# Immunoglobulin light chain (IGL) genes in torafugu: Genomic organization and identification of a third teleost IGL isotype

**DOI:** 10.1038/srep40416

**Published:** 2017-01-18

**Authors:** Xi Fu, Fengjun Zhang, Shugo Watabe, Shuichi Asakawa

**Affiliations:** 1 State Key Laboratory of Biotherapy & Collaborative Innovation Center for Biotherapy, West China Hospital, Sichuan University, Chengdu 610041, China; 2 Department of Aquatic Bioscience, Graduate School of Agricultural and Life Sciences, The University of Tokyo, Bunkyo, Tokyo 113-8657, Japan; 3 School of Marine Bioscience, Kitasato University, Sagamihara, Kanagawa 252-0373, Japan

## Abstract

Here, we report a genome-wide survey of immunoglobulin light chain (IGL) genes of torafugu (*Takifugu rubripes*) revealing multi-clusters spanning three separate chromosomes (v5 assembly) and 45 scaffolds (v4 assembly). Conventional sequence similarity searches and motif scanning approaches based on recombination signal sequence (RSS) motifs were used. We found that three IGL isotypes (L1, L2, and L3) exist in torafugu and that several loci for each isotype are present. The transcriptional orientations of the variable IGL (V_L_) segments were found to be either the same (in the L2 isotype) or opposite (in the L1 and L3 isotypes) to the IGL joining (J_L_) and constant (C_L_) segments, suggesting they can undergo rearrangement by deletion or inversion when expressed. Alignments of expressed sequence tags (ESTs) to corresponding germline gene segments revealed expression of the three IGL isotypes in torafugu. Taken together, our findings provide a genomic framework for torafugu IGL genes and show that the IG diversity of this species could be attributed to at least three distinct chromosomal regions.

The adaptive immune system (AIS) functions via a diverse repertoire of antigen receptors: immunoglobulins (IGs) and T cell receptors (TCRs). The key effectors, IGs, which are expressed only by jawed vertebrates, are primarily involved in antibody responses^1^. A typical IG molecule consists of two identical heavy (IGH) and two identical light (IGL) chains. Each IGL chain has two domains: a constant (C) and variable (V) domain. The IGL C domain is encoded by the constant (C_L_) gene. As for the V domain, individual variable (V_L_) and joining (J_L_) gene segments rearrange somatically at the DNA level to generate V-J regions, which, after transcription and translation, encode the functional V domains of IGL^2^. Primary diversification of IGs occurs early in B cell development during V(D)J recombination. The IG repertoire is further diversified by the action of activation-induced cytidine deaminase (AID), which catalyzes IG somatic hypermutation and class switch recombination (absent in fishes) in mammals and other tetrapods^3^,^4^. V(D)J recombination is initiated by RAG recombinase, which recognizes recombination signal sequences (RSSs) flanking each V, D, and J gene segment and cleaves DNA during V(D)J recombination^5^. The RSSs are composed of conserved heptamer and nonamer sequences, separated by either 12 ± 1 or 23 ± 1 base pair (bp) spacer sequences^6^. The V domain consists of three complementarity-determining regions (CDRs) of highly variable sequence and four framework regions (FRs) of relatively constant sequence.

Jawed vertebrate species, with the exception of chickens, ducks, and snakes, express more than one IGL isotype^7^,^8^,^9^. It has long been known that mammals have two distinct IGL isotypes called kappa (κ) and lambda (λ), but additional IGL isotypes have been described in other vertebrate groups. The current classification system groups all vertebrate IGLs into four main ancestral branches: kappa (mammalian κ, elasmobranch type III/NS4, teleost L1/L3/F/G, *Xenopus* ρ), lambda (mammalian λ, elasmobranch type II/NS3), sigma (*Xenopus* σ, teleost L2, elasmobranch type IV), and sigma-2 (elasmobranch type I/NS5, variant sigma-type in coelacanth)^10^,^11^.

Traditionally, different vertebrate IGL sequences are classified by: (1) sequence identity, (2) IGL gene organization, and (3) spacing of RSS heptamer and nonamer motifs that flank V_L_ and J_L_^12^. Additionally, Criscitiello and Flajnik^13^ have proposed CDR lengths of corresponding V_L_, specifically CDR1 and CDR2, to be a valid criterion for the classification of IGL. Another IGL classification criterion using a set of 21 conserved molecular sequence markers to distinguish κ, λ, and σ IGL isotypes was later proposed by Das *et al*.^14^.

Teleost IGL genes, as those of cartilaginous fish, have been shown to be in a multi-clustered configuration^15^,^16^,^17^,^18^,^19^,^20^,^21^, defined as independently rearranging mini-loci consisting of few gene segments (multiple V segments, one J) and one C domain exon^22^. The IGL loci in teleosts form tightly linked clusters and there are significant differences in the number of loci for each isotype among species. The presence of multiple clusters on one or more chromosomes, similar to those found in zebrafish (*Danio rerio*), three-spined stickleback (*Gasterosteus aculeatus*), and medaka (*Oryzias latipes*), suggests a major role for cluster duplication in the generation of IG diversity in teleosts^18^.

Torafugu (*Takifugu rubripes*) has a recognizable adaptive immune system and one of the smallest genomes (~400 Mb) among vertebrates^23^, which makes it a good model for research in comparative immunology. Two partial annotations of torafugu IGLs have been reported^18^,^24^, revealing IGL assemblages with respect to gene segment number, cluster orientations, and organization on three scaffolds and two clones that contain L1 and L2 loci, respectively. In this work, we have scanned the torafugu genome assemblies to provide an extended annotation of torafugu IGLs as well as their genomic organization. Our research showed the identification of a third teleost IGL isotype (L3) in torafugu and an expansion of the IGL genes that were identified in previous studies.

## Results

### Identification of IGL genes on torafugu genomic chromosomes and scaffolds

**Figure 1:**
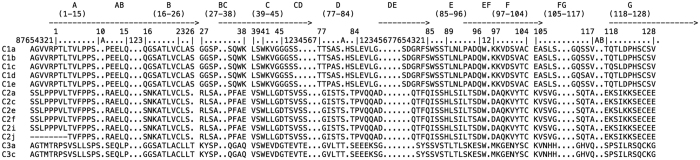
IMGT protein display of in-frame torafugu C_L_ representative amino acid sequences. The protein display is shown using IMGT header (IMGT Repertoire, http://www.imgt.org).

A total of 82 IGL gene segments in torafugu were found to be localized on three different chromosomes, i.e., 2, 3, and 5, and were confined to 45 different genomic scaffolds (see annotation details in Supplementary Dataset File). Of the scaffolds, four (scaffold 10, 158, 54, and 139) were assigned to separate chromosomes, whereas most of the IGL genes could not be anchored to chromosomes. Altogether, 48 V_L_ (Table 1 and Supplementary Dataset File), 13 J_L_ (Table 2 and Supplementary Dataset File) and 21 C_L_ (Fig. 1 and Supplementary Dataset File) gene segments^25^ (except for those that might be present in gaps and cannot be identified at present) were identified.

### Identification of a third teleost IGL isotype in torafugu

Homology in the C domain is the most reliable criterion for classifying a teleost IGL isotype^18^. As mentioned, two IGL isotypes have been reported in torafugu: L1 and L2. Here, we used the published IGL sequences from various teleosts to search the torafugu database (http://www.fugu-sg.org/). As a result, three scaffolds (scaffold 2422, 2488, and 3698) were found to carry C_L_ sequences that had homology (47–53% amino acid identities) with the L3 C domains of zebrafish, carp (*Cyprinus carpio*), and channel catfish (*Ictalurus punctatus*). This degree of homology in the C domain exceeds the limit used to distinguish mammalian κ and λ C domains (35–37%), thus further strengthens the identification of a torafugu L3. BLAST^26^ searches with the V_L_ segments on the three scaffolds revealed similarities with L1/L3 V from other teleosts. After amino acid identity, RSS orientation is the second most common characteristic used for distinguishing IGL isotypes^13^. The torafugu L3 RSSs have the V12-23J motif, similar to that in mammalian κ^27^,^28^.

### Type 3 IGL organization

**Figure 2:**
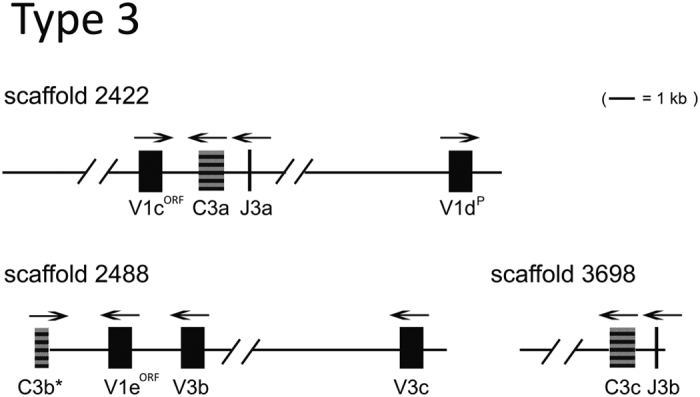
Overall organization of representative type 3 IGL genes. Scaffold 2422 of 14,667 bp, 2488 of 13,611 bp, and 3698 of 3784 bp, are shown to scale, with exon size exaggerated. The transcriptional polarity is indicated by overhead arrow. Each gene is labeled, and an asterisk denotes incomplete coding sequences. V^P/ORF^ denotes pseudogene (P) or ORF sequence.

Of the three scaffolds (2422, 2488, and 3698) that carry one L3 C sequence each, scaffold 2422 contains one each of a functional L1 V (V1c), L1 V without leader sequence (V1d), and J_L_ (J3a); scaffold 3698 contains one J_L_ (J3b); and scaffold 2488 contains three V_L_ sequences that belong to L1 V (V1e) and L3 V (V3b and V3c) within the same cluster (Fig. 2). This heterogeneity suggests an organization of multiple clusters. If a region harboring one C_L_ is considered as one cluster, at least three clusters should exist at the L3 loci. The L3 C sequences share 48–75% identity with each other at the amino acid level, which suggests their divergence from each other, while they are nonetheless distinguishable from the L1/L2 C sequences (10–31% identity in all inter-type pair-wise comparisons). The functional V_L_ segments fall into two groups and correspond to L1 V (V1c, V1_d_, and V1e) and L3 V (V3b and V3c), respectively. Within a group, they are 88–92% identical at amino acid level over the V_L_ coding sequences; between the two groups, they share 34–42% identities. All five V_L_ segments are arranged in the opposite transcriptional orientation to their C_L_ and J_L_ on each individual scaffold, similar to that described for other teleost L3 genes^10^.

**Figure 3:**
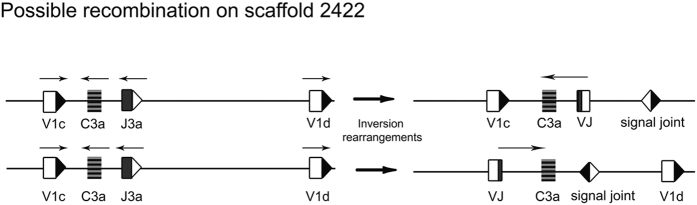
Inversion rearrangements on scaffold 2422. The transcription polarity of the rearranged VJ, at the right, is indicated by arrowheads on the top of VJ-C. The J_L_-RSS is indicated as a white triangle, the V_L_-RSS is indicated as a black triangle.

The V1d sequence was defined as a pseudogene due to the absence of a leader sequence in the current assembly. However, it may rearrange functionally to J_L_ with its identifiable V_L_ exon and the downstream RSS sequence. Therefore, the V_L_ on both sides of the J_L_/C_L_ will likely undergo rearrangement with C3a and J3a through inversion as in other teleosts. For example, V1d will possibly invert to join J3a, while V1c will recombine through inversion of J3a and C3a (Fig. 3).

### Type 2 IGL organization

**Figure 4:**
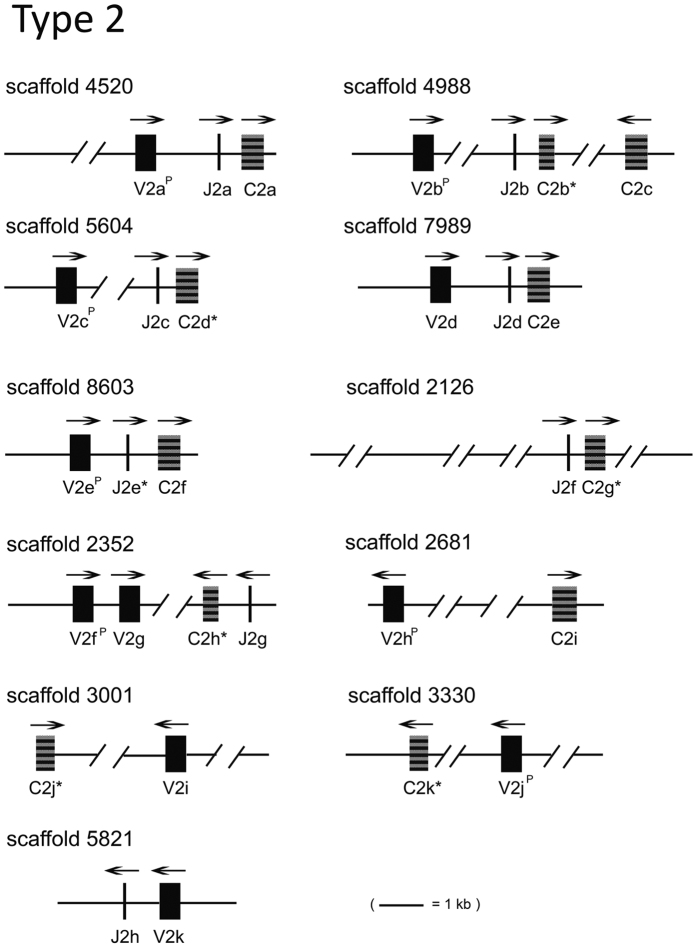
Overall organization of representative type 2 IGL genes. The transcriptional polarity is indicated by overhead arrow. An asterisk denotes an incomplete coding sequence. V^P/ORF^ denotes a pseudogene or ORF sequence.

A search with L2 C sequences from various teleosts showed good matches with 10 scaffolds (scaffold 4520, 4988, 5604, 7989, 8603, 2126, 2352, 2681, 3001, and 3330) in the v4 assembly. Other scaffolds were found to contain either L2 V or J sequences (Fig. 4). The torafugu L2 loci contain 22 V_L_, 8 J_L_, and 11 C_L_ gene segments. All 22 V-matching sequences (some were found only as fragments owing to gaps in the sequences) were summarized in Table 1. The genomic organization of L2 genes was depicted in Fig. 4. C2a, C2c, and C2i are identical with the published L2 torafugu C sequence^18^. Other L2 C sequences (those with complete coding sequences) are 92–99% identical with C2a in the derived amino acid sequences and only share 15–35% identity with L1/L3 C sequences, suggesting that they duplicated among themselves and diverged long ago from other types. The L2 V gene segments are either in the same or in the opposite transcriptional orientation as their corresponding J_L_ and C_L_, which is topologically similar to the three-spined stickleback L2 genes on chromosome 11^15^. It is worthy to note that although all the scaffolds carrying V_L_ in the opposite orientation as C_L_ and J_L_ are missing sequence information between V_L_ and J_L_-C_L_ (e.g., sequences in scaffold 4988, 2352, 2681, and 3001). For example, the orientation of V2f and V2g on scaffold 2352 appears to be opposite to that of C2h and J2g. However, two possibilities should be considered: (1) the gaps between these gene segments may contain novel C_L_ and J_L_ segments with the same orientation as V2f and V2g and (2) scaffold joining might reveal additional V_L_ segments that are downstream of and in the same orientation as C2h and J2g. The L2 locus is most likely occupied by eleven clusters, and on average one V_L_ segment resides in each cluster. Conventional recombination at the L2 locus would occur. For example, rearrangement between V2d and J2d on scaffold 7989 will occur by deletion of the intervening DNA to form a V_L_J_L_.

On scaffold 54 and 139, assigned respectively to chromosome 3 and 5^29^, only one L2 V was detected and no corresponding C_L_ or J_L_ could be identified, based on both v4 and v5 assemblies. The other L2 sequences identified on v4 scaffolds could not be assigned to v5 chromosome (s) due to the presence of gaps.

### Type 1 IGL organization

**Figure 5:**
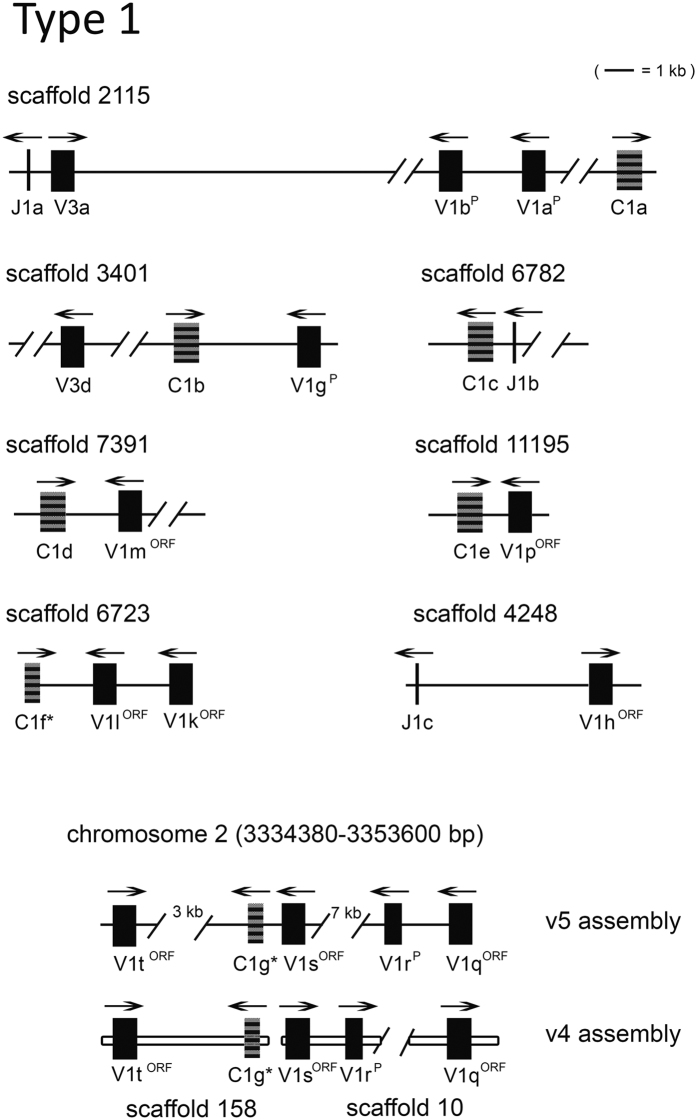
Overall organization of representative type 1 IGL genes. The transcriptional polarity is indicated by overhead arrow. An asterisk denotes an incomplete coding sequence. V^P/ORF^ denotes a pseudogene or ORF sequence. Scaffold 158 and 10 were assigned to chromosome 2.

L1 and L3 V sequences appear to be intermixed (discussed below). We described L1 IGL genes on at least seven genomic scaffolds (scaffolds with L1 C), thus they might operate as seven loci. As expected, L1 C sequences possess high amino acid identity (≥96%) with each other and the divergence from other types was evident (15–35% identity compared to L2/L3 C). As depicted in Fig. 5, the transcriptional polarity pattern in the L1 loci presents as V_L_ in both orientations to J_L_ and C_L_. In fact, in all but one instance (chromosome 2), the overall impression is that the L1 locus is organized as V_L_ opposite to nearby J_L_ and C_L_. On chromosome 2, four V_L_ segments were identified, with three placed in the same transcriptional orientation to the C_L_ (C1g) and another one in the opposite direction. On the other hand, sequences on scaffold 158 were perfectly assigned to chromosome 2, including V1t and C1g, while scaffold 10 was anchored to chromosome 2 in reverse, that is, it has the same V_L_ segments (V1q, V1r, and V1s) in opposite directions (Fig. 5).

### IGL cluster estimation

**Figure 6:**
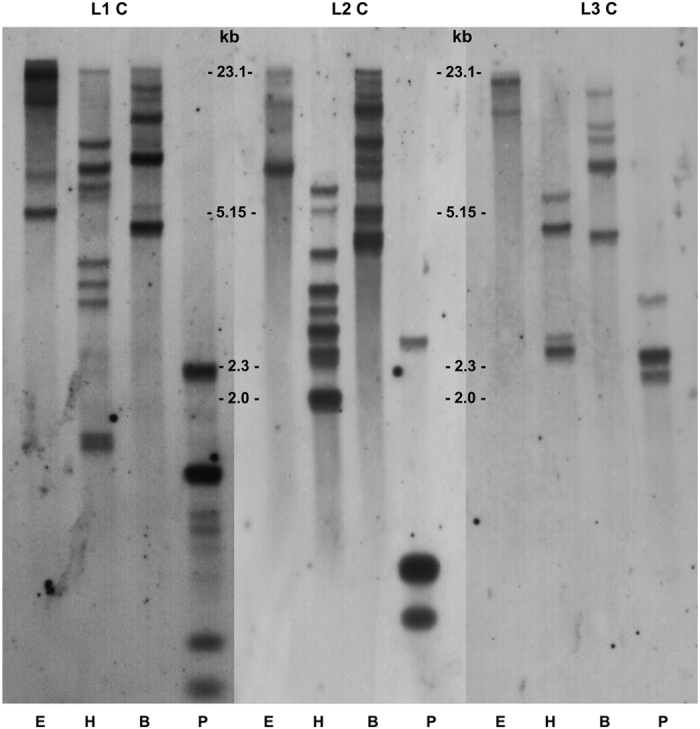
Southern blot of genomic DNA from torafugu sperm probed with torafugu IGLC. Restriction endonucleases are indicated at the bottom: *Eco*RI (E), *Hind*III (H), *Bam*HI (B), and *Pst*I (P). Figures are cropped and the original blots images are available in Additional File.

Southern blots of torafugu genomic DNA from sperm probed with different types of C_L_ reveal that the IGL genomic organization in this species is of the cluster type (Fig. 6). More than two bands in most digests suggest multiple IGL loci. Judging by the number of hybridizing bands, seven and three IGL loci are common in L1 and L3. For the L2 isotype, the number of clusters is lower than predicted. It is noticeable that the two bands digested by *Pst*I are much stronger than other bands in L2 blots, which is attributable, at least in part, to the fact that there is no or limited polymorphism with *Pst*I and many bands are hybridized at the same spot.

### Phylogenetic analyses

**Figure 7:**
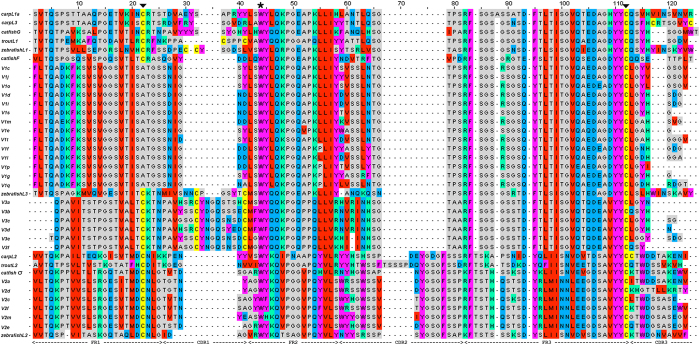
Overview window from Jalview of alignment of teleosts V_L_ representative amino acid sequences as determined by MAFFT. Hyphens denote gaps. FR and CDR regions are labeled according to Kabat delineation^42^. The conserved Tryptophan (Trp, W) in FR2 region is indicated by an asterisk. Cysteines (Cys, C) that are expected to form intra-chain disulfide bridges are indicated by solid black triangles, with the exception of torafugu IGLV1 group sequences (wherein Cys is replaced by Ala).

The V_L_ domains of different teleost species and IGL isotypes were aligned (Fig. 7). Similar to the report by Criscitiello and Flajnik^13^, the comparison analysis revealed the conservation of a long CDR2 in L2 V (relative to other isotypes) and a long CDR1 in L3 V. The torafugu L1 V sequences were found to possess both short CDR1 and short CDR2, and were missing the key amino acid 1st-CYS in the FR1 region; this may be a torafugu-specific finding.

**Figure 8:**
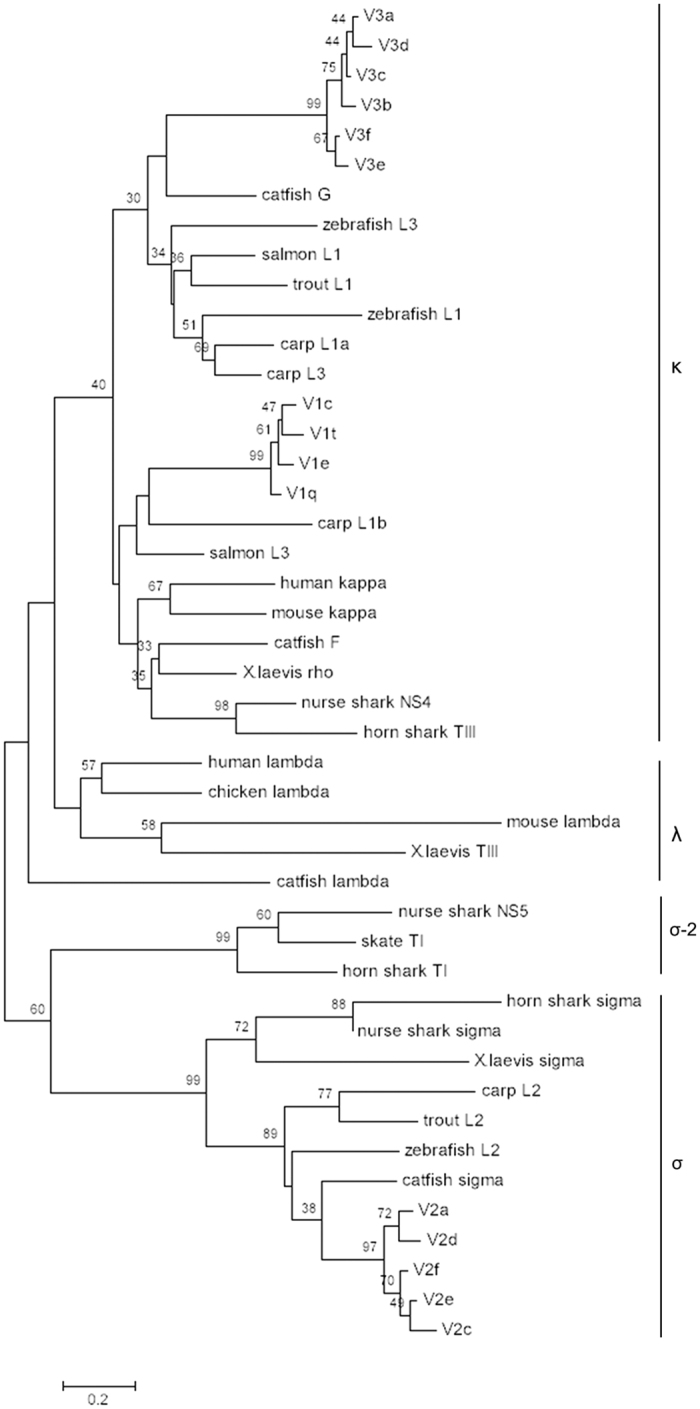
Phylogenetic analysis of representative V_L_ from various vertebrates. The NJ tree was constructed using MEGA 7 with 1000 bootstrap replications. GenBank accession numbers are: zebrafish L1 (AF246185); carp L1a (AB073328); carp L3 (AB073335); zebrafish L3 (AF246193); salmon (*Salmo salar*) L1 (AF273012); trout (*Oncorhynchus mykiss*) L1 (X65260); catfish G (L25533); carp L1b (AB073332); human kappa (S46371); mouse kappa (MUSIGKACN); salmon L3 (AF406956); catfish F (U25705); *X. laevis (Xenopus laevis*) rho (XELIGLVAA); horn shark (*Heterodontus francisci*) TIII (L25561); nurse shark (*Ginglymostoma cirratum*) NS4 (A49633); *X. laevis* TIII (L76575); mouse lambda (AY648665); chicken lambda (M24403); human lambda (AAA59013); catfish lambda (EU925383); nurse shark NS5 (AAV34678); skate (*Leucoraja erinacea*) TI (L25568); horn shark TI (X15315); horn shark sigma (EF114760); nurse shark sigma (EF114765); *X. laevis* sigma (S78544); carp L2 (AB091113); trout L2 (AAB41310); zebrafish L2 (AF246162); catfish sigma (EU872021).

A phylogenetic tree was constructed based on the alignment of V_L_ amino acid sequences from various vertebrates (Fig. 8). The torafugu L2/σ V sequences (V2a, V2c, V2d, V2e, and V2f) clustered strongly together and were distinct from the κ group (including teleost L1 and L3), which seemed to be mingled (V_L_ sequences from the same scaffold are not necessarily in one group). Interestingly, although all the torafugu IGLV1 and IGLV3 sequences belong to the mammalian κ isotype, they clustered to separate groups. This suggests that they are probably associated with different sub-isotypes or a teleost-specific IGL isotype, as is the case in stickleback^15^.

**Figure 9:**
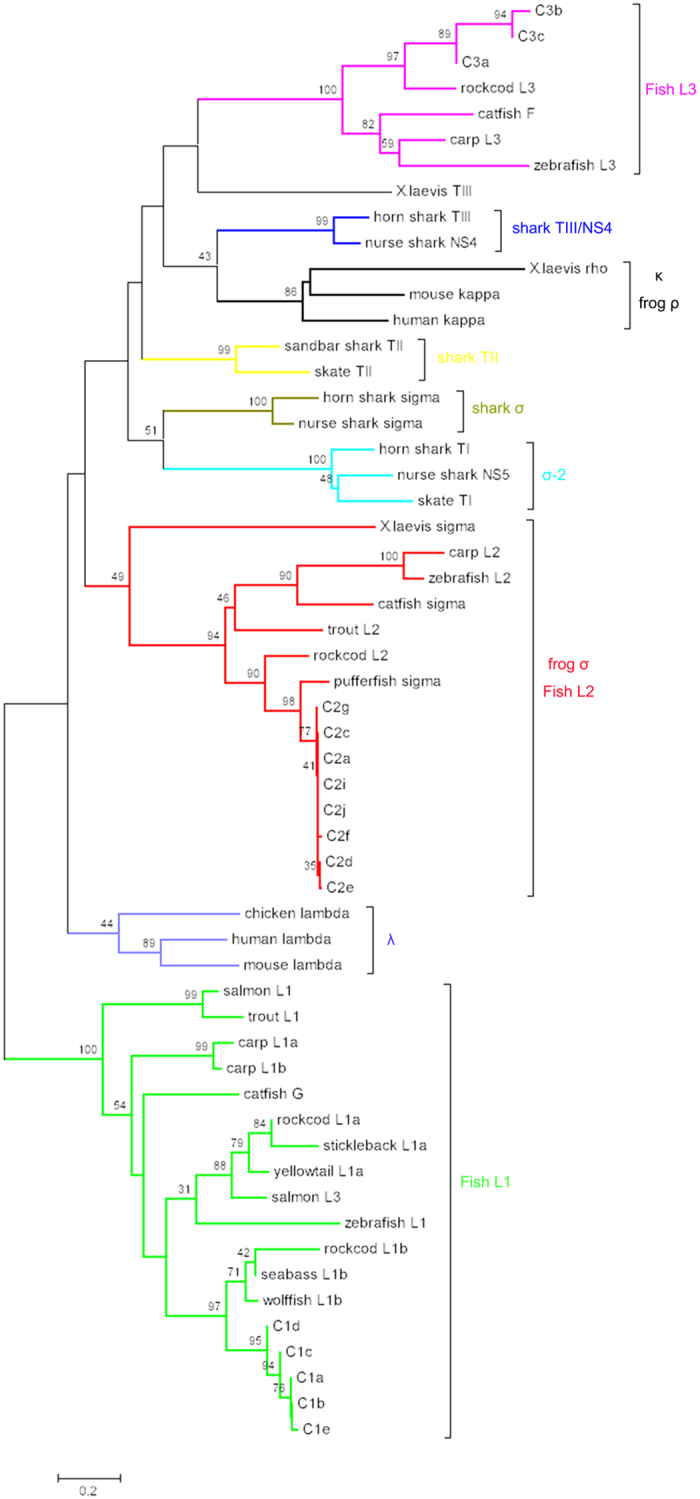
Phylogenetic analysis of C_L_ from various vertebrates. GenBank accession numbers are as follows: wolffish L1b (AF137398); rockcod L1b (DQ842622); seabass L1b (AJ400216); zebrafish L1 (AF246185); salmon L3 (AF406956); yellowtail L1a (AB062619); rockcod L1a (EF114784); stickleback L1a (AY278356); catfish G (L25533); carp L1a (AB035728); carp L1b (AB035729); salmon L1 (AF273012); trout L1 (X65260); chicken lambda (M24403); human lambda (AAH07782); mouse lambda (J00592); *X. laevis* sigma (S78544); carp L2 (AB103558); zebrafish L2 (AF246162); catfish sigma (EU872021); trout L2 (AAB41310); rockcod L2 (EF114785); pufferfish (*Tetraodon nigroviridis*) sigma (AJ575637); horn shark TI (X15315); nurse shark NS5 (AAV34681); skate TI (L25568); horn shark sigma (EF114760); nurse shark sigma (EF114765); sandbar shark (*Carcharhinus plumbeus*) TII (M81314); skate TII (L25566); mouse kappa (AB048524); *X. laevis* rho (XELIGLVAA); human kappa (M11937); carp L3 (AB035730); zebrafish L3 (AF246193); catfish F (U25705); rockcod L3 (DQ842626).

Torafugu C_L_ segments were compared using phylogenetic trees to evaluate the C_L_ relationships among vertebrates (Fig. 9). None of the torafugu C_L_ segments cluster with mammalian κ or λ IGL sequences. However, torafugu C_L_ segments group strongly in branches with sequences belonging to the same teleost isotype (L1, L2, and L3), suggesting that teleosts share a common derivation and that three or more IGL isotypes may have been present in a teleost ancestor. A close relationship between torafugu (belonging to the Tetradontiformes order, Acanthopterygii superorder), and other species from the Perciformes order (Acanthopterygii), such as seabass (*Dicentrarchus labrax*), rockcod (*Trematomus bernacchii*), and wolffish (*Anarhichas minor*), is also evident from the tree. In addition, phylogenetic analysis consistently revealed the tendency of C_L_ clustering according to taxonomic group rather than the isotype^13^,^30^. Taken together, the results of the phylogenetic analysis of the torafugu V_L_ and C_L_ sequences revealed different selective pressures on the two domains, wherein C_L_ tends to cluster according to taxonomic group, while V_L_ tends to group by isotype.

Isotype distribution was assessed for the J_L_ segments and J_L_1, J_L_2, and J_L_3 sequences were distinguished (Supplementary Fig. S1). Of all J_L_ segments identified, those belonging to L1 and L3 were most similar to each other.

### Analysis of V_L_ gene 5′ flanking regulatory sequences

We examined 5′ flanking sequences for identified V_L_ segments to reveal possible regulatory features. The 5′ flanking region contains two conserved motifs, namely the octamer motif, which is critical to correct transcription of IGL genes, and the TATA box for the general transcription process^31^. As summarized in Table 1, all 5′ flanking sequences of functional V_L_ segments exhibit considerable family-specific conservation i.e., (1) all the functional or open reading frame (ORF) segments of the IGLV1 family contain sequences completely identical to the octamer consensus (ATTTGCAT) and the TATA consensus (TTTAAA); (2) IGLV2 sequences show slightly less conserved octamer sequences and most functional members have single point variation (ATG-T/C-AAAT) in the octamer sequence; the TATA consensus (TATTAA) is well conserved across functional IGLV2 genes; (3) members of the IGLV3 family have consensus octamer (ATTTCCAT) and TATA (TTTATA) sequences.

### Functionality of torafugu IGL loci

A total of fifteen torafugu EST sequences associated with IGL expression were identified from the NCBI EST database. Alignment of torafugu ESTs to concordant genomic V_L_ segments revealed that all functional IGLV3 genes were expressed, while only one IGLV2 sequence (V2k) was expressed. Additionally, expression of all the IGLV1 sequences was observed despite the fact that they were missing the 1st-CYS in the FR1 region. Expression of all the complete C_L_ segments was also observed with one exception: the C1d on scaffold 7391. Upon detailed examination, 9 ESTs and 6 ESTs were found to be concordant with the L2 locus and L1/L3 loci, respectively. Interestingly, ESTs associated with L2 and L3 C sequences were found to lack a V_L_ segment, except for EST AL835785, which carried a complete V_L_J_L_-C_L_ (L2 C). In comparison, expression of L1 C sequences was often found to be with either IGLV1 or IGLV3 sequences (Supplementary Table S1). The identity of all the retrieved ESTs to genomic V_L_ and C_L_ segments is 95–100%, suggesting the feasibility of using this method to assign ESTs to concordant genomic sequences.

## Discussion

In the present study, we have characterized the torafugu IGL genomic organization based on available genome data sets. It has been reported that torafugu has two IGL isotypes, L1 and L2. Here, a teleost L3 isotype was newly identified, demonstrating that torafugu possesses at least three IGL isotypes. All the IGL genes have been found to be partitioned over multiple scaffolds (v4 assembly). Currently, we can only speculate that torafugu IGL genes should be assigned to three different chromosomes due to incomplete sequence information from the v5 assembly. Our observations must be taken as a step forward in the elucidation of torafugu IGL genomic organization and future studies on more complete genome assembly may help to address the current issues with gaps and false assemblies in the whole genome sequence.

During vertebrate phylogeny, IGL genes have undergone major evolutionary transitions involving genomic arrangements. One extreme example is the presence of a single IGL isotype (λ) in bird species, such as chicken and zebra finch^7^,^32^. Unlike mammalian κ and λ loci, which are often arranged in a translocon fashion, teleost IGL genes are organized in distinct clusters of (V_L_-J_L_-C_L_)_n_. Herein, we show that torafugu IGL genes are arranged in a compact multi-cluster configuration, supported by both the genomic organization and the Southern blot result. This observation is similar to that found in other teleosts, suggesting a conservation of the cluster IGL organization among teleost species.

In regard to the comparative analysis of the sequences of torafugu C_L_ with those of other vertebrates, the relative distances are in agreement with the phylogenetic relationships. The torafugu C_L_ share the same cluster with teleost L1, L2, and L3, respectively. Moreover, a sister-group relationship (Fig. 9) in the superorder Acanthopterygii between torafugu L1 C sequences and those of the L1b subgroup (wolffish L1b, seabass L1b, and rockcod L1b) is supported by the observed high bootstraps values. At this time, we did not find an L1a C homolog in torafugu, but if such sequences are found in the future, this would further support the hypothesis that L1a and L1b subtypes exist in the Acanthopterygii L1 isotype^33^. In addition, the identification of an L3 in torafugu (Acanthopterygii), together with the presence of L3 in rockcod (Acanthopterygii) and Ostariophysi (catfish, zebrafish, and carp), suggests that the divergence between L1 and L3 took place at or before the emergence of Euteleosts^18^.

Finally, screening of the EST database indicates that the majority of IGLV1 and IGLV3 genes are expressed. However, most of the ESTs associated with the expression of L2 C do not have a corresponding V_L_ segment. This phenomenon has been previously described in zebrafish^34^ and medaka IGκ^19^, and it may be related to the low efficiency in eliminating aberrant IGL transcripts^35^.

The observation that torafugu V_L_ and C_L_ from different isotypes (i.e., L1 and L3) may join together to achieve potential expression at the rearrangement level is somewhat reminiscent of the previous finding in zebrafish wherein inversional VJ-rearrangements leapfrog C_L_ occur between clusters^21^.It is plausible that (1) torafugu L1 and L3 clusters are close to one another in the genome, which may allow recombination between different isotypes, (2) torafugu with multiple C_L_ on a scaffold are poised to reconstruct the IGL locus by inversional rearrangement, which can bring V_L_ from one cluster into another, similar to that of zebrafish^21^. With efforts to sequence additional genomes, it will be intriguing to investigate whether the inversional inter-cluster rearrangement is teleost-specific or a commonplace in other species.

## Methods

### Retrieval of IGL genes from the torafugu genome

Genome builds of torafugu (assembly v4, October 2004 and assembly v5, January 2010) available from the Fugu Genome Project^29^ (http://www.fugu-sg.org/) were searched to locate the IGL genes. Published IGL amino acid sequences from torafugu^18^,^24^ and other teleosts^15^,^17^,^20^ were used as queries in TBLASTN alignments (cutoff *E*-value of 10^−15^) to retrieve relevant scaffolds and chromosomes. Genomic sequences that contain matches for both V_L_ and C_L_ were downloaded for further analysis. The identified genomic sequences were subsequently used as queries in BLASTN searches against the EST database at NCBI to retrieve expression data. Expression of V_L_ genes was determined by BLAST hits using a 95% threshold identity and a 10^−15^ *E*-value threshold, while ESTs were assigned to concordant C_L_ when a ≥99% identity was met.

### Annotation of torafugu IGL

Artemis^36^ was used to annotate the IGL loci, including the transcriptional polarity and relative positions of V_L_ and C_L_ in the genomic sequences. C exons were discerned by comparing resultant genomic sequences with published IGL mRNAs. V_L_ genes were determined based on the presence of canonical RSS (allowing 2 nucleotide mismatches), with ORFs that match for IG signature sequences using IgBLAST (www.ncbi.nlm.nih.gov/projects/igblast) and IMGT/V-QUEST^37^ (the Teleostei unit), and finally by pattern searches for 23RSS or 12RSS flanking ends of gene segments. To identify the J_L_ genes, which are too short to be detected by BLAST searches, we performed pattern searches to find J_L_-specific RSSs among the initial genomic sequences that contain V_L_ and C_L_. The pattern is a consensus RSS heptamer and a nonamer with a 22-24 bp spacer (CACAGTG-N22-24-ACAAAAACC) region. Splice sites between leader and V exons were discerned by FSPLICE (http://linux1.softberry.com/berry.phtml). Exon boundaries of V_L_, J_L_, and C_L_ were refined by alignment with known VJ-C cDNA sequences and torafugu EST sequences (from Fugu Genome Project)^38^.

### Nomenclature

Identified IGL genes were annotated according to the IMGT^®^ nomenclature^39^. For the V_L_ genes, all retrieved sequences without a truncation, frameshift mutation, or premature stop codon in the leader exon and the V exon, which had conserved residues (1^st^-CYS, conserved-TRP, and 2^nd^-CYS) in FR1, FR2, and FR3 regions, respectively, and possessed a proper RSS, were deemed as functional genes. For the C_L_ and J_L_ gene segments, retrieved sequences without frameshift mutations and internal stop codons were regarded as potentially functional genes. In addition, examination of RSS was implemented to determine putative functionality of J_L_.

### Comparative phylogenetic studies

Phylogenetic studies were carried out using the MEGA7 program^40^. Multiple sequence alignments were performed using MAFFT^41^. The neighbor-joining (NJ) method was used to construct phylogenetic trees (pair-wise deletion, Jones-Taylor-Thornton matrix) and to enter range-activated sites by gamma parameter 2.5. Evaluation of the veracity of these trees was done by executing a bootstrap procedure of 1000 replicates.

### Southern blotting

Genomic DNA from torafugu sperm (5 μg; extracted using DNeasy^®^ Blood & Tissue Kit, Qiagen, Valencia, CA) was digested with *Eco*RI, *Hind*III, *Bam*HI, and *Pst*I. The digested DNA was electrophoresed on 0.8% agarose gels for 16 h and transferred onto Hybond-N+ membranes (GE Healthcare, Piscataway, NJ). Hybridizations and subsequent detection were performed according to the manufacturer’s instructions (Amersham AlkPhos Direct™, GE Healthcare). Torafugu C probes consisted of the entire CL domain of L1, L2, and L3. The probes were amplified using Platinum^®^ Taq DNA Polymerase High Fidelity (Invitrogen, Carlsbad, CA). The conditions for the thermal cycler were: 94 °C for 2 min, followed by 30 cycles of 94 °C for 30 s, 55 °C for 30 s, 68 °C for 1 min, and a final extension at 68 °C for 5 min (see primer details in Supplementary Table S2).

## Additional Information

**Accession codes:** The annotation data (Supplementary Dataset File) of v4 scaffolds have been deposited in GenBank under accession numbers KU350660 to KU350678, KU359177 to KU359180, and KU365386 to KU365407.

**How to cite this article**: Fu, X. *et al*. Immunoglobulin light chain (IGL) genes in torafugu: Genomic organization and identification of a third teleost IGL isotype. *Sci. Rep.* **7**, 40416; doi: 10.1038/srep40416 (2017).

**Publisher's note:** Springer Nature remains neutral with regard to jurisdictional claims in published maps and institutional affiliations.

## Supplementary Material

Supplementary Dataset 1

Supplementary Information

## Figures and Tables

**Table 1:** Genomic features of the torafugu *V*_*L*_ genes.

IGLV family	V_L_ gene	Fct	Promoter				Gene structure			RSS		
			Octamer	(nt)	TATA	(nt)	L-PART1 (nt)	Intron	V-exon	7mer	Spacer (nt)	9mer
IGLV1	V1a^^^	P^a^	—	—	—	—	—	—	114	CACAGTG	12	ACAAAAACC
	V1b^^^	P^b^	ATTTGCAT	27	TTTAAA	64	40	137	249	—	—	—
	V1c^^^	ORF^l^	ATTTGCAT	27	TTTAAA	64	40	137	285	CACAGTG	12	ACAAAAACC
	V1d^^^	P^a^	—	—	—	—	—	—	267	CACAGTG	12	ACAAAAACC
	V1e^^^	ORF^l^	ATTTGCAT	27	TTTAAA	64	40	137	291	CACAGTG	12	ACAAAAACC
	V1f	ORF^l^	—	—	TTTAAA	64	40	137	300	CACAGTG	12	ACAAAAACC
	V1g^^^	P^a^	—	—	—	—	—	—	300	CACAGTG	12	ACAAAAACC
	V1h^^^	ORF^l^	ATTTGCAT	27	TTTAAA	64	40	137	300	CACAGTG	12	ACAAAAACC
	V1i	P^a^	—	—	—	—	—	—	300	CACAGTG	12	ACAAAAACC
	V1j	ORF^l^	ATTTGCAT	27	TTTAAA	64	40	137	285	CACAGTG	12	ACAAAAACC
	V1k^^^	ORF^l^	—	—	—	—	40	137	291	CACAGTG	12	ACAAAAACC
	V1l^^^	ORF^l^	ATTTGCAT	27	TTTAAA	—	40	133	291	CACAGTG	12	ACAAAAACC
	V1m^^^	ORF^l^	ATTTGCAT	27	TTTAAA	64	40	140	285	CACAGTG	12	ACAAAAACC
	V1n	ORF^l^	ATTTGCAT	27	TTTAAA	64	40	137	300	CACAGTG	12	ACAAAAACC
	V1o	ORF^l^	ATTTGCAT	27	TTTAAA	62	40	137	285	CACAGTG	12	ACAAAAACC
	V1p^^^	ORF^l^	—	—	TTTAAA	64	40	137	288	CACAGTG	12	ACAAAAACC
	V1q^^^	ORF^l^	ATTTGCAT	27	TTTAAA	62	40	137	300	CACAGTG	12	ACAAAAACC
	V1r^^^	P^b^	ATTTGCAT	27	TTTAAA	64	40	133	168	—	—	—
	V1s^^^	ORF^l^	—	—	—	—	40	137	291	CACAGTG	12	ACAAAAACC
	V1t^^^	ORF^l^	ATTTGCAT	27	TTTAAA	64	40	137	291	CACAGTG	12	ACAAAAACC
IGLV2	V2a^^^	P^f^	ATGTAAAT	107	TATTAA	97	40	92	326	CACAGTG	12	ACAAAAACC
	V2b^^^	P^b^	ATGTAAAT	107	TATTAA	97	40	92	206	—	—	—
	V2c^^^	P^h^	ATGTAAAT	107	TATTAA	97	40	92	323	CACAGTG	12	ACAAAAACC
	V2d^^^	F	ATGCAAAT	101	TATTAA	97	40	92	302	CACAGTG	12	ACAAAAACC
	V2e^^^	P^k^	ATGTAAAT	107	TATTAA	97	40	92	326	CACAGTG	12	ACAAAAACC
	V2f^^^	P^a^	ATGTAAAT	—	—	—	—	—	329	CACAGTG	12	ACAAAAACC
	V2g^^^	F	TTGAAAAT	88	TATTAA	97	40	92	326	CACAGTG	12	ACAAAAACC
	V2h^^^	P^b^	ATGTAAAT	107	TATTAA	97	40	92	215	—	—	—
	V2i^^^	F	ATGCAAAT	101	TATTAA	97	40	92	329	CACAGTG	12	ACAAAAACC
	V2j^^^	P^d^	ATGTAAAT	107	TATTAA	97	40	195	225	CACAGTG	12	ACAAAAACC
	V2k^^^	F	ATGTAAAT	107	TATTAA	97	40	217	182	CACAGTG	12	ACAAAAACC
	V2l	P^b^	ATGTAAAT	107	TATTAA	97	40	92	230	—	—	—
	V2m	P^c^	ATGTAAAT	107	TATTAA	97	40	92	329	CACAGTG	12	ACAAAAACC
	V2n	P^e^	ATGTAAAT	107	TATTAA	97	40	92	326	CACAGTG	12	—
	V2o	P^g^	—	—	TTAAAT	97	40	92	326	CACAGTG	12	ACAAAAACC
	V2p	P^i^	—	—	—	—	40	92	326	CACAGTG	12	ACAAAAACC
	V2q	P^b^	ATGTAAAT	107	TATTAA	97	40	92	302	—	—	—
	V2r	P^j^	—	—	TATTAA	97	40	92	329	CACAGTG	12	ACAAAAACC
	V2s	P^a^	—	—	—	—	—	92	326	CACAGTG	12	ACAAAACCT
	V2t	F	ATGTAAAT	107	TATTAA	97	40	89	323	CACAGTG	12	ACAAAAACC
	V2u	P^a^	—	—	—	—	—	—	329	CACAGTG	12	ACAAAAACC
	V2v	F	—	—	—	—	40	92	329	CACAGTG	12	ACAAAAACC
IGLV3	V3a^^^	F	ATTTCCAT	38	TTTATA	65	52	84	303	CACAGTG	12	ACAAACCCT
	V3b^^^	F	ATTTCCAT	38	TTTATA	65	52	85	314	CACAGTG	12	ACAAAAACT
	V3c^^^	F	ATTTCCAT	38	TTTATA	65	52	85	317	CACAGTG	12	ACAAAAACC
	V3d^^^	F	ATTTCCAT	38	TTTATA	65	52	84	315	CACAGTG	12	ACAAAAACC
	V3e	P^a^	—	—	—	—	—	—	306	CACAGTG	12	ACAAAAACC
	V3f	P^a^	—	—	—	—	—	—	315	CACAGTG	12	ACAAAAACC

Fct functionality, F functional, P pseudogene, ORF open reading frame, R reverse strand,

^ V_L_ gene segments depicted in schematic diagram of the genomic loci,

^a^L–PART1 is missing;

^b^3′ truncation;

^c^1 nt deletion and frameshift at position 659 R; 2 nt deletion and frameshift from 637 R;

^d^1 nt deletion and frameshift at position 5176 R; 2 nt deletion and frameshift from 5154 R; ^e^1 nt deletion and frameshift at position 4359; 2 nt deletion and frameshift from 4381;

^f^1 nt deletion and frameshift at position 4666; 2 nt deletion and frameshift from 4678;

^g^1 nt deletion and frameshift at position 3685 R; 2 nt deletion and frameshift from 3673 R;

^h^1 nt insertion and frameshift at position 540; 1 nt deletion and frameshift at position 586; 1 nt deletion and frameshift at position 608;

^i^6 nt deletion and frameshift from 1896; 1 nt deletion and frameshift at position 1936; 2 nt deletion and frameshift from 1955;

^j^1 nt insertion and frameshift at position 439 R; 4 nt deletion and frameshift from 456 R;

^k^2 nt deletions in CDR1-IMGT and CDR2-IMGT regions and frameshift mutations at 1418 and 1487; 4 nt deletion and frameshift from 1429; 1 nt deletion and frameshift at position 1462;

^l^1st-CYS replaced by Ala.

**Table 2:** Torafugu J_L_ nucleotide and AA sequences with associated RSS.

*J*_*L*_ gene	Fct	J-Nonamer	Spacer	J-Heptamer	J region nt and AA sequences
J1a	F	GGTTTTTGT	ACGACCACTTGATGAGTTTGTAT	CACTGTG	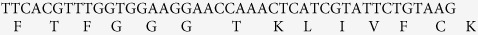
J1b	F	GGTTTTTGT	ACGACCACTTGATGAGTTTGTAT	CACTGTG	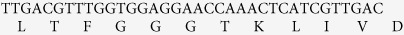
J1c	F	GGTTTTTGT	ACGACCACTTGATGAGTTTGTAT	CACTGTG	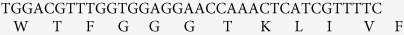
J2a	F	GGTTTTTGT	ACAGCTGTGTGTACAAACTGAAT	CACTGTG	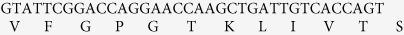
J2b	F	GGTTTTTGT	ACAGCTGTGTGTACAAACTGAAT	CACTGTG	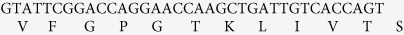
J2c	F	GGTTTTTGT	ACAGCTGTGTGTACAAACTGAAT	CACTGTG	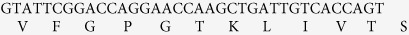
J2d	F	GGTTTTTGT	ACAGCTGTGTGTACAATCTGAAT	CACTGTG	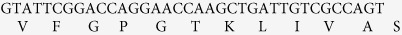
J2e	F	—	—	CACTGTG	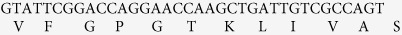
J2f	F	GGTTTTTGT	ACAGCTGTGTGTACAAACTGAAT	CACTGTG	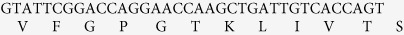
J2g	F	GGTTTTTGT	ACAGCTGTGTGTACAAACTGAAT	CACTGTG	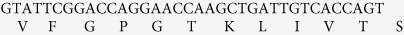
J2h	F	GGTTTTTGT	ACAGCTGTGTGTACAAACTGAAT	CACTGTG	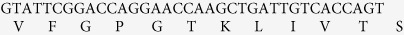
J3a	F	GGTTTTTGT	ACGACCACTTGATGAGTTTGTAT	CACTGTG	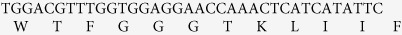
J3b	F	GGTTTTTGT	ACGACCACTTGATGAGTTTGTAT	CACTGTG	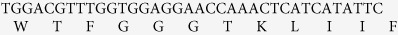

Fct functionality, F functional.
