# Supplementary material for: Immunoglobulin light chain (IGL) genes in torafugu: Genomic organization and identification of a third teleost IGL isotype
Source: Sci Rep. 2017 Jan 18;7:40416. doi: 10.1038/srep40416 (PMC5241823; doi:10.1038/srep40416)
Supplement: Supplementary Information [file srep40416-s2.pdf]

Immunoglobulin light chain (IGL) genes in torafugu: Genomic organization and identification of a third teleost IGL isotype

Xi Fu[1,2], Fengjun Zhang[2], Shugo Watabe[3], Shuichi Asakawa[2,*]

[1]State Key Laboratory of Biotherapy & Collaborative Innovation Center for Biotherapy, West China Hospital, Sichuan University, Chengdu 610041, China

[2]Department of Aquatic Bioscience, Graduate School of Agricultural and Life Sciences, The University of Tokyo, Bunkyo, Tokyo 113-8657, Japan

[3]School of Marine Bioscience, Kitasato University, Sagamihara, Kanagawa 252-0373, Japan

Author contributions statement

S.A. conceived of and planned the study; X.F. analyzed data and wrote the manuscript; F.J.Z. performed the Southern blot experiment; S.W. supervised the study.

[*] Correspondence should be addressed to S.A. (asakawa@mail.ecc.u-tokyo.ac.jp)

The PDF file includes:

Fig. S1. ClustalX amino acid sequence alignment of the J_L segments of torafugu.
Table S1. Identification of V_L, J_L, C_L gene segments in torafugu ESTs coding for IGL.
Table S2. Primers used for probes of Southern Blot.

```
         ....|....| ..
             5    10
J1b      LTFGGGTKLI VD
J1a      F......... .F
J1c      W......... .F

J3a      W......... IF
J3b      W......... IF

J2a      VFGP.TKLIV T.
J2b      VFGP.TKLIV T.
J2d      VFGP.TKLIV A.
J2e      VFGP.TKLIV T.
J2c      VFGP.TKLIV T.
J2f      VFGP.TKLIV T.
J2g      VFGP.TKLIV T.
J2h      VFGP.TKLIV T.
```

Figure S1. ClustalX amino acid sequence alignment of the J_L segments of torafugu. Sequences are grouped into L1, L2, and L3 isotypes. Residues identical to the first J_L are denoted by dots.

Table S1 Identification of V_L, J_L, C_L gene segments in torafugu ESTs coding for IGL

EST	V_L	J_L	C_L
BU807888	IGLV1 sequences^	J1b*	C1c*
AL834643	IGLV1 sequences	J1b	L1 C
AL839526	IGLV1 sequences	Stop codon	NA
CA330233	IGLV3 sequences	J1b	Partial L1 C
AL835462	IGLV3 sequences	J1b	Partial L1 C
AL835814	NA	J1a	C3a/C3c
CA330333	NA	L2 J	L2 C
CA330247	NA	L2 J	L2 C
AL835572	NA	Partial L2 J	L2 C
AL835410	NA	L2 J except J2d	L2 C
AL835785	V2k	L2 J except J2d	L2 C
AL834874	NA	L2 J except J2d	L2 C
AL835992	NA	L2 J except J2d	L2 C
CA330627	NA	NA	L2 C
CA845761	NA	L2 J except J2d	L2 C

^ More than one V_L, J_L or C_L gene segment can be assigned due to they present equal homology with certain EST sequence

* Segments depicted in schematic diagram of the genomic loci

Table S2 Primers used for probes of Southern Blot

Primer ID	Sequence: 5′- 3′
L1Cf	GCGTCCCACCCTGACCGTCC
L1Cr	GAGGGTCCAGGGTTTGAGTG
L2Cf	TCCAGCCTCCCTCCTCCTGTCCTGAC
L2Cr	CTTCACACTCGGACTTCTTGATGC
L3Cf	ACCATGACCAGACCCTCCGTC
L3Cr	CCTGAGGATGGACGGGCTCTG

Supplementary Dataset File: Annotation details of torafugu IGL genes.
