# Supplementary material for: Immunoglobulin light chain (IGL) genes in torafugu: Genomic organization and identification of a third teleost IGL isotype
Source: Sci Rep. 2017 Jan 18;7:40416. doi: 10.1038/srep40416 (PMC5241823; doi:10.1038/srep40416)
Supplement: Supplementary Dataset 1 [file Supplementary Dataset File_SREP-15-27950A.pdf › Supplementary Dataset File_SREP-15-27950A.pdf]

Immunoglobulin light chain (IGL) genes in torafugu: Genomic organization and identification of a third teleost IGL isotype

Xi Fu, Fengjun Zhang, Shugo Watabe, Shuichi Asakawa*
* Correspondence should be addressed to S.A. (asakawa@mail.ecc.u-tokyo.ac.jp)

```
LOCUS       KU350660                 2781798 bp    DNA     linear
DEFINITION  scaffold_10
ACCESSION   KU350660
SOURCE      Takifugu rubripes (Fugu rubripes)
  ORGANISM  Takifugu rubripes
            Eukaryota; Metazoa; Chordata; Craniata; Vertebrata;
Euteleostomi;
            Actinopterygii; Neopterygii; Teleostei; Neoteleostei;
            Acanthomorphata; Eupercaria; Tetraodontiformes;
Tetradontoidea;
            Tetraodontidae; Takifugu.

FEATURES             Location/Qualifiers
     source          1..2781798
                     /organism="Takifugu rubripes"
                     /mol_type="genomic DNA"
                     /db_xref="taxon:31033"
     regulatory      2769583..2769590
                     /regulatory_class="promoter"
     regulatory      2769618..2769623
                     /regulatory_class="TATA_box"
     misc_feature    2769686..2769725
                     /note="leader"
     V_segment       2769863..2770162
                     /gene="VL"
     misc_feature    2770167..2770194
                     /note="RSS"
     regulatory      2772451..2772458
                     /regulatory_class="promoter"
     regulatory      2772486..2772491
                     /regulatory_class="TATA_box"
     misc_feature    2772556..2772595
                     /note="leader"
     V_segment       2772729..2772896
                     /gene="VL"
     misc_feature    2781060..2781099
                     /note="leader"
     V_segment       2781237..2781527
                     /gene="VL"
     misc_feature    2781541..2781568
                     /note="RSS"
BASE COUNT   743096 a 617434 c 620919 g 727781 t  72568 others
ORIGIN
        1 ttggcacacg gcctggcagg tggaggaggg gcaccaggtc cggcacgggt
ccaacagcac
       61 ctctgacaga acagggagac gagcaaaatc ttaaacagtc gcccatctgt
ttatcaagct
```

121 gaaggaaacg gtgggattct ctttgcggga gcatcttaga cacgttttct
ctgggcttct
181 ccgagtttat gacactaaat tcagatttca gcatcttcta aagatcctca
ctcttaatct
241 taaatgtcag atatgggatg tcagtctggg tccctggcag ttctggacgg
tcttccaggc
301 tcttatctgt tgggagctgg tgttaagtcg caggatcccg ttacagccca
gagaggtgtt
361 gagctggagc acctgtcaca gaacaccgcc ttcctgcctg agtcttagcc
gtccgccgct
421 gatgccagat ctatatttta aactacaagc ccaacaggcc cctctgagtc
agccgctcgt
481 catcgacacc tcgtcactaa cgaagacgga ggcgcgccga gccgacgagc
ttcgggctgc
541 tgagcagaga acccacgctg ggaatcgatg gtgaaacacg tcgggagctg
acgccatcgc
601 ttccaatgcg cgaacgccga cacctcgttc aggacggcgg cgaggagctg
cgtctgctcc
661 tgggctgact cacctcgttc aaactggagc ttcttatatc tctgcatcac
ctctgtggcc
721 accatgcact cgatctatca gggaggagag agacacgagg acatgaaagc
ttcagcacgg
781 ctgcaactag agccaactgc tcatcgatct tctcctccag ctgccaggtg
ttaaaggtga
841 atgcgtagga aaatgtcctg ctgaatggag aatgtaaaca ggtagccact
tccagccctg
901 ctgtcacaaa caggccggct ggcaggaggc cgagtcccat gatcgatctt
ccttaagttc
961 tctgacattt cagggaagct tcgaatccat taaagccgcc tgaaagcgct
cagattgacc
1021 gtcacaaaga cggcgggtcc attttagggt gataaaacag catttactag
catcacaaca
1081 cagtatctcc cttttcctct atcaatgaac tcatcagaaa caaacaagaa
caaacttctg
1141 gttggttttt tccgaacatc tacctcattt tcctgcaggt gtcctctttt
gggacaggca
1201 gagtcgggac agctaatcgc agtctccagc ccttctttaa tcagaagttc
cacatactgc
1261 ttcaggcact gcggggggga tgaagacaca tgtggaatta gcttatgcta
acaggaaaac
1321 acacagcacg gatagaggaa cgccaggaac gcctcctcac cagagtacag
aagacgcatt
1381 ggcactgcgt gatggtggtc atctgctcca gagggaactc tccaaggcac
agcttacagg
1441 acaccagggg gtccatggcc agctccctgt tgggacggta gcgcgccgtg
gtcatcttca
1501 ccgccgctga gctggcagac agacagacag acgggttgtg agcgccacga
atgaagccat
1561 gagcgagaga gacacttcct gctccgcgga agatggagaa taaagtccgg
aatgtgggaa
1621 tctgctttta taaaccagcc tgctggacct ctcaggtcgt tccactcatc
tttctgaggg
1681 aaaaaccact agaaaagtgc taaaactgcg gtagcggcgg cccgatccat
ctcagctcgc

1741 tcctctctgc gtcaccgggg ctcttttgtg ccagacggtt attttaggcc gtctcctcgc
1801 accccgtcct tccgctccga gagcagagag cacccactcg ggcccgtacg cgagacgtca
1861 gcgccgccga gctctccggc tgctgcctag caacgggaag gagggaaatt atggaagaga
1921 agggaaaaaa aaaaaaaagt gctgaaaaaa ataaagagga gagattgagc gccacaggcg
1981 gggggacagt aacagcaggg ggcggagaca gctctcctca catccccaga cctgtgcgcc
2041 gccaacatgt gacagctgag aggaaccgag ggcgggaggg gtgggggggc acctttgacc
2101 aacattacat ttcagcaaat acaggaacga tagctcgcgc ccctccccca cacattagca
2161 acaggagcta actcagggtg aggaaagcgg cacccatcca tcactctggc cagccggccc
2221 ccagcggggg ggggggcaag gtgctgaaca tggaaacaga agcataaacg gcgatttgtt
2281 atggagatgg agctgaggga gcctgacctc tgaaccactc tgccgtgagc gccgcgcctc
2341 tgcggaggca tttgcatcac ctgtggaggt ccagctgcac gggtcaggct tgtttgcata
2401 gctgagggca aaggtcaggc agacaaacaa atgtgggttg aggacgcagg acgtcgacgc
2461 cctgacctct ggtcatgtgg acgccaccag gagacttcag caacaagcac tcggggctgc
2521 agccgcacct ctgtggcagg tgaggactcc cccccccctt cagaaccact ggaaccagcc
2581 tgttcattta ttccgctcta agctccttta gacccgctgc caaaaccacc tggagctgaa
2641 aactcacaca acaccgagag agaccggcta attcattcta atctgccctg ccggggaggg
2701 gggggggggg gggagaggaa aggaaaagag aagaaaggag gggaggagag aggagaggaa
2761 aggaggagag gggggaagag gaggggagaa aaggggaaag gaaaggaggg gaggagaaaa
2821 ggggagagga aaggatagga aaggagaggg gaagggggga annnnnnnnn nnnnnnnnnn
2881 nnnnnnnnnn nnnnnnnnnn nnnnnnnnnn nnnnnnnnnn nnnnnnnnnn nnnnnnnnnn
2941 nnnnnnnnnn nnnnnnnnnn nnnnnnnnnn nnnnnnnnnn nnnnnnnnnn nnnnnnnnnn
3001 nnnnnnnnnn nnnnnnnnnn nnnnnnnnnn nnnnnnnnnn nnnnnnnnnn nnnnnnnnnn
3061 nnnnnnnnnn nnnnnnnnnn nnnnnnnnnn nnnnnnnnnn nnnnnnnnnn nnnnnnnnnn
3121 nnnnnnnnnn nnnnnnnnnn nnnnnnnnnn nnnnnnnnnn nnnnnnnnnn nnnnnnnnnn
3181 nnnnnnnnnn nnnnnnnnnn nnnnnnnnnn nnnnnnnnnn nnnnnnnnnn nnnnnnnnnn
3241 nnnnnnnnnn nnnnnnnnnn nnnnnnnnnn nnnnnnnnnn nnnnnnnnnn nnnnnnnnnn
3301 nnnnnnnnnn nnnnnnnnnn nnnnnnnnnn nnnnnnnnnn nnnnnnnnnn nnngggagga

3361 gaggagtgga gaggagagga gaagggagga gaggagaggg agagggagag
gagaggagag
3421 gaaaatgagc tttctaggct aaaggagaga cagtgtgcgg tggcgtcccc
ctgctgcatt
3481 attaaccacg tgtccattat cactgtggaa cacgtgggtc tatgttcagt
ccaggtcctc
3541 tccaggacca gcctggccct gtgaacctgg accggtggat ccaggctttc
agacgagagg
3601 tcacacatca acctgcaccg aagaggagaa aatacagcca ttatctgctg
gctgtgcacg
3661 tttgtgtcag gcaggctgga ggctgggcct ggcactgccc ccccccagag
agacaacacg
3721 caggggaaaa tggacctgat caccattata tcctgagcag ccacggaact
gccagatgtg
3781 aataataaat ttgtgcacgt gtgcgctcgc tcgttccagc tgtggcgaag
caggtgagca
3841 gacaaagcgc ggcgcctccg acaccagagg gaccagaaga gtccaggagc
agcggcaacg
3901 tgcatcgctc cgctaatatg aaacatcagc acgtgtctcc aacagagccg
ccgttcctcc
3961 gctcaccttt agctccattc tgctggatta tataatccca tatcaaagca
ttgcgtaagg
4021 ggctatttat gtagctcacc taatgtgaga caaatgtggg ggaaagccgt
tagccactcc
4081 gcgcgctgct agctgtcaca tttccatgca gcacgtggag acggagtcct
cgccgtggag
4141 gagcgtgcca ggaggagcgt gccaggagga gcgtgccagg aagagcggtg
gtggtgagga
4201 gcaggaggag aaacagctga ggtgatgtta ttctacctga agcttgggcc
cagccggcac
4261 cgcgcacctt cagatcgggt tattgtgacc tacagctaaa ccatcgcaga
cagcctgggg
4321 gacacgctcc tcccagctgg tccgaccagt ttgatcttgt gatccacctc
aaaaacaacc
4381 aggggaatca aataatcaaa tatggctgag cacacactcc acgggcccag
ctaacagcac
4441 gctccactgt ggagggacgg agggatggag agagagggag aaagggaggg
atggagggat
4501 ggagagaggg agggatggag agagggtgag ggagagaggg agagatggag
ggatggagag
4561 agggtgaggg agagagggag agatggaggg atggagagag ggagggatgg
agagagggag
4621 ggatggagag ggtgagggag agagggagag atggagagag ggagagatgg
agggatggag
4681 agagggaggg atggagagag ggagggatgg agagggtgag ggagagaggg
agagatggag
4741 agatggagag agggagggat ggagagaggc agggatggag agagggagag
atggagagag
4801 ggagggatgg agagagggag ggatggagag agggagaggt gggtggtcag
ctggaaccaa
4861 tgcctcaaaa gtgggaatac tgggaacatg tcacaggaag ctcatggaac
tatttttcac
4921 cataatgtgt gtgagtgaag gattagcagt gttctgtgta tcagacacgt
tccaggtgct

4981 gttgtctgtc ccgtcccgtc ccgtcccgtc cccactattg aagcagtaaa agactgcccc
5041 cttttccacc ctctggtgcc tcttttttta cagcccacag ttatttctta tcagctcgtc
5101 gtgaacgttg agactgtggg acggaccctc ggcccacgca tgggtcacac ggatgatgtc
5161 atcgataaaa gtcggatcag cttgggacac attctgtctt cgggcagatt ctaaagatta
5221 cattacgaca ttcttgacat ttccgtacgt atctgatacc agtgagctca taagataatt
5281 aaaacttata ctcacacaca cacacacaca cacacaaacg tacttttgtg agatctatga
5341 tccctctatg atccctctct gacagcatcc ctaagacaac atcttaagtt tgactgagga
5401 ccagacagca tgatctgcac aaagaacaca cacacaccct atttttagaa tgtgagaggt
5461 catgtgagag actctgtgtg tgtgtgtgtg tgtgtgtgtg tgtgtgtgtg tgtgtgtgtg
5521 tgtgtgagaa cggtggatgg ggacagtaac catctgtcct tgacgtgggg tgatacgact
5581 tatttttggc cctcaaggta ttacacaatc acacagatgt attttagcac catgacctga
5641 atgtgtgtgt gtgtgtgtgt gtgtgtgtgt gtgtgtgtgg gtgagtgtgt gttgcccata
5701 acaatccaaa gtcccctcca caggctggac caggcagtgg ccggggtaag tggggcagaa
5761 ggccggactg cagtctcgct ggtcccctgc cactgttgct gcaacaatga gtgacagatc
5821 tgacccagtt gatccacaac aacaaagttc tggttctctg gagacagtaa tgacacacac
5881 agtgtgttta aagccctatt tgctaacttg tctggtcctg ctgcaccaaa ccacaacacg
5941 gttccggcaa aggtgtgttc ctgggcgtga cccgcccacc tcggcactgg atgttgcgca
6001 atgcaacttc ctcgtgtagt tgagtttctc tcccaacctg taattaaatc tggaatgttc
6061 ttccaggctt tccggccgtg gcaggtctgc tgttctggtt gcacaagcga acccggttgt
6121 gacatatcga gtgaggaaac ctgcagattc tgtccaacac gagcccgtcg ctgtagtggg
6181 ccgcagctct cagaagctct gttctccagt ttcttcttct tcttcttcga acgttaccgt
6241 caaaggaaaa acctccctga aaatcctgaa aaagcgcctt ttcccagcag cttccttggc
6301 tgcaaacgcg tttggacgcc gtcctcttca tcatgttacg gtgtttttgt cacttaaagc
6361 ctgcggctaa agttcttgtg tacttttatc caaaaccgga atcaaacaaa tcaaacctgg
6421 cagaactcac cgtttgggaa tgttgtctgt tagagctgct agctcaattt ggaccgcttc
6481 ctccttcctg tgccagtttt ccccaccttt gaagtaagtt tcgttagaga tcccgccgag
6541 atcccgtcct gcgtcagcgt gtgagtcctg ctggcttgtt tgtccagggt ggacgactgg

6601 attttgccaa aaagacacct attttcctca acggtaaaca gactcaggag
cggatggaaa
6661 caatggacgc attcacggga tgaaacaatt tgaatttgag ctaatttcag
gctttttgga
6721 atttaaaagg agccgcaggc tgtttccata gtgggaaaca tctgcaaata
aatatgttgg
6781 cgcaccatgg aaccaactat agttgctttt tctaactgaa cagcttttga
aaacctctga
6841 aatcctttat tcccattcta aggacgctct gctgctgtgg gcggccaccg
cggggaccac
6901 tggcccggtc ttcactgggt tttcatcatc taaaggagac agcaccagtt
agcatggtcc
6961 cagagtgtcg cgcccggagc ctggaggaga gagacggtgc agcagagacc
tgcagaagtg
7021 cacatctgtg cacgttaaga gccaatcaga ctgtctccaa gtgagaaggc
acgtccgggt
7081 tcattacagc gtctgagacg ctgtccactc acggcgccga gcaatccagt
tttcatggtc
7141 acgggggggg gggggctcca gggctactct caccgctgtg cagccaaact
attcccaagc
7201 aatgttagga atcacaaggc cgggagcaag gtccatttaa aagtgattaa
ctggtgtcgt
7261 cccgggaccg cgtgcactca atttcctcgt tcaatctgca gccatgccgg
tgccaacaca
7321 tcatcatcat catcctccta tacaataacg catcctgtct tctcctgccc
ccggactcac
7381 ggagggaaac aaggaggaag cctaaaaggt accttaggaa atgcgccagc
gtcccatcgc
7441 tggctctgct ggtgacgtca cggtgctcat ggttcgatgt gctcattaga
atttataagc
7501 ccatttagag gcttttgggt ccccgagtaa tgcgatcctc tttgagccag
agcagcgtta
7561 gcgcctaaat gaggcgctcg ctatcaggga cgtgacataa tgttacatat
tagaggaaat
7621 cagacaataa taagcagggg aggaaatatc agaagcactg attattctat
tgtttccaga
7681 gctgcaggct cgttatcctc aacgctgcgg aggaaagagc aactaaaacc
gagaccggca
7741 gagtcataat cacacctctt catttattga ttaaagtggc atcatgtgac
cagacccaaa
7801 agaaatccac ttgtattaag aataaatagg aacaaaagtc tgcttaccgt
tgacttgtga
7861 agaggcagaa cgaggatgac agcctccgtt tgagtggagt aaaggaaagt
cgctaacccc
7921 aagtgactaa aaacgtgaga acgtgacgga aacacgaccc gctcgactga
caggcgctcg
7981 ggtccatctg agtgggtgga aagacccaag acgtgctgct tcctgacttt
gttttgcagc
8041 ggctaacttt agcgcgacgg ccccggagga gcgacagcaa cactccaagt
gtcccattca
8101 ggttctgagc tccggactca aaatgaaaac tttactgttg ctgctcgttt
ctgtgggaat
8161 caaatttgtt tacacattct tgctaagcgg tggagccagt cagcagatcc
tgtaaccagt

8221 ccaaacggag cgagtgagtt cgaggccgaa aattgaggtc agctatgaaa aattccaaat
8281 ggctctcctt gaaattacaa tgttatcttt atttcaggct acgcaacaac gaatatcatg
8341 acatttctgc ttgacttgct aagaaagaag aataaaaaca agccaaacca cgtcggaggc
8401 agtcaaacat catagaataa tgtgacgagc gtgatgctaa ccaaaacgca gaggctagct
8461 ctgacatatt aaaatcatta tcaggaggcc attttggtag cttcttaaac actccctcct
8521 cttcctcaca ccaaaacctg aacaactagg aagtgacacc acccctaaca ctgacaacac
8581 caggctgaaa tggcatttaa gctgttgcgt aatctttggc tcaggcagac ggcaaacccc
8641 ggagacagat gacacacaca aatcactcct ctagatggac caatgtcagc ggaataaaag
8701 ggcaattcaa caaaaggtgc agtgcagcaa cacacttcag atcacagaca gcacgcgacc
8761 tttagaggag acgctccctt aataactaca cgctcacgtc tctctggttt ggtccccgca
8821 tgcctgcaag acaaaaaact tttcatttcc gtgttaaccc ccgcaaacgt ggttgctaag
8881 cgacggctaa aatgctcatc agatcgcgac aggccgtcga aaacaacaag actttagaag
8941 tttgccttca ctgtgttatc aagccgcagc atctgcgggt ggtcattgac ttaagcagga
9001 atttagtccg acacatctgg gctcttatca caagtaactg cgagaatgtg gaaaagaact
9061 ggaggaatcc caactggaga aactggagcg cgcacacaca cacacacaga tacacacaca
9121 cggcggtgtt gtgtctgccg tccaggcccc gagtgtggag aatgtgtctt tattttcaag
9181 tgaagaagtg cttctggggg agtgacgcat cggcgaggac gggagttcac aggcgctctg
9241 taggtggacg ctgctcacct ccagccacca gttcaggggc tactggtgcg tgcaaatctc
9301 agctccaggg tcaatatttg tgtctgactt tggcagcaga accgcgatgg gatggctggt
9361 cactgctagg caaccctttg cgatcctacc ctgggagcag tttacagttt tgtcgctata
9421 gaaaccactt ttttttccca gtttctgact aatggagagg cagtttgacg cgtttatccc
9481 acccccccg ggagaggggg ggggggttac actggaacgc ctgcaataat actggaagca
9541 tgtcacagga gagagaatca atggcgcaga atgaagaggt tattagcata gcaggcttga
9601 tcctgtaggc taaatgtaac gttgaaaaag gcccttaaac gcaccatgac gtgacggtga
9661 cacgggtaaa aacccaaaaa cgtacgtgca cgatcccttt actctcggca tgggagcaca
9721 aacccacact cctcatggtg gcctggtttc agaactatgc ctataatctg acaggaaccg
9781 gggggggggt cgctgcttaa aatggaaatg gcgggtcagc tggtgacccc tgagggctca

9841 gaggagccgt ccgctctgaa aacacgggag gtttcgagct cagatgcaac gcacggagaa
9901 aggagcgcca cacagcccgc agcagccacc tgacagcaca gatgcgtcta aacacgcacg
9961 cacacggaca catgaacacg tctgtcagca acacaacctg cagtgcacac caagacgttt
10021 cactgcgaca gccgcccctc ctgcacgcag ggagccgtac ctggctggtg ggatgacgcc
10081 gtgctggtgg tggaaccccg gggaaggctg agcagatgat ctcccgaatt ggattaacga
10141 agcggcggcc aggcggtcag gcggagcggg aagacggctg ggaaggggtg atgcccatgg
10201 cggctaccgt gtccctccag cagcggcggg gtggaaacat ttgacctgct cctgtcgccc
10261 acagcgcaaa tgatcggacc ccggtggaac acaaacaccg acgaccatcg gcgaccgcgg
10321 gaggggggt gtttttgctc gacagcgggg ttcggtcctc ctccctcgcc gccgacttcc
10381 gcgagtgcgc cgcgtccagg cgaggttcgg cgactaatgt gaagctcgtc ctcgctgcgg
10441 aggaatccgg aggcggtgtg tttacctctg agcggctcct gctgctgtgc gcggtcgcat
10501 atcagctgct gcgcactgga gagacgccat ttgttccttc tgctgctccg cttcaagcgg
10561 agcagagcgg agcgtcactt gaacaacagc gccccctgct gcccggagcc gccactgcgc
10621 cgctctgaga cggtcatttt aaacacgtct tatacaaaag tacatatata tatatagtat
10681 tttcttttct accagtattc cattcttatt aaaatttaac atccagcccg aagatctttt
10741 ttcctttta ttgtgatcag tgatttgtcc aaaaatggat ccagacagtg atcaatgatc
10801 tcaagaggca gagaaatata aacaagggtg atctgcactt aaaatcgatt tgaaggaaaa
10861 gacaattaaa agagggattt gtaaaagcat aataaaatgc agtatttaaa tcataacaat
10921 tacactaata ataatagcat agctcaataa tagctctgac taaatatttt atttgcattt
10981 ttaatcgagt agttaaaaac tacatttgtg ccctggaacg actgctacac tgtgatcaaa
11041 tggatcacaa ctaaaactta atggaaaggg ggagctgtga acttatccag acaccccgt
11101 gactccctgt aacagaaaat caatgtaaat aagccatttt ttaggttcac ttcaagtaaa
11161 actttatgta tgacctcagg aatatactta tttagagtga aatcaaatat ttttttaaca
11221 caaacgcgac caacatattt gcattttaat gctgactcac caacaaagta aacaggaact
11281 gaataggata ttaatgagag attactattc acatgactca acgaagccac gccccctaca
11341 agggttccgg gataacaaca acactggtac attttaaatc ggtataatga caaaaatata
11401 catcttttta ataataaaaa aacttcttag ataattcaaa tgcagaaaac ttttgcacta

11461 gtgagcaaca gcgcttcaac tatcagcaac aactaaaaag aaccaaaagt gcatcaaagg
11521 taatcgcaac acagaccctt catgtttttc aatatttttg attcctcggc attttgacaa
11581 atggtgtcgc ggatgttttc accactccag cttcatgtcg gctttgctcc acacatattt
11641 ccctcctctt ctcagaaaca tcttttcatc aaagccactg aagagaatgg cttctctcac
11701 acccacatca agaatggact tggacgggtc tttccggccc tctcgacagt ccaggaaacg
11761 catctgggat aaagaacaaa aaaaacaaac agttaaatgg acgcaggaag agatatctga
11821 ggtcaataag gtcgttctgc cgtgaagaca aatataatac ttacatattc atccaagatc
11881 aaataggaat ttctcatctg gaaaaaagaa aaacacattt taaaatagaa gaacagaaga
11941 aatttttaca aatgtctatt tacccaagac aacaagaatg aacacgtgta gagttaaaat
12001 ttttaaattt ccaattaaag tctcaaaata cagagctaaa atagcaaaat gtctgtaaaa
12061 gtgggaactt taacctgaca tcatgtccag ttaaagtggc aaaatgtgca tttagcatta
12121 cacccatgtt tacacccatg tttacaccca tgtttacacc ccgtcaccat ggtgacaggg
12181 tgtaaatgat tagtgtgaat ttctcaacat ctccgctgag gtcacctgca acattccatc
12241 tgacgcccac atcaacatgc caaagtttgc ttgcgcacct tctcattgga ctctggaacc
12301 agacaggtga cgctgctgtg cctctccagg aagtcctgga actgcaggtc tctgatgacg
12361 aacctctccg cctctctcag ggcttcctct cctgcgttct cgccgtcaat cagcaaacac
12421 tggaacacct ggagacacac gcatgagggt caagacgggg ggggtcatac ccggacctcc
12481 gtgtcgcctg tccctgcgct caccttccag cggaccgggt ccagcttgtt gatgttctcc
12541 tgcatgtcct cgtccacgtt gaaggtgttg accaccgtgt tgattttgaa cgccactttg
12601 taccgcgcgc accagccgcg gatcttgtaa aggttctcga tgtggctctt cttcccctga
12661 gctctgccga tcagctggtt ggtgtcttca ttgaaactgt cacaggagat ggccaggatg
12721 tccagatagt cacctgatgg gacagaaaga acgttttgat tgagccaaag actttcttcc
12781 cctttgggct cgaaaaaaaa aaaaaaggag ttgcttcctt ggataacatc ttaccgtact
12841 tctggaacca cctttcctga atcatgctgc cattgctgac aatgctgaca ctcgggagtt
12901 ggagatcctg tttgcaaaac tgaaccagtt tgccgaggaa gtctcctttt tcgtgcagga
12961 agggctcccc tcctgagaag ttgatcttct ccatgcctgt atcaacacaa aggtacactg
13021 aagagctcca tccacgcccc caaacagccg tgtttgtgct ctcctggggt ttatttacct

13081 gcatccttca ggagttcgag tcccctcttc gcctcctcca ggggcagaac gaaggaggtt
13141 tttgcagtgt ggaagcagaa gccgcacttg tagttgcatt tgcgtgtgaa gtgatagttg
13201 acgctcgtcg ggacggtggc agtttgctct gctttgccgt ctggaagaat cgtgctgtcg
13261 tcggacggcc gccggacgtt taaaaagacg ctgctccaaa ggttgcacag agttctgatg
13321 cagaactgca gcagcagaat cggagacacg atcacggagg agaaatgcat cttgctgacc
13381 agagatggac ctggatctgt tttggatgtg cactccttat ataaaggtcc cacccgcctg
13441 ggcggtttgt tgacatgaaa tgaatgctta accggtgatc agctgtttct gttcccaaac
13501 atcgaaaccg aaactcaagc tcatgactca tacggctcca aaagagataa ctggacttag
13561 ccagaaacca actactgact cattgtttaa aaggcacacg ttgcagtatt cctgaaggaa
13621 ttttacgact ctactttatg actcaaggtc taagttcccg ctaagtttcg ctttcgtttc
13681 tccgcctttc aactcgcttt cctttccccg cctttcaact cgctttcctt tccccgcctt
13741 cggatttcag gtgccgaaac ttatcgcagc cgatgcagct gatggctcga cgcaccatgt
13801 ctgtcgtttc ccagtggtcc tcccgcatct tctcggtgga gctggatgga gcagagtcct
13861 actttgcgat ccaagaaacg cgcaaaggcg acgttttacc ggaggcgttc aggagagcga
13921 accgggacag gttctactcc gtcctggtct cctgcagcga cagactcaga caagtcaagt
13981 tccacgcaga tctcgaggag aagcttttga aagctctccc cgcaggctgt gccgtggacc
14041 ccatgtcctc gtttttgccc ggtaccaggg gctctctcat caggggctac tttttgaagg
14101 ccagctcaga ggatccgtcc ttttcagacc gacttttgcg agacttgata aaggacgatc
14161 cagtgcttgt gtgctcgtac gtaaaatgcg aggacggcgg gacgtggacc cagaacctgt
14221 ggccagacgc gcacagtgag atgattaaaa cgttttatgt ggtccactct gaagcgccaa
14281 aggttcaccc atctactctc aacatcatca actcagacgt gttctacagc ttcgaagagg
14341 cccgcgaagt tctgaaggag gttaaagaca tttaatcttg tgtgatttta ctgtttacat
14401 gcatttatgc gaaacgacgc catcgtgcgt tagacgttgg tactgcaata catgctaagc
14461 gatccaatgg gagattcgat tattccattt tctccctgta agtgccaggt agttcgcagt
14521 ggcatgcgtg cgagacatgt gtatcacgtg actccctctc gcgtaccctc ataacaacat
14581 gtctgttctg cagagcagtg acatcatccc agaggcagcg tctgttctgg agctgctgcc
14641 gagcagagcc gggcccagag gcagaccaga ctttccagtt attgtcattg agggcctgga

14701 tgccacaggt taatgccatt accttacaaa taaataaggg acgggtttga
tgtatagatg
14761 acttgctaaa acgaaggccg gctgttctgt gctgaagcta tttgtctgct
ggggatcttc
14821 tgctaatgga atatcaagtg tcatgacctc agaagcatta cctcatggtg
gaagattctg
14881 attgtctagc gctcttgcag actcaatgac tacgacggca agtctttatg
gtttttttg
14941 gtttgttctc ttctcccatt gcaggtaaaa ccactctgac ggagtccctg
aagggggctc
15001 tgggcgccgc acttctgcgt tccccaccgc agtgcctgtc cccattcagg
gagcgcttcg
15061 atcaagaacc gcccctcatc cgcagggcct tctacgctct ggggaactac
atcacagcgg
15121 atcaagtcag tcaggaagcg tccaagggcc ctgtgattgt tgacaggtag
atgggtcaca
15181 ggtgtcgggt tagaattcca tctggccgta aggatgctca agatcgttgt
cctctgcctc
15241 caggttctgg cacagcacag cagcgtacgc catagccaca gccgtgggcg
gcccagtgtg
15301 caacctccct gacgagggtt ctgagctgta ccgctggccc agtgacctgc
ttgagcccac
15361 cctggtggtc ctgctcaccc ttgaccctga ggagaggaag aggaggttga
gggacagggg
15421 tctgattaag acagaggagg agcagaagct ggaccacaat cagctcttca
gactcaagtg
15481 agatgcctga agtgctttta caggttataa ttgcgcgttt aacgctggtc
cgcagaggaa
15541 gcgctgctga tatcgtgctg cggatggttt tctgtagctg aactgcagct
cacctcagca
15601 ctcatgaaca aacacgcttt gctgacggaa ccattatgcc gcacttataa
agggtaaaat
15661 ccaaacattt caagccttaa atcaatctca gcttctcatt gcatttcagt
taaaaaaaac
15721 ccaaaatcga ctcctttcac aaactcacct aaaataccaa aaacaaaagc
agctgtgcga
15781 cgggcagaaa aaggaggaaa acaagttctc ggtgagtgtg aaagcaccga
agcacagatg
15841 ggctacaaac tggcctgcat gtctcactgc agctcagcct tgctctgatc
catcaaacgc
15901 ttaacaatca attactgtcc cgtgggggag gctgctgatg aggaagaaca
ggtgattagc
15961 tgccaagctg ctgtgctcca ttaagctggt caaaactctc aggacctggc
ggcccagtta
16021 gccgccgcgc agacagcgga aggctacttc cattcatccc ttgtcctgat
ataatggcct
16081 ttccacacgg gccggccaga tgttgaaccg gcgcaattag aaataaagtc
tgccgctgag
16141 agagtttgca caacgggtca atctctttcc cgagggcgct tcttcatttc
tcctccagaa
16201 atggccgctc tcggcgccat ctgtgctctt tctcatgcta aattttccac
attttatcac
16261 tctgacaaaa ggctgagcca gcaaacgacc ctcgtagtcg tggggagatc
agatgaagtg

16321 tgacgcaatg atctctgttt taatgctgtg cccttatttg ccacacttgt tcctgtgtct
16381 ctgcagagtg gagcaggctt accgaaggat cgtcggtcca gtgtgtgtga ctgtggatgc
16441 gagcccttct gccgaccaag tgctccgaga agtgcagctt ttaattaaga ccaaatgcca
16501 cttgtaaaca gttcttcctc ttcccagctg tcggccacgc tcgaagcagc tgcagtaccc
16561 gtttttgacg ctaggtgcca gtaaggcttt atctttgtgg tactttttgc agacatgatt
16621 gtggagagga gcctctgctc tgatcactga ccgtctgtag ttcagcatat gagtgaaggt
16681 tgaatgtcta gaggcctgaa ttggaggcca cgttcagaaa tgcatattca tgttggcatc
16741 cgtgcgcgtg cacacacacg cacggctgtt tctaagaagt gctgttgtac gtttatggat
16801 tgtcctctgt cctctgtcca gacactaagg gaaaaaaaac acatttaaat gtaaaattaa
16861 gtaaaaaatt tgtttaaaaa catcttcaaa atggtttctt ttttaatgta tgatgcattc
16921 agggtctagg atgctacccc gactgtggaa cttttgggca cacacatgca attaagcact
16981 gtatttgttt atctttcgaa gacaaaccct aaatgcacac gcacgcctca cgacataaac
17041 tcgtgtccag gcctgcacgt tcactcccag cgtgcagcgt aactcatttc tatataggag
17101 gaagtgtata tttggccttt gtgatgggaa gattaattgg gggttttagt aattatgagc
17161 ctggcaaagc ttcaggctgg ggaagacaga gtgacccatc tcttacaccg tgatgcacca
17221 gcagagcgcc atccactcca gaccctattc atatggcaat taatggagcg ctgtatatca
17281 atgtcactcc ggcgcttccc cgcataagcg gcgagattac tcctgctacc aattacagcc
17341 atccggtgtt ttaggaggag cagaggtgcc gccgatgcaa atgctaccgt cacgtgtaat
17401 caagctagag cggggggcac cggtacgttt accaaagccc gcgctaagat ggaccccccga
17461 ttgaggtaag tatccgaggg catctttgca gggccctcgg tggtgtggtg acatagcgat
17521 gtgctgaggg atggaaagtg tgttctcggg cccctgtggg actttgttca ctgtgaatac
17581 agtgggctct ttctcaggag agggcagaat ttatgaagcg ggagcgctaa gcaattagcc
17641 attactccat gcgtcgggag tggacgtaaa gttttgcggc cacgtggcgt cgggcgtgtt
17701 tccatataag atgtttttct tggcaacata aggtggtgat atgccattac tggccctcaa
17761 caggactcta atatgacatt aatggcccgt aatcatagta cattacacat aacgccatgg
17821 atgtatagct aatagctttt tcccctcaca cgtgaagcat ttatggggtt gtttttgtca
17881 gataaactct gagtaagtca gatccttgca tgatactcgg tgaatccgag gcatttccag

17941 gaagaattac gggttagccg catcctgctt ctaatctgca aacccgctga agtctggaga
18001 tgaaagtctg cccacattcc acgggaaaac ccaggccgct cgctgggaag agcgggcgga
18061 gcgcaggcgc tctgcgcagg catgtccgcg ttaggctgca ccgcacgcgc gcacctcgct
18121 ctgtatgggg ccacttcgcg ctcctcaagt tgcgctcctc aacttcaata tttcatcttc
18181 acaacagaga agcggcagca gagaggagga aaaaccctcc gcgaaataat taaagatcca
18241 gcagggatgc ggcagcacag cagccgccgt cccatcgtaa gtaatagatc aaatgaaagc
18301 tgtccctcag atcattaata atgagcgcgg ggatggagac tgcggggcgg agggtgggag
18361 aggagcggga ggccacgggc tggtacagtt tgggggcag ggagaagggg catcagggag
18421 gcacaaggtg ggtttttcat gtgtgtgtct ttggagtgtg ttctgtgggg ggcggggctc
18481 cgaatgggag tctgggtcct ctggcaaaga ccccagtggg cctcacttgg cagccacatg
18541 aagcggcatg gcaagaggag tcatgcatat gatttggcac cgagcggctg ccaaaccctt
18601 cagacccgac agcgccgtcc tggtacgccc gccgtccagt aaacaacacg ttacattacc
18661 gacgcgcagg ttagcgggag ctaataatgg ccccttcaat cagcggggcc ctcgcagcat
18721 cggcccggct cctgagactg gcagggatgg gctgtctggg gttttggggg aggagggaac
18781 aagaggaaga cgggaaaagg aggagaggtg gtttgagagg gggggggggg gggtgctgat
18841 gttgtcatct gtggcctgca gatctcccag ccggcaggag aaatatttca gcagataatg
18901 gaccactatg ctggaatcag gtccctcctc aggaaccagc aaccaccact gggtcgggga
18961 gtctgattct gggatatttc ctcgccttta cccattagat gtgcagtttc tttagggaat
19021 caggatctga cgctgttatt tatcattatc tgatatttga gtcaaggaat ccttattaag
19081 caatgagcaa acaagcacaa acacacacac aggcacacac acacacacac acacacacac
19141 acacacacac acacacacac aggcacacac acacacacac cttccctctg aactcagcag
19201 ggggtggcag acaccattct ccccacatgt caggtggatg gatgtaaaaa cacgacggcg
19261 gcggtgagat ggctaacgaa aacacacggc gaggctccaa aacgaagtgg aaaaacgggg
19321 cacagatgcg agaacgggga agagcgctca aaaacgctcc accccatcat aaagcagatg
19381 tataattcag cctcctcctc ctcatcctca ccttttgtcg cggcgcgtga cgacggcgtc
19441 agcgcggaga ggtgccggtt gtgggcccag taggccacgt attcatgacg gcgttatata
19501 ttcaggctta ttttctgatg ttgggggggt tagcgtagca tagcctgcga tgattgaaac

19561 cgctctggtt ttgcctggtg gctgttgggg aggttttacc aggaacggtt aaatatttaa
19621 acaccgctgt gagatctcat taaaaacagc acgtcaggcc ccagaaggcc cccgcagcac
19681 tcccccacac acattccccc aaaaaacccc agttaggagt cggcggagtg tgacaaaaca
19741 gttattgtct gtttgcgccg cagcgctcgt gatgaatcgt cacatcctgt caaacgtggc
19801 agctggcggg actgcgtgac gagtggcgag cggagagaga gagatcgagg ctcggtcctg
19861 tctgacagct gcgagcgcgc cggaaaaaac ccaaaaccaa ataacaggac aaagcggagc
19921 agaccaacat tcccctaacg cacatgtgaa aacaggagcc aacttctcct ctctggagtt
19981 gccccccccc cctctcgcaa attaattccg acgctattgt tttggggcct ccctattttt
20041 agtccggctg ccctctcaaa taaattgatt tcctgtggcg aacgcagagc gttccttgcg
20101 cgccacacac ccctccaaaa cagaggaata cagtcctttc tccccccaga gggataaatc
20161 aaaggcaacc ctgccaagct gtacgcggct gcttctgcgt ctcttcttat acttgaaaac
20221 ggtttatttt cgtttccact tccattaact ctctgctgcc tcatcaggcg gcagcagacg
20281 gaggagctgc tcgagtcagc acctccaaaa agacccaaac caacaacctc tgcagctctg
20341 gttggcgtct ccccgggtat ctggttatct ggtcctgccg aggtcagaac gcagtcgtct
20401 ccccacatgt gcaaagatat ttagacacgc cgctttacat ataaacaaaa gtagagcaac
20461 ttgatcctgg aatcttcggc gagcgtcagg ggacccgctg gtctttgatg atcacgagca
20521 gagacagatg cacggaggct gggggatgcg aggcttctct agacggcgag aataatgccg
20581 aagaatatga atcattaagc agagcaaatg tttgctcaac ttcacgggga cgacggcgtg
20641 aacgtgcggc gaggcggcgc acacaaagct cgcctaagtt ggggaacttc cgccacgtct
20701 cggaatcgcc agatgaccgg ggattgggcc gagctttgcg tttcaatttc cgccaccacc
20761 ctgcagttgt cacggcagca gcaggagaag gaacggattg gaaaataagg cctttggggt
20821 gtggagagag agacagagag agagggagag agagggagag agaaggggag aggggggggag
20881 acgcaaatga gagaagcata ccaccagatc ccagtgcctt atcctgacta agccatctca
20941 atccctctac acccatttag cttactcact ccatagttgt agtgcaggag agacgtggcg
21001 ttcgcctact ttacagtctc ctcactgcgc cgtggaaaag attgagttta tagctgctca
21061 tcttcataat aaacactata tgcccagtag gctacaggag gtatcgccag caaaccgggg
21121 aaaaaggatt tacctcccag gtcctcccgc gtgtgacccc tcctgccttc taaacggacg

21181 ttagcatgca gtcctcaggg gctaatattg aaagcagggg gattttcttc ctcatgtttc
21241 taactttcca gctttgaagc acttcccctg ctgcccaccg ccgataagga aaatggtatt
21301 agtcaagaac gtggtcaact gcatgtaaaa cgtggggaag gtttcatcct tttaatgggc
21361 tactaaaccg gacatgggtg gaaaatattg gggagccaaa gattgtagtt catctgtgtt
21421 ggttgccccc tgttggcagg ccacagtact gcagggagcc ctgtcctcca tccctcttac
21481 ttattcctta aaaagggtca tgggggtgct ggggcccatc ccattaatca ccaggtgaga
21541 ggcagggata caccctggac aggtcaccag ttcatcacaa gacaggcaca cacacacaca
21601 cacacacaca cacacacaca aagagggtaa tttacagggt ccaatcaagc taatgttgca
21661 ctgtaggagg agaggcctac acaggctcag ggagaacaca gaaaggagca gaacagcctg
21721 gcagaaactg aacccagaac ctttgtgcaa tgaggtgact cctcattgtt gactgaaacg
21781 ggtttcattt agttttcatt ggttatttga tgctgaacct tgaatggacc agcagagcgt
21841 ggtcagagtg gccagaggaa ggtgacaatc gtctgatatc tgttgtcatg gaaatgaccc
21901 aaaagacttt tctccctgtg ccctgacata gacatgtttc ctcccctgga aaccagaggt
21961 aaccctacag agatgagaag caacaacaat aaaaagaaag accttacctt ccaaacactc
22021 tctgcacagc caagtgatgt agttgtccgg aacctctgct ggagcagtta tccatcttgc
22081 tctgcgctgc cagctgccag agcgcgcggc tccagctagc tgccagtggc aggtgcaatc
22141 aatatggact ctacggagag ttgcaatacc agaggccagt ctccactctc gaacccatct
22201 ctgtctctcc ggggggaaac aggaggcagg cgggcaggga gagcgggttt gatgtctcct
22261 ctgtggctga gacggagcgg ctcgccgtac ctctgcggcg gggagatgat taatcctgcc
22321 ctgttggata cacaagcatg gctggactca gacacactgc aaatagtagc tttatgggaa
22381 aaaacaatcc caaaaacact gagcgatgga aacatcacac ggatcgaagt gctgcgttta
22441 caccttcgac tgtgagaaga ccacaacttc tgatatttct tgtgtttaaa gtttgtccac
22501 gcgtctccgt atagaggtct gagcgtcttc ctatacagtc acaacagtgt gcagttatta
22561 aatattcacc aggctgtcag gccgtgtagg agcgttgact gatgcgtgat cattaacggc
22621 aatttcagca cgacagaaca aaaaagacac acacgcgcag gcgggagcac gcacgcacta
22681 atggccctga aggtattcac ttctccacct aatttaaagc gctctcaaag cggagcaacg
22741 gcgaagaata aacaatgaat ttaaaacgat ctttcgtttt aatgacttgt tgttgtcttt

22801 aaaaaatcat tttctgaaat gttttcaggg gatctgtggt ggaggagtcg gctaattaaa
22861 aaaaaaaagg gggggggggt aaaaaaagag aagagccgcc aattccgccc ggagacacag
22921 atcttacata tttgcacgct gctctgcgtt tttcagcagg cggcattttt ttttgggggg
22981 gggggggag ggtctttcac acacaaacgt gacggaagta cgtgagacat ttagggaaca
23041 cgtcggggat ttcacgtcga aagtctcgga ggcatcggtg ccgtaatcga ccgtgtggtt
23101 ctgttccgta gcgacgtcgt cgagtttgtg tttccggagc aaagtattcc tgccgttgac
23161 ccctgaccct ggggtcaccc tgaaggaagt ccagcagccg ccggcaacgc tcagttacac
23221 gtgcgcgcac ctgcgctcgc gtattagcaa caggagataa gcgctgagct ctacagagaa
23281 gcaaagccag aataaaagaa gcagctgtaa taatctcgtg ttaaagcgtt tcccctcaaa
23341 ctgtcttcaa tatgtaggca aaagcaattc cagagcagcg cccgttcact ggtaattaat
23401 ggacattcgc cacagggcaa gtaaccaaag taccctgagg gtgcagtagt cctctagcct
23461 ggtgtgatta atatgcacag ctcagcgttc tgatctgaac atccagccgt cctctggatt
23521 tctctcgtgc agccacgtca gcgccggaga cgccgtgaga acgcgacacc tggtcgtttg
23581 acttccaaac actcccagac tctgtttttc ttccgtcatc gcctctttct aaagtctgcc
23641 ctgtctcatc cctctgtctc gtcccggttc ttgtaacaat aataataatt tttaaaaaag
23701 acccttgtga aactttcaag agggagaagc tgccaaaaag gcaatttatt gctttggcgc
23761 aatcaaagat tgaagagaac acagacttgt aaaagtggtg acttttatag tgttctctat
23821 attacacgaa ggagaatatc atttcttggt agcctgagga tgtctctctc tcgccgccgt
23881 gtccgccacg gctctgaacg ccacgtgcaa gtgctggctg cgtgtccgcc ctcattccct
23941 ttcgtcttcc ccctctgacc tcaattttct gccctcctaa acgtctgact gttcccccga
24001 ccccgaacag agccagccca ctatgggaat gatagatggg agcaacattt gaagctaact
24061 tctccatccc ttctctgatg caacgcagac tcggagctgt tggcttggtg cggagctcag
24121 gcagtattca tgggctgagc ctcgcaagct gttgctgcac agatttggga aggccgccgt
24181 ttggatatac ttcaaggaga atgtgttcgc gcagcccgta gcctctgcgt cgatagcggc
24241 aattgtggat agcgtcgagg cagagcagag ttcacacctg ttcacatggc gtacaaagtg
24301 taaccggagg gatgttcccg acgctaatgt ttatttcacc ctgcgtgtca tccgccacag
24361 gccactggaa acaccaggga ttatttaata gcgcgctacg ctgatggcag gctacacttt

24421 taccaccatt ttatgatgtg aagctggcaa agctatccct catcggagca gatagggaga
24481 gcaagtgaag gtgtcattca ttactctgca cggccggctc gttccgggcg gcgtgcgcga
24541 gtcacggggc accgcaacct cctcgtgcgc attaaaccgg ggcctggccg cagaacggcc
24601 tgatcgacgg cgggttttgc cgcagcattg acgcgctgtg aggaaactgc gagggtctca
24661 gaggcaggca gacagggctg atgtcaccgg cagcgtgacc cgtgtggtgc cgtgtctcgt
24721 gtagcaacca caagtcatat tggcatgtta tattacactg cagtctccag gaagtctaca
24781 catcctgggt gagagggtaa actgataaga taatgtaatg aggagagaga gagagaggag
24841 agagactgat gcctgttttc cactgcagag ctgcttcgac tcccctttta ttctgctaat
24901 ggaagaccat gcagtgggaa agggttcctg tcaggaagag cgagcatcac tgggcgggag
24961 aggatcgtac tggacccctg actaaggtgc tcagctcttt gtaaagccga gctaaaagat
25021 gaggctctag gtgtatcccg gaaactggat gtttgtgaag gcaccaggcg ccgcgcatct
25081 ttcagtttgc attatgggcg ctggctaatt agtcccagtg gagaccgagg tgggcctcac
25141 tggaggttca accagaggct acgagtctca gcagctcgta tttcactggc ctctatgtac
25201 caaaaggttc aggcgggtgt cagtgccgca cgcacgtgtg agtctggaga cgtgtctcgc
25261 tcacgtacct gcacaaatgc agggctgaag ttacagtaga ggaagatttc cagttgtttt
25321 agcagatgtt tattagtcct agaagaagaa gaaaccctgg tagcattgtt gtgggcatgc
25381 tagcgcttac ttgctacttc aataagtgaa actggtgtaa caataaattc cactcagttt
25441 actttgaaat ggaggcttta gtggcatctt tgcatctgtt gcatatgcag gttttaaact
25501 caccctgaca tggtttaccg gctggataca cgagtggaaa atcacagttt tagctgtgga
25561 gtcagagcta aagctagagg agaatattaa agaaacaaat gtatccatat gcacggtgct
25621 ttgttttaca gcgttttaat tgtaaaggca aatatttatc cggctgcttg ttgaggaaag
25681 ttttacactg ggatagaagg tgtttaaggt taattattgt gaatttccaa aaagattaaa
25741 tgttttgatg acagtactgg gatttttctg cgtattgctg tttgtgccct gcggcagttc
25801 tcacagattt gaaatgctgg tcgcattcca aacacttttg ggaacatgtg attctccacc
25861 acagaacgcc aaaacggcct cacatcaagt gaaaacaatt agcaagaatt aggccttacc
25921 cgaacaaaaa agaaaaagaa aaaacgctta ttggtttcca catttccaca tccaggcttc
25981 tagtcagtgt ctgctgaata tggatgagac cgacgacatg gaaaaactaa aaccctacaa

26041 tgtctcgtcc cacctgtagg caatcagtgg ggatcacatt tcagaaaaag ctctttgctt
26101 gcatcatccc gtccggttcc atcccggttt gttcacatca aacagtcaga ggaagttttg
26161 ttgcattaaa tatacatccg ctctgccctc tccgccccgt ctcctcgctc accaccggcc
26221 agtgttttgc tttagagcaa agtgcccagc gatgtctgat caccggactg agcggatccc
26281 accgcgaggg acttgagggg ctccgctgtg tgattaagca agcctcagta ccaccagagg
26341 gccaccggag cagatgttct cattaaaaat gcaggctgtg tgagcgaagg agattgcctt
26401 gttccaggca gaacctggcc ggaggttttg tcgaacgccc cctccctccc tccctctccc
26461 tcactcggaa ggccggccaa gcctcgatgc ctgcgcgagc tttaagcaat gtggcaaccg
26521 ctgaaaagga gtcgttccaa gggcgcagca caatcgcgca agtgggttac ttctctttaa
26581 ttcgtcgctg aaaggagaga atgggtcatt tcagttgctg tttatgtagc accgccgctc
26641 ctggcatcct cgccgctgca attacaaaga aggttttctt attaaatgtc tcagctgagg
26701 gctgcgggga agagaggcag atatttcagt gttttcagca cggaaccaaa gtgcaaatga
26761 atggatacca actctttcga gggccttaag aacaagctgt tgacgacagc ttgggatatt
26821 ccggcccttc cagctggcgg ttagatggag actggcacaa ttaacataaa actgcggagc
26881 gcaacagagt gatgcggcac accagagggt gcacacacct acagtacaca gctgctcaat
26941 agattcagga ggatctatca aaaagctggc gtaaactcgc tgccagtgtg gattctttgt
27001 tggattgtgc taaccggcag ttagcttaat gacgctgaag ctttagcaaa gtttagcaga
27061 gggttgtgtc ctctgattgt acaccttgac tcgtggtgca cagttacatg gattccccgc
27121 atgcagccgt ctgctgcaca tgtgggagga aaagttactg atggatgata gtcctcaccc
27181 ctgtggatga ccccattact gccctttgcc tcaacccttc ttcagcttct tcatcagccc
27241 tcctctctaa aaccccctcc ctactgttct cacacacata cacatgcgtg cgcgcacaca
27301 ggcatgcaca cacacaaaca cacattctca tattttcaac tttgtgggga ctttcataga
27361 tattatacat ccttcacccc accccccacc ccccagcctc taatcatcct gactaacacc
27421 cgaacccagt tctaacctca accttaaaat caagtcttaa ccctcaaaca gccctttaaa
27481 gttgtgggga cgagcaaaga gggcctcact ttgcaaaaat gtcctcagtt tgccagtaga
27541 atgagaattc tggtactctc atttcactct catttatcat gtaaaaggat gcacagatag
27601 acagacaggc agacagacag acagacagac agacagacag acagacaaca gacacacaca

27661 cacacaatgt tgaaaatgcc atgcttgcta atcagaatgt gccaatatta ttcccaaagg
27721 ccaaagaaat ccatgaatca tgaaaaactc ctggaattat ccctcatcct ctcacacata
27781 ttctaattta tttcatggat attttattcc ctctctacgc tctggatgag gcctcatatg
27841 acagtcataa aagatggatg aaggtggaag aaagaagtgt ttagttgggt ctgggtattt
27901 gttgctttga ttttctttcc tttctccagt tatttctttt tctgcgggga acattcatgt
27961 ggggggtaga ttattaagga atgattcatg cagaagaatt tactggtcgc tcatgaatgc
28021 cgcacaaaag gtcagaaaga atagcaaatg atgacacatc cgatagtgga attactgcag
28081 attaaagcag cttgtcccaa attagctgat agcgatccaa cgagaagaca gagagagaga
28141 tttatgaaag aatgcttgca gcagcggtcg cagtgatgct ctccggtgat ttacaacagc
28201 aatatgagat gggaaattca gccagatatt ttacacaatc ccccccccta aatatggcgc
28261 tgatgtcact ctgtagatta tcgtgggggt gcggcgtcat taacatcagc tgctgtaaat
28321 ctctatctgt tgcttctgaa tgcaagagca gaaaagtgtc tataaaacaa acgtcatttg
28381 tgcagaacag attgtgttgt ggagacaaac actgcttgta acagtagaag ctggggccaa
28441 attcagcgta aaaaagcaat aacctgtatg ttaccaagat ttatgcatcc ctagatggcg
28501 cggttcctcc gtaatgaggg ctgccaggta gtaaattaag aaatgccttt tggcaaagag
28561 cgttcttcac tctcgcacac acacacacac ccacacgcac aaacataaac acacacacac
28621 tcacagctat tgcactaggc ctttcttaga tctaatgcgt tcaccagctt tcaccgctac
28681 gttagcccct cccctgacct taaaccaagc ctgtcatggc cttaaattca agtcttaact
28741 ctcaaacaga cttcgacgcc tcaaacagaa gagaacagaa aaccctcaat cagaagattg
28801 aggagcagcc aaaaattgta atgttctgat ttccacaaaa cacgtcctca gtttgctagt
28861 aaaattattg ttgctaatca atatatacta tgtacaagaa cacacacaga agggttaagc
28921 actgacttta tgaatccatc tttgaaccct aaccaactgg cgttggccct caaatttaat
28981 gtgaaactac aacccaaata tcgtgagatg aactagtttc attttcaaaa aaaccccagt
29041 tggaccccac aaacccatat gttttgtgta ttgatggtgc agacgtggct gcatgtctaa
29101 tggcaacagc tgctgtgagt gaagaggccg ctgaatagga tccatttgta tgcatattaa
29161 tatcagagca cagtgaggcc aggacaagaa gatcggacta atttgaccat ccatcatcag
29221 gacgcgtctt cctggtcaga aacaacacat cattaccccg cgcacaccac agggatggcc

29281 gtggtaaaac aacccccccc ccccccacac acacacacac acatatagaa
tgtgtctgtg
29341 atcgttcgct ttctcccaca ctctgggaag ttggagcgag tcggcagcag
gcgcggcgaa
29401 cgtccgcaga ctgtaacgat gtcacagctc ggttcagaaa gatccttgtg
agctgctctt
29461 ctgtttcagt aatgaagcgt ggagccacag caagcgcccg tctgtgaaaa
ctaacacccc
29521 gaaaggacat acagacacct atttgtctgc tcgcatgaga tcgccaaggg
ttaccgcgag
29581 ttccgatatg caataaatgt catccgagtg ttcggagtga tgctgggctc
gggggggaggg
29641 ggggtccttc agcggtttgg gggtcagggt tgaggttggg gtagttggtc
tctggtggca
29701 gagtgtcaaa cctcagccct caaagctgat ctttggggac tttaggcttg
tctcacagcc
29761 tagagtgaga gccaatgggc cgtgttgaat tacagttttt cccagttgct
aacgcagtaa
29821 aattacatgt atagcacagt tatttaaact gacacacaga gagtaaatcc
tttttctcaa
29881 gtcttcaaaa ctacacacac attttcagtt caaaatgtcg cgtttcaaac
gcattgaaca
29941 ctgttctttg cacaagacac aacaatctaa cgtaaagtca caacacgtat
caaaacgacg
30001 ccatttgaaa tgatactaca tgagctaaat gaccaatata ttagctccat
ctgagcaaac
30061 acttcaatca acaaagttta ctacttcatt caggaagtaa gcaacctcag
cacgggttag
30121 cgtgagggac attgaagagg aagagggagg gtgagaggta ggagtagagc
tggtagagtg
30181 ttcatggcca aaacaaacaa ctttaaaatg aaatctcctc aaggtcctgg
gtacaggagg
30241 atggctttgt aaacttgcat ttggaaaata aagcatcgtc ctccaagaca
acattcaact
30301 agtccgtctg tccccaatgg ctcgcattct ctagcgaaac caaatcagaa
tgaaacaact
30361 tcattaggca gcttttgagg gaaactctca acgaaacaaa gagctcagat
gaacatacgt
30421 ggctgtaagt attgactgca gcacgctaca cgcaacatcg cctcccaggg
tactgggtaa
30481 catcatattg actgagtttt attctttgtt ttcagggagc cctggggatg
gatgtgcatg
30541 caagtccacg tgagttgata gatgaggctg gcttcaacca ggaatagatg
aggagaaagg
30601 gaagcacggc catttccagt acgcgtggtg tcctccacca tcataccaac
cttggaccat
30661 acaacacagc catattctca catttctggt cacactccac aacgtggaaa
tgagtgatgc
30721 agaccctcaa agaaagcggt accccataga acccccccga gagaagagtt
cttttcgact
30781 tggcgttctg gaggggatac cacccctggc cctttgtgca tgtgccttct
gtgcagtctg
30841 tggaagaggg gtgtgatgaa attgatgtag gtgctattca gggatggatg
aggcactcga

30901 ggcgttctac cctccatgtc tgtcaagggc agattttgcc tgcgatgcgg
acgaggtgtc
30961 gtggccaaac cagctgtggg gcaatacctg ctgcctaatc ttttattttg
ctgttgttct
31021 gcatatttat ctcgcaatcg tcttgttttt gttgcctgtt gaatgtattt
ttgttcagac
31081 tacctgctgt tgcactgaca tgttttcaca atagtttcac tagcaggtcg
cgtatgtgca
31141 aatatttatg tgcatgtgaa atctgaagaa cgttcttggt gccaaaggtt
ggttttgaca
31201 gtgcttggac acgtgctttt ctgtattgtg tttgtttttt tgataaaacc
agcctcacgt
31261 gtaaaatcat gctttaacga ttggaaaaac aacaactgta gaccagcagc
ggtttcttct
31321 gaaggctgaa gcgcacgttt caacgatttt cctcattctg cctaacacgt
tctgaaaacg
31381 acacagttga ctcgtttttg gcatcccgct cccatttctc tggattatgg
atcgccaact
31441 gcgcttaacg tctgcactca ctcatctcag aaccacgcac gggtggcagg
aagtcccatt
31501 cagggtgccg tcgcatcatc aggacgacac atgctcacac actcaagatg
caggaagtaa
31561 agagtcagca acttgaccag cttcatacct aaagagggac tattttacct
gtgaaacaat
31621 gtgtgtctca cactgtctac ctacacgtga tgcatggtac acaggacttg
ggcagctggc
31681 cggtataaaa ttggatctcc aggtttcctg ctgctcagcg ggactcaaag
ccatattaaa
31741 ggcacctcct gcagtcatgc agcagtggga ggacatggat gatggccaag
gcaagcagct
31801 cggtgccagc cgcgcccctg gaggactgca tctgctcgcg ctctccgaac
tggccccttt
31861 atgttcaggg tgacacatat gccagcggtc ccagactaca cgcgctttaa
ttcaagatca
31921 cggcgcacca cgtacagtag aggcaataaa cctcgacaaa tacacttttg
attgcgcaca
31981 tcagggtttt atgcacccat gtgtaaatag atgggaggaa gtgtgtgttc
atgcatacag
32041 aaggtcgcct ttttccaatt tatgggtcag cgtataaatc cagacatcaa
tcttgtgaga
32101 gctgatgata tcctcccttt ctttccaatt tacgcaggct attatttctt
ctgttaaaag
32161 cctcggcgct gaaggggagt gtgtttgtgc gtccggcggc tgctcggcac
cggtcgctta
32221 gagcggtgca gattgcgtgg gcagatgccg cctcatgggc ccagctcgtt
ttttatcgcc
32281 ggcttgtact tcacaccggc cacataaagc cagcagagca gtcaagcccg
acgaccgtga
32341 gtaggggcgc ttgtgaaatc acttcccttt tgtctctcac acataattga
gccccactca
32401 tctcctgcca aagcaaatac ggcggcctac atttttgaaa aatgcggcgt
gaaagcgcga
32461 ggaaactcgg gcagtttggg agaatcggga tgctgaggtg taaatctgcc
acgaagggaa

32521 gcaaagctgt ctctcaaact gccactttgg cggcggccct gggagagccg ggaggataat
32581 gggagtcgtc taataagttt gtttggcttg ttagtgtgat tgttttctca caaagctaat
32641 tagctgtctt ggccttgggg accctccatc ctatatcttg ccattaccac ctatccacac
32701 acacccacgc acatacgcag gcattcatct acccgtcctc acctgcctcc gcccccttc
32761 tcaccctcgc cgtgcagcag ctcgccgtct ctctgtcttt gctccctgtc atgtttaaat
32821 tgatggaggg tctagtgatt gtctgtatgg tcccaaggtg cctttcctca ccatgcaggc
32881 atcctccttc ttgctccaca gttgattacg ataaatcaat ctgggggggg ggtcaaacgc
32941 ttggactggg gcagcgtcat ttccagtggc tgcggtggta cgccaggctg ggataagagg
33001 gtgtttcctg aaaccccgct gacctttctg gacgactctt gtttccatgg tgagaccttc
33061 aataaggtac aaatctggtc ctgtagtcaa aaaccagtgg gggaggagag atgctctcat
33121 ttttcatctc tttctttgcc tccaatggcc ttatttcccc gtctggcagg gctggaaact
33181 gacaggtaac ggtagagcag agcaataacg ccatccccct gtcctgtatt gcaaataaag
33241 caggagattt aatcaatggc caggaggaaa agcggggcca aatcaataga agcctggctg
33301 tttgctcccc tgtttgaagg tgaatccagg ccttgccaca taaattggac taattgaaga
33361 gcatgctagg aaacaagccc aataacaaag catgcctttg tcagcatgat acttgtatgt
33421 gaatttcatc gagccagaag tgcagaggag gagataatga gctgccagga gtctggaaca
33481 gaaagcagaa gggataaaca cgatattagt tgcatttggg cagataacat ccgtctagct
33541 catttaagtg ataagcgtgg gcgcgcggcg gttttcagaa gcaatagcac tcggctgtgg
33601 gatgagtcga agcgtttctc tcgctctgtg tatcaagatg ttcccctctt catctatccg
33661 ctgtcgtttc ctgctgcaga atcacgccag tactaaaaga agccattgct tgccgaggcg
33721 gggacataaa tccagccatg gtgatacatc tcatcagcag aggcaacaaa ttaaaaaaca
33781 gtcgcccacg agacgccgct gctcaataaa ttggccctat tttcgagggg gaaacggttc
33841 ctttcccaac gcaaaacaat caatcagagt aaatgactgc gattcacaaa tctgtcgcag
33901 aggtgcatcg ctgtattaac cgtccgacct gtgtgctgat cagcttaaga tcttccccgg
33961 gacctcattc catctcaggt ccctcatggg gacgggaccc tgagtgctcg ctcctggggt
34021 tggctttaat taataagttt tgcagtgatg gcaaagaaca acaggaggtg ttaaccctgt
34081 tagtggagac gcatgttgtg gctcttctga ggggatgggg ggaagggtgc gggggtgggg

34141 aattaaaaga tcgttgccat ggattggttc acaatgcacc aggaagcaga
tctgagtcca
34201 aatgaaatga ttgtatcaga tagaaatcga tagcaatcca tgccttcctt
aatccagcag
34261 tagctcccag atcactcctc ggcacaggca atctctcagc catcattacg
acacggaggt
34321 cgatagcggg cagcgaggcc tccggttagg tttctggagc agtgacgaag
aggcagaggg
34381 agcagaaggc gagacagcga gcggcgaacg gcgaacggct gaggggtgtt
agtgtggctt
34441 tgggtacctg acaaactaca agcttgttgt tgttagaggt aattaacaaa
aggagagggg
34501 aggaaaacag gtaatccagt gcgccaaaaa aactttaaat cctgccaaag
ctctaaagcc
34561 caaaatgttt tcttgtgtga ttctcccaca ataggggatt tgagttaagc
gccaggcata
34621 ttctgcttca aaagatggac gatgaaggcc ggggctggtt taaggaggta
atcagagcag
34681 gtgcaaatcg tctgtcaagg cgtaacacaa ttttacagag tgccttcggg
tgaatggaag
34741 catctcctac gcctcagaag acctgctgga atgaacgtga gagactttag
ccgcatttgg
34801 gaggggtaaa ataagaagaa agagtaaaga acatccaaat gctttgagcc
gcaacatcgc
34861 cggtaaacca aatggatccg atgacttgtt tttgtaccaa accctccttt
tttgaagaat
34921 taattggcac atttgtcatt aacaccggtg gcctgagcag ctgggtgctg
agaaggcgcc
34981 ttcgggtttc agtctggctc ctattctgca gtccggtcac agtatttgaa
acccctttca
35041 aattaaaacg cagggtaggg cgctgcttta atgatagctg cgattttaat
cagcggcgca
35101 gtattcctaa taggtcctgg aggcctggag gcctggctgc acacatcaac
ttatctcctt
35161 cgtaaagctc cttttctaat ttcccaccgc catcaaagtc tgcccggctc
tcccggcgct
35221 cacctccgtc ctcatctccc cccccccccg cagacctcgg tggtacgatg
tgcatattga
35281 aaaaaaaaaa gagtacaaca caacaactaa ttaattccat taacatcaat
attctgctca
35341 tgagcgggaa acacaggaga tgaagagaag gaaggcgcgg caaaggatta
tgagtgggaa
35401 gatggatgga tagaaagaag tggaaataat tgtttactct ctccattctt
tatctttgtc
35461 ttcttttccc tccgtctcgt ctctctgaag gagcctgttg gcgtgaagga
acttctctca
35521 gcaattaaga ggagccgcag acgctcggca taattaccgc cgcacgctgc
tgctgtcaca
35581 aaactcaggc ttgccctctg gtcataaaaa aacacactga agaatattta
gaaatgaata
35641 attcagagtg attgttcata cttaattatt catgggaccc cagcgaaggg
agatttgcat
35701 ctcgccgctg taaatattat taatcttgcc aattacaaaa ttctctgccg
ctccgaattc

35761 ttcagatgat gctacgtgat gtgtgcagtg ttttggagct ttttaggtct
aagcggctct
35821 aaatgaacgt tctgcagatt tcatgcgcat cttaaaaaaa tcaaagatgg
agctttcgtt
35881 tgggggctaa aattcaaagg caatggcctc aactgctgta gccagaggtg
aaatagagat
35941 tgttgggacc aaaggtgaca gttttctttc caaatgatta aaaacgttta
aaggttattt
36001 aacctttaa aagctccctc tagaggtaga tgagtgtaat tacatgacca
ctgttgcacg
36061 catggaggtt gttgtttatg tttagagcaa aagtaaaaat aaatgtatcg
ttataccatt
36121 atttccatgg ataaaagctc ttaactcaaa ttgtgatgat cgggaaatat
aaacactatt
36181 cctatgtttt ccttatgtct acatctcata ccacgctcca tctgggcagc
attgaagtga
36241 agcttcaaaa aacttttcac atcaaatcca tgatccctgg actccatctg
ctgaatcaca
36301 gccctaaaat taacgatgac aattgacata acaaagtaca gtagatggaa
tcagagacga
36361 gatgagttac aataacatcg ttattctgat tagcctccta aaagtgcgca
gtatttagct
36421 ccaaacattt tcaaaatcgg aagcccatta tcagcacctg aggtcccggg
tgttatcaat
36481 agcatgttgg gataataagt catcccagct catcatgggt tggaataaat
aatgcttcca
36541 gggcaactga aaacaccatc agctcacata gttatcaatg actctggcgt
ggagctcaga
36601 ggaggagaga aaatgatcat cacttgttgc aaaagctaac gccagggtcc
attttcactg
36661 tttattatca ggattcttgt gatgaccaca tggaatccca gatccataaa
ttactgccaa
36721 tcatttattt cctcataatc tgtttaaaaa agattacccg gagctcattc
cttatctttt
36781 attatgttct tgaaaaagca ggactgagcc ttataatctc agaaatgcaa
atggaggtcc
36841 atgtccagac acttaattaa atttatgtca actttactat tacagtacgc
ctttctgtac
36901 ttgaacaatg tcacagatcc ctgataaatg aaacacgtgc tgggccaaag
taataatttg
36961 tcaagtctgg tgaaaatctc ccagtattag gttggaaggg ttgatgaacc
cttggacggc
37021 tccaggagga tgtgtgactt ttcccttatg cagagtgtcc ctgacctgaa
tttaaagtgt
37081 gtgtgcatgc agtttgactt tataggactg taatgggaga ctcttggggc
tgtttatcac
37141 atgtaacaca cacacacaca cacacacaca cacacacaca cacacacaca
cacacacaca
37201 cacacgcgca cacatgcaca cgtacagttg ttattttcct tgaggtcaca
gatgcgataa
37261 acatgtgact tgcagacttg caaaactgca aaagaaaaca caaaaaacct
gaaccaaaca
37321 agaagaaaca aacaaaaaaa acgcacaatc aacaataact tccgaagaac
aagatctttt

37381 aacacgtgtt tcgtttgtgg gaattcacaa ctgcatctgg gtctacaagt aaagaggaac
37441 aaacgatgat gcccggtggc aaacgagagg ggaaaatgcg ccctctggtc tgtttcacct
37501 gtggattaaa ccctttgttt atacttaatg gtaacgcagt cttaagtgat tcgataacgt
37561 gcggccttgg aggagcatta aatctaaaga agagctcggc attcatccct tcagaggtgg
37621 agacaccagc cctccacatt tttaatagtt agcctgtttg tctcttggtt gcctgccttc
37681 tgcttctccg agcctcaggg tgaaccagag agtgacactg tctaatgctt taagctagca
37741 cctcactcta ataggccctc ccttaattag agtgggaccc cccccgcccc cattatgaga
37801 gataaagccc cacccccaga aagcgaggca gctgacactc atgcaaacac aaacacacac
37861 tgagtacaga tgtctgtatc acggtggcag tgtgtaacgc ctgttgagcc ctgacgtggc
37921 actgataatc accatttcag attgtgccga cgtgtccagt ttttcccctc cggttccttc
37981 ttggcctcac cctggttgcc gtgcaggctc cagagtgatg tgtcatctta taatcctacg
38041 ccggctaaat tacattagga atctcctctc attaattaaa gcggctattg gcagcacctg
38101 agataaaaca gattttaaga gctcagagtg agaacgcggc gcgcagaagt gccggcgtca
38161 gcactttgtg agtgtgtttg tgtttccagg gggagagaga gaggggggggg ggcaatagta
38221 aagatctgct ggggttctgg agtctataga tggtgctata gcacacacgc cctctgtgat
38281 ttaaagctta tccttaaaga ggtgtttaat ccagaacaag attagtttta gggttaatgg
38341 atgccattca cgtacgctgc cgaggtgttt ttgacgtgtg gtttaaagaa tatgcagtaa
38401 aacagaaccg gagcactagc tgggatttat attctgccat caagctattg ctaatctata
38461 actttgtcag cagcggcaat taaactgaag cgatcttccg tagattactg ctcaaattgt
38521 ataatatgac cgtcaagtga gtcaacaaca gacaacaaaa aatgcttcaa gttgctgaca
38581 cgcacggcaa agtttaaaaa tgtacaactc tgtgaaataa taataataaa atataaagtc
38641 attaaaatgt atcaggggga gcggcttcaa acaacattaa tcacggtgaa aatttacatt
38701 aaacacactt ctcttggtag ctgtcaaaac tgattaatgc aaaggaaagt gagagaggag
38761 gggaaaaaaa agttgtataa atattaagtg agtcctgcat gttgcctttg tatgcaaacc
38821 atgtaagagc cattgtgtag ttcacctggg tccctcgcaa atgaataaat cactgcctct
38881 ggacaaatta atcaaaatgt ccagctgttg ctgagcctgc tgggtaccgg agctcagact
38941 gcatccttgt gtgtgtacct gtgtgtgtgt acctgtgtgt gtgtgtgtgt tctgccctcc

39001 tttcctctct taacagcaga cctgaaatga cactttagct ttgtgatagt tggtatgatt
39061 ctcacccaca acagttctgg gatggctgct gatgtctgtg gcctgagcta gactctgtcc
39121 acaccatagc cgggtagtta gacccccagc ccccccgctt tgagctgtaa agtctatttt
39181 tatccactca catttggaga ctggttgttc ttgacggatt ggtgcctggc tagatgggcc
39241 aatcatgagg tggagccctc ggtgacctaa agtgttgagg atgttgccct tgggcaacca
39301 gaaatggtgt tttggctatc agctaactga gcttagcata cagaaaaaca ttaaagacca
39361 caataactgc gccgctttga tctttcaaac ctcacccttc ccgtcagtca gtgttggcgt
39421 tatttacagt gatctttcct cgacggaaac acaggcagag atgggtcaat ttccccgtaa
39481 atgagttcca tccatctatg tgtttcttat cagctgctcg tttctaatcg gggtcacagg
39541 ggtgctgata tctaccttag cagcctctcg gtgaggggca ggaatacacc ctggagaggt
39601 cagcagtcca ccagaggaca aacacgctcc tgatagaagg agccagagct ggaatcaaag
39661 ccggctttga tgttctagag agggttacta tgttctggtg ctttaattca gtcatccagg
39721 aatgttctgt ttcccaggac tattggatcc agtttaaagc atcaacagga caaatccacc
39781 ctgcatcctt ctactgtgtt ctgatgtcat taatatccac gaatatgaac caccggcaag
39841 tgtgtgtgtg gtctgtgtct ctaggtgtga gctcaagctg aaggcctgag tcttctcagt
39901 ggaggtctgc cagcagagca gatgaaccag gacacagtga agtctccatc agctctgctc
39961 gcagacgctc ccggtttctc cagctttctt caaatcgtgt tactgagtga cctttaacac
40021 ccccgacacc tcttttggtg taattcaccc ctgtatcctt gagaccccgc agcagatata
40081 accccgcgcc tgtagttgcg aggcggcgag catgtgctaa catcacggat gacagacggc
40141 gagcagatgt gacaccgctg tcatccgccg tctccggacc cgccgtccac ttaaggtaat
40201 ggacgcgcgg gatattccgc cttccgctcg ctcttcctcc tcatttccct tggaaacgtt
40261 tagtacggtc tgactgtgtc tgtgattctg aactgacggt gaacccgccg ctctaccccc
40321 acgcctcttc acccccccca cccccccgcc ctgactgctg atgtcctctg catgaggaat
40381 tgctctcaga agtttacttt gtgttaatct cataaacgcc tgggtctccc tccccatgca
40441 ataaaccact tacataaacc tttcctttga gacggggatg gggcgggggg gggtagctga
40501 catgttcaaa gttccactcg gcctctctct ctccccctct ctgtctctct ctctctagaa
40561 gggtgtgatt tccaccagga atgctttatt tggacccttt attacacatg ctgtccggga

40621 ctcttgaagg gcagttttaa tataaggaat cttcgctctg tagcatgtga agcagccacg
40681 aggagttgaa aaatgttgac atttacgagc gcagaagcat ttcacgggac cacttgttgt
40741 caggtgatca atatgggctg acaccagtgt cactggtact ggttattgta gtagtggcca
40801 aaaagggata gtctgactaa tactagatct gctgggattc tatatataga gcactctata
40861 aggagcattc tgacgtgcga tcaaacagta cctacacctg taactgtacc tacctgtagc
40921 tacacgttta gctcgactaa gatcgcccct gcaaaatgtg caccaatggc atcacacaga
40981 gcccaacaat agctaagcta ggtgagctag gaagctggtg tctgagacag gtacagggct
41041 tttgcacatg cgcggagcat ctcagcgtct tccaggataa aactgtccac aaaaaagtgg
41101 atggactttc atttgcgttt tctgtgtgcg ttagtgcgac ttttaaaact ttgatttctg
41161 ttaagatagc atgtttacat gtattattaa tgtccagctt tagttggact cataaaaaag
41221 gttaaatgaa ataatttgtt tgctttttct taatccaaat gaatgtgaat tagtgagttg
41281 cttgttgaat ggaaatgaaa taaaatgaca cttttagcag tgggtgctgc cgggaggttc
41341 cataaaaata aaccaaaaca ccagaaaact gttatggggc ggcgataatg taaaagagaa
41401 atgaagactc gtgtttgttg gtcgttatca gcattatctg ttcatttcta gtctgaagaa
41461 caacccgcac agatgaaggt agcatcaccc ccaatgtaga ctaaattatt aacgcctaat
41521 tatcatttat tatctgtgtt tttgtgcttc tgtagtaatg gaaaaaggtt tcactcagag
41581 ggaatctctt atccttattg ggagtcagcc aggcaacatc accattcatt accggctcct
41641 tccactcctc ttctcccacc actcacacct ctttccatct cttttcagtc aattctcttt
41701 tcctctcatt tgcccgggat gcttccacac ctcccaatcc catccgatgt gccatcattc
41761 acctccagag ggccgttccc actctgaatg ggatcataat cggcccagct ttggttttaa
41821 tagccaaaat gacaatagct cgcacgagcg cacggctgaa agcgtgtgca cgtctcgccg
41881 cttctgacac ctctgtggct ctgcggatga gcgtggacag atgtgcccgc tggagcagaa
41941 ccgccggaga gcgtggagac ttggacatta atgagatggt gacaaaactc gcactcgcca
42001 attacaagaa agtgaatggt ggatgtttga gagggaggga gggaggaaga gaaatgacaa
42061 ggatatccca ttgagaggct gtgctatagc gctccggcga aaaagaatga ttggatctag
42121 tgttcacttt ttaattctaa agatttaata tgtgtatcct tttttaatca tttttctttt
42181 actaatctgt gtcagcactg tgtaaagtgt catctggtct ttttttcagc catcgtgact

42241 gatttctgta attattaatt ctattttaga tataagtgcc ccattcaagg tgacacattg
42301 tgacaaaagc ttcaacagac gcctacaaat tcttggatat taacatagca acccataatt
42361 gaccctgaca acggcaaaag gtgcataaat gaatgtacac gttccagagt ttcatggaac
42421 agagcatata ctgcatactg tcatacacca tatactttat gtctgatttc tctaaagagc
42481 taaattaaca tcagctaact gttgccgagc aacagtacta aagggctctg caagaactct
42541 ttcagcccca tggaaatcat ttctttaaat taaatcacac catttcatca cacaaatagt
42601 gacgctgaac attaaactcc ctggttgttc tccagtctgg aagctagtgg aataagaaca
42661 aacaaccaaa cttgtttagt ccgattagca gtggtaagtt tgggatacct gtcagcagcc
42721 tggagcacat ttaacgccac taggattagt tagtaaggca ataatctgat tggctgttta
42781 gatttagtga gttggcaata gagcacttta ctgcccctgc tggtgagaag aatcattgca
42841 tctcaagcag acagagaaag caaaatctca aaatgttgct tttctatcaa tagctcatga
42901 tttagatcca ctgagctttt gcaggaatgc taaacaggtg aatcaccact aaagcactaa
42961 ttcaaattca ttaggacact catccagttt aatcacttct ttataatgaa ggaacggtct
43021 gtgaaacacc tctgcagatg tgtgaaatga cacatttgta accgtggagt ccctcacctg
43081 gacatacagt gtgttgcacc atgttcagcg gtcagaacac agccgccggt ggctgggctt
43141 gttatcggcg ctgtatgggg cttgttaaaa aataaaaaaa taacaagggt gtaggtgtag
43201 gtccatgcgt ggcagcgctt gctcctaacg tgtgtacact gcatgtttgg tatgtgggac
43261 ccctctgcag gcggcattga tcgacctccg tccctggacg ctcgatgcag cctgacccct
43321 ccagtaacca gtcagtaatt gatcctgtag cagcacggtt gtggactccc acggcacccc
43381 gaatctccac ttatgtgtga gtgtgtgtct tctgtgtctt cctttggcta ggctcctagc
43441 agcacgcccc acctcgctaa cctctctgtt gcattcagaa tgcatccaaa gggcatggag
43501 ctgggcacgg tgtgcgaggc atctatattc tctctatcgc tctgctcgca tctcgtttct
43561 tcccccgctc cgacgctgtc cacagcagtt acccgacggc tgatgaaacc tccctccttt
43621 tggttagggt tccttcacat ggcggcacaa gtttgccggc agatcaaaga gcgccgtgat
43681 ttcatgtaga tgaaagcaaa tcgcagtccc gccaagttgt ttggacgctc cgccgcgctc
43741 gattgtctca cggttttcga gcgacgcgct cgccacgaca ggacccgttc cgctgccatc
43801 caattccagg caggcagagg aaaccgggct gtgccaagtg tgccaggttc catcgtccct

43861 ggttttgttt tctttctttt ttggcagaag attagcagct gccaatttgc cagctttcag
43921 atcctccaaa gacacacaca cgcacacaca cacacacaca cacagtaaat gaaaatatgc
43981 atatgcccag tcacaatctg atagtttcaa acataagaaa cttcaatcag agcacttgca
44041 gtttctgtag ccatttatct gcaattcaaa gcgctgtggg tttctgcctc tgccaaatta
44101 caagctccat ttgccagcct aattaacggt tcgcagcgtt cagtgaaatt tattgcggcg
44161 gctcgggctc aagccaaagc aaacaggata gacctcacag atgaccgtaa actcggctat
44221 ggaggccgac accttgaaga aaaacaaact tgttacagcc gccgtgttca tacacttcga
44281 tttccctccc tctgcctctc cttgctccac tgtctcggct gacagttact tgactgtttc
44341 acttcacggt ctccgtggcg atggttgtta agaaggaaaa gccgggctcg catcctattg
44401 ctgttgactg caattcattg tgtgtttgtc agcggttgcc cagacggtca atactgttta
44461 gatgcacggc tgcggcgtgc atccagggag gaggaccgaa caatttcacc ctaatttaga
44521 gaactcgcac aagcaacctc cccatattca ggggaaatga acatctcgag aagaacattt
44581 tcaacacgtt acagaacagc cgtgttcgag ttggggttgg ggcacatata gtctgcttca
44641 gtcacatcat tcctatggaa accctcattg agtcaattaa ccccgacagt ttatagctaa
44701 agaaaatggt cataaagaag cttcctttgc ctctttcaca tcagtttgtg gccaaactga
44761 ttagagctcc aaataaatcg gaaaatagtt actcagagac gacagcagcc catctctctg
44821 gctcagggtt agtatatttc ctgaggacat taatgttctc ccctgtcttc tgcaaatgaa
44881 gccaaaggtt ttcctttggg caacagatct gtcctgttta ttagacaacc caaattagca
44941 ggacggggtt gaggttggga ctttaaggga tcttaaaaag gtaggagagc ctttaatgtg
45001 atggcttcat tgacaacaac aatgaaaaca acactaacaa caacaacatg aaacctctga
45061 aatatgcaag aagaatcttg gccaaacaca tcacataacc tataatttct ggcttattgt
45121 gctgtaataa actggtgcat cgggggnnnn nnnnnnnnnn nnnnnnnnnn nnnnnnnnnn
45181 nnnnnnnnnn nnnnnngggg gggggggggt gatcctttct tctaatccat caaagaaacg
45241 tggtttccac ttttgcacaa gcaactctgt ttgccacaga tacagttgct gaaagcacct
45301 tttggagtaa acccctgctt tacatgcctg atgctggttc cttattggaa cctgagagtc
45361 gtcttcttct tcttttgtat taattcattg attcaccccc acgccccccc cccccagcgt
45421 gtcccagacc tcactgctaa aatagacctt tgtgtttcct ttaaatagaa cacgtacctg

45481 cacatctgtt tcgaacatac atgcacgctg ctacgaattt aaaggcaaga tgaccaaaca
45541 gcatcttcag ctctctttct ctcaagcagc catcaggaaa attgcctctg agttgcgcca
45601 cgcgatccgc actccccccg atgtgcagcc accacgagtc gataaagaaa gcaacaaaga
45661 cgaacagatt gacagagcga aagcctaaaa aaagaagaga aaagaagaaa tcaagaaaag
45721 aggccctttt catgtttcta ggttagcgtt gcactacttt agcatgctac tcttgggaat
45781 ggcttttttt gttggtggtg gtggtgaaat cagtgtatca atcaaagcaa agtctaaatg
45841 agacacagag ggatgcaaac gtagctctgc tgctaattga tctatggttt gttcgaggaa
45901 ttaaaagagg aggctggctt cattagtgca aatgttctaa atctccaact ccggctcctc
45961 acttttgcgc cgaagggcac aaaaaaagaa gaaagaaagc agataaacag ataaatggga
46021 acagaggatt gatttgatgt gaatgaaggg tgaccatcat cctctaaaat gtgttttctc
46081 gcatctctaa cttttccccc tacctcacgg tgtgtctcct acaagtaggc cacattaaaa
46141 taagaccgta cgacaacaca tgtcacagca tgcgcgctcc taagagggtg gccattagtg
46201 gaaaagaaac tggaccggct ttaacttgtc agccggcttc aaagaaaact gtgtgggcct
46261 ctcgtaagcc tccccggctg atggagaatt attgattgtg tgttcagata tcttgaggcg
46321 ctgctgtata acactggagt ttaaattcag ccctaatgct cagcccatgc ataatttatg
46381 gagttaccat caaaaggtag tagcattgca aactaaagag gtctccagaa gcttttcctt
46441 tgtccccgtt gaaagttttc acacatcaac agtctcaaaa gctgggggta attttacagc
46501 aataggtcag gatcctcaaa cagcggggat aggagcggat caacgggtgg gcttccaggg
46561 gttcgcaact gtactgccac ttttacaatt tacgttcctg ccaacttatt aaccgagcgt
46621 ttccaattca tggtctattt tgttttgcag ttaatttctc atacatgcat aatcagggcg
46681 gcgcaaagga gcagagacac acaagcggtc ttctgggctt tactaatttt atgtttctgc
46741 ctctctgcct tgcatgctga atcgccctgc attagctgaa taatgaaagc accgttcatt
46801 tattgatctg tctgtctgta ttgaccaatg ctgctcccag ctatgctttt ccacatgcta
46861 cagacattta tttagatgta gagacccatt tacttgtaat tacaacccaa aagtgaaaaa
46921 tgaaggtgga aagaaaattc agactcccag aacccatcag aattatcgca gctgcccccc
46981 tgtctgcact aatggctgat tacctatgac tggttctata ttacacacag cagagctgca
47041 ttctgctcaa aaagcacctt aaaaaaacat ctttgtcatt ggacttgaaa ttagcactgt

47101 caattaaagc ttataatcag attaatcaca gggcggcttg ggtttaatca caattaatgc
47161 ccattcttct taattgtgtt ttactttgta tgggggaaac aaccatagat ccacttaatg
47221 ggtgctttgt ttaaccctaa aaacagattt ggtgtccacg tatcaacttc actgatgtct
47281 ttgcagataa atccttcatg ttcaacttct gttcacttcc acttcaggtt atgagtttca
47341 tcttgggttc ttttctaccc aaactttcca ccaggaagcc caccaggatg gctgtactgg
47401 tccattttga tggagttggg cctgcgaaca gacagtgttg aaaccctgat cctcgccatg
47461 gggagacatt gcaggatttg gattgtgctg aaatgtagat taaaatgtgt cgtttttcct
47521 ttagacagtc cagagaatga aaattaggct tcagcacaga gaagagaatt ctcatgcttt
47581 ttccataaat atgaagagtt tgttcataga agagatccac aggtgaagtt tgtgaatgac
47641 atatcgacct gttttccatt tacatcattc accacatcag cctctgacag tttctctata
47701 ttgtgatggt caaacaacag ttgacacctg gacaaacaag actctgagct aaaacacaat
47761 gctaatattc aagcctttga gttcttccag ctggtgggaa atttgaattc tcctccaact
47821 tttcattgcc tgcttcgctg tcatacgtgc aggactttta cggcagcatt tatcgccttg
47881 aaggtacgat gcgtgagtgt tccgtgtttc agcctcggcc ttgagcagct gctttaatat
47941 cagtccatca ccagatgtca ccattaaaac ggaacgcaac gtcttaaagc ttcaacacgt
48001 cgttggcaga aacacgtttc caaggtccat aaggaggctt catccaccta ctgctagttg
48061 gtggcatatg gttgaaagct cgtgaagcga tgccgcctgg actcctacat gcaggtattc
48121 actctttact gtccttttac tacgtttccc tccttgcatc cattactgag tcttggctaa
48181 tagttactca aagcagcact tgactgtcat aacagacgtc atgcttttat gaagtctgtg
48241 ttgatggttg gccccaactg ccaacgccag gcttcagtat ttaacggcag ataaatgaaa
48301 ctcaatgtca acatattttc tgccctaact gtacaatgga aacactgaaa gaggctcaac
48361 gtgacaccgc agagcgtgcc tgtggaaaaa cgtcattgcc tgtattaaat tctgaactgc
48421 tttatggagg caggtgagaa aaataaatct ggaaatgagc aagacttgtt tcaaagagac
48481 cgtgtggtga tatgataact cggatgtact ctgggtcagg tgcacccttta gttttactct
48541 gaaattacgc taaattttta taatccttca tggagggagg actccagatg aaaagtcact
48601 ttttaagaat cggttaatgc ggatttaatg tggagaatcg aaagttgaca gagttatgag
48661 gtctcttgag agccgtggag caaaaatata acacagtgca gacgctgttg agaatcacta

48721 atctggtttt tagacaaggt tattaatctg gcccttttcc agggagcgtt cctgctgtga
48781 gaaggcagaa ttcatgcatt actcctgtcc tgagtatgga aacgaataca aagacataaa
48841 aaaacacatg tttaaacttg agaaatttgc agtcaacccc tctaattacc cttaaggtag
48901 cactttattc tacatacact gcccattttc gagacagcga cagatttttc tcttcgagct
48961 gattatccat cattctatct gcacttcata tcgtcattaa aaaaaatgtg ctgatgaatg
49021 cgctttgcct tgagaggcct tgccaatggg ctacaccaca cttattagca ccctggacag
49081 accagtccat cgcagcacac acactcacgg tcaaacaacc tggggcaggt tagagtctcc
49141 aaacacccta acgtgcataa aaactccaca gaaaaatggc agctcctggc aaccatgcta
49201 acaatgcacg gattaactta taagacacac aaatgcacat aaccgaagcc gactggctga
49261 tataataacc tgcacttcaa agattctata ctgaaccatc ggtgtggaaa gtgtcctgct
49321 ttctatcctt tgaacagcac ctgacacttc tgatgagcct tgttcaacgc agacctcaac
49381 tttcattaat aaatgccact tgagcgcata tgctcgcaat ggaaatgtac actccaggcc
49441 gcatttaatc aaatcatgct aatcattgtg caaagggggt atacgggagg ggggtgggag
49501 gcatcaggca cagtagccgc atacagaata cataacaggt ggtggaacaa agcaaaagtt
49561 taaatgtttc tttttccgct ttccagacat acagctcatt atgccctcat gtgtgtctcg
49621 cgcagcagga aagggggctt tgacagtgcc cgtcctcatt actgcaagcg agcacctgag
49681 agatgagatg catcctgaca cctcgggctg attctcacct ccattaacac cggcaggggg
49741 tcaccgtgat ctgattacag gagggaatga agagggaaca gaaatgttgg gaaacaaggg
49801 gggatggaaa gagggggcgg tggtgggaga ggaaagggca gaagattgca tatatagggg
49861 atgtaccaaa ggggagagga aggatggaaa aaaggcgaga gtaaagtagc tggatgatga
49921 aggggtaagt cgtagctgct tggttggggg ttgatattag aggagcttgt ctggagtaca
49981 taggccagca gccgtggggc ctaatgatgg aggtcattgg catacacctc gatatgggcg
50041 actgcatgtg atcaatacca cctgatgagg tgtctgagtt ccctctgatg tgcatcaaaa
50101 tggaatatgt ttttttcctc ctcctttaat cgataggaag agtgtgaaac taattgggca
50161 aaattgcggg ggcatttatt gattaatcag gcagaaaaac gagcatatca ggaggcagag
50221 cctcgacatg tgagggcaac gacatggtgt tttgattggt gaagggaatc gatatctttg
50281 aatcattggc cggggcgtga aagttggaca ggcatgaaat atttaaaggt gccatggcgg

50341 aggcacctgc acatgcagga aatgcctgaa taaaaacaga ctggcatcaa ttttgaatgc
50401 cagattgtcc tatactggaa tgcacttgcg tggatgatgg gattctttcc tcctggaatg
50461 ttatctttct tttcccattc ttctcacata taggacatct tcaatatata cccagggtgg
50521 cgtgcttcag tatggagcat gccttataga ttcctgtatc tttctgccac catgacagga
50581 atctcacggt gcacgctccg ttttgcattg cgggaatgtg ttcaggaatg gtttgcacgt
50641 ggtgcaatat cacagcgggg tcctctgaag gacgagagct gtgaggctat aatctctttt
50701 ctctgtagcg ttgaaccccc agggagagtt ggatcaagcc acttgattca gtcttttagt
50761 tccactgtgg tgcactgcac tatgcaccag cacaaaagcc atctcccctg tcgaagaaga
50821 tggaaataat gttggtggcg gcgagaggga gcgtcaccag tccgccaatg tgttccactt
50881 caggaagcat tcctatttat ctgccctctg agaacatttg tagtgtgtag aaatgcggca
50941 accacgggga accgggggcc acccttgctg ccgatatgcg cctccctgtg aatggataga
51001 agggctgatg agatggacag gagtggaatc tgggatcaga ggagagtgtt cagactcaaa
51061 caataaggaa atcaaaggca acttcaacat ctttctggat tgaagcagga agaggacgaa
51121 gagactcaaa acaagggaag agtggaaatc aacgttgccc actgaggtcg ctggggtgtc
51181 tgactagaag aaccctgtca gtgactggga cacactattt tcttaaggag atgttaacgg
51241 tctagaggct actgggaggc ggttgatgaa tgtctggttc acgaattgac ctctctgtgg
51301 cctcaaaatg ctgctgattg cagagtgaag tcagcaggga ggcgagtggt ctgactctca
51361 cagctctcgg gctctgagtg cctgtcagtg gaaagactca aggccatatt attctgagca
51421 gaagcccatt gggtcttgtt gttttgaccc actacagtgc tgtttacata ctaatgagag
51481 atttactctt cgttcggcac cagagctgaa tagtatcatc gttttctgtt cacttgccca
51541 agagttgctc cacgtgtaca gcaaaatcca taaaaaagac agcaaattga acacaggctg
51601 catatttgga attttaaatg aattaacaca tgggcatgtt tatggatgcg gccgggtgag
51661 gtggtccgca aatcaataga gtttagttgc taaattaagc tggtaagcta actgacaccg
51721 ttcaatcaat acctaagcta aacaactgtc aagttttgtc aaatttcaca gacactaaaa
51781 aattttgtag cttctactaa taatgtagct attgaccaag tatgtaatgg ttgtttttgg
51841 actgaaccat cttgcattcc caattctgag taactgatct gtactctata atgacaactt
51901 ttaacttcct tgattcttat agatgttctc cttttaattt tttcttgcat atttaaaaaa

51961 aaaaatattg tcagatgtca agatgtcctt gttatgtgct acagactgag taacaccgcc
52021 ccaaactttt aaaatcactc gcaaaatatg gttgcaaccc aacacctgtg ctttatttcc
52081 actcagagag taactgtata tggactggag acactttgac atgtccacgt gttgaacgtg
52141 gccacaattc ctccctgacc accgggaaga gttttaacag atcggacttt ctcggtgtgt
52201 ttcataggct cctgcttttg ccatcaggac ctgcaaaggc atccggcaca gctccttcta
52261 gcagtgtggt gatcactaac ccaccgcacc accacctccg ccctccggtt tcaacctgtt
52321 acgagttaaa agccgacgga aggctttacg ctgaaagatt tcttacactc tttaatactt
52381 tcttcgacaa tccgggtgtt acgccgacta actccgcctc gcgctaggcc ggtaagcggc
52441 ccccagaaca ccgtcaagca aatctgtgaa cactcaaacc ttgggttcca gcaggcgaat
52501 ggtgatgtag gcacagggac tagaaaggca gacttacatt tacaacctgt ttgggatttg
52561 gctattgcac cgtgactaac acccctgctg aaccacatat ggcgttgaat tttaagtctg
52621 gcattctcca ggaggtgcag ggttagtaag agcagagaac aaatgccatc tgttaaataa
52681 acaaacacaa cgcacgccac ctccgttttc cgtgcaggag gaaaactttt caatccccac
52741 aatgaatgtg tgccattaca cctcagctcc agacggccaa ttatatttta ttttggtact
52801 taaaatttgc aaagggttgt tggatatcgc ccataatata tacactggct ggtttttgcc
52861 acacggctac tgcttaaaca ggccccttgg gggtcctgag gtgggaacgg aaggtaccac
52921 ctgcagattt ctcctcatac tctatgtgta ctaattacac actgacagac agagcagcga
52981 gcctgacagg ccatcgccgt ttggcatatg ccacacacaa atgctctgtg ccgcctcggc
53041 atccaaacga gtcctcggtc taacaactcg gttggaccgg ggccaaggaa gaaaatctaa
53101 gagcggtgag aggagggttt ttgggggca gagggaggat taatattaca tgcattttct
53161 aaaaagtgca agagggttat gacaagcaac acatgaacat tgggagaaag tttgtaggac
53221 tgtcacagag ctttgaacat gaactattta tgaggtagac agtaggtgga aaggatctta
53281 ccatgctggc caaaggcggt gtggtggctt tgtgtttgaa atttggattt tacataaacc
53341 tggcaggtgt cataatggct aagaataaac tgaggagcca aataatgaag cacgactacc
53401 cctccccttt ctccctttca cagccacggc ccatctgtgg gttcttttt cctgctttgt
53461 tttaactaca gacttggttt tctctttcct ctgtacggcc tgttgaattc atggttaaaa
53521 gctgtcacac atggtaatat cattgtatcc tgccattttt acgcagatga ggggggaatc

53581 tggggccagc gattgtatcc tcttatttac agtatcagct gcattaacag ggacactgct
53641 gacactttca cgggtgctca gacaatgaca atattttgat tccacctcaa atatgattga
53701 aatcaaaaca aagtcctttc ttacagctat tatacactgc tcaaaaaaca taaagtgtga
53761 tttaagatct gatgaatgac acatcccagt ttaaaatcca gaaacccaaa taatcagccc
53821 atttggggat aattgattgg cttcatgtct ggggaaggca ggccagtcaa tagcatcctg
53881 cctttgtcat ccagaactgc tgatgcactc cagctacatg agcctggcca ctgtcatcca
53941 gcagaaggaa tccaggtccg acattaggtc tgaaggtctc atcttggtgc ctaacagcag
54001 tcaggggacc tctggctaca tgtggaggtc ccaaggatat gcctcctcac accatctctg
54061 acccaatgcc aaagccatca tactggagca tgttgcagag gccggtggca aatcagccaa
54121 ttttgatgtc cactcataaa cacatatcgg gattttggag gtcattttgt agggctccaa
54181 cagtgctcct cctgctccct gcataaagaa gcagactgtg gccctgcttg tctgtctgat
54241 atcccctgta cgtttctgat gctgtgcttg aagagacaac aaagccagga gaggctggag
54301 aggctggaga ggctgacata cctgaaattg tgtgcgtgtg tgtgtgtgtg tgtgtgtgtg
54361 gggggggggg gggggctcac cccctgtaaa accatttctt atatctcaaa tgtaggacag
54421 agtgatagaa atatgatgat aatactcttc cttttatttt ttaaacactg cgttactttg
54481 tcctattgaa cagggaaaga gctttagccg ggcggaagat gcctttgttc aggaatcggc
54541 tattcagcaa tgcaaccgcc agatccaacg gagagattca aaagagtgac agagatagat
54601 tttgcagtga atgaagaggc gcccctcagt gaattcaaaa cagcatgtgt gctggtaaga
54661 ttcgacggcg taaagatgag ggaatgtgcc ttcttatcct caaggtgcgt ctgcgtgctc
54721 gtgcactggc gtgtctgcgc ctgatttcag aggctggtga aagagatggt ctcctctgtg
54781 acattcacca gctctgtcat ccaggtttga tatataacat gattgcatac acacaaaggg
54841 aaaatgcaca tgcacgctcc cacataacaa cgaatctggg ttccgatttg tctggctcca
54901 cgcgagcaca agcccggatg ggttgcgtga caccgccgca gtgttccagg atccctgcgg
54961 gcagccttgt gacgagacgc tcatcatcac tttttgcact ccaggtgaat ggcagggggg
55021 ggtcacgctg tgatgagcgg gacggagtgc aggagaggca atagacccga ggcctattgt
55081 ggagatggtg caatatccac atctgctcca gtggccactc aaacaggcac atggaagtgc
55141 acacacacag gagtgagaaa gctactgaga tgggcagtgc tgtcaccagt gattaccctc

55201 ctgccactct gacatggaga gattggggcc atatgaacgc ctctgtagcc ctaagcgata
55261 tttaggggca ggagctgcag agacgcggca gtggctgctt atgagaggtc ataatcagat
55321 gtgaaagctg tcttgtacgc ttacaggcag cattgtagcg aaaggcactg taatattgat
55381 gcagtttttc agcgggtaac gctaagagga cggaagaaaa ttggaaacac attagcaaca
55441 cctgatagct ttgagctcat gcagagcggc tcggaatatt aatcaccagg cgggaatgtc
55501 atcccgcttt ctgaaagaag gcaggaacca aagtgggaag cttgcgactc aataagcaca
55561 acctggaaaa aggggttttt gaatgaaaat aatagtctat taaattaaaa atgtatgcat
55621 tattatattt tgttcttatc aactatgcag caaaaacaag ttttttaaat ttcattttta
55681 ttccctgtgg ttttgacata gaaatctaaa tgcagactgt gaaacaggat gagatgtttt
55741 gttttccctg cttgtttccc cggagtcatc ggagtggaaa ctatttatta actagacgct
55801 gtgttggctg gtcagttgga agctctgtaa taggtgaggg gaaacgacta gtggaaccgt
55861 agatttattt gatgccactc tcaggttttc caatctagtg catcttgatg gttccagcgc
55921 tcacatatgg aggggctgga aatagcagag agcacatctc cggatgaaag gtgcctcaag
55981 ggtggctatc agggccgagg aacacaaagt aagcacctcg ctcacatgca caccctagcc
56041 ctccgggcct ttgcgagtga tgagaactga cagcgctttt tgtgcaatga ctactcctgc
56101 gtttgtgatc cacggctgct gtggcagccg tcatcacaaa cctccaacat ccgcccctga
56161 cagcgggggc caaaagatct ttctgaatca cagagcgagt acttccctgg tctaaatcaa
56221 gactctgggg acagggcgcc ataagaaaac aaacacagca gctttctaaa atgttgcatc
56281 tgcagattgc gagtggtgag cgtagaggag aagccgtcag gaatcaatga tatcagctaa
56341 aaatatgttc gacgggagat ttaaagggct atcggaaggt acccacttct tgaccagcat
56401 gcataattaa tatgtgcgtt agacacttaa caatggttaa tgattccaaa gtgctgacct
56461 gttaaatggc atacatttaa ggctgtcaaa tgaaggtctg gcactgtaat tttgtacagt
56521 tacactcatc aacctttacc tctcctccgt gagacacatt tatgttctaa tggctgatca
56581 catatgtaaa ttatgagata gcgctcactt taggatactg gtaaaagtca gagaaaccct
56641 gcagttcaac atcgcaagag caaaacatct aacagcaggc cgtgcatcgg ccgtcgtatt
56701 ttttgcgagc ttgtttacca taactggcta aacgtcacat caaggtgctg ctttttagtg
56761 gtatgatgat gagaaaccac gtcacttact gccacatcac tgccacatca ctgctattaa

56821 ttcctatatg caaatgtagg acacaataga cctcgccgtc attttaaata gcttgaaatg
56881 ataaattcat ccctataatt atggtcatga ttcaagtcaa tgagcaaaca aatttcatta
56941 atggattaaa agctaaaaat ggtctgttat ccagcactta gagggagagg attagcccac
57001 caccaaatta gacttatctt catatgtaaa tgaaaatgat taacaatcag tgcttcccct
57061 gaaagcagaa gcggcaaata aagaaagtta ctgaggtcga aataataata ttagctactg
57121 gagagaaatt agtttgggtt ttaacaacga aagcaaagtc aatcaaatgc aaatgcacgt
57181 gttcctgctg atttgtgtaa gtggaacaaa agacggggaa agcttaacat aatgttgtac
57241 aacacggcgg gaaaattcac ggcggatcca gcagaaataa ccataaagcc ataaactgtg
57301 tgtacaacag ggggtgtgag ataatgggta ttttttggca gagatgtcta catcagtttg
57361 gacctcttct ttttatattg gcaatctacg gtacttccgt gggctccagg acggcctgtg
57421 ctatctgcct catcttacat tgaggtgcct gttggcagct gctgccctta gcatcaaaat
57481 gcagcacttc ctctcagtgg cgccgtgcaa ctgggagccc ttatctcatc tccagcagca
57541 tgagctggtg gacctttggt gagggctggg gatggctggt gggggtggg ggggggcac
57601 actgtacctc ccctgtccct gcagctctct aacaacgctt cctctctggg tgaatggtct
57661 ctgagggctt aatttagact gtggatggtc acccctgcct cagattaatg ttggagcgct
57721 gggccaattc acagggattt gctctggcat gttcagcagg ctgtgttaaa ggtcttggag
57781 aaacaaactc tgacacgcat gtaatggtta tacaactgga ttcacttgta agatctgcct
57841 cgtggtcacc ggctccgctt cctgcctcac tgtccccgcg acaacactcg gacaaggcct
57901 ctgatgcatc tcccaggtgc agaatcctcc cctcaaattg gctgtgcagg cagaccgcgc
57961 tgttaatctc aaattcatga aggaaaggct ggatgaaatc ctaatccctt acctcgttat
58021 aaaaacatca tggcacaaat tatctacagt atggggccat agtcaaagtt gcttcttatc
58081 tccaggggcc caaaatgcac tctgactgac tgtgtcagag taagaaaaca tacaccctgg
58141 gaggactcac actgactcct cgtcaaagtt tcttagtgca gccagcttct tcggccaggg
58201 cttctctgaa gcggggagcc tggaggaagc ccttatccct ccacagctga ctgggaagga
58261 tcaagcaaaa ttggagcaag tcccatcaca aatacgaaat ctaaacaatt tattgtatta
58321 gagaaatcct gagccgatgg agcacattaa tttgctccct tggtgttatc aagatttcca
58381 catgtggatg caattccttt ttttgttgtt gtggacatcc cccctccctc ataaaaaaaa

58441 aacaaagaaa agaaaggcct tcagccaatt aaggtataaa gataatcatc catttaatta
58501 aatgcagcat ttagtgttgt tggatgaatt taaaacgaaa ccctcagtag tcactgcttg
58561 gatatttaga ctattacaga tatcatagaa tgggtgaaaa catcagttac ttcgaatact
58621 ggaggtcttt aactgagcta aaactgcagc aatagtcatg aaaccattaa aacattgcac
58681 gttttaaagt tggctctcgc cctataacta agtgtaacat gctccaaagc gctgtttgac
58741 agatgaaacc aaaattactg atgctctcac attctgaaat cctattacta tgaaagcctt
58801 ttataattga gttagaaaat ttcacagtct attactaatc acgcgcgcta acatgttcaa
58861 agtcggtttc catgtcagac actttctaat ccctcccttt aatgtatccg atccgacggt
58921 ccccttcatt agggggtcta taggttcctc ttcctctctg taatgaaagg agagtgtatg
58981 aaataaggat agagagtgtt tgctctggag agaaacagca aagttttttt ctctttagac
59041 tgtgtcctta ttagggcttt tctttccaaa gcaaggtcat agcatccacc aactttcagc
59101 ttgattagcc ttgggcttgt cctcgggcga ctaagtggct ctcacgcaac accacggaat
59161 ccaacctctt attgtgcctt atgcctccct ctgtttttca tgtttggcat tagatttctg
59221 cctctctctc gctctctcat cttcctttct gctgctcttg gtattgaagt cttttccaca
59281 cagcccggcg ctcgcctcca cctgagcagc tgctctggct tcttcctcca ctccgcctca
59341 ttagattctc cctggataac ctgagaaaaa gagctgctct gtcttcttct cctctccatc
59401 atagcccccg ccaccccgac cacaggattc atctgctggc gacggatgac ctctgacacc
59461 agcgcctcgc aggcaggatt accactgagt ccggcacagg ctttgttcct ggttgccgag
59521 ggagcggaga gagtctttaa tgaaattcaa actttgggcg tgagcagcca cagacttctg
59581 aagacctcac ttcttctcac ctcactcgca aatgtacggg atcaaaaaaa aaccttgagc
59641 caagctgtat ggaggaatgt tgctctgcga cgtgaatgca caatgattat gggaaacaaa
59701 gggggggttc aggtgagagc caaggctttc tgaaggtctc actcactcct ctttctatgg
59761 ccattccatt ttctttaccc aattacgaca aaaaacacca cacacaatat ggtgaaaaag
59821 ttacatccag aagggccaga ataaaccatc ctgtgtaaca gacgttaaca tctctttttt
59881 catttaggtt ttcatttagc accacgagga acgcaccgaa ctagctgagg ccgaggccac
59941 cgggtttatc agtctgtcag gcacatctgc ggagccatac atcttctcgc tcgaggtgcg
60001 gagccataat aactgcggat tccatatgtt tcccсgccat tcccagactg tgtcagtgaa

60061 gtttaagcgt tttcatttcc acgcggttca ccccccgtgc aacaccacag gtgcaggcct
60121 ccctcggtgc cagacgcggg ccgccggcat catgtgagcg gaagaatcag aagaagatcc
60181 aactggtgcg atcagctgtt cacgcacagg ccaatcacac gggtctgttt acttgagacg
60241 cataaaaaga gccacctggc aggaaaaata agtaaaagca ctttttaatg atggtaacta
60301 tatattgaca ccacagggtt gggagacgtg tgctgttagt cactgattaa cgagcgattc
60361 tttcaaaata gaaacgttta atcacgttaa atttcagcac gcaccttcag cctccaccat
60421 ccccgcagct ctgactggga aataaaaacc ctctgtgaaa tgtgggatga tgcttttctg
60481 ctggaaactg tacagagaaa accaacttgc ggggtggcag ctgttggagg atggtgtcgg
60541 cggcaccccg atcagtgggc aggtctcaaa aatagggcct cagttgatgt gagaagaaaa
60601 aaataaggta aagaagcaca gcagagtggc ctgctaaaaa gacaataatc agatccgaag
60661 gcttcatttg ttgacctcta caacaagcaa acgtgtgttt agatgtctta ttaaaggttg
60721 agcttgtttt ccacgtggtg ttttgacacc agactccaaa atctgtacga caaaaataaa
60781 aaataaaaat cctagaggcc tcatcaatca tcagggaggg ggggtccctt gctttaagct
60841 ctgctctggt tttgtcattg acaaaaaatg gctttccaca gaaaaatgaa ggagatcgca
60901 catgcgcgcg cgcacacata cacacacaca cacacacaca cacacacacg ctctctggtc
60961 ctgtattgat ttttatggcg gatggtgggg ctggtgggta gtggaggcac tgcactcaga
61021 gcagatgtct aatggtgttt agagactctc cactttatca agccaggcca gcactgacag
61081 ttagatgctg gagggcaatt aaaattaaag cctgcctgat tagaatatca tattcatctg
61141 gatggtaatt actatgtctg gctccatata aataattaat tatgcaaaac ccatgcagct
61201 gccactggct agagttaact gactttctgt caacgtctcg cagttcgaga aaggataaag
61261 agatttggga aataagctct ttagccgcta tctgcgagcc cgctggggtg cgcatgcagc
61321 gcccattgaa acagtggcag gctgccctcc gcgtgcaggt ggctccagca ccactttgcg
61381 gggttccctt ttgaagatgg atttggtttg cagggaaaag attgctttta aagacatcca
61441 ctttgggacg gcccgatatt tttcttttcg aaatgaaagt gttcgtgtga ccgtgccagc
61501 tgcagtcctc agccccgcct tagcctggtg ctgctgggtc tccgggacgg tgccagaaga
61561 ggtcattaca actgattggg ggggtggggg ggcagaaatg gacgagaggg catccaaatg
61621 ttacactcaa cgcattgtta ttctaatttc tgatcacgca aatataaaca attagaaaat

61681 attgaaaagg tgaagctcca atcatgttct cggtgtacac ttcattactg
tgaccttctc
61741 caaagacatc catagctctc aaattagctt catgctgtta aggactttaa
tatggtaatc
61801 aaatgcagaa ggttatataa gcagtgaagg ttattcccta tcacatgcta
gagagcagaa
61861 aatgataccg tctgtttcac aaactacttt ctttttcttt gttctgtttc
ggtgggttcg
61921 tttggagacc tcgccaccac agagaccctg gcagatctta aaagctgcat
ctgtgtgtcc
61981 tctgtacctc acaatatctt tttatttcat tgttatttca cagaaaactc
atataaaccc
62041 ctcttctttc tttgcacgtg catgcattga ggcgctattg ttttactaat
gttgttattg
62101 atttaaggtt ctgttgacca ctgggataag gtagttaatt acactcagca
atgttttact
62161 tgatgtatgc atgttttccc tgttagcctg gaggactctg tggtgaaaga
tgacgtgcat
62221 tctttgataa tcttttctca gtgggaggtt tggtgattat ggagatctca
aggctgaagc
62281 tgtgggatat aagctccata caggcctcgc gccgccgccg tggaactgaa
tacaataccc
62341 agagtgcttt gggagttcct agatgtacat ttttcattaa agaggttgtt
attttagagt
62401 ttttaaaagt ccagatatta gcaaaacgtc tttctgatga gcctatgagc
cagtttatat
62461 gaaagctgct gttgactaaa ccgacatccg ccatcctcaa catcagtcaa
tattgtcctt
62521 tctgtgctga cgattattat gtaaagcggg cccggatggc ggcccgcggc
tccattgtgg
62581 ctcccacaaa cactttttgg tgggcacctg gctctgctgg cacagtggac
ctcgcaacag
62641 ggaaggaggg tgaggttttg gctgcggttc tgagcttctg tttgcaaggt
cacagctgtc
62701 tcattggggg ctcccactgt cacgtctggc acggatgcag cgtgtgtgtg
tgtgtgtgtg
62761 tgtgtgtgtg tgtgcagcag gaaaagcttc ccaacttccc tgtgatggca
ctttcagaca
62821 gcgtgtttgc acatgttgtc tccccagcgc acagtcacac tactgggggg
ctattctgcg
62881 ccagtagggg gagaaggatt acaagcaaag gtggagaagg agccacgagg
aaccttacct
62941 atcagctcct gcagattgaa cctgtttgct acctccgcca acacaagggg
aggggctgag
63001 gtcttcatat gttaactttt ctttaaagtt ccacagggat ttgagatttc
ctgattgttc
63061 ctaaccatcc aattcagata aaaccagatc acgctgctgc tgacagatga
tcaggtcaat
63121 gcggtgatac agtatatcaa attaaacatc agctttaaaa tcaaatgaaa
tataaacaga
63181 caaaagccag gataaaatag aaagaagtaa agaacaaatg aattacatcc
acaggctcaa
63241 acggcaaaaa gcttcttggc agacgaaaga gatttaggca gcataaacct
taaaagtctg

63301 tgtgagtcag cctcaaacaa cccacatcta ggctggagcg ccaccccctta aagccttaaa
63361 cacaggcaac aatgtttgaa aagtctcagg taccactgaa gatgagccag gactcaggtg
63421 cccacatcct ttaggccagt ttaaagcctg gctgcagccg tgtgggccag ctccagattc
63481 tctttactga tatgcaagtc aaacacactg cgatagtcaa tgtctggagg aaagaaaggt
63541 acccagagct aatggccatg tcctccactg gcaagaggct ccatctgatc ctggtccaag
63601 gtttcttcct gttcacagtg agttgtccct gccattgctg cttgttcaaa attgaaaatg
63661 taaagctgtg tggctgatgg agtttggatc tgtcgaaacg ggatggacag actgctaaag
63721 tcccgaagct tcatctcctg ctcacctcct cgccacatcg gcatcccaaa acacaaacac
63781 aacccctgct gataatgagg agcgtccata cacccagaac aagatgaatg acagttatgt
63841 gagcatcgtg taaatgaagt tcctcacgca cactctcgcc tctcaatatc aacgctatct
63901 ttggactcgg cacaacacct gctgcgggca gatatcgacg agagaaaaga ggtgtgtgtg
63961 tgtgtgagcg agtgtgtgag agtgtatgtg ttgctggagg actgtggcaa ccgtccacgg
64021 agcacttccc ccgttgcttt gagggcgcgt taaaaaggca caattccagc caggggggtgg
64081 tgaaggtggg ggggatgtgg tgaaaggagt gcgtccaggg ttgtttgaag acttttgcga
64141 gaggagctgc gggagagttg aatcaagact gtttcatatc agatatgaca gatatgagat
64201 ttgcattgag ctttcctgaa cgagcctttc tgtcgggaag tgactgaggg tcgtaatggc
64261 cggtcccttc atggtggatg tggggggggg ggggttgcca attctggaaa taaaggaaaa
64321 gatggagaag cagctccgag tccagctcat aatgtggaaa aaactgtgtt tttctcttgc
64381 tgctgagtaa aaagaaaaat ggagaatcag tgacttcctg ctagactgtc tgaactgccg
64441 ccgcattccc atgttttccc tacaaccaat aaaagcacag aagatcccca caaatgttgc
64501 tctttatata cgtttgtgtg atcccattct atagcgaata agcacacaat cacacacaag
64561 aagagcgtgg agagtcacca gactgtgacc gttctcgttg aggcagagcg ccggtggatg
64621 tgctgttgat tccaattaca gaggcagcaa cttgctggag acgagcggca aaaccatgca
64681 agcggcccac actgcgattc catgtgttgt tgcaaacatc atccggctgt ggtgtcagat
64741 cagtttgaag gggctaagtg aagaaaatgg aaaaaaaagg agaaagagag agttccattt
64801 ccagccacgt aaacgccaca atacagtgtg tttcatcacc aaaggctgtg aggtgttgcg
64861 aggaaatatg aaacaggaac aggtctgcta attgaaacag tcacgctccc tggtttccat

64921 tagagtaatt ggcatatctg tttaggacat acacattctt tggaaaaggc aactgagctc
65981 taaatacagc caacggttcc aaagacttct tctggaaaat atccctaatt tacatcccaa
65041 ctatctgcaa caagatgctt cctgatgcat ctttagatgt ttttgtcgtc gttaccggtt
65101 acgatagttc ctttagacct cccaaaaacg ctgctttcca tcacatcgtc tgtttggctg
65161 tggtttgcct gcagggagcc acctgctcat caggagttgg aatccacgtg cattcgccaa
65221 cagatgaccc ggcatctgga gcaatttggg attcgggagc ctcgctcagg gacacggcgt
65281 gaatgccggc gaccagccct ccgccatcat tctccgaaca gatgtgttgc atggttctgt
65341 tacagaggat ctgatccagc ttctcctcag gtcttactgc aggatgcatc gttcaaaggt
65401 ctttgacaaa gttttatcag ggatccgtca tgagtgaccc aaaagacccg gttttgaggt
65461 cagcccggtg ggttcctgtg ggagagggct ggctgctcct caacagactc cctccccagg
65521 cctggctcca agatgagacc cagccatcct tgatttgtag tggttttgat gggttaaatt
65581 tgtggctgtt tggtcaattg aaagtgaaaa gcgtgaaacg tgtgatttca cttgactgtg
65641 agccagagcc aacacatgat gtcgcgtctg cgccgttatc aggacagaca agacagacaa
65701 ctcatctagt gaagctcgca ctgtcctgtt gcctctaaat gaacatttac tacttcatta
65761 tattttacat cacaggctag aacgcacgag ggctgcgagg acgtagccaa tgctaaattc
65821 cgcattagtt ccacgttaca gtcacttcgg ggttttatct atcatcttac tctttccagt
65881 aatttttgaa cccgagctgg agatttccca tcagcactca ctgggcttgg gggggtctaa
65941 tcttcgggat gacccggttg gaaatgtgca gatctttccc ccatctgctc cactcgcttt
66001 gtggcgcatt agcttgtggt gcccttgaac aaaaatgcca tcaaacaaac agcgcgatga
66061 ggaaatatga gtccacgcaa acacgtttgg acgcagagtt tttaatgacc accatccgtt
66121 gcatttgcaa tatggtattt ctgaccagcg gaggtccatt aacctctaaa gctaaccaac
66181 gatgaggcac gatcaggctg cttgttacaa tattaggctc tgaaaattga agaagatgat
66241 gaaggaggat gcagaggctg gcctaaaagg tgtgcctgca aaacagcagc cgagctgttt
66301 gaaaaaaatc agcattcttc ttcttttttt actatttgaa gtattaaaca atgcatatga
66361 tttaaaggac actcaatttt atgcattcta tacatcctat ttaaaaactg gaaaaacgtt
66421 cccctgcagg ttaagaagca acggagtcgt cttcttttca tccctcctcc tgatgactgg
66481 gggaatatcg gatgtggtta aagaagaatc tggatctttg tgctatgctg ccaccattcc

66541 aatgcgctga cggttagagc tgcatgtaat gacagctctg tatgctaaat gcagcaggaa
66601 atccaaataa cctcaccccc acacaaaata agccgtcctt gtgctttaat tgtttacacc
66661 agttggctgg gacggctctt ttctttaagg aactgtaccg ccgccgcctg agactctcat
66721 tgtgccggtt tgctgtgctg ctcgctcctt tcgtttctca catgtggagg cagctatctg
66781 gagcgtccat gctgctggat tatcccacta accaacaatc ctttcgcaac agttgagagg
66841 atgagagcgc ctgctggatc tcctccaggg tctactggtg cctcctctct tatactcgcc
66901 ttccagagcg gctccgggct gagaaccggg tcccgcatct catcggccca acagcgactg
66961 gcgcggggca gggagcgggg cccttctgag gcctgccatc tgctttgtca gtctgtccgt
67021 cagccgtttt ctcctctgaa ccgagcgcag gtgggagtac aacgttcttc agcagacaga
67081 aaaacggagg tgggatttgc atgcaggtat tgtaagggaa cagatgcacg gccgcggttt
67141 gagtgattac atgcgcatgt atgcaaatac aagcatgttt aagcataaaa acactctgag
67201 acacagagga accctcccac acactccttg tcgatgcagc gttcagactg gcgccggagc
67261 cccggctgcc ctacacaccc acatcagctg tgcaccaaag catctgggct ctgtgtggtc
67321 cgtctccatt cagtcggcgc cgccccggaa ccttccgcac ggccccgacg gcgacaaaat
67381 gagggttgtg ctcgtgtttg caagaaaagc tgatgagagt gaggactgct agtgatgggt
67441 agaggaggca ggtgacaagt ttcaggtgct ctgatattaa catctctttt tttcttgaaa
67501 tctttaaaaa aatgcaaagt gagagatatt agaatgtgtg gctccactca agagcccgtt
67561 ttctaaatca agctcgctta aaggttattt gtatgcattt ctcagaggct ggaccttggc
67621 gctgccgccg gtcccacgtt ggggatggac agattggctt ctaatggtta tccgatacgg
67681 aggcaggagg gtacctgcgt gttgtttaat aaggtggaca gatctttcac tgtggctata
67741 aatcaatgac actcgcagac agcgaagccc tgcacgcaga tgaaaaacgc acacggagtt
67801 acctgcagaa agctaatgca tttgtcaaaa ttcacacatg tgcatccccg actacacccc
67861 ccccctcaa gaaaataaat aaataaaaac aaaccatgct ttctgcctgt tgatgaagtc
67921 aaatccaatc atttcctgtt gctgcagcct ctgaaatgat ctttgcaaag tggaggcaga
67981 ggagacagga gcagatgcag aaagattgtg tttgattgcg ggtaaatggt ggatgtttga
68041 tgttgttcct gaaaggacct gctggccttt ctttgtcatt actgccttct gcctgagcgt
68101 aatggctgag gtacacatca gtaatgaaag cattatcaac agctgaatag aggaacaact

68161 gcgctgtcca gattccactc atttacctcc cagataatca tttacctctc cggcagcagc
68221 agaaatagct gcaaatatcc catggagcgc catcccgcca gtctgggaga cccatataaa
68281 aacaatgtat ttcttattgc tctctctctt tctccatctt cttaagcacg agtccaaagt
68341 aaaacagagg ctttcactgc aggaataaag tgcggagctt cttatcttgc tgctgctgtt
68401 ttccctatat cacaatctat tttctgcatc ataaacacaa atgaaaacaa taaacatccg
68461 caacgtagtt tatccttatg ggccctgctg ccaccggccc agttaggaac atagacctat
68521 gtggatgtgt gtgtgtgcgt gtgtgtgtgt gtttgggata cattccctga ctttctaata
68581 agccaccggc tcgatgccac cgctctcctg catgggggga gataagaact ggcagagcgg
68641 tggtgtgggc tcccattgaa agcccccccc ccacccagcc cccagcacaa gcccagaacg
68701 cacccacact cgcgcacgta ggcaagtccc aacaatagga ggaagcccca taccccgccc
68761 cccccccaaa ctttgcaatg aggtaacgct gagattacac atgcaagagc gcaggccgcg
68821 cactgggaat atgaggtgga gggagagagc gggggagagg ggagagatac aaaataacaa
68881 gaattaagtc caccgagacg gcatcagaaa aagcacaatg ctccgcttgt tgttggagaa
68941 cgagcggagc tactcaactg aagaatttcg acttctcttt attcctcgcg cagccgaaaa
69001 attaccaccc cagcgaagcc tgtcccgccc tcatttacca gtttctttat ttaaataaat
69061 atagttttac atgcttcagc aaagccgaca attactttat tgaaggtttt tttttagagt
69121 ttgcaggagc tgagggggac gtcatttgca tttctaacct tgttattggg gctgcggggg
69181 agttgagttc ccttctttaa ctgtttgaga ttaaccccat gcaaatggag cagatgccaa
69241 agtccaataa atcagcactt aagagagaga gcccacgtct gtggcagaca ttgttacgga
69301 ggccaatcgg atgcttccac acccgcccgt cggtgatggc gaaactgttg ccgctccatc
69361 tgtgggctgt tttgtgaggg agagacttga agtcgaccat tgtgatggag gacggctcca
69421 gagccattct gcctttgtgg ggaaattcta tcacctcatt agcacgttca ccagcgagtg
69481 accgtcagct gtcggccgta aaagtgagcc gcctcaggcc tcctcgtcac tctgctgtct
69541 ccccctgtgc tccctgcatt catttgtaaa tattttggcc gtagattagc aggaaaaaaa
69601 aagcttcttt gatggctaac aacgaccgct acggatcccg ttatcagcca gaatctgtgc
69661 gagcagttta atggaaaact aaatgtaggg aaatactggc tcagaattta acatccagaa
69721 acaaatatat ttccaaaata attaagttca atacaactca gtctgagggt atgggtaggt

69781 ggtaggggtt cagacaggag taaataaaac accagattaa ataaaagctg
ctcaattcat
69841 ttgcaggtcg cataaaagac taaaatgaca ttacgagcga cgagacgtgc
gactgagcga
69901 tgccgaggct gtgattcatt gcgctctgcg taaattggat gtttaatgga
gtgaaaagag
69961 cgttttgact gagccaacac cttcagaaag tcgcatttta attattggag
tgtgactttg
70021 agatccaatc atggcagcgt ttaaacttgt ttttttcata cttctacgtc
ctccggtttt
70081 ccccatccag ctccatcacg gcaccatttt ccctgttcgc gggcagcgtc
agactggagg
70141 aacttcgtat tttcacttct ccgcccgtct ctcatgcgat cgacataaat
tgctgtgttt
70201 ggactcgcga gggcatcgag ccgagacata accgcgcgcc ttgagctgag
agattcctaa
70261 aatatggaag aaaaaagatc ccagtgatgc aagttttgaa tactagccga
gctgaaggag
70321 ctcttctgct gcgtaaacac gtgttagctt gtatcgctgc gctagcgcct
ttgacagtcg
70381 gcagggccat actgatgcct gagaaagcag tgaaaatccc ccccccagga
attcttttaa
70441 caccagaacc tctccagagg agaggagaaa cactgaacgg cagtaaggag
gaaaaaaaac
70501 agcaggattg atgtggagga aaagcccaca gtgagatgta tacaggtggt
gagaatccgg
70561 ggggaggaaa aataagatca ggtttggagg atttagtagc tcagctcagc
tgtcaatatg
70621 atggggtgtt agagccgacc tgagtctgtg gagggagctg cctgtcaggg
cagtgatcag
70681 gtccgattta cagtttacag tttattcttt tccccaaaaa gcaggaaata
ttaggaggct
70741 acattcgtga tgtctcatta aaggaggaga aaaaaaggcc caaactctta
aagacttgaa
70801 ggagaactca tccatgcaag ggtttattca aaatggtgct ccgatgccaa
aaaaggcctt
70861 aatgagtcac aaatgtggct gcatatggct ttttaattag atgtcattac
ttccagaaca
70921 atggcaccag aaatggagca tatgggatca tcaacggaac ctcgcgagtc
tttggaagga
70981 tgaaacccac cttcctaata cgtccggtcc aatcaccaac ttgggaatct
gggtgagacc
71041 ctggcgccgc caacactcaa acaccctctg gatttgatga gaactggcca
aatttcatga
71101 gaactgccat taaatgccgg cccagtcagt gaaggaaaca gatttgtgac
gtttgacggt
71161 aaagtttgct ggattgaagc tctaacccga agcgcacggg gggtgggggg
gggggtgggg
71221 gggtgggggt ggtccgtgtt tctgtcctga ccacgtcaga aagatttccc
atctccaggc
71281 gctctcagct cagatgaggc agcctccatc ctggccctcc gcagctgttc
atctctctaa
71341 aaaggggtcc tgaaaactgt cagaggagtc ccgataggca gcgaccctgg
cgggcacgtt

71401 cccacattca ggcctgactt aaaacccaaa ccagcgatga aacactccag aacctgcaga
71461 actgctttga gatcaccgag aaagtgtgat ttgttcgtca cagcctccga ctggtggcag
71521 cgcctcatag ttttaccagt ttatcttgtg tggaccttga acatctttta ctgccatcat
71581 cagacgtcat ataacctgag ctactgacaa agtcaagccc attattgtgc aggcagagtt
71641 gcttggtgtc aacaaacacc aaagcagtgg aaagaagcca agaaagctcc ttcagtcttc
71701 catcagccat ctccacacag cactttcaga tctgaggcaa agtcggagga gagcgactcg
71761 ctttgttctg agtaaactgg atgaaataaa gcgaaaacaa gcagtcggaa gcaaaacaga
71821 acagctggat ttttaaagag atacagcacg tttggagcct aggatggctg atgtgactta
71881 gagccctccg tgattgaagc tcctgctgaa agtgtcactc gtctgttctg gggaggtacc
71941 ccagcagatc cggggagcgg cgccgaggac gggtcgtctc ggacgggtcg tctcggacac
72001 ttggaggctg ctggcctcgt gcagatgtgc tccccattcc ccgtttgcca tccctctgaa
72061 tcctccctgc atccctcctt taaggggccc ctggactgtc atgcatatca gtgctttatg
72121 agggatggag gagaggaagg aaagacccgg gggggggggg ggtcagtgga gggggcagtg
72181 aggcctgggc aacagttgga atcaggtcct caggattacg gctccagcaa gatgtgatca
72241 atagaacctg agcactaacc cctgccaggc gggccggggg ggtaaacgag agtccgaaaa
72301 agctgctccc caccatacca tatcccaatg cccccccct cccggttagt ctcacatggc
72361 aaaaggcaaa gatgttgttt ttgtctgagg agaagagggc agacactgct tcctgctcca
72421 ctgcttccta acaatgcagg gggagtgttg cgctgagctg agaggagcat ctacccatgt
72481 acccctgggg ggcttttgta ctgctgggtt tactccttcc ttgttgctga aactttttct
72541 tttaatcctg gccccccaaa aaaggccagt cgtgttagat ttggattgat attcaactca
72601 gaggaagctt ctgaaggagc tgacggagcc gtgaaagcag cggtggtctc cttctactca
72661 aggggagaaa taaaagtttc acgagtctat aatctaaaaa agatggttgg cagaaaaggc
72721 ccaagatact gacagcactt ttctcacatg tcggcaaata atgactgaat ttcaggaatt
72781 ggggtctact cttgaacccc cccccccctt ttctttaatg ccgacatgga gggggcaatc
72841 ccggccctca ccagcagatg gcagtgctgt aatcctcatg caggggcgtg cttatgcagg
72901 cagagctcag ggtgcgactt ggtggagaaa aaccatcacg ctatcagcca catggtctct
72961 ggggggcag tgaagttaca aatgttcaca catgctgccg cattacaatg cgacgcccta

73021 ttctgtgttg ccacaaaccc acagtttgag cagcttgtgg tgtaaatgaa atcacaggac
73081 ctccataaat gatttggcca tcccatcatg gtgttactaa tccttttaac agaggtgcat
73141 gtctctgagg gggtgtcttc ctcaggaggg actgtttaaa atcaaaaata gtgatctggt
73201 catccgatca actgacagca gctctctctt accttttcat tcactcccaa acacccatac
73261 agggacagac aggcatgcta aagtgcacgc taactaagca attaggcatt ttccacaccg
73321 ctgtttcaaa gcaaatgaat ccgcaggtta caagtcaaga agcgccacga ccagcgccgc
73381 aggtcaactc tggaaacgga gggttgaaca caaaccaaca cctgtattta ggtcgggctc
73441 catgtgggca gcagtggtga ttcatccaac tcttcattcc ccaataaacc cctttgcttt
73501 tgtttctaag aaggtatttt ccgaactgta ttcccacagg gcangacgtg ttttacataa
73561 tagcagtttc cttcagttta cagtggcgag agattcagag atgatgtaaa cagaattaca
73621 taagtgtgat ccgccgcagc gcttccagga tatgagaaac agaagcagag agaggaggaa
73681 ttcatgcgtg caccatcccc tcactgcgcc gccggcaggc ccgggaggac gtctgcgggc
73741 cggtgtgtct tgggcaggac ggtggccctg cgtgggggtc agcgcaccac acaggtcccc
73801 cagcgagccc acaaatcacc agtgggctga ggaggctctt aaacaagtta aagctgcatg
73861 tagaggcagc acaaaccgcc acaggtgttc caaaggtggc taaagacaca cacacacaca
73921 cacacacaca cacacacaca cacacacaca cacgttatgt gatctgaacg ttagccttca
73981 aaagccagac acttcctacg gtgaccagga ggggattttc taagcttttt cagttttatt
74041 agaactggac ctgctctttg aacacagaac acagagcctg gctcagagtc cattcctgaa
74101 aatgtcaaaa caatctatca ctcaccctca gcgcgtgtgt gtttccacta gattacaatt
74161 gtgaggcgtt cgggtccgtt tctggaagtc gctgcgtctg tattcaacaa ctgactgatt
74221 gacctgaacc tttattttcg atgtttaata tctggaaaag tttcataaaa atgggaaaat
74281 accccccccc ccttccacac acacacacag agaccacaca ctctgcatat ccagactgct
74341 ttcatctcct cctgttgact accgttatat cctgtggcgt gatcggacca caccagacaa
74401 caccgagcag cgtctgtatt cctccccagt tcgcttgttc cctgcctcca tccctctctc
74461 gcccacccgt tccctgcttc catagcccta caacaactcc ccccgaccca ctatcacccg
74521 tcccgtgttt gcttttcatt agctttcatc tctccagctt attgaactcc accgctggca
74581 tcaaagcggg atgctttgac ggtgtggccg ctcctccaca taaatccata tttacccctt

74641 tgctcatttc catgctaaca aatggtttgc cgttctcggg tgatgatacc accaagtgta
74701 tcatctgttc atagtgcgct gatgcgttgg cacagtcctg cggtatacag gggcacctga
74761 agtgctctcg aataaggtca acgtttgtat ctaaaccaag gctaacatgt atttatggat
74821 ttgtttcaca ccttaaaggg cactgggaga gagagagtaa tgtatgggcc attggaattc
74881 tgctatatgt aacatgaact ccgtccaacc ctttccaaac accaaacttg agtagttttc
74941 tatcacctaa tgtcacaaat tcagtttta ctccggtggt gtgttcaggc actgcactat
75001 gtgcccctaa atatcaacgc tggaggcaaa agtagtaact taaacatcag caacagcaca
75061 taaacatcac aggaggcgta ttgatggtat aatacacaat taaaagagga ggctaacttt
75121 tacacaaacg ttgtctccct cagttaagta attgtaatgt taactgggca acatcagcat
75181 ttagcattgc atgtgaatat gtcagtagct cctaaaaaac aaccattgag catttaaatc
75241 tgcgcactga atttgattct catagtgttt aaatgttgaa aacagcaaat atgtaaatat
75301 aaaaagataa ataaaacagt gtccaacctt tggaagtgga caacaaagtg aataaggtgt
75361 gatatttcag atttgtgtgc acggcagcta actagcgatg gtgttgtgta ttaaataaat
75421 caaacacaaa cctaattgta aagagctgtg actcgatgaa gttgtcggcc gcctgccacg
75481 atttcctctt tttcttttca gcacagttcc gaccgattct atcaaaagga tctacgtatg
75541 gatgaaatat gctgcctgct ttgaaatgct gaaggtggcg aacaattatt tatgaaaaat
75601 tggctcggag ttgcgtcatc ccatgaaaca cggcccggcc tgcacagccc ctttactggg
75661 aaagcagaaa gattttacgg gcggtgtctt gttaggaaga ggataagagt cggtctggag
75721 tattaattgt tttcctttgt gtgtcaaaca agccgggaaa caaggcgatt acaaaaattc
75781 gctgagaagt cggagcaaaa acccgacgtt agcttgtcta acttgcattg caaatgaggt
75841 aaagtgacgc tggcctttca tttgaagtcg aaccttttgt tgtcggctcc aactgtactc
75901 tgtttttaaa cccagcgagg gaccttttaa acgtttcaac ggggaattcg gctttgaata
75961 attcagcagg ctaatccagc gttgctagcg ctttggtctg tgtggcctgc tgttcggggc
76021 tgcagccgcc tcagagtcaa tacagagaga gacaggctga ggcagagaag cagaaagtcc
76081 atgaacgcag acagacacag acacagtgat ggggtactta atgctaatta gaatactctt
76141 aggattaggg gtttttggca ctacaggcgg tgccatgtta cactgtggct aacactgtca
76201 aacaacacat aatattagag gagaggtaag aactctagag tggaataaaa caaatctaga

76261 gttgaataca cacaatatgc taataatgca cgagcacagc ctggaacgat aataccatgc
76321 tgtatacaca tgttgcaatt aaagtgattt atcgcagtgc acctttaatt gtctttttga
76381 actgttcaat gatcccgtta atgttgtctg cttcactgtt tcccacagtt tgatcacatc
76441 cataccttca tttgatccct cctcgtgaag cacagctctg ttttgatacg ccggccgtac
76501 gctaaatcat tcctgattgc ctctctgaag gttgatacgc gtgttggtgc gcgtgtaatg
76561 aattaatgcg ctaaaagact taatctgcag gaaaaaaaaa gccacagctg tgagaacgta
76621 catcgtgttt gcatcatcag ccactctgat gttcctgaag aacctgaagg caacacgttc
76681 tgttaaactg cacctttcat tccaccgata acccaaacaa tggctttaaa gctatgctaa
76741 cgtccacttt aaaaagtggg gatgcagctc gtgcacaagg aagaagaatg actcttcctc
76801 tgaggggggg gcgaaaggtc ccaaactctg gagtccctcc tctaggaact gataaagttt
76861 cagcctcacc tcagcggatt taatgcatgc acactggagg atgcacgtct atgagtagga
76921 cgaaactcaa gagcttctta ttttacttca agttatgcaa aagccgactg cggagcagga
76981 aagatcaaaa aaggggcggc catagggcga gcacattagc agtcataacg agggccttta
77041 gcagcaactg cttaattatt gaaaggagaa acatctaaga tgctttaaag agcttctaat
77101 aatgcagctg gaaacagagc agcccggcac tgatgaagag tgcagcacag acaacaaagc
77161 acctttttaca ttgtagttaa agcgtttgtt gggctatttc acaatagccc cgagagctcg
77221 gcccaattag cagaaaggct tttgattagc ggctttgatt agcattgaca aatctctgtc
77281 tctctcgccg gtcgccatcg tcgccgtttt aaagctttga ttaccagata attcaatatt
77341 tgatgctttc agattgtgca cggtcagcaa cgacttggtt ccacggggcc ctttcattca
77401 taatatgccc ctgtttccgc accctcatcg ggccccacag ttcaagcgtg accctgtggg
77461 cttccaacac aacgctgttt tcattcgtgt tgactatttc cattatttcc acagctaaaa
77521 acactcaaag gtcatatttg aattgcttct gattggagaa aaatcggcac accagaggca
77581 ccaagactac ttctaaatac atgaaagcct tctgaagggt tagcgtagcc tctcaacaac
77641 aacagcaggg tcacttcata gagcctcccc ccaacctttg aaagctgata agaaattcag
77701 gcatcgaaca cagagccggt ttgtgaggcc aaactctcct gcgaccgaac acagagggag
77761 gaaagagggg ccgggggccg cattaatcca tgtcaggccg ggccaaatta tttatcacct
77821 tgtgcaaaca aacagtgtcc tggacagcct gtcggaggag gggggtgtgc acgtgaggtc

77881 cgtgagggct gccagcgacg ctgcagcctg accccatcac tcttcctctt
atctcccctc
77941 cttggccccc tcctcattac tccacacatg cagcctctga agctcccgtc
tccgtgcgca
78001 gggctgcacg gcaacggtgg ccgaggtctc acgtcttcgt ccccgacttc
ctccgactcc
78061 ttcctcgatt cacagctgga atctttgcgc cacacatcaa gaaattctcg
gcggagggcg
78121 cagacttccg cgcgagggcc aggccgcctt tgggtgccga ctttaaggtg
cgggagggga
78181 cgtagagagc cgggtggggg tggaggaggg gcggggtaag ggggctgcat
ctggactctg
78241 ggaagcagac agatcggcct ctctcaccca cagcatcgcc caggcagccc
gaggagctcc
78301 aatcaataag cagtccctgt ggctgccccg agcaagctgt ttgaaaagtc
agagtagcat
78361 gaaatggcca ttattaaatt actgggatgg gcgcatttag aaggccattg
cattaagaga
78421 gcaagctctc ccaattagtg gatcagcaca ttaatagccc tggatgagaa
caccatctga
78481 tcagagatta gagccacaca ctttctattg acccatttga ctgaggcact
ggtggccacg
78541 cagggccctt ccttttcttt taaagcataa cactcactca cgacgtccgg
cctgaataaa
78601 tggctggatt taaattcccg tcttgatgcg tgattcatat tttggaccat
tttctgctga
78661 atcaaacctc atccatttgc ctgcaaaggg actaaaggaa ctgccactaa
atcaactttt
78721 ttttttctta agagggaaaa taagagctat actttatgtt cttgggtgcc
ctgaaagcct
78781 gaggctcatg ttaaaaatga cagcgaggag gcgcattgct gcgaaaactc
ataattctag
78841 tgtgactcgt tgagaaatag aggctctccc ggctcagtga cctgcttatg
ttaaggatat
78901 attaggtgta tttccaagtc tgaaggggta aaaagagaaa acgcatatgt
gaaactcaac
78961 tccgggtctt tatgcaggac tggaacaggt ctggtttaag tttcttgtac
tggcactgaa
79021 tcctcctctg agcaggagga ttcagtcagt ggttcccact cacagtgggc
acctttcttg
79081 ggcttttctt ctatttgagg gaggctttta agaggaatta tttaccctag
agggacacag
79141 ccgtggatgc cagcacttgt cttgaaggag caccttaaag ttacagtcgg
tcatgcagga
79201 gtacttgggc taacctcagg gtcaggccaa cagctgagaa ttagcgtttc
cctccggtac
79261 ttttcggcat ggttgcaggg cctgtgagcg cacaacagag gctgagtgca
cggaggcaga
79321 ggttacacag tacctgaact agagagatgc caggatcatt acttaactct
tcagggttgg
79381 atccaccccc agtcctgtct ctccacagca ccagcaccag cgccgctatc
acatatcaat
79441 atcgagcgct caaacctctc ccacctttag actgtgtttt gctttcttag
tcaccctaaa

79501 ggcaggaatg tttccaacag gccatgtaaa ttgtgtgcct gcgtgtttac ctctgagtgt
79561 gtgtgtgtgt gtgtgtgtgt gtgtgtgtgt gtgtgtgtgt gtgtattctc gcctgtattg
79621 attacacatt tatattcacc cacacagaac agtatccagt ttcacttgtt agccattgac
79681 cttttggaat gtgaactact tactacgtat atagaacttg tagtatattt atatatgaga
79741 cacagtatat cattattgtt cttgtgttat ttctaagcag gaagttggat tttcttcttg
79801 ggtgtttcat atgatgtcta ttataccttt tttattattc gttttagtac tttttagcta
79861 aaataaagaa catgtttcca taaaaataac atatccctca aacctttta ataaattggg
79921 attgtcttca cagcataagt tctagcaaaa gaaaaccaga agtagaactc atcctttcat
79981 gacctagctt gtaggtctgg gtttaccgct gttccttaag ccttagactt tttcagcgat
80041 attgtagata aattctacaa aggttccgag ctaccaaaaa accccatctc agctgacaca
80101 actgtgtcaa gactccatgt gctgtaggta aaacctgtag aagcattcat ggatatcctc
80161 ctcgatcccc ttttgggtat gttcaagggt tgtacctaca attccacccc ttgaatggga
80221 tggaccaaaa aaaaggtcaa ctaaatggtc ttacagtctt acatcagaat cctccaatct
80281 gattctactg ggaaccacat caaccagccc atcaggcttg gacataaata tttcagcctt
80341 gaggagcacc agcgcactgc tgttgttgcc gccctctgtg gtttactggg ttcagtcctt
80401 ccacatctga ttggcaggct tcatcttgaa gccacagagg tcgtgctgct atctctgata
80461 ccgatgcaaa tctcccctcc ccatttcctc cccaacatcc tcttctgttt atcaaccaca
80521 tacgagcaac aacaccgcgt acgcctgctg ctggcccaca tggaagtggc ccagatctgc
80581 tggtgaaagg agggagctgt ggaagcctgc tgccaggacc agggccaagg tctgatcccc
80641 agtcggctgg cgtcattcca gcccggagga gcgagtgttc tacagcagag caaagcatgg
80701 gggctctgca ccccccgcc accatcaccg ccatccccc tgaggatgat ggatgctccg
80761 tcctctccct acagcagggg ggaagcatta gacatccgta taattgactg aggatgagag
80821 aaataaaagg atggaaagag agaaaaagga aagacagatt gcaaaggtgt gtgtttggac
80881 ttgagtgaga tattggactc agagtgtgtg ttttattcat ttcataaaca gcaacaaaca
80941 acgtggaaga tatcacaaga caaaaagaca cgggaaaatt gagggaacag acggagaggt
81001 agggagctgg cgcagctgaa aatggctccc gctcaacagg acatcctttc acccacatta
81061 cacatgtcct cgccaatcct ctcaacgaga atcacggcta cttgtgtcga aaacggcccc

81121 gcatccagga cggacagaac tacggaggca ctgcctggcc gagcacgagc
atcgtcccga
81181 aatctccagg caaccaagtg attgagtatg gcttactctc acaataggac
aaggttttgg
81241 ctcgtgatcg aagggccaag catttagaaa agcctttgtg tggagtgcac
catcaccccg
81301 aagagatgac tgaagggggg ccctgaccaa taaacccata ttagttccag
aaaggcgtgt
81361 tcctttgcac tcgctagagc catagacctc cgctgcctct gaaagtgcca
cccaatggcc
81421 ccgtctgaag aactaagtgc tggtctgcat ccacgtatca aagtgagagc
gagagctgct
81481 gcacaacttc caaaggaaaa taatacaatt ataggagtac acagacttgt
tcattattga
81541 tggcgctgct tttcaacggc ctcgccgaaa agaccccgtc aataatttat
caccatggag
81601 acgcatgcca agtcagtctg cttggacaca tcatgaatat caaataaata
tgcaaaacaa
81661 agggggggata catcattaag cacactttaa acgcaggttt gtaaaaaaaa
aaaatcaaaa
81721 aaatgcaact ccttgattgt tttttgcacc gtaacatcaa tcacagagct
tgaatttgat
81781 ctgttatggc tgaaagttct ccattaatca ctcagatgtc tgtaccactc
cgagcgcgat
81841 gatcggttta agggaaacgg gcggaggggg aatagcgcgg ctgttaggtt
atgctttaat
81901 gacgatcctc catcacttat taccaagaca atgttcctca tcagttaatt
gaataagtta
81961 ccgaatggag ccccacgcat cctgcgattg tggagtcatt cgcatcatta
gaagatgatg
82021 aactccagat aagaggtctg agtgatcctc tctctttatc actgctaaca
gcctgacgag
82081 acgtggaagc ggagagaatt ggagtgggag gctcctgatg tgacagtgac
agtcattaat
82141 gctggagcga cggtcacgtg tgaatacatc gactctacgg tgaaggattc
attatttaat
82201 agtgcttcag gtcgctgctt agcttgaagg caatgtgagg gaaggatgtg
cacattttag
82261 agagaacaac gataatcact gtaactacaa atgttctttc acggattgtt
actggactgg
82321 tggactatgt cttaccaaaa tctcaacaac aacaagtgaa ataagtaaat
atatttgctc
82381 cactcggcag tttgtaatgg ttccagataa atggttcctg caccagcgat
gcaacagcat
82441 tctataatcc acaacataaa ctgttaatag gcagggttga tgcaggtttg
gcagatccga
82501 taggacgtcg cggagcagct agctgtcacg ttgcctcgct gataagcccc
atgcaaatga
82561 ctggatctcc cagaagacct ccactgctca tttggtttgc tgcatgattt
ggttgcagtt
82621 ttgcacctca gacagctgtc tcgtctccgc ctcgtgtaca gtagcgcttg
tttttaatag
82681 ccctttatct gcgcatcgca tgttactgaa gtcgccaaac aaatcttgtt
tcgtgttcct

82741 ccattttgag aacactggag cctgaatgcc acgaagaacc gctgccagca
ttctgatacg
82801 ccacggagcg ccgcaaactg tgtctcacat gcatttttgt gtccatctct
ctattctctc
82861 cgggtggaat tcgtgtttgt ggatgcacaa cacatcctgt tagcttagcc
gggtcagagg
82921 tgagggtcac cgcgtgtgtg cgtgcgattg cctgtatgag cgtgtgtgtg
catgtttatc
82981 aggaattgca gatatgggac catttgggga gtctcaaagc aagcaggtga
gagtcatgct
83041 gggtgttctc tcggttcagt gctaatggag ttcaatccgg atccctcctc
gcccctcagt
83101 tcccagtgag caatcattat gggtttgcta atttaggctt aggtctttgc
aatggggggg
83161 agggggtggg gggttctata tgcattacgg ctatcatgac aaaaggtaca
gtacagttgt
83221 atccagatat ctaggttgtg tatgttgttt taatgattct ttcacgctga
agattcattg
83281 atatggaggt tatggtctca ttcaaagtca tgtatcccag tatactgttg
gttaaggcac
83341 acatggacat ggaagtcgac catgctagcc gatgttagct gagtggattc
tcttatcttc
83401 ctggcaaaac ctgaaacaaa tgctccattt aagctgtcta aacctgcgta
ggtttattgc
83461 tccctctcaa agccatttat aaagccacct ccatcccaga tcagtctgac
tctactctga
83521 ttagctcaat atcccatccc tgctgtctgt cctatgctgt cctgaacatt
cctgttgctt
83581 ctccattcca tggcagcatg tggaaagtca gggaggtaga gcagaacgcc
ccagagaagt
83641 catgccacca aatacataaa aatgtgattc tgactgtgaa attacagcgc
agtctggctt
83701 aatggctgaa aaaacatacc tcatttttat aatatatgct gtgtagagaa
ccatagatgc
83761 aacaccgata aatggacgaa cttcaggatt agattttaca gttgaagatc
ttggaattag
83821 ttgcttctct tctccctttc agaggagcgc cgctgatccg ctgccatctc
tttaccgcag
83881 caaatgcacg ctgcatgtgc cgcagcagat gcaccttttc atcagccttc
ctttgagcct
83941 gtctccgcga tgtgggctcc atttcatttg tggcagcatc aaggtaaagg
aggccggatc
84001 tgaaagactg gacccgttcc agcacttggg tcaataacgg gtgcctgcta
atcacatcct
84061 catagatggg aaaagctccg agcacgtgta caccaccagc ggtacttatt
tgtgtgtgtt
84121 gctgttttct ttaggactag catcttgcgg tgtctgctat atgagtaaag
gctgtgttaa
84181 cgcagggctg aatgaaccag gattgatggc gccgtggcac ctaatctaac
ccacagtgaa
84241 taagctgcat cttctttaca ttgtctgata atctttcaac tcccaattac
tgtcagaatg
84301 tcctggttct aatgatactc caacagcaaa tacatttcca gtaaacaatg
aagatcaaat

84361 acaaacaaaa gtgtgtttta cgagttagtg ggtctgaaat atccgtcttt agctatagaa
84421 tcatttcatg aaatgatcca ctcgctcctt gttggtgact gcagcagctg gcagacgtgc
84481 acattaaact ttgcaccatt agatattctt gacatgaaaa agacttttaa ggttagaggg
84541 gtccactggt tctgctctgc cagtaaggga ggtgggctac cggggtgggt gaacgctgct
84601 gcacaaggcc gaggggagcc cagcgtccag ggcgcgctga gccgccactc atttgcggac
84661 gcttgtgcgg tgcataacaa tccagaccga tctgtcatta acatctgcag ccaccgccgc
84721 tatgaatgac taatgacctg aacacacaaa ttggatgaga aaggcaaaca acccctggat
84781 gctcccagtg atcatgaata acaaagcgcg gacttttcgg ttccttggga attcagtcgg
84841 cggacacagg cagtgtacat tcttggtaac aagttccact gtcttgggtt tcctctaaag
84901 aatcaaagac ttggaactga aaaaccaccc tcatattctc aggaaccact ttattaaagc
84961 tttgtgaggt acgctcccaa aaggacctta aacaatgaca aacccgctgg agtcacacag
85021 gagaagcaga agcaacacaa tcaactcagc tctattcatc tgcttcccct tgtttctttt
85081 tgcttacggg caacgcaaaa aacacaaaag catattatct ccaccagctg aagatcagac
85141 gttctgcaga cgttcagcca gtcaaagtga tggatgtaaa gaagaagtga agggctgatc
85201 cggacttaag attggatgag tggaacaacc ccctagtgta ccgctggacc tctcctctcc
85261 agcctcccca tggaagtcca tcccggtcag cggaggttct gttccgcctg catgcgtcac
85321 gtgtgctgaa cgatcacccc gactcacgcg gttctgctct ccacctctct tccgggtgtt
85381 gcgccgggct ttcatcatcc agggcgccgg cttggcaggc gcttcctctt attagaaaat
85441 gaagacgggg attgagccga tgttaaaaag gaagccccct ttatatcaac ccgtgatgag
85501 gagcctgagg tgagcacaag gttaaccaaa tcccgttgga cctgcatcat cccagctgtc
85561 ccgctggacg ccctgcaccc cggcgtcgcc ccatatccac acgtggcagt tttgtaagta
85621 caaccgaggc aggagcggcg cctctctgcc ttaaatcgct gtgaaattta cttctctcgt
85681 tagagcggca gcccggtttt ttggccgcag gctgagacga ggcatctgag gtatttctgg
85741 gtattcagcg ttggactcgt gcgatgctct ctcctgacag tgactttgtc tgacagcaga
85801 tataatgtga tttctcccgt ccccggggtc cagggccgat gatggagact tctggggtct
85861 gccttgctcg ccggcgacag ccgtgacaga aagcggggga gaaaaaaacc cacttgagct
85921 gatgatcggt ggcatttctc acacgactcg gagatccctc agtctgtccc cctgctgccc

85981 agcatcagag agagaaacca ggtccgaatg ttcggcgttt cccctcacac acgagccaac
86041 tcacccgcta cccaccgatt tctacgcctt catcttatag accttcgtat tttgggcttg
86101 ttctgcagcg tcgtcgcaca ttagtgcagg agagcaaagg ggacgcccgt gtcgctccta
86161 ctgtaagcgt gtcacatcag gtaaacccgc gtgtgtcata cccgcgggcc acacacgggc
86221 ctctgtctct gcagcttatc cacaaaaaca aagctgatta tttctgtcgt gcgtctttta
86281 tctgctttag tttaaaaaaa aaacaaaaaa aagctgcctg aagttttggg gagaggcagt
86341 gcagctctaa acgggcttgt tcttttcttc ttataaggac ctcctgctgt gaggtgacat
86401 cctgctgtgt ttttggcttc ttcccttttt cctgtttttt ctaacaaatc agggtgggag
86461 ggtgagctgc agccggcctt tgtttaccag gggcccgttg acacgacaac caaaaaggct
86521 ggaaacggcg aaggtttgca aatgtctgac tccgcaaact ttcaaaaact gggcttcgga
86581 ggaaaaagat gtgcaaacgt gtaaacaggg agtggtgttt tcattcagtt tcaatagctg
86641 tgaccaaata ttgggcagcg ctcgtgccgc tgtactcgct gtgaaatggg gatttgtctc
86701 atcaatactc tgatacttcg gcattgcttt ggttatatta gtcaaggacg gaatcaatat
86761 ttatattttc agttgttatc gtggatctgt gtgagttcgt atcgtttaca caccaggtcg
86821 tgtcatgtgg ttaagacttg tgtgctaatt tagccaaact ctacgtggtc tttcaacatc
86881 ccagtgatct gggagaatgc agagaacaca ctggacgagg tcatttcctg tttcctgtgg
86941 ggactttatt acaggtgtga ctgccctatt tttagcagta cacaactccc acatcattga
87001 atattaaaca tttggaattc ttccctcatt gagtctttga catctgattt agatgcactt
87061 gtatctgtgt aaaacatttc tgatattacc accgttgcac aaaggaataa agataaaccc
87121 aaaaaaaccc cagaaagttt tagtcactga atgttgacag tttatgacag aaaagaacat
87181 gtggcaaagg caaatgttaa tgtggtgctc aacacatttt cagggcacat gggtgcagca
87241 ttcatgccac atgggaaatt ctttaacgtc tgtgcgcagc gctttgaaga aaaatgatac
87301 cggtcgagtt gtaattactg ccagagtaaa gggcacgggg aaaaaaatga agtccctgct
87361 cttcaaggga agagggaggg gtagttctga cggctggcaa gtttggagtt tcggagtgtt
87421 tcgaaggtca gcgtgtttta cctttgtgta gaggtattgt tgttattgtt gttattattt
87481 tgacctggct aacgtgtcca tgttgtgtta gtatcacaca tttcagaagt ggtgctggca
87541 agagttgggt agtgagcttt gctacattga tcctgaaatt tgtacctggt taaacaagtc

87601 ttgctgaact ttcagggtag tgggctccat taaaactcca cggtctgcac tgctagagct
87661 ttgatttcta tttaaaatat atagtttttc aaagtgaaaa tagtttttca cagctctgtc
87721 cctggctgga gccggagttc aaagatgaaa caaatcatta aatgaacgct ggacccaggc
87781 gtgaggaagc acaagctaat aaaaccaggg aacatcggag tcaagcatac tgggaaaaga
87841 cactgtcacg aaggccgccc agcagggact ccactgctgc tcctggcgct gattcttatc
87901 tgctcgggtc ctgcatcaaa attgctggca tctgctgggg actgtacacc gggggcctgt
87961 gccaggcagc tctctgacct ttgacctagc cgcatcgtga tcacaggcgc cgcacacagg
88021 gggagagaca caggtttata tagctcccac cagctctggt gcatctccaa gacacctgac
88081 aaaagattaa gaaagacctt cttcagctgc ctgtaaacag tgccctcacc atgcgctaac
88141 ccagttagcc tcatgctaac tggaaatgtt tcttcttttg ttgttgtata atatttattt
88201 taaaaaaaaa tcatgtgacc aaattaaaaa cacgtgtgac tttttgccta ttatgagagt
88261 agttacaaca gctgctagga tgctaatgct aaatcaagtg ctatgttttg cataatatcg
88321 catccactgt tttttgccct gacaaatata tcgtcccact actactttta cttttcagaa
88381 tacaggaaga cgacatatta gctgcttgtg gtccactgag ttttgactgt ctaatccatt
88441 aaagtagcct ttgaaagcaa gaccagacag cttcaacttg tctgtcacct cttccacaca
88501 cacacacaca cacacacaca cacggccaca tgaataagtc gtagggattt atttcacact
88561 aataactccc tcggtagtgt acaaatgcag ctcgagtcct tgttacaggc tccagacaac
88621 atttagacga tcacagctca tgaagcacca agtcccagct tccttcctcc ctcgcttcct
88681 tccggcgttt tattccatct aaataatgca gccacaagca gtcattgggg ggatccatta
88741 tgggaggact gatatagaat gctgggatgc atgatcagac tctgttatca ctttcacaga
88801 gcatgttatg gtgcagtggg gagacctttg tattgtcggt ctcttctaag tgcttaactg
88861 tttgcttgat gtgatagcat tatgtaatct gaacaggtgg gggacatatt acagtattcc
88921 ccaatctaca taacatccag atgatgtatg attggttgtg ggtatgtgct cacggagatc
88981 ttaaagggcg tttcgttggg ggcctgggag gaattcactt tgggctggaa ccgctggtgt
89041 gaagtgagcg gctacagccg tcacggttgg tgagagaaaa ttaaaacagc ttttccacca
89101 gccagtcatt ggataaagaa ctatcattca tccagaggga agctgcaaaa tcaagagtga
89161 aaacaaaaca gcgctagcag agcagcaggg ggccctgcca ggcaccttga cgtttattca

89221 ccgcgttagc tgcttctctg agaggagact tttaattaca tgtaatctac
cggagaacaa
89281 acgagtctga cggtggcacg ggagggagag cgcgcgtctg cgtgggcgtg
cgcgctaaca
89341 ggtggagccc gcgatgctct ctctgtttcc cactaatctg cctgtcagta
atgaagtcaa
89401 aggcttaagt gttggagaga gcaggcgtca tccattagtc tcatccattt
ccattaatgt
89461 tcgcggatta cttattattc cctgattaat aaacactctg cgagctcgtc
cttatagcag
89521 cctcaataag gacggcttca ttaatgatta gcactgaagc taatattcca
gttcttggca
89581 gcagctcccc tcgtgttatt ttgagtgttt ctagagcatc gacggttgat
gttagcctca
89641 gcttcccagg taatattcca caaacaactc acattacctc agtatttaga
catcggtgag
89701 cgaaaacaag taatccaatt agcaaactca gtgaggcgct attgatttca
taaaaaaaaa
89761 atccctaatc aatttttgag agggaataat gaaatcctcc tacttagtgc
tccacatcag
89821 caagagagag cgcatttgcg cacatatgtg cacgtgtaca ctgtttgaaa
actgcataaa
89881 tctacgttag gcatgcccac cagaacacac ccggaggttc ctcacctctg
cgcgtgcaca
89941 aagtgcatgg atataaagag gaatataatt tagataaaaa tatgagctca
tacttctgtt
90001 tggcccaaac cagcccgaca ccggccgcac cctgagtggc agaaccggcg
gcatcctaaa
90061 tattcatgct aaacatgtag cgctgccact ggaactctta gagggattat
atattggaca
90121 caattagatt gtaggggagt ggtatgaata gcctcaggat gtttgcatga
attacagcgt
90181 gcaggaaact actgtaatgc actaatccca tgaatagtcg gctctgcctc
gtcttatttt
90241 acaccgggcc caaggtcttt tcaaccacct caccggtgag ccagattcca
aaattttcaa
90301 gggtgaaaac tattgatcgt atgcaaacgg cattaatctg aggcgcagcc
atatttcttt
90361 cactccctgc ttgaaatcag tggcgttcac gtgcgcattc caatccaatt
atatagataa
90421 atagactaat catgccacaa aatttcaata tgaggtcttc aggcagcctg
ataagcagaa
90481 tcattcccag tgccaaattt acagcacaat tttagataag aaagatggaa
ggcaagagtc
90541 agggagggag agagggtgca aattagacag ataaatagtt tcatattaga
tatcgtcctg
90601 tggatcgtaa tggtggagac atttaattaa tggaaataaa cattcaatta
aaagtagttt
90661 tttttgttcg ctccattcgc cgtcgttcac aggattgtga cgggtccaag
tgttacggag
90721 acagtttgta gagggtgaca aattggaatt ccaaaaggct ccatccggcc
tgtgagtggg
90781 gggggggagga agatttacgg gagacactga ccccacctca gcgtcccaga
tcaaagtggc

90841 ctcattcaga ggtgtggaaa tgattcttct gataaagaat ctaacaatta caacaataat
90901 tgtttgctaa cccacttcag ttggaagaga cttggtggac tatttcacaa cattctgttc
90961 aacttttaaa agcagctttt tatgctcact gtgtgaaata gatccagaca tcatctgcat
91021 ccaatattga cctaatgcgt gttgctgcag agcaactgtg actaacgaag ggacgagtag
91081 cgttagctgt ttaccaaaag gggggaaacc aaaaccaaat caattttgca gtgatttttg
91141 tgaaactata aatgtgaact aattagtgtg tgaaaaatgg gacaggatac tcgtcatgtt
91201 caggcccctg ttttaaaagt ataaatgcat ttttaaactc tttcatactt gcgaagacga
91261 ggctcttgga taattcaaag atcctgcagg aggaaacgtg ctcagtgctt ctctctcagt
91321 cccgatgtgt tggtttctaa tctgtctgta tgtgtgacat gtttagtacc ataatactca
91381 ttccactagc aaagtgagga ccttttgtg atgtggggcc gtttctgcca tttgccacag
91441 cttcaaagac ccttttgtgg gtcaagccat ggctttaggg tgggggtgta ggttaggggt
91501 taggggagga ggaggagctg ggtgatgcat tattattcca gaaagtctcc gcaaagatcg
91561 aaacatgagg ataagagtcg gctcacggtg cctcggacat tcttccggag tccacgcgac
91621 cgtctcagac taaacacaga gcagcaggac ttcattcatg gtggccccac cttcagctcc
91681 agccaggacc tccacagagg aggaggccag agcatctgta gcaggcctcc aaagaatgaa
91741 tcaataaaag aggggaaaaa aggaacatgt attttggtaa gcctgattta tttggaaggt
91801 ggagacagaa ttaggaacat gataaaggta atgaacatgc acccgccgga aagaaaacag
91861 aaccttagag ataggaagat gcctgatgtt tgtgtccgac agcaaccaga agagtcgtgt
91921 ggagcgatgc gtctcccgaa cgggcggcgt gctgccgctg ccgctggcga gccggagcga
91981 cgcctcgacc attacggctc tgatttcatt acgcttctcc agtgacagct taagtgccaa
92041 agtcacctcc gctgacgacg cttccgctca tgaaagcgac taatgatcaa taacaaaaca
92101 acgtctggcc gcgctctcgt aaagcggggc gagctcagcg tgcacgtggc acggctttga
92161 gcaacggtct gctaacgccg ccatcgatgg cttcaaccta acgtctccga gtgcacacca
92221 ggcgcccgtt ggatcaacat cgccgagcag gaccaggtag acccgatctg gacccaagcg
92281 catcaaaggc aactggactt taactatccg gtttcacctc ccagttttga cgcatatggt
92341 tggaacggca gccttgagtc tgctggtctg ctcgagttcc tgtttccatg aataattcca
92401 gtttgggatg aagcacaggt gagcgaaggg gcatggaacc tcccctgtgt cagagcgttt

92461 cacgctacct gtgtagcatc tgcagctagc cccacccagc ctacgtattc
aaaggtgaaa
92521 agcaaaaacc atctaagctg gaggacaagg ctccagagga gaggtaactt
ccgccctaat
92581 gccccatttc tgccgttgtt tgtttgccga gggctgctgc tcgtctttga
ggcatatcca
92641 gaaaaccact gcagcgttta tcaaaatgcc gaacgcgaga tgcagataca
attaaacatc
92701 ctccgacatc aaacaggatc agcaaaacct caaccgtgga tcaaccggcg
tttcctaaga
92761 agccggatca atacgagcgt tgttgggggc gacgggcaga cagtcggatc
tgtgctgatt
92821 tgaggaactg cctgtggctg gaatacggtg catggcccgg aatgaatgtc
ctcttaatca
92881 gccaatcaat tgttaatgct gggggtgaga gtgagggaaa ggatcccttg
ggtggctcgg
92941 gggagaaatt tggattatat cgcatcctcg tttggcaacg aacgccgttg
ctgggagcca
93001 gagtggacta atcaaacagc attactgtac cagatcgata gaggggaaat
aaggattcgg
93061 tctgcgctcc ttcatacctt atctggggtt tgtttgatta taattgtggg
atatttgatc
93121 aagagtttgt gcagatgtgc tttgagccgc tttggtttct tagaatctct
ccgaacgatg
93181 tttcatccgc tacgagcggc gtctcccgac acctcgccgc atcttatcgg
cgcaacgcaa
93241 gaggcaatct acaagacaga tatggaatcg ggcccacaga ggttgaaaga
tggcgccgat
93301 tcacggctga cggcggcttt tttttctcgg cgcagtcggc gctgaagcaa
acagtcgtgg
93361 aagaaaagtg acaacgcgag tgtgattttc ccctaaaaca gagtgaactg
aaaataacaa
93421 cgcacgctga aatggtatct ccattgaaag gaagtcgata taggtgtgtg
tgtgtgtgtg
93481 tgtgtgtgtg tgtgtgtgtg tggggggggg tatagtgcat attacccggc
cctgcagccc
93541 agtgcccacc acggttcgag aggggctgca tacatttctt tcacaaataa
tctaatcttt
93601 ttaatttgag agataaggca gaatggactg taattacgtg cagcnnnnnn
nnnnnnnnnn
93661 nnnnnnnnnn nnnnnnnnnn nnnnnnnnnn nnnnnnnnnn nnnnnnnnnn
nnnnnnnnnn
93721 nnnnnnnnnn nnnnnnnnnn nnnnnnnnnn nnnnnnnnnn nnnnnnnnnn
nnnnnnnnnn
93781 nnnnnnnnnn nnnnnnnnnn nnnnnnnnnn nnnnnnnnnn nnnnnnnnnn
nnnnnnnnnn
93841 nnnnnnnnnn nnnnnnnnnn nnnnnnnnnn taatctttcc tctccccccg
tctgtctccc
93901 tcactcctcc acctctctgt ctgtctccct cactcctcca cctctccgtg
tgtctccctc
93961 actcctccac ctctccgtgt gtctccctca ctcctccacc tctctgtctg
tctccctcgc
94021 tcctccacct ctccatctgt ctccctcgct cctccacctc tccgtgtctc
tccctcgctc

94081 ctccacctct ccatgtgtct ccctcactcc tccacctctc catctgtctc
cctcgctcct
94141 ccacctctct gtctgtctcc ctcactcctc cacctctccg tgtgtctccc
tcactcctcc
94201 acctctccgt ctgtctccct cgctcctcca cctctccatc tgtctccctc
gctcctccac
94261 ctctctgtct gtctccctca ctcctccacc tctccgtgtg tctccctcac
tcctccacct
94321 ctccatctgt ctccctcgct cctccacctc tccgtgtctc tccctcgctc
ctccacctct
94381 ccgtctgtct ccctcgctcc tccacctctc tgtctgtctc cctcactcct
ccacctctcc
94441 gtctgtctcc ctcactcctc cacctctccg tctgtctccc tcactcctcc
acctctccgt
94501 ctctccctcg ctcctccacc tctccgtctc tccctcactc ctccacctct
ccgtctctcc
94561 ctcgctcctc cacctctccg tctgtctccc tcgctcctcc acctctcacg
tggttgcacg
94621 tggagaaaat atagtgttgc ttacaaggtg ggggggggca atggaagtga
gaggatgaga
94681 gggaggtgtc agttacagct ctagtagtca tagtactagt actgctaata
ttaatagtag
94741 tggtagtagt agtagtactg gtagtagtgg aaggagcaat agtattagta
gtggtaatat
94801 tagtagtagt ggtagtacta gtagtggtag tggtggtggt agtagtggta
gaaatattgg
94861 tagtagtggt aggagcaata gtggtagcag ttttaatagt agtactagtg
gtagtagtgg
94921 agcaataagt aggagcaata agactagtag tggtaatatc agtagtagta
gtagcggcaa
94981 tagtagtagt catactacca ctagtagtac tggtagtagt gttggagcaa
tagtactaat
95041 agtggtaata ttagtagtag tggtagtagc agcagtagta gtagtaatac
tactactact
95101 agtagtagtg gtagtagtat aaatattagt agtactagta gcacataata
gtttcctttg
95161 gccttgtccc aggcgagagg tcgagggtgg tgagcaggga atcaggcgtg
tgtcaacgtc
95221 caccccagtg agacctgaag cacaggtgaa cctgacccca ctgatgctcc
cagcacacac
95281 acacacacac acacacacac acacacacac acacacacac acacagacac
acacacgcac
95341 acactcattc taactggggc taaagctgct tcgtgttgac ttgattcaga
aactgaaaag
95401 tgtgcacccc cttgaaaccg atcagaagtg aaacctccta cttctggacg
cacaagaaga
95461 ggtggaaaac aaggaaaagg ccacaattac cacggaatgg cgaaataatc
cagctgcaca
95521 tgagggaggc tggtatggaa cactgattct agaagtgtgc actttgctgt
ctgctgccat
95581 ccactctgcg ctccatcatt ccagagnnnn nnnnnnnnnn nnnnnnnnnn
nnnnnnnnnn
95641 nnnnnnnnnn nnnnnnnnnn nnnnnnnnnn nnnnnnnnnn nnnnnnnnnn
nnnnnnnnnn

95701 nnnnnnnnnn nnnnnnnnnn nnnnnnnnnn nnnnnnnnnn nnnnnnnnnn
nnnnnnnnnn
95761 nnnnnnnnnn nnnnnnnnnn nnnnnnnnnn nnnnnnnnnn nnnnnnnnnn
nnnnnnnnnn
95821 nnnnnnnnnn nnnnnnnnnn nnnnnnnnnn nnnnnnnnnn nnnnnnnnnn
nnnnnnnnnn
95881 nnnnnnnnnn nnnnnnnnnn nnnnnnnnnn nnnnnnnnnn nnnnnnnnnn
nnnnnnnnnn
95941 nnnnnnnnnn nnnnnnnnnn nnnnnnnnnn nnnnnnnnnn nnnnnnnnnn
nnnnnnnnnn
96001 nnnnnnnnnn nnnnnnnnnn ttcccatcgc ggagacgcac atttaccacc
gagacgtgca
96061 agttacccac accaagcgcc cgagtggcgg cgcggaacac gcgccgcctt
tgtccttacc
96121 cgtctttaac tgtgcaagtg tgtcatgact ggagtaatag cagtgatggc
tggagggggg
96181 ggagatgtaa gtgtgcatat gtgggctgtg tgtgtgtgtg cgcgaatgca
cgtgtgtgtg
96241 ctttaatgag aaaagtgctg ctaagcagtg ccaatcacac tgatagacac
tcttcatctt
96301 ccacccagca aaagcaacaa agagcagagt taataagcag acatggctca
gaggacacta
96361 aagtcggcct tctgaccctc acacacacac acacacacac acacacacac
acacacacac
96421 accattacga caagataatt ggctctttta ttcagtagtg tcctgagtga
agcgttccta
96481 ttgaggaaag agaaatacgt caacatttgg aatttggaat cagatttgga
atctgatgca
96541 gattagaaag agttgatgta aacactgatc actgatcagt ggattctgga
gggtaaagag
96601 attgaaaaga tccattcaga gggaggttct tcaattttac atgttcggtg
aattgatttg
96661 gacaaaatat gtcctcgaca aaaccaatac agaccatcat tatcaataac
cttcgatgtc
96721 tcaccgtgcc ctgtcctcct gtgggaccct tcacaccttt gctacctggt
ggacaagaat
96781 gatgtgtcac atgatcactg caccctaaag ccatataatg taaatacacg
catgtatttt
96841 gtcaaacgtg cgttgaaatg ctataattct gaggacagcc tgtaatcagc
cgcgttatga
96901 acgtgcacag ctatcaaatg gtgtctgggt gaagcaaccg caggctggtg
gtgccatcta
96961 gcacatagta gcactaacaa gtttaaaata attatcccga gctttgtttt
tgacacatcc
97021 ccgctgttgt ttgggcatca gttggacggt ttctgcctcc agacagcttt
gaggactctt
97081 ttttcagacg agcacgagct gcggtttatt attgtcctgc tgactcaggt
ggaaacggtg
97141 caagatgagc tcctccgacg ctcaggtcca accggcatct gcgtgttttt
attttggttc
97201 ttcaggaata agaggcaaca gggacgcaac tgatccacag gctattttct
ctccctctct
97261 ctcttttaaa aacacacatc tttctctctc attacccagn nnnnnnnnnn
nnnnnnnnnn

97321 nnnnnnnnnn nnnnnnnnnn nnnnnnnnnn nnnnnnnnnn nnnnnnnnnn nnnnnnnnnn
97381 nnnnnnnnnn nnnnnnnnnn nnnnnnnnnn gttagacatt acatcagggt gatgtaatac
97441 tcccaggact ggctagtgtg tcgggcttgg ggcgggagga gggagaaggg atgggttggc
97501 agagtagtga gtgatgaggg gggttattga gcagaagaac tctctctctc tctctctctc
97561 tctctctctc actctctagc ctgcagccag tgaatgtgca tgtgcagcca ccacagaaag
97621 cctctaatcc caaagcacac agcggtgctt tggagaagga gagaaataat caagactgtt
97681 tccataggcc cccatctaaa ggctggagat aagctttgct gtgcatgctg ccgtgtgtgc
97741 gcagcagctg tgtgtgttgg ggatacatac acagagcaga aatgggtttg ttttcttcct
97801 tttttttggt ggggggaggg gggaaacgac tgaaattcaa tcattaattg caaatagaca
97861 gagaggaaga tgattgtttg gcaggacaga aaatgccaag gcatttggtg tttgtgcata
97921 atgactcaca gccaatttct cacttcagtg cgaggtgctt tttcatggat ccaaaggtgt
97981 taaagcatat ctctgactgg agatgggctt tgtgaacagc gctgcccagg gcaccggcaa
98041 tgtgttttta cctgattgct ttcacgcagt gacagacagg gctctgcaat aaactgcaaa
98101 ttcaagcctc agaaacgaca ttaaccaagg acagcaggag gagagagaga gaaaaaaaag
98161 cctccatttg tcaaataaaa agaaatataa atctgatttg gaaatctgtg gctgtcaatc
98221 tatctaaagg gccaaataac tgagcagagg agccagaata cgcagtaatt caagagccag
98281 tgaagtgtga cacagccaca tgtttcagaa acaccaagac ggccaaatgt taaattggct
98341 tcgcaaactg gaagtcagac gtccaggcgg tccaaggaaa tgtttcgttc ccggctccat
98401 ggatgccttc cattcaagag tttatttgga aaaggaagag gcgctcggct gcccaaattt
98461 acagataatg aattcagagt gctgaggcag ccacaggtaa atatagcaaa accacgctgg
98521 gagactccaa attaaagaag gtgcatttat gattcagggg gaaaaaaagc aaactatggg
98581 attggagcat aaaataaacc tgtcaaattt ggaagggaac atttagtaat gaaacaagac
98641 attgcataaa atgtgcgatc taaaattatg aaaggttaaa agggaacggc ggaaccctga
98701 caaagcctat ttcctgaaat ggtgtcataa aaagcccctt ctttaaactg gtttaaggtc
98761 gtttatagcc tctagcatag atcatccttg taaagttaga aagcaaccga aaacaccagg
98821 gttgtctttt tatgactcca ctaagttaag aggaggtaat tcttattaaa caatgtgaaa
98881 tgaagatgct aacttcctgc taatatggaa acccaaccgt gctgtcagcc aggcagctaa

98941 cggaaccctc tgaggtaata tgaaaggaaa gtcaggcagc ctttgaagga aacggggaaa
99001 cagggaacaa aaactctacc tgacaacttg tcagtatgaa agactgcaac ttcttcttta
99061 gctgcgggct cggaatggag acgaggtaac gactacgatg atcgcgcaca acaggcactc
99121 ccaggctgac gccgacacac gatggctggt tagttcaaaa ggccgccccc agaacctgtc
99181 tcattgcaaa cacaattaaa atgaagtttc ttcagtttta agggatgatt caggctgagc
99241 gcgttagctt tagcgtcagt gccctcacgg ctgcaggtct gcattaacca ttgactgcta
99301 acagattcca gtaggcacat gggagtcaca gatctctgtc accagtgtgt tattgccgcc
99361 tttgagaggt caaggatgac tagaaaggca ccggccaaaa cccactttaa tcctaccaac
99421 ctacaggtga acatctggcc caggtcgacc aggaatccca ttaagtctgt ttagctactt
99481 aaatgacagc atgtgaatta aaatagcttt taagctgcca gaaggaggaa tgcgtctccc
99541 cagaaggact ctgtgtgttt taatacacag cggcaacacc tcatttatta tttaaatctc
99601 ctggatcata gttgttaatt cgtgtccagg ctcctccgca cattttaaat ggcatgcagg
99661 cagatattca ttttgctgct taaatatctc tttgcagaac tgacttcact gaagatttgt
99721 gtggcggggt gggccagaag ctcagattta gagacccgca cagccggtaa ttcatgttgc
99781 acatgatgtg tgggcacaac attattactt ttgtgttttc ctggacttta ctgaacacgg
99841 tcgatggttg tccagccatt tcctgtttac cggcattttg actggtgttg aaagtaccgt
99901 atattccagt agtggctggg tgagaagcag gcatacaccc cggacatgtc accagtccat
99961 cccaggacac acacaaacca cacattcacc tctctcttcc caaaaagcac atttcttttt
100021 acagttggag gaaactgggg gtgtggctgt acccaccact ggaccaccat gactggctgt
100081 acctggtgaa ttttggactc aaggtccaaa gatgacttgc caccattgtg ttcttcatgg
100141 tttgtgtttt gtagttgttt ctgtgctcct ttctttccac atggcataaa cagcagcctt
100201 atttgtatgc tatatcctct tacactgagt cccgttttgg ccgagctgtc agcaggcgtc
100261 tctgagagcc ctgcaccagc ctgtccacac tctctgatag ttcatcccac cacaacgtgt
100321 tgtaacgcgt ctccccctgc tctcaggtct ccgtgtttat tctccgcctg catcgtctgc
100381 actcttcatg cggctgtctg gacctcccgg cactcagctc gccgtgacag aggtcaccga
100441 gtgctcggga gccagctgac gtcatgttgc tgcaaaaccc cacagacgcg ccagcgggcc
100501 ggaacacaga cgctccgaca gacagagcga ggagcggacc gggtgtattt atagcctctc

100561 ctctcctgta gccccacagt aagcttcaag gccagatacg ccctgaagga aacccatcct
100621 caacgtgttt gtttactatg cggcgtctcc aagcgtccct ttttggaaat atgcacacgc
100681 atcatttcct gttggaagca aataattctg tttcccttct gaaaggtgct gcgatgctca
100741 tctgcttaat gagcacattc tggtgcaaat cagctactga gatgacgcac tcgccacaat
100801 gctttacacc attacttatc taataaactg taggaggcag ccgccattaa cactggcgcg
100861 gacgctggaa tccacctccc accggcaggt ttcttgaagg ctaatctgcc gtgtagtgta
100921 tgcaaatagg aaactgttat tgtcgggatg caacaaggtt ccacatcatt ctccggtagc
100981 gggccaatcg ggttgacttt gaaatggatc gcgggtgatt agccggctat gctgggagaa
101041 tgccagggcc tctgcggtga accccgcagc tggtgccgga gtcagcggag caacgatcca
101101 ggtaattaat tctttgacaa gcatcagtga tttggaaaat cagtaagaat ggcagacgct
101161 gatcaaaagg gaaacacgtg gagagctgtt taaacagtgg gtgtttgagg cgtcagtcac
101221 gtggggccaa gacatctacc aatgtccagt gtcctgagga cgcccccgct tgccaacacc
101281 cgtccaaaaa gacaggctca tatccaaact gtacaactga ctccacaaaa gcaacctcgc
101341 gtatatggga atacactcaa aacacgtgct aatgagttgg agcccgtcgc agcgtgcaca
101401 gactgatgtt tatgacaagc actaaactat tacagtcggc caacatgtac agtaaacaat
101461 atggatttaa catcaacata cacataaacc acacatgttt attccggtca caaacccccg
101521 tacatgggct ctgaatgcac accccaggac cataactcaa tatccccacg tggacatgtg
101581 gtaaagatca attcattttc agaatttatt gtacgtatgg cgctgagcct gtatctggtg
101641 aatgaaatgc aaacgccggc accatattca tgggcgtgag agcacataga tgcgggcgca
101701 gctgcggcac tcttcctaaa cacacaggag gttgggttga cgttaccacc gttcctttga
101761 ttaaatcatg cgtatatcct tttatttgat tgttggggca gaggggtagc gcacaccagc
101821 cgaaaaaccg taatgaaaca tcattggcag cggctattat agatggtgtc cagaatacaa
101881 attccccatg caggtatttg ttttcatcca tcagaattca ttatgcgagc gaatgcatca
101941 ttggagtgta gctattgatc cggtattaaa tctcattgtt gcggcgctat tcagcgcgcg
102001 agccaggtga agaggaggga aaaagagaaa gcaaggctga ccaaatgtca cggtgacaac
102061 atgcggcatt gtcaaaggta gctttatcga tcaagaaaga cagaggggga gaggggagat
102121 atttcagcag gaccccaact gcctgacatt ttagaaaagc cgtcctttct gtttttgtca

102181 ccctctattg ttgcacgcaa agagcatgga tgtcttgact gaagccgtga gagctacagg
102241 ccagcgggcg gccagcagag agcggagagc ttccacgtaa acctggggct gctttttgg
102301 ggaagatgga ccatgagcgt taaaatggcg tcccgctctg acattttaaa ggcctggctg
102361 tcacacttta tcaccagata atctgaaaca gctccggctt ggtgagggtc caattagctt
102421 ccatggtcgt gcgctggaat ctttcagaca acttagatga ctcagacatg tccccagtgg
102481 cggatctttt ctatttctgc tccgattcaa ggaagatctg cagaccgagg agacacaatg
102541 aggcacagat gaaggcataa ctgggggacg gtggggattg attgtcatct aggctggatc
102601 accagcatcc aagacaagac gagcagggtg tgaggtgttg actgggccca aaaatcccat
102661 tttaaagcaa gccagaaggt gtggaagttt gccggctgag ctcaacattt cactccctct
102721 ctgactgact tgctcaggta attagctcag acttgcgttt ttccctgtgt atgccttaaa
102781 gcgtcttgtg ttaaagcagt ccagatcaaa aaggcatgtc atagcaaatt atcctcaaag
102841 gtgctccacg gcaacagctt ttagtcagct ctgctcttcc tccacttccc aggatgttta
102901 tctactgcca ggacagggaa atctccacgc tcattgtttt gcaccgaagg gaaccgaaga
102961 gcggattaag cagagccccc gatctatgga atgatcttaa cattgtgacg ctctcactgg
103021 aattctccac acaggctcgt cttttcaagc agagttctaa ttggaagagt accaggattt
103081 tatcatgaaa tgaaataaaa tggagtaaag ggcaaatatg ttgcaacttt cacatcagat
103141 ttcagaaaat gattttatat aaggtctacc tttttatata aaaatgcaat gcacgttcag
103201 accagcaggg ggccggtgtg tgcagtcgct atcagcactg ggcagatggc agcatgtttc
103261 tatttcagca ttttaatttc acataaattc agcgcatgtc agaccctctt aggtgtcatg
103321 atacttttca tcattgaatt cccaggcaag ccactgcgcc acgacgttgc actgctgcga
103381 tgtcacggtt ggtctggggc cgacgccgct acagtcatct gtcctacata tttttaaaca
103441 taagccatac taagttgtgc catttaaagc cgtaacatac tgaagtgctt agtggaaaca
103501 tacttaagtg ccctcagaca tgacaggatc ccctctcagg gtctgtgctt gcaggagtgg
103561 tgcaggaggg gggatggggg gtacacacaa gtaacaggat tgaaccctat aggtaacaag
103621 accaaatctt ataggttaaa agattgatcc agaggaacct ctttttcag ggcattactg
103681 ggcatggtat tacacttcct gtctgatatg agcgagatgg ggctggactc actgtgtgtc
103741 tgcaggctgg tgcacctcac tacagcttaa tggaacatac gggtcgggct tttttctcat

103801 ttacagtcat gggttcgcct ggatgtgcag cgtggcttta gtagttacct
gaaatgggca
103861 ttttctgtca gccacacaat gtttttagaa ttttttacag gcgaactgct
gtacatagga
103921 agaagcagta cccaggtcat tcatccacac catcgcaccc tttgatacat
gttacagctc
103981 cgtaatcgtt cttcgctgct gttacacaag aatatcttca aagtggaact
caaaaggtct
104041 atcttatctt atcttatctt atcttatctt atcttatctt atcttatctt
atcttatctt
104101 atcttatctt atcttatcta attgtggttg gaagtaaata ctacgaacac
aatccagtag
104161 agggctccac ctgggagcaa tgttcaatgt tacgcacact cagttatgca
agagttacat
104221 ccagagtaac attttgcaaa gttcactgga tttctcctgg aaaccccgtc
tgaatctgtt
104281 ctaattgttc gaacagcact tttaatgtta ttggctttta aagcctaatt
aaactttctt
104341 gttgcaacgt cattgatttg attggagttt ctgcactttg tttccttttt
gtactttcaa
104401 gggaaaactg cacacttgtc tttggaccgg tagcaacaca gctgagatgg
actttgacca
104461 gcagaaccgt caacatcatg aatataagat ctggaaaaac aggagaaaaa
gggaggcaat
104521 cagggaaagt ttgcgggtta atatcaaggg gcagcttcag taaatggtgc
ggcagaaact
104581 ttgactgaaa accttgcaca gcaataaagg gatatacaca tgaataatgc
agaagcattt
104641 aggtcagtgc tgaggcgatg gggggaatca gatgtccctg gtgccctgaa
ggattccagt
104701 ctactgagcc agcgtctccg gtcaagaata gagcagcaca gatccaaagg
agaatcccac
104761 tgctccacaa agtccagcca agagagacaa aacacacgca ctgtgtgcac
acgagcgtgt
104821 tttaacaact gtcgatgcct cacacataat cagcacacat gttgaaacac
acaaacccca
104881 gagagacacc cggccatctc tgtaaaggaa ggaggttttc acagaatcgc
caataagcca
104941 gtgtgaagat gtagtgtctg caggaaccga tgggatcgca tttaaaagaa
ttccagcgaa
105001 gaagcagaaa tgcgtgaacg atcacacatg aacggccgcg tgtgtcgaga
gaagcccgga
105061 atgattgcag aaacatcagc ttagcgctgg ctttactttt aggtagtaat
tgtgggattt
105121 ggggtttaaa tacaaatcta tgtcttaagg actgcggagg cgagtaggca
ggagtcggag
105181 tgttgatttc acagaactgc tgagtagatt ccgctctgtc gtctcagctg
cccacagaat
105241 caactaatga ctaattaatt acaacacata tccaaatcaa ataatctgct
tccctttggc
105301 gtgcgtcgtt ctccttggct gcccttcaaa acctctcctt atggatccag
aagaagcatg
105361 tttaggagtt aaaagatgcc atcattccaa agaatcaaca cttatgcttt
ctttttagtc

105421 agctggtgca cgtactgaat taacagtgtg tgtgtgtgtg tgtgtgtgtg tgtggttaat
105481 gtgttggttg acactgcttc tatttaccgg ctttaactca acattaaaac catttgattt
105541 ccattcagtc aatgcaaatg agagcgtcgc ccctgcaaca tatgcggctc ccagccgggg
105601 gccagtgagc aggggccagg ggcggggacg ctcatcacac cggaatggta attcaccaat
105661 cagcgaggtg acacgcttgg cgaaaagtag ggagagccgc gcgagcctga caggaacatc
105721 agtttcacga aacatttcct cctctgcacc tgaaagcgcg gcggcataat cacgagcggg
105781 ggcgatgcac catgaaatgg aatgggcgcc cagatccggg ggagcggaca cagccgcgtg
105841 tccacagaaa tgagggtcat cctttaccga gagatggcag cgtcagtcga acgggtcccg
105901 caggttcaaa tcagacagct gacgccttcc acacgtcttc acgtcaaaat gtaattcctg
105961 attcggctgg aaccatcaac gacttatttg catttctatt tgtcatgaaa tggttaaaac
106021 gagatttcaa gttaaaaatg ttccggctct gagctaaagg gtccctttgt gctggacttt
106081 ggagtcagtc ctgcctctag gttttctcca ggtgctccag cttcctccca cgtgctcttt
106141 aggctgattg gagactaaat ggtgtgtctt caatgacctg tctggggtgt attcccacgc
106201 ttcccagatg accactggaa ttgaccggaa gtttccgacc ctgattatgt taacagaaga
106261 gagaaaaaca aatgaatcat ggactccatc tctccattgt taacacagag gatgcttctg
106321 tactttatca atattaaaat acgtacaaca tgtcccgccc attttctttg tttggatcgc
106381 tcgcgttacc tcgacacacc tccctacaca cacccacaca ccccgaggtc ttatatattg
106441 gaagacacgt ttcagttggt ggaggaacaa cgtgagataa caaacaacag cgactaatag
106501 actttgctcc cacagcaagt tcttcccaaa taaaaaaaca tggcctgaat ccacaaaccg
106561 ccacctctct ctatctgcca gatgtatcaa tgttcctccg agcaatcaag aagtaattcc
106621 ctttatcggc cgacatgaat attaaagttt tgtgctcggg ggatgaaggg aataaatgct
106681 atcgtcaccg gcacctgtca gggaaggcga tggatagctg aagaggaggg tgagtgctcg
106741 cagtccaata gcgggcccgt aaagactcca gtggcgacat cggatggcga gagtgctgat
106801 tagtgggagc aattagcatc tttgtctttg ggtacttgtc atccaacaca tgtaaattta
106861 aacaagcgca gccgcagcag accatcacat tagcgttggt tccactgaat gcaaatgaag
106921 agtacataaa caaatatctg aataaccaga ggaggagttc cccagactgg agacctttca
106981 aacagtgcaa atagcagcac gaccggcagg aaaattaaaa caaagtggaa gaattacgac

107041 gaaagaaaca gtccccgctg atcttcaacc atcccccgag ggttagagcg
gaacactgat
107101 ggtcttacac tccgtcaact tttggacacg gacgcacggt cacaaagacg
gcgagccaat
107161 aaacagcaga ggctgctgga acacacacca ggtctggtaa tgcactcgga
ggcaggagga
107221 ccggcggcgg ttgggaaagc tgtcgacgcc tgcggatcat tgctaatatt
ttgcaatgtt
107281 cccccaaaat tcctccaaag acgccatgaa atgagctgaa taagctaatg
cacaaaacag
107341 gcttctccat ctctgcactc cacctgctgg cgcgcgccag gcgcatcatc
gcctgtgttt
107401 acagtagcat tatagatcgt gcgtgtagcc acggttaatg gaaagagtgg
gagaaaggga
107461 cgggcagggg gagaccgtcg tccttctgtg gagcactgct agagtgttga
ctttgtcgcg
107521 gtgccatgtt ggattgcgtc cactccatca tagccatgcc cagatgttac
aagagagata
107581 accggattgc atggagcagg cttgtcaggg atcccataca ttcactcaga
atctctggcc
107641 catagaacac agataggctt ttaaagtgaa gtgatttaga ttggttttct
gtgttttttt
107701 cctctttgga ttatttcact ttgacatcaa cttggctcca gcttgtacgt
ttcttggaaa
107761 caaagacaaa ctgctggcag cctcaactgt ggcttcaatg gctttaaggg
tccacataat
107821 gtaacattag ctacctattt gaatgtaaac tggaactgga acgattcctg
ggatgtcctc
107881 agtgcaagtt gcacgaaaca gcacatttaa taggaaagca gaaaattacc
atcggatatt
107941 tatattgatt ctggtgcacc ttcttttatt atagtaacga tgcacttgag
ttcctaatga
108001 gtgttttaaa ttgccaaaat gaaacaaaat tgttaattta cacctctttt
tattggatgg
108061 ctgagtcact cccgaggttt attgctaact gattactctg cggaatgccg
ccctcgcaat
108121 aacgccactg aagcattaag tcgagataaa agcaatctaa atatgaacat
gcatgatggc
108181 cacgctaaat gcacccagct aatggtttca ttattccatg ataaagggtt
aatgtatatt
108241 cagaattcca atttacttaa atgactcccg catatgcaga gctcgtcaga
tgggtttgtg
108301 tttttcgttt gagttcccag agacaccctg aagtcgtccc cctgagccca
ccattcagca
108361 gagggttcaa atacagatta gtcagcttcc tgtcccctgc cccaaaaccc
acaatcacgc
108421 tcacacccgg tgctacgccg catgaccccg tgtgtttctg aacctcagcc
agcgctcttc
108481 tttaagactc caaatcctgg ctgcttccat cccacttcaa agcggtttgg
catcggccgg
108541 cgctcttaac ctcccaatct ggccgctctt gttacagctg cgccgcgggg
acagagcggg
108601 cagtgtttga gtttgttgtc gttgagttta cgtgcgcaca gagtcggcag
tgtacggggt

108661 ctgccgatta acccccaggc cagggctgct ctccactagc agacagatcg gctttcagcg
108721 ctccctgccc ctgtaacgag agcccccgct ctgaccggcc acttcaaagt gagagcgggg
108781 gccaggaatg tcaggttcac caccaggaga tgatgagagg ctcgatggag gagaaggctc
108841 attcgggtcc actgtatgtg cacactttcc ttgtttgagg agaaatactg tagttttaat
108901 gcggttaaag agccttttgg gattgattgg cctcagcagc ttcttagagt cgaaaactac
108961 gctgaataaa agcgattgta aatatgtacg acggcgcgat taaagtcagc acttaaggtc
109021 aaattgtatc aatatagtta aaatgactga aaattgagta gcatgttaca tgttgaattg
109081 tctgaatctg actcgaatct tttagcggta gctctctgac aaccagcgac agtaactgtg
109141 gagtcatgga agaaaaacag cgcaatcaaa acccttgtga ttttctccaa tcatgcaaat
109201 tcgtagatca gcaaataata tccaatcaaa tcataatggt cctgcttggc aagaggtcag
109261 atcccaatca aagtcacctc ttgcgtcgcg aggatgccga caccaacggc gctgttagca
109321 acctaatgac cgccacgatc acatggttga cccgtgttgg cgttgagagg gagcgcctct
109381 tgtgatccaa ccctcagttc aagctctgct cagaaatatg tgtcatctaa ttcaattttc
109441 tatgtagtgc caactgtgat cgtgcatcat gctggagtta ccccggcaga ctgagagggg
109501 cagcaacaca caccagtcag gccgccacca cacacaccca cacacacaca cacacacaca
109561 cacacacaca cacacacaca cacacacaca cacacaccat tgactcaaac actaacccct
109621 cagggcaatc tccagtcatc ctaatgtaca tgtttttggc tgtgggagga aattggagaa
109681 accccaccga ggtatgagat gaacacacaa acaccagata gaaagactct gaactggcat
109741 ttaaacccac agttttcttg caaaagcagt gatgttgcat cttccatttc cttaaaggcc
109801 cgaacagctg ctcattttca tatgggcttg tttgctctgc ggtgaagacg atccctggag
109861 gtcagggaga agaacctcct gcagaggtca cgtttgccgg cctcagacgt tctgtcgatc
109921 acccaacact gcgctgcctt ttgtggcggg cagacaaaag gcctctgccg gctgagggag
109981 acacgcaacc tgaacctgac acaggaagct ctgaaagcta cacaaaacaa actgctgagc
110041 tttggcattt tgaccccccc acacacccag aaagactatc tccgctgtca ttaaaggtga
110101 actcaatcat ctaatctcct ccaaagtcac tgaagccacc ggcacctcca cctcctcccc
110161 tccaccgctt cctccttcct tcctccttct caccatcccc ccccgccccc agtcgtggag
110221 gccttcaatg atatgccacc agagatctcc aggggggggtt agaggtcata ggacaaaaat

110281 gctctgttga atgctccccc tgaaaggaaa ggcggcgtgt tttgtgttgc
cctgatcgat
110341 ggacggaagg tgggagcgct aggcgacgcg cgcgattgtt tccaaacaaa
acgcttcatc
110401 cgaacttcgt gcgtgttgtc gggcagatgt gtcggcggag gccgtttaac
gctcccgccc
110461 ccacacgtaa aagtagcgtt atggatttca tgcgtgttat tgtctaaccg
cgcaaaaata
110521 acagttggcg atcggatttt tcttttttttt tcgtttcacg tttgaattcc
ccagcaggta
110581 atcaataccg agcagtgagc agtaaataag ctcctgatgg gaaaaaagaa
ccaacagaaa
110641 gtcgtcagcg aggacacgcc gaaagcccgc gacggcggga gcgcatgctt
ccacgcacgg
110701 acgcatcctc tgccaccaca atacacgtca tttttcgtgt cagccacatt
cggttgaggc
110761 catttgcacc tcacgcatca tattctgccg cctgtgcaaa taacgctgtt
tagaattcaa
110821 ttatctgagc gtctcctcct gcattcaggt gggatgaaaa ggcttctgtc
tcggcagcaa
110881 aggtggaaaa tggtattcac ccccctatta tcgcatcaaa atctgcctgc
ccattgcaaa
110941 tacaggctca ttaccctgct gaaaaagcca ccttaaccta ttttacaagc
cagccctgta
111001 attaatgact ttttaagcta ttgaaccaaa agcaattagg ttataaagag
ggccttgttc
111061 agttaggtga ataccttttcc tattagtcgc atccttgaag gggtgatgct
aaccgaatgt
111121 gacagtgcct ggaattaata tgcaaatatt gtcaatctga gagatatatt
ttaatttgtt
111181 gcagattttc tgaaaagtaa ttcagcctta caaagacatt tgaaggtcaa
aacgctcccg
111241 tacggtcaaa tagttctgct gtttctccac gtaggcgctc aggtgcaaac
ctggcgctgc
111301 cgcctcccgc gagcgcagca tgcagcgcgc agataagact tcatacaaca
cgtttcctat
111361 atatttgttt tcgactttat cgcatgttgc agttgaggta gacacagccg
agacatgtgg
111421 tctacattct aatgcctgca agggtcagcc cgtggtcaac tagcacatta
ctctatgtct
111481 acccagacag gtcggaagcg cacaagacta atgctgcggc tcatcgcaaa
ggtcatccgt
111541 ggatattaat gcgtaacttt aactgaacaa cccgcgccat tttgcgagga
tgcggttttc
111601 cgcgagggat aaacacgccc gcaggatcct gacacgggtc ataaagtgcc
aaaggcgagt
111661 tttgtcgaag cagatggagg caaaatagcg cattgatgtt tgtgctctcg
ccccagacag
111721 caatcgaact gcgcgtaatt aggagggctt ttggagagaa aaagcaaaat
cagattgatc
111781 tctttatgat tcacaaacgt ggaataaagc ctgacaataa acctcgccgg
ctaacgttta
111841 ctgaattgat ttcaatctca aagccattaa aacggtgata caatttctgt
caaaagatgt

111901 aaaactgcat cctatttaat tgaagccggg tcgatgccct ttgttgtccc gggtttctgt
111961 ccgcgatctg cattcagcaa acaaaccagt atggtcatta ccgcatgatg atggtgtgaa
112021 ctgggttact tatgtcatct taaccttgtg gtatatttta attgtaacaa acaacaaagg
112081 ttcacctggt caacctcaga ctggaggcat tggtaaatta ttcccccatt acagcagcag
112141 ctatcacatc ttactgaagt ttgtgtgtgt gtgtgtgtgt gtgtgtgtgt gagagagggg
112201 cgggctccta accttcggct cctaactgcc caccccgtgc tgaacaggca gcagcaatgg
112261 cgttattata agggtcctgc ccccctgatg tttccaaacc cagaacattc ataaatattc
112321 atcaggtctt tttctaaatt atttatttat ttcagatatt tgtaaactgt attgatcagt
112381 gtgattactg taattacccg ccatgtcttg cggttactcc agatcctttg aattagcggc
112441 accagagatg atccttcacc tttaggacta ttaggcgccg tggttccgaa tcattgtata
112501 accagtaaac aactaggttt tttttctcct ggaactcgtt acttggcccg aaatgtgaaa
112561 agaccccttc attcattaaa tccttcccca tcaggcacca gccgtctctt ggaggtccag
112621 ctaacgatgg taatccggca tctgtaaact ctgtgaggtt ttccttattg gactcgggcc
112681 cgctgtctaa ataattgtct cattagggtg tgcagcgtgg ctacggcaga aatgcaaact
112741 ttggaccagt gtgtatgcaa atcggatcgt ttgttcgacg ctctttattc atgcgctcaa
112801 aaatcagccc gtttatggca gcgacctcag ccctgaaaat ggaaattttg ggataagtaa
112861 tgcatttcca acaaggtgaa cagtcggcag gttgtggatg aaaacagatg atgtgcaaac
112921 agttgacagc tttcagcctt gctggcatat atattagata tgtgtactca ttttggaaaa
112981 caaaggctgg aaaaaaaccc tcccaagtgt cagtcatgtg acacgccatc agggcggtgg
113041 cgtgcagagg gtgattgtgt tgttgttgtt ggagtgacaa aattacccca gattctattc
113101 gacacatttt gaaaaaaaag aggacaattg tgtttcaact caacagacgg gattctgtaa
113161 aaaacaacag tttggctttg gcgtgtagct acaggtttgg gatgtctgga tgcagccgaa
113221 acgaacatct caaagtcgct cgattatact tgtaaaacca gagatttgta caacacatta
113281 cccagattaa gcccagaggt gaaaacaaca tcacgggtaa ccttgtcagc agcgattaat
113341 gctgttcaaa cctgctattt gcagataaag attatgctaa cgttattagc attagactgt
113401 ttggcgggta gatttcaggg gcggcgtgcg tttttattta catttgcagt aactccatga
113461 caagtacaaa agtcaaaaag tacggagagc tggtggaaca cgttctgaaa atgggctgtt

113521 tacatattag ctcttcattc atttacatgg aatctgtaat tggaataata gttccctttc
113581 ctcgaatgca aagcttgaac cagaatacat tcacgacttg acgagtgcag acgattcgca
113641 tcaaagtagc ccccgccacg atgctgctgt actactgccc gtcttcacaa atttcatgaa
113701 atattagtgg gagcaaaaaa aaatcaaagg ggcaccagca gaacatttta gttgcaagga
113761 ttcaaatgtg agatgagatg atgaaacagc tgggagctgc agaaacagga aaaatacctt
113821 aaacgaggac tttcagtaac ttttttgtca atttaggacc ctcaaaccct caatggagga
113881 gcgagagctg ttgaatagag aaggaaatgg caaagacctg atgcatcagg aggaggagac
113941 cgtggcggac gcgtgttatt cgtagatttt cagcacgatg ctcaaaaaca cgcacctgtt
114001 tatcccagtg cgaggggagc gggaacacga tccgtctcag atttcaggct gggttgatac
114061 atcacagctg cgcaggatac tgttcatcaa ctccagcatc gggggtgtcg tcctgccctc
114121 ccctcggcgg cgctgttagt attcacccag cgatctgcag ccaagtagca aagagcatgt
114181 taagcacttc aaaaatagtc attatggaga tttcgattga accgagcctc gttcggcctt
114241 aaattatgga aagcatgaaa gcgcatccgc gtgggcagac cagcccgggc tcttttcaga
114301 tcttctggag aaaattaaaa tcgcactgat caatatttta tagtaatatt aggccagatg
114361 aggaggaaaa gtcattgttt ttgtccttca agggcagact tgggtgtcct ggaagcagcc
114421 tgggtggaac tgctgggaat ttaggacttt ctattttcag ctaaatgtga ttaaatctgt
114481 ccccgacacc ctcctctttc aaaaacctct gaggtctgat ctgaaggcag aaacgaaggc
114541 tggaattgaa tttttatgca ttaaaagtgg gagattatca cagaatgctt catttaaggc
114601 tcttcactgc agacctccac acacagtaat cacattagac ctggtttgct gtgttttggc
114661 gggttttaac cagcgacctc tcgggggagg ggttcccccc agaagtttag cgtagccata
114721 gcccatatgt aattaaaaag tatgttatct ctgcccagaa tgttatggct gagcgttggc
114781 cgggtccagt cgatatgatc ccacccaccc ctctttctct ctgggtgggt tcaccaggtc
114841 accaactgcc tccctgtgtg tgctgttgag cagcggcgag acggaagggg ggggctctct
114901 ggagagtttg agttaggggc tctgaacata gtatttccca gcctgctttg tgtgttgaaa
114961 cagaaaataa accaccccac cacccccaca cacattgttt ttccaattgg cagaagggaa
115021 gaagcggcta aagggtttta ctcacgtgtg ccttaaacaa aactctaatg aagttcagag
115081 aacgatctca atggccctga actcttatct acataataaa tgatttaata tggctctgtt

115141 ggagatccag tggacgccac tcactcctct attgatacgt tctgatcata tcaggtgacg
115201 gtcgatctcg ggctcattag cgtttggcca aaccgcgccc gtggctacgg ggagctaaaa
115261 gaaagtctac agctcgatca gtaaagtctg agctgacctg ataatcaacg ttaaggagcg
115321 gccaaatgga gggcaaacaa aacttcacaa agttcccggt ccatcgggga cagagaccga
115381 tcacggactt tcctcctctc agacattcgt tttaagatca gctaaacttc agcagccatc
115441 aactacggtg cgccgctctt ccctcctctt tcttgagtcg catggctctc agacccaact
115501 gaggaagcag tgggtggcag agacccgaga gtttggaaaa agagaagaag tctaagggtt
115561 tgaggcataa ttactgacca cgggccgcca atggctctcc agcactgagc atagaaaagg
115621 tcgggaaagg tcacgcgcaa gcaactcgta ctacaagaaa atatatattt atatctatga
115681 gagtttgaga tgaacaaatc tagcatctgt aatgaaatcc cattgggcag ccgcgttgct
115741 attatgcaca tatattagca cttttttttcc agaaaggaga acagaccttc cctcatttta
115801 atcctctttg aagcgagctt gcgctgtgta cagtcatgta aatgataccc cctctcatgg
115861 tttctgcgcg gaaagcagac gtcgtaattg agcgaggacg cggttcctcg ctcgagccga
115921 attagcagaa gatgaccgag ctgtgaaccg caggagtttt taattccccg cgcttgcctc
115981 agcggcacgg cccttctgtt ttgtttgatt caccacgcta acacacgctc ctctcaggtt
116041 acacgccgca aacagaccga cgcgcagcgt ctgactccgt caataaactt gcactgggtg
116101 tcaagatatg aggaaaataa caacagctgg agaatagatg cgtattactc acaggaagag
116161 agccatgccc ccccccccaa gtcgtgcttt gaccacgatg ttgggaatac gggcgatgta
116221 aaaaggttgg gctaacacat ttaagtcaat gtttccttgt gtatctgtct catttgccca
116281 cttcggttgc aggtggaact atttttcatt ggatctgggc tcatctctcg ctagcacaac
116341 acacagcaga tcacgatcac ccaggcaaat gtggacggag ctcaaggccg attctttgtt
116401 tcccagcaaa ggtcccggaa gtccgccctg ctgttattgg cccagatcga tctgggattc
116461 aaaatcaagc gcgacaagca aaccgctgtc actgttggaa tttatccagg taatcacggg
116521 gacggaaaaa ggaaattaca agttttagaa aacattagac atccaagctg ctgcaacaaa
116581 gaggtgctat tatgcaaatg tttcgctttc aaaggatgaa gagaaattga tttgtctgaa
116641 aaatcagaga gaattacagc tgttgtcaca gaaagtagta aattactcca aactataaac
116701 ctcctattca gttccgtctt attctttgca ttgtattagc attcgcccag tagctaggct

116761 aagtcctacc agataagtga ctaaacttgt gttgcttatc agggaccaaa
tgaacaaaaa
116821 gaaacaaacc aacggtcgaa aaaggcagaa tgcaacaggg gagggggac
cacagactcg
116881 cacttgtcac tccaaacttg tcaagttctg cctcaggctt cactcagctg
gtgcaggagg
116941 taccatccag agcgggggtg tcaaactcaa ttacagacgg ggccgaaact
caaagctcgt
117001 ttacgctgca gagtgaacat ttttagagcg ctacagctaa ctatgtcttg
aacagaaata
117061 tgagtcagaa caggacacct gacctctgtg ggccccggtc ttgaccttta
gtgtgcgcag
117121 gagtgctttc agctccatca tggtgtttgc aggtgcttct ccacctgcct
gtatccgagt
117181 gaaccacaag catctccgct catacgcatc tgtgaccacn nnnnnnnnnn
nnnnnnnnnn
117241 nnnnnnnnnn nnnnnnnnnn nnnnnnnnnc cccccccccc cccccccccc
ccccccccgt
117301 tccaggaact gatgttttag cgtgctcgct gtatcaccca ggaacatcag
cggagacctc
117361 cacgcacggg ggagtcttca ccgtttgttc cgtctgaatt tgccaaaatt
cccttattaa
117421 gtatcaaaag gcagaaggag gggcaggtga catctgcatc gtccacatgg
cctgaatggg
117481 atggaggacg aggtcacacg ctaacagcca ctaacgtccc tccaaagcgg
ccctgttcac
117541 tgaccccccc ggcccgagtg ctgagcactt ccttggctgt gactcgagat
cctcggatgg
117601 gacattccga cacttcttaa aaccctaatg ggcttggccg ctgcgggaga
gggggagggg
117661 gtgaggggag gttaaactta attggaatgg acttttccac ctctaaattt
taatgaacct
117721 ctgggctgaa tgtctcttta ttaggtgaaa tcggccatta gcattttaaa
tgaggggcta
117781 tcttcattat aatcagagcg agatgcatcc cagcgcccag gatccattga
ttgctccgca
117841 atgtcaaagt ctacatgaag atgtggaggc agactgctcc tgacaggctg
tgaggggatc
117901 atcggtgcag ctgcaggggg ggggggggat catccatttt gcaccctttt
aaataattac
117961 agtaatatga agataattac agtggtggca gcgcggctac attacgaaac
aaacatgttc
118021 taattcaatt tatgcttcct gtctgtttat gtagcaaagg ggacacaaca
ccaaactgag
118081 cttttttttg ctcaattcct ggactccgat gtcactttta catcttaaac
aatcttaaac
118141 atttatcaag caaacaatat aaaaaaatga aggcagcaga gtcgtctgct
gaaactcaga
118201 tggtttttgg tgaaccatat gaaccagtac tgtcatattt gcttctgatg
tcttcattta
118261 atcatcttta ttaagatggt ttgagttgtg cccgacttcc tctgctgctc
tgacagtttt
118321 tcaatcttcg actcaaagcg cctgcagcaa atatttgaag gcctttttta
tcttccttcc

118381 gccccgactg aagcaatccc gagaccaaac atttgccgtg tatgtcgcgc agaccctcat
118441 gtgaggcagc cacagtcgct cattatcgag ctccaatcac aataatctct tttttatttg
118501 ggggagggtg ggggggatca cactgtcccg tttggagagg tttgagaggg attccagatg
118561 gcaccgatgc agcaggctgg cactgaggag acgcccctcc ctcacacatc atcctccacc
118621 ttcacacctc caccccatcc ctgcctttac cacattttga ttctcatcct caccattcct
118681 gtcctgtggt gctcttgtgg tttccttctc tcccctcacc cttcttcgtt atgccctttg
118741 ccccccctccc tactgattgg tgcttatcta aacagaacct gtcgcatgtt gtcccaccgt
118801 aggacacagc ccccctcctc tccgcccctc tcctcgctcg tcctacgggg ggcgacagag
118861 ccctgccctc cgattacacc ggggcgcggg ggttacaatg cagccctgag ggccggcagc
118921 tgtcaattag tgataactgc caagaaagag acacacacgc gtgtgcacaa acacacacac
118981 acatctctgc ctacattaat caggggggcca tcgcctagca accgtcctga gtaagtcctc
119041 aatggggatg cctggcgaga ggcacacaga gaaatgacaa tgagccggag tgagggatgg
119101 cgagagcggt aggaggtaga gagaatgatg agagatggag ggggtagatg aaaagaggat
119161 aatgaaacct ttccagagga caatttgacg agacgtcgcc accgtcagta cgaaatgtca
119221 ctgttatttg tgtgtccctt ccggttccac aagtgttgcc tttttagcat aaatagcatt
119281 attagctttg gttttataaa tgttattacc ctcagaactc ttattaaaag attttatgtg
119341 gaatggagct gtacctcctt tattgtggcc caactatact ttgaaaataa cctaaattgg
119401 agtaatcaca catattcgac ggaggacgat aaggataatt gctggtttcc tttgaactac
119461 aataagttct ttttttgttg ttacggcaac tgcaaattgc cacaattggt tgtaaaactg
119521 gaccccacct tggcctcaca ctcactgacg tataaaacac acaagtgctc acttataata
119581 tgaaatacgt cattgaaaat aggaaatttc tcgggcaaac tggctcgatt tctatctgta
119641 tatcgcaggt gactcctctg gagaaagcag ggtgaacatg acttgccaca gtcacccccca
119701 ctaacagcca cttctgcctg acagagagca atgaacaggt gataaatcct tgtcagcttt
119761 tattaaaacc tggccaggcc gatcccaacg cctcatattg agctgtaata cctgaggaga
119821 cagtggactg cacgagagcg gaagactgtc cctacttggt atgctcgcca gctgccgtct
119881 cctctctgat cctgttaaag tggagaaaga gaagctctaa gaacactcat tcccatccac
119941 caccactctc aggccgaggc gaacgctcct gccccctgct ggctagttac accagtgggg

120001 ggcaaactgt cactggtctg caataatctc agattacagg ctgctgaata
gaaagccggg
120061 ccgctgtaaa tcaacacaag gtgaacgctg acgtctcaag agaagcagaa
attcaggaag
120121 atacgctcat accatatgtt ggggggtgga ggggtggggg ggcaatctga
gtgcactgaa
120181 tgttcctgaa aagcactaaa gctcttctgc aaatgaggtc tgcaaggcag
aagcctccaa
120241 ctctgatctg gacacacata gaacagcaaa actgaattat tctcacacca
gttatgcatt
120301 tctttaaata ttccatttct ccccctgag aggcgccttc tcggttgacc
tctccgcagg
120361 tcggcctgct ggccggacaa caatggaaat cctcacttcg ctcattattt
atttacctgg
120421 cagaggagca cttttcagac aattaatcaa gcgggtgaaa gatttatcag
cagcttgacc
120481 ggagagcccg gacattaaac gggtcgagtt gtcaaatact gggagtgatc
tgtgcggacc
120541 aattactccg tcgtgtcacc gcgacccaaa taacccaatt tacggtgttg
ttgaacaacg
120601 taaaactgaa aacatgtgtg cggtgtgtgc aacgctggag tggcgggctg
gacaaagcac
120661 ttgaagggaa gaacgcccat gtgctcctct catggaggac gtccagtcgg
gtgtgcagca
120721 ataccttaac ggtgctggtg cggcttcacg actgactgca ctcaccatat
gtaagagctt
120781 gtgtcatctc agcacatttc agaaagccgg cctgaaagca gatagccaca
cactccctcc
120841 ctccctccct ccctccctcc ctgtctttca ctgcctcctc agggtctttg
tccttgatga
120901 ccaagccgtt gggtggtcca tctgaccgcg ctgaccctca cgctccgtct
gacgtaaatt
120961 cactaatggt taccgtcgcg ctcctcttgt gacaggtgct cctgcgctca
gctgatgggc
121021 tcctgcagag acggaatgga gcgagaggcg ctgatatgcg ggtatgtctg
tcggcggaga
121081 ggggggata tctggaatgt ttggaaatat agttgcaatt cccaggctgc
ctagaaaaag
121141 caacaaggga acatgctcct ggattaggtc tccgtttcca ccagagccat
cgttcacaca
121201 acagagcttt aacttctctc tcatcctgct tggaccacac cagtatggac
tcactgcttc
121261 agcatctatc tcatttgaga tatgtggcaa cgtcaccatc caggaccgta
tatctgacag
121321 cagccacagt ggccgtaacc acggatgtca agaggtgaca ggttcatgaa
gcagacatga
121381 ccacagcgca cagcgtgtga aaatgcccgc cgttcagcca cgaaagaggg
atttatggag
121441 ccgacgcatg tcgtttatta acaccggtat cgttactgcc aagcaaacat
gatcacatga
121501 aatacacgtg tcaggaacac agtttgacag gcctctgcag gaatgcagca
gtggaaatat
121561 gtggtaagag tggtggtaaa agctaagcac aggaagctcc atccgacatg
gatgttggtg

121621 catttttact tcaatgatgt gtaacactgt taacctgaaa gagaagtgat
gtaataatca
121681 caggacgtcc ccacaatgag tcatttttgt gggttgattg gaactgtgtt
ggaaagtcac
121741 cattgaggag aacataatat gtgcaggaac ttgagtgaac ctgggaggtg
gtaaacaatc
121801 ctggcaagtt ctggatttgg gtagatctgc ggttctgggg cggttggttt
cttcgatgga
121861 gggatttact cagagctctg gtgtccaaaa ccgagaacca aaccacaaga
taaggtgata
121921 agttcaagta ccttatatgg tgttgggaca actcctagag agcaccttta
aatacgagtt
121981 ctcaaataat taccttttt tcttgggctc ccaaggaagc acattcaact
ccaaggctaa
122041 aaagagactg ttagcgttta ttattgttac tctgtgactc tcacagctgg
aatatctttg
122101 aaaaccttgg cacaagtgtg ctggatggtg ttcattttca gctgtgaaaa
ctgtttgccg
122161 tctagctaaa aaaacaaaga gcgggagaga gtgctgggat tggatcgctg
ttgctctctg
122221 taattcagtc atccattttc tgtaaatata cagctgggaa aaggcaaaga
gccgtgcatg
122281 caccgatgtt tggatggctg aattaaaatg gaatctcaca gcgctgtcct
ctgcatcgct
122341 ccatcccaat ctcctcctat acattggccc ttctctataa acaccgcaat
cagcgcatct
122401 ctcgtcccct caaaccaaaa ccagacctcc gaaaacagct ttgcggctac
ggcggcgctc
122461 aaggaaccaa gtggagggaa acctgctgga cttttaccac tttaggcagg
aaggaagctc
122521 aggagagaga aagaggagtc cgtaacgagg gccccggttg acatgggctg
tgccaacagt
122581 ggttccatac aggcatctaa gaggagaggt tggcatcttc tagagaagaa
aggggaaaca
122641 ggagaatgtc accaagtaaa ctggacgcca atgagtcatg ttcaggtttt
gatctcataa
122701 tctttttgaa gagtgagcat caacagttag agttgagtgg taccataacg
ggagatgtta
122761 gtgtgctggg gaggtgctga acgttacacc agggaaatga aacaccagac
ctgccgagac
122821 attttggtcc taccagccca gaatgtttct gaccacctgc ctccttcata
ggagagagat
122881 gagaggggga cgtgggggct gtcatttggc cccttgctcc tgcagggact
ttggggtgac
122941 actacaggtc tggagtcgtt actgcgtgtt tccacggcag gaatgtctcc
tgaacagcta
123001 tttacaggaa cctgctcagg ggcaggtact tagaatggcc tggaaaactc
caggtggggc
123061 ttgagtatta ctcagcattg atttgctttt cctttcattc atcgcatccg
ccatgtgtaa
123121 aaagaaatca ggaatgatgc aaccgtctgt cttcttttac agttgaaaag
caacacaaca
123181 cgtgctgttt acttaatatt tatattttca gcttgctcac aactagaaaa
aaaggtgcag

123241 ggggaaccct tcatagctgc actcttcaca gagctgtagg acaccatatt ctcatagaat
123301 aggacaccat ttcagatcct tccatgtttg tctccatccc cattgcagtt ttcttcatta
123361 ttatttctgc tttttgaggg ccaagtgagg ggaacggttc ctgtaaagtt cccgctccct
123421 atatttacca ggaactcttg gagtggaaac gctccattat cttgcccctg tttgtgtgct
123481 tgtgtgggtg tgtgtcgggg ggattgactc aactaccttc tctgcatttc aaagtgtctg
123541 cctttgtttg tgccggggtt tcttattttg cgctggggtc catttggcga gcgtctccct
123601 gtgatctggg ccacaatgga gcagaggggg acaagtgctg ggacaccgtt tggagcacac
123661 tttcttcttt catcttctat tagtatgaaa gccatggaca tcaagtcatg acggaaagag
123721 ggatgcttat tatcatcctt taaaccaacg cacacaagaa catgggagac catctttacg
123781 aggtccagac agacacgtat aaccctgaaa atgggaggca aaagcaaatt atggatccaa
123841 ttttatttta ttcaatatcc tgatgcctcg tgcttgacaa agtacctctt ttacgttgat
123901 tctcgagcgt aggcatcata acagatgaaa agcaattcag cgatacggcc aaagccggct
123961 cgccaccaca ctcaggcaat cacgcgctgg atgcgcggac acgtaatcaa ttgagaaatc
124021 catgacggtt cggccattcg gaacacacgg ggagacaggc agtcatccct cacgcaccct
124081 ttcgtgactg atgcatctac ttcccgtcta cggcgtgtac gaccacattg cacgcgcgca
124141 acattggatt gtgcataatg tatagaggtt gtctaaacac catgatggtg aaaccggcaa
124201 tgagaatcag tcattctgcc gagactgcat gttttcattt tgagtgattg gccttttggg
124261 ggttagggtt ggggggggacc tgactgcagt tgcgtctgta ggctttgacc ctacaaatag
124321 aagtgacctt aataagctgt gctccatcaa ctgctgtctg ggtcttaaaa gcttccgaaa
124381 gaataaagaa atgcaataca gtaaatgtac caatatcttc agattgtggg ttgtgaaatg
124441 aaactgcttg gtgtgttgat cagatcttta gagattctat tagccctatt ataatcattc
124501 tgattgtttt ctagtacatt actaagatgg taaacaggca actcgctaga actttagatt
124561 catatcaggt ttgacagtta tggcaatgac caaaaacaaa atattaaaaa tgaataaccc
124621 atcatgaatt aatgatccac tatgaaaaga gttgttccac taaggcagtt taaaatacaa
124681 atgcacagta atacaattat ctaaatttga ccattattta agatacagat tgcttcccag
124741 atcattttgg cctctacagg cacattagta ttgtaataga gcacattatg aggtcacaga
124801 ggtgtaaagt gctagtagca gttagttgct aactcaaaca tgagaaacca aaatcgacct

124861 aactgcagag acaatgcagg tttctgctat tacaggaacg gtgaagggtt ttttgtcaca
124921 ttatactgtg ctgctactgc tgcacgtagt gttgcacaca ccttttaagc tatgccagtt
124981 tattattgtg ggatgcaggt tagctggcct actgtgcatg agatcccagg ttcaatatcc
125041 tttgctcaag gacactttga cacgtaaagt acagtagatc agacctccaa ccttcacatc
125101 actggacgag tgttcagctc attacagcct gtatttatag tatatttagt gcgaggcagc
125161 acccccggtg acaggagtaa ttatgacaga tggataggta gaaggcatgc tatgtaatat
125221 aaagggcaat taagtgtgct tatggagaag gctggggtgg ttgattaggt caaacacacc
125281 cttcatccat ggggctgcgt ggaaccctga atcagtgtcg acttatttca agtttagact
125341 taaacaggcc atcacagtga tgctaaatgc tactcaatca gcgtccttta aaagtggacg
125401 tattcatgac accgacagca catgagccac tttctttcgg ggtttccagg aacacgtttt
125461 ccttttttgtt aaacaaagag tataaagaat cagatctgct ggcagtacaa gcaacacagc
125521 agtgtctctt tcaccgcctg ctgcagtaca gggacgacgc gtcaccgtac gccaggcagg
125581 ccggcacctg ggggagttct ggcagcgctc ggctgctgtc gcgcccgtga ttgagctgag
125641 cggagatgcg aggaagcagg gatgcagata cgagaggagg agaagaagag gcgggaggga
125701 gaggcggatg aaagaggagg ctctgacaga tctgagaaag aggtggcagg gaagagatgg
125761 aagcgatggg aagagatgga ggcagggagc ccggcagggg ctccggctgc cccccctcgg
125821 ccagggggaa ggatgagaca gtggaaagac ccaataacca gggacgtgtc ctgggcatgt
125881 cctggtttcg atgctatttt ctgtaaaatg aactttccaa gagtccagtg gctccaaatc
125941 cccaacagcc acggagtttg gtagccatca gtcggctgga agggagacat ttatagaatt
126001 tgactgagag acatcctcta actgtcaggc cttcagtcct ccccctaatc taatcccggt
126061 tcagtgtcag atggtggggt tttattgcgt ctgacaccag gtggattatg tcagttaatt
126121 caccacaaat caaacaaagg ctaacgctaa tccagcacag tgctccggtg tgatgtctga
126181 cgctctggat ttattcacaa cattccagca accagcctaa tccttaacga accagacggg
126241 acccgtatcc caactgacag tttctgcctc gttatggctg atgtgcttca actccccttt
126301 ccccctaaaa tcatctcaat gaaggagaca acaaaggttg aggatttata tctataaatc
126361 tatctatatc tatctgtctc tctctatata tatatgcaca cacacacaca tacatgtgcc
126421 tcttttcccc agcattcccg cagcagccat taagcccata ttcagagtgc attctgcttc

126481 tcttaaggtt taagttttga aattttcaaa tcaatagatg gcagagaccc
cccccccaca
126541 cacacacatt catgaagttt ggctgaaaac acttcaatca ggccctctga
ggatgtgaca
126601 caggagacag attgatcgat ctgtaatgag tggaggccta cctggggttc
cactccaggt
126661 ctcatctcct tccagctact tccagcctaa agctgccatt cccacatttg
ttgattacaa
126721 caacttgtgg aagtagattt tcctttttgt ccaaaactac agaaatagaa
agaaaattgg
126781 agaacaaaac caaactcctg atttggtcgg tcagtaccaa ggttgtgctt
ttatgagtgc
126841 agcacagtta gagcctgtct gagcggaggg ccgcaggaac aatgagtagg
tcacgatcgc
126901 agattgatga ggaaagaagg aagaggaagt aggtaatagg gaccatttgc
tatttaggga
126961 ggatgagaaa atgtcttaaa cctgcgagtg gggagccgt tttggtaagt
gaggacaatc
127021 tggctgctct tcacaactcc aaggggctgt ttgagggtga agatttggat
ttaaggaagt
127081 caacaggcat gcaaaataca tataaacaaa gaaaaaaaaa ttcctgactt
tgtattaatg
127141 gtatagtcaa acctcttttt ttatataaaa actattcaaa ttgagcctat
ttggcttcta
127201 tgtctcattc tgtgtaatga ggcaaaacca gtggagttaa actggaactc
agatgtctgt
127261 gatacatcag gattggaaca aatgtggact ttagcaatga ctgatgttac
attctaggcc
127321 tgattattat cccagtctct gtgtgtgtgt gtctgtgtgt gtgtgtgtgt
gtggtactat
127381 gaatgctgtc tactactgga cagccccca gcccactact gcagccccac
aactgttcca
127441 gaaatgtatt ggctgtttca aaagatgcta ttgtatcatc aagccagccc
agctaattag
127501 gctgcagtcg gccttcctct gttgaataca cagtccctct gggccctgca
gctgctccta
127561 ctatagaggt taagtgctaa aacatgttcc ccccgctaaa tgactcacag
agctacagag
127621 ccagtgaatt tgccagttgc taaagagaac tttgtgtttg tcagagcagc
caaacgacgc
127681 agctcagagc agcgatccgt tcacaagcag gacagtttgt ctttggaatg
atgcgtttaa
127741 ggtccatctg gattattctg gtgtgagctt cgtttatagg aaaatgttgt
atctctccag
127801 aaacaagtca agatgatcca gaacttcctg tgagcagttt gacggtggaa
tcattctcat
127861 ttccacgtct ctgcacaaat gtacccagac aacacgcagt ctcatagatg
ttgaccataa
127921 gttacaccag caagatgtaa cacagacaag agttactaat tggcaatatg
ctgcaagatg
127981 acagctttta atcatgactt ctcctgaaac aatcttggga taaaagcatg
cgtatagctc
128041 actgatgtca ggctgatata tagagatgtt tgctggatgt catttggaat
ttttgtggta

128101 attcggcata tttcacagac atttttggat ctaatataca gtgaccacta gatagcattt
128161 ggatgatgca gttgtcagca acagatttgc ttttcattta acgtttacag tgtgttgggc
128221 acatatttga ccatgtaatg tggaaaaact acatacctca ttcgggtggt ctgcagctaa
128281 tttagcttct tcttctgcct tgtgttgatg gcagcctaca aacggtgagg tgggtgctgc
128341 cacctatcat cctgggtgta tcacttcatg cttggtttta atggggtggt taaactccat
128401 gaggccaaca gtgtttatat gctagatgtg taggatctgc ttgtcatcgt aggcattagt
128461 gaccactagc tgtaataatg ttcccatcat gcaacaggcc gctaactcaa tggcatcctt
128521 ctaacatgga ggtgcaatat ggagatagat ccttttttgat acaaatgagg tctgtggatt
128581 atgattaggt ggcatttttg aaaattgctt tgaacgcagg gtaaactgtg ccagctgctg
128641 tccacagatg actatctgcg tttgccgtaa atcctccgtg gacggacgtg tgcctctcgc
128701 tgacttcaaa cacggtgacc taatcacact tcacctcctg ctcccttcat ctccaggaat
128761 tcacatgatg tcatcgggcc cgtgcccagt cactcccccc caacgcagcc gcgcgcgcag
128821 gcgtgcgcca ggactttggc gtagttcatc cgccatccgt cagaattcat cgtggagcca
128881 gcagtataca gccggcatcg actgtaaaaa ttagacagta aataagtgga aggcagtaaa
128941 agtggtccaa aatatcacat taaaacgcca tgttgcgttt tggatgctat taaatctgca
129001 aaaaaggcag tgatttgagg cctcccctct aatggtgtca cttgcctctc cacgcgcgcc
129061 tcgctgtaga tttgttatat ggcggcccgg taaagtggtc caatgtgaaa accatctacc
129121 caaagtcaga gaaatgtgat gtgaatggac ggcgataata aatagcggcg ataagcgggg
129181 gagggaccca tcattcgccg gggagatgag tccgcgcgag gacgaagcgg ccataaaagc
129241 ctgaatgaag agatgtaaaa caataaggcc atgagaggtg ggtggtgggg gtgggggctg
129301 gagagctgcg acgacccctg gcaagtgagt gcgggatgga gatagagaga taaacgcaga
129361 gcaacagaat cggaagagaa caggaggcgg agaaaagaaa gattgcgagt cagacgcgtc
129421 tgcactccat caaaaactgt gtcagtcatt cgctaccagt gacccccccc cccccccca
129481 ctgcaccact cctctctctt caactaacac attaattcca cttcttttaa ccgtcctcaa
129541 ctgaatgtca atcctttgct cagggtgaca gaggactgag cgagggaggg gacagggaca
129601 tgaccggtgg agagctgctc ggaatcctca ggggcctttt ctatccggag gtttgactcc
129661 agacgaccct cctccttctc catttgaatt atacccсttt gattaggccc cctgtctcct

129721 ggactggtgt gcccaacggt ttcccagtgt ttgatgggag ggaggacagg acatgtctgt
129781 ctgtgtctca gacgttttca gaacactgac acagaatcaa gactggtgac cacgtcctga
129841 cacagaagcc agtacatcac agcataaacc gccatcttgc acctcgctgg ctgcctttac
129901 ttctggaacg ctcatctgtc caccatgaca tttaaccctg gtttttttaaa aactgtactc
129961 taactaaatg tactctaacc tgaaacgagg aaacatcgga gctgtacaaa gacaggaaac
130021 actcaggagg aaaagtttcc acgtgtcagg ggcacctgtg tgtgcacatg aacgggtgcg
130081 tgcacgcgtg tcctgccttt ccctcagatc agaaggcctg atgaatagaa cacaagtggt
130141 gtatatctgg tcaacatggc gtgcgaggac aataattaca cttccaggct gacacccaag
130201 gtcgacgggc tccaacagct gcccaatcac tgccggctat ttttcctcct cctgtctgga
130261 cttggcagcg cataaaaaaa aaacacatca cggtgttttt catgttgtca catgatagca
130321 gatgaaggcc cgagtcggta ccgcacgctc ccaacaggag agggatgtcg agacgagaca
130381 caagtcatca ccgatgtttc cccactcacc tgttagcttt gctctcatat tcgtccgcca
130441 ggagtccaga gggaaaggac gaattaagat tgaaatcagg attcaaagtg gggtgtaatg
130501 ggggggctgc gcccctgttg ctgtcttcgg gggtagagtc gatacgatct gtttggaaga
130561 tagaggtgat gcatccgcct gctcaggatt ccacctttta cggctcgtcc cccacatgtc
130621 tcgctcacca tcaagaagga cacctgatgg acagaccgac tccccccccc ccgacttggg
130681 atggaaagaa agatccattt gacgatatca gatggccgct gaacgctcat ctcactgtcc
130741 ctgagcgcgg cgcctcctgc ggctaacggc taacccttgg ctaggggtga aaaatggaga
130801 cgggagacgg gggagcggtc ctgggtgaaa ttcaaggcca acacactcac gtctctaagg
130861 tgtaacagtc ctggatataa ccacacagtc ggaagctttg gacgtgggtg accataactc
130921 atgtgtgtgt gtgtgtgtgt gtgtgtgtgt gtgtgcaggt gacaagcatt tctgtttgtg
130981 tctctgagga aatgacagga ctacacatcc catcatgagg gttcctctcc agatgattct
131041 tttttattaa catgtcccct tttttttttca tcaaccgaaa aggttactgg ttcatttgta
131101 ataccccaca tgaaccgtct ccaccccgcc aaagcccccc ccccccccca tcagacgtgt
131161 cctttagctg tgagaggaca tttcaatggc gccgtcctcc cctctgcctc ccaaacacag
131221 gtagggaaat atcaaccggg gaataaataa cccagccagg gttaattaat tggcggacac
131281 gtttgcagat taataactga gtaaaaaaaa cggcgatgga cggaaaaggt gcacgtggat

131341 gaggcggacg gggatgaggg gcaattgagt ggagttattg gagggcaaca
gtgcacgcgc
131401 cagcgtcaga tagatgcagg agcaaagttt agcgcccaag gctgcgaaca
cggcgtctga
131461 gagcagagag attaactgag cctggtgaca tcacgcctgt agggacggtt
acgaggagca
131521 tccatcacag gcaaaatatg gcaaaaacag aagcagcaca ggtccgtttc
ataaagattt
131581 atttaaaaag aaaaacacat atgtaagagg aattaagaag tggttcttta
tttgggttca
131641 gacctgcacg cttctgcctg ctggaactgt tccctccacc atggcgaggt
gttattcctg
131701 tgttataact cttcctgcct cgctgatagt aaaatacaca cgcagaccag
cgcacatgtg
131761 agatcctgca ggatctgatc acttcagcag caacatgtgg agttattatc
atcacaaatg
131821 cacaaacaac ctaaataccc ttaatctaga caaggggtaa agcctgtatt
gacctagtgg
131881 tgtgtgtgtg tgtgtgtgtg tgtgtgtgtg tgtgtgtgtg tgcgtgtgtg
tgtgtgtgtg
131941 tgtctgacct agtctgcttg ggcagtgaca gaacccctga gctcagttca
cctgaggagg
132001 gcagactggg gtcatttttc tctttttgaa gagagagaga catagagaga
catagagaga
132061 gagagagtcc ctgttgtttg tttcccactg ggaaaatctt gacacagcaa
gaacattagt
132121 ggtaaccaaa cctgtgtgat ttcttatttc gtgacagaag gattcttttc
tttatctttt
132181 aaaaccttat caatgtttat ttactactct cactctcatg ttatttttgg
accctgacga
132241 ccagcagaac tataaacctg gatccacaca gaatacagag ctggtcgtgt
ggtgtaagac
132301 agcacatttg aggctttatg aacataaaga tccgatttta tctgtgtggg
agtttggctg
132361 cctttgattg atgcttctcc ttcttaaagg tcgctctctg gctgaccctc
cagactccgg
132421 gctgctgttg ctcccatcac tgatcacctt caggcccgtc ttcagatgga
aggaggcgga
132481 aggggaagcc aaaggaaaga gctgagactg agcagaagag gaatcaagga
ctcctcggag
132541 gcctctggac gtgttcctgc accaggtcat ttgtgtgtgt ggtttttgcc
tcgctccctt
132601 cccccaccca tccatctatc tgccctccac ccaccgatct cccctctcac
ccctctggcc
132661 ctgcagggca ggccctgcct tccatctgtt gtctgtccca gctaggagca
gcctggtccc
132721 ccctccactt gcatttacaa ttcaattaaa aacaccaacc ccttcttcct
cctccctctc
132781 ctcttccttc tcctcctcct ccactttttt tcccctgccg tgcctggatg
agcacagcag
132841 aaagaccccg caccccatgc ggggatgcgg ccatgacaca cacacttact
gagcccgcgg
132901 atgacgcaaa cacacactga cacacaaaca cagtgcgcgc gagggagcag
aaagggagtg

132961 acacggggag acggtgtcgc tcacgcacag gcggatcttt ttagccccgt ttggacggag
133021 tgaatagaaa acagggttgg tggggctaac ccagacaaca ttaccgctgc agaaagtggg
133081 tcagactttg cattagaata acgtatgtaa tgatgatgta acagcccaag aacaactgaa
133141 aaagtgggat tgtgcgatag atgagggggg ggttacggcc ctgtgtgtct ggaaaaggag
133201 actacggagg gaagctaagc gctgctagct tgattctgct aatcataaag ttctcctttt
133261 tatgtgatag gagttagagg ctgcgtaaga gtgcgagcgt ctacattcaa gcccttcgtg
133321 aagctctgtt gctgctgtaa gaaggaaata tccacaggca tccctcttct tggaaaaaaa
133381 gaagattggg agggcgagtg tttaatattt aattacggtg gtgccaacat gcaactttta
133441 tcactttctt gtctgaggcg aggccagaag atacacgcgg cgccacgggg gcgagagaaa
133501 gacaaagact aggtggttcc aggtttggag gggcgagagg aggcggccgc ggcggggagg
133561 aaaggccgcc atcgggaggc ccgtccggcg gggagagagc aggaggaagc aaggaaagca
133621 ctcaaagggg gctgctcgtg tcaaatatac cgcagagccc ccccccgtcc accccatcaa
133681 acggcttccc ctgcctacct tcggctgccc gttggcgctg aaccagaaag cagtaatgac
133741 tgataacgtg tgtaattacc agtccaacag ccaggcacag ctgatgagga atcagaccag
133801 cacccccccc cccaccccga cccccccccc cccccccccc atcgttcccc ttcattcacc
133861 ctgaatggtg gcgcgctcct tattggatgc tgctgctgca gtcggtcatt aagacggccg
133921 tggaaatgaa aggaaggggg gagctgccat taacatgaga atacatgtca aagccatttt
133981 catattgctt taatttcagt gcaaagaggc ggcggctctt ctctcgctgc atcatcctat
134041 agaatcagca ttgtgcttta atgtaaggat ctctgacagc acgcaaacag ctggataatg
134101 gctgatgagc tgccctctct tttgctcgct tcatcctgat ctttggcttc aaagtagccg
134161 aactgctgta aatatttgct cgactgaacc cagacgggtc tggagtccag gacctccact
134221 tggataaacg tggacacctt taggcttctg ttcttccctc taaacgggaa atcttgggag
134281 catctcgggc cacgcgcacg tttgctggct tcccgggacc ccggcgcgct ggcggctgga
134341 gcagatgtcg gctttccagc tcgcagaacc tccgcgtttc ccatgacgca tcgatggagg
134401 gatcgcctcg ggccaggagc gttcagaatg aaacgtggtc ggagcgagga tacggggaag
134461 tggcgcgcag agggaggcga gagccggcgc ttggaaacca ctcagcacag gagggggcct
134521 gagaatgtgg tgcactgccc agggtccgct ctgttcggct ccgcgctctc cgctggctct

134581 caggaataaa tacttgactg tgtttgaaga cttttctgaa tctgggatat ccccacagca
134641 ggtttctcag tctgctggga tgaaaactcc tctgatcttc ctccgtttcc aactattaca
134701 tgagactctg aagaggatgc ttttaagcta tttatgtaac aaagtccaaa ggaaaagttc
134761 tgacagcatc agaggccatt tgcaattaac aggtcgggtg tgttgaggat gggagtgaag
134821 cagggtagct aattaatctt gaagctaaaa ttaaaaatcc tgtagattaa gatgagtgat
134881 catcatacat ggaggattct gctgttaggg ttagggttag ggttagggtt agggttaggg
134941 ttagggttag ggttagggtt agggttaggg ttagggacat gaactattca aataacacat
135001 gtttataaag atatcagcag tgctaactgc tgttgctaac attagccccg ctcatctcag
135061 cggggtggtt gccagttatg ttgttacatg ctcacacgcc tcttgagagc aaaaagtgag
135121 ggttagcatt tcatttcact ctgttaaaca agcgcagtgt agcatgctaa catgtttaaa
135181 atgactacta actacaacag tatgctaaaa ccatttaaaa acgcaggttt tgttccactg
135241 ataacaggta cagctataga tggtggcgtt ttgcacccaa caccttggtc atttttactc
135301 acagtaaatt gcgtttctgt agcgttagca tctttcatct tccctcttat ctaaatgact
135361 ccatattgca ctcagcatgc atgtgcacat atggcgtagc cccgtctcca tctgtgtacc
135421 tccgttcaga cagaaagtgc tgagctggtc gtggagttgg ggctcccgcc acgaggacct
135481 tgattagaac ctctgggtgt ttccttacca tgtaaatgat tacaacctct gacactcgtt
135541 ggaacggggt gtgacttgtt ttgagatatt ccacagcatg atggagaggg taaccgatgg
135601 gtagggtggc ggcgcggtct aacagggctt tgaggggaga cttcactctg atgaaggagc
135661 tgttttagag agagacctct catccggccc actttccgat ttgttggtcg acaactggga
135721 ctcaaaccag aatcctcgta actacccccc cacacagatg tacagatgta cagagtctgt
135781 caggatggag ttgagccaac tattgtagtg gcgacttctg gtggatccat caaatgcgag
135841 gccaatttcc aaaagccagg ctgcacctgc atacatgcta atgttaaatc aaagatatta
135901 gcacagctca aacatgtgct gtgcagagga ggaagcacat gttccttcat ccacatgtgt
135961 cagtggttgt ttaaagagag gaaacaggca gagttggcgt ggagatctga gctggtgtct
136021 gttttcggct ctccctgcag agcttcaata catctggctc tctgagcagc atgttgctgc
136081 agcttaagag cagcaaatat gacatctccg aggaatggag tcaccaagtt aatagaggtg
136141 cacgcaggag ggggttgaga gagcgctatc actgaaagta tgtctgttaa tacacgacag

136201 tggcggagaa tgggatttcc agctttgtca aggttgcagc ctggcgtcca
ctctgcatca
136261 gaacaaagga gagggaaagt cagacctcat taattcccct cactcgcctt
gactttcatt
136321 ctctcgttcc cgatgagttt gttatcattt gtataccatc tgatcaatgg
gtcaatagat
136381 caatacataa caaatgacac cgcgggtgtt aatgacaacc tgggattagt
acggatcagt
136441 ccgccctgga agatcaatcg attagcagct agcctccgct acagcacacc
ccagacgtgc
136501 gccagtgctc acacgcacgc aggagctggc gcggatcatc cggggccacg
cagccccccc
136561 caacaactgt accataacac cctttcatcc ctcccagcac acgctctcca
gggcccggag
136621 aagatttgcc cgaatgccaa gatgagagac tgcgctaatg cggaaatgtc
aaagaacctt
136681 aatgaacacg ggggggctat aaccgtgatc aaggctcccc ggaggagcag
cagcagcgaa
136741 caacccaatc tctgggcctc agcctcgctc ctccggatcg atctcaatag
atttcctgct
136801 tttgcacaca cctgaacgcc gccaacagga ggagcgagtg caaatttggc
tagtttttca
136861 cctcctgcct gacccccgtg ctccattcct gttgaatcca ttttttttcc
cagcacatgg
136921 aaacctttgg agctgttttt ggcaggcggg acttgttata aaacagagga
gggtgtttgt
136981 ggtggtctgg atttaagtcg gctcttcgtg ccctcacact gaaaggctcc
tttggaaaac
137041 gaggccctgg acggctttgg tgagcgttcg gtgaaagaca cacacaccca
cacaacacac
137101 acacacgcac gcaggcccgg gttcacatgg gtcgctctct cattactcag
cataaggctt
137161 tcacgcttga cccgcttttt aactttctga tctggattag tggcagataa
tttagggagg
137221 agaggagccg ggaatgtgag gctgcctgag ctgagcggct tacaggtagt
gcgtagctat
137281 cattcaggag cggaacccaa gaccgagttg acacagctgc ctcggagatt
taccgctgct
137341 gccgccgctg ctgctgctgc gtggaggaag aaccagaaat atcagccccg
ggcacgagcg
137401 ataggactgt tctcagtcgt gctgagtgcc agaatacaat aatgatgata
ttacagttat
137461 ttcctacaaa gaattctcag tacagcttca cctatgttgc ctttacactc
acaataatgt
137521 ccttttcacg acagaggaac tatttaatac atctttcttg ccttgtttag
atcattggag
137581 ggttgaaatc attacaatag aagctacaag tggcataagt gagtgtgatg
ctgaacctta
137641 gttttttaag tggtggatta caagtcatca ttaatgcata tgctatttag
catcaggaca
137701 ctcaagcctt tccctcttgt atgcatgtga taatggataa tgttgcatgg
ttacgcttca
137761 aaatgaattt gccttcacag gatgatgtca gcttacatca tttcacaatc
aaagttagct

137821 gtgctaatgc aaccagaaaa aggccccttc agggtcggtt ctatatctgc gtttctatga
137881 atggctgtga gtagtagcac ccagctagct gtagccagaa ctagctgttt acacgaacac
137941 taattaaaag aatgaaaagc ttcaaacatg aaatgtgtag ttcaacaaag acagagtgct
138001 ggaaccctcc aaagctggat ggaagtcatc agcaacagtt tccaacaagt agcaaatagc
138061 ttacagaggc aggtgggacg gggttccatc taaagaccgg cctaattgaa gaaatatgca
138121 ttaccacaca tgtgtgtgtg tgtgtggggg gggggggtgc tatgctgtgt aataagaagt
138181 aaacccacat tttgatttca gttactgtaa atcaaacata aaaacatcag aggacctgag
138241 tttgctccag ggtgtgaatg caggatccaa ggcggtggtg gtggtggtgg tggtggtggg
138301 gggggggtct ttgggtgatg gggatcagtg ataaatccat ttacatcctc cctgttccat
138361 tctctccccc agactttgcc tgggtctcca cattcgggct tgtttacctg ttaagctgag
138421 tgtgactaaa gcaaattccc acagcccgag gtgggatcgc agccgggggc tgacggggat
138481 gcgggatgcc aggtttaagg taggcctgta agaagggtga ttgggagggg gggcgtaggc
138541 catggaatca ctcagagctg taacccacct tccctcaccc agcccccatg gcttaattat
138601 catgtgcagc ctagtggaga tgaaataacg gagactctca cacgaacacc cacacaggga
138661 ggatctgagg actcctcatg cacacacaca cacacacagg cagacaaatg cacaaatagg
138721 agcaaagaaa aaaacacggt ctgcttttaa tcgaagctaa tcggcaggga aaataaacag
138781 gctggcaggg ccgccatcta tctgcagcgg aggctgaggc tgccagataa ggagcagcgg
138841 cctcacagac acgtgttctc ctctttaaaa caccgcctgc tcctgtcggc agaattaagc
138901 ttaaatatgg gtcttagagg aaggcttctc cagggtgaaa aggtgaaatt atgggatgga
138961 tcacccccca gacacatcaa ttacattcac tgttgttatt ttttatttgc tgggggtcaa
139021 agccggcccc ccgtcgtttt cagcctttcc tcggaggagc ttctgcgact atcggattcg
139081 ccgcaccctt cgttagccga gaagagctaa gagctgaatt agcagcactt ctcatgatct
139141 ttgttgatta gaagtagcca aatgagattt aaaaagaaaa aaaaaactaa aaaaggagcg
139201 agggagcagg acctggaatc agagagggga taatcgtcca tccagtgggc agagtaaggg
139261 atccactccc taagcagctc tggtagctgt ggaatccaga cacacgtgcc tgcagaagct
139321 tttcgagcat ttaaaaagac taaaacgctc aaacttacaa tcctggactg aattttctgg
139381 ctccgtttcc gttccccatc tgacctttct gtctttatag ttgggaagtg ttgcaaaccc

139441 caggcagtaa atcattcccc acattactgc gctcaaacgc agcaattaga cgctgattaa
139501 tagcttgctt tgaagaataa tataaaaaat aaacctattg taaatcaaca agcacttcat
139561 ttgctgaact tagtttcact cgggcagtgc tgatgcgttc ctgctcctaa acggtgtttg
139621 agtgaccaac aaacagagaa gaagaggaag aaaaatggag gatgctttga tgtctcacaa
139681 aggggaaaaa aagaagaaat cccgaccatt ttgtcaagcg tctgaggcgg cggagcagcg
139741 gggcagcaga cgtgcacaca cagagccagg accggggcta atttgcgggg atcactgcac
139801 tgaccccgag cagataaggt gccggccgac gaaacttcct tcatttcctc ttgttgttgc
139861 agagtgggag cagaggtagc cagcacccgg cttaaggaca gcgggggcta aaaggtgaac
139921 gccacgatcg gctgaacttc gagcttgatc aaaaggaatg cgacctgggc tcggtgaggc
139981 agcagagctt atcggccccg cggtgaggct ctggagggag gagagagaag atcccaggcc
140041 ccagatagcg ggcggtaaaa aacgcctttt ctcaggaatt aggtgaatcc cccttttttct
140101 ctttgtcccc cccccccgac ggcagcgctg cacctcagct gaggcgacag tcggctgatg
140161 tgaattaaat gatctgacat ccaccaccag tagacacgct cacacgatgc gctgccagca
140221 gctaaaaacg caattattcc ttcagacttt ccatcagctt cgctagtttt aaacagaaag
140281 tccccgtgtt tacatatgcg ggaacccccc cccccaccat ctttactcca tctgtcctaa
140341 tgtatcattc atgattttaa ttgctagtaa ttaactgggc cccaattagc aagagagata
140401 atcaatactt gttgatcaac acacaggtgc attatcttcg gtgccattgg tagcatgttt
140461 aatgccaaga ggaaatcctg ttgactgcag atttacacct aattgaatca gtttggttta
140521 attcgggact ttacttcaaa cagattacac actgaactcc ctcgctctct ttctctctct
140581 ccctctgtct ctctctccct ccctctgtct ctctctctct ccccctgtct ctctctctct
140641 ccctctgtct ctctctccct ccctctgtct ctctctctct ccctctgtct ctatctctct
140701 ccctctgtct ctctctccct ctccctgtct ctctttctct ccctctgtct ctctctccct
140761 ctgtctctgt ctctctctct ccctctgtct ctctatctcg atatctctct ctctctctct
140821 gctgtaatcc attttaactc ggtgctaaca attatctgct gactaaagag tgaagagaag
140881 agggaaaaac acactctgtg aaatgaaagg aacaccgatc agagggaagt gaaagaggga
140941 gtagaaccag cagaaatgaa tgaatacaca acgtggactt ttctcctgta tggaggaaaa
141001 aggaatgagt tggaagtcct atgttcccct cgtccggccc tctggcacca ttcgcagggc

141061 gatgtgatcg atattgacac tgtcattaag cgactctggt aatgagggtg gggaagcggg
141121 ggtccgaggg gagacgttac ctgcaggtca gcccaccagg cgtataaagc tgccactcac
141181 cctgctccag aacctccctc gctttttaaa gattccatat cgtcacacct ccctgaatta
141241 cagcttttgg gccggtggaa gcattccacg gtggttccac gagtgcagag gcacaaatca
141301 cacgcaaaac gtgacaggat ggcaagttta ggggtaagaa ctgagcgagt gtgtgtccgt
141361 gcgtttgcaa catgttgaca accaaaacat gcattattct aacaaagcgg ggctgttttg
141421 gctgctcctc gcaactctag aggactgttt gagggtcaag acatgatttt aagggtgagg
141481 ttagacgggt tttggtcagg gttagggggt tcggggttag ctggtatgtc agggttgcgt
141541 taggaagctc cctgttgcat tatatcgatg aaagtcccca ctaagataga aatgcacaga
141601 agggtgtgtg cacgtctgtg tgtatgtgca tgagtacgtg tgattgagag agcggtcttt
141661 cattgcttgt gacggggtca ggagagtacc caactcatcc ctcggcgggc ctcactaata
141721 gccgtgcaaa catgcattta tctctgacac caaaatccaa gagctcaaaa ctgtccatca
141781 atgctcctga gaagaaagag cccccgactg aaaggacaca ggaagggaga cacgatggac
141841 ggagcgcagc cacgtcccga cggatctatg cggcgttcac ggagccggtg aaacggagcg
141901 actgggcgag cgtcacagaa tgagataatg tgcagaacta gatggcacgt tggggttgcg
141961 cgcttcaccc gggccctccc cgtctctgtc tgggtcagca ctgccgtcct gctcctgcag
142021 ctgctgcggc gaggcccgcg gtcgccgccg tgtcggccga tgactggatg acgggcagcc
142081 tgagcgcacg ggcaattcca ggaagtttaa agaaacagct cacccaaaaa ggaagtcact
142141 ccatccagaa ctatgggctg gctgtgggtt cagctcccca acagaccact gctgtaaaaa
142201 tctcatcggg atcggagcac tcggcacagc tttacgagcc gtccactttt tcggtcggat
142261 atttgctaat ttgaggagaa taatgaggaa atcatggggg atgaccggtt actgactgta
142321 gctctcatgt ctcgtccgta tttaaaggca ggaggggatg gcactgaaga aatgtcccct
142381 ctgaagcgtc aggaaggaaa cgccagtggt acgtgtggct ccgaggaagc gcctcctcat
142441 taacacggga cattcgccgg ccattccttc cagacgcgcc tcagcgggtt ttgatagatc
142501 taacgcgtcc ttgcatttga tgtacctcgg gatggtggct gatgtttcat tgtgtgctat
142561 agaggatgta ggagctgctc atgtctcttt tcccccccac gctcggcctg aaatattgcc
142621 cccagattat aggctcgcac gaatgacaga agctgcattt catacaaatg ctgtgaatac

142681 ataatgcata gcattgtctg cacgaaccct ttcacatgct aatgccaacc tttccaccgt
142741 gtctcaaagg tacgtgccgg tgactgccga gtggcagcag agcttcaaaa tagagaagga
142801 attcacaaag caaaccaaaa aaaaaataaa aaatcattag catcctaatt gccatcactc
142861 acatactgta tgtttgctaa ataattcacc ttggtgggat aaaagaagac aggtagagcc
142921 gacacggaac tggtggaggg actggaagct tccagaaatt cacaaggtgg gagaagcaca
142981 ccaagggtat gaaggattca tctacatcgg agattagcat gtcttaatgt gtaaccacag
143041 ctaaaggaag tgctctcagc agcatttgag ccagacacta aatcctgacc cagttaattg
143101 tatgaaggga cagtaaaggc caaatgctgt tgagaggcaa ttgaaatgct cctttagcgt
143161 ttcaggaaag ttcccgcaca taatgatgct ccgatgacct ctacagctgc agcaggggtg
143221 gttataggtt caaaactagg tttagtgctc gaggatgcac attaaaggtt gaaccagtca
143281 cctctgtatt tgaaaagaat cagctggact caagatgctg cgatgtgtgt ctttacccaa
143341 aagtacaatt tttaactttg cttttaactt tgttaattgg ttgactgatg aaggaaataa
143401 ggaaaatgca gttttctgtg gtttccaaaa aaaaaactgc tgcgaggagt tcccccctgt
143461 ccagtgacac atgaaaggac cggggtacct caggcccatc tgtagctcca tgtggtttcc
143521 tcaggaatga tcatgtgatc tccaacatgg tgaacgttta accctgcacg ctgcatcagt
143581 ctggaatgtt ggacagtgac tcgtcttcag gtgcagttga gtgagtgaaa catggctaaa
143641 ataaatcctg acgtgctctg ttagattaga acactcagaa ccattaacgg gctcatttaa
143701 aagagctgca aggatgcttt actgaagtaa aagaacaata aaatatcaca attttacaaa
143761 tggaaatctg tttttaagca tttaatcaat gagcagggct gaacagggtc atttctgcag
143821 ctccaggggg ggagagctca cgttcagcac tttaggtgtg ttttcttttc cttcttttgt
143881 gtttggacgg ttctgggtga attatagctg cctttgttga caacgcccca gtgaccttgc
143941 tgccctgcgg gtgccaggaa cactggacgg accgacatgg acagggcccg tgtttaccga
144001 gtggtcctca gctgcgcttg cacccgtgat tgcttatctg ttccagttct atgctggtcc
144061 cgtcgctgtg aggtgacaca gagagctgat ctctgaccgg agtttgcttt ttgagacgct
144121 cgactccacc gtaggtctgt ggtctcaggt ttctctggcg cttgtgtcca tttagtgtcc
144181 tccccactgt tgactttcta gcctgctgct atgtctaaat aaacagcgac tgggtttagg
144241 ctagctctcc catccctgcc atcgctactc ctcgctctta tctgatgcag gcctcagaga

144301 ccagactggc aacccccccta ttttggaaca ttttaaagaa taattacagt ctttttttatt
144361 cccagaatat gccctgaact ttgactttta aacccccaga cagccacaac taatggaaag
144421 acgatgtctt cctcagagga ctattgaggg taattatgtt attcaataaa aaagctttaa
144481 caaattactt atggtatgga aaaatgccct ccattgctat tttttcctct ttcatatcat
144541 gcagccttcc attattgggc aaaacaatgg agaattggat ctgcgctgtt attgatttca
144601 aatgaattgc aaaagtaaat tattgattgt ttgcaaacag gccagcgaag ttataaataa
144661 agaaaaatac tcgcctctcc atcttccaat aacggtcatt atgtcccaga aagaaagtgg
144721 attatggaac caacctgacg aatgcaccag aaactctcag aggaatcctc tgctggcttc
144781 aagcagaggt tctaacgcac atttcagtga cctgttaaga gcattatggg cccccggtcc
144841 acattggccc actgccctgg taatctggcc catcctctta aaagtggaat tgattgaata
144901 aattgatctt ctctgggttg gcagacgcag cctgaataat tacctcacgt aaatctcagc
144961 cctcacgtat gaaatgtcag cgacattttt gccttcagcc tgtttctcag ctaaagtttg
145021 gcctggatta aagaaaatgg tgagatgcta cggttcctgg tctgctagca aagactagtc
145081 cacatgatga atgtaacaat aaaactcctt ttgtggtctc caaacttgca aatatgttgt
145141 tttttcctga ctcctaagat cccaggtgca cttcctgaaa ggaattgttc actcatttgt
145201 taggcaagat ttggttggat ggctggttaa tgagcttttg gaaggctggg ttatcaatat
145261 ttccagggct ggtttgtggt tactggttac actgggagga tagatgcagc agtaaactac
145321 ttttttaggt tttaaaggaa tttggaggac atgttagaca gaaaggaaaa acattggtcg
145381 atgcttatta cgcactataa atgtcaaaag agtttgacca gcaaacacac aataaaataa
145441 agaaacaaaa ctcaactcaa gcaacaagtg taattaggtt ggttggaatg atttggatat
145501 gtctactttg ggaagaagaa aaaaacagaa aataacctag gtatgaacaa aaccttaatt
145561 ataagaccgt ggctcaacaa acagatagca aatatgcaga aggagccaaa gaaccctaaa
145621 aagcagctgc aaagatggga gggtgcagga ttagggcctt agcccactca gatggggcat
145681 ttccatgaca gtgagctgca ccgtggctta acagcccgcc cctgtttatg ctttttggag
145741 aagaagccgg attccctccc cgtgacagat ggcctttcag ccacaaagtg ctgtgtttag
145801 acttagcttg ctctggggca agacaaggca aagctctgat gacacagcat tcggcaaaca
145861 actcccagat gcaggagcgg tgaccacgga aagcacagag aactggacga cccaccgagt

145921 cagtaaccgg ctgaacagag acggctcggc ttcgattaaa cctgccaatg agcagggagt
146981 gtaaacaagg gacggaggag ataatgatgg ggcttacaaa ccccaaaaag tggcttggct
146041 taaacataca tgacacaact taatttagat tcaacccaaa tggatctaca aaatttgctc
146101 acttcacccg aaccacccga ctggatcgac cctctccagc taaaaaaaaa aaaggaaaaa
146161 aaaacagact gctttcaatt aagaggattg ttaaatttgg tcaattgaac tctaaagcaa
146221 gtttttaaag gggcagaata acagaggagt ttgcgtttca gggtgaagtg aaaaggacca
146281 gctgctggag agaattagcc cagtctcatc tgatccaaca cacacccagg ctgctctcct
146341 tgtgctcata cttagaaaaa agaaatctgg cctgacccag tttgaccccc cattcaaagc
146401 cagggaggcc ctggaggtgg tgaattaatt gtagctcacc gagtctgatg ggccatatct
146461 catacaggac tcgggctcat gttcagaagt gaggagcaga ctataaaagg gcagttttgg
146521 tcggctggtc cacagtaaac aacataaaca ctacgtatgc atctgcttgc ggggctgccc
146581 ttctaaaagc agaaacgggt tagttcagag ggcgttcaca ggctttgacg ctcgcagaaa
146641 gacgtggaga acacggggca cgtctcagga cagctgatgt aaaatgcgct gaaatcatct
146701 aaagagtgga ggcgtgcttt tgccgcattt cacgctggct gctcacactt caaagaaaca
146761 ccgctggagc cacctaaatt ggcctctcac accgtataga ttttcccatc acgggccggc
146821 cagcatctaa agaagacaga gggtgggggg cagccgggct gaccctattg tttcgaataa
146881 aattaaacat ttgtcggtcc gttaatagaa agagatggct tgtttcagtg tgaaaggtat
146941 acctgacata cctgctttat ttacacgagt atgagatgca tatgttaata aataagaaga
147001 actaaaatgg ccattatcat taactgactc aaagcatcca ggtgccccag gatgaggctg
147061 gaggtcttcc tggacagtga tagtcacata acagttatgg cagcatggga aacaagaaat
147121 gtcctgtctg ggggcttcct gacgtcagtg aatcgatagc gcctgaccag gaattttaaa
147181 agataccatc agtccaactg ctgggggcaa taaactgaag attaaccttt atgttcctcc
147241 cagtccaggg aagcgccctg aagggcatgc tctactgttt actaatcaaa aaaagttgca
147301 gcgtcgatac cataagacca gggaatcact tagtggctct gcactagacc ctgaaagtta
147361 ttgacttctt cccttaccta tatgagcatc ataaatgtga aagatggacg cctggcaacg
147421 gctttggaga tacccaggca cgagtttgct tattaaaaac aaacactata tattattgtg
147481 atgtgctgca gcacagatgt aataattcct gttgttgttg ccgagcgaag aaacagaaca

147541 tatgcagtca catgaccgga gaaccccggg gttgttgctg gtcgcttcgg ctggctgcat
147601 gtgtcctttg tgagtttcag gcaaacaaga gctccttttc tgctccagga gcgtgatcct
147661 gtttcacccc atacctttga gctgggagcc gagggttttg aatggcggtg acctctgtgg
147721 cctctcttgg cccccactgg cctccaaccc tccagctgtt catccttcca gtccagcaca
147781 ctggccagta cccccagttt gcagaggcaa tgggagaagc acccctccga gccttagaag
147841 tgaccccaa gaccagggcc acaacactgc cggcgtaacg ctgcggttcc cacatgattg
147901 ttggagcatg gtgaggagga gagagaaaag agataaagca gtgggaaatt gactggggtg
147961 tgtgagnnnn nnnnnnnnnn nnnnnnnnnn nnnnnnnnnn nnnnnnnnnn nnnnnngagt
148021 agaaggagat agatgtgctt cacacaggac aggaacatat gaaaagagtg aggagagagt
148081 ttaaggggaa gtatgtcgag ggtgctggga gcacgagggc gcgtgatcgg gagacataag
148141 cagtgacttg gcaataagac gccactgccc tccccccccc ttctgcatgg ctggctccga
148201 tgacaggaca gtattccctc atcaccgatt actaactgga tgatagctcc aatctctcgc
148261 taccctttct cacattctgt ctgtgtttgt agcgttgtct ttctttcttt ctttctttct
148321 ttctttcttt ctttctttct ttctttcttt ctttctttct ttctacatca gttcatccta
148381 aaatggccat caaaggctaa cattagccta tggtttgttt acacgtggaa gttcagcgcc
148441 ccgggcccgg ctcttacgtg agcgctccaa gaaaaagaca agtgcaactt gagaacgcta
148501 aaaaaaacgc caataatacg atgagagcat gtatgtggac gtgtgtggga ggggaagggg
148561 gtaactcttt ctgacttgca gggagggcga atggaaatgg gctctgtaca ggatgaaagc
148621 ctctgaggac gatccattga ctatgaaatt tgatgatctc ctttcacctt cagacgcaca
148681 gagagctcag cttaacattg ccctgggctc taaaccctgc agcctgcagt gtgaaaaggg
148741 ctccggagtg agcgcttata aattatattt tttgtttttt acccattctt tgtcatgaaa
148801 agaaagcaag tgcattattc ctcctccctt tacattgtat acaaaatatt cctaaatgat
148861 gcagactcca tacggggtcg gacgagttgt aagtggagtg tatattttaa tacattactc
148921 tcgggctcat aacagaagga ttcacaaagt tctctggagg acggggagtg tatttgaata
148981 tgaatgcgtg tgtgaaattg tcttatgcgt ggctgtgcat atggtgcctg tgtgtgtctt
149041 tgcacacgtg tgtttgtggt gtgcatggat ctgcacacat gcgcctggtt ggttagtggg
149101 gtgttggcag ggtttggtac cctgctggga tcaaccctgc ccaggcagcc tgttggaagg

149161 gacaagctgt ggcatgtatg cttaacacac acacacacac acacacacac
acatacacac
149221 acacatacac ttgcgtgcgc gcacgcagag cagctgctcc ttctgcagct
gctgctgtcg
149281 cccaacccag aactccccgt tccaaaggag ggtgagaagg gaggtgagga
ggggtcgatg
149341 gaatagaaga aaaataaggg gggggggcag agcgagagga agagaaatgc
tagaaatgag
149401 agaagaagaa gaagagagag ctgacaacct aaattggaaa gatataagga
agatgtgaga
149461 aatgggaaaa aggaaagtaa tgaaagtgag aagggaggaa aagaggcaaa
aagaggaggg
149521 atgtgggggg gggtccgaga cacttacact cagcaatcac acacaagatt
ctctcaccta
149581 ttaataggtg aaaaagaaaa atctccactg ccctcacctc cattaaaagt
ccacaggagg
149641 aggtgaaacc gaagcctttc catcagaaaa gcttctcctc agccgtctcg
agcaaaagtt
149701 agagacaaac accagtttgg aattaatacg caaatgtgaa catctacaga
tgattctggc
149761 catgaaattc ccacaggtta agcacagatt aaactaaatc ctggcctata
ttccattgac
149821 agagagtctc cgttgaagat aatacattac tctgcaacta agcttaatct
gggtctgaga
149881 aatcaacatg gaccaatcct ttcacttgag atgaggccgt ttgctccact
tttgaattaa
149941 atcgccacgt aagacatttg gatactgaat caacgagttc taatgacgat
ttagagtaga
150001 tttgatttga aggacggccg tgtcgggttg acagtgggtg ctactagatc
gtagaggagg
150061 gattggctgt ggggggaggg cggcgcaaaa aacgtgtcca attaacaaag
taattagtta
150121 attaacatag acgaccctga aggccttcct cagccttgtg tagacctcca
aaccaactgt
150181 cagatttgga agacttcaac aaaacccgcg cactgcagtt tcaccgccaa
cctcgaccat
150241 catcaatgat gcacacacac acacacacac acacacacac acacacacac
acacgcaccc
150301 tgtacttaga atcatctcca catatggttt tgcatccaat gtcagcttcc
tgcctcttcc
150361 agccacaaac ccgcagggac acagactgta gcgcgtcatc agatggtggc
gatgacgaac
150421 ccttgtcctc cagtgacatc agtgaccatg tgtgttgagt gagagcattt
aacagcgccg
150481 ataccctctg aactcgggtt tatagatgca tcagctttta cagtgacatc
acctgatgta
150541 gcttttacaa cacttaaccc ttataaagag ccaagtgatc agtggtttct
ccatttaaaa
150601 gggaaggagg ttagaaaagg agagggcgag agagtcagaa gaccgtctcc
ctctcatctc
150661 tccatagcca cgggcgggtg ctcagcacag gagaaatgtg cttacatcat
cacctatggt
150721 ccaggcatcc tgcagagggc agtgctaata gcagaagcac agagcagagg
aggcaacgac

150781 cgactcagga gaaactcatc aaggcgctcc cacacaacta ggatttctga
cattcttggc
150841 agcctttcag agaggtcggt cctaaaatat tcgagcagag ctaaccttga
ggaggcactg
150901 cgttccagcg agcagagacc agaggcttaa ttcatcatta atgaatgtct
gcatgggttg
150961 gagaggtgag gatcagcagc acccccgcca aactattatt tatgtcattc
tgttagatgt
151021 tttgctccgg taaacacttt gcttccttgc ataatttcat cctcacagag
caggacagat
151081 agaccgccag gctccacact gtgacccaga tgaggacaga agagactgca
catgtaacat
151141 ttgagttatg gacgtttatt ccgtgcaaca ggaatagaag tatccagaat
gcccacatcc
151201 atccttggct ccgcggaata ggccgaccga gggcaccgag tccaacaaaa
tcagcgtcac
151261 tttccttgat tatatgttca catgagggag acgttgtaat cactgtaacc
agcataaact
151321 ccattgatcc ctatctctca actgttccga tctagtctgc aggcatgcct
acaatggcac
151381 acagctgcct cagtaggggt atcaggcccg gcaacatttt tgtaggtgca
cgagcagata
151441 ggatcacata gcaaatgctt gagcaaagga tgagagctat gaatacactg
cgaccaggag
151501 cattcttctc acgacttcct ttgaccaccg ctgcaactga atctttttgt
ttgtgtaagt
151561 gcgtgtgcgc agggagggtg catgtgaggt ctccaaagca tgggtagagc
ccgttttctt
151621 tcggatactg attggaaatg agtgttgagc gctcagaaca attatgggat
cattagcacc
151681 gccaagccag aggcaaaata tgaacaccgg gccatttgtc atccgaccac
tgctgtctaa
151741 tnnnnnnnnn nnnnnnnnnn nnnnnnnnnn nnnnnnnnnn nnnnnnnnnn
nnnnnnnnnn
151801 nnnnnnnnnn nnnnnnnnnn nnnnnnnnnn nnnnnnnnnn nnnnnnnnnn
nnnnnnnnnn
151861 nnnnnnnnnn nnnnnnnnnn nnnnnnnnnn nnnnnnnnnn nnnnnnnnnn
nnnnnnnnnn
151921 nnnnnnnnnn nnnnnnnnnn nnnnnnnnnn nnnnnnnnnn nnnnnnnnnn
nnnnnnnnnn
151981 nnnnnnnnnn nnnnnnnnnn nnnnnnnnnn nnnnnnnnnn nnnnnnnnnn
nnnnnnnnnn
152041 nnnnnnnnnn nnnnnnnnnn nnnnnnnnnn nnnnnnnnnn nnnnnnnnnn
nnnnnnnnnn
152101 nnnnnnnnnn nnnnnnnnnn nnnnnnnnnn nnnnnnnnnn nnnnnnnnnn
nnnnnnnnnn
152161 nnnnnnnnnn nnnnnnnnnn nnnnnnnnnn nnnnnnnnnn nnnnnnnnnn
nnnnnnnnnn
152221 nnnnnnnnnn nnnnnnnnnn nnnnatgtgc ctgtgcgttc gtgtccgagg
agggtgagga
152281 atgccctgcc cgaacgcaac tgtccaaaca catccccgtc agctgtctcg
cccctctcct
152341 gtcaagacga aataacttca catggattta tttatttatt tcgccacgcc
ccggaccatc

152401 atctagcatc ggctttgtcg tgcgtgtccc tccgctgccc taaaactaag aaagcactct
152461 gcaaactagt gcgggaacct tcaggaggat aacaagaagg aaggaaagaa aggctgccag
152521 aaaacacctc gcactcctct ttaatcattt cattttcctg ctgacaggaa gcacctgcaa
152581 tccatcttcc catccgtcgt tacgggtggc gtaaatgttt taggtagatt taattaattg
152641 atttggtgac tgatggctaa gagcatttat tacaggggca ctcgattcac atttgcaaaa
152701 acgtggaata acatttttag attctcatta tcgtccgagc ctttgacgct ccagggttgt
152761 gcatacctga agcagcaact aaaaaagtca aatcctgcaa ctcgagcatt tctaaattcc
152821 taaagtgcga atgttcccta aaaggttgtc attccatttt acagttcaag acctgcacag
152881 ttgttctgct ggtgtctgag gacctcgtgc tggaggaaag gccaggaagt gaagtggtgg
152941 ggcctctgac ttgtgtgctc gcgccggtcg gccgctgaga tacgactgct gtaagagcac
153001 gagggattag cagcgcaaac attgatcact gataagctgc taaaagtggc catgcatctt
153061 gtcgccgata ttcttcctcg cggcgcccaa ctgagcccgt ttcttgacgt aaaacaacac
153121 aaaagaagac acactgcaca aattaaatcc ttgtttgaca tagtcagctt aaatcccacc
153181 tctgactaaa acaagcnnnn nnnnnnnnnn nnnnnnnnnn nnnnnnnnnn nnnnnnnnnn
153241 nnnnnngatg ggaacaagta aaacattgat agtggggaga aagatgaaga ggacgaaggt
153301 gggggggggg gaagaaaaga actagagttg agataggtgg aagactaatg gtgcagggac
153361 agataaagag ttgggggggg tggggggggg ggcaaccaga ctgccggttc cctctgagga
153421 aaaggaactg gcaaccagtt cagagtagaa gcaaacatct tcatctcaaa gcctgtggag
153481 caatagatgc ccgggacaaa tcagcaagtt ttttattttt attttttatc ctgcataaaa
153541 gacaaatacc agcttcaaaa actctggtct cccccagagc acatgagcag gggctggttc
153601 ctctgggttt accatatcgg tctgtggatc gatcacagaa gaagaaggaa aaaaaaactgc
153661 atttcaaagg gttgggggat ccaccgggtt ttcctttatt taaaaaaaaa aaaaaaaaaa
153721 acttgaacaa aacagactag aactcagcaa ccctgcagac acttgtggct ctgggggtcc
153781 caggacgctc aagtgttctc agcacccata agagtgcggc caaaccatat cagcacgatt
153841 gtgagcgccg gaatttaaga caaaagaact tcaaagtaaa cagtaaagga aaataaagga
153901 ttcaggcaaa cagagcgcgt ttggcttcag ctcggctgca gcacaggaga cgacggtaga
153961 gaaactttca aactacacaa gatcgccttt gcaacatgtg ttttggctat cagtgccatt

154021 aaaaagccat tacacagtgc agcaacagag cttgtgagtc ttcctcagac ttctgggatc
154081 atctatcatg caaatttacc atgaatgatg catttagagg cgtgcagatt cacacagttt
154141 gacatttaat atttgcgcca caattcatca ttttcataat cccgcaaata aaaagcgacg
154201 gtctaacaag tggggatcga gaaaaatgac ttttgttttc cggaccaccg gagctctgct
154261 gtttagaatg aaataaataa cagaacgggg tcatccgttt cgtgtttctc ccggagattg
154321 ttattgtcat tccaaatgct gctgcattct taacagtgga ggctaatggt gtcttgacct
154381 ggaggagagg aatgccaact ctctccgcag ctgcactctc aggccaagag ctggagtgga
154441 cgcaggggtc agaggtcacc catgatgtga tttgatcccg tgcaatcaat ggacgcaggg
154501 tcctggcctt tattagcgcg ctcctaatgg agccgagcaa ccgaacaatt gggaaagaga
154561 gcagacaaga gagaaggtga gcataaaaac cccttggcat gtttaagtga catttttcat
154621 ttgaggaata ttaaatacag accacgaccc aacaatttct ggatgcaaat ggaattttcc
154681 tcctgattta gcccaacgaa aatggttaaa taaccattta tcagagcaaa taaaatcctt
154741 tctttttta ctttaaccga acaacctaat tgcttatgaa cccataaatc tgagtttcca
154801 aactaagatt caggcgcgca aatggtcata aatgtggagt cacctccaca atgagcaaca
154861 gacacagcag agagagactc tgcgctggtc catttcacac ttcataaagc agcgacatat
154921 ttattccata cattcatact cacatgttcc tccagccagg taatggctgc acaactccca
154981 gtgattcatt tgctctgtca ttttctccac ttagttcggg actttaagtg caagtttcag
155041 aattgcgtat tgtgccccag ttaatttatc tgttggtttt ttttgttttt tttttcaaat
155101 ctcgctgcag ggaagctgtg tgctgtaaaa gagcctctct ttaaaaaccc aaatgcacag
155161 gagctaaaaa gaggtttttt taaaaaaaaa agctgcaaag acacatgtta aaaatcccac
155221 ctctgccgga gcaatttggt aaaaccgggt gaaacgccat aaaagaaaag aagcgtgtgt
155281 acagctttct ttccttccaa attagtcaac aatcatttta tatgcacata taggagttta
155341 tgacagaggc acacagagtt aatcaggaat aaaaattagt tcttttgagg cccacgcggc
155401 gctggggtca agagggtaag cctcaggtaa atgcgaggca gtgggagtgc acaggcctgc
155461 cctcttattc ctgtctgatt taaaagaatc catagggggc ctgtggtaaa cacagcaggg
155521 acatgaacct tctggagcag gagaatgagg ggctggtgtt gtacttaggc ttcatataat
155581 gaccaacaaa gcccccacct ccttcagtcc cccgcatacc aactcccact ccaccattcc

155641 tcctcctatt ctatacgttt ggtcactaag aacctccatt cagccgccgt
ataccaggaa
155701 cttttctttc ccctctggtc tctcccttct ccagttgacc gctccctcgc
tcggtgagag
155761 gcaggcccct ttcaggcggc tggccgggat cctgctgttt tatgggaagt
attgtgatcc
155821 cgcaagctct ctcaggggga tcagcactta cgagggccga tggagacggt
gatttgatct
155881 tctcttcttt cacaggcttt cctcgagttc tcatcaaact cgtgggccct
ctcgccccgg
155941 gaagcctcgg cacaaatcga attccacgcg tcggtagaac agccagcatc
tcggagacga
156001 gggcggacct ccgctgcaac acaaccgcac agagccggtt gctgttcttc
gtcctgacca
156061 cgtcgaacag gaagtcgcgc ttattgaaga agtagaagac aaatcagttt
atatcagtgg
156121 taataaatgt tccgcatcat cacaattcca cattcagcaa tcacggacag
aggtgaatca
156181 ttcctagaat tatttattta tttatttatt tgcccaacac taatcgggtt
agcaataact
156241 gtaatatctc caatgccagt acagacgtcc taattggatc atgggttcac
aagcttgtac
156301 gtgtgactcg cctgggtgcc aattaatgct gaccagctgt tccaggctgt
tgctgacgag
156361 ctgagtcacc aggatccagc tctgcaacca tgcgaacatt tagggtggct
ggggaatttc
156421 ttttcacatt ggaggacttt tccaataaag gacgttttac aaacgcctat
gaaaaaagag
156481 ttgatttgcc tcattttgag ttgattttga gacatttgac tggagttcac
tgaggaattc
156541 cccattagga acgactgtga caacactttc ctctctttcg agaactccgt
caacatcctt
156601 cacagatccg gcccggcact ggcgcggagt gaggcatcgc agacctgttg
tgccgtgggc
156661 tgatctccgt gtggatgaca gcagaatgca acagttattg taaactgaga
ggagggaggc
156721 ttttgcgggg gaaatcccat cggcatggcc agcatttact ggtgaaccaa
aacacaaagc
156781 caataatctc cgtgtcatta aggctgcttt tccaccactc aagctaatgt
gatgacacag
156841 ataaaaccat gttggtgtgc gtgtaggtgc gataaaagac atcgcttaca
tggacccacg
156901 ctgcattttc tgtattaatg tttcagttta aaaacatgga ttttaatagc
cccgcttcaa
156961 atctgaagaa tattcgagga ctctgaggtt ttgtccttgc tatattttct
ttttatggat
157021 gctttacagc ctcgttgaat catcgcgtaa gtgtaacggc gtgcttcaaa
gtgcctcccg
157081 tcagaggaaa aaagagctct tagacaacgc ttttcaaaaa atacagcttt
tagtgcagcg
157141 aacggcggtc tggtcctcgg caggggcgcc gaatgggtca cgcattcatt
ttggaaacaa
157201 gcggaaagtc gttgttcttg gcggggagaa aagctccagc gtaattaacg
tgttttcaga

157261 taaccccgc aattttccca gatactgaga cttccgctcc acctcacaca cacttgcacg
157321 attgtctccg gtcctttgag ggcctcgtta gacatgtttg ttttgctttt gacaggttgc
157381 cgtcgcacag aatcagagac gacctcctcc aaaggccaaa tatcctccca gttgttcagt
157441 ctcagttgtt aatggcttcc tccactttct gatggctgca tttatggcgc atgtttgcac
157501 aaacgcagta aaatgagtcc acacatctgc gtgaaaacta tgcccccccc cccgtgagtc
157561 ttggttttgc tgtacatcag aggctatcgt aataatgctg aaatgagcta cagctctccc
157621 atggtgcaac agttgcttta tgtttcataa acgtcatttg gaagtaattc agtaagcgct
157681 cccgtgaaaa agaaatgaaa tacagattgc cattgggtat attaacacct gtcacatcgg
157741 gccaacatca cgtttacccg actttctctt attttcttcg ccctcccaat ctgccggtgt
157801 aattacttca tcgaataaat caacaaagac ctcaaagcgg agcgcgagga agccaggaaa
157861 cacggagtag aaaggagaaa ggcatcaggc cctgacgcat gggagatgtg tgacgttcgg
157921 agatcagggg agcgaagcct tgagggcaca caatcaatat cacattttcc atcgccacct
157981 tcatcgctat tgtaaatggc ttgtgtcaaa aataataaca ggttgtatca ggaaccctgg
158041 gtttgcaatt tcgcccctct cgctcggagg ccctcgtctc cttctccctc tctcggcctc
158101 ctttactccg gccccaccgg gtgttaatga cgccacggtg gcagacactt ctcacaccag
158161 tgccgacttt ctccaccccc ccaaccattc tttatctcct tacccttgac agtactgaga
158221 actggaggga aactgtgtat gtatgaaagc gctcagcgct agagatgatt agccctctga
158281 atgtttcgcc actcagcccc taacgcagta ttattagcag cgctggagag atcgaatctg
158341 ctctcacttg tgttgtcata gcactctggg cagcaataag caagatgagt tgctgcacac
158401 tagagcaaca gagtcctctt catgggaatc agagctattc taagttcttc tgtggggaga
158461 agaggtgggg gtggggggtg ggggtcgggg ggcacttctc tgaattttgt ctttgtttga
158521 gctgattggc ctctctcatg cagtcagcct ccagtcttga aagtccatgc agaatatctc
158581 ttttaccttc attttctgtg ttcctgtcaa cctttttttc ttgcctgctg ctgtcagaga
158641 ccaatggaag cagtaggagg gaaccaaaat agtaacagtt cagaggaaat cagctctgca
158701 gagttgacgg gaaccagatg tggtatgagg aggagatagc aatggcacat tagctttgtt
158761 aggtagtcga gaggaaaaaa ctgaagaact caaaatgttt tcactttcta actaaagaca
158821 tgcaaacagt gacaacaagg tgtctttcaa atgacatgta attagcttag atgctaaaat

158881 ccaccctatt ttaaggatca cagaagaaaa atattttttt ttgcagaatt
ttgtttgtat
159941 gttacaagaa atgagtgcgt ttattttcat gttcctatta ctgcaaccca
aaatacagaa
159001 gaaaaaggag tcagagaggc cagacgtcgc attctgcagc attctcagct
gacaaaaaga
159061 ggaagtggca ttgatttcct ccttactagc tccagtcaca ctaaccagat
aactgtcctg
159121 cagtgtagag aatggagaat atgactatat ctggaatttc tttatttcat
tagagacatg
159181 agccagaatc ctgtaccttc tgacgttact ctatggacta gcgcacaaaa
aaggaactgt
159241 ttttataaaa acctagttct gctggagggt ggaggtgtag gccactgtca
atgcctaagc
159301 cggtcactat aaacagcacc aaagatcaac aacagcaagg tcacgtcaaa
caaaggctta
159361 aacggggccc attctctcca aatctgacaa gggagttgaa aaccgccagg
aaagatgttt
159421 gagaggtgac ataaagtctg aactggcttt gcagagcaaa gtgcagcatc
attaacataa
159481 caaaacagct ccgacagaac cgttaaaacc cacctgatgc tgattcagtt
gagaagtgat
159541 tcttctaaac tttagaatct aaaggacaga gagagctgct ctgagtgatg
taggattcat
159601 atctggaaac agacatgatt cagagcagga acaaagtgat ccgcggtgac
tcacagcacg
159661 tcaaacctcg ccaaaggcct ttacacccga aaagtgttga ttttctagag
cacagacgct
159721 aataagcttc ctattccctt ccaaaacagt gtgttgtcac actcccagac
acgtcccccg
159781 tgaatctgcg gtgtccatct cctgctatgt gcaaaatgag tttcggcaca
gccttattga
159841 agagtgcaaa cagcaaaagt aatacaggct gacaggacga tgctgaggcc
aatcgttggg
159901 tggacgaggg ggctctgcag ccctggcttt tgtaccggtg ctcctttacc
ccaggctcat
159961 ggagaggctc tttatgctaa cgtctgtggt gtttttaaac tgttctgcct
tatctgaggg
160021 gaatcggcag gaagtcagtg aatccctgct tatcttctaa gtgtgtgcat
ttgtgtgtgt
160081 gtatgtgtgt ttgtgaaaag gaggaggagg gacccgagtc gaatgctgca
agatccaccg
160141 gagcaagtca ggacaagagc agcgggggag gcgcccggca agactgacag
acgtgacgct
160201 aacatttgac gcgtactcat agcgagcaca taaacaacaa cggccaggaa
cactctgaca
160261 caaacactca gggcataggt gacatttaat ccatccgtca catcccggcc
gaaaggcaga
160321 cacgaatccc tcgcacacgg gtgcgtttgt gaaataaata taagttgatg
aatgacatta
160381 ttgagtctgg gggccgacgt cgcgggctga gattgtttga aacgggtcgc
atttgtgtcg
160441 caggtgacat tgcgtggaac actgacgcac tgcaaacaca caccaaagac
atgcgcagca

160501 tccagttgtg caacaccaga gatagctccg aagggcatct taaggaggtg ataaaaaggt
160561 tcctcccccc cccccatttg aaagcccgct ccctgatttg tgagccataa ccacggagag
160621 tgttgtctga acgcaggagg gtgggtggtc cgccgtcggg ttcatgcacc tttctctttg
160681 cagacaggcc acatttacat ggttttaacg gccactatgt cggctggctg ctgtaaatca
160741 ggggaggggg ttttaacctg taggagccat taggggttaa tgccgagggc aacggccact
160801 gctgctctgg cacaggaccc aacatatgga cgtccttgaa agcaccatcc tttagagggc
160861 attggagtca cttttggcta acgctgacct ccaggcccaa ttaaaaccac gattagcttc
160921 atctaaatgt ttcatcggaa tttgcaattt gtatttaagg ttggttccat gtatttcaca
160981 aacacacagt gtccagaaat aaacattaaa aaaaatcagg atatcgtaag ctgctgtatt
161041 tactgaacgg aatgaagcaa ctctttgcgt taccttttct ttatattcat gagcagcgca
161101 gctccctgtg gcggtgctcg ttaccatcat aagggacgca cgcacacgaa attcccagga
161161 aaaatgagca attagtggaa gtgtaacaca cctggcaaag gctccgcgtc tctccgaaag
161221 gccatcagca cgccgggaga ataggcttcc ttgccgttta gatccgagct aattggacac
161281 atttgattgg ctaatgagca tctaaacggg attccgacca tagcagaagc agcatgtgat
161341 ggcgggaggc gtggtcgtgg tcgcgctgta ctttttggg acctgagata taaatttatt
161401 ctcctgaaac tgtccagctt gctaggaaag tgcagaatcc ggccgatagc attagaaagt
161461 ccggcagcac gcatgactct tcctgactcc actccagaga aactgggatc acaattttcc
161521 aagtggcaat ttttgacctg aatatcattc atcaccactt cctcgatgct cacgggccaa
161581 ttccagaacc tgcttccgac aggagaaaca tgggcgtgac cttacccccc cagcattaac
161641 acgcagggga atgacaccac accgtagaca tcatcacacg aatgtaccct aattcacaat
161701 aaaggaggga cggtggtcgt tggacaggac gccgcttgcc gccagggcga gcgctcagtg
161761 tttaatcaat gggagaaact gtctatctat tcaagtattg atgtatccta tgacaactgc
161821 ttatccatgt tgagcaggca gagaggcact ggaggccatt caatcatcca ccagctgaag
161881 tcagtgttga gaggtggggg gtgggggggt gggggggtac ttcatgaatc tgaagtactg
161941 cattcagttt gcatattccg attattccac tgccacgatg ggagcttaag cgcaggggtc
162001 aatcaccatg caaacaggct ctcccacaca gcaaagcccc cgcccttcct gattcgctgc
162061 ctcggacaga caacaaccac taacaacagt ataattgcgt tggtcggaat gacacccagt

162121 tctggcgccc ggcaccagag agggcgagaa ggtgttcttt caaacctttg ttgcatatat
162181 cacctaagga gataccgtgg agatgtatgt ctacgtagcc atgtagcgcc agcccagtcg
162241 accttattag cattgtgttt gcaatagact gtcaaaccaa tcaatctgta gtgaggaaag
162301 ttgtgttgcg tctccgaggc acacagcgtg caccatacat gtagctattc agtcccgggt
162361 gatcaaataa tgaagctggc tgtgacccca gcgggtcaga acagcagaga ggtgatgcca
162421 cagacaccct tccacctgtt gttgttcatt acaggcagga gctacattac tttggaatgc
162481 tagatgagga ttctagctct aaatatgcag caaaactgga tgttctctac ctacaggtcc
162541 actatttatt ttaagctgaa ggtcaaatac agatatagat ttattgaaga ctgaagtcag
162601 attaattatg cacccatccc aactggttcc cttagctttt gatgactaac gtggcttttt
162661 catctcgccg acaccgtgga cctatgacag atctgtgagt cagtggaacg cacgaacata
162721 ataaaggcgc tcttcttctc actcagcgtc tctcatatta atattttcat cctgtaataa
162781 ctaaaagcca tttgactgct cattaatcag tatctgaagc tccccagtac acacagaaat
162841 atgatcccac aattccacag gaagagccgt ggataataac cctgcagctg acaggctaac
162901 ggaacatgac gtgcatgttg tccattggga aaacctacct gggcatgacg gaaacaggcc
162961 aaagggatat gcgaacctcg ggcccagcac tgagaggcgt agggctccgc ccacgccctg
163021 gtgacaaatc aatacttaaa accaaagcag gacaatgtgc agaatcagcg tctgaaccgg
163081 tgacagaatt gatttgtggg aatttaaatg gagtccaata ttctgatact tcatcaagtc
163141 catatcaagg agcggcctcg tgagttctgg tgtttattaa aacaacaaga ggtaaacagg
163201 tggggtcagc ataaaataac atcacctgaa actgtacaga ctctgattta tgcgtctggt
163261 catttatgtc agaagctgca tgcctgcctg tgtgtggtgt gtgtgtgtgt gtgtgggtgt
163321 gtgtttagct tttttgcgtt taaatcaatg gtcatcccct gatacaagat ggcctcataa
163381 atcattgttt gttgatcact tgtcaggaga caggggcctt acgtcgttct cctaattatt
163441 ccagctcgca gccccggctg gatgaatccc gacaggaatg ccggagcaga tcaaggtggt
163501 ggacttcaga aacgccaccg cggctccgtt tcagcctgga cggacaaggt gggcatctgg
163561 aaaggagcct cggggggtag gaaacgctcg tctgggagtg cggtagcgtc gggcaggttg
163621 gactgttagg tgcactgaag tgagaaatct agtgtatgag atatcagtca cgcaattgca
163681 acgtgctagg ggacatgaca ggagccggcc cctcgcctga ttgaaaaggg agcacaaaaa

163741 aaacaactaa atctgttttt cagcctctct cgtgtcgcat cagtctctaa acaagatttg
163801 atcatgtctt tgtgccactc aataccagcc cctcagtggt gctgcgctca cacaacaaga
163861 ccggacacac actcttgtcg catcagtagt ccccgaccgc gacacctggc agaggcggtt
163921 agcgttcgct ttctgtcgtc tgctttagcg gagcgaccag gacgcaccca aatgaggaga
163981 agtgtgacaa aagaaaatcg ggccagcact ggagccaaag tggtcggggg gggggggcag
164041 tattggcttt cagtgccatt aacttttcag caggtttctg tgtccactta attactttaa
164101 atgtgcagca attacccagc cgaggctata gggcttaaat gatgagaggg acgagagttt
164161 atgggattat tggcgagatt tttatgagag gggaactcac agtaatgctg gaattaagat
164221 tatacattcc aatggaatct cacaccagcc atctgatcct ggtgtaatta ctggatcagc
164281 ctgttctgat gaagagctgt atcatattga ttcgttttac attgtcgttg acatatagaa
164341 gaggcctcgt ttggtgttgt tggctgagcc aaagtgcgtt ggctggaggt ctaaatatga
164401 tcttagcgtg tacaaatcat gctgagcata tcggggaggc cacgcaggct ttttgtcttt
164461 gatatatatg tttgcagcat aatgagagct tcttgctttg atcaccaccg tgtcctccgc
164521 acgctttatt tgaccgtact ggagcatgtc tgtcgcatgc gtgtgcagtg gaggcctctc
164581 cagtgggtcg cctgggctta cggattaata tatagcacac aggagttgta tttgagatac
164641 ttatccaagg cctttaaagg caaggtggct tggggaggtg actgaaattg atcaaagcat
164701 caaaaattta gagcatagga aataccgctt ggggaaaaag gttgcgcagg tgagctaaca
164761 ggccgcttta ttgtgcttca gccaatacgt tgaagcagtg gcatcaatac ctgaagcgta
164821 aggggtgacc cctattgtga ggagtgagaa ctggtgccag ccgcttacag cctgacagta
164881 aatatcccaa ctaggcagca aaaagcgtcc gtgtttcatc acccatgggc accaatcaat
164941 atttagcaaa tcactccttt aaacacctac agagattatg gggaattcgc tggatcaatg
165001 gtgatcacaa ctttcaaagc agtgcttcaa aaagttgatt gttttcaata aaatataggg
165061 ctgcggtgtt tctgacgcac cagaggcaat aactcagccc gtgcaggctt tccagattag
165121 tcatccatga tttcatcagg tcctatatga aatgtcttga ggagaaaaat gtaggtcatt
165181 cagaacagca tcaaacggcg gcggagggac ccacacttgt ctgctgcatc tgtgagggcg
165241 ctctcgaccg cccgcgggta cttgtaacga cccggggaca gcatttctcc gccgccagct
165301 gaggtgcttc acaaaatgaa taactctgac cagccggggg gggggacaga gggaagctag

165361 ctgctgcgtg tgtgtctgtg tgtcacaagg agtgaagcag catgtcacct
ctctttagat
165421 gctgcagctc tggtacccgc tgattccaaa acaatccccc tccccccaac
ttgtctgcca
165481 ccatgtccgg atcaagtgtt cctcaggtat gagccttaaa gaggtgaaac
ttaccaatgt
165541 gacttcacca gccgtgacat cactttagtg ttagcgctac gtcttacgtc
tcttagaaca
165601 tcatcaagac catgtggcct aatggataag gcgtctgact tcggatcaga
agattgaggg
165661 ttcgagtccc ttcatggttg aggtctgtat tatggccact ttaagggctg
ttttgatttc
165721 tttgatccca gatcagagca caatgtaatt agtaatcaat tgctttaaaa
tcaatacatt
165781 tagccctaat tttctcagac tttccaggtc ttgcgcattt ttgttcttct
gaatcaaatt
165841 tagatttctg actgtcttta ttttagcggt caaccctaca ggacgacacc
taatggttcc
165901 ctaatgatcg tctttgaggc gaggagcttt gcaggttgtt gctgtcttga
aattttggag
165961 cgagaagtaa ccatatttgg ataagagggc agaactaggg acgggatggg
ggggtaaccc
166021 acccacagac tggatcataa gacaggcatg tgaatgaaag agacacctct
cccctagtgt
166081 taaattattc atatctaaat aattacggga ataatttcag ttgggccagt
aaatcatatt
166141 ttaggatgat gtaatttagc tgttaacact acatagttgt gttttctgga
gccggccccc
166201 agcgaatcca ggtggtatcc gtggtggtat tgtacgtaaa gtcacttgtg
cgttggcttc
166261 acttctaaga ccgggagtcc atctttcata cacagtctat ggtttatgcc
cggcttattc
166321 cctgtttcac tctttcctca caatcacatt attcatccca ctgagaacga
tctgaaacct
166381 cagatatcca aatttctccc tttctgtgtc gcatgttttc catatttggc
cagataaatg
166441 taagattttc tgtgatgata tacagccaga tgatgaattg ttcatataca
tatgtatgaa
166501 atattcaaat gttttttttg ctggcctttt tttaaagggt ggctacgtcc
gcaggaggca
166561 aagcaaactg caagttttca gttcaatcag gtcctggcac gtgttctcgg
cgtctcctct
166621 tcgtacggta acagctgtat tcgtttttgc atccctctgg atggaatcac
tccagtctgt
166681 gtgcagttaa tggagtattt ggcagctctt gtcattttat ataagcaatc
ctcagggatc
166741 attacttcgt cggagcatcc tgtgtggcga tcggcggggc caagcagctc
tggtctgggt
166801 aaagatgtct tgctttacca gacaaacaag cttattctga aaggcgcctg
atttggaata
166861 aattattcac acaaatgcag gactcccgac aacttacacg acatgttggc
atcatttgct
166921 gcattaattt tttagcactg ctgtaatttg aagggtatat aaatgttttt
tcacaatcac

166981 actaacgctg acacattact tatatgtgtg tgtgtgtttg acatagaggg tcttgtgagg
167041 gagacagggg ggctcgccat caaaaacctt gcaggatagc aatcattaga agctcagccc
167101 ccccggtaag caagccatgc ttcctgtgta gggccccaaa ggattctgtg tgctaattat
167161 ttactgaagc agcaaagact atcaaatgag accctgtgtt gagtctaact gtgagggggt
167221 accctgacag tgtaacctgg gtaacatgca aatggaagga gaggcggggg agggaccgga
167281 gggagggagg gaaggagaaa ggaggggagg aagtgtgggt ttataatggt cagggtgcgt
167341 tggaggagca gaggctcact ggtgagtcag gaaaggtggg gccacggagg ggcacagcag
167401 cagagtatta gatttcagat gcatatgaat gaccaactgc tggctctgct caagctttgg
167461 ggggaaattg cacgggctcc ctttgagccg cagttttgaa tctagattac tgtcaagctt
167521 aatcccgggg tgactcggcg gggggtcatg aatgtctgtg gctgctatcg ttaaatcgaa
167581 gacctgcaga agggtgtagg ttactgctcc tcagctgagt ttttccccat tctgatctct
167641 tttttttttt tgattcagct acaaattcat gttaaacgtg tccagatctg ctccgctgaa
167701 ctgcatttgc atgcgcacac aggcatcctt gtacttctac ctttttaagg actttcacag
167761 gcgtaatgta ttccccagct cctcacccta atccctaacc attccaagta accccctaac
167821 ccgaacccta acataaactc aattccaacc tgaaccttaa agccacgtct tgaccctcaa
167881 gcgatccttt gaagtgacga ggaccagcca aaatggcccc acttccccaa catgtcctga
167941 ctacagtaga atgactattt tgatgctcgc tatgtagcaa atacaagaac gaacacaccc
168001 acacagccaa aatcctcaac tcaaatgaag ggggaggggg ggagctgtgg tgttaaactc
168061 gtgcatctgg gggcaaagag gtaaagaaaa ccaagcagac tttcctcaac agatgcacac
168121 acgctgtaaa acagcactga tcttttaaga tccactttat ttgattagag acagcacacg
168181 gaccacaaaa accccaaatg catgtaattt attagaaacg cacatattct ggctgtaatc
168241 tggattaagt tctgatttac cgtcatcacg ctgcatacat tagagcccat tacggccctg
168301 ttggactaat aaagtgggct tacggcctta gaaagttcga ggctgctgca gctgccgcaa
168361 agctcaaaga taaatatctg acagagtcac aattaaatcc aaaaaaaggt tatgtaaatc
168421 ctcatctggc cacaatcagg ccagccgaga gagccgtgtc attacaagcc gccgctaatt
168481 aaacccagct cgttaatgtg gcatttgcaa aaatcagaat agggtcttgg ttttttttgtt
168541 ttgttttgtt ttttcctttc catgtggacc gatacaaaga gtccgcctgc ataacactta

168601 ttgaactgga atctgcaaaa cgtcaccagg gacactcacg tttaattggt gcaacactca
168661 ggaccggctc tgaaaggaga agcaggtgcg ccgtggagct ccttcccctc tccacggcta
168721 attcacatga ctgatgcgcg acctccagca gctccctgca cacaaaaaaa aacaaaacag
168781 agtgaccctc tcccaatgaa atgtcaccac gtgtcaggac atattctggg aggagtgatt
168841 gaactgtttg tttcagtgct gcttagtcaa tagcaccgct agcttaaact gctcctgaca
168901 gcctgctgtg tcggctctcg gcgccgccgc tcgcctaaac tttattttcg gagcctcttt
168961 gcccttgata aataatgaaa acccacaaag gaggatgtga gagcaaaatt aggcagcggc
169021 agcacctgct gtactgcatc cccgccacat ccagaatgtt taaccaaaca gcaggtcctt
169081 tttcccctct ctcttctatg aggcgattgg gcctttgcca cgcgcccccc ccccctcctc
169141 gtaaatgggg gtggtaaata tcagcgcggc agatggggct ggtgctccct ctcggcctcc
169201 ctcctgcact cagggagggc agagcaaatg gatagccatc aacagcagca ggagtcgccc
169261 cgggccacgg ctggagtaga ggacaaagtg agcggactgt tgctcaggca ggcaatttta
169321 cagacaaatc ttcccccgac tgcggccgct cgctcttcga atcgacttaa aaggtcgcgg
169381 cgtccaagat ttggtgggga aggctactgc gtttagcttt gcttttgggt gataatgaat
169441 tatattttac gccgcaggct gttcggactg ttacgggaag gaaggggggg ttgaaaagag
169501 cgccatttcg gcagccgggg cagacggcgt gccgcgaaag ggcaggatgg aatggtaggg
169561 aagtgacgcc atcccggtaa caaatggatc aaatcacacc cgctcatcaa ttaccagaca
169621 gtaaacaggt ttaaaattag acgagtggtg ttgttattgc cgttgcggac ccccgggcac
169681 ggaggagcgt ggctcggtat tcccagagtg atattagaca gcctcccaca gggagcagag
169741 gagcacatgt ggagcagcga tgacagaacg cagaggctca ctgcgcagcg gcggcggcta
169801 aacagtgatg taatgcatct gagctcaccg ggcgagagtc agcagcacgg atggagggga
169861 aaaatgaaat catacgtgac cttgttatcg ccggattcat tataaaagag ctaaaagtga
169921 cccaggccgg gagaagcttt agagtcgagc ggcggctcgc atctgcataa cataaacggc
169981 catttgaagc aacccacgac ggaggtgatc caccctcagc cttcaggttc atggcgtggc
170041 tttcaactcc cacatcccga gttatatgaa tgatttaaac gctccggcga tcggaaatta
170101 cttcactttg tagcggcaaa atccctcccc ggcctcccgc tgtctccctg aaaggacggt
170161 tcccctgctc ggcgtctccc ccagcattgt ggatttccat ttatttattt aaggaggaaa

170221 tagaaggaaa tttcaacgga tcactaattt catttcaaca tgtagcggtg gcattagccc
170281 tgagcagata tttcagagca gcataaaatc aatgttgagc ttctactggg aatagccctg
170341 gaacccatta gcaactttga ccaataggtc aggcaaagta atagctagat aaatgtttta
170401 ttacagattt gcaaagcaac ctaatgggga ggccagtacc tgtgcagccg tgcatggcaa
170461 atattcatca ttagattctt ttgctgtaat gaacacgaat ctctcggcgg gcacgacctc
170521 tgtttcttca gaagcgatcc gttaggtggc ctcggcggcc gacgggcctc tgaaacaacg
170581 atagcatgat agctttggaa gcacgacttc aggccgggtc actggactca tattgggctg
170641 cgaccagtgc atttaaagcc cggtgatatt gtgcctgatc ccttatttac aggccgtttc
170701 cttaagatgc ctccaacctt catgtaaagc tgcatctgca tttgttttcc atcagcttta
170761 ccaacctctg gaattgctat tttaagccat ctttatatgc taacctcact gacctactgc
170821 ccctgtgtgg atttacagtg tcccccccac ctccccctgc cccgagcaaa catatgtgac
170881 atttggatcc tttatttttt cagcctttca ttttttttgc aatgctactt ttctgaatgg
170941 aagccagaca aacctagagc cccccccccac acacacacgc acagccagca gcagctcctg
171001 tttccagctc gtctccatgg acacgacgag ccgaccaata acatcgctcg gcacgggcag
171061 gcgggtgatt tgatttctta aagggacagc gacccctccc ccccagggga ggaggcagaa
171121 acggcacatt ttcaggagag taaagcaccg caaaacaccg cagatgcaaa tttttccttt
171181 ttattaaacg tgaattcaca tgttagttgg agcccatcca tgtcagagta tccgcctata
171241 tacaaaaata ggcacgactg cgtcatcaca tccttcaaca gagaacatct gtgtacattt
171301 attcacaaac tgaaaggaca gttgatggtg gtaggtaatc atttattaaa agatgagtca
171361 aagacagatt cacactcgga atgtgaagtt actactgccc agaacatcca aaacaaacca
171421 aaaagaagaa gaaagtctcg gcatataatt aggacgtatt ccaaaactgt tttatcaaac
171481 agacttcatt gtaagaccca cataaaaata aacaatatct gctttttttc cttaagtgca
171541 agaaataaaa aatacaggtt tatggttgtt ccttttctta aaaaaagaag gcagtcttcc
171601 gttttaaaat aaagtttcat ttcatgaaac aggagtatgg aatgtttgtg tttttgctcg
171661 cccagaccac cacttttaaa ttcagaatat gcccttttag caaaactcgt gtttccctaa
171721 ttttaccgcc tggatcttta aatcgcagct gttctggcat cagatactcg tgagtcggat
171781 taacattcaa ttcctggtag ttgtcatata ttatgcatca caccatgagg ccgttttgag

171841 ttgctttgca tcagttccag ctgatcagtt tattttgttc cgcccagacg ggcttcagca
171901 tcacaacatg aaatgatgtc gacagtgcac acgtctgttt tcccatcacc tgctacaact
171961 gttgatggtc tgtatcgtaa gaggatatat tgcacaaaaa aatgagaaaa gggggaaaa
172021 aacagtatgt gccataaaaa ataccaaaat ccagtttcac acctgcgatc gggacccatc
172081 cacatatcac gagtatgata tacataaact cctaacttta ctatattaca gtacatatac
172141 aatcttgaaa ctagctccag ctctgaggta tgtgttcccc atgatgccat tgtatcgtct
172201 acaaatactc gacaaatctg ctctacaact gtacaaaaaa aacctagttc gaagtcacaa
172261 ggcatttttg atgcaagttc ttaatttaac ttaatcctta aaaccaggac tcccttgcat
172321 gcgtaaaaca aacgataaag ggaagaagtg cctgaaactc actgaaagca cagtaagaac
172381 attccctttg aaactgtaca aaaatatgcc tgaaaatatc ttacttttct tttaaaaaga
172441 tgaggtctgt tatgtatcaa aatgtttgcc ctccacgctt gtcgtcagca gagggtttag
172501 gctgcgtaaa tttaattttt tacttttccc aaagaaaaaa aaacccagga ggaaaaacta
172561 aaaaaaaaaa aatcacttac aaaaagaaaa acatttgcgg catttagaaa ttacataaac
172621 cgttgtaaag taacgtcctg aacaggtttc agttcacaga tttgactcag tccacagtct
172681 tttttcagct ccctgcagtg tttttttgtc tgaagaggat tcgatgcata gaagttcacg
172741 tatttttccc ttaagaaggt ccgtattttt aaagatgaac tgtaaatgca gacctacacg
172801 gagagggggg aaaaatccat aaaaccctgt catgctgcca gaaaaaaaac cgtctgcaga
172861 cttctttttt tttttgctac cggcttcata agcatctcac ctcgccgcct ggccccgcac
172921 ctccctgcag gaaccttcag aaaacaaacc ctgcaggagg cgcagccgcc accggaccag
172981 gatgcggcta gaaccgactc tcgcctcctc tcttattact agagaacgtt gaatctgtgg
173041 ggttgttgtt tttgtctctc tctctctcgc tctctctctt ttttgcatgt cagtatgtga
173101 agaccaggtc ggaaaagttc gcctccagcc agtccccggc gatcatttca ctgagctctg
173161 gcgtgcaata gtctgggaat tcgaagtggg agcccaggct gccctcgctg aacgagtcca
173221 ggtccttatc cacaagagac agggagaggt ttcctgaatt cgggttgccc agttccgagc
173281 cctgggcgcc ggacgcgaaa ttcaaactaa aatcgaacag caaatcgtcc gcgtcctcgc
173341 cggaagagga gctgccgctg ctgctggagg aggaggaaga ggaggaggat gtggagaccg
173401 acctggagga tgctggtgag acggaggcgg cgtgcagtgt gctctgcttg gtgatgtttt

173461 tgatgttgta gaacagtctg ttgttgctcc gcacctcctc gtacatgctc gccccctcgg
173521 actcggcgga cgagctcaaa gttgggctcg ccggaacttt ggctacattg tacggcctca
173581 ggcgctcctc gtccccggcc ttaatcccca tccggtagtc ctcctcgtac tcgtcgtcgt
173641 cgtcctcgtc cgtcagctcg tttttcaccg tctttgcaac ttttaagctg ggaaaaacac
173701 agtcgtcccc gtagccgtga gaggacttgg agccgctctt agtctttatc tttgagcact
173761 tcttgctggg agtctttgta atctttccgc acttctccgg ggagggtgct gatgacttgg
173821 aactgtcgag ctttggtttt ttcttcggtc tgtatttgta gtcggggtag tcggccatgt
173881 gttttagccg gagccgctcg gcttcccgga taaacgggat cttttcgctg tccttcagca
173941 tcttccacct cttcccaagg cgcttggaaa tctccgcgtt gtgcatgtct ggcgactgct
174001 ccataatctt tctcctctcg atcttggacc agaccataaa cgcattcatg ggtctcttga
174061 tgtgaccggt ggctgttttg caccagtccg ggtttatcga cacggggctg catgccataa
174121 attcactctc ctccgagtcg gtagcctctc tggacatgct cccgtccgtc tcgctgttgt
174181 cggtgtgctg caccattgtc ccgttttcaa gttacttttc tttgcgcccg gacgctgcag
174241 tgtgtgtctg ctcccgctct cccccccaaa aaacccggat cagctcactg tgtttgcgcc
174301 ggtgcgtcct cgtccgctgc ttaaacgtgt ctcaggctct gcactctttt tccagagtta
174361 caaactgcct tcttaactac ttatcagctt tttcatgttc tctgcgtcac cgacggccgc
174421 ccaatcacgg cgcttccgct ccgccccgca gactccatac aaaactccca ttaacccttt
174481 tcgttcctcc tacccgagct ctcattggag tggaggtggg gatttgtggt acacggcgtc
174541 tggggcaaca cgccgggcga gacagacata ttaaacacac atttagcacc gtgcgctccg
174601 gagatgaaca ttacgcatca gagcgcacgc gagaataagg tttactgtct tatttgagag
174661 cagcagaata aaaggaatgt gaggagaggc gattctccag gcagtgtaca gatgtggctg
174721 gaggacaaga tgtaggctat ctgatcattt gctccccccc tcccgtaggt tggaaactga
174781 aggtggcgct tggacactgt tgcagacgcc tggatgcgcc ttcctgcctg ctgtaacggg
174841 atgcgtttaa acagctggta atggctttag aagcgggctc tgcggtgtca ttaactttgt
174901 aaaaacctta cagaatccgc ggatatcgct ggctgaaaac ggcgtgatca tatttccccg
174961 atttctgaaa cgtttttcag cctggatggg tttcagtgaa ggaaataggt cagtagcaca
175021 agctcccaaa cagatcctta gcagagattt gttgtgtgca ggcacctcaa acagaacggg

175081 accccgcccc ccccccaaaa aaagtgaata atgcctctaa gaagtacttc atgcacccaa
175141 aagcaagcct ttaatttatc tgaagcgtca atttcatttt ttaaaaaaag ttttgtatca
175201 aaacacgaca gccacatgct ctgatttcac atgcgtgtgg attagtcctc atcctgtaaa
175261 gtgtaaagca gcagggctca ttcacatggc aacatttgat tgcaggggct tcagctgagc
175321 cagagcagcc aggcatgggt ggaaggctcc ctgctgagtt tgctgtgtca agaggcactg
175381 tagcatcaag ctggcgtctc ctgtaccccc acccctcact ccctcccagc catgggtggc
175441 agccagcagt gctattggag gccggcgcga taaggcgtcc tttccccccc cctccctctc
175501 tctcttttgc ttcgtcccct agagggcaat cacttaccct tttggaaccc tccccccacc
175561 aagccatcgc atttttccca gctgggactc aagtgtctcc agcagtacag agggaaagca
175621 gatagtgagg aaggagagga gggaagggaa ggagggaaga agaggaagga cgcaagcagg
175681 gctgaggagg aagcagcaca gacggatgga gggactcctg ccttctccag cctcgtgtct
175741 gtgcctgttt gtgtgtctct ggtgcagaca gagataggac tgaaatgctt tatcatggcc
175801 ggcctgtgct gattaagcct tatcagatat atgtgtcaga gcgagagtct ggggaggctg
175861 gcacatgcaa atgcgctctc ccgttagcgt gtttactgtc ctctgctgga tccatcccgc
175921 caattgtcga tcagggtggg tgcatatggg ttcatttcac acggactgac agcatggaat
175981 catatgggtg tgcgactgca acggccctct catgcatcat gtgcgggggt ggggggggctt
176041 aagtagctct ccgccattgc gatttgcaca aaatagcact tatatcttaa tgaaagcaca
176101 atatcgatcc atcacttcat tcaagtcgcg gcgctacctg caatgcagcg ccttgctgct
176161 ccctgtccat ccatccagcc tttgcccccc cccccccgaa ctctgatggt ttcatgctgg
176221 atatgaatca cagatctcct ccaggacgag tgtgcggggt ggctgtcctg ctcattagca
176281 tggctcatta ttggctgtaa atgaggtgct ggtatcagcc catgctgcct atgactcatc
176341 tcagggaggg tctcatcgcc atggctgcct cacacagatg caccaccgac acccccaccc
176401 ccccaccccc aaatcaggcg agggtggaga cagtggagag agtggtggga tcctcagacg
176461 tgttgtgttt tcatttcatt ttcactccag agatggcaca caattaaaac agtgaaaatc
176521 cagttatcag catgcaattt ctaatacccc cggcctccta ataccccccca aaggcagatg
176581 ttttaatgct tatttgttgc ctttttttgtg catcagggga gagcacattt tattccaaag
176641 caatggagtg ttgaattaag tgatggtttt catgtaggaa tgctgaaaaa gggtttcagg

176701 aaaaaacaat tccctttaca ttgcttatct aaaagccccc caaagcaggt
gctgctccca
176761 caaagggcca gtgaagcatt agatcaatac taaccctggc tggaggctat
ctgatgctca
176821 aaatgcatgg atccatctcc tctgcatagc cttaagacag ctgtggtctc
ctggaagctg
176881 ctatctgatg gcttcacaat ggggtaaaaa aaagggggga ggggggtgac
gggggtaggg
176941 ggtgtgcgag ggtgctaagg cagaaggggc tgttttggaa gtgcataaac
atccaaatta
177001 acttgtcact tgacagtttg ttaggtgcca cgtggtttta gattacagag
gttattgcaa
177061 tatcctgtag cggagtcata cgatgctaat gtgttagcat agctggggtt
tatgtgctag
177121 tttacgaggt aaaaacaatg cttgtcttta ctacagccct ctcagttcaa
cctatagctg
177181 aaggcaaaac ccaatacgtg tttttaaagc tgaggattta ttgctgggac
acgggtgacc
177241 agctccttta gcccgttttg ccgccgtaac tcttgagacg gtactaaaat
cggccccgtc
177301 agctggcccg ggatttggtg ggctctcatt cacgacaggg cctcattctg
ccgccttccc
177361 aaatgtaaaa tgaggtttgg cgatgaggag gacttcccag cagcatggag
gccgtttctc
177421 tgcttgatgt ttggtggttg tggagccatt tcatgctcta ctgttgattt
gcattatcaa
177481 ctcaatgcaa atgtttctcc ggggtgtcag gctgtttgtt gacctccttg
atgtggcctc
177541 tgtctctcaa ggtctgatgc ttaacagata cttgagactg gagtaggctg
tcatgaatgt
177601 aaacagctga tgcacgagta ctgtgccgga atgtttgtca cacacacaga
tgcaaatacg
177661 ctcgtctgct gccaccctgc agacgcccac tcacacaagt cctcgggatg
aaagagccgg
177721 cttgcgtttg ttagatgcct cttttgtttt tagtcttttc ttttgggaca
atttcagaag
177781 aaagaatgaa aatgaatgca aattccagat gagatatctg tgattttttt
tttttaattg
177841 gagataaaca gaaggattcc agtgtaggaa ggcagccaaa aaggtacgaa
aataaaatatg
177901 gttggtgacg attggcagct gaatggaatc gaaatttaaa gttcaatata
ttgtcacctt
177961 aggtgaggtc atacttgacc tcttttgacc tttttcctca gttttccacg
agctacagat
178021 atttgcattt ttctcaaaag cctccacaat taattcattt aagaggtgtc
aacatgttta
178081 accttcattt ttcttacaaa cgccccctgg tggccaattc taatgtcaaa
aacatccaaa
178141 gatcaatcag aatatggtga gtatacaact acaagtacaa taacacaaac
atttttgtgg
178201 ccgtacaggt ttggaaatgt taaataaaat aatttccagt tttctgtatg
atctatttta
178261 tcataggatt gtattaatac atttaagaga ataatgtaca taaaaacttt
aaaagtggag

178321 acaattttaa gccaaacttt tagctgtggc acattttgat gcgagtctgt cagctaaaac
178381 tccattttcc taaaaggctc aacacttttc ttccactgct gctgatctaa ctcaattcaa
178441 cagcccggcc agtggacact tactcaccgc tccagcatgc attacaagcc tgagggaggc
178501 tgtaaacttt gtcaatgtga gacacgagga ccggcgtggg tgtaggagta agtcaatttg
178561 taactgaaca ctttgtcgcc gaggcaacgg cggcacccgg gaaggtgaga tatgagaatt
178621 aagcctttta ttaaccgacg gcttgttacg ctctcagtga aggtgtttga cgggtggaag
178681 agacgaagag atgaagaggc tcccgtcgct ctcctctcct ctctctcctg cagaccagac
178741 gctgccctac aaacgatgtg cggcggcctg caggagatga catcatcccg gagcagctcg
178801 ggctcacata gaaccgcgtg ttcttcgtat tgctgcgtgg ttcaggccat ttaaatcctt
178861 taattatcta ccaggagtgg cgcagccgtg tttactgtaa gagtccactg cagctcgttt
178921 gggtttgtac agtgtgtctt gagttaataa acttgtgccg aggaagagcc ccgcagcaac
178981 tgtaatggga gcgtgtttag tctggagacc ggtggactaa tgctgatgga ttgtatggct
179041 gcaggtcagg tctcgcgctc acacacactt gttggaaact gaagcaagag aaggaaaact
179101 ttcaaacgca gatggctgct ccggaaaatg cccacgccgt ctttccttta aacgctcgct
179161 acgatcgctc atcaataaag cgcagccgtg ttctgataaa gctgtttacc gcacggctat
179221 agctgggacg cctgccgctg aggccacgtg cccccccac ccgtcgaccc gcccagactt
179281 gccccgtagt gagataagtt agtgttgact gcagaccatt gcgtgaagac gggtgagagg
179341 cctccgccgc cgcttaaggg gccggcgtcc ctggaagcgt cgaccttctg agacctgatc
179401 ttgtcagaaa atagactcag ttcatggcct gacgccccga tcaggcagga acacgttcga
179461 ttccgaacag tcatacctgc cgcttccgaa gaaaatgtgt tagtcaccct gagatgaggc
179521 gatcccggag ccttcctgcc gtctcgtctt tgcaccgtca gtcagcgtgt gacaggtcgg
179581 agcagcctga caaattgagg atccgtgccc cgcgggctca actcagaaag gtcgagattc
179641 cagaattagc aggtaagata acccggaatt aaaggcaaac actctgagct gggtggctct
179701 tatggcatta ttaccactga tgtttcactt gatttcattt ctgcaaataa aacgcaagag
179761 tttattttgt ttatcttcga tttcaccatc cgatcattta atgtggattt aattaaatcg
179821 caaatctcat tagcttttcc tcgcaatgct atcatagctt ccttgtcaac tatccattgt
179881 ttacatttca ccccccagat gaataaagcg atttagtcac acgcaccagg aaaaaaagaa

179941 aaaacagcat ttcaaggaaa aagaagtcag cagcatcaat caagctttaa ccttgaaaac
180001 aaaaacaaga agaaatggac ccatgcggag acaaacacct gaagcaaatc ccagcattcc
180061 ctcctgtttg aacagtgacc tgataagata gccccaaagt tatcagcaca agatgaattc
180121 ccagaggaac ccagaggagg ggtttcatca gtgtagcggg tggaagaaaa tcaatctctc
180181 aaaccacaaa tagtgacaaa taaccccga aaagtgaagc aatattagct gtaacctctg
180241 gagcattgct ttttcttttt ttttttcag tcaaaaatga aacaaaaaag agtctcagac
180301 agagcgaaca ttctttaaat caatcatcac ggccgcgctc ccgtaaaaaa aaaaaggggg
180361 ggggggattt agagagagaa gcttttaaaa cgtcaaccgt ggacaaagtt gcttcagtat
180421 ctactgacta aaaccaaagt ttccctccaa tgtttctgag tctggtggca ccacagagaa
180481 gaggaaggct tccctttcc tctattccgt atgcacatat agatttgtat tttcctggta
180541 tcggaatctc actccgtcct ctccatacac tgaatcgctg ccctttcctt catgtctttt
180601 aaggatttct tacacaggct tcaaggaaga ttttcataaa tgtcctcctt tgcaaatccg
180661 ccacagcccc cacggctcca gtatctgcgt ctcaacagta aaggtcacca aaggggactt
180721 atggatgtgg aaaggctaag ctgtgtgcac ggacgagcgt tagcaactat ctggaaatca
180781 ataatagaag gtctgataat gagcgtgtcg aatcacagcc gcccgctcag ccacaagcat
180841 ccgccagact ttggtctttc cggaaggcct gagtgaggaa aacaggagga agtttgcacg
180901 agtatttccg cgcacgcagg aacgtaatca gaccgaacac aaaaaaaact gcgagaagga
180961 ataataacca tccctctttt gctattccag attcatttgt gtctccttta aaccgcgttg
181021 ccgcatcagt agcggaggat taaggcgcga gagctgtaag acaattcggt aatgaaaagg
181081 aaagcagagt ttataaggaa gcacggaaag agaagacttt ggtccccgaa agtgtaagcg
181141 gattagctcc tcttattttc aacatttcct ctttgaaaca cccgtgcgct aatctcatta
181201 taagtgtctg tcaagcgtat ttgacaattc attttaacaa ggcgccgctt ccttctccct
181261 caactcgcgg gctcccgctc aaatggaagt acatgagagc cgcctgctga acacacaccg
181321 gctaacacgg gggaaaaaag tgcacgaagc tcggtacttt taatcatctc taacatgcac
181381 taactgacgt acgctaattt caacagtgtc taccttgaaa tgtgttcccc acaacaacgg
181441 cggcatttta acgaccttgc acacaacgct ggcccgcaca cgaatcaaga ccagagttag
181501 ctcgtcgctc tttaacacac tcaagtgtgt gtggagggaa tatttgccgt tgactgtaca

181561 aacatagata ctgtaaacta tcgcgtttta ctgcaaaaga caagaaaaag
gttcagtaga
181621 taatgaaaca tttagtttca aatatagcta tgagacgcta cagataatta
gccttttgtt
181681 aattgggacg aaccaatctc gcgttcttct gagttgttag aataaattgt
ttgcctacag
181741 aacatcatta tttcataact gctgaatgtg ttttgagtaa caacaacaat
ccgccccccc
181801 cccccccccc ttttcttttt ttttcctttt aacaagaaat gtgaaaaata
tcctcaggca
181861 ttatttttga actactttta tataacaagc tgactccatg aatattacat
cggctccccc
181921 gttcgcagcc tgtaatgcac cacctcttat catggatagt gatatatgta
actttcaggc
181981 ggctgggatg ccctgctagc taaacgtgga cttagatcat gacccctcgc
gatctcgatc
182041 tgtctgtcac agcagtaatc tgaccattgt gggtaaatac tgcgaaaggt
cttcaagaac
182101 gatgaagacc ggtaccttaa tcacggctaa tccccagttg ccagacatgt
cttggaggga
182161 catgggatgg acacatcacc ccaattagta gcagctaaag ctttcaaacc
ttgtcattag
182221 gagtccataa ccttcctgtc actcagcgga gctgctgcag cctcttattc
ctgcagtttg
182281 tcacattttc ctgcatataa aaatatctgc cgagcgtttc gctcggtggc
agcaaagtgt
182341 ggaaaacggc aaattaggca tctgccgtgt tgctacaggc gtgaaacttt
aataggctga
182401 tttatgtatg aaaattccta tgagcattag cacgccgagc agaggagtct
tatggcaggt
182461 ggagtgtact ccacccctaa tgtattcttc caggaaatga aacaggcatt
ccagatacct
182521 ttatttgtgt taatagatga taggggcaca cacatgcatt tgaatgtact
gagctatcag
182581 ctcagcatgc agatagcatc cccggtgttt tagtgccaga gaggccgcac
ctgccacggc
182641 agacagtaga gcattagcgg ttatcgcggc ataaaaaaga gcgctggtat
taagtctgca
182701 acaataagag cagggtcaag gatgaagttg aatgacagct ccgaatgaat
acaatttccc
182761 aggacgagat aaagaggggt cttgtactcg ggctaaaaat cccaataaaa
catatatttc
182821 tttactcttc agctctctgt tccgacatgt tttcattctt tgtgccattc
tctagcgagg
182881 gggtcaataa ttaaaggagg acaatggcca cgccgggtga gattgatgga
gaggatgggc
182941 agaacctccg ccgaatcaaa gctattcacc gctaatgaaa ataagagaga
gcccgcactg
183001 taaatcatct tatggcctaa tgtattagcg ataatgaatt cacgcagcca
catttaacct
183061 tagttttgca attcattata tttgagatta acaagggagg gaacttaagt
gaactgtctt
183121 cagggaatta cagcgagggc ctcattgtca atcacggatt gtgttccgat
gtctttcatt

183181 aggcctggtg gaattaaata agactttctt ttaatggcgg atggctctcc catttaacgc
183241 tcctcttgct ctcccaagat ggttaactct tcattaccca aattaaaagc atccatctcg
183301 cccggctcct gccacattgc catgacctct acctgtcaaa actttcatgt aagctcttga
183361 gtgatggatg ataccccccgg accccattga caaaattatc cagcgactgt ccaaccctct
183421 ctgatccatt caatccatct ccccaggcta catcccacta gctatgatac aacaatgaag
183481 ccgagtcgct aatttactct catagaaatt agaaactacc ttttaattat atgcctggga
183541 gtgttctgag ggttcatggg gatcaccccc gaagctttta actcaacttt ctgggggttg
183601 atcgttcata cgcttcaaac gtgttatttc aggggtcgtt caacacacct agggccagag
183661 ggaatttcag gagcttcggg gaattggaag gaaagaaagt attgaaatca cttttgaagg
183721 tcagggtcat tcctgaagga cataattgga tagaagcaca cttaagagac ccatccaaaa
183781 atccacactc caagttgtgg aaattaggat gtatcgcgat agctgaatct gaacaccatg
183841 tcgtacatgt gggcacctaa tcagtggagc aaacacagat acgtgttcag atctgcagat
183901 gcatcacgcc acacttttta cagacggact ctgttgacaa aggaagttct ctgaagctcc
183961 ctctctttcc tcctctattt gcatcacctt cagacacatg taaatattca aacgcaggcc
184021 actcgtcata cataaaacat gatataagtg gtcccattct tgtctgacct cttgtccccc
184081 tgcttctatc ccagacagat gtagataaca ctccagacac acctcacttc ctgagaggag
184141 agttatggct tcctgggaga ggcactgccg tcccccggag ccccgaggag tcagacagct
184201 acctatggag agggcacgtc tctgacttgt agacctgctc tgaggcttct cctctcactt
184261 tgagatcctt cgtgtacctg tcagggggtc aaaagggtac gctcgctgca gacagccaaa
184321 cggcgctgtg tgtctgggag tgtatgtgtt tgcatgtctg catgctggtg tgtgtgtgtt
184381 tgtgtgtgac accccccccc ctcctccacg gtgacagatg ccaggctgtt tatgtgtgag
184441 gcgcaggagg gccgggtggg caggtgggac gcaagtggag gcaagtgtgc gggggtctgc
184501 ggtggctggg tttgagtagc gcggcgtgag agaccacact tgtcaaagtg tgcagacatg
184561 cgtgtgaacg catttgcata cacgggcata tgtgcgcgca cgtgcctgta cctcggcgag
184621 cttgacagcg cggagagtca aaacgaaaac ctgtgctctc cacttccccc caaaaatgta
184681 ccgtcccaga accacagcac gacagatggg gcagcggcgc cgctgctctg gcaggcgagc
184741 aaatgatgag ctaatggtag ctaaggaggc ggccgcggtc gcccacggct tattcgggat

184801 tatggctcaa tcacggaggt ttagggtgct taggtaatta tttaatcaaa gagtggacgt
184861 gtcaatagaa gtcgacgaca gattaattat gagtcaggga tgactttaat tcatcgtgtt
184921 ttactgctcc tctgccacga agtcggcagg ggggagagtt gatggtgtga gggtgagtga
184981 cggttccggt ggagtcgacg tggacggacc cactgctggt tcgttacgtt attgctcttc
185041 agatatatcg gttttttta gtttagttta ggggccctgc acagaagaaa tgtgtccaaa
185101 tgtatacagt aatattcccg agggcacccc cctctggctg gtggctcagc cccctctggc
185161 tggtggctca gccccctctg gctggtggct cagccccctc tggctggtgg ctcagactat
185221 aaaaaaactg tgaatgctgc acgtctgtat gtcggaatga ggtccagcct gcaccggttt
185281 atatgaggag atgggcggca catctccaac aagcgcgaca ccacaaagtt ggcgttagct
185341 tcgcacttgc tctcaagaac agtgacaaaa ataaaacatc tccaacagcg cagctggcca
185401 atcatggtgt gaagtaagtg tgaatgttcg gctcgtttgc ttggacacag acgccccccg
185461 atcttatgtc ggaaagaatg gggggtgggg ggggggtttc caacgctgtt gactgaagat
185521 tctcgcctct cgagccttct gtgacagctc tagaagaaga tgcaggcaaa ttacagcgtg
185581 gagatggtgc gtgagtcacc cggcccaccg tcagtaacct gtcattattt atagcagacc
185641 aaagaagatg aaaacgctgg tgaattctgc cttcgttacc actaacagct ttctactaat
185701 agccgtcatt tgctgcagca aggctcccgc gacccggttg cagcgatgat gtacaaaaca
185761 gacaagcaca tgacccgggg aagtggctca ttcacacttc gatcttttaa gtgtttaagc
185821 tttagtttga gtgaggaatc tgtctttgtt gggtttcgcc cgtctcggca gcctggatcc
185881 agaggctccg tgaggcttgg tgtttgctga ttctggggat tcccccgcat gtgctctatc
185941 cagtccttct gtttcatttg gctgatggag aacaggatgt gaagtttaca ccagcgcaga
186001 gaaacggggg caagttgaga cacagggatg gccgttcctg gtaaactccc tgatcatgtc
186061 acaggagaag agagataaat agcgaaggga aaacaagcga ggggaagctg gctatttttc
186121 taaaagctcc acggaagttg ctaattatat ccagtcggag acctgtaacc tcggcagggc
186181 cgcacactga catacatcca ccggcatgta cgctcacact cattgttgct gaggcctaac
186241 ctctgccggc cctgcggttc cacccgagca acaaccaaat tagaaggctg gagcgtaata
186301 gcatcccctg tctttgctgc tgttaaaggc cagtaatctg tcatttgctc tgacattaag
186361 gagaagacgg attttacaac aagatattca tgtcgatgat gtcgtggaga ccagcagagc

186421 acgtgggttt ccgaggggtt gattgagggc aagctgtcac gcctggtaat acggaggctg
186481 agtctcacag cccccagccc caggcgggca gatggtaggg tgggcagagc agggcgaggg
186541 tctcctgtct ctgattctca gctgcacttg caagatatgt ctgaaacacg ctggcagata
186601 tccatgtcct tcaactcctt ctgcacccct agcgtctcca cccgagccag ctgtgtctgc
186661 acgagctcgc caggctccga cagcagggat caaaacagca gacgaaccaa acgtgacacc
186721 gaccaccatc aaaacggcca cggcgaggct cccacagcta catcacagga ggcgcgtcgc
186781 gaggaggcgc cgggcgagct gcgagctgtc tctatcaaac gaagcgaagg ctagcacatt
186841 agtcaaccag tcgtgtcgca ctgtgatgtt gcttaacgca attagtcatt ggagacacgg
186901 cgggagctga acgtgaacca acacgatcat tgcgcagctt tgttttcaga ttaaagggga
186961 aagttcaaag ctctcaaact ttgttacctt tcctcttttg ttgtttgcac gcaagctcac
187021 acagacaaaa acctcccaaa gacgagggga tgagagcgag tgagcaagcg gaaggaaaaa
187081 agtgagaaga aaaacaagcc ggtgcgcgca acgtgcgtgt atattgatgg tttacgtcca
187141 tatgggccac aggcaggaaa tgtctacctt cctgtttgat aatattggac cagcagtagc
187201 tcaaagacag tcaggagatg ggaaaaggga tttgatttgt ctgcttgttt taactgcttc
187261 acaggtgttc tcaatgaagc aaccattcac ggacctgaga aaataaaata gggaggggga
187321 tgaaaggatc gttcggagga taaaagggag caaaatatca aagcagacag atggaggagc
187381 tcagtcgatt gataaatgcc tctggatctg ctcccagtag caaaaataag ggtttcattt
187441 tggtctcagt aaacaagatt tcagttgaaa atcccgaaac aacaacagta gttccacaaa
187501 tgctgtatct gtcggaaaga agaagaagtc cgtaatgtga actgatacag gcaggtcagt
187561 cagaataaag tgtggatatg atgggcgtca ccagggcgac tagcatctga tcccaactgc
187621 tacctttttt tctcctttca gataaaaatg tctcatcaga agctgtttta ggaaccaaaa
187681 acctttggaa atgagaatat gtgaacatgt gaaaatgtta aaaaactata aaaaacatac
187741 ggaaatctga tgaccagttt agccacgtga gttattctgg ggtttataaa gttgcagggg
187801 ggagtttggc ttccccccta caggaagcag ctggcaacag cgccgtcact gaggtcacat
187861 atgagaggtc agccagagaa tcctggtgtt catgcacaca cacgcccaat ggtcttccac
187921 acaaacaaac acacacacac acacacacat acacacacac actgaagtcc tgagtgacac
187981 gtcctggcag aggaaggtta catcatgtaa ataacggggg ggtagggtta gagccagact

188041 ttaatgacat cagaactgaa acctgcttcc gagtcttatg tccaacacac
acacacacac
188101 acacacacgc acgcgcgcac acacacacac acacacacac acacacctgc
cacttaccaa
188161 gtgttgaaac catatttatg ttaatactgg acttgattgt gacatgtgga
ctcaaagttc
188221 gtagaaccgg ctcgcatggc gagctgtttt tcagatatct ttctggctga
cggcgcagcc
188281 ttgctaacca tatgccatca aaaagtgatt attacgtttg attaatgcgt
agcacgcgca
188341 attaggggaa tataattgaa ggcagtggtc ttctttggca ccgctccctg
acggtggaca
188401 tgtgccgacg cgactggggg agttaaaagg gccgagcagc tgcggaggca
gatgagcctt
188461 ctgccgaagc aggaggggga aaaaagttat atgctgaaag tatatttcag
tttatggttt
188521 atctgaagcc cccagatgtt gtgaacatgt gggtgtttta tatggcagtt
aacagcaggt
188581 catgctcaga ccgccattcc ctcagaaaca attaataacc cataaacagt
cgaacggctt
188641 ctccgctgag cattaggttc acaacggctt ccatgcagag aaaaaaggct
gtgaaaatct
188701 gctctttttc tttaaggcgg gggtctttta actttctttc tttctttttt
tacagctccc
188761 cctcgctgca gaactgtagc tgtataaata atgtgattca tcccgccagc
tatgtgcgca
188821 cggcgctgag acagtaaacc ggtggcgctg ctgaaccgag tgacccggcc
tgttgttgtt
188881 tgcctccatc tgcacaacgc cggggcggtt tattaccggg gcttgttcga
acacggtaca
188941 agtaaatatg gctggttgtc tggtggcgtc tgatctgatt actgcgctgt
gaaagggtcc
189001 attgagcaca ggaatctggt ttttgctcct cttataaatg cgtaaatgga
tgattagatt
189061 gcagctttga aggcagatgg agaattcaca gccgtctaaa tggggaatat
gaacataaac
189121 ggctaatgtt tacgtccaat ccaatatctc catcttcacc tgcgcttaag
gttaaatgta
189181 tcagacctgt aactgcttgt caggtcagac agggacggcc tggcaacgtt
ctgaggtgaa
189241 taaacaaaag ctatttgtat aaataaagtt taggcggcgc tgatgtacag
ggtgggttaa
189301 gccatcaccg cctcgggagc cctgagattc atcatcgcat gtggctgata
cctcacccag
189361 gatgctctcg caggggtcag gggtcacgca gccatcagag gaagccatca
gtgagtagct
189421 gacccctcct ctgcacaccc cccacctttc atctgtctct ctgattagtt
tgcccatgtg
189481 acagatagca acttaaaatg aaacacccca cggacaccct ccagggagga
acgctcaagc
189541 atgatatata tatacacaca tttttgctgc tcggctagcg agcgcacttt
caattacaag
189601 gagaggccgt tagatgttga ggagtgacgg cgcagagctc tgccgggcct
gtcggctctg

189661 gagaatgaat ccatccacct ggcttcagct ggatttggat ccttcggctt
gtctctcatg
189721 tgtgatcggt gtgtatctag cctgcagctg cagatggtgc tccgtttgat
gtggacggat
189781 ccgatagcca ttcggtcaca ttatgtaacg cagtggacgt aacgcagctt
caaagtgaag
189841 ttggcacagt tcaagctgca tttctgtggg tttggatctg taaaaataag
tttgtgtcca
189901 gactagaagt cagccggctc caacataaat cactgtccgt cttaactccc
ccgctgactg
189961 tgaaatgtgg tcctgggagc gtttttacct gcttatacgt ctccatagat
ggatgaaatt
190021 acaatcggtg ccacagtgaa gtattgttgg tcgtctttct gtcagagaaa
acctgaaagt
190081 acagtgacgt aggttttgtt tacctggtta tcttaatata attataacgt
ttacgagtca
190141 taacaaccaa tttaaacgta tcgctccttc gccacatcag aggcgacgtt
ccaaacgtgc
190201 gttacagtgt tctcagggta actggtagcc gcgacaggct tgatttgggg
atttgccgtt
190261 gccgtagtga agtgtaggct gtgtgttgat gaagtcatca gggggtgatt
tggctgatat
190321 agaaggggac ctgtcgctcg tcagaaaacc atccgccgtg ctgattgagt
cgtgcgcgga
190381 gccactggga taatttgcca caagcgggcc ggctcaatgg actttgattg
gcgcgctgta
190441 tgcagtcgag ggttgtcact caacgctcga ctcgcctgtt cgcacttatg
aaagatttgg
190501 attccacttt gattttctca ctttgtcttt tatctttttc ctgtcaaaaa
aacccccaaaa
190561 aggatccgcc tcctctttgg tctctggctt tcctctcgct ccgctcctcc
ctgcgcttcc
190621 gtggcctctc cctggcctca gcctgggggc ccactcagct cgctgaaagg
taacaaaatc
190681 ttccggaatg aaatcgccgg ggctgattcg ctgattttcc tgtcaaagag
gcgaaggagt
190741 gtattttccc cgtcacaaaa caaaaggtgt ccattgcagt gatctgtgtt
tttttaaaaa
190801 aagagaagaa gaagaagaaa agaagaggcc ccgcacctga ccggcgtatt
ttaaagtgcc
190861 agtaagatga gaagacgagt gcacatgcag tcacgtatcc tgcaggagga
cagaaagctt
190921 ctctgaggca cctgttcata ttcctgttaa gtttgaaact ttgaaacatg
tggctcagcc
190981 ggggagctgt aactctgcca aattccccca gttcagctgt gcactggaac
tttttaacac
191041 cccaccagtg aaaaatcaca acggcggcgc tcctgcgcgg aggaaaactg
cgttcccctt
191101 cattaccttt attccggaaa gaaaaaggga gagagaaagg acgcgagtat
ctgccaggag
191161 gcgcttggca aaatacctgc agcaagtttc aaaacaccac cggatctcac
atctgactgt
191221 tttggcaccg ccgggtggag gcggaggcct cggcgctgac cataacgcag
gtatgcctaa

191281 tcctctttca tctgcaggca tcaaaaagga agtatctgtc caagtgagga gagattctgc
191341 agactttaaa ggctgctggg ggatgggcgt gtcgggacca accccgtcct gctccttatt
191401 attctttgca cagagctgta agtctaaaca agccgcttcc agagcagcac cgctgtgcac
191461 tcatgctggc ttcagtgttg ttctgctgtg gcccgtgtac gtgcgtgcat gcacgaggcc
191521 gctgcactgt tgagatgacc cccgctttct ttctataggg gctttcactc tgcaagagct
191581 caggccctgc acagaagcag cctgtgcttg taccttgccc cccccctct ttctctctgc
191641 aacagcttga gtgcttccag ctgccgacac tgaacaccgc ctcccacaca cacgctcgtc
191701 ggtacaaagc tgggccagca taggacgcgc atggacacat gcgcgcggca cctcccttat
191761 ttggtcgcga tggctgcgag agcgcctcgc ctgttagcga gcgccacgcg ttagcacgca
191821 ccctcccgga ccctcccgcg cctgtgcccc ttcagacagg atggatatgg gggatgattt
191881 attgtccgta cccataatag accctcccgc cgagaatgcg gccgacaggt aacggggcgc
191941 aggaggagcg ccggcgagaa acggcgtcga agccaaacaa agcaaacaaa aagaggaaac
192001 gacacggaga tagaacgtag agatccatat cagggaaagt gccataactt gtatgttaga
192061 aatgcacaag aaaaccagaa tccaggtgca cacacacaca cacacacaca gagtcacatg
192121 cacacacaca aagacaaaag gtcctccaga agcagcagtg tgtctctcag tcctttcatc
192181 atcttatgaa ataagaggag agcaccagag gaacctgttg ctgggctata agactgaagc
192241 agcccaggaa aaaaatggta atgctagtga tagcggcggc aggggtctgt cggagtgtaa
192301 ccatagctgc taaacttgca ttcaggccct ttccagggct gttctcgggg tatgcagacg
192361 acccaggggc tagagtctta gggaggactt taagcctctt tgtcaagctc ctcggagctc
192421 aggcgaccgc tgtgaggagg ccgaggcatg cagctgtctg gcaacaagag gctcctcatg
192481 gcgctgacac cggcctgcgg ttcagggatg tgtgttccag agcgcgtaca tatttcacat
192541 tgttcatctg aacatgtggg cccgacttga tcaaaaggag gctgttggta aaacgctgat
192601 aaaaggcgag gcagacgttg tgcagaacgc tgacgaacct gagctccgca ggaactttta
192661 cactaacttt ttgtctcaac acaccttcat tagaacaatt tatggcattt tcctgaatag
192721 cagtgttctt tgctcgcata tgttgttctg aatttggagt tggagctggt gtcaatgtta
192781 cgagggcagt gctacattca tatacggctg ttttcagcaa gaattccagc aaaaaatgga
192841 cctaaactca gtaatagcac cgagtaggat gtccaatttc accactttag tcatcttgtg

192901 ttacttttta cgaacataaa ggaatatttc tttgataatt gcctgacata
aaccaggctc
192961 caatgcagct gcgataaaat gacaaactgc acatcaacgg tgggggggata
ttgttttact
193021 gatgccgtcc ctcataacga cctgacaaaa gactctcacc acacttcacc
ttcatttatt
193081 agacacctca gactctttta aacaacgctg tcgtcagcgc gccgccgtct
cctgctgcag
193141 ccatgtgacg tggcctgagg acaacacggt aaacccgacc gctgtctccc
cacccgggta
193201 ttatcagtga aaccaacacc tcctttggaa tgcctcagaa tcgcctggag
acacggagga
193261 gcgggtttta tgaagcccgg ccccgcgccg ctccaaatga gccgtgctga
attctccacc
193321 gccagccggt cccgggggag gcggttcgag aaaaccctat tatatttggg
ggcaggcagc
193381 gctcaaactg acagcaactg ctgcgacagc aaagccggtt gagagagggt
gaggcatgtc
193441 tgccgtgtcg tcgtgacaac aggcggaatt tcagacgcgg gcacaaaaaa
aacctaaata
193501 caaaccagat tttgcccccca tcccttagtt cttctgcacc cgactctcct
tgttacataa
193561 ggctgcaact tgagatcatc ttgtgtcaca ggccactcca ggtggccacg
ccccccgcgg
193621 taattacaac gcacacttgc taaaaacggc gcacccgtgc gtgcgcgaag
aggaccgcaa
193681 acctgccatt gaatggcact ggaacggggc aattactgca gggaggcggt
caagagtctg
193741 gcctctgtca gaccaacagg gcaacgcacg ggaggacacc acgtcacgag
tggtggcttc
193801 aatccgaagg ataacgcgga aaaggtcaag tcggagtcgg cggatcacac
ataatcacct
193861 cccaaacgcc gccgccgtat ggcgaggtag cctcctgacg agagacgaga
gagagggaag
193921 gaaataaaga gtgaaataag aggagaagga ggcattatca ttgaaaacat
cccccccgca
193981 gcagatgtgc cgcttgtctt gggctgtgaa gaagtggcgt gtgaccccgt
tcacctccgg
194041 actaatgaaa gattgactga ctgactcacc tcgccttgct agcacagaca
taaactcagc
194101 caaatattat tggatgcagg gcctggaaat gatctgggag cttaaggaag
aaggggggag
194161 gcacaccaaa ccatggtctg actgcaggcc cttaaagtga gctaatcaag
aggacgactg
194221 ctggtgtgag agggggcag aaggaaatga aaccagagcc gtttgagtct
ggtcgcatga
194281 tttgtcatgg aactggtctg gaggttgtag gattatcctg gacctccttt
cagatcacag
194341 atcagagggg aaataatctg ccccacatac aggcatacac actttatttg
ttgtgctaac
194401 ttgcttttta ttaaaaaaaa aaagaatcag atttttcaga ctttgatgga
tctgccactg
194461 atctgtcggg agctttacct ccctttacca ggtccctctt tttgaactgg
ctatttcaca

194521 gcgtagggtt gagtccaaca ttaaagggat taaagcagac atttctgaag
cggattctct
194581 ttctggccaa cctgttctaa ctcctagttg gcagataatg aaagcctggt
ctgggtcctt
194641 gggcttggac gtgactctat ggatgctcct ctggtctctg ctccacacat
agaggctgtt
194701 tcatcagtgg gattccccca acatctttgg gttagcttta gcggaccgtc
tgtctaaata
194761 aacggttggt taaggcagtg aaggtgcatc aaagaattca tcaaagccac
aaagtgacgg
194821 gaatctcgtc ttgggtaact aaactaaatc gttctaatta aatggccatg
tttttaggca
194881 ctagctgcat ttccattttt cacacatttt gctaatgcac aattgtgttt
tgtacgtgtt
194941 tccattgaag ggtgttttga tcgcaagctt cataattcat gtgaaatctc
atcccaagag
195001 acgccgctgg aagaaagatg gacgacattc gtagttacat cggcggccgt
tgccttgaca
195061 atagtttcta ttgtcaatat gaatttgtag cgctaataaa atattcggtc
tcctcctcgg
195121 tccatattta ctgttccgcc acgcggtccc gttaaccgat acgtcaacac
ataaacacag
195181 ctttgacact ttcgcggtat atttttacat caaaaatgtc taaaattccg
cctcatgagc
195241 ggcaaaacct ttgagcggtt tttgaaaggt tatctgaaat acaggtgttc
ctatggaaac
195301 ccagctgctg atcaacacgc tgggtttacg agatgaggag atcttttcaa
cggatgtttg
195361 aggcaagcga aatcacccag tggtgctgcc ccactgagtg ggtgacaaat
aaatacagat
195421 atattagtcg tcaacttttt gaaatgatct gtgtgagata agaatgtctg
accaccaact
195481 tcagcaacgc tgataaaaag ggctaaaagt gtcccaaacc tttgttatcc
gtccctgcaa
195541 cagctttcta ttaacattca aatgaaaccg aatgtcttgg gagattattt
gttcatttgc
195601 cgccctgaaa tgcgtctgca ttgctagctt agttcccagc tggctactat
tttcaatcag
195661 ctgagcgagg aaggaaattg caaataaaat tccgcccccc cccattccca
tgatacctca
195721 ctacattatt tgtttggttt taatttgaac ccccgcctcg accctataag
acagcatgaa
195781 acatgcatac aaatcttcac ttgtgcgcaa tgacacacat ataaacatgc
tagcacgtca
195841 ctcaagactt cctgtggcgt gtggaatctg ttttacatca aatcaacctt
catccggccc
195901 gtcagtggtc aatttttata cagaaagcca gagattacat gcagaactcg
ggcagggcaa
195961 ccttggtttg agtgcataaa tcattgacag gctgcgcgtt gctccgtatt
cgggaaagcc
196021 cacatgtgat cccaaaagca tccagtaatg aatgtatgtg ctggaggtca
cagagccggc
196081 gaaagcaaat aaactgggct gaatgcaaac aggctgcgca tctgcctgcg
agaagatgat

196141 tggacacaat aattagacga agaagtcgtc gtttttggca gctcctctca cgcagaggcc
196201 ataaataagc tctgggaggg tttgtgtttt cagtcagaaa taaaccccac agagcgttca
196261 aaatagaata ttagatgctg tgaatacggg agtcattgat tcctgatcaa tggagagaag
196321 atcagaggat tagaattgac agaaaagcaa ttgctcttta aaaacatgtt gttttgtttt
196381 gttttaatgt tacgagaaat taccttctca gacaagttag ctggcagaaa ggcaaattat
196441 gcttcaacgg atgaattaag ttctccgaat cacgcgcagc agccttaaat catcggttgt
196501 caaaattaat ggagaaggtt cttcttttca acatcgggac aggacacgac tttatctgcc
196561 acatccaatt agtcgcactg catcttcggg gatgccttta aagtgtcagt caggaggccg
196621 taaacattgt ctttcagcag cagtcccacc tcgaggaggg gggggggggg gggctctaaa
196681 caacaaagtc agcgctttga tatctctgct tctcaccttc ccataaaaca ccgccgcaaa
196741 acactaacgc atccctcttt tttagacttt acggcctctc ctcacactat tacccatgat
196801 cacacaatca gcgtcgggtt ttacgaccgt gaaaagagcg taggggcgcg cacgcgtggg
196861 gggaggggca gagggtgagg gacgttttgg acaggtgaga aaaaaaaaaa gcccccccctc
196921 ccaaaaaaag gggaggacag ttgcgtcaca cgcacgtaca agtgcacaca aacacaaacc
196981 tgcccacccc tgactgctgt gtcttgcccc cccccccgc tgcccttagc tgtttcctgg
197041 attccagatg acgtcctgac aggagggctg agtgagtgga ggtgaggctg tgtgatgaca
197101 gagcagcctc tttatcagtg accagcacac cgccagcttg ctgtttgaag attaacgggc
197161 agattggagc ccaaaactgc cgccgccccc gtgtgacacc ccctcccccct cctcctcctc
197221 cttcccccct ctcttttttt ttaaacattt cgtttattta tttgggtttt aacccctcgc
197281 tcgccggctg ctgaagctct ccgggtttaa tgtaattggc ctatttcctg aatgtgaagt
197341 tcctgcttcg ctgtaattta ctgggacttt tatgggctgt gagacaggtt caatgggaac
197401 caaatggaga ggcaacacag gaagctctgg gccggacaac ctgatactca tcaagtcaac
197461 caacccggac gcagcacaat gaagtgacga gactggccgc agagacttgc agggctgctg
197521 agggatctga gtgctagcat gtaaccggcc tgcacaagcc agtcggtacc tctccatacg
197581 tctttgtccc agtatacgtg cacagaagtg taactgattt attgtctctg ctaccaagag
197641 tgtaactcag taagtttttc agcttgttag ggtcatttaa tggcagactt caaaaccgta
197701 agctaactac ttagcaagca acaaatagcc tggcaatgag cagcgttgcc tattggttta

197761 ccttttttgat gattaaaact tcaatttcca tgaggctaac atgtttacat
gcactttaac
197821 attccagttt tggtagaata ctttgatttt tgctaaaggc acagaaacag
gcatcattcg
197881 aacaagtgaa tgttttagca ggtgtaaagc ttcaacctcc tttttatttt
gttgattatt
197941 ctgtttagta gcccacataa ttgccgggca cttttctctc agcagaaaag
acgcgttgag
198001 atccaactgg ttttatcaca acatctcatg gcacgcaacg cgacaaagca
gatattttgc
198061 tctttgatgt ctgccagagt aaatggacta tcgatgcgga gtacaaacac
tcctctatgc
198121 taattctcct ccggggctct ttgatggcat cagttgtata tttgaagtgt
gttttgaaaa
198181 attgcatcgg tgagccatcc aaatagttag aattaaagcc ttttcactca
caacacctcc
198241 ccagtcacgt gcacgagggc ttctagattg agaaaagagt gctcagtagt
ggctcaaact
198301 ggcgcataaa taaccggcag cagcacgggc gaaccaagcc gccgcggtgg
cagcggtcca
198361 gtcggctgcc aggtgacatc atttggcatt gtttcaacac tccatttgtt
tgattgtgct
198421 gattgtcctc ccacattcac tcgatcaagt gggtctctat tttgtgtgcc
cggttgtttc
198481 ggtagtccag gtgcagcgcg cttgtggagg cttcctgtcc ccccagaggt
cctttcaatg
198541 cctctcccgc cgcgggtatt gtcacaaccc cacagtgcat ctgaaaagaa
gcagcagccc
198601 tctgtaaatg tcaaagaggt gacttgtttt taactgttcg tgggcacttg
gtgtgtgtga
198661 gtgcaccgtc gggtccacgg tgtggcgccc cctgttgaaa ttcgatggtg
ggattgggac
198721 tgtttttaaa atgcacagag aacaggcttg cattaattta cacctgcttc
cttcctcctc
198781 agtcagcccg ctttgtgatt ggctacaatc tgttccttgt cacgagtcac
agagtcctga
198841 cctgagagtc gttggctctc cccacacaca tcaaagattt tgcttgtaaa
tgctaatatt
198901 tatgattgtc ggcccctcat ataggacacc acgggagaac tttataagac
aaccttacca
198961 agctaactgg gcgtttgcct tccttggtag ggaagggggc aatcaggaca
aaaacagccc
199021 cgatgatatg tacatcctga tgtacatgaa tagatctttt gagcatccta
cgtgttctgt
199081 agttcctcat tcctgcgttg agcagaaaag atcaacataa tggcagattg
tagcataaag
199141 ttggtatttg tggcgctacg acactttcca gaaaacttaa cacttgccag
caaagcggca
199201 aatttatatt tcatccttga ggtgcaattg tatcactcac aataaagccg
acaaatgtgt
199261 gttttagcag cacagcagga caataaggcg agcgaggcta atggctatag
catgagcgag
199321 gagacaaaag agagacggag gcgcagatgt gatgtcatgg caggttcaat
cagaccatct

199381 gagcgtctca gcagacggac gaagacaaag tccaatttgt ccaacagttt ctcagacacg
199441 tgtgcttcct gctcaggccc tgcgttcgga caaatattca ccattttccc gcgtgaccgc
199501 cggtgggaaa gatgtcctga tcttttcccc gtgggttgag ttcgtcaaca attagagtga
199561 cttccctcac ccccgccaca cccccagcgt gagctttcag caaacaggag ggaaaaacga
199621 gaccttgtcc cttgtcctcc cgctgtctcg cctttttttaa gacacccgcg gccttttctg
199681 ttgtacacac gctgcagtct ggcgggcggc tcgcgggcgc catccagtta gctgggtatt
199741 ttggtcggtg tgagtgggca gcggtgtcat tctgcgagct ttgagctcag tggtgggaga
199801 gctgtgaaaa gccgtcacta acaagcggag agattccctc cctgcatccc acagccagat
199861 ggtgaagctc cgaggaatca gaaacctcct ttatagtccg acagaaacac gggggaatca
199921 aacgcaccgc ggacgagctg cacaacatct cagctcctgt cggtgatgaa aacagccgtt
199981 cgtgcgcagt cattttagaa tttagtatcc cgtaaaagca aggaaaggag ggaaaagtgc
200041 tcctgaaggc cggggtcacc gctattcctc gacctccaat atttgacagt tagttaacaa
200101 aataagacta aagtcaacta tctgtgtttt atcagtaagt gtatacagac cagagacctt
200161 tcaaatctaa agtcgggaac acatgccaac gtatttactt gatcgtgtac agatggagga
200221 caacaagctg gaccccgggg aggcgtcctg ttcctcagct ggaggggaca tcaccagcag
200281 cgctcgctca cctccagacg tgcatctgga tcctgtctcc gacaggctca tctagttagc
200341 tgacctgagt gctggtggtg gagccatgcc ataaagagtt aaaatgataa taaaaacaaa
200401 ataactctac acacacacac acacacacac actttctctc tctctcttcc tcgtacttgc
200461 gattcataaa taaaagacat taagattttc agccacattt gctcactttt atcaaagtaa
200521 atttgaatct caagccataa aaatacttta tttttctttg tctaggtggc aaaacagaca
200581 ccagagctcc gcatgtgccc tgcgatcggt atcaattcat gatttccggg ctgaggtgaa
200641 gtccaaaagt tcgctgaaat gagacagcac ctactggcag ccacagaatc acctcccgtc
200701 ccatctgcct ccaaataaca ccaaaaaaat aaaataaata agtgaaaata cacccgctct
200761 ttatgcctgc ctccgcccgt ggcctaaatt atgacaagct tgattcatgc tggtgcagtg
200821 ccagtggatc gcccgaggat gcccccgggt gtctgagcca gcgtatccat tagctacctg
200881 tgtcggctgc tcctccgagg tgtgcagaca ctgatgtctg cctttctttc ctttaaagag
200941 atccccgagc cacacacact ctctccatcc ctctccatcc ctctctctct ctccatccct

201001 ctctctctct ctctccatcc ctctctctct cgaagctcac catctggcta aggattgagc
201061 accatcctgc aacagcctcc gatggtctga ccacttagcc cttttcctgt cactcagcct
201121 catcaacaac cgccctcggc acaggtggca ttcccttatt cctcctccat tgggggaaga
201181 agtactgaca gaaggtttgc gctaaacacg gtacactggg aaaataataa taataaaaca
201241 tgcattttaa tgcatgacgc ctgtcggtgt ttgaatcagc tcttttggct cacaaggggg
201301 tgagatattc tcctctggtg tgcttgaatg tccctactgt agcccagaat ggacactaag
201361 cattactgtt ccattgttgc agagatctgg atttccaatt ttcatttgac agcctaaatg
201421 catcgtcacg agtgaaagta gtgtggtgga aacatcagtt ttgttcccca gtagccacca
201481 tcaaaacata tactgccgtg gtaatttcac cttttattcc taccagcaga caccgacaat
201541 ccctacatgt ttgactccaa agaattgaat aaaaaaacat gctacctgtt gcatttaaaa
201601 acaaacaaac aaacaaaaaa aacccattta gctctcttcg aaacatgcat tttcagtcag
201661 ttctgtttgg cgtcggacag cctaattaat gttatttaca ctttgcagca tcaggcgctt
201721 gattttctga acaccagcaa actggaacag ctgtttgata gagagaaact aatgatgccg
201781 tgcccggaac caccagcggg gagcccgaca ccacgggcgc cgtcgctcaa caacagaatg
201841 tcaacgacag cagggcctgt gtttggggaa ggcacttgtc aatcacgttc ccagccagcg
201901 ggggatcagg tgcaccggcc aatcagagaa ggtccctgca cagcaggttc aggaccagga
201961 agtgaaacgc catctggagt gacaagaagc cgcgagtggg caacagaaac aacgtgccga
202021 cgtggataca acaagggcgt ttggaaccgt gctgccgttc ccgccaagtc tgttgttggc
202081 tgcagtcgtc cgatggcgct catcgcagat gtgcacacgc gtttcttctc agggcgttgc
202141 taaaaatttg ccagctgata gatttttttt taaaatgaaa agctggaaat ggccaaattg
202201 aaaattatga cgtgaaagaa aggatgaaat gaaactccat taaaacaagt tctggatgga
202261 gagagcgctc tcttctcccg tctttggtgt aacgaggatc ttttattccc ctgataagag
202321 acagatctgc tgagagatcg gtcggaggaa atggaactga atggaaatct ttgcgctaca
202381 tttactcaat catcaatgtg attaatagca gttaacacct cgtaaaaata gagccgcaat
202441 tatggcggaa aattagggtc tggcattacc ctcatgctaa taagtaccga atataattac
202501 atttatctca gtattgtcac agagattgat aaattcatgc tcaatctcgt taatctctca
202561 ataacatcca gcaaagactc gcgcaatcgt ctttttttggt cacactgata gtggggaaaa

202621 aagcctgtgt gggagaaaga ggaggagata gtggcgagag atgacaggag gacgggatgg
202681 ggaaaggcag cgccagtgag cagatatgcc tctgtccatc tcgagtattt ttcatcctat
202741 tcccagcaga ggggataaaa cagataatta ccaactctaa aaactgtgtc tttatgtgca
202801 gctctgaaag gagaaactct ccagcgctga agagataaag ctcaaaacaa aagcaggctg
202861 aattggacca ggattagctt taatggtggt aattaccaga ggggataaca gcccggtcct
202921 ctctgcccat caccaaggcc cagagcgggg gggggggatc tgaatcaggg cccacccttc
202981 gggggcccgt gtggcttcat ggtggcagca gtttagtaca ttcaattgta aaacacttgg
203041 acggccgatc cgtgtctggc ctctcctgca ctaaaagcct ctatggtagc tcgctagcac
203101 aataatggcc ttgcataaaa aagggaggtc cttcacactg ttgctccttg aattacaaac
203161 tgaggtgatg gcacagatag gagtgcccta aattatagca gcgggggcag tgggaccttt
203221 cttccccttg tctgggtagc atgggtagta tgggtgtgcc aataaaccgt tattgccccg
203281 ctgacaacga tgggctctgc tgctaaaaaa caagagaaat taaaatgcaa catggtgcca
203341 ccttatcagt gaaagatggc cccccagaca ccagaccgta caaaagagag acgagaagag
203401 ccgtgtgcgg gacaatggag tgttcaggag gctataattt atctgggttt aaatctttgc
203461 gtctgaactc caatttaatg tatagcctgg agcatgtgcc atctaaatca gactgctgca
203521 tatacccgtg tggatccttc agccccctt acttgtgtac agtatcagcc ccacagattc
203581 ccaaggtgta cgtggatgtc tgacctgtgg tccgttggtt tcctgaaact cagatcaata
203641 aagcccagcc aagccaacaa cctatccacg gggaggccgg atccataaat atctcatatg
203701 tccagctgag ccagactgtc caaagcctga ccggcataaa gtgatctcac tagttcagga
203761 tgtgtagaat aagacgccct ctctgcgctg cttaacttcc cattgatcac ataaatgcgt
203821 gaattgtttc tgtgaaaacg tgggaaggat gtgttcattg tttcgttatc agcgtcaaag
203881 agacgaggat aaagagccgc gggcctacaa ggttgcgctg gtgatctatt actgcctcat
203941 atttaacctg tagacgctca ggtgaggcag ccagggtctg gagttttctt ttaattagct
204001 tttttttttt agctccgata gcaaaagctt tcacacggga gaatggagct gccactcagg
204061 actcaaccgg ggacagcgga gcttcagcct atagctctta tctcagggat gaatcaagag
204121 gcatcatcat gtttccgggt ctgttaatgc tccctgggtg actgcccct cccacacaca
204181 gtcagccctc tgcggaatag ctcccgctaa tctgttccca aatatagggt tcacctcaaa

204241 tgcaatgtgg attatttaca ggctgatatt ggaagttcat gcggggaaac
atggctgcag
204301 ctcgatttgg tccccacgtc tctgaattca aacgatgctg ccccttgtca
ctcagaaggt
204361 gcgagggata actcgtcaaa gatgagcaac gttattacat ccagcgacac
agcgatgagc
204421 catttcccgg aggatggcat cagaactcca gccgctcgtc tggctgcctg
taatcaaaaa
204481 ccaccatctc tctccctcca tctttcataa ataagtttgt ggttatcatc
tatttttccc
204541 ctctacagct tcattttaat ccaggcgaaa gggagcagat gaggcgttaa
tgtgggcgat
204601 cgtcacagcc cccacacgca cccaccgtgg caaattaaac gccgcgggca
tccgtgttga
204661 cccaaacctc gcagaggcaa acatgcgcgg tctccagcac cctgtgaaga
agaggcaggc
204721 agcagcaggc atccacaatc acctctccac aactgtgtgg ctgcaaagat
aaagctatat
204781 tgtaaacaca acaggtgacc gactgcacaa gaactgcccg gggacgccgg
ctggtgagtg
204841 gaaggaagcg ggtgcagcct tggttaaatc acctaaatat ctgagggatg
cacgccttcg
204901 atctgaccat aatatgactc tgacgcatgt gacatggttt ggaccatcgt
ggtccatgtg
204961 tgccagatgc acatggtggt gttcatttga aacacatgac tcttgcacaa
cgtgcactcc
205021 tgtgcatgca ctctactctc tctctctctc tctccctccc cttttcacac
agacacactg
205081 agcccataaa attcagatat ctccgtactc catcctctgt gagaccttat
tgtcctggtt
205141 attaccccgg gtattgttac gaagaattgg tatgcttatc aaggcctgta
gtctaaagtt
205201 gatacgacac tggcctggca gcttcattaa gattgatatc ctgccacagc
gcactcagat
205261 gagccgatcc aggacacggg aaggagcagc cgagcggagt tggaggaaga
cggagagaga
205321 aagggactgt aggtctacct cccaagtagc tcaattgcca tctattgagc
cacttgagct
205381 caggaaaggg accaagcatg catacctctc ctttaaggga acgctcccct
ctgtaacctc
205441 accctgccag tcataaatta ccttcctcaa ctcactccct ttcacagtga
atgcacagaa
205501 ggcccctata gcctctccac gagcaattaa ggcctcaata ggatggtatc
aaaccgccct
205561 gacctcatcc aacccatcct gtcattgtca cctcctgaaa ggtctcctcc
accgcgtctc
205621 cagagtgcaa ctcctctgtc agggcagaga ccattttact ggccaccagc
tcattttcca
205681 gtcttatcac gagccataca aacagggtca gagttcaact ccactggaag
ttcgtcatat
205741 attctgagca atctttacag ctagtttaac aatgccagtt caccctaaat
gtacccagaa
205801 ccaaccgcgt catttaacgg attactttca gtccagctga cggcctctgc
agccacgctg

205861 tcacgtgggc cccattcggg ccgtccgccc ggctggaacg aaaacttctg ctttatgaga
205921 agtgctaaac atgggctgta caggtcactg aggattgctg agcgtacatt aaccgagcgc
205981 accggcatgt ctaatttgct gcggcgggca agacaaagac tttcattgat atcaccgtat
206041 cgagtcaagt ctggacacac tggatatctg tggtttcccc tggccagacc taatgattta
206101 ttgtgtcacg ctatctaatt ggcaaggccc tgagaaaaga catatatatt tttaatcact
206161 gttaaccatg tcaataagaa tactctgggt aaggaaacac aaaaaacaaa gaaaagactc
206221 ccgatttcct tttctcttat cagagaattc agttggttca ggcattagtt ctaagctttc
206281 ctttcacctt taaggacctt cattagagag aggagcttca aatgggagcc actgagtaaa
206341 catctgttat tgaaagacat aatgtcaagc caaatgcgcg ccgctctcct tgtcttgact
206401 ctgtacattg cgtttaaaca ggtgggggac acgataagac caacagtgag ggctaaaatg
206461 aaatttcttt cattaagata atcatatgca gacacttgtt caatgccacc tatgacttct
206521 acgtaatggc gcagtccttt attttttgtg ccgcatgaat gcaaaaatta tgcccgacca
206581 caagagttta atgcttgtgt cctccgcttt ctgggctttt tattgatgct acggtattct
206641 gagctggctg tggatgacag gactcagagt ttaaatgtca aactctacca gacccactgg
206701 tcagtctggt gcacacagaa cagcattatg ggagtggggc agttgcagca aggcctttct
206761 gatggcctat agagtgctgc ctatatgcac acagagtcat caggcagatg tgacctcggg
206821 agcacacaca ggctctttgt gtccgaggga tttagagtcc cctgggcctc ctgactctaa
206881 acattaacag acctactgca ggaggggcga gagagtaaga gagggggaga gagggagaga
206941 accttactct gctgacatgc aacattacac tgcgttaaac tctcttgctc tacttaaaat
207001 ccttggaagg agggccatat atgtcttgct gcatgcacaa gtgaagacaa taagttgatg
207061 caaactccag cacacccaca ccgcagaagc agcgtgctta gtttatagat atcacttacg
207121 tacttgaggg gatatactga aatataccta tatatgtctc tgtatctata tctatagatc
207181 gctctctcac tctctctctc tctatatata tacagtatat atactgcagt tcacaattgt
207241 tcccatgcaa taaaggtaag gtttcagagt acttaaagga gtcctgaacc caccaatttc
207301 tacgctgcaa cctgagcaga ggccgccccc cccccgattt gcacaattca acacatcaag
207361 cttgactgct gtgttgtgac gtgattgtgc gtgcaggaaa ataaaacagt ggcttcggat
207421 aaaggaccca attatctgtc agcgattgat gttatgagcc tgacgtacag gaacttatct

207481 ggattcttgg tgctttcttt cccctctgta ataacattat tattcaccgc tgtggctaaa
207541 gctgcctccc agttcacggg tattctcgtc ttttttgcta ataataatga tacaacattt
207601 gcataaagga aatgaaacag gttacatttg catatattaa acatttatat gtatgcatgt
207661 aatatgtttg ctcatttcag aaaactaggc aagataatag taagactacg acaggaaata
207721 gtagcagcac aatccacagt aaatattggc cttcgttatc tgccatcccc tcagacagga
207781 ggctgtatca tttttcagca aagagccccc tgttcttgag gactaggcca ggccttgctt
207841 tattaaatta tctcccttgc ttcgcaaata caatttcctg cataaatcac tcaaatgatc
207901 cttcagactg tcaggtccga gagagcgttt tctttcctat ctgcctccga ccaggttatg
207961 caaatgcttg ccagtgtgtg ttcgatatct attgagatgc ttcaattgtc tgtgtttggc
208021 ctcaaatcct tcgcttgtcc ccatattatt cccctgccct tgatggaaga cacgctaagc
208081 atcgagccat caccaactcg gtaatccatt accgaaaaaa aaaagagcat gccaatttac
208141 ctaagccaga aacactgacc ctgcaacctt gaccttgtaa acattacaat agggggaggc
208201 cacagtttgt gcatcggtga tccgctctga agcccattgg aagagcttat tatggccctt
208261 aagctggggc tagattcaat catattggta aaaggactga aaaagacaaa tctttgctag
208321 cggtcagctt aaggtcccaa tacagaaata actgtaccaa aaagaaggcc acattatcat
208381 tgctgctgca gtcaggtagc cagctggagg ataaacatct gtaattcatc ggctcaaaat
208441 aagagccagc ttaaaattac ttcagcttga tcccagacag ctgtttaggc agcagcactg
208501 gcctgactgc acagggggtc tcagagcagg tcctgaaaag gggacctcca aaaaaactac
208561 aaagagacaa aggattcttc tgaacaatgt tctcgcagat acaatggtgg tgcacagggt
208621 ggaagggcca aaggagcagc gcctattctg aggaaatgaa tgaccagtgc attcctctgc
208681 agtcaccgag gagtgggcga agtgaccaag agtgacaagt tttcacgccg ctttgtcgac
208741 agtcttcctg cttgaccaat agattccttc tgctttccac ctcatccctt cacgttgttc
208801 tgcatccttt ctcctttaaa ctctactttc agtcattgat taaaccctca acggtaatgg
208861 ctgcctttct aaaaacatta gcattaaact agcaaattgt caatgtttag catttcagac
208921 atcagaaacc tcagctccgc ttcagccttc aacaccggta atcctcttcc tgaccagttg
208981 ctggatttgg ttttgttgta tctgagttgt cttgacctca acttctgtgg gtggatttgg
209041 agaaagaaga gagaactgct cagttgtgac agatttggct cattttagtc atacaatatg

209101 tcacagtgaa taaaaaatga aatcctacca atgtgacaca gagtttagtt ggaacgggct
209161 tattcaagtc ctacgtgggt gtaacaggag tccactaaga attgtcagtg cttatattca
209221 caataaaata tggtgtggag cgtgcacata aatacacacc ggggtcaagt ctctgcatag
209281 cattactgac atgcatttgg agcacatgtt tattctccat acaagctaca catcagtcct
209341 ttgacaagtg aaatgggtga tgtaacgtat gggccagaat cgaggggttc accaagagga
209401 cagcgaattt gcaggatttt atcttctgtc acacacaggc gccctccctc tttctctagc
209461 acacacataa aggttgagac tggctgggtt gttgcacttt acttgagagg aatttagcta
209521 ggtacagata gaaagcgggc agaaaatcgg caaagaaatg caggttcatc ctatattttc
209581 gtgcatttcc gtggctagga ggctccgatg tgctcatttg aacgtacctg gtgtcaacag
209641 agtgacagtg ggaaaacaac actacagaga gcgccatttt ggagcccagt ctgcgaagaa
209701 agtgtacagc tattaattga ctaggtagga cccgtggtaa tcccgccgag ggatggcatc
209761 agcattcgca cctgtgacag atgcagaaaa atgagggggc tcattaaaaa tttgattcag
209821 atctgttgca ggcaccctgt ggagtcgctt taaagctagg cgcccatcgg aatgataggt
209881 ggcaaatata gtagcagggg gaaattggac ttgaaaagga atgtctgtct ctccattttt
209941 gaaaggcttc ctcttaataa cctattttcc tttgtactct caaacaaaat ggcttctata
210001 gttgtacgcg ttgcctttga aggtgcgccg tgactggcgt acatggatgt ctccttgtag
210061 ggccttaatt tgacgcttga gcactgctga aatgttgctc cccttttgaa aatagtagac
210121 ttttgcttga gttaaagtaa accagatcaa tttttcatgc acctgagcta aacataggag
210181 ctgcgctttc ccggcacagg agtggaacct gcgtcatttg acttgaactg gaagctcagt
210241 tcactgtgac ctctgcagag gggttcagct agtccacggc ggggagtgtg tacaagcgct
210301 ggaaatgaat tcggtaaagc gcattactct ggtgatgaga aacgttccgg taaattatgt
210361 cacaaagcat aattcatgcg ataaaagagg gaaatattgc atagacggaa cagattattg
210421 gtttggaggc acgtaactcc aggctatgaa tatctcaact acctagtgta agagataata
210481 agcaccatct taatggttta tgaatcccat ttgatttatg gttattaacc agaggagaat
210541 tatagtggcg aggtattgat gaatgggggg tgtttcagga tagggcccag gtgattgaaa
210601 tggtataatc tgggtgttgg taggccagtt ttagcaccaa tgatctgaaa ttgttctact
210661 tgcatttgaa tcaaaccgat ggtaccacag aaccagcaaa ttatttccac ttcttaagtt

210721 cacgttgatt tatttagatt tgttgttttt gtaattggct attattagaa
aatcctgttt
210781 agaaaatcct acttcaagat tgcctcctac catacaaaca tagtggaaca
actttttatc
210841 ccaaatgtgt aaattgtaaa attatacatc tgactttagg tgcgaagcac
atttagtaga
210901 atccaaagga cacagatgct tgattataat aacaacagtg cagttgtatt
attattaggc
210961 actatttgtt attcccacca tattacttct aatcctcaaa aaaacctccc
taaaccaaaa
211021 tgtcacgaca agcaactttc tcatttgttt gattttgtat tgttcgcaat
ggcaggtgta
211081 aaaaaatgac ggcgagaaaa gaaataagag atggttctat ccagggaccg
atattattcc
211141 tgtcccacac agggcttcaa gcgagcagat ccccttgatg atgcagcccc
ttcctcccat
211201 acacaccacc aacaccaacc cataaccacc ccccaaaaaa gaaaaaatag
agcatcagtg
211261 aagtaagtgt ggcacagatt ggagaaggca tgagcagatt ttcaatagga
tttcggcaga
211321 aggcacaagg ggcagaaatc ttttgctttt gtctgccgtt caaatgggtc
tgatgagcct
211381 cgagcagcag gctcagcttg tcatacgggt cctgatctct tgtgagaatt
gatttgcatc
211441 atttgggggg agcggtacat gcctgggaga acaagataag ggacaagaga
actgagacgg
211501 agttactgta aaagaaagag ggcccccgac aagccaaagg ggaggtgatc
cggggcaagc
211561 ggaggaaaac agtatagaaa aaaataggtt aagtcgtccc acggtccatt
ctgaaatgtt
211621 ccactagtta tcgcagggta agaagctcgt tcctgttgta acgttttctc
tctgccaggc
211681 atttcatctg attatgagta ctttgcaatc tgcgacagca tgattgatgc
tccactttca
211741 tgaggggaat tttaacagca cctcttcagc gctcaatgcg cacccaatta
tgcgtctgag
211801 ctggtaacct tgaatttatg tcaccatggc aacaaaaccc ccggtcatgc
attatggatt
211861 atcattgaaa ttctacaccc ctacctaaca taatgagagc ccaggactta
atcaaatcag
211921 cccattctgt gtgacacaaa gtgaagaagg agctctcatt gtgttgaggc
tcgggctggg
211981 agaagccagc caggcaggca ggcaggcagg caggcaggca ggcaggcaag
caggcagaca
212041 agcaggcagg gaggcaggca ggcagacaag caggcaggca gacaagcagg
cagacaagca
212101 ggcagggagg caggcaggca gacaagcagg caggcagaca agcaggcaga
caagcaggca
212161 ggcagacaag caggcaggca ggcaggcagg cagacaagca ggcaggcagg
caggcacgca
212221 ggcaggcaag caggcaggca gacaagcagg cagggaggca ggcaggcagg
caggcaggcg
212281 ggtggcagac tggggtgtga cggccccagc agctgcaggg ctagcagcct
gatagatggc

212341 tcccatatac gtcagtgaac atatagacag atgggccctc aatggctgga atttccagcc
212401 caggaagcag ctgctcagcc taacctctgc tgtccgcctc accttgttgt catggaacat
212461 tgagcagaga aggagaatgt ggacggggac atggtgcgga gtgtcgcgga gatccacaat
212521 gctccaggct gggacaagcg gggggggggg gggggggggg cctttaaaaa attctgaggg
212581 aaaacttgaa tttnnnnnnn nnnnnnnnnn nnnnnnnnnn nnnnnnnnnn nnnnnnnnnn
212641 nnnnnnnnnn nnnnnnnnnn nnnnnnnnnn nnnnnnnnnn nnnnnnnnnn nnnnnnnnnn
212701 nnnnnnnnnn nnnnnnnnnn nnnnnnnnnn nnnnnnnnng aagccccgaa tgattcaatc
212761 tcttctctct tttcagcatc tgaatgaaat aattgctgaa aatcactgcg ctggatttaa
212821 cgcgccctgt ttttcatgtt ctcaaggctg ctttgtgctg tgcttgcagt tagacgtaat
212881 attgattgtg cttgtctatt tgcctcataa tgtaagcact gttattagct aacaggaaaa
212941 ataaacgata tcacggaaca ttaaaaccag tatgttggct ctttaaagca tcttcagtgt
213001 ttttcacaca gaaggaagaa ataagggctg gacaatatgt tgggatgagg aaaattgcga
213061 ctttttgaaa caacatgacc caaaattcca tatggagaaa tgtcaattta attctgccta
213121 tcgaacgact tttctaataa ggcaggattt aaatctggtt tccctccaca gaataagaag
213181 tttaataaat ttaagctcca atttagaagc agaacatagg gaaatctata taatctgatt
213241 catttaatgg gaacagtcat attgaaacat aatcagacac gagcaatggt tcaaggtcta
213301 caaaaatgga aaaggtgaat caaacctacg agtactattt atcacagatt tgttttccaa
213361 taccсttccc ataccttgct cagaaaactg ccagtaaaga acaaaatctg ccatgaataa
213421 aaacaaagaa cgtgaaacgc ccgattcaca actgcaagac ataaaaaaca gatttcatga
213481 atgcatattt tggccacaac agagaagtga cctcctaaat agtcacatat gcatttattg
213541 tctctttcca aagcaatagt gacagttttg ggaggctctg aaaagggacc atgaggcacg
213601 ggtgtcttat tcattcattt atttatttat ttcattttcg agtctataac aacccagttt
213661 tggaagcaaa acaaagtctt ctggaggcac tcaggcatat caaatttatt ctgagcacag
213721 cagtgagagg ggacagtctt ccagcatcgg aatactaata tccagtattc agcgggagaa
213781 aaatgaaacc aaagtgtttt aaagtaatgg ggggaaaaaa gtttatgtaa aacaaatggg
213841 aattcagcca ttcatcggct ccctgggtcc tcggcagcac tctaatacat ctgcatattt
213901 ccaataagca caacggcgta ataaatgtta ttttctgtga agtaagtgca tcacaagcag

213961 aggtgtcatc taaaatgtat ttgtgatgcg cttgatgaag cctgctcatc
gctctgtaat
214021 gacaccccag cctctctcat gtgttatgca tcacgtcttg ttaccatccg
ctcctgagca
214081 cgcctgtcat cgtgtgccgt ctcgggcccc tctggaatcg tccctgagac
tgaagggtgt
214141 gtgtgtgggg ggggcaaaag gaggggatgg gagtggtctg attagatgcc
tcttcaactt
214201 ctcaaactcc gggaggtgtc attccgccac tggggtttgc tgaggctacc
cccccgcccc
214261 cgacgccatt acggagcaga acggagacgg tgtgataagt gaagtagcga
gtgtgggatt
214321 cgtctgttca ttattgatgc agtatttttg gttattagaa cgtctttcct
gcattatgca
214381 tatgaagttc atggagagtg ttgtggttta ccagccgagg acgagaacgt
cccagcctct
214441 gtgtgaactt cctgcgtgtc aaacatcgtg tgtgtcgggg gtgggctttc
tactgacata
214501 gaagtcgacg taaaaataag cttttctgca tctgactgga gttttggtct
ttcatggctg
214561 cagttagccc ggatgtttcg tcaactcggc atatcgcaga agcaaatcag
gtcaacatcg
214621 accacaacga caacaacaat gttcattctg gatcacacaa gtgaaataat
cactgttaaa
214681 tgatgctcct tagccagcgc gtgtggaatt ggactgaggg atacaacgga
cagatgcttt
214741 catggcacaa acacccactg acagtagttt actgtttatc ctccagaggc
tgtacccttg
214801 tatacgtgcc tgactgtcaa taccggtgca gcactgggaa agccttccaa
agatcactga
214861 ggagtaactt cttcattaat catcatggat gaagactgat tatatatagg
actggaatga
214921 aataatggct tggcctcatt tgctaacctt cctctgacag ctatgctact
gggttacaag
214981 gagagtcagg cggctgcagt ccccatcccc tcatttgcat cgaattaatg
atgcatgcgt
215041 atgcaaatga gcattttaaa caatgttgat tactgcctat gtattcatag
gccaacagtc
215101 acacgtgtct gacatgccac ttctgcatgt aatttgcacc acggtaaagg
gcaactggtg
215161 ctgtctctca gaaggcgatc acggccgtag gcgccggtgg taactgattc
atccgcgctt
215221 tgactggtgt ttatttattg gcctgaatct ctggcattat ttatcactga
taatgtgtac
215281 agagtcagca gctctgtgca tggaaatgtg agatatgccc tcagccagga
ggaaatggct
215341 gctgccccat acagtaaatg ctagcagtgc agccgcttcc atagctgtgc
cgtggaggct
215401 cacgtctatc gcccctttaa caacaacatt cccaggagag tttaatacca
gggatttatg
215461 tagggtttgt gcttccatcg cagctaataa ttgtactgaa gtcagttttg
atgacatacg
215521 ggggagtgac acaaaggtcg gagccaccca gaccttgtta tggtccttta
accccttatg

215581 atcttgttta ggttttggtc aatttttcaa gaaagtcttt gacaaatacg cttaaaacag
215641 gcgcttttct cgtttaggtt catcttacaa atcaacaaaa ttaaacatat agacacgaag
215701 caaaggcaaa aaaaaaaaaa gtagtccagc catttcaaaa gtcctaaccc aaaaacaaaa
215761 aaacaaaaca aacccagcaa tgcaaattaa tactaaaaaa gaagtgtgta agctggaaga
215821 ttacataatg acgctgaacc tgtgaagaga aagtgcccgg tgacctgatc tcacgataga
215881 ccctccaaat actcgaccac ctgagcacgc ggatacatcc tagtgcactt ttgcccagct
215941 ccgaccccca tagaggaagc tgttcacacc aaccctctca tttggttaag ggttcttcag
216001 ttataggcaa atagggctga gagcctctct ctgtgctact acccagccct ggggtcagcc
216061 cagttccaag atggttgcat ctctaatatg atcatgtctt tctatgactt aacgccggcc
216121 tgcagcaaca gcctggagtg gagaaccttt tttttttttt aaatgagcga gggatgaagc
216181 gcacatttta tagcaagaaa gcggatgaca cgtcgagacg tccgtacgga agataaatata
216241 agcggccagc gctggaaatg ggtcattgtt ttacgagctc aaagaatttt atgatgggaa
216301 gaaatttgca cctttggagc catttttgct cctacgatca gggtcacaac ccctgctttt
216361 acaaggttcc attccagcac ctattacact ttactgtgtc aatatttacg atctgattgt
216421 aacctacggc cacattacgt ctacgctttg tcacaatttg catttatagg cctcctctga
216481 atggcctatt tcacttatct gcactcttgc aagggtaacc ccaaagttat gagtgttgtt
216541 ataacacatg ggctcgggac atttccccca gaaccccata tcttggggct gacactaaac
216601 aaatcagatc aatatactga tgtatttttt ccccttccaa caattttttt ttatttcttt
216661 tctttgtcct gacataaaat gctaatgaat gctcagggga tgtctacccc atttagattt
216721 catagaactt ggagcctgca ttgattaatg tgtgcctaac ctctgttttc attgtggcgt
216781 cggaggtggc gtgcacgcgt atgtttgtgt cctggttcga ggttatattt tgggttgcat
216841 gattgaaacc cccgtgtttc catgtggagg taatgaacct gaagacaagc agcagctagc
216901 ctgaaatttc atgcattgag gaagcgtcgg gccacacata aggctggctc ggtcatttgt
216961 tatcgttaat cttcacgttt atggctgcga atatgttatt aggaggcgtg tcttgaggat
217021 ttcactgtga ttgggccctg ctgcgatgac atattgatca gccgtgctgg atagagacgc
217081 ccagaagacc aaagtgaccg agaccttgac tcctggacat ttgcaaaggc atgaccgtga
217141 atgtctggct gatattcagc atcccaggta atttcccttt gtaggggaat caagggcaca

217201 taaagcccat cacatttagg ccccaccatg agccggctgg gtgagtaagt
gacagcacag
217261 cccagaatgg agtccggcaa tgaccagcag ccgcctacgg attacattct
gggaaatact
217321 atccatcttc attcaagcat ttcagtgctc actgaaatga tgtggtgggg
cggaccggat
217381 tcagattggt tctgtgcatc gtgtgtgcgc tagtgcgtac ttcagccatt
ccaggctgga
217441 ctatgttgga caccactttc acattcacac tcacaaccca ctgctgcact
gctgaatcta
217501 gtcctaataa ggtgacatga ttcctgtagt aatcacccat gaaaattttt
ataggtgagc
217561 tgtgactaga aaagggccca ggtcaaagcc tttagcatta ccctggagac
ggctcacgcc
217621 tgcctccatc caggagcaac atctcttgga tctcaatcaa ttatacagca
ggctgatgct
217681 ataacggcgc ttgctatttg ctagctcaag cagcaaatcc catcataaaa
ccaacagcaa
217741 cccatcatat gcaaaaagaa aaatgcgcaa gctagcttat taatgaaccg
ggcctgcgcc
217801 acctatttat gctgcggcat cacttgcgcc ttatcagtct ctgtacgttg
ggaaacaata
217861 cgacattagt catgtgaaaa ttcagggacg gagaatttcc aattcatggg
aacggtccac
217921 acacacgtgt gttcgggggc agtgttgtgg aagtcactga tcagtgtctg
atgaaggtag
217981 catttctctc tgtctgcagc tagacggatg taaaggggaa agccatttgg
cacaccgcac
218041 ccgctgacat ttgggggaga tttagtgata tgtagagaaa gacaattaca
ccggattgtg
218101 tgtgaatcag cattactcca caaggtgatg tcatgcccgc cgtcgctaag
cttcctcgct
218161 aaggctcatg gatgggggct cagtaattac gtggaatgtg tttaatcctc
atcctgaaat
218221 gtaacgcaga ctcactgaat gtctctctca gctctgaaat tggattttcg
actcatttag
218281 gacagtcatc cggagttatt ctgcatgcca cgagctagct ctggttcaga
tagcatgaaa
218341 taaatgcaca aataatcaaa taatggacca cggcatgcta taactacatt
agaacaaggt
218401 gtttccagtc cagcaacgac aaagaagtgg ttgttgagtt attgtgttgt
cctttctgga
218461 cataggctga cagtgtttgt caactggaaa tgaggattaa tccatgagtg
ttgacctggc
218521 tgcggggaga ctgtaatccc aatcacagct aggccgtatt ttatgctaat
aactgttcat
218581 tttattaatt tgctattaaa agtggtgatg tgggtacata tgtataaata
aattatcacg
218641 ccaaaatgtc atcaagatta atggaaagac caacagacgt tgcatttatg
gaaattacaa
218701 cactgctctt tgtaaaccgc cgtccccgct aatggatttt atgcaccttt
acggcagaaa
218761 gctgcggcac ccagagacta gagatcagct ggaattgtgt aggtcattat
gcatgtggac

218821 tcccaacctt cctctctcct tagtcgccgc ctggctgtaa attagaagga caagttgcgt
218881 gcgacaatca ctgtttattg ctgtgtttaa attcaccctc tgtttggaac gacgcgttac
218941 agagtgtagc tttaaataaa aaggagaaga aggcatttaa tataaatgat ctggtgttga
219001 ttgtgatgga tatactatgt agagacacct tgccttcaag ggcatgcagg gataagctat
219061 cattttcctt cccttacgcg tggagggaag aatgacggag ggtccaatgt cattgcagag
219121 tctacaataa cacgccgcaa ctcagggcgc aacatgctcc tgataggcaa ccaaagaggt
219181 gccttttaca ggatgattcc tcaatgattt ttgatcatgt gactgaaata cagaagaaaa
219241 aaataatcac ttcaaaccct ggcctgggct gccgtgtatg tgagcgcaca ccccagattc
219301 ctccctccat cggtgagcac gtgaccttcg tcacatattt tgctgaaacg accacaagtt
219361 ttcatcccgc tgctgtttta cctcctctca cctttgccgg agctccaatg cgcagagcca
219421 aagtgacccg gctttataaa tctgcgctca gcataataag gctgtgttac gattaatgag
219481 atacccaggg cattatttgt tatcttatat atcttattga ttgcattgtc tttcatcttt
219541 gcatgtactc gctctccctg cgactccctc caccgactct tccttccacc cttcggctgt
219601 cggcttcgct ctccctcagt ctcgctcact tgcaccctcc cccgggtccg ccgtcccggc
219661 tttataggag ccgttgaagg ttgaatagtc atggcatgca gagtgcaagc ttgtataact
219721 acgcttccat tacctgcagc gtacacgcgc taataagtca ttttaagtca atgacccgta
219781 catatcaggc ttctttaagg agcgatccag gatcagtgcc catgcaggtt cccccccaca
219841 ggcttattta taacaggctt caaaggctcg atgaggcctc ccacttggaa atgtttgggt
219901 tgaaaccacc tggctgcagg gggggagtga gagctcacta acgtacaggg cagacgtgcg
219961 gatgtatgcg cacgcatgag cgttccttcc gtgcaaatac agccccaagt gttgactgac
220021 ctcgcctcgg ttcccggatc cagagaggga cgcctgaagg gagccggtcg catccggcag
220081 cgcggccgct ggcacgtttc aaacgctgct gatttattaa aggctagctc cgctgtttta
220141 atgaaggcgg ggaggattgg aagtgacaca gcataaacag gaactgtcaa acaattaaac
220201 agtgtaaaca gcacagatga gcctctgatg atactggctg tcgggagcca tttagcaagg
220261 ccgcgccaag tagcctgaga ggtggttctg tctggacacc cgcccatcgg tgacagacct
220321 tctgccaaca aaaatacaat cttagaggcc gttgtacatt tctgctgctg actgttgcat
220381 tcaaagatct ttccatgtcg tttatttgat tacagccagg agaggactag ctgctagcat

220441 ctatttgtgc tacattaact acaatattat aactgcagat gtaaaacgct tcatgtcccg
220501 gctgaaactg tcatctatcg tcatctatct gctataccca atagcaacca ctgggggcac
220561 actgagagtc ttacaaagct agagcatgac taacaaccct ttttggaggg tccggtttca
220621 aggacacgtt ggcttcagga gtagaaccaa ggcccctata agcaaaccag atccaatatg
220681 ggtagggaat aaaaaatgta tgtgttaaac atcatgtgag tgaggttaaa aatactgcca
220741 gaggaataaa tacttgatga atctatgcac attactgcct aatgtacagg ggatgctaga
220801 tatcaactca ttatgcacgt gttatagagc agaatgaagg tttattttga gttctgcatc
220861 gacacagttg ctgacatgtt ttagcgcgtt tcttttttt tctgcttgtt ttcgtgcgtg
220921 acggtaacgc tcagagctgt tttcttgttt gctcagatcc tgcattttat gcagctctgg
220981 aagtgccact cctggttggg aatttgggaa tatgcaaggt aagcactatt actaaggcta
221041 ttaacctcga caccattatg caaagcgaag aggaacggaa gagaaaaaca gagaaagcta
221101 ccagaaatcg tacgaaggga aaaggaaaaa aagaagaatc tgtggtgaga attctgatga
221161 aaagatggtt gatcgttaca gtaaggctat gaatggtgag gactcgggcc cccacacctc
221221 tggtgtgttt atgataaatg agaactacct ctctgttgtg tcagggtccc ttgaccgaca
221281 aatagcaggc taatgacaat ggctagaaca tatggtaatg gcgccgcaca aaactctgct
221341 atcagtgaca cctccaatgt gcggggccgt gattcatctg aactgcacac tggtcctcat
221401 atttactgcc tagtttgtat tgtttgggat gattgagggg ctgattagca ttatgtttac
221461 tgctgggatt attcaccccg acccattgtt gcctcgttga aagggaggcg ttttacggac
221521 gaggatattc tggcactctc tccagctctg acgcagagga gtcaaattta gaggtttggt
221581 tctaattaaa acgggtgatt tcccagactt tctgcaggta ttaagaataa aaatccaaga
221641 ttgtatgcac acacgaactg tgatgggaca tctcccccag tgtgttacga tgattttatc
221701 cattcagtgt atctcccaaa ccttgggatt aactttaatc tttactttaa ttcgacttca
221761 aacatcttaa gtcggacaca tctagcccaa ctctcatcta tactccatct cccagctgac
221821 agtgaccaga atcctgaatc ccctcttaac tgcgtgtgaa atgtagaata gctctcctct
221881 cccctgttct aatccgggcc cgtccagccc cacagtaccg gcctgtgaca gatacgaagg
221941 cgcaaagctg ctaatctcag cgcccctcca gcccgtgggg tcagtgggga gctcctcagc
222001 cctaagcaga gccatcctag gaggccaggg gggaggagat acttgggcct gggccttcag

222061 catcaaacct tagctgtcgg attaatgcac ctacagggac gtgatgttcc tgatgggccg
222121 caggctcact ggatcacagg ctcacaacac atgggactac ggttattaca agagggagca
222181 ggagttgagc gctgtcgcgc tgctagcgtc agagccaccg gagcattttc cacagaggta
222241 gaggctggaa tgacacagcc gctctttgca tagtgacaca atgagtatgt tgtgtttaca
222301 tgctgaacgt gttcgcgtga atttagctcg gaggcagtac gcgaagaagc agcggaggac
222361 tcaaaatgta gcctacatta gccgagagaa acttgttccc gtggcgtccc tgaaggatgc
222421 ggcagtgcac ttttatgttt atgagaaccg gctcaataat taatggtgat aaccatcatc
222481 agcggcccaa atcgattctt atctttgccc gtgttgagcg acgttacatt tttaatgttc
222541 ttgtcatttt gattaggatc atgtcgttgc ctttgtcctc actcataata cgagtcaggg
222601 aggtctgaga gcactttggg tgttaaactg ccacgatcca atgcgtcctt ttagctgcgg
222661 tggagcttct ttgcctgcaa aatgcatctg ttggaaaatt taaaactgga atgcgggaga
222721 agcgtcaaga ctaaatactg tgtgggctca tcgtggcagc ggaggaataa acccgagttc
222781 agctgctctt ctgcctcctt taatagttta gacgccactt aaagcagctc tgatctaatt
222841 tggggaatat gcagctatga agtctacatc ctcacttcag ctatgatgct gcattaataa
222901 aggggggggg gggggggggg acaaatctaa aaatgacttt ggcttccctc tgctgtgagt
222961 acatgtttac aggatccact cacacacaca gtgttggcgg caagtgttaa taaaagcttt
223021 gcattccagc aatatatttt aatccacggc tgtgaaattg acaagctctg ccagcgcata
223081 tttaacaggg acgtggtgcc gagttccttc ttatggaggc aaccgtctct acagccgata
223141 actcctctga cctgaaagat ccgtctgttt gatcagggtg aggttcaccc tctgagcccc
223201 caaccagatc tccagaatag gaggaggatc agccaatgag agcacagctc caacagaata
223261 acaatctaaa actcttcata tttatatatt taatacottt attctgtagc aatgacaaca
223321 cactacacag cgccgcatct taacaacttt ttatccttca cacatttgaa ctcccctcac
223381 ttctacagtt ctgactctgc tgcctgaaca gcgcctacta gctgtgatac ccggtaataa
223441 aagaaaatgt tctgctcaca tgtgcacact tggctctgaa cacacagcag gaaccaggaa
223501 gtgacactaa cacacctcac gctcgcttca aaaccctttc ccaaccagag gcatcacaga
223561 ataagtgtgt gggctaatat tgtcccttaa ttaattgtat tctgctgatt gaacttcctc
223621 ccaaatacct gtttaaaatc attcacacac acacacacac acacacctga gcacatgctg

223681 caagatgagt gttggctggt acacacacac acaaagaata gaaaactgag
ggctcaggca
223741 gccatgatga tgggggatta ggagtggttg gttgtcagca ggatgcatta
cttagtgtgc
223801 gtttgtgtgt gtgtgtgtgt gtgtgtgtgt gtgtgtgtgt gcgtgcgtgt
gcgtgtgcgt
223861 gtgcgtgtgc aagactgagc tcgcatgtag aggcttttcc cggcctaaag
atcgtcctta
223921 caccggaaat cctggaaagc gaattgaaag tggcgaggca taaatatgat
gcccaggtct
223981 gaactcatct gataaatatc agagtgaagt aatttaatat catagttgta
gcaacaaaaa
224041 caaacaaaaa gggctatcaa acacattcat gcccccccca ccaaggatac
agtcagtgct
224101 acagcacagc tgttgaatca cattcatatt taaatattca tttataggtt
aaataaaata
224161 atgtcagcca cacaaagcac ctttgtgagt ctggatcgta accctgattc
atattcataa
224221 accctccacg tgacattttc ccactcgggt gtgatatcct gtgtccacac
agctctcctc
224281 tttagagtgc cttctcatcc agcctctaat tcaaggccca aaatgacagc
agaggtaaat
224341 gttctgttcc taatgctctg aaataatgct aaattataga cggctggttt
tatttgtgtg
224401 aataaatttg ggagcttaat ggcttgtaaa acacatttgc tgagcgtcgc
ctacaaaagt
224461 gtaaatttgt aaatcggtgt ttccagcaaa gtacaattag cattaatttt
tgcgacatgc
224521 aataattaag aacaaaacaa gacagacaaa taaactccct gttgttgaga
caggaaataa
224581 ttcaataaat ggctgatcaa acccactgaa gcaaaatgca gcatgtttct
catttctgcc
224641 taagctataa aggtcaataa ataaataaat ataggctcat ttaggcagat
gatagtaaat
224701 atgtagcata tctgattggg ctctctggaa attctatctg cttctgacag
atgggagaag
224761 gctgcccaca attacacaac acatggccaa gatctgtcca tttgtggaaa
ttaaagctgg
224821 catattctgc agttatctgc cagtgtctga cattaatctg ccaatggagg
gggggggggg
224881 acaaaaaaaa acacatttgg catggcgcat gcttcctgtc tttgagaaca
cagttttatt
224941 tgcttttgat tgacaaagat aatcccgact gattctcaag tcatcctcta
aaaatatcat
225001 cttaaagact gaagaaacac aaatttaatt gactagaacg ttacaggttt
gaactatgac
225061 gacccaaatc ccccaatctc taagtcaacg ccgcgcaagt caacaccttt
ccaacatgtg
225121 gtattgcctt gattcacctt gactcacctt aaagtacagt aaatttgtaa
aattttgatt
225181 tattttatta gggttacgat ttaaatttcc ccaggacaca ccgttagcat
gtagtagcta
225241 agctacagcc atcaaatcta atatgttggc tataaatctt tgtgtttcaa
atgagtacat

225301 gttcagaatg tccgcaggag tggggaatat cagtaccttg aaaatatttg gtaaaaaatt
225361 ctctagtttc ctctttagca ttggtgtgaa taatccaaaa aggctctatc ctgccaccag
225421 aattatccat ccatctcaga aaccaaataa taaaataccc tccaatgaat ttattacctt
225481 ttaattattc agtatagact aaagaaaggc tttaaaggtt catttaaaca tgttagttgg
225541 actaaaattg atgttttaaa cacatccagg cgaggcaact gaacgttgaa taacccctgc
225601 attggtagag agcctggctg acctgtcata cagcaggaaa aaggtaaaca gttgcttcaa
225661 cagaagaagc cacctgaatg tggcaaaaac tgtacttcta atgtggtcgc gccagttgac
225721 ctctgtgatg ctaacttgct agctctctat taactaccgt tgtggtctgt gatgtcaaag
225781 tgcaactgga aaaggcccaa tacttacaag aagaatgctc ctatagggga ggtgacatga
225841 tttcaagaag gaataagttg catccctctc catttatact ggggcaaaca aaatagtcca
225901 accaaaccgc tacacccata gctcaactgg ctgtgcctgt gtcactcaga atcagtgtca
225961 tgcctagaag gcacttaaac agtttaaaaa gtttaaagtt gcataatggc agatccagag
226021 acagacaaac cttcttttta atgggacctg atcagccatt ggttgactcc aaatgcatct
226081 gtcacgagac cccctaacca gaagaagcga acgtttatac cagcactgct ctggctgcac
226141 gatatccccc acaaaaatag caagcagcca tctgcttcct gtttcaagcc cagtgaatgt
226201 aaatgaatga tggtaattgg acagatttcc actggagtga aaacggacct gaaaccacaa
226261 atatgaaaca gcgctgttgt ttgaacgtca cgcgagaaag ttctgcagca tattgggatt
226321 gtcaattatg tacatcaaga ctgatatgag aatcatctgt caatttatca gcattacaga
226381 gtccgacatg ctaaatgcgc gcggttgtcg gcggcgacag atcagaacag gccggcccgc
226441 tcgactcgat caagattgag cagagcaata atccaaatcc tctggacaca acacctgcca
226501 gcaccgctga gatcccagtg tgagaaagat gaaggctctc tagagttggg gtaatatggt
226561 gtcaagaccc cctccccgct ctccacacgc agggaggggg gggtatcagg gccacaagca
226621 gtcctttaat tttcagttaa tgattgaaca aacaaacctc gcctcagagc tgtgccctct
226681 gatgggtgtg gctgcccccc cggcgtgggg gcaggtgccc tctaataagc atcctcgaga
226741 aaagaaaaaa aataaaaaga gcccaatatt tgctcagcaa ctggagacgg aggagccgtt
226801 ggggagcagg atattggttg gctatgacaa tgcacaagaa ggccgagctt gacagggagg
226861 gggctgaggg gtgaaaaagg agggggtct actaaaggag gagcagaagg gaggggatga

226921 agtggtggtg agtggagatg gttgggtagg gggccttttc tgccacatat agtactatga
226981 ggaacagatg gggcctgata aatcctgaaa gggaggcatt acctgcccaa gtcaccaagc
227041 agccacaatt atcacagggc tctgggttca ttgtcaatta gagtggcagt taaagccagg
227101 cagcagctgt ggcagatggc tgggatccag gaaggctggg ggagccggga cagaggccag
227161 cccccaggct ctctgcatct cctcctactt ttctctccac atctccgtct cttcacgggc
227221 ccggggtctg tggtggagaa ccttctcacc gggcctgggg aaaagatatg acgaggcaga
227281 gcggagaacg cgccaggaaa gattactgga gaggcagccc tgtttgttta tttcacaaaa
227341 agtgcattaa aacagcataa aagtgcagga atgatttatc ttgtgcacag atatggtcac
227401 agctatacat agaatggtgc ctatgttgta ggaagcctgt gctttagtaa aaatagcaac
227461 caaatatatt cagtaaatcc ataaataagt caagactcac tagcttgcac gatagcagca
227521 actatcagtt gttgacgttt gtacatttgc attaattaga ctctgattgt gccacaaacc
227581 acttgttgct ggaaataata aaaggaggaa catcataatt taaaaaaaac aacattatat
227641 caaggcggaa gaggaggagg aagggcatat gtgtcttcac tgaagctgct ctacagtgct
227701 cctagtggtc gtcattggat ttcaccataa caagacgcgc agcatgccca tgttatagac
227761 tgcacagcat tgtcacaata cggagattca gcctttaaat ttttatattt ggtgttgtaa
227821 ttttctctcg agcatttctg acaagttatt tacacggaca ggcggttcac atgcgctcct
227881 aaagtcttaa gtcccggctc ctgctgtgtt tttgtcgtcc cctcacctgt tttgcaccag
227941 cagccctctc tgtgaagcat acatgggcga ctgaaggaca gactttcttt ttttatgctg
228001 agtttgtctc tgtctgcctc ccagcctgag ccaaagctca ccagcaggct tcactttcag
228061 cgagtaccac cattcatcac caggcagggg tgcggggagg ggtttgggtc gggggtacg
228121 gcagggggcc agtaatgggg cagagtgtgc ccctcacccc tccttttctt ctccacaggc
228181 tgctgagaat acaggcctct gccacagaca gcagccacac tcctctctta caacctctac
228241 aatggagctc caaatttatt caggggaggc gcgacctgct cccttctctt gctcccttgc
228301 tccttcaccc ttgtctcttg cccctcctat tcagctcttt tcttccctcc ttgtcaccct
228361 ctttccttgg ctcttctccc ttaccctcaa cccctctgcc ttggtccctc tcctctcccc
228421 attggggcat cattcctcct actttgttcc actagtcctg gcccagtgtc tgggctgtcc
228481 tgatagcaaa gaagaagtag cagcctatcc atctggctgc cttgggcttg ccaatggaaa

228541 aaaaagagga gcagaggtcc tcattcataa caatagccat taacaatgct aacagcagtg
228601 ctgttgctgc tgtagagact ggggagcgaa aggttgcagg gactgggaca agggactacg
228661 tgcactggga ctgctgtggc cagacagtaa agtatgagcg gggggccagg gagggcttgt
228721 cgccaggcag agttggagat cgcagagatc caataggttc acaggccgtg cggctctcta
228781 aagcatgttg taggggcgtt agacgagccc tccgagtctc cgaggcctgc aaagaaatat
228841 tgatttctct tttttcttgc ccatggccag acagtgtgtg atgccccgtc catgtgaagc
228901 tagctctcct ctcacagcgc ccgggtcatt aaagagtctg tggctcagcg cgactcactt
228961 ccaagcgtgg cggagtggtt ttgttgtttt gaagaatgct ctcggattta acaaaataat
229021 acctaaggtt cgaacacgcg cgatagtccg aagtgcgtgg tggatggaag cgacagttcc
229081 ctgcatcagc agaaacacag ctcagtgtgg gtaaactcac tgacagtcgc acaatagctg
229141 taatgatgcc cgagtagacc agatctacct ccggtacttt cccggcttcc ctcctgagtt
229201 ttgacacatt ttcccattta tcagggatgc ctatctgtct ccagtaacaa caaaaggcgc
229261 aacaccgcat ggagcctcct gtgaaatggt tacagtagac cctccctccg cgggctcctc
229321 attcctccaa tcaagctgtc aaaaaagcac agtcctttgt attgagtgtt ttaattagat
229381 ttggatagat ttcaaatctg ctcttgtttc ctggaatgat tcggagtgaa agaagaaacc
229441 ctgcaacaag attaagcgct aatctatcct ttcattgaag ctcctcaaat ctgtccatct
229501 gcccgaccat tcaaacgcag aggagcttct gaagatatta accttttgag cattctcttt
229561 gaattcgcct cataatggac ccctgcccct aaaaacaaac acaaatcagg tgcgagccaa
229621 aactttggga ttctcttccc cccactgtcc agactcttgt gggtataact tgattaatca
229681 gatatatata taattctcct attcctccca catgcaggat gggatctgca cttgctcctc
229741 tcaacatgct tccatcaata caattgaatt tgtccacagg gcagtgaaat ttactgtatg
229801 ccatcaatga atttgatcaa taccctcccg ttatggcaac ttattctgtg tggcacaatc
229861 aatatcacac atcgtcaacg ccgccgtctc ccagactaat aatcttcaga atccaatatg
229921 gacttgaacc gtctcttccc aatgactcag aaaaaaagtg tgaggttatg caaaggtgtg
229981 gacggccctc aatccccccc ccaacagcaa ttacagtgga taaacaactt gatggctcac
230041 aaggaaattg cccggaaagc tgacacagac tatgcataat ttgctgacaa aattgtcaat
230101 gagttaatga tcacggacaa ttggcagagt cgccgcgctt taactccgct ctgcctccat

230161 ttgtgttggc gcaagcggct gtccctttc tctccccttc tctcccctt
gatgggagca
230221 aaaacaccaa caaagaagta agtgacaatc ccagagaggg ggggatacct
ttatttgatg
230281 tatgaattca cctcgactaa aatcagtgca gtttggagac tcagttagct
cttttagcaa
230341 tagcatgaag tggacaaatc acagaataga gaatgattac agcaagaaaa
cagtgagtaa
230401 aatgatacac aacaaaagaa cacaatcagt gattatcaaa gagccatggc
tgttagtaag
230461 gttcagccat gcattaaaac agtgcaggtg tctgcgttgg tgggggcctg
ttgcttaagt
230521 tacccaggag agtctgaaac tcaagccaga aaatccagcc ggagtgacag
attaaagagt
230581 ttaagcctta aacgcaccaa aacgctgctc aacattaagc aacggccaca
gcaggttttt
230641 actttgctca tagaatcggt ttaaagcaga ttactgtttt actgtccata
agaccaaagt
230701 atcactattg gaaatataga gaattgtgtg tagagcctgt taacctgtgc
attcttcttt
230761 ctcagtggtt catgatgggt tcatgatgag ttgaattttg cttttattta
tattgaattt
230821 cagaaggaaa ataggtttaa tcatatagat aacacaaaat tagcaatttt
ccagttcaat
230881 tcctaatgag ttagaatagg aggaacacct cagacgcctc tgactactct
agggaagtta
230941 aatgcatcag cagccgagat ctgagagtct ttaaacaata accagttcct
tgtgcttcaa
231001 atccatgtga gacagtaaca aagagaaatt ccagtcgtgt ttatgtagac
atgccgcaga
231061 cccccctggc ccaacttgtg ggaggtttgt ggtgatctgc atgtgattgc
agatcaccac
231121 caacgtgtgt tttccggcag aaaaaaccct taaactcaag atgtgaggag
atttctaagg
231181 acgtgtgaga aataatggaa gaagcttgtt tacaggtgac atcagctcac
atcaggtgtg
231241 ttttcagaga cagatgaaat gccaacagta caagcagatt attttctttt
caaataacta
231301 gagatagttt taattccttg ctggcatcgc cactgtaaac cccatcgcgg
acccctgagg
231361 aacagcaaaa cgtttccatc agaatctgag cagtgttcag aaggactgca
ggctggaccc
231421 ccgtcgctct ggtgaatcac agcagactga ggacaatcta caacagacga
gtcctggctg
231481 cttatatata gaaaaggatg gctgccaaga gaaatcttat tgcttttaga
ttattatggc
231541 agctgatatt tttggaccgt tgtggtcttt agggaaaaaa tcaatatctg
tccttttacc
231601 tttgcaagcc cccccccct ccccccttccc aatccaggac cccttccact
tctcatccag
231661 gtttacggtt taatagatgc tctgtagaaa taatataata ccgcttgact
aaaagacacc
231721 aaaatagaca acaaaaatta agctttttcc aacaataaag tggataatgc
tgccgtattg

231781 acgtacaccc atttatgttg ctgctgcaaa tccccgtaaa ttgactctaa gtcagctttt
231841 cggttcagat gggggcttag tgggggcctg accccgtcat acatgcatga ctcaaggaga
231901 catggtccaa atagctcctg agtgtctgcc tgtgtgtcct tccactcttt gcccttttt
231961 tattgggtta tcgctgcagc atgcataatt taatgttgcc gcttcattta aaatgagatg
232021 aaaacatgta aaaacagtga ccctgcccgg agagggcgca gcattcatca caccttcatt
232081 ttgctcgtcc cttcttttga aaacacccga cccctggaac ctctaaaaac caaaataaaa
232141 gaaaaaaaaa ccccaccctg cctccaggta aaatcaaacc tacatatata aaaatggctt
232201 ttcctcacat tcttccccct gcattggatt ttatgcagcg gctgacacga ctcaataact
232261 gaaagctctt gatacatgtc agaaagcgtg aactctgtct gctgataatg aagcaggtga
232321 ctgttgtatg gctccggaat ggtctccaat gcacacttta atgacatgtg tgcgtgcacg
232381 ctcacgcatg tgtgcgctgt gtgtgtcacc cttgtgtggt gctttttttcg caaggctcct
232441 gtcaggtccg taattgacag tctaagtggg gcagccgaga ggccaggccc tgttaattta
232501 ttttctcaga ctctccatcc atatttatga gcccctgctg ttttgtctat caccaccgat
232561 caaactcagt gcgtaattgt gctcgctaaa gcaagagagc cacgtgtgag tggtcactct
232621 gccccacagc acccggcatt agtctttatt atttcattaa tatcttcaga ccgggaggga
232681 gagagcaaag gaaggagaat tagaaagatg ctggtggcgc cgatgattag ggatcaggag
232741 ccgaatgagc cgcctgattc aggtcattat gacaacagcc tctgatctgg ctcaaactaa
232801 ttgcagcggc tcctgtgggt gagggtcccg tctcctttta gccgtaatga ccgcgtccat
232861 cacgatgcgg cgcaagccca ttcatcacga ggtgtcatta acgcaccgat tttctaaaca
232921 caataaaccc ccccaacttg gggggaaaaa tttggaccgc cgataatcag tgccgtgcat
232981 catctcgctt tagtgcgaga tcacctgctg tgcctttacg tccaacgaca ttgcgatgaa
233041 ttgcattgtg ggaagatggc gtcggccgtg agcgaggctc aaggcatgtt gctgtttacc
233101 tccagtcgac cctctttaat gaatttaatt tgtttacaaa gtaacctcca ggaaatattt
233161 gctggcgaga cctgggtgtt tgaaggtgtt atgcgagggc atacctgcgt cgtcgggggt
233221 aatcacgtaa ttatgagcta ttttgatccc ataaacatac attcatttcc attcattacc
233281 acttgttatg ctttcatcta aacagcggcg cagggcgacg caggcgggca taatgggggt
233341 tagcgtgagg ggccgggtgt cagtcggctc tggattctgt cttaaccttc attaaaagaa

233401 gttgagcctc ttctaccttt tcaactaaag tttcctattg tttgattgat tttgcattga
233461 ttatttccct ctgtcggagc tgtgagagaa atatttattc actattagga atttgctttc
233521 cacattaggc cctcaaactc gccggtgcgg acttaacaag cagcacttaa tgcacgccgc
233581 agaagtcatg taagtcactc atttactcgg gctcaaaagc tttaatttgc atgttgttgt
233641 tggggttttt tatccaccct cactttttat tcaccctcac cctcactccc cacaacccca
233701 catcttcaaa ccctgatgga gtgcggccac taatggctca gttgtgagtt ataaaacaca
233761 aatgaattct cttcgctcca aatgaaactt gctctgatgc gggctcccag catgctttga
233821 tgattatagg agccctcccc atgaaattgt ctttgtaatt gtctcaccgc tcgtggaaag
233881 ctcagatact tccctaataa gccaaactga ggaggcaatt tgacaatctc gagtcaaaat
233941 ggccccactt tcacctctaa atttcaaact cccacagctt cgttggagtg aaccactgca
234001 acatattcta tatttcatta cgtacaagat atcaaatgac gctttttggg gctcctgagc
234061 agcattgatt atgcgatgcc ggtttgttta cttcgagtaa ttttggactt ctggaatgtg
234121 tctcagctga agcgactcct gaggtcgaac ctgcgaggcc tgattaatcg gcgcgccgtg
234181 ttttctcccc tacttgaacc caaggccgag ctatatttca cagaaagcga ttgagcggtg
234241 gactgacacg ggcctgacca taaagccaaa ggcaattagg aacctctgaa agatgctccg
234301 tgaaaaaaaa caggtcagac atattcaaca gccgctgctc atgacaaaca gaggcgaggc
234361 tttagtctgc gcgggagcgg ctcacagagg gaaagaacgc tcttgggggg ggcgtgttgc
234421 gcattactgt gttgcaagtg tgcaaatttt gataaataac accttcgcat cagtgcggta
234481 atttgatttc ctgagagata atcgaaacta attgacacaa gaattgagcg agcgaggcgc
234541 tgatgctaaa gcgtcctggc agagttttca cagcaagatg ttcccgaagt gtcagggata
234601 tatcacgact tgcagcgctc ggtgacagtt tattggaggc agctttcctc tctcatccaa
234661 taacgggccg tcttcattac gctgcgaggg ccactcgcgc ttcctgatgg gctccgctcc
234721 tgaccacacc ttcaatcttt gttgggatgt aaaactaaat aagtgcaaaa caagatgtgt
234781 tggagcacca aagtctggaa agcattcaaa ctgaagcagt cacacatcag aagaactcca
234841 gttttgcacc tgttagcccg ggtgctaagg acaattgtat taaataatca aattataaca
234901 caaactgata cttctgtcct aaaaagagcc cccccggggg tcttttcctg taactgcagt
234961 cctataaaat ccccattgca aaaagtcatc acattacatc atcgataaga ggttgttttt

235021 actacactgt ctttggttcc atttcattct aaatatagag aactgtgaaa cttccttttt
235081 taagccattt ttatgtttta cagtgagaga agaagagtcc cataatttgc tgtaagcaat
235141 taagtcagtg attaagagga aacaacatta aatctatatt taggtttaca gtttggctgt
235201 tggacaggat ttactactgg ctgccactcc catagctgcc attcccagat gcattagcta
235261 ttggctgcca ctcagccatt cacagatgtg aaaactggca aatgtcaaca caaaagatcg
235321 aaaaagtgtt gcattatttt agaaacaagc cacattttac agttttaaaa gggatttttt
235381 ttttgcttgc tttttttcag ttactgtata tcaccgggta tatcagggtt taggctccgt
235441 ggcagtcggg cagaccttcc agatggcatc ggcagcatat gtggcatgac cttccacagc
235501 gcgggtacac actggggggt ggggaagggt gtaccgagtc accgtgcaaa cagcaggcgg
235561 agagctcaga tgtgggtgac cccccgggga aaaggcggca gaatgagatg cccatcactc
235621 tgtgggtctg cagatgctcc tcacaactgc caagctttca gcttcattag accaagatca
235681 gatacataca gaggagtgca gcctgaggct atcgctgcag cctccccgcc atgcaggcgc
235741 acacacacac acacacacac acacacacac acacacggtc agattcacat ttggaataat
235801 ttctaaaaaa aggcagaaaa gatgtgatgg ccaagaactg ctttcttctc cggatttggg
235861 ttctgaacgt gagcaggctc gataaaactg tcacagcatg aaaggagtca gggagcgtgt
235921 gcgaattcac acatctttca atgaacacag cgaggaaata tgatgggaaa tgagtgatac
235981 cgcgacgaca tcgatgctgg ggagctttat ttagtaaatt attgatacca aacacggaga
236041 agaaggggac gagcaggggg acaaatctag agaaatggcc gggatcagat tcttactccg
236101 agtcattatc ttctttactc cagacccagc ttgattgata aagatcagct ttttaccccc
236161 tcacttccct gcgagcagtc ggacttttat tcgtcaccag ccgagacatt tccccctctg
236221 cgcgatatgt aattagtgtg taatcccctg ccttttttaaa atgagaccaa aaaatgatga
236281 cgcggaaccg taatgagata ttaattcaga aacactgcag ataggcattt attttcttcc
236341 cttttttttaa tcttctcacc tcaataaagc aatgctgcat aagttattac aaagctttaa
236401 tgtggacgtg cttattacag caatttgtat gggcaaccag tgattcattt ccgcttctgt
236461 ctttttctca ttatggcctc aatccctatc tgtcgctcct ctgtttattc ggaataactt
236521 attcgagtaa ggacataaag aagatagcag aagaaggagc gccggggatg ctaattttag
236581 gcgccattct tgccaaaaat gcacatttgt gtccagccta gcgcgctgaa cgttgtcatg

236641 tgagtccaat ctccgccgct tggctccgtc atgtaagatg tgtcccaggt
gatggaagca
236701 ggtggtccat aatttagccc cctccgttcc cccagctacc attgctgttc
tgccccactg
236761 agacggggaa ccgtctcctc gcagtgtgtc acggcgcccc cgagatggaa
cgccggaatt
236821 aggcagaaat taccctgact ggctgccgct ttccactgag gatcagaaac
ctgagcgagc
236881 agaggggcat tcgtccaaac gtgattaatg tgaaatcgca tttgaataat
tgcgtttaaa
236941 tatttggacg atcgatcaaa aaaaaaaaaa aatgaagcag tgacaattca
tctcagctgg
237001 aattcttctc aggaatcccg ctcgacagtc atcgctttga atgcgaaacg
taatctttaa
237061 ccggatggcc tccagcgtgg cggcggcgct attaaattat tacacaggcg
cgacattgtt
237121 gctaactgaa aagctcacat tattttcttc attcatcaaa atgtcagaaa
gcctctgtgg
237181 tacctcggaa aataaaaata agcagacttt cttccaaaac acatttctat
ccaacacgtg
237241 gtccagtcaa atacaaaatc acttctcaca atagttttcc acattggaaa
cgctgctttc
237301 caccattgca tcggtgttag cgcgcacgtg atgtcgccat tgccagcctc
atagctcaca
237361 tgagaaaggt actctcagat tgcatttttg gcactttgtc aggagtgtaa
ttgagcatgt
237421 gtgttttggt ggcagcggcc atgtgtgtgc gtgtgtgtgt gcgtgcgtgt
gcgtgcgtgc
237481 gtgcggcggt ggtgggggtc atcccgttgc ctggtaacaa gcctcagatt
gacaaaggaa
237541 gagcaattta ggactacttt ctccagaaat caatacgaca ggcctgatgt
gatgatgtga
237601 gattagccga gggagggaaa gaaaaaaaac aatgtgtttg gcagaggtta
ctttataacc
237661 aggaggagga cgtggaggag attgcatctg aaacatggaa tataaagata
taaggtgggt
237721 gcctacagta gcatctgggc tcaaaatgag acttctccca ccgtggcacc
tgccttcggg
237781 taatgagagt tttagcaaag tgaacactcg tggggaggta aaaaatccga
ttgtagcttt
237841 cttgtttaat atcgtctgtc aggctgtcac agaatatcgc cctgggtgag
ggaatacact
237901 gtcgggctgt gatatgagaa aagatttgag tgagagagaa atggcagtta
aatagaactt
237961 taggatggcg actcccttca tgcataaacc attctcggtg cctcccgctg
aaccagagag
238021 gttagaaatc actgatgtcg agggggaaaa aaatcatctt ttatgccgag
aatacgatgc
238081 acggccccat tagacgtaaa cgaggggtac gtcaggaact gtttcactta
aatatgaaaa
238141 gttcaagaca acttcttcac ttttcaggga aaaaaaaccc ttccagcaca
gctagaccat
238201 attagtttag gagtttatgc atgtaggaat tcacctcagc tgtaaagagc
gcagctttta

238261 ggcactttta acccttttggg ggtctcagag tgggtgtaag agcctgttcc
agctgcacac
238321 agatacaccc ctgaaagagt cgcccgctca tcatcacggg agcctatgtg
gccattaggg
238381 ggtttgttac cttgctcaag cattcctcac cagtgctgtg aaggtgttgg
cagcgctcca
238441 gctaccagaa gaccacccaa gttttgtcca cacagggact tgaaccaagg
accttccact
238501 tcacagccca gtccccttca gactggtttt tactccattg gggataatc
atggtcaaac
238561 tgagcctcgg aatagggaag gagggtgaag gtgcaggatt gcggtgcaca
atctggcagc
238621 gattgtttgg gggaataaaa tgcggccctc agctttccgc tgcgttttcg
tgctgtttct
238681 cttcgcttat gacagcggtc aaaaacaact cctgcgggaa ccattcgctc
ctaagtgctg
238741 cattatgttc atcatccagt ctcggcattt gtcggcacgc aaagtgccgc
cgatgtgttt
238801 atctgggcat tttaacttaa tacagccaca cgctgtggct gcaaaaacag
gcttcccgaa
238861 tgttcacagc acctgttgta attctctgcc tttgagctca ggttgagatc
ttgaaaaata
238921 actatactga aactttattt atttggttgc atttaaatat gttttttgc
agcaaaggat
238981 tttgctctta gcactttttt ttaaaatctg tgcttctttg ttgttgcagg
aaatggcaca
239041 atgccccatt cagcccagca aagttggatg ctagcgcgaa acctgttcca
ttcaaggaat
239101 tttatcgagg gaagagtgga gtaacctgga gatcgtaacc tttttcttgc
ccctgctcta
239161 agcagggttt ccctgattta cagtaggtgt ttgtgcaaac ggtctgtccg
gaggcgttcg
239221 ggttagctga actggaaact ataaattgcc tgggattgtg aatataaatg
caaaattgtt
239281 tcccttgtct gcgcttgagc tctgcaagag gctggaaact agcatgtgtc
ctccggtgca
239341 tgctgggata ggctcaataa agcagctgtg accctgaaca aaatgtgcgg
tcgtaaatgg
239401 atggaggact tttgtctaac aaccttgtac agttttcaga cattaacctg
atcagtttct
239461 aaaaacgtgc tctattaagc taaaaacaca tttctgcata tttgtgtttg
tgtttgtgtg
239521 tgaaagggat tgaaaaaaac aattaggagt aagctagcat gaaaacatca
taaaatgccg
239581 ccacaattac tgcacgatcg gaatgatagt tagcgtcgga cagtcgctat
atttaacctt
239641 ttataccagc atgattaata ataattgtgt ctgcataaaa aggctgagag
gaatggagac
239701 acaatgagga atctgcctga tttgggttga aaataagaga tgacggatga
gggaaacatt
239761 cacacagacc tttctcctgc tacataaacc tgtgtaacca gagccctgac
aggctggaca
239821 gtggaagctc ttgatgggca ggaatgtgga ccccgagcgc acagagagca
gattgatgga

239881 gaccgagagc ggggaggaat gtatcgactc ccccggccag tgggaacaac cgcgggaggg
240941 gatcaccatg acctgcgctg acacctctgt catcgcacgt cccctgtctc cagccggaga
240001 aaagaggggt aatggggaga tggagagact ggaggagtgt gagaaggaaa ggaggtaagc
240061 agcgaggggg tgaggtgggg tgaggtgggt ccccttgcct gacagcagga ggcggactgt
240121 ggagacatcc cagtggagaa atccgcttct tatgtagccg accctgcagg ggcttcctac
240181 acgcctgtta ccgagggggg gaggacagag aaaaaccctg gagacaatct gtcaacaaca
240241 tcaacacaaa ggaaaggaaa taggtgatgt caagagacgg ccttcaaaga tgtccctcgc
240301 ctaatattca tcgatatttg gtgtagaagt ttaaggtgag aaacggagaa ggtgagccat
240361 cacttcagaa acctgccagc tcagcaggtc ggcagcagcg ttcggtgtca gccgcatatg
240421 caatcagccc tcataacaag tccataatca atgcattgat tagtgcctgg aaggaagtca
240481 ggcagaaaca gtcacccaga ataaatgaac taccatgact ttgtgcctgc cttccctaca
240541 caatgatgag gcaattgcct ttaataagcc ctccctctct ctccctctct gtcatttacc
240601 agtgtctgca tcattaggct gctggtaccg gccccctgga ctgatgtgtc tgtgtcagga
240661 cctgccgcat tagaccactg ctcaggtgcc ataattcacc tccgtatggg cagcaggtca
240721 atggagagat gaggcctggc ggccattagc tactcagagg aagacagcca ggttgtgtcg
240781 gggtcagcgc gggtcagcat tgttaagcag aatacagcag aatcgaagga atgaagggtg
240841 ctggttgggg ggaatgatgc tattaaaatg aaaggaactg cgatcgaaaa gtgtggaggc
240901 tcgttacgat gcttaagctg gttgtctgga agcggcgggg tcggcgtgtt gttgtcgccg
240961 tgaggcgtca aaacgagaaa cgctgacaga cgagacgatt cggcccagat ttagaacgct
241021 catattttaa ggttgaaaaa agaagtgttg ggatcaatat tctgccgctg tctgtttact
241081 gtcagtgaca tcattcacct tttcgctgtc tggaaattcg atcagctcat ccagtcgtgc
241141 tctaaaagag aaacggccca ttgaagggga gcgcgcatcg gacttggctg gcaaatcccg
241201 tgactcaaat atctgttagc gcactgcttc ctggcagagt tacatcagct ttggcaccag
241261 gcaggcacac acacacacac gcacacacac acacacacac acacattagt cagcagtgtt
241321 cactctccag ccactgcagc gcatccggtg gctcactgat aagctgataa ctcatgctga
241381 gcagcattta ttgcaatctg ggtcagttgt ataatgtgga aatactgtta gggttttata
241441 taataatatt caatatgtta ttatattaca ataacaaaca gcagcattaa aacacaaagg

241501 acatttaaaa gaacattaaa tgatgataag ttatgacatc acataagagg
gtactttaag
241561 aagcttagga acaaccagaa cggccccacc caaggatccc aggctggctc
aacaattctc
241621 ttaaaaggct tgacgtcagc cgcatacgag ctttaaaaga aaaatcttaa
aatcaatttt
241681 aaaatagtca agcagccagt ggaggggggc caggatgagc actgatgtca
taatgtttgg
241741 aaggactcgc taattttgaa ctaccgtagt tgtaggcaag ataggaatat
ttggaagata
241801 ctggactgag gtgcagtagt ctagccttga taaaagcaca ggtgagttct
acaacaatag
241861 ataaatgggc aaaagattga aggggtgaag actttgttat ggactaaaca
ccaggattcc
241921 agtttttaaa tgaagctaca agttatgagc catccacctg caccaattca
gaccaagcca
241981 ccaggtgtta gcagaaggta catgcatcag ttgtatgtgt aacaactgac
aacgttcact
242041 gacacagatg ttgacaaacc cttttcgaac tgcgtttcat gtcacactca
ggtgccggca
242101 gatttggcca attagttaat gtctgtgttg acggcgaggt ggaacaccag
gagggaaatc
242161 tgctgtgtgt aatctatagc gggacgtgcg acacatcggg gtgttatcag
cgacgcagcg
242221 ggggatgaat tctgccattt tctctccacg gggccacgac agagtgctcc
tttctaggac
242281 tccctgaggt gggagagaag gagggtgggg acactggaga agtgtggaga
aagggaatgg
242341 gttgactaaa agctgctgat accttagagt agtttgctgt tggagatggc
tcagcggatg
242401 atggaaaaac aagccaaata ggagactaaa gggtcctgcc tgcacaacca
gtagcagttc
242461 agtgagaggc tgcattttgg aggagaacag gagcattttt attcttgggc
agcgtttccg
242521 attagcttcc aaagcctgaa ccaccaaaga aaaagcctgt gtagaatgac
agggctcaat
242581 accgtttgcg gacataaaca acagctgtac gttcttgcga gcggcagcat
ttttcataat
242641 ttcgcccgcg gtgacatatg ttcgttttta cgtgatccac tggaacagaa
accagcctgc
242701 gttgcatttc cttaaatgaa tcatcaaaat tgttgcctcc ccctcagaaa
atgcaattct
242761 gtgtcaggat cccgacagct cctaataatt caacttcaca aagtaatgcc
taaataatta
242821 cacattctaa ttacgatggt agaggagtgc gatgcatcct ttttttaatg
ctatgctaat
242881 tagcatattt tcattatctc gctgaattaa taattcaggg ggtctgtggt
agacatgaat
242941 tattggatta cagtgcaatg atggatttaa aggggcacta cactcaattt
gcatctcaat
243001 ccctcccaag atgacatgaa taattagcag aagaactctc ctcctgcgat
cttttcaaat
243061 catgaaaata tacaagttgg taaagttgta tgacacagta ttactgcgtt
gttagcatta

243121 gcttgttgcg acttgtatca ttttcgccca agcgctcatg cgtttccatt acatttcgtg
243181 tcacttcgga tccccgtttc tctttttcct agtttctgtc tcaaatttac gtcccgcttt
243241 ctcagccggt catctccggt ccggttctaa acagactctg tcagggcgag atgaaaataa
243301 aatgattcat gctggccccc tccccacttg ttcaaagtaa tgagattcca tatttcagcc
243361 agcttgacag atggattgga aattaaaagc cttagataag taatgccttt ttgatgtcgg
243421 actgtgaata actcacactg gttgattgat ttcaaaactc cggtgacaaa gtgcacttgc
243481 tgtacaggag acaacatgcc catacccctaa tatgtcacac attaggagag caatacatta
243541 gaataccata ggttcgatac tgcaagtgct gcctcgccca gaggagaagc aattattgca
243601 ttttgtagga gtcggtgttt cactagcata gtgcgagtgg tgtaaaccac acagctggaa
243661 ttgctggagg agtttgacag aagtagaaga acatcagtga tatcgctttt gaatgtgcac
243721 atttacctgt gtgcttttcc aaaactggag cattccccaa tatgcgcttc tctctcatta
243781 cagcgcctct tcctgccaaa tgcacatttg gagaattgaa tcaatatcgg ataacagctt
243841 cagctcactt actttgcatt tttgttcatg gtctgcttat ggatgagtcc aaaccctcat
243901 tctttcatcc cggtcatgca ggttacatga attccgcctt tatgaccctt gttctccgct
243961 ctgctttcat tatatcagcg cctgcgctca atttgcgaga acagacagct gtctattgct
244021 ctgtgggaga cagagtgaaa atatcgaact ggctcgttcc acaatgagac tacagtgcca
244081 acataacagt gattcatcaa aaaagtgctg gatgggcaga gtcctggctc cactttctat
244141 ttttcatcaa atagtttcag tcataagaag taaagactga gtgactggtg tcaatggtaa
244201 tccattggta tccaatggta tctcgcctga tactgagata cagaatttta aacatgacgg
244261 aatgactgat tatgatagag aggttgtatt gattggaact gcgagatgca aacaattttc
244321 tccacgatca gagcaaactg acaccgcctg agtgaatttg gagagactcc ggaggacttc
244381 aaacgcctaa ttttcaattc ttgttgtaaa gaaatacgct tttctctgtc tcaaatgtgc
244441 taattttgcc ctttctgcaa ctggaaccta gctaacctta cgcagatgtt gtctgattca
244501 catctgtcat aaaatttaac tacaagttgc gtcaacttgg aatgcaacag gtatgattat
244561 cctccttttg gcatggattt cctcgcggcc agtcccaatc ccgttcctgc tttctcgtgg
244621 gccaagtcag gagagagtga tcaaataatt atgcaaatct aaatgtttgt gtgagtcttc
244681 actaaagcat cacaaacatg ccagatggct gcaaatccag gggaaagcaa tttcccaaaa

244741 aattgattgc gcatcatcaa gtgcataaag taaatccaga gtggtacaaa gccaccagtg
244801 agtgaaaacg ccccctacat gtgtgagcag cagcgtaggg acctcgttag agccatggca
244861 cgtaaaataa cgtaaatcct ggttaaggca aaaattccga gtaaatgtac attctcaaga
244921 ggatcccacc gtcccctgtg atgcacattg gttgagctaa gacaccaaaa taaaatacaa
244981 ttctatttga gtcaacaaat gtgatgtgct cccccccaaa aaaattgaga cagttgtgaa
245041 tatgctaaat atatagacgg atatatagaa ataaataaat caagatgttt tcctggaact
245101 gcctgccacg attatctctt tctcgtgtct cagagaaaca gtgggcactc tcattaggcc
245161 cggggaagac atctagttat tcatcaccca ctccatgcaa aaccctgcta ttagaactct
245221 aaaagccatt agagaaatcc ataatgaacc ccaccagtgg caggtgcaca tgggggactg
245281 gcactcggag atgacacaaa caacgtgaca gtttgcgact ccatcggcca ttagcatcct
245341 cgccgccgcc gccgccgtta taatctgcgg gttttcagca gtcacttgca ggtttttgat
245401 tttttttct ttaaacattc cttcggaaca ggctgctggg tttggaaaag ggaacatggt
245461 gcaacagtac tgacagttcg gattaggatc ggctaaatgt ttattagggt agatcgatac
245521 ggcctgaaga actgagcaat gggcttgata ttagtctccc ttcactttac cgcttctgac
245581 ttcccctgtc tttttttttt cccatttgct gttggaccca aagctgcagg atagattctt
245641 ctgttgtaac gtggactgat ctgtgaattt cgtccgaggc taagtcagca aacctctctg
245701 ctggattact aaagaagaac aaatgcaaac cagcaggatg gaaaaagaac acgatgttgc
245761 tggttttctt gggtcccggc tccccgtcat tataacctta gtcatacttt agacttcctc
245821 tccttttatc tttgatacat tccatctgtt cctcatcatg tgaaacctct gtgaaaatac
245881 agtaacaggc cacatcatgt acaacgcgta gctctggcac atatttctag gaacatgttg
245941 tctaataata tattattttc ctgagttttg cgcctggatt gtatttcaaa gcaagtaatc
246001 ctcaaaacta atctggaaaa aatgagagtg agaggtggaa ggtgcacaca gttcatgcaa
246061 cctggaccga gcaagtgcat tgacccagtg gcagacgggc gccacaggta actccccggc
246121 ctgcaaacct tccatgtcag aacacgctgg tcatgctgac ctcatgggag aacaggggtc
246181 gatgccgact gacccctaaa ttccccatca aacacaaaaa caaaacgatc tgcctcacgc
246241 gagggtcgcg gttgacgcca aaaggtgccg ctgcctttag cccacttcaa cctgcgaggt
246301 gtgtaagtgg aaggaggact gatccgtggg gagagcaggt gggggggct catgaaatga

246361 agtatgtcca ggctggctgt gattgcccca catcggtaaa cctcatctgt
acctcatccc
246421 ttcttccagg gtatgcaacc atgaagtcaa tcagcatgat accatgacat
caatcactcc
246481 ggctcccgtc ggggtgacct gcctgctctt gcacgaatac acttaaaaaa
aaggacatat
246541 atcaccaaaa ggccatttgt tgacaagtta tctgggtcca acattcatct
ctcaggtgtc
246601 attatttaaa tctcctggtc atacattctg ttttgtttga ttaaaatgat
tcattgcata
246661 aataacttcc aattccagct tccgataaag gagagaaaat cccccccccc
cccgttcccg
246721 tattatctca catgtacttc caaacaaatc tccccctcca aagaggcctt
ggccccatta
246781 ataattccct ctctaatctc cctcttccat ctgatcaggt cttttctttg
tagcccctct
246841 ccatttctca cctgcccaga aactccatca acacctcctt ctctccctct
gtgcagggtt
246901 caccactttt gatgtcccgt tgttacttta aaatgagctt taacaactgt
gtaatctgaa
246961 ataacaccgc ctcggccaat tcctggaagg agcggaggag aggacgggga
agatgaagga
247021 gaggagagaa tatgttgcac taatggatgt tccctctaat gtaatgattt
tagtggaaaa
247081 agagcagggg acagggagag gaggaaggga gaagaaaaaa agatgaaatg
gatggatgag
247141 tagaggaaga aatgaataga ggttagggtg aagggcagca agtgaaagag
ggagaaagga
247201 aaggggggaa agcagatgga gccagaatga ggttgggctg attgaatcta
ttggcggggc
247261 ctgcggttgc tcctcgcagg ccgctctgac agacagaggg gatttggcag
actgatcaaa
247321 attataatct cttagccttc tcacatcaca tcggcagatg gcatgcatgt
ggtcactcgc
247381 ctctgttctc cctgccaatg atgccattca atatgtgata atagcccgat
gatctcctga
247441 aaggagccgc gactttgagg ccgtggcacc attatttcac ccatgaatct
attacgcagt
247501 tacagccagg caaagcgttt tttaaaatat tccagatctg aatttttaag
gtagcctcat
247561 ccgtagatta aatgaatcgc tgtgagctcg catgcatgac tggaagttcc
tctgatgttt
247621 aattcataat cgttatgcaa ctcagcaatg gtctagcctt tgcctgacct
cggtagtctt
247681 tgagggtttc attattagag ttagccatct ctgatactgt ggggaggggg
gagaaaggga
247741 gcaccatcta attcactcta ccatgactca tcattacact ctagagcctc
agcaactcct
247801 cgctgtcctc atcaggacta attcccattt ccttcttccc tttacctagc
attaagtagc
247861 cggtcctcct ccctctcccc attcaaaccg ttggtgttgg atgtcactaa
cagcacttac
247921 tggtcctgca atacagcgag gaagcctgca gctgccagtc ctccagtccc
tggtgtggtc

247981 agagtaggag ggctattgtg gccgcgccat tgatcgggag aagtaatgga tctggcttag
248041 gctgataatg catgatgtaa ttgtcagagg agtttaatta tatgcttatt acatgagaca
248101 ctgagtattc atgctccctt tccattgact ttacattccc cgtgtagacc atgtgaaaag
248161 aagagtaaaa tcatgtgcat catgtaaatg ggagacttaa gatggagcac gttggtctgc
248221 agaaccagtg gataaggatt acctagtcac ccaccgttaa cgtcagttcc actctccagt
248281 atttaaacac tgtagttgta gctgtagttg ccaagctgac tcaggcgaac tttagcgtgt
248341 cataaagtcc tgctggctca ctgccaccat ggagttttta gaacagggtg agctaaaggg
248401 aacacatgca acatgaactg attcatcttt ttttcttctt catgtttcaa tcaacctttc
248461 cggttgctgc tgttcctata gcaaccagct caggtcagca ataagtctaa attgctgtaa
248521 tgacattaca ccaagagtta tttttttcata aagattctat tagtaaatgt gtatcattac
248581 acacagcagc ttaggaagtt taattactgg atccacacag tgaagctccc tacccaattt
248641 atttattaac accacaatac taatgatgtc gccagcacat cattctttac cggttctgtg
248701 caaaatatat cacatggata gtgttggctt cttgtaacgt actaatgata aactagagtc
248761 agacggatgt aatgaaagta gagtaaggac tgatgtgagt gccagaaaaa caaggagaaa
248821 tgttaaaata tggacagtgc acggcgagaa attatcaaat gagcttcccg tgtccctctg
248881 cggtgcttgg aatcattttg gatggagccg aagtgcagtg ggtgggtgga tttgtttcct
248941 ttttaaatga acttaaagca ttgttcacca atgactatat cagctgcact tttctggttc
249001 aaatcattat ttgtaatgat gtaataacaa acactgttcc tttaagtgga gttgaagctt
249061 gttcgcttga tagtaatgaa gctaacaatg agctctgctg cactggtctt aagaggagct
249121 gagctaattg gccatgagcg agatctgtga cacagcaaac taccaaaaga gatcaccacg
249181 atatgtttac atgcataaaa attgattctt aaatactccg attaaaacta caaagtgcct
249241 gaaacagtgc gtgttgtacc actgctagtg agcctcataa gccatatgct ccacacattt
249301 aaatttatag cccatgtata tggtatattt atggtttggg gatttcaggt gttaaatata
249361 caggcatccc taacttcacc gctgacattg aaaagtgtgt gagggtccct gttaatctaa
249421 aatttctatc agggcgcaca cctcttctat ctttccatcc ctccaggcca tgcagagccg
249481 taatggtggc ctcatcgcat gtctgagtgc ttacgctaca ctgagcaatg cttttcaatt
249541 acctgcgccg gagctccggg atgaaaatac atagcgcata tttctgaggc aaaatgctta

249601 ggtcagatgc gccgagaaat aaaatatacc ttatgtcatt taatttgaca
gttgtccttt
249661 cccagctgtg gaatagaggt cgtcacaagc gcagctgggc tagcgtggcc
gcgaattggc
249721 gcgtagcggc atgcagtcca tgcagacgtg tttataaatt aggcctctcg
ctttgaaaga
249781 gatgaaacag tgtaaatctg tttgcagcgt tgcaacaaca ctgttaactt
cggcaggaag
249841 agacaaacaa ggcagacctt atcacctcag catcacccgc gtgagttttg
tgtgccggta
249901 aactgtgagg ctgtaaccta agcgctatca aagacagcat gaagatttcc
aacttcctaa
249961 ccctgatcag cacctgtgaa aagtgcagca agcagagttg ctagctgagc
cgcatacaga
250021 cagagaaagt ctgcaaataa tgaatcaaaa tagtgctaat ttcctgcatc
atttaattgg
250081 ccacatggta gatcaacagc aaatcccccc ggccatattt ggagggaaaa
agaaagtcat
250141 ctgtcaagag ctcagtgttt acggcttcat ctgcaattac cgcaacacag
gaatatgtca
250201 ggacaagtgt tcagcagcag acttctacac ggggttcagg gtgccaaaat
aaatcctcag
250261 gttaatcatg tttagggttt gaaaagaaga aaagaaggca aaggaggaag
gggaggggtt
250321 ggaagcctcc tggtgtccgc ccatgatgca gtctgcatta atgagcttca
agtagggaac
250381 agaggctgaa accagggcta taatggtgtc ctcctggaat agactagtgt
gtgtgtgtgt
250441 gtgtgtgtgt gggtgtgtgt gtgtgtgtgt atgtgagaga gagaggcaga
gaggaaaagt
250501 tcgaatacat ggggttgttt gtagcgggta aaacaggaca cctctgatct
ctcaccaatc
250561 tgaacgaaga tcagtgagtc cgactgctct gtgctcatcc caggttgcac
aacatccagc
250621 tgggctggtg cccagctaga cttctatgca tttgctgcct ttctgatatg
gaattgcaga
250681 aactttggga aacatgcaat gaaaagagca tttgaaccga aatacaacgt
cccggttgcc
250741 cctgtaaaca cccgtttgga ggttacactc cggtcctgtt caaagccttc
agcaattagg
250801 tggaaaagct cagatcaaat atggaacaac aaacactggc agttatcgtt
ttttataatc
250861 tcatgtatgg aaatgagtgt ttgattttct cctcatgcaa atgagaattt
caattaacta
250921 aaaaaaaatc cggggcttgg caaagcattg cgcgcttgtc tattgtgtcc
tcccctcacg
250981 gaaataaata agaataccta aatggcattt cacatttgag ataattacag
tgctggtgag
251041 agagtgggag agcgagggac tctcctttaa catgcaaata aaatgcccca
ctttgttttc
251101 ttctgcaaag atcaatcata attgggcagg tacgcacaat caccggggta
atttgatgac
251161 ccattttgtt tttggatcgc ggaccccgaa tctgtgctcc cgcgctgcga
gcgattatca

251221 aaggctgctg gcgcggctca ctgccacggt tcactttatt gttcctgtta ccaccttaat
251281 gagacgtctc ttccatcaga aagtgaagaa tggcacgttt caggccgcgc ggttcgatcc
251341 ggcgcgctgc ctccttctga gatgggacag aagtgattca cttacacgct gatctccagt
251401 tgttctgaaa tccttatcgc aatctcgccg ttcaaaaaga taacgggtat gaagctgtga
251461 aggagctcct tgtgtgccat taatgaacat tccggaactc aattcttgaa tgaggctgca
251521 gagaagtcgt taacggtgag gagcatgaat gtacagataa gaaacaaacc ccttgttcct
251581 ctatagtact caggaggtgc agacacgtga agatcaccca tccacccacc cacagaaggg
251641 taatatactc tgaaagtcaa cctgatgtgt ctctacatca gactcctgtc tttgccctcc
251701 tgtagggctg ctgggatgtg actcaggcct tctaagtcgc ccctttgctt tactcaaact
251761 cccactgggc tcccggagtg gctaatgtag tcccggagac tccagggagc aaagctgcaa
251821 gaaaaagatg tgtggtcact caaagctgcg cctggaacag aacgtgagga actggacaac
251881 tcaagggctc gtaaagaatg gtgaaacatt ctgcatgcca cctacgtctg tctgtcagcg
251941 cgcacgtgcc cccgccttgt tcgcaccgaa gtgctaaaag gaaccaacat gtgaagagac
252001 ggcgtctggc tccgcgggag tttgtttcaa ctccgcagtt gttcattgac catttcagag
252061 aggaaagtgc tcacgcatgt gagcgcgggc atagacggat tgcgcgctgt gagatgggag
252121 ttctgggatt gatgtgaatg tcttttagag ctgctgggtg tgtttaccag ttgacttcga
252181 ctgtaccagg ggctaattcc acagaactct ccctcgcagc catcaatctt ctctgatcac
252241 acctcgagcc gcatcctccc agaggaagtg ccgctcttct tcttccctgt tctcattcca
252301 tttcactcct ctgcagccct gctttaattt cccccatttt ccactttccc ccaaacccat
252361 tttgactcct tgttccacaa cccacgaccc gtcctccttc ctctccgctc cgttctcttc
252421 ctcttcctcc ggctgacctc atcctggcac taaatctccg tcctgtcagg cggcgcgccg
252481 ggatcgcgga agttgtgaac ttgagaggag cgagatcaat aaagcaagga caggctgaat
252541 gatctaccca gagaaaatgc gttttgtgac gaggatgggc acagcaggcc ataaatcctg
252601 ccatccgggc tgtctgccgg gccgtgcctg ttggtcgtcc tcatcattgt cctcgccgcg
252661 acggagggag tggaggactc actgtaagca gttacgtcag cggtcccatc accgccggca
252721 aaaccgataa atccatatta gcaggaatga tgaaaggcac tttgatacat gttcatgtct
252781 gagttggctc cagtgaaata tgagtggtct gtgtcatata tgtcacgaca acatactgca

252841 gatgggctct cttatggagg aagaacatac acttagtggg atgagctagc tgtaagctgt
252901 cgatgataag tgtgattggc gaagcataat ctgcaacctc cgagaacctg aacgcatctt
252961 tgacctttta tatgactctc tgtaatgatg cgtgttattg tagatgcatg ctgatgcacc
253021 gacagatgac agatatgtaa attacaagcc ccacagagaa agacccacac aaaccgagtg
253081 ttagtctgaa aatcctgtaa cgctaacgga agtaactttt tggggcagcg gatgagagag
253141 tgttcgggtt cagagagccc tcacgaaata ttttattttg tattccatac ataaatagca
253201 ggctgtttgg tcccatccca gcagtcttca tatttcccgg tgacattatc agcactctcc
253261 atcgtgtgca gagcgggaaa taaattgcat tctgctgttg gttctgtggc tgcagacggc
253321 aggaggagca ttataatata aaacatgaat aaacaaaatc actcatgagc aaatgtgcag
253381 gagcatgggg aggtaatctc ctttaggcat ggtccttgaa attaactcaa agaatctgcg
253441 caagaaacgt gttaagaaga ttaataaata cccccccctca accatcaccg cagccaccca
253501 tccccaaatc ctgcaaccat tccattttct tttccttccc attttttttt aattcaaaca
253561 ttatggtaat gacttattat tgtgcttact tgaggaagat tgcttggcca caggtgaatt
253621 tgcgtgaagc aaaggataat ggcgtgcgtg tgaaatcgtc atatcatcta tttcctaacg
253681 tgatttgcgc tcgccattaa cgtttttcat gcgcggggag aacatggcgg tggtaggtgc
253741 cgtgcagcgc gcacagggac agtggaggag aaagtgggca gataatactt ttaatgtgtt
253801 ttgaatcgct aagtagttta cgctcgtcga gcactgaatc aactcggctg catatcccat
253861 aatgcatctg tgtcccggtg gtgaaatttg caagacaaac atcattctct gtatctcgcc
253921 gctcctgacg gccatacatc aattaccgta tgtggtggga gccgcgttgg ggaagctaca
253981 gttaaagaat acgggatcag atgccacttg taagtccttc atctaaaatg gctgtcagac
254041 ataagcgcag gtaaatcact catctgtcca tataatcatg caaatttgca ttattcctca
254101 agtccccgac ggctcttgtc actcatttat gcatgttttc ttcatactcc atcgccctca
254161 tgatggtgga cgctttaaaa tatacaatgg gggtttcttt ggttgagtat ctggggattt
254221 tagtcagctg gtctggaatc tcaactacca ggcggatgga catagacgag gctacggaca
254281 tccatcagcc tcataggcgg aattctaatg gtcctaacag taacttgtga catcagccac
254341 cctccaactt ctaaagtttg gcttctggtt gtttttccgg aggcctccac tgccaccaat
254401 gtcacgcatg gattagggcc cccactaact ccgtagctac gctaaggcaa gaaagtgcca

254461 aatttagtta ttcccatttt caaagtttta ataagatacg gcacaattta catcaaaata
254521 aagagaccta atgttaaata tttccatagt aaatctttaa aatgctatcc aaatccataa
254581 caaccaggaa caaagtaaga aaatccatct tgaaagagca ggcagtgtga caaaacaagg
254641 cgtggtggcc ttcctcctca cacacacttg ccagagtgac aggatttaaa aggcaaagtc
254701 acactcagac cttggaatac gttacgctgt ggcttcagcg gagggtatgg atattgatat
254761 ggatataggg gacagggaag gaggggccga gccgccaagt atccctggtg accggaccgc
254821 gtgcacggct gatcggtccg tggcaacggg ccggttggaa atgactgatg agttggcagt
254881 ggaccttgga ggtttgggtg atagcctttc agtgtgtgaa tcacaggaag cgccctgtgc
254941 cctgacatcc tctcaggctg tgattgagtt cacagtcctg cagtcctccc gagtacacaa
255001 gcttcccaga taaatacaat cacgctacgt gaaagtcaag agctgtgaac acacccaaat
255061 gtgatgagta ggacgacagc gaatcaataa ggaacacaac tctgtatgat tacttttgaa
255121 tacacatgta ttttcgtgta attaagagta gtacagtgag gggacatgag gcagatttca
255181 aaatatgcaa ctaggaaaaa cagctttgaa tgtgaggaaa atttgccagt ttttaaatct
255241 tgaacctcca gtttgtactg aaaactcttg caactctgaa agcgaatcag agagctaatt
255301 ttgtgcatgt tggacacctc aaagccacag aggcgctgca agcaacttta ccagcattgg
255361 ctattagcta atactctcta ggatctatac aatatgatgt cactgctctt tattctcagt
255421 tagacaagtg gtgtttacgt ctgtgtcatc agagcacgtg aagtctgagc aggttgatgt
255481 gtcacttctc agacaaactg caccccccc ctcacagatt cctctcccaa cctgatgatg
255541 atgtgtcaat gaatgctgtc acatggtgtc actcatccca acacgctccc ccgccatctc
255601 tgccatcacc cctggcaccc acatcacatt tagtgccgct ggaaaggcag cgtgtcgcac
255661 ctctgagatg catttgatgt tttacatgcg tttgtatggc agattagaca gaagtttccc
255721 tttctgtgct ggtcaaggtc aaccatctcc aatttatgac ctcagcacag ccgactcttc
255781 gcttatctaa tattcccatc cctccttcct cttggttaaa tcatcatgcc gtccttcaat
255841 catccatatt ccttccactt taaagacaga ctggtggctt cctcctgttc ctgtgtgggc
255901 accatttgat atcacataaa aaggggattt gcacaaataa ttccataatc tccaacagtc
255961 aataaagggg tgacgcaatt gttgcctttt cggaataacg aggcaacacg gaggtgagtc
256021 attcctcaga gcgaatgggg tcttagccaa agtctttaag cacataagct tgtttggact

256081 aatgaatggc cagatttgaa gatgtcatcg gagatccccc cccccccggt cggcgtaaac
256141 agcaacatga cacactgaag acgggcactt gaaggtgcat aaatccacca aagtaaataa
256201 aggaattaca gaatatccaa tttaacaatt cacatgatac tgactctcaa gatctccctg
256261 gtaactttat tataaacaaa tgaggcacat gaataatgac aacttgcctt ttgaggtaga
256321 caataaaagg cttttggcca gagtgatgag ccagtcactc tggtacggat atcaaagagt
256381 cactaatagc acagcaatgg ttgtgtttca ccactgtgcc attattaagg gtgaaattag
256441 ctgtgcctga aaatgagtaa cagctgcaca ccggaatcat cggctgtggc ggacagagat
256501 aaggatcagg gaaaggagga cacaccgtct gtccagacct cagatgttca gtgtgagagc
256561 tgctggtgtt atcagtcccc tgaaatggcc ctctaattgc ggacatgttt ctataaaatg
256621 tgtataaatt gacggtaatt gcttttgtat gcatggaaaa tgctaatttt ccttcataat
256681 ggctatcaag agaaacacac aaactagttc agtggtattg cggtatcatg gacgagagga
256741 caataaataa acgccggcca tgtgcggaga cctaatgtaa agcaaatgca aatttgaaat
256801 ggaagtgcta cagtatatcc acagctttcg agtgctgcca cggtaattac cgactgaaac
256861 acacacaact gtggcctgcc catctcccgt tgtctctccg gaggatagcg tgcagacatg
256921 aaatgccgta tcggagcagt tttctgaggc aatgcaccca cttgagatgc tgacgggttg
256981 tgataactcc tgggttcaac gtcccatagg cagagctttg gcataatcca ttctccatac
257041 agatttctcc cacagaagcg gctttatccc attatcacag gcattataca aatgtatacc
257101 gctgtaatcc agacacttag aggccgacgt ctttggaatc agccatgttg acgacgacaa
257161 acagcttctg agagagtaca acacaaacac accataaaag taggagcagt gtcgtctaac
257221 agaccttttc aaattaaaga tatgtgtgta gccatgcaag gaattctctg agggatgcag
257281 catatagaaa ttttgatctt ggccgtatgc gggtgtgttt aacatatcca agggctccag
257341 cagagacaca cagagacacg ggcaggtcat gggggacgcc gagagctctg aactcagcag
257401 gtgtccgagc ctgaaacgcg ataactagat gttctcgatg acaacagtgg aggattcagt
257461 ctgtcccaaa tgtactgtat ttgcctaaca acgcagcagg cagtggagcc cccccccac
257521 caccaccagt cgagatcaga aacacagggt acgatgcccc atacatcttc tgagccatgt
257581 gatccatgaa ggacaaagca caagagatca agaaaaaaat gaagaatcct gctgctggag
257641 gtagatgaga aaatccagga gaagatgaca acactaaagg cttcacaacc cgttttctag

257701 cagtcgtaag catgttgaag tgtccttggg caaaatactt taaacctaat
tgcccccaga
257761 ggcaaagcca acagggtgtg aatggatgca tgtgacatgt gttggaaagt
gctttgagga
257821 gccacttatg aacgggacca ggctacatac ctgggtaaga ccagctaaag
tcacctgaat
257881 agattaccta tattttacac tatgaatttt tagttgaaat tttacatcaa
ggctatgaca
257941 tttgagatgt ctgctcagct ggcttgtgca ctgctatggg agacacggtc
tagcagcaga
258001 tgcatgcagc gcgccgctct tcgctgccac tataattgac atctgcatgt
aggagaatgg
258061 cagaggcatc agtctgtgcc atgtttctgg gaaataaggc ggcgaagaag
cagagcagat
258121 cctgagaggt ttgcatcaac tgtctcgcta atgcctgcga gtgctctggc
attaatggca
258181 tatattatgc agcaaattat ggaatcatat atagcagctg ttgatccaat
atggatataa
258241 tgctacagct aaaacaagcc cccccaggga ggagtggtta gttggggttg
atttagcaag
258301 atgctaatgt attcagctgt tgtttgtggt ggtgtgacag aagtcttatt
agctttaaat
258361 gaacacaagg aggatgaaca tgcaggatca cccagataaa catgatcaat
agatctgcag
258421 aggactttta acaagccatc tcttgtcagg tcatccaagt gcatcttcca
tccctcattg
258481 tctgacgatt tttataacgt caacactggc tgggtattgt agtccgtgta
atgtttggta
258541 ttgtttgact aagttgactc tcctctttga cggaactaca aacatggctg
gattcatatc
258601 aaacacggtc atttacttca acttccaggc ttgttttctg tacattttga
tctaaaacac
258661 aagcaaggtt aataattaat taataattaa taataataat tgtcctattc
tacatttcag
258721 gcttaaagat ttgaatttgt ggttagggca gaacaaactt ccagcaaatg
catataatgt
258781 acttagatgc atttttattc tctttgttgc ctcatttggt ctgaatcatt
gcggtctcga
258841 tgaacttatg tttctaatct aatacagaac acactttcag acccattaca
agctggtcct
258901 ctttcatatc ttcaattatt ccatacacac ttggcaaaat taacttctac
cctcaactgg
258961 gattaagcac agccaccata ataaatcaga tttatataac tggtagaatt
catcgagatt
259021 ttatgccccc cctccccatt tggggttaat gagtgtcttc cgcagcctcc
cctcagccgt
259081 ttcatacctc aggggtctaa ataattccca ttgatagttt caaactctca
aatgagacag
259141 gattaatcaa aagcagagaa agtggagctg atgggggtga agtcgatgat
gaggatgtcc
259201 aaagcaggag gaaggcgaat agttatacac agcgggataa gatattcatg
tgactaatgg
259261 ccgctggtga tggctggtat gagcagtacg agagcttcat gatatttagt
gcttccccac

259321 tttaatccaa atacgtttgt acattcagga gcggtcacta agttagtcat tcacctcaca
259381 cctagttttt accccctttgg gttcaaatat gctcgtccat tacggcgttt ggcgtatgtg
259441 tggagctttt attaaccaga aggaaaccgg ttctccgggt agcaagtaaa tttcagttga
259501 agacatttgt gcatgattaa agcctctcaa atgtcagttt tcctgaggtc ccacagtgtg
259561 acaagaaccc catggacact ctgtcatgca ggcagcatca atttaaatcg gtggcgtgtc
259621 agccacggcg catctcagct ggatgatttg gtgattttgg tttagttgtc cttcatttgt
259681 tgttgctttc agaaaaagtg ggcggtgtgt gcacttgcat atgcgcatat aattcccctg
259741 attgcctttt ttttttttta aatcatgtct aacatacatt tttcagtgtg tcgatacggg
259801 ggaggttcct gtgtggtgca gattgatgag gtttccttcc aaattagcgg ttttctgcag
259861 cgcgtgactt tgatcctgca tctgggaggt tttctgaggg acttgagtcg gatggattag
259921 ccccagtggc tatgggggct aacatgtcgg ttaaacgcag gattaaatat caggagttca
259981 catcctcagg taaggttttg ctatccgtcc acgtcgggct cctttgtggc gatggtcttg
260041 gcattcaatt tccttgggat gagtcactta catcagttca gtaactggtg aggcatgaat
260101 ccatcctgtg aattccgttc agacatgatt cattttaatt gtcacactgg gacgtgctgt
260161 tgcacctctg taggacgggt cagcgcataa gaaaattaaa gccgaggctc taatttgaaa
260221 tctatttgta aattctggcc tgtgggacga agatatgctg agtgcatgta aatgctgtcc
260281 cgcagaggct tgaacttctc ctaaaccggt gaacagtcac ctgggtatgt cgcccccccc
260341 caaccccccac ctggatgccc tccgctcctg tgctcactct gagggaactc gcgttaagct
260401 gcagcaggat gcccgtttta catgactgag cgcacgtttg cggtagctgg tgccagctat
260461 gaggttaaca agtgtgggcc acgccggggg ctacttatgt tctggatttg atgagctcca
260521 attccacgga ccattaaaca cctcctttta tctacttttg gggatgcatc agtggaaggc
260581 agcgatcagc tactctcttt ttgaaatatc agaaacttct ttgaaacttt gtatagaata
260641 aatatggctg tcagaccctg gcagaacgtc atattttaga gccagacgag tcaggacctg
260701 cacaaccccg gcctgacagg tttgagtgca cagagaagct cagtattccg ctctttcaca
260761 tggcctcttg tcctccgttg tcatcagcgt cttccctgct tcccatctta tagctgcacc
260821 atttagggaa tgctgtgttc cctctgctca cttcaagggt gtagcagagg tcacggctct
260881 gtttagacta catgtggaaa agaagagggc ttctctcctt cgtcagccaa gtcttttctc

260941 tgtctcccat ctctcaaaca gcatcgccgc aggaactgcg atgaaagacc aacttttaga
261001 atgaatccaa ctcgggctgg aagccacacg ctgagaacaa agagcatctc tttagcttta
261061 gtgcgtaggg gtgtgtgtga tttacattgt gctataaata catttttttt gaatcactga
261121 aacacagcga tgacgtcggt tccaacaatg taaagtggat gtcactgtaa ctttgtggga
261181 attaggaaga ccgagggaag tctgttggaa caactcggaa taaaagtgca gatctgttca
261241 tttaaagaac gaccagcgct ttgagatctg cgccgtgtgc ttagctcatt aaaacttctt
261301 tgaaccttga atcagagcag gagctgtgag aagattccat cgcacaactt tagctcggtc
261361 tccgctaact tggcattcaa attaaatcaa ctgggtgctt tgaaggaaat ttgtccaggg
261421 ttcacatatg caggcgggtc attaccagag gtgatgcagg tcaacaggct agacggtccg
261481 gccgagtgac tgctgacctg tggaagtgca ccgaatgagg aaatgggatg caggtgagga
261541 ggcagtgaag gagccggggg aaggaaatga ggtgattgca gtggtctgaa atgacagaag
261601 gtcacaggag gagcacagac aatcaccaaa ttaacccaaa actccggaat tttgaattcc
261661 aaacaccaat tccaattatt tgtattgatc ccgtgcgtgg tcttacgtgg tcttataaaa
261721 agcaataatg gttgaggcct gtgaccatta aacagcaata tttgatatat cgatcaggat
261781 ttcaatgatt catggttcat cagcgattca cagaactctg tgctggaccc tccccccatg
261841 caacatgcac tgttgcgacg aaagtgtcga tcagtgtctc gtggacattt attttcagga
261901 ttttcgtgac tatctgcgct ttaatgagtc agacgcgcgc tggtgtttgc ggtcgatcta
261961 tcacgctcca gtcggcctaa agaggcagga aatgaaatgg aaatgagcgg accaatcacg
262021 ccggcttaat aaattcccca ggaagcgcca cgtagacgtc tgcgtgagga ggcctgtgca
262081 gggtggtttg attgggaatg aaaccctgat tccagcagaa ccatctgcag cccgattctc
262141 tgtcgtctct gctcgctcag acatcagaca cagaggaatc gcaagataca atatttgaca
262201 ccaaacggct aaaacagtag atatgtttgc atatgtaaat gatgacattc actgactgaa
262261 tatataagtg atttatgagg ggacatggca gtcctcctgg aactgggaga tttacatgcc
262321 catcatctca gctcctcctc agaagctctg acatgcttat ttctgatgct cccagctcat
262381 acgctcgctc ttctgtccca caaaactggg taattgattt gccttggctt tttattttta
262441 gatttgaatg acattcgcag gaatttttcc aattgtcatg atctaattgg cgcttcctcc
262501 tttaaccagg agacgtggca cagaggggag gagaggttct ggaggcattt tcgaaaggag

262561 gacacgttac acagcacttt tgtccgagca ggacggaaca tctgctgttc ctttttttaaa
262621 atctttctat accttattct ccaccctctt gtcagagaca ctcggctaat ctatcatcct
262681 ggctcggctc tcaaactcga ctttacgatt ggaacgagaa ggtagaatga aaggagaaca
262741 ggatctcgtt atttcagacc caccgctcgc tcgctcactc tcttcatgat tgatcattat
262801 ccaaaaagaa gaaaaaagac catttggaag tgagtttgtg ctttagaaag caaaggccta
262861 ttaaaatcat attcaaagtc ctggcggaat aaagcaaaac tactttctct ccatgcctgc
262921 gctgtgatgg aaatgatcaa tctttcgcta tgtgctcatg aaaatgcttc tctataaagc
262981 tggttatttc ctctctgatc ccccaccccca ctcttaaatg tcactgcagg tgcaacggtg
263041 cagtggaact aatgtcataa atgtagtagc gcacatttga ttcacttcag ccgttcgagt
263101 cctcccattt catggcacgc cgccgctcct cgggcccggt ctccttttcc taaatacctt
263161 tacagatgtt aacctcggag gcgaatttcg caacgttttg gcgcctttcc agcgggctgc
263221 tggaaagctg agagagtgat gaaaagactc ctgtcttctg tcggtcgtga agaaaaaaaa
263281 aacacattta aaaaaagact gtgattatgt tagcgtctgc tttgacatga caagtcagca
263341 ctcggagggc ctgaagaaaa ctggcgacta cggctgggct gaagggtggg aaccgaaaag
263401 ctgccccacg ttttcctgtt taaccatcgt atatatttac tcttgcatcc ataactcaca
263461 acagctgcat ttctgttgaa ctaatcattt aagagtgagc aacgtcacca tttgtagcgt
263521 ttattcaccg tcaccctgaa acttcccatt tatcataact tctaattgga gatagagaga
263581 aggtgcggtg aagaaggcat catccttatc ctcttttta tcggaatggg gggagggaat
263641 taactcctga catttgtttc ttgggcctga aaacgagtta gattgtacca acaaaagggt
263701 ttaatctccc ccgccagcag ctccgcctct gcaccttcca aatggtgcga cagatgttgc
263761 tgatgcagat gtgacgtctg gctcggggac gtcccagcat ggaagcggtg ttggtttcat
263821 gacgatgagt ccactgtgcc ctcgctcctg cccggcccgg tgacccctgc tgacctctct
263881 gaggtgttac caaagacagg gggggagaa atccaaagag gaaagcagaa gtgaaggaat
263941 ggaggtgaac ccacagacga gctttaacca caggaaagct ggatggttac gttggcttcc
264001 tttgatagaa catctgtaga aaagcagccc caaatgacag gggccgtgtg aaaggcttaa
264061 tagcctaact tcctgcctag cctggcagaa atatctgtct gacgctgaca gctgatgaag
264121 caccagcact tgattgttct cccaagtgca gaacaagtaa agacctatct ctgcttcccc

264181 tcagtcctca ggcagcgagg cacaagtggc gagaagagaa aagaaagaaa aaaaaactcc
264241 aaagttcttg cagtggctgt ctcctcctca gggctaatga caaatttagt gataatcgcc
264301 gtgttttaat tgatgtttcc gcgccagtga gccgaaacca ccggtagatt tcatcaaggg
264361 agcggagata cgagcggagt cgcgtctgcg tggttctctg gatcagctca catctgatcc
264421 atcctggtgc tcgaggcctt gaatgcacag ctcacctctg ggacggagca acctgctctc
264481 agtcgccgct ggaggtaggc ggcgcacgcc gtcccatctc cacggtgccg ccttcggacc
264541 gggcggtaaa tggcgccata tgaaaagcag gctgtttgat tgcacggcgg ccccccagtg
264601 tgacactcca cagcgaggac taatgtattt ataaaccccc tcatatgtgt actgggaaca
264661 aattagagac aaatgaggca agattcctcc actcgccccg gaaagccatg aaattagggc
264721 agcgtcgttt acaactcgca ctagatgaga gcggggcctg atgcaaagtt aaacacgctc
264781 tcaatgtgtc cgtaggagga attctacaga gtttttaatc atcggtgatt caaataaaat
264841 ggagacagtc ctctacatgt aaatggctgt ggctgatggt gttggattgt attttaaaac
264901 cttctcttct aaatgattct aaagtcacgg atccacaaca gtattgaaca atataataag
264961 aattcagtga tttatacact tgtttaacat atatttatgt attttagcta aatgctaata
265021 tgttgctttg atgtgctttt ataacccaag tgtgagacag aacatgacat tttttttccgt
265081 tagagtggaa ctgcattctt gcgttatcaa acacacttaa cacacacttt aagcgagcgc
265141 tgtccttccc cttcatctcc gagatagtaa ctatatttgc gtgtgtgatg tatttgtgtc
265201 ccttcctctt acacaaccat acggacatct gtgggactgg agcacacctt gggtccagaa
265261 cttcaaggaa agtgaccttg agcaaggtta aatagctgca atacacacat gccctggtgt
265321 tagctgtcat cggctaacat gctaaaggca tacaagcgac gtgtccgttg ctctgcagtg
265381 gccttctgat ctttttggac aaattctgat gcatatttaa aaagtcccct caaagtgaat
265441 gtcacatatt caatagctca ctctcagaca cagtgtctgc tcagcaacaa acagcatccc
265501 tctatatatg taaatcagat caataggata tccatacaaa cggcctgcag ctgtgtgttg
265561 ccatcaagcc gctcacctaa tgtaataagg aacatcaata gttgattttc aaacctagtg
265621 gtgcagagaa agaaaatgag gaggatgaga ttagcgcagc ttggctccaa cgttgggctc
265681 tattttataa acaggcacaa ttaacgcaga ctgtcattag gcaggggaca ataaaatgag
265741 tcggggagag cggattaata acctcaccgc atgagtcaaa cgctgctacg ccggagacgc

265801 actcggattt gtttctgaca gtcagcgctg acatttggac attcggatgt gataattaat
265861 cacggggaag gcgaccgctc acaggctcag acaggaggtg acagccagca cgtccaccgc
265921 gcgcggctcc tcagacgggt gtaatgatgc ggtttgacaa tcgcctgcac tgtacgctaa
265981 cgccgaatta gcaggtgtgc gcggcagcga aaaatgcttc cattaacgct acatcctggt
266041 cgttgttccc tgaggtggat taggggccag tcttaactaa ttatgttttc actgggaacc
266101 cacatgttat tttagccaaa aggaggcttc atatgagaag tcctcaagtt cagctttagt
266161 tttagttgtt aaatagtttc cgtttgcgcg tgcttcgtgt gcgtttcaag gtattacagc
266221 taatgcgttt tcgtgtcaac ccgtcactca gctgattcaa ttcaagattt aattgattct
266281 ttttttgccc tgtgaatggc tgtaaacgat gtgacagaaa cagggcgctg tgtacattta
266341 tccatatcgg ctggcattta tgacatcatt gtgttatgta acagtgctga ccccctttgt
266401 gctgatatct aactccaaat gggggctctg ggggatagag accaactttt gtgtgggttt
266461 ctgtgcatca gcggcttcat ttatccccat ttgtcaaagt ccggcattca gaaaacttac
266521 acctacgcga catcagagtt aactgaaggt tcgccttacc ggtgcgctgt cagacgagcg
266581 cactcataat cccgactccc tgcgccgcca caggctgcgt ttggagtgtt tcactggagt
266641 cgggtgtcgg ggggtgatga agctcttggg cctgctctct ctccctccct ctctccttct
266701 ctgtttcatt ggattattca gcacatggtg aatcagccat ttgtcactga cacagaaaca
266761 gcctgttttt atgggcgcca tgtgaacaat ttaatgaaga aaaaacctgg ttcggtatct
266821 tcggggtatc ttaatggcag ttcatctgca aggagtgcat gaaaagagct gtgataaaca
266881 tcatacctgg gggtttttgg agtttttccc ggctatcttg ttttctaaat ccttccgtca
266941 gcctttcact ccgctgctgc accagaaaca gagatttaac tttaaaaatc tgccttcaca
267001 aggctataag ttagggcttt agcaggctga aggaatatct cctgccttaa ttgtgttttt
267061 ggggcactat taatgccatt cacggtgtta agaagagaaa atgactcttc ctgctcttgt
267121 acacataatg gaggagccag aggacgatta cagctgcaga ggaataaaac aggagcggga
267181 gaaaggggca aaatggatga agcaactggc agaaacatcc aggaggagaa agccggccag
267241 tggaagacaa aacatgtttg gaaagcgttc agtggcgcgt ctcaaagtca taattaactt
267301 ctgaaaaaac ccttcacgct cctgccagcg gagccgtttt accttcttcc cgagcagatg
267361 cgacgcggcc acgtttttac tgcgctcgtt tacacgtttt tcctcagcac caggcgtgca

267421 cgaggttgct gcagggccag ccggcactca ggacatttgt gttggcccct ggccggtgcc
267481 aggctgggtc tgggtctgtg aacaccactc gtctcttcca gttgtatttt aatgaagcac
267541 taaagggcag aacaccatga aactacctgt ttctctaata attgctgcac cttaggccgc
267601 cgtctgcaga gttagttatg tgaccaataa aacactaatt gtgcaaactg tttgctgtgc
267661 agcttgcacc aaacaatcat ctcacactag gaacagtttt ggagcgccgg tgaatgtttt
267721 ggacttaaga tgccaaattt ggagtcctgc agccaaagtc agaaacaata gctttggagc
267781 aatattggat tttatatcgg ctattaactt gttgactaat tgatcagtac aggtgtgttt
267841 ttgagtgaat ggaatcattt ctaaggccca ttatatctcc tgcgtcccaa agtctctggt
267901 ttaatcactg accagcagat gtggagtggc aggccctcgt taatcctttc ctttctttga
267961 aataaattat gcatcaggag aagaaaaaaa gaagcttttt ccacctcagt agatgtgtcc
268021 cttaatccct gcctcctaaa acctgcttaa tctgaactaa gtcattaaaa cagtaaaggg
268081 atatcacaaa ggactttcaa accaattggc ttaacggtaa catgattaga tcattgatct
268141 ctcggcaaag ccctgggctt ccaggtgcga aatgactatt taatgaaatc agagaaatct
268201 cgttaccaga aaattactga tcaaagttaa gaccaggtga ccggggtggc gttggtatcc
268261 gtgtatccgg acagaggtat ttattctcca gatcccctct tgacattagc aagaatgcat
268321 tgggccggaa tgggagacgg tgtcgttaat ccctaatcag ccactatcca tacctctatg
268381 catctttaac aagggctgca gctttcagct atgatagtga cccccccaaa gaaaaagacg
268441 tgcaggactg gaggagaatc ggtttaataa aagcagcatt tggcttattc cagttctgct
268501 ttcctggcca gcagcctccg gatcatatct ctgataacgt ctgcttagtg tcagataatg
268561 agccaaattc accgacttct acctaatgag ctctgaacag gacttccggc cgcacgccac
268621 gccaaacaca cctcagattt atgcacgccg ccgcctcacc tgatgcgtga tgacacccgg
268681 tatcgctcag aggattagcg ctgtcctctc ccgtggcgag aaataaataa ttatgtcacc
268741 ggcgaccttt tctaattaac cagaagaaat gaaatatcac ttaatttcaa atttaattag
268801 gagccgtaac gtttccccgc gtccgactgc ccgcgtggcc acgccgcggg cgaatgtgct
268861 ccgtgttaaa gtcgggggcg tttacgattc cccggactgc acgtgaaatt tcccgcgctc
268921 tacgggtggg aattgccagg acagccccgc ccccaacggt cgggcaccta caatcattcc
268981 atacgcgcgg cgggtcgtta tcgggccgcg caagataaga aaaggttaat aaacatttgg

269041 taatgaaaat taatgacaca aatcacacag cggcccgaca cacttttccc
tgtttaattg
269101 aagttatttt taattacaga tatggctgcg ctcaggtcct tcaatgtcag
gcggcccaaa
269161 gttctcacgt gcacatcgct gcagatgagc cttccgggcc attatcacaa
ccaggacaaa
269221 ggtattgact cctaccctca gatgagtttc gcttatgcaa taggaactgc
cattaattgg
269281 ggtaatgagg cctgctcgcc aactttcacc gccactttta gtctcgatca
tatattgatg
269341 atttggcata aaaggaggtc gttactgagg tacgggtagt acggttgtca
cgtcaagcac
269401 atacccgacg ggttcaacct gacccttgtg aatgtttggc tttatcaaaa
ctccccatct
269461 gaagatggcg cctccttcag cgagatggcg tgatgtcaat cagcgatcga
tgcacctgac
269521 aaaggcgctt taaactggat tagcgctgcc gaacacgcgc cggctgcaga
ttccgtggga
269581 atcccgcaaa ggcccgaaac taaattgttt accagcgact gtctgcgctc
gtctctgggg
269641 gccgggagaa tgtgcaaacg gcaacttgct gaattagcac tatgctgcaa
gaaaataaga
269701 aataaataaa taaaagaatg attctcctgc tggctacaga tggatgatgg
attttgaata
269761 atgcaaatat gataaattac ccaacactaa gcatttgact aattaattat
aattaatcat
269821 gttgaagttt tcggaggtac ggccggggat aatgttgtct gtttgatgta
ttgcccagat
269881 gaatcccagc atgtggatca actgggcaga agcttcagac tggtctctta
aaacctgctc
269941 cgtgtttgtg cctgtcttcc ctccaacgtg tccccctctt tccactccac
agatgggatt
270001 tcattttaat tgacagttac ccggatccat tcgtcaatgt gacaaactga
tgagtgggtg
270061 gggagggggg ggggcgaggt ggagggggct tctgttgagg agcaccgtgg
atggaaccag
270121 cgaggtggag gcgtttcatt tccgtctgtc gcgcctgccg ttgtctccag
ctttttcgga
270181 tgggtcgatc tcgccgtcaa acgccgcctc tgtgtgtttg tttatgggcc
cggggagggc
270241 ggaacgcccg ccgatccatt gatccggggc tttctgagaa aagtctggcc
tttgtctgcc
270301 agatctacct ctcccggcgt ctctcttttc actgtcattc cctgacaccc
ggctctgaca
270361 catcttgagt gctgcaccgg tgggaaaata acacatatct ctgacctcct
gaaccctttt
270421 cttctctgaa aagaaaggag gctttagctg cccgcgcctc ttttaatgtc
taccaaggga
270481 tgtggccact gagacaagag ggcagggaga cgtttcagga caggtttaga
tcttcggccg
270541 gtgtcaggtg cgggcatcaa gcaaaacaac cagagattta cggcatgcac
gcttcatgtg
270601 cggagcgtag actgcagcca ggctggagta atggacagtg ttgtcagcaa
ttcctccact

270661 ggggtctgtt ctacgaggtc tgttcagtgt taaatcacag actgtataca
atgctggtca
270721 atgcatttcc gggcggctgc agtcactgtt tactctatcg ggtaaagatc
ctcaaacaca
270781 ctgacacgtg gaacaccccg aatacacgtg cgagccgaga cccagacgag
acggaattgc
270841 tcggttgaca taaacagctt cagatgatgt cacagcagtg cttcataagg
cccgctcact
270901 attctgctca gacacgaggc aacaaatgcc cttaaacgcc ccacgccggg
cgccttcgca
270961 cattggaggg gggcgtggtt gaggagcagc ccgcctgggc cagcagaggc
agcgtcatgg
271021 ctcactcgca gagccggtgt tgtatacatt aaggcaggag gggaggggat
tggcagaggt
271081 ctctccacag caacagagga cagcctatgg aaaatgcccc ggctacccat
cttagacgat
271141 gatgtcatca aagtgaaacg tgcatttccg cgagctgtct cctgcagtgc
acgtgtgtac
271201 gcgtgtgtta cacacacaca cacacttcct acatgctgca tcaccttcag
gctgtcctca
271261 gctgtagtaa gagggatgtc tgctcctaaa tgatttcctc atatatattg
aatgaaccgc
271321 acatcaagct catgttcacg ggtgatttat cttgtgcagg tgtgataatg
tcaatatatt
271381 gattaatcaa gtcatgattg agggccgtgt gctctactga tgattaatat
tgataactgc
271441 gtgtcacccc acacttgaca gtacaaatct aatcacacac acacacacac
acacaacaca
271501 agattgagta tgtgtgttta ggaatgtgga agtgtgaccc gggggattac
acctaacccc
271561 tcccaggctc ctggttgcat tgtctgctgg cggcggcgta ttgtaccaca
gcgggccgcg
271621 tctcaaagag cccaaagaag tttgaaaata tggacagctt gctcttttat
tcccccgaat
271681 tctaatccag tttgggccca caatggtgct ttactcagtg aaagctgtga
aaggggacga
271741 ccgcgagtgt ggccaccaag tctgaacgct gatttcacgc atctggaggg
ggaggggggg
271801 gggggcatg aggtcatgag tgtccactac tgttacacat taccataaaa
cttagtctga
271861 gtttgatcaa actgagtctt tgtcatcact gtaatgtgct gatcttattt
gctgtgtgtg
271921 ccagcgaggg gaagtattga agcgatgtcg gcatcgggga ggctcctgcc
tgggaaatat
271981 gcttctaatt gtcaatttac atatttttat gtttgattta atgagctaaa
atccaggatc
272041 tgagcagtta acagtgatga tcggtgggta tcgtaatcct cctcatgaga
tcattgtttc
272101 atctagttgt ggtgaaacat ttatgctccc tgattacagg atagtttgag
ttttcaagta
272161 gccccaaact ccacttgtgg aggggggcag cagactcatc aggatgtcaa
ctttcctgaa
272221 agtgcaagac ctctcaggac gtttcatttc cgctcccatg ctccacatta
ttaatcctca

272281 agcctgtaaa ttgcccagat tagctccacg tctattcagt gaacctttga
cctctaattc
272341 tagccttcac cctggtggtt tgttggtgaa aagggtagaa atgtcaaaga
aaataaatgt
272401 atttatgttt ctccttcctc agcgaggtat gaatatgaca cttcgacgtg
aaattaggtg
272461 aaattattgg ttcgttattg tttcagccga ttcacaggat ctgacttcct
gacttccttc
272521 ttgcagccct gacagttctt tttaccccca gcacttggcc acggagagaa
accagtgcct
272581 gcactgattt gttgtgctgc attaccttga cagatcgact gattgttgac
aaacagcttg
272641 actcaatgcc aacgccgcca tggccgccgc cgctccgccg cctgactgtt
ccgcagtcgc
272701 gctgtgattt gctggcgctc cccaggatga caaccatgct tcatccctca
ctttcattgc
272761 accataaaag ggccctctgt ggtgctggat gccgctgttt acttgttagt
ctttgtcatc
272821 attaccgtgt cacagacagg aaaaccagag cgggagacgg gggcgagggc
ggggactgca
272881 caaccgcggc tggggcagga gtcacccagg tccaccggta tcacatcatg
cgcccatttg
272941 tcttcataca ggtgaatccc aaatggcagc tgagcacttt gtaaaccccg
aagggaaagg
273001 acagagaccg acgccctgaa taaaatatct gacaaagcta aatcttcgtt
agcaacaagc
273061 tgggaggcta aactgcgcag ggtgaatgga aacgatcaat aaagcccttg
atttggctgt
273121 gaaacctgat ttagaaaggt ataaaaagat tttaacagga gaaacaggga
ctttttgatg
273181 aagagaatga tttgtacgct acctgatcgc tatgctgaca ccacaggctc
ttttgggtgt
273241 gtgccagttt ccacgtttct gtgtttatct gcatctgttt gtagttgcgt
gtgtgtattt
273301 aaagagctct gctctgttca gcacctgaaa tagaggcttc acctgtggat
gaacctgcac
273361 agagcactgt ttcatcctct cttttatata catccaaaaa aaaaagaaaa
tatctgggtt
273421 atgctggctg cagacaggca gcatctcgcc tatttccctg cctgaaaaga
gcagaggctc
273481 atccaggccc tgcagcagtc cgggataaca aggagggggg gctctgatct
catcgtgctg
273541 cagctcactt tgccgggaag gagcaggggg atgatgtaat gctgtttgtg
cggcaccatc
273601 agatgctctg tgagggaagg ggggagccgg cgcaataaca acaccttcac
tatgatttta
273661 cgaggcatca tcgtcttggt cgtaagccgg tttactggaa caatcatcat
cagactcccc
273721 ccatatatat actatatttt atatatgcag tgtacgtgtg tgcgttttca
cattaaattc
273781 aaatctaatt gaaaacagca gacgtgttga aatcacgcca atcacgctgc
aagaacgtgg
273841 ggcaaattca gataccccgg gcatgaaaag ccccccaaag ttaagaaacg
gtgtgttgga

273901 cagtttaatg gtaaatcaca cactccgtcc agttcacctt ccactgaaat atataaaaga
273961 ccaaatgaat tgattgcttt ttactgctgc ctctgagagg agcacatcca ttctcattac
274021 cttcagctct ccccttcac cagtacgaga cagtcacctc cacagaatca tacgtctcca
274081 gtccagtata atgccggggt gagaagatta tgctgtgggt tttatgccgg gtaattaaag
274141 agatcactgt tttatgtcct aggctggaat agaccttccc tgattcacag ccctgcgcta
274201 acgtgcaata gccttgttcc tactcttgac tccactggtt taattgccct ttgtgtccgg
274261 cttatttctc gtctaactac tgtcatatat aatatgctga gtgcctgcat cctttttaca
274321 gatattaaaa ggaactgagg aagagagtgc ccgcccgtca aaaaccacgc tgatgagtaa
274381 gtctgcaccg actgcctccc ccttccttac ctcacctcct tcccacttgg cgtagcgtaa
274441 tggcacaact ccgggagcag cccctggtcc tgtccaacca tgttcccctg tacaaggctc
274501 agcacgtgtc agtggcctga ggtgggggg ggggggggtg tgagtcagat gtacacttgt
274561 caggtgatgt catctccatt gagccctgat gagcagcccc agctgtttca gtgcactgag
274621 ccacagactg tagcacctgt ctgaagccca tttctgcagc acagaacggg caccttcgta
274681 atcagccccc cccccactcc caccgctgat gaagtcaatg caggtcgaac aggctggagg
274741 gtgcatctcc aaccgagcgc gcttccgtgt tgaaagggcg aggggaaaat gcatacatca
274801 gcataaatct gaacagtcgc gtcacaggtc tccaactgtt gccgatgaca tcattcctga
274861 cacacactta tgggtcatcc atatgatgct aggacggagt catacggtgt cacatcctcc
274921 ttaggggagt caaaaaaaaa acttatttaa taacatagaa gtcaaagttg atttctatgc
274981 atggattttt tcatttattt atatttttgt aataaagctc caggcggtga ctggaaatgc
275041 agacacgccg tagaaactat tgctgatgga accagctggg tacccgtgca catgcctgca
275101 tacacagtaa cacacgcagc tcacgacaat aggctgcaac aaggcagcag agcctcactc
275161 atagtaaatc aagaactgtt tcactgtaat agctgtactt tgtaatagcc atcgcagtgg
275221 aataactctg atttaatcta gccggcaatc aatattttct gactgctatg attgatgact
275281 attaagtcgg actattaaca gagtgaggag gggaaaaaa gtctccaagt ctgaatccca
275341 ctatatgccg ctcagtcaga gcgctaatgt gaaagggaat aatatgtcag ctaattcaca
275401 aataggctta caactgttga catatttttc ttaatgaata tgtgaattac tcgcgcattg
275461 atttctactg tgtgggggaa aatgggaaac gtctgaggag ctgtccgcac aggtgcctgc

275521 agaaacactt cattgacgtt ttccctggga gagcgggaga aatactgcat atgctgaaag
275581 cagcagattg tgtatttaaa gcctctggtg gatcagggtc acgtcagcga ccctttttagg
275641 agcagtctgt cacgcattta gcagagtagg ggatcatttc aaactgtcct ccatcacatt
275701 atgacagatc ttaattgact acccatacgg cttttgctga gttggcggaa aaatctgatt
275761 ttgatctaat cactgcaata tacctgattg gcctccagca ccactcggcc cttttagggc
275821 atttgcattg tggaaacaga tcagaactta gcgaacaact aaagccttca tgcagcacat
275881 cgacttccta aattaattcc ttgtggcttt tatattccgt aatagaagtt tttggagcta
275941 aatgtgactt tgtgtgcatg tttaatgagc gcacgttaat gggattttat cattttacaa
276001 tataccttca gttatacttc aggctttatt gaagttctat tcctgctgca cgacagtcac
276061 attttatcca ctttttaatg cagacatttg gggaaatgat ccaagtttga aactttaaaa
276121 gaccgaactt tggtcatctc ctcgttgggc agcccttttt atacgacaca tcttatagct
276181 tttgtgcatt tcctctgacg cagagacgat tttcagccag tcgtccccgg tttatcactg
276241 aaccacatca ttaagcgctg ccccacggtt ccctgtctaa actgcacgtc agcgagcagc
276301 cgctcgctcc ctggataaaa agcccaaggc tgagcacgct gacatctccc cccgccctcc
276361 ccgtacataa ggtaaatgat ggcgcataag atggtggtcc agagtacctt cagtggtctt
276421 gggggagagg agagcagaga agaatgatga tagattgcgc ggggagtgga ctgagcagac
276481 agaacgagcc tcatccaccc aaagagcacc atctgctgtc agctcaggtg actcattcca
276541 ccacgctgac atgtgcgggg aaaggagcat gctgggaaca cgtcaggctg ccgtcagcca
276601 ccgcgatgat gggctgaata tgaaagagtg ggggggagga ttgtgggaat aatataagtg
276661 ggctactgca aacactgcgt gtttcttttt ctttttagtg tctcttctcc tgatttctgt
276721 ggagtttcaa agaaatcggt ggctgttaaa gtccgccgga acacttaaat ccagctcagc
276781 ttcggtggct tttgcagaac acatgttctc cagcatgttc tcaccctctt acggtgagat
276841 ttcagcccag acaaactcgt gtggacctaa agtgtgaaat aataggataa ctgaggggca
276901 agaagccagt ccaatggtgt gagatgacag tgtccatccc tctggaaaca atgagctgtt
276961 tatgggccgt tctcccagtg tggagccact ctaactgttg tcttcacctg taaaacccgt
277021 ttgcaaagag caaagacggg ctgcgtgtca ctggagaaag ccaataatgc tgccgattgg
277081 gcaaacatga aggttggagt catactgtcg ccccgtgaag tttagtcctt ttctttacca

277141 gtaaagtgta tatttccctg gaggggcagc aattatattt ctggaatgag atgtcaggtc
277201 gggatcaaag tcgggatctt gttgtcttca atttcatttc aaggatccag aggatccaaa
277261 atgtcgataa gaagcagctg tattgacact tgtggtatga aaacagcccc cccccccaca
277321 cacacacaca cacacacaca cacatcttgc actcccagtc tcccactctg caggtctcgg
277381 gtaatgacca gcgctgccgt ctccaggagt gacggggcgt gccgatactg aaaaggtcgc
277441 aatttatgaa aggccgtgac aaagctctga tcaatggctc tcataggcct caaacaaggg
277501 gatgagatcg atccgatagg tgatattgtt actggagatc tgcagacgtg ctgcaagtgt
277561 caacgggggg acgccggctc aagttcaaag caggcaattc tataggattt gcgtcagtca
277621 ggcgaggacg gttcggacag tgatgggaga ccctggtgac cccgcagggt gatgcgcggt
277681 tgtcagcctg agacaggttt aaactgccgt ctacaggggg aatattatat tatggtgagc
277741 agccgcctcg ctggccggtg gcaatcagac agcctctctg ggctggggtc attttattag
277801 ggggtgggtg gatgaatggg agtcttatga gatgatccag acagatcttc caacgggagg
277861 tggggtactt tagctcacat caatgctggg gcagatattg ctgtgtcaac cccccaccct
277921 cactctcatg tagataggaa cattctcata accttggaag ttacctaagc agcccgggct
277981 cataggcaga tagaaatata atatacttag aagggaaata ataacccagt tcaacaaata
278041 agcagctgcc tgttatttgc agatatttct ggtttgccaa tgccaaaaaa gttgtgtttc
278101 tttctttatg ttaagcagtt ggattcctca ttaccaagac aacaaaataa taccggcgac
278161 acacacacag tgtgcggcca cagctacgtc ccagttgtga tctcggcacc gacaccagtg
278221 tcactgcctt tacaaagaca acgacagaaa caacgcggat ggacagaaag actccggggc
278281 atcatgaata aatcatagca ctcaaaaagc aagctgctct ttgtattgtg atgtgtttgg
278341 ccacacttgc acaagcgcag ggatgagggt tcgagggcgg ctgtgagcaa attagtaaat
278401 aaggtccaca aaaataaaaa aaaagttata cctgggtgac ataaaaacat tgtctggcgt
278461 tgttttagca ttttcactcc acgtccatga ggctttttat ctttataaat tgcatttatg
278521 acatttcctt tctcatttaa ggacttttgg aagataaatc atagaaaggg tttttgtcac
278581 gtttaagaat aaaaaataaa aacccaacgt ttcttctgac agataagact ctcgcccttc
278641 ggtgagatag gaaagctttc gtttttgata aataacagcc aggctagaaa taaataaaca
278701 aacaagagtg atgatgtgga ataaatccaa tttgccatgc aggagataat caatacatta

278761 acctggaatc ccttgccaaa gcacctttag tctgcagtac agtatgcgca tgttgcctat
278821 aattgcaata ttactggatg cagaagcggt gggcagacaa ttgatagtag ccattagcaa
278881 atgattaggc ggacagagat gaactctccc acattgattt ggccggctta catttccgag
278941 ttttgcagga gaataaataa atccagcagc aaatctataa tgcatgactt tatattcata
279001 atttccagcg gggaggctcg gcggccgcct gcgtttcatc atgtacgcag agaaaaaggt
279061 tggcagctcg ctgaaaatcg ggatgtccac agtgtccgct ttgtgccaaa tcataactgt
279121 ttattcagcc aaacacggca ttataagatg tatcgctgag gagatttata atgagaatga
279181 cgccggggaa gccacaggct tccattcatc ctgctttcta ggcacgctga agccttcaaa
279241 atatcaaagg ccttcattat catttctccc cctttttta gggcaatatt gtcatttgag
279301 ccatctgaac caccaagtct aatttgttta cttcaagtgc ctcgaagagc gtctttatgc
279361 ttgaaaagaa aacatccctg tgggttgcct gctgccccga gaagaaaaga cagattgcac
279421 aagagaagtg cgaactgttt gccaataact gacacagtga tcctttcttc ccatccagag
279481 ggttgccaag tactttcagt caaactcaag gtctcatcgc aggcccacgt tctgctgaaa
279541 aagagtgtta aaggtattaa aacgcaggcg cgttcagcag cactctctgg tgatccatgc
279601 tcccctgctc tccccatgtt ctcacacaca tgggtaggca gtaatcaaat agagttgggg
279661 ggaagcccaa acctaatttg gctgcttgca gcgctgaccc ctctgacctt tgacattcca
279721 acctaaccgg cgagggtagc ggatcattta tttactttgt caagacataa gccagacacg
279781 gggctttcag ccttatggca agaatctttg gaactcaaaa cacaaataaa agccagatta
279841 aaatgtaaac agccgccttg tagctgacct cggccacaac agtccaaccc ttctgagtaa
279901 taatcagtat tgattggcgg gaaacatctc acgataaaaa ccctcctttt cgttgattcc
279961 cacggaccct gatcaatcga atcaattatt tgcccaataa ctagcggtgg cagaaaatac
280021 tcatcaataa aaatcgtcca actgcagagg acagagcgcc cgtgagggcc aatatgcata
280081 tcaataactg agactttatt ttaaagatat gttagcgtcc tcacaaagaa tctgttgtgt
280141 ccaacaaatg gcattaatcg aagatcgttc cggggtcaga ggtgaaaagg agaggacaga
280201 aacctgagac gccacttata gcagaaaaag gccagcctct ttcttttttc cttcctctct
280261 gatgcttttt tccaagtgtg tctcaggagg attatgcagc cgcggctcgc cacacatctt
280321 atttcagggt tgtagctctc ctcactcttc agacaaactc tcaaagcatg cccaacatta

280381 aaaagcagtt tttggagtta acggtgcctc tgtgcgagcg agtcatcgcc ggcccctggt
280441 cccccgctga gttctcacag ctccttcaac agccaaactc gctgcatatc cccacatgtg
280501 ccattctccc tccagccatc tcctaaaata gacccatcgc tgggcctctg aacatttggc
280561 tgcttccttc ctcccagctt gtctgcgcct gttagagggt tacagctgct ctctcgctga
280621 ccacagtttc cattgctcat cactgtttaa gtcccagagc cctcctggag tctattacca
280681 aggctaactt tcccagattt atcaccgcta tgacctgcag cagccaaaac aacggcggcc
280741 tggatacaca gagtgctgat taggctgtgc tgaggatatg ggatcaatga tcctgcaaaa
280801 gaaattaggc ctatatctaa taaaagtatc tgtcagggat aattattcaa atatggctgg
280861 ctgctgtaaa aaaaaaaaaa gggcacaatt gatagatgca actatgctaa acggtgctac
280921 taatgagtga aatcagcgcc agttatttct gtcaggccaa cattgtttaa aattcacaca
280981 tgagaatgaa atatagttct aatcttactc ctcagcagtt tttttctaac acttttaagt
281041 tcttcgataa gcagagcgcc gagatgggcc gcagctgcca aggaagccct gttggcagca
281101 cctctgtttg tttccacaaa ggactaattt ttagcatgca ttatgcaacc ctaaaaaatc
281161 cccccacccc cacccgtcat caatttatca aaacctgcaa gagggagcgc tggcaagaac
281221 agtgggaggg aaagagaagc tgggacggat tgaggcagcg cggtcatgta tccctgaaaa
281281 taaagggggg aaaaaggcca aaaaaaaagc ttacggggca gacaatgagt cttgagaggc
281341 gaagctgcag tatttttgtt tctgatgacg gccttattaa tcacagtaat gaatcaggag
281401 aagctgggat cgcgggtaaa caaaagcaga tgagctgagg ataaggtggt atccttggga
281461 gatggaagaa gcagcaacga taagctaagc aaagctaaaa tttagcctct ttggtcgcct
281521 ctgtaaggtc aaagctgcct tgtttacatg tttgtttgct gatcaaccta ttcatttgat
281581 gacttgcttt ataacacggt aaaaagctag ctgtgcatcc aagtaattac actgtaattg
281641 atgagttaat agaggtttaa attatgcagt agtgtgtctg cagtagaaag agtggagtcg
281701 gttgtataat tacaaactcc agctgccgcc ctcctgtaca gcagccataa attaggcgca
281761 caaaatttga ataaccgaga atggagccgc tcccacagca ccagaatgag cccggttcct
281821 cgatgccggg ttcttttcta acccttacac gggagcacgt ggttgtcatg agttaggagt
281881 tcatttcccg gtggggtcac aaactcgcac tcccgttact gaaggggctt ctgggtaaag
281941 caatctgcaa acaacatgct gagctgtttt actggtgtga gcgtggtgtt tagagctccg

282001 ctttcagtaa cgtccctgca gtaatgaggc ccgtttctga cctttgaaca
atttacattt
282061 ggcttttaat gatcgtatgt tacatggaag tccttggagc tgttttactt
cacatcagca
282121 ccaagaccac aaacgacatg gtgtcttatc ttttcagtgc tgatttgggt
ttcttttata
282181 tctggaccta aatacaatat agaccttagt ctgaagtcat ttacttttat
gtcagtctag
282241 attgctcttt tctcagggtg atgaatggtc tgaattgagt ttctcgctgc
cagttgtgtt
282301 gaagggagct gacagatgcg gttgaaagta actcgctata cctcgaaatt
ttgcctcccg
282361 atccgggccg cttcaacctt ggcatctgca catcagagca ggaatataaa
gacacaccga
282421 gaaggctctg atggttcccg ttcacgataa gaagatgggc tcaagacatg
ttcagagaag
282481 ccgcacaatt attgaaacaa ttcatcatgt ttctaaccca cgtgtcaacc
tttcccccctc
282541 ttatagttcc atgtggtgct ttctacttcc aaggagaagc tgttggtgtt
tacagtctaa
282601 aaaaggtcag acctgttggt ggcacaggca gaaggaaccc ttgattacaa
aagaggcatc
282661 agaggagtca tccgtccagg ctgacagctt gtagcgctct gatgctatct
gctactggta
282721 ccatcccagt cctcctctgt gctcttgcac ataattacag cagttaatct
tctggttccc
282781 cccctctcct gccctattct ctcctttctt attatccttc ccttgcccac
ctgagatttt
282841 tgtctttttc ccctccaatt tttcctctgg tggtcagcac agcggtccag
tcggagttgc
282901 cattgtttat tgtgatatgt tgggataatg aacaaatgac ctgccacctg
gagcacttag
282961 agcctggtgt taaactcagg ttataaaaac ctcttccagc tctattgatt
tactggcaac
283021 atttgattgt acccatgggg caggggctct gcaaggctgc acagagcttt
tactgtttta
283081 cagtccttat tacaccaaac aaataaatct cctgaccatt aattcactgc
atgcaccctt
283141 gttttccaca ctgataggcc aaaaaccaaa actgatgcag gttgctcgga
aggctcagcc
283201 acattaccag gcactgagag cgtgtaccct ataatatatc agcttaggac
acgacagaca
283261 agtgtgagtg ttttcagaaa actttacgag tctttgggtg aaatacatga
ggaaatgatt
283321 tgagtcgagc taaacagttt ttgcaagcag cggtcattat ttcaatatcc
tgtgaggctc
283381 ttttatagtt cccccacagt tctgttgccc ggctcttcag tgacgcgcag
aatggcaacg
283441 tggcacctca gagatgactg aggacgacca cagagggtca aatggcactc
atttctacaa
283501 tggcgagcaa ctccggggcc ctgacacacg gcgccctcac ccacggcccc
ccggagcaaa
283561 agccacgttg gctagaattt cagcatggat gttaagttgg gatattaaaa
tgtggccgta

283621 aaacattaat aatggtctat ccaataaaat acaccaatta atgattttaa ctttaagtac
283681 gcaagtatat aagtcatccc cccccccca cacacacaca cacacacaca cacgcacaca
283741 cgcacatgcc cctacatcca cacaatactg tgccaggatg atgaataaac tgcagcctca
283801 gaagatgaaa gatttcctga ttagtcactg ccaacatgtt taccatagtt tgaatcacta
283861 ccatacactc tgtggactta tgtaacgcct gctccatcct gctggtttcc ctgtgcaaac
283921 caacacgtaa ttgtcagcat atgtggagga tactcacaat ttctatcgtt cctgtcggca
283981 cccgcgtgat gttcacacag gcacacatgc aaacacacac acacacacac aaactaaatc
284041 aagtcagtag atcctcaaag tgatgtcacc ttcaaacgta aggaacaaaa atctgccgaa
284101 cccacgcaaa actcaatgac tggctcccat taaactgttt ggttagcgca cacaaacaag
284161 cttggataaa cacatttaca cacatacata aatacatatg tacgcactta tccaacgctc
284221 acaaaggccg gcattccgag gaagtggcca cattcggtcc tgattttgca ccatccggca
284281 gagaccagag ctgcaggaac ggtacgcctg ctaatcaaat aggctttgaa cgcacctgga
284341 tggtataggg cagatttgac ttcctctgga ggccaaaagc agcatgtaaa tattcgctta
284401 catgcgagaa caaagtcgct gtcgacgcaa agatttaaag cgggcatcaa tacctctctg
284461 agggtctcac aaccaaagta gttctctacc tgcaacactg tcccacatgg gattaatggt
284521 gatataatgc gctttgtcag ggcactttgg tccggataaa agacaagacc accagacagc
284581 taaccagcca atctcagaaa tactcgctta atgtgacagg ccgtgttgaa taatgaatat
284641 atacattatc acagaggggg gtatcgcagg ggatgccgcg gatcgctctg tgtgtggtag
284701 gtggacacga cgtgtgttat cagcgtgagt tctaatgagt taattcacag ctgagtgtac
284761 atttaccagg ccctgcatgt gtaattatct ggagggtgac ggtatcgtta tcaaaaagga
284821 gctaagctct tatcgatgaa cgcctcccat caaatctctc tgggacataa tgagccaaat
284881 ggaaacatca agagggccag attgatatta aaggcttgtt tttttaaacg ataattgttt
284941 ttagcgcccc ggtttcaaaa catcacacag taaaaatgca ttttcatcca tttctgtgtt
285001 ccccaggtgg atttattcca gtattgtgcc ccattctatt tcctttaggg agtggggttt
285061 gacactacct atcaaccgcg acatgctacc tgtcacagtg tggaacacac acaaacacac
285121 acacacacac acttgaatgt gtgaggcatg tgacatctgt tctctctccg gtaggctccg
285181 cgtgtcctcg acctcatcca catcgcttct aagcatctca gagggaggag ggggagatat

285241 atgacaaggg gacgaaaaag aaaaaaaagc agggatgaaa ggaggttttt
tctgcgtggc
285301 catgcttggg ctcaggtctg ttatggcgag gttagcagcg tggtggacac
aaagcagtca
285361 agcacgacat cagtcaagtc cgccacggca aactttctcc ctcgctgacc
ttgacctgca
285421 gtctgcttct ttttgacagc ataggtcctt gttgacacca gctgtgctga
cacactgggc
285481 cagatgcaca gaaagtaggc tgccgcgagg ctgaaaaatc agaggctggc
ctgaaataat
285541 cccacacact acgtgcacat tgaagaatag ctgcagagtc ccgtgacctt
gttgcctccg
285601 atagcaggct aggtgcttaa atggatagct ttcctgagct gatgctggag
gctggaggtt
285661 ataactgtga gcaaatcaag atgggatctt cagcagcgat gaattaaagg
agagctggat
285721 gaaagtgcaa catgaaagtg ttagcagtgt cacactttga ttttgaacat
ttcaaagagc
285781 agagatggaa agctagagat acttttgaaa atgactcaac tagaaattta
gaaccagtgt
285841 aacgagtcct taagttgttc attttgtact tatgtaatta gctgaatttg
aacacaccta
285901 agagctgaaa aaataagagg gggcattaat tataaccaca tgtttcctca
attaagatgt
285961 caattcttta tattattatt atttcagtgg gtcatggaaa acacacaggt
cactagatcc
286021 tgccagtcgc cctctactcc aaacagaaca tttctttaaa atggatccgt
gcaagtcaaa
286081 gacacgagag taaagagttc atctatttag aataacacca ttcctaactc
acgtgttgcc
286141 gctgccctgc aaaagaaaca gcagcgtttc cctttggtca caactccacc
aactctacat
286201 attactttgg ctgagaggaa gcaaatgtgc ttcccagaca gcttccacag
gagtcgttct
286261 gcagtagagc gaccggctga ccgatgaggt caaagcagaa cggcttcatt
ccgtaccgca
286321 caactttttt tccgtcctct tcggggcgcg atcataacag gaaaatcttt
tttttatgac
286381 actccgatcc cacgcactga atcttacttc gacctaatgg ctactcctct
ctcctctcca
286441 ctctgctccg gtgaacctgc accattatca tcaccagcat gagaatctca
gcagaaacgc
286501 aaccactgca actccaaggg tgaaattccg agatttatct ggtgatcagc
tccatcctcg
286561 gcggatattt gtcgatcaga ttaaaaacaa cctttaatgc tttaatgctg
cagtttcact
286621 aatgaatcat actgaggcac aaaaaaaccc tccgtcaaca gaaaagcacg
gagcttttct
286681 cctcgattgg aaacctaaca gggcgctgaa ccccagagaa agccgcaaac
taaaattctc
286741 ttcgagtttg tcaaagagat aattgcagca gaaataatat gatgttcttt
atccgctcat
286801 agttccatgg tgagagtaga gcagctgctt ccaccagagc cagagtctgt
ttagcacatt

286861 gactcagggt cacgagttca ttgggctcag ctttgcagat cattaggtaa tgaagtgcca
286921 ttcaaatata aatgcatcca caagccagca tcctttcatg ttccaactca taaaatactc
286981 ttttagtttc ttcagagtcc aaataccaat cacagccact tccaacatct gctgcccgct
287041 tgataaaagt gttggatgtt gggtttagtc cttgtaattg tccaccaaag tgttgcagaa
287101 agaacagagc aagagagtta tgatagcttt tccttaatct gagaattgat cattgcgtaa
287161 tctgtcaagg atcttggctc ggagctcgtg cgcatgtgta cacgcagtgg gtgtgtttga
287221 gccagattta aaacacacac aaatatgaag gcatgaggag accagtctaa aacacagcca
287281 gatgaggaaa ccatgagact ctgattattg agggaagcca tcgctgacct tggaagacaa
287341 catgtccttc cccttccgtc catccagctc ccccacacta ttgtggcccc tggcctacag
287401 cttattttgg ggggcctaga gccaattccc tatttcatac agtccctaat ctgggaggca
287461 aacgataagg ctgaggttta caggaggggc tttgcacact caccaaaatt tgcatttaaa
287521 ggccctcaag taatctttag gcttattcaa atctcagaaa gacactggga aaagagacgg
287581 ggagggagaa tcctgcagat ccacctccga tgtttctttc ccatggtgga acaaatcccc
287641 accgtgaacg agccgacggc gtggatggct ccgccgttct cacagcagct cgcttctgga
287701 tcatcaatgc catttaattg tcagagtttt ttttttcttt tacctctttg caagcaaata
287761 aacccaccac gtgggacaaa agctttacac atcagcataa atccaattcg tagataattg
287821 ctgcaagaga ataagaacaa gatggggtgg gggggggctc agaataacgg gggtgagagg
287881 attttactta taaacttgtt taaagccccc ccaaaaggac cgttagacct tagtgacgcc
287941 atatttctgc aggttcttac aactttaccg cctcttaacc gctgtgctgt ttcagaaatg
288001 gctgactgaa caatcatcct gtcccacttt tgaaggggtg tgtgtgtgta cgtgtgttta
288061 ccatccacat tatagcaggt actgtatagc ggtcaacata gtcagttgtt taccgccaat
288121 acatccgaca taacagcaca atgcttctgc tcagccaact cgcgtcagca tcaggggggt
288181 attttcagca tggggttgta tggtgcatcc tacacatcca gaagggattt gaaagattaa
288241 tcctcacctt tccgactgaa tgtatgtaat ttggtttgac aaacaccagc cctacaggga
288301 cgttttcttc attaatactt gacaatgtgc cttgcgtctc aggcctgggg aaatgtaggg
288361 gtgtgacgga cgagagcatt aaagtgaccc agattcaatc tttctctccc cacttgcagg
288421 cttttaacac cgagtacagc acctgtgata caaaattcag ccgagagagc aacaaagaca

288481 gggtgttaac agccattaca acaggccaca gagaaaagcc gcgatctgat catcctcaga
288541 ctgtaatacg gcgtgaaata ttcagcacag tcgacgtctg tagtttgcaa agacctcaaa
288601 aacatttttc caggcggtgt tttggggttg cctcgtggtg gttttgagag gttgccatga
288661 tttcagtata aaggtcagaa aagcaccagc cgggtaatgg attgacactt tttatttgcc
288721 tctacttatg tataagctac agtattgtta aacttctact ttccactgtt agctttgtgt
288781 tagcttctgt cttgctaaca aatcatatag acactactaa actggtctca gcctaatgtc
288841 tgattttata ttctacaatg ttttattttc ttcctccaga actctcttca cagccataag
288901 acaggtcaaa tcaagctcct gcctataaaa gcctctgccc ctggcaagca gtatagatga
288961 gcaccagggc ccggcttact gaacaataca aacaaataaa aactcttccc accgccccag
289021 ttggtggaaa gatgaagaga gggaagaaag gaaatctcgg gactgcctaa tccatctgag
289081 aatctaaccg tgagcttggc ttttcagccg caatatggaa acgctgctcg ttcttccgtg
289141 ccacagccac ttcctgctgg ctctaaacaa tgcagactcc atttgggcca gcgtaatggc
289201 tgactgcttg acataaccct tgtcagtgag agccgagatg ggtgacactt gacagatgtg
289261 taattgctgg gagagctcgc tgacaggtgg atgagcttaa agacacctga tatgcttctg
289321 aagccgttga cggggggggat tacaccttaa agcagcgccg gctgctctga gtgttttctt
289381 tgctgacttc aaaatagagg agggtgggca ggaaatgttt gggtaattta ttcaaagtga
289441 tatcggcttg atttgtttga gccaacacat gagccagcca tgacagccag agaggttaag
289501 tctcagggaa gaaccctcgt ttcatgcatg cagatggagc gtctctgtga tgtaatgcac
289561 catctgagga ggcttgtaaa gcccgtgttg aggcgtgata gctttccctc ccgggtcagc
289621 ccaggtgagg tggtaccggt aggtagagat aacactccgc ccctataaaa gacaggaagt
289681 gagataaaca ttctcctgtt gattaggagc ccggctgcca tgtgggaggt gatgtagtca
289741 tgggggtgaa acagttggat gggatgtgtg tgtataaagg gttgtgtttt acgtgggggt
289801 ctgtaatgca aatcaatgat ctccttcagt gcagagcaga gcagcacaaa gcctgcacac
289861 ctgtccatca ttcaacagct tctgttccac ctcttcaagc tggctggaaa aaaaaaatca
289921 cttcagctca cagtgaagga cttgggagca ggaggagggg agtgtatcca ttttgctcag
289981 aggccccagc cattcaccct ttttttcttt atacccgcca agatgaccac gatattagat
290041 ctgcacttac taaattggag cgaaggagac cagagctttc tgaagcgacc tcaattgcac

290101 cacgacacca tctcaggaaa gtctaatttc acttaaaaag aaagggggg gggggggaga
290161 agaggaatcg acagtatctt ttgtatcatc atacatagac agcgaatagt gatgacactg
290221 gggcgtaggt ggctctagct gtacccaaca aggttggttt cagttcattg ggggtatttt
290281 cacttataat tgggctagct ttcatctcgc ctgccaacag gcatagcggt gaagaagagc
290341 accaacaggg agctatcact tcccctattt cctggcccca gacaaaagca tccaggcagg
290401 cagccctgct gaagatgatg tatttctgat caaagagcct gaagttgtca gcagcccggc
290461 ctagcgaggc aacctgattg ctcctggata gatttgtccc tttgatcaga gcagactctg
290521 actgcaggca ctgtttatgc aaagaaaaga tggcagggag aaatctaccc tgtgttatga
290581 ggaagttggg ctggagaagg gagtttagct ccgggatatg ttggttcata caatattaca
290641 agattatatc tgttgtaatt accgctaatg agataacatt gcaaaaagcc agtttttgca
290701 acttgttgcc gtgatgcttt tatgctgaca gcaaaaaatg tacttatgtt agacgtggtt
290761 atctgataga cccctgcctc taattacagc agcgtacatc agatatatga gagagataat
290821 ggaatgttct tgaacgtctg gcgtttctgc aacacgatct gcaagtttta ctgcgcaaac
290881 atcttccacg taatgcaaat aaccgtgggc tatgaattta tgcggactgg aaaaaggcac
290941 gtagttttgt cataataaat tgatgcaaaa tctcaaggct agacttagga tttagccgta
291001 caaatgtgct gcaaagaaat attaatggca ttagtttgga gccgtgtacc tacaggtccc
291061 tacggtggca gcggccgtgt tttcgctgaa gagaacaaag tccttgtata tgctgacaaa
291121 ggtccgaacg ctgaaatgtc aaacacactc cactctgaaa gcactccggc tgtggggtgt
291181 gatttgttta acacaaagaa cagggatgag gctgaactgc atcttggtgt ctttctcatg
291241 aggctcctac aagatctttc tgtttttggg ggttttttg catgcatgtg gtgaaatgcc
291301 acatttagtc tttaaagtat tgagaaatgt ctttagattc atttaatagg tgcacactta
291361 agagctgtca gaacatacag ttttagtgtg gaaaatctct tagtgccccg ctgctccctt
291421 ctagaaaagt acctgcgatt ctctttcagg aaaagtctgt tttctctgct gtcacgttaa
291481 acattaaata gtcccgctca gcaagctggg tcctgggaga cgtcaaagtc aaggtcattt
291541 tgtggatgca aagtacctga tctgtggttt taaatctctt ttattcatct ccacccccc
291601 ccccccccac ccccattcca ctggtgacag gtgttattta aaggagatcc gggtgcacac
291661 acagtacagt ttgaccacag tttaagtcct ggaaagaggg ctggttggtt atgtaagcca

291721 tgattaacat atctgacggc tgcatttggg tcaaacagtg atgggtaatt
caggtcaatt
291781 ttgacagctg atgaaaacag aaatcatcgt ggcgggcagt gggcgtaatt
atgtatttat
291841 gagtgcaaga ggaccttggg ggaagcaggc agaagagatg agcacacaca
gagcagagcc
291901 tgagtgctac tgtaatgaca gctcatttcc ctgtgcagat gtttttctca
gctccgcgcg
291961 aagcttctgc gagtcccacc agtttgcggt acctgccagc ctggcctttt
atggacaata
292021 caggagggtt aagtgttgta ttattaaggg atgaattctc tgttgagatc
tcagccaagg
292081 aaagaaaaaa aaacagtaca gtaggctcta cccaaagggc ccctccgtgg
cagcttgttt
292141 gtgttgtgaa aaacactgct ggggcttggg cacatttaaa gatgcatttg
atgttgaata
292201 tggggttaat gcataaatac atacatcagc ctcctgcact gaagggcacc
gctgctttcg
292261 gcatcatcgc attgttctga atacttaatt cttctgaaaa tatatacata
tgtatttatg
292321 gtttcgtggt aaatgttgca ttgtggcacg gcgcgctcaa tttgcatccg
tgtccttaac
292381 ccctgctcac tgtcgaatct ggaacacaat agactgtgaa ctggaaatgg
aggatgggtg
292441 aatctggatg caaataacct ccgagttaaa tcaaagccat cctggaatca
aaccgccagc
292501 aaggcttttt cctttctctg tcaatccgct gtgacatttg tgatatgggc
aacactttca
292561 atccgcagct gcctactcca ttggccacgt aaagtttaat gagcaccttt
tgtggagtga
292621 tatgaataat gtatacatta cagagcttta aagcgcctat ggtgcatctg
tctccaggat
292681 ggacggaagc cacagagaag aaggaccggg gaaaattaaa aagcctgggg
gtaaaggaga
292741 gagatagaaa aagggaaaag ttgaaggcgg ccacattaat ttgaatgcct
cagtgtgctg
292801 ctaccaatgc aaaatagaac tcatcagcaa tcgcatagaa attcagcacc
ccccaccccc
292861 cattactcat ctgtgtcaac ctgctctcct tttggttctg agcatgcggt
gacaagaaaa
292921 atgctcactt ggaagctctg caccatttga aggatcctaa gcacaaataa
ccaccatcag
292981 gctggtgttc caggaaagga gaagatggat atagagttta ttttaaaaga
aaagctccct
293041 tttgtgactt taatgtcttc agaatctact tttgccagca tctgggacag
gatctggtat
293101 caccttttca tgaatgtatc tcatcttgat gatcagagcc tcccgttggt
gttaacggtc
293161 attaaaatgc atataatggg gtgagaaaac ttccacagaa ttgagggcat
cttattacga
293221 tttatctctt ctgactgcac ataaattacc tgttggtact tttaacatat
attcccagtg
293281 gaaacaagtc gtatctgtag tgcaatcagg gtgtagaggc acaaaggtca
aatacgatgt

293341 ctccaaggtc attttttagt attatgacac acctatatgc tgatctttgg tgtattcggg
293401 gcattacttg tacaatattg aaatctaaac aacaacaatg taaagaataa tctcgtgtca
293461 gggttaggag gtggtcaact gttgcatgca ggacgtacta gcctcggggg ctttaccgcc
293521 acagctcttc ctgcatcatc acaagcccct cagtccacag cgccttggcc accccaggca
293581 gcagcttcct gctccagacc ctccccaccc taattccaat gattggtggg cttctatctt
293641 ctcagagatc tgctcccctg ttgaataagc aatggaacct ccccacctca cttgtctaag
293701 ccgagcaggc ctgagcgacc ggtccctcct ttacccccca ccacacacac acgtccctcc
293761 cgcccctaac cccagtatcc atctgcgcag attgaccccc ctacccacca ccaccctcac
293821 gctcccctct cagccctcgt tcccagccgg ctccagcaca caaaagaagg ctctggctta
293881 gtggccttgt gatggatgca ttttcatagg ccaatctctg ttatcttggt gaaacaggtc
293941 tcagcctcca ttctctcagg gctttacagt tttttctctg ccactcaccc cacccacgcc
294001 tttccttcat ttttttccca cctcctccct ccatccctcc atcctccttc cccattttcc
294061 gaaggtcacg cattaatact cctgagcatt gacgctgacc ccgtgaccct gggcttgaaa
294121 gcctagccct ctcatattga gcatccaagc ccacgtccca gcattctcgc ccaggtctga
294181 gcgaggcagc gtggatccgc ccccccaccc caccccacca aggtcaagga ctggctgaca
294241 tagggcccca aaacaatggg agacccttcc tcttcacatc cacatggacg ccccctggga
294301 tctccgctct ttagattgat tggctacgcg gaacatctcc gagtcaatca atttcatttg
294361 tttaatgcag atgagacaaa cttgagaatt taccctgaga tctcaatatc tgccagaaaa
294421 ttgaaaaacc tgacaaataa tgattctgtc aatcacagtc caggtgtaaa caagcgctct
294481 gttttcctct agctcttatc aaatattgcc cgttatcttt aaatgaagtc ttgaagaaaa
294541 ctggctgcca ttccatctgt ttaatgaatt taccctgttt ctatcccgac gttatctttt
294601 tatttacttg tagatgaatg agagccagga aggtgcaaat tatatgtaaa gctagcggca
294661 gacggtctcg cttttggctt atttgccgag tggattacaa tgacaacatt tcttgagtaa
294721 ataaaaccat ctatcaaaac ttgccttcag ttccgatgaa gcaaccgtgg ctcaccccgg
294781 gtagaaactg tggtgttaaa acacactccg ccatgagctc catcattttt ttttcgctca
294841 ctcggcagtt tgctgcctcc tcatttccct gctgtggcct ccatgtatca tatatctctc
294901 tgtttttcct gcgtggctct ttttgagcct ggggtcatgg ggtgaaataa agcagacccc

294961 cattttgaag ctgacagggt ctccaaagtc tgaacccgag ggcagcacct cccctcccct
295021 cccctcccag ccccagcctg acagaggccg cgtccgacaa aggtgtagtt tgttacaagc
295081 ttctagggga aaaaaaaaag aaacactaca gggagggtta tttggtgttc ctttgaccct
295141 ttaagccctc tgagatgaaa aggctttcag tttttaccag acaaggaaga ccacttcagc
295201 acggaaccac taaaggcttc aaagctcagc agaattttta atcggagaca aattactgta
295261 tattgtggtg tttgctgcgc tcggtccaag cgggcaaagg ggcaaaaata gcattatcct
295321 cgcggtgggg ggggtgaagc ttccatatct gctgtctttt taaatcagga aaaggcacca
295381 ctcaattatg accagagcag tgttgtgcag gaaagtaaaa acgcggtgag agtaccatct
295441 taaatcacac ggtaaatatc agaaccacta tgctgtttaa gggcatattc agtcttataa
295501 aaacatatcg atcatttaca gcacaagggt aagagggggg aggggagccc atgagcacga
295561 tgtgccttct gccatttact aaacatttta tcaaatttta caaaggctgc ttaatattta
295621 aagcctcggt catgcagcct aatggtgcag ggcaggaata agctattcag gggtcaattg
295681 ttttattaag attaatgaag gggagcaagg ggggacatca tatctgcact gccttccctc
295741 tttctctgag cgccggtgag gtatattaaa agaagcgagt gaggttttgc cacttagcta
295801 atctacataa gtggcttacg gatgtatttg tccaggacgt ttggggtgat tttttttttc
295861 cctccccacc atctattcat ttttcatcaa gcaaagaact cacttccctc gcagaatata
295921 tcaagaaaag cctcagttcg ttttacacct cgccaacatt caagagcgcg agattagcat
295981 tgaggggaaa aggcagactg tgcagttttt gatgttatta tatgctgtaa agccttgtaa
296041 aatgtgcaag taatcttttt taatgccacg cttttcttta ttgcatgaaa aaatgagcaa
296101 ctgtcctttt tttctcatct ggaaatggca acctgatgga attagagacc tgtgtaggat
296161 taaaaaaaag aaaaaagata ttgatagaca gaaaaataga tattccaaat aaagtgttaa
296221 atgatttaat tccctctctt ttgctgattt gacattatgg tggaaatgtt tctggttgag
296281 agtggaatga tgtaacgtgc tgggggagca gtctcctgac acatgcagat gttacgccgg
296341 ggtaactgcg agaaaacgca gagcgtgcat tagcgtgctg atccacagct gcacagtacc
296401 tgtgattaat taccttacat gctactgcaa cagtcaaaca tagatcagag gaagatattg
296461 gattcatggg cgttgaaaat tgttgggctt gaaaaacaga gacgtcacat gacagcaggt
296521 ccagcggggt catttccttt ccaataaagt gccttttctt ctttagacgg agcagaatgc

296581 ggacagtgtt ttcgagggcc atcttcagcc gccatccttg cagattgcga gcctttcatg
296641 tgtcagtcac tctcctcttc atgcgcctca catgttgagc tgcctgggag tgagtcgagg
296701 ctgtggctga cctggaacgg ccaggttgga gaggtttgcc gcacactgtg agggctgcaa
296761 aacaacctac agctgaaaag ttggtggggt acttgagaaa atttcagagt ttagaggccc
296821 ccaaagcagg tgactatcca ataaaccgat ctctggagtc acattagacc tgctgacgtt
296881 acaggtatgg tttcatatca gtgtgaggct tatgttgtag ctcatctgca ttaacaaata
296941 gcagatgatg tagatgtcag gagaatagaa tatgtgcctc aacatgctct atatttgaca
297001 agtaagggcg ggctaagtcc accgtgtgga taacaaacta aaaacttcct gcataacatt
297061 gataagcacc ttgtacttta cactaatcct ctctgttcct aaggatcggg gtggtttata
297121 tcaaagaatg ttgctctgga ttgtacgaat acccagaaga cgcaaacctt gatggatctt
297181 gatgtgatca tactggttga ggatggagaa tgaccagtag agaagtgtac aggcgggttt
297241 aatgctgtgg ttggacttca cggggagatg ctttcgtctg cgttgctttc accgatgagt
297301 gaaacagtct tggaccattc acaggcaaat gatcaataat tctgattgca gagggggaat
297361 attgtcgttg cgatgtcgcc tctaatgagc ccacctgttg gcatcatgaa gaaaacaatt
297421 ttccacttga tcgcatatgt aaactcacat taaacgtaaa accactgaaa tgtgaggcag
297481 ctgcagccca gaattatgca gcatccggta aagttgaaca aacgtacaaa acaacccgtt
297541 tcctccaaag aaatagcaac ttgtctcttc tcacaaaaga ggaaaaagtt gaaagcctgg
297601 agcatcattt tctattgctg tgaacttatt gaaatttgaa actggagctc agaccgagac
297661 gcccagagta actgatgttg catttattag atgtgtttgg cttactgttg ggaatataaa
297721 aacctgtgaa gtttgaacta tgcggagcac cacaaaaatg gaatatgggg tgttttgttt
297781 ctgcactgca gctcacttca aaggcttgtc ctgaaaatga ggaggacagt tattgatcca
297841 atccttcagt aaatcaggaa ctgataactg gacactgcag aatgcacttt tatgtcttct
297901 cactttgaag ctgtccaacc tcagagaggg gaggtttttt caccacagtg cttcttaaaa
297961 gtttaaaatg cacactttca ctgacagtct gtatcggcct gtggctccag ttccaggaac
298021 ttttattacc aagaattttt tttcctgggt taaaaaaaaa aaatcctggt aatttgtgcg
298081 gtttgtattt ccaccgcact gttctggaaa agggattcaa atcaggctga cgaggaggag
298141 tgcagccgtt gggcccaata ttgttcataa tattgctcaa taccgtttta ttgtttattg

298201 taatattgtt tacttttctt tgacttttcc tgaaacttgt tgtgaacaaa caggcttttc
298261 ggctcggttg tttggagttg ctgaaacact cgagagcctg gaccccatcg cctctccacc
298321 ggcttctttt ctttcactca gtttccaaat aatcgcaaca caaataaagt gaagcttgta
298381 taaatagcaa taaaggcacc aactgtgttc caccaatgag cattcttcag tacacagccc
298441 tcaagaatct aaaaagtcct attttttggt caaggaaagt ttggagttgg cggggggga
298501 atttctttcc tgtgaatatg cgctgaggtt tttctaaatt gtggctaaac aattaaaggc
298561 actgcggtgg aaaaggggct attatttgtt ctcaaagggc ttgttgggaa tcagttttcc
298621 gagtgtaaat ataaattagt ggggacgacc aaaacgctcc tgtctttgtt ctggatgagg
298681 ctgaacaggt tccaggacag gtcaggtgta actgaaagga gaagcctgtc cgtttggact
298741 ccgcgctccc ctcgctccca ccggggcctc tttaaacctc aacatctgtc ccgctttacc
298801 actcaattgg tggagaaacc cctcgcatga cggacacacc gcggcgcagg ccagcgttta
298861 gaaactgctt ccagacatct ccgagcgctg tcacgtgtaa ttgtgtgcgt tcgccttccc
298921 gtttatttgc ctacgatttt ccacaactga tcgcaggtgt gtttgcgcga tcgctacaag
298981 aatatttata cggacaaagc ccacaaagaa ccaaagcacg atgcagttcg gaggaaaata
299041 tttacagagg cataaactgt tgtcgctgct ctgaaagcca tttgtttcca agcgtcacgg
299101 tgagacgcag caagttaaat ttagatgatg agctttttca cagttttatt tatgtagcct
299161 tcgtacgtgg gaccaaacca agcagacagg gtccaaacag gaagccaagt gattctgcaa
299221 caccatttca gacatcataa actcatgcta atccaaaaca ggatgctata tattttgcaa
299281 aagatgtttg gagcagctag cacattgtaa ccaaatgcca gtcttccatg tgggagagaa
299341 aacccgtctc cgtgtgtttg attggtaagt gtaggcctct gctgtctgct gcacagccga
299401 tttctaccct ctaactagac aagagggacc agagcaaaga gagagacaga cacgaagcgt
299461 caacacgtct gcacagtcag tgggtccgga gtgtgtgaac gggggaaagg acggagctcc
299521 atctacacca gctgtgtgga atgaaaagca gctttggttt gtcagttttc tgactcgcgt
299581 cgtgaaccgg gagatgccca tggctgcgcg ttctactccg gcgcggtcca ctacctgcca
299641 tttctccccc cgtctactag gcaaaagatg gggaagacac acaaatccat acatgcatac
299701 gcaaactggg ggtccctcag gtgtgtttgt gtttgtgagc atttgttccg aatcaggcac
299761 ctggcacctt ctgggggggg aaggggggggc tttaccaata ggccccccctg gactgtcaca

299821 tgtctggatg cagaccggtg cccatcatgg caatcccatc accccctcga acctcacggc
299881 ccaataggca tttcactttt ttatccacac acacacacac acacacacac acacacacac
299941 acacacacac acacacacac acacacacac acacacatca tgaaagcttg tgaatacttc
300001 tccactgatt gaatattcca gacagggttg gaatttaaat tcaccgctgg ttaaattgga
300061 ccctctctgc cgactgctgt aataccccat agcaggtata atcaaagtaa ttacaacaac
300121 acacaggtgt tccacgccgc acgccactgc acatgcaaca caagtcgatg acagtacacg
300181 gaataaacgg gggaaaaatc aaatcatgtc ccattacagc tactttataa gcaaacatga
300241 aatgcacaaa caaattgctt tttttacatt ttttttttaaa atcatggaca ctttatgctc
300301 cgtcacttca agatgctttg ctcccacttt gccgttatta taatttaaat ctgtaagagt
300361 gaattataga ggaatggcat gattttttcc acgctcagcg ccgaaggaga agcagatgga
300421 gctacgagtg ctgccaatgg ggctctctca aaataaaagt ccatcattat tctgtctaca
300481 agggtttttt tttttttttt acttttcact aaaatggacc aaaacaccta gaattgtagt
300541 ccgccagagg taccgctggt ttagttggct cggatcaggc agcagagaaa aaaggcatac
300601 aatcacatta aaagctcccc atctgcatcc tccaaatctc atttctttga gaaaaaaaaa
300661 agagggtggg gggagtgtca aagaaaagag aaggtgcaat taccacaagc atcgtctgag
300721 ggatcaactt cacttcaaag agacccgggc cctgtgtccc aggctccaca cagccccgag
300781 tatcagcgcc gtgcgagtgg gtaaacatga gcagatcaca agacttttaa tagcagccag
300841 atctgaagag gtctccacct tgctcctgcc tggaaggaac agcctctccc ctgcacattc
300901 agctacttca ttgaaaagca cgagcgctca agagctttat ctcaacagcg cttcctgctc
300961 ccgaggacca gcgcacaacg ctaaaccgcc acgcaacagc ataaacacac attttgcaac
301021 tgtgcgagtc cttgtgcggt agttgccgtg tggacacagc ggtcgcggac accactgcat
301081 ctgtctgtgc tgctgaatta ttgcagcgct cactttgaga cttattgcca agaattatct
301141 tgttccagta tgtggtcgac tttgtgagca ggatttcatt catctttttt ttggggtaaa
301201 aaaaaaaaag ctccatgtct gagccctccc tccccgaaac aggctgtacc aatccgaagc
301261 tatgtctact gtatgtgaag tgcatttaac gcccactaat ctgtcagcac tggataaata
301321 cgataaggat agctaagagt cccttgacgg tatcgcaggc ctgacggcta aacaggatta
301381 aacagcttta tgcaaagact gaatagcaat attttacagc gctgaccccc ccctcctccc

301441 ctcgccaaat acgtaaacac agcatacatt agtagtgggt aaagctgatt gttttccatt
301501 atccataggc tattttcaat cacatgacag ggttcagaac cccttgtcag gacgcattca
301561 ttctgttcag atgtcattga acaagacgct gggtccctac cagctctggc tgtgtgtgtg
301621 tgtgtgcgcg tgtgtgtggt gcgtgtgtgt gtgtgtgtgt gtgggtgggg gttatgcggt
301681 gtatgacccc cgcactctga cctcccccac tgggacagca gggcagaaaa aaagaagctt
301741 tccctggagg gatcaataaa gaattacatt agcatacatg atgtatatca aagtaaatct
301801 tggaccgaca tgtcttctgc attgctaatt ctctaagtct cggcacactg tggattgagg
301861 caggaccggt ggttgtcttt taaacccata agattggtga agcaatcaac tgggaaggct
301921 gacatctgtt ggccgagacc agtaactgca gcagcgtgga tggtgtcgaa cgtgcagggg
301981 gcagatgtga gcggggtgtt gctgcaggac cgttgccctg gtagaacagt gcagaaaaca
302041 cttaaagaga cacaacgggt gaaactctca agtcccgaac aagatgaaaa gatgcctcca
302101 ggctaacctc acagctgagg cggaatcatc cggagtgctt ttagtgtgtc tctctcctcg
302161 gatgtcgttt gttaagagac tacacccctt catgcctgtg tgaaacttaa ttcctcgctc
302221 tgtccttgag cagtaaactc tcacccettc cctggcggct gagagtgacc cccccaccct
302281 ctccgtttac aaaaaaaccc acacacaagc atagctccgc tcacctctct gactgcctcc
302341 acaccagatg ttacttttcc gtctgtgatt tatcacacgg tttagataag ggcaaacaaa
302401 tagtcattgc agttataact gcaaacaccg cttttctgcc aagcttatca caggtcaaat
302461 acaaagttag ccagagacgg gacgagggag cagggagagc aaaccccccc ccaaagaaaa
302521 gtcaaaattg catttttcta aacacaactg tccttctgcc cacatgcaac tgcgacataa
302581 cacgtaaccc cgagccctga cctgtaggaa tgtgtgctct ctgtggaggg cgattagccg
302641 gctagccctt tggctcccct aacacagaaa gctcattgtg ataaagccca gtcaatgttt
302701 gctgacacac gttatcgccc gtcccctctc cctgggcctc gggtcggatc gagccgccgc
302761 cactctttgc gacgatttaa gggagctgtt tgaacagtga ccaccttacc acacaaggga
302821 cggcaggggg gttggactcg tttgacatat gaatctcaac gtcggcaggt tttatctgca
302881 acaacgaccg tttgtttttt tgtttttgtc ttcgaatctc cgattatcac gggacaggac
302941 gataggaagg aagaaggtgg ggttttgtca gtggcccttg atacgtctgt gagagaagga
303001 aaagaaacac ttatcttgtt tgtgtgcatt cagaggaggt gaggtgataa aggagaatat

303061 tcctcaagga ggatcaataa ttgttattca gccccgcgcg ggtccctcta
taggaagcac
303121 cgacactgtc ctaagagtct gctggccgta ttgattttaa tttatttttt
acttagtgat
303181 ggatatggga catgtcacga aagagaggca ttggtcaaat aattaccaac
atctattcat
303241 taattgggag gcaaaagaga tagaccactc aatattcatt aaggcctgca
aagactttgc
303301 aaacgtgctg tctgacacaa gggtgtcaga aagatatgtc gatgccgtaa
tcacggcatg
303361 cagatttggc tcctgtgaag tggacccggc acgaaggttt cggtgaaaaa
gagaaaagaa
303421 agcttcttaa atcctgattt tattgcattg atcaaccagg actggaaaac
ataaagtgtt
303481 ctcatttcca aatgtttgtt ccaaatgcca ggacaggacg tttcaacgcc
cgagacagtc
303541 agcactttct gaaaggctta caaattggat ccttgcttaa tcctttcaca
agttatggaa
303601 atcaacctca aaataatgag cggcgattga attcaaaggc tgtaagagcg
gcctaattaa
303661 ctgagcatat aaaagtgatc agaccacagt gttttggagt tgcaaccctg
gaaattttgg
303721 cctctggaga atacaaatga gtatggagca gttccagaat cagcgccctt
gtcatcatat
303781 atactttgtt aatatacagc atcaaaacac gtgcagtatt ttttttctgc
gcctgaaggc
303841 aaactgacta agtccctgct gggctctctc agacagacac ctgtggttat
tgagtacatg
303901 acaaaaaaag taattatctt gaattcaagc taaccctttg agagaaccaa
acagctgacg
303961 aaaggctgaa taatcacacg gagaaaatta gcctggccta gttgtttgca
atttggttga
304021 agacttttga gacgtccgaa gtgctcaact gcgtggccaa tctcgtgttt
tgtgcgtgat
304081 ttacaatgta aagctgctgc tgtctgtgtg agtgagcaca tccaagagga
gcttttgtga
304141 gctttgttgg ccatactgta aatggcgaag gggtaacacc cccccccca
tccctccttc
304201 cacagctgag taactggaga gtggagaggg agagttattg ctcactgcag
gagggccagg
304261 ccaacagcat taaacattaa gatagagtag atttggtttt ctccatgcca
cagtggctat
304321 gtttactatc cttttctgtc catataaatt atccccacca aagtaagctg
agtcgaatct
304381 ttcgtttctc aaatatggca tttatattgg tcacattttg gccactgaag
atagccataa
304441 tttaaataaa gatgtcacca agtgttacct gtattcggac actctgataa
tataaagccg
304501 caggatgtaa gtaccaaact ttacctgagg agagcatcat tacgcatgaa
tacaaaaaga
304561 atctaatgga tgcaagcaca caacctggtg ggtccccata actacaactg
ctgtccatgg
304621 aggacctttt tggagttccc atgatgctaa ttaaatgatt taatcttagg
acaagatgcg

304681 atcattgata gtattaaatg gagacctgtg atctacgtgc tgagataaga ccctcatctc
304741 atttcatccg aagatcatct gtaaccttga ctgatgccgt ctcatcgccg ccgaaggtgg
304801 tcggatgacg cctgattgat gtcggcgcgc ttatgtaatt gtgaaaggca agtttcgcct
304861 ctgctcaagt aggctggcgt ttacacgtct gcggggtggg gaagaggcag cgacttccag
304921 aagctttctg tgtgtgtgtg tgcgtgtgtg tgtgtgtgtg tgtgtgtgtg tgtgtgtgtg
304981 tgtgttgtgt gtttcacatc acatgccaca tcgcagcctc cttttcccac acagtctggg
305041 ccaaggtcgt ggttaagtta tgattacact atgatgcact cagcagagac acgtaataaa
305101 gaggtctctg ctgaaccgta aaactgtcaa gatatatcac aaaagtgtca gattcaccac
305161 gcataacatc gcaacagata aataaataag actccctttc ttccactctc ctctttaagc
305221 tataaagcct ctccacttga caacagctga gtgttgcatt taatgaatat acaacgaggt
305281 atacatgcgc ggatggggaa gaaatgtaca atggctttaa aatcccggct ctgctgttct
305341 gctgcgctca gtaatgatca attatttggc ctcaaactgg catttctttg ctgcgcaaca
305401 tcaccaaagg gaaggaaccg ctttcatccg cagctgttgg gctgcaccac ttattctctg
305461 atgcaccaaa ccgagggagg gcctctttct cgaggattac caggactgaa cctgtggcac
305521 ggtacaattt tctgttgaaa atgtgcatta agctgtggaa atacgagctc taaaatgata
305581 atctaatgcc ttatgatgta atccgctctc aataaatgta tatttttcct tctatgttca
305641 cataaaatat atatttatat atattttttt atttatatat atatatatat ataaatgtct
305701 tttttttcct ccctttcctt acggtcgcta tggggacaaa gacatgtgac gtgttgccac
305761 actctgtttg aaagcacgac aatcaatcgc acttcactac aaggaagctg cactttggtc
305821 caatctttaa gcctacaagc cccccgcaca cacacgcaca cacacacaca cacacacaca
305881 cacacacaca ccatcaacca aactcgcacc acaatccact ggaccgacat ggggaggcgg
305941 cctgaatgca ccatcagcgc tgaggtttgg ctgggccggt acgctgtggg tattatgttt
306001 caaagaggag cgcaggggtc cttttttct cccctaacaa ccccaaaagt cccctttttc
306061 cccttgtgct tcaaggtttg caatcagaca actagagttg tcgcatctta agtttggggg
306121 acggcagaag aggtcatttg agcaatgaca agaaaaaaaa gttgtggggg tggagggggg
306181 tggaggcggt gagtgaaaga gcagaaagga gagaggtgcc gggggcaggt gaaagctgtc
306241 gccatacaag gtggcgatta ggagaaagtg gcccggtgtg aggcaagagg aacatctgtg

306301 cctattacca cagcacacaa aagaccagaa gggggggagg ctggagaata
tccacggtct
306361 cctacatgtg cagggcccca ctaaacacca cacgcttgat ttcaatggtc
caatttgagg
306421 ctttcagagc cgcctgtcat tagcatttga aaaagcgagg cagaagccgg
ctcactgagt
306481 aactcgtggg atggaggaga gagacttaag agggtataaa agaagacccc
ccccccacac
306541 acacacacac acttacaggc acatgcctgt tcagcgggtg atgttgtgga
gtcacattga
306601 atgtttattc caacctccct aataaaacag gggagaactg agcacaagtc
cggctgattc
306661 ctgattcctg tctgaatgaa aagaaacggc atccatgaat ccgcaaatta
ttcagcacca
306721 agaaactatt ttatttggac aaactgacct aagagtctat aggtcaccgg
gtgtaggctt
306781 cgataaatgg agtaaaagca gccttgcagg tccgagcaac acaaaagtga
atcctaacta
306841 ccggtttgaa atgggtaaat ccattcttgg ccatttttgg gaggctggcc
aaactataaa
306901 ctagatcata taaacctcga ttggaatcac aagtttgtag accatgcaaa
tctctcatca
306961 ttaataagga tctcctaaaa aagcttcctg gttagctcag gaaaatatat
ggggggggaca
307021 aacgatggac attcctgcag ggctttcagg caagagcaca agagtccttt
ggttcctctg
307081 atgggggcag ggtcaagaag ctgaccgaat agtattacat cacctgcagg
catcttaagg
307141 cttcagtgtc tgagaaattc agggttcctc ccaaagacgt cactacagtg
gccgtcttat
307201 ccgctcagag cctgtagacg ccctctgctg ctgtgatgac tcaaaatctg
ttcatgcctt
307261 tcaagaaaaa gagctttctg ttgctgcgga ggtgtttcca ttgtgtctga
tatgctgccc
307321 cggtgtgtca ctacatgggc aacaagtcgg tgcggtggga ggtgcagtct
gttccgtcct
307381 cttaaaacac ctgactgtga gaggaaggga ggaagtccat cctagaaggt
aaaaacagga
307441 ttctcaccgc cagaactttt cctttaactc ccgtcataac aagtgcacgc
tgccggagag
307501 agacgcacga cagttagcaa ggcgccacgg actcattttc cggttgcatc
catatgcatc
307561 agcgactctg gcgtctcagg gcggcagatg aatgtgactg gaccattttg
aatttagaaa
307621 aaaaaaaact gtgtcaacct ggttcaaaaa cggctaatgg gaggccaagg
catgaaaaat
307681 aaaacactgg ttctggtgag cagagcatca gacctgggta ctagattgca
ctcagtatcc
307741 aggttttttt tagtattctg gaattgaaaa cacttgggaa gaagatgtat
tacttattta
307801 aatgccacaa ggcatatatc tgtttactta aatattccaa tttattccaa
gacggtaagt
307861 gccttgctga agtgaattat ggatggtaaa tcaagacact gagttattag
ttatgacacc

307921 ataatgttct gtctttgtca acatctctct ctctgagatt gctataattt agctagggag
308041 aagaagctcg ggtgacgcat cagctttaac taattgacac aataggattt cctttcagca
308101 tcactttagg acaaacgggc tgctcgatga ataacgattc atcaatattc cgctaatcct
308161 gttgctggac gtgcagcggc gttttactgg gcgattcacc tgagtcattc ccatgattcc
308221 agtcatggcc aggtccacgc taagccacct ggaccctgac aggaagtgac acattaccgt
308281 ctcgtttttg cacattatta ttcaaggccc agggagcggg aacagacaag tttgcacctc
308341 ctgctaacct gtggggatct gcgagggtac gagtacagca caaaacagga agtgcaaact
308401 tggcatcgac agacaaattc ccagtgaaac tggtgcattc tggctgtttg taaaattgat
308461 ttcacagcaa agcgaatggg aataattcat aagactggga tttacagttg actgaaaccc
308521 agggtcactt cagagacagc tcgatgtgct agacagcaac agggatgctt caaatttgtt
308581 tgaaatcata gtttttttcaa gccacgataa ccttgtctgt gagacacatc tgtgtgagca
308641 gcagcgccgc agggtgggat ctatttagca gaagcgagga acttttgttc tttaaccaca
308701 acaagaacta acaagaactt ttgaatccaa ttcagtgcaa ccgtgttccc ctctgagccc
308761 acgtgattaa aaacacgcat ctttaaatgc tgcacagctg caagtatctc tatgttacag
308821 atgggatcac aataggtgac agccagtggg aaataaccac ccctcctccc ttttatttac
308881 cataagcttg aggtaaatgt gcggaggcat aacaatacat tatgcatgtc agagaagtaa
308941 tttggtccta tctgatgaaa tcctgcatta gtgaacatta aagcaggtag gttcaaaggc
309001 agggacgcaa cgacgggggg gcaaatgtga atctatgaag atcaacaggg aagagcaagc
309061 tgccgttaac ttgtgctctt gcaggccttc caacagcttg tcttcactag cgttagccgt
309121 gaagtgagtt ttgaaaggaa tattgagatc cgtcccgttt tccaagggtt tcaggacaca
309181 tgaatgtttg agacaccact gaggtgatgc aagtgggtcc cattgtgccg cagaagcgtg
309241 cggtctgaag gtcatcagat ccatccatca gccggtccaa ggcactgccc cagttgacca
309301 caggaagccc cgtgtaaaaa aaccactgtc agcatgttca tctttccagc acagagcaaa
309361 tagggagcat ttgagtcaag actggtcggc ctggggggtc acaacatggg caaaggcgct
309421 ctgaagagac acgtgtgctt tgacagactg tatctgcgct tcactgtcca gacttacagg
309481 ataatggtta tttgcatcct cgcagattgt gatcagtgca cgcagctaac ccctccacga

309541 gtgcagcagt gcaggcggcg gctcaattcc ctcgcagcgc atgacgtaaa
tcctccacgg
309601 tgcacattta atgtgatgcc attgtactat cgacatccag ggatggactg
gaggccgatg
309661 ctgatttgtt taatatctag ctgagagaat tctattgact ttcctttatt
tcctcatctg
309721 ttcacaagat gaaaacatat cttcccaccc agaagctttg acccactatg
cctttgaatg
309781 tttttcagaa ggacacggtc tacctcgcat gggtgtgcac gtctctgtgc
cgtatatgtt
309841 tgtaggggtg tgtgtgtgtg tgtttagctc cattcaagac ttatatcgat
ataagccagg
309901 cttaatcctt tcactctgat ggcttcaatg acagcagcac gccatcagat
aagaggtcat
309961 tgaactgcag cagacaactc ttcattcaga ataaatgggg tgcacccaga
gtgcacctga
310021 ctatgtgcca cattgctccg gtcataatga aaatgtaatg cattttggaa
tccagtcgcc
310081 atcacggcca ccctgagctg cactgattct gttgataata cttgtccaac
ttgttcaatt
310141 tgttcccaga cacggtacaa aagaggaatt tcaagactcc gtatgtacga
agcggttcgt
310201 cggatccctt caaattcaaa cgattcaatt tatccctttt ctgttattct
ttgctctgca
310261 tcatgagggg aaacatatgg tggggtgttt tttgtggggc tgttttgctg
ttgactgcag
310321 ggatttgcag cctgaaattg gagggaaaca tacagtaggt gcaccttcta
cattccaaat
310381 gtatcaactt gaggccagta tgtggtacgg tatctatcaa agtcgatgtc
agactaatgt
310441 tctttcctgc atcttgggca cagcatgacc atatggaagt ggagatgttt
ccttttttctt
310501 ttttttcccc ttgctaacca gttgcgttga aaggagccca tattgtgaag
taagatttat
310561 tcttttcctg caaaaggaag ggaggagaaa agcatgtaat atccacatgg
ctttgagatt
310621 tttctgcttc caatcctgcc gagcaacatg aaaaccagcg accacggtcg
tgctttgatt
310681 gaaggtgggc tgacacatgc taatgccccg attgtgtttt cattcggagc
tgctacttgg
310741 cggcgccttg tcgtttgatt tcctcatgca tcgaatcgct ccgtgtgaac
cgccgcggtg
310801 tgcgtttctg ccttctgatg cctcaccaag cacctatctt tgagcaatca
tctggggacg
310861 aggttggatg tttattttgg cagaaatccc atcatgcatt catgcctatt
tcctttggtc
310921 tgagaagtgg ccctgagtgt catgttatac aagaatgaaa agagcgggca
tgcatataca
310981 acacacaaga cgtccacagc tttcacttca gcaatttgta cttttactca
tatgtgctct
311041 tgtagaaacc tttttgtttc cgttctatac aatcttgatg gctcaaagcc
gttaaaacca
311101 cctcttttta atggcagcat gctaatgtat cacaagagag cagtcctttg
aatttcattg

311161 atttaaattc aatcctaact taatgacagt ttgagaataa tagcgagggg cacatgatga
311221 aggtgtgcgg tggctgtgtg gtgacatctt ttgttcattt ttttgtttct tctttccctt
311281 cccagattgg caagataatg gcaagggctg ctgctaaagg gggggggggg gtcagaaaaa
311341 gtggagctta gtgcgcaggt tatctttcat agtgtacagg ataatcacag agtggtgaga
311401 gctcggcagc agccgagaca tatcttcttc gagatcaagg tcaaaacaac tcaaaggaag
311461 gtggaaattc agtttcaaaa acactcacag ctgcctgaaa catattgatc tagctccttg
311521 ccccctctac ctctaaactc attcctctga tagactatgg tagaaataca ttattttctt
311581 gtcatgggca ccacaatgga tcacggttaa gaatatattg cacttaaagg ggagaggaga
311641 gggagcgatg ttaagagcct ggtgtgctct atgtggagga gcaatctaag aagtgacctc
311701 tgcagccaaa cccccccccc cccactctct gagatttcca tcactcacaa aacgcgtggc
311761 cgccaattag gcgcatgaag agccgagaca tcggagatcg ccgataggtg aggtggttct
311821 cagaaaatgg caatttgtca acggtgcaat agctataatt agagtttgga gtatggatga
311881 gtgccgcccg tccagcctgg actccagcac gtctttaaaa atgcatggct gtttcataca
311941 acagaatcac tctgcagaaa ctttacacag agatcaataa ccgtgcagac tagcgttgag
312001 ccctccgagg cgctgcagta agctcagtca ctgatgtatc tgagccgagt ggcctttgca
312061 acaaagaaac agcaactgcc tcttattcct ttgaattatg cccttggagc gcggagcaga
312121 ttcccaccaa agagcatcta accatttgat tgaagtgctt gatgtgaagc aaaattgtta
312181 ttcttaattc acaaagattt ccagatgaag tcatataagg caggatttgg ccagtggccc
312241 attttgcatt tgaattaaat cctctcaaaa tgataaaagt ccattttggt gcttagcagg
312301 ccagtaatgg acagtgagca tgtttatgag gctccacatt agaagcactg tcaataacaa
312361 gcactataaa cccatttata gtcacgtatt gaatttgagt cttctccagc tggcctcctg
312421 ctggatttcg ctcagacatg gacgctccca agtctcccac ctctcctcct ttgttctgag
312481 cacaaatcct tcttttatta cccagacagc agcataaggt cctgacccca cttccccgcc
312541 gtgccttcct ccatgtttga tgccttcctc ttccaatcct ggattattgt aatactctat
312601 gcacttgctt tgatgtaata actatgtgca acttgctgcc agggtggaat ttgatggaag
312661 gatgacccga cagatgcaaa cgtgagagaa gacagctcgt gtcaaacgca tcggtgtctt
312721 gaagcatctt taagagactc gtggaaacac cacaaatatt ccactgcagc ctgttttgtg

312781 acgataataa tgagaaactt tccaaaaaat aaaacaaaac caaaagccac agcagacttg
312841 cctgtctaaa tgcatggatt gagcaatata atcaatttgc caatgtcaaa ttaaggaata
312901 agtggcagct ctgtagtgta tgcagtacag tcccgcttat tattttctgc cagtttaatt
312961 tattgaccgg ggacttaatt gatttagtga cagtgttctc aaagtcacct cagctgcaac
313021 gtttcagaag agcaccatca gtgagcagct catccaggta attaaatgtt catcagataa
313081 ggatgaatca cggaattatt cccaggcagg aaaagagcta aaacattttg gatgatggaa
313141 attctgatta ctcatgtaga ttctttccat gtctcagctc ttgatctgag ctgagacatg
313201 gatacagaac atgtcagtct ctgcaaagtt gccttttatt tcctgagaac aacccaacag
313261 cgttgtcccc tctgctttgc tttggactca tccgtcccgg gcgtgcacgt tcccagcctc
313321 catcttcacc tgggcgcacg ctaacacggg cctgcggttg ccgtttggtg tcataattga
313381 gtgccgccca cggttgtcga gctgtgtcag tgtcatgtta gccggcgtac cggcgagtcg
313441 aagctggtgg gatcaggaaa ggtcagagtg gcggcgtctg ccctttcaca tgtccacagg
313501 acacaaacag cccctcatga ctcaaaataa ctgatctgca cctccaattt gagcctttag
313561 aacattaatt tcacagacta gttgcaaggt ctcctttgtg aaccgctgct gaacgcgcac
313621 ggtgggtcaa aaatgtttgc ccaagtgctc ctgaggccga gtgcagctcc acagcagcaa
313681 actcccgaca agtcctccaa attaaacaac ctaatatgtc acttgtctgt gccctcaagg
313741 acaacacatg aaagcatttc cagccggcca gcggtgggac aacataattt attacacacc
313801 ttccaaaatc aatgttgaaa aataaatcac tgttgccaac atgacaacac tgtagctaat
313861 agcattttag tcgaacgccc cacaaaagca ccttttccac acatctgcaa acttgatttc
313921 cctggatatt tcccttttc gtgtgtgtgt gtttatacat ctgaacgtca cttggcacaa
313981 caatcgccct tcttgctatt gtgaaaatga agaacgcgag tcgtgatgca tgttaatccg
314041 tagcgagggt ccggcacctt gacacatcaa cgcggatcgc cgcgttcctc cgcacttccc
314101 acctgaatcc agcggccctt cgtgtgtgaa agttcctgaa gccgcagtga taacagtgtt
314161 ggggaaaggg atgaaggctg acaggtggag agacacaaac aggaaacggc gctgatcctc
314221 tgtctgccaa acccttctga tacgtgtcca aactgacggg gctgagacaa aaaaagaggt
314281 gggggggggg ggggggagca agtcccaatt tatttcactc agctgcgagc ctttcatttt
314341 cgtgactggc gccggtgcgc ttatcaactg cataactatt agccgcatca aatcaatgtg

314401 ttcattgctc tctcttgcct ccatcactct cctcccatcc ctccctcccc cccccccct
314461 cccctctcat tctctccctt tctaccagac aggtcagata ttagaaacct gtattatcag
314521 gtcttcccag gatgctggtg gaaaagttga tttgcaacgc ccgattgaat ctacttatcc
314581 ttcatccctt tccagccccc aaccagctgc cccagcagac aggagcccaa tagcctatct
314641 ataccgaggt gccaaggaat aaaaatatct atctgcagaa agagggggag ggaagaggag
314701 gccgagagag ggaaaagagg agagcgacag tgagtgtcag cccacctgtc acggctttgg
314761 acatctaggt gttggcagag aggggtataa atgttcactt tggttctctc ccatcccaac
314821 ccagacccca tcccctttgc aaagctttat ctactagcat tatacagcca tttatcagca
314881 gcaaacaaaa catgtcaccc cccacccctc ctgaatacca ccacctctgc acacacacgc
314941 aaatggaaaa agctttcagg cgatgttggt caaagacggt gcacagagga aaggtcacag
315001 agggaattca ttaaaagact gtaacgtgtg ctaattgagg ctttagtcac aataaagaga
315061 gcgaccagaa atattcatct gccgacccgg ctgtcagggc cacaaaggga gacatttggg
315121 ctttatctgg gtaaatagag tgaagatgag ggatcaagag gagaaagagg tgagtgggcg
315181 aggtgattgc cagggaggca aaacagtggc agcagatggg ggccagtagc cgacgggagg
315241 acgtgtgggc cggagtgctg cttaatttgt gaatgcaatt actgtagacc tcccaagtct
315301 gaatgatgaa tcaaatcgac gctgcggtgg cctgcgatca ttcagcatgc ttcccattca
315361 aagcacagag taatggagag caaggcaggt gtccccggtg aaagaggtgc atcactactg
315421 agggcgccgg accacaggtc ctgctgcagc gtctctgccc ccataagcgg aaatggagac
315481 gtcactggac aaatatcgag gggattctgt gaggacagaa tgaacgctat ctctgtaaat
315541 tcaacccggt cgtgtttctc tcagcaaatt cccagagcct cctccagttg gcaattaaaa
315601 cctaccaaag gaagcgccat tagcgcctca atgacttttg tggacgcttt ctgacatctg
315661 agccaaacaa tgtttgacat ccgggtcttg gaaattttta atggagttca gttcgcaatc
315721 aacctggccg gggggagcag cgtgaactct gactctgccg tattgatccc cgcgctgtgt
315781 ttactggctg aggtcaccag aatcccttct ccagcctgta gaggggtcaa taatgtcaat
315841 acagtaaatt aggtaattct gctgtaaaac atccctggat ggtcaatttc cattttagct
315901 aactttatat ggccgacaca caattacggc cacatgtcaa cgtgtcaaag tgtcacgtac
315961 tgtcacggga cggagagccg gaatgaaggg tggggggggg tgcagggtgc atggctaaag

316021 ggggcgggtc tatgagtaaa caaatcagcc ttttgtcaag tccggtgata aatcacttgt
316081 ctatagaaga tggggcatta atggcagtta accctttact ggcctgcctc taaagcgtgt
316141 gactgtgtgt tgcttgggga actttaaagg tgtgaaataa tgcaagttac attaaagaga
316201 tccatatcaa tcatattagt tattgtcgag agttttaaac ctgaaatggg ccacagtatc
316261 ctcaatggca gatgaagatt ccaggacttt ttaaatcctt aacagtgtcc agacactctg
316321 acattccaca tcctcgattc ccacgaacca gcgcctcgtg tttctgctgc tctcatttca
316381 gtttcaaagt agaaagcatc atctttggca gcgtttctga tttttttctt tttttccaga
316441 agttaaactg ttacaagact tttccagtaa atatggaatc aaatcgagga agcaaaagaa
316501 cgacatgaca atatttcgtc cagcaacaag tgaactacct aattatacgg aggctgcgtc
316561 ataataagca ctcagttatt cattcataaa cacttgttcc tgaggaaaaa aaagattatt
316621 aaaccagcat tcatgagaga ttttatgcgt aaaggaaaca tccaatcaac attgagaagc
316681 aggagagggg aaatggaggc tgtaagttga catatttcct gaaatactgc tgctcttttt
316741 acatacaacc cacagtaaaa aatacttctc tcagacataa atttcattct ttttttttta
316801 agcctgtcag cagagtttta tacgtcaata aatctgagaa tcatcctgga ggagatggat
316861 gtgtattata tctgtgccat ttcctcctga ttttctgcaa gacaggcctt aaattgagtg
316921 ttaaaaaaag aaaagaaaga aaaggtgctc caattttcca aaagctgctc ccattctagt
316981 tcggacacaa aagaccgagc cactggtgga tgtcggtgaa gttaatggta gacggtgagg
317041 cttgtataat cactgggaat tggagggggg gcttcaaact cttgtaaagc actccagaca
317101 gacacgcatc acaccaggcc ttcagagatg ggagcctggg tcgggcctat tgcctgatgc
317161 cacaggggaa ataaacggcc taaaataggc atgggggcag gaaatggatt ttattggaga
317221 actaatcaag ggggccattt tagtggggca caatttagtt tttgattgga ttcctactga
317281 catcggggtt atcaatgtgt ctcatactga gaggtgtaat ttgatctttg atgtccacac
317341 ggtggtcttg atgtatgtcc tgctgcatgg gcaatgactg ctgtgcatgt ctgttgctgc
317401 ggcgagtaca tgtgtttgtg tgtgtgtgtg gggggggag agagggggca gatgccagcg
317461 tgcaaactgg ggtttcatgg atgtgagggg aggagttgtg gattaaagtg gccgccgttt
317521 gactggagtc cccccccccc caggattcag aaagagtaaa aatttccatt aactacgagt
317581 aatggtgttg gaggagagcc cagcgtgcgc tagagcccaa aagagagcac tggagagggc

317641 cgagagtttg gggaaaacca caagcggtga cgtcttcctc tttgtgtcag cacttatttc
317701 ctacaaaggc ctgaaacgat tcttccagtg agtgaacgat gctacctgct tcctttatta
317761 aacatcctcc aactcgaatg gactgttgga ttatttatgt tctccagcca taacatgtaa
317821 ccagtgtcca tgtgctcctg atttctggtt attgtttttc tgatcctttg atcagaacgt
317881 gtcatcatgg ttactgtgat attgcacaga atttcaaatt tcatgatttt ctgtttggat
317941 gaacaaataa tcagccaaat caaccaataa atgaagctgc acagtgtaaa gattccgttt
318001 tgagccgtca aaaatgggaa aggtttcccc cttttacact acattgtcgt ctctgtggga
318061 tacagctcac tctcttatct gctctcctgg gcaaaaaacg tatacctgac aggatatgtt
318121 agacatagag aagaaattca gcaccattta tttatttatt ttaaatctga tagatcgggt
318181 ttatgcgggg gttggaatct gtctctgcag gtactgaaag agaggcacga atacaccttg
318241 gacaggtcat cagcccatca caggacccac atgcctttct ctcacacctg caggtaatgt
318301 aggatctcca atattcaaac ctgcgtgttt ctggactgtg gaaggaaagt ggagcataaa
318361 aactaaaaaa aaaaaataag acaagcacat catttcttgc agtggaaaaa caaggcagga
318421 ttttatggca ggagcttcat tcagctgtca aaccatggaa ggattcctcc caataacaaa
318481 aacagatgtt ggcattaaca tgtgcattca taaagggaca ccttacttgt tagggactag
318541 aataaaatgt tagtgggggt tcagtccaca gaataactac aaatataaaa caaagacaca
318601 gcagcaagaa gcaagcagaa aagctttaga atgaaggaac aatgcaaaac cgtacaacct
318661 tgcatcagta ttttatattg ttatattgta aactaattgc acagtgatta ctaagaaata
318721 attcaatttc ttccaaatat aactgtgacc acctaacaaa agcctataca ttctatacat
318781 tttagtcatg cactctttcc ttcaaggctt tttatttatg caggtgcatg atctgtgttt
318841 gcagcagcag gagcatgaat aatacatgtt gtcttccagc cattccattg atttttttc
318901 tattttttt cattaaaaac ttgataagtt gcaagtaccg gcataagtct ggtgatcact
318961 ttgcatcctt acattattat aaagatgcaa ttgtgttcag agtcaattaa aggagagaaa
319021 gggaggtata agagcaaaaa acaataaaat attaagtgta gcaattaaat acatttttac
319081 tcataaaatt agataaaaat attgtttttt tgcagtgtga cccgacgtgc tgatcctgcc
319141 tgtttcagag aggaaacccc tacagtaaat atacatatta atcacagctc tagctggagc
319201 cgttcaggct gccacgccgc tccagtctcg atgcgagtgg atgaaggcca gaaatgcagc

319261 acagcttggc agctaccgac gatgattaaa gcagcgaatt ggtagtaact cacaatatga
319321 acgtgcatct gtgaacagcg tgtaccgaat tagcttccat ttgaatactg gcttctaacc
319381 ccagcctggt ggttccatcc gtgccagcag agcgccgagc aagcgtcgga gttgaatctg
319441 acatctggaa aactacgaag ttgttctaat ttctaatatt gttggctggc tgtctcaggg
319501 ttacacttga cactatagtg aaagctttat tgaccgactg cagccgccga aggtggagga
319561 agtgatgaca ggctcctgaa gatgcacctt ctcttttcca gcacccatta ttgctccctc
319621 cgagttctga cattgaaaag aacttctaat atcctgttac tgtcctgttt gcagcgtgca
319681 gcactctaat cccatttaag ctgtagccaa ggcagaccag tgccaacccg tcgtcaaatc
319741 caggccgctt tgaacctcca gccaagcctt taaagacaga ggaatgtggc aatgggggct
319801 cgtgccaggt gtacgccacg gtgtaattag gcctgagctg aattatacaa cagcatataa
319861 aatacaatgt cacttattag caacacattt gagggtgtta cagctacgcc ggttgcagat
319921 atggaggaat gacatggaga aacaccggct agcaattaag tacaaaaggt tcaaacgagc
319981 cggggagggg gaaaaggcaa cttgagcatt cctgtgagca agaaacacat gttccgaagt
320041 aggtgtcaac aatttctaag aggagatcgg tcagaaatct gcctctagaa gtacagaaaa
320101 acccaaacgg cctctatatg caactatttt cgtacgcgag ggattgtgat taaaagcact
320161 cagatgcaat gctccgtgtt tgcatatgaa agaaagttga tgtcacatca cttatctatt
320221 gtggaaaact gtgctgctcc tcttgtcagc tgcaaataag cccatcgtat cccgctgata
320281 aaaggcaatc aaacaaagta tataatagtg gagtcaggca ttaatagcct gtataactac
320341 cgcctaatgc tgaaataatt aagaatgtct cttactggga actggcaatg caattatcac
320401 cttgataact gtgcggcaca aagagattca gaggagaaag actgagagcg ccagagaagc
320461 aaatagctaa aatgacgagg ataatgcaat tagaacacgg ccaagagggg aaggaaacaa
320521 aagagggagc gccttaaggc ttttttccaa caggcttcac atgtctacac actccatttg
320581 cattcttata atgtcacaca attatgctag attagcatcc aaaactggga gcttgaagca
320641 gcactcacat accgtttggt gccacacttg tggcaccatt accgccatct ctccgcgagc
320701 agctacctca gtaattccga cggaacactg tgtcacttcc atcagtcggc gtccaccttc
320761 cacagaacgg caggacccac gccttaagct gtgggtcgac atttttagca acacctatat
320821 tacgtagagc agcacttgga atgtgatatg tgatatatac agctggacaa aatcatcgtc

320881 tataggagaa gggggcaaat gttctatttc tcactttgca ttattaaagg taaagatcaa
320941 attaaatatt cagccgctga tatgctgctt taatattcat agaattgaac tttatattag
321001 agaacacatt tttatgagaa gcagaccaga ggccactgat taaatttcat tgtttgatca
321061 ctacgcgggg acattacggc gcattaagag gtccagtctg tggatcccct tcagcaaaca
321121 tttagcgcga agtcctcaag gcccggggca aggatggagt gcaaatctaa catattcaaa
321181 tgtgtttcaa aactgcaaaa gacatatttc acaagttcag atttagaggg ggaaaaaaat
321241 atattctctg aatcgattaa aagggacacg tatgagaaat ggacggaaca cagggaggca
321301 tcttcatatg tttactgatg atttgagaag gaagtcaggg ctctataaat atgaataaat
321361 cagactggtc cctggagagt acttttacat ggatcttgtc ttttgtttta ctcccttttt
321421 agttgtttac ataagagtat caaagagtta aagtttgaat actagcagac actccattac
321481 ataaccacca ggagagaacc ttcaacaaag atgtagccta cagttgaaaa caatcagatc
321541 attcatctct tttttcccct caacacacag aaactctgcc ctgtgactaa ggagatcaag
321601 agcaacggac gggcgtcccc gctgtgactc aaaatggcag cctgtaaggt caaggaggtt
321661 gctagagttc ccctctacct taaaggtgga tgaaactctg ctcaaggttt tctaacagtt
321721 atctaattgg attaggcctc aaaggaaggc cccctttca tccctggctt ttgtccattc
321781 gtcttagcac tgaatggagc tcagagggct ccagacactg aggaggacag gcgatggatt
321841 aggtgaggta tcctgactaa ttaagacgag gccaggtgtc atgctgattg cgggaaaatt
321901 gagacctcat gccgccatgt gtctcaatcc ccctcccccc tttctcaaca accactcatt
321961 tcactgtaca ccacccgctg ctgtcctctt ctccctccct tcccttactg ctgaggtaac
322021 ctgagctgcc tttccttgac ccctctcttc cttggagagc taatcagacc cacctggagc
322081 tccccggggc tccgttagct gctggttcat ccagcaacag ctgatccaac ctgagtcccc
322141 cgggccaacg ctgcaggtct ggcacccaac acacaccggc ttggcatcat ccggggagat
322201 tttggtaact gatggttcac tgaagccccg agcgcaatca ccgcttagca atgacaagtg
322261 tgcgtgtgtg cgtgtgtgtg catccaactg ggcccacatg gggctggggt gcaccatgtg
322321 tcagcaaaaa tcgacaactc ttttacaatt gcagttccaa ttaaaacggt ggggtgatga
322381 cacggagagg ccaatctggc aggtaccgct cttacatttc aggtgacaag caatgtcacc
322441 atagcaacag aggcaagacc gacacacaga aagcagactt ttaggaaaat atgcaatata

322501 aaatacaagg agatcaatta cagacgtgga tgtaagtaaa agcatggctc cacagttaca
322561 ataaggatgt ttggctgtat ctaccaggat tggaactttg tcattagtgt ttcaagtcca
322621 ttcagagaca tgtttgtttt tcttataaac atgttcaact gctaacatat atgtattctc
322681 tttcagttgt gaagtgcagc tgaaggtaga acaggtgttc tagcttgagg aacaatgcta
322741 ctgcagtgtt cctgtggttt cgaatgtccc gagctgaaat ccaagcttcc tgattacctt
322801 gctttattgt tgtttcaagg tttcactgcg gcattcactt ttggagtgtc cacagctcaa
322861 gttgacgagg acgtgcacaa atatgccaaa accattaagg gggaaaaaag gcacaccgct
322921 gcacatcaaa gttggacttt ccttctgacc ttcggatcgt ggtgaggttt ttaaatattt
322981 tctttggtcc gctgaggcta aactgaagtt gtattggcac attttgcttt tagttggcaa
323041 acgaagcgaa atgccttcag cgttaaaacg acatcccccg cagctgggag gtaaacgcta
323101 ggggccaatt ctgccgctgg tgcgcgaggt cgcaaagatc acggatgcca tttcacagta
323161 cggtttaaag gggattcaaa tgagaataac aataaaacaa agatgatcca aaatgtgaaa
323221 aaaaatccat ctcgctcctc ttaacccttg atgggggact cacttagata cttggaggtt
323281 ccaaattaaa aaccaagacc atcacctgtg gatagtcact ggccactgct gatcggttag
323341 aatttatcct cttctgcgat tggctggcag aagtcttgcg gttctgctgc ctcacaaagg
323401 cgggaatgtc tctttttggc catctgcagc agtcaccaag cacgatcact tgcctcataa
323461 agggttaaga atttgggtgg tttaaggtaa cggtccatgc acaaatatcc tctggatgag
323521 ctggaatagg tcacggagtc tgagaatgga actgcatgag gtcacggcca gctgtggttc
323581 acgccacggg cagccagtca gctaatattt tcctccgttt ggtataattt ctcttttgat
323641 tcagctcaac ttcacttcag ttcctggttt ccttggacaa ataaagacat tcgctctgtt
323701 tcacaaaggc gacataagtg cagaagctgt aataagcatg acttacccat caagaaagag
323761 cttaagcatc gaagaggtgg ccagaaatgc aatatggtaa tgttattctg catctaatgg
323821 gcatgtaaat agaagaaaag cagcctccaa ctctgccaaa tgacgaataa ataatttttc
323881 ccatcaaatc ttttacagta tgtgttcaaa ttcttcacat ggaacaaaca ggatatgcaa
323941 atgcaccaaa tgaacagcat attaccatga aacaaaatcc ttatatgctg cgtataacca
324001 gtaaaaacaa tgtttgcttc attatgcagt gttgtacaca agcatatata aatcaaagaa
324061 aatgattcat ccatagttaa tatatgtgcg tctgctttac actgtttttg ttacagctcc

324121 gtttcttcag agcaaattga cagtcgcgag caaacaacct tgcattttat aatacacgtg
324181 acgctgtttg ccttaactat tcatcaaaaa aagcaatagt atcaacgttt tttttcgttt
324241 ttcatattat cctgagcaca aaattattct aatatagcca aaggccaact gaaatgggac
324301 tggtacatgc agatccagtc cattatgcat tgttaaacat tgatctatgg tttaatattt
324361 gcttcccaac agcaatgaca atgtgttatt accttgacag agagccataa tgtgtgttga
324421 catgatggtg attatttaaa taagccgagg caataaatac ggctccacgc gagatccgga
324481 acatgagaga gaggagggag gggtggcctt gaagttttgg ggctgttgtg acgtctgaca
324541 gtcagagaca taaaatatca gagaaattgt tgaattttgg acttagatta gttcccagag
324601 caaagctgga atggaaaagt gatttatatt aatgtattaa gactttaggt gttatagaaa
324661 gcccaaaatt tgattattct tgtggaacta gcgaaactcc acagcctcag gagctggtga
324721 ttccggtgag gctggctatg caccatctca tcagaagttt cttgtagtta cacaaacttc
324781 taaatcaata tcagagcgtc tctctaacag acgtggcggt atctccgcgg cgcttctggg
324841 agcagttggc atccccggcc aagaggggtc acgcgcattt ctgttcgaac ccgtggatgc
324901 agaaaaattc acactgatgg agtcgtgagg cttggcaaat catcagctta gacaaacact
324961 cgagcctcct ggcaaacacc aactgtcgcg aacagcagcg cgcaaactgg catccacagc
325021 aattagggga gaagagttgg cgagggctcg gcctgtccca gacacctgtt ccacagagag
325081 cggcaggcta ctttgtcgag gcgacacctt agcggagcat taactaatga gattagaagg
325141 gtcactaatg agctctgagg tataaaaaaa aaagaaaagt caactgctaa gtgctgaagc
325201 caaggtcaga aattcagatt aataacaaaa caagtgcttg gaacgaagcg cccatcgtat
325261 ttttgttgga ctctggaaac tttgtgtctg agcatctcaa atccaaatca ccgatccaat
325321 cacttggtgt tgggtctatc attatcatca cggtcgggag aggctgaaac attgccccaa
325381 aaatgtagct tttgcccatc ctttatttgc tccatgacct cgtggtgaga taagcttcgg
325441 agccgcagca gaggcacctt taataatgtg ctgacaggaa gtcatacatg tttaaccagg
325501 taatagcatg ctgaggaata atgatacata ttgggtttta ggacaaggta ttaatctcta
325561 gcatccttct gcaaccactc tctcccccta ctttattcac tgggggttaa aagagattga
325621 cctcccttca agtaaaatta attgctttag ttattccttt tattttcctg cagataaaag
325681 ctgcagagct gccccatatg gctttattgg cacacaacca cgctattctc ccccccatat

325741 actggtgaaa gatttcacgt tatgtataat acactgtcaa ccaatgacaa aaacaaattc
325801 cagttttgtt taagtgggag atgtaatcaa cgcgcaataa gcttaagtgc acaaaaatat
325861 ctcttttctt gttagcgctg catgtttgct gaagaaatat tacatgccaa agtgtattaa
325921 cggggaagga cgtcatgcat cttcgcatca gacctgagag atccttgtca ccgccatccg
325981 ctgtaatgtt taatactgtt tatgtccatc caaatcgagc acatgcatgg aatgactgat
326041 gccgccatca taatttagtc ataacagccc cgctccctat agtagccatc cacggatgct
326101 cagtccagga gagtgccgca tgaatagtga tccattcacc cctcaccttt aaaagccctt
326161 atggcgagcg ctccattcga ctccaactgc actgtctggc tcaaaccatc cttcagatga
326221 gacaataaag atctgatgca gacatgtgca ggtttgtgcg tctccgtcgg tgtgcgtgtg
326281 ttgagaaagc tcgctcctgt gcgggaatga tctcactgac ccgcatgctg aaggagaaag
326341 gggaagagag tgcttgaggt gacgacttct ccactcactt agggaacttt gacttatgag
326401 gtgcactgca tttagccaag ctaatgcaag cgagggccgt gccattaacc tcgggaagag
326461 aggcaggcct cggcagtatg atgcattcag attggcagag gacaatattt caccatgaca
326521 ggcattacca atgaataatt aagtgtttaa tataatgatc ttgggagagg aagagagaga
326581 gggcaaagaa agaaagagag acagagacaa agagaggaga aaatggtaat tccacaattc
326641 attgccctta aaacctatct ggcattatta cccagcacca ttgctttttt tttttttttt
326701 ttttgttgaa acaaataggt ttttgatggc tctgccagag tagtaacggt tattaggttt
326761 gagaactctt tcatgatgga agtggtaccc tgccgcgcag agatcatgaa attttaagcg
326821 gggaatttac agcagaaaga ctgtggggca gcggggccgt ctgggccatg ccgagggaca
326881 catgtgtttc agagagggat aatgctagtc taatgctgct gcaaataaat tgttaacaag
326941 ctggagatag ttgcgcagcc tggcgcacaa agaggtttta tttattcagt ctttacgagg
327001 gaaaatttgg gttcgaaaac ggagcgacga gtcatttggg agtgatgaag cgacggcagt
327061 cgagccggat cttactttaa caaaaccgtg tcaagctgca gcgcccagga gaggggtcgc
327121 tcaatcaaat ggagggtggg gggggtgaaa aataaataaa gaattaaagg aaatgctgag
327181 attaagcaaa actactcaga cctcttcagc gtgactggca caacatcaat acagttattt
327241 agtgcaagtt tgtttcagag gagaagctat aaatgtttta ttgtttttct attgatcaaa
327301 tatctcggat gagtgagtga tgacaataca cttccagttg tccttttcat aaagcatcaa

327361 ctctgccagt actgacgttc tgagctgatg aacggtgaca caaacatgat
acttctattt
327421 ttttgtcttt tatcacagat taaccggcat cctatcactt tccaacatgg
ccgcattaaa
327481 atatgacatg cagaggtcct atgaacacat gcaaccaagt aaaaaatgaa
ataaatcaat
327541 aaatgtgaag ttcgtgaatg attattttcg gttcattcgg ttcactcttg
tacatttaaa
327601 cttagttcag caagtgttga ctctgtagtg gctgcaagca gaattacaat
taaatttgaa
327661 tgaagcaaag acgtccgcgt caatatgata caatatatac aattgactta
tgagatttca
327721 gaattggcgg ccgataaaaa tcagaaaaca ccaagtggtc cagcttttca
atcaatgttg
327781 tcctctacaa gtgcgccaag gactcacgcg tctgcatgat tcccagtcag
cccacgcctt
327841 cgaaacacag ccgcacatcc tcccaaagcc caacagagcg ctaatgcttc
actcctcaca
327901 ctgctgacct ttggcattct agatgctgct tgttgcctgt gtgacagatt
cacatgcata
327961 ttcataccta catatggccc acccgaatga agtcagccta catgaggaag
ttcattaccc
328021 agtattctca gctgggtccg gtcaaactcc gcggatttgc ctgtgagctg
tgtttcggtg
328081 ggataattgc gggttgcgcg acgaagtggg actgcgctgg aaaacgtgct
tatgcccgtg
328141 tgtggcataa gaacggtggc agcaggcggg cgtgtgcggt gggggtggtg
cgtccccgca
328201 tccgtgcatc cgcttgtgtc aatgagcgat gtgtaggtgt gtgtcggctg
cgagtgcgcg
328261 ccacggcttg tttgtgaggt gagaagagag aaacgtggag tccctcagca
tgcctccacc
328321 tcctctcgct ccatctgccc atttcgctca ctcctccata aattaaaaac
gctgtacatc
328381 tgctttgggg tacattgtta gttgtgtgca ttcgccggtt cttatgttta
taaaaaatta
328441 aacacttttc cacattcaat tatagcccaa cttctgaaat cccaccatag
caagcatgat
328501 ggaggcctcc tttccatcca acatgtggaa aggacctggc ctttccggct
ttctgaccga
328561 ggcatgtcgg gagtagaaaa actgtaatta acagctgttt gtggtgagac
gaaacaaagc
328621 ctgtaaatgt catttggcct cggcataaaa atgtgctttt gcagcagtaa
tccaccacag
328681 caaagaggag tttgagtctc ggtgacaaca aaatcgagcg atccaggtga
gaaatgtact
328741 gtatctgcgc cacgttttca cgcgcaagaa ctgtttcagt gaaagaactt
ctgccatcat
328801 ttcacttcag ctgttaatta tacggtaccc ctgtgctgta aaaggtccca
aatgtgcact
328861 tgtttccttt cctaaatggg ttaattgatg aaaggactga ccgggcaaaa
cgatctcccc
328921 agcagaccat ctgtatgcaa gaagatgtgt gtctgaagac aaaacaccag
tacaagggac

328981 agtgaagtta attgggacaa aagggacagc ccaacacctc tgtacattgg ccagcattga
329041 gcagaataat tagtgcaatc agaggttgtg gtcacaaagt tgccctatat ggaacaccca
329101 ggccactggg gggacccgtg tttgcctttg acatggacac gcggggctcc ggggcagtca
329161 tatttggcca tggtttggta actgtgagcc ttgcagcaga gcaggtgtag cagaccgatg
329221 aggccatcaa tctctccctg gcctgtctct gtaggattca gggttaatta tcatcaaata
329281 agccattaag gagaaagatg gaagcataga ggacgagagg atgcaaaaag ggggacagct
329341 ggatggttga gttagctgtg tgtgttgcta agatactaaa agcagcttgt ttttatacaa
329401 aagagttcca cgactgtagc ttcctattta cttgtctact ttaagctaac agctaactag
329461 attgtagcca gatctttact ctttaatgtt acgtttgtcc tgtataagta attgatataa
329521 ttttattcac gttaattcat gctcaacaag tcttagcagg tcaaacgaga ccaagaatat
329581 ataataatct aatagaattt acacttattt tgaatctttt tcgcagcgat tgcttttgtc
329641 caactgtctt ctgatatagt gcaaaataat ttgaccactg cactcttctc tcacaagggt
329701 acacatgttc aggtaaaaag taatgactag aagcatcaaa ggtttgagtc tatgttaaaa
329761 ttatatagat tcatacgtac attaaatacc ctcataaagt gcttcttttt attacactgc
329821 acctttagtc ttgacagtat ccatctcagc gtagattact tcataatgca taaatggaaa
329881 atgccattag ggcttctttt catgctctta acatattaat tggcattacg gcgaatgtca
329941 gatcacgcag taaattgtat ccggagaagt cagtgacatc tgacctattg aaaatttcac
330001 agtgttctta taccttcatt tatctactcg atgtgcccat gaatatttaa ggttgacaat
330061 taaaccccag ctgacctgcg ggatggaggc agcctggcgc tgtcaaggcg tggaggcaga
330121 aaccagcgct aaccctctcc tcaaacacct taaaactccc tccacctctc cccccatcct
330181 ccccccttcc aaatacacag aacgctcaac gccatgctgt cacggtgaca cagctcgatg
330241 tatgacagcc acattacaaa tgtgtcatgt ttatgggggc accaacagac acggttaatg
330301 tttctccagt gtaaatgttt tggcggggtc agagttgatg tgtgccagtg tcccacctca
330361 ccttggtcac attttatttt tgtgtgcgca gtgtctgctg caacgagggg ctgctaactg
330421 catgctttca tgcacgagtg gagtttctga taacacaaat gtggacccag agttctgagt
330481 attgtgacag aagggtccat tgtgtccgtg tcgcaggggt agcagcctca gacacttctc
330541 tcattagccc attccaccag ttccttcaag ggaatactga ggtgttccca ggccggccga

330601 gagatatgat ctctctaacc tgtcctaggt ctgccccagg gtctcttccc ggatggacat
330661 gcacagaaca cttactccag ggagccgtcc aagaccccag cgaccccagt ccggataagc
330721 ggttttaaga cggacaggca gaggggtcat cagctccatc gtcaatggaa acctgtttga
330781 cgttcttgtc cattgtttgg tctgcgaggt taaaggtcaa ccagaagaag aatcaggatg
330841 aacaaactga aagaaatcat gtccggtctg cactagtgcg tttggacaca tgcggaacaa
330901 gggcaaaggt cttgtaaaat tgcttctaac cagattgaac cgggcacgga ctgatgtgag
330961 aagagtctac tatattgtag agtagctgaa cttttttttt tttaatcatt caagaatcca
331021 caatcatgag catactcctc acaccaccaa aagatttcag acatcttcaa ggcttcatat
331081 gagtgtgaaa ttcggaaaaa ttcaattcga agaaccaatg gtactccagt gaactactgt
331141 aaggagttca gcctgtcaat caacctaaat ctgatgtgat ctaaaactaa cagcatgcct
331201 tatttccaga cagacagatc catatgtaca gtctgaatgt aataaccagc acattacggt
331261 agaccgagca ggctgactta agacatcgct gtgcagagag ctgacgctgt cagcttattt
331321 ccatccctgt attaatgcat ttgttcactc cgcgtgggtt acactgtagt cctggatttt
331381 aatcatgtaa ataatgcagt catttccatt gaaattaaaa tatgtccgcc tttttactta
331441 taaggcacta accagtgact atacatcaga ttattttttc aaacggtatg taatattaat
331501 ggaccaaagc tttttcccac ccatagaatg ggtccatgag aatggagggg aatgacttca
331561 ctttgtaatt attttgccga gccctcaggt caaaccttct gattcacagc cagagccttg
331621 atatgaaata agatttcaaa tcaagaaaaa acagaaaggg accttctgct ttccctcagt
331681 tagcatcaca caactgaatc tccaaaaaag gatttctcta gcgtctcaaa ctctgatgaa
331741 ctctccagga atccttaatc gatggagcat tcgggtatga aagtgatttc tcaaaatgtg
331801 ttccttaccc cgtttttgag tctttctgag cgtacgcaag ggctcgtaca cgacaaacgg
331861 gcggttagag ctgtatcatt atgtcgatca atccctcgca gttgatgcaa ttttacctgg
331921 aacacacgtt tcatccgaag gtccgcagtc tgtcccagag cccgcatttc aacacacact
331981 gttattttga acaaactgca agctccacag gcaatctaca tcttgctgtg gtgtaaattt
332041 gctgtgggaa tagaaatgag ccaactgtct gtgcggttgg gtttttttc tttaaaaacc
332101 aactcctacc aagcagtgtg ccataccaat gagcgaaaat acttgcttgt agagtaagag
332161 ggactcaggt tataaaatag gaaaggtatt actgctgtca ggcatcgggg actaatacaa

332221 ctcacctcct tgtcgcctct atcagcactg acatctgggc cggtaatcat actttaaatg
332281 gaatccccat aatgatagtt ttgtggattt atcatttggt ctaaatacat ctgcaataga
332341 ttactttttc ttcttcaggt ccaattttcc atcacacgtt gcaatcacat ctgtgaaacc
332401 gtggaagcag aaagagctgt ccgtgtttcc aaaatactga cttacaaggg ggaaaacaac
332461 ctaaagccca aaaagatgac ttgaacgatc agttgtcttc atttaaaagt ccgttgaaag
332521 cagcgtcgtt aacaatgagg tgcaatttag agcagtctgg tgtctcaacg tttccccgga
332581 ggatgatttc tactgggtga tgtcaggtcc tctgggaaat gacagcagtt gagaatgcca
332641 tattaacaat tgttcctttt ccctcaatgt aggattagca tcaactgcag atccacaggt
332701 agatctttat aaactaggaa atgacttaaa gagaaactat tgtatatttg ttgggacgct
332761 gagactcgct gaaactgtgg gatggaagaa agtcaagtct gattgtatcg gtccatctta
332821 tttcttgatc caggatttga ttcaaggcaa aaaaaagctg gtgctgaaga tgcaggagca
332881 cttttctcag gctaacctgt aagcaaaggt aatgtgagca gaccttctga ttagagtctg
332941 cgtgttcatc caaacaaaca catgaattaa tgaaggggct tcatgtgttt cactgcacac
333001 tacaggagcg tggagggagg ggcagagagg gacaccccag gcacaacatt gcaaaagtgg
333061 gtttggacag cctgcaatac gatcccttcc atctcacact tcgagtatgc cccactggct
333121 ctgacccgtg tccgctttct ttcactgttg tcagacctac aagtacacac atttgacaca
333181 aggcaacacc agcagagaca ccctcacact ggcattctgt taaaatgaaa ggaggcgggc
333241 ctgtatattc ttctctggac cctcccgacc gcaaagaccg ccattattca gatgcaaaac
333301 ctttgtcttt ttttgaatga aatggtattg atttctgtta aacaccctgt gtaatttcag
333361 ctcttgtatt cacagcttca ttaatctgtg gctgaatcca tctcagcctt cctcgcgatc
333421 acgcggtgcc tcaatactct tcattaattg aacctttttt tttttccagg aaatattcag
333481 tctgcacgtg agccgggtca agcaggacca cacagccagg gattgtgatt ctaatcatgt
333541 ggaggacagc atacggccag gcttttaatt ggctcatata cacatgttcg gctgattgca
333601 tcccacatgt accggttttc ataagatgga taacacactc cccatggtct gcagaagtgt
333661 aggggcaagc caactggtag tgcacttgag ataccttgac agtaatttta ccatgtacat
333721 ctgagccttt atatgggctt cattgggccg ggcctatatc gaaaacatta atatgcacta
333781 gaaatgacta agaagagaag gagagggttg atgtagtgag atagatgccc cttaaatctg

333841 tcttcaatta gtccctacta gcttacatct tccagttgaa ttctgattag ctaatttggc
333901 cagagagcac ttggtctctt aagtgcaacc tcgcttcttt tgagtattaa aaacctttga
333961 tcagcatccc ttattaccta taaaagccaa catgattacg ttttgtgttg catcagctcc
334021 tcatccgcag cacttttaca ctcggcatat ggaagatcac aggctccagt aaggtccaaa
334081 aaatgtattc agttaagaag agcaattgac agaagagata tgttaatgtc acccatatct
334141 cccctgtgtg cctttgaaca cgtggcctga cttctatctc tgtagctagc tacttccctc
334201 ttgtggccaa atggaacaca gaacatgtgt ttggcatgtt tgcttctctg cttcctgcaa
334261 gttttttatt ttattttttg catcaaggag acagagatca gaaatgtgta aaacacaaca
334321 gtgtgctaac actcctccat atgcccatcc cccactcctt ctgctcttca gaaagtggat
334381 caccagggaa gctaaccccc cctgcccatt aacatataga ctctttattt aaatcccatt
334441 cacttttcca ttcatttaga agagtgatgc cagcaggtta cagtgatggc attgcgtgaa
334501 gccctgattg agcctcaatt tgatcatttt atcagattgt gaggtaaaca gtaccatgat
334561 ggataagatc tcattacaac aggaaagtga gagtttaatt tcctgtacat gttccatctt
334621 tattaatgtc aaaggcagca agtcgtcaat tggattacag gtcttgatac tctggctagg
334681 cacagtattt gacacaaatg aggccagtga gctagcttaa aggaattaca tctggcatac
334741 tttccaattt attgtggcat aaatacatgt tatggtacag aatgttttag aaccaatgag
334801 tgtaaaaaca tgctggaaac aatacagtat aatgaatgcg cacaaaacag taggaggctg
334861 cctatcaaga gagaaactac agcacaagac aatggtcatc acaaactgac actacctgtc
334921 caaagacaag gaaaacaaac ttcaataata aagctgtcaa caccccagct tgaataccca
334981 ggagcaatga ccatctcaac tctcatcccc tggtgatagc catcatcatg gaccctctcc
335041 tgagagacag catctcatct atcaatcagc cacacgcata cccacacacg ggctcactgc
335101 cccctcacca catcatctct atttgtccca ttatatcctt aacacggtct aaaaaacctc
335161 tgctaaaggg aaaactgtgg aggtttggcg ccggtcaaga cagtgtgtgc agaggtacgt
335221 gtggtcagca ggttctttgc tattgcccat ctttggtgct aagacaccgc cataacgctg
335281 tttacttgtt tttcccgctt taatcctcag ttgaagtttg ccgtgcagcg tgcagattta
335341 cccgtgttac ccaaaaccct cagggaaccg aagcatcctg gcgtgagccc ggaaatgaga
335401 aagaggcagc agtgtagatg tacctgatca ttaccattct aaatttatat tgattgttga

335461 agcttaagac ctaaatgcga gttattcccc aggcacagtg tgtgtttgtg tgatttgtgg
335521 ggcttggacg gaggctaatc cacaggaacc gcaaaatgtg tgcagtgcgt gtggccacag
335581 atatggcagc acacgttata tactggcagg ttatctttgc acgctgctta aaataaataa
335641 gaacaacagg aacacctaat cattacaatt taatgcatca gtctcttcag ggacatcaat
335701 ctgtttagtt agatagtttt acctactgtt gtgtaaatga ggcaaaaatc aaactaaaag
335761 taattggcaa gacaatacct atcaagcaac ggtgttgcag gtggtggacg ctggctgttt
335821 cactcatctt ttctgaccca ttcttggcta cttgtccaac tttacagagt cctcagtagc
335881 atgcgatggt acctgtagcc catatgagtg gctcaggaag tgcatcttgt ccaggcttat
335941 gagtagtctt aaggaaagtc acaggagctg gatcagtggg cattatataa ccaagctatt
336001 ttcacagttc gcgaatgtga tttagtctat cccagcagga cctttgatgc acagctcttg
336061 gatttcacct tacatgcagt cctgttgtgg agggttgcac agggatggtg aagtgctgat
336121 acagagggca ggacgtggat gaagaattgg agttgtcaag aggtttctag tttctcctgc
336181 cgtagcacca tgggacaaag ttgtgtgttt gtgaggcgtc tccatctcta ggcagcttcc
336241 atcactcaca tgcatatgtg cttgcccttg acattatcat cagcagattt gcacgaggag
336301 ccccgaggta aggttgaaat actgctgagg cttttgaggc actttagaga gcgtctaagg
336361 gctaaataca caccaggaga cagacagagg aaggggagaa agctcagtaa ctgaacctaa
336421 cagagcacgc ctcaggggac agcagtaatt aaacagtgat taaattaagg ggcaatgcgg
336481 gcaaaatcta gactgattga cactctgctg gcttctcttt cctctcctcc ctctctctct
336541 ctatcaaacc agatctctgc ttctctttgg gttcaactct gtgacagagc gccgcctttt
336601 caccgacctc caattactca cccatgctgc atttctgatg caatatgggc cacacaagaa
336661 aaatatctgc acaaagagca ccttttgtca attattagtt ggctccagga ctgtaacact
336721 ggtgcatact cccgtactgt tattgtttgt ctcagaaaaa ctactcttgc tctcgaattg
336781 caccgcaagt actatcagtc ggtcctaaag ataaactcag cacaatcacg tggagcgatc
336841 tgtgatttac aaaactgcac tcgtattgag tttctcctcc ttttcaaaac tgtggttgag
336901 ctttcaaagc gccttatcaa atactctcct gcggtgggga aatacaataa attatctgtg
336961 atgcctcctt atacccaggg taaatagaca gacataagag ctgagcaaca gcaataaaac
337021 cagctttaac aaaacaatca gaaaaaccac ggtggcgaaa ttgccttcct ccaaatcgga

337081 aattctccac gcgttccagg ctcaccccgc tcaccactgt gcccccggtc ctcagctccc
337141 cgtatagatt ttcatgaacc gggtcttgcg ttcatgaaaa tatgagggtc cttaaacaat
337201 ctgttaagtc tcccgatggt gaataaggtc tttccggtgt gtaatacaaa ttcaaatggc
337261 tgtatggaga tggattatgg cgcagataaa gaacaggggc ccactttaga ggatgagttc
337321 aagctgacag gtattgatta agtgctcaat gagaacacgc ctatgaaagg ccaggcagca
337381 tacggctcat gaaagaatca tatacctcaa atgctatacg cttctgcaaa catgcagact
337441 tgggaatgat gcgcaagctt ctcataccac aggccaaaca gttgacaacc tgcttgcccg
337501 tgtgggtaaa gtcattaaaa aaatatatca aactttttat tttgaaagcc tattaatacc
337561 tatatcctcc tgttaagaag tgaagaaaga tgtgtttttt tgcaagatgc cccccccccc
337621 ccccccaaac ccgctgagac cagcataatc acatatgcca gcgtgcatta ttcttttata
337681 tgacaccaca cactccctta ctcacactgg cgatgtatgg aaaagtggga ggatgttctt
337741 taaaacaaca catggacttt ctgggtaaaa cagcaacacg taaccatttc aagcgtaaaa
337801 tgtgcagttg tcgaaatgca tgaggatgac atccgcactc tgtctgatcc tcctcatcat
337861 tcccggcagt tggcaagcag ccgcttctct acctctctgc accataaaca aatgtttgaa
337921 cttggagaaa gagggcacgt agtcatacta aacataaagc gttcaccgtg ggaggaggga
337981 gagggaagag aacctggaaa atgaatgagc tgaataaaca agcaagataa agccagcgga
338041 aagaaaaata caagaaagcg tgtgcagacc agcggggagg gtgttctgaa cggaccaccg
338101 cctcacaggg tatctcaagt tatctgaggg aaaccgtctc tccctgccag ttgcactctg
338161 atggaaagta ggcaataaat gcttccataa gccatcgtaa atatgcagct ttctgctcca
338221 cacacacagc tcagacctgg gtacgagctc attatccgtc atcggcactg gcggattcgc
338281 acgctaagtt aaggacaggg atcgcttaaa agccctttaa cttcaacagc ggttggattt
338341 gcggctcact tctgggacca ggatgcatgg tggtcaaact gtaatatttt tctcttaaag
338401 aatgatttgt gtcaaaaagt gtatacccaa gcctgacatt tgaggtttgg ttaaatgtta
338461 gatgcactca aacaacccgt aattgttcaa tgagctgaaa aactttcagc ctggaaccac
338521 agaaattaga tatgcatggg taatgttttt ccaccatgta taaaaactat atttagttta
338581 aatgcagatt atatgaataa tcgaatggct tggagacgtc tcctgttcag tcatatatgt
338641 caaaaacgta ggcattaggg tcttatcgcc caaatacttt caatatacaa tttgcgatct

338701 atgaatagca ttgacaaaag acacatttga cttgattacc tcttgacatt tctctccatg
338761 aatgacagca gtggtagata aagcaatcac agcactgaca cagtgtaagt atataaatat
338821 ctttatttat tctgccctcc agataaagat ataaaggcac attaacgcat gagtgaccat
338881 ggttagtagt tcccagccta cctgcccatc tgtggaggct gacagtacag cagcgtgccc
338941 ttcattatcg ctgggtaatg ctcatcacaa cagccgtggc ttttctccat aaaaagaccc
339001 caagtctcac tcacaatgag gaaaaaggcg agataaggtg ccggagacca tttctcttga
339061 cattcatgac ggcaactcat cagtcatgct gtcaaaggct ctggtctggg ctagtttggg
339121 atttatgaga aataatgatg gcttgaaaat agccagtgag ggcttgtctt ttacctttgg
339181 tttgggagtg ctgtgaattg aggcacgctc tgcataaaac ccaatttctt ttgtcagtga
339241 cccttgcact gcttttgcat tcaatctcag catgtttggg agggtttggc tgagcctgtt
339301 tgagtggccg ctgacatttc tgcctcacgt gatgagataa gtttatttaa aaagaaggct
339361 gcatctccgt gagtgtcaag gctgccgacc attcgctgcg tgtcattcaa acgatggtcg
339421 gcgcagctct gttgttaatt gtgtttaaat gcacactcaa aagagaaaaa acgccaactc
339481 tgacaaagtc ctatcctcac ctggatcctg tataaaaagg ggacagcaag aggactgtaa
339541 tagaatctgt caaacactca ctttacaccg gtgacaagca gtgacagatg ctcccatcgc
339601 aattctcttt gtaaacatgc agggacacat cgcagggacc cgtctccggt ctcaaaaagc
339661 ataagaaaat gattcagtcc aaagtcattt gaaagtggat ttcaattcca atgcaagggc
339721 gacacgtctg aatgttagca caactgtaca actaaagatt atagtcagga ggacttctaa
339781 tgggctcccg atgggaccgc gctacaggga tgtcctccag caccaaatta ttagtcaatt
339841 tcagattatg aagtttatta attactattc ttaatatatc ttagcagaag ttccttgagc
339901 tatagcctaa ctacctaaac tcagcaaatg aaatgttcca catatattaa atatattaat
339961 ggaattctag attggttgta cctgaagttc acctttttag aattataaag gcaaatcaaa
340021 gcagccaagc cagtttaaca ttgcttgtga ccaaagcgtt gtgaaattag gggacaagag
340081 tctttggggt atttttcccc cacattcaag tgtggataag acctctccag gctcgagggt
340141 gcttggatct gtggcagaaa ttaagctcag agaattgtgg gttatgggat ggtggattga
340201 cagtaacatt agcccctcgg cacctctgct ggagcacgcc gccgtcatgc aaaatggatg
340261 gtaaattaag tcttcttaat agtaggaagg gatgtggaga tgtggagtct ggttggttgg

340321 aactacttgg accttgcgag agaaagataa aaagggcggc gcttttagct ggataaaatc
340381 aagtggctat ggagtgtttt ggttaatagg cctgctcaac ttcacgccat catatttata
340441 ccaattgggg aagagctcat tcaggctctt cagacaagtt cacacgtcgt actctgccct
340501 ttgctattca tctcaacgca ttcatgaaag tcactatagt attagtggga aaagagttca
340561 cagcaaagag ttctcagcat gatggagtaa acatttgtag cagctggctt ttgctgtggc
340621 tgttgtttgt ttttcaccac cttctttcga ggttcagtga catctcattg ttgttccaca
340681 aagcctaacc ttaatgtaga taaagccaag tcggcaaatc tttgcaaaag caacatgcat
340741 ggacgcgcac acttttaaga gtagggaggt acacagaggg agcaaggcgt tttcaatgca
340801 tttagatgag acctttaaca aaggtcccta atgcagatat caatgaacca ggggcccagg
340861 ctatcgaagc gaaattggct gacggaggga tgtcacccct gcgcatgaaa aatgatggat
340921 cccatttgta aacttctcct ctgtcccccct gccctctacc tccgctgcca ctggttattc
340981 agcaagatag aaatcccaca accgctcaaa aataacaaga ggttcggtaa aattggcact
341041 ctgccgggcc atttcatcgg accgcgcggg gacccacaga ggctctgtgg gatttcagtg
341101 atgacaggct tgattattat aagggacttt gtttttccat ctacctttca gttcatctcc
341161 acggacatca acaagatgaa atcaatatct gccaatcttg aaaaatagat atacacttct
341221 aaaacattag cttttgcatg ttggacggac aatatcctgt ttcttgccgt tgttatgctt
341281 ggttggcctg gcaaaggcac tcatcgtgaa gggatggagc agataaatag aaagatcccc
341341 tccaatacga acaaagaagc agtcaagagc accagtttgg gtttatctta gctcgtggtg
341401 gtggtgaatg aaaaaaaact acagcaaata tttcgacaaa taacaacttt ttagaataac
341461 cctcattaga ggttaatgtt tgttttaact gagctggtcc tgcaggaata aagctctgca
341521 tatcaaatct aagcaacatt atatgcatag taagcatatc aaaggagagg taaaagacat
341581 aatcactaac ctttttctta atcattgtgg cttttgcgtt aagacaaaga cgcagaggtg
341641 attgtgggcc aacaagcatc tctgttggga aaaatggaaa cttcctcatt gatgacggct
341701 gactgctgac attgtggttt cagtggcaga gtgactttgg gatggcttgt ggtctgccgc
341761 tgttcaaagc tgtgccttga gggtaagggg taaatttgcc aagttaatca cataatgagg
341821 ccaattaaac atagaggagc ccaggtgaaa tgggagctgc cgcagagcct ccgataaatg
341881 aggcccggat gattttcctc tccggatgga ttggacaacc tagtttcagc cccсttccaa

341941 tcaattcatc ggaaatgtaa aaacctatgt taccaatttt tcaagtgtaa aaatgtgggt
342001 ttaatgtaaa ttggtgtggt aaaagtgttg ctcaattttc aacagtgaat cactgcaaaa
342061 aaatactgcc ggttatctag tcagccccat tttcacaggc ctttaatcaa aaagcgacat
342121 gaactacact ccacctattt agccttacct tatctcaacg ctggtgactt tacacctgtt
342181 catcccaaaa atcatttcag tgtttttctt cattaatcca gatagtaaga gcgtggacgc
342241 ggtgatggtg ggagtatctg ggtgtttaag tgctctttgg gggtctgtag gaggaggggg
342301 gatgataaat cactccagca gcaattagac ttccaagtgc ttcctgcctc catgtacctc
342361 ctggcacagc gggcctgggg tcaacaatgg tgaggcatac acacggaact caactgaaac
342421 ggtgtatccc tgtgccttcc tcttaagagt gtcaccctcc tctcagccat ttctggtgca
342481 cacacagttc agaaggattg caacattatg gtgtgttgta ttatcgcatc tctgttatgt
342541 ggtttccccc ctcccactca gcacggagcc aaaagcccgt ggctgtggag gctaatttcg
342601 aaaggcagaa attcagccga ggagagaaaa tgctgctagc tcgctacagg gcaaccacaa
342661 atggaaatct gtagcgctgt caaacacaac acaaagtaat aaggtacagc ggaatgagca
342721 gcatgttaaa acctggaaat acggagcgag acgctgaaga tgatgagaga gtatttcatc
342781 agcatctgta aaaaggccgg ccgccctgcc catcacgagc aactaaggcc gacgtggaag
342841 atttggacca gggaaattaa gcccggcaca gtttacccgc tcatattgtg atgatttgtt
342901 aataaaatcc aaaccgacag actagatgtg agtcagtggc taaaacgcgc cggtgattta
342961 gacttaatcc gtcattatcc tgcctttgaa gtgacctcgc ggtgtacgtt cccactgtga
343021 cccagaagca cactcgcagc ttcttcatta gccaggatga aatagagcca cagaacactt
343081 tgatgtccca tcttgtccac acactcacat acacatgata ccttcacact cacacattac
343141 tgttatcctt atcattctct ttgctggggc atggaaaaac attatggttt ttaatcaatt
343201 atggctccca cctttggggc agcacattca ttacatatga taggagcagc aaagggacaa
343261 tattccaatt gcatgccgag atagaaatga aagaaattgt atggttagag gctagacaaa
343321 gactgcaatt tcatactgca ggacatcctt gactggattt acatatgtgt gcgcagaagg
343381 cttataccac atgcaatttc accgatataa tttatgtaca ctgcgtgatt tatcgtcctt
343441 atatcgtagc aggaatcaag ctcagatctc ctgaagaggc ctgtacatgc tgccggcaaa
343501 ttatgggcat tgtaaaagtt tattgcggta atgaaatgga aagaatcatt tgaaaatgca

343561 catgttacta taatgctaaa cggtatccaa tcagagggag atatcattag
gaggcaaaca
343621 cgtgtcgaac ataaatccag aggacatttt gtttacatct ctgcagttcc
gttgcacaga
343681 tgttgacagg aactgtgata ttggagtttt aaatacagta cgcagtggaa
attgtacata
343741 tattgtacga ggttatgatg gatgaagaat atgaggcaac cgttgtttta
aaacattccc
343801 tgaatttttc cagaaaaggc agaaattatc aagtagaatt atggtttggc
actaatttat
343861 tacgtcacag cctatttgaa aggtgcatgc gtcatctaaa gcttttgttt
tttacaaatt
343921 tgatgggttc tttggagatt tttcttgctt tgtgtacagt tcctctatac
tcttgtacac
343981 agcttgctta gaactaggcc tatgttttaa tgatatgctg ctaattaata
gtttaaaatc
344041 atctcaatcc atctataatt taataataac aaaataaatt atccatgtaa
attaatgtat
344101 atccaagcag ttctacaaaa gaagtgtgat cccttcggaa acggtccatc
tgtctccttg
344161 gcaggcagac tgctgcggcc tatacttcac aaaagaagaa gaaagcgtga
gcacctgtgc
344221 gcttcacatc acaacactca acctttgccg cccgccgtaa aagccacatc
cttgccccag
344281 gcggtggccc gtcgagaagg aggaggagaa gaagaagaag aagaatttta
atgttcccat
344341 aagaagaaat tcagctttta catgctctgg gggttgttgt ttgtttgttt
gtttctataa
344401 acaaatgaag ttttgaactt acaaatgaat gggagataaa gcgagagccg
ggtacaaaga
344461 acaacactaa cagagccatc tgcctgagaa cacacatcca tcttaccgag
gacaacctgc
344521 ctcagacaag atggggaatg aaaaaatgtt tgtgtaacag tgaaagtaaa
ataaataaaa
344581 agcagatcac agcgtgagat agcgccacaa taatctggac ttgcagcatc
aacaaatgaa
344641 tacattacag aaacaccaaa cacacacacg ctaaatgaag tgttactgtt
aaatggactg
344701 tgtttctatg gcgctttcct gctcagcctt ggggacgacc ttctcgttag
gagcaatgat
344761 caccaacccg cattcagatg ccagccattc aggagccagt tgctcaaggt
cgctttctga
344821 caagtagatg gctgagatca aaccaacaac ctttgtatca ctacagctgt
cctctggtcc
344881 aaggtgaccc tccaaacctt ctgacagtgg acacagaggg tatgttgata
cttctgcccg
344941 ggatcaacta tttactggaa atattccttt tatacgcaga aaataggact
attttaatca
345001 ctggattctt atagtgaata atttataggg tccaaactgc cagtaatgca
gcagtaaccc
345061 agcagcaatg tatttggttg gctgaaaaca ggccagaaga cagaccacct
cacactcttg
345121 gtgtagtctc ataaccacag aagcacctgt tacttttaaa agcatgatga
aaatattagc

345181 acagatttaa tattgaattt caagacacac acagttcgat acacacctct gttaaacagg
345241 gcattgggga agtaaatgca gttagtggtt ccaccatctt tgcatcataa ccagctggat
345301 agaagtgcag tcagcaatct gggacggtga tgtaattaaa gatttgtttt atttttagac
345361 cgttttctca tcactttaat cagtatatat ggtcccagtg atacagcgct cttatccgct
345421 caccgtctga cactgcatta ccttatcacg ctgagagctt ttagctgtgt ttccagtcat
345481 taccttcata cactgggcag ctagcagccc ggatcagagc gagggatagt ggctgagcct
345541 ttgcatgaaa aacattttg ttctatttct agtctgtact cattggcttt atttattcct
345601 gtgacttaaa aactgagagc actcctggca cttagaagta tcacatcacc atcacttcag
345661 cgctgcagta taatgaccag atggatatat tgtggataat ttcatgtgga tttaatgaca
345721 ccttcatgca aacgttgatt tttcttactc ttagagtcac gtattagatg gaacatgaat
345781 cacattgaat gactcttgaa tgcagtaacc cacctgctac cagcagggag agcagtggcg
345841 ctgatcctca ggcttcgtgt gcgtgtgtgt gcgtgtgtgt gtgagagaga gagaaacact
345901 gacatttggt gtacaggtac agtgtttgct tacatcaaga tagtaaaaac taaatctact
345961 tacaattcaa gcaggttccc acacagagca atctgaccac tggtaacact ggagccagtg
346021 acaacagcag cacacaggaa gcacattaac agaggcggtg ccgttccaac acagggcagg
346081 tgaggagcaa agtgaacact gagggagaca agaccggaca ggaagtaaac tggagaacta
346141 aaccaggggg aatcagtcat ccccagagcc tgacaacggc agaaggaccc agcgtaaaca
346201 tgagaagaat aatatgagaa aacatgtcaa atcagaccac gacaaataca tgtaaacaga
346261 gcagcatatg gatctccata acttcatata aatttcctaa aaaattacat tttacacaaa
346321 attgaatgtt gaatgttgca gctcataaag atcaaatatt aaagcattaa taaggcagag
346381 aatttaaaaa aaaacaatgt tgtgagtaat ttattctggc attggaggag gaattaaata
346441 attataaagt ggcaaataat ggagttcgtg aggtcctgtc ccagaatggc gcccctctgg
346501 ccagagccag actgattagc caccatcagg acatggacct ggatgccact ctgcagctaa
346561 gtacacagaa ttagtattcc caccacttcc agacagcttt gtgaatcaaa ccctgcaaca
346621 cttaagcttt aatcctcttt tttgaaagca aaacagccaa tttaacatca cattaagacc
346681 agttgttcag ccgcacgctg aagccttaga gctggccaca gaacccacag cacgcagcgg
346741 ccgacgcatt atcgacacac agctgcactc tgaccctcca ctacaggaga cttctacacc

346801 aaacaagccc tggataccett agcgtcctcc cattgacatg gatcaataat
ttacatattt
346861 gtggtggcta ccagaaagcg gcctaagtgc gctttgcgag cgccagactc
acatgcattt
346921 tacaggaggg cagcgggagg aggccggcta ttagcccgca tttgatcctg
cagtaaggac
346981 atgctaactt tgcgcccagt ggctgtgggt cagcagttct gattgtcagc
cttgcacttt
347041 tattgaagaa tttatcgtgg gtgctggtcc tatatttgaa gcaacacgct
ggaagcctct
347101 tttattcatc ggacgacaca aaactgaggt caagccttaa acctttctgc
atagagtgca
347161 ttttcaactg aacataacga tactccgtta gggtccgcgc ttatactgtg
tcgctctgtg
347221 cagggggga tctagtttac cattccttgt ttagtcggcc gctctggact
ctttccttga
347281 agtggctcgc gtgaagtata aagtgaagca ctgcttcact ctgtgtaatc
tgaagcgctc
347341 tggtttgctg cagcgggtaa catcactcgc cgctccctcc agtccctccc
acacctccca
347401 cctcccccgt gtcgtgcaga aatattaaag acgtgccccg gggtcagcgg
caatagcctt
347461 cgctctacca tcacggcgcc cttgtgttct tcatagccac cgctgcgaga
gggagtgatt
347521 cattcttctg atccactcca ttaaggtatt agttgattcc agtggattta
atcagcatag
347581 taattcctct ccatgggaag ataagtctct ctcaagagca gttatgccat
ccactatgcc
347641 tgctgattag acacaaggct ccaaaataat tcatcagaaa actgcaaaaa
ataatttatc
347701 aaaagtgctt tgcacaaaac ccgctcagga cctttgcttc ttaataatta
aactctgcaa
347761 ttaggcgtct taccatgcac tcagtgtgag gctccatgta atattcataa
ccatacataa
347821 ttatgcaata tatccactga aataggaggc tatatattca cggtggggct
gtcaagcggc
347881 aagacgttta tatttggttt ataacacagc ttcatctcat acaggagcag
ctggcatttt
347941 ggtgacggag gagaaagaaa tgttctctgg tccggcgtcc tcggggaaag
tgaagtgaga
348001 aggggagaaa aacaagcact aagtggtgtg attctcaccg caggagccgc
taaatatcaa
348061 agtgtaactg ctggactcaa caaagactca cctttgcagg atcaaacatt
gaccatactc
348121 acatgcagaa agaaaaaaaa gaggcattgg attaaaatgg ggttgagaag
gggtggtgtc
348181 ttgggatgta ttgtatgata aattatacat aatgctcagt ccagcatggt
tcagatctat
348241 agaataaata atagtctggc agcccttgat ggtgcctgaa gcaaataagc
aacagcagcg
348301 gtcggcagtg gacacactgt ggacaaggtt ggctttcatc tcgagaagca
attcaaagca
348361 atggcccgcg cacacaacaa accggcaatc aaagctaatg tgcaactggg
cagtggtgta

348421 cttcagagag cccgccgtct cgagtgcagg gggagatcac acgccagcgc ctcgctcgcg
348481 tcacattgtc tgattttcaa cttgctgtct tcagaactta ttatatttca ctatctgaat
348541 gcatttaaaa ggcccgctgc tgtcgggagc atccggaatt ctgagggcct ttcatgacac
348601 atactgtgct taatgaagaa ggagaaaaac atacatttgt ctctgttgca tcacagtcgc
348661 tctcaaaggc tcgttctccc gtagcggggt ccctgaaaca taaggaaata tcaaacaaat
348721 gttctatttt atagttattg tagtagatga tgacatatac aacatgcatt gggaggatta
348781 atgagaattt aatatttgga agagtcccat ttaacgtaaa atgatgcatg atgaccccat
348841 agaacacata ctgtgttgta tatggagctt gaaacggtgc acttttatat gagattctta
348901 cttagattaa tgacatgtga caaagtttgt gatattacac aatgctgacc taaaatcagg
348961 attttatggt gctggtggtg gaaatgatac agttatgttc cgaaaaactc agatggtgct
349021 gcaatctgga tcattgtctt tataaattta aagttacctt caagtatttc atttaagttc
349081 actttgtttc ggcaaatttc agggtgaaac atttgtcact ggtccagaaa acttggatgg
349141 acttcaacac ggaagctgct ttttttctgc agggaagaga ctatccagct gacgtcgtgt
349201 attggttgtt tggtttttc aataacacag ggaaattgta aggggcatag cgaacattga
349261 gaacatttaa gaaagtattt cgtacacaac aggtcaagag tccaaacact tggctgtttt
349321 gccttccttt tcctttcccg gtctgctgca aaacaatgac cttttgggat acggatattt
349381 tattggctgt ataatacgga tatgattaac ataaataaga cctaaagtca aataggcgta
349441 aaggtcgtca ctcaggacca ggcccgggtc tgtgaggtga tttacaacaa cttaatttga
349501 ctgtgcggag taatttatct gtgacagagc caagccacgc gactcgccgc atatcgcgcg
349561 cacaagcagg cgacagcacg ccgtgacggc acacgtctca ctgatctacc caatcagtca
349621 agatgcttta caacttctgt caaaggccct aacagttctc ataaattaca aagccattgt
349681 gtatggtgcc aataactttt gaaggagaat ctcatctaaa ccgagcagag gagcttctcc
349741 ctccttcaga cgtggaaggc agaatgggat actgcaataa aagtacatga aagaagcagg
349801 acgagcccat tgatgatgga ttgccatcag aaacgggcct ccaagtttcc ctccagctgg
349861 tttcaaagaa taaatctggc tcaggtattg acatttttgg ttcaaagcag gtttaagcga
349921 gtgcgttgga ttcccttgca gatcactcgt tttcacatct ggcttccctc cgctgcagcc
349981 tccagtctcc tgttgcctcg catctgtgtc atctccatgt ctaagctgac agttagggaa

350041 tgggaggcat aacccattca taaacagcaa cagtaatgtg atggaagggg gttgggggg
350101 ggggtagaga attgagtatg taggtctgga gtctgcaaac aataatgtgc caccatttat
350161 ttcaaatgcc cggtgtagcc cctgcaggga aatcacagcc agagctccaa gccaacctgt
350221 agtgctgtca atatgtggat aattgaacgg tttcacatgt gctgcatctc tgttcgggtg
350281 gatcagggaa aacgtgcacg ttgaaaaaac atgcgctccc ttgttaaaca aattgtgtag
350341 ctgtgtagtc gtattatagt gcgggtggaa accccggctg gggttgggag catgctgcaa
350401 acaggaagca gcgaatttcc tggtgtcacg ccgacagggt gagctttaaa ttgaagaaaa
350461 aagataacca cgtgtaggga tttacagttt ttcatttccc attcagcccg ctttggagtt
350521 ttgaccagaa aggaccgacc gcacccgggg taacggtgaa cgggctcagc tttgaaacac
350581 cacaatggtc aattagaatg aaactgccca agcatgcctt atcatgatca gatgtgtgac
350641 agcccggcat caaggcattt atctctttgt gaaataaaat cttggcatgg atttcggttc
350701 gctagcaggt aaaacctggc gcaggtccct tacgttgttc atgcgaacag actctccagt
350761 gaactaaaga gaggaagtga tgtagcgtaa tcctagcagt caagcagagg tggttggaga
350821 atgtcttatt atatatagtg agttccctgg actaaagggg ctatagggga aaaaggaggg
350881 gcttaagtta gacaacatcg gttctaaacc ctaatgttgc agcttctgtg tatgaaagta
350941 tttggttggg tgattccccc ggtgatgtga ccagtgccat taatgtgtta atctacgtga
351001 cttgtaatgt ctgactggtc ggagatgact agaaatgttc tataaaccat ctacataaat
351061 tagtttggct ggatgttaaa atgtggttga tctggtgata gaaggaacta gtaatgcaaa
351121 gtgtattatt ggtctattta tgagcatatt aggtgctatg gaggggaacg ccagggccgc
351181 cacttctgcc agtgagctgt aaccactttt tatgctttga tgaacctacc cttgagaaac
351241 caggacatcc gtgactgcgt tctgcagagc acgtgagacg ccttcctccc ctcagccttc
351301 tcaaacacat aaatcagttt ctcatatcat catctagtca tattcaatgc ataagcggct
351361 ctgatcggta aatctgtcag ctttgtccct tttcagtgat gtatcagatt cgtaatactc
351421 tcccacacat gcagtaaatc tctgatgact gtgtttttta aaattcagag ccggagctgc
351481 tcgaaacaga taactttact tgcagacaga ccgctcggag tggtgcgttc aggcggcccc
351541 tcggccgttc atgtcagctg aaaacagcga cctagacggg aggaccggga tgcatttctc
351601 gaattcattg tccccgctgt gaaggagcag aacatcgccg tctgattaat ggccgtacac

351661 cgtctaatga acagtaattg aatgatataa ttcagtaggt ccttaaaagc
ccagaggagc
351721 cgtcttcctc tgcaaggcgg tgacattgtc tagagttgat tatagttgac
cttgcatggg
351781 gagaatccgg cctttgaaat gccgggccta agcttcgggg aatagaagca
cagaaaaaca
351841 agtgcagaga caaatgaaat gaggccgcat tggaaaaaga aaagggatgg
atcgatgcac
351901 cggtcaataa cgatttgatt tttttatcac caaattctta gggagaggaa
gacgattgat
351961 caaacatctt gggttgtatt ggcatttcta acaatattaa ttaagaaaaa
tgcttagctg
352021 actggttgat aaatcagcca atgtggttta atcaaaacaa atgatagttg
taaacacaat
352081 tccgcaacgg tgcgtccaaa tccacccttg tctgtgtttc cgtaaagtgt
atgacatcca
352141 cgacgcagac ctcaccgccc cgcgaaatca atcagctctt taacagagtg
cactctaaag
352201 gaaaacccaa ccttcagaga gcccaacaaa agtcaggcgc gtgattataa
gaaaggcttt
352261 gataaatgcg catcagactc cggccccgcc atcctccacc ctctcccacc
gagatcgccc
352321 gccttcgctc ccagcggaca tttccacttg gcagagatgc tgaatctcgt
tattctttat
352381 ttggcccgcg gaccgcacgg gtcaataacg ccggcgtgcg gccacctcgc
cgctgtactg
352441 tctgcagatg tgttctccat ccaaatgcat tccaataaat catttaaagc
ttcggctgaa
352501 aatgtaatca tgtaattgaa ttcggtcatc aatatctact gtatgcatac
gcgccctgcg
352561 tgtgtgattt cataagcgcg cccgagcgca ggagtgggag gaaatgcgtc
caaactccca
352621 ttgaggtatg actcataccg gcgcctcgtt atcatctcgc catccgattg
atgagggcgc
352681 cgagactgaa gccgttgcct ttccagcctg ttttgtccat cacgatgacg
caatgatgcc
352741 taaaagttca tcataacagt tctggtctta gcgaactagt ctagggaatc
atgtcgggat
352801 ttgttagcaa acaactttga cgcttcctgt aaaaaatagc tgactctgtt
cgtggaaata
352861 ttattttttc attatctaat ggtcttcaat ctatagcgac tctgaatcag
caacagtcat
352921 gtatgactaa aagcaataag taatgaatta ggctattgat ggggtcttta
gccggggtcg
352981 gaactggccc cagaagtcaa taattacctc agcaagaata tctttattgc
aaaagtgcac
353041 acggcagcaa aatcatcgcc acaccaaaac attcccctct ggtgtaattg
ccactcctta
353101 gaaattatag ttttcattaa aacaaaatgt tcaagattat tgattgctta
aataagactc
353161 aatatccttt tatttcagag ctgaacgaaa ttgcccggaa tagtaatatc
acctcaacta
353221 ttgtctgcat ccatcacgcg tgcacgcgcg gcctcgttgt tgtcgtccct
ggaggattat

353281 tagagcaaac gaagtgtatt taaatgagtg cagctactag gtagcacggc
tccatgcaga
353341 ggcaccggca tgcacgtcgc cacagcacat ggaaaagaca gatgtcgcac
acaagcgtgc
353401 gctgcgatgg gcctccaacg cctgttgtgg atctggaaca aacacgtggt
taaatacgaa
353461 gacctcacat ctgcacatgg gccaggacgg tgatccaaac tgctcagtgc
ctttccagga
353521 gagttggact ggtaaataac acacagagga gacacccaca cacaccttat
acacaaagtg
353581 tcgttgctgc gagcaggtct gcgcgatacc cgcataatta tgaaaacggt
gattactgta
353641 attggcggtt ggaccatttt cctggcagct gatgtaaagg cgtatgaaaa
agaccggact
353701 tgataataaa gtaaaactga accaagcgtg aaatagtaac tacctggaac
attcattggg
353761 agaccactta tgtgcctttt tcctggtcgc cgcgcacaaa ggacctttca
tcccgacact
353821 cgtcgtcagc tgtcttggcc tcaactttca gtgggggagg aaacaccatc
cagctgggtt
353881 gcgccaattt ttaatggata tttctttgtt catcttattt tcatttctgg
ggctcattct
353941 gagtaatttt atttttctga ggccacagag ggcccctttg ccaaattatt
ttagattatt
354001 agcggcttca aaattgaatt ttaacaagaa tgattgtgtg gtaacacctt
ctcttgggct
354061 gcgctacttg gcaaggtaga gaaaatctct aatgaatgtc tgtattacta
aattacacct
354121 ctgaaacttg tttaatttag aaaatatttt cttggttctc tctcaaacac
ttaaattaat
354181 cgggttttcc tgtaagacca acctcagttg ttgctgctct acaaatgtaa
tgctggactg
354241 gtaaactgga cccagaacag gtaaatggag tcagtccatc accaccacaa
aaggtacaaa
354301 ggcactgctg ggacttgaac ccaggatctc ctgtttacta gacaggcact
ttaaccaact
354361 aagccacagc gcctgtggca gccttatatg atggtgagta gaaaatggca
gcagaaaaga
354421 agaagcgacg catcgttgca ttgtagatat gtcagatctt cgcccacaga
gatgctgttg
354481 gtgtgtatct gagagtgacg ggacgcggtg aaaataaacc ctctgaaagt
gaccgttggt
354541 gatcagattc cctgagacac acgcagagac agggcgacat cagctcctca
gagtggccgg
354601 gaaccacgta cggatgtgag gtgaggaggc ccgggcggtg gtgagatcag
gtggtggaca
354661 ggagctggtt tgctttgagt cgcatcaggc ttgttgtgag tttgagctct
gcctgatcaa
354721 agtccaccct cagctgccgg ccctgacgct gcccttcggc tcctgtcagt
ctgcgttgaa
354781 cacggtgccc tctgagaaga gcgattaatc acaattacaa gtccggcctg
gcagtctctt
354841 atgctccctc tctgaggagc cctccgttct ttactgccct cccgtcctct
cccgctgccg

354901 atcctgttca ggagatcaat gttgctgtcg aggcttttat ttatttagcg tagagcctcg
354961 gccgctctgc cacaactacg tccggccggc gtgaactttt ggtcccgggg tcctgagctg
355021 acagtgacgg ctgctttcac cagcaccaga tgacaacggc gtcttctcac aaacccactt
355081 ttgtctttgg ctgcggaatg agacacagaa tataaagcga atgataaaat acagctgcca
355141 gtgatttaag tgacagcagc ggcagtctcc aaaacacaaa ttcagactgt gaagggcttt
355201 cagaaagaaa aatgatcgtg cgctgtcaac ccgcggctga tgtttaatgt tgtaaaagca
355261 acataaaatg aataaatgac cctactgacc attctgattc atcaagccgt ggctgcggac
355321 aacagtctct tccttcgagc gcgtcgcagc tgatcaatca tgtggtaggt ggggaggaga
355381 gccattgata tggagattag gacttaaggt cagacacaca gagaggaacc tcaacacaga
355441 ggtcaaaggt caccacatgc aggatggaga aactttaaaa ctatgtgttt tgaatgctac
355501 tttaatgtgt ttgtgattaa aatacccccca gaggcacaca cattcacaca tagaccccccc
355561 cacacacaca cacacacaca cacacacaca cacacacgca cacacacaca cgtcacagta
355621 acaaggctnn nnnnnnnnnn nnnnnnnnnn nnnnnnnnnn nnnnnnnnnn nnnnnnnnnn
355681 nnnnnnnnnn nnnnnnnnnn nnnnnnnnnn nnnnnnnnnn nnnnnnnnnn nnnnnnnnnn
355741 nnnnnnnnnn nnnnnnnnnn nnnnnnnnnn nnnnnnnnnn nnnnnnnnnn nnnnnnnnnn
355801 nnnnnnnnnn nnnntggggg agggaggaga gatgcaggag cccccccccc ctgtagggag
355861 aagagggcag ggattctgga cacctgcctg tttgttgttt ggtcatcttc agctcagatg
355921 tgtgtttctt ctggcccctg gctgtgcacg cgagcccgcg cagggggagc agagggaaag
355981 gggcactgat gaaggcactt ccccgcttcc tctccgcttc cttattgact gttttttctg
356041 ctttttttgca aggaagaaga caagagacga cggggcgtca cttgtatttg caacgctttt
356101 atttttttttc ctgtctttca ttttacattt tcctttgacc tcaatttttc atgactgtgg
356161 ttcccctgcg attcccactg tatcgaattc atctgcttcc aatacatacc cagcacaacc
356221 tatcaatcac cggggagcgt ggcaaactaa tcatttattg atgaaaacgg aggccaccat
356281 gaatttctaa cagcatttgt cttatttatc aggctagatc aaacaaagag aatccaaaca
356341 agcttaaagg ggaaagggaa gaagtcacag ggaacaagtt attcttcaga cagcagcagg
356401 ttgtggatgg aggatggacg gaagaaggta gggaatacgg aaggaggagc ggtggcgaat
356461 tccgagggat gttcagcagg gtacacatct ggattaatgc ttccacataa ggcaattttc

356521 tcttcgcagg ctcctggtcc cctctgcagg agagcaaggt gagggacggc tcccacgctg
356581 agatgagatt tgatgtttcc cctcagccgc agctgcccgt ctggattccc gagctggctc
356641 tgggggtggt gcagagacct cgggcttcca ccagaacaca cgtccggtgg cggcgctcgc
356701 gagctcgcta acatggactc tggggaaatg gcagtgtttt cttcaattgt ttgctttctt
356761 taatgcagag gcacagcctg ttggtattct tttgaacatt ctgcagttat tgctgggctg
356821 ggggtggggg gtggggtagt tggagggggg ggggtagtgt ggggggtaa aaaacaacaa
356881 aagagaagcg aagtttgttc gattcgtacg acacgagggg agttgaaaca gagttcaggc
356941 ctgccgctga ggtgtccacg tagccgctcc agatcaaaaa ggaaggtgcg gcggtggcgt
357001 agcagcgggt ggcggttaat ttggtcccgc accccacgat tcgtcttgga gtgacgggga
357061 aggggcttgg cggggaagtt cggaatcaaa gtacagctgg gcgatcaaaa attgagcagc
357121 ttgttttgcg tgtttacagt ctgacagtga gatgtaaaca gatcttattt aaatccacac
357181 agcgaaatca gggccgcgtc actcggggag gagaaatcag aggacgaggg gctgccgggg
357241 agccaggaga ggcagcacaa gcagcaggag gactgggaaa acaggcagca gagaagctga
357301 ggtcagtctg cctcctgcat atctccagca cggcgtcctg tgggagcagc cggctgcctg
357361 cgtggggggg gagagaggtg gagctggtgt gaaagaggct caaggggacg gagcgacggg
357421 taatctgtgt ccctctgaag gagcggggag caggagtcaa caagaggaca ctgtgatatg
357481 atagccaggg ggccagaaag tggagcccga gcggcctgta gccaccgtgg ctgcaggaga
357541 cactccgggg gcctgtgagg gcgggctgtt tttccgtggg gctgggaggg ggaacgctac
357601 caggactcgc cagtgagctc atccagaagg gagtcatcag tgatgtcact cagcgtggtt
357661 aataaaagct tcagtcggcc ttctggggtg ctcctgagtc gacccgggcc acgtcggcgc
357721 tgctcactat cagaacccgc ggcatgaaat tactttgagt cataactctg ttctcccttt
357781 cactcgtttg cagccccacc accaattcac ccgctcttca ggtccatgaa gcccctaact
357841 cagcacatcc acgagcgttt gacaggaaca cggcgtgctg gggagcttca gttcaggctt
357901 cagcggtcgc cccagaccag atgtcccaat cagtagtgca gaaactgctg ggtgctctgt
357961 taccccctct ctccctccct ccctccctcc cctgtagcac aggcctgcag gaacaaggtt
358021 atgttttaca cagcctcagt gggcgtgttc cctgcagatt tttacaggga ttttagacca
358081 gatctctcct attttgtgag tctggacagg aattaatggc agacacgacc ctataaatgc

358141 gcccgcaatc agtaatctaa acagctgtca gaggagacgg agtgggcaga ttttgtcaca
358201 tgtgtcaccc caccccccaa aggttgctta atggtcagac ggtgattatg aattcatgtg
358261 gatcagagac agagttggag ggagtcaaat ccagcagtga atgtctgatc ctggactgaa
358321 actgctggat gcggaaccca gtcaagggtc tgtctctgtc tccccttaat ggccatgtgt
358381 ggaactgcaa ttgctgccat ttaaacagaa ttcattcatg ttttctcaaa cctgagcatc
358441 aattgagaag attgtgtgtt gttgttgttg ttgttgtgtt aatagtttct gagcgtagaa
358501 tcgttttcag catctcattt agcctcagat atgctggcaa taaatattca tgctcctcaa
358561 ttggagacaa cagaaaaacg ggccgggaca tttttctcca tctgaaaaaa aattggcacc
358621 tgccacggcg ttgtcatcct tcatggttag aactatcaga tgtaagacgc ggcgctcacc
358681 cggggagggg ggccgccatc tcccacctca gcctgctgac tgggcatgtt ctcattgtca
358741 cagggtgatt tggtgttcat ctgcagccct cggaggcccc atcctcccat ggattcaaag
358801 cactccaccg tctacttaat cacaatacaa atgctcccag ttctggtttg aataagagac
358861 cttttttttt cattttagtc actgaagcca ccccaccaca cccctcccca ccctactctg
358921 catatgatgt gtctacgagg ctcgccgcgc ttgatcaagt gggccagccc gtctctgctg
358981 ggattagcca tcaaagtgag aggcaaaggt aacttgatgt tgctcgcaga cagtgattga
359041 tgatagcgcc gtttcataac gaattgtgca cgcccccccc catccccccc aacgactttt
359101 gcacatggga aatgttaatt gaaatgctgc gtgtgcagag atgtgctttt cagctgttac
359161 actcggcgtc gcgacggcag gaatttagca tgtcgacaga ccaaaaccac tctcgcctgt
359221 cttgtgtgtc ggtaaactgg cacaaatgtg ttggcaggag gcgaataaga tgccacctga
359281 gcgcatctcc gcagatactt actctgctgt taaatgagtg cacgttttcc ccgatcagat
359341 tacacgtttc cgcgccaaca gcagcacgct cgacacgtaa aaacagtcag gtgaattgaa
359401 ctgtttggga aacagattat tttaatgcct cggaaacacg aggaaggctc atacgcacaa
359461 ctccaaaggg gtttcagaga attttgtact atatcaagcg gcctggtgac ctcagaccag
359521 cccaggacct ccacgtccag cgtcttcacc tccgaggtgg tttgggagcg gccacccggg
359581 cggctgctgc aacagtcggt ttgcataaac aaagaatttc tgccagaaag tgtcgtctgc
359641 aggctcctgg tcctcatcag ggtcgccacc tgactgcagt cggatcattc agtcatccac
359701 caccttcacc tcatgctgta gcatgatcgt gcacgtcccc acgttgccag gatctcaaca

359761 caattcctgg aagctgaaaa catcccagtt cttacaccgg acatgtcagc cattcagcat
359821 gtttgggaag ctctgggttg gaggagcaga gcaacattcc acaggtcaca ttaccaacct
359881 gctcaactca gtgaggagga gatgtgttgc accgcttcag acagttgttc tttatttcag
359941 caccaaacat gcagacaaac ccagacgcgc gttgatattt tcagtttaaa catgcctccc
360001 ttattcgttc atggaccttt ttctcatcaa aatcagctct tctttaaaca agactttgtg
360061 aatagagtct tttcattaca gtagagggat ggaccacggc tagagtggta caaaattaaa
360121 cttccatttg gagtggaggt ttgggggagg gggtgtttta tccccagcga ttctgctcac
360181 atttatcatt aacaagactt ttctgcccct ccgattatct gcctgtccac cgtcggacgg
360241 agctgtccgg gttgtttacc acagtggtaa ccagagattg caccaacaat ggcacctaat
360301 tggtttatgc cagacaagtc ctggtcaatt aaatgcataa ttcatctccg cattaattat
360361 tcatttctta agggactccg ggcttgcatt attaggagaa ctgcatcatg catattcaaa
360421 ttccccttttt tagagcgact cggtcggtta tcatctcagc tgggatgccc atcctaaata
360481 caatagcagc caggggtgga agacagccgc tattaatcac acgacaaaaa caactgcaga
360541 ggtttcccaa aagataaaaa tgtcataaac ttaaaatatg tcttttttttc tgctgctgtc
360601 atgttgttac ctggtgcgcc ggatgctgaa acgcccccctg tctttagatg cttcatgttc
360661 tacatgatgt cagcctgttc agctccaacc cgccgtctcg gggtaatttc agcctctttt
360721 gctgcatctg cccaatcagc gtcgtcaaca tcagataaat actcagcttc caaaactaat
360781 actttcacgc agtgtgaggc cgctacggac gggttggatc cgtggggggg ggcgctgcgt
360841 ggcggtgatg taatcacttt gttgttgacg ccccttgtgg ctttttttcg cccaataaac
360901 aaagttgtgt ttacgttcag tgggatgcat ttcaattatg agaggattaa ccgacgtgat
360961 tgctggttgt ttttcctact ttctttgttg acgtctgtcg tactctccac tctaaatatc
361021 cggggctggt ttacgcttta aacgttgcat aaaactgtag cagttattta ctaattttac
361081 acgcggacta cggttcaaat agtttaaatt ttatttccag ccttcctata tgtttgtagt
361141 tttatttaat cgttttaaat taaacttgca gacctttgca cctattcgtg acgttttcct
361201 gcagtatagc ttttaaccac tagatggcac gtgtctgaca gctggatcta attcagcata
361261 aacagaacta taaaatgaat gccgtcggcc atatttccct gattgaaggc agccaagtgg
361321 aactaatatg acattttaat gtgatattct gtgccaaatc tcatcttttt catttgtcct

361381 gtttgttgat aaaatgtaat acttccatct gagtgcacag tttgcataat
gtcactcatg
361441 cagctgtctg agacgctaat gaatcgcagc gtgagattaa cggcgttggt
agttaaagtt
361501 aagttggcaa taaagttctg ttttatttac agactgtggc cccaaacaca
cacaggtttg
361561 tttatttccc cagtggttta tttgttgttt ttctcatatc agattctgtc
cttgtcaaac
361621 tcacaaatga agtacatcgt gggctgacac gccacatctg tccactcccc
agacgccagc
361681 atctctacac agtcctcggc gtcacaggcg ttgtcgggct caccgttcct
ccacttgctg
361741 aacgcggtca cgggggagag gtccacgtag gtgaagaccc cctcctggtt
caggtcgtgg
361801 atgccgatat agactctgct caggcccgcg gtggttatgt actcgccaag
ggccgcgtta
361861 gccccctcgt ccttgggcat ggccaggtgg cccccccctcg cttggcagta
gagctcggcg
361921 tcggcgtggc gcttctcctc cttcaccaac agatagacct tggtgtctgt
ctccttaatg
361981 ccagcaacag ctgcagggag ggcagtgctg aaaaatgagc acttgtattt
ctataaatct
362041 cacaccgatg acgccggcgt tcgcagaaca acaggtggag cagaaacatc
gacgccggga
362101 cttaacgaca ccgacaaccg actgtggaaa tttgtgtccg accgcacacg
gtggagtcga
362161 taaatagtgc tgggaacata ccgttcttaa tgaacttcag ctcggaggag
aggtgggcca
362221 ggaggagatc catctctccg atcacattcc tccgtggcgt gcactcgcac
ggagctcctg
362281 gtaaagcaaa tatgcgagga ttacagcaca tttctgccta ttctcccaaa
tggattcatc
362341 tctctggcag tgatctgctg gataaactgg gtatcccacc tggatctccc
cttggaccag
362401 gatcccccat gtctcccttc acccctgaga tataaaccgg agcaaatcat
taaatccaat
362461 ctttgctttg aaacagaaat gtttgattac ctttgattcc acttgggccc
acttttccat
362521 aacggcccat tatacctttc tgacctttag gcccaggttg accttggaaa
taggaaatgt
362581 ttgcaggtta aagaaagaaa agttaccaat tttccactga ggtgaaaatg
cgccgaatgt
362641 gtgtaatctc aaatatgtga gtgacctccc ctcctcatta gggccttcag
taaacacttg
362701 accacttctc ctcccatctc ccaccctaat gtaaacactc tctcggcctg
gtggtgctgc
362761 aagagcacgc tatcagctgg agcagcacaa acgttgcccc cggattgaga
tggaacgaaa
362821 acaataaaca aacaaacaag aaattattgt tgttcataat agagagaaag
aaactacacc
362881 agtctctccc gttgggccga ctcttcccgg cctccccggt gcccctttct
ccccttttc
362941 acccgggtct cctttaaaag aatataaata gccatttagc cattttcctt
ggttttctgt

363001 ctctaatggt cactgttgtc gtacctttga gtccaggaac caggatctga acagtgcagg
363061 actcttctgt cagctgctct gcacatacgg tgtgtcgggt cagtgaactc agcacggaca
363121 tcagcatcag ggacaataac accttctctc tgctcatcct caacaccggt gacgctcggc
363181 aaccacctac ttaacaccag aataaagggg ttaaataaag ttaggttagg tctttctttt
363241 gctctattat tttaaaagca attgtcaaag aatgaaaagg aaaagcagct ttggtgattg
363301 atttgagggt acctaaagta gaagtccagc aaataaaaaa cattgatatg aatgggtcaa
363361 gatctgaatc acctacctgc agagtgtgtt tttaccccaa acagacagaa gaaaaagatc
363421 cccggtttac aaagcttgaa gcgttggtgg acctacagaa gcatttcatg ttcctctgca
363481 cacgcactgc agaatgtgtg cacgattgga cttttataaa cagttcacag ataaagtctc
363541 aaacagctgc agcccgccag ataattggct gggaaagcag cggcgtgatt gacggtggaa
363601 ggttcctgag aaacccgagg acccgacttc atcacaacac agcaaaaatg tgtttcagct
363661 gtttcataaa atcacaattt attcagtggt tggaacagat ccgttttatt cattcttaaa
363721 actcaacaga agctaaatag ggaaatgatc atcttttta atattatcgg tagtagtaga
363781 acagcagtgg aggatacaca gttgagtctc ctttttctaa attgaaccac aaatatgtga
363841 ctgtatctaa atgcatattt aatttaatat attttcctat gtttctgcta ctttgatttc
363901 cccatagtct gcatgtttct ctggtgatcc tctccagtgc accctgtctt cctctccaca
363961 cttcattgct tccatcttaa ttgctttgag tttgggatcc tgcggcttct tctctgtggc
364021 cacccggggg cgctattgcc cacgcaagga ggcaggggcg accagaagtt ctctgagctc
364081 acacgaacaa gaagaaacat aagtgccatt acaatagggg aaaaatacgt tttcattttc
364141 aaactgactc aagtccacac tatttgcaat attgcttttt aaatgactca agaaaatgtg
364201 atgttcacat gctttcatga ggagtgagta ataagatatg gtctaataga ggagtgtccg
364261 atatcctaat tagatttaca aacccagtgg cagctttcaa gggtggatcg ggtgtctcct
364321 tcaaaccccc ccaaaaaagt tttacattac ctccgtttca aatattcatg gccctcaagg
364381 caacatttgg tgcctctccc atccagtcag taaagcagtt gcgcgttggc taatgtgtag
364441 tttagcaaaa actgtggatt aaatgtaata aaagactacg tagagtattt gtgttcatgt
364501 aagtacaggt atgtgtttgg ctgaaaagca aagaaatcac aaaggaaaag gaaaagaaaa
364561 cccgaataca ggatattacg agcagtccgc caacgcgcct tcatgtgtgt gacgtcagcg

364621 gtgctcaccg gaccgtgagc tgtcatcctg cacctcccgg tccaggacgg agccgccggc
364681 tgcacctttg ctgcatccgc acagccgcat gaaggctttc aactccccgc agtcgccctg
364741 aacgcacatt tgaacctctg gaggatcctt taaatcgtaa gcgccgatgc tcttatataa
364801 ctcttacact ttttttcaa acttgtaact ggatgatgac ggactttctt gaatgaatgg
364861 tgttgatgct aatatctgtg tatgctaatt attagggtta gaattaattt aaaaaaaata
364921 ataatgttgg tctgagcctg cactgtgaga aatgcgttaa acttaagtga gacataaaca
364981 cagtaaaaag gcagtaaata tatctttttg caacactgcc ttcacaagaa taactgtgtt
365041 cataggaagt cgtgttataa taatccctaa ccttgacaat agtttaaaca ctctttggca
365101 cggtttcttt aacttgattg gtataagcgt cgtggctgtg gccaccttta gaggctttta
365161 tggactttat tttgatttct ctctcttcta attcctgttc tgtgttcctt tacatttggt
365221 ggccggacag gaaacaagag acaggatgtg tgtgtgtgtg tgtgtgtgtg tgtgtgtgtg
365281 tgtgtgtgtg tgtgtgtgtg tgtgtgtgtg tgtgtgtgtg tgtcttggcc tacacgcagc
365341 acccctctgt tttgcattgc cttgcaaatt attggtgagg tttatttccc agatttgaat
365401 ctgccggagc gtctcactgt gtccatctgg attagggtca aacactaaga ggatttagtc
365461 aaagttacct atgtgaaact gctttgggct gtcatggaga ggcctcagtg atggttctag
365521 tgcgtgtaag gctgcctttt gacttgatgt aagaaacaag aaaagagagc cggaggatcc
365581 cgaaggtctc caatgagcag aagtcagaga cctgaagagc agcaccaata tttctaccac
365641 atttcccaat caaaggcggc acccttgtca ttttgaacct gcatttcctg cattgaggca
365701 ctttaattaa acttggattg aagggaggaa tgtagctttg tgattatctg gcatctgagg
365761 acagtcttta ggatgccccc ccacccccca gcgcctccct gtgaaggctg aagttaacgc
365821 attgtgaagc taatcttatc ctgtgtcctt ccacattttg taaagtcctt gttatgtcag
365881 aaagtcgact ccagttattt gttcaatctg ctgtgcgaca agtttgtttt ctctggaacg
365941 acgatctcgc ctctggtgta aaggcctgtg ttgacagcag ggtaacgagt ggacttctaa
366001 acactgatta aaaatgcaaa taaggcatca caataatcac agatgcaggc ttgtcttccc
366061 gtcttatcgg agtcataaca ttcagtagca gacacacaca ttccccaaac attctggggt
366121 tttctgtgct ggtgcttttt ttttaaaaca agcatgaggt gatctatgaa gaatgtcaga
366181 tcataactgg ttgcgtgttt ccagctctcc tgtttgctga gtcgctgtac ctgttcaacg

366241 gtgagtccca attaaaggat gcgtcagcag tttccaggac ctgcactatt
ctctctggtg
366301 tttgacctta atcctgaaaa gaggataccg aagcgtcgcg tcagcacttt
tcccagaaga
366361 tgctcggttg tttgtttgat tctgtcaggc gtgcagcacg gctttaacct
ccgacccgat
366421 gttatcggaa gacgctgttg tctgaatgtg ctcgtaggat gtgtaactaa
aaatgttcaa
366481 ggccgtgcac ttaattcaaa gaccccttga ccttttttct gaggggagtt
ggttcctttc
366541 aggtttccct ctttggtttc ataaatcatt tcagattctg ttgtagtgaa
aacgttggag
366601 gcgctgaaaa agagcagcgg cttgttgggg ggggggcag cttttacgcg
tctaaacaag
366661 cagcgctgag ccgaaagcct ccagtttaca tgcaaaggcg cagcgtgtaa
tttggatttc
366721 tattttcggg ctgcgtgttt tttgtttttt ccttcccctc taacttcatt
gtgtccgtct
366781 ttctgtcact ctctcaccca gttgtgttgc cgtgccgacc agaagtgagt
catggaggat
366841 gggaggccag tgtgggcgcc ccaccccgcc gatgggttcc agctgggcat
gatcgttgac
366901 atcggagctg acgcgctcac catagagccg ctgaaccaga agggcaaggt
gggtcacaca
366961 cacacacagg tacacacaca cacacgcagg tgaacacgcc ccagaggtcc
atttccagat
367021 ggagctacac acacctattc gcagtcacgt atgaaaccat ttcaactatt
tttaacgttg
367081 ccacacagtc caaagaggca gtttagaaag gtgctaaacg ccaatgtggg
aaatccgagc
367141 gcacgccggt ataaataagt aaagaagcgg cagcagcagg cgtgctgata
gtgttgcccg
367201 gagggtgagc tgctcattag gaggtaaagg ccacatgtta cacctgagcc
gtctcttgtc
367261 cccagcaagc cactcgtgac acacgtgcgg tgcgccgcgc tcaagcgtgt
gaagcgttcc
367321 catcgcagtt aatgcggaga attttcccag ctgagatgag aacacatgtt
cacatgtgtt
367381 gctaatcctg ttaatgacag ctctgctctg ttcaggtgtc ccaggaagtc
ttcactctgg
367441 acacttggtt atatatgaag gtggttcttt gggacttaat aacaatcctt
gttgtaacgt
367501 attcttttat atatgttttt aaagagatat tttagaaaat agtaatgcta
actacatgca
367561 tagcaccagt acccccccat cagtgtttcc tacaatttgc acaaatacac
tgatagcatc
367621 tttatttctt cttttaactt acatgtgaac aatcagaatg tccaggctgt
agtttcagag
367681 taaaaaaaaa aacagaatga gccagagtga atctcatgtt ttgatctctg
gatgcccctc
367741 atcatttatc ccaagcataa atgcctcaaa attccattca ctgagagaat
aaaaggccca
367801 ttgagggtca tttctttcac cgcgttgctc attagcgctg caggctccgt
gttcgaacct

367861 cataccagct tccacttctt ctctgtaggt tgctttggtc caacttttgc
cacgtggttg
367921 ttgtggacag gcacacagag cagattgtgt ttatgcttac tgcctgttcc
taaacctaga
367981 gtggtgttga tgaggggact tcatgttacc gttaccaacc actggtgtgc
tttcatcatg
368041 tcacatgact cacaacagtg gctgctgcat gcacgtactc actcaggtgt
tcagatctgg
368101 ttgctctaca tccaaattat tcatcactct gccatgtctg cggccttgtt
tctctcacag
368161 ccggcttgtt ccgttgagtt tatttccatt aactcagact cttaaacaat
agagcgagaa
368221 catcgccggc taatttagcc cgcgaacgcg tctgcagaac atcattactg
ggatctttaa
368281 gtgcgatgaa tgattcatgt ttaatcttgt ctgttaatct atttatgatc
tctaatatac
368341 attttcggag gggggggggg ttggcgaaca attgttttgt ggagcagttt
tcagaggaac
368401 cagtaaacct gggcaataat ctcaggtttg actggttcgt ttgttttcca
gacctttctc
368461 gctcccatga gccaagtctt cccagctgag gacgacgtca acaaacacgt
agatgacaac
368521 tgtaagtgaa cgcaccggtg gctgtctaag tattgaacaa aaatcccgat
aatgagctca
368581 tttatctgac ctcactatca ccctcaggtt cccttatgta cttgaatgag
gctacgctgc
368641 tcaacaacgt cagagtgcga tacaataaag accacatcta cgtatgtgct
tctccttcct
368701 ctcatttagc ctttttttccc tttggtaatc tgcagtaact gaggtcttat
agaggagtta
368761 aactcttcag ggaaccaaca ttgcatttgg atctcttatg gtgttatctg
tcgctatggg
368821 ctaacgctat actggttcct gtacgtttcc tgcatccctt ctgttgtggt
gcatcataag
368881 cagatagagt aacaggttgc aggacagacg gagagaggga tccttaccct
tgcttcccag
368941 tggggaaaag ctgcaggtgg taaagaggga agggtcaaac tgtcagacta
gcaaactcaa
369001 tctgacccca gtcacccaat taggatataa gggacagatg gtagatgggt
gggtgtgtcc
369061 cagtcggctc attttcacct gttggttctc attatttaat catttattca
gttgttgact
369121 ttctccatgt gaagtggatt attttctatt ccagacttat gtagccaata
tcctgatagc
369181 tgtcaatccg tactacgatg tccctggttt gtatgggcct gatgccatca
agcagtaccg
369241 ggggaagtcg ctgggaacgc tttcgcctca tgtatacgct ataggtaaga
gctttacctg
369301 ccaccgtacc cgtagtggga agcttgagtc actcatttaa tattaggtga
taacacaatt
369361 atgcagtaca gctaaaggtc ttttctacat tcatttgttt tgcgtgttta
gctgacaaag
369421 cctatcggga catgaaggtt ctgaagatga gccagtccat catcgtctcg
ggggaatccg

369481 gcgccgggaa aacggaaaat accaagtttg tgctcaggtt agtgtgaggt
catctttccc
369541 agacggcaac acgctgcagg tctgtctccc agcagaggga caacatttgt
gtttgctctt
369601 catcttctgt tgcttcatcc tcaccgctgc ttggctctca cgcctgttgc
acagatatct
369661 caccacaacg tatgggagcg gtcaagatat cgacgagagg attgttgaag
gtgaattaac
369721 aacagagcaa aactatcccc gtgctgtatg tgggggcagt ggggcaatga
atgggcgttt
369781 tctgtcttga acctcagcta accctcttct ggaggccttc ggaaatgcaa
agacggtcag
369841 gaacaacaac agcagtcgct ttgggaagtt tgtggaaata cacttcaatg
agaaggtaac
369901 gcacgctgag aaatggaaca tttcgctccg ggtagtgcgt gcgttgttga
cggcgactcc
369961 tgtcctcaga acgcagtggt gggcgggttc gtctctcact acctgctgga
gaagtccagg
370021 atttgcgtgc agagcaacga cgagagaaac taccacatct tctacaggct
gtgtgccgga
370081 gcgtccgagg agctgaagca gaagctgcac ctggactctc ctgacagctt
cagggtcagt
370141 gtgtgggaga cacagccgta cgtgcgactg ttctaaatat taataatccc
gccacttctg
370201 ccatccaggc gtcacatgag cttattttta gtgtcgatga ggagttccct
ccggagaaga
370261 gaaagtctta cacagataaa ttccttcctg tttggatgtg attataaagt
caatccaggc
370321 gcgtgtaaag gtgtttttcc ctacatgccc tacaaaacaa cacttaacgg
aacagcaagc
370381 agctccagtt gttgctgtga atgtttttca cagagtcgtt atcgggtccc
tgaagtgaca
370441 cccacatcaa cagagccaat tacccaacgc cgttcgctgt tagtcagctg
tgaagcagca
370501 ctggaataga gcaaagcaga tctttcagag gctggaaatc aataacgcgt
ccccccctgcg
370561 agaaatatta gcgcagcctc tgattgtcat ttgtcaaacg catcgttttt
ctcatcagtg
370621 tttcaatgaa ttcctctcgt tcgcagtacc tgaacagagg atgcaccagg
ttcttcgcca
370681 gtttggactc cgataagcag atcatgcaga accggaagag tcccgaggta
acgtcacatc
370741 ccatttcact tgaatcgccc cagtggataa ttgtttcgga cagctgtggt
ccggtctctg
370801 aggcaacatc tgcactcacc actggccgct agcaatattt aatgctttaa
gcaatagaga
370861 acggacgggt ttggatggat tgtatagaat aatgtatccg gatagagaac
gtcagcctgt
370921 gctccagcct ttagggcagg gagctcagag cccagtccgc cggggaccac
agcccaaccg
370981 ggttttctac actaccaggt aggcgacgtt cacctgggtc caacttgcct
tgatgaaagc
371041 gtttttgcc cgagagggac agaaaacccg gctggattgc agcattcgag
gactgggttc

371101 tgacaccccc ttattaatat cataaaacaa ttagccgagt aagtgtgacg cgctgctctc
371161 tcaggggcta attatgtgtt tcacttcgcc ttttctcagc tggaataata ttttatgtta
371221 attaaagctt cgggaaaatt gggtgctggt aaataaagct gatatcgtgg catccttgtt
371281 agaacagtta tatttaaatt tgcgagatac aagggaggca ggaatttaaa tgcacgcatc
371341 catttatgtt tccgagctca gagagcgttg gatttacatt tatttatttt gaaatagtct
371401 cctcgttttg gcaggagcaa aactgaaatt tcctcttgaa ttaaggattt ctcagggcaa
371461 atatgagctc aactctgttg tcttgcatgt tgttcctgtg gtttgaattc ctttggaagc
371521 actgatactg ttgtcactat attgcagatt atgaacatgc actaacttta ttgttttcca
371581 cctgtctgtc catccctgta ccataggaca acaaggtgat ccttccttcc tcatcagctg
371641 tgttcttcct gctctctttc tcaccctgaa accctcaggt gttgcttagc tgccttttct
371701 ctaaagcaaa caggaagcaa gaatgcacgt ttctgcctcc ttcttgcatg ctcgttggtg
371761 tgtcgcctta gtgggaaaag acaatgtttg ccgatttatc gttttcacgg cactcggctc
371821 cacagtagaa tattttcatt gatatttacg tggatctttg cagtcggtgt tctgtcccca
371881 tgttagctta cgtgtctgtg ttgacgcttg catcatttca ccagctgata ggatgtgaat
371941 tatttgggta aatgctggat ttttgaagat aaatctttaa catctttatt gtgtgcttgg
372001 ttagatgaag gagatctgtt cagcctttac atgaattgca tgtttgattt actctttaaa
372061 tattttcttt tatttcccgt ttaaatcttg actgattctg acttgttggg cagcatctta
372121 aggtcggcgc tctgaaagac cctctgctgg acgacaaggg cgactttaac aggatgtgtg
372181 tggccatgaa gaggattggt ctggatgaca cggagaagct ggatctgttc agggtggtcg
372241 cgggtgttct ccatctgggg aatgtctgct tcgaagaaac gggaagctcc tcaggtaggg
372301 ctcttttagc ttaataactc cagctaatag agaaatctgg gagattgttc agtttgtact
372361 tgaccttttg tgtcgttctg ataaccctcg cgtttttagt tgctttgaaa gtcttttata
372421 taatcttttc taagccagtt ctgaataagc agtgaagcca cagtgatata agactgtaat
372481 tgtatgataa atgcttgcag tggggccacg tttttaaact cttccatgct ttaggtggtt
372541 gcattctgaa gaatcagtca agtcagactc tgcagtactg tgcagagctg ctgggtttgg
372601 accaggatga tctgcgtgtg agcctcacaa cgagagtcat gctcacgaca gcagggggcg
372661 ccaaaggaac agttatcaag tgagaacagc agcactctgc cacgaagtgt tctgcatttt

372721 tgtgtgtgtg tgcgtgtgtg tgtgtgtgtg ttgtttttta acacattttt aacacacagt
372781 aacacattgt tctgtggaaa agttaaacca acacagagac aaacatcagg attgaactaa
372841 cgcatcagcg ggtgaatact gggggggtgt ttatcaagct gattatattc agtctagaca
372901 ctgaatattt atgcagcctt attgtcaagt attacaccga tggaaatgtc aactttagcg
372961 aatcatatgc aagtgtattc ctgcatgttt taataaaatg tctgcacttt ttatgtactt
373021 tggtgatgat gtggtgtttg agcgatagag ccgggtctga cagattaata ttgcaggagg
373081 gccagatgct tgtctggcct caaagtgttt tctccacata aataatgcaa cctgtatctg
373141 aagaggcaga gggagtgtgg cgcagtacag taaaggctca gtgtctggcg agcaggtgca
373201 gcggggggag gggggggggt ggagctggct ttgtcattat gccccagaca gatccaccct
373261 acccctggga ggttaagtag agaggagggg tgtgtggggg atcactgggt ctctaactca
373321 tgtgcatatg atatcagcta gatgaatttt aacactttac agcatgacgt gtgtttaaaa
373381 cgatcaggtc ttcttctatt cttaggagga cagcttgaat atcaaagaag gggttgctta
373441 gtatctgcct gcaaagggca gaccttttg atttcacatt caccatcgtc cctgttggga
373501 cagcctactt gatctctggg tcacgctgta gctgcgcttt gtgcatacag ataatccttc
373561 ccgataattt aatggtgaat tgttttcaag cgccactgaa gtatttatca gaggttaaag
373621 ttcagcgtgt agttttaata aacctccgct atagaggcca gagaggaaca tccctgtggg
373681 cgaccacgcc gcatccagac aacagcagcc gcggaaatgg ggccgttgac gagagaaact
373741 ggcgagattg caacaatcta ctttaagaca gtttcttact gtcccacttt gcctccgttt
373801 agggtgcctc tcaaggtgga gcaagcaaat aacgcccgcg atgctctggc caaggccgtc
373861 tacagccgcc tgtgtgatca cgtggtgaaa cgagtgaacc aatgcttccc cttcaaggaa
373921 tcttccaact tcatcggagt gtttgaacat cgctgggttt ggtaataaac ccgatctgtt
373981 ggcccgtagc tccctcccaa tcagatatac acgcctgtta agtttatgct tgggaacaaa
374041 cttcccctgc tgccctaatt agccgcgttg catattgtta tttattttcc tcccatttca
374101 gagtactttg agcacaacag ctttgaacag ttctgcatca actactgcaa cgagaagctg
374161 cagcagttct tcaacgagcg catcctgaag gaggtgggtg ggcagcctgg ggattaaggt
374221 gatttgctgt ggctttatta attatcccag gaaaacccgc agcgacacgt cactcgcaac
374281 acaaacttgc tgatgctttc acaagctggg agaaaactgg cacgcaaggc ctccctttct

374341 tcattccctc tgcttttttc agggtttttg cacttaaatt gatcctcgca gactgtctca
374401 ttagaatgat aatttctcct gggacatgac gaggcttatc tctctgtgat cttataactt
374461 cattcttttc caagttctcc atttaaagtg gaactgctct gcttcagaca tcctaatcat
374521 cttgaatttt gttctttctg catctagttt tcatcgcggt ggttctctag ccatgaaaat
374581 atgaaggaat ggtttcataa cgctgcgtta acattagctt ttcctatttg tgccttattt
374641 aggagcaaga actgtatcaa aaggaaggcc tgggcgtgaa cgaggtgcat tacgttgaca
374701 accaggactg tataggtgag caaatgtcag cnnnnnnnnn nnnnnnnnnn nnnnnnnnnn
374761 nnnnnnnnnn nnnnnnnnnn nnnnnnnnnn nnnnnnnnnn nnnnnnnnnn nnnnnnnnnn
374821 nnnnnnnnnn nnnnnnnnnn nnnnnnnnnn nnnnnnnnnn nnnnnnnnnn nnnnnnnnnn
374881 nnnnnnnnnn nnnnnnnnnn nnnnnnnnnn nnnnnnnnnn nnnnnnnnnn nnnnnnnnnn
374941 nnnnnnnnnn nnnnnnnnnn nnnnnnnnnn nnnnnnnnnn nnnnnnnnnn nnnnnnnnnn
375001 nnnnnnnnnn nnnnnnnnnn nnnnnnnnnn nnnnnnnnnn nnnnnnnnnn nnnnnnnnnn
375061 nnnnnnnnnn nnnnnnnnnn nnnnnnnnnn nnnnnnnnnn nnnnnnnnnn nnnnnnnnnn
375121 nnnnnnnnnn nnnnnnnnnn nnnnnnnnnn nnnnnnnnnn nnnnnnnnnn nnnnnnnnnn
375181 nnnnnnnnnn nnnnnnnnnn nnnnnnnnnn nnnnnnnnnn nnnnnnnnnn nnnnnnnnnn
375241 nnnnnnnnnn nnnnnnnnnn nnnnnnnnnn nnnnnnnnnn nnnnnnnnnn nnnnnnnnnn
375301 nnnnnnnnnn nnnnnnnnnn nnnnnnnnnn nnnnnnnnnn nnnnnnnnnn nnnnnnnnnn
375361 nnnnnnnnnn nnnnnnnnnn nnnnnnnnnn nnnnnnnnnn nnnnnnnnnn nnnnnnnnnn
375421 nnnnnnnnnn nnnnnnnnnn nnnnnnnnnn nnnnggggcc gacgggccca ctcgatgcgg
375481 cgggaagatc gcgctcgttt ctgtcgtctt tgttttcaaa cggatgctcg tgttagtcgc
375541 atgaaggagc cagcgttccc atttgtatat caaaggctgc ggctgttcag gtgtggggtt
375601 aactgacagc ctctgataac atttatgact tgcaaatgac actcggcatg tcgattaatt
375661 caccatcaag acggatgatt tatgcacaag tgcctttttt atgcagatct tctccctgag
375721 gacctcctct actgggtagc atgttgcaaa caaggcatga gataaataat actggagcat
375781 acagtagtaa aaatgatcca gaggtttgtg acatcatcgc gccagctgtg gtctcgtttg
375841 cttccggctc cacgtacctg cttattattt cacatggcat tttcataaat gtccggttta
375901 gaattcaggt tctaatgaag cgtcactgct ggttccagat ctagtggagg caaagatggt

375961 gggagtcttg gatattttgg atgaggagaa ccgtctcccc cagcccagcg accagcactt
376021 cactgtagct gttcacagcc agcacaaaga ccacttcagg ctgacagtga ggcctcgtct
376081 tactctccct tcttcgtttt attctcactt aatccctcta gaagttattt tatgagggaa
376141 acggactggt aattgcacta gcgttgacat catttgctca ggtttgcttg ttttaaaagc
376201 ctttatttaa gtgacaccca gtcccacccc atcctctgtc ccttgtcagt gtcactattg
376261 ttccccgtgc tgcctgtatc tgtgtgagca tgaaatccct ttagatctct agccttgaca
376321 tcaattcaat cttaagatta gaggttgctg gttgcctcag ctctcagctg caggaaaagt
376381 tattacatag ataaaggccg gtcctccggt tgtcaaacct ttttacatta gtgaaacaaa
376441 aatgacacag attatacaat gaacccattt gatgtgattt tgtttcccag ctgataaaac
376501 atctcgtttt tagatagtaa catgcttgaa actgttacat gttgccgcta aggtaaagat
376561 tcgatccata attcccgtac gattgcatat tttcacatat attccacttt tatcccaaag
376621 aatactctcg gctccaccga tttgtttcat tttccagctg gggtggataa acagctctgc
376681 tagaaagatc ctgacgatgg catttgtttg ttttgctccc cttctccaca ctcaggtccc
376741 caggaagtcc aagttggccg ttcacagaaa tctgagggac gatgagggtt ttatcatcag
376801 gcattttgct ggagccgtct gctacgaaac agtagatatt tgtttatgaa gtggaatcaa
376861 gttcttattc accttttgag ttttgatttg aacggccgcg tgaaatcgct gctgcataac
376921 cctctgttcg tgtctacaga ctcggtttgt ggagaagaat aacgacgccc tccacatgtc
376981 cttggaaagc ttagtgtgtg aatccaaaga caaatttgtt caagagctgt ttgagaactc
377041 caacagcaca aaggattcca aacagaagtc gggcaagctt ggtttcatca gcgttggcaa
377101 caagtttaag gtgacggatg aaagtttcct tctcttgttc tgtcaccttg actttgtgaa
377161 taaaaagggg cattttggat tataatttcc tgctgtggct ctggttatct cctttagacg
377221 gcctttatgt cagactgaag ctttgatttg ctttgacttt gtccttttaa gtcaaatggc
377281 tccatttaaa atgtttcttc aggccatttt tcttcttctt cttcttcaca cagatgcagc
377341 tgaacctgct gctggaaaag ctccggagta cagtgagtat ttagctgcga ttacgcctca
377401 gcgtcagatg ggcaggcgtc tacgcgtatg tgctgctctg aggtcagtcc tttgcactct
377461 cagggctcaa gcttcatccg atgcgttaaa cccaacctga agatggtcag ccaccagttc
377521 gaaggagctc tgattctgtc acagctgcag tgctcaggtg ggacaaaaac gctgcgttta

377581 tggtttttaa gccactaatt cttcacatcg gttcccacgg aatgccgtgt attcttgtat
377641 taaatggaag ctgaagtgtg ctcctggcag ggatgaagca gaggcgtgtg tgacgtgctg
377701 cgtgtgtgtc tttgctcagg catggtgtcg gtgttggacc tgatgcaggg gggcttcccc
377761 tccagagctc ccttccacgc gctctacaac atgtacaaac agtacatgcc tgacaagctg
377821 acgcgcctca atcccagact cttctgcaag gtgcagcagc agtgaatccg ccaagcttct
377881 ttcttattct tacttactct gctcatgctc catcccctgt aggctctgtt caaagctctg
377941 ggactgaatg acaatgactt caagtttggg ttgaccagag tgttcttccg ccccgggaag
378001 gtaagggtgg caccgaggtc aggaaatccc cgaaagctcc acacgccaat ggtcccgtgt
378061 ctcctcgcag ttcgcagagt tcgaccagat catgaagtct gacccggacc acctcgcgga
378121 gctgctgaag aaggtcaaca cctggttggt gtgcagctgc tggaagaagg tgcagtggtg
378181 cagcctgtct gtcatcaaac gtaggtgcag cacactggaa ccttcatggt tttatcagaa
378241 accctttttt taatttatct ttttaaaatc aggcctcact caccacaaat gtaccactta
378301 ctgtgcacat ttggaatgga gcaacttgca aacaagctgc actattttct aatttctttc
378361 atcactgatt taagccccag gctcgtgaca gactttaaaa agagatgaac aactatttaa
378421 aataagtcac cactctgcag cctcttcagc gcaccgtcgt gataaacagc ctcttccatc
378481 aggcgccgga tgagtggagg atttagctga catgaatcat attcacatca catctactct
378541 cgtaatttaa aactacagcc tccagcagat cgcaccagta ttttagagca gatgccaaag
378601 ccattacgct ctatgggaac ttattggttt tatcatatgc agacgcgcgg gctggattct
378661 gctgatttgt tttatgagca gccgcggtga ttccattacc cgctgcccct gcgtgaacac
378721 acgtgcctct caggtgtcca aaggaactcg atgcacctac agatttcctt cacaactact
378781 taaacgttga aaatggtttt agatggcgtt atcgtgcagt catggctttt ctcctttctt
378841 ttttagttcg gaacaagatg agctacagag ctttggcctg cattaagatt cagaagacgg
378901 tccgcatgtg gctgtgcaga cggaagcaca agccgcggtg agtgtcccct gtgtctccat
378961 cctttactgg acaatgggcc ttttggtttt ggggcttttc tgtgaattgg agacctcaaa
379021 tcaatgcgga aggttatctg tgggaaagtg atacatcact gttctgttta gaggaaaata
379081 tacagccctg ctgcaccgga gggtgttttt aatggctctg gggaatgcag ccatccagat
379141 gattgttttc agatagcttt ttaggaacac gtttggatcg tgagcggcag tgtgtatcta

379201 tggctgtgag ggaaatctta catagcagca tgtacacgta ctgtctggtg ctagctgata
379261 tgtcatatac ctgtttttaa tgcaaacaaa acagaatggg gtgtattccg tgtagaatga
379321 ataatagtga tgatgattaa gagcagctgc taattgttca gtttttggta ggctgttgtg
379381 ccttttgctc gtctaaatct atgttaaagt tgtttctggt gctttacagc atcgatggca
379441 tggtaaaggt caaaaacctg aagaagcaca tggagcannn nnnnnnnnnn nnnnnnnnnn
379501 nnnnnnnnnn nnnnnnnnnn nnnnnnncgt ccttcttcac ctcttttata aacagaggat
379561 gtcagcacgg tgaataactt tttttttta ccttttctca gaatcaaatg catcgactca
379621 gttttaaaac acagacacat tcagagaaaa aagggatcga taagaattta gcagtttgct
379681 ccattatatc agctattttg ctgtcgtctc tgtccaacca acaggtacct gtacctgagg
379741 cacctgtgac tcatgagccc tctcacccag tccacctccc acgtcgccca tggatccctc
379801 tagcgtatta gcatattagc atctctagtg tcggctgtta tggcagtagc gctcccaact
379861 gctcaattgc gatggtgtgt cggggtgaat aatgcatgag ctgcatcagt tgccatctag
379921 gcctcagtac ctcggggaca ggaccttgtc attattccac ccggctcaac tgttagggag
379981 cccactaatg aatcgttcca tcgcggtgac ctctcatctg cctgctgatt gtctaacaag
380041 catggtgggg aatcggcctc agagtgtcag tcagcaccgc cacatgggaa agctgatgca
380101 gaagacgccg agtcacggtg aaagacccac tagcaaggtg ttggaaaaga gaagcaataa
380161 aatgaagatc cctatcccgc catttttag ccactgaaat cttacacaaa ggatgcagta
380221 ggtatggaaa gtattagact tccatgttta tggtgaagtc tcaggtcaca atcactgggg
380281 gtcctgggag tcttccactc tgccataaag cccagttttt tggttgtcgg actgaaacct
380341 tctccacccc acacaggatc agacatccag ctgtaggaag aaccctggaa gagtcctaca
380401 tgtctttcgt atgggaatga tggagcccgc cgtgctctta gaaacaataa ataaagcaga
380461 tctttttct aggctggtcc agatctgtct acatctccct ccctgagctc tccaggcagt
380521 tctattgact cttatttttc ctctgatctg cactatcaac cctatttaga cagctgtaag
380581 ccttaaaatc cggctctaat agaaacaata gtgagtctgt ttagagattc agtgtattcc
380641 ctgattttct aacatcatct cttcttcctc ttgactcact ccaggccaca ctgaccatca
380701 cctggaaaga gatcgacaca gagtaccagg ggctggtgaa gcgctcggag cagctcctca
380761 cctccatgca gaagaaaaag caagaggagg aagagcgaga gaggctgaaa aaaattgagg

380821 gggagatgga gaaagagagg aagaggaggg aggccgaaga gcagcgacgc aagcaagagg
380881 aagatgacag aagagtgtaa gctaaggaca cggaggggac agcggagggg ggagtgaatg
380941 taatggtgta acagtgagcc tttttcctct ctccacaatt tagtaaagcg gagatggaga
381001 tgaagaggaa gcaggaggag gaagatcgga aaaagagagc ggaggaggag aaagcgatgc
381061 aggtcggatg ttacttatca tccctcaaat tgtcgcacgg gccggactcc ctcccgtcat
381121 cagtgcgatg gcgagcgagt ggtgaactat gtggtgcctg atttatttca ttagtaatca
381181 caatcacgtc tgaataattg ggaattcttt tttccctctt tggccaagaa aatacgagga
381241 ctcttcagtt gcatcgattt aaaagtttct cctcggccca aagggaagct ttttctcata
381301 ttcctcatat tcaccttatc aatatctgat atctttcatc tatgttaacc cacagctgtc
381361 gtacagtttg cacatcgacc ttcctctgtc ctccacggac gccaccgcgg tctttgttgt
381421 ccccggatcc atttgtcatt ccggtatctg ggcagtttgc tctaagctgc cataatgtgc
381481 atgactccag agataccctg caaaatctgc ctctgcggcg tgcgtggatc ccatctgcaa
381541 cggggaacat ttgggaccca gacgtacccc gttctgactg ctttctaaca cgtccactcc
381601 cgcgattcac cagaccctca tcagtttgtt ttatggctgc agcacatcca gtaatcatct
381661 ctctcacagt cgtgtaatga atgtacttca gatgtgagcg atcggtctca tgagatgttc
381721 ggggggggtg ggggggcggc taccgttatt ctgagagaac ggtgacttct gtttcagcat
381781 ttagatgagc tgttctggaa tataatgcta tataatgacg ccacttatcc gctgcagtaa
381841 agagccaaca ctatgaataa tatatattcc taactgggga ggcgtcggta tataaacctc
381901 ctccattctg gacattattt gggccgttct acatcatttc acatgtgtgc tgccactgtc
381961 tgaaactgca tataaatgaa aaggctggaa gctgctgact gctcttgacc cgcgctgtgt
382021 cggatccctt gttttgtcta gacaaatgcc caacagagct ttattagcag cataaaacct
382081 ggataaaaat gtcttaatcg ggattgggaa agcagcaggg ctgcagagtt ctctgaggat
382141 caggcaggaa gccggaaagg aggtttatgt ggagtttaag agcagaaatg ttgtttttgt
382201 ttcccaggcg gagctggagg ttcagctggc tctggagcgg gaagagcagg cccagcgggc
382261 caccatgctg gagcaggaga ggagggaccg ggagttagcc atgaggatcg ctcagagtga
382321 ggcggagctc atcagcgagg aggggcagac ggacgccagc ctgcgcaggt gggcggcgcc
382381 agcatcagaa cacctccggg tcacacaacg ttgtcgctct gacgtcagct tgactgtgta

382441 accgcctgtt tccttcttgc tgtcttttgc cgtagcgctg agtctttttc
agagcttcct
382501 atctcgtcat cctcagccag agccatgtaa gatcccagct cagcctaacc
gttgtcccga
382561 gcacgccgaa cacgagcagc accttagtcc tgaacacatc cgctcattaa
aattgataaa
382621 gcagtcataa taatgcagcg attcaatgac ccggcgggcg ttatctgcac
cgtctgcgtg
382681 gatttatgtg tacttagtgc ttagagaaca gtcataattt agagtcttaa
ttactaacat
382741 tcattagctg tgtagaatta tagggtgcct tcaggtccat acaatcctca
tctatagttt
382801 ataatcaaat gctgttgatg gttaattcat gaaattcact tggatttatt
tcgctggggt
382861 aacattgaat ttttaaggtt cccctccaat ggagacaaat tcctgtagct
cgtccccgtc
382921 cattcggtcc tgtcctgtag ttttggtcta atccttttaa taacaacatc
accaaccaaa
382981 ggcgaggtga atatgtaaca ctaattatta cttatggact gattgatgcc
taaatgcctt
383041 ctaaaactgg actcctcacc aaaaggtgag gtctcgagat ctagcgcatt
atctcgctgg
383101 catcacctga acgccacctg aacgccacca caagcagctc tgctgccaag
aagcctccga
383161 gcctcacgtg tgtttcacgg ccacgagcgc gtgcagccag cagcagcatt
cacacacccc
383221 tctcgctgaa gcttctcttt cccgctcacc tcctgccaca tcatcggcac
acgcttgatc
383281 cagaataatc ctgaatccgc ttcatcgttc cagtccatag gaattataaa
tgctcattgt
383341 aatctggcta accgttgaag gatcagtaat cctgcaccct gagacattag
cagctacacg
383401 gactgtaaat agaggagcta aatccttttg tattattgaa ttagcagact
cagctcagca
383461 ggggagctgg acacctggct ggccaacaaa gtttggcagc ggattcctgc
tgcctcattt
383521 ttccattttt ccgtgtgact ttgcctgatg tcataattct gggggtaaaa
ggcattttac
383581 aattaaaaga tcatggtgtg agaccacaat cagctccccg ctgagctcct
ttaatgttca
383641 tttaatcgga cgactcgagc agaaggacag acagacaaca tgatgcagtc
aaagcataat
383701 tatacctggt ttttcctctt catgctcgtg gcttctgcct ggaaattgac
ctcctcctct
383761 cagccagatc caggaaggtg acataacaga tttgttttag ctgggggggg
ggttctcggc
383821 gctgtaactc cgacgaactc ccgtgtgcat taagaaacga cctcccacca
gagtgtccga
383881 acaggatcag atttatagtc ggaggaaggc agaagccctc aatccactca
ctcgtctttg
383941 tctgctcctc gccacagggg tcctcaggtg caggcaacca aagccgccgc
cggtgtgaaa
384001 aagcacgacc tcagcaagtg gaaatacgct gagctgcgcg atgtcatcaa
cacttcctgc

384061 ggtgagaaat tcccaacacg cccacattca gcatgactcc gccttaatca aagaaatggc
384121 ctgtgtgtgt gtctgtgtgt gtttgtgcgc tgctcttccg ccacattatt tgggacaact
384181 gataacggct ttctgcggcg tgcaaatttc ggcccccacc ttctcgtctg tgtgcgtaat
384241 ctgaggatgc agctgcatga agagcgtgcc cagaaagtca aaagaccaat ctggacgctg
384301 caacagctgt tagctgctca tcagcaggca gttaaatgtc ggagagggga aattatcgcc
384361 attacggaca atgacaggcc cctgcccttc ctggccgttc cacgccagtc atctcacaaa
384421 aagcaccgcc aacatggcgg cgtgctcgtg gccctttgat agctgtgaag gcgatctggt
384481 cagcgagggt cccgttgtga ttccaggaac atctggctca tttgttttgg tgggactggg
384541 gttgattaaa gtggaaaaca gtgcaagcgc cagaacccac aacaaagctg ctgcagggtt
384601 cgcttgaacg cagcaccgct accgctaaaa cgctcagtga cgtatttgat gcagtcgctg
384661 atgtttggag atgtgatcat ttgtgtcctc acagacatcg agctgctggc ggcctgccga
384721 gaggagttcc accggcgcct caaggtctac cacgcgtgga agtccaagaa caggaagcgt
384781 aacgatgacg gaagcgatca gagggctccg aaatcggtca ccgattatgg ttggtcttat
384841 atttgatgtc ctgcgcgtgt ttcctgctcc actgacgtga cctacctgca cacagatcag
384901 ctacttgggc gcggatgaac cgcagcctcc ctcctgtctg gttctgccag aaatatttgt
384961 tctaattagg aaataactgg gagccatgcc ctgtcaggtt tgctagctgc tagcgtgaca
385021 gccttgccac tttgctaagt taatgcaggg cgcaaccaag catggtcacc ggcaccaaca
385081 catgcacgcc cacgtgcaaa tgttatttag ccgcccgaga gctcagcggt gctctgcatt
385141 tcctttcttt ttccatctca tcaacaaata ttaaatttga cggggaacgg ggacattaga
385201 tccgccggtg cctagtgctc ccgctatatt tactcccctc tctcctttga tgtgatggct
385261 cctgaaagcc tctccgcgtg atgtttggat cctgttctgg gttcttcctg ttcttagcct
385321 ggtgcagcaa aggtctcaaa aaggccattt ttaaagaggc ttaaagctgt tttcatcctg
385381 tcaccgctgt tctttgatgg tgtatgtacg gaaggctttg aagtgaaaca acactatttc
385441 catttcgtcg ctgcagctga gcagaaccca gctcccccaa ccacggggca gcatcaggaa
385501 gtgacaatga accgccagca gcgctatttc cgcattccct tcatcaggcc cgcggaccag
385561 tacaaagacc cccacaataa gaagaagggc tggtggtacg cccacttcga cggaccctgg
385621 atagccagac agatggagct tcacccggac aagcaaccca tcgtgctggt cgcaggtgac

385681 tcggcagtcc acccatggag agcaagaaca gaatccccac gtatttactc
atttgtcact
385741 tcctcaggca aagacgacat ggagatgtgc gagctgagtc tggaggagac
gggtctgacc
385801 aggaagaggg gcgccgaaat cctccccagg cagtttgaag agatctggga
gcgctgtgac
385861 ggcatgcagt acctaaagaa agccatcgag aacaagcagg cccggcccac
gtacgccacc
385921 gccatgctcc agagcctcct caagtgaaac cagcgcggcg gtcagatatg
agcacagact
385981 ttgcccggca cccaaatcgt gacgctgatg cagcacggtg tcacctgaat
cccctctctc
386041 gctcgctctc tctctcttag atacctgcat tttatacttt tttgccgtgt
aactcgtgtt
386101 gaatcagcct gtgtttgaat gtgaaacacc acagcggtgc cttaactccc
catagaggcc
386161 tcatattcct gggttacgct gagaaaagcc ttaacaggaa cgtagcctta
atgctaacct
386221 caatgctatg caataagcta aaatgttaca ctgtacgtag gaacacttga
gatagctttt
386281 ggtctatgca gataattcaa aatgtttgaa ttaatgatgg ggatttgtca
tcggcccagc
386341 cgtgacatca gtactggaga tcgaggaatt agcatttcac tgtcatagct
taagtttttc
386401 ctcattttgt gcggcgtatg catgctggat tagtattgtt tagcaatcag
ccaggtgaca
386461 acaacataga ggttttcatg tttgcttggt ttctctcaat tggatttgcc
tcaacggtgt
386521 aatctgcgtg tgacgtgctg gtgcgtgtga gatagtttta agctgattat
tcgcctccat
386581 cgtcgatctt taaacttttt cctcgtcgga aaaagtcacg cggtacctta
attttgagtt
386641 gagttgcatt tctggaggac aaacggcgac acagtcgctg tatcaagacc
ccacttaatt
386701 cacgcaaccc gcgatgctcc gtgatgccct aacagcagct aacaggatta
gcgcagctta
386761 ctgtgacatc caggctgccg taccactgag gtcatccctg agccgaggct
cctacccatt
386821 aagtaatcag cttgattaaa atagaattaa ggcccactgg tgtagatatt
tgcttcaacg
386881 ttaattaacg gcagcgcatg ttttgcttcc actttgaatg tgttttgtac
tttgcttcct
386941 gtcgcatctc ctttgtgttg ttttttttag ctaaaacatt aataaacacc
aggtaggacc
387001 cagactactg cttgtctgcc cattcttcca ataaagtgtt tgacaaatcc
cttttattgg
387061 aaaagaacat ttatttcctt tcaaatgtgt aaaaaaaatg acagcgataa
gtgggaggat
387121 ttctatactg tctaaactta aaatgtttgt acacaataaa tacctcattg
tgaggaaacg
387181 accacctttg ccaagcagga aatgttccta ctgtataata cgggaaataa
atatcaggtt
387241 ccacttcgag accacctaag ttcgccgtca cgcctgcagc tgtctggcta
atgagacata

387301 atcactatca acggtagtga gcaccactct gggatgaccg aggtcaccgg gccccctccc
387361 acacgtcggc tcctcacacc accgtcacgt cagaggagtc gtctgacggc cgtaagacgc
387421 agcacctgtg tctgtgttca cttggccgtc gccacgtctg gaaagaaaag acgggagggg
387481 tgtgtgtgtg tgtggcgggg gggggtgaaa aaggatcatt tcagttggaa tcaccccaga
387541 aatgtgggaa aagtaagatc atttcccaga gatcagtccc gagattccta tcaaatcaaa
387601 acggcggcaa agtattggag actgagcagc gattattaga ggtcatttca aggtcttttt
387661 tttttaccct ccacgcatag aatttgtcaa aactgatttg cttagtatga gagacaagag
387721 ttaaagagct ctttttttac ctttggaaac atcatagagc tgaaaaatat tgacgtgaat
387781 gtcagtaaac acagagtggg ctcctcgaaa ctgtctctct gagaacaaaa gggggtcctc
387841 acctcgttgt gtgggataaa gtggtggaag tccaccatct ccgacattaa cctttcggat
387901 ctggtggtgt agagctgccg ggggatctgc ggagacgaca ggcacagagt gattggcatt
387961 tgaacgaaag agcgtatcgc aggagcagag gcgactcggg gatgctggag ggaacgctag
388021 aagagcgtgc aaagatggcg gccacgccgt ggtgttaggc ggctacgcag tttgaggtta
388081 gtcgttagtc ttttgagccg cgcggcagca caaagtacct cgatgctcag acggatattt
388141 tcaatttcat aatgttccgg ctcggagcgt tgggattggg aggaggcggg actgggcgcg
388201 taaggcaaag tggacacctg agtcaaaaca ccgtcatcgt tctcaaacac ggggttgacc
388261 ctcagggtgt tttcaaaggc ataaggcgcc agagtctgcc tacaacgtaa aggagacgac
388321 gtcacgggga aatggcgaca caaaaacctg aaaaggtgaa ggtgggtgtt gggggtgggg
388381 ggcgggtggg gggtgtcgta ctcacaccac ggcgacggtc tttctggtgc ttgagcactt
388441 cttgttgatg aagattaaaa cgccgatgac ggcgcagacc agacagaggc tgcccacgag
388501 gcaggccgcg atcagcgggg ggtccacggg cagcgacacc agctcgttgc agcgctcgcc
388561 gtggaagtgc cagtatctgc cgacgggaca cctgagagga cacggaggaa aatgtcacac
388621 gcctcaaaag ggcagaacta ataaactcag cctgaaaact agcataaatg agcatctcgg
388681 gctttttaaa gggctggtga tgagttcagg gggggtttaa tctgtttcaa atgtccttaa
388741 cgtccacact ctttttcaca tgagtcagct tttggtccga tctcgacaga ttttactgaa
388801 gaatttggta gaaaggacag tttatcatca tctcagtatt ttatctgaaa atctggttct
388861 gcgttttctt tgaacgcacc atcgagtgag cgctacagcc caaaccaaca cacacaggct

388921 ctcttttacg gtccagatgt gctcaacaca tcctgtcagc cccacagaga agagtaccgg
388981 ctgcaatttt tctgcaggaa gaaaccttga gcaggaccag gctcatgggg ggaggtaagg
389041 taggtgggaa ggagcgacac aagagaacag acatgtggtg tatgttggag tcgtctagag
389101 agagagtccg aatcacatct ggattattcc acatctaatc taggtctaaa cgagtctcct
389161 cccatcaaag atccggttct cctgttctct ttaacctcat cagcaccatc ggctgcttgt
389221 gctcctgcct gtctcctcgc gtgttctgga ggttctggat acctgcaggt ggctccgtgc
389281 ccggggatga tctcacacag gcctccattc aggcagtagt cgggctgcac ctcgcaggtg
389341 ctctggcagg gcagcccccc cacggtgctg tagcccgggt cacacagaca ctccgcctgc
389401 gccgtccagc tgttcaccac gcagcgggag aactcgttgc acgccagtaa cttgcagggg
389461 tccacctggt cggctggggg ggggcaggat agcaacaatg acaccatgag ctcctcccag
389521 cgggttcaga ggaaggccgg aaccagcgcc tcacctggtt cgacttccaa agaccgactg
389581 tcgatctcaa tgtcgagccg cttggaggcg gcgctgcaga agtcctcgag tacacagtgg
389641 accgcctggg tgacgttata cggaaccggc ttgtccagct tcatcctgct gttcaccacc
389701 acgctgccat tcctgaagtt gaggatttcc agctgcttga atccggtcag attggactgc
389761 agatacggca gaagctgtcg gggggggggg tcagacgaca aaagcagatc aatttctctt
389821 gcgtcagttt gaccatttct tgtctaacgt actaaaaaaa aagacagtgg ggagaaagac
389881 ggagtctacg gggacatgaa gtttccactt gggcgattag gttaatgaaa tgttgttcgt
389941 gtcagtcaga gagacctgaa tcctcctgag aggagcgcag aagactcagg gtctgacctc
390001 cccagtcaag ctgaggtcgt cgttcccctt caaaaggtct atacattttt catgaatttc
390061 ctggaaacct aaaaccgccg tcctgaccgt gccgtccaca ggcagccggc acaccgcaac
390121 caccatcaag aggatctgta ttggacactg gacggcgcta atttagtgtc acgcgtcaca
390181 tctggtgcca cccgcgtcag cacgttcaag atcagtcgtt actctgtgct ttttctcgcg
390241 gaaaagcaga aagcacatga aaatgtattt attgtccaga tatgacagat ttttcactgg
390301 ttagaagctt acggacatcc cgcagggcaa caaagacacg tcaaatccaa aataaatcct
390361 gggaatgaag cgacagcgac cgtccagtga cttacgttaa gtgctgatgc gcagaacgca
390421 tacaaacttt gtttgaactg cagcttccct acaaagaaaa tgtactggaa tggcagcttt
390481 aatcttattt ataaattatc cccatggaga tgttgggtgc ctcttcaaag ttaataagct

390541 agcattgaat aatgctaatt tagtactctg ttcttcctag ttcctatggc tagaagttaa
390601 gatctatttt cattcttcat acagtacaat tagtcgctga gctcattaag ctaaactaac
390661 aaggcagtaa agatcataaa aaataatgtc attacaagta aaatgtagag aaaatagcca
390721 ctgtactttt atccattcca gcgctaagct aatgcgctat ataatgatgc tgatccagaa
390781 ataaggttgg gctcaactct gtaacccaaa gtaaatttgg gggagcacgt cgtttctact
390841 tgcaggagtc ctgcaccttc aggtaactga tgattgcagt gaaactgagg atgtcaacca
390901 gcttaggtaa tgcttatcga tctggaccgc acacctcctg gccattccac cttcctcaca
390961 aacgaggtaa acatcaaacg gctgcagcgc cagagggagg agattacgat actttagctg
391021 gccccggttg acgagcggga gattagagac cttaaggagc tcttctctga tcaggttact
391081 tcaccttatc accagcagac agcgcacaga gggttagctc catatagcta atagcctctg
391141 aaaaaaaaaa gaggggccag ccagaggggt catacatcct gaacaaatgg ctgcgagcca
391201 acagctggat aaggctcgtg gtttggtcag ccatttgtct caatgcacga atgtcaatta
391261 caacaagctc actgcagatt gaatacaaat ctggagcgct attgccttcc acgcaaggtc
391321 aaagtcaaac ccccagcaac cctcggttca gcctctagag tggatggtag cacaacaaca
391381 tttctgctca aagagctttt gtaagaggca gagacgccac cgacagccac attaacgcca
391441 cgtcagggct catctgtatc tactggttca tgtctttgga aacatctgtt ctctcatgtc
391501 aagaagaaga aatctgaccg gcatgctatt atgctaaatc agttccaaac tccatgtcca
391561 atttttacag gttgcttcac aaaccagtag gtacaggagt gagagaaggg ggtggggaac
391621 attggggact gcaagccagt ggaagctaac agttcatata tagcgtatat atgaggaaat
391681 atctgagtaa tatacgaggg aagaagcgta gagcattaca aatggggcct cctcccctgc
391741 tagacattat gtccgcttaa atgggctgca gaatagatac aatttgacaa ttcatccatt
391801 gcatattttt ctgctttttc agtaaccaga ctgaaccggt tcggaatatc tgctcccaac
391861 tgtccctaaa agcccattta aaggataact ggttggaata gcagagagca gatgcaaata
391921 cttaccattc caaacatctg gccacattac ttttgccgag ggactaaata tattgattaa
391981 tagggagaat tgcagagacc acagtttggg caaccattaa agattggaga tggagtgtta
392041 ctttcaatgc agtgggggga aaaactgttg gaaaatggat aataaattac tctgaaagca
392101 tacattcagc tatagatcta aatctggaac atttgttcct cacatgctcc cgtagctcac

392161 atttcctccg tgacagaaaa tgcacaaatc caatatccat caatccttta tgtagcgttg
392221 tgaatctaag caagtctaaa aatatgccca tgcccttggg gtaatctgaa actgagacca
392281 ctcttcctgc tgtgaccctt gctgtaaggt gctgggttgg tgtcacacct gtaagaccgc
392341 ggctaaggcc aacttccaac gttgaggaga gataaaggat gtattgccac atgataggga
392401 agaggaaaaa tggcaacaat agtagcagtt ttcacatgaa tgtgtgatca gaaaatgtct
392461 ccaaactgtc ggctgttgct ttttatttga gttttgttgc atagtgtaaa ccagcgcgtt
392521 atagcagcca ttgatgcaac atgcatgcta acgtccaagc aaaactgact ggtggcttaa
392581 gcagattggc tccatttata gggcagagtg tgtgtgtgtg tgtgtgtgtg tgtgtgtgtg
392641 tgtgtgtgtg tgtgtgtgtg tgtgtgtgtg ttgtgtgtgt gttaccagaa ttacataaag
392701 cttcttagca caagtcaaaa acctctgagt cctacaatga ctaagggaga cccaagtctc
392761 atgattccaa acttccctgg ctagtttgca ctgtaacatc tacaaaattg atccagtttc
392821 ctgatataca aatttcacaa agctgcccaa ctctcgctcg tcacgttgtt ctgcggtacg
392881 ccgatttatt tacactgcag atttgttttt aaggcttcca acaaacaggt tagtcttctt
392941 gagaacagac aaaaatacaa aaataaattc tggattttct aaaataaaac atatgataat
393001 atccatggtt gctagtacaa aacaccttca acaaggagca tctatagctt cgcatgagca
393061 catcaaaatc agattaatgt taaaagccca acatctggat attgctcaga tttccaccca
393121 gtccacagac aatgttggaa gtctttgtcc acttgggtgc actggtatca cgatttgagt
393181 tcccaacttt tagcacattt gtaaacatta aggcaaaaca cagaacaaaa gagccacaaa
393241 gtggattaat gctaaagcag accctaaagc caagggtgtc agtaatgtta gtgacgatct
393301 gattgttaga tactatggtg caggaaggtt agtgaaggta tcaaaagctc tctgaggcat
393361 cggagaggta gttccaacat ggaaagcgaa aataccggta tggaggctaa agttgtctgt
393421 ctcctaggcc ccgatttact ggaagctcca gggtttggcg agtttctcga cccagagtgc
393481 tgtgtctgag tgaaacacaa taactatata gcttagcttg taatattact gcacattcat
393541 gaagaatttg gcgacggatt accacatttt tttaaaccat gcaaatatat attaatgtat
393601 aattgtttac cgtagcataa ggcttcctat aaggtgcact ctcccgtgtg cagaacaaca
393661 ttttgattaa acatcaacta aacaacacaa agattgtcct aaactaaaat gtaaagtagc
393721 aaaagtttcc ttcaaacttt tcccacactt taaggttgac ctataacaca aacaaatatg

393781 acaatgtagc atgcatgagc caaagatcca gcacaaatgc tagtgtacct
accagctcca
393841 gaaaggtatt ctccagtgat ttatattctg gcgagctctt gttgaacagg
tcatcagaaa
393901 acattatgtt agtgaccctt aagctaaaga aaaccactaa ctcttggttg
ttatccacta
393961 tgttggtgag aggagtgctg gcttgtcgca aagatggagg agcggtggat
tcatagggac
394021 cctcgtcaga ctccgaggcc aagccgctac cactcccttc ctcctgaagt
tcgttcacat
394081 ttgcctccat cacgttggga ttggaagcaa cgtcctcctg aatgtctgag
ttctccagcg
394141 atacgtcagg cttaactcca ctctgccctc catggcctcg gccctcggag
tcctcgccat
394201 cctctgaagg cggcggcaga ggctccactt ctgtttctgg tttaatgcct
tggacagcgt
394261 cttcgtgaga tggtgccttc gaagaggggg ctgctggggc tggagctgtt
ggagctggag
394321 ctgccaaggc cgggggtatt gaggctggtg ctgtagatcc tgcgactgac
atctcagtaa
394381 tggtaaaaac cgcagcctcg gtctcagcaa gcgtggacgg agtttcatct
tttggttggg
394441 tggctggagt cgccgagtcc tcagtttgcc ccacaggagc tccaagatca
ggttctgtaa
394501 gtacaacgca tgagaaaatt gaaagaaaat attgataatg tcaaattggt
atgatgtaat
394561 ttttatggtg tttttagtgt gagaccagca gaaatagagt ctcactgatt
ttacattcta
394621 gagtccatta attaactatt tttagacatt ttatacacaa gcaatgctaa
tataaaagac
394681 agtgtttcta ctctcagtcc agaaagccta gctaatgtgt aggatctcag
atggttccat
394741 ccagctaatt gtttggttct ttgaaatcaa gtacaatagc tgagctgtac
cagcgccact
394801 aagcagattt gtgaactttg gaaaattccg ggctaagtat ttcctcctgc
ttcactctgt
394861 gtttaatcaa attacaccag tcttcatcgt gatcataaat gccacctaaa
aacaccattt
394921 ctcataatct tttagctttt tcacaggtct cccgtcatac tgatgatata
attccacatg
394981 attctggttt ggaaaagtga gtaaattact ccaaaatatt tttgctgttt
gttatcacca
395041 cacagattct tccctctttt gcaagtgtct catcactggg taactacgtc
tccacctcct
395101 ttgagaaatg ccccatgcag gtacggcgcg tctgggtatc gatcatttta
cgcgacttgg
395161 gtcactcggg agctcgtggt tttaaagaga gctaaatgag acatggattt
atacataaaa
395221 agaaattact tttttgcttc ccaggtttga gtgaatacac aaatgattca
gctggtttgc
395281 tgagcacttt ccaaccatct tcattgtgtc gcctttccat tctgtccaat
cattgcagtc
395341 ttttttatag atcaaagggt gttactttct cctgttttag aggttctaaa
tatctccgca

395401 tcacactcat acacacagct aatcagtaaa ctttggctcc ttcaatttga
ttcgattaac
395461 ttattgcacc ctgttttaaa taaagccaaa tgatggggtt ttacctgcag
tcaggtgctc
395521 ttcctgttca gtctcacctc cgggaccctg tcctgtgaca gtttccagtg
ttcttacatc
395581 agcgggttcc tctgcagtcc cctcgggctg ttcccacagt cccgataaag
aagcctctgt
395641 ggcagcaggg gtgacctctg ggaggatgtt cgggatgtaa ggcaaaaaag
tgtgtgtttc
395701 cagtttgtgg agactttcta aagagttctg tatcgccgcc acagtgggga
agaaactttg
395761 agtgggcgga acaatctccc cgagtgtttc tacaatggtg tcctcagata
tgatgtcctc
395821 ccgaccaaac tgatccgcag gatagattgt ggctactgaa gaagagcagt
taaacattta
395881 gctttaacct tatatgtcaa gaagtcttta aacttgcgtg gcgatgatgc
tcaccggtct
395941 caaaactgag cgtctgaatg tccaacggca gcagcggatc ctgtttcaag
gccttgacaa
396001 tgatgtattt cagttttggg gcattagcgt cctcgtccct ttccttgtct
ggcccctgcg
396061 gaatgtcggg atcatcgcca agctcggtct ctccgttaaa gaccacagca
taacgtacag
396121 acacgtcctc caagctgatg gaccgagcaa accgcagtca agaaagtgag
atttctgata
396181 tcccaaccag gaatactcgg atctaatcct gaatggcagg tctaatggaa
aaatgtgtgt
396241 ctcacgatca gctcaccgaa atcccaaaac acggatctcc ttgaagcctg
ggactttttc
396301 gagtacacga cttagctgtt gagaaaaaag aaagaaagaa aatataattc
atgtgtcaga
396361 aaacagtcct gtgctttatt tggctctcac ctaaaaagaa cccctgatgt
tgttgcattt
396421 ctccttagaa aatacctaaa aatctttaaa gcaataagct acacaagaaa
ttctctcacg
396481 tatgtatcag acccagaaac gagacccaag cacacacgtg tttgctgcat
tattttcttg
396541 agcgcactgg ctgtcaacat gagatggatt ttatcttata atatctttaa
tactgctgca
396601 atattgaaat gttatctgag tccttacagg caacattcag ctctttaata
acaaaggaac
396661 tttaatcagg ggtgagtttt acaataaagc ccattagcgt catctcccaa
aatattacta
396721 cagtgtcttc cagccctctg aaatcgaagg tcaccttggc agtgagttct
tccttaatgt
396781 cgttgtattg tgacccatcg ggatcacgaa gcagcttgct gtactcgggg
tccacgatgg
396841 tgaccctgaa ttcaacaatg tactcctcgt ttttctcttt tttggcgtcg
gggtcttccg
396901 tgatctgtca ggtagaagga accgttttta aggatattac agtcgcagta
agttggatgg
396961 agttcggaaa cttactgcag cagcgttggt tgtaagctct gtttgcatcc
agttgcctga

397021 gagagaacaa cgactttagc tcatcactgc cgaagtcata aagttgaatt agtgttccat
397081 taactcacat tctcttcctg gccctgaagt agcaaaccta aaaagcagga tttaatcgta
397141 gtttagctga gggacagtca agtaaatgag atcatttcgg gagcttggca cgtacccttc
397201 gaggccgcct ccttcggcta ctctctgcag gtcagaaaca gacggtaagc tgctgcaggt
397261 ttaaagcaga acatgaacat tggctgtgga ctcactctgg ccaccacgtc ctggtgctcc
397321 tgggagctac tgaagttctg cgccaggtca tccatgcaca ggttctggtg ctggcaggcg
397381 tagacccaag ctctgtattc ctcagaattc gggaccctgt tgaagaaaat tctaaatgct
397441 tcccaaatcg cctcctgaca aactgcaaag atgtaaagaa aggcaaaaat aggagatttg
397501 agtgtgtctc cttggaaaaa cattttaatt cacctttatt gcgacatgaa cgccctaaaa
397561 ttcattaaaa cattcattaa ttcgccaact gcttcaaccc gatgtgattt aaatggagcg
397621 gcagcggcgt ctgttccggt aaaggtcaaa ctgtgcatcc aacaggaaga aatattaaaa
397681 ggctgagctc aaagacgcca cctgaaatta cagtgccttg cgttttattt gcatcagtgc
397741 gagaaactaa atacacattg tccaatatcc ccaagcctgt gggaaatttg tgcgaaacta
397801 atttaggaaa cgatttgatt accgccagca aatgaaacca accttgtttg gaaattgcct
397861 gctgccgcag ctgtcgcaac catattttaa tggctaactg cctccgacgg cggcggcggc
397921 ggcggcagaa gtcgccgaca aaccatcctc tctgaacaaa gcgcgcaacc aattttcttc
397981 aaatgcatca gagcaacagt ttcaaaaagc ctaattgtgg aagggaaaca tctgtttcct
398041 aaacagttag catgtttaca catgtgctgt ctgtgcaccg aggagatcta caggaacgtt
398101 gacaccttcg gggaaattcg gagttgaatt cacctctcag cttgtaatat gtgcgatggc
398161 tgctgatgac ctccttcatc gtgtcctggg gacacacttt gacccctgag ccaaacactg
398221 gcgacctctt cggccggtgg ttaaagcgtg ccgccacgtg cctcgctggt ttggagacat
398281 ccaagaaatg tcggtacttt acatcccgga gtcccggtaa accgtggttg gagacgtctg
398341 tgaatcagaa tcaaaaaatt tgtaaggaag gcctcgatgg ctgtgatcct gggggtccag
398401 tttcactcca gagagaaaaa aaatactgtt tactgtctgt ttagtacata cgccggatca
398461 tatggagaac acgggctgtc agggaggaag ttgccctcaa gaggtgggct gaacattggg
398521 gggatggtaa cttgcttaag ttagctttaa ggtgctctga aaatgtctca gcaccacttc
398581 tactcccagc ccactttctc tctttttggt ccataactgg gattttgatg tattttgccc

398641 atgtccttgg tcaatggtgg agctaattat gttctgctag tgtaataaat acgatgcatc
398701 aactggatgc ggagctgttg ccatggggag cgctagcatg ataagagcct ctgtggaaag
398761 actgattgaa cactgatgaa ctcaacaaag atcaatttag gtgtggtcac aacccatccc
398821 tgtgctggga gagggctctt caaggtgaag gtctgtggac atctccaagg attcgggcgt
398881 ccagaccttg ttttgcacgc cagaaccgtt gccccgtcac aacatttgca ttcatcacaa
398941 tttacactat aactgatctg tttaccagtt tatcagtgaa tttaagatcc atctgtgctt
399001 gttggcttta aaaccctgct gggttgtttt gaaaaacatc acagagtaac tcttgctgcg
399061 gcggagaggc agcggtgatg gctgcataaa taataaacat ccaatcactc ccttttgatt
399121 cttccaaact ggtcacagtg tcacacggac gtgcactatg aataatttta tccagttttt
399181 accgcaatgc ctcggctctc tcgtgttcct gaaccttaaa tgtcactgtt tttttttttt
399241 ttaaactgag atgctcattt ctcgctggga agaagaaaac ccccacggtg gcaaagagcc
399301 aagaggtgtg aagaggagtg cgtgaagaac actctcgtgt ctcaaaggaa ccgttgccgc
399361 ggtgcctttg ggtgcctttg ggtgcctctg ttgcaccgag gctctgaaac aaggcgagtt
399421 ataaacggca gagcccaaaa gaaccaaaat aaacatccag gtgagcaaca gcatgtgaaa
399481 aatcaggtta caaaagacct cagcgacgtg ttgcaaacgt tacaaaagct gacctcattt
399541 cctgatcacc tcagtcaaga gaatgaatta gtcattacag ggaggcgttt ctatactatt
399601 gtgcaagtca gagcgacaaa caacacagtt agcaactggt gtggaagaaa ggaactcatt
399661 gtagtgtcag gtggaagcct tcagggcgct ttttataata aagcagcctt tatattgaca
399721 cacaacttct gagagccagg gtgcacataa tttgagcaaa accttcttga gtggtgagat
399781 ctgactcaat tcacactgaa tttgaatgac aaagaagtaa ttacgaacag tttctctttt
399841 gcttttcggt gcaggggaag acaaaaaaac tctgacaatc agcgttacac ttggacatca
399901 acaacgagct ctattgtttt tcttcattcc aaatgttggc ctgacaccag tgacacgcac
399961 tggggcaaat atctcccagt ttggccttgc gaggcagaac tcattctctc cgcacacctt
400021 ccaattataa accacgtcca cacgttagcc tgaaatgtag ccaaatcatc agtgccgacg
400081 cggcgctttg aatgggttct cgggaagccc gacccgcaca tgcggaaata gaaagggagg
400141 cctgagcgag ggggggtca tggtgggtca taatttccaa tgcggccaca gaaagaccgc
400201 gctgtagggc caggagagat tagcatccag caacactggg aacagaatgg cttttgtttc

400261 attcaggata atgagctcac ccacgcacac aaagtgaccg taggcgcaag tattactgct
400321 gtcgctatca cctgctcccc cttttacagt gaatatctgt ggcctttaaa ggctccctgg
400381 ttgtttgagg tgcttgcaaa agtcaaatgt ttgcatcagc cagcacgaga gagaccttaa
400441 ttcgatatag cagaggatga cgttaccatc acttaagttg ccgatgcctt aattagaccc
400501 ctgccagtat actctcacag gtagaagagc aagagagagg aaagctctgt gaatctacct
400561 acatgaaaca aacagttcag atatggaact tttttttct gatcaagact cagactagat
400621 taaaaactct caaataagtt tggagcccag aaagtctgca ggattcttac ctttggcttg
400681 acttgattgg agcacaaaca cacacaaggt gaggaacagt ccaaccttca ggagcatggt
400741 gggctaattc aagaggtttc ccaagtcctg agctgtcatt tgtaaagaga agttctgcaa
400801 acctaataaa cctgagacag actgaaaaag tcccctcacg agggtgacca caaagtcttc
400861 aggttgtcct gtccacttct gtcccagctc cagctcagtc ctgcagggat ttaggctcct
400921 ggacaccgtt gagtccctgt gacggtggtt gcatggatgg atgagcagag caaacaaagg
400981 agggaataga aaaagaggaa tgagagatgg cgaaccggtg gaattttggc tcgggagtct
401041 ccttctgcat ggtgtgacaa cagttgcgag gttaaaaaag ctgctctgtg gagtttaatc
401101 tctttacctt cttctcgctg cttgtaatcc gtattgggcc acgtggccgc ccagcctcat
401161 cctgttaggc acctgctggt ccctcatctg tcagctgagc tgaacttgga gatctaaacc
401221 agcccagacc agcaaaccac tcccaacagt ggccactggg cgtgatcgct gacacccggc
401281 cccttctgag cgtccgtctg aataaattca aattcctccc tgggagcagc aaagttgggt
401341 caggatcgta atcagtcatg acattcaacc gtttgagctt tctgcatggt ttcacccctt
401401 ttatcaccgt ttccccgagg aaaccctcac atcagtagcg ggaataatcg ttagatcaca
401461 cattgaaaat aacagggagc tctccttgga tgccagctgt gaagaatcaa gggtcagagg
401521 tcaagatcat tgtgctgtgt tcagacaggt gggatcaaat cattctcagc tgcaggtgac
401581 acgtctactt aggcaaacaa cagcattgaa tgttgacaga gagttgggct tctctatcag
401641 cacagatatc gacctgtttg ctgactttaa ccagttcagt tagcccgcct ggtcttaaag
401701 tctttaaaga agcagaaaga tccgacggga ggccaagttt ataaatagca tagcacattt
401761 tggttttagc atgtcgctgc ttagctttga gagttgccca aataaatgtc agtcacaatt
401821 gttatttaac tcattacacc cccaagtgaa gtacaccaac agtgtggcaa tcaggtgaca

401881 gtcatgtgac tgggacagga agtaacctgt aaaaatggtg tgatcatctg tatacaaaag
401941 aattttgata agcttgttgt tttttttgc agcaaacaga attctaagca cacacacaca
402001 gttcaagaac tttaaaaaca tgtcctaaca tgtaacttgc tcttgttaag tttgagctta
402061 atgttagacc ccagtatgca ggggcgaaaa acactggctt tattgccgca agtcaagcaa
402121 aacaataatc ccagcaacat tcctctccag agtcaaaatc aacaacaata aaaaggaaaa
402181 aatcaccaca gcttgcaggc aaaacagcac agtagactaa ctggagtaga atttactgga
402241 aaactgtgaa gccgattgga cggcgcttgg ctgaaccaga gagtacgacg ggtatttgaa
402301 gcccttgtga ttgggatgga aaccaagcgc gcctcattgc caggatcact ctcatggttc
402361 ttatgattga aggtataatt tagtatgcta cgctaactaa ctcatccaca gatgttggga
402421 tgaacataat tagcttttaa taataattat cttatgtaaa acaaaacagt aaaaaaaaaa
402481 tcattatttc aaaactcttt tttttggtgg attcatgctc ataatttccc ccagcataaa
402541 caaatcctcg tcagaaccta ataggatttc actgagataa gtttgacctt ccctggctga
402601 taacatgaat cactgtccaa ttagagggat cagtttaaat tatgtaacag aaggaaagtg
402661 gtacttgcca tctgtcgaga ggagttattt cattagggac acaaccgggg tgtctctggc
402721 tggcagcgcg ttttttcggt tttgtttcga ggttttttgg tttttttcgc aaggcggcct
402781 ctgctggttc atccatcatc tggagctccg gaggcaaagt tcccccgcaa aatatattga
402841 gcatttgatt gcatgagaca aattatttat tttatgtgct gcgaattagg ctggtgatac
402901 tttgtcactt tgaaaaatgc cactgagttg tcgggcctca agtatcttgt cggtcgtctg
402961 tgctgaaaca tctgccccaa tgccaaagat cactcaagtg gtttgagctt cgataaatatt
403021 atgtttattt aggttttttt tcccagaaat gcagacagat aaaactttgg tttttgatat
403081 ttttttaat caagccgtcc acagaaacgt accgaggcat gaatgacctc tgacctctgt
403141 gttctactgc aggcacaggg tctctgccag cctcttgtgg ctccaatttg ctgttctctc
403201 acgttttgtt tgctcctcac acacgttgag ggagggaaaa gcctgtgttt ccagtgagat
403261 cttagctttt atccctgcac taaaaggctg aggagttctg cagggctgat gtctaatatt
403321 agctgcattt gtccactcag gtttgggaaa atcctgcgtg ttgtgatctc tgctgtgcct
403381 gctgcagccg cggtggcatc tacggtgtga aaaagctcca acacgaatct gaccagttaa
403441 tcttaaagtc tagtgatgtt gtgctttgat gttattgtta aattattttc aaaatgtggc

403501 attgctcatt aggttttttt ttttttaatt tacaaataaa atatatgcaa atattgcaga
403561 cagtgtccag caaataaagg atatccatcc tttgatacca caattggaag tccaataaag
403621 gtttaggata catataggat ttaaagttga gaaaaataac tgaaatccat cataaattct
403681 ggcgtctgtg cctgatcctt gtttaaagac aacaccaccg ctagagggca gcacagacgt
403741 ctgtcctaca ggaggcaagt tggtaataaa gaatctatga atcctgaaag aaatctaaag
403801 ctaaaagtaa atccacatga tttaaggatg aaaggaaatg ttttcaggag atgagggaag
403861 ccaaaaactt aggaagatac cgaactaatg tgagaagtgg atcaacatgc agaaatagaa
403921 agggagcact tgaatttaag tgaccagatt ttctgtgtac acagtttaaa gcagtaatga
403981 atctggacat gacaggaagt tctgattcaa cattatttgc tgcaacaacg ttgtcgaggt
404041 ttaacaaatg tttttccaac tccagacctc tgcaagcttc agaaaggaag gggaaaaaag
404101 gcagtgtggg gaaaaaatcc caacatatcg agtgagacaa aaccatcagg agagatttgt
404161 ccctaaatga gtccctcagg agatccttca gggttgaagg aagagaaacg tgatcaccga
404221 ttgtctgaca tatctacatt ataatgaact catacatcct cttgtgtgca gtaaaaaatg
404281 actgagtcgt ctgcatacga cagaagacca gaggtggatc gagacgtcca ctgaagctga
404341 gactttgaaa aaaagacaag tgttcatgag gaattatatc caaagctctg tctggcaggt
404401 ggaagtctgg agattaataa ctcttgtctt tcttctccgt atgaggtgcc agtttagatg
404461 cctcagagct gttggagctg cagatgagtt aaaagccaca gttcccaggg tggcgcaaga
404521 tagatggacc cgcggcggcg agaatctgat gaagtgattc ccacttcatc ccgtttccct
404581 cgggcaggct tcattgtatg cagagtgagc aagaccaggt agacagatga cacagaaaag
404641 caagagggtg agaggcctga tgttggagga ggaagaggaa gaggaggagg aggagagagg
404701 gaggcgaaag gagggagtgc tggaaaagga ggtcaggtca attgacagcc cttcagggat
404761 gcctttgagt tgcggcatat taatccgcac ctgattgcag cggagtaaat ccgctgcata
404821 atgtaggacc cgtggaagct gcgcgagtcc ggtggcgaag ctgtgaagaa gccgagatgg
404881 aagtggaggg ctgaggcgca tgagcccact ctgagctact gtagaaacag aagcatcatt
404941 tattcataaa gctgttttca acatcaaaac ggcagtgtgt ccgccgtggt cacgataccg
405001 aaatctctga cttggtttcc gtagctcgcg tggtttcctt tctaaattga aagtttgttg
405061 gaggatgttt tacagtgaaa tcgtagagga gggagctctt gattaatttc acacagggga

405121 tggagtcatc gcagggggca cgtgaagaaa aggaagttat taaagcaaca
aagaggctgg
405181 aggccttact ggatatgagt gtgtcaggaa cattccgtgc taatatatgt
ggcatttggg
405241 gctgttatta taggcaaaat gtcagaatta ggaacatgtt ggtgcttttg
atgcttaatc
405301 taaaaaggtc aatagacgat atgttaatga atcccgaggt gaaggaaagc
cttcatttaa
405361 atccctccag gctctcgaca aaactgaaat ccatctgtgt ttgatgagtt
taaaaagaaa
405421 taaatcttgc aaatatgcaa cattcagtgg gaaaatgaaa ggtggtgagt
gggatggggg
405481 gtgggggatg tggcgtcgtg cggcgacctg ccatctcctc tgttgtactg
ctcttgattt
405541 taaaaaaaga ggactcttga cctctgagcg gagggttcag cgcatcagac
gagaacaaga
405601 catccagatc ccaccagctc tattttaaaa cagtcactta cttttcctgc
atctcatttg
405661 ctgcctctcg gtaatgagag ccagattcag aggatggaca aagttgctga
gctggacccg
405721 ctgactcgtg ccgcaggttc ctaaggtgca cactggagtc aaatacctga
tacagaaacc
405781 ggacctgatc tttgaaagat ttttttcttt cgggtttcaa atcaatcaga
gaaaataggc
405841 agggtttggg aggaggacaa ccgtcttagg gcgccgccac cgggtccctc
ctctatctca
405901 cttaacctgt actcattaca gaagccctcc gacatcaatg tagataagat
aaaatttaga
405961 ttattcactt aacaacaccg ttttgttggg agtttaacaa aatcctcatc
ttacaaaggt
406021 gaatttaatc tgctgactct ggttaaaacc gttaaaacca ccctctaatt
ggggctataa
406081 ggctctgtga atgatgctgg ttaattgtca ctgttcattg agcaacgata
cacaaagctt
406141 aaggatgcat cctactgcac gctcagcagt atccatccct cctgaccgcc
gctgcatcat
406201 tgcctcatat atttcatgtc aggcccaagt ggtcgcgctg gctaattgag
acagatttat
406261 tgcgttgcag gactgctggt gtcacacatg taagcacagc caattcatta
ccactgcggg
406321 gacgttattt tacacgaggc ctcatctttt cctcattttt cctcattttt
ttaatccagg
406381 ctggtatggt ggatcatctg cactctgcag cagatggaag gcaaaaatct
gtgtcctgtg
406441 caacatcgct ggaaaaactc ctgtaaataa ctgagttagc cagttagcct
ggtttttgca
406501 cgccagccgc cttcaaactc tcatccaagc gcatctgtcc ttgcagcatg
tcggcaaaac
406561 tctccaatcc ctttgagtga caaaagatct ttgattaata caaaccgttg
tcttcttttc
406621 cttcttctga cttgcttttc ttccactcaa ggtcgaaggt tgtccccttg
gaaaggcgca
406681 ttattaaagg cagctcggct cttgctgaaa tgatgaacct gtacacccaa
actccattca

406741 ttaaaagcat attttatagg ttcattatac atgtgttctg ccccactggc ttgccagcgc
406801 ctcagcagtg tccttcgctc ctgtgccgag ccggcgcgtt caccttacag gagaggaaga
406861 atcattgcac gtcaagaaaa taatcatgtg ttctgtgatg cacctgaagg gaagggaaca
406921 gcatcagttg cataaatcca ccattgtgtg caatcaccgc atctaatttc taccgggctg
406981 aatgtgttta ttcaccaggg tgtattcaaa tgcatgccgt cctgctgata tggattcaaa
407041 tatccaatat gttacagagg agctttgcag attttatgca ttagagcagg gatttacagc
407101 atctcccgtc atacgtgcgt gtatggctgc gtggccgaat ctccttgata tacctgcagc
407161 aaaagagatt tgtgtgattt gaggtttaaa ctggccttgg atgctcacag gctgttattt
407221 cgaggtaaca atgcgtgtgt gtgtgtgggg ggggggttat tttgtagttt agttctattc
407281 aaatcggtct ttttagttgc aacagtgagt tcgttatgac cttgaaacag tgaccttgcc
407341 gcccgtgaca cagcaggcca catcatttta ttgtggtttt tctttcgtat aaaggaagag
407401 ggagaatgcc cgattaaaga tgcccgattg tgcttgtgtg gtatcagatc gcctgattaa
407461 tctttactac ttgggtaatc tgtggacgtc tgctgaatgc tttcccctaa cctacagtag
407521 tgaaagcatt agaatcataa atacactgca tcgactgact gcccgcttat tttacacgca
407581 tgaaactgta catgtgagag gctgaaggga ctctgcagga tacatgtgtg atgacacggg
407641 agtttccccg cgtgtgtgtg gaaacattgc ccacagtcac tcctggccgt gacagcctct
407701 gactgctcac atcctccttc cctgcaagat ggcagacatt ggctgtcgct tgataaagct
407761 gacataaatc tgacatttct gaccaccatt gcgacttaaa gctgcattga aactcgcacc
407821 caagttaagg aaatacttct ttattgcact gcatctgcga atggaacatc atgcctttat
407881 ataacacaat gctgacaaat ccacagcatt ttatagctct cctttatagc tggcttcagc
407941 tcctttaacc attagatggc aaacatgaaa ataacatcct gtagataaaa actgtagcag
408001 cgcaggttag atgttcagca atattctggg ggtgggggt ggtttcaatt ctaaaattga
408061 catttccgtt ttgcgcaaag actgtaaatg aaatgatagt tgatagattt gtactcgtcg
408121 cacatgaact gaggctccct tttgccttat ctttattctg atatgcgtta tcagtatgct
408181 gcttctgcat taatggcttt tcgtcgcata gaaacacaga gcatcgacgt atgatccaga
408241 aatgcttaca agatacgaac accacagcaa taaattcacc tattgacgat ggagcatatt
408301 tacccacgag gggcgtttta tgtaaccaga ttaagggatt tgcgccatgc gtaaaagttg

408361 ggatttcgct cgaaattgaa ggtttgtgct gaacaaaggc tttctttttc
tggatgaaag
408421 caagctctgg gcggtgggaa caagtgcagc agttcgttta tttgacattt
attgaccaca
408481 tgctttttct tcgggtgttg atgccgctac ctgtctatgc gcatggtagg
gcgtcgttag
408541 gtctcctata tatatatata tatttttta tttttttttt taaatacaat
tcaaaacttt
408601 tatgccaaag aataaatatg ataataaatt acatctgatt cgttcttgac
ctgcagttaa
408661 agcgcacaag tttatggaag gctttcttga agtcctcgtt tgacattgtg
tagatgatcg
408721 ggttgatgag cgagttcagg tatcccagcc acgtgaaaaa gtcgaataac
tcgggaatga
408781 aacacgtctc gcacgtcgcg accaccaggg tgtaaatgaa aaacggtagc
cagcaaacga
408841 tgtaagcacc gaggataatt cccaaagttt tcgtggcttt tctctctctg
gctgccgaaa
408901 tgcgcttttt ctccaaaagc gcgtccgaca ccgtcacttt cacttggttc
tcactggccg
408961 aagagccggt gtcgctggac agagtgtcgt gcctcccgca ctgcagagag
ttagtggacg
409021 cgacggatcc cggggagttg gtgaccaggt gcgcggaggt gagccgcttc
cccacccttt
409081 tgggggactg cttcaggatt ctttttcgag cttcgacgta aatccgtcca
tagaggacaa
409141 taaggagcag ggtggggatg tagaaagctc caaaagttga gtatatggta
tagaaaatat
409201 ggtccgtatt gacgctgcag ctggtcagtt cctccgcctt tacctgcctc
cagaagagag
409261 ggggcaggga gatggagatg gcgatgaccc aggcgatggc caccatcccg
gccgcacgtc
409321 ctggggtgcg cttcttcgag tactccaccg cgtccgtgat ggcccaatac
ctatccaagg
409381 cgattacgca caggtggagg atggacgccg tgcaacacgt tatgtctgag
gagagccaga
409441 tgtcgcacac gatctgtccc agcgtccagg tgtggatgac cgtgtagagg
acgcagatgg
409501 gcatcaccag gatggacacc agcaggtcag tcacggccag ggaagcgatc
agaaagttcg
409561 cgggtgtttg cagcttcttg gagtgggaga tggttgctat gacgaaggcg
ttggacagag
409621 tggtggccag agtgatgagg gacaggatga cggccagact aatttgatag
gcgacgctct
409681 ccgcgctgat ggccgctgtg gatgaatctg cgaggctgag aatgctgtcg
tttgaagtgt
409741 tgacaggctg ggtcggttcg atttgacccg agcgctccat gactctcagt
cacttggtgt
409801 catatattat gtcgcattta tgttgtcagc tgtcccggtg aacgtcacct
tccatagaat
409861 gaacagcaga ccattgtaaa cgggcctttt cttcttcatt ttcgatgggg
atccaaatat
409921 ccacctcgct caacttttct cattcgtaat taagtctgtg cgtaaaaaag
actacgagtg

409981 gagcgtcctg gtgcagtagt ccaacatctg gtaatgggat cggggataat ccgcatgcgt
410041 ggcagaagtt ctccagaact gttcagtctc atcgggcccc ctgtggtcga atgccaaaac
410101 caacccaaac tctcagtttt atacagactc tgacatcgaa taagcgccac agatcacagc
410161 cttttttttt tcctcgaaga gaagactatc tgctgggtca tagtgcccag tgtttcctcc
410221 ccgctccctt atctggtatt taattagctc tctgaaatga cttttatact gtttcctatg
410281 cgaataaaaa taatgagttt atttataggt gcccttggga aacactcaag gacacgaaat
410341 ggacataaaa aagaaaaaag ggaacatcta acagaagagc agaacaatac aaaacagtaa
410401 aagctacaag aaatataaat aaggctttaa aatggtgtta aaagtggcaa aaatggtggc
410461 taggctagag gtggggacag tgaggcactg atgtagagga ggtcagacag gtaaggaggt
410521 tgtggatgga ctcgaatgaa aatcagagga tttcgggact atacagagat taactgggag
410581 gtggtaaagc tgctgcagga cggggttgat ggggaacaga gggagcttgg gaagctctcg
410641 gatggacgag acaaaagaag aagggagttg caataatcag gacaagaggt gacgaggccg
410701 cggtatcaag gtgagggagg gctggaggcc attgatgttg aaggggtgga aacgtgttta
410761 ggggggttat tgatgggaat gaaagtggac ttgtcggggg gctgtgagcc ggtgaagagg
410821 gctcagagaa ccgtcggttt cagagagggt tggtgctgag gggagtcggg tttgctggag
410881 aggtgagctg ggtgtttgcc ctgcagcagt gaggtgtaga tctttacttc atgtgtcctc
410941 tcgtttcacc aaacagcacc ctccatctaa tagcttgata aagcttctca tattcaaaaa
411001 ccaactcatg gtttcagttt gaccagtgga ggcggagctt gtggtctgtt actgcagtca
411061 accaactgag atgctttcca gttttatccc gggaaggaaa cgtaaaaact cataaggctt
411121 ttaaaggcga ttaaaaaaat aaatccactg ccatgctttc acagaagatc caacactggc
411181 gagggtctaa tgacagactt taatatgtaa catcctccat cttgaaatat caacaataat
411241 ttcattgtaa acgatttctg gagctacttc cgacattcta gaagcgcttc acctgatgaa
411301 gagcaacttg gcagcagctg aaatcagccg ccaccgttgt taacactggg tcagcgccag
411361 cagagctgta aaactgattc attgttttag ctcccaagtt tagccaggct gcactgggcc
411421 cattgaaatg ctttaatatt aaatattaat cttcagcctt gtgaatcagg atccaaacca
411481 cttgtcagga agtagatcta gtgttggtgt gtgctccttg ttcttgacac atcatttata
411541 atttaactcc agctcggcct tggtgttctg tggatcaggt taaaagatgg tttaatcgct

411601 ttagaagttt cagctgcagc cctcaacatg aaggacactc ctctgtagct ctgaaaaacc
411661 agtcaaacgc aaagttgtta ccaccaaaaa ccataaatat gaagaatacg gagattattc
411721 atccgagact ttgtttgaag ttgctcactg gtgacagaac caacctttgg cggtcccaaa
411781 agtcggtaag tgtttgatgt ggtccgaggt cggcatcacg aatcctctga tcctctgccc
411841 tcgggcgacc agacgaacgt tgccgccgtc ccaccgggag cacattgatt gtgtttggta
411901 agtgctcatg tcatgcgcat taagctgaat ttacaagaaa cggtaaaagc ttttcacatc
411961 gaggcgcggg cgcggtgcct gcacggtgct caggtaaata tttgaaaaag tgcatttata
412021 ccagcagtgc tttatgtctt ccgtcgcggt gcactttgtt ctgagtgaac tggatttccg
412081 ttgcagtgga acctccagat tgatgaaacg tcacttaacg ttgctttaaa ggttcctttc
412141 ctttcaggct atgacaaacg caggaattcc ttccttcaga gagtcggttt tcagtcaccg
412201 cagtaacctc tgatgccatt cttggtcaag gcccggtgct tttgggtgtc cactcaaaat
412261 aaatgctgct ctggctgagc tgcagctgaa aatgaagcgg tttacaagcc agaagcaaag
412321 ggctttgtga gcgacccagt tcagcagcag cagcatccgg ggcagcttct ccgttatctc
412381 ccctcatcac gcagcacagc gcctcagccg cctacatcca atttcatact gctgctaaaa
412441 aaactatttt tgggtgggta ataaggtatc gagctaggtt aagtcaacac gctgcaacac
412501 gtccaatcac aggccgatgt ggatccttaa tcgtttttc ttttctgttt gctgaacgtg
412561 aatgataaaa taaacaggaa gtaaaatatt cagagaggtt ttgatgaaag tcatctttga
412621 tgaaacgata aaaactgata atagtgaata aaaaggctgg ctccagagct caaccagatc
412681 ccaccctaaa cctatagatg ttaacgctaa aaagacacaa agcaatcctt tagcggcttt
412741 ttattctgac gctggccacg ggtcgagttc cattacagat gaatttttat tttctctgtt
412801 taattggctc cgtgctgaag tggcttgaga ggctttttgt taccgctgtg aagtgcgagc
412861 caaacaggaa aggtgaaatt acctcgctgg gaaattagga ggtggtttaa ttacacttca
412921 tcaagaacac ttttaacact tttactttt atttcagggt gacctttcag ttattgtggc
412981 cgtttatttc tgtccttgtt caatgacagc tgtagcgaac caaatagggc aactgtaaat
413041 cagggcagat cccttgttgt tatgagatta gaccctccct gtccacctac tcaaaactcc
413101 ttatcttatc tgagcgttta tggcccgagc tgagaggcca tgtttgtaac ccaaatccta
413161 aaagtatctc aggcatcgca gccgcgtcgc tgctgggatt ttaatcagga gagtccacgt

413221 tacagcctgt tgcctgatgg gtatgtttaa aaatgtgcat ttaactagaa atgttaagat
413281 gtcttcagtg gtctaaggtc tagtttttaa taatcctgtc ataaaattaa atatacaaca
413341 cttagatagg attttatagc ccaataaatt taatcaccaa taaatattaa ctagaacgtt
413401 acatctgtgg agggccagaa cagaaaacgc atgtaaatct gtggtttgtt tgtccatctg
413461 tggcccataa agatggtaaa atattaaatg agtacacatc atcatcactg aagtttgcct
413521 ttgcaccact atcaaaatat tattggaaga taaaatgatc attggaattt caagcccaaa
413581 taaaaccttt tttgtatcca gattagattt tgtactgtta gctccatagt aataaactca
413641 aatttctgag gctgtttgaa ggtcagaaag gtcgttgtga cccctgctac atctatgagt
413701 aatttcattg cactgtgaaa ctaacacaag acatcacaca aattgagcat ggctctgatg
413761 gagatctgag aggagtctgg gaaaagaaac tattcaccaa atgccaaaat tcaggagtca
413821 ctgagacgtt gaggtagaaa tttagaaaaa caaaaccgtt tgaggaaaat aacgcacagc
413881 cacacacaca agaaccatga aacccaacta gtggtgtcga ccatctggtg tcaatcaccc
413941 aactgactca gaggaatgag tccctataga ttagcagctg attagctaca gttctgccct
414001 gcgggtgcag ctgatcaaca acctccagac ccagacaggg ttaatagatg cccctggctc
414061 gtgttgatgg taaagtcatt gttgactgga catcaataag gttggaattc tgtggagtta
414121 agaaaaagaa aagagcccct tgccccttttg cccttttctc cggctctcag agggcattat
414181 gggtaatgtg ggcactctgg gtttgctcgc acgaccaata aatgacttgt tacattgaaa
414241 tagagcaact ctaacaatgt gtcagtgcac aaaggaacac tcaaatttag gacaccttaa
414301 attgcaggac ccctccccac tgaagttgtc ccagtctgcc cagtctgata acatatggtc
414361 tcttctgctg cgccagtcag ctctctgagg cttacagctg gttctttctg ctctagtgat
414421 gatttggggg ggggggcgt catggagaga agttattgca ggacttggac cctcacacag
414481 aacatgcacg tgtgtgtggg ctcgcgcatg tgtgtgtgtg tgtcttccat gttgggaaag
414541 aggagaatga attcagagcg aagcgcagaa tcgtatttgg agcagaaaaa gaaaaaaaac
414601 acgggtttga gaaaacacgc ttagtggtca gtaaaaagtg ctgactggtc ctttttttggc
414661 accagttgga gtctccagtt ccactgagtg acaccagggt cagaagctga ctgcaggcag
414721 cagcgaggct tatcagcact tctgagaact cccagtattt agagtggaat actgcaaata
414781 tccggtacta ctgtatctca cactgacata tggtttatat atttactgta gcagaatcat

414841 cacaggcctg agagattatt ataaagattg gggtggacca cccaataggt gcgggaatct
414901 tgtgccacaa atacagtagc tcacaatgga caaaataaga taggagattt tgtcttctgt
414961 tgtgaatctt ctcctggtgg tagccgagat gtgttgatga ggaaggaaac ttcgcagaca
415021 ccgacttgat tatcaaagat gtttattgga gagaacagtc aggcatcaat cgaacactgc
415081 agtttgtccg agggaggtct cgaggtcccg atggtgaaga actccggggt gtctacctcg
415141 atacctcctt cttatagagt ccagtaaaca cattcttttc tgatgacctc acgctatacc
415201 gtatggtcaa cccaatggta tggtgttcag ttatgaccaa aaaagggaat agtgaagatg
415261 cttcatgcta tacatcctcc tagtgagaac caccaggggc ccacaggctc cagtgttgtc
415321 ttttaacagc tcctattacc taaaggaaga agacaccgga cacacatgtg gaggatgtcc
415381 taatatggtg cttaggttgg ctgttaaaca cattaaggcc ttacagacgt aaagcctgca
415441 tagaacgggt cagatactac acttcattca ttaagactta actattaatg cttgctacat
415501 attgcatagt ttatcaactc aactttattg atacagcatc tgttggaggg gttctagtag
415561 aggaagagaa gagcgatgcc tactgcagga tactatcgac gtataagtaa attctataaa
415621 acacgggtct caacccagtc cagctgggtt ttatgtcccg cccaggttta tatgaaacta
415681 ggatcccagg tgaaaggagg ttcctgcctg gttgggctaa aaaccccggt ggattgtggc
415741 ccgtactggg ttgaagacca caactgtgaa acataaacgg ctgcacttcc tgacatctgg
415801 attttggttt ctttttacag ctcacagtta attatgcagt gtgaagtgtg aggcgagact
415861 tgagcctgga ggtgtgaagc gtgggcattt ctgggttgaa tggtggatgt gtgaagattg
415921 agagtgagga ggtgcctctg atcttcagtg ggcaaatgtg cgaacacaca cacacacaca
415981 atcacctaca ggacacacct acatcactgc tggtggtgca aaagccttct cacgtcaccg
416041 gaaggagcat ccccgacgcc cctgtcaaca ccaggttgtg gatcaatgat gagaaacatc
416101 attagttgtg taatgaaaac cttaaatccg cagctgctgt ttcagggagg ccatttcttt
416161 gatgaaaggt gatgttatga cagcaagggt ctgcagagta aaacccttcg cgcccccca
416221 gcggggcact aaacaggagg aggcagagga caaggagatc agtacaacac catctgtcta
416281 agtgtgtcaa cctccgtgtc agactgcacc ccgcgcaccc attagcagcc tccccggctc
416341 taaagataaa cgctgccagc taataacaat ctactgttgg ttctctggat ggggacacca
416401 aacagcgttt tacagcttca taagagagac cgagaagttc agaaagttgt gaagaaacta

416461 gacttagatt taaatatggg gacacctttt tctacaaatg ccagctttta
tattaggttt
416521 cttttcctgt tccagatcac gtgaggaaat attacaaagg tttggcctga
aaataacccc
416581 tgctgccttt aaatctaaac gtgttcttta tatagttgat ctttagcacc
aggtttatat
416641 aactcatgtc tcgttttttt cccctacatt tgtaccagta gtttaaaatg
attctgtcca
416701 cagatgtaac agtgaagaat tcacctgaaa ttagtgacat attcacagta
acgagtcgcc
416761 atataaatga atttattata gcatttagag gaaggactgg aaagagactt
ggagtcatta
416821 ttgcaaaggt tcatgcgtat aatgtgtctt cgagatgtta taaataaata
aacaaaccca
416881 ctatgtataa aagaactacc atttaagtgt tcttgtgctt ttgagtgcag
gtattccagt
416941 gatttatgtt tttaatatct gtatgcgagg atggtgatga agctaaagaa
gaatcagcta
417001 tttggggtat tttttgaggc agaaaacccc cccaaaacgc atcacgaaag
cactgctaag
417061 catcattcat ttcagtgcca actccccagg gcaaccgcaa caccgccatt
gatccgtttg
417121 ggttctgtgt attatttggt catttttcag ttgcgtgtgt caatctgccc
atctctgtgg
417181 tctctttacc tccctctctc cctctctgga cttccaaaca ggtcagatat
ttttagctgc
417241 atctccttta aagtttaaac cgaaaaataa ataaataaag ccacccccgt
gaaactttaa
417301 aataaggggt gaaaggtttt atttctgctg catttgttgc agcagtacag
ctcaggacaa
417361 acacgtcccc ttggggagta acggcatctt tcggctgccg cagcttttga
atgacttgct
417421 ttatcgtgtc accgctgaga ggtgctgcag ggagaggaac cagatcagga
gagagcagcg
417481 cttcatcgcg gaccgtccaa tgcatctctc tggttagacc tttagcttcc
acttttataa
417541 gttaagaatg atatatctcc aaagttaaac cctttactgc gatcaacatt
ccattttggg
417601 cgataatgac tcgcgtattg aaataataat cccatttaat gacagtttta
ttcatatggg
417661 agaacccatt ccttgtattt gatattagtg tcccagtatg tgattatttt
attagtgttg
417721 ctgttaaagg caacaacaag aatatgattc taatggcgtg aacgcgctga
gctccttctg
417781 gctccaaacg ttgtttcccg ggacggtttt tatgccagtg taaagttgct
aacataaaaa
417841 cttccatgga atcttgattc gaccccagtg tatagcgccg cgcatcaccg
gagcaaaagc
417901 ccacgcctcc ttcgtctccg cacagtcctc gggacaccag tgcgttttta
cgcgcagcct
417961 cgagatgagc gtgtggtcgc gccggtgcgc tctctgggtc tgcatctctc
tactggcggg
418021 gccagtttgg accttcgagc tgacgctgct tcacaccaac gacaaccatg
cgcggatcga

418081 ggagaccagc gaggacttgg gaaagtgctc atccaggggt ccctgtttcg cgggggtcgc
418141 ccggaggttc accaaagtgt cggagatccg caaaaaggaa aagaacgttt tgttcctgga
418201 cgctggggac caatttcagg ggacggtttg gtttaactac tacaaaggcg cagaagccgc
418261 gcactttatg aataaacttg gttataatgc gatggtaaac gactatttaa gtgtctttgt
418321 gtgtcttttt aagggttatt aggcgcttct cagtttggaa tctgtctgat ttgattcttc
418381 tgtgtttaag gctttaggga accatgagtt tgataatggg gtggagggac tcctgccttt
418441 cctccagaat gtgaactgtt ctgtggtgag tgcgaacatc caacctgacc agagtctggc
418501 tgcaaagctc agcggtttcc tccagcccta cacggtcctc aacgtgggct cagagaaggt
418561 ggccgtggtg ggctacacca ccgccgagac cccccttccta tctatgccag gtaaaccctt
418621 aattcccctt gaggcagagg tggattttat ttctactttt ttcgtgtttc aggcccacat
418681 cttaaattta acgaggaagt ggaggcgctc caggtccagg tggacaaact ggaaaccctg
418741 ggttatgata agatcatcgc cttgggtcac tccggctttg atgtggatca gcagatcgcc
418801 aagcgagtga gaggggttga cgtcgtcatc ggaggacaca ccaacacctt cctttatact
418861 ggtatttgtg taaatagctg aattttagat aagatgaaat gctcggcagc actacacagg
418921 cacatgtgtc tctgcaggaa aagccccgtc cactgaagtg cctgtaggtc cttatccttt
418981 tatagtgagg tctgacgatg ggagagacgt cccggtggtc caagcttttg cctttggaaa
419041 atacctcggt tacttgaggg tcacatttga cgatgctgga aaagtaatca aggctgcagg
419101 aaacccaatc ctactggaca gcagcgtccc tcagggtggg cccatctact ccgctcaatt
419161 catcaacagt gcaatcacga gttcttcttg tgttttcata accctcattc tgactctcag
419221 atccggacgt cctggctgag gtcaacaggt ggaagaaaga cctggctcag tactcctcac
419281 agtatgtcgg gcaaacctta gtttatctca acggcacgtt tgaagagtgc cgatttcggg
419341 agtgtaacct cggcaacctg atctgcgatg gcatggtaac gacaatcaga gttcatcctt
419401 taccgtagtc cgaaaacctt gctcttcaga tggtttagct gggaaaacta gggttagcca
419461 ttagcctgcc aaggtcaggt tcactgtttt ccttgttgca gatcgaccac aacatcaaat
419521 tctccagcga gctgcagtgg aaccacgtga gcctttgtat gctgaacagc ggagccatac
419581 gggctcccat cgatgagcgc tacaagaacg gtaaatgttt gtccatgact gcaagacaag
419641 tccctgcttc tcgtcatcgt ggtatttgcc attatcttta ggctccataa ctatggagga

419701 cgtcctcact gtcttgccgt tcggagggac tgttgacttg gtgcagataa aagggtcaac
419761 ggtgaagaag gcgttcgaac atgccgtcca cagatttgga agcatgtcag gagagtttct
419821 gcaagtctca ggtctgtttt ctggctctct cttcatcctg ccaagttttt ccttttattc
419881 ttgggtcttc cacagtaaca ctctttagct taattgccaa tacatttgca accagttagc
419941 ttagagttac ataaagaaag acttggccga gcctataacc agcattacat tatcatctac
420001 atagaaaaaa ccttggttat tttatttttg gacttttgca ggcttccatg tcaagtacga
420061 cctgtccaaa ccagtgaacc aacgcgtcac gtctctgtcg gcactttgca ctgagtgccg
420121 cgtacccaaa tatgagccca tagacccgga gaggacgtac aaggtgacca tgccgtcata
420181 tctggtggac ggtggtgacg gtttcagtat gatcaaggag gagttactga agcataacac
420241 aggtgagcgg tggtgtttaa caaattctaa tctaatggtc tgaaataact ccagagttaa
420301 tgctcattgt catgacgata gaatctggag caacagccag gagcaaatca atcagtttta
420361 cttattttga aatttcctat gaagtcagag ctggccctca ttcacattac agcacagcag
420421 aataacatca cattcaatta tttatttatc tgagcatttt tagggacttt taacattatt
420481 aaatataaaa atatagaaaa aaatgatcac ttataatttg atttttgctc tattgcctgc
420541 agttttcata aatggagcca cgcctcaaca taaaaaccac acaaacattt cacctccaga
420601 ttcactccag ctgcaaatgt cacataagca tgtgaggatt agggcatatt catagagaaa
420661 ctcaccctg accccaacac atctgttaac agaaaggatg tgtcacccag acagagcttt
420721 ttatctggat caggttcaag ggtcatcaaa aaaaaaagaa agaaaacaat tgaaaagtca
420781 gcctttggaa aagagagaac caacaataat gaataaggaa ggaaggaaga gaagaagcag
420841 cggagaaaaa ctcagactgg gggttgttta tttaggatga atgccacgtt tcagatgctg
420901 gtcctgtaat ttgctctcta aactcaagct ccatcagtgc agccatctct ggtcaataac
420961 cgcgcagtta tgtcagcctg tgctgctccg aaaagctgtc agcatgaccc accacaaaaa
421021 ataaccatgt gggagttgcc tgtgcaagcc agttttagat tgtagttcct gtagatggga
421081 agtaaaaacc aggatggtta attgctctaa gttgtgtctt taatgaaacg gacgcacttt
421141 ggagaagtaa atcgcgggag attttatcga gagcagaaaa gagatgtttg aaggacaata
421201 gggtcattcc aggatcgatt tgggaatttc aaacagtgtt ttgtttgaaa tcagtgaaga
421261 tggtttaaat cttggcattt acaccctgga agtacagatt tgcctaaaat aataattaat

421321 gacactgact tcaagagggc cgaggtcaaa aagacacaaa cacaagggga
tatcagccta
421381 aagtgatgcc aagtctttat agaacagcat cacgttcagc agcgtatcct
ggaaaacgcc
421441 aagattctta cgctctgact cctctctttc aggtgacttg gacatttctg
tgttctctaa
421501 atatatctcg cagcagaagc gcgtatatcc agcagtggag ggccggatca
cagtcagggg
421561 ctcggcctcc tctgcagccc acagcctcgg cgtgctgctg ctgctgctgc
cgctcttatg
421621 tccgtctctg atcctctaaa gctcgtccaa gactgataaa actgaaacct
cctccagaac
421681 atggacagat gcccacatac cttcagaact agagggcagg ctggtgttag
agacactttc
421741 taaagctcaa attgaatttc catatatatg tgtgtgtgtg tatatatatt
aggaaggttt
421801 gcactgaatt accacaaaca cactgcaaat attttataca tatatttgtt
acagaatgaa
421861 tccagttgcc aatcatttat gtacacattt taaaaagtgg ttggcacaaa
acaagagaag
421921 cacaggtgtt agctgagcag gctttaatca cagagcaacg cttcaggctg
ctgtaatggg
421981 ctgtttcaca tccacggagc cacggcagcg gcagcctcct tctgcccctg
aagaagaaca
422041 acaaaagcct tatgcgctct aacaatcatc cccctggttt ccctcgaatg
aatccttgag
422101 ctatttaagt tcataaggtt cggaacgata agctaaaaat aactacacag
gaacgtcggg
422161 tgaagggtgg aagaagcagg cgtaaagatg cacaatccct gtccatttcc
taccattgtt
422221 acgcaaaaaa atagggctaa aataaaggat tagatcgatg gaaacgcaga
gggcatcagt
422281 gttaccctgt tcagctccgg gaaaagctct tgaatggcga tgtccaacag
cacatacgtc
422341 aactgcgtga gaggagggag aacaaaaaag caaagtgctg tttagtatct
ttgtcctccc
422401 tttcccagca gggggtcaga ggtgaacaaa cattcgcgag tttgtccttg
taccttcgta
422461 cctgtttgtt cagaatcggc tgctgcaatc catcaaagag gagacgtatt
ccttcatact
422521 tggcctcttc tcccatacat ttccccagga agtctgaaag ataaatggct
atagaaatct
422581 cccgctgacg tgcgctgcgc ggcttgattc tcgggtggga ggcggacagt
aatctggggt
422641 aggaacagca gacttgtata cctggaatgt aagacatcat ctcctcaaac
gtcctcttcg
422701 ccctcatctg cttctcttcg agcgaacgcg gcggactgct ctcgcagaag
acgttgtctg
422761 ggagcataaa gatgcagagc gactggatcg tttccgtggc catttcagag
gcagcgttct
422821 ttatttccta acgcttttct ttttacctct gagcagggtg atgagggaca
ccagccggtg
422881 ctcctgcacc acctggttga gtttgtagtg caggcagtgg tctgtgtagg
cttccagggt

422941 gtttttgaac aggatcctgc ccgtcatcaa caggtgtgca gccagtcagg gatgtgaaga
423001 cgactctggc tgcagacagc gagaggggga aagagtcaat atatatgtaa tggatctgga
423061 gaagtgatca cgatgatggg gatccattgg gggaaaaaaa cagagtcctg gagggagtgg
423121 gaaaaataca ggaaacgagc ctgaggacat gaagagcttc agtgatttca taaaaacgta
423181 tgcacgctta aagaatgtat attacaagtt cattttacag caccatacaa ctcaaatgcg
423241 cagtaaaaat gtctggagac tttcttattc cctgcctgtt tccaccacgc attcaaggtc
423301 tctgccagca gtggaacgat gggattaaaa gatactccct gtttaaatgg gttaggacaa
423361 acaagagtat gaattcagtg aggaagttct tctgtgttaa aaaaataaat caatcaatct
423421 taaattctga ataaatgtca gactgattct tgactgcagt ggttttagca tcacaggaca
423481 ctgaataaga aagagaacat ctaccggtgg tgagtagaat agtgcataag cagtgggcgg
423541 gacttaccca catacatcaa gtagtcgtac accccgtcca cagtggtcat ttccatgaag
423601 taattctggt tgtgtctctt ctcggccatt tctgaccggt tggcgttgtt tttgaagagc
423661 tcattgaaga gctgggagac aaggacggag caccgtgtca ttttcttctc atatttgatg
423721 gtttaaaaac acactcgggt tattgcgaat gagtggagtt tatgtctcgt gcagagagtt
423781 taaatggttt ttgtaaatgc aggacagaaa gtcgaccccc ctgtgtggaa cagatgtcct
423841 gaagcccaga aggtgactcc ttttaaagtc cttttacttc ccagaaaggt tccggccaga
423901 tgagatctgt gtgcgaagaa ccatgccaca aacctcgttt tacacctaag ttttactggc
423961 aacaggatgt cctgttacac aacgcgagcc tgatgagtcc cgcctgggtg aggacttact
424021 gatttaatta ccgagacctt tgacacggat gggacttatc caggaacaga tagagagcct
424081 agctgctcgt tccatttatc actgtcaggg actgtgggac ggtgattcag gttgagggca
424141 tctaaggtcc ctgcagatgt ttgagtaata cttgaagaca tcagttttaa aagagtgata
424201 aggcagtgag tgcgcacagc aaaaactggt ttatgttctg cgtacgttat gtactgtgta
424261 taattgtgta taatttgctt aatacttttt aattaaaccc cgcataatta gtgttaagta
424321 gaaagtgtcg tgcagacgtc aataatgcaa gttaaaagaa acagaaggct gaataaacct
424381 tgcagatacg tttctgttgc tataggaaac gtaaagtctg tatttaaagt ccgcagtggt
424441 gccgttaggg ggcgctgttc gtgctgccgg aacaaatttg cgaaacggct ttttcgctgt
424501 tccacaattc aaaaatttaa aataaaatgc ggatcatttc actggaactc ttttgaaaga

424561 aaacagtcat cagatcagtc attttcttct ttgacctgga aagcatgact
ggccgccctc
424621 cagcgtaaat aaatgccaaa ctaaattttg agcttctgat gggaagccag
gcagcggagg
424681 aaaagccccg gagagtctga tgaccatgag aaagaacccc taaccttttt
gtcattttcc
424741 gaggtagggc tgaggatggt gagctcaggg cggctgggct tgggtttggg
ggattcgcag
424801 gagttgaaga acgactggat gaaaggctcc agatgttgcc ctttctgatg
aaggaaaagc
424861 atcacttgga aacgctaata aaacattgca ctgttgctaa gacaccaccc
acaaggccct
424921 gcatgcacac acaccccagc agtgcatgac tcacctcttt tatcagtttg
ctgggcaccg
424981 atttaatgat tttccctgca aaacataaat attttatctc attgatttat
tgcttcccaa
425041 caactcacca tcaccatctt ttcactcaaa cccaaggtcg ggctgcgcaa
acatctctgc
425101 accccgctgg agaggtttgg ccgcccccga cccaatccat cacatcaatc
cgacacactc
425161 ttactgtacc caggttcacg tcaggcagca tcctgtccag gaactgagac
tcggtgctgt
425221 tgggggacag aaagtcggcc agcagctggc tgttgctcag ctccgggtgc
tgcagaagtt
425281 tctggagagg aaatgcattt cagcatgtct tgactcaaac aagtggcctt
gaacaactcc
425341 cgagctgccc tttgttttcc atgttccgcc tcataattac caaatggata
gaatttgtca
425401 gagtcttttg ggacccctcc tggacaacaa aattccttta acaattggca
attttatgga
425461 agcggtccct ccgtcctctg agaataaatg cgccacattc gagactgtac
caaggagccc
425521 tacctgaaga tactcctgga actcctcgcg cttcgacgtc aggaactcgt
aattcttggg
425581 gccgatgatt ctctttgatg gcagctgcgt gtctggaaac gagcctgcag
agggtgatag
425641 gatgagggaa aataattcac tggcccctac acacaccccc accacacaca
cacattgtgt
425701 gactcctata taagccataa aaacggcatg acgtatggag gtaatgttct
gctggtgagt
425761 ccggatgttg ctcccttctg tgtgaactgg gattttaaaa acggaaattt
aaaaaactgc
425821 tgacgtagag aacttaccat gaaactctgt cagctttgac tcgaggacat
agaactccag
425881 gtacctcctg tagaccgacc agtgctcggt ctcatgcccc actgcagaca
cacaaaagca
425941 atagttcgtc tcaaattaac ggcatcagtg agacgtattt gcagcccgtt
ttcactttac
426001 gtaagagaca tgttgaaacg ctgcagcact ccagcagagt ttactccttt
attcagattt
426061 atatgaattt gaatcctccc caaagtatct gcttagctcc agaatatcac
cagggacaca
426121 tttgtgagtt ctcacttgct ggcattaaac cagtcccatc tcctgaggtt
ctttttccat

426181 tactgaaata attgcgcgtt ttcagaaaga agcagtaaac tgtttggtat
cggtaactca
426241 tggagcaaaa caagggagag gaaatatgta ttctgttgct gggaggagcg
tttggagaaa
426301 cggaaaagac gggaaaagtt cttccagttg gacaggactc taattatacg
tggagtcact
426361 tcttttgatg tctggggtca gtttttcta acctaaatta gcttagaatg
aacacagctg
426421 agaaggaaac agacgacgac gcgtattcag gttcatttca acaactctgg
cacgtttaag
426481 cttttccatc tgtttctcgt ctgtccaaat attcccagag atgtctgcca
atcattacca
426541 ctcctacagt gtcgtgcatg taacttcttg cccatattat gtaaaaccat
caggcagcga
426601 agcagaagaa ccgcttcctg cctctgatgg cagcaggtcg ggtccttcac
ctcacctgct
426661 ttccggtcgt tgcgctctac atcgatgcag aacacgggga tcctctcccg
cttcacgtcg
426721 tcgtcgaaga aatcgatgta tgggatggtg atgttccagg ccaacaggtt
tcgtggagtg
426781 gtggggctgg atgctgcctc catgggggaa tcatcctcca gcaccatcat
ggcttcctcc
426841 accttgaata gcaggcgaga cagcttacat ttgagtggag ttagtttttt
tcccccaaag
426901 ttaaagcgtt cttattcaca acagtttaaa ataaaagtcc taaactctgc
tgaacaccat
426961 ggatgatgag catgtacaag tattcttttc agacaaaaat agctcaaaaa
atacactttt
427021 tccccttaag aataataaat actgacttga gaaccacata tgcttctgga
tatattccca
427081 gtgttgtctg actgtacgga acatctgagt tcaacattct gggaaccaat
ggaaggtttt
427141 attcctctgt gtctggagac gctcatcgag ggtcactctg cgcggcaacg
cccccatgtg
427201 gtgactaaga ggagtgcttc cgatttcaac aggttgcgcg acagtccctc
tcaccaagtc
427261 atcctctccc tccaccaggc ggaggacggc agcatcgcac cctccatggt
cgtgctcttg
427321 aacactcctt tgattttgct gccgatgcgg ctgatcccga aggactcgcc
cctcttcgac
427381 gtgttcctgc gggcaaagag ccggacaatg acatggatgc gcgacggaac
atataagact
427441 tctaaaggaa cgtgacaaac aggaaccgcg ggggaatgag caggaggggg
gctgaggaaa
427501 taaccaagca tctgtatgaa aactgctgga ggagaagcaa aaagcagaca
cactgtcaca
427561 tcacagccat ctcacactga aatgcctggg ttcaacactg aggtcaaacg
aagcgatggt
427621 cttcggcgta agaaagaatg tgccgtgaag tactagtgta cgtgacgtca
caatgttttt
427681 ttactttgga ggaaaatatt agactgagcg cgagggatat cagctaatac
gttttgatat
427741 tgttccggta caaaagcaac aaaattccag aatagagtga tgtcattact
ggtgcaccat

427801 caccggtgca tggcaacatg aaataaatca atgactgacg gtaatgttgc tgagccccaa
427861 attaaacaca ccagtgaatg caacagatgg ggctttagca ctcacgacat gagggacaga
427921 aaaaagggat ccatgcatca gggcccatca tgcaggtgtc tgggctggac tggggggtag
427981 ggaatacagc atgacatcac actgggtgag cggtgacacg tgaatggacc ctgacttact
428041 tgggtgatga gtggcggtgg gatagtgagc cgtatgggaa agttgtgagc gtgggctggg
428101 catggaggga gctaaaagat gcaggtgaag agctgccaca ggcggtgaac agcgaggatg
428161 gggactcggg gctccagtcg caggaactaa aggcagaatg gggtcacat ggcccccca
428221 caggcaagag gaggaagcga accgtggatc ataccaggac aggcagtatg gtggtcattg
428281 aatcagacaa acacagcacg gtggaggcgt gacagagatg aagggaaggg tggagacacg
428341 ggcagagtgg gaagggaagt tactggtttg actaaatgga aggagctgag gctcgctgtt
428401 ggtcacttgt cacaaaatgg actgtcagta gaagcgcgga tatccgtgtt gctcgacctc
428461 aacctcaaaa cgatttcttc taagaagttt cccctcatgc ctaaccctca gaaacgatac
428521 gtggaaaaac ttgcacaacg aaaagttgct ttgttccttt gattaacatt tgattaatga
428581 agacatgcgc ttcaatttta aaattggtta tcgttgcttg tctaatcctc atttgcatct
428641 gcggcgccag cagattaaaa ccccaactgg agctgaatta gcatccgaat gctacgccat
428701 cgtcacatct tcagctagcg cttatggaga caaagccctg attgcggggg ctgtgagcgt
428761 tttaaaaaag gcagcgtcac gttccctcat aagaccagtc agcgaaggat ggcgtttgaa
428821 tcggcgtgca gtggcactgc tcagtcaggc accggttgga tcagtacact gatgccatat
428881 actcaccgaa tgtcatccag actgaggcta tttctggaac aatatgcaca aggagttagt
428941 ggcagggccc cgtggccaca gacgcaccac accccgtgac agaaaacatc acagttcggt
429001 cctaaccaaa ctttttttag gccgggtttc atgaaacaaa tgctagtctt aatgctaatg
429061 tagatcgttc tcaactccat taagacaaac atgaacttta tatcatgaaa aacattaaaa
429121 aacattgtta cattatcaaa ttttggcccc agcattcaat attaatggat caatgcagct
429181 tttatttggc acagaaccta cctgctcatc ctggagctcc tggcggggga ctctgcccct
429241 cggagaagct ggcggaagta ctgtaaaaac acagaataaa tgaatgcaaa cgcagcagga
429301 acagaaacca gaggcaggca cgtacagaag acagacggac ggcgctgcat gggtcttttt
429361 gtcttaaatc actcactaga tcacatcacg gctcacatct aagagaggca aactgacctc

429421 gtcgctgtga caaaacatgg gcgcgaaaac attctccagg agggagagga
cgtgctcgta
429481 ggcctcgaac aaacacctca tggtctgcag cctcaccacc tgtggatacg
gcccctccgc
429541 aactgcaaag tcaagcaaga atcagcctct ttaccagcaa gcacacgcag
caaaggcaga
429601 gaaggcgcct gccgcgtact gttgcggatt tcctccacta tgaaaggatc
gaagcggatc
429661 ttgtcgatgc tctcctccaa gcagtaggtc tcatagatct tcctcagttc
ttcgtggagc
429721 atcatcttct gggagtcgga cagctccggg cacaggatcc gatcgttgaa
ctcctctgag
429781 aagacaaagg tcacacgggt cgcccgtttc tttattgata attaacagct
tctccatcat
429841 gagttcacac tctacattta caggacactt gaaaaaagga acagttgctt
gaaatcagtt
429901 tattttggga tgtatatgat ttaatcggag tgaaactaga caacgcaagt
gtgtctgtcg
429961 cactccccgc gtggccccgg aacaaaaaca tctaagaaag ctataacatt
atccatcttc
430021 ctgcaaatac ccatctgccc cctaacgggt gtcgtttatt aggcgatggc
ataagtcaac
430081 cagaagactt attaacctgc tgagcagacg catgtcgcca cctagtgttg
aggaggggga
430141 tatcttcctg tcagttatgc gattttctct gttgcatgta aactgggaca
aggaacaaat
430201 tccaaagagg cgccaaaatc tagttttaag catagcttga ttaatctgtg
catgtaaacc
430261 taccgagtgg ctgtcgcgta gctgcaacaa acgtggttaa acttcagaac
gtgcagattt
430321 atacggggca ttttatgaag ccaaggtttg tgttcaagaa atgcaaatgt
ttgggctgcc
430381 cttaccgact gccaggcaga actgaagcac gtggacggcc ccttcctgct
tcaggaagtt
430441 catgaaacgg aacagaaggt cctgctgttc cctgatttcc ttcaattcca
gcttcagcac
430501 ctgaagaaag aagacagggt gcagatttac aaagaagcta cggagggaca
ccagggacag
430561 aacctgaaaa cgcttacaga cggctttttg ctgcgcgtgt cagagtactt
ttgcaggaaa
430621 ggaaccaagg ctgaggaggg ttctgtcgct tcttcaggct ggaggaacaa
acatcgcttc
430681 agtcatatgt ttaaacccga gggaacgtgc acatcaggtg cagttttatc
atagacttac
430741 aggcgagttg tccatgaata tcaagagtaa atgattgacg gtgtcctgaa
agttcagcag
430801 agcagtcaga caaacacaca gaggacataa ggaccagctg cacttcaata
agaataatac
430861 gtgcagttat aaaattcaaa tttctaacaa ctctgcctgt acagagggcg
gagtttaccc
430921 atcaaggtgc gacctgatgc tcagaagcaa cagccgatca cacttagggg
gtcagaaatc
430981 tgaagattaa cgttgacttg aacgcgtcta cactcacagg atcagccaag
tagtccagtg

431041 aaggaagaaa gacagaacca gccaagactt ctcttatcag gagagtaaga gatctaagag
431101 gagtgaggag ataaagcagc aggaaatgag agtgatgctt atctacgaat gctgtcagaa
431161 ctcttcccag aggagaggag gaggggagga gggggggag aggaggaggg gaggaggggg
431221 ggagggaagg gctaaagatg gcgagccata aggagcagaa gtcccttacc tgcagtctgt
431281 agccttcggg ggcagaatgt gggggaagag catctcggtc agcttcctga ggtagaggag
431341 ctcatctctg cgactgcgca gggcgatgtg gaggtcaggg ccatattcct ccagggcagc
431401 ttgttggagc tgctccgcgt tctttactat agaaagaaag ttttgttggg cttccttttt
431461 tttttttaag ctaaaacgtt ttaaaaaggg agtccgtacc tttttgtctt gctttgctaa
431521 tgatttcaat gtgcttcatg gaaactttaa gaagcttttt agtgatgagg gacgccacat
431581 ccacctgaga cagagataga agaatgtaac aacaatctaa tgtgacccca gatattctga
431641 cttatcacct tctgggttcg gcggactaaa acagcagcaa agaatcgcag cgtcaccctc
431701 agttcatcca caaacgcctc gtcatcggtg atgtctctga gaacaaaaac acacttctgt
431761 catcctttat tctacactac tcaaataaaa atacaggaaa gcatgcttac ctgtaccagg
431821 gatacacaaa attttccaaa accagctcca gaatctttag aaaaatggag aacatagtaa
431881 aaatcaatga tactgctgtt attgtcgtgt tggtgtgtgc atattacctc tgaaagagat
431941 gcatccactt tagaagggat ttttaggtca agccagggtt ggtacttttc cagtagtaat
432001 gttggtctac aaagtgggga aagagagtca aatacaaacg ctaggtggcg ctgtggaacc
432061 ctgtgttatt ctataacnnn nnnnnnnnnn nnnnnnnnnn nnnnnnnnnn nnnnnnnnnn
432121 nnnnnnnnnn nnnnnnnnnn nnnnnnnnnn nnnngcggtt tctccctggt aggatgcaaa
432181 acctggctgg attccagcct tcatggattg gacttgccca tcagaggagg tcacatccat
432241 tcgtggaggg ttgcattcaa gccgggatta catcctacag gcaggaaacg ctttcaccag
432301 gtaaatggaa ccccaggtga gtgtaggaca gaacgtctgg cttggatacg gccctcgagg
432361 actggatttg cccatccctg tatgtgttag taggagaggg agcatatgtt gaatcaaaca
432421 gagcagatct atgaggcttt tttattttaa accctttcaa tttgaaacat ttaagatatt
432481 acagtgttta attcacaaag aatatgctga tgcagtatat acacttgtct ttctcctcct
432541 ttaaatggag gacacttaaa aagagtaagg agaaaaaagt atgataaaaa acaaaacaaa
432601 gagccctcaa tcagttgaca gagaagctgt tgcagattag gaattcggaa gaactgtgta

432661 taaatactaa actaccacct gtaaataaag ggatacattg tgtgaattta cattatagac
432721 acagaaaggt ttcaactctg caaggaaatg gtagtaaaat gaatattaaa agggaaggtt
432781 tttctcagcc ggtgtatcac ttatactgaa caatgtttcc tttttgtaaa taggtgggaa
432841 aactggacat ttggacacaa ctggacacaa cgctggggct tcctgcagcg ctgctcatac
432901 tgagatgagc aggagtctca cctgtgtctt ttgcacttga tctttccaca gacggcacag
432961 ctgtggccca gtggaaacag ctcctgctgg tatgactaca gggcaaagga aataagttac
433021 cctgagcttg tagatatatt gccattgttg caaatgcatt cagcagcacc acacctacct
433081 tcggctttgg tttgatggag acaaagatgt ttggaagcag agactcggga cccagagagc
433141 agtagaaggt gaccactcca gccaggaagg accaaaacac cattatgatg tggatatatc
433201 tgtggaaata aatatgctgt aagacaattt aaaaactcag gcaggcacat tgtgtgtgtg
433261 tgtgtgtatg tgtgtgtgtg tgtatgtgtg gtgtgggggg gggacctgtt taaaagcacg
433321 gtggagaaca gcaggaccag caggagaaag cagaagactg gatactgtct ccccagctct
433381 ctgaagcgtt cgagcctcat tctgcgccgt attttctgaa aacacaccca gatccatccc
433441 atttcaggcg ccgtggcggg gaacaagcgc ggtcatctta gtgtaaaaca cggacacccc
433501 caataacagc ccgaaggcca ctgaagtaaa acgccctaat gctactgctg cctccccgag
433561 cagctgcgct gttatgattc cacactacgg agggtgaaat gggtcatttt tcgcgttagc
433621 gcgcaagcga aatttgaaat gtattttcaa gaggaaagag ggattgcgtg aaggatcgga
433681 gcggacggcg tggctcacag ctgcgggatt acaggtgaaa ccacgacagc tgtctcccaa
433741 cgcggggaac gttcggctaa tggccgcggt gttctggcat ctctgccggc tgaggggggc
433801 ttctgtccgg gtagaggcgt gatgtcacct ccgctgtgcg gcggcgccgt ggatggcaac
433861 caatccccta atttcatttt tatatatgac ccattgccga ataataacca agaaaatgcg
433921 gcccggctgt agaacaatgc gatgattaaa gtggttttcc ctcagattgg accgctcgta
433981 accggaagtg catcgacaag gtgttgaact cgcgaccttt gagatgcaca gatttgaact
434041 tttgtacagg ggagaaatgc aatcagtact ccgaatgctt ctaatgcaat ggtgcccaat
434101 ttagaagaaa tatttagatt ctaccaaaat gtatattcat caatgggatt taaaagtagg
434161 agagatgata cagctgaaaa gaaaatggct ttgggtaaat caccagcccc ggagggactt
434221 agaactgact tctgtcagtt ctgagatgat attagagaat tgctcttgtg tatggcaaca

434281 cacttgtaac aacgaagcaa ggtataatta ccttaatacc aaaaccaaac agggatagag
434341 cagttattga tcatttaagg tcaattgcac tttttattat agattctaaa atctctactc
434401 atgcattggc cagttgactt gagattatga attgtcagct atgatttttt tttttacaca
434461 gtaaatgtaa taaagatttg agagtattag taacacagga atgttgtctc ttcccaactt
434521 tttttaaaga ctgatgctct caaaattaga agccgatatt tgttaacttt gttcactaaa
434581 atttttgagg atgagatttg atttggagga taaaaaaagg ctattttgtc aagacgttat
434641 tgaagcttaa gaacatctat tctacagctg cactatggtc agaaggtttt ctgaagtcat
434701 gttacctggg tttgaatagt tctgtcaagt ttgtcttctt catgcaaaac actgtaaaat
434761 ttgtagttta ggtttaataa tattgtgatt attggacagt tttatataca caaatgtaga
434821 ttcttaaagt tacttcaagt tttattgctt ttaagaatga cttaaacatt gtctatgagt
434881 taaagtgcct tgagacagtc caagcttcca ctggtatagt gactaaccca tcaagttcat
434941 ttcatctatt ttcttttcca tgttcattta tggctgctgc tgttttcttt ggacggtctt
435001 aaatatttta aatttataaa atattttgta atgatgtgtt tgagatgcac gtgtttttac
435061 tgaagctgta catcaacctt tttggtttat ttttgtggtt cttgcagcta ctggttacca
435121 ttaatttctg cacaatatct ggaattgttt acccaatggt cgttttgatt aatacaataa
435181 aaccgcctcc agcccgtctc catctcagct ttttatccaa agaggaggac acggatagaa
435241 atcaaaaact ttattacaaa aataagttac actcacctcc attttacagt ataaaacaat
435301 ttatttgcag gaatgcaaaa aatgttgttg gccaccaaca atagtttaaa actgctaaca
435361 ttttttttc caaaaggtgg ttgttatgat tttactgagg gagttaactg tatagaaaac
435421 tgggattgaa gagcagtttc cttctttttg ctatacctgg aaacagaaat ccttcactgc
435481 agtatcccct ctaccactgt gggacgatcc ctcgccgaag cgtggcggcc gccatagggc
435541 gcacgagctc accggtgggc ttaaatatgg caattcagtc acctgtcaag tttagctgta
435601 aagttccacc tggttgaggc ctctaggagc cgagaaggga tcgttcgaca tgattcagga
435661 ctgactggta gcatcttaca tgattcaggt gtcgaaaaag catcttaacc atataaaaac
435721 caaccactgg attaaaacaa tggtaaaaaa gggacactgc agggcttttt ctttttttt
435781 ttcttcccaa attcaaggca gtttgttgcc cgtgatgttg aagcctttac aaaagtaacg
435841 ttttatccga gaaaagaaaa aaaacaaaac tatacaacca gtaactacat actctgaaga

435901 agcaaggcgg ctaattcagt tcaaaatagg ataaaggcag ggcttgttcg gactcatcac
436021 actatattac attaagtaca ttgtttttac actgcagctc ttgtgcatat tttgtataaa
436081 aaacaagccc tgctttcatc aatatagttt ccctatttac agtacaggcc gggtagttta
436141 gtcctctgta ctgaggtagt caaccaaccc tttaaaagac aagttctgaa aagaaccact
436201 ttcctggaat gcctgactta ttcatgccag gtgggtacac cctaaaaacc tgcaaatgtt
436261 ctgtcaaaca tccagatcaa acaattaaga ttaacatgga tattccaaca ataaaaattg
436321 cgattagcac aaaaatatac aatagcatca agctcctttg agccacttca aacctaagaa
436381 cttaaaaagt gcaaattctt caaaaatcat ttttaaaaaa tggcatgttc agggacaggg
436441 aagggggaga ttgtaagggc aattcataaa accctgcata cgagggcttg cttacgagag
436501 tttggggcaa acgggcggtg gcgagtattc agaatcctcc agtcaacttt tgttagcttt
436561 tctttctttt tttttttttt tttttaaata tagggcgaga gttccagata taagtgggaa
436621 taaacgtata ggggagatgc ttcttctgta aggctggaat atcattggac catgttgttg
436681 ggcttttttt ttttttgctt tttttttttt ttccataaca tccattgcag cttatggtgt
436741 aaccccacac atcaagtcag tatccactgt gacaggtcag gaccgccctc gaccccgctt
436801 ccctgctgcc aaggagagca aagattgatt agaaaagctg tccaccttta aaaaaaacaa
436861 aaacaaaaac aataatcaac actgtgtgat ccatcggtta aatgtgctgc ctttaaatag
436921 caaaactttg cgttttgact tgcctctgct gctcgttcca gggcctcgct gggagaaacc
436981 caatcggccc ctgggtggtg cggcgcctcg gcccttgttt tgactggcgg cgccgcggag
437041 tcccccccttt cctcgtcctc tcccagttcc ctggaagtcg tcgtagacgt agtaggggtc
437101 gtcgtagccg ccgcggtaat tgtggtagtc gtatccgtag taactgtaat aatcttcata
437161 gccgtaataa tcgggagggt aggcgtagcc cccgcgcgct cccctgcctc tgcctctcga
437221 gggtgggggc atgtgagggg ggccgtaata atagtattca tcatacctga aagagtggaa
437281 aattcgacgt gaacgtggga tctggttcga tcgtgttggc taaaaggtca aaggttacgg
437341 ctcttacatg tgtgttttag cagcttgcct ctgagctttg cgctctttcc tcttctgatc
437401 aggaggcttg gcaaaaacta tttcaatgtg ctcgccctcc aggtctttgc cattcagatc
437461 agccagagcc tggtgggggg ggggggggaga tgatgacatg ttttagagcg ccagcaactg

437521 gccatcagaa gtgaccggct tttctccctc tccatacctt cactgcacca tctctttcct
437581 caaagtggat gaaggcgtaa tctttgagtt tcttcactcg ctccagcttg ccgaactgac
437641 tgaaggcctt ctccagtaat tcctccgtga cgctgctcgc caggttcctc acgaacaaca
437701 ccttgacctg gtgacggcca gagaaaattc agggtcagcg gaaagcagat acagacttaa
437761 atagccaggg tggtcgccct cggccctatt ctgaccttgg ccatgacctc tgggtccggg
437821 tcctcgaggg gatcagccca ctcgacggtg accaggttcc cccagacctt gaccttgccg
437881 ctcatcagcc ggcggcgggc ctgagctgct gttttgtgat cttcatactc caagaagcaa
437941 aagccccggt tcttcttttt gtcgactggc tggaggtaca atatgacatc gtttagaccc
438001 tctgtggaga catcagagaa caccagtcag tcacagattt gctttaaggt cacttttaac
438061 tcaacccgac cacgagatga ttctggctca acgacttgtt gcatttagtg tccggtcaga
438121 tcttagcggt aaactgtcat ggtgaaacca gtcactccag tatagtcatg aggtcatcat
438181 ttggacacaa acagattccg tgatcaggtg gtcaatgcag gaaggggggg gggggggatc
438241 tactgaaatc cagagcagct gctaactcac ctgtgacttt tgagaactcc tcgataatct
438301 gctcttttgt tttactcttg gggatggagc ccacaaacag tctgttattg gccacagaga
438361 tgcaaacacc gatgtgttta ccaggccgga tttcattgtt gttgcactgg aggaaaagtg
438421 gaggagaaga aaagctttat ttactattgg gtaaataagg attggaacaa cgtgttgccc
438481 tgggaggggt gccgagcgga gaaaaggggg acttgtgctg tgtctggact ggtgatagat
438541 caaactccta ccaatttcac cgcctgctgt gcagcttctt tagtgcagaa agtgacaaag
438601 gcgtagcctc tgttcaggcc gctgagtgga tccatcatca gtcgcaggtc ccagatgggc
438661 ccggctttct caaagagcgg aaccagctca tcctcaaaca ggtctctggg tattttgccg
438721 acaaatatct gacagagggg agcatgaaga cgtctcaatt tcaactggag agaagaactc
438781 cagctggatg tcagttgaat acgtttccat cagctcttag cggtaaacta tcaaagtaac
438841 aagagttact acagtggtaa gagtgtcatc acgcggatgt ctaaatcctc aacacctgct
438901 gggaactgct cgtctttacc tctgtaccga tggttggctg agcccctgaa tgaacagctt
438961 caggtggggg accgccatac ttcctctggc ccgttgtcac atcaagtgtg tagccagtcc
439021 tctccagcaa agcctgtgat tggacaccgt acaaccaaaa aaatgacctc atttctatca
439081 gacggcgtcg tcgctttgga aattaaacaa atggcgccct cagatgtacg tttcttactt

439141 tgattttggc ttcatctggt cctttactag tgtctgacac tttggtccct tgtttctctc
439201 tctgcctgta tgtcttcatc acaccacaaa gaaaggcact tttgttctaa aggcagaaaa
439261 tagcgcagca gtatgaaaca atgattaaaa catgatgtgg ttctggtgtg caaaagtgct
439321 gcacatggtt ccaaaagaga caaacgtcaa attaagttca tgcagactcc acgtggaagt
439381 caagtgctct ttaatccacg tcgttgtgaa ggcggcggcg gcgcggacgc acctgaacat
439441 gtgagaggtc actgtcctta aattgcaaaa gaacttgcag agcgccctcc tcgttgaact
439501 ctttcagcgc ctcgatggct cgatcatcta agtcactgtg tgacaccaga cctgaaggcg
439561 ggcgacagag cacaactgct gagttacggg attaaacatg ctcgacatca tcacaaccac
439621 ggctgagatt ataatagcag ccattgtgcc gcctcgcctg aaatctttca aattgcgtga
439681 ggccaataaa ttagagttat ttctgtgaaa ggggggaagc tgcttgcagc cctgacctaa
439741 ttgaactgta ttctgtggtc aaagagcact ttgctcccca accagcacag tgttgcagaa
439801 ctaatcacaa cagatgtggc agtgcaactc gatatgcctg tacccccgtc tacactgtgg
439861 gagatttgaa tgagctgatc tacagtctcc tctgtggaag ggccatcacc tgtcccagac
439921 tgccccgcac cagacagctg ccctcattgt ttctagtctc gccccatctg gttgaaaagg
439981 ccaggccgcg attttccgag gtgtgactga acgtcctaaa atgaccccga ttgtgtctct
440041 ggtgcttctt ccgtattatt tagtggtctt ctaaccaagc agcaggtggt gtggctggtg
440101 tgtctactcc aacagctctg agatgaacgt cataaatact cagggtgaaa atgggacggc
440161 aaagatcaga ccatcacggt tacatttata cacttgtgtg gggtcatatt ctatgataca
440221 tatttcacac tgcagtaccc attttggtag gaaaccacaa aaagttgctt atattccccc
440281 attccaaaaa ggtgataatg tgacaataca tgcaagaaat tgataaacag agaaaattgc
440341 atatttcaat atgcaagtat attcactgtg ccttacctgc tatgtaaatt tcatctagtt
440401 tttcagcaac tttctgtggt aaaccagctt ctaataaagt ctggaagtgt tcagaatggg
440461 taactgcagc agaggtgtcc attgggtctt ctggaccatt tccattaata tgttctgtgg
440521 ccatgtctcc agaaatctgt gcaaatgcaa tgagccaaat taatctcgga tcaaggggaa
440581 gacctgggat ttgttcagaa gagtggaacg tctcagactt ctcatggaaa cgtgcatcaa
440641 cgggaaaatg tggatttcgg tcatttagcg ttagcgatgc aaaaaagaaa aaagaaaagg
440701 aattgcccac ataaatgaca tcaagtgtta aagcacacaa acatactggc tttacaaagg

440761 tgtgtcggtt tgctcttcct tgctccaggt tagtgcacta agttcacctt acagcaccgc
440821 tctcacggcc ttctgacata gctggacagg aaccctggtc tgcactgctc tctaaattac
440881 acgttagctt actttcccat tgctgacttc agcacaagct agatagttga aacacgtgtt
440941 ccaatggaga gtgactcccg gtttccggtg gctgaccccg ttagcggacc cattagcagt
441001 tctttcttga cactttgaca gctattatcg tgagaggaac gtggaaagtc caacgaacca
441061 tcgatctaat cttaacatgc gcgaatccaa agaaaccgtg ggaccaactt ccaaagcata
441121 aatctatatc attaaaaaaa aaccctccct tcatactgtt gaatttggca aaacgacgag
441181 cgttttgctt aaagttaact cggttgtcgt cgctgtctta aattacaatg cagcaaattg
441241 tatccagcaa tgcctgctaa tcaagcttat attggaaagc acgtgttttc tgtggttaaa
441301 taagttctcc tgcgtttta gtgcttgagc acttaatttc ttacatgaat aaatgaggtg
441361 ggggggatta aacgaagatc ttgcacacaa agtcacgtac cctcgggtat attttcgtgt
441421 ctgaggttaa ggataaggtt agatctaagc tgggagggac tgactcgcac acgttgaggc
441481 tagctagcag gctagcttgc gcagctaatg ttatagctat cgtgctaacg gtgaacctaa
441541 aagacaataa cataacagac cgtacaccat tcctacgtgt gcacctctta tttggaggtt
441601 tgatgggagt gcggctgtca aacggtgcag tagcaacacc gggtcgatta ttcttgctag
441661 aagagctgac tcggttagca tatgatgaca atgaacatgt caattccacg atctgtcaaa
441721 ataggttagc tcgggctagc attgctaatt cgaagggaat tacattgagg ccattgttag
441781 tcatttgccg acacgagcat tacttattac tgaagcacgg ctacgaaatt aagtcaacta
441841 caaccaggcc ttgaaagcta gaggtctagc ctgctaaatg cgtggtttgt caacgtgccg
441901 ccatatagga agagtccatt ttcaaaaagc cggtctgggg aactcgctcc caaccaccac
441961 cagtttccac attgtttaga acagcagcac acgactcaga ctttttactc aaggaggcaa
442021 caatgacgtg accgatcgat agcattcgcc ctacctgccg ctggctcacc aacgtgtatg
442081 gtgttggtaa ttaaggaaga taaatgtgat tttaaaaaga caggaaaagt gtaaaactcc
442141 cggcactgat ccggctccag tcattcactc ttgatgatgt aaaatggctg ccacgttcac
442201 gcctcgagtt ttgtctcgtc gatggggttt cttggacagc cccgcacggg ctcgccttgt
442261 atgcgttata gagcggcggg tcagctccag catccatcct aattattagc tacggacagc
442321 cttttgtgat tatacagcct gtgatccgca taaaacgccc catccgatgc ccctttcgag

442381 agataaagag gatgtttaac gtaggacaac tgggttgtta gtgtatgcaa tgctcgattc
442441 atcatgaatg catttgcaac atctgcacat atcctccgtt aaaatgtggg tgggaatggc
442501 ctcccactta taaaaacgca tgtccacgtc gcccgagtgt ccctgagcaa gatttgaact
442561 cgctgcctaa tttaatgcag ggagcgaggt tagttccgac tttccgccgc acggtgtcgc
442621 tgttggcgta gatatcgcag catgaacaaa agacttccaa attcagcgag taaacaaaac
442681 aaaatgcgtt ttgagcagac tcccccctc ccatacagag agacacattc aaacggaacg
442741 ttctattaat tagtagtaga aaattgcgtc tttttaaaaa aaaatgatga cacttccata
442801 taatctggtt gctttgcagc ttcgtacgtt caggtgttgg tttgatttga atttacttta
442861 tggcatccaa ccaaatcaaa accaaactgt tgaacatgga ctgtgtgcag cacacaataa
442921 taaaacttca caaatcacaa ccaaaggcaa tgattttggg gtcactatga ccaggagtga
442981 ccttaaaacc ccaccctcag gtccttcaaa tgcatctcac tatagtgctt caacaaggtt
443041 gttcatgaaa gcctgggttt tctgcagctg tggcttccca cataaccgtg gctttcagcc
443101 tctcgtcctc atgcagccag atcaatactt tataaagagg gcgtccttgc atgtgggctc
443161 tgttacatgc agaacacgag aagcttggtg cccttccatt cgtttttgaa tgaggaatct
443221 gtggagcagg acaataaaac cgcttttggg ttttaatgaa caacctttaa aatatttgtc
443281 atgccccaaa catgcacatc atggccaggc aaatcaccgc aatatggaca agaatcagca
443341 gttaactcaa aactacatag aaaagaggga acagtgttcc gtgtcagtgg ttaaaataat
443401 aggacacagt ccggaacact gtcttctgtt tattcgtgtt tatcagtgaa ttctcaaaca
443461 gtttgtttcc tttggaagag ttgttgaaaa aaaaaacgat gcctcagacc tcacaaatag
443521 ctttaaacat cataacataa ctttagcaca tgttcttcgt ggctcatttt gaagtcaatg
443581 atcaacacag ccacaaaaag caaacataga tttgtataca ggtatataga tactccaaaa
443641 aagctctttt gagcttcctt ttaagtattt tccttaaggt gttgcaggca ggcaagaatg
443701 ggtttatatt cgtcccggct gttaaagagg tgggcaacat ctcatgtccc tgaatttatt
443761 cagaataaat attttactaa aatgacctcc aagcacctgc tggtcatgca aaaagacaac
443821 aaatgttaac atctaagaag ctaaatccca gagtttgggg cttttccctg ttttaacctc
443881 ataagaactc tttattaatg gatttttctt gatttccagc aaataagcag cattagagta
443941 taaccactgc tgtggtagta taacccgaaa tgtgaggcca ggtcacggga ggctaaagaa

444001 gcactttgta aaacaaggat ttttacaaag gtttgggatt ccgaacctga
agaaatccct
444061 gaagatattc cagaacacac ttgggccact gtcagcaaac ccttcacgtc
cttcgtcatg
444121 acaaatgaca aagccacagt tacttggtga caaaagcctg cggattaact
tcagcatcca
444181 tagtcaggac gtgaggagat tgatattgac tttccgctgc ttttgttatt
atttgctgca
444241 taaagggaaa ggtcacgaag cggaaagtcg gcgtggacgg cgataacatc
agtaggcttg
444301 gctcggaaag tcgtcacagg ccggcaagac ggcggtataa atgacccgga
gggaggccga
444361 actccggaaa actaaacaca ggtgtcccgc agacggaaaa tgaagggaga
atcttcgctc
444421 aacaaggtga cggtccgtct tttgacttcc tgtggctatg atgaaacggg
ctttgacccc
444481 ctgtcttttg cctctgtact aggtgacagc aatgggctca gccaaatcag
ccggagcggt
444541 tcttcttctc ttgtctttcc ttctgtatgt agctgactct taccccaact
ttcccaaatc
444601 aaacagtaag atttccacag aagattccta gaatgaattg cgcctttgac
ctttgcgcct
444661 ctgctcaatg acctttgagt ttaaactctg gatgctctct ccccagtggg
ctgtgaggag
444721 tgcacgctga cgaagaacag cggcttctcc agggatcgtc ccatctacca
gtgcatgggc
444781 tgctgcttct ccagagcata cccgacgcct ctaaaggcca tgaccaccat
gaccatcccg
444841 aagaacatca cctccgaggc aacgtgctgc gtcgccaagc agagctacca
ggtgccgccg
444901 acaacgtctg catgagctca acgccccgtt cacctcggaa gacattaaaa
cctaccgttt
444961 ctgcctcctt cccgacgctc ctgttcactc ctgcgtctct gttcctcttt
cagacggagg
445021 tagccggcat aaaggtgagg aaccacaccg agtgccactg cagcacctgc
tactatcaca
445081 agatttgaca gctggaagat ggagaccgct cttcagtgtt catcctggcc
ttgagctctt
445141 tcttttcatc tgcacaagct gttataaaat gtaatttcct gcgtcgccag
gtctgttctg
445201 tgactcagtg tttaagatta ttattattat tgtgcgtggc tagatgcaac
agattaactg
445261 tagattgaaa agcaattaaa atgtgaacat cgatagatgt ccgattattt
gggaacgggg
445321 atggagagga tgaacctgga ggtggaggct gttataaata ggtcctaaaa
tgtaacacat
445381 acacactcga agcctctgat gtgaggagaa ggccacaaag gaagctttat
tcaaaagaaa
445441 taccttctac atcgtgttcg gtgcaatcaa tttggaagga cagtgtgctg
acctgtttaa
445501 cagtgtgctg ttagctgcct tcaggtgagc caaaataatc acccccgttt
gtgaacatct
445561 gctcggctta agtgtccttg actttaaaaa gcatgaccag gccaggaaaa
taacaaaaca

445621 aataatgtgt cacggagggc ggagatggat tagtttgcct ggttttgctt ttggaaggtg
445681 gatgagctcc tggtgcttat tgcctggagg tcatagctga cctggagctg catggatgga
445741 ccagggggaa acacagaact cctgcccgcc tcggatcagc tctcagcggg ttcgctcacc
445801 tgcacttgtg cacgagtgca attttttttt taatcgtgca cggtcttttc cccccaaaat
445861 ataaaatata tttacacata tatatcacca tttctattag attttgttga caggttggga
445921 atgggtatgc ggagcacaca gtgtgctttc ccttcccccc tgcgtgcgcg tgcgtgtgcg
445981 agtctggcag cgtaacatct gtgcgtacgc ggtcgtgatt gttgtgaaga tggcggaggg
446041 ggagacagag aaggaatatg acacacggaa tgctatcggg gagctccgag agcggttcca
446101 gggcttgacc acgactttga aagagagctc gctgtctccg ctggaagcct cgctgcgttt
446161 ctgccaggac ttttgccagg tttgcccgtt cttctgctgc tcatcccggg gcagcctgca
446221 gcccctctag cttcctggcc gaggatgcgc gtctacgttt gctacaacaa tgtagctaag
446281 gagctagctg ccttgctagc agcctggcat ttaagatggg gaaaaaaccc cgctggcgct
446341 tttcaggcac tatctctgca atatgtgttc gcgatttatg cgtatttcca aagtaaaaca
446401 gtgcacgcag gcaggggaga gggcaatgag tggtaattac gcatttatta accgtatttt
446461 ccaggccaga tggctaatag cagatggatg ttgctagctg gtatgcaggg tccccctgtc
446521 ctgtgcagtg gtcatgctgt gcaaaaatgg atagctacgt tagcgagatt ttaccaggcg
446581 tttgcaccga cgtgcacccg attcgccttc cctggaaagg ttcgaatgct acgaccgtgt
446641 aacaacgcct attaaagttt tcgtccggcg ttaaagggct gacttcatgg agccgctttt
446701 atcgcacgga aagatcaatg attgttccaa ggtgctggcc cgggccttgt gcatcaatta
446761 atccagccaa ccacatcagc gcacaagccc ttttccagca gtttgagcag gaatggcatg
446821 aaatggtgat tcggtgtcag tgttggcgat gtggctgcag atctttttct tttttttaa
446881 aaaaaaaaga gagaatagag aaaataaaat ggaaagtgtg gctgtggtca gtgactccat
446941 tacacctccc ctcattaatt tgtcctgatc agttcagcag catgcgaggg gtcggtcgac
447001 tcgatacctc cccgaaacct cctcgatcac cgcagtttca ctgaaatagt aaaagtttgt
447061 agggggattc atgtgtccag gagggacccc atgcagcgct gaagtttttg ggtctacacc
447121 cgggtgggct gcagcttatt gcgcagaaaa gccacgatgc agattttttt ctctcctttg
447181 tagctcaaca atgaatgaat gaatgagtga agtttttcag ggcgacgctg atcaataaaa

447241 aggcttttgt atgaaataga tacacagagg gacatttcag agatggggat
ttgctgctgc
447301 agggctgtta aaatatggct gaacaggaaa cactaaaaaa aatgctgcaa
agcttgaatt
447361 tgcttgttta cattaggcgt gaatatgcat taattccctc ccaccaggtg
gagaaacgtt
447421 tgtgaattat tcgttttgct gttcaggcct tggtttaggt aaacagcatt
gatttgtaga
447481 atcagctgag cataatgtgt atatatgtct atatgcacag atctctcttt
tgtgttggct
447541 caatagtagt tcagttgtag agaatgtttg tttttaatta aatgtcagca
tttacttaag
447601 taaagtactt aagtattgtg gtattattct atggtaggaa aaatgacaca
taaaaatccc
447661 caaaagtcac aataaaaaca ctagtgtgaa cacattttag tttattgtta
agaaaggcct
447721 caagatgttt atgtaaagca ttagacatta aaaaaccttt aaacatgctc
caaagctttt
447781 gacggtagct gaagtatact gatggggtgt cgcgatgttt tccaggtgct
agtggaacac
447841 gctagccttt ggaaaactga tgaggatcca ctgcctttgc tggaggtcta
cactgtcgcc
447901 atcgtcagct tcgccaaggc tgtctcccat ctcgcctccg aatgtgaaaa
cgcgccactc
447961 ctacttgaaa agttagcact gtgagtacga aaccctgact gggattctca
gtcagatggt
448021 gatgagttga gctgtgaatt gtacaacatc tctaagtttt ggttcccccc
ctttgtaaac
448081 atttcacaaa tgacacttaa tacatgtcag gaaacaaaga aaccatcata
gaaatataga
448141 ttgagtactt ttactggatg atttttgcac acagactgag gagacagcag
atgaagctcc
448201 accgccccgc tgatgttgtt cttgtttggg gagtgtaacg tgtgcacgga
tccttgacct
448261 aaatgtatat ttaccaaatg actgtgttga ctatgcagga gctgtgcgga
gctgttgctc
448321 ttactgcccc agcatgtccc tggtgcctta tgggaggagt ttcagacctc
catgaaggtc
448381 agtatcatta ggcttgttaa gatccgatcc cttgaggctt atctttgcat
ccactggcaa
448441 tgaaaaatca ttgaaaaatc gatgacagaa gcgattctgc aggttctcct
tgcagaggct
448501 gttgcgctat ttagcacata tttatctgct tttacagttg gcacacggcc
tcttgcaaga
448561 aagcgggagc acacagcttc tccttctctc atttctggca cagcaggatg
gcgtctggtc
448621 aaacgccaca ctaagcagca tcttatccaa tcaaatccct catattgaac
aaggtcagtc
448681 tttccttgac attactgtta tggtgatcag aaatcccacc ggtgtgcaag
aactgtctca
448741 tttatagttt ccccttcgtt gctcctctgt tgtccagttc atgagtatct
tgagttggaa
448801 ggtccagcac ttctcaccat gcgaataaag cacctgatca aagtggaaag
cgttgacaaa

448861 gctgccattc tggccaaggt gtgctccgag tacccgggat atgaaggaaa aggaaacttt
448921 aaacagacct acttggtttg catctgcatg acgaaaagtc aggagcagct gatggaagag
448981 gtatgaaata aaacaggttt ctggagccca caaatcattt tatcaataat ataccgtctg
449041 caaactgaaa aaaaatcatc aaagttgcat tataaaatca tgtaaaggca caactaatgg
449101 atttaaaacc gatttaaaga gccaaaaata tgcatttaca ttccacatga tctgatttaa
449161 atgggctcgt tatttcgttc tccctgcctc ccctaataga tttcatcagt ggactgcaaa
449221 gatgctctcg aaatgatctg caatttagag tccgagggag acgagaaagg agcgctgagc
449281 ttgtgttctg cctttctcaa gaggcagctt ctgcaaggag atgtttactg tgcctggtga
449341 gttgtgcttt tcattgggaa aaaaagattt cattgttagg taacaagttg aacgttttct
449401 gtaatgggag caagcacaaa agggaaaaac agtttaaaag cgggaagact ggataacatt
449461 taggctattt tagtcagctg aagcagaatt attgtgttga ctgatcccaa cctctcattt
449521 ttacagtagg actctgtaat atttttgttt tggacgcaac agaaaacagt attgcgcaat
449581 tcacctttgt ggtcttgtgc gcttgctcaa aatcactgaa aaggctccga cctgcgttta
449641 tcccaccatg aatgtggtgg cgtattccaa atgctccact ttggagcctg gtttcgtctc
449701 gctcctcttt caggcgcttc catggaaggg gacccaaaac gatgtcgttt acacggggac
449761 ttagttcagc tctggccgtg gtaacgttca gctgctgtga ttttattttt cagggaactc
449821 acgctgttct ggagtaagct gctgatgcgt ttggagtcat cggccgacac gtttcttggg
449881 aagagcaaag agatggctct gctctgtgga agcgtctgtc atattctgtt tctcatcaag
449941 gtgatccaaa acgaggtgag gatgattaat tacgaactag cttctgataa ggttaagcgc
450001 cactgttgac gacacgaaga ctcctgtaag ggaacttacc ggaagattcc atagtaactg
450061 ttttattccc ctaggttgga gagctggggc ttcccgtgtg tgtggaaatg tgcattcagg
450121 ctttgaaaat gacctcccct gaccataagg acaccaagtc taccatctgc aagaccattt
450181 cctgcctctt accgaccgat ctagaggtca agcgtgcatg ccagcttacc gaattcctcc
450241 tgcagcccgc tattgactcg tactatgccg ttgagtcact gtacaatgaa cctgaccaaa
450301 agcccgagga ggacgggagt ctaccagtgc ccaactcgtt gcgctgcgag ttactgttgg
450361 ccttgaagac acagtggcct tttgaccccg agttctggga ctggaaaacg ctgaaacgca
450421 actgcttggc actgatgggg gaggaggcag ctattgtgtc atccattgac acttttaatg

450481 acacagatga acaggaagtc gaaacagcgc ttggaaagct tcctgaatac agagatctgg
450541 aggatttcct gctaaccact acaaatgaac tcaatgaaat cacagatgaa agggagaaga
450601 acagagaagc taagaagctc cgggagcaag gttttgtgtc agcccgtttc agaaactggc
450661 gagcctacat gcagtattgt gtcttgtgtg acaaggagtt cctgggtcat agaattgttc
450721 gccacgcaca gaagcatttc aaggacggca tgtatctctg cccgatttgt gcagacagct
450781 ttgaaagtag ggaggtttta gaaccacatg tagcatcaca tgttaagcaa tcctgcaaag
450841 agagactagc tgccatgaaa gctgctagga aggtaaccaa accacctcag tcccctaaaa
450901 gtccatcaaa aaacccaaaa gttgctgtca ctacccctttt caaagtggaa tctcagatta
450961 aaccagaaca aaccacatca gatgccaaca tgagtcaaga ctgtttctgt cctgtcaaaa
451021 actgctccaa ggctttcaag tttttccgta acctcatggc tcacgtcaga tctcacaagg
451081 acgacgaaga ggccacgaga tttttggaga tccagaaaca gaaagtagtg tgccagtact
451141 gcagacggca atttgtcaat gtcagacacc ttaacgatca cttgcaaata cactgtggca
451201 ccaaacccta catctgcata cagctggatt gcaaagctag ttttaactct aattctgaac
451261 ttctcatgca tagaaaaacg catcccgagt ttaaggccca gtgcatgttc cccaactgtg
451321 gccaagtttt taacgaggcc tacttgttgt acgatcacga ggcgcagcat tatcttacct
451381 acacctgtca gacggataac tgtggcaagg tattccattc gcagtctcag tacctgtctc
451441 accaagagat tcacaacata agttacgcag tgaacagtct acccctgact aatgaaaacc
451501 ctcctgccac tccaaaagta caatcaccgc ttgtgaccac gctgagcctt gaaaggatta
451561 acacagatgc tgaaatgaag gaggcatcga gggcaacagc atcgccgtgc acatcagagg
451621 agatggccaa agacggttgt cccgtgagag tgaaacactc aattgagagc atgctgaatt
451681 gttccgatca ggcgctagat gagccggaca aatgctacac tcccctgacc aaccccactc
451741 cagtgccaat cgacgtggcg tcggcgccac cagaagcggc attagcacaa aacgatgcgg
451801 ccccagcgcc cgaggctcct cagccgcccc aaaaccttcc cgcgcatgtc gagatcaccc
451861 cagtttacac acacatgcct actgccccga atgctgtggc aagccaacag agagagggac
451921 ttaattatat ccaaggcaac gacgctccca aagcagttat acaagcagta aatcctgaaa
451981 aagtcaagac ggaaattcct tcctcgctgc aaggttatcc ttccaacaca cctgtgactg
452041 ctggcggcga ggagaatcag cactgctgcc cgtttaacga ctgcacgcgc gcgtacagca

452101 cgaacaaaag tttatctaga cacgtgaaaa agcaacaccc cgaaatattt gaggactgga
452161 aattggcaaa gaaatacaac agagtggcca aaattaccag caaaaaagca tcaagagggc
452221 caggtctccc cttccagcct gagaacacag gaaacagacc tccaaatcag ccaggaatac
452281 tatgcaaaaa acccgggatg cagcaaatgg attatccagc ggaatgttca tcctcaccag
452341 cccagtgtta tcctggatca atggagcctg tgcctatcac tccaatggtg aacccttcac
452401 tgtacccagc atggggaagc tcaaacaatg ccggcggaat aatgcagtca gacatggccc
452461 agtcatggtc cgcacctcct gttaataact gctattcaga tgccttcaat atgaatgaat
452521 atccctcccg gagctaccct cagtggcagg cagaccccta tcaaaccaca gcacctctgc
452581 aatccgatag agatccttca atggcagcta tgcatggccc cagcgtgtca cacacacctt
452641 cagattcaag tttgatgtct cagtatgtgt ccagctcttt gatgctcgac aatggcggac
452701 agatgcataa cggaggtcat catcagtatg gaataatgcc tcctgagggc agcgcagaca
452761 gcgtagagtt gagaaagagt aatgaaaata tgacaagcca ggatagtgcg cctaatatat
452821 caactattga cagtctcccg gagggaaatt accacgctca gtatgatcag caagaaaact
452881 ccagtccggc agaggccgca ccagttgata ttcacatgaa atgcctgagc agagaagcac
452941 aggtggcttt aaaagctgca gaagaaacag ttaaaacaga cagtgtatct actccctacg
453001 aaccaatgga cactgcggag ccaaccagtg tctctcaccc agaattcccc tatgaagagg
453061 actgttccaa tgctgactgt gagatgaaca ctccagagga caaaggcgtt gaccccgaca
453121 cgcagaacgc caaacgcagc agaatgagca agaggaccaa atggccagcc atcattaagg
453181 atgggaaagt catctgtcgg agatgcttca gggagtttgc gagcaccaag tctctcggcg
453241 gtcacttgtc caaacgttcc cagtgcagac ctgtggatga aatcgacttg acggccgatc
453301 tgccgacgtc tttccttgat tttctgaacg acccccacgt ccccgacaca aacggtgcga
453361 tgttcatgcc aaacggcgat ttctcacaag aatcttgcag tctgactcct ttgaacgcac
453421 ccacgcagtt aaaacaggag ccgcaaaata ccaatataac agactttccc aaacctgcga
453481 ctgttccccc tgaaacccaa caagagaagc aggcagatgc agctaatcca gccctagcag
453541 tatctcatca ggaggatcac ttggtggaaa tatcacacgc atttcaaagg ttagatttga
453601 ttgaggctgc ccaagaaaag atgaggagcg ctctggcgtt agggcagacc gtcagtcatg
453661 gtgccacaac cgctgttcca gacaaaagca agctgctgac taaaggtgat gacaagccca

453721 ctgagaaggt gcacaaacct ttcaagtgtg atcaggagga ttgcgattac
tccttcatga
453781 caaaggaggc attattcaga cacttgacca agatgcacga ttacacgaat
gagatgatag
453841 aagagttaaa aaggacccca tccaagctgt ccccctaccc ctgccagatc
tgcccgaaga
453901 cgttcactcg aacaacgggc ttgaggattc actacgagaa agtgcatcgg
ttgtcgaagg
453961 cagaaatcca gaagcttaag attagtgcac gaaacaggcg tgcctttcgg
ttaaataaag
454021 atggcacggt ggttagccag gcaaccgctg attccaacag cagccccgcc
acgcagtctg
454081 cagcagcatt acctgctata aagcaagaac cacttgacat ttcaatcgcc
cagcaggcag
454141 ccgctgacaa caatccagaa actgttcaga taaactcaca aaacgaagca
ggtaaccagg
454201 acttgaatcc cgaggccaca actgtgactc aactaccgtc acctcctaac
aacttgactg
454261 ctaatttgtt tggtgaggtt cctccttcag ccccagaaaa cccacaagtt
gcagtggtac
454321 acaacactcc agatctaatg caaaaatacg aaattcatga gacattaagc
ccgaccgtga
454381 acgcagccct gcagcaaggc caaacggatg aatcaccaaa caaagcaaat
gagccaaagt
454441 ccatccctgc atctgcatcc tcctccccag acaagcctgg cagttctaaa
aacacaccga
454501 ccaaggatga atcccagaaa agagagagag cccagaaaag gttgagtctg
aaaatgtgtg
454561 agacggacaa cggcttcagc ccgtacaggc cctaccgctg cgttcacgaa
gggtgctccg
454621 ctgccttcac catccagcag aatctgatcc tccactacag ggccatgcat
caggcggcgc
454681 ttccccccat taagcacgag gtggagcctg aagtggcgag cgtgacgagc
gtgccggaaa
454741 acaaccagag cccagacaaa gacgacgagg tcagatgtca ggtgaaagac
tgttccaggg
454801 ttttcatggg agtcacaagg ctagtgcagc attacctctt acttcacaag
tttacccgtg
454861 acaaagccac ggcgatgatg gccagcatgg acatagggac tttccactgt
gatcaggctg
454921 agtgtgcgct ccccttcaac tctgtcgaca aatacataga gcacatcaaa
aactttcaca
454981 aggaaatcgc catctccgag agcggctccg ttgacaggac ttttaagtgc
gagtatgacg
455041 agtgtgaccg cgtttattcc actaaatcta acctcctccg tcacctgata
aaaaaacaca
455101 actacgttta cgatccaaaa agaaccgacg ggagaagaac taagtcagta
gggctcttca
455161 caagcgtcac cgatgggaag gagaacatgg aaagtaagtt gaaactgaag
aagaaaaaca
455221 ctaaaaagaa agatgggaag tccattgaac actggaccag cttcgggaag
cctacgctaa
455281 agacccacga ggaggcgtcg gccatgtgca ccaagaagtc ttccctacag
tacccgtgca

455341 tgataatagg ctgtgacgca gtggagtttg ctgaaaggaa tatatttaag cattacacca
455401 cccatggcct cacagaacga tacattgaag atcagaggag ccagttcata ttctgcaaaa
455461 agttcccacg tgcgagattc aaagattcaa acaaaacgga aggggcaacg agctcatctt
455521 ctgaggagac cgaggcagag gacggggaaa agcctgggga ggaaaactcg gaggagcccc
455581 tgtcggatag cagcagggtg gaacaagaag atggcaagct gtctaacgac gatagtgcgg
455641 agtctcgatc ttccaatggg atagaaggag gagcgaaaag aggccgcccc aaaaaaacctg
455701 cccacccaac accagcgtgt gcagagagga tgcagaccct gaggaatcgc gccggtgtca
455761 cgacctcgag agagaactca aatcctggca cccccacagc ccaagaacaa cgtgaagacg
455821 gagttacacc cgtatctttt aaacctttag gacttgaaga ctcttttctt aagttcttgg
455881 aagcctccga gtcaacacaa ccttccaagc gcaaatttaa cgataaatct ggtgccgagt
455941 tgccgcccaa aagacaactg actcacaaac agaagtctat cagaggtaaa attgccgaat
456001 gtgaaattct cgtcgacttc agaaatcctg tcaatctcaa atcagcgaac aatgtcaaga
456061 ttgtcataga taaaacattt tcagacggtg ctgatctttt gctaaagcag ctacaagaca
456121 tgaagcccct agtcataatt aagaagtggt tctatagcgg atcgtagccc agtgttttgt
456181 ttttaactag atctcactca ggatttgaga tgttatgctg cagaaagggt ttgtctgttg
456241 atgcatcggc ttttcaactt ttaacagtca cagtcacagt tcatgatgaa atggcctatt
456301 ttgtacttaa aacacgtctt ggatgatttt aagtatgtat gttagttatg ataaccaggt
456361 aatctatagg gcatagattt tagaaccatg tttgtattta tgcttagtac tgaagatgtt
456421 tttttttttt cttttgctaa atggagactt ttaaagaaaa acgattttat atatccctat
456481 gggattttca agttctgttt gtatttattt catccttttt aaacactagt agacctatct
456541 gtaatattta catgtcttcc acagtgttca ataaaataat actggtcctt ttttaaaaga
456601 ctgcatcatc tgtgtttttt aatattttaa tataataatt tattgtctat ttgcggacaa
456661 gagggcttta tatcatcctt gcacaggtaa ttggcatagg ctcaataaaa taaaccttgc
456721 catctattaa gttagccgca accaaactct aatgtaaatg aaacattcat ataaaatatt
456781 ttctacaata gtgcgaagat atgcatgttt ttgcatgtct gtaatattta ttgtaattca
456841 aatcaacagg aaagcagcct gtgtaaattg atattgagac gggtgggggt gttcaagagc
456901 agcaatgttt aaagagacgg tgccgtaaaa ttgtagttgc gaataaatgt atcagtggac

456961 ttcaagttat ccaccttata attaataatt tcagtagtgt tgggatatgc attgcgtttt
457021 gtaataaccg ttgggctaat attaatcgaa gtactaaaac gccttttata aacgtgttgc
457081 gtcatcgaac ccagcctctt ccgtaaacgt cacactggcg ggaagttgcg gctccgttgt
457141 tccatgcgag acgcataatt tggcccatta ttttgtagtt tagcatgaac acaaaataaa
457201 cgcgtcttcg gaaaatctac cttgggtgct gctatgtcca cgtcatccgt gtcaaaggta
457261 acgtgttcat ggtacttcct tctatttaga tttagtgcta acttgcatgt ttatgttcaa
457321 attgaaataa acaaacggca cacctcaccg agtatacaca ggtagatctc attacaaaaa
457381 ccaaaacata attagcaact catcgggtat tacctgtcac tgaaacacgc ttacaccgat
457441 ttcagggctg ctttgttttc aagccgagtg ccaagaagaa aaggacactg agtttgggta
457501 agttgcgatt atattgacgg gaggatgagt gttttaaagg accctactct atgtatattt
457561 ctgcacagag gatcatttta ctcaaggctg tgaaggggca aaaaacagtg agacgcgatt
457621 taaactgtgt caggatctgt gggacaaaat taaagcagaa acggaggtaa gatcaatctt
457681 atcactgcat gtttttggcc cttctgtggt gttaatgttc tcagcctgag tgtcatctac
457741 ttgtcaaatt caataaaagt taaacctact ggtttgagat atcaaaaacc agccatgaaa
457801 cattcggtag gtttcagatt gcttgaataa caatgggttt tgtatgtaag gttttacagg
457861 atgaactcta cagaaaaatc ttggacagct tgctggactt caccaggaag tgttcttcga
457921 ctcgtcaaca cagcgactgg acctcccaga tgagggccag tgagattccc acagcggctc
457981 tggttctggg tacgctggca gtttgatcta tgcagcgtta tgtttgtgac gcctttggca
458041 tttaagggga ccgtgaatcc tgatctattc tactgttgtg accctcctca ggtgtgaatg
458101 tcccggatca tgacatgact tttcagagtt tgtctgaact cctccagcag tcggtcagtc
458161 cgtttgtggc ctctgtgcag gccaaagagt gtggaggtga gaggtcattt gccttctcag
458221 cgacttctgt gtcgcgcgtc accaacatcc ccacgtacat gtgatcttgt tttagcatta
458281 aagcagttga tgaagagggt cctggaaagg ctaatggaca ctgttgtgac tgtggaggat
458341 gatgatgaag aggaggagga ggctaagcag acttctactc agcaccacgg gagggttcac
458401 tgctccctgg gtgcgctctg tgattggtac agctgccaga caaaggtgag gcggcgtagc
458461 gcggcttgtg ttcgtgcagc cagcagcacc aaaggggtcc gattaccgtc accatggaca
458521 cgttctcaaa ttacagaaat ccagcaacgg cacacctgga aaaaagcgca gctctcctgg

458581 cggagatggt caacagcagc cgccagttgt aattattttc aaggatttgg aagctttcag
458641 tcccaaagtt cttcaagatt tcatactcat ctgcaggtaa acccccagtc atggtttatg
458701 tgcttgtttg gatggatgtt gagaatgtct gttctctact ccacagccgc tacattgagc
458761 gtcttccgct gatgttcatc ttcgggattg ccacctcacc cagcaccatc caacacatgc
458821 tgcctcactc ggtttcctcc ctgttgtgta tagaactctt ccagtccctg acctgcacgc
458881 agcacttggc cacagtgata gacaaggtga tattgcagta gtagaaacta caagtacagg
458941 gtcaggcttg ctgactgaca tgtgggtgtg tttaagacaa aacgtttgaa atatctttca
459001 tttctcctaa aagccagttg cccaggtaac atgttggaca actttttgtt ggtaaagtca
459061 gctggagggg gagagaagtg gtttaatatg tgccttgaat gtttaatcct cacacacttt
459121 tcttttagtt ccaaacttgt ttacctcata ataacttctc attgtgtcct tgaggtccca
459181 aaagcagcaa aacctgcaaa tctcccttaa gaaaaaaacc tcttttcctc ccccagttaa
459241 tcctgacgca tcacttccca tttaagctca atgctaaagt gatgcaggtg ctgatcagca
459301 tcttcctcta ccacgacttc tcagtgagga acttcatcaa gggtgttcag gtacaaagcg
459361 gcttccagat agttttcagc taaatcctgt cggttgtgca caatcacagg ctttttttcct
459421 gtctcaccac agctggccct gctggagcac ttccactctc agccgctcag cgtgctgtgc
459481 tgtgagaagc aggaggctct gcggaacgct acgcagcttg gctgcaaaga cctggagaga
459541 atcaggacct tgccatcatt caagaggtgc ttggcagata atatttattt aatatttact
459601 ctgttgagat ggcattgttt tactgtttca aacctaaaac tagcccttcc aaaacaaatg
459661 ttgtgcatat tttttccagg tatgttgaga agcaggaatc tcaggaacaa gagagtttgt
459721 tgagagatga tgctcacttg aaggtgagca tcatcatcgt ttattttaat aataacctac
459781 aggtgacaga gacataaggg aatgctttct ttctgcctgt ttttaggaag tgtgtcagaa
459841 gcttctcaaa gacctttgta aatatcacaa gaattattat cctaccctga ggtgccttca
459901 cgccctgacg tcttctttac ctcgataccc tctcggaaaa caggcagggt tcaaattgtt
459961 actgcaagaa gtgggttttt tttaacgaaa atgggaactc attcaccaaa taaatggatt
460021 ttgttttgaa caggtcagag agctgcattt aatctgtatg gagaagaatg tgtgggagaa
460081 tgaggagtac cagatggcca tgaagcttct caagtaagtc tgtggattga ttaaaaagaa
460141 tgtaccttaa aacgcctcca gggatgtttg gagacatgct cctcctgctg gtagatgtcg

460201 ctgacgcacc caagacgggt gccggttatg gttttgataa attaccccta aacgagccat
460261 ttgtccattt attctcctca gaatgctggc taaagacgag ctgatggctt cgctgcagaa
460321 gtgtgcgaac agcctggagt cggtcaaatc caaagagatg aagaaagctc gcgttcggct
460381 cagagagctg ctcgccaagt ttcagcagat ggacggtgag acgtgacagg gaaggtcggg
460441 catgttggag ggatgaaccg cagcaggctg aacgttgata agcgatgatt catgtttgct
460501 aagccgccag gatcagctgc tctgtcggtt gttatgaggt ttggtctgaa agggtgtgtg
460561 ggaagtggaa aaacaggtgt tgtgtgtgga atgattttga accattttat caaaaatgat
460621 gtgacagaag ctgatgtttt tttggttttt tttggttttt taatttttaa acttgtttca
460681 atagctccag aagcccccgt ggaagaccca gtcacctctc ctgtgaggga ccttcaaaag
460741 aaaaccgatc tgtttcagct gcaaaaggtc ctttatcatt ccagcgaaga ggtgcaacct
460801 caccgttgcg gtaacagttt ttgtcttcct gcagaccctc ctggagatga acgagtctcg
460861 cagggccaaa aaactgagtc cgtttgagct gctgcgaaat gaagttctgg agtttatcga
460921 tagtttagtg aagtaagtag cttttcattt tcatacacat gagtctgtta aaagcactca
460981 atctaatgcc accccccccc cccaggaatc acctgtcccc ccctgaggct cagacgctgt
461041 atgaggtttg ttactacagc tcgtctgcca ccgtgaggcg ccacctcaac gccacgccgc
461101 gcatgtccat acaggccgcg ctcagcagcc cttactatta cctgcaggta cactcaccat
461161 gggggtgttg gcgtgaagag aagtcccgag tgtcccactg cttttagtct tattaaaatc
461221 ttgtctggtc ctgcagaatg agagcctgaa aacggagggt ggctcagtct ccagtgctgc
461281 tcctgacatc tgcatcgcct acaagctcca cctggagtgc ggcagactca ttaacctcta
461341 cgactggctg gaagtgagtt tacctgatgt ttgacaatat tttgaactcc tgcctctctt
461401 gcacgggggg gttttatcga gcaaggaggt tcaatagatc gccgttatca gagtggaccc
461461 ggagctgacg tgtttgtttt caggcctacg ctactgtggt gacatccgca gagggcgccg
461521 acgcggacgg tttgggccaa gtggatgaaa tcaagcagta tcctttctca gcttttctgc
461581 catcgaacca aagaaacaac ccacttgaga gactggagtt cctcaactcc cgctgcagcg
461641 ctcgattcat ccgggccgtc tcagaactcg agtttctggg cttcgtcaag tccacaaagc
461701 agaaaaccga ccacgtggcc cgactcacct ggggaggctg ctgacagtcc agatccatcc
461761 agcaatggct gcgcttcagt taatgaaccg ttaatgaaat atgcgctgag gtcatgttta

461821 atgtccacag cggacaaacg tggaatttat tcagaagagg caaatatgca tcgacagact
461881 ttctggcagg aaacacacaa ccacacatca actgagattt ttattctttt gagcaataag
461941 tacatttgtt ttaaagtgaa acaaaaatta aaataaataa aacactggag aggaggggaa
462001 aacaaaagta aaaggctcat taaacgctca atttaaaagc aatacatgat ttttctctac
462061 caagcctcag ttctttaatg tcctctcaga ttgaccagca aacttaaacc tttcaaccct
462121 tttatattta caaataacaa aaaataaact gacggagctt ggcgtggtgg gattcattac
462181 aaaactgaga taactgtaga tatacgtaca gcaggaggaa cataggccac tggtagagat
462241 atggtccatt aatttacatc aggaagcacc tatttgctta caagagagtg gtaacaactg
462301 acaacagcat acaaaccctt gcccaaagac aaaaatggca cagttaaagt ttcaacaagc
462361 taaactgaat ctcttacgac gatgactttg tttttatgcc ctcgagacgc cgcgcaatcg
462421 tgagcaggag gaggccttct tgcagagatt tcagtgccac actcaggaaa catctggaga
462481 aaaggaaggc agaaaaagcc acctcaagat ccatcactaa tgtggaagta tgttcaattt
462541 tagaagaagt cttacagctt gcaggttgct ccccaaatcg ccgcattaac tgatcgtggg
462601 tgaacttgac aaaggtgtcg tattgttctg gagttgaaag gaaacctgga tttattcatg
462661 cttttggtac aggacactta taatacattc aatcgaagca gtggattgtt ggtccacaga
462721 ctacctgtca atatccatgc tccactgtgt accaacctgc cagttttgaa gtcatggttt
462781 cctcgtattc ctctcgcacc ttctcttccc tttctttgag cagacgctca caaataattc
462841 caacttgtct gagggtgaat aatggctgtt cttttctagt gggtgaaact cctccagagg
462901 atgctcctgt gcacaaaaga aacaaggacg gaacaatgat atcacagcta caaggtggca
462961 atgactaaag tcctgggaca ctttttggtg tgagggaacc aaccggacat gcttgaaata
463021 tgcatactag acgactggga tggggactct ggggaatagc tgcagtctga ctggaggtac
463081 cctccatcta gatgcttcct cttttgaatg cgtttgtact cttgcttgat gctttgaagg
463141 atttgttctg gtggaacaaa agagaattta gactggatca actttagcac gcatttctcc
463201 tttgcaatgt gacaacatgt tgctaatggc tgttaccaaa gcaggccgaa tttgtgacat
463261 tagtagcatt ttacccgcac taatggtgga cgagaattcg ccgaatggtg atggttccat
463321 gcgaagatat ttccgaggag atggggacga agtggacacg gggacgcatc ttcgtctttt
463381 cggggaagta ggactcatca gcggatcgaa atccatagtc cgcttcaggg tggcaccaca

463441 cgccatgact tttttttttg gtttaaaata aaagatgcag tataccgaaa caagaaatga
463501 atcaagttaa ttgtcggtta tttgacaaaa ctgttaaagg tgattctttg tttctaatgg
463561 tcggagcgtg aaaggttcgg agaaagccgg ttaccaccgc tgctgagcat actacgcaac
463621 tgaggcttta aatcaatact ggaaagctaa tgttagccat gcttgctagt gagaattaaa
463681 aaggaaaata ccgctaacgt tagctttagc taccgtggtg gctccggtgg tataagacag
463741 ctgtttctca ccttatacag gattgccgac gtgtccgatt ttgtcaaatt tgtgtttcta
463801 agaagtttct tctccaaact tgatgagtgg gccaagtgtc tactcgcaga tagagttagc
463861 aactgtaacc tagctgcttg ttattgatac aagttgacgg tttttcctgt tcctttacgt
463921 cacatgagat aatcgcaagg cattctgggc tatgtagtag ttccgagact atagtggata
463981 gtacactagt ggatatacac taaatacaga tttacgatgt ccgtatcaat cagctaaaaa
464041 tgagtttcac gtctttgtct caatacaatt ttcgatgtca aacttaatta tgtcatttag
464101 aattggttcc atgatatcca caagagggca gtgttaccca aaattaatgt atttgctcga
464161 tcaactatac agtacatcga tgcggtaacc tgatggtgga ttctgttgac attgataaaa
464221 actaacaaat ttcatgtgtt tattcaatca cagagtggga catttgatgc atcacatttg
464281 ttgttacatt ctcatattac caaaaactca agattttatt tgatcaataa gtcattcata
464341 attcaaagta gtggttcacc attttgaaaa cactcacatt agaaaataca atgaatagag
464401 tgaaataaca caaaaacagt caattctgtt ctcacaaatg agtaaataat tgattgatga
464461 tgcttcttta aagcagtgtg ttgaccatga aattacaggc agtttgatgt tggaatgcaa
464521 tcacaatgtt ttcatgagcc aaaaataaat gaaaaggaca aaaacccttt tagtactgag
464581 gtctgcaaaa ttatgtgcac catcttgaca agaaacaagg caggacatag gcttcaataa
464641 gaaaactgga gttcgtaata taattaaatt ggtatagtca ttctgtgcaa ataagacaca
464701 attttaagag atgaaacaga accatgatta cactccacca tgcagtcaga tacagttaga
464761 tgacaaatgt atgattcaca acaccaaaac cttgtttttc agctcgttcg ctcggtcaca
464821 tttggcacct ctcacagtaa tagagcgctc agccgatcag ggctgtttga cagtatcttt
464881 agagttaaat cagatcaggg cagctgaccc ttcacaccgc ctctgcagga gaagagtcag
464941 tcactccaga aacagtaacc tgggctactt ttacttgagg ctttccaccg acagatcggt
465001 ctttccccgc cctgcctcct gtgctcctca cgctcctgct gttccagtcg ctctcagcac

465061 tgctgtccag agactgcgtt gttccccgac acatgtggca gatgttgacg aaggccctgc
465121 gcaggtcttt gctcctcatg gcgtagatga cggggttgat ggtggagttg agcaggcaca
465181 gcatgctgca aaaggcgaac acagtcttga tgaagtcgtt caccctccca aagaggtcat
465241 agaccatgat ggctagcaga gggccccagc agatgatgag ggccaccagg atcagcacca
465301 gggttttagc cagacgcagg tccattcgag cctgctcggg cctcacagtc tgcactttag
465361 tcccctccga ggtgtagacg atgatgctcc tctgagagct gcggctcagc atgcggacag
465421 cgtggtggtg ggatttccag aggatgaaca tgtaggcgta gataatgaac agcagcaaaa
465481 cggtcgtcat gccgatccag aacattaggt acttctcgtc aattagggga aatatgtctg
465541 agcagacgga gtggaggcgc ttgcagttcc accccagcag cggcagcaat gagaacgcga
465601 tggagatggc ccacatcacg ctgaaggcga tgaccgcttt ggtcttggtg atgatgcgct
465661 tgtacgccat gggcctgtgg atggacacgt agcggtcgat ggcagtgaga aacaggctgc
465721 ccacggaggc cgtgaaggag gcgatgaccc ctgccagttt gaagaggaaa atactggggc
465781 tgtctttcct gtggaggacg tggaagtcca ggaagctgta cacgaatatg atgctgccga
465841 tcaggtcggc cacggccagg ctgccaatga agtggtaaga ggggcgggat cgaagggtgt
465901 gcgagtggag gatcacgcac agcaccacaa agttctcgag caccgtaaat gtcccgagcg
465961 tgatggccag gatgacaatc acaagctgct gtgcaggggt aaggatcatg aaacactcca
466021 tgttgtccac gatgtcctcc ccacactggg tggcctctcc gctgccgtta ctcagtggaa
466081 agtctgacgc attggtgggt aaaatggtgg ataggccgcc gtagatcagc tctttatttc
466141 cagggatgag tctgtggagg gagctgctcg ctgagtaagg tttctcgaag ttgaacctgc
466201 tcttcatcag agctgcgtcc gcctggggat cctcgtagct ggcgtcgttg gagcccaggt
466261 attgcacttc tgtcgtcagc gccgccatgg tggcgcctgc tatcctgtgc agagccaact
466321 tcatggctgt ctgtatggag gttctacgca gaggtgaaac agcatcagac ccttgccaaa
466381 gggcccctgc gaacacacaa cggggtaaag aacacatgtt aacacaaatc attcatatct
466441 acgcatcaag cagcatcgat tattagtgat taatagggtg gaaaatgaat ttgttgctgg
466501 gagatgctgg tgagatccgt gcatcagcca gctgacatga gcagatgcat cccagacaat
466561 agtgggaagc caaacagttg atgacaaacc tgtgcagcct caggggaaat cattgcatta
466621 agtatgtagg acagacagcc tatcttatta attaaaggag atcattaagc attaagccga

466681 gttttcctca ataatcattg gattaattag gcatcaaggg agaggctgct tgtctaatga
466741 tcatgtcttt cttgcttaaa atgacatatt caatattcct caaagtaaat ggtcgataag
466801 atatagtttg aaaagaccag ggaacaagaa aacttaatat ttgggttaaa tactgattta
466861 gaaaataaac atcaggaaga ttaatcgaat taatagactt aataaataaa cccacctcaa
466921 tcttctttgc gcaatcatcg ctcctcgatc catgtcgaca aatattgaag tgatgatatt
466981 aatgaattta gggaacaaca gcttgttgtt gttttgaccc tcttaagtaa ctccgcgtaa
467041 aagaggcaaa aactaatgta aaataatcat caatgctgat gtttttttta atttcccaaa
467101 cgtctgcggg agacacagaa taaagtccga cacgcagccg ccggagagcg cgtgtttgtg
467161 cgggcgtgcg gcggctctca cgcagctttt tgtgcgctgc tgtcacgtgg ttggaggtgt
467221 tggaggcgca tccatccaca cctatttata ctgaagcttc tgctgctgct gctgcaggca
467281 gcgtattaca aaaggcagca gcagtgcccg aactagtcat aaaaagcaaa tcttgatctt
467341 acaatagaag caatcaacac agatcagggc tgtttttcat gacaaagctg ttgattacac
467401 tcaacacacc ttacatctgt cacctcatta cactgagaca gtagcaagat agtttactgc
467461 tcagggatga actggatcgg aaacactatc aagcttatgc atcagtgaca cataacggtg
467521 ataaaatatg aactgaacct ttgtttattg tatgtttatt aggatgttgc actgcactgc
467581 aggtttgcat tgtaataatg aaggttctct acaatccgaa tctgctgttt cttcttcggg
467641 gtctgtgcac ttattcaagc aattcaagaa agtaaattca agatcgtttg tctgtaaatg
467701 ttctgacact gtatttctgt tgctattttt aattcaactg attcttcttt ttggtgtttg
467761 ctaactgctg tttgtggtgg ttgtgcgcgc acacacacac atgcacacac aaacaccaca
467821 caaacacaca cacacacaca cacacactgc acagggtcct ttttcctgtc ccgtgcagct
467881 gcaccattat ttccagtatg cactcccagc agagccatct cccaaattac atgtatctcg
467941 ctgtggaaaa tgcatgacat ttggcgatgt agcatccaga ggaaacaatg aaaaggcatg
468001 tgtgatgccc ttctcccccc agttgtggcg agaacagaag agggggctga agtcgtccaa
468061 cttcttgcgc gtgcgttcat ttgcagaggc gttagtaact tgtgggggta aatatcctca
468121 gccatttaac actaatcgca tcggttaatg tctttttaac gactgtggat tctggaattc
468181 caaaactgaa gtctgccctg tcactctttc tgttttcctt ctgatcagaa tcgggcgtca
468241 ctgcgactct ggcttgacag cagcatccac catccaagtc atccgccgtc tatatccatc

468301 ataatattca tcatcaatat ttcagtgttt ttcttaccaa agcgtcagtg agggagacaa
468361 agctgtccgc catgacaaaa gacatccata cagctgtaat ggcgacctgt caattcctct
468421 taatcctctt tcagcatgtg ggttgtgcag ttatataggc ggctgttgtt tcacatccat
468481 ccgtcattgg tggggaatat tgccagcgat ttgcagtgag tgatcttttg caacagtccc
468541 tctgaaaaga gccataatgc agctccgagc tcatttaata ttgatccttc accaattacg
468601 gtccattgct atttgagctg ccttgctgaa atgatttctt tttggcttct gcagacaaca
468661 tcacaaaatg caggaccatg ttagagaaac ggggcttttg caagagaaaa gtggctcatg
468721 tcgagtgtca cttggggcca aaaaacccct aacgagtctc ctgcatacgt gttctcttca
468781 tcaagtatct tttttaggca cctcagcgct atggtccttc tgtgcctcat tatggcccgc
468841 attaattaga agttacaacc tggcttcatc aagaatgcac catccagcac gagttcacat
468901 gtgggcagca gcttctgaaa aagaaacagg gggaagtaaa gtgccaaaca gttttacagg
468961 gtagatctac cacgtagggg gagttaaggc acagcagaga ggctccaaag gtgtacaaca
469021 gtgccttgaa atggaacagt catcaatcaa ataccagcat ctccccccaa aatgccgaat
469081 gtacatatta aaggctatct aatcgctagg atgtagccgt ggagcggcga atgcacacgt
469141 gtgcaaacag atgcagctga atgctacagg cacggattat gacgaggttg aaatttccag
469201 tgggttcaca attcgtggct ttcaggtgat gcagctgtag tcctagagcc tcagagcaga
469261 cactgaaaac cagaggggag ctgacatgga atcacctttg agtgtctttt ctaaatccag
469321 atccaaatat taatccattt gcagtacaca caagggctaa cgagtggccc aaaatggacc
469381 tgactgccat gagaccagca gtctcctata atcacatggc tcttttctgt cagaactaag
469441 tggggctgtg aaattagaat tatttttagc acatctctgc cttattgtgc cagcattcag
469501 cgtgcgctgc actttaatgc agatcatctg gatcatgctc ccacctgaag caacctgtct
469561 tcaaggattt caacattggc tgcccatctt gtcatccaga cagcaaacta tgctgtgttt
469621 cattcagcgc tctgcagatg catcaaccgc cggcgttctt tctcctgatt acaaagtaaa
469681 tcaactgtaa ttaagacagt taaattgttg ctaattaaat tgcctatgca caaacctgaa
469741 acctttaaca tccgctagtc acgcagaagt gatttggttt tttttccttt tttgtgactc
469801 aaagaactcc aagagcggtg tgtatttgca aacctgtgtg ctcgtcgcag ctttcgctca
469861 tgggcagcac agcacttatc cttctcattt ttctcaccac attggttatt cgccctttat

469921 gagcactcag ttaacagaat gctctctcat ctccatcact gatgagatgg cattaaagta
470041 aaaaagctga catcttttca ttcgtattac ttcttttctt ctgcaaacaa ctgagctgag
470041 agcggattgt ccatcagtct ggatgcaatt tcagtaagca gcgaggagca cttcaactta
470101 gatatccttg atggatgtcc gccagggttt taccatcaat tccacaccaa gcattagtgc
470161 tgatgatagt gggatgtgtg tgacctcatc aggctgaaga gaaacaaacc aaaatggaat
470221 aattaaaagg gaaaaagccc ttttgatggt gaaaaagctt tcaggagtgg ctctcctacc
470281 ttttgttgtc atttttcctt attgtgcatt aattgagaac gtaacaaaag tccaacatgt
470341 ggtatttggc gccacctagc ggtgacacaa cagagtgctg aaatatagat attgtatatt
470401 gtataaatat tgtatatagt aacctagcat tttagaactc tttaccaaat tcctacctac
470461 taatgtttga cctgaacgtt tgtgtctata tttttatat gtctgtattt atttgagatc
470521 agatgagccg gcgtagacat aatatcaaat gtgagtgggc tcagagggcc ctgaggttaa
470581 gattactgta tactatgtcc ctattaatgc aaggtcaaag gcatatatgt ggccagtggc
470641 atttaaaata tcaatggtat tgtggaatta cattattcca ttgttatatt ggagagaatg
470701 gacagggagg ttattgttgt ttttctctga aagttaaaat aattccacaa tcatgctgcg
470761 tgctctaatc aacacaaaca ccgttccact cttcatcttc cgtcagggag aaatgacaat
470821 gcggcacctg tgtgcatcgg tactttcagt aagaagccca tttagaaatt accagcacat
470881 aatgagacga gatttgatga ttatccgcgt catcggtcct cctctgcagc tctcatgacc
470941 acctgggctg gaacagaccc atgaaacgca ttagcctgca gccatcatgc cggtctaaat
471001 aatccaataa acatttgttt ggctaaaaaa gagaaaagta aatactgcat aattgatcgg
471061 tcaacctttg acagcaggag cttacagatt tttttttaaa aaccatcgat gccctgcagc
471121 aaactaaatt atcgtccttt tttcctcact caaaacctga ggggaccctc tgtcggtgct
471181 agcgtttcag gcacatcgcc atggagactg ctgcactcac tgtaacacaa ccaggtcagc
471241 tggtgtaaac ggctatactc cggcatgggt gtgtgtgtgt gtgtgtgtgt gtgtgtgtgt
471301 gtgtgtgtgt gtgtgtgtgt gtgtgtgtgt gtgtttaaag gatgatggtg taaaaggagc
471361 tgattcatgt gtatttcatt gctagttact gtttgggctt atcaggagag cttttctcta
471421 acttttcttt tcttttacct ttggtatttg cttgaaacac gtcacagtga agtttcactg
471481 tggagaagag accggatttg agctgttttt tctctgcttt ttgacttttt aaactgttct

471541 gaacacaagc aaagaggcac cgagttccat taaatgtctg tgaacatctc aaccacacag
471601 caaacgtgtg gttgatgctc ctccccacaa aggagcggtt tacatcctgg gaggctccca
471661 gtctgctctc cctccaaacc catctatcac tgaggtctgg tgagacccta acactgcagg
471721 attgggggtc cagtcttatt acccacaatt gggacacaaa tttgtgatcc agcgggcaca
471781 acagggagca ggtggatgaa cgggtgtacc gtaaacggac cagcttggac tgggatcccc
471841 ctttgatacc tgaagacaga caagaaggaa agaagaagaa gatggagcac aatttgttaa
471901 tttgtataaa taatcagttt acttccttta atttgaggtt tatgtacagc ttttctctcc
471961 ggattccagc cgacagcatc ttagtctcat tgattatcac cagtttatca ttaatcactg
472021 gtcacaacgc caaggctgcc tgtcagaagg aacctttag tcccgttgga acagcaggtg
472081 ccctggtttg cctccgccac acctcagaaa atgactgatg tgaggagtcc tgtcagattc
472141 ggctcagcgg ttttccgcct ctaacccccc agactctcgg ctgcatgttc atggatgagg
472201 tcataggccg cgaccgacag agtcaggact ttattgttga ggtttgattg attaatgacg
472261 tgtcacgcgg caccacctgt ttctgtggaa aaattatgca cattatctgg atttttctcc
472321 tgacgatctt tgaacgcatc catgtcagaa tttgttctca agtgtgtgtg tgtgtgtgtg
472381 tgtgtgtgtg ttttagagaa agagagagag acagagagac acaaagagag aaagtattaa
472441 aaggtctgca gcatcagagg ttgcctctgt gttatgtttg tgatgtatga gctctggggg
472501 ttcaggagaa acctaatata agagttccat tgtttttatt taaagccatt aagataaaat
472561 gaattcgaat gacttttaat gagataaaac attaaattga taaattacca tcctgctgta
472621 aaggtgtcat tacagtccaa acagccacct ccgctggccc gttccccata tctggagacc
472681 ctctctggat gtaatgacac tgaattctat tcactccttt attgcaggtg caagaaaaac
472741 atgagggaca agagaaaagg gagtagcaat gctgggaaag ggcattgtta gctaaaaaca
472801 acatttttat gtcatccctg tcctttctgt atgtttcttt tttatttgtt gacctctctt
472861 tttttttttt ttttaccagc atcaaatgag aatcgtgaaa gagagtccag acaaagtgtc
472921 tttcagagaa ggactggagc agtgagatct attcaatcag aaaggttcat ttcacagagc
472981 gcattaatgt ggcgtctgaa caataacaat gtgtcattct cagcacaata ataaccaata
473041 gcttctcttc tggggacacg ttggcccttt aaactcattt acatttgtaa aattgttcta
473101 ttacattata aataatgtaa aatgctgtag gtgtcgaggg aatctgagca tgttatgatc

473161 aaaaatgcac cacttagatg agttacaagt tgttctgtga agttcccatc catccagaac
473221 cgtgcgaatt tggccaactg ttacagtgtc agcgtgttac acagatggat ggacgagaag
473281 atgcaacagc acatgaaaac agcaccaggg cttcaccagg gctttggaat ttagtgcagt
473341 cacccgagcc ttgttgccag ggatgcattt aatttctcct tttgccaact gttccccacc
473401 tttgcacatt accagtaatt attagtcatt tcttacatta ttatctcata agtggagctg
473461 tggcctcaca gccagaaggt tcagacctcc tgggtggagg gaaggtcccg cacgctggtc
473521 gataaacaga gacgagagtc tcggctggac attattaaat cggaaacaat tttttttccc
473581 agacctgttt agtgggaggt atttttcaca gcggtctttt aatctgacag tgctgcttcc
473641 tcttttattt ggcaccctgt ggtgaacaat cctgtctcca cgtcctgtat aatgactgtc
473701 tgattgttta agaccacggt ggtaatcaaa cggctgacga gtgcttcagc tgcagctaga
473761 aaaaaaacac tttctatgag attgttttgc atggaccttc tgcccttttc agcagaagtt
473821 gatttattac cactttaaag tgtttgtgta tgactcactc tgcaaaacaa cccagctcca
473881 ctggtgtcaa gtcctcgtca tgaagacatt ttggagaatg taatgtctga ttcacgccac
473941 agttcagaag gtggcagcaa tgccatctgc ccgataaaaa aacaactcct gctttctttg
474001 caggtttctc tccttctgcc agtcttctct ctgccagcgg tcgataactt atcataactg
474061 ccgcttggac gttctttctg ggggtccgtg ttcccgtact ttattggaag gtgcactttt
474121 aaacaacgga atgagatcag atcacatcaa aatcagttag cttcagggcc aatcgctttt
474181 cactcatcgg tttctcaagt ctcaagttgc ggccatgtga gctaaaaaac aaaaaagatt
474241 tctgaattgt gctcaggcac accagggtta gtttgttccc catcaaacag gtcatcagta
474301 catggaaaag aatagattaa cttcttgtga cagtatcttt tacaagatct aattaaccca
474361 aagcaatttc aggggtacac gttttatctc tcaactgttg cgcgtgcaca tttcactcct
474421 cagctccaga aatatgaact ccgtgggccc cgcggcagat attagaagca aagctcaatc
474481 aaatcgactg aagttgatta aattttccta tgatagacac actctaatat gaatcactcc
474541 actgtgccgg ggcagtggag tttgatagat gagattcagg tcaataaaag aacacaatga
474601 ggggcggtga ggatggaagc ttcattttac agcgagttca gtctggaata ttaatcctgt
474661 ctttgctgac atgatattaa aatgaaaatc aatcatggga ctagtttagc ctttttggta
474721 atgtgctgct tctcctctca gcgtgcagtc aaatgaacag agcgatacct ggacgctcgc

474781 gcaaatgcaa cgaccacgcg ggcgaagact ttgtgtcctc cacgcttccc
aatgcctgaa
474841 ggaaacaaag ggaaagatgt tgtttggaat tcacacacag tctctcagct
tcatgttcat
474901 ggatgaggtc ataggccagg acgaagatcc tgccccctcc caacaggaag
aaaccttgaa
474961 cagggccttg atcacacctc ctgctgataa acctccaggt acatgaggag
aagctgaggt
475021 atgaaagggt aggatagaaa gagaatacat acatacacac attatatatg
caatggtatt
475081 ctatgtaaat tcaacaatct atgtgttaag caaagttttg attgtgtagt
tgtagcagtt
475141 cttgaactat atctgttgtc caatgacgta gaaacgtcac cttgacgtat
atttaactca
475201 tcaatacgct tttattgtca aaatgatgct tcatgttaat caaattgtct
gttgtacata
475261 aatccagctg tgctgttgct gcttttatta ttatttctgt catggttaca
tattattcaa
475321 gcccaaaagg tgacaggact ctctgtggaa tataaaacag gccagcgaga
acagtgaaaa
475381 tcgttttagc agataaacac actggacaag gaaaatgtcg acgagttcct
gtgggagagc
475441 ttccaattaa cactcgagct ttctctccga cttggcagct ggctccagaa
agaaagacgg
475501 agacggagag agtgcacagg caaacaccag gaggagaatg catttttctc
tgaaggtgag
475561 atgaaaatat gtctgctcaa atgcatcaaa ggcgccgaca gaattctttt
cattgtcgat
475621 gaaaagctgt gtggagacag atttacccag cctttcttta ttttttacac
atcttatttc
475681 tcacgttctt gatggattgc tctgggttac tcgggttttc agcttttcac
tagactacct
475741 tgaaccagca tgctcttagc ctgggtagga aaaacagttc agcgggtgta
aatgcgacaa
475801 agaaatgtag ccaccttgag gactatttta gagcagagca gaagcgccct
taaaatgaga
475861 ggttgggagt gtgacatgaa gcctgggttg actcacgaag gggcgcacca
aagatggaac
475921 agaagcccgt ggaactggac gaaaaggtca acgctgatat gatgttgatg
ctgcacaaac
475981 atatggctcc tcctggattt gatggcagct gccagcatta gcgtccctct
attcagtgct
476041 tccgctccaa ttaaggcaat tggcccatgt tatggaaacc gcagtatctc
gctccttctc
476101 ccataaatgg cagataaatt acattttgag catagcagta aagactgatt
aaaatgattt
476161 ttcctcatgc acaactcccc acgtagactt gtatgttttg atggattctt
gcatttctga
476221 tgtgtatttc atttaacaga agacagcagt attaacccca ccatctttgt
ttttcatccc
476281 atcgcacagc aggagataaa tgcctctgag ccggcatgaa aaagcaacaa
ctgtaggttt
476341 ggggacagca aaatagttgc ccggttgctt tagtatttta tcaccacact
gaacccattt

476401 taaaatctga ctgtgctttt tctcatcgtc agtcacgctt tatttacatg atgtctgctt
476461 gtggcattta gcagcgctgg agatcctgtg aggagaaaag ggcgaggttt gagccctgct
476521 ggggggcagg acaccaaaat taatgttgta gcaggaaggc agcaaatcca tttcgagagc
476581 tggaaactca atcgcaattg tgacacacac gagtcctatg aaagcaggct gagcagctca
476641 ctagagggtt tgaacaagtc tctgactaga tgagaattgg gtttactaaa gatgcacggt
476701 gagcagcttt tgctccatgt aacacaaatg aggaattcat tcccaccctg agcccagcta
476761 cctatttcac gtctgcactt cagctccacc ctgctgacgg tttgagctat ttgaactccc
476821 accggccaca gaaggctgac gttattgata aagacagcag cctttccagc tctgtcaaac
476881 acagaaaggc cacgactacg gcagagctgc ctctgagggc aaatgtctgg aaagacgagg
476941 aaatatcctg cacgctgcac ctcgacgcga agccaggcaa acgggcctgg tttatgagaa
477001 acccctccct ctgaactgac aacagagctt tctcctctca tatctttcat tttctttctg
477061 taataatgca agccatcgag ttttccttca agccattaca aacaaataat gtttgatgca
477121 aactgaaatg gaaaaactct tccactgcct gtagtctagg attttatatc tgcgagctga
477181 tgctaagaaa tcaatgggca gtgagtaaaa gaagttaatc tttttgttgc tgaaacacac
477241 acacacacac acacacacac tgtgtataaa gtatttcttc ctttgtgggg aactgaccca
477301 gctcaacctt ttgtgttcac tgttttaaag ttgtgttttt caaagtttcc tcacaatgct
477361 agtggagtta atattttggt aaatgtagca atatgttgca aatacaagga gacacatgca
477421 cacacacaca cacacacaca cacacacaca caaacagaca cgtttgaacc cctcctgtgg
477481 ggcagagtgg cttgatcatc tgaagtgtta atatattcca tcttgaagac cgcagacaca
477541 tcgtgtgcac tgatgtgaca agacaggctg cagcaggact gtgaagttgc acattcaccc
477601 acacgctcac atctcagagg cccgcgggtg acattctgca acgaccaact gcatcgcagt
477661 gcgctgccaa aacacgcctg ggaggctcct ctgagggcga tgggtttgtt cagtagcgat
477721 gaggacaaag caaacgtgac tgacacattg tccccctctc tctgtcactt ggagcctcat
477781 gtttaagaat taggctcaca gcttcgacag gcagtgcttc aagtgtatat cagataaaac
477841 tcattttcct gtgtgtcatc ctcagacaat aaagttgaat tttctcatcc gtgacccatg
477901 ggtaacctgc tccgtgagga agcagcccgc agtgtgtatg tgcaaatcct acacttcaga
477961 tttctgtgtg aagtggttaa attttgatgt aacatcatgc ttggaaaata aaaaatattc

478021 cccctgcact ccagaagaag acctcccaat agcactatta tttaatattt
ctagctattt
478081 ttctcatttt ggacaaggtg tgccagcaca tttcagatga gtttttttaa
gcttgtcctg
478141 cacaaattgg tgctattgga aggtgttaca catcttaatg tgtttcacag
catgttcgga
478201 gctccaccga agctgtctcg gcagccttca tcctgctgtt gtgacacctg
cctgcatcta
478261 tcagaagaac tctttcacca cagttagtgg tgatgagtaa tggctcaacc
ctaaaatatt
478321 cttcttactg cgcaaatgtt gtcatgtgtc attggaggac ggaggagttt
gatgtgtttt
478381 gctagatgga atctattagt gggtcagttg tgtttcttcc tttcctgagg
tgattttaat
478441 gcatcacaat atcaccacaa ttttacacca atgtttacac acccacgcac
acacgcacac
478501 aaacacaaac aaacaaaaag gatctttctt tttggaaaca atgcatgagg
ccaaatctgc
478561 ctgtaatcat gtgaaatgaa acacatgctc acaggggcaa tccctgagtg
acacgtcgct
478621 gccacctttc catgtcattt aattgatgca caatagagag tgtcagcatc
ttcggcggtg
478681 cgtaaagtaa gctgacagac aggccatgac aggaggctgt catggtgtca
gcctgctgag
478741 cgctgttcat cgtgtcccct cctgtgattg gcaacgaccc ccaattaacc
tacaaaaaaa
478801 agcggttaag aagaaccccc caaccccccc ccacacacac acacacacac
acacacacgt
478861 acacatagag ccagacacga atgcatgaca gatgatgagc ttcatgttaa
ttcagtacta
478921 aatgaccttg ttttcagagc agaaagtgga tgaaggtgaa gcactgttta
catatgggac
478981 acaaggaaag gaacatgcag taaattaata atgtagacat gttttttttt
taccgacttg
479041 aatcttttta ttagcattcc aggtttaatc gagtaatctg tgggtgcaca
tcttttccaa
479101 agtgtgtccc tctgggtccc tctagtgtag tatcaggcaa atagaagctg
ccatgtctct
479161 gttctcaata tttactgtga ttaaaacaag cacaagaatg ataatcttat
ggtttttagt
479221 tggtcttgac aatagtgcta taaatgtcat tctcccaaat aacaggcatt
ggtaggtatt
479281 ccatgtactt tggaatgctt ttgatatact aaagattagt ggttataagc
tgcagacata
479341 atgcagctgc agagtttcat ttatcttcgg acagcctagt ttgtcctaat
gcagtcgtac
479401 acaaccccag ccagtacagg ctcttataaa taaagaatta ttaaaagcac
atatgaattt
479461 ttcatttaca tgccatcaaa ggaaggatac atatgttgca gtaataagct
agttttatca
479521 catttcctcc ccattttcaa taaactgaag cagaaccagt agggaactgc
agcttttata
479581 ttaaaggaac agtcattcaa cagtctttat ttgctgaacc ttacagctga
accttttggt

479641 ttaatgcaca ttcaacaaaa cattcaaaat aaaatgtata gttttattca tcagctaaaa
479701 gaggagcaag gtgagtcaca taaatgcaat gcaaatgtgc ttaaccaatt aggaatagtt
479761 ttaacaaaaa aataaaaata aaataaaaat aaatctatga gattgtgatt gatcataagg
479821 acaatagttg ttataatggt tgttattagg tgatatgagt gtaactaact tagagtcttt
479881 aaacttttat aaaataaatt gttttgttaa acctgatcaa gataatgacc accggaagtg
479941 tatgtatgat gcaaacgaaa gaactaaaat aaaataatca aatttatcct gtacattaaa
480001 aaaatctttt cccgtattca taaaatactc aaccaactac ttataattat tatgtttctg
480061 tgattcataa aaattcaatt atacaaaatt ttattttgta tttaccgcta tttcctgcag
480121 gtgggggcgt ctccagagca cattccaccg gaagttgctg tcagtgtgtt ttgttttttt
480181 taattgagat tgaatttaaa atatttttct gtcccccccc ctcccagaaa agattctggc
480241 acttaataac attgtagttt gcaattttgt tcagattttt cggcgtctcc ggttgtagtt
480301 tccaaatgga gaaacagtta aaagactccg tccgacgctg catcgacgcg cgtaaagttg
480361 acttgcacaa tttaagccgg gacatctgga gccgacctga actcggcgga gcggagacgt
480421 gcgcgcacga caggctggtc gacctcttct cccaggagga gtcatggacg gtggaaccac
480481 actacacgct gccgaccgcg ttccgggcca cttggggtcc tgtctgcggc ggggagggta
480541 acccggtggt gaatgtggcg ctcctgtgtg agtatgacgc gctgccgggc atcgggcacg
480601 cctgcgggca caacttaata gcagaggttg gagctgccgc ggctctggga ctgaaagctg
480661 cattggaaca gaccgaaccc cctttggcag tgaaggtacg agttaatgtc acaacaccag
480721 agcggaaatg ccacaatttt tagataattg gagcaacttc cataggtctc attaatgaac
480781 aggtcaccta aaacctagat tgatataacc aataaaccac caataacacg tggtttaata
480841 ctaaaaaccg atttagatgt tgcaaacatc gaaataaatg aattataatg ccaaatccat
480901 cttctaccgt ccttcattga gtgggagatc gcttaagtgc caactgagtt tcatactcac
480961 atcttccttt gtgttaatct gcttcttaca agctgtaacc ctgacatgaa ttgaaaatgt
481021 ctacactagc tcactgttct gggaacgcct gcagaagagt ctgatggagg aaagattgac
481081 ctgattcagg caggagcctt tgctgacgtt gacctggcgt taatggctca cccagctcag
481141 aaagacgctg cgctgcagcc caccccttgca gtccttgagt gagtttgcgt cgcatgtcct
481201 caaacacatt actgatccca gatcaccctt aacatcttgt tatcaataca cactaggata

481261 tctgtgaagt accgtgggaa ggcatgccat gcctctgcat acccttggaa gggagtgaat
481321 gccctggatg ctgctgtact ggcctacaat aacatatctg ttctcagaca gcagctcaga
481381 ccagactgga ggattcatgg tcagtttaag gggaaaaaaa tctattgaag gagaataata
481441 gttcagattt ctcacctcaa tagcccatct cattcagacc tgatgaggag atgtgttcat
481501 ctttttgcac tgacaagatt ccatttacat gtggatttat gtcatcttaa caaatgcaaa
481561 caataaaagc atcaagcaat aaaaagggta agttggtgtg tatagctact ggataaagtg
481621 ctaaagattc ctcacaatat ggaacataaa catcatcatt tgcgttttct atatattgta
481681 gccataaaaa aaggtcatga acggttcaga aaaacatatt catgcatttc tatgtaattt
481741 tgccatctct taacagcatc tgccacttat gctttaatta tacatgtgca atatgcgaaa
481801 aatattttaa ccacgggtgg tgttttatgg ctcttttcac ctgtagagtt ttttttattt
481861 ccacttcagg catcatcaaa catggaggag agaggtctaa cgtcatcccc gcctactctg
481921 agctgaacta ttgcgtacgt acgacctggg ttaaggaact gcctgaacta aaagccaaag
481981 tcgaggcttg ttttcgggca gctgccgaag ccaccggctg ccaggtaacc tcacctgctc
482041 ctgtcgtccc tttgtttccc cttacagggt gaacatacat tatttgtaat aatatatgca
482101 aggttgccct tttaacgtat tctgatgtcg tgtattgttg ttacattatt cactgctttt
482161 tttacaggct gttttgtttt ttcctttagc actattcatt cacgccgcag ggtttttata
482221 ctgcgtcata aattattcat atgttacatc tgtttcagat ttgggctgca tttataaagt
482281 tttagattga ggcagcattt atgtttcata taggtggaaa taagttaccc aggccatatc
482341 tactatgaca tcctgcctaa ctcgactctg gaaggcttat ataagaataa tggaaaagct
482401 ttggggattc agtttccaga gcaaccaggc aaattctccg gtatgtgcaa cccactgtca
482461 tcttactaaa gcatcaaaga gatgttatca cagagatttt gaaatggtct ggcagggtct
482521 acagactttg gcaatgtatc atacatcatc cccggtatcc atcctttctt ctacatcggc
482581 acagacgcat tgaatcacac ggaggagtac gcagaagcag caggtgggca gcgggtctgt
482641 cgggaaacta tgaaagctgc atcaggtgat tccttccagc ccgccaattt ttctcgacgc
482701 ttatgtgttt ttctttcctt tcttgcctgg caggagctga aaaggcccag tcattcaccc
482761 tgaggacagc caaagcttta gccatgaccg ctgtcgatgt ggtctgcagc ccagatctgc
482821 tcacgcaggt gagggaggat ttcaggctgg ctaagctgaa aacagagatc tttgacactt

482881 agatggctgg acgttggtag caaatttcca tagagaagta taatttacac
cacagtaggt
482941 tttatagtga ttcaatatga aactagatct ctggggcaga tttgtgctgc
tctgtggcga
483001 tctgatgtac tttaaattct ggtcaggctt tcaccttcta acgctttgaa
aattatttgt
483061 tgccctcgag aggttgggga ggaggtgagt ctttttgatg tgattttttt
tttttcctgc
483121 tgatgtgttt tctggagagg taacggtgtg ggatttgccg tatttgatga
aaaagggtaa
483181 agaaatcatc agtatgatct acaatagttt caaagtgaga agacggattg
aaataaaatt
483241 tggattttat atgcagtctt tgttctgcaa ccagacagag cagcagagaa
gaaaggcggc
483301 ggccggtggc agttcagtgt ttaattcctg tgaaatcttg tcctgcagca
acgtttaaat
483361 atactgtgta agagagcaga aaactggata gagaatctgg cacagtgagg
ctgcgccaga
483421 acctcttatc aagagcttcc actgacatca cttgttagtt ttcctgtgaa
tttaatagac
483481 atctgcagaa gctatttcag agcaacattc agccatgcaa catctgctat
atgccacaaa
483541 ctctaatcat ataaactctg ctcacggcat tttcatatgt cacaaaccgt
gccttgtgat
483601 tgatttctca caattatatc ttaatttttt tttttttac agtaacatct
ctctgaattg
483661 aaaaagacaa tagaactagc aactaaaagg tgacaatcga ccttgaagcc
tgaaaacacc
483721 aaacagagga ggcgaccttt ttaaaaaaac agagaaaaaa ggaatacatt
caaaaaagtt
483781 gattgcttgg gatttaccaa aaaaaatgaa ataataatat ttagatttta
acagacttat
483841 tatgttgcat taacctttct aaagtcacac atgtgcactg caaatatcaa
cagcaaaaca
483901 tcagcattga aacaagattc acaagataca tactgaacct tttgaaacgt
gctgcaacat
483961 aaatggggga cgtcggattt ttaaagaaaa gttccacatt ttgggaatct
ccttcgctta
484021 agaagaggat tattgaagct gtataaaact ataaagtcac agcaggcagt
tggataaaat
484081 tagcacaaag aaaacatggg ggatgccagc ttatgtaata atcttaaaat
gtacttcgga
484141 tgctcagaaa tttttgccat agtttttcta ctccaggaga aaaccgacgg
ctaacagcaa
484201 aagtccttct ggaaacatat tccacattgc tttctttcaa atgatttttc
ccttggaggt
484261 gctcatggtg tttgtgcaat gctaagctaa cctggcatag cctgatattt
aacaaacaga
484321 tctaacagtt gggtgcaggt ctgtctgaca ctaggataga aagcaaacaa
atgcaactct
484381 ccctttaatg taggattggt ggttcagtct gtcagcgact gttttgttct
atttttaatg
484441 tccatttcca gaaaaaaaaa gaacagttca gagctgtaaa tctcaattct
ctttttcagg

484501 tgaaaactgt cactgcattc agaactattt acagcacaag aatgcacctt gtacagcagg
484561 ttgcagcaaa atccagatcc gtgtgtctgc agtgacggaa cgtctcacct tgttatcccc
484621 caaactcttt cgttttaagt ggctgggtcg tgaaggcaat gtcaccaatc tgtcagtggt
484681 cccggtgcat cttaagagca gttttctctc aaacgcaccc atctccatac tgctgaatag
484741 tgtctataag tagatggacc agtagatgat gttaaagagg atgtatgctc ctggaaacac
484801 caccctggag tacttgtcta tggcgtgggt gtctatccaa atgttcacat agcctctgga
484861 tggcttcatc tggctcgcca tctgcgggtc attcagtgcc acctgggcca gcattcgttc
484921 ctgccgaccg ccattctctg gcatcccata attccccatg gtgtttatat ccattgaccc
484981 gtagccagtg atctgggggt cgatcatcat gtcgtccggg ttgccgatac cacatgtaca
485041 cggcagctga ggagataaaa gatttagttt tttttattgt ttttaataag gagtttaaac
485101 tgccctctgc agagtcgcgt gcaggagaat tcattcaaat agttttcaat aaattatgag
485161 ctgcactaaa gcagccactt taatttcaaa aggagtaagt caggggggaaa aaaagctggg
485221 ggaaaaagga ccacaggata ccacaggata gggcacttac tctctccctc agcttcctct
485281 ctttcctctc ctgcacagtc gagaggtagt tgaccgccgc gtactctatc accgacagga
485341 agacgaagac aaaactgacc cagaggtaaa tgtccaccgc cttgatgtag gagaccctcg
485401 gcatggaggc gttaacgccg gtgatgatgg tggacatggt cagcacagtg gtgatacctt
485461 tcaaaaaaaa aaaaaagaag ctgtttatca catcattatt tcaaatcctg tgtccttcac
485521 tcgtgacact cttacctaac ggcaccctgg cgggcacggc cctgcggtct atccagaagg
485581 acacccagga cagcatgacc atcagggtgg cagggaagta ggtctgcagc aggaagaaga
485641 agatgtggcg ccgcagagtg aagtggatgt acaagcgatt gtaccagcct gaaaggaaga
485701 cacatcgaca aggacatgca tatgaaagaa attcagctgg atttatcggg acacaatgaa
485761 ctgatgtggg aggaagctga agaaaactct gcatattctc acctgtgctg ctgtagaaag
485821 ccagcttggt ggtggtgtga aatttctgga tgaggaactg ggacagggat atcctgtcgt
485881 ctgtgttcag ggactcgttg cctttcttcc agtacagcat cagatcgtcg tccgtgtacg
485941 cgtctgaggc gggaagacgg aaagaatgct ggttttcctc tgtcaagctt ttgtactctt
486001 aaaatcagcg cctgctctgc agcgagtaaa ctacccgcag cggcacaact gttgataaat
486061 ggaacaaaaa cgccgacatt cctgatgcaa cgctttggaa tagcaggata acaaaaacag

486121 cgcagaagcc tgaacatgtg ctgcaaccac aggaaacagg ggagcatatt tcccagtaat
486181 tacagagcaa actggtggac ggctgaggca gaataatgga atgcaccctg gagcgtgccc
486241 tcggcaaatt tatacccatc actgctgcgt tttcctgcgg atttcctcta tcgtcccgtt
486301 ttcgtagaag ccggaatctt tccacaatgt atttttttta aaagaagttt ctactcacag
486361 ctctcgatct ccagcgagca ggtctgggtg tccagaggga aacggctcag gtccatgttg
486421 cacatggcag tgacggtgac tctgcacgac acacaactgt ggctgtgact gtttgacaca
486481 ctgctcaata gaagggacac tggtggcatt tggtgattaa actacaaaaa tcaattgggg
486541 gtgttctcag cgttggtgat tacggtgagg aacaaggaag cctccgctgc tggccttctt
486601 cctctctgtt tttcctgttt acctaaaggc tactgcttca gctacatcac acgtagacaa
486661 taaaatctgg cacgacagtg ctgattacag ggctcctgcg gtccacattt gtctgctgat
486721 tgtgatcact gaattacaaa tatgcgttca ttctcatctc agcgtgtgaa agccatccaa
486781 acaaaccagg tttgatcttt tataatttct gctttggatt gttttggtgt ttttgtctct
486841 gatgtacaaa gattgcattg ttgggcacag aaaggactct tcagagtgat ttcttttcat
486901 tgtctgccag tgaagaacca gcagaagccg cgggacgagg cgacataatc acagaaacgc
486961 cgctggtcct ttctctgagg catcatatgc tttacctcca gcctcgtctt cctccctctt
487021 tgagtgtacc tgaggctgta gaggacattg ccatcggggt aaaccctcag catcacgttg
487081 tccgtggtgg tgtcgtggat gaaggagcgc ttggagtgga caaagaacat atcggggacc
487141 caaatcttct tcaccagacg gctgtcaaat gtcatactct ggttgttggt gctctggaag
487201 gacagacgct cgtccttcca gtagtgacgc aggtacagag tcatggtgaa gtcctgtcgg
487261 aggggagaag ggagggtgtg gacgttctgc gtcagtacat gcagctaaaa tgtctgcaaa
487321 ccatctacta ttattaatgc ttctgtatga ttacacaata tttaaatatt aatgttattg
487381 aattatttaa tgctgacaga ataacttgga gccacagatg ttctgctgca tttaagcttc
487441 caagtagcac aggactcctc acagagccga ccaaactggg taatctgggt cttagagacg
487501 tgaccacgaa cccagggacc gagcttcagc gttcctgcat gcagatgggt gcttttgtcc
487561 ctcaataggc gctcaatcac tagattccac cagatgttgc acatcagact gtgtttgaaa
487621 actaatccag atccactatt aggatcatgg atgaatgtgg aaagaattta gaaaagtggt
487681 ttccttacac agctttggtt ggaggctctg gattcagagc aaatgtggca tttaattctt

487741 aaccaccacc cccccccccc accctccatc acaacaccca cactattacg catacattga
487801 tgaaaaaaaa ccattatttt tatttttat tttttaact gcatccaaat ttcgaaattc
487861 ctgatcccgt taatttgtcc ctggttcgca cgcaagacat ttacctcaac atctgcactc
487921 acattttcga gtgatgctgc taaagcacaa agaggtgaaa acattaaaaa aaaacatcag
487981 aacctaccat gtccacctcg gagatggtgt ccaaactttc aacttgcacg tcgactccaa
488041 ctggaatcgg aggacctgaa atccaaatgg aaaagcaaag gcacaggtgg ctcccactac
488101 tgtttgttgg tgtctttcac taaaggaggc cataaatctc tgtagatcgc aatcttacag
488161 tcagctacag ttttctgata aacatgaaag ccttgttggc gtttataacc gcctaatcct
488221 tcctcggcta caaaaagcct ggaatcaaat ccctccaata tacagtgatg cagcgtgcat
488281 caacgctcgt gtgatgtaaa taactgagct gcgattggaa actgcggcgc agcttgcgtg
488341 ggtggtgtca ttaagatctc tgagggctta atgcctccac gcctttttga cctgaacctg
488401 actgctgcag ccttgtacgc tgtgatccag tatcctgcag gtctgtagat tagacatctg
488461 gtttggtttt attctgacct ccaaacgccg gcctcatagt aaaatcgtgg tcgtctattc
488521 tcagcagctg ctcggatttg gtcatcagag atttcgtcat gtccggactg cgtttgtaga
488581 tcgggctgcc aaatagaaga ggaggaacga ggcatgagcc cttaaataag ctgccaagag
488641 aatcaaagca tataaatctc accgtcccgg tttgtgactg ccgccctgat ccatcctggc
488701 ctcccttcta aatctgagga gagagacgtt gtgttgttac atcacaaatt cactgacagt
488761 gaaataaaca agcctcaaac aattagcctt taataattta atccccaaaa ccgggtggat
488821 taaatggtca aattggttgc cgtttccaca cttttggtgt ccacatcaca cactgttaaa
488881 tctcctctgg ggtcactgtt taatacgcaa tagttttgtg ttccaaaccg ccttttcagg
488941 tcacgagcag agcagaaata gcgggcgaga cagagggagg gggggaaaaa aagaattcca
489001 aatatcctaa agtggattat tagagtagag cgtgctgtga cggacagtac caaatccctg
489061 tgacaaccac agatatgcat gaaggaagta ccaaagagaa ccagagcaag gggggtgatg
489121 gggccgcaca aagaataaaa taaatatgat aagaagagtg tttgttagca gctccgccgt
489181 gccacaggca ctgtttgtct gcatccatcc agcagctggt ttctaatcct cttggcagaa
489241 atgcttgccc ccacccccca acctccctac atcgtatagc agctcgtctg ctctgtgtgg
489301 ccctgcgtcc attctgcagc cgaataccag cacacgcctg ctagtacagg tttcattgga

489361 tggaagtcac actggagccc tgctgctgag gatatggcgt gagaccсttt aggacgatgc
489421 gacaaaccaa gcacagaaaa aaaaggttaa tctgagtgta actggcgtgg gttgaaactt
489481 cgttatttaa tgtggcttta gtcctcatta acatttgatt ttaccaatta gcacagttat
489541 aaattatgtt tatgagaata gctgagggca acagacatgg tttactgtct gttttagctc
489601 ttgtgttcat taacacgtct tgcaccctgc actggatcct gtttgggttc agggctgttg
489661 gaggaattag gcatcaattt aatataaaat aacagttgtt ttcctcggtt tcatgtttct
489721 tttgaaaagc cttcagatta aattcaaata tgttattaaa acatctggat ctctctgcac
489781 atttgcatgc aatattacga aggaagtgag cttaaagggg ggaaaagcaa gctgtgaatc
489841 atccggcgag tttactcagt aattatactc tcaaatagct tcctccactg atactcatgt
489901 aagcgggaat gttttgcaaa tctgcctctg cttttcctcc tgaaggcagt gagcaggggg
489961 gaaaaaaata acatctgagt ggtggaaaaa ctccctcaac ccacgccctg ccgcatccag
490021 ccgctttcac gatttcacac tgatgaggag gagaggagcg aggagaagta cgggggagaa
490081 gagtttggct cctgcatacc agatttgtgt tctcagactc cgcgtgtctg ccgactgacg
490141 tgttaggact tctaacagcc agatttgata acaagcctgt tctggtgtgg ttacagattg
490201 aagcctcttc tcggtgattg tgatttcgtg gctcttgtct tgtgatgatt ctaatcacca
490261 tatgggtggg atgattactt caaacgcccg tgctttcatg gagagtcgct cagattcacc
490321 atcagtccag aatcatcaag ataattacag atatcagcct gctgcttcac gcttttgcaa
490381 acgtgaaaaa acacattcta tccaaaggct gggggggggg gggggggggg gttgtcgctg
490441 taaatgtata tgttactgta ggtcgatcta tattatcaac tgtgacagct tctttttaaa
490501 atcactggaa gaaacagcca tgtttctgct tattttcaag tgtccctgaa agttttgtca
490561 ttaggtttgc tgaagttaga gctatttaaa ggtcctgacc ttgaactaca gctgattaat
490621 cattcaaatt aatcaagctg ggaacgactt ttccctttgg ggaacgcaaa ctgaatattg
490681 atcacgactg tcgacagctg gatggataaa atacagacaa ttgtgaatta ttgaataagc
490741 aaacacactc gtagattgtt ttctgagggc agttatcgga gttccatgaa ctataatctt
490801 tggctgtttt aatcaattct tacaaattgt aagtgaaagt gacagaggag ctttgacaga
490861 tgtgattcag atgtgattaa agggcacaag taaaatgtta aaatgaatga ggttgaatgc
490921 gattcttttt gccgtttaaa tgactctgac acacgtgcgg acacgctttc aatctggttc

490981 aatcctgaca caaacgttta atgcagtaga tgcacaaatg aggcagcaat tagcctaaat
491041 aatcacctgg gagccacatt gaccacaacc gtcaccagtg ggggggaaac agtcattaac
491101 tggacgccag tggttgtggg gcaagagaac acagcattca ccaatagtac ataaatagat
491161 agggatttag gtctccctct ctctttctct ctctgctctc tccaatctgc tgctttaata
491221 agtctgtttt ctggtggggg gagggtggac aggtgacaca ggaaacactg gacaaagcaa
491281 agtaaacctc aaaaatagtt acatattcac agatcacatt gtcagcacct agaactttgc
491341 aaacggctgt gatggaaagc tctttagatt gtattaagaa aataaaagca tgcagcccag
491401 tgcttgtttt attttcctgt taatattttg gagcccttca actttacctg ctctgcgtgt
491461 aggtctccag cttgtgaccc cgcgactggg ccatcctagc ggctgcaccg gcgagccaga
491521 cacaaaagag cactagcgca ggatccacct gcatgtttcg cattccacag agggcaagcc
491581 tctcctgtca gtcagaatct tctcccctcc acactttgga gccctctgcc tccctgagac
491641 attctccttt gtccagcaga agaattggtg aaaggaaaga aagaggcaac aacaggagct
491701 acacttggtt attctacggc ctgagaggaa aacatcctac ggtgcttaat ctccacagtg
491761 ggaaaagcaa ttttgactaa acccataaat ccttgagtgt cagatggtac agatttgatc
491821 cctccagcgg cggatgtgcc tgcctctctg tgcgctgctg tgctctgata ttctctgctc
491881 tgctgtgctc tactccactt tgcaccgccc agaaacacca attctcacaa tgatgcggcg
491941 tgctcatttg taaggtcaat ggtttagaga atctgactca cggtggctga gaggaagatt
492001 tgtgcatgtg tgtctgatgc tgcagcctat ccaccagcat gtccaaaaag aaaaatgttc
492061 aaatctgctt tttatattta ttttttgcag tgttttgctt tcaataaagc tgtttggata
492121 ctgtacatat tctgttgccg agggtgtttt ttaaaatata tttttggttt agcttttact
492181 cgtgggactg catttgatag ttcaggaacc agattttcag aaggctaaag ataagataag
492241 agaataactc gcgcttcagt gtcaaacggc atcgattcag ctccattaac tcgatctcaa
492301 agctcaggac atcgtttaaa aaaaataaat ttctttgttg caccagttta actggtggcg
492361 cgtcatccat ctgcacctgc caatcataac cgcctcagca ctgacatcac taccatgcat
492421 gtgccagccc tttttttttt ttttgtgcag aataaacagt tggattataa gatcatgaat
492481 ttgggttagc tgcacagacg tcagtctgaa tagtggtgta agtaatcttt gggattgttt
492541 gtatgttttt tttaattatt tttctcctgt taaacctaat cgtgctgact aggagaaaga

492601 aagagctaat aagctaggaa acgccaataa taagactgac ctgggtgtag aatatgtgtt
492661 aataaacaca caaaatcttt taagatccat tgtttttgat ttatgggggg gggggggggg
492721 tatgaggtgc agatgatgga atctaattat attttaaagt ttccatttgt aggagtgtac
492781 tgtcacacta acatgcagtt cagtctgact tcaagatttc tctggtccat ctgcacttga
492841 acatctgagg aaaacctcca aaggaactta gaatgtggag acatgatgta ataggatcta
492901 aggtttttga tgttgggagg catgggcctt cacagactgg acctgctcca gctctctatt
492961 aatgctttta gtttgtaaaa aaaactcaga actccattgc cagtgattaa ttcaaatttg
493021 taatattgaa tattgaatgt atctgcatat tttactgatg taatatgcta ctgattgctg
493081 tattgcagca ttccaacttc aatgttaagt gtttttcttc caactgatga gacagtaaat
493141 tatgatgcag attagatgct gatgtgtctt taatatccgt cttttataaa atacagtaaa
493201 atgatttcct gttgttacat tctgttctgc acaagactag gtgattagat ttaagcatga
493261 ccactggaca ttggtaggct gactatgtct ggataattgt ggattattta actggaaacc
493321 agttgacatt gaaaccagtt atattttaat attcaatcca ctaacttcat gttcagatta
493381 gtagattatt ttactaataa aaatcaagat ctctatttca ttctttattt atttagttca
493441 gccagggatt ttttttaact tttttataaa acccgtaatt ttggactgcc aggtttacag
493501 ccgacgccat aacttggctg catacggtca gaagaaaagt cttcacacaa tttcgttgtt
493561 ttaaagcaac atgaatttaa catttttaaa ttgatggcta atttgaatag gcgatcttta
493621 cagtcagccc agaaccgagg tagaacaata actgcaactc ttgcctgaca gacaaatata
493681 gagggcgcaa ttacctcggt aaggtcaggc tattggtctg cgtcaccgtc tggcgcgact
493741 ttgcaaagtt gacagaggaa ctacgagaga tgtgctgact ctcaataaac aattaaacgc
493801 gatcggatcg acactgatgg tttggcgaag ggccgactca gtgaaatgaa gtgagacacc
493861 attttagttc gcttggtgtg cttccagtcg ttgtagtcgt tgtccagcgc gctgccgcgt
493921 cgttctcccc cagctgaagc gtcgcgttag ccgctggcta gcaccgagct atcatgagct
493981 ctacgcggga aatcaaacgt aagtattgat acaggaacac cattttaaag gtcgttcggc
494041 gttggttctg ttgcatatgt ggtttctgtg catgctttcg tgacgtgatc ggcccgcaga
494101 gctccagaaa gcaaagagca aggcgcagaa ctgcagcaac ttgaaggagg aggccaacat
494161 ctgcaaccag ctgggagagc tcttctccaa aagtggtaag tgtctgatgt cgattgtttt

494221 cattattatt catcttggca ataagcacag cggtcgcaaa ttggcaggaa taactcataa
494281 aggtgacgta tctgcatttt aaatgttaca cactaagggg aaatttgcgt gcatgtacct
494341 gatataaatt cagaaccaaa gtaatattat tcttgagcct tttggaacca gcagaactct
494401 gcacttgcac tatgcaccaa tccaggtatt atatgataat cataatgagg ataatatttt
494461 aagcctggtg tgcttcctgt gtttaccagg tgattataag gctgctattt cagagcacag
494521 acaagagttg gtgctctccg aggcgctcaa cgatgtcatt ggccgcgctg tggctaatcg
494581 caagatcggc gagtgctacg ccgagatggg caactttaag gctgctttga aagtcagttg
494641 aagtaattgg gaatacaaac atttttatta ggtgtgtggc ttagaggtgt tttttcctct
494701 tcagcaccag cgctgtcacc ttgacctcgc tcgctctgtc agagaccacg ctgaggagca
494761 aagggccctc gccactattg gtcggaccta cctgttccgt tatgagtcgg accaatccag
494821 gagcagtttg gagcaagccg aggatgcctt caggaagagt ctggccattg tggacgactg
494881 cctagaaggt cagattgatt gttacctcag tggctgtgta actttatagt gtttgtgact
494941 ttcacatgga catgggtcat ttttcctttt gtaaacacag cgactgtgtc ggtgcgagaa
495001 atcagtgaga tgaaggctcg tctgttttta aatctcggct tggtctgtga ccatcttggg
495061 gagcccaaac gctgcagcga gttcatccga cggagcgtct ttattgctga gtaaggaggt
495121 ttggcgcgtt cctgaaggca gcaggctttc agaaagtcaa tggtgatttc tcccttcctt
495181 tgaaaggaag agtcagctgc tggaggatct cttccgtgca aactttaacc tgggcaacat
495241 cttctaccgg aacggtcaga agtccaatgc cgtgcgttgc ctggaacagg ccaaagagtg
495301 tgcgaggaag atgaaggaca agtttggtga gagcgagtgc ttccattgca ttggcaaggt
495361 aagccatcgc cctctaaacc tctgagccaa aagtgcttta cgatcagttt gttttatttt
495421 gttgcttcat gtgcctacgt gttgtgcgtc tgcaggttca gctgtctctg ggggactttg
495481 tcgcagcgag acgttctctg aaaaaggctc tattgttggg ttctcagcag actgtggaca
495541 ggcagtctgt gaaaaaagcc ttcaaatatg gtaaactagt tctctttgtc tagagtcaaa
495601 tatttcaatt aggtttactt aatatttgta ttaattttac acttgactgt gaatttttta
495661 gcggatcgtg gctgtaaatt agaagaagag tttggggatg atcaggccac agggctcagc
495721 tcccatcagg ccgtggggct ggcagagcag ctcggggatc tttactgtaa agtcggctgc
495781 tacagcaaag ctctggatgc atatcaggct caggtaaggc ctagtctata tatgcacatt

495841 cataagagtc ttttagacga gtcaaaaaaa aaaacatgga caatgtgctg
acaaatgacc
495901 tgcgttgtcc ctgtgtcaga acataaaaga cccgtcttta attcctgctg
tactttcctc
495961 catgttcttg tccagacaca tttgtaatat aaatggcagt tgtcctgacc
cgaaactaat
496021 catgtttttg atctctgcca acccagatat ggtaatccca ctggacgtga
ccaccccccc
496081 cccccccaa aaaaacaaaa aacaaaacaa aacatcctga catcactatt
cgcaacatcc
496141 aatttctgcg cgtccgtgca gccaaatgtc ttttgtgcgc tcagtttgct
ggacttgatt
496201 gtgtgtgtgc tgcagctgaa gggagctgag gctttgggaa agccggccag
ggagctggct
496261 gtcattcacg tttccttagc cgagacctac acggacctga tgcagcacag
cagagcagtg
496321 gagcactaca aacgggagct ggcattacga cagggcagcc ccgctgaggt
aatgttaccc
496381 agcatcgcac gcgctattta tgcgacaatg cgtcgtcaag tctgacaggg
gctgtcggtt
496441 gtatcaggag tgcagcacct ggctgaacgt ggcggcagct caggaggagg
gcggctttcc
496501 ctttgaggat gtccacagca gcttcagtaa agcctcaaac tgtgctcaga
cgtccggaca
496561 ggcccggctg caggtgtgtg tcggtgaaag acggccgcgc tcggtgaaac
ggcccaggct
496621 ctgatccaaa tttccctcat gcagaaacgc gcgctgagga tctggctggc
gttccagaga
496681 cgacgtggtc caagtgtgtc tggagacacg gaggccaaac tgcaggagct
gtgcgccgcg
496741 gagggctgga acccagatgg gagtgacggc gaggacgatg aagaggagat
ggacaacagt
496801 gaaccgctgg acgacagcga tgtcatcctc tcggaatctg gtatattata
ttcggccact
496861 ctgctgtcaa gaatgcaatt ttggcttcat tttgtgtttc cctacatggt
ttctagacga
496921 tgatttggag ggttatgata agatggtgac aggacggagg aagacgggga
gggtgagtca
496981 tttgttagtc ttcccttgga atctttcctt tgacaacgtc catcatttat
ggccttttct
497041 tactttagtg gaacaaaagg aacgagaagg gcgagacgtc tctgcatcga
gcgtgtatcg
497101 atggaaacct gaagcaggtc cagtacctgg tagagcaggt gagctaacca
cgattgtgtt
497161 agtcacgttc aggccgtttc tgttttatta ctttatttta agaaaatgat
gggaattgtt
497221 ttaatgatgt ctctgtcagg gtcatcctat aaaccccaga gattactgtg
gctggactcc
497281 tcttcatgag gccagcaacc atggtcacta tggtaaccga ctacttccac
tcttcctcgc
497341 tttaaaagct ggcgtcatag gatatgaata aatatggcat tgttttattg
catcgtcctc
497401 cagatatagt agctgttctg ctggatcacg gcgccaacat caatgatacc
ggcggtccct

497461 tgtgtgaggg agtgaccccc ctccacgatg ccctctcttg tggaaatttt aaagttgccc
497521 aaatcttagt ggaacgcggc gcttccgtca ccctgcataa ctccaaggtg cgtattacac
497581 ctctttccca ccatgattat ttaattccat aaaaacgcgg gatgaacaat tgcttcctac
497641 ctgaagaaac aaataactgt tttattttca atcccagggc tacactgcct tagacactct
497701 tcatcagtgg cagcgaacgt acaggagaga gctggaccag gaggccagac aggcctgcat
497761 caccacagag agactgctga ggaaggctct cgccggggga ggtgaggctg ttgcagcgtc
497821 acgctacttt agcctcagtt tgctcttgag cctgtatttt catatctttg tcacagtgtc
497881 tgctgccccg gctccagtta cgccgtttga cgcactgcag gacagtcagc tgtttgacgc
497941 tgagaactct gagccgctgt cggcctctgg aggggacgga acgtcgagcc aagccaggct
498001 gcagaatatc tcggaggtag ttccagcacc tgccagtccc aaacggtggc agaacaatgg
498061 ctccacgcag cggcacaggc agagaggaac agggagcgct gtcctatatg gggtaaaaaa
498121 aaagatcgat aagttgttga attcaatttt ttttcaaaag attcagtttt ctttagtctt
498181 tgaatcatcc tctaactcta gtaggtgtaa aaacaaagat tatttaggtg ttttttttaa
498241 tcttgtagga cagcagttcc tctgattcaa gtgactctga ccgctcggtc agtcctctcc
498301 gccctgtgcg gccgagaaaa agtttctccg caaccagtca gaaggagttg cctccaaacg
498361 cggacgccaa ctctgccccc agctcagttc gagaaaatac cagggagcct tctgtggctt
498421 cggtctttgc aagagaggag taccaaagtg ccatccgaag cttgggaagc gccaaaactc
498481 gacttcaggg tcccacacag cctcagttct ccagcacgcc ctctttgtca tccaacaaca
498541 cctcagccct ggtgcccgag gacgagtacg tggctgacga ctggctggag gatgatttgg
498601 aagaaatcca gccaaagaaa aagaggagga tccgtatgga ccagaatgaa atgagagggg
498661 aggaccttgt ttcatcttca gcagcaagga gtcaaaacag gagcatccac tcagactttg
498721 tccccagagg taatcaggct tctatttgag ttcttcatta tgccaatacg tccaacatct
498781 tagtgtactt acgctctctg tgtactgcag gtacttcttt gtccagcagg ggtctctctg
498841 tgcaaaagaa caactctgtg aagcctcacc aagtcaaaat gactcagata ccgggaatgg
498901 ttagactcgg aaggcgtgaa gtcagcaggc cacaaagccc catctttaca gaggtggacg
498961 tcatgagacc aaacgcaact tcaccacaaa tacgtgctca agtgagaaat tcaacctcag
499021 atgtttcaga tctcaggcct cctgacacag cattatatag aatatattag acaatctgaa

499081 aacttcccat tgattctatc tgtcatagtt ctgtcacatt tcttgatacg tgctcaatga
499141 agctgctcga aaacgagaaa tgatcctgag tccattttaa tgccctgtat ccaccaattg
499201 tagattttt tttatcacaa tttaattta gcatttttct ccagttaaat atagctgaag
499261 cagtattttt aaacacattt tcagtttgta actgagctga tcacagatta gaatcctaac
499321 caacttgact caacgtttgt agacagtgtt gagtgaagaa agtaacgcac cccactgtga
499381 tgctgtttta ttctaatctg cacatatatt gtattactct cctccagctg tttcactagt
499441 acgtgcattt atctcatatc tgtcagcctc ccgccgtgcc ttcgtgtctt gctccgccaa
499501 tcagagtgag ggtcagagtt caggaagatg tgttcctcat tcctgttcct caaaggttgg
499561 ttgttgcctc ttttattaat ctgaatagat taaaatgtgt tcaataagct actgcttgtg
499621 ccttttagtc cttttcttat cagtgaatgt gtcttcattt ttcgccccag tgaggccgac
499681 tcgtgcacgg tgtcctggct gtgcgaacag gcatctcagc gttactacca gaagtgtggc
499741 ctcctgcctc gcctctccct ccagaaagag ggcgctctgc tctccccgca agaccttctg
499801 ctcactgtcc tgcacaccaa tgaagaggtg agtacggtgc tccgcacccc tgccgttgtt
499861 tgttatttac caggagagaa ttacagtgac gtttccatgt ttttaggttc tggctgaagt
499921 ttgttcctgg gatcttcctc ctctccccga acgctacaag aaagcctgtc aaagcctggc
499981 agtgggtgag aaccaaacac acgcagaact tcaaacagcc gcactattag agcttgtgca
500041 gggacgcatc acctctttat ctccctgcag atgaaaacaa gcaggtgacc cggctctgtg
500101 aggtccacgg aagctcctct gtgtctgcgt gtgggctcag cctgggcccc tcctccctca
500161 ggccgctcct ccgcgccctg aaacttcagt ccagcctcac cgagctgcgt ctttcaggca
500221 accggcttga cgacgagctt ctgcccgagt tggtctccac gaccgtcacc atgccccgtc
500281 tgcaggtgct ggacatttca gcctgttgca ttacagcaga agggctggag aaggccgtcg
500341 gcgcattaca gggacagagc caatcggcgt ttccggtaaa aagctcagct gggaaccagt
500401 tttcaaacgt aatccaacgt tggtcacatg aaatctcgtc ctccttttcc ctccagtgtt
500461 tggaggagct caacctcagc atgaacccgc tgggcgacgg agtgtccgag gccctgtcct
500521 gcctgctgtc gggctgcccg gtgctggtga agctgtcgct gcaggcctgt caactcaccg
500581 ctcgattcct tcagcagcat cgcttgctgc tcgccagcgc cctggcaggt gactgtctgc
500641 attgctgagc ctctaacctt catgaaatgt atagatcacg ttgaaaacag gatcacgaac

500701 cactgtttga tttgaccttt caggcagagg tctcctcaga tcggtgtctt tgtcccataa
500761 tgctctgggc tccactggct tcgagctcgt cttgaagact ctgcccctac acagtctcac
500821 acaccttgac ctgtcagcag tgtgccaggg tgttgtggat ttcctgccat tgcaacacct
500881 cacctctgca ctctcacagg tttacacgcc aatgttccgt ttgagtctga ggtttaacaa
500941 atctagcgtt gaacatgttt gaggtttatc atgtttgtat tttagtagag actaagaatc
501001 tgtctttcaa caggacaagt gttctctaac ccacctgagc ctggcagcta acgggctgac
501061 agatgctaat gttgccacct tggccaggta tgtgtgtgtc tgcgtacctg ctttgataca
501121 tactgggaac tctgggcgta atctagtcca tttcttcttt aggtgccttc catcttgtcc
501181 aactttggtg agcttgaaca tatcaggaaa tcctcaagtt acatcagcag gacttcagaa
501241 cattctgacg cacctcaagg aagcatctcg acctttgacc cttctaaacc ttgaaggtac
501301 agacacactt atagaggaaa gacagttttt tactacagga agtgtgatcg taatgtgaac
501361 gcagtagcag gtaaatgcag ggcttgcaag ccagactgtg ggtgctgatg agccgtttgt
501421 gtcacaggat gtcaggtgtc cggcccctgg gacactggag ctctggatgg tttatcagag
501481 ctggtccagg atctgcgcct ttgctctcag gcgctcaaca aactggaccg gcaggcttta
501541 aagcagagct ggaacggcag cacgcataac gtcgtcgatc aaaacagcaa atggctgttg
501601 tctgccgcag ccccctcctg agggggagaa ttctgactca caacacactg tggggggtca
501661 cacgtttcaa aatgtttcca ttagctctac atgtctgatt atgatgtctt aaaggtgatg
501721 cttccaatgt accctgtgta tatagactat ctgtccttta tctgtaaacc ctaatttatg
501781 tgtttgtatc atggtgcagt attttttttt gatttgctcc attaaaaaat gattaaagtg
501841 actagaattc tgtcttctgt tgttttacca acgggtgcgt ggagataata tatattttat
501901 aatggaatgg ttagactcgg aaggcgtgaa gtcagcaggc cacaaagccc catctttaca
501961 gaggtggacg acatgagacc aaacgcaact tcaccacaaa tacgtgctca agtgagaaat
502021 tcaacctcag atgtttcaga tctcaggcct cctgcacagc attatataga atatattaga
502081 caatctgaaa acttcccatt gattctatct gtcatagttc tgtcacattt cttgatacgt
502141 gctcaatgaa gctgctcgaa aacgagaaat gatcctgagt ccattttaat gccctgtatc
502201 caccaattgt agattttttt ttatcacaat ttaattttag catttttctc cagttaaata
502261 tagctgaagc agtattttta aacgcatttt cagtttgtaa ctgagctgat cacagattag

502321 aatcctaacc aacttgactc aacgtttgta gacagtgttg agtgaagaaa gtaacgcacc
502381 ccactgtgat gctgttttat tctaatctgc acatatattg tattactctc ctccagctgt
502441 ttcactagta cgtgcattta tctcatatct gtcagcctcc cgccgtgcct tcgtgtcttg
502501 ctccgccaat cagagtgagg gtcagagttc aggaagatgt gttcctcatt cctgttcctc
502561 aaaggttggt tgttgcctct tttattaatc tgaatagatt aaaatgtgtt caataagcta
502621 ctgcttgtgc cttttagtcc ttttcttatc agtgaatgtg tcttcatttt tcgccccagt
502681 gaggccgact cgtgcacggt gtcctggctg tgcgaacagg catctcagcg ttactaccag
502741 aagtgtggcc tcctgcctcg cctctccctc cagaaagagg gcgctctgct ctccccgcaa
502801 gaccttctgc tcactgtcct gcacaccaat gaagaggtga gtacggtgct caacaaactg
502861 gaccggcagg ctttaaagca gagctggaac ggcagcacgc ataacgtcgt cgatcaaaac
502921 agcaaatggc tgttgtctgc cgcagccccc tcctgagggg gagaattctg actcacaaca
502981 cactgtgggg ggtcacacgt ttcaaaatgt ttccattagc tctacatgtc tgattatgat
503041 gtcttaaagg tgatgcttcc aatgtaccct gtgtatatag actatctgtc ctttatctgt
503101 aaaccctaat ttatgtgttt gtatcatggt gcagtatttt tttttgattt gctccattaa
503161 aaaatgatta aagtgactag aattctgtct tctgttgttt taccaacggg tgcatggaga
503221 taatatatat tttatacttg tttaatatgt gctagttgtg gctcataatc cattctctgc
503281 atcctgttgg atgcctgttc agcacgctgt tcctaatccc accgatgctg gtcgnnnnnn
503341 nnnnnnnnnn nnnnnnnnnn nnnnnnnnnn nnnnnnnnnn nnnnnnnnnn nnnnnnnnnn
503401 nnnnnnnnnn nnnnnnnnnn nnnnnnnnnn nnnnnnnnnn nnnnnnnnnn nnnnnnnnnn
503461 nnnnnnnnnn nnnnnnnnnn nnnnnnnnnn nnnnnnnnnn nnnnnnnnnn nnnnnnnnnn
503521 nnnnnnnnnn nnnnnnnnnn nnnnnnnnnn nnnnnnnnnn nnnnnnnnnn nnnnnnnnnn
503581 nnnnnnnnnn nnnnnnnnnn nnnnnnnnnn nnnnnnnnnn nnnnntgtca gaaagaaagg
503641 ggacttttat gtgccccct ataccccca tccccaaatg ttttacctga atgtggagga
503701 gtaggcgaaa cagctactgg gtgggtcatc atgacagtac acattaatgg tcgcgtctaa
503761 attcctttta actttacata aatagtcaga actgtttgta caaaaaggtt gaatttgatc
503821 ncagttgatt tagttgcctc aaacgagatt catgaaattt tctcttcccn gcgacgtgta
503881 ggcaaaccag cctgnccaat ttaaactttc ttgtggtaac gacaacagca tactgtcaca

503941 ttaggggttc tgtttttttc tgtctgagta gatataaaag cgaatcatcg tcaccagcaa
504001 gatcgtattt cctagttatc tgactatttt taggaaatgg gaaagatttc ctgaggagaa
504061 aaacctgcct ggtgtgttat gtccagttgt tttaagccac agaatcagct ccagtatatg
504121 aacacgctcc acacacaaag gttcaatgct caagatcatt tattgttcac tagcaaagtg
504181 caagacatgg gcacaggttg ctaatatata ttttttttaa aaagaagaaa aaactgggaa
504241 aagatctttc tttttcttgg aagttgaaga catcctttcc catgtgctcc cctcaggcgt
504301 actcattcat ctctaactgt gaggaaaaac cactctggaa gagctcctcc agctcctttg
504361 gcctaaaaca gttcgagaaa acaggggtga gataaaagtg atgagagtag aacccctatc
504421 caaaaccaaa cgtcaaacgt ttgcctgcac atgtgcctgc tcatctctga aatgtcaatg
504481 ttggaccaca attgtgagga tatctgtcct cttttggctg aggtgtggac tgtagggtta
504541 ctggagaccc tctgcaagtg tttataggta cttagacgta taaaagtgct ccatcatcac
504601 gaaccgagcg cgatgaactc acttgcaggc cttgacgttg acgcagatgg ttctcacgtc
504661 atcgctggtg gtgagttctt cgatcaatac ccccaggtgt tttgtcacga atttgcggca
504721 taaagacttt aacaggccta tctggttgca gatggacatc agctttgcct tgatggcctg
504781 gaaaagtgga caaaagaagt cacactgctg ctccttccag caggaccctc cacctctcgg
504841 cacctttggt gtacaatact ttacctctga gttgctgttg tttccgatga ttatcttcac
504901 tttttttaaa gcccacttgc acgcccagca cacacctgga agctacgggc aaaagcacaa
504961 ataggagaaa accccactat gtttgtgcac aggcacgtat acagtttgac attaacagtg
505021 tggatctgag cactccctgc gtccctgtgt atgcacaacc aggttcgact ttgactcata
505081 tggggtcttc aataaagtca aatggttatc tttgtaaaca acaactttaa atcaatgcaa
505141 gcacgcaccc tgccagcttc tatggcaagt tcgtcctggt cttcatcgtc gtcatcagtg
505201 ctgaccttta aatttcttgc ctggacagtc caaactaagg agacaaatag tcacttgaac
505261 taggatttaa aggggaagat aaaggtttga gtcccgctgc agcctaccag aacacgtcac
505321 caggatgcac agaagcagga tcgaggaggt tgccattgct ggagctctga gtgcctttca
505381 ggttagtgaa gaacctttta tatgcagcag aagcgaagag gtgggtccaa gacagatgga
505441 aattcacacc acaaccactg tggctgcaaa gaatgaacat gttgagggga ctgttttcac
505501 acttcttcac agaaccactt aagaacgtca cattctagtt acaggaaatc agagagcctc

505561 tccgtttaaa ttcccatcaa tgtaaaatct aactcaacgt atggacagga ggataacatt
505621 ctagatatgt aattcatcta ttaaatcatg tcacgttact ttaaagttcc aatactcacc
505681 ctaacatgtc atgcagtcat gaataaacac cttttgtcac attttgttgg tttccataac
505741 aatgattcaa acactgcttt taatgatttg ctgtatttat tttatattta ttttatgaaa
505801 atcagaggtt tttttttttg ctgtaaaatc ttacattcta aaagtttcca ttaattctgc
505861 acatgacaaa atacacagtc ttacgctatg caggcaaaca tcagctgtgt gctttagtcc
505921 tattgaaaag ttaatatcca accatctgat atcacttgaa acaatgaacc ctctaaaagt
505981 caaatccttg tggatgccaa atggaataaa gttcccgaca gtgccgtctg caataatcat
506041 atagatgaat ttcatgactt cgcatagaat tagctgttaa tgtgccacac acagagagac
506101 ggttgcgatg ttacattttt taactgaaac caggcttgaa gcgacttcct ttcacacctt
506161 ccagagcatt ttcaaactga cacatatgca gcgtcagctt ttccctcgga cagacggcct
506221 ttatttgtaa atggttcttt ttctcctaca taattttaag cggacacaaa tttgctgggg
506281 tcgtccgccc tgttctaagg cggacatcag tttgtctttg aagcgtgaga ggaccttgtt
506341 acacagagat ctgagaggtc ccgtcttctt gcagacctag ttcagcaggt tgttgatttt
506401 cgcctttaaa tgaatgcgaa ggaaatataa tgatgaacgt cagcggtgaa ggatttgcag
506461 agaaggtgtt gaagtgaatg atattcatta cccttgagtg atcgctgccc aggctcttaa
506521 tcactttgga cacgatcatc ttgcaaaccc tgcaggagcc tgggaaggcc tcatcccgca
506581 acacctgcca acagtcacct cgcgccgtta agatctctcc caaaggaagg agggaagatt
506641 ctccaccgag gtcaaaagta ctggcgtgag acttcagcag aaatctgagc ggcagtttct
506701 cacctcccct gagtggtggt cagtcaccgc cctcacaaca cctggcaacc tcatcggaaa
506761 agcttcagta tggtgagcag gtttacgggt ggcatgagca aaatctgaaa gcaacacaaa
506821 gcacgtctca gaaaaaggtg tgagcaacgg gcatcagaaa agatgacttt cttactggtg
506881 gagatgagaa aaagagccga gataatggcg agaatcatgg ttgggcccgt cagtaacgtt
506941 cttcatcagc tcttcttctg gggctcttta tattatatct ataatataat atatattatc
507001 tatatatatc ttctgctatt tgttgtttgt aggctcagca atttgtcata agtgatactt
507061 ggtcagcgaa ttagcaaagg tttgccctca gcaaaacatc ccatgccaaa caggagggcc
507121 gaccatagac agatcagcct gggaaccttt gaatgtgggg tgtagtcttg gaactccaag

507181 gtgataaaat caactaaaaa accgaattaa accttttaga ggatattgct
ctacaaatgt
507241 aacccctcag atgtttgcaa atatgcgtta acatatgctt tattattctg
tagaaaaggc
507301 agaataccac aactgcaggt ccaccatttc cttcctctat cacacttccc
ctcactaaca
507361 ctcacacacc ccactcacac cccccccccc ccccccaca cacacacaca
cacgctgtaa
507421 acgtggccta tataacgcag cttttctgtc taataaacca gatggagatg
gatttaaaat
507481 gagttgtatt gtccgtttct gtgtgaaaca cccggtttga ccaaaatgaa
agacatgact
507541 gcttggacag ttctgtttca ctaaaacagg gaacattctt ccttcatagt
gctcacagat
507601 gtaccaataa taaaagaaac acaaagttaa aaacacagtt taacaacaca
gtttaaaaac
507661 agtgtcatag aaaagaaagg tgtcctggga tgacatggac agagaaggac
agtgttcggt
507721 ccacctgact ggccagatac ctgaagctgg tgtccctttc ttgcggcagc
acagccgtag
507781 agaagaatca tcatcataca ggtcagcaaa ggtcacaagg tcattctttg
acctgagggg
507841 agaaacacgt ggttgtttac aagtgatggg agggtctggt caatcaacag
gagtggacag
507901 tttggacgaa gttcagcctt ggtgaaaaca aatgcaacag gttttctttc
cccaaatcat
507961 tgcattaaaa tcagggtgat tttaataaaa attctattgg aatgnnnnnn
nnnnnnnnnn
508021 nnnnnnnnnn nnnnnnnnnn nnnnnnnnnn nnnnnnnnnn nnnnnnnnnn
nnnnnnnnnn
508081 nnnnnnnnnn nnnnnnnnnn nnnnnnnnnn nnnnnnnnnn nnnnnnnnnn
nnnnnnnnnn
508141 nnnnnnnnnn nnnnnnnnnn nnnnnnnnnn nnnnnnnnnn nnnnnnnnnn
nnnnnnnnnn
508201 nnnnnnnnnn nnnnnnnnnn nnnnnnnnnn nnnnnnnnnn nnnnnnnnnn
nnnnnnnnnn
508261 nnnnnnnnnn nnnnnnnnnn nnnnnnnnnn nnnnnnnnnn nnnnnnnnnn
nnnnnnnnnn
508321 nnnnnnnnnn nnnnnnnnnn nnnnnnnnnn nnnnnnnnnn nnnnnnnnnn
nnnnnnnnnc
508381 aagctcactg ttcattttgt caggctgcag gaagttttgt gtaagtaaaa
tgtgccacat
508441 tgctgcaaag gccaaaaaaa taatgcacag tgttggggaa aatggggaaa
tagaaaagag
508501 aagaataaaa agctgtggac tgaatgtgcg agtatgttgc ttcatgtgtc
ttcttttttt
508561 ttgcaaccat ccaaagtgat taaatctcat atccaaaaag tctttgtttc
gcttaatcta
508621 tggtaaaaaa aaaaagaact actgagaaga cacattttta aaaaaccaaa
ccaaagatgt
508681 tcggccagtt atgtcagctg ctgtattttc ttctgtgttt tgtttgaaca
ttataccaac
508741 aaaatatatg taatcaaaaa gttaatatgt ggaagttttt gataaaattg
tgcgttttaa

508801 aataatataa ataatacacc ttgccatatt cggctgctat ggatgttttc
tgccagcagg
508861 tggcgctagc ttgcgtgtct gaaagggact tcctgtgatg actacggtat
cattaaagac
508921 acacaacagg aaagcaggca gacacataaa tagcaaagtg tttgcgattg
tctcaacttc
508981 catagatagt ctgcgcctac agcgacaatt tagagacgca gctgcttagg
agagactggt
509041 gaatctgcag aaaccctgct gccaaactgg gatccgtcca gtaaaccatt
cctgcttaca
509101 gatatatgag ggttttctaa agtaaatgtc ataaatcttt acagagcaga
ctgtccagtt
509161 ttgtgataaa gaagaaactt tattttctca tgtacagtat gtacaataaa
ctggacattc
509221 agttgggttt aaatgcagaa cattccttcc gaccgacatc agatctacct
ccgtgcaaca
509281 cctggccact taacgcatat gtggtcctga catcacgttc acagtaaagg
cagcatgcan
509341 nnnnnnnnnn nnnnnnnnnn nnnnnnnnnn nnnnnnnnnn nnnnnnnnnn
nnnnnnnnnn
509401 nnnnnnnnnn nnnnnnnnnn nnnnnnnnnn nnnnnnnnnn nnnnnnnnnn
nnnnnnnnnn
509461 nnnnnnnnnn nnnnnnnnnn nnnnnnnnnn nnnnnnnnnn nnnnnnnnnn
nnnnnnnnnn
509521 nnnnnnnnnn nnnnnnnnnn nnnnnnnnnn nnnnnnnnnn nnnnnnnnnn
nnnnnnnnnn
509581 nnnnnnnnnn nnnnnnnnnn nnnnnnnnnn nnnnnnnnnn nnnnnnnnnn
nnnnnnnnnn
509641 nnnnnnnnnn nnnnnnnnnn nnnnnnnnnn nnnnnnnnnn nnnnnnnnnn
nnnnnnnnnn
509701 nnnnnnnnnn nnnnnnnnnn nnnnnnnnnn nnnnnnnnnn nnnnnnnnnn
nnnnnnnnnn
509761 nnnnnnnnnn nnnnnnnnnn nnnnnnnnnn nnnnnnnnnn nnnnnnnnnn
nnnnnnnnnn
509821 nnnnnnnnnn nnnnnnnnnn nnnnnnnnnn nnnnnnnnnn nnnnnnnnnn
nnnnnnnnnn
509881 nnnnnnnnnn nnnnnnnnnn nnnnnnnnnn nnnnnnnnnn nnnnnnnnnn
nnnnnnnnnn
509941 nnnnnnnnnn nnnnnnnnnn nnnnnnnnnn nnnnnnnnnn nnnnnnnnnn
nnnnnnnnnn
510001 nnnnnnnnnn nnnnnnnnnn nnnnnnnnnn nnnnnnnnnn nnnnnnnnnn
nnnnnnnnnn
510061 nnnnnnnnnn nnnnnnnnnn nnnnnnnnnn nnnnnnnnnn nnnnnnnnnn
nnnnnnnnnn
510121 nnnnnnnnnn nnnnnnnnnn nnnnnnnnnn nnnnnnnnnn nnnnnnnnnn
nnnnnnnnnn
510181 nnnnnnnnnn nnnnnnnnnn nnnnnnnnnn nnnnnnnnnn nnnnnnnnnn
nnnnnnnnnn
510241 nnnnnnnnnn nnnnnnnnnn nnnnnnnnnn nnnnnnnnnn nnnnnnnnnn
nnnnnnnnnn
510301 nnnnnnnnnn nnnnnnnnnn nnnnnnnnnn nnnnnnnnnn nnnnnnnnnn
nnnnnnnnnn
510361 nnnnnnnnnn nnnnnnnnnn nnnnnnnnnn nnnnnnnnnn nnnnnnnnnn
nnnnnnnnnn

510421 nnnnnnnnnn nnnnnnnnnn nnnnnnnnnn nnnnnnnnnn nnnnnnnnnn nnnnnnnnnn
510481 nnnnnnnnnn nnnnnnnnnn nnnnnnnnnn nnnnnnnnnn nnnnnnnnnn nnnnnnnnnn
510541 nnnnggtgat actctgggat acatagaata ctgagtacct gcttggaaag agcaagaaaa
510601 tcaaagacat gtagcgtaac tgtgaaatac tgtatatgaa ctgtaataag tgtgagattc
510661 gttttctttg gcttaaactg gagttttgcc tttaaaaaaa ggattaaaca gctgaagggg
510721 ggacacaaac atcagacact aagaagatcc catcttcaac acctcagcac ttccaaatgt
510781 ggcaaactcc ttcctccaga tgcttgaggc caatttgcag cttcgctatc gctgtggctt
510841 ccttcttcat cctcccettt tttcttattc ttaaatttta tcttttattt tcagatgact
510901 tccaggacgt cacagggcac gaaaccgctc ttctttccac tcgccactct gatgaagccg
510961 cctcgttcgc actcgctgct cacacaaatc tggggggggg agaaatgcaa ctcaggaaaa
511021 catttccgtc cggaaaatac aaacacgcgc ggtcattcat caaaaacaaa tccaagtcag
511081 taataactca cattagcaat aacggagttg aggccaacag catcacagct tatttatttc
511141 tgagcgcccc cccccccccc ccgggtnnnn nnnnnnnnnn nnnnnnnnnn nnnnnnnnnn
511201 nnnnnnnnnn nnnnnnnnnn nnnnnnnnnn nnnnnnnnnn nnnnnnnnnn nnnnnnnnnn
511261 nnnnnnnnnn nnnnnnnnnn nnnnnnnnnn nnnnnnnnnn nnnnnnnnnn nnnnnnnnnn
511321 nnnnnnnnnn nnnnnnnnnn nnnnnnnnnn nnnnnnnnnn nnnnnnnnnn nnnnnnnnnn
511381 nnnnnnnnnn nnnnnnnnnn nnnnnnnnnn nnnnnnnnnn nnnnnnnnnn nnnnnnnnnn
511441 nnnnnnnnnn nnnnnnnnnn nnnnnnnnnn nnnnnnnnnn nnnnnnnnnn nnnnnnnnnn
511501 nnnnnnnnnn nnnnnnnnnn nnnnnnnnnn nnnnnnnnnn nnnnnnnnnn nnnnnnnnnn
511561 nnnnnnnnnn nnnnnnnnnn nnnnnnnnnn nnnnnnnnnn nnnnnnnnnn nnnnnnnnnn
511621 nnnnnnnnnn nnnnnnnnnn nnnnnnnnnn nnnnnnnnnn nnnnnnnnnn nnnnnnnnnn
511681 nnnnnnnnnn nnnnnnnnnn nnnnnnnnnn nnnnnnnnnn nnnnnnnnnn nnnnnnnnnn
511741 nnnnnnnnnn nnnnnnnnnn nnnnnnnnnn nnnnnnnnnn nnnnnnnnnn nnnnnnnnnn
511801 nnnnnnnnnn nnnnnnnnnn nnnnnnnnnn nnnnnnnnnn nnnnnnnnnn nnnnnnnnnn
511861 nnnnnnnnnn nnnnnnnnnn nnnnnnnnnn nnnnnnnnnn nnnnnnnnnn nnnnnnnnnn
511921 nnnnnnnnnn nnnnnnncgc aacgcatgta aagtgccagg caaaaattag cataagcggc
511981 tgatttggcc cccacaaaga aaattgtgca caaccctgtg tgtgtgtgtg tgtgtgtgtg

512041 tgtgtgtgtg tgtgtgtgtg tgtgtgtgtg tgtgtgtgat ggagccagct agtgtcttcg
512101 tgtccttatc ccttgtagga tttctcacag aaccaggaac aaataggtac ttaagttgaa
512161 ggactactca tgacaaatct gctctttccc aggagcacat gtctaagtat ggtgcaaatg
512221 acatcctatc aatcacgctc taagcacggg ataaaaccag tttcaaacta gagtggatat
512281 gataacacca ccacaaagac ggaggcgcaa gcatctgccg tactttccag taaaaatgtc
512341 caggactaca cacacagaat atttttgtg acatggacta aacatgaaac actgtaaaac
512401 tacattgagc agtgggataa atataaacac actaattggt gactgatgaa cgactcattc
512461 atggactctc ctgcaggacc gatgacaatg atttaaaatg tcatccgcag aggctgtcat
512521 tggtacgctg ctctcaactc aaaatgtgcg caagcttgta gcagatggtg gtttatttac
512581 tgtctgaaat acaactgtgt ttgtgtgtgt gttgtgtgtg tggtgaacag ctttctcctc
512641 cctgtcaata ttaataagct ttccctaaga tgcttgttaa ttaccacctc cctaattttg
512701 gtctttcatc atgcacactg gctgaagcca ccatgttcga tgtcaagtac tcaggtaagg
512761 gggaaaaaaa ctaattagct tccttgtttt aggatgtagt ctcaatggga atgctcgtgt
512821 gaggttaact aatccgtttc tggaactgtg aggggtgtga gtgtgtgttt aactcacctc
512881 atctccagat catggctttc ttgtgcagag aagctctgca gggccacaca ggtgttgagc
512941 ttgaacacat ccgtgtccag ctggtggtgc ttcaactgta cagcagacac acatgaaaac
513001 aggtaaacac ccgctccagg aggaaatgca gcaccggcgc acacgcaaat gtcatttttg
513061 tcaccgttga gtgttgtttt ggcactagca cccgcaggag aaggctgcgt ttcccgagga
513121 ctgacagacc gaggagatgc ttaatttgct atgcagtctg ttcttgttgg gggtgagtgg
513181 gaaaatacag gatccggatt ttgtgtgtga aagagataag gcaagatggc tgcaggtaat
513241 taagagggag acgtgtcacc ataccagcac ctgcacatat ggctggattt gccacacaca
513301 cacacacaca cacacacaca cacacacaca cacacacaca cacacacaca aaatgtcctc
513361 acttcccaaa aatgtctcca ctttattggt agactgaaga ttttggtact cagtgtgtag
513421 aatgtagaag aacatctatc tatctatcta tctatctatc tatctatcta tctatctatc
513481 tatctatcta tctatctatc tgtctgtctg tctgtctgtc tgtctgtctg tctgtctgtc
513541 tgtctgtctg tctgtctgtc tgtctgtctg tctgtctgtc tgtctgtctg tctgtctgtc
513601 tgtctgtctg tccgtccgtc cgtgtgtgtg tgtgtagagg gagtgagaga gagagagagg

513661 gcatgtgcat ctttaattca taaatctaag acggcagtag tccattaagg gctataaaaa
513721 gcgagttatc aaacctcaga tcattcttaa ttccaatgaa gagccagaga aaggagacaa
513781 gaacagatga agggtgtttt ggcctccggg gttccgctgg cgagatgagc tggctgatag
513841 atgagttggt tttaagagcg tctgttgcct caggttgcaa acaaaaacat aaacctcgtg
513901 catcccattt cagctaacta agagctgttt actggccttg agaggttctt gacgctgggg
513961 gagtgtgaaa aagctcagta gaaggtcggg gcaacatcaa gaaaacggcc tctcgtgtta
514021 actgatctat cggacagaga actggggttg gaaggaaaat gggagtccag cactgctgtc
514081 acttaggccg gaggaaaggt cgacaaacac aacttgtcat catgtttgat aattgctgaa
514141 cagctttagg gtagatcacg tgtcccagag gtccagagct gtggattgtg agtgtgtagg
514201 tgggagggcc ggctaaagac gctaaagcca tctataaact atctgcacga gcagaagctt
514261 cctcactcgg cactgttttg gcagcactgt tttgctctcg gttgatttcg ggtagtttaa
514321 cgctgaacct aaactgtgcc aacagatggg gatttgaaca tggccgcccc tgctcccgtt
514381 tgggcagttg actcccccgt ttcaaagtgg aggggctccg tctctggcag aggagtagtg
514441 ttttgagtgc ttctctccgc tcattacccc acagagccgc catctgtccg tccaccagcc
514501 tggtctgtcg gcggacagac aaacagcccg ctgccagctg tgttcaggcc agaccgataa
514561 cagcaattca gcctctcccc caaaatggac ggactactga actgttctgc agagagcagt
514621 ctgtgtttgt gtgggtcggt gtggcccgtg tggttattta tttatttatt tttggtatgt
514681 ttcaatgtct gtgtcttttt ccactgttga caatcaagat aaacacgcta atctaaacag
514741 gactgctggg agaccgtagg aaaggcggcc atgtccaaag aacgttgatg ctgaggacac
514801 ctttcccaca atcccgctgt gcgaatggga acaagcaatt ctgaaatcgg cacatgaaac
514861 attcaacgtt gctcttcggg ggatggaatg ctcttcaggt gggaaggttt ttttttttgtc
514921 tcttcctgtt ttcctcccat ctcctcatca cctccgcgtc tccactgcag cgaagagaga
514981 cagagctcag agccgcacgt tcgcgcacgg ttgtcagccc ggcgtgattt gttacggcag
515041 gaagaaaaga tttccatcag agtcctggga aatgcaaccg tgtggacgtg ggaggggcct
515101 cgtggacgcg ggacggaccc agttacccag agaaaccgca cagagtttcc agccaacgga
515161 gccggttctc gcgccagtaa caagcgtttc agtcagtgca cattccagag aggaaagtgc
515221 aacctcaaag ttgctgggaa gacagacgga gccttgacat ttgaacaaca cgagcagcag

515281 attgtcttta aataagacag ctggggatgg gggggggtg ggggggggc
atatgatggt
515341 ttttatatga ggaaacctgt ggaggtgagt cagagactat atttatccta
aaataaatct
515401 tttttaatta tcaaacccag caaagggtcg ggaataaata catgaatacc
acgaactgat
515461 atgttttcat ttgacctcat gctggtgtga ttaaagtgaa agagaaaaga
atatgaagaa
515521 gagcagattt ctcacctgaa aactgtctgt ttttatgtca gcctggtcaa
aatcctcagt
515581 tccattctct atcactgcaa acactgaaga aaaagttagt cagcataaac
acaaacaaac
515641 atgacgccgt gtgtttaatt tccacaaagt gcatttgaac cgtgtcgtgt
tgacagtaaa
515701 cgcatcgtga aatacaagtg acagacagca aagaaactga aacagcaacc
ttctctgact
515761 ctttttgtg ggcagtaata gaaataaagg agtctgtttt agtatcggga
cgctcagctg
515821 agcagcacag atacgcacca tcatcactgt gtttccgtga cagctcctgt
cggctgctgt
515881 gagccacgac ttcctctgga actttgtcca agtcgctggt ctgcagagat
tacacagacc
515941 acataaaact gggaagacag atttaggtaa agtggataca tataaacaat
acataataac
516001 tctgagagac agtttacgag gccggttctc cgcaaacaaa tccgggtctc
gcttcaaaat
516061 gattcgaatt tgccactttt ctcacatgaa cctgctgttt ggaggaaaca
aaatcaaaga
516121 aagtggagca aacatgttgt ttctgggctg ccatttggaa actattatca
gatcccctaa
516181 tgagatcaca ttgtctctga gctaagagcc tccacaaaca cacactcaga
ggatatctgc
516241 acatgacagg acaaaatggt cgaaaccaaa gcgctctcag atgtcaggga
caaaccaaga
516301 ctgcacgtac agctttttgg ttgggtaggt ggggggaggt gtgtgggggg
tagctgtaca
516361 atcagcccag accatcttca gagctgtaac atgagtgttt gcactgacac
ttccttttca
516421 tcacaggctg aaccagactg cctttattgg tagtgtttac agaccagcgg
tggccctatc
516481 tcattagacc atcagctgca caaggtctgt tgcagacaag acacgacaca
ctgaggactc
516541 tgatggtgtg taagacactg agccttgatg taggaaaatg cacttttatc
ccgaattccc
516601 aatgcttctc agcgttcccg tctaaatagt taaatcgttt cttaccgaat
ttctgtgtgg
516661 agactcggag ccgctggccc tcttggcttt ctgtgccagt gacgtcccga
acctcagggc
516721 ctcgtaaaca gggtccacct tattaccgca ggcttcaggg aagaatggag
acaaatgctg
516781 agagttgagg cataaagcgc ggcgacggaa cggaagcggt tactattacc
gatcggcatg
516841 acttctttga tgcagccgta ttgttcctgg atcaataatg gagagctgta
gtaccgccga

516901 aagccttttg gctgaggaga gatgcagagg agaaaacagg gtgaggcaat agttgctgga
516961 gaacaaacgc tgcttgagac aaccgcctga aatggaaacg ctgctctgcg actgctcgtt
517021 tgagcaagat tcttaagtaa tcctgcaaaa tatcatgcag aaaatgcaaa ggaagctaat
517081 gtttttggaa ctaatctaac atgacacaaa tcctattaca gtgtcgtagg atttgtagca
517141 gatgtgtgtc aaggaatctg caggaaataa taaatgaact cttaaacata ataatgaatc
517201 atgagttctt ttcagccaca tcatttcttt tttgctagtt tacagcctgt gagagcttca
517261 cgccagatca agaggaaaca tctgacagtc aaacaagagc ttcaacacaa atcaccagga
517321 aaaaacatca ttgtgattta agagcacctg gggtttttgg agtggaactg atattttaca
517381 actaaatgaa tctatcactt gcatctaatt ttaaacagtc ttataaatcc aacctggtgt
517441 tttcagtcag ttagaaacaa actgaagagg tgctgatgcg ctcacaggtc ccgaagatta
517501 cttaatttaa tcgctaaaag gttctcaagc caagagcaac aagcacattg cacaagtgtc
517561 caagtataga gttagctgac tgcagccgtt ctccgctggg gaatacgttc ctttttattc
517621 ttgacctctc aacctcattt tccactcctg agaacagacc atatctccaa gcttcatccc
517681 ccaggccttt cataactggc caccgctgct aaggctgcgc tgagccgtcc gtgttgatcc
517741 agtatttcct ccatttagca aaggaacctg actgatatgt gtttcgttgc ggaggaacaa
517801 ggcgggaacg gctggaaatc acgcttaata tgacccgacg gagctccagc ctcgcgctgt
517861 tccgtgtggg acgagtgtga acgacagaaa gagcaagagt ttcagctacg gaagtgaagt
517921 tgatccatgt ttccggcttc acacgctgac aaactctcag caaacaaccc cccccccccc
517981 cccaaaaaaa aacgaactca tcgatcctga tacacagtaa tggtttctgc tgatggcagc
518041 gtggcgagca agggtcagtg ctgctgtatt agcattatct aacgcacagt tggagaggtg
518101 acataacgat gatgacggag attaatggct gccacagatg taacagcagg ctcacacgca
518161 gactgtgatt tctgaataag cacattccgg tgggaaacga atgccggttc aacctatttt
518221 ggttgctaat gacatcagtg tctataaagc tgaggttcac gacgaaggca ttctactgcc
518281 ccttccgtgt gcttcttagt gtgattccgt attgtgcgtc cttaccagtt tgcccatgca
518341 gcgctgctgc attcccactc tgttctcaca tttgtggtgg aggctcattt tgcaggcttt
518401 gcagcggagg ccgtgctttg ccgtgttccc tgccaggaac accacatgct tggcggtcgt
518461 acctgaaaca cccaggaaaa aaaagaaaaa acggttcacg ttcacgtcag gagctggact

518521 tcaatccact ctggcccgaa ctgcaattta ctccaacaaa aagggatgga ggaaatggaa
518581 aatattattc agaattacat cgcctgtgga acaatttagc aaatgtgaca cgcgatacac
518641 acttctgcga ggccctttc gaccagaccg cctgtaatcg agccatctcc aacacgtgaa
518701 accaggccaa ccctttacag atgtgcagcg catctcgact tttttttaat tattatgatg
518761 tatcggctgc agctgcgggg ataaatgtgg aatttttgga ttgactcgtt agccgcaagt
518821 tacgcaacgt cacaagtgca gaaaaatcac cacgtctgca ggtgtgtgga agaagatgac
518881 gtgtggagaa agatgcgacc gcagaataaa ggctgtagaa acagaggtgg ggtcacgtga
518941 cctcgtggtg atgagcataa tgtcaaaaag caaagtcacc aacgtgcagg cagcagcggg
519001 gcatcgactg acactcagaa tgttagagcg tcacagtact ggtatcagat gcagcctggc
519061 agctacgctc tggctcttta ttgattaatc aaaggtcacg tgatggtgga aatgggagtt
519121 attgagtgac tgttgggcac atagtgaaaa acaaaacaaa acacattcta ggtctccgtc
519181 aagcctgatc acggttaagg tgaccgagct ctgagctgag cagagccgca gcatttgggt
519241 ctggatcctc ctcgacacgg catgatgagc ggagagccgc tgttcggggg gagagaggta
519301 acgcggtatg accagtgtga aaccaagcgg aacacctgat ccctgatctt gacagcgtac
519361 gtgggataag gtgcactcgt gtacaagtgc agggcctctt gtgtctcatt tatctgtcca
519421 aaagggggg aaatagttgc tcagcagggt gtaatgtgaa ctggtgtcac ggctatctgg
519481 agccatgagg ctctgaaact caggaacggc tggttaattg gctgtgcatt tccaatttta
519541 agctcacgtg ttgctgtgct gggtcacagt gacacgggcc tggagcggcg ttcattaacg
519601 tgagacgaaa tctgagaggc gcgtcagaca gccgcggatc acatcaacaa cagtggcggc
519661 gggctcaacg tgcgtgagga tttttgggtc agagcgaatc aagtccgctg acgccacctc
519721 acgagcaacc agagagctat tctccgctct taaggtcagc aaaaaagcaa gattactgcc
519781 cgcgtctcca gatgaacatc tcaggaagca aatgaaggat tgagggcgga gtgatgagct
519841 tgtctgaatt cgctccatcc atccatgttg actacagggg acaaaaactt gagctcttct
519901 tccagaggtg aggggtgcag ccgtgtgctt tccttccggc tcagacccaa ctggggagcc
519961 tgaaggcatc acggccgtgc cggggggtaa aaggcattag aactccacca agtctccact
520021 tctagacctt cttggcatca cctcctcttc tttcccacac taacgttccc actctttaac
520081 catgcaagcg ttctcccctc acaagaacta aacattgatc cattaagctg ctgctcttca

520141 cgcctccttg ttttcttgag caaataacct ccaccatgac aaaagatcct ccacctcctt
520201 ttcttcttaa gctcccccaa aacactgacc tgagcagcgc tctttattgg aaggctttaa
520261 ttagcttcac ccacagaggt ctggattttt tttttccttc ccacattgta aaacacattt
520321 ccccatctgg ataattagag aaataaagcc taatctgcct tataggtgca acaaatctgt
520381 ttttatggat ccgtctgcga tcaaaggctg acatgtgcag ggaataaatc tgtgttttta
520441 agaattacat aggctaaggc ccagatggag gagtgcagtg gaaaaactgt tttcattctt
520501 ctctgtcaaa ggttgatgat gtgtatgttt agtatagatt tgttgtgtta taactgaata
520561 tacaattgat ttataattgt cacgggttca cctggactac ttcagtggca ttatggacat
520621 tatgttttat tttatgtgtt ctataattcc tcttaacttg cttgtttggg ttttagcttt
520681 cctataagcc ctttaaatga tttacaaata aatataaatt ttgtagatgt agatgtgtgc
520741 taaggctcct attgtccttg aaggctcagt aaaaatctgc tataaatgat gaaaatgtgg
520801 tcatttgtta taagtgaccg ttcagtttta atgaagacgg gcttttgctc tttgaccctg
520861 gacacacaca catgtacaca cccttctatc tttgtgagga ctttcacaga caatacagac
520921 aaatcttctt cccttaccct aagcatcaca aaagcctaac cttcatccta accttaacct
520981 aaagccaaat gtaaccataa aacctaaaag tcttaaccct taaacagccc attgaagatg
521041 gacaggccaa aatctcccaa ttctccccaa ttccctaaaa cgtcctcact ttgctattga
521101 atacatgtcc ctcaatatgc cacatgtacc caaacacaca aacaataaaa caggacaaaa
521161 gaagagggca gcacagcaag aggagcagac cacaggaact atcagaaggc cagacaaagc
521221 tgacatcaca ctttaattat taacccgctg ctggagttct atcccaagct ccggctccag
521281 gatgtgcctc catgaacaca caccagaacc agtgtaaaca acatgcatta aaatgtttta
521341 cttgtgtgca cattgagaca cagacactcg tcacacttgg aacacctatg tcgctaagaa
521401 tccagactac tttatagctt tgtttgacag ctccagtaaa cttatattaa tgtatacgtt
521461 tccatcaaag tgacaacctg gacagaaaac ccattacgag cggcacggca gcagcgtgca
521521 caatcctcct cccagaacct cggtgacaac agcccattct gctcgtcagc aggttgagtc
521581 acacctgaga cgaatgtgtt gctgccgcag gaagggtggg tatatttaaa tactggattt
521641 ctctttggaa attacccata taagaccgtc ttttcaatta aaaatgcttt ttttaacctc
521701 cgaaaaacac tgggggatta ttcacgctgt ttaacgccat cttgaatgtg caataagtgg

521761 ctcaatgatt gtcaaaccct taaagactaa ttccatctgg ttttatcgcc atcatcaccg
521821 tgatgctcta tcgtgatctg tggtgataaa aactaagtgc tccctcagtg ctgagagtta
521881 atggggctaa atggggacat ctgttgagga cagaggtgga ggtggcgcca caacaggtgc
521941 tatttacggg cccagcagcc ataaggagag agctggctct cgtgcaaaaa acaaaaaagt
522001 gagaaatttc tcagccgtga tgaaacgcag ctccttcctt cctgtttgat tctgctccat
522061 ttctcttaaa gaaaatgggc ctttcctgaa actgctgccc aacttcccat ctgctacggc
522121 ctcatcagcc actgaatctg aaggattttc aaccctacct acaatcatgt ggttgcagac
522181 gtcgcagaac gtgggcttct tgaagatgtg ctccatgaaa cagtgacccg ctgagtctac
522241 ctgcagcggg ttgcgcgact gtgaacgcga ggtgggagga ggcgcgcagg ggatggccgg
522301 aggaacaggg ggaatgggga ggttggcgtt gtggggcgga gccatcccga tgctgcagag
522361 gtggccggtg ctgccgctca cgtccgacag cagctccgtc ttgttgtcgt tgctgctccg
522421 gaagaagttg tcggcgctct tgctgcggat gctcttggtc ttgaaggaca gggagcgctt
522481 gagcctctgc agctgaaacc agaaagaaag agtcgaagta gaactgagca agacagagta
522541 ggaagaggga gaaatcctca aatcataaaa cctggttgga aatgattaca atttctctgg
522601 tgaatacatg cagtgttgac attttttcaa ggttccctac ctggaaaata atatggtcct
522661 ggtctttgat ttgacaactt cccccatgct gtgccgcagt ctatcagtct cattcatttt
522721 tagcagtaat attattcact ccactccaca ttggcagctc ctgatgtgac cttttctctg
522781 aaaccctctg aatcttaaca tacaatgaca atgcctctaa aaagtcctac aatgacctct
522841 aaagtcttca gcttagacac gacagcaaag aaataaacgc atatttgggt gttctgtcat
522901 gtagtctgct ttttgaggcc ttcttcctga aagccacagg cagcgacaga aaatggggtg
522961 agtatagaaa catttacgac gtgagctgcg gctatttttc aacttggcgc cttaacgaac
523021 tgtgcaggaa cgctggtcaa tgttgctttg ctgtggtgga aatcaatcaa ttctgtggga
523081 cactctggaa acaaaatcaa tgtggatcct ttctctacca ttaaggtcac acacacacgc
523141 acacacagac tataccaccc tacagagata tataacaata aaatccacaa ctctattgct
523201 ggcagttcat ctacagtatt cttctttaca gctggacttg tggtcaatag agacttctct
523261 acatcatcaa agtgaaagac tgcgtccatg catggaggag ttcatgctgc gttttgacga
523321 gggccaaatc tataagagcc gtgttcaata cgtcagggag ggcgacggag gcggcacttt

523381 gtgatcaaaa gcaaacgatt accacaaata cacagtcagt gctgcggcca ttgaaaacag
523441 agtgcacgct ataaaatatg agtgctgggc ggtgacgatc gggcccaaaa tgcaataaaa
523501 gttgcaactc ggggcaactt ttatctccac ggacatgcag gtacagtgct gtgatgggct
523561 gggtcgtatg ctacgttttt gcacaagagt ggaggctcac cttgcgcttg tgaagttcag
523621 gcctcacagt attcttcaag gaggaataaa aacacaaaca tggcttttac tttgggggat
523681 aatccataat acatgtattc taccttccag tctgtgcgaa ggatgagaca cttaaacacc
523741 ggcttgttgt attttggagc tgtatgataa agtgagctgt ttggcagcag cttttgaagt
523801 tatgtgagaa attcagtccc ttaaaaaatc ccagtgacca aacatgccaa agaaagagtt
523861 cacatgcaac agagatcatc tgtcatcctg actggaagct gggaaaaaag gacaggttga
523921 acagatgtat tcattttaac tttacagaga atagatcaaa taagctacct gtgtctcaga
523981 gcatcagtag cgaccgttgt gacatgttat ccactatttc tgtggtaatt gctgtattta
524041 tggcaatttg tggcccagat agatggcaaa atgctcgagg cagctgaggt tctctaccaa
524101 agcctatttt ccaacttcat tgtaaactgc aaacatatac gccaataaat ctttaatatc
524161 tttctaatat aatgcctagt ttgtaggttc cactcccaca atttgcattg tgttgtttta
524221 ttatgtttat ctggatagtt gtagtgacat tatttagact tttaactgtg tggcaaaatg
524281 ctgtgcaata ttttagtcta gtttaacaga gaaactgtgg tgtaaagtaa aaaataaaac
524341 agttttatgc atatcaggaa caataaataa caactggatc agcctacctt ggtttcctgc
524401 tgactggcgt tcccagtcgg agatttggga gtctcattct gctcctgttc ctcctcgttg
524461 cccccactgt cgtcctgaat catgttggac ggtggtatca tcctgttttt agggatctaa
524521 aaaaaaaacg gttatgtaga gttcgttctg tccactttgt gtttacagcg ttcccattac
524581 tcccagtcgt ggtgacccga ggaaaaagaa gtcatccaga tctaccgagg ctgcgacgag
524641 gaggtttgag tttcatctga aaaaggatca gaactacgtc ttgaggagtt tgccaaggac
524701 gacttttccc caaaacaaag cagttaaata ataagtccag atcgccttca aactgttctt
524761 ctacagaagg ctgtctcgat cctggcgtcg tgcgtaacgc tgccaagaca tctccaagtc
524821 cacgccgtgc gccgtgcgca ccacaccgcc ggcgctatga tgggcggaga gatgtgaacg
524881 cggagaaggg aaagacggga agagaagaaa ggaggaggaa taagaggcga gcgtcctgcc
524941 aaaactgaga tgagggcacg ttcatgtggt cccactgaag cgccgcagca acgtcaacaa

525001 aacgaacaga tgttttgcct aaaagacgga tattttaaaa aaaaaatcag ggcacatcta
525061 atacttttat ttacacgaac aatcaagtcg cttgcgaaat gaagttgttg tgtttttgtt
525121 taatcattta atcagtctaa actgagtatt tttcataaac aacccctggc aactgaagac
525181 tgcgtttaca cattgaacat gaccggtcat ataatttttt aaaggttggt taaaaacaca
525241 atcttttcca tgttcatcaa ccaaaatgat atattataat tatgtttaaa gttagggaga
525301 gggaagagga aggaagttag gggagaggga agtctgggct tctctcctta ggctgctggc
525361 cccgcgaccc gaccccggat aagcggtaga ggatggatgg atggatggat ggatggatgg
525421 atggatggat ggatgtttta agttagcctg atcaccatat ttatatgtat cccgcagtta
525481 agttaaaaaa aaaaaaaaaa aacctttgg gattgtaaaa atgtttgttt tacttttatc
525541 atttaaaaaa gtaactagta aatgtaaaac attgtcaccc aaagttcaaa tgtgtatcca
525601 ttttttaaaa aaataagaga tgacatgagc cagacgctat gctaagctag caacaagata
525661 gaaagctagc agatagaaaa catgccagtt aaaagttgac ccatatttaa gagtgaagaa
525721 aacactggca gcatttgtcc tgctttagtt gggataagca tcttccctag ttgtaccttt
525781 tttactatag caaaagaaag aatcaaaata aacttcacat ttacttcagc aatagtaatc
525841 aggaggtcat gttcaaagat gaaactatac aaatgagtca ttttacagag agtaaatgac
525901 tcaatttgac acctcaaatg agccgcagtg ctctgctgtc atcccagcac cagacagtga
525961 caatcgatca tccattcata ccacccagaa cttgaagcac ggtggtatct gtgcatgagt
526021 gcatgtcctg catgtgcgcc tgttaatggc gtcagcagaa aaagccaaat gtttgaccag
526081 aagccagaag acaaagctgt gagtctgagg gacggcgagg gagcattata atttcctctc
526141 agctgccgtc acgagcgcgg gatgagatta gggaggaaga tggagaggtg gaggaggagt
526201 accagatgat gggagcaatg gaggttaatt attgcaaatg aaaggtttca ttaaagcagt
526261 gtgaagagtt gggttgagtt tcagccctgt ttatacactg caccatctgt ttaaggacta
526321 ttaaacaccc tagtgtgcct ttctctttgc cgagaagtcc ccaaataacc ctacaattcc
526381 ttcattttgt tccttaataa cgttttcctc gaatgccttg aaatcaccac attttccgac
526441 tcccccaata cggcctgtat gtgcaggagg cttgttggtg tattttgaga cgtgccagaa
526501 tgtgttcgtt tgtggtgaaa attgtgtggt gtgacttctt gcgctttgct gcttgcaggt
526561 ttctgctgca catttgtagc tggctgtttg agcacctcgt tgcatctgta ataatttcac

526621 acatagatgc gctgtttttt tttaaaaaaa acgtgtcacc acaaatccag tggctctctt
526681 tgctagattc attcacttcc atttaaaatc aattttgacc cttgttttgc atcaatactg
526741 aatgtttatg tgcttccagg acctagcttc agcccgacca cagaggaacg cacaagtgct
526801 gatcagctct tctaccggag gccacatgag tggaaaatgt ctccgccatt gacgagattc
526861 ccaagtcacc atttgtgggg taatgagcag ttttacatta catcacaacg tggtttatta
526921 gagatcatct tatgttgcat aaactgagct actgtgagcg atttaagcag gctccatggc
526981 tgtgaggaca taaaatccca tttttagctc tgtcacacac tttcacagtg ttttctcagc
527041 tactcttaga cgacatgtta tccagctatt tctgggggt ctgtggttaa agacacggca
527101 ctttaggtgt caagaagaca acatcatgga gttcctcgca gaagaaagag aataatggag
527161 tgaatggagc acaagaaggt ggctgtggtg cattccttgt ccttaataac gttcaacgaa
527221 catgggatct atattaacct gagctgtgag tgatggcagc aagctgcttt aacatttcac
527281 acgtctccca gcatgtgtta cagtaatggg aaaattgatt cccttagaag taaacctaat
527341 tggctcatct cattacggct ccttctagaa atagtctcag cagggtggat ggcgatagac
527401 agagatggag aagcccacct ccctcccccc gtgggggttg gaacgcggtg tgtgtcctcc
527461 tgagcgtggc cttggtctgt acctgcggtc tctaaagctc cactgagcga cgccccctca
527521 gccactgcgg cggttttgaa ttgtctcagg tctaccgtcc tcttctttga cccggggatc
527581 tgccgctgac ctgccctcag catctagctg ggctccagct ggtccttttg gtgtctttgt
527641 gcgcaccagt tggagtaaat gagacggtag atgccaaaaa cagctcagaa ctgtgactca
527701 gacgagtcag gttcacctgt cactgttgct ctgatggctt gtttgtgcgt cgggggaaat
527761 ttatcaccta cacaaaatgc cctcgcagtc caagcagtcg atacattacc aagacatctc
527821 actggaacta ccccccgcgg taaattataa cttcacatat gtctgattaa taatccatcc
527881 attttctgca gcttgtgtct aattagggtc atggggccgc tggagcctgt cccagcggtc
527941 gccatgccaa ccctggttac atcagggaga gggaaccagt ccgtcacaga ggctccagtc
528001 aacccgatgt tcgtgttttt ggactgtggt tgcaaactgt acatcgggga gaacgtgtag
528061 aaaagcccca gatggaactg aaccagaagc ttttggtttc ggctgtttgt gatttcaact
528121 atcacattcg tttctggttg gttgaataac aaaatattct taccgctttt aaaattcgac
528181 taatttctaa ataaactgca gcgaagtctg cgaagtttag ccaaagcttc tcttaattat

528241 tgttagaggg cgccagtttc cattaaacac tgaaatactg aattatgaaa ctataaatct
528301 taagtcatca tctctcctgt aacaaatgct gatacatgac cataatagct ccagttgatt
528361 cacacagttg gcctgaagtg aggtgtttta tttatccccc cgccactacc accagccggt
528421 tcagccgagc agggtgattt attaaagatg ggttccagcc tctgtgttaa tcacaataaa
528481 acgcagcttg ctcgctttca tcagcggcag agccagtttt tgtggtaaca aacggggcca
528541 gatagtgtgt gactgctgtc tttgctcctc tcagcgttgc agcgagtgca atgttgatgc
528601 aacaccattt ctttcaaaca cgcaaaggct tctttctcgc cttattagcc accccgtcac
528661 tgtttttcct gtgttttgat gcacctgatg actgctaagc taagcagcgt ggacattttc
528721 catgacatga cctcctaatt ttatcactac ccacaggagt tttgatgctg ccctagagac
528781 attaataatt ccttgtggtt tatatttgtg gccatgcttt ggaacattgg ttaaaatagg
528841 aaaagctgat gaacaaatag gaatattagc catgtttaat cacagaatga tacaatatga
528901 attcaacagg gataatatgt aagtttgaac atgagaagtt attccttgtt cttaataaca
528961 acagtttgtg taggatctgt ttcatttatg actgttggcc atgatactgg gacaccagct
529021 gggactgaca aatggccctt atcaatttct tatcaaaacg tgactctgac ccaaaaagga
529081 agtgatgaag gcatgatgtc tgtcttcctc tgggactcaa attctttacc catttctagt
529141 tttaaaatgt gttctgacca ggctgctgtt cctgatgtaa aaaaataaaa agagcctttt
529201 tgtcaaaccc ccagcccttg gatgactaat agcttctatt agcagggcgc aatgatcaga
529261 aacgcatcgc agacaatgac ttcccataaa gaaatgaaca atttttgaaa atttgattcc
529321 atttttttgt tgcggatggg atggaaacgg tgaggcgctg ctctcactac tctcgcaccc
529381 aaagctcgag gccagagatg agggctgtga tggagctaaa tagagagctt ggccctgcgg
529441 gtcagcagcc tcttcaccag tgtcggatgc agctgccacg ctacagcggc ccctcccgta
529501 gacgcaccac gggtgtccct ttggatcccc aattagaggc tcacttgcaa aacacacaaa
529561 ggcattatta ttatacaaat cttagttcag taattagcct caaacatgca gttagattgt
529621 ttttagcttt gtcgcatttc tgtgccggca ttgccaacca gctgcagctt tttgggggac
529681 gtaaaataat attttatctg cacaaaagca tccggcacaa aataactcaa ggttctttcc
529741 ttcttttgat agaaatttca atctgctttg cattttgagc tgacaaactg ctcttgaaca
529801 gcagcaacag gttcaccaca ataagaggca gggccatata taagtgctat ccacagttcc

529861 acttgtggat ccaatttgct ttccatcaca aagcagtcag gttttcaaca actaaccctt
529921 actaggctaa gacagaagcg ccacaaaggc tctcaaaata gaaacaacag cataatttga
529981 atggagctgc aggcgctgtt gtggagccgt tcctgttgtg actcataagt gttggttttg
530041 tgtactgatg acgtgggcgg atgcgtggtt aatggcgggg gtgggctggg ggaggtggcg
530101 gcagtggatg catcactcgt cttcctccag acctcttgtc atccactgag tcaagcagtt
530161 gttgttatag tatcatgctg tcaaatgttc cgaagctgac acgaccgatc aatgatttta
530221 actggatcga tctcgatatt tcctctatga gttccaaagc tttgggccat tcagcattga
530281 aatcagtcca aaaacaaccc aaaggtgtat ttaaatacac ctataaagtg gcatttgcat
530341 taataaaatg acatacgggt tctggtgtga gtttaaaatg gtaaaatcta tttttctctt
530401 gccgaccaag agattaatag ctgctggagt gatctatgtc ccgcctggtt tagctttagc
530461 tcagaatgca gcagtgctca acacatttgt cagaacaatt catcaaaagc agtgtgggga
530521 gaaaaaagga actccccacc caaaaaaaac ccaatatggc gatatttcta tcaaagacag
530581 ggcgcgtcta cttaaatggc ataaagcaat tttgtctcgg aaaagtgaaa cgaaaagcaa
530641 ttccttccat catccagctc acaagggtaa caaatgagtt aacagtccga cggcctcttg
530701 tgattggagg gccgtttttg ttcctggtga taggtctccc cgtgaaagct tccagctacc
530761 tgaggagaca ttgatgaaag agttgtgggt gatggaggaa ccttgatgct tccattgttg
530821 tgccgtttgc tgctgttgag agcgactcct ctacgtgcag ggctgatgga atcattgcct
530881 ttgagccact atagagtcca gcagagccgt gtcatgactc ttgggctcca gctccagaca
530941 agtcctaaat tgttcccacc aagcaaacca ctcagcaaaa agacatttta aatttaaggt
531001 aatttttaag ggaacagtaa ttctcatgca tttgcacagt tcagctgtgg taaatagata
531061 ctgtggtgaa atggctttca tccgcttaaa atctaaaagt gatggtccag atgcatatga
531121 acctgcttta atattccttt tgcagggctc actgaatctt tgccaatgtc acatcaaacc
531181 aaggacctgt atttaggatt aataaagcct agttttgaca ctgaactcta ttaattgagc
531241 cctccacaga catctccctg catccaaaat aggtaaaaag tccatcaagg aggtgaagag
531301 ggactaagta ggccaaggac ttgtggaaaa tagactgtga gtcactgagg ggcctgacat
531361 cttgggaaag aatgtgggtt aacaataatc ctcgaaaatt aagggtgtgt gagtacaggc
531421 tccctgtgga gtgatgaagt aaggaggaat tacatgtctg tggaccttaa aagtggtgat

531481 aaaactccaa attacctgca tcaaggagtg ttaatgggaa atttacactc aacatgcatg
531541 tgttttacat cagtcattag atttatgatt gagatacgaa tgttttagct tgctgcatta
531601 aaaaacgtgc atacatctta attctttcca ccatttgctg ctgcagtgag tgacttgaaa
531661 tgcaaaacta aatgcatata cattaatttg aagtacagcc tcatcgcctc tggcgcttaa
531721 tgttggaaaa cagttgtatg tagaatttca gatcagaaaa cacgatgctc aaaacacaat
531781 tgagcatgta gcgttcgtca acagcgacac ctcgtggaaa ctattggctt cgcacagtga
531841 cgtcaaaagg aaaactgctg gtagactaaa cagttttaca acatcaacca ctctttattt
531901 acaagttatc acgacaaaac aaacacacca atggtgggca gcccagtcct aaagaggtgc
531961 agtggtgtga agaaactgaa caataaaagg gagagagaaa aaagacaaag tatggaggat
532021 ttggttcaat cattctagca ataggctaat tatctcaggt caacataact gaatttgcaa
532081 aaaacatttc ctttacttgg gtggggaaga tgggctcaaa ttcctaattt ctcacactgc
532141 aaaactggaa taacttttcc gaggccatga cagaggaagg tggagtgatg cgtgatgggt
532201 aaacatgaca tcattgaatc catgtggatg ctaaagcgga tgaataagga tggcacattc
532261 aggaaaggac aggcaataag aagtgggcta atcacaacaa tcccatggca cttggaataa
532321 atatgggaat aaaatcaggc attggaaaag gcaaaaaatc tgcagtgagg catccaaaca
532381 aatcaccata gactgcacat ctgacccaaa ctgcatgtgg gtaaaaccac atgtcctctc
532441 ctgtctcaac tgttttcatc tgcaatgaaa tcgacgaggg cagaactgca ggatgtcatt
532501 tcagattaca aaaactgtga acccgactcc catccaaatc cacgtgggag acagattgtg
532561 tgtactgtag agacaggcat aagagagtgg agcatgtaaa aaaccaataa gaagcaacga
532621 tagatatgcg acagcaaacg tcgccatcgc tcacatgcac actacgcaat acagctctgc
532681 atttacacca gagcacaaaa cgggttttca agtactcaac gctcaatcag aaattaaaaa
532741 aaaaagagca ggtggatagg atcttctgtg tacatttaaa tcacaggccg ggagcctttg
532801 aaagcaggat aaaacacaat taaagggata tttattcatt taagtacaaa agcctggtat
532861 tattgaaagt cagctgtgtt attgcgagtt gtgacattct taaaatgagt cgactcacaa
532921 cagcctgaac agctttaaac aaacaaacaa ccaaaaaaga cacagtaaaa ctaatgttag
532981 aactgaaaac ttgactggtt tcccttcaca gaaaaccagc agggtgaagt ctatattgga
533041 tcttcaatat ctgagaaaag cttattgctg caaacctggc agagagtatt tgcaaagttg

533101 tggatccatg ggtacacaag tcaggtcagg tcattgccta agaggtcagt gaatgcatca
533161 catatgataa cctaccacat gcttttcaat ggttcatata attaaaaaaa aaaccagact
533221 atttcttctc ctaacttacg cagagcagca cggtcagatt ttccttaact cttcatctgg
533281 ctactttttt gcagaggcag tttcttcttc cttcaggaca aactttttcc tcagagcttc
533341 tgaaatcaga gtggcagagt cctcttctct gagggacgtg cagcctctgt tgtatctgca
533401 gacgcccgga ggatttaaaa aaacaaaaac aaaaaaacaa acaatcaaac aattagcagt
533461 ttattaagcg tcctacctgg tggtgttatt ggtaaatggt gctctggaac atttatgagt
533521 cctacctctc tttactcatc ttcatgcggt tcatgtcctt cagaatatcc atgacgtcag
533581 caaaactggt ggactttgac agagacaccg tatcctcttc agcctcccga aagtccatgc
533641 tgggtctgtc caggtcaaag gttgccgacg ccgtcatgga gactgttggc agagggtcgg
533701 gcaccagctc agacaccatt gagacgtctt tttcatcttc ctgctcttct tcttcttcct
533761 cctcttcagt gacatcgctg atgacaaagg aagcggcccc ggcggtggcg ggctggtagg
533821 acgccgtttc gaacggtgcc atagagaagc tcatgctcgt gtcgtcaggt gagagcaggt
533881 caggtgtcaa tgggttggaa cctgaaagga agaagaggag cttgagtatt tactgaaaga
533941 tatcaaaaac cctacaaaca acaaccccaa catttttatt atttattaca ggatgtgaaa
534001 agtcttgatg tttctacacg ttttgatctt atggactcaa gcagctaaac tatgagaata
534061 tggacaatga caattgtcat gagttgtagc tcttttcggt tacatggtaa catgattggg
534121 gctaactgtg tgacaatggt gcagacaaaa agttcaataa gggacacgca tgtgccactg
534181 gatggcagag attatgtttt gacactgacc tagaaaccgt tgctaagaca tcttagtacc
534241 agagagcttt agaagaatca gggcagcagg cttgatagca gaagtatcag agccaccagt
534301 gaatatgggc tccacattgc aattttgatg ccctgatgta cacggcagcc attacacgcc
534361 ttcatttacg cacctgaatc agttgccaca atcttggcaa tctgtgcacg aagtctgctg
534421 agctcgtcct gcagggcggt agtgcggttc agtgccgtca cgccaggctt cttaagacct
534481 tccattcgtt taccctcgcg gcggaagtta ggcacagagg aattcctgtg gagcacgagg
534541 agtggtgtgg gcctccattc atgcttcaga ggacgacagt cactcctgag aacagacaag
534601 aggacgatca gtaaatcagt gattaccttt atatcacgac tgtctttccc taacctggca
534661 ccactccata aactttgttg gtgactagat acttaccgca ctctggcata tgtctcttct

534721 tcatctgcga caatccaggc tatatcagcc aaggtaggaa ccattggacc
gtccgtgggc
534781 cgaagagggc ccaatccggg taattcctta agtgtaaatt aagtggaaac
aataatttaa
534841 actgttgtga agaaagactt cctataatgc atataaagtg tttttatgag
tcaatattta
534901 cctggaaaca tgccctttgg ggaggcttga gtggaagtgt ggaacctatt
cttcttacaa
534961 cactacgagt tgagccatga ggcttattct gccaaattgg aataatatcc
tgcaaagcca
535021 attaaaaata aacacaagtc tctcttatta ttcaattata ttttgctcat
tgcaatattt
535081 ggggggaaaa cacattcaaa aatatggcta agcaaaacta aaagtaatat
ccaaaaaaca
535141 aactcacagc ttctgtttcc atggagatgg cagctcggtc tcatgcactg
agaagttgca
535201 cgctaacagc tagagctaag attggcttac tgtccagcga gtcagcagac
aggtgtcatc
535261 tcaatgagcc tgaaaatcct taggtctgaa taaacaactt aggaacttag
agcatctgtt
535321 gaaggaaaca aacaaggtag tgttaatcac gcaacttcaa tcttgtgaat
actgtccttg
535381 cacgaatgtt tgactttggc cagttataat aatcatcaac attactaacc
tgcaccaaca
535441 ttagcaacct gatgtgtacg acgcatttta catttctaaa acattgaaaa
tttatgctat
535501 tatgttgcat aacccccaaa acccattttc ttttagcaac atcacatcga
ctctgcattc
535561 actagacttc accactcaag atcagattta gttagaaagg tttgatccaa
acccttcatt
535621 cagcaaccag cagcaaataa aaatggattg aacttttgtt tcggcgtaat
ttaggcgtca
535681 cattaacaca aagaccactg ggaaataaac atcaacgcta accttcactg
cgtattacta
535741 aatctgtaga cgttacatta cattgccgaa cgggtgaaag cattttacta
tagtgattta
535801 aagaatttcc gtcctgtcat cttgtacatg cggtagtcca gcagcccaaa
ttaccggagc
535861 agcgatttag ctttagccaa atcagcagca agcccctgat tgacccccaa
gcgcccacca
535921 ccattacaat aaacgttgtt atttctggtt ttatatcagt tgaccagtgc
tgtcatagcg
535981 caattaaatt ctgtccgcaa cttacaagtt atccggccgc gatacttacc
aagcagcgtc
536041 tcctcttcgt ctttcagttg tgtttgcgtt cattaggccg gaaacctgct
tccttcatct
536101 ggactatgat tggctgtagc cccaccagct tcctacccga cagcgacccc
tatagggata
536161 aagtggtaag ggccattgtc atggcagtac gataacttca aatcattgtt
aagcatgtga
536221 ttaaagacgc gcacgcacac acatattaaa ttgaccaaat gggccgatta
cacctctaaa
536281 agtcatcatg ctttacgcgt ttattcaatg taatgtgcaa gcacttaagt
caagcataat

536341 ttgccattta tgtattagat caaaacatga acaacaaatt cgctgtgata
aaaatgttta
536401 gatgtaactt taaaatatag tttgctagaa atatatagag aaatatatat
agaaatatat
536461 agttaaatta aaattattaa tacatacgcg tccgtctgaa acacaagata
tgtttagcgg
536521 ctcacttccg ctttcgttgt tgaattggac tgaactatct tgacactccc
ttcaaggtcg
536581 ccatataggc ggagcttcac tattgaagtt gctcacgtta tttctgcagc
gatatcataa
536641 cactgcatta tgaaggtgcg ctcatatgac caacaatctt cattgctaaa
acagacgcag
536701 gttagtcaag atatttcttc gtcaattacc tgttcttctc tccggtgctg
agctgttgat
536761 gccatattct ttcctaccac agcggcaacc tcagtcattt gcataactgt
cgcctaaaat
536821 agttacatcc tcagcagtga ttatatcgca gaaggaagga agccagttgt
gttcacttgg
536881 tgatcgacaa gcatttgaaa tcttgtgact taaggaagaa tagagaaaga
agctacgcta
536941 accccgctgt tcttgtgggt tccgtggcta aaaacatcaa catccgcagg
gatctgtcat
537001 gagttgcggt tagaacgtct ctgtctcatg gccataatgc gaaacattca
taccatccat
537061 cctttattta tctgatccgg tcaacgttat tgagccacaa aagagactaa
aagcatactt
537121 atacttatag gcagattggc gtgtcaaagt ggattgaacc actggctgca
tcccattgca
537181 aaatgtgcag ctccctttct ctgcgatcct gtggttaaag gtagaaaaag
gattacccgt
537241 tttctcccct gtctgtctac atgttcagta ttttcagcca gcatttctga
cttgtgtagg
537301 tatctgtgtc aaagtgaaac aatggggaat aaaaggagaa ggaagtatac
caagtgagcc
537361 tctcatccat ctgaaaccgc ctgcctcata cggaagtacc agtgaaaatg
gctcagtccg
537421 tgaggaggcc caacactcta atggccaagg atcaagaatc ccagagggac
aagtccagga
537481 ccagctactt tggcaatgtc aagaccaggt taggcctgta aacacacaga
ttactggcac
537541 aaaatggagg ggaaggttca aggaggcatg tgacatgttt acccgtttcc
cagtttaagg
537601 cacagtacag tcaggtcttt catcaatgaa aaatctgtgt gcacatttta
gcgagtctta
537661 ttaccattca gccacttacc tcttagtcat tacagactcc tacgctgctg
aaaataaacc
537721 acactgattg ttacttgcag taatctaagg aattctcctt ccccaaccta
agaccttaat
537781 gtctgttttt ggcagtttta atggctgaat aaatagtatt tggtacctta
agtgctttta
537841 ttcgccatgc tgcacaactg cagctaggtt gcattttaaa tttgatatgt
accatttaat
537901 aaaagctatg taaagcagtg tgcaatgctt attgatctca cattttaaat
ctttcctgca

537961 actattgagt gagcataaaa ttaagttcta gtcaccgtag tgactattta tagtctaatt
538021 taatcattta ttcatcaata tctacagcac cattaaagta attaattggt gtattctttt
538081 attgtatctc actgagcaat taggagaaat acaagtactt ttaattactg ttagtagcat
538141 ccacctcagg gtttaatatt aaaaaagatt tgttttttct ctcttaggtt gggttcaaag
538201 ctccctgcag gtgtctccca gcctccacag tttgccaaga agccctggga gactcaacct
538261 aaaacagacc cctggaacca ggatcagaac cgacccattg gccgtcctct tggccacact
538321 ccctacatca gaaaccccaa gctgaggcag tactacctcc aaggtaagcc tgcagagttc
538381 catcaccccc acatatggag aggactgttg tcaagtggct ctttgaggaa tttcagcctg
538441 aatgtgtatt gatttttcac tcactgagga tctttgcaca cttgcataga ggttttagca
538501 ggcggtggca ggaactgtgt gctcatctta tttggaacca gggaactgaa gtcaacgcaa
538561 cgcaacacaa cacaccaatt gtgccttctc cacttctcag ggacgtcatg ggatgtaaac
538621 aacactgcaa cgaagcatca ggacatggag ggtgcatcat atgacaggac agacatcatg
538681 gtgagtcact tgttttctta aaatcttctc ctggcatgaa gcaaatgtgg ggctgcggct
538741 ctatgtctgg tgaggtatgt gataacaggc aattttacag tactgcgtca aagtttatag
538801 tcttggtaga cctagataaa gagagagaac aaggtacagg gtgtcagagc catccaagtg
538861 acaccatggg caagcagagc attttccaga gcctctacaa caaatacagc cggaagaata
538921 ttttttcaga tttctattca aaggtaatta tcacccttgt ttttccatct agtgccgatt
538981 gcctctgtgc tttatgacat atttagctct tctcatttgc ttgttcagcc aattaaaatg
539041 atctttaaaa aaaaaaaaaa aaaaatatat atatatatat atatatatca gtagattttt
539101 actgttcagt gctgtttaac tgagttgtct ttcacatttt atgctgagga ggtaccttgg
539161 ttattacata actgttaccc ttttgctgac cggttttaag cagataaaga agaaaaaatg
539221 gtgttcattc agagttgagc atttatctct ttcttttttg ttgtgtttgc tctccgtatt
539281 tatctgtatg tttagcattt gtcatgcaga tcttgctgtg ctgagtggat attgacattg
539341 tgcctttagc gtaaagagca ggcgtttgaa acagttaagt ttatgttcgg ggacatgtat
539401 gttaaaactt acatagttta agcctctcca ggcttactct ggcacaaaac cgcccagctt
539461 ctctatggcc tcataatccg cgaggtggct gtaatctaat ttaggtcagt gggtccttgt
539521 tttcatggtt gatagttgtg gacatggtgc tgctgttcgt ggtgttcttc aataatattt

539581 ttgctcttat attgtgcact ttttgaacta atgtttcggt tttaactgcc gtttcccaat
539641 attgtagatg cacattttgt ttctcaggat gtgtctgact atgaatgcca aataagctca
539701 attatcctgc acattaatag atattgtggc catgtggtat gcaaggcaaa caggtaccta
539761 ttgcatcaat atctaccaat gtggaaatct gattattaca ttccttctcc tgattatgtc
539821 accttaatta ttatcttatt ttaattgctt aatcatttat ctgcaggttc tgcaggtctt
539881 ggagctcttt tttattgatc atggaattac atgatttacg atgacaatgt tttatggact
539941 cttgagtact ttggtttact gtttgatttg cggtcgggtt tctatgtgac tgcagatcta
540001 caacccttat gaaccaattt aagttataat ccagacttat gtgccctctt ctttttccat
540061 ccattgttct gccagtgcag ggttgcagtt gccatggtgc gtattgtatc tagactggtg
540121 gagctgatgt ttttttggtg tatcttcttt tagggtctcc ctggggacac agtgttcact
540181 gttgcagctc cgtttaatgg aactggtgca tcctcagtgg aagaggggaa aatgggtcac
540241 cgtaccccct actcttcctc accttttttcc tcctttggtg gtgacttcat ccctagaaag
540301 gtacagatca gctcattgca caatcagata caggagcagc aaagtttttc acatgctgct
540361 gtggactaga tttgaaatgc aaaacaggac ggctgaatgc attcaggtct cagcaggaaa
540421 tgtgatgata gatcgtcaat atccctcgga atgtctcttt ttattagcaa actgatatcc
540481 tttacacctt gatagcttaa agtcaaatat catatgttgt aactgcgtac ataagttccc
540541 tcttcacaac cctcatgttt cacatgcaac caagatgcta ctgcaaacaa agagaagtag
540601 agaaaaaaac aggttgttaa aagaggaagt agcctcccat tttacctagt gtcagtgttc
540661 tcatggcttc cactccggtc tcacaggcag gtgctaatat tcaaggatca tctcagccag
540721 aactggatgc agacgcgcag gttcagcaaa cagaggagac tatttcagac tcttacaaca
540781 cactcgctgc tcagcctcag tccccttac cagaatctga ctacgataaa cttctggtga
540841 gtagaacttc tggggggtaa accctttcac tgattatttt acaaaacaat ttggggaaga
540901 atttgctcta agttttaagc atgacaagag cagcaggaga ggacaggtgc gctcagggca
540961 aagctctctg cctgaagaca gatttgtagt tctgccactg ctaatggtga actttatata
541021 cttcactact tgcctgacag tgtagttatt taaggtgtta aactgacagt cttgaacaaa
541081 atctaacaat tgctacatta agtgtgtgag ctgtaatgat taaatctgta gttatggctt
541141 tttcccaagt tgagacccat tgtcacaaat gtgcagccta aaaatcagtt taaaactgtc

541201 aattatgtta gttacttttc agaatcacag atgatactcc tcagctcatc tcatgatctt
541261 tcacattatc ttatgactcc aaattgtggt ctaccacaat aaacaatatg aaacaaggtt
541321 tactacaaca gcaaaatgtt gcattaatgc accacttaat gtgatattag caaatctgtt
541381 gcaatttaat ctaaatagaa atgatctagt ttgagattgc atccaaaata tgcctgtttt
541441 tatatttcat tttatagctt gcatattgtt tgaaattaag gtacaggagg catcaaagat
541501 atcagacata catttcatta tgtgttcatt ttcaaccatg tgctgtactg tatattatat
541561 gcttttaaat atatcattga ttctctcttc atgatcattc agctacttaa aagcttagat
541621 gtcaaggaaa catctaaaaa caaggaaatt agaaataatt agcatcacta gctttcagat
541681 taaaactgtc caacttgtca gctcatctgt aattgttgta aaaatggaca tgttgacagg
541741 atgtggaggc ggtaccaatg cctgacggac agctctgctt gttggctctg ccacctgaat
541801 tctgtgaggg agatggacca gaggccgttc agtacctcaa actgctctgc cgctacatca
541861 cagaccgtaa ggtgggtgcg agaggccttc actgtcctcc aatcctgtgt tattcgcttt
541921 cactggactg acttcagtgg ttttctgcat cagggtgtgg tttcaggtat cctgctggtg
541981 acctctaata agatcttctt tgacccgtgt aagactcacc ccctggtgaa agaacacggc
542041 tgtgaggaat atctcctgtc gtgctctgtt gacagtctga cttctgtttc cttcttctct
542101 gacatctccc atattcactt caacacatcc cagcagaggt cagcaatgct tcactcctct
542161 caaacagcca cgcgggctga ttcacactct agatttgttt tgtgtctcag gaaaaaagga
542221 aagaaaatcc tccagcagtt gaagaggcaa gcctacggcc ttctgaagca aaatggggag
542281 tcgaccccag ctttggagtc tgtccattca gagttatctg gtctgcctct aaggcagacc
542341 aaagaagttt ctggggagga agaggaggct gagagcatgg acatggatga agctgagggg
542401 gagcttggtg ggatccatgc cgagggctct ctgggtattc tgagcagcac tgccaccttc
542461 tgctctggag gtcaaggaac cgtcagcacg cagcacacgg tcgaaacaaa accatcagag
542521 actgcaggta agaatgtgaa ccagtcggcg attatagttt atcacctttt cttcatttct
542581 ccatgacaac tgttggttgt ctgctcactc acagcacccc ggaggtcgtc taccggcagc
542641 cccggcggtc tgatgtttgt gcggctgcgg gttcagctgc ctgcgggaaa gaaaagaggt
542701 gcaggagccc ttcagatagg ctctgccaag acatcacgca aacaggacgc ctggcttgct
542761 ctctcacagg agaggtgaga taactaaggg aaagtgaacc gtgcttacac gtctgggccg

542821 gtgtttaata ttgcaacagc tccccgttcc ttaactgctc ctgtaaatac
tcctgccttc
542881 ttcagttgaa tattcacttt cactcagctc tcagtgaaca ttgtcaaaac
attacaaagg
542941 taacatgtaa aagtaaatca gtcactcatg tactgcacct atactttcac
agcattcaag
543001 agggaaaaat tattttactt taatagattc ggttataaat cgctccttta
caagaacagt
543061 tcactctttg cctctgcagc tcagacgagc tctacgccta ccttacacgc
tgtcgaccag
543121 acttgtgcat actcgaggga ggggaggagg agggggaagc tgaccgtgat
gaagaggagt
543181 ttgtcctcat tgaggacaag gatgaggtgg agcaagagga ggaggaggag
gaagaggagg
543241 tatgccacag gcttcggagc tcaggagagg actgggaggt aagcgggttc
ggtcttctcc
543301 cactgggcag taaacaagaa caaacgttcc atagatgcac cttctctgtt
tacttgaaaa
543361 gatttgataa tatttaataa ttccttgcgc agatggtgtt gatggaagac
agcagggaca
543421 aactaagcct ggtccaagac agggaccctg aaggtctcag taatatcgta
gagagcagtg
543481 acattttaga agcttcgcat atcagagagg tgagtgatgc acagaaacca
actcttttgt
543541 tgagtctgcg atgatggaaa acatgctgga agaatttctt ttgagactcg
agatgtcctt
543601 cagtttcttt aaacagtcca gtggactctc tggttgactt caatggaaaa
aaaaacttga
543661 tctggatgaa tgacaactta cacgaatatg ttaacaagtc ctattttgat
gttggttcag
543721 tcagctcatc cgaagttagg gacatgtaaa tatttaacaa atgttgctgt
atgaactagc
543781 atcaatttgt tttcaaatct ttcaaacagg gctagtggta gcttagtgag
gtagtggtag
543841 ctcggtgggg tggtgataga tctatcacta ggggacccgg ctgggaagca
gagggctctt
543901 gatcaaagcc ctgggtgcag tcaaaatttg gaaggtgctc tggtagtggg
gaaaggtaca
543961 aggacaccaa ggtttttatt ttttctcagt tttgttcatt tttctcagtt
atcaaaggag
544021 cttcccccca ggacaatcgg ccacacctgg aacctggcct acagtacatc
ccgccatggt
544081 gccagcctga agaccctgta caggaagctc agtgcctcag actctcctgt
gctaattgtc
544141 attaaggatg ctcttgatga ggtagattga atgttatgaa agtaatacta
ttgcttaaaa
544201 atgaggccga taacatatat aaagatgcct ctggacagta ttttacattt
aataaccatt
544261 ttttactccc attattatta ttaacagtgt tttggtgcca tttctctttt
atgtaagata
544321 ttcggggcct tcctttccca ccccctaagg cccagtgaga cgttctatgg
cacaggagaa
544381 accttcctct tcatgctgca tcctcgctac aaggtgcgcc acccgaccca
cgtcctccat

544441 caagtgctac tgtagttaaa taggagttga aatttaaagg tgtctttttg ttttggcaac
544501 agtgcttcag gtggactgga gagaattcat tttttgttaa aggagacctg gactcttttg
544561 ctattggtgg aggcaggtaa tggccccttt tccagcattc tctctttaac tttaagctca
544621 gaatttgaca atttttctga tcattataca aaggttgtta ttggggtttt acattatagt
544681 ccaaaatgtg gaggtgtgga ccaataagac tgtactttct ttcatcagtg gtcactttgg
544741 tctgtgggtg gatgagaatc tgtatctcgg ccgcagcagc ccctgcttca ccttcaacaa
544801 ctgttgcctc tcagaaaccg atgactttcg aatcatggat ctggaggcct ggacgttcag
544861 ctgaagacac actcgtggtc gcctccacct gcaccctcac caccgatcaa ctctgtccct
544921 gatcattaac acatacatct gttaattcgc cgcttcatcc cctcaagtgc tgggtgtatc
544981 ggtgttcagt ggattttact ggctgaaatg aagcgaacaa agctgatctc agcagcctcc
545041 cggcgtggag gaacttagac agtggaaggt ttcacggtgt gctcattcgt gtgggtttgg
545101 ctgccagact gctaaagctg aactttcacg aacctggacc gagcccttgt ctttaaatgg
545161 aaaaggttac acgtcctaga cactgcactt tgctcttaat gtgaataagt aactttatat
545221 gtgtgcctgt gatctgaagt aatgcatgac cgctcatttg agtcgatttc tctatttaaa
545281 tctgcacatg cacgttctgc acatgcactg tccaattaaa acctgaaata caatgcaagt
545341 gactgattgg ttgacccctg aaaagaccac aagtcaataa aaggctgcat ttcaaatttc
545401 gtttggagtg tctgtgtttg gtcaatgaat aacatatgag cgaggattct tttgtaaaag
545461 aacacatttt tacctttact catttagtaa attacaattt acatgtttgc taaatctgaa
545521 atgaggcaag cggttcacca gagattgtga cttttttttt aagtcacaag tgtaaaagaa
545581 caacactgtc atctggaaaa gatttcttta ttttacgtag tagtaagtct ttggcttaaa
545641 aaaaaatcag tcaaatcctt aatactcatt caaacagcaa acaaagcttt agttttaaag
545701 taccacagtt gtattgagtc tgccctgctt tagtgccccg aggtggagga gggtttctcc
545761 tgcagcaggc ttcagcagaa acatcagacg ctgcccagca cagagagctt taagacaaca
545821 tcttcacaaa ctgctcaagc atgactgaaa aggcatcacc cgcccacatt ccctctctgg
545881 gtacgctcag cagtgtttaa actcgctcct cgtttgaata ttgcacccca gaatagagga
545941 gaggattatg tgtttctgag tgttatgatg gatttgcatc catttgtctg cagctacaga
546001 ctggttgatt tatgctgctg ctcactgtaa aaaccacagc atcttaaaat ataccacact

546061 gtcagtaaca atagctttag gaggccacag ttttcctgta aaaaaaaccc caaacactaa
546121 ccaaaagaga taccacaata aatagtaaca aagatgagag aaggccaaaa tgttttttttc
546181 aggagctgaa aaagcactgg ggttttttcc ttattaaggg tccccgagga attactaatc
546241 atttcctctg catgaacatt actgtggcat gtacgcccgg gctctctcta gtccctcttt
546301 tagaaagctg atgtaagtgg aagtggaagg gtcctcgaac ccgcccgaga agctgaaggg
546361 cgtgccatcc tcctctggtt tcatggctgt ctgatccagg aagaagttgg cattgatgtg
546421 gtggacctag aaccaatcac agattgtaaa tgtacatgca taatttgatt tatatgccct
546481 gaagctgtat gaagtgttca aagtagaact gagcagaatc aaagaatttg ctctaaaaaa
546541 aaagatcgga actagtacta ctaatctaat attagcctag cacaagcacc aacaaacagg
546601 ctttcaaaat ctctgaccat ctataaatgt tataacaact ttttacttgc acaagaggta
546661 aaatatttac tatagttcca ttaggcagcg ccaccatcat ctccacaggg aagctgtact
546721 tctccaggtg taatcgggcc ttttcactca acacatggtt ctgctcgtca gcctgagagg
546781 aaaaaaaact ctgaatacac tgacgacaaa taggttgtga aaaactctac atctaaacaa
546841 agtacagaga actgggtcac gggattttta cctgcatgct ctccagctct ttgaccagag
546901 accagctgct tacgaagctc tcgttgagca gggccaggac gggcgaactt tccaggactg
546961 tctcccggag agttcgcccc gaacctacag agggcagata gatattagtg ctgtaactgg
547021 agtctctgta aaggttttgc ataagttttc tcacctcagc aggactggtc atctaatgct
547081 ccccagagca gaatggaatg caccagttta ttttctgctt cagctcgctc aaaggcctct
547141 gagaatggca ggtaggacac ctggaaaaga gcggcggaaa aagtgatctc ctgggttaaa
547201 ctctgctgtg tgctgaataa acaatgggtg tttaccttct tgaatgggta gaaggtaaca
547261 tccagacgtt gagcagcttc ctgatgactg atctccgatg tccagtggat gtcctcgaag
547321 acaaactgga tcggttcgcc gctgtccttg ctgtcgatga cattaccctc ctcatctgtg
547381 ataacagtgg gtgtggacgg gcccaatgac agcagctcca gctgtggaca catacgcttc
547441 ctacgtcagg aaactccctt ccctgaggtg aaacatcatt gctgtaacag agacacccgt
547501 ccctgcagct gtacctgtgg cagatatcca atatcaacct ccatgttgct gctctcactg
547561 gcaccgtaca accactccat gtccacattt agcgacctgg gaacggaaca gatcacaatg
547621 aagtcactga caaagacact tacagcagca atctcatgcc acagactcac ctgttgttgg

547681 ggacatagag gcggaattgt cggacgtggg acgcgtcttt ggacagaaca
atgttgccag
547741 tgaattgccc aggggtgaac cagaaaggga agtttggaac ttcgttgagc
tggaattccg
547801 catgaattct gcaggaggaa aattgcatca atgagtaaat gaacttgccc
cctgatttta
547861 atgtgtcatc agcttattta catgcacttt tcaacacttc cgccaactta
taatggctca
547921 tgctcttcct ggtcgccact gaatttcctt cagcttgaaa atatgcacac
gtttagcgtc
547981 agtgggacat aaatatattc attgtgttga tgctgcctgc cagggggctc
ctcacctgaa
548041 cacgatgtca taataaaaat cattgctggc acggatgcag gcgacggtgc
cctgaggggc
548101 aaagcgagac ttgatgaagg gccgagggtg gaacatgctc aacaaggagt
gaatgagaac
548161 ctgtaatggg gcagcaacac ccgttaataa acacactcca gtcagggacc
catgagtgct
548221 ctttacaaat acctctttgc ctttcggcga gggaggatgg taacgattgt
tgggcaggta
548281 gccggtgaag atgttgagct cgctgggaat aagccaccac gtgtttccta
cctccagctt
548341 attctttgga ggtaagaaaa ccctgaattg tctggcggag aaggaagcag
aaggcactgc
548401 tgggctcttc caggagcgca ggccgctcag agagctgtgg gacacctgca
gaggaaggat
548461 aaactccagt taccacccaa aaaccaagcc aatgtgccac tacttttaaa
aagtttggct
548521 ctccaatgtg ttaatcagca ccaaaagaaa ataagaaatc atcatacccc
aagaaatcca
548581 tccctgcttt ttgtcatgga gtctaggatg agaggctgca tcatggcttc
cactgtaaat
548641 gtctctccat tgagttcatc tgtctcttcc tcctcaaact ctacgggggg
taggattcct
548701 tcagaaggcc aaaaggaaaa atcctctcag gcttgtaggt gagattcaaa
agggcaataa
548761 caggagaagc ggcatacctg tcagtttctc agctataggt ttaaactcct
ctgcgctgag
548821 ataaaggtca tggtcaatgt ctaaggaaga gaaaagaaaa tgtccctctg
cgcccagcgt
548881 tcgaatgctc tgttcctatt ggaagaacat acacgtttgt cctcatttac
acaagcatca
548941 tgggttttca atacagcctg aaatctggga cagtgacctg gatctcttga
aataatgtac
549001 aaatgtgctt ttctgtcctg tgtccatgtg agtccgagca ctcatttagc
attagctggc
549061 tggatagaga tcagcccaga ggccacagat tccttgcaca tgttcaataa
gacctttcca
549121 gacagatgcg tgtctgcaag accttacaca atggagggaa gggtgggtgg
gagtttccaa
549181 acttcagacc ctctctagtc aaataaaagc ttggattaaa gcaagttgag
tttaaccagc
549241 tgtggcacag tcccgcccca ctcataaaaa gaaacagggg tgcaaaaact
gcagatgtgg

549301 gaaaactccc tccaccatta atcagactgc cgaacttcat taacatacat
atgcaatgca
549361 ccttgtttta cagtataaaa cacatttgtt ttgcaagaat tttacaagac
ttttactcga
549421 tatgctcaac aaatacaaca ttaaacaact gcgtttcaga tgtgtggctt
acgtgacgtt
549481 tcaagaggtg ggcatcttga taatacttga ccccgaaggc aaaaagtgga
acggcagtca
549541 caagaatgag agtccacagc cccttgcaga tccacgatct agagcccggg
ttcgcattcc
549601 cagcatgcgg cggggttttg ctcccatttt cgggggtgtt tttatccacg
tctgcggcca
549661 tcctggatct cagcggcggc tactgtgatg acggcattgt catgaaatga
cggtcagaaa
549721 ggtgtccttc ggattgtgaa ttttccagca aatgatttat ccgagtgtgt
tgtgtcttcc
549781 ccagctgttg catgcgtggt gacgctatgg aaccttagct ttagctgcac
agatttcacc
549841 ccctaccaat gtcgaaccat tggctgcggt tcagatacgt caaagaacaa
acgacaccaa
549901 gagctaaacg gatatggttg caatttcaaa gtaaaagtaa attggtattt
atttatttat
549961 tggtacttat ttaatgcaac aataatatac atatataaac actatactgt
atacactata
550021 gcaaatttgg cttaaacgtg tcaagagttg cggttcgagg acactaattc
gtttattttg
550081 aaacaataac cggaagtagg ggtgttggta gctcagtctc gtgggctgag
aagcaaaggg
550141 atgttagtgc gagccccagt acagacaaaa catggaaggt gttctggtat
taggggaagg
550201 tgccagaatg acttcagagc actgctgagg tacctttgag caaggtacca
aacccacaaa
550261 tgctaataaa gggcccacaa aagctaatat agggccttgc aatgaactca
tccagggatg
550321 gacccagcct tcgcccattg tttaccttcc ccatgacccc aaaagggata
aagcagttaa
550381 gatgaggcaa gatgagaaac aaccagaagt ttccttattt ttgattcagc
aatgattact
550441 tttgccaagg tgataacagg catttgtttt cctgttaaaa taattctaaa
agttttgaac
550501 aaactttagt gaaatgtctt tggagatgat aatggactca aataaatttt
gatgatgttc
550561 gtacgggtgt aagtcacaat aggttggtga gtcagacgcc ggtgagctgc
ttgttatgta
550621 atgggatggt gttagcattg ttcttccagg attttttcc ccccagtgaa
acatcaatta
550681 catatttaaa ctacaaattt acatatttaa ttacagattt aaacaataat
ttcaccaatt
550741 tcagagtgaa atgtggcaca tatatctgat actgtccaaa aaaaggaatt
ctttgtatgt
550801 caatgttaaa atttattggc attccacatt ttgatatacc atacatgcaa
tatttacatg
550861 gaccagttgc actatacatt accaagttca atggtaatgc ttccaagtta
ggtttacagc

550921 taggttgaga ttttgtttat cagtccagca cagatataag cagctactcc
atgtcctctt
550981 ccacagactg tgtcttcact tccactgtct ttggagggat gtatgacccc
atacctgcag
551041 ctctctctgc ttcctcctga gtatagggct cctccctgag ctgtttcaac
ctacagagaa
551101 aaatatttaa taataacgtc atagtcttag ttatcggtgg catacatata
ttgtattacc
551161 ttttcttgtg cactttactt ttgaagtgat cttttaacga tttcatgtcc
acaaaatatc
551221 gcctgcaaga tatgagttgt tcacaggtta agtggacatt caaaagcagg
atttattttg
551281 caagacatta atgaaggagc ccaaaacctt ctatttaaaa ataaaaccta
cgcgcagtgt
551341 aaacagtaat gctgtgcgca gcctgtgacg tcatagtcca cttcttgatg
tagcagcttt
551401 gctgcaatgt ctggcttcat atcggagtgg atctgatcca ggtctttggt
tctgcgcttc
551461 gttttccaag ttttagcaat gttcttcttt ctgccattgt tgtggttacc
cgtttgttta
551521 gatttcccca tggtagattt gacgaaacag tctgtcatac cacaaggaaa
acacacaatt
551581 gagacacaag attaatatat tacaaagcta tataaaaaag caaagctgag
tacaatgaca
551641 cgatgtatcc atgtgtaaac tatacccaca agtcaatctt aacaagcctg
ttgagaacga
551701 aaagcccaaa tttacaaagt tatgtatcgg agaatgataa tttcgcaaca
aatcattatc
551761 agagtagtaa aatatccagc tatattaaaa tgtcagaaca aacgtcataa
atacacgtta
551821 tatacttata gaagtcgccc gtacaacaat ttctgtttac ctactgtcac
acgtgtggct
551881 aagaggaaac aattgtactt ccgggttaaa gaccgtggcg ttataccaga
gttacacctc
551941 taggtggcgc caatcagtta gatagataga tagatagata gatagataga
tagatagata
552001 gatagataga tagatagata gatagataga tagatagata gatagataaa
atagtttgga
552061 gaaaaaaaat ctgataataa gacagaatta tgaattacag acaaattata
ttattttaac
552121 ttatttataa tgttgctaag ccaaaccaaa gttaaatata tataaaaata
tgagtttaaa
552181 atggtcatcg acgtagttta aaaatttctt agaataaatg cgaaaataag
aatagtgacc
552241 tgctaatgct ctttcgttat ctttgtgttg catttatgtg cccatcagaa
ctgttgcagg
552301 caaacggcgt tgagattcga ttagaataaa agtactgtca tttaattaaa
taatttatgc
552361 accaacaaaa agattacatt ttaagggata acatgtgaga gaaaaagctt
tataaaatct
552421 aaccttccaa actgacaact caaagagcca actgaattga attaaaatga
tttaaaatct
552481 gcagcaataa ccaatacctt catcgctacc tcagctttca agagcagtta
taaacacact

552541 gaaaaataga ttttaacaaa ctggggcctc ttgtgtattg tagtgagaag tttgtcagtc
552601 gtctttatat attccgacag tcagtgaagg cgttatcaga ctctactcta ctgcatacgc
552661 gctttggtgg agagcgaaga tggcggagaa taatgtcgaa ccagagaacg aatggagcag
552721 ccgggccctg gacacgactg aggacactct ggttgcaatg gaaacactgc ttgcaacgct
552781 gagagccttt gaagacgtgc tcaggcaaca agatatatcc gttgcatcct cggcagagta
552841 ctgcgacaac ttctgtcagg taatgtgtac atgctacctt caagctaccg cacggctaat
552901 atcgctagct cagccacgat ttgttttcat tttcatttgc cgccctcact cttatctgga
552961 tcggctgcgg gttcgctagc tgcccgaaaa cgtgcagggt ttgtggacaa gcggggagca
553021 atatagcgcc gtgtgtgtta ttgttcatac gcgcgtctgc atatcatttc acagagcaat
553081 ttacctgtca aaaacaattc ccgttgtagg aaaaccatgc acgctgcttc gggttacttt
553141 taatgacata atgacctgcc ttcccattat cacccattct ctacgaagac gaagttttaa
553201 atatctttgc acgcacatgc tatggtccgt attttcgtgc gtttgtgtgg ggcggggcgc
553261 atctgtctta aatttgccaa aatgtgcgct tcatacttgt gccttcgttc acgtttgaat
553321 ctgatgcagc gtccaaaatc agtggagcaa cgtcgcattt attcgtatgc tcagatagac
553381 tgtaagtgta attgtcaccc agagaattaa agttgtgcgg gaaatgaaag catgaacgct
553441 ctattccacc atttaagttg tctgatgttg aaattggtta gatactcaaa tgtgcttcag
553501 acaatgtatc gtgcctcagt tacactgagt tacacattag ccagcagccc tttgaccttt
553561 aatccctgca acctgcactg tgtcactatt aagcagcaat atccaactgg ttttatggtt
553621 atgtttgatc agtttggaag ttgtctggat gtaattagta cactctcaaa ggaccttagt
553681 ttttgctcgt tttgagggtg tactgtggat taagacgtca gggatttgct ttggtcccat
553741 ctagcatggt tcctaccgtt tcataaatag tactccaggg aggttgtaac aaacccgatt
553801 taatgcatct cactccatcc aaggtgttga actgactaga cccccatgtt ggctcctctg
553861 caccagggga gagatcccag cactatttaa acatcagtca ttaaagcatt ttttttcatt
553921 gcaaattttg aatcagtagg tctcagaatt gatttccaaa tctctattgg cattttaatt
553981 ttacttatat atggacagat attataacac acactgtgat agaggagcgt gtttgtgcgt
554041 gaaccccagt ttattttgtt tacagcacac tattaaaagg cttcaggacc atctttttct
554101 gtaaaattgt gtcccattag acccaatcag tagcatttca catctgagag attttcagct

554161 gggatcagtt aggtgaaggt gggtctctcc ccagatggat aacccctctt caccctgaca
554221 gatatctcat ttatttccct catgttattc ctcactcttt acattttcct gtttgtttgt
554281 gtttataggc actgatgcac tatgctggga gcaggaactc tatcgagcat ggtcttcctc
554341 ttcttgaggc ctactgtttg tccataaact gttttgcggc agcccggtcc ctcctcactg
554401 cggagtccga cagagtagct cttgttctga agaggctagc tttgtgagta ctgggcagtt
554461 tttgcatacg acctctttag tatttaaatg tttgagagca aatgttccac ctgcaagttg
554521 ctggtgcttt gcatgaggta tatattcagc atttctttac gtgagttgta ttcatgtcat
554581 gtgcttccag ggtaaaaaga atctaattgt aagaagttgg aaggaagtag gatgtagaaa
554641 ggcagaacat ggagtttgat ttatccattc aacccacaat ggatatttta acaaagaaaa
554701 cggcaattag atgataagca ggttcaagga cagatgtgct ctggttccta attactacat
554761 gacacacaaa gcagagagaa gggctgccgt caacttcagg tgttgaattc acatgtggca
554821 gctgtttgat tagggcatac ttgggacgca ctggggggat ttcgtctctc atctggcttg
554881 gaagcgctct agtgagctcc cagaggggtt gtaaaaagtg gctggggaga ggaaggacgg
554941 tttgggcctc cctgtttaaa tagctgccct tgtaacccag ataaaaagat gagcacaaaa
555001 tgagatgatg gttttagagt atcaggaaac ccagatgttg ctaaacttca ttagatttca
555061 tagaatatag gtgctccaga atgacagata tttagttttt cattttctta tttatttatt
555121 tatgttcatt ttggccctga atggagggaa aatgctaact tttttgtcac tgatgacctc
555181 tttcttcaat gtctgtcagc ttgtcagagg tgattgtaaa tatcaacttt gtgtcttgca
555241 ggagctgttt cgaactgtta ctgtcagtgc ctgagaatga aatcccattt gaggcctggg
555301 ttcagtttca tcattcagtt caggtgcagt attgtgattt actaaaatag ctccacggga
555361 ggatcccagg caatatcagt ggatttgttt gcctgcatat tgtgtatcat ccatatgtaa
555421 agcatttatt actagtcctc aagttatact gcccacttat atagtgtcac tgttgactga
555481 tgtgtgcatg tgagggatag attcactgca aacaattata attgacattt atcatgaaat
555541 agcccagctt tgcttcagag aatgaaatga tttgcatgtt agaatcaatg caaaaaatga
555601 acagtctctt ttcttttgca gtttgcccat gaaacattat tacagtatgg cagcacagac
555661 ctccaggcat tacttcagat cacaggagag ggtggagcgt ggagcaactc tgtccttact
555721 tccctcctca ctggccaacc caccagtgca gaagaaggtc agtttagcgg agccatttag

555781 gtcctcaggt ggcgacttct cactaccctc cataaccaga ctttctttgt tgccagtgga
555841 agcattaatc agcttggagg gcgagggctt catggaaatg cgggtcaaac acctgcagaa
555901 gatgggcgag atagccaagg cggtggtgct ggccaaagcg tgcactgaat gcagcttcat
555961 ctccaaccag gctacatttc atcacactta cgtctcattg ctttgtcatc tgcttccgaa
556021 tgaagaagcc atcatggagg tatttatttt ggaaagagtt ttatcaggac atgctgagcg
556081 gtatcagctg gctatgattt ccgtgctaga agtttttgct tcctcatgat aggaaatgtt
556141 tattagttct aaaaaataaa aagtctgtgc acatttccaa attagatata gaacatgcta
556201 aaaatagatg tcatcacttg atcaaagata taaatagatg ctgtctcacc agaaaagcat
556261 tcaaaacaca gtttgtgatt ccgtttctca aaatattgag aattaatcag tgaaatgtac
556321 caggaatttt atttctcttt tcattccagt gacataacaa tggtctggtc tttcaggtct
556381 tttagcttct gttttaattt ctcctcattt acagagaaaa aagattggtg atagacacct
556441 ttcttctgaa agcctaatct tcagactcta agcttctcat ttgactttga cttttaagag
556501 ccaagtcaaa gatcttttct gtacagtatt tataaggttc actgtgcatg ctgctttctt
556561 ttaatagtaa agtggcccaa aatcagagtt gttttgtggt tatctgctct cccgatggct
556621 tcgtatctct atttcacgct ccttctttgc cctgctttgt tgggaggtta caatggtaat
556681 cagaggtttg ggttttgaaa ctgagcagct gctgttgcat gcttgccttt tctctctctt
556741 ttgttgaatt tattatcctg ctgcattatc tctgcagaca aaagaaattc tgctgctatt
556801 tgcacttttt tttagctttt gttcttttag tcatttgtgc aaattttaat gttcagttac
556861 tgaaacgttg cagcagccaa aattagtcac agcataaatt ctggtggtga agaattccca
556921 tgaaaggtgt tataaagggt aacaaagaca caaacaagtg tatcagcgtc ttcaaatttt
556981 gtgacctaat aaaaaatagt tccaaacgga tttagaagat cctaaggttt atatgaagcc
557041 ctaaacaata attcaaatgt ttgcataaat gatgttacct gttaagaagt tattttcttc
557101 aattcttagc tctttcaatt ttctcttttt ttagatatcc aggctggact gtaaggacgt
557161 cctggagatc acgtgtaatt tggagacgga gggagaagaa aacacagcat acatcttgtg
557221 cacaactttt ctgacccagc agcttcagca gcagagcctt tactgctcat ggtgagcact
557281 ttaactgtat aaacaaataa gtgaactgtc taaattgtga gttgaatata aacaaaaaaa
557341 atcacaagaa tgattttggt tttgttcttt gctttgaatt caaagggagt tgactctctt

557401 atggagtaaa cttcagagga ggatcgaccc ttcgctgatg tctcttctgg atagatgcct
557461 tcagctcggt gctattgcca agaccgttga ccatttactt tctctggttc gtgttattca
557521 gacagaggtg agtccagcac cagtctcttt cggcattcgt atcaccaccg ctgaaacggc
557581 agcctggaat gaatgatggt tgtctaacaa gtaaggagaa acggttgtgg cctctgatct
557641 ttaaaaagcc agccataggt tccgcagtgt agggaatcat gaatcaagaa tgaagtgttt
557701 gaagtacaca gacgggtaga aatactacag tttccagcac atcataaatg gttaagatgt
557761 aataaaagtt atgtgtgaat cacatgcaaa atatataaat tatggttttt atgggagttt
557821 gagtgtttat tattctcctt gtttcaggca gaggagattg gggtgcctgc ctcagtagag
557881 ctttgtgtca aagcccttca acttccaaag caggatgaca cagaaactag gatctcaatc
557941 tgcaagacag tgtctcgtct tcttaccgat gacctggagg tgttacgtgc ctgcctgctc
558001 acagagttcc tgctggggcc caaccaagag gtcttcagcc gcctcgagga actccacttg
558061 cgtcctgatc aaaagtatga ccaggaaaat gaagtgattc ccaactcgct gcgctgcgag
558121 ttactgcttg tactgaaagc ctactggccc ttcgatcctg aattctggga ctggaaaact
558181 cttaagtacc actgcagctc ctttctagga ttaatgccag agtcagaaag tgaagaggag
558241 gggacagttg acaagcaaga gaaggactca caacctgagc tacagggatt cccagttaaa
558301 cttgagtctg atcaaaagag gttaaatgga acccttgata gtcacaaaca gcaagatgaa
558361 aatgtctgta gttctaattt atcagaaagt ccagcaggaa cctgcaagaa gaagcataaa
558421 ttctgctgtc ggatatgtaa aagggtgatc gctgacaccc aaatcatcca ccactctaaa
558481 aggcatgcac aggatggctg ccacacctgc cccgtgtgtc tggaaaagtt taagagcagg
558541 aaggatctcg ttcctcacgt gaaaagacac attgaggaag aagaacctct ttccaacgca
558601 gttaaacaag agagttcaca gaaacagctg gacgaggatg acgatatcga gcccggggag
558661 atcaccatcg acccgtcctt aatgtcttat tataaatcca cacacgatcc agatgtgctg
558721 cagcacatcg tgcaacaggc caaaacggtg aaagacaagc ccgtggacga agacgagcac
558781 gtaacattcg aatacatcgc taagcacttt aacctgcaga accgagacga gtatcagtgc
558841 ccgggaaccg gctgcagccg tatttttaag cattccaagt acttgtacgt ccacttgaag
558901 tctgaacaca agggcgacga aaacgtgaag cattttcatc agatgagaga caagcgagag
558961 aagtgcattt tctgccgacg gcatttcgcc acggcgtacc attaccgcaa acatcgaaga

559021 ctccactatg gcgagcggcc ttatgcttgc gtggtcatgg gctgcggcgc
tcagtttcgc
559081 tcatccaatg agcttgtcac acacaaacaa acccacggct atcagctcaa
ctacgagtgc
559141 gaactgagcg gctgcaatgt cacctactcc gatttaggcc aaatttatca
ccacgaagca
559201 cagcatttca gagacgcagc atttacctgc gttagcgatc agtgtagaaa
atactatctg
559261 tcaaaaaagg aattcataaa gcatttatcc acacacaaca tcaccttctc
agaagaggac
559321 ttcgaggcac agaagaaggc aaagcagaaa ctcctcaaca ctgttcgaga
agcgccaccg
559381 cattttagcc gggctgacag cacagaggag atcgctcatg aagatatgtt
gaactcttct
559441 ttagggagct gcgattctga ggagccgaaa gcaacaatga cgctggtggc
cgtctgtttc
559501 gatggcagca aattcacctg tggctttgag aaatgtggca tgaccttttc
cagagccaga
559561 gatgtccaga gacatctgaa atgtgcccac ccagaacacc taaagctgga
gaataaagaa
559621 cacaagcacg acaaagagcg agacccaaaa tccaaggggg cactaaagga
caacgagcca
559681 gatgttgaac ataagggaca aaatgagccc tcggctcact ttcaacctgt
cgagccttgc
559741 gatgaacgta caaccctgcc ccatggcagt gaaacaaatg cattgagcct
ccagacaaaa
559801 agcgatgctc tgaaagaaat ccttcttatg ctcagtaaac tagatctgaa
ttcttccact
559861 ggtattgtgt caagcgagct ctctcagtcc agcctggagc ctaatgcatc
gcagttatcc
559921 cttcatcaga cagcccccgc caagtctcct cctgtaacgc ctcctgaaat
gtccccaccg
559981 catcctgaag aagacatcga gactgaagaa accttagcag cagatgatga
agccagtttg
560041 caaacattag ccagtgccaa gccatacatc tgtgagataa gtggatgtga
cttcaggact
560101 gctcagaatt acagtttaat gagacactac agcactaaac acggtcgcac
tgttgaacaa
560161 gcaaaaagac tgacttcttt aaaagctact tcttttaaac cttatgcgtg
ccatctttgc
560221 tccaagagcc acagacagaa acatgctctg agggtccact acattcatac
gcataaaatg
560281 agtcaaactt tggtggacaa aataagctgc gcgtctgttg ttgagcctaa
atctcagcct
560341 ttgacaaacc atcatgtcaa aataaagaat gcagtccacg agttgccgca
ccatcaagag
560401 aaaaaaaact ccatggatac tgaccttcat gacttcaatg acaatcattt
tctgtctgaa
560461 gaggaagcag aggaacaccc agagaaccat gacgacggtg aaaaaaagga
agaaaacagc
560521 gacggggccc cccaacaggt cagaacccga cgtgtggtaa ccagaaataa
cctctgctac
560581 atattggaca agttcagtaa accctttcat tgtgtcgcaa aaaactgtga
cgctgcattt

560641 tccacccagg gaggccttgt gcgtcacctc caattggtgc accattacaa tcgctctcag
560701 cttttattgg acaaagatga ggatgaacat cacagcccag aaatcaggaa agagcccgcc
560761 aagaaaaggc cactttcgaa ctcggatgaa cctcagcccc aatttaaatg tcactttgcc
560821 aactgtaaag cgtcctacca ccttagaagc agcctagtac gacacacccg ggactgccac
560881 tcccaaccac cagaggtaat ccagtgcaca tacgaaggct gcacaaaagt gttcaaccac
560941 aacgaggccc taaaaaaaca catgctttac agccactgcg aatactatga ttcactggtt
561001 gtacgacttc agagctctca caataaaaca gttgctggat gtcaaaacaa cgtcattgtt
561061 gcaccacaga gtccccaaaa cgaagagcct aattcaaccg ctactaaggc tgagactttg
561121 aatccagaac ctgaagtgcc tccccagatt gaggaaacta ataaagacga tgtggttgaa
561181 agaaaagtgc catcagaaaa agtgtctaaa agaaagggct acagtcagtt taccttcagg
561241 tcttatgaag aagctctgca gatgtgccaa gaccgctgtt tgcgagtggc ctatccgtgc
561301 atggttcaag actgcgagtc cgtcgtcacc tacatgggaa gtctgcatcg tcactacttg
561361 aaagttcact tcatgcgacg ggaagatctc atgaagaatg aagataaact tgtttttaat
561421 gccgaacaac tggaggagct gatccagagg aattctgcca gattaacggc aacaggaaca
561481 agcgtctcta atgaaggttt taagatggaa tgcgaggctg agccagagag cacagacgca
561541 gggcagcctg cggccatggg tgcgcacatt gttaagtcag acacaaatga gaaggaacca
561601 gatccattga cttttcctga ggaagaagca aaggagccac caccgattga gagaaacggc
561661 gtcctagttg ttgcggatga agtcctttat ggcgagccaa gcgccaatgg acactctgag
561721 gtgccagttg tagaaaagcc taaaagtcca aagccagagg gtttatcctg ggagaaaatc
561781 aaaccacttc ttcgtcctgt caccgttgat ctctcaccac catactcgct gcgctttact
561841 actgaagagg gcttccatga tgtgctgccc tccaaagatg ttgggaaaat gttgaatggc
561901 tctgtcttgc tgcctagtcc tcctgtacgc cagcctctaa aacggaaaaa cgaactctct
561961 gaacaaccac caaacctgaa agacactcag ccttgtagcc cttcttttga catcacagcc
562021 tataaaccag tcggctttga gagctcgttt ctgaagtata tacaagacac aaccactaaa
562081 gacaaaaacc agggtccagt gagacgggag tctttcagac gcagttgttc cgttaaagag
562141 aacaaccagc tgggagtctc tctgacccgc agcaaacgta attactcccc actgtcgaag
562201 cgccatgctg tgaccggaga cctcaactca gtccaaaaca taaaatccat cttggacaaa

562261 gccctggctg ggtgcggaga ccttgctatt aaacagcttc agtacctcag acctgtggtg
562321 gtcctggaaa ggcctgtgtg ctgcaacacc accactacca ccacccctga tctcttccca
562381 tcagaaacca acaattccaa attacttctg ggaagttagt tttaagaagc gttattttct
562441 ggcagttctc tttttcatca gtcagtcagt atttttcttt attaaaaatt tagtattgca
562501 ttgatctttt tcatttaaga cttgaagaaa aaaccctgag cggtgcatat ggaatacctt
562561 ttgttaaaaa caactaactg taaatactgt agttttccaa gaatgatata cacactttca
562621 aagactagaa atggtcagta gcatattgca aggccttttt ctgatgtgtt attgtgacta
562681 caaaaaagca tttacttcct agaatcactt gagtgttttg ttatctctca ccgatttata
562741 tcacaccagt gttgggatgc agtgaacagg aaatggaaat gttatatcca ttgaatttct
562801 cataatcttt tttactttct taattatgta aagtgatatt tgtttacagt atgctggaaa
562861 atgtatttta atttaatagc acataaggtg atttttttggg ggggagcgca tggaaatgtg
562921 atgagtcctt tttgttggaa ggagtaatga gaacgataat ttttgataat gatggatatt
562981 gtggatgtct tggttgtctg tgctgaagta tatgtggctt tgctggcatt ggaatcatgt
563041 ttacattacc aaagcaagtg taagaaaagt acctttttaa taaagaacta tcatttcata
563101 ggtatatgtg ttgcacattg aattaaaaaa tatgtaaata ccactttttc ttacatataa
563161 tacattttat tttgcataaa tatgctgcag tgatcaaaca ttttttaaca tagttatata
563221 taacagttta acttgtatca taagggctaa aattgaatac agtactgtct gaaagtattt
563281 ttcactattc attttcacag cacattgtaa atatttataa aggccaagag tgttttaacg
563341 gttcttttga ccgatcttta ttggtcgaaa ttctaaattt agattcagaa gtatcgtaaa
563401 agcgcagcgg aaccaaatgc tctcgcggct gtttctagag gggtggattt tgttgacaca
563461 ctacgcgttg cacgaatgga acgatattag ctttaaaacc ttggcttatg aatggaaact
563521 ggttgcaaag atttccgagt tctgatttgt agtcggtatc tgtcattata acacagggac
563581 agtttcctaa aatatttttt tacgtttgtg ctgagttaaa gctaagtagt tcctaactac
563641 tgatcggttt cgggctggtc aacatacagt atcattggcc ttttagctac tagcagctag
563701 tcacccccttc acctagaaaa ggccaaaaag aaaccaaaaa atgttggaat ctatagttga
563761 cctaccggtg gagaagcaga tattttatgc tgttttggga ttttcctgga cagtgtatct
563821 gtgggaggca tacctttctt atagacaggt gagtaaaaca gatgtcagct aacagacacc

563881 accaagaagt ttctaccgag gtggtgtcta attcaaacta aatgagacat
aagtgaatgt
563941 ttttgtttcc tctggaaaac cgaacagcac tagcaatttg atttcctgaa
aatgtgtcca
564001 aaagctagtc atttttataa tcaaatattt ggggaacaca actcccttat
ccagcgtgct
564061 aatgcactaa tgcgataaca gaaaaagaac gaaataattt tttataatgt
ttttgtagga
564121 tccaacagaa cgattgctga tgtctttatg ctccacagag aaagatatac
agaacaacaa
564181 cacatgtgcc tccagaactg gggaagataa ttgattctga aacatttcaa
aagtcacgtc
564241 tttatcagct tgacaagagc aactttggct tttggtcagg gctgtattcc
gagactgaag
564301 ggacggtaag ttaattaaat gtctatatat atattcaata ggcttgtaaa
ttgataatgc
564361 cataattcaa aacgttttag ttgatcctga tcctgggtgg aattccattt
ctctggaaca
564421 ttgctggttc tgtcacggcc cgctttggtt tgggttcgga gtatgagatc
acccagtcac
564481 tggtttttct gacgctcgcc actctattca gtgccgtgac aggactcccc
tggagtttgt
564541 acaatacatt tgtcatcgag gagaagcatg gctttaacca gcaggtacac
tggtgactgc
564601 aaccgaccaa caattgcact aattattaga acacactttt gtaagtttat
ttacatgttg
564661 tcttatattc tgctcagaca atagggttct tcctgaaaga tgcagtaaag
aaatttatgg
564721 tgacccagtg tatcctgcta cctgtcacct cgctgctcct gtacatcatc
aagattggtg
564781 gagactactt cttcatctac gcctggctgt ttactctggc tgttactctg
gttcgtgctt
564841 tgtgcacaaa caggaaaaac atgttgcaga aattgtttca atgtgctaac
agctcatgaa
564901 attgctgttc tctaatgtgc tcaggttctt gtgacaatct atgctgacta
catcgctccc
564961 ctgtttgaca agttcacgcc tttgccagaa ggagagctaa agactgacat
cgaggctctg
565021 gcgaagagta taagcttccc cctcactaag gtttatgttg ttgaaggtaa
cgcaatacat
565081 agttatgata ttcaactcta tatctaacca tcaatttaga ctgtaattgc
ttatacattt
565141 ttaattacac ttggagtatt tgtgcaggtc ttcacacagg tctttgtttt
ttatttccag
565201 gatccaagcg ttcatctcat agcaatgcct atttttacgg ctttttcaag
aacaaacgca
565261 ttgtgctgtt tgacactctt cttgaagact actcgcctct caacaagtct
ggagagcctc
565321 agactgagca accagagagt gacgagtcat cccctgaatc aaaagccaag
ccaaaggtaa
565381 tctactgaga gaagttcttg cttggtctta aatcaacaac atggctccaa
aatcttaaat
565441 tagtgttatt ttattgtggc tgaaattttg gctggtgcta aatagggctt
ccaacagtaa

565501 gacctgttta cagtaactct tttaacgatg tttttatgtg tagtatagta ttattgacct
565561 caatcttatt tgcagaacaa gaaacaagga tgcaacaacc cagagatcct ggcagtgctg
565621 ggtcatgagc ttggtcactg gaagcttggt catactgtca agaatattgt tatcagtcag
565681 gtgaaattct tttatatttt attacaatag caaatgtttt tttatgtaca tgtgcagaaa
565741 taatcttatt tctttctttc tgcagatgaa ttccttcctg tgcttctcat tgtttgccgt
565801 tttaatcgga cggaaggagc tgtttgtggc ttttgacttc aatgacagcc agcccacatt
565861 gataggtttg atgatcatct tccagttcat cttctcccca tacaatgagg taattttgag
565921 agcgagagat gttgatttag gtgtttttgt gtctgcaacc ttgatttttc tcatcattct
565981 tatttaattt ttgcagctct tgtctttctt tttgacggtc ctgagccgca ggtttgagtt
566041 ccaggcagat gcctttgccc ggagtatggg caaagcttct gagctctact cagccctcat
566101 caagctcaat aaggacaacc taggtttccc tgtggctgat tggttgtttt caatgtggca
566161 ctattcccac cctcccctgc tggagcgcct gagagcactg ggcaatgtca agcaggactg
566221 acggggctgg aagggggagc agcgacttct gtctcctcca aagggcctcc gcagacctct
566281 cctagccctg tgaagcatcc tacttttccc cccaccaaac ccctatattg ttttatccat
566341 tttttttagc tctttctctt gccatgcttt ctcttgtgtt cccatttaaa cctttgtttt
566401 taaatcaaaa tttaaacatt tccggatgag ccagccttaa aaccctgaag cgaagtctct
566461 gccctttctc actaaggtcc tcaaatgctg tctctgagat caaaccttgt gggcaatgtg
566521 tgctgagcaa aaactactgt cggagcacaa ttcacacttt tgtgcagtta tttatgcata
566581 atgagaaaat atctgattcc aaaacaaaga tgtaaagact gtctgacgtc taatggctga
566641 tggtatgagt gatttttttt ttcttctctc agttatacct ataatcttgg gttttgttga
566701 tatgatagaa tccatgaaca agttgaacat gcgctcctaa cactggttgt tgcaggtaca
566761 caggctaaag ggtttgattt gtctctcggg tgaaactttt ctttgtgaga aaacatttca
566821 gggaaagctc aagagaaaaa cacaattgat cgtccactca aattcttcat ttctcaaaaa
566881 gggactgtat gaatgtcacc aaaataaagc aaggacctcc ccttttagtt attctttttt
566941 aaaataaatg gacttaaaat gagcagcacg tagtgtttca gtttaggcta aatgtttgtt
567001 ttcttaagtc cattgaggga gattttattt tactttttgt aaaatgcttg cgttatgttt
567061 cctactatac ctgtacatat gtaatttggt ctgcacagta acagtgtacg tatgtgaatt

567121 ttgaaaagcc ggttaaatgt gttttgaaat tttaagttca caccgtgtat cttagtttct
567181 tctcttggtt cattttagag taaacctggc atgcaagtac ttgttaggtt tggagacctc
567241 aaacgccagc atttggtttg ttcagagagg gacctgtatg cttttctttg ctctgttcaa
567301 taaattcctt gtaggcgata tttgtggttg atgattttca caaggtttga aaggtaagtt
567361 tgtgcttttt ctgcatgttt gagttgtttt acatgtggca gcctatagag ctactagtaa
567421 gatttttacc agcattttct ttgttaaatc tgttatagcc tttaaattat ttcacttcgg
567481 ttcttttttg cacattatca cagtggaact tgttaaaaat gttgcctttt cctgcttttt
567541 aaaattgcct taaacctgta catatgtagt ttcctgacat gatccctgaa ttctgtttta
567601 agatccattc agcctctaaa ctcttgacgt aatttgattt ataaatatat aggtagaaaa
567661 ccagacagta aattgttaat agggataata tagaatatgt gatttgctat taaaaaaaaa
567721 gggcgtgtaa actttcaaaa taaagatgat tttaaagaaa aaatatatat gttagtgtca
567781 catatttgct aaatctaatc tctttttttt taaaaaacta cttgtacaag gaatgcaatt
567841 gaaattaccg cctgacaagt caatcaggac acgaatctga gagacttgga cacatgatct
567901 tccaaagatc ataagctaac ctgtttatat tgtataaccg tattttactg ctgctttccg
567961 ctatactgca ctgttgctag tcattaacaa actacagcca actaattgca tgtgcgcact
568021 ttttgctcga tttacttagt tctcctccca accaatcaaa taatacatta tcctcagttg
568081 gtatgaggat tttgttttat tttttcaagt gtaggtttct gtttatgccg tattttggag
568141 aaatagattt gatgctactc cggaacgtgc ggtcagctga tcacgcagac gtcagaaaac
568201 gtgcagtgtc accctgcttt cagaccatac cattgtgttg tcaggcggaa taattggttt
568261 tcgcatcaca ataactttaa tcatgttcaa aatactacaa aacaataaat gctgtagata
568321 ttgcactttg ctgatgacaa cgctgcgtgt gtgctcgctt tctcattggt tgaccccgtg
568381 aggatgataa tggcgccgtc ctgtggcgtg tttttgtgga caaggacatg atcgcagttg
568441 cccaactgtg gtgccagtta aagatggcga ggtttttgag gcctaacatt aggagctttg
568501 tctcttctca gctgagaatg tcatcagacc aggtaagcgc attcagaaaa ggccgatatg
568561 atgctgtttc tgattgtgtt ttctaaataa ttaccgcaaa tctaccgttc tttgcatgaa
568621 agggcgtttg acagttggct gttgacgtga ttgtatgttg tagtcatgct gttcctggac
568681 tgtaagcata aagtttcgtc taattatgag atgttggcat gttaaatttg tatttaatat

568741 gtggttattg gatcatttta ccaccaacag tttcaagtac caaagcaagc
tgttggtctg
568801 agtgaaatga aaccatgacc ttcacaatgg agggtggggg agttatggtc
aattgggtcg
568861 tttacataca aaaggtttca attatcacta cataatgcgt ttgtctttta
actcttctct
568921 attaacgtta gctgggggag ctggggaaag gtgctggaaa aggtggcggt
ggaggagggt
568981 ccatcagaga ggcaggtgga gccatgggga agaagcaggc cgccgaggag
gagatgtact
569041 tcaggtaagc cagtgtttct tttacagcaa acaaatgtgc actgtcatgt
catgacctcg
569101 acctgtttat ttccacctta gacgcaaaga gcaggagcag ctggctgcat
tgaagcagca
569161 tcaccaggaa gaaattgacc accacaaaaa ggagatcgaa cgcttgcagc
gtgaaatcga
569221 ccgccacaag ggaaagatca gaaagctgaa gcacgatgac tgagtgaaac
aaaccttccc
569281 aaatgatgac actcaaccta tcagtccagc cacatggttt tctgtgatta
ttccacgcct
569341 tctgtaacca ggccagacaa gaatgtgtgg aggagtgttc tgtaaatgct
tgaaatgatt
569401 atagatggtc ataacgtttg tttccatgac atgctctgac acacaagttg
tttatttttt
569461 ttaaacattt ttgttgtggc tcagcctata tgtatcaaat gaaaaaaaaa
tctaatataa
569521 tccatgatat ggttgcacca ttttatcttg ggtgattaaa aatacatttg
tattgcaagt
569581 ttacttgaaa gtcattaggt gtttccttac ttttttcttc atttttttct
acattttctt
569641 acatgtgaac agaaatgcaa actgacagta cacattttta attattgcat
gaacaatata
569701 caaccacagg ttgtacacca cgccatgcca ctgcttgttt ggaatttgag
gaattaaaaa
569761 atacaagcaa cctgatatag ccagtttact ccactatcac gtcagtgtag
agatgaactg
569821 agatttgtta catgacatgg gttacataat cataggtaaa ctagttaaaa
agccatttac
569881 ggcaattcta ttttacttac ctgcacgtag aagtctattc taaatcaaat
gaagaattaa
569941 gataatgctg aattgttaaa tgggactgta atcttccacc caccaggtgg
cgataattct
570001 gtcttgaacc acttctacaa cacgacaaaa tgtcgtcact tccggatacc
cctgcctttt
570061 accacgagca accccgtgca tctttctttt tgcgcatcat ggcggaccgg
ttcaataagg
570121 tgggcaatcg ttggaaagaa ttttcaatct cttcggtgtt ttggttgaag
tcaggccttg
570181 tatttaccct taatctgcat gtcaaagcca cagaacatgt atttatagtc
cttgcgttgg
570241 tctgcgttcc ttgaggattt tatattgttt cgtgttttag acagccagcg
cggcttgagg
570301 agacgtgcct tcatcatggc agcttcctta acaagcaatg ctgttagctt
gctaactgcc

570361 tttgctctcc atattacatt aatgcagaac taataactaa tcctctgcca attataacgt
570421 attgtcttcg cgccatttca tttgacacta aatacctaca ttatgttaaa agggcattac
570481 gatgggatgt cattgctaac gtagcatgtc catgcgtgga tgatagcgga cttgtgggta
570541 aagtgcgctt gatattttag tattcttact tggctgcagg tacgctggtc aaatatatca
570601 gtgctacttt tacaggggaa acaccgaggc tttatttaac aatgcaataa cgcatctgtc
570661 acgtacaggt tctggtgctg gatggcaggg gccatctact cggccggctc tctgccattg
570721 tggctaaaca gcttttgctg ggtaagtgct gttatcgaag gctctagact acatgttgtg
570781 ttgtcaaatg accattgaac gctgaatttc acagtgaatt gggtgatcgc tgcaggtctg
570841 tcagtgatga tatgtttgtt tttttgcttg agtttagcga ccatgagaaa actcacatgc
570901 actaccatct gagacggatg atcatctgta gtttaggtta tgcatttgcc tcttgccaca
570961 agcgtgtaac acattagtat tttttatttt attttttgca ggacacaaag tggtggtggt
571021 gagatgtgaa ggcatcaaca tctcaggcaa cttctaccgc aacaagcgta agtagtgtaa
571081 acctctaaac tgtggtaatt tattaatatg catgtctgtg atgtgcttaa ccaatataat
571141 tcgagtttca ttgacaatga gacaacctca catgcactac catctgagac atcagtgcga
571201 cacattgaaa tcacagtgga gcatttggca tattctaaag atgccttgtc tgttgttcag
571261 tgaagtacct ggctttcctg cgtaagagga tgaataccaa cccctctcga gggccgtacc
571321 acttcagagc ccccagcagg atcttctgga ggactgtcag gggtaaggac atggctatac
571381 agtttctata tttgtggcat tgtaatacaa ttggaattgt gacaaaaaga ggtagttatt
571441 ggcactttgc tcaaataaaa gtaggggtta aagtgcaatt tacagagcag tggtaaatgc
571501 ttatcagaaa tatatatgtc ccataatacc aaatcagagt aagttgactg cccagaaaat
571561 ggctacattc acagagccag cacgttgttc ctgttcttac tgtgatgagc actttatctc
571621 aaaactgatc ctctgtgatg agacgcaaga tacggatctg attactgagt tcagcaggaa
571681 caagcgcgcg tgcttggaca acctgatgct tttaaaggtc acaggtatta aacctgggtg
571741 tttttctctg accaggcatg ctcccccaca aaaccaagag aggccaggct gctctggaga
571801 gactgaaggt gttcgacggt atccctccac cctatgacaa ggtgatgaaa gttgacctat
571861 ctgatctata gttttgactg tcttcgcttg tatgtggtgt ttgctgttca tctcagtctg
571921 acctttgagt ttgggtccac agaggaagcg catggtggtt ccagctgctc ttaagattgt

571981 gcgtctgaag ccctctcgca aggtgagctt taaaccacta ttctgttcca attcgattca
572041 acttgacaat gaagatcaat gtaaccctta actgatcatt tattgatgag tcctactttt
572101 cggatctgat tgctgagtcg agttgcacct ccaatttgag aactcgttac tgaattctct
572161 tctggatcaa aggctggatg tggttcagat tgccagatca acactgaaat gttgttctcc
572221 ctcctcgcga cagtttgctg tccttgggcg tctggcacat gaagttggct ggaagtacca
572281 ggctatcaca gccaccctgg aggagaagag aaaagagaag gccaagctcc gctatgccaa
572341 gaaaaagacg gtcagaaaac tgaccaagca ggctgagaag aacgtcgagg caaagattgc
572401 aaagtacaca gacgttctga aacaatatgg aatcctcgtc tgagctggtt gtttctggct
572461 aataaagtta aataaatgtt tatagaaagt gttggctttt taattcatgt aaaggaaaat
572521 gacttctgga ccaataaggg tgaacttctg tagttaggag ggtcctccgc tccagaaaca
572581 taaaggtcgg gctgatataa ggaaaattat gctctcaact gagcagaact cggtttccta
572641 cacacccctt aaaggtttca tcctaatcta acctttgagg tttctgtaga aacgcacagc
572701 agcaccgaat cgtcatgccg gcaggtggcg ctgcagggcc ttcaagagcg actatttcca
572761 ctttaggacg tttaatgact actgtgtctt tgggaaaaga atgaaaacac gtaaaactgc
572821 acaagtggca tttggtggga cgaattttgt gtgaagtagg aaaaagaatc cggattatca
572881 gcagatgagc tcattatagg atcatcacag aagcggcgct gcatcaaact gggtttgact
572941 cctggattct gacaccaagc agctactgaa gaacagcaaa acaaaatatc tctggtttct
573001 gtctgtgaat gcgcttcttc caccttcctc ttccacccta agaggattat ttacgcacat
573061 gtgggcttta tgttatttct cagatttaga taaactaacc acgtctgaaa tatattctcc
573121 agatgaaagc aggacgcact ataaagaatc tccatccctg tattctatga ttatgtctgt
573181 ctgtctatat gaggacacag taaagtgcac atgcctattt aattgcacgc tcacggaaca
573241 ctgcctcccc ccaatctgac acctgtccac ctgtgttaag gtggacaaga attcaacagc
573301 aaactgctcc tgtcacagga aacagtttca tggcaaatgt tggccactgc acccagaatt
573361 ggaaggcaaa taagaaataa tctcttaaaa ccatcagggg ctagaaccaa aaatatggtt
573421 taaatggact aaaagccatt ttggaattgg gggggggaca catctgttga gggtttcaga
573481 gatacaccag gctgactgat gtggctttga atggtttatt aaagcttgtt tgttatttct
573541 ttccttttgc agaaagctga aaaatgcctg acagctgata cacttgagcc atgacagact

573601 gcatttagcc agagtttctg atattagcag ctacgactcg ttcttcatgt cgaaatgatt
573661 gtgaacgtta cccattaacg ggtttttata atgaactgaa agataaagca gtgtcaagct
573721 ctatctggtg tggcacaatt cttcgcgtgt gtcatcaaga cacagctaaa tgagtgcttt
573781 gaaacggaat taacaatagg aattaattag ctctgatgtc acacttgacg aacaacgctg
573841 aaaagaaaac tgacaacacg acctgttttt agtgttacac tggagccaaa gacgggttta
573901 catcagccac atattaataa aagcattcac acaaagtctc tttatttaag aaatcgatgt
573961 gaatgacttc tgtctggagg ctttcccccc tttcaccgtg gtaaccccag tgtgtgcgag
574021 tggctctcct gcaccaaagt tgtgaggatt ccaatgactt tcatggtaat tgctatttag
574081 accgcgggta cactgtgcat ttagatcacc cagcgtgtga gtaatgagaa tagctttcct
574141 gttcgagtga tcatttctta tgctttatga gtcattgctt aaccaaatac agcagcaatt
574201 tacccccact gttaatagag ggaactgctc cttgaatagt tgcttattca ttttcaactc
574261 ttgcagcaaa atttaacatt caagattaga aaacgctgat gggaaaatga aggaatgatt
574321 tggaaaagca ggtgggagga cccggatgac cccgctaacc tgtgcccttt gaccccgaac
574381 tgacaaatca cgtcaacgcc atgctgacca acctgaaacc gctagagaat agcgtctttt
574441 tatcccaacg ggtttgaaat ttactgcaga atttagtcaa aaaggcaggt gaactcatga
574501 cagccctgcg ggttccaacc atcggtctct tttgaaacgt tgcagatgat gcgtctgcct
574561 cctcttcctc atcttcgctc acgacaaatg ctccacacca gcttttatgc aaatgcatgc
574621 agacagaaat gaataagaag aggaatttgc ctgtcgccgt ggacccaagc attcatcacg
574681 gcacgagcag cttgtcataa gtggctttag aggagtcaaa aaaaagagaa aaaagatcta
574741 tatgatgtat gctctggctg cactgtaatg gctgtctgta aacatatgat gcaggtgggg
574801 gcactcggcg cctccttcat attcaccgac ctcagtgcgg aattggaatg atatagaatg
574861 acagcggggc aacttgattc attcgttctc gccgaagcca ttatatccat ttactgacaa
574921 gagcagctgt gaaattcaga acaggcagat tgatattggc cgccgagagt cacaaattga
574981 attgagaaat tgagggaacg ccgagaccca acaaccctta tcgactccgc tgtaatgaag
575041 cacctctgag gcgtcagacg cgtgttcgca tagcctcaca acacaaagtc aagagccagg
575101 gaaaggttag gccctaccgc ggtgcacgcg tggcagagtg tccttgagca aggcaatatg
575161 gaaggatttg ggcacttctg aagataaata caccgctggg cggttaaata ttatgtaaat

575221 gatggagaaa atgattccat ttaaacctgc agctgctgtg aaaacgcgtg gagcttgtta
575281 ttcacactgt actgaacagg tcaccagggg ttctccccag attatggatc cattcacata
575341 atctacccag ttttattcct gcactgagct ggaaggtttc ctatgattaa caacacaaac
575401 ataatcagaa gcattttggg ctgtagcacc agaaaaaagg tttaagtctg tattttatgg
575461 tgaaaacagc tcgacaattt ctgggcatta taactccatt cagcagtgtt gctccttgtt
575521 ttaatgctgc agctcctcat gaggacaaac ggccacggcg gtgcggcctt attagaaatg
575581 aatggaaaga agcgttttaa tattggcctc aatggagtaa tattgattcc tgagcagaat
575641 ttgctgtaaa gaagcagtag gtgctacagt atcaactagg actgacacag ttgattgggt
575701 tccactaaac caatatccca gataaactgc tgtaaaacag ccgtgcacat atctgctctt
575761 cacacataaa gatatttatc ccgctaatgc atctaatatg gggtccatga cgtcccccag
575821 gccttccctg ctaattttcc tggccatagt caccacgctc ggggctgtaa aatgccgcag
575881 gagtgtaaac ttgggtcttg gctgcaaacg ccagagcccg tgcagcaccg acccctcctc
575941 taaacgatcg atccgagtcc tgcgacgcga acggcgaaat cagggaatgt tctgctgatg
576001 gaatcaccta catttagaca gatgccgagc gtcacgtcaa gttgagggaa actcagcaac
576061 gtgtgtgaat ttcccctgcg agcgtgctag cggcacacgg aggatatgaa aacagccacg
576121 gtgtgttttt catgcacgct tgggaataaa gcatatggga tggttttaaa gatgtcactg
576181 caaagattcc cctgagtgtg ctgcttatcc acctctgtga gagcagacca caaccagctt
576241 tttatatcta tactctccag cgggggtca cgaaaggcta cagaaaatat agttctattt
576301 tttatttccc tccatctgtc cgctgctttt tgcagctcac gtaatcagag gccgactctg
576361 aagacgccct tgtcgacgcg gtctgaaagg cctgagttga aagaagtttt gacttggggg
576421 gtggggtggg ggggagagac cccaacaata aaccaaccca tttacgtttg cgcgcatgca
576481 atttgcatga gacgctacgt gtggagcagg atggcagcag actgttgctg aataatttgc
576541 aggagttgat gttgaagagg tgtctggcgt atgtataata gattctgaag agagcatatt
576601 tggctccgga gggaggagga accgaccagc aaggctccat ttagctggag agtaaaaaaa
576661 agttgaggtt catctgtgag gcgcccactt tgagcatgtt ttgcgatgaa agtaaagttg
576721 catttccttc tataattgct gtgtatgcac gagtgcttta attccctctt taaaagaaaa
576781 cacaaagcaa taaccagatg gtcacagatt tccatgaata catcaaaatt cctcagcaaa

576841 aatagagatg tgaccaccaa tggccactct ggcggtttaa aggctaaaat catctgattg
576901 tgaatgcttc aatatggcct cgctaacaca cgtgctctgc cgcccacgat gagaggtgcc
576961 tcgttcacgt gcgcgtgcga ggactgacac gtggcgtgag ttatgaatgt gacctgatcg
577021 gatctttaat aaatcgggta gcggcggctg ccagtccaga tgagctcagc gtggcgagag
577081 aaaaattgga gtcgccgtcg gactcaaacg accgcacggg cccctcagcc tcgacagaat
577141 caaaggaggc gcgtcaggtg tccatgggac gaggcgctga agccgcgacg ccgttcgtta
577201 gcgagccaga acagcagcga gcgctttggt ggggctttaa cggtataatc agaggagttt
577261 atccaatttc aagctgtcca caaagagttc cttttaaatc tcttttcgac ggtttcgctg
577321 atcattgagt cttggacggc acacgtgagg tggagaaaaa gactgataag cagaaacccg
577381 cgcagcgatg aacatcccta atgaggtttc ggcccagtgt gtgagagctg gcgctctact
577441 cccacatacg aaaacccaag aaaagaaaat gcttgaagca acattctctg gtaaattttc
577501 cttgcatata cagccaacat cgtactttag ggacagtatc tgcgtagaat ggggaggagc
577561 acccatgaat tgatcataat gagcggagct gtattgattt tctctccagt aacagcattc
577621 tgagtcaact ctggttagac ttaagctttt tctagaggat caacgatttg attatttaat
577681 gggaacaaaa taattgtctc tatgctgccg ccgcggtgtc ccggggaggc tcactttttc
577741 cacattcccg ccagaactca gaaatcagcc ttccgacaag cacaccggtc tcaacagcgt
577801 taacagtctt ctactttcca ctctaattcc tctctccttc cccagcgatt tggtttttcc
577861 ttttggtggc ctttctttct gtaacctttg agagacaaaa gcacagccta cagccttcag
577921 ccgtgcaacc gaaagacttt tcaccgtctg aatcatgcac gacaagcgtg tcacgctctg
577981 aaatctcacg gtgacgggcc ccgagatgtc cagagcgttt tgcagaacaa tgtgaacgct
578041 tcttagatgc tagcctggcc atctgtcgtc ctaaacgtgg cacaagcatg aaaaatatca
578101 agagcgtccc tcagagatga gatcggtggt gaggagggat ggacaacagt gccccagtga
578161 tgtagcctga tgttgatagc gtgggctggc taacagagga tgactacgca gaaatgtctg
578221 tcataaaaga cccgcctgca gcacacaatt aaaatgtaca cgtgaattaa atgcgaataa
578281 ttaaagctct cagtctgaag atgttttcac accagcgtta ctactgtcca actgtcaaaa
578341 tgtgccaaaa acaaatgtgc aacaaagact tcttttcttc tttatatctt tgctgaaagt
578401 ccagagctcc tttatacttc cccttcagcc cgttaattgg accgctcctc atgacagaac

578461 tatccatttg aaccgtttag cacagggaat tcattaggat agcccactta ttcccaactt
578521 ctccacttct tctttcttga tcacgacaga agtttggaaa gaaacagtcc agttggggaa
578581 aagtctgccc gttgacccat ctgtcctaaa catggaatcc ccagtggacc ctgtgtgcag
578641 cttaagttct cttttgattg ataatttagg atttttttgg aacagtggtg cggtaacacc
578701 acaaactctg ctctgtttga cctcattaac atacctgaaa acccacattg gatcagcttg
578761 gtgtgcagtg taggtgtata tctatagaac ctcgactgca tgtttctctc tgaatctcct
578821 ctccctcttt tttctttccc acggggatga aatgaactaa tcagagggat gtgcctgctt
578881 acctacctgt aaatttattc atgacgcgcc cctggaaatg cccagaaata aatcactttt
578941 ctcccatggc ccttgttcaa tctcccagct ctcccttagt gagggaggag cttacagaga
579001 aagggagcaa gagggagata aagaaaaaga aggaaggaga agagaaaagg aggctggctg
579061 accccagagg tggagttagt tagctcgctc agtggtacag tacttccagg cggtgctgca
579121 gcggcaccat caggaagagt aaatatcaga tgtgtttgag gttcctgatc tgagagcggt
579181 gaaataaaga agctgacgcc ctggagtttt gactgcaccc cccacccccg taccgtccac
579241 aggaagagcc aatggggccc agtttagttg agcagataaa aactggccaa cgtgaaagtg
579301 taaaaggaaa ccagaagacg atcagcttcg ctactgtcac gtgacccccca aatcctgatg
579361 tcattctcct ataggatacg ccaggtctaa agcggcatta atatttcagg gcatttttaa
579421 taaaacatgc taacatcgtg aattctttcc gctgaagtgg gctcctcttt aacgtctgaa
579481 ccctaatcaa aatggaaaac cgccccagtc acactccacc tctcattagc atatttaata
579541 cccaaaaggg aagacaggac ctttcaaagg tccgaaccct ttagcggctc catctgggga
579601 tgttcaggat ccaaaggcac caggctgact cggtttaacc ccctcattct ttaaacccgg
579661 agctaaatgc ctttaacagc caattacgag gctcagcgat gacccctatc gaaacccttt
579721 aacacaatca ggaccgttga tggccattaa gggcacaatt acagaaacac cgagtggctg
579781 tgacgtttgc gtgttgcagc agattatcag atggttctca gtaccactga gtacctcttt
579841 tctgtagtta atttgaaaat cagtaagaat tctttattct tcaacagcac gccatcaaca
579901 agcccaaagc atgtttgaaa cggtcgtttt acgaatacag atccccagaa atagatattt
579961 tagtattccg tgtagtgcat cattgtatca atgacggcgc ctcccccctt ccaggttgga
580021 ttctcctgcc acatccgagg cccgatccag gtagctggac taatctatgg gttttattta

580081 ttccatgcag gaacgtgttt cttaattttt cattccaatg atgcagctaa
tctcaaggtc
580141 acgggaactg acctttctag tgcgctacca agaattagct aaggctaatt
atgctcatcc
580201 agttagatct gggtattcag gttcaacata accaattaat tttcatgttt
tcatatgtcc
580261 ccccgtcctc cttggctttt cttttccagt ggtttcacgt gagcattagc
atgacgatcc
580321 ctctcttcat gtgtgacaga catgtatgtt ctttccctgt acagtagtgc
attaatagga
580381 aaagcaaaac atctgagcat gtcaggtggg tcaactccca ctgtctgtct
agaacagatg
580441 ccagtgtccc acatgagcgc tgtcaaaata caaaaggaga aaacacccac
ccacatcctc
580501 cagcatggct cagctgtcag tcaggtagtt gcatgtgttt tgttagggga
agcttgtaca
580561 tccggtgcat tgcataaacg ctccaagagc cgtaacagat gagcatctgc
gcaccgtgac
580621 aaggtccgag gctttggctt tactgtgtat aggactgatc ctaaaatccc
agataattac
580681 ataaatgctg aagaattctg aagaaataaa gggcacaaac acgcctaatc
tgacataaga
580741 aagcaaaaca cagttaagag aaactgaggt ggaagaggcc agtgtgctca
cataatatca
580801 aaagcagggg gatattaaaa cctgctctct tctctgctga tttcaggtgc
aacatttcat
580861 cagatctcac tgaaaatccc gccgaggttc tgctgaggat aaccgacatt
ttagctccaa
580921 aaacatggcg tcagccgtct ttttgctgca aggttggcga agcagcagca
gatgcaacct
580981 tggggagctc agcgatgcaa gtaactctgg gaaaaagttt ccgcagtcac
tcctctggtg
581041 cgtcagcatc atcggcagta accatcctcc tctctcctgt caggtgagcg
tgagtcaccg
581101 aggctgcatc gacgcttttg ctttcatccg aaccttgggt ttcagctgaa
agtttacctc
581161 cgggtcatgt tgggtttgtt tatcttttac agttaaagaa aaaaatggag
acgtcagatg
581221 tgtcggtcag gtacgtgcac tgtgggcatc agaggagact ttaatcatga
ggtgtgaagg
581281 gaggaagacc agagaagcct tgaatggatc cattgtttgc ctctggatcg
gcgggataaa
581341 taaacaacgg gatgacagct gcaggtaatc attgtttttt tatgctctcc
tttcaccccc
581401 tggatccgtc tccctctctg gctcgatata caaaccaaga ggagaagctt
tgggaaaact
581461 cagccttcac ttcacctatt gaagaaaaac ctcacgatgg ctttattgca
ggctttaaat
581521 taatccccaa atgtcgtata aatccagtcg ggtacttgag gttgattttc
tcaccatcct
581581 gacaggttcc gtttaaaagt aaactttatt ttttttgtca gtttaaaaag
gggaaagcaa
581641 aagtaatggt gagtcggttc tgtgtctgct gagacagcac tggttgcaca
ggttagaatg

581701 gtgactagtg gacttcacat tccggtggga tgtaaacaga cgtcacttcc tggaggacca
581761 catgagtcgt ccaaacccgt cacgtccacc tgggatatat acacagcaat tcctgcatcg
581821 atttggctct caagtcttca ataaatagca gagtttgctc tgctgttttg gacttaactc
581881 tgtctcagga gcatcaatat tcttggagct gaaacttctt taatctgtgg acagcgtgag
581941 aggggcgtgc ggggaggtga gaggaaaaca aatccatccc attaatcacg acagctgatt
582001 gtggtccgca gcgtgagcat cttaaattgc tttgccgtgt cagcggctct cattaacatt
582061 tctccctcct cctgcttcta tctaaccttc cttctgtttc tgattttaaa attagcagtg
582121 acagcgaggc cttccagtac aaaaacccgc tcccaaatgc aacaaattaa cctcaacagt
582181 acgtttggac aggaatgaac tgttgtccct tattggtgga gactttggtg gcatttatat
582241 aggtgtgaaa ctaaactagc aacccagtgt gcatgagttt agctgttcac cagtgtgacg
582301 tgcataactt tattaatctt aaaaacaaga gaaagcaatc tctgcactgg caggtaagga
582361 catttgggtg caccaaatca caaaatggat tcccaccgat gaagttaaac atacaaatac
582421 aatataaagt gcagaaatcg atttcagcac atggttgacc ttgaataatg gcggatcgca
582481 cagctcctct catcagttcg atactccagg gaccaccagc ggtgtaaaaa gactgcttac
582541 accagcaaca actgcagcac ctcacaaatg attgttagga ttgagttgca ggagtggccg
582601 tttccatgaa aagtgtgact ctgcagcagg ctttttaaca cccactggaa gaaaacaaat
582661 cattatctca tgtctggttt atttgttggc ttgtcttcac agggaaatcc aaaaggaaat
582721 tcttacccag actttgtgcc acagttttct caccttttaa tgctttagct tagcttgtat
582781 ttggcatcca ggcatgaatg gatggaatat aagacctcct caaattatat atcaccaaag
582841 tttgacatgt aataagaata tatagcctta cagggacatt tatatatgtg ttcacctatt
582901 gtgagtcctt taatggctgt atttgtcccc cagtgcgtca gcatatcagc gggaaatgtg
582961 ttcgtatatt gtatctgaca aatctagtga tggaaacagc tgaagactgc agaagacggt
583021 aaaggctggg gagaagtaat tgtgttctat ggctgcttaa ggaaggtgtt tccagaaggc
583081 tgaatccaca tttattcatc caacaatctc ttcttataac gagactggac tggcaaatct
583141 aataatgcaa ataaaaagat atgaggaagg tacaaaaatg cccatttttt tggttgaggg
583201 ggtgttctaa tgacagattt aatcaaaaag tatgaatgga aaaaagtttt ctgtaaactt
583261 taaaggcaga aaattgtgtt tggaatattg aaaaattcct caatacttaa tcagcttgac

583321 tggctgagag tcttcctccc ttctggctca taaaatatca cgaaatgcaa
atcctggccc
583381 cacttggtcc cacatttgaa cggaagagaa acttattagt cttttcagca
tcattaaaag
583441 cggagacagg ccggagacag gcggcacagc ggcgtcaaaa tacggcaaga
aatcactgac
583501 aagaacacgt tcggtttcat cagcggccgg ctctgtcctc agctttccgc
tattaacact
583561 ccaggaaaga catttcataa tcagattgcg tggggggggaa aaaaatcaat
gaatggaggc
583621 cttaatgacc ctggttaaca tgcttatgag gtcacacgtc ttgtctgaga
tgtcaggtgt
583681 tctcagacgg agtaatttag tgccggagca aacagatgag agccttgaaa
cgagacgttt
583741 tagaactaca cttaatggaa aattaagctg atgattttca tggaaagcta
gcagccgtta
583801 aaggccgtgg catgtctttc catgcgccac cagtagatgg cgctttaaag
agcaaatttc
583861 cctataaatc tggcgtaaaa gggatggatt tattttgata agggacaaga
tttgacctgc
583921 agttttaatc ccttctttca gaccgaagga catggacttg tgtagctgta
gcaggctcat
583981 actggcgaac aagaattata gatttaacca tagatggaga aacagtggtg
tgctttggaa
584041 ttaaaaatga aaaacaagta gaaatttaga tgaagtaatt ttatttattg
atatggaagt
584101 aatggaaggc ctttaaaact tccccttca ggacagagat tttctagcaa
cgtttcaggt
584161 gttctaaatt tcaaagcacc atttttgctt cttgttcggc tgtttaattt
tttcactttg
584221 agacatcagt ctcaatttca tcctttttgt gagtcccatg ttttcttttc
agggcacata
584281 aaaaatatct ttggggcata aaattcccat cctttttttc ttgcttcact
ttcggaggtt
584341 ttgacagctc tgaaatgatt gtaaatggct gttatcattg tgagtaatga
aacatttgca
584401 taggcaccgc cgggctgcgc acacacatca gaccgaaaca gaaagtcttt
cggcaccagt
584461 ctgccgacac ttcagcccgc ctcacaaacg aaagtcaaat atgtaaatat
gaagccaaca
584521 ggactgagac aaatttattt ggagcctgtc actcggtgac aaacaaaacc
ctcacaggac
584581 ggtaaaaaca aggatcgaca agaactcatt ccactcttat ctgtgtgggg
ctgatttatt
584641 ttcctcctct gctgttgtga ccagcgacac gatcaaagaa tgcagagaaa
ccaagtgcag
584701 cgggaacatt attctgtcct gacagcatct tttgttcata acaaagaaga
aagtcaagaa
584761 atggaccgaa tgtgatgtgg atgttaacga gagccgatta taacaggaca
gagaggctca
584821 tgggattcat ctgtgggtta taattggaga ctaattttag aggtcaaagg
tcagatggtc
584881 taaatgtaga agtcctttgc ccgtattcaa tgtgcgtact tctctgtcct
tgacttctcg

584941 ccggccaagt tccaattcta aactcacaat ctgtcaagta cgccccacct tgctccagtg
585001 attttatcag gcgggacttg agcgcacttc ggcaggctag gtatcccaca atgcatttca
585061 ccctgtccac ggagtgcagc ggcaacggca gcatgtagga acttagtgac acccaagtcg
585121 ttcacaaacc tttcatactg agtattgaca agactataaa aatgagctcg cattcgtatt
585181 ttgagcttaa gaggttccaa ctctagttta ttttatgcaa tgactattgt aattctttat
585241 aaataatgga acttttcaca gcaatcacac ttatcagatt tttgattgag gcgcttgctc
585301 aactacagcg tgaattattt ataagtgagg tgggagagaa attggacttc aatatattct
585361 ggaagactca caaaatccca cactggatgt cctctgtgag gggaaaaaaa tgcatattta
585421 tgagctgttt ctgtaaaaga gtaagacagg gccacttcaa tgtgaaatta gtttctcaaa
585481 aacagcaaac aggtgccagc aaggtcacca gagcccccgg ttgagaggtc aaacccactc
585541 accaaaacta tatgcttaca ccttcatggg agcaaaaagc tttaaaaaaa atgccgaaaa
585601 tcggaatatg tctgccttta tgttactttc cctaccttaa aatgcgccct gaccttcaga
585661 gcacactcaa ggtcagggcg caagcaagtg ccaagtttga tcttctgctg atccttcatt
585721 tgattaacct cagataagtg tgagactttt aaaaggcaat ttattccccc aaagccctgc
585781 acctgaacat tgaagcacac catgatgcat ttttagtcgc aagtcagctg tttgcgggga
585841 gggccttgat ctcgcggcat gttattaagg cctaataagt tttcgtctgc gtaggtgtgg
585901 gggcaacgct tgtgttgcgc aggttgcgtc agcgccgtgg tggccttctg aagttgagga
585961 cttttaaatg ctaattgccg tcgtcggcca tctgactggt acggaggaca aagccttcat
586021 ctggcagcag cgtgggtgtg acagcgcgcc aaaacgccac acaacaggct ttgagtcatc
586081 gcgatggccg aataatcagg tagatgagaa gtgtgggagg agcagcccac gttcacctta
586141 gcgttgacca caaccgactc ttacataacc accttcttca tctgaccaaa cgtctttgaa
586201 acgacaacac caaacagtaa agtaaacaac aggctcgatg aggagcgctc acattgaagt
586261 tagccatgcg cactgcgggg tttatgtctt taacatgcgc gcccccggct tattttaaga
586321 tcctcagaag gagtaatgag gccattaccc ataactgttc ttattcgtct gtgcatccca
586381 tcctcacaga gcaaaacaag caaacgtgct ctgaaatgtc aacgagaagc attaaagaag
586441 atgtccagag tttgggcaaa gacccatttt ataggaacgc aatggattct tgcatggctg
586501 ataggaaata tagcttagag tagatccgtg gctgagccat gtggatctaa cgtcctttgc

586561 acccatcaca acaccctgct cctcccacct cagatggtag ctgctcctcc accctggggc
586621 cttccttcca tggaccgtgt tcagcatacc tccgcaagga tgcgtcaggc ggccacactg
586681 cataatccaa gggttcccga gggttccttc ttatgaggtg tgaccctccc gttcctgagg
586741 gtgagtcccg actgagcgag gagagagctc acctcagcct ggagagatgg tggtctcact
586801 ggatcacaac agcaggtgaa ggtcgaaaaa ggagaagcta attggaggta acgttttaag
586861 ttttgtgtta gctctgctaa tgctacgaac tgtatgccca gtttgcagtg gcgccctggt
586921 gtttcagtgt tgctctgcat ttactaagtc attcctccac ctcgttgctc ctgctctgac
586981 acccacacgg agatgtgcgc caccatctgt cactgttggt gacgcgtgat gcctacatgc
587041 atgtagtgtc acttcctgcc attgcacaaa aaaaattgac ggcgaacttg caggatttgg
587101 tctctggttt ctaatactgg cgtctgaaat tccaatctgg gtttatagtt tccactcatt
587161 agagttaatt aagggacccg tttgtacagc ctttagtttg gcccatggga caagtaaaat
587221 aatagagtta ccaagtggaa ctgtccacat gactttgcac tgcctcagtg ccagatcgtg
587281 ataatgttta caacagtgta gcttgacaac aaattcaaag acgtggagtg gagaacatct
587341 ccgggggtca ttcgtgccac attagacgct gaccttttga aataaagtaa cacaaacgcc
587401 accgaatcct tgctcggtgt tgcggccctc gccatccaaa agtattgatg cacaaatgca
587461 tttaagcagg aaagagagga cgtttaggct ggtctttagc atgtccgata cctgttaatg
587521 ttgctttaag gttaaactaa ggtttacttt aaggttaaag tcggggtcag ggttcaccag
587581 ttggtggtta tggttgtgat ggttagggta ggatgctgaa gatgatggcg gtgagtcctc
587641 acaaagatag aagtatcttt gtagtctctg tcagttctga atccaccacc gtcgagcccc
587701 tgacacattg taatagcaga gtcatctttc tcttcttcgc cggatgacca cttatcagca
587761 gtcagttaat ttcctgccag gcctgcggta aacagagcac acacacacac acacacacac
587821 acacacacac acacacacac acacacacac acacacacca tagtggatat cattattttg
587881 gttttatata tatatactct atagttatta gtctatagtt agtgaattag caaggaatgt
587941 tgccataatg gaacatatac cgcactcaga atattttact ttatgttatt ttgttgattt
588001 tatttatttc tgtgcttctg ctggtcgtgc aatgtcagtc ttttggattt tgaaactaat
588061 ccttggtgct ggtgttacgt gtcagaggcg taggtttaaa ttattcagtc ttcgcaacaa
588121 agcccaagtt acattttgcc aagttacaca cacacacaca cacacacaca cactcacaca

588181 cacacacaca cacacacaca cacacacaca cacacacaca cacacacaca
cacacacaca
588241 cacaccagga aattgatgaa ctatgtaagg gcttttgaga ttatacaaga
ttcctgaagc
588301 acacatttga tattcacaag agaaactttt gctttgagtt ttagcaagta
gaatgaaata
588361 tagtccaaac agcaaacgca ctaaaatctg cttttgtggt tgaaggtgca
gcgaagaata
588421 aatgtgtttg ttgagatgct ttgtataatg cagcagcaat atgttctgat
tgtatctgaa
588481 gaaagggcca gattcatgaa tctgaaggca gggtggaggt atgcacttca
ttaactgtta
588541 tctacagacg gatgttgtgc caggcggctt atttaaaata cacccgaacg
tcggagtggt
588601 agtaactgtc acggatctgc cgtgcatttt tttaatggca agcaaaacaa
gacatttttt
588661 aggggtgtgt aatttgatca ttattgggtt tctggagtta tctgatggtt
catgttaaca
588721 gctcagttta tcagctttat caggtaacgg cagctagaca atgcaggagt
ggaggggaac
588781 aaaggggatc atcagatcac tatttttatt tatcttgctc cctccagact
gcatgaattt
588841 ggtgtttacg gatcctcagt ggggcgtcct gctaacaacc ccgccagact
ggggtgctga
588901 tgtctgatct cctgtctaac cagtaacagg gcaggatttt cccagcacat
gacatttgtt
588961 ttgaatttct cagcctccaa ggttggatcc accctgcctg aatatgtact
aatgcatcgg
589021 cgtctctttg gacgcatagc aatgaagcag cctattaggc ctgaacacaa
cctcgggtca
589081 tctttccagg gtgcaaactt ggacccaact atattcagaa gctttggatc
gcgcgtctgc
589141 tttgtctcgg aatgttgaca taattgcaga gcttttctca gtgcttccag
cagggccgga
589201 gttgaaagag aagctggtgt tgttagaggc aacgcgtggc cgactttggc
tttaaagaga
589261 ttcttgctga ctttatttgt ggctacctgg agtttcggac tttgctcttt
catcatggga
589321 actgctttct ttgtgttcta tttattttag tcccccatta gcagtagaag
ttccatgacc
589381 tcggtagaga tgatggggac aagtttttga acttttatca ctttttgtca
cagctgtaag
589441 ctacagctct tctaatgcat cccgccattt gtttcctctt cctcggtttc
tgcatctgga
589501 caagaacaaa aaaagtacac ctacaattta atcacagtgc tacataagct
gtcagacagt
589561 tgcaatgagt ccaaacttaa ttttgtcaaa cagggatcct ccagagcaga
gtacaaaagt
589621 gaatattcac aacagaagct actgcagaac aaacaaaagc cccttgggaa
tatcaatgca
589681 gccatatgcc ccagctatgg tgcctggttt gatgcagagg catcgcacgg
ccttaaatcc
589741 agcgtcagta ccttgcagag gaatgttcac tgcgccaaaa taaagataag
acagcacttt

589801 attcttcttt tcaaaggtgg atgaggtctg gggctaagat tagcataccg cttcattaag
589861 agcacctacg gtaattgttt agttcggatt agtcaccaac aaatcgatcc ttgtgtcacc
589921 cacccaaaga caagattata tcaacccatt gaccgtatct attgatccga tcacaaggca
589981 ggttttcccc acatattcta agcaccagtg ttttgttttt tttaattctt ttatcccata
590041 aattcccatt tgcctgtgaa cctttagtca gggagtgccg tcctgccaag aatgcacaat
590101 gttcgccgcg acgcagcctt ttgtttacag acaagtaaac aacccttgat taaaaagtat
590161 aaagtaaaca tcctgtggaa atcccaccaa tgatgaatag cagcgctcag agggcgttaa
590221 tagcatctga ggtgaagcgt tgccgagacg cctttgaggg acggcagagg caaagggaag
590281 tcacggtgag cacatgcggc acaaacacat ttacatgcat gaatgcatgc atgcgcatac
590341 acacaataca tgttattaca ccttaaggtt ggttataaat ctaaaactgg agctttttca
590401 gactaaatta aattaagggc tgcagaagct tctctggtct taaatggatc agcttaactc
590461 tcctccacgt ttcctttgta accagcgagg gttcgcctcc ccacatcttt cacaaggagg
590521 tctggtgcaa tgtgggtttc agtcggttct agtttgaatc agatctttga catcattccg
590581 aatggtggga ttaggaagct atactggaac acaggaaaaa aagcagtgac aggaacccaa
590641 tctctccgcc ccggcagtcg ggatgcttcc gactgtttcc cagatgtatc agtcttcgcc
590701 cttctggtgt cttttcttga tccccgctga cagaaaggcc ccagtcgctg agactcacta
590761 ctttagagtc cacatcatat caaagaggta aagcgcactc tagatcccgt attcctgttt
590821 taggcaaatg tgtaaaatcc acagagctct ctattgtggc tggcttgcac tttcagttta
590881 ttgatgctta aatcatggct gaaattctca gggaaacatt tctaattgtg tttttgaact
590941 gagataggaa atattcctct tttgacactc tgtccactcc tcccgctgtg cccggttcag
591001 cttgtatctt taatatatct cccagccaga ctgttgctga aacaaaccct gctgagtgct
591061 ctctgtggtc tcactagaac cccaaacaaa cgatcagaaa tgagcaagat taatgctgct
591121 gcccttcgaa gctctttaat aataaaatgg tttcttggca aaaagtctta ttaaaattgt
591181 gaagatgtct tctttcagaa aaagttgttt ttaaccactc ctctggggtg ttctagtctt
591241 gaagtgacac agatattcca aaaaaaaagg tttgctttta atgattgaca gaggtgtttg
591301 gccctgtaca catcactatc aatgttatcg tctcgtcatc ttgaattttt atagttgggt
591361 tcattcacaa cgcggaaaag aaacagacga gctgtacaga cagtttgagg tggagatgat

591421 gccttcgtga tgccgtttaa cctgacgttt catttactgc gaaaggagga gagaacagga
591481 agaggaagat tgagtgactg ttgaaggatt caggagagcc tgaggttcgt ttattcatca
591541 tcctcagtct ctttctgtct ccatcctcat tgttgttatg cgtggagggt gttttgtctc
591601 tgttgatgat ttttctcttt ccagccttct ttcacagtca ataaaagttg tcccgttttg
591661 gccagacaca cctgtcactg gtgacgtctt tcatttcacc gattctgtct ggaacacaaa
591721 cccaattcct gctttccatt gccctcagaa cagggtctgg attcaaaagc agggtcgtgc
591781 cgctccatta gagggacaag cccagcaaca aaaggaggtc agccctgctg aggcattaaa
591841 attccacctg cacagtcaca aactcacctg taaatgtgtc taatcatccg cggcctcaaa
591901 aagcttagca actgtttttt gtgtaggaaa agttctagtt taactctagt ttaggttaac
591961 tacgctatta atttaactct aatttaactc acctcctttt ggacacgtcg attaatatgc
592021 tatagtttta ccaatcttaa tccaaataat taaaaggatc agaagtttag ctaaaatgtt
592081 cttggcattt ccaggttaca tcacatcaca aatcatcacc aataatgaga aacattagca
592141 tcagactgat ttgaatagtg aaaacagtga aaactcaaag caccagatta acagggctgt
592201 tttttttccc cctagaaata aaccttcctt taaatgttga tgggtcctgg tccaattaag
592261 tggaattaat gttttattaa tctcaacaga ttctttaaac cttctcctgg ctgaaaactt
592321 ctcacttgga cgctgcagct gatgtgaatc tgaactgagc ccatgtgcga ttcagcagga
592381 gtaattcatc accttttaag taaaatcgat ctgtttagga tgtcagcaca cttgaagatg
592441 cgcattaaat ctggagaggc tttcagggaa catctggaaa agtcacgttg tgcctctaaa
592501 cagggcctgg aagaaggaca ggctgaatgg gcttgggaaa agtttgatat tagtcctgac
592561 aggagtaact gggctgtatc atttcctata tttaacaaca gtttaaaaca aacttaaata
592621 aactagttaa actttccagt ctatttcata caattactta aacagtaaca aagcaagaac
592681 ctcgcagcac aaattactgc catagtcaca aagcatatca tgctaattga ctgaacataa
592741 ttttctaacc tacttgctaa cactgttata gttgctcttt cattagcacc tttgagctct
592801 gcaagtgcag aacaggagtt attgctgcac cgctgcaggg cccagatcat tatcgaggca
592861 ggctcctaat gacagcggaa agcagaaatc tataggaaaa caacgcactg caccttatac
592921 attattatat tcagcaactt tacaggcagt gtgagatggg agttagcaca cggtactttt
592981 ttccgagcta ccacttaatt tgcacattcg tcaaatcgat gtgagaacta agatgtgctg

593041 agcagggggc caataggagc gcgctcgttt tcccactgca ctaaaatgtg atgagtgtgg
593101 tgaattagtt taacctctgt ttaaatgact gtaaaatcta cccgccagct cctgggtgat
593161 aatattgagc cgtccttaaa atatataaag aaataataat tacatgtgta acatcttttt
593221 atctgcatta aaaaacaaat agacatgctg cagttacaaa caaatgcatt tatttgtttt
593281 tttgatggtg cagatcatga catatgatgt atgtgtgcag ccgtcacaaa gacaatgagt
593341 ctggattttc atgatggaaa aataattagc agtgagaaag tgatgacaat tacttgatgg
593401 caatcaaatt ataaataata ggccttcagg aatgtgttat acgtttggtg cattgatttt
593461 attcattcag tattgagttt atatcgactt ttaagcagcc ttgtacttta aagtctgatg
593521 cagcacagat aatttcttaa aaagcagatt attgtgatta tatttcatac atacatacat
593581 acaaacacac acacatatac atacacacat acacacacac acacacacac acacacacac
593641 acacacacac acacacacaa tatatatata tatatatata tatatatata tatatatata
593701 tatatatata tatatatata tatatatata atacaagttt ttgtgttagg tctgaacagg
593761 agaaataaaa gtcatttacg tttaatgggg cataatgctt cctggactac acaccagctc
593821 tgcttttttg ttatccatct gccttctgtt agcaaagtga gtttgttcaa tcagacttca
593881 gcttgctgtt ggttagagcc aacatgttaa caataagata atgaccgtgt cctgcgacca
593941 taatgatgcc tttttcagca tcacagcagt ttggctgtta ctgaggcatg aactgctcct
594001 caggataaaa agtaaatctg aaagattacc tctgcattgt caaagtgaaa aagtgcagca
594061 cctctctgca gcagtgaagg aaaaaagggg gttctccaac tcaaatactt atttgggaac
594121 tgcaaaaagt atttttaata atagcacaag aaagttacat tcttaaggaa ccttttggct
594181 aataccccac tcgatctgct gacatatgca ctgtaaaaaa ttcagagaaa cctgctcttg
594241 ggcattattg atcgtgagat cagtgtccac aataagcacc aacggaccat ctaatttgtt
594301 gttctattgt acaacaaaag caaacatgta cagcacacca accacagtat ttagaaaata
594361 gaatgctgtc tgaaaatgag tagtaactat cgaaacattc agtttaagat aattttcaat
594421 cactctgtca gtgacattta aggatttaac tataagtgtt acgtgtcggc cacaaagaca
594481 gaatgaagaa taattttaaa aaatcctgga gctccagatg gaggagccgt acccagactg
594541 acaaagggga gagagtggat gcatcttcta ttagagcctt agtaaaacca cctgtgctgc
594601 acagctgtgc cacgccggct gaaatatcag tctccaaaca cgctgttttc tgaaatgatt

594661 aggcgtgtaa ttaagatgaa aagtcactgg gagctgaaaa tgcgtctttg tttatttgca
594721 attataaaag caaaaagaga cgacagtgaa tctggacctc tcacataacc tgctccaaaa
594781 gtggatataa aagatttctg caaataaggc gatatacgac cgtgtccgtc cgcgtcttag
594841 aaaataaata accacaacag gcggcatatg cagtttgtca ggccaacaat aaaaaaaaca
594901 aaacccaatc tattatagtt atgtgacaca cttgacattt aactctgcat cactgatggc
594961 agcttcagac agagagggat ggccgatgga gggggacagg acagtattta tgatcaacct
595021 ggagtttgtt taccacactt agggaccacg ctgcataaaa tataatccat gtggattgtt
595081 gggccatgtg atgaatacta aaaaaaacaa gccaatttat ttgcctcttt aaagattctc
595141 aaaagcacag atgaaacaca cacagttaaa tgaggtgtta ctgagtaaat aaacagaaca
595201 gaatcgcatt agcgggacag gcgcgccgcg ttcacaggag aggctggcgt ctgatttgat
595261 gagcctgctg tcatcaaatg tcaccggggt atgaaaattt aacacgagga tgattactgc
595321 tgggattaca tgcgattcca ttccccacgt gatattaatt ccattttttt tctccattta
595381 aaacattctg cgtaaagctg aggcgctgtc ggttctgcta cacttaactt tgcattaatc
595441 gttgctgtct atgaatatag attgattgcg ttttgcagat tttagtgttt ctaaaagtta
595501 ctggaagaca gaaacaacaa atctaggtag ttttagcatt tgcctcaact atagaatgtt
595561 tgactgggca tctgatactg aaagtcttgt ttctctatgc tccatttcac ctgttcacac
595621 atgacagcac ctgtaatagg tattgctgga atattctaga gcgggcttct tctccgtgtg
595681 tctcattaac tggtattgtt ggctgagggg gaggtctgta gtcctattgg aggggagaaa
595741 ctgctgattt gatcccaaac attctctgga ctacaacacc ccgctgacgt aaaatctttg
595801 cttcatggat tgtttttttt ttgttttttt tgaagaattc agtgtttaaa gggtcatggc
595861 cgatgataat aaaatgtaaa tctgagaaaa gaagcagctt tattcttcct gttttcacaa
595921 tacagcctct gtcactcagc aaattcgccc gtagcctttc aggtagaaaa catcaatgaa
595981 gaatttcctg taaaccagtt ctggctctca ttgacctgct cttggttaga atattggaac
596041 actgagttgg gtcttgagaa gagccctgaa gtcggctgta cagatcatcg ctcccaacgc
596101 tcaaatcagc agatcttaca gccccccccg ccacacacac acacacactt acacatttga
596161 ggttgatcag atcagcagct ctcagcaggg cttaaggaca agaatccccc cttctgtagt
596221 cagacaccga gtcctccttc cctatgaagc ccgccgcaat gactgtccct tgtgtctcct

596281 cctcctccca gccgccgctg attgtccccc tgggctgtcg gaccgcagtt attaaaagct
596341 ggtatttcat cttgtaattc ttatcagcag gagaaacgcg agaaagaaaa acctcactga
596401 gccgtgaaaa gagatgagga gcatcatggg tggcttcaca cactcaaatg ttgacaggca
596461 ttgaaagttt tagcaccccc acacacacac atacacacac acacacaggg ccaggcaggt
596521 gtggggactg agctgataga ttgcccccag agcatggaaa tactctctca gtgttatcct
596581 tttctttggt ggattagagt gtgatcagca gtgtactgac agcttctaca ggcaggtaag
596641 acgaagacaa acaaatgcca aaacaaaagg tttttattca tcaaaatcca aacaggggca
596701 tccgggatgc aggaggattt ctgtccgagt ggtatgcaga gtaagattta gaaaacaggg
596761 accagactgg ctcgcgtccc cccagcctcc ccgggcgacc gcctgacctt tgatgtacgc
596821 atcctaacag cccccccaa gtcattgttg ttgctaacag gttagatcat ggtcccgtct
596881 gtctcacctt gtttatttgc tcagccctga accgtcaaca ctgtctgcag gaagacacag
596941 ttttaggcaa ccaatgctta gcgatgttgt cttattttta ggtttgtcct cacaaagtgc
597001 acaaatcacg gtcggaatca aggcaggagg ggtttccaat ccaatccaag gtctgttgca
597061 acaaaagtga attgtcctgc tggagcagaa gctccctgag gcggagtgtc tgagggagac
597121 ctttgcaaac ggaaagggaa aaaaaaacat ttacacggaa tcagccagtg gtcagtggcg
597181 gctgatgcag cagccgggag cattcacgcc tggcgaaagt tgggcgttgg gtttttgcac
597241 ttcagtgtcc ctcaagaaat gatgtgagcg aggggaaaag gagacaagtg agccgttgaa
597301 gatgccttcc cggcaccgac catgtctgca tgcatgattg ggattggagt caggatgcac
597361 tggaacgaac cgctgagaca cacatccacg ctaaccttca gctgggagtc agaaatgagc
597421 acagagccgg tgtgtttttt ggccagcagt gggtggtgag tgggtggacg tgcccaccga
597481 acacctgctg cttctgattc atggatgttt ccaacgcgtg gaggagtttg ctctttgctc
597541 gcaacttttc cacctcaccc catttgctgt gcaaacatat aaagtaaaat gagtctccat
597601 aatggaggcc attgcagctg gaggccattt ctgccttccg ccacccttaa agtaactgtg
597661 gaaatgaggc gtctccccct ccccctgact cgcagccccc acactctgtc aggaggaggt
597721 ggggaaggtc ccctgcgctc ccactttgaa gcggctgcct tcgcaaccat gaatgaaata
597781 aaacatgaca tgggacataa caggaacacg accatgtgct gctgactccc agaatgtttc
597841 gtacgttgac atgatgtgct ctctcctaca tacagtcatg agggaagagc agtcactatc

597901 tggaacatct agtttaatgc tggtacgctc tcagatgttg tggaagtagc
aagacatcat
597961 ccactgctca atttaggaaa gttcttgtca tcttgaggat attctctgga
tgtggagcct
598021 ctcttcctct gagcagatgt caataggtca aagggatgag ctgaacattt
ggcccaatgc
598081 aggaccggca aaaccagtcc tgttcttgct agcgtttgaa gtcttagtgg
agtctgcaga
598141 ggcctcaaca ccctgagatt caggagagga agacacatca aatcagattg
cataatgcat
598201 gtcctgtctg acctgctcac cccgccagct caattataca tattttttat
accatgactg
598261 catcatgaaa aagaacttgc atatatgtcg cgagtggagc agctttctat
cataatcttc
598321 atgcagaagg tcaacctggg gacataaact ggaagagttc cccagaggaa
gaggtcatca
598381 atttagtttc attttcatct ttagttttcc ttcttctgtg gttgtgaggc
tccagggagc
598441 caacgctgct ttgttcaaca gttgggatcg attggtgacg aacatgttac
cgacatgcag
598501 aattatactt tagtcccaat tgatctgtta aaatgcttca gagggcaatg
tcctcacaaa
598561 tatgacaaaa ttgtcatgga atgtgtttta attgatttga atttagccct
ttttgggttc
598621 gtctccctcc tcttgtctct acctcctgtg taaatgctgt gctccagacc
tggttcaggt
598681 gtcacctgtc agtctgtcca tgattgttct tgtgtttcac ctctgcagtg
tccttgtctc
598741 aatctatggg ttctcgtgtt tagcgtgtgc gttgattggt ctgatcttgg
catcatgccc
598801 ccatccacgt gtttgcagct ctgctacagc gactggcccg acaacacctg
ttccttactt
598861 ttagcacttt aaattacaga ggataatggc tttcctccag tggttcatat
caagtaaaat
598921 ttggcattta aggctaattt agaacaatga catgtttttc ctggagtaat
gaaacgaagc
598981 agacctgatg gtccattagg acacctgcga ggggttgggg acatgtccat
tacatcttac
599041 taggaagaca aataatatac agcgttattc ataaagatta cagaaacatg
aaggaaaaat
599101 aagttgcagc gtccaagatg ctattttgat tatattatga gtgctgaggt
cagacaaagg
599161 gtcggctatg catcttctaa actactaaac aactataatt tattcaagaa
aaataattct
599221 ttttctaacc caactgaagg tgatgtttga aaagacgcca gttcctggct
tgtagaccag
599281 attgtacctg cataggagga ccacgggatg ttcatgtcaa ggtcattttt
tctttttagt
599341 cctagatacc agatgtttca gagaagaact taaacaagtt gaactgggtc
agtttttctg
599401 cattcccatc tgaccagtgt acattattaa gaagagcgag ctaaagaatc
tattaataaa
599461 tccatgcagt gataaagcgc acagagttct gaatggaacc gtctgaatag
tgcctctgta

599521 gggggagggg ggtgtcattg ctttaatgga ggcattgtta cctgaaagtc aagcagacaa
599581 acagagggga cggtttatcg atcgcacatt taaatcacat tagaggaggc caaaatttaa
599641 acaggaccat tcagggctga aggtttctca gcaaaacaaa aacaaaataa aaaaaaaaca
599701 ttaagttcta gtgtgtttac tgttgcaaaa atagaacagg gattttaaat gggaaccact
599761 aatgtataaa ttactcgcac agcgtacatt cagtctttgg gggcttgttt tcctggatgg
599821 gtgtagccta ttatcgatgt gcagccaggg ttttagcatt aatcacagtt aagtttctct
599881 cgctcggtta aacactaatc accgttttag tgacaccaac aatagcacag acccagaatt
599941 caataaaaaa attgcaacag agttcaaagg ccaaaaagaa accctcataa taaatggatg
600001 actttgaaac tctgaaacat tccgtggagc tttataaata agatgactga ttagataaac
600061 cccaaaagta caatcaaatc taattttacc acacagattg ttttaaatat tttccattcg
600121 gcaggttttt ataattgcag cttgctgcct ttcaatattc aattatctga taatttctgc
600181 tttccccttc actccccatg tcgaatgttt atctccctgc aataaagagg agccccccatc
600241 aagagcagaa caatgaaatc tcagatcacc agcattcagt ggatcatgca gctcagtcac
600301 cctgaaagct gatgacacaa caggcgcgtg catggttcag ggtctccgga tcttgacacg
600361 gcgctttttt cctattgacg tttttccctc tgtcactccg aacaggggct tgaacaaaga
600421 ccgtttagga gtcaggagtg ataaaggcag aacccacttc agggaattag catatacatg
600481 tgtgagttct gcttttttttg gttacatgga agctttaaaa cagtgtgaag agcagaaacg
600541 cctgttacac tttgattgtg tccaacaaca caacgtgagg ctcatgacac tgaaggaaac
600601 tcttaataga ctttaccacc tcatcaaatg gtttctaagc acttgactgt acattcattg
600661 tctcacatca cacaaaaggt ttcatacact gttctgtgag aaattcttct gcatcccaga
600721 gcatccagct tgtgaatgtc cttgaagtag acatcattta tggaacggcg gtgaataaca
600781 gtacgattgc tgctttccac aatccaaaat gattgttttt acttgtaatt tgtcattgct
600841 catattgtgc cagttaccaa aagtcttatt tggaggcatt tataagaacg gagcgcaaaa
600901 atatatctgt gaccagtatg aagggaaaaa accacccttg tgcagtcatg aatattcata
600961 acgatgtggg cgtttgcttt tcaactgtga agtcagattt gttttttttaa aataatatat
601021 aaaagcttaa gtttccactt gatgccagtg tggagaaaat gctcttgcca tctttgacct
601081 ctggtcagcc aaaagttttt caaatgtttt gttccattgt ttatttttct tctgtgattt

601141 tgagcctctt cttttattgt aaacctgtat aaaatggatt accgtactgc tgaaatatag
601201 tctgttattt ttgtggtttg tttgttcctc ggtgttgggc ttcatccttc ctcgcgcctg
601261 tttggcttct tgactgttac agtttgtcta gtctcatctg tcccctctga ataaaaactg
601321 cactctcaca cttttgattg tcagaacaca aagtctgatg cttcgattca tcacatcact
601381 tttcgtatgc tgttctgagt ttagttggtt atctttgttt atttcttaaa gctcatgatt
601441 aggaataagt ccgttgccat gtggtgatga tggtcttcca aatatgatgc aaacagcacg
601501 gcttccctcc aggaaagagt tctgctagcc tgcctttgag gacattttta ttaatttata
601561 aatataatgt gtgtgggcta aaccaagagc cttaaacttc tcccggggtt ccacagtgat
601621 gccaaagcac agatggagcc tttctgaggc gactggaggt gaaatagcct caaaacttac
601681 agacacaaag tggcgatttt cgcttgcaag agcaaaaaca aagtcatgtt atgtaaagtt
601741 aatgttgcgt gtggacgccg cccggatcaa agccgccgct agagcccatc aaagcggttt
601801 caggaaagtg caacattgtc atcaggcaga aaaatcaaag tgttatcgat actttgggaa
601861 tagaacacaa atttaatgaa acaaggagga ggctgatgat cacgcgtgtt gtattaactc
601921 atttttaact atgtaaacta cctgtatgag tgcttgtaga gggtctgtgg agaacactgt
601981 gcacctcaaa cagccgtggt ccatccgacc acggctcagt gtgccagcag ctgttggttt
602041 ctgatgagtg acagcaggga agtgtgaaac tgcttgttaa agagagacca ataaaaaagg
602101 gagcaaaaat ttaatcatct ccatccacac aaacggttgc aaatgaatcg gtggcaccag
602161 agctcattga aaatctgcct gcaaatttgt attacacagg caatcagtgg aactgtgcac
602221 gccgcgataa atcttaagcg ttgcggttac aacttcttca tcaatgtttc atgggtaatg
602281 ttttgcagca cactgtggtg gatggaagcc aaattccact gttttctggg taaacacaac
602341 aacatataaa atccaatatc cctgtgttgt cactgcgtct aatactttat aattacctga
602401 tgtgctggtc tgagccttta tatggcagca tgtgatgcct aatggaaggt ttatcgtatc
602461 agccctcgat gtccacaggc ctgatgtgcg tctggagctg tgacacatca ataatcaacc
602521 attctggagg agaaaaaggc tgatccccca aactctctct tccatcctaa ggccagcatt
602581 tagccgcggc gtcctctggt tggaaacatt tcccctccca ttgatgttcc ctcatgcatg
602641 ctgctcaaat cctcataaag ccagaattat agtgcaactg ttgaaaaggg ggcgtaaatg
602701 tagcggatcg ctgcagacag tcaaagctcg tctttcatcg ctggcggagt ttcacacacc

602761 aaacacaatc aagttcacct gccaggatct aaatgatcca tcctgaccgc tacaattaca
602821 aatgtcttgc cacttcaatc atggtaaaac aatctgggta aactgattag atcccaaaac
602881 ttcacatgcg ggctaaatga gatcaaatat ttccaaccct ttccaagaat taatatacaa
602941 attcagcaca tatctgatta aagatttaga agctgtcagg tgaacttggt tatctagctt
603001 tatgaatttc ccctcgggtt ggcagggacc acacaaacgc cccttcagtt tgctcaagcc
603061 tgttagtctt gattccaggt tattgggaga ttatacagtg gcagcgttca tctgccacat
603121 tgttttattg catgttagcc accattaggc tgaagcttta ttgcagggtg ctttgcactg
603181 tgtttgtttt agataactgt acctaataaa ctggatggat cgatgacaaa cccgctgctg
603241 ctctgcgcaa agagtcagag tggttaataa tttaacattg atgtcatcat tcaaagtgca
603301 aaacactacc aagtgtagca gaatccgtca ggcactgttc tcagttttga atgtaaacta
603361 accgcccaat aatagtaatg atgatagagc cagaatgttt ttacagcacc tctgaaaaac
603421 cctccttaaa tggttaagtg tttaacctct gttgctgaat tagtgaccgg gcttgtagag
603481 tgagcgcaga gttgccctcg gcagagaaag tccacctccc acagcctgat aaagggaagc
603541 gggcaggaac acagggtgag aaatgatgga gaggaggtag atcggaaaag gagggctgct
603601 gcagtattca atataaaatc atctgctcgc tggtcaggcg aatggcagca ccttctgtca
603661 acaacttggt ctattcaaag ctaaaaaggt tccactggtc acatttatac agagaattta
603721 gcctttgacc tctgtatttc acccaactct tcagatcagt gggcagccag gatacagcaa
603781 ctggggagca gagctggagc cagaagtgta ttcatgctct cagtcatcaa tacttctgta
603841 atttaaatat gtttttcact gcaatgctgc cttctgtgaa gatatttatg ctggattggc
603901 ttttatattt gcaggggctg cgagtgggca aagtgctgga tgatttgggg acagaatttg
603961 aatattcagt cttagtgaaa gagaattaaa gcctgtaaca ccacgatgca ctgtggtgga
604021 aaggttaatg aaggcctacc tctgaagcac ctcgaaagtt acaatttgca ctcacattta
604081 cattagattc aagtcataaa ggtcacccga aagaaccaat ttaaaggtgg acataatagg
604141 aaattagaca aatgaggcag tgggatggaa gagtcagagt atcaggttaa aactcgagag
604201 agccacagat gtagagaatg agcctcagga tgcaacgaga ccctttcacc ttcgctactc
604261 aatccgacgc cgtgaacact tgaggtcgcg ctttgaacca actcctgtac tttatctgca
604321 gtgttttcag attttgagca gctgaacaga aaaaaaaaaa aaccgtctcg gatctgcttg

604381 cgcacatgac gctaactggt cttcacactt tggatttctt aaagaactta aaactagcat
604441 gtgaatggaa atgggagctt gaatgttaaa atatgtaaat gctcccggtg aaacagatgc
604501 tcgttaaata tttctccaca caccatttgc attaggttaa tatatgagtt actgcagtag
604561 gtgctggaat gaattaaata caaacagttt ctgatcataa atgattcagc agttgggctg
604621 tggagaacca gcaacaggag gaggagaagc caagagcccc acaaaggcca ctctggagga
604681 ttctggaaga tcagatttga actttctccg tatctgtctt ttcacccaag ctggaaaaaa
604741 cacctaaagc cccctttctc atttacaagt cattaaatca cgaagcttgg ttgttgacca
604801 cactgtaaaa ttgagtctta ggaagctcag tgtgatccta ctctcattta ccaaagtgga
604861 ttggtggatt gctggtggat ttcacatcct ggtgttttat aaatgccagt gaagctgcac
604921 agtgagcggc tgtcacgctg caggtctttc actactcgca ataccttcac acttttgact
604981 ggtttggtcg ggaacaggca taccagtaac tcaccgaagg tcatttttca gacacctggc
605041 acctttcccc tcgttctcct acacacaaac taagcacctg tggcaagttg gtactgagat
605101 gaagtcaaac agtagctgtg cgcttgtctt cagacaggag aaggagcata aatgacaaaa
605161 atgctatcaa agattgaagg atagatacag aatgatcctt aatggagaag ggaagagtta
605221 gcaagcatga acagagaaaa agatgtgcct agagagatta taaatataca ccaaaagtgt
605281 ttgttttcaa ccattctgga tacagaaaag tataaacttc atctaaactg gatgatggag
605341 acattttgtg gagtaagccg gttcaatgac attctaggat atggattcca ttaaatactg
605401 atagttccag cccaatcctg taatttttaa aaaaaattat cagctcatgt ttgtgtataa
605461 aataggtgtg taaagatagc tcattactgt ttaaatatgt aacttgctat tctaattgcg
605521 tttgctgccc gcggagcctc tgaagctgct ctctcagatg cgaaatacgg tgtttttgca
605581 agccatccat caatcttcca cttatgtgac gcatttgttt ttctccttct tatgtttcct
605641 ctgataataa gatcagagag gggagcaatg tggaatatgt taatctcttc gcccacctgc
605701 cgttattagc cgcatgtgct gccaacagca cattattagc aattacaaaa tcctgtaatt
605761 actcattcac attcatataa tataaaaata tctcaaattc tgtacaggtt tagcatctac
605821 tgcagaaact gagcatgcat aaaatcacat gacccaataa taaatgcacg gcatacctaa
605881 aaacacttgt atcctagctt gttgacatga ttatatggga ctatttgtgt tgttttagct
605941 ctcaagtagg tctgattagt ctcattactg ttaactgagg ttctccttaa aaaatggaga

606001 gtgctgatcc aggtgacaat gagaccatct ggagtttctc attgaagagg cctgatggaa
606061 aaacaagaaa aggactgatc gccacagaaa aagtcacgtg accactggct tcgaaacagg
606121 gatccaattt tgaacaaagt ggtcatgaca ggaggttaca caccacacac cagggcagtg
606181 aagcgtgggc atcaagtgag ttgtgagtgg gaggagggtt tgggtgtttg aggaccccac
606241 aacttaacgt ttgggatgga cgtgaggcag gaggtacact gagaaataaa tgcttttcca
606301 gggtttatag ttttcaccca ttcattcatc ctgcaggagc agtttccctt cagagaccct
606361 ctcgttccgc cccaacctag gctcctcggt gagtaaagta cattgaagca tctgcttttc
606421 tgttttacac tccgagtaaa cacaggtttg tctcagacgg tcatgtgggt caagttcgtt
606481 aacttaagtt tcaccctctg tttaggtggt aggtgtctgg tttccctttt ccattttccc
606541 ctgcaacgat cacagctgga taatctcctc aagcgttgtt aagtttatct gcaggtatgg
606601 ctcaagctca ggtaaaagaa ggaagaaggc ggcggacgct ttgaccacac acagttgctg
606661 aattcatttt tgtggcaaat agcggcgaag cactgcaggc tgcagatgat ttacgaaact
606721 aaaggagctt tacgatggca gaagacacta atttacatgc accagggctt gtcaaacaaa
606781 gaaccatggg taacgagaaa agcggagatt taatctcaac gtgaggtttg tgtgaataaa
606841 aagaggcaaa gccgcattgg cagctgcaga tccttataga gcgaaataat aaacagtcag
606901 gggactggaa atagttgatt tttcactttc tgacaaatgt gtatgtaaat gtataaaaag
606961 ggcaccacgg aagcgctatc aatgtttttg tactccagtg ggcttctatc tatctggctg
607021 ctattctcat tagagatgat cacacagcaa tgcaaatgca agcctgatgt ttgcagctgc
607081 agggaaaaaa aaaatctgag aggttcttct ctgatcaccc tcaacaacac caccaaacgt
607141 cactgtccta ctgggctgct gcttattaac ataatggaat taaatgattt cccttgacaa
607201 aatcaagatg tgaaatggtt ccatttagtg tgggtgcact acagccttat tttatgaaga
607261 aaaaaaaaag gtcggaataa ttatccgctg ttcttcatgt ggacggatta tactgatatt
607321 aagataataa caataataac attattctca cacacatctc ttcaacaagt gctgtcaaat
607381 gtttccttga tctgtgtaat tatgtaacca ctatcttttt gtgctggagc tcatggaaaa
607441 gtaattaatc tcaatctact gaccaaaatt cctcttttac aaagatgcct aaaatgagaa
607501 tttagtctta caaattgtgt tttgaacagc acacacacac acacacacac acacgtattc
607561 ttgtactcct aacttcaacc ttaaaaccat gtttcaattc tcacaaagtc ctttaaattt

607621 aagaagccaa aatatcttca ttttgtgagc aaaatatcga ttttggtaca cacacacaca
607681 cacacacaca cacacactca cgctcactca cacttcaggg cattttcagc gagctttaaa
607741 ccagactaac ctgcgtgttt tcggactgta ggacaacaat aaaatacaaa gatatgtcct
607801 caatgtcacc atgccgacct tccagtcata cttttcacaa gagatgtgca atcaataaga
607861 cgatttcact gttatttgtc cattaaatga cgtgagttcc ttccagcgag gcagggtcag
607921 acctgcagcc ggtaatgcaa tgttgaaacc tgctgatagg ggagatagag atgcaatttc
607981 gacgatggag ttttcagccc ttgcaataac atcatctagg atggaatcgc aaaactcctg
608041 tctgctaact tctaatccct cgtcttaata gccgagtctg acctgataga cagtgccgag
608101 ccaaatattg cacaaattca gtggcagctg gcttaagaag gattaaaacc gatcttgagt
608161 gcatagttct gattgcagtg ttgaaagaag agtggagccg gtgcgttgtg tgagcagcag
608221 tgtggatatg tgctgggacc gccagagtta actgcatcag acaggtgagt catgtggaaa
608281 caacactatc ctggagtacg ctgacttggg gagaatcccg ctgggattag aacgcgttgt
608341 gcttctagcg gtgggaaaac tccaggtaga acacaccact ttcatctctt tcatgcatcc
608401 agcaaataat gtaatatgcg tcaggctact gaggtattcc cccagtgcag tttaattgaa
608461 attaattaaa ccaaataatc tccttcaagc tcgcagtgta tctatagaga cgttaccgtt
608521 tcagaacatg actaatagga tatagtttct ctgaatgata attaatttga agaaaggatc
608581 gtcgctggca gcttcccttg gaaattgatt caaccacatt tcggtggtca cacggagcct
608641 cccgactcca tatttccagg acaaattaat gtatacaacc ctatgcattt ttccaaatga
608701 ttgcattaat gaggacccgg actcttcgag gcattaagga tcacatgggc tcctgtctat
608761 cgatcggctg tgtgcaagac tccagtttat ttgccaaaac gtatgctgcg gacacaggaa
608821 ataccgaaat gtgactgcat acattcaata tggcgctatg tatggctgac tgaaaggatc
608881 cgcatcgacc ttccctggtc tattattgtg agtggactgt caatactttg attaggaggt
608941 gacaccctgt taaggagtcg ccgccgccac tgaaccagcg tgtgaaaaca ctccgacagg
609001 tgaatccgct ccagccagtc ggtgacagat ccgctgactg gctggagatc tagctgattc
609061 agaacatgaa tgttaaagcg taatataaat aaatataaca ctaaaaagtg cattcaggct
609121 gtgttatagg ccccttaat gagtcaacag acctacctgt gattattgta tccttacagt
609181 tccatttgtc gccattagta ctgtattctt tcctgtccgc agtttaaaac acttttcatt

609241 tccgtttcat cattagtttt ctagtacttc accagtgttg ccaagatgtt ttcattgcga
609301 gcaacgagga aataaaagcc atctttgcag ccaacgtgcc ttcaggctgt cacatgaagc
609361 cgttgagaga gggaaacaaa tagaatagtt gctgcacgag gacatttgaa gtcacataaa
609421 aattcaaatt catgaccgag acaaagttct tgcatcacca tcgccagtaa ttgctgtttt
609481 ttttttttta tgaaaaatga ccataaaagt ccgtaaagaa ttcatcagga gctattttgt
609541 aaatgtgggt gcttactcaa gccttcagtg gagaataatg acccgggctg ctgacaccat
609601 ctgctgtcgg ctgccttttg ttgtagcagc tccacagtgc tgcgagagaa aacaagccat
609661 tcactgaggt gctcaaggac ttgttctata gtttcttttt gatcaaaaaa ataatagcca
609721 ctgtgtgagt ctgtctccgt gctttctgac ttctctccaa agacttatga taattccatc
609781 ttctaggttt ttgccctcca aaccagtgat cccagatgta tcttagagcc agataacaac
609841 agtcatctca cacattctgc actttcactc aaaaacaaga ctaatcctct tagaaacatc
609901 catttttaca ttaattactc tcacatgtga acaatagtca tgaggggacc tactaatact
609961 ctatgcaggt gcagtatttt acatattttt aacaccttta atgatgaagg acaaaggtga
610021 gagcagcatc caccaggttt aataatcacc gtcaccgtag ccgccgtgtc tctgtaaatg
610081 tcccacactg ttatatgtca tcgtatagta acacagaaac gcgtcccatt tttccccctc
610141 atgtccccac cggggctgat aaactgtatc agccccaggt ctaataacca cctgcaggtg
610201 attcaaatct tttatttaag atgtgctttc tccctccata tatgaatgca tatttgtata
610261 atccatttta tgccttaaat taaaagagat ttctctcatt agggggacac ctgacgaagc
610321 taaacacaac tattgattct gaataatccc atataagaac gatttaaacc tttctccgtg
610381 tctatctgca ctgatatgga gagtcacatt aatccttcac gtctctctcc actgaagggg
610441 acagcatttg tgatgttagg ctgatcgcaa aagtttagat tatcttttaa agccatataa
610501 tgctgtatga tccagtcatc cctcttgctg tcctctcttt tttatgggat atttatgctt
610561 tgtgggtttc atgataacag cctctgagtc cttaagtgtt tggagtgggg agacaataaa
610621 agtatctgat tccttcttgg taaatctcca gcaaagtaaa aagtgcacgt tcatcctgct
610681 tcatcactgt tctcagctac aagtgacagt agaattatga aaggcttgtt ataataaaaa
610741 gggtgtaatg tgttgatcat tagcagctat aaaactgatg aataaataca accaaccaat
610801 aaaataattg aagtgctaca tcatagaagc ataaatctgc ttttgaatta tttgaggctg

610861 aaaccagtag ataaaaactg gtttaaagag ttttttcatc agtcagggtt cagaagagaa
610921 cagcatatgt ggacaaagac ttatgctaca tgctaaaggc tacaggggaa agtcatgaat
610981 tcaccattat aaagcacatt aagaactcag tcaaggagtt tctttccctt aatcaccgta
611041 aagttgctcc acgaaagctc acaaagttta ttcataagtc taaggaagca ctaaaattaa
611101 tcctactgac ctttttgtt aagctgatat tgtataattg agcagcattc tactctccca
611161 actcatcgaa ataaaagatg cttggtcagc cacttcagct tcacacaata aagatgacga
611221 tgagaatgaa ggacagacgc acgcatgcca tttactgaag cacttttaaa ttctgatgga
611281 tttttgggtt gtctataatg tacaagagac tgctaaatgt aaatgcccag tgtcctttcg
611341 ttgaatataa atggcattga attcagatac gattaagagc gaattcttca taatacccctg
611401 atttgatgca aatctccata ttccaaaaca aattcctcca cttggagttc agcagatttt
611461 catttcctct tgaacttgcc aatcaagtgt gtgcactgtg tatctgtaag ctgtcttgac
611521 tcgaggagcc acgtaattat ctgtcaagga gcagctattg attcaattaa agataatata
611581 atgaagatga aagcgagaaa gagtagttca gtcacagcgt gcgtgatgaa ccaacaggga
611641 ctttcccctc attctgtgca caattacaca ttaacatcat attcatccac aatctttaag
611701 cttttccaga taaccagtca ggttgcctga ccatatgtaa caccaatgag gaaaacaatt
611761 aattcaggat atattagatt ggtgattttc agctatctgt gacttctcag ggtcgttctt
611821 tttccacaaa ggtaccgcag tggctaaaat atatgcaatg tgacaaaaaa aaacgcccct
611881 gacttgttat gcatttataa aaaagtaaac atatctattt cactttcata actttaatcc
611941 ctcgcatttg tgaagatcag gctggaagct gcaggtttat tattgctaac tggctatttt
612001 tgtatctgtt ggtccaagtt gctgaccgtg aagaatgagg aagactgtag ggtatcacca
612061 ctgtgcagca gaagtcccta ttcttcacag gtctgttaaa tttcagtcat ctctgaccca
612121 cccccgtcgg cccctgagca tccttcacac taccattatt gttggctgtg caacagaaca
612181 ccctttagca acaatggaga tagttctgaa gatgagaatc acacataaat cagggtaaca
612241 accagattta gacctcatta aaatttgtta ggcgtccagt atgttttgta taatgatctt
612301 cagtaaagat cattatacaa atgactgcag ggtttatatg tgttatatgt gttgggaaat
612361 taaaaactgt ggatttgaaa tattattttt ttgtgtatag ggtgtatgtg tgtgtgcgtg
612421 tgtgtgtgcg tgtgtgtaaa tatagccagt tagaaaaaca tttgttgcaa tctgaatgta

612481 tggaaaaagc agagaagctg atgtaatcat atgcaaccat agtaacgcta tagattacca
612541 tgtaatttta ctgtgatggg aatcagctca ccaacttgat tatcttgtgg gaattgtgtt
612601 actgtacgaa ggctgctgga tttccaagga aggatacaat aagtacgttt taaatgcgcg
612661 cgcttgtgcg tgtgtgtgtg tgcgtgtgtg tgtggagaga gagaaaactc ttcgcgcgtc
612721 tctgccatag ctgcaattct gacaatcagc tgcatcaact ttaacgcgtt ggcgcctgaa
612781 ctcgcgtgtt tctcaagtcg ttccgtgaac gggacgggga cgtaattcgg agtgactcga
612841 gttaacttag ctgtgtttct gtccagacgc acagaggagc gtgcgagtga gctctgaagc
612901 gaaggcagcg agccgcagca acacctccag aagctgcgcg ctgccgccgc ggcgctccgt
612961 ctctcgcttg ttttaacgga cgtgcgtaag tgaagaacgc ggctcctggg gttctgtgac
613021 agtccacgag tacgcagagg acaagcaaaa tccccccaat aaagacaaga atggtgagtc
613081 tgcatgttta tgatgattat cattattaga gtccaactcc atcagtccgt tacgcacgga
613141 ccgggggcgc aggataagaa ttgcgttgac agttgtaaag tccataaaag atcaaatctg
613201 tggtaatatt tgaaactcac gcgtaatgcg cacatctatg taaacctcat agatgataca
613261 gcaggacagt aattaaataa tcccgcaggc tgtggtaacc agaggaacag tataataaag
613321 attgtggatc caaataaagg tgtcacaaag tccagataaa gtcagtgcag gattctggtg
613381 acatagacaa gatatttcta ccatacatga aagaattgga gacattcatt ttaaatattt
613441 gaatttatcc atctctgctc aaggtcataa ttaggtccgt ttacatgaca gctcaaagct
613501 gctgacggtt actgagacca ttttttaaat cgggtttgtt ctaattgcac ccttggtgta
613561 tatgcagttt tttgttcttt tttacattta taaagcagtg gataagttaa taaaatgatg
613621 atgcctattt gtttttgtta tgcagcttta ttgacatcat gagcaattga ttttgagacc
613681 gctgaaataa aaccattaaa cgaagagatc acttcaaatc catcaaccaa taaggcttat
613741 atatatacat gtgtgtgtgt gtgtgtgtgc gtgtgcgtgt gtgtgtgtgt gcgtgtgtgt
613801 gtgtgtgtgt gtgtgcagtc tgagctccaa gtccatttca gtctggaaaa attgaggctt
613861 ttttatgctc tgaagcctat ttcacttctt agagaaggca gatagagcag gcgttgcttt
613921 tttactttag ctgaatgatg tgtgagatac tctggaaaaa tgaaaaagga tgagcttcat
613981 ggtcacacaa tgtccctttt attttatcct cacaagtgtg atttctctta ggtctttgcc
614041 cacagcagac cattcacaca cacacacaca cacacacaca cacacacaca cacacactca

614101 cacacacacg cacgcacgca cgcacatata cacacattca aagaaaaccc aaatttgcat
614161 ttactctctt atacgttaac agcactgaat taagacagta tctgggattt taccactgct
614221 gagttttgcc ccacttattt gagacccttg aggacgaata acaaagagga aatgtttgca
614281 gctcattaag caaagcatga tttttaatcc aatgatggaa gggagggaat ggtgatgcat
614341 aatggtttcc agaatgcgct aatgaacagt aatatcaatt tcatgcatat tattgtcacc
614401 taattctgga atgcatagct taccatttat aaccactgta ttgtcctgga aaagtttcat
614461 acagggactc aatgtctcaa agcccgaggt ggaatgatgt tcacgcgttc ggtatttgcc
614521 ccaaaagtga ctgacagctg gtacgagaag gatacgatgg ggaaaaaacc caaccgaaat
614581 ttaatttaag ccgcttttat tcataccacg cccaacaagc cactcacaga agtaaaactc
614641 tgagggtcat tccatctcat ttgaagtttg tacataaaac aatcactgcc atatctacca
614701 atacgctgtt gattgtgcta actgtggcca tcttgtggac tacgttggta ctaccctcgt
614761 gaatcacaca tttgcacctg ctgagatgga cataaacatc tcggatgaat gaataaacca
614821 taatgtttgg ggactgtatg gatttgggtt tttgttcgtg tctttttctc cataaaacca
614881 tttgtgattc tttggggatt tcaactcccc cgccccccctg gagttgtgcc tgaggtactg
614941 ggtggttcaa cagtgatctt tagcacatca ggtttagctc actgagccat ttctgtttta
615001 gtcatagcac caacttactg cacctggttt cactctggtt gggaaaacga gcaatgtttc
615061 cacaacgtgc acatcagttc agcatggcag cagtttggaa cagtacgcct gattcgactt
615121 gcatgtcctc cacagggtgt gtaggcccaa gagtgacagc tgcagtccac ataaacgtgt
615181 gacataacag cagagcaaca cacaaagaca ctgtcaatgc acttggaaga atgtgaggga
615241 atagctgcag caggtgttca gggaagagaa ggtggaaaga aaacctcacc tcagttggtg
615301 aacatcatac tggtattttt gatgtacatg tttcatagat tgatgacatt taggcagtgg
615361 tcaaaagagt ccccgacatc atttgtcggc attttgggtt gggcacgtac gctccacggg
615421 aatgtctacg gaaccggact cactgaagag tgcaagctca tttagaagag tcatcacgct
615481 ggttgtcctg caggctgttt acagttctct gcagtacatg gctcagcagc agatgacgag
615541 gctctgtctg cgaacaggaa ttcatccgaa tatttattgt ttttgttgcg acactttgaa
615601 gttgtcccag aggtgtaaaa ccacaagttg gaagtctcaa taattgaata aagactgtga
615661 ataacaacat ggatgcacaa gcagggtgga tgtgaatggg aagcgagtgt aaacagaaga

615721 ttttacactg tgttgacagt cagatgtggt ctgaggctca aagacctgca aaacccaata
615781 tccagcattt ccttaagggt gcggtctaac cccaactcca actccaatcc aaccctaaac
615841 caaaaacact aaaccctaac tttaacccta acagtatcca atccttaacc cctaaccctg
615901 aaccctaact gtaaccccaa caccatcccc taaccctaat tcacactaac caactaacca
615961 agcgctaacc ctaaatgcta aacctaacac taattcctaa cccctaaccc ctaaccacaa
616021 ctaaccctaa ccccaacctt aacccttctg ctgcctgtct ttctagaatg tttctgctcc
616081 ctgctttgat gctagtgacc aaggtgtaag gtgatacaag acatgttgaa tttgttagga
616141 ttaggggtta gggttaggat tagggttacg gttagggtta gagttgggtt aggggttagg
616201 gttaggggtt agggttaggg gttagggtta gggttagggg ttaggggtta ggattaggat
616261 taggggttag gggttagggt taggggttag ggttgggggt taggattagg gttaggggtt
616321 agggttaggg gttaggggtt agggttaggg gttagggttg gggttagga ttagggttag
616381 gggttagggt taggggttag ggttgggggt taggattagg gttagggtta ggggttaggg
616441 ttagggttag gggttagggt tattcatgaa catgtttgaa cacacgaacc gtcgtgttgc
616501 ctggttaaaa ccagaccccg gtgtttattt catcacatgc cctcccgcca aatcttcttt
616561 tcactcagcc gaatcttttt tcctgccgag gaattaaagc agttcacaaa actgagacgt
616621 atcagtggta atcccatcgg atttacagcc tggattattt acatgggatt gaagtgcagc
616681 agacttcaac agaccaagta tctctttctc tctttgctcc cattcattct ccctcatctc
616741 tcatctccat tattcaaact cgcttctttc catatctctc agtctataat cagtggtttg
616801 gaaacttgtg taattagagt cagaccctta cattcagccc tctagatcaa agttgtaatt
616861 atgaaccttg ataggttctc attttgcatg aaactaccca caggggggtc acgtatcagc
616921 cggcagctcc tcacagtagc atctaaagcc attagtggtc aatgtttcat gattgacttc
616981 ttactgaaag acgttggtgt ccattatctg aaaccggcaa agaaaactca ctcaaatgtt
617041 gaccatcagc agggaaatat gttaaaactg tactgcagag gagaaaaaga gcttttactg
617101 gaatctcttg cagctctaac tctcggccac cgtattagct cgctggccca cttgtccatg
617161 ttggggaaga tggcttgttt tgtcaatcag tgtcaatgtt cagatatttc ttcttcaaaa
617221 taagcaagaa caatggacaa tatgtccaaa tatccaagaa tatttcatat acggtactcc
617281 tatttcttta gaagaaaaca atcaatctcc tgttaaaacc cattcgaagc caaaattggc

617341 taaaataatt ggagtgaatg aatgagaatg agaccgaggt ggtggtgtta
ataaagttat
617401 gtacaactta gtggaaaaaa atacattgtt attgataagt tatgtgtata
catctgttag
617461 aattggaaat cagaagaaac ttaatttgac taattatgag ctctgaatcg
agatgtttta
617521 ctccatgaca gcataaatta cgttggattg ctgcattact taaaactaat
tccaacaaaa
617581 tccccgtgaa agttacacct gcccctccaa aggaaaaaga tttagtgctt
aaaaacccaa
617641 acgcaatatc agagtaatta aagcagaagg gttttagaat acttctaaag
ttaaaagtgt
617701 ttgtcggcgt gtcgatactg cagttcagat ctttgtgcct gggatgaatc
atacagtggg
617761 ttgatcaacg cagcccacgt gtgagcgtct acgctgagat cacgctacac
tgggtcatca
617821 gctgccgatg ctgcagatga agcaccaagc gctcaggcac ttcctcctcc
ctgcgtgttg
617881 agggctgcag ctccgcatcc tgtctcagca aacatgctca tcttagacca
ctgtcgtgac
617941 agttgcacct gaagctccat ttccatctcc gaaatctgac tttctctcac
ttccgtcaat
618001 ttatgacagc gtttttggct aaatttcgcc aacagggagt tgtagcgaac
gacatttgaa
618061 cttctattag ccgcagtcca tcagttttcg attagttatt gtgctggatt
agtgtttata
618121 gagtcaagtt gggttattac tcacccccga acaggaaagc taacatccgc
cgttgtctcc
618181 aacgtcggtt gtagcgctcc cgcttcgcat ttatgactca cagacactcg
aacataaaag
618241 ataaagatga gagctctcct gcatcgatca caaatgttca gtttttactt
tgttttggca
618301 cagctattca tgtgttggtc agagtttatg catatcatca ccattattac
atgttctttt
618361 tcgattaaaa acaaagaaag aaagattcag aaagcagcat tactcatttc
tctacgtgac
618421 tgctgcttta gtgctgtttt cagactaatc aggattaata ctgagcatca
gcctctgcct
618481 gaaattatga actgaaatag cttttgcact gtttatccta tgtttctggc
agtaaataca
618541 ctgcaagaaa gaattgaaaa taaatcttcc cccaaaaaag aactaattcc
cagttttatg
618601 catagttttg ctttgttact gtgacgcagc ttcacactca gagctggtgt
tccactctag
618661 tgaagatgta tttctcagct tggctacaag gttacgcata ttgaaagaag
acgggacttc
618721 ggggtgttat tcacgctggc ggctaaaata aataagtggt gctccccgtt
tgcaaagaaa
618781 ttgggcttat tgttagctag ccggagcaca ttagcagatg catccaatta
gcagatccca
618841 ttcatcgcgc tgtttaagct accctgacgg cctacttttc ctgcttcctc
tgcaagccgc
618901 tttgcaaccc acctccaccc cagctttcat ttaatcagcg tcatttatgt
cgttattgat

618961 gcctgctgag gtccgtatcc agggggactt tgatgctttg tgccatgtct tggcctttcc
619021 atgcagacac gggcagggag gctccaacag agccgtacac cggaaaatct ttcatttcac
619081 gcaggtcccg gcgaattctg tggcagtagg tgaatgaact gcttaatttt taatgtactg
619141 ccatggccag tggtgaataa atatggtaat atttagtatt gcaaagacat ggacatgttc
619201 tcatggtaat tgcaataatt gactcattag cacagactaa tgtcatttgt aggacctatt
619261 tcagtgtctg tagctcatct acatattcag cgaggtatat gttaccgggc aatgattgct
619321 gtctgcagtg agaggtgttt ggtgtgcatg taaattgatt tttttttaa aacccacatt
619381 gatttaaaaa aaaaaaaatc cactagtgtg aaaaactaag gactgaatag ttctcaatac
619441 agtctaatga gctgtgttct gagaccggca ttggaattgg gattggaact cctgcagctc
619501 actggaatca ggggcctcca gtgccagctc ctgtaagatt taattggtat tttatgtctg
619561 tgcttacatt ggagggtgcg agtaggagga tggttggaag gcagtgcgga tggcgatcgt
619621 acgtgcacag cccacgcgga ccgagaaaaa tatctactct ccactctcgc aggcatctac
619681 caacaatgga ggatagatcg accctgagga agcgatgcag tggcatttca ctcttctttg
619741 aagtgctgcg cgatttggca acgtcttact attcatagat ggtagccatt cataaaaagc
619801 ttccttgcac agtgcattat cgcacttcag tgtgattgcg gcgtgcactg cgatcttcag
619861 gctgttcttt gacaccatag ttctgagttc atgtccaatg ttcaaagaat cctactaatc
619921 tatattttat actttcattt tcctgcactt gatgctggcc agcatattat tcagcattcc
619981 cagcgtgcta cgcaagctac agcactcaag tggggggatg ttacagtagc taaatcaaac
620041 aatgggggct tggattgcac gacgagcaca ggaacaatat tggctgttaa aatgcggctg
620101 tgccgtgaga aggcaaaagg tgtggtaaca aacacggggg acattgaagt atgtgattta
620161 tgggagctgg tgagagtgta ctatattatt ttccttatga gggcataata tatcagaaaa
620221 aaatgctact ttcaggagaa tgtcaatgaa aatgacagcc cgcggttggt atctgttaga
620281 ataaataaat aaaaggtaga tattcagagc actgatagcg gcaattctgc ttttggcccc
620341 gccgcttgat gctttctgcc aagaaatcca tccaacggag agccacttct gtcagagcgt
620401 attacaaaga ggtagagagc ctgaaagcct ggacggattg gcagtgtgga gcgggaataa
620461 ggtttgcgtt ctgtgcccgt ctttctgttt tcagtgatta tttttagttt cttctaatgg
620521 ttcggttaaa ttgagaaaaa gggttggatt tggattacac ggcaatgaga cccgatggca

620581 ctgaggttat gaagccgtgg tttgggttga aaataagcca tcccattaca ccactgcacc
620641 attaagaaag ggagctggta aatctatatt tccacctgag ccagccgcac acaatatagt
620701 atgtcatcat caacaggacg atcggcccgt tttcagatct gtgtggtggc gagggaatga
620761 atttcaaatg gttgctgaag ctttttgaag gaaatccaac tcaagcactt cccagtggaa
620821 atgatttgct tacttgaagc caaattgcca agaaaatagc tgcaatttca ttccaggtaa
620881 acttgccatc gatgtatcga tgctggcttt ttacccccta cacacacaca cacacacaca
620941 cacacacaca catcggtacc tctatcacta tgaggactct agccataatt tccaagaccc
621001 taaacatcac gattaaactt caccccggtt ttaatgtcgc taaggccaga cacgatgtcc
621061 tcactttgct agtgaaatgc attttactag gtactccctc agtagcatgt gcaagaacac
621121 acacaccagc acagacacac actccaccct tatttaccta atgtttacgg cacgctcgag
621181 tagacagcag acattagctg acagcggagg cagcgatggc tctccctcct ccgagttaaa
621241 tcaattagcc catctccagt ggggccatgt tctgtttgca ggcctgccag gttcatcagg
621301 tttctgaccg acgagcacac gctacaccat ctgcttggac ttggcccaaa ccttaaagga
621361 atgatttggt ttttgtagtg aatgggctgt gatgtaatga ggccagggct tgacatggat
621421 ccggttgtcc cccgctcctc ttttccacct agttttcttt gatttttttt cctcctctct
621481 ttctaaaaaa aaaccccccct ttgatttcga ggagaaaaaa atgcatttgt ggtgagccga
621541 gctatccctc gtcgtatttg attaatctgg aaaaccagat gaagatagaa aatatatttc
621601 gccggagcag tccactgtcc aatttcagca gggcctgttt ttgatcaaaa ctcctttcct
621661 ctcattcatt catgattgag cccattacct cattctgcta taaagcagac gtttggagaa
621721 aagcctcttc ccagactact tcaactgttc agcaatcttc tcagacgcgc tgctgagtgt
621781 gttgttgaat gcgtgtctgt tttcttgtat ctttatatgt ggacatgatg gttttttttcg
621841 tattgttaaa agcactctga ggccgaggac agagtgtgca acaagcacga ggcagctgat
621901 tgaatacatc atcccttcca tgcccatttt ctcacaacgc tcacacacat atttgagatc
621961 ccaccggcag gagtttggct cgtatcactg tcatctctgc tgatgccagc gcgatcctca
622021 cgctgtgtaa tgtttcgcac atcagcagtc acggctttct ctatcgcatc tgcacatcac
622081 gcgttcggtc tccggagctg aggggtctct tcggagcaat ggcggcagga tgttatcaaa
622141 aacaagtgtt atataacgtc ggctgaagga tctggcctga tgatgctgcg gtgaccatct

622201 gtggagtatc ttaatggttt tacatacatg gaaagtcttc ttaaatgttc aaaagctgtt
622261 ggaaaaggag gaggtttttt taattggttc ataccattct ttttattata tggggaacac
622321 ataaatactc ctgtataccc ccatccagcc attggattgg tccatctata cattaatttt
622381 agaccagctt caaattcttt ggatggatga atttttatcc atccatccat ccatccatcc
622441 atccatccat ccatccatcc atccatccat ccatccatcc atccatccat ccatccatcc
622501 atccatccat ccatccatcc atccatccat ccatccatcc tgtgttcact cccatttgag
622561 gcaccttcca tcactgtaac gtgcacactt ttgtgggagc aactgggagt gaagagatct
622621 tcacatagag ggaacctgag ccagattcaa acccagagcc tctctctcta tgaggcttcc
622681 tttgaatctc actttaaact ggccatgtag cctctctgcc gcgctgttct gagatgatga
622741 gatgatttgc tgtcttctaa aacactaaag cactaaatgc ttttagtgtt ctgcaaagcc
622801 tacgtaactt attgcgctca tcagggggtg catcaaattc aatatccaca ctttaggcct
622861 gccacaaaaa atgatgattt ctactttagc tttgcaataa aatcctgaac accttattcc
622921 ctttattgtt gtagtacact gaactctgag cgtgcacatt tggacattat tgcagtaaag
622981 cattaacccc ttctttagaa ggttttaatc acaactgagg agtccatcag gatgtttcca
623041 tgtagcgcac gatactgtac ttccttattg aataagtggt cagtatacaa gcagctgctg
623101 ttaaataatt aattaaaaca aacccagcat atgtgaggag agcaggcaaa agccttccat
623161 ggtgattaac agtcatcctg cgtatgtcct cttccccact gggatgtaag accacatttc
623221 cacatttcag atctcggctt tctcattgta cttgaaagta tgtcagcatc cctcttttga
623281 tcttattttt cctttactcc tacctggggg aaaaatagag ggagaaaaga gtctttaagt
623341 cattatttgt agagtttcgt agccttcacg gggacgtgaa acacactcta tgtttgtgct
623401 accaaattgg actaaatgag gcaaaaagtg aagggctgtt gtatgaggtc tcttgactgt
623461 gtccattgga atatttaaag tcccctcaaa ggaaaagctg aagatgcctg acaatgaaaa
623521 acaaatggtg gaaatgaaat gcaattactt ttcccccctt ttattgtttc tagaggaatt
623581 ttgtgatacg accactgcag actcctgaga gtgaagcact tctgatctga taacatcctc
623641 ttatcttatc tcatatgcag cacaataaat taaaatgctg cactttgtac taaagtaact
623701 gctcttatat taacattttt tcattcattt taagacagat taatataagt cctgtgaaat
623761 gtttcatgtt tatgcctgct gtggatttta gtgtgcaatt taatgcaaga attcagtaca

623821 gtaactgcaa tgtgtgggtg gtttgtctgt attaagtgct aatgcaggtt aaagttacag
623881 gcttttactc tccctaatgt gcggaggtat ggataatgtg tgtgtgcacg cacatgttta
623941 cattagaggg gaggggataa aggaagtgaa gttcaataca gctaatgctc cctctcattt
624001 gtttcagcta ggatgaagac actgcagagg aagctggaca caagtactca ttttgcgaca
624061 acagtgagga gaagctaatg tatttgactc tgggaacctg aaccgaagct tttagaatcc
624121 ctgcgcatca gcatacatct ttataactgc caactgctgg tcggatatcc agcgtaatgt
624181 acaggagtaa taggattttt ccctgtttgg ctttgatgtg gaggatacag ctgtaatgtt
624241 tggactttcc actgccagcg gagacattag acacagccaa gtttcaatag aaacttcaat
624301 ttagaagaat tccgtggacg gttaaatata aggtagacag caagggaacc attgtgtctg
624361 tatagggcat aatctttgtc ctacatacag ccgcccgctg agccttattg aaaaggagga
624421 gcccattaaa gcagttggta gataggaagg tttgaaggtc caaaggtgtt gttgctgcct
624481 gtctgccact tggagatgag ataagagcag agggaagaga aaagggaagt gcctgtctga
624541 caggagccga atacagatta ggattttctc tccagcagct tcaccagtga ggcgaatcaa
624601 tagaagaagc agaatggaca aggtagtcag cttcaattac aggtgcatta gatgtaaacc
624661 caaatgatga aattacacac ttgaaaagga atgatgtgaa atcataccgc agtaccaggg
624721 acggagaaag tctgcagcca acaccaacag tgtaataatt cctctgtaaa aactccctaa
624781 aaccatgaat gccatttacc aaccatgatg tggatggtct gaaagttggg acaaaaatca
624841 tggagctgga caacaactca ctggactact tttctagcaa tttcacggac attccgagca
624901 acaccacagt cgcacactgg accgaagcca ccctgctcgg cctccagatt tccgtgtctg
624961 tggtgctagc tatcgtcacc ctggcgacca tgctctcgaa cgccttcgtc atagccacca
625021 tcttcctgac gaggaagctc cacacgcccg ccaacttcct gatcggctcc ctggccgtca
625081 cagacatgct ggtgtccatt ttagtcatgc cgatcagcat cgtctacacc gtcagtaaaa
625141 cctggtcact ggggcagata gtttgtgaca tctggctgtc gtctgacatc accttctgca
625201 cagcctccat cttgcacctg tgcgtcattg cactggatcg ctactgggcc atcacggacg
625261 cgctggagta ctcaaagcgg cgtaccatgc gccgggctgc ggtcatggta gcggtggtgt
625321 gggtgatctc catttcgatt tccatgcctc cgcttttctg gcggcaggcc aaagcccatg
625381 aggagctgaa agagtgcatg gtgaatactg atcagatctc ttacacgcta tactccactt

625441 ttggcgcctt ctacgtcccc acagtgcttc tcatcatcct ctatggacgc atctacgttg
625501 cagcccgctc tcggattttc aagacgccgt cgtactccgg gaaacgcttc accacagcgc
625561 agctcatcca gacctctgca gggtcgtctc tctgttctct taattctgcc tccaaccagg
625621 aagcgcacct gcactcaggt gcgggcggcg aaggaggagg atctcctctg tttgtgaaca
625681 gtgtgaaggt gaagctggca gacaacgtgc tggagcgaaa acgcctgtgt gcagcccggg
625741 agaggaaagc caccaagacg ctgggcatca tcctgggcgc tttcatcatc tgctggctgc
625801 cgttctttgt cgtcacgctt gtctgggcca tatgtaaaga gtgtagtttc gacccgctgc
625861 tttttgatgt gttcacctgg ctggggtacc tgaactccct catcaatcct gtcatctaca
625921 ccgtcttcaa cgacgagttc aagcaggcgt tccaaaagct catcaaattc aggcgatgat
625981 cttgaagaag aaaagtgttc taaaaccagg gacaaagtta aaacaataag actaatgaga
626041 ctcctgcact taaatgttcc atcctgtgac cacacaaggg ttgtgacttt agtttgtttc
626101 ctagatcaaa cagctttgtg ccatggcgtt ttagattcct acagcctatc cttcttgtgg
626161 acagcatgca tgctaaagta aaagatttta aattgattca ttgtggaaat cgtcgttaaa
626221 aactcaaaga aaatgggatt ccaagtcttg attttcttgg tacaacgaat ataaaaatag
626281 tcttataagg tgactgtatg gaagaagtgg atggggtaac tgcaggggga aatgaaaagc
626341 catcaaaaat aagttgtctc ttcatcatac tgtatctgct gctcacatat aacgtttctg
626401 tggtacagta aaatacctaa atactagaga cccagtaatt tatttgaagc tgtgcgtctt
626461 tttttctgtg tgagtgcatc tgatggtagt cactgtaata tatgcatgtg tgagcgatgg
626521 aggattgtca agtgtttgac atttatacat gatgttccaa agcgaggcga ggcttatctc
626581 atcgtggctg agttggaaat ggaaaacagc attttcactc tgaaatatac tagcacaatg
626641 gcaagaaaaa ataaaacaaa ataagatatg tatgagaagc ccgggtgaag gtgagagatc
626701 aaactgacag caactgcatt tttagtagtt taacgtgtgt gtgtgttttc taaatttatg
626761 tgcttgattt atcggaacat cggcgtgact ggcaggacca acgcattaag atgttagatg
626821 ttaaaattga cggaagggga agaaacagct aattttagtc cctttgaagc cataaattca
626881 tgcttctgtt gacattatga atcaaaaaag caaaaaatca ctataaatta aacaaagcta
626941 gataataaat agcagacagg gtttgaggtc ataagcttaa ataagaatat tagctaatag
627001 aattccttgg tgacttgtga ccctagatgt cctttctaaa gaaatttagt atgtgtgtag

627061 gtggtggcag catccaataa ttaaaataca aacagcatag ctgtcctcga
tctcctcagt
627121 cttgaataaa ctaattttct tgacagctac tttaggggtt agcacaggaa
atgtttactt
627181 ttgtaatggc ttttcatagt ccaaatggtt tatgcgttct gatctgactg
tagaaacatg
627241 gtagaggaac cggtttgtta agccctttttt tatattggaa tgataccgct
tccattttca
627301 ctgataatgc agctgtgatg agccaaccgc cgattgctga atcaactcct
ggattcattc
627361 cagaagaacg tctggtggga gtgtgcagac atcaggtcaa aggctggtgg
caggaaatca
627421 gcgacacact cctaaagagt cgttcacacc tgtcctgcag gtgacatttg
atgttgctct
627481 gtcgcttctc aaccgtctac agaatgtggc tccgctcaca cgctggctaa
gcagtcagtg
627541 gtaccatggt ggaaaagacc tcaggttaca aatgttgaac tgcatacatc
atacatgctc
627601 cctggtgagt gaggtgactg tggagtggac aggagcctcc acatttgtgc
gtaagtgtcc
627661 aacttcatct gattgtgtaa atttacctca cgcactttat agtatcaata
ttcaatcatc
627721 acctttaatc aaaagtgtgt gtacacatca acacaaagct ttgcctgtat
ttgcttgaac
627781 ttaaatgggt gtgtttggta catcagcgag tccccctcgt taattgggag
ccccactgtc
627841 aataacagag tgggtgagga cctctatgat gttgttctcc tccttccctt
tgaagctgtt
627901 aatgaacatt tacagaacca catagataca cacagtggag aacataatgg
tatgaaacat
627961 ctacaaaaga tagtagaagc atacagcaaa atacaagtcc caatttaaat
atgcatcttt
628021 tcttatcctg gtggtctatc aactgcagga tgacacaaat ttagcaccat
ggctaattaa
628081 atatgccagt ccagacaggc cctcattatc ctgaaggtgt aactaaaata
aataaattct
628141 cttcaaatat gcaggagctg aaacacacaa cagtgttaaa caaaagaatt
aagtcatggt
628201 gatggaccca ccagctattg atttgtgccg ctgggtaact gaggccaaca
aatgtgctta
628261 tatcaattat tgtgtatgag agatgttgaa attcaattta ataagcggtg
tgcctggatt
628321 aaataagcct ccattccaac aaaataaaat aatgtccgaa gtagtttttt
ttagggtttt
628381 ttggtgggtg actgagggaa aaaacagaga gagcccaaag tggtgctgtc
agtgtggatt
628441 agtgtgaata ggagctggat tcaaacaagg tgagattgcc ttgagcaggt
ataccgtaaa
628501 ggtcagggct cacttaatct cttgggatga gggctgtcac ttcttgactg
gtgtatgaag
628561 cagggagctg gaagggagcg tggagggaaa cagctgaatg aggagacggg
agtgagaagc
628621 atgtaacaat ggggtagaat tcagagagaa cagatggagg ccccttcaga
tgtggttgtc

628681 tgagacagat accagcaggc tttattaact ctaccaccta aagagtgaca gagaagctcc
628741 gagcatcatg ggaagatcct ctagtctaca cctccagctg ggccctaatt atgagcttaa
628801 tcaaaaggta aagcggtgac tgcaccttaa cctggcgggg aaagacactt cctcaagaca
628861 ggagctggac ctgaagagga aggttctggc tcccagagtg catttatgaa tttttgggag
628921 ctcctgtaaa tttaaatagc acgggcattt tcgtaaggcg ctcacatttc tacattataa
628981 atggtgtcca aaatgtctct tcccattagc ccacatggaa ttagtcccgt gatggtttgt
629041 tcctctctga gtgtggaatg ctgtgggaat atgacctatt ttgcacgtat tccaaccatg
629101 aactggaacg tagtagaaaa tgaaaatgag aattctttcc gaagctggac cctcagtcgt
629161 ggcagtgacc atgtctgact gggactgctt gtacaaatgg tgcaggttga attcactgtg
629221 cttctactga cacatttgaa cagcctctgc cgcacagaca ttggtttctc cccaggtttg
629281 attccacttt cacgcactct ctaaggttcg tagggcctgt aaattagttg tttctctgtc
629341 tcctgttagt cggactctct ggtccaaacc ttgtgtctgc tgtcgagaga cctgtcaaat
629401 ctttcatggc aaacttcttt gagccgcatg tggttgtaca gcagcagcgg cccaaccaac
629461 cggttgatct gggcaattag gagcggtcca aatggtccaa atcacatctg atctccatat
629521 cattctttgg actgatgcaa ccaattgcag tgagccagca agagataaat tatgcatttt
629581 ccaccctcaa aatcagacag gaagctgtcc taagtgatgt cgcactttca acagtgtgta
629641 atatgcatac agatccatac gtgcatggtc ataactcacc tttgttgaag aaaagaggaa
629701 tctacatgat ggtgaacatc agacgtaaat gattaaatgc gtgcatgaat attttacatt
629761 tccttcctcg tgcatgataa tcaagcctaa ttcacatgta ccgcgaaggc acaacgggga
629821 cattcacgct gcgggaagtg gaagatacga caggctggat tccaaggaat gaaatgaaat
629881 ttagactgat gcagcgctaa taatacatgg aattcttttg tacagtggaa gagttggctc
629941 caaggggggc agcaactgag gcttacgagg gttaaataca agcagcctcg caccagcaac
630001 cgtcacttct gcctgaataa ttgaaggcaa agacaggagg gaggacttta accacggatg
630061 gaattcattc agtgataaat ctttctttgt gtaattgtca cctctgtatt cttgtacatt
630121 tagtgtcccc acatgcgctg cagctctttt gagtttgcct tgttaatgtt tcccatccgt
630181 tcgccaaagc gtgttaagtg cagagtttta tagttattcc accattttca tttttcatct
630241 caaagcagat gtgttatcac acgtctgcac ctgggaccaa accaaacggg gagggagggg

630301 gttcaaacaa ccggctgcaa aaagggttga aattcatgga gaagcacttt ttatttgctg
630361 aatcagtgaa agcacagatg tgttgaaggt taaccaagca aggaaatggc ctctggtgaa
630421 agctatgtgc actgaaaacc ctgataaatg accgttgagg ctgaagttaa aagggatcca
630481 tgtatggagt ggagaatcat ttctttaagc actagaaaac ggtgtgatcc tcctcagtca
630541 atacagttta ttggaataca cagaccactg taggctgctc aggtagtgca gcgcatgcaa
630601 acgtaggagt aagtaaaggg ccagaggagc cacagtcgag gtaatttcag cgagaatgga
630661 gaaaagaaag aggtatcagc aggggagtca ccgctgaacc ccgaagtctc atttctaatt
630721 gttacaacac acagccgcca tgaatcacag cctcttctgt tcacaatcag gccgtgcaga
630781 ggtgacatca cccacgccag atttacctgt cggtgagctg acaaattaga acatcagtta
630841 aatcagcggt ttggattcaa atttaaaatg atgacagaat tttaaaccca ctgctagacg
630901 aacattttgg acaagttgac tcataatgtt ccccaaacaa cagacatttc tatgaatatg
630961 tttgtttgtt ttttaccaaa ctgcagtttt tcccattttc tcattctctg attctctcat
631021 tcactttcaa cactttcctg tcaaaggcag tttggcatca cttttacttg gaatgggttt
631081 ttacaagagc aatcttttag taaatattca gaatggcgcc ccccgagacc actttgtcac
631141 tctaacgcct cttttctcca gttttgacgg gtggcagtat atcaactaca gcttaaatca
631201 tttagaaagc cgtgagtttt aaaaaagctt gacaccacgg agaaatagta atgaatagtt
631261 tatttatttc tacaaaaagt acaagtaatt ttactataaa aaagaataaa ttataaataa
631321 atagagaaaa acagtacgag tgggctgtca tctgagcttg agtctcttat taaaaggaaa
631381 aacccagagt aattgaagcg agttaatatt tccttgtagc ggaacatctg gagaacatct
631441 ggatctggaa ggtctcactt attagtgcct gaatcctcct tttatggctc ttaagtagag
631501 gctttggagg ttttacatct aaaacattaa catctcatct ctgactcact tgcagatgtt
631561 tagatatgag gggaaatca gtcatcacaa aaaaagtcat aaatgttttc attttgatgc
631621 tgtagtaaaa aaaatatatt atggtaatat acatttttgg caggaaaaca attgctcgta
631681 aacacacgca cacacacaca cacacacaca cacacacacg cacacacatt cacaacacag
631741 atgaatccct cgttattttt tcaatcccag cttcctgtct tagtaaactg ctgccaaaca
631801 tccgtgctgg gtgatatgtt atttccacat ttgctgcgat gccttcaagt aacagtggga
631861 ctaattagtg tggaatggca aaccaaatgg tttgctttct acacatttgg aaatacacca

631921 ctaattattc acatgattac tctaattatg caaatctcaa ccggtccaac aatcacgttt
631981 tttcggttat ctgactgcgt cagcttttag ctgcaacatg tttattaaaa aaaaggagat
632041 ttcctcagtg gtaaaagctt tgaaacgtgg tttgacaagg ccgtcattag tgatcaacaa
632101 ctcccacaca gtaatgagca attatgtaat tacgcattta aaagctcttt atttaaagac
632161 cgatgattga tgtaagatga tttacattca aagccctgtt ggtgtctcat gtgtttgttt
632221 gttttaaact ggaacatttc ttttccatcc tcctttggtt ctgaagtcaa attgaaatcc
632281 cagagactgg gctgagtttg atctttcctg catccagaca gcagcgctcc ctctgcagca
632341 gcgctctgct gcaggtatag gtcaagctgg atcgtatctg cttttagggc acaagcacct
632401 tttataaatg tggcaacgtt ggcctttctg tcaacccccg tttcagtctg cagccacctg
632461 tggagaccgg attagcccag ggaggcgatt cttttacaga ccccaccaga agggaggaac
632521 agagccgagg cagaggggag cggagaggct gcagtcgggc ggagacgaag aggagcagaa
632581 ggtcctcaga gatgcagaag gaaggttggc gtgaccacat gatggacaga atttctattt
632641 tcttctgttc tcacccctaa ctcatccatc agttcagacg gaggcgttgc tcttggtgaa
632701 tacggacgca ctaatcctga tatcatcata atcgaagcct gttaaactaa atacacatca
632761 tttataaaat tccttaaagc actcatttaa cttgttcact tgttagtatt tcatcttttg
632821 taccctgaaa gtacttcctg tgcaagccac gcccactctt ggctatggag cccttctcca
632881 cacccctgc aaccaccaca gaaggattat tctcctcctc aacaataacg ataacctcag
632941 accgtaagtg aatcagagat gcatccgctg gaactaaacg gccctaacag gcagattccc
633001 tgaatacaga tgagaaaaag tgttgaaatc cgacgcaact gttgctgtca cagagctggt
633061 cggagcgctg aagtaaaatt gctttgttat gcagtttcac gcccactcag ataaaattag
633121 gcatgctttt gtgggattat gagtcctgca tgtccaagat ggaccttaga tgtctctttc
633181 cagaacttat gacccaagat acagctttat gtgaatgctt tagaagcttt tgaagtgcag
633241 aataatttta ggagccagtg aagggttccc atatttcaaa gtgcttcccg gatggtcagc
633301 tattctctga tacgccgtct ctcccgcaga gatctaatga gaagaagagc tgcatcctgc
633361 tgtatagggt ccaatacaac actgagtttg aactcatcca ccaagcacat atatactgtc
633421 tttttttttt ttttaaatcc atctttaatt ttcagaaaca cgattctagc cgcccaccgc
633481 agcgttcctc taggatggca gataattaaa tgtctgccaa atgtctcact ttgcatagaa

633541 attaaaatga actgactgtt gagctctcag agctttcgtg gtgttgttaa ttccagtctc
633601 catgtttcat gtatgagaaa aaaaagacag ttaacaacat gtatccaaca ggtcattaaa
633661 tgctgaactc ttgagtgcca ataaatgaat cagccattgg tgaaaaggtc cagaaagttg
633721 ttgttgtcat cctgcttttt agaaaaaaaa cctaaaaaac attagaaatg acaaagactg
633781 ttctgaccac gacaggggtg aaaaagagaa ccaacgtttc tcagatttga tttataaagc
633841 ccgactaata tgattaaagc cacgtgcaat ctcagcaatc tggttcaggg acacgaagat
633901 gcaaaacatg ggaggcagat tgcttccttg cttctcctgt ttgctgcctg gagagctaac
633961 aggaagctaa actgctgaat tggtgaaacg ccacctgatg tcctttatta cgacccaaaa
634021 ctgtggcggg gaaaaaaaat gttgtcacaa cagcaggata gtagctgtag ctgcaggctt
634081 caggtgcctt ccagcaccaa cactttaagt tcagatcttt tcgttgacga tgactatttt
634141 gctggaaaat ttagctgtaa aatgacttta cagagcagga agctgccgtt accctgcaga
634201 gacactgctg tactgtaatt tgggctcgtc aaagtgattt gtgtgtgtga ggatgcgcgt
634261 tcatgatccg tgcctgtcag cgcaggcgtg tgtgggtgtg caaatgcagg catgaaaggg
634321 gaacggcgtc ttctttgtac tgaagtcttg atattctggg gtcagcgttg gaatattgca
634381 caaaataaac tgacaaatga tagcaaaaga ccagcttggc agtcacctta gggttttttt
634441 taaatggggt gtcagatggg ggggctttgt tgatagcata aggccgaaga gctgcaggat
634501 taatgtattt cttatttact acttttcaag gagttatatt tatcaggttg atatgcagca
634561 ggagcctgga ggtatttgca tattgcagtc tgtcaagcag cgtttgacag aagaaacaaa
634621 tacaacaata aacctggata catccaaaga tgataaaaat ctgcttctac taacagggat
634681 tatgctaatc tagctctgtt tctggtctca gcatgtctga cttgatttat aatgctgggt
634741 ttgtattttt aaatgctcta ccatttgtgt atataatgcc agttggggcc atttgacagc
634801 tgtttgctta tttacatgca aaagccacag tcgatgcaga tgatgctgca gataatgata
634861 agatttcagt tgcagacctg cgcacgaggc ttctgcttgt cccacaaatg ggatgagaaa
634921 gaaatctgtc tgctatccag aacctactgc ttgatgaatc aataaataaa ttcactatta
634981 aatcagtttt tgctgctttt taattgagtg agaggtgaaa ggcttttctc aacatgcaca
635041 atcagaaaca aagactgttt taaaattcat tgcattcaag gtctgatatt ccgagtttaa
635101 cctcatgtat aaggacaaaa ttataaaaat gtgataataa tcatgagctt gatggtggga

635161 ggtctccaac acccctagga aagaacagaa ggttgaaacc agtggaacgt gagctgaagt
635221 ctgcataaca caagagtttg ttaaacatca aggtggtagc tatgtataca ttcagggaaa
635281 ggtggggggg tcaccttgaa gaaagtgaaa cgttgaatcc cagcacggcc aggacattcc
635341 ggtgtttgat gctcttcaaa gttcatcaaa cagaccatct gccagggcga ggtgggatcc
635401 ccgggacact gggacgtctg cagagcgaag ccgctccatg ccggattgta tcccagatca
635461 gctgatctca gagatcccgc acatattttg acactaattt aaaggtttta gaatcacatg
635521 gacgtgcaca tgtcttgctg agctgacagt cagaacagct agacttattt gctcagatga
635581 cataatagag tgaggctgat tttcattttg gctcagcgtg gaaacacaat tactgccgct
635641 gtcattctca aacgtttgca agtgtggcaa aaacagagaa gattgatgga ttttatgcat
635701 taccttggat ttaatgatca agaacagact aatgaagctt tatggttgca gtttactcac
635761 cagtttttaa aggtatttca aaaagcatac atctttacat tctctatttc tttactgaga
635821 catttcttcc tgaatgacca ctatattgca atatattaca taagctgcat aaatgtctta
635881 atgcttgcat tcatccttta ttagcattaa caataacaac aaaccctggt ttattttcac
635941 ctcaggtctt cagttgaatt tattttgggc ctttacattc tgtggagaat ggggattccc
636001 acatgtcagg cactactcgg cccatctgat cggttccact gaggtcttca tcttcctgga
636061 gaatatttta gcaaacagaa ggatgagccg ctgtgtgact ggatgtgtca cagatcaata
636121 caaaattttg tatgttatcg ccccgtgcac ctgcataaca atacattcaa tccagccgtc
636181 ctctctgacg accccattcc tccgttatcc tttgtccatc gcaacaagct atccatcttg
636241 ctgtcttcta ttatcttgtt ctttgtctcc tgtggaaaag taaacaggct ctggtggagg
636301 agtcctgctc attagccccg ttggtttgac agctacctca taaactaagc tgctgttatg
636361 gtaactaggg tgtaacatac gttcaaacag ggaactgggg gatcttaacg agacaaatag
636421 cagagaaaat aacccggaag aagagtcagc aaaggaaaaa aacattgaga tgccactgtt
636481 cccatacaga agctgagatg taacaccagg attttgtgga aatacactac tttattcatt
636541 tattcaggaa gagatacaaa tcttcatgga agagatacaa tcttcatcag actgtaaact
636601 gtcatcccct gcatgtgtga ttgaaaacat tttttaaaaa tggaaatgtt gtgctgtcac
636661 atactgtcct ccattaaatc caaacatgcg cacacaaaaa atcctctctg aacattttcc
636721 cctgttgccc tgagaggaaa tgaaagtagc catatcagcc agtaataacc ctgtgttgac

636781 aatgaagctg tggacaggga ttgaggcaga gataaaaaca caatgattcc
gagaacagaa
636841 ggctcacagc ctgatcgata cacattggcg tactgtatca ccagccggca
aagaaaatcc
636901 tccacgctgc taatgcaaat ctgttccttc tccagtcaga aattaaaaga
acaactataa
636961 aaattatgtg tttgttgttt atattaaaaa catgcaaaaa gaaataaagc
agctgccgtt
637021 tggagtgttt ttactgttta ttgtagtgct gaaggtttaa atctttaaag
ctggagggaa
637081 tcagagtggt ctttttaata ctgttcattg tgtctcttgt taatgggatt
caatattcaa
637141 gttcccctca aacttgtcca tcaccggagg gtgggcatcc tgcaataaaa
taatcccaat
637201 actgccttca tgtggacact ttaattcatt acattgcgat ttggaacgga
atcaaccaac
637261 caaaattgct taaataaata gatgaataga atttgtctgc ctgccttgat
ttcttgcacc
637321 aacctggact ccaccttaac gcttcacgct aacaaaaggc tcagtgttca
tttgagtctg
637381 tttcagtggg atcctgccaa actaggttga ttatggctac gaaacgcagc
atttttggac
637441 gtaaagtgaa gatgcagaca gaaagtgtgc gcacctccac acccttaaaa
aacaaacaaa
637501 caaaaaaacc tccttattga agctacagcg ctacacgtca ctgcccccct
gctgtgactt
637561 ttgagtgttg caatctggag catccctcgc ttggagtcca ccactgggca
gagagtgcag
637621 cagcagcagt aacggcagcc tgaagttgcg cacagactgc gaacataatc
ttgtcacagt
637681 gaatcatgag gttttaacaa tgcgcgaatt gttgcgtggt tccggaagag
gcttcggcga
637741 gtctgggact cagtcacatg ctcggacggt cagtcgccaa atccccaatc
agtatgtgga
637801 gccatgctga ccgggttcct gctgtcagtg tgtgacaaca ctgccgacct
gctgacaccg
637861 gggattcctt cctcagcatg cctcacaaga gcaaaaaaga taaggtaaag
ttattttcat
637921 gggcttttgt ttgagtcatg caggactccg cgcgttgtgt ttactgtccc
tctactgtta
637981 ctcatttttt tgtcaggaat ccaccaaagt agggagaggc aaaaaatctg
ggggtaaaaa
638041 cgggcctgtg gatgatcgag atgtaagcat gacttttcta aacttctaac
cctacagaga
638101 gaaatgtgtt taagaaacgt ccggaatacg acaggatcta cctttttaat
ttgctttcgt
638161 cgtctcaatg gtgtcagaaa acatttatat tgtgtcttaa ctgctgatgt
gtttgttttg
638221 tttcagtttt cttcctttaa tacacaaata acaatataac atcagattaa
aattatatat
638281 atttttggaa aaataaaaaa tgaccacaag catcaatgat ttccccatca
gacatgagtt
638341 catcatatga gagtggtaat cattttctgc tttttctcct aacgagcttc
tgaaaaaccc

638401 tgctattcat ccgtgcttgt gaaataaagc gaaagctgct catgtgacat
cacatctcca
638461 tcttatgact tcattttctg tgagaaggct tgcaatcagg gtctcaccct
gtttgtctgg
638521 ggtagcaaag ctatttctgt ccacttcata tctgacctaa ataaatcgct
gtgcggcgag
638581 cagcagagac tgattgattg actgctgggg tcctgctgtt ttggccctat
tgacagctcc
638641 atcagtgaca tctgctcaat tattctgtcc tctgggatct gtccaaaact
gtgtcaacag
638701 atcctgccca gtgctctgct gacagttatc gggttgcctc gccgagaatc
actcgatctg
638761 attcggaagg gaacttgacc ctccaactac aaactctcct cggctccacg
ctgacacttg
638821 agcacacgag ttgtcctgca gtttgctctg gcgctggcac cgccataacc
cgtccttttc
638881 tctgcagggc tccggtaaga cagggcctcc tgcgactcag ctaatgaggg
cgaagcagcc
638941 gggctcccac tctgctgtga agagagagaa gagactcagc acttcctctt
tccccctcag
639001 tgctaacagg gagctgcaga agctgcttgc gctggcaggt actcctcctt
ccgaaggtgc
639061 tctgactctc tttgatgcag ctcatctctt tgataactct gtctcctctc
ctgcgagata
639121 accattcctg gaactgaatg tttggcctgt ctggattaag ggagttgtat
taacaattca
639181 tagacctggt ggatcctcat aactgccttt ttaaccttga ggtcattgct
tgctgcataa
639241 tttatagaca agtagattgt ccggcagctt tatctgtagt gcagatgatg
tgcgatcagt
639301 tgcatctcca gccatcccag gaggggaaaa agacaggtct tacacaccac
agggctcaat
639361 catgcaggca tgtctgactc cggctggacc tgacagtgaa gatcctataa
cagcctaaat
639421 caaagatgag ccctggtgtg tagcactccg tgcatcttaa gagtcccaga
atacacttta
639481 gcctcagcag ctttgggatt tgagtacacg taataactat agctagctgc
tgcccacccg
639541 acagtgttct catcgatcgc ctcatgttag caccaatcac attggtgctg
ctattaagag
639601 tggaacagtg aggttcgagg cttgaacatg cacatcaaac tttcatggct
tctttcttta
639661 cacacagcat tacaggcagg agctggctta agtgggttcc tgtaaactat
accctgccgc
639721 actttgtttt catgccggga tgaacaacat tgctgagcaa tagcacagtg
ttcataataa
639781 caataatact aatgttcatt taaatcaaag tgtaaggatg catttagtcc
atttatttat
639841 ttggcattac gacccataca ccacagctaa gttactttat gcaagagcca
gaagagcatc
639901 gatgacagag cagatttatt atgtacgaga gaaaatgaat actgatttga
agaatccttt
639961 ggcaaacatt gtttaatgac ttaaatccat gatcctgtac cacatgcctg
ctccgagtgt

640021 atcactgccc cttactcgtg gtgtgtgcgt gcagcattta acacagctac ttacagagaa
640081 aactacacaa acatgcaaca atattctgag taacaaatac ttgtatctca ttcaagatga
640141 cgggaatcat ggaggttttg aaggctggat gcttacaagc ataagaaatc caacttctgc
640201 ttgtcaaatc tgacgaggac gcgccgtcaa ttatttaaaa ttatcgttgc tgccccctgc
640261 tggacacgcg cgtgaattaa tatagtattt tttccaaaaa ttaattagct aattttaaac
640321 tgtatttttt tttatagtgt aaccttctta tatttggaac tctcagtgac cctctctctg
640381 tgtgtgtaaa tgtataggta tatgtttata tataaaacaa gaagatatca tcagacgtta
640441 gccaaatgca cctgaagcct ccagtaaaat cagataatac caagttaaat atgtatcagg
640501 atgatctatc acaaccctca gctggtccag taggaagtca gtagcagctt ttagaaaata
640561 ccgccactcg ttgccatggt tgtggaatgt aatgacacct ctcaaagagg ctttttcccc
640621 tctgtgagaa aataccagta cacttgtctt tcattttgtt tctgctgcag aaaaaagggc
640681 accaatcaga aaagtcatta atgctgctaa aaacatatct tatatattaa acatatctct
640741 ggagcaggtg actttatgag tatgaaataa gccattaata atctgtcagt ggcagaataa
640801 ttaacaatac agacttttat gtaaagtggg aaaatgctgt ttaaccgcat tgttctaaaa
640861 acaaccctgg tgctctggtt gtggcttcca tgttttcacc acaggaagag gggggcgcct
640921 catcatgcaa agataaaatg cagattttgt atctatcgct gacaatctgg aaatattttc
640981 catgcagatt atttgactaa aaggactcta acgctatagt tcatgtctat catagaaaac
641041 aatgctatgt ttccatatgt ttgctttatt tcccatcctt catgagtact ctctaaactg
641101 tgagtaatgt ttgtggccac ttcttgtgtt ttaaaccttt attttgaatt tctttattgt
641161 gcatgactag atagcacatt tccatttcat ctgcagtgtc attatgaggg agctttgaaa
641221 tttcagtata aacttctcac cattccgtct tcagttcctc aaagctcctc cagctcattc
641281 atcccaagga tttgagaccc ccccccctgat cttctcctcc tcccctttga taagaaacca
641341 ccaatgtgag atgacaggaa gaaacaacat aatgagtgac ttcaaagatc cagccagtca
641401 gggcccgcct gcctgagcag gcattcggag aaaattctat ccacctgctt ttcctaagtc
641461 cgattatacc acagcccaat agttaaactg gaaatgattt aactcaatct catccaagat
641521 ctcaaggccg gccttccata gcagggacca gtgagcgtgg aggaggagtc gtgggggggt
641581 ggagagactc catcacatgt cccgcctcct ttcccgactc aacgaatcac atttgtcctt

641641 cccgggcaca acagccaatc acagcgcccc ttgctggtga cgtcactaac
tagccagttc
641701 cctccagcgg agcagggagc ttcattttct gatctgcttt tgtgctaaaa
tggaggctgg
641761 cctgtcgccc tgagtggatt cccttcctgg ttgaggttta gatgttgaca
tgcaataaaa
641821 cagacaagat ggttgtggat gcacccaatt ccaacgggcc atttcagccg
gtggctttaa
641881 tgcacttcag aggtacgtac ggccgaattg ttctccctgt gcgtcccagc
gacaaggagc
641941 gagagcggag ctgtgtgttc atctgctccc gttccccttc actgccaagc
gggtagtttg
642001 tcagcattct ttcggctatt tgtgcgcggg tttttggaga tagccacgtt
acattcgaat
642061 agcgaaggct gttgtgtatt gctgtgagat gcggacgcta cgtaaaaatc
tggttttact
642121 agttagctgt gctgcctgcc catcccccag ccaaccgagt gacgtcaaag
gaagtgatgt
642181 catagagaag ctgaggctct tttccttagt ttttctcgat attaatcgta
atatacgtcc
642241 ttggcaacat tgtgctccag taacactgca agacactgtc ataggatcat
tcggcgtcgt
642301 tgtatttccg tcggttcacg ctcctgttct gtgcacgaat tgatcccggc
gtggaggctg
642361 tagacgacca ctttgcagca aactgaccct ttctccattt ccccacccta
cccctccgtg
642421 caccgctgtc aattccgtgc tcgcacaggc tgacttgtct tggttgtgtt
ttccagcagc
642481 aacatgagcc ggtcgtgttc gttttattcc tccagagcat ggcgtccatg
tcttcaggtt
642541 acaggacagg gctcggcctc ataatgtctg ctcaatgtac cgtgtcatat
tggaaagcct
642601 ttggttgtga tcagcctgct ggagtggcat agatgaagcc ttgggcattt
attgataaca
642661 ctattgagcc acagaggtct ctggcctcgg gtcaaccagg ctctctttca
cagaccaatc
642721 ggttccacat tgtgtctgtg ctggttcccc atgcctcagc attaggaatt
agtgaatgct
642781 atggcgaagg gagaatacat ccaaatgcat tctagtttca tctcagctgg
atagttagtc
642841 ggggatccag gccagcatta ctttacacca acaaaatggc aacactcagt
agtaaggttt
642901 taaagttgta ctcagttccc ttatatattt tatcatttgg attccagatt
ccagaacatg
642961 aagccttaat aaaatagagg aggttaataa aaggccatgt tgtgagattt
acaggtcaag
643021 cgttacaata ctgtgaggct aattgaccat tttagcaaat gcacagggat
tttttaaatt
643081 aaataggatc catcagcttc tctgtcgggt acacgtgcac aacagttttc
tggcaagttc
643141 ccaacagtga gggtagacat cattttctta gccataaaca cccagtttct
gttagctgtt
643201 aattgcaaat acctaagttt gatatcaacc ccccctccc ttcttccctg
acaaggctgt

643261 gaatgatgtg tatgacatct ctggtgtgtg ttcctgcctc ctgtccagtg tctgctgggg
643321 cagcaacaag tcatcctgat taaaccagtc acacactagc acacaaaccc acaataataa
643381 aaaacagcaa cagaaggaaa tctgtaggta cccaccagca tcaaaggaac ctataccatt
643441 ccaattttag aaataataca ctttaatctt ttggacctgt tcagtttgag attatgtatg
643501 ttttgtgata aagagggggg tatgaataga gctggagtat tgccccagta tccatgcaag
643561 cctagatgtt aatgattaga taaaagaacc caagaactta aatcacaagg cagctttgta
643621 aatgcagctg caagtatgtc aatgaagtgt attcaccaaa aaaatctctg acttctgctt
643681 attctttatt gttcaaataa aaaaacctcg ttcaatggac gaatttatct ggctttgttt
643741 tgtatgcatg taaaatgcaa gactgcttag gacagttggt cagtgtttaa atcaacatca
643801 ggtcgttcaa tcttgttttc tgaactcccc aggtccatct tacgctgcca ccccgccgtt
643861 caaagaatgg tgtttcccaa agttggacaa ggtggcgaat gtcacttagg ttggggtcac
643921 gggaaacggt cagcgagaaa acttattcta tctttgggtt tgtggtggaa catgtcaact
643981 ttaggatcat ggcactttgt tttcatgtta cgcaacagcc tagtgggata ttttaggagt
644041 gtacacaaat gctataaggc aggccggcag gcatacttgt gcaacagtgg caccatgagt
644101 attacatagc cagaggtgga aggtatggta atacattcct cggtcctcat gttccagctg
644161 gcagcggcag tgttttgtgt cagaagctga gactgactgg ctctcccgtc cttgcacatg
644221 tgtccaataa ctgcagcact aaccatctga aaactgactc gtccttgtgt aaactcatta
644281 aaatctgggc aggagtgaag aggatcggtt atctgttatt tctcacatac agaaacattt
644341 ttttcttgca ttttgccaca taaacctcaa gccaaatgat tcccacgtgg tctgtacaga
644401 gacaacacac ggaacatttc cacttttatc cagcaccccc ccccccaagc agattgtagg
644461 aatatcatta cctaatgtgg ttttatttgt ctttggatca tactgtgtct ttagttgatc
644521 tttaacttct ttcctatcct cctaatttca gaaccttgtg ctaaatccat aaatcatcat
644581 ttgtcttggc atctgattat gtttatctct gtaattagca gtgttctgcc gcaggctact
644641 atacagtcat tgaagaggaa taggattcaa ccctatgcat acgctatgct tatatctgat
644701 ctaatctact gccgtcacgc ctcagtaaca gccttttata aataccacat caattattca
644761 cccgcactat ggctgatttt tatggttagt gtcacaactg acccatgagt ctaacgcgga
644821 gtattccgtc acaatgggtt tgagagttca aaaaagataa ttgtgtttgc gggaatgaca

644881 gccagcgaca gcaacagctg atttgctact gtcagacttg gtcagatgga
ttacatttgt
644941 gatcgctgtc acaaatgtaa cctcttcaac cagcccgtgt tccattcccc
tcgccatttt
645001 ctacataatt tgtgaaaata tcctgtctgc cacaaataag gagagccaac
tgacataaaa
645061 ttgggacgta taggaaatta gcttgttttg tggttgccta gagccagcct
ttctctttaa
645121 aacagccatc aagtggcagt gtaattgaac agttggggga catgtcgaat
agcaatgata
645181 ctagaaaact ccatcaaaga gagataaagc actttaattt tgtgcctcat
cattatggtg
645241 ttggcatgta ctgctatcaa gccaccacgt agaatgactt aatttagagt
tacaaaacca
645301 aacactgccg ctctttattt ggggcctttt tgctctttga cccacatcag
actttcgtcc
645361 tttgtccctg cagacgtggc ccctgcagag caggagaagc tcttcatcca
gaagcttcgg
645421 cagtgctgcg ttctgttcga cttcctgtcc gacccgctga gtgatctaaa
atggaaggaa
645481 gtgaagcggg cagcgctgag cgagatggtg gagtacatca cccacaacag
gaatgtcatc
645541 acggagccca tctacccaga agtggtgcac atggtcagtg tgagacgtcg
cctctgctgt
645601 gtaaacccaa aaatgagggt caaactagtg gcttcctgtt tgtgtcattt
gttttttgtc
645661 tagttctttt gcacaatggc agtttcagaa ttagatctat aatcttaagg
gttaactcag
645721 agacacacca atgtccttct tttcatgtat gtgctctgtg cgaaatccaa
aatgcataat
645781 ttaattcagt ttcagagatc tgaacccaga aagttcagtc ttcactctag
gctgaagttc
645841 agtgctgctc actttatcta cctctcagcc tgtcactaca aacaaagagc
cccagtgtat
645901 tagcaggcca cctgcaccat cccatgatct gtctggcagg aaacagtgac
cctcggggta
645961 gtcagttgac gcacttgcac tgttatagac ccagtgcccc tggaaggcag
ttagagaggc
646021 aaagattcta ttgatctaaa aataagaaag ttatttttaa atggaggaat
aaaaaaaaaa
646081 gtttggttga aaatgtttcc aggagtggca aactttattt tactttaaac
tttactttat
646141 tctgtgcaat ataacttgga ttttacaaaa aaataaatat atacatatta
ttattattat
646201 tattagaata ctacccagag gaattaactt tcctctactt ccagtttgca
gttaatatgt
646261 tcagaacttt gcctccgtcg tctaatccca ccggggcaga gtttgatcca
gaagaagatg
646321 aaccgacgct tgaagccgct tggccacatc tccaggttca gacttaatca
tttattcatc
646381 cgcaaaaccc aacataatca agctgcttgt atttattcct gcgtatgttg
tgtttgttag
646441 ctaccacaaa taaacccccg ttttggtgac gattacgtta aaatgattca
ctccaaataa

646501 aatctccatt gtgttttctt tggcagctcg tctatgaatt tttccttagg tttttagaat
646561 cacctgactt ccagcctaac atagcaaaga aatacatcga ccagaaattt gttttgcagg
646621 taagaaagga attcaactcg cagctctgtg gaatagcttt gaaaggtgct gacggtcact
646681 gttgagtaac atttgaacgc tggtgtattt cagcttctcg aactgtttga cagtgaagat
646741 cccagagaac gagacttcct taaaacaacc ctgcacagga tctatggaaa gttcctggga
646801 ctgagggcgt acatcagaaa acagatcaat aatattttct ataggtgagg acagttttcc
646861 ttcagcactc ttctcagcct gtgttctagc tgagaaggta acatcataaa tagtttttaa
646921 tgtaaaggct ggaacctgtg aaatagtact tgatgatatg cagtttcatc agtatcatgt
646981 cttcaaaatg gcaacgactg ggatgaaagt taagatgtcg aattggtata ttttttttat
647041 gttctttcag gtttatctat gaaacagagc accataatgg tatagctgaa ctactggaaa
647101 tactgggaag gtaaggccct gatttggtat gaggtgtgaa agtttctcag ctgtttattc
647161 taccgtcttt ctcttccaat tactcagcat aatcaatgga tttgccttac cactcaaaga
647221 agagcacaag attttcctgt tgaaggtttt attgccctgc acaaagttaa atcactcagt
647281 gtctaccatc cacaggtatg cctgatgctc acagacgcat ctttagaaac acatttccat
647341 atttttgtgc cgacttcatc ccgcagattt attcatatgt tcatcctgtt tctccagctg
647401 ctgttccctt tttttagttt cctcttcacg ttttaatatt cactgctcat gtgttttgca
647461 gctggcctac tgtgtggtgc agttcttgga aaaagacagc actctaactg agccggtatg
647521 taaataagga tttaatcatc gtcaccaaaa tgatctttgc tccatcaaag aaatatttta
647581 atgtcggccc acaggtggta atggctttac tgaagtattg gccaaagact cacagtccca
647641 aggaggtgat gttcctgaat gaactggagg agatcttgga tgtgattgag ccttcagagt
647701 ttgttaaagt gcaggagcct ctcttcaggc agctagccaa atgtgtgtcc agcccacatt
647761 ttcaggtagc caacagataa tgaaaacagt ctggttcttc aggtgtggta ggtaatgaat
647821 gtcagtacat tcctgactgc atggtttaaa ttcctgtagg tggcagagag agctctgtac
647881 tactggaaca acgagtacat catgagtctg atcagcgaca atgcagccaa aatcctgccc
647941 attatgttcc ccgcactcta tcgcaactcc aagactcact ggaacaagta aggcccctgc
648001 acaggttctg attgtcgcag catgtgacac cactccaaca ccggggcgat atctctcaag
648061 tccgtgttga tttttgagca aggtcacgaa atgcacgaca ccgcagcgca gttgcacata

648121 agtcaaacag gtttcgtcat tatggagcca acggtggttt atcttcaggt ccatgtttga
648181 ggtcctttct ctaaaatacc agatgtttca tttagtttca taccaatgct gttaaagaat
648241 gatgacttgt gcctgctgtg cgtgcaggac catccacggc ctcatctaca acgctctgaa
648301 gcttttcatg gagatgaatc agaagctgtt tgatgactgc acacagcagt tcagggctga
648361 gaaaaacaag taggtgccgc tctctttgtc catcccaaag attcgccgtt cagcctcggc
648421 gaggttttgg atttaatggt cacctttggt tcttttctgc agagagaagg caaagttaaa
648481 agaacgggag gaggcctgga tcaagatcga gaacctggcc aagtcaaatc cacaggtgag
648541 tgtcaagagt gcagcttcac accttttcct cctgtgcttt ggaacgtgtc atcatagcac
648601 tagtgtgtgt agaatgtact gaaatggatg gggtttttaa tttatggtgt tagctggtga
648661 atttcaattc ctttgtccca tcttatcaca cgtgcgtgca acttaggctc atttgttcat
648721 attgtcatta gtaaagagct taattttggt gattaaaaca gtcgacagta agcgtttatg
648781 ccgatgaaca cgaggtattt ttaaatggca ccgctgtgtt cagcgcatgg ttattggaaa
648841 gaacacacac acgtcgtttc tgatactgtg gtggtgtctt gtcatcaggt gagaggtctc
648901 ctttgcatgc aactgaaagt cttttctcca tccttttgtg tttgtttttc cctttgtttt
648961 caacatagtt ctctgtgtat gttgattcct ctgacctcaa tagtcctgta gcaatggaga
649021 cggatatccc tttgatagaa gatgttcaga ggttaaaaaa gacagttgag gagggcgcga
649081 ctcaggtaaa cctgactttg tctctgatgt ctctgagcct cactttactc gcatcgcagc
649141 ttttaatttg cgtttccaga cctgagctaa agaaagcgct gcagtcgctg aggaataaac
649201 ggtgtgtttg tggcatgtcg tttctgcatg gagccccaaa ctgttcagta ttttttggat
649261 atttattaat gcaaaaggtt ttctgtctgg ggggttttct tgccggcatt gcttgctcaa
649321 gggttgggtt ctgggtctct gcaaagcacc tgcagacaat cttgattgta acagatgcta
649381 cataaataaa gtaaattgaa acgaacgcta caggacgcag catgggtcaa aatatagagg
649441 tcagctctca aggctgagac aacatgattt gttttatcct ttaagaggat aacaatgtct
649501 ccgttctcga tacccatgct tttgagattg gtttgtttgg atatttaaat gtcacttatt
649561 ggcagcatta cggttgccgt ttaattcagt ttgacctgaa taaatgtttt tgttccagtt
649621 gtaaaaccta aagaaaaaac tccagtatat ctactgactg acttgaattg acaaaagata
649681 attttgttct ttggtacaat actggataat ttcggggttc tgtagattta gtgcatggcg

649741 tgcaatttaa cattattatc gttatttgac atgttgcaat acaggctgtg aattctgaat
649801 tcagtagtta tatttaatag aaaaagggct taattaatgt tattttattg aaaacatttg
649861 ctggtgatgt gagttttaag gataattctt ttttctgcac cctcaatcac tgacgtttag
649921 actgtgcttg tctcaggtga acgctaagct ttgctgcccc cttgtgggtt aaagtaatct
649981 tgatgaccgc catcatgctc tttacattgt acagacttgt aatggcaccc tttgaatgtc
650041 tgccatttat cttttccaca tccttgtatg tgtagcacat tcttttctgt ccagttgtat
650101 atgtgatgga attgtgcacg aggacattat gattgacttt gggcctcctc tcagttgcag
650161 catgaccagc ggaaagagcg gcctatgatg cgacgcaaat ctgagttgcc tcaggatatc
650221 tacaccacaa aagccttgga gtcccaccgc agagctgaag acatgttgac cactcacgat
650281 ggactctagg accctctacc tgccagcgtc tggttcagct gccccctcta ccatccacag
650341 ttatggacat ccattttgtc cacgtgtcag agtgacactg ataacccaca ctgggccttc
650401 catccatatt tcctaccatg agtttaccca caatcctcat ttgtccttca tccttgaagc
650461 ccgcctccca cagatttctg ctttctcagg tgttttactt gattttgcga gtgcgtcctc
650521 actacgttct gtgtgttctc ccgtttcttc ccacctcatt tcttcctctc ccgccccgtc
650581 accccgtgca ctcactgtca gtgtggaatc tgggagcatg gactcctcaa cactggttct
650641 ttcattccac cttggaattg tgttcttaag tgattggaca gtgtaggaaa cgattgactc
650701 tgaaggcaca tctgttggca tctgtttggg tttcttttct gtcgatggga tcaaatccag
650761 aacccgttgc tcagataaaa gaacggccct cacctcttgt ctgtcgtctt tttttttctt
650821 tttttttggc ccgatctatg gactcttgtc agtgccaaga ctgttgtgtt cactgtttta
650881 tgtttgtgcg agaatcgaac aggggaacct gccttttgtt tttcactgat ggttgagatt
650941 tctttatttt aggacaccag accaaactga ggcctgtcct tagccttgca atgtccaaaa
651001 aagatggcaa ggaaacgaag tgtcctgtgg acatctggcg ctttcttttt ttcgtgccat
651061 acactgttct ttatatgctt gcttccttat gtttatcagc aatatttaat aatcaggcca
651121 taatgatgcc tagctggttc aaacaatggt actcgctgct ttctagtcgt cagacaggaa
651181 actgtatgac tgtctggtgg ctaatttctc catttaaaaa ggatggagtg gttatatccc
651241 cattactgtc ccatgactgg gatgtaaaat cgtgccctgt caggtgtcgt gtgtttttca
651301 tcccgtatct gcagtagcaa cacaaaactt ggctgtaaca aatggccggt tgagattcgt

651361 tgagcagctt cacaaatata tgctgtatta cagagggagg ttgtttataa agcatcatta
651421 ctgtaagatg tgttttagtt gagttagtaa gatgatgcag acttcggtca ttgtttttct
651481 ttttttcttt ctttctttta acgctgcact tagccgaggc ggcacgatta aaggttttaa
651541 tgtctgtaac taatcctgga acataattta ttaacacata agctaatcat acacagacat
651601 tacagcaaat gatcaacaga tttgtttaag agctgacaac accacgcaaa taagcaatcg
651661 ggtgtttttc tgttatgcac tcttccctcg gtcaaattag ctcaggcagt accattgttt
651721 aaaccaagac agtctgcagg agttcttgat ttgaatcgtc gtatttcttt gtcttgtctc
651781 ttgaggtcgg cagtgcggat gtcactcttt tttcctcgtc cttctggcct tccacggcca
651841 caaaaaaaaa agcccagtgc tgaaaataaa cattgtgact tggcttttta actgtgttaa
651901 tactgtattt gcttgaatgc aaaatgatgt aaattaaaac taccatgtta aacatgtacc
651961 tttaccctgg aggaatgcca ttcaccaacc tgctgcggag gtcatcgcgt ttaggaaaca
652021 accccgcctg ccgggagagg cgtttattgg attttacgag tcgagtgaag gcagaggttt
652081 tattttagcg ctgaaaataa gtcaccttcg atgatgagac catttaaact aggtggcttt
652141 tgtccgtttc tgcaggagct tcgctgaact tttaatttta acatattatt tccagaacat
652201 aacttaaagt gtccttttgt atgtgacctg tactatatga ttcatttaat attgtatata
652261 tgatttacct tgtttcatgt agtgttatat atctagatta attttgtaca aactgtcctg
652321 tacagaataa accgtcaata gcaagtgaaa gaagtcagac tggcacatac cgtcttattt
652381 tcttctgttt ttggatgtac tttgagccgt caaggattta gaaaatgttt ttatttttat
652441 tataagattt gcaaagttct ctgcctcctc tatataacta aaacatcttc atgcccgttg
652501 tgtgctcctt tcactgtctt tagtggtgta taactatata atgaataaag agtttatttg
652561 aatatatatt ttcaatcatg catcaatttt ggttttactt gatcagcatc cttctatgaa
652621 tatatatata tatctatgac tgctctagat tgttttttat gttaaacatg ttatgtttac
652681 tatggtatct aaataacatt ttctaatagc cttagttggg tatacgcgct gctcgcacac
652741 taggggggcgc caaacatcct tcggtttctc cagcacattt taatacgatc taaaaacacc
652801 caggttcaag caaacagata tgatgcagct aaagttatag tttctttcac gttttaaact
652861 ggcttttaaa ttgacacact aatattgtta attatgttgt aaaagcaaca tcatttcgag
652921 tttatttagt atttctgttt agacaaactg gtgcagtgct gaaatgaact ggatagtgtg

652981 aggtataaat attttatgtg accaccgttt aagctttaat aacagtttga tgcatacacc
653041 tattcaattt agtaacaata aataatgttt atagtttaca atctagagct gacgggactg
653101 atgcattcct cctcagcagt tgcagctttg attaaaaaat aagttgtttg ctgttaaaat
653161 attcagaagt gacgtcaaat ttaccgcctc ttcattgcga aacgcacctc acgtaccccc
653221 tgtaataccc cttaagccat atcacttcaa tcagcagagc agattacatt ttagggagtg
653281 acggtagtgt aaaatcagcc aggttcctct cagaaaagga gggaaaacct gacgcgaact
653341 ttcccaatgc aacacgtgtt ttatggatta cttcctgtta gtgtaatgag agtgttgaga
653401 tgatgcttat gaccttgcac ctgttggttt gtagtagctt atctggtgct cttgtgagag
653461 tattaaatca tgaagtcaca caaagtgaag atcaagcctg gagtggcagg attggtcagt
653521 ggagagcgtg ctggtaatgg agcggccata ttttggggaa atcaggcaat ttggcactaa
653581 tttcttgaaa tgaacccggg ctgcacagct gcactgaaga atgagggttg catctgcttc
653641 cactggttgt tatttttata ataatgcttg aaaacattgt ttgatttaac tcagagttaa
653701 ttacctataa catcacactt gttttttttt ttttttatgg tttctataat ttgtttttat
653761 ttctgcgtga agagtaatta tgaagacaca gtccttttaa gtggccttat gtcattatgt
653821 aatcaaattt atggtcctga ttaatgaata aactaagaca cagcacataa tatggagagt
653881 ttttacatgt attgtttcaa ttacagtttt atatgaacat tatgatgaaa taaaataaca
653941 tgtcagtaat tatttgcatg tgtgtataaa aaccacatgc aaataagtgg agtttcttta
654001 tttatgaaag aataactatc aaatgaataa aggtttatag agtgctgttt gttttacaag
654061 tagtgatcat ttaatattgg taacagtgag attggcagca aatagttcta cagggctgca
654121 agggagccaa acaactgact gaagatgtgc ttctgaaaga gcttcaaaga attccacatt
654181 acatttcaca gaatccttga aacccaacac cagcctactg gttcaaaagc tgcacaccat
654241 cgctccctgg ctcgcgtatc actgtgcacc gttgccaagt tgtcctcatt cggcctacct
654301 gactcagagc gggctctctg ccaaggctga cagactgctg gaaggcagac ctgcttccat
654361 ctggaacatc acaggctcca aggtacagcc acagcccgct cctgtcacag tcaccctcct
654421 gtttcattga aaatcactgg gttctgtagg tgttagcatg gccacacagc cacacctgaa
654481 acctgtaaaa ctggccataa ctacaggatt tgtctatatt ggtttattca taactcagca
654541 gttaaaggtg ctcatacata gaaatgttaa atgtcttctt tacactctgc ccaaagttta

654601 aacaaagcag cactcatgcc tatgctaact gggtaccagt ctgatctgca acataaaccg
654661 gcatcggtac ataaacgcaa cactatcatt aaactaagac attttctggt tattaaagtt
654721 ttcttttttg ttagtttttg tgtttttgtg ggcttcctca aaaccatgcg ctttttgtac
654781 aaaatatcag cacagacttt aatgggactg attgttgtgg cacccccagg aatcacctga
654841 tggagattta aaaaataaag atatacaata agttacattt ccgctgttta tttattttat
654901 taattccagc tgtaacgaag gaactttgaa gaataactct aaccctgatg tagtactgca
654961 ttgatctcgt gggggcgctg tttccccaca gagactgcac cggcagactg gactgatgga
655021 agacgtgatc tgggagaggg cagatgaggt aagtgtaggg gggggcagcc gcagtctaat
655081 gcctcacaac agccccagca gggtaatcta atcaactagt ttagagagtc ttcttggcct
655141 ctccatccct cctcaaacga gaggggatcg tttcttcttt tcaagggttt ttccgcgaac
655201 caccgtcttt aatttgagtt gcaggaggac agggttgggt gggatgtgtg gcagaatctc
655261 gacacatttc acgcttgttt ttggaagaaa caacactttg taaaatgggt tagcgtatac
655321 ctgtatgcat tactgtagtc ctgttttaat gggcgtggga tctcatgcac ataatggctt
655381 aaatgaaact aagaacaaga gatttaaaac aaagcacatc cacttatttt ttgagaggtt
655441 tagtccgaat aaattgcacc aataattact gtatttggtt tgaaggatga atggcattgt
655501 ttatgatatt tattaaatca gataaaacat tatatatcct ttaattacag attaaatccc
655561 aggatcagac gggatcagat ggtgtttgct gtggacagac aatgctctta cctgcttgtg
655621 actggtttga ggtgacattt ttctttacag atcaattttt taaattctct tttaattcta
655681 aaactaggat aaaaacttcc aggataaaaa cttaaggtca aactataatc cacgattggc
655741 aaatgctttg gcccagaatg taaacattaa aatcattaaa aggcagccta agggatgttt
655801 ttaagactga tattcaatta gattatttta gtctttcttt ttttgaaagt tatgaattca
655861 tttaaaatga ctttcctcat tttttatgga ttgtgattat atttatttct tcatttatag
655921 catgtttatt ttcttttctg catcactgtc ttaccctctg atcagtgcta aataacaaaa
655981 aactcacctt ttaattcaga ctaaattaat taatgatcta gttttgtgta aaatgagaaa
656041 cttttagaaa tatcttaaac ctttgcatcc aaactatgag gaaattattc atttgaattc
656101 agtcaaatct ccatatttaa taggaattct gttaatatct gttcttttaa tatctaatat
656161 ccaaatatct tatggaaata atgttcatac ccaccagaca catgcccact ctgtccatgt

656221 ggaccgtccc atcattctga tgcaagatga cctaaaatct gctcatgttt aaactgtcag
656281 aagattctgg tatttgtgaa gtaggtgaaa aggtgtctct gggttctctg acaacagatg
656341 catcactgaa gccatatgga agattctaca ttatctccaa atggagaaac aaccttagca
656401 acagtccatg aaccctacca aacagacatg gtgcgttcgc ttgcccccgt taaaggcagt
656461 tttcataaca tcaaaggatg caggacaaaa cccaaaccag gcacaggctg acattccttt
656521 taatatttaa tttatatgag actattctaa aacattctac agtaaaatga atgtgcatta
656581 atacttgatc ctaattccat gaatgtataa cttcaattgt tatcgttttt ttaaggattt
656641 cattttagaa tatagttaac agggtaatga acatttaaac agcaagcaaa acaaaatatc
656701 caggacaaac tacagattaa agtacttgag atcaagctga agcataaaag tgataattct
656761 aatatgttat ttctctttct gactttaatc tggatagaaa gaacactgag cagacgtgaa
656821 tgcaatgaca tcaaaacctt aagtacttaa acgatataag aacaaaagtc ttacccacgt
656881 aacaataatc cctccttaag aactagctta gcagaagtat tcaaataatc aattttcttt
656941 ttcccccgat atgaagcaag tttgaccaca tgactcattt tctaatgacc ttctgtatta
657001 gcggggcact gtgtcacaat ctaacatgat tggttctaag cccagtgacc tgaagattca
657061 gctccatctc agttaccttg gtgatcgtac gagtcatgaa ggctgccaac gtctgacata
657121 atctacatgc ccagtaaatc tgtcattatt tactgcatca tttgtgctgc gctgtgcctg
657181 aaatggtttt taacttattt tgactggctg cgattcttgg cttttattgt gttaaaggtt
657241 aactgggaac aataacatta cagtcagcta tgggaataat aatatatatt ttttttaaat
657301 attgtttatg aaaaggatgg atgagcattc aaaaaacttg aaattaagcc actgaacttg
657361 ttttcctcta tctgatgatt ccagttggtc accagcagac ttcaccttca atttcatcca
657421 gtttctcttg ttgtcaaaat gacaataagg tcgcacgtct gtgccaaatt gcacattccc
657481 tgacccctcg tctttcccat ttatccctgt gagtgacacc cagaggtgaa atgagtgcag
657541 agaagtgtag ggggccctgg cattgtggtg gctgagctta ggtaggattt aaacactact
657601 gcttaagggc tcttaacctt actgaccctt ttgggactat tacaaatcct gttgtgccag
657661 agaattacat taagcccttg ctttgattcc agccctggtg agcacatgga ccgaagcctc
657721 tgccgtaggt cctccagcat tcatctgtgg tgccaagggc gcacaggtgg aatcacgcag
657781 gttgtggact catactacac cgtgatttct tgtggtgggg gtaaattatt ttcaaagact

657841 catcaaatag aatcacatgt tggattgaaa cttgggtcac tgctaagtac tccgcctggt
657901 tgcatgtcgt tccctctggc aggtcagccg ttaagactct gcctgcccag tgatgcccaa
657961 tgggcagctg gcatttccag gaaaaggaac tggagacgag tcagtacacc cttattttta
658021 gctaagcaac tacctgcaaa gtagctgtga tctttttac atcacctcaa taagaaagaa
658081 gtgatcacag tgttacacag tgagatttat aaaatgaata aacgaataaa aaagcatgca
658141 ggggctagca aattagcttg gattagacat gataaaccca ttgttgtcaa atgtggggac
658201 caaatctttc ttacattaat tattgcaata tcaacaattt taccaagaag tggtatcaga
658261 agtagaggct tcagtttgtt gatatggaaa atattgcagg aggatgaagg aatctgcacc
658321 cccccccccc caaaaaaaaa cacagagccc cgctgagcca atttgaggca atctgttgtc
658381 cattcaaatg aaagcctttg gcttgtgttg aagacgtggg aaaagacttt gttttaattt
658441 ccacaggcgc atctgcatat tttccaaact tggaaatcaa atagatttaa aatctacccc
658501 tttccatcaa ttcaatttca attcaattac aaaaataagt tgccattttg cagttatgta
658561 cctcctctaa aagtaagcac attttcactg attaggtagc gtagctttac tgtgccttct
658621 tttaatcatt tcattaaccg cgcgtctgcc tgcgtgtaat tacagcctgt ataacctgaa
658681 gagagcctga gtgacgtcgc tcactggcct caatagctct accaccatct tgttttagca
658741 gtgttgctac attaatgttt tatcacttct atacatttat ggatgtgcga aagagagtgg
658801 gaatttttg agccggacca caattacatt tatttttggg ccaaagcagg ctcacgtttg
658861 cactgaacaa tcctccagca cattctgcag caaagattcg gcatcacaac gctgcttggc
658921 cccaaatccc ctctgtgtgg aaggaagaga agcctaaaaa ctgcccacta gctattgact
658981 gagataattg gtagtttgtg taaagagttt tgcacttcag aaatgtctct aacttctggg
659041 accttgtcca taacgcttcc ttagacaaaa gctgcggcct gtaatgacct ttttaagcat
659101 tgctatccca cagcgatgac tcatgacgaa caagtttctc agtcagagga ataaataccc
659161 aaaagagtga aaggtctaaa aatgaattgg cttcacagct cccgctcggc tcagttggta
659221 ccatccagct tctgttatgg agtctagaca tctattctcc ggagggctga ccagagggcc
659281 gcttcttgaa tacttctcaa acgcagcaca gtgcacctcc tgccatcaac agcactgaat
659341 agagctgcca cagtcaaccg ggcagaatct gggtgtcact gtcagtcccc cctctcctct
659401 gcccattgga ctgcatcaca tgccaccgct tgtgacagcc caggcagaag gaatgagccg

659461 tatctgatgt gatgttttct cccacgcctg cctcttggac cgcagcgagt
tttcttcttc
659521 aaatgctttt atgctggtta ataccaatta ggtgtataag tactggagta
agcatgatga
659581 atgaccctgc agatatacat ttatatacag tatacttgta cacacacgca
tgtattaatt
659641 ggacttttca tgtctggctt ttttgtttta tatcttatat gtaattaaaa
cattgatggt
659701 gctgatatgt agatttgcaa attgaggctt cacacagtcg tagtaaaagg
gatatttatg
659761 gggaccctac catagcttca ttgctcccca aatgtaggtt taaccacaaa
ctcatgggaa
659821 catatgatga gaagcaaagc tacggacgtc tctgagacat cgttgtctga
gatcgtaatt
659881 aaccttcctg gttttagaag ttgtatgtga gaaggaccca aaaaaaaagg
tcccgatcgt
659941 gtatgctgtg ttaaagagct cgcgcagcag actttggtgg ttatagtcga
gctccggtga
660001 gtcacggtct ggtcattctt tgactcttcc agtaagagtc ccaggtctcc
ggtctctact
660061 ggaaatggat gacttcttcg tgattggaag ctgagacgtg actaaggcat
ggcaaatcac
660121 taaagctttc ttattttcaa attcgcctcc agtgtgtccc ggtgaactgc
catgattcat
660181 aaagcagctg aaaaacccac atgcaacacg tgtccaagga ctcccccgcc
cccccccccc
660241 ctcacaaatg accacgtgca taaggtgaaa taaggaaagc aaccacgggc
tacgctccag
660301 ctccctactg gttatttaga ggggaggatt tcaggctgat gttataaaga
cctccaccac
660361 ccccctccct tttttttttt tttttagttt gaaagctgtt tttggttaac
agaccccccgt
660421 attcttcaga gagcccccat agaaagaggg cctcactgca tagctgaagg
aaactgaaga
660481 gaggaggatg agctatacta ctccgaaacc ttggacatac ttgagcactt
ccagtcaacg
660541 ctgagctttt ctcagcacat gaatttacag ttcaataaag caaagctaaa
tgatacgagg
660601 gaggcgtttg gagacacgcc ggtaaacaaa cacgctgccc ctcacacatg
cggatcacgt
660661 cccgtaggac cccagcacat atgttctgct tctcattatg ctatcttaca
tggttctcat
660721 tatgtttaag acgtccttcc ttgagcccta tggactttat ccatttgtgt
gtcaacagaa
660781 ttaccacgga ggagactttc tgtcttagat gggcgcttgg tctttcttta
gacttttgtt
660841 ctgctgaaag gctccacatg tgatccttcc cacaggcctc ttatggtatc
catatgctca
660901 cacaatacag gcaggaaagt tctgaacatg aaataaatgc ttatttcata
tacccagatg
660961 aaactggacc atctgttttc atgttgaaga tttgatacat tttgttctcc
ctccttcgtc
661021 ttcacaatag accgtggtta atttcaaggc agcaagtgta cgagcctgtc
ctcgcctgaa

661081 ctttcgggtt gatgcacagc ttaatccagc gccgctgcga agcttccctc tccttctctc
661141 catctccctc tcctcggcgt tagctcgctc tgctccgccg cctcccgtct gctcacagta
661201 tgtcggtgcg taagatctga gatacagccg tgtcgggttt ggactcgcat cagctccgaa
661261 tatgtgagag gaatgtcgtt cggaatgcac agcggaggaa gttggggcga agctgcagag
661321 ggaaccgcgg aaggatgtag tcaaagaggt gcacgcacac gctgacccac actaatgttg
661381 cataaacagc agagggaagc tatctggtca cctcgcctgt aatgaaaagt gcaccgatga
661441 catggatgta aaaaggggct ttatttccgt ggcaactggt gatacggtcg gagcagggaa
661501 actgaaaatg ctgcattgcg tcatagagtt acatcccagt caggtgcacg tccatggatg
661561 catctctcaa atccaaaata aggatgaaca acctgctcaa gatgtgaata ctgcatttaa
661621 aagtgttaca ctgggggctc ccagttcaaa tacaccacag tttccgtcac ctgtcctgaa
661681 aacatgtccc ataatacgcc ttcgttgtcc tggtgtggtt aggaacaacc ttaaaatcac
661741 acttctcttt tcaaaagatc tacaatataa accgaaagtt cagcctcagt tgactggttt
661801 tcacaatatc atcggcgttg caaacacctt cgctttgttc cagatggccg tcacctgaca
661861 agtgcagctc gagggttcca gagtgatgcc caacatcaca acagtatcag aaccactggc
661921 catcgggtca aagggtttaa gtgagggaga gaggcatggg ttccaaaggt tttaggccaa
661981 gtgagaaaat gagaatgaaa aatggaattc ctggtttgtt tagtaacctg gcatgtgatt
662041 gtttcttcta ttttcttcat gacactaatg ggatacagct aaagtgttcc cattgttgtc
662101 acacgtatga ctagcccggg gggtggtagg gcctgatcag acacgtaaag gcattgttga
662161 agctagcgat acctgccgat atgtatgtgt gtgtgtgtgt gtgtgtgtgt gcgtgtgtgt
662221 gtgcgtgtgt gtgtctgtaa atactgggta agcgaataaa ttgatcctgg gccccacatc
662281 ctgaagggac tgtttgaggg taaagacttt gggtttggat tatgaggtgg gtcatgcatt
662341 ttgtctgaat ggccttacaa atatggaagt tcaagcctga gtgtgcgtga cctgttccca
662401 tgtagaggct aacaacaaca acaacaaata ataggtcaat gcggatgtac agagcagtct
662461 attgagcttg catcttgcta gctctaacag attggctggt acgtaggcag gtgtttactg
662521 ttaaaaaaaa aataaactct cacctccccc acaaacacat taaatatgga acctcaaatt
662581 gaaagtagac ttgtgctgag tccttatgta agagcctgac agattgtgat actgctgaag
662641 gagaagtttc tcagttgtgt gtaggaggca gagggcacca aaaaaacagc ttttaccaga

662701 ctaaaactca tgtttggaaa gctttttttt ttaaccccta gctcttcttc tcctaagaca
662761 aactgctgtt ggagaggtca agctacaaag ataattggtc atatgtttaa taatttctgg
662821 gaagtagtga aggttaaagt atgcaaggat ggcggggcag gtttaaaagc attaagtgcc
662881 attaatcttg caaacagttg aacgttcctt ttttgttgtt gaccttcctc tttgctttta
662941 ccataaagga acgggtagga agagtgcttg gaaaaattgt gaggacggtt gtgcaactga
663001 aggccacatg tgccaccagg caaacaagat aatcaagtca ttattaacgg gataagggtt
663061 aagatgcaat catgaattga tcatttaaat tttctagctg caaacatgtg ccatagtaaa
663121 aacactaaat agttcccctg aaaaaaagca ttcaagcccg tagtttgact gtttccaact
663181 acagtttcaa actgaaaaca tatttcttcc atttaaagcc attaaaacat cattaaagaa
663241 cgtctacgtt tgactgatat accgtctcaa ccaggtttcg cactgccaac attattcgac
663301 tgtaaagaat atttgaaata tccaggtgcc ctcaaacgtc agggactttg tcatcagaga
663361 taagtgtgtg tgtgtgtgtg tgttgggggg gggtggctgt tcatacactt tctatattgc
663421 ttcggtttca taaaaatgaa ctgagctgaa ctctgctcac tctgaattta ttcaggaggc
663481 gttcagttca gttctgttgg ctactggact tcttgccctg ctggagggtt ctcggacttg
663541 aggatttgaa tctatagtga ttgaatcata cctctggaat gcaaagcttt gtcagggcga
663601 taaagatgtg ttccgaattt gcttttgttg tgtctatttg catatgtttt cttaagatgc
663661 agagtatggg gctcctttgg ccaccgttca caggcctaat taaatgtgga tggtgatact
663721 ttcttaagag gcctgtgcta attttccatg ggttttgtca gcacttttgg cttactctct
663781 tcctgcccac acaagactct ttgctagttt ctttcttagt tatctcaaag tcttggctaa
663841 acctgggtcc tctcaacctc ctttttattg ccctagagtc cttatccaaa acaccttgcc
663901 cttgccttac tggcagttta cagttttcac agaacgtgtc ccaatgtttc ccagggcctt
663961 aaaccttatt gctctcatct aatttatttt gtatgaagcg gcccatcgat tcttgaggtt
664021 caccttgaag tgagctgtaa aaccaactaa tatagtaaat aggagattta ttgtttgtta
664081 aacagcatct atattcttga aaaatacttt tgcaatcttt taggtttttg taatgtttct
664141 tcaatcttta aggttttgta atgtttcttg ttgaagaaat agtttgcctt ttttaacttt
664201 ttgtgtttta tcttgttttg gaaattgtca cgtcgcattt tctcatttaa tcagactata
664261 atctcaaaat cccggtgcat tcaggcttcc cgcgccttta aaccatctca accattctgt

664321 tcagctggat ccaaaagcaa acgggaaccc ttgacgcact gggcttgaac atcacgctct
664381 gcaacctgat gctggtctga ctgagaagcc acgaacagac cgcagactta ttggccagtg
664441 gttcagttta ttcagggctg gccttttatc tgctggatca tttttgcatg tgttttaaag
664501 tcagactgta ggtattttg catggatgat gaatgtgtgt catggcgtgc aggtagctca
664561 ggggtcggtt ctgatgacaa cagaaatgac attaagctca aagaatttta aaaggaagaa
664621 atgaggtgaa acttggtggt aaaatctacc aaccagcatt cagccaagct tagtttttct
664681 atcctgcctt ccttttaagt cctcacccat gcccctcttt gactgcagat ttctcctgat
664741 gctcggattc caaaatatga cacaaagtgt cctcagaaaa ccggaacacc aaaaaaaccc
664801 actaaatgag gcgcacaccc tctcccttta tgccgctcct ggagttgtca ctatcttttg
664861 cctcttgtag ttgctctaag ttaatctctc cttccctctc gtgctctctg ctcggaaacg
664921 ggacctgaaa tgaccagcac acgttcccac tggggggctc agactgattc atgtgggagc
664981 agctttgcat ttcttgtctc agcagatgaa aggggaaata gagtaaactg atggcagaga
665041 ccaggctgca tttctgctcc tttgaaaggg gggtgtgtga tgccaggtct cactaggggg
665101 tctcacaaag ccaaggcaat aatgaagaga agaggaggtg tgtagcgtgg acttgtgaga
665161 gagagagaga gagagctggg ggatataagg caaacaaggt aaacaagccg gaggaaaaca
665221 cgagcaacgg tggatcatgg caaacggtaa tgctcaacaa gtactgtgga tttttttttt
665281 ctttttatac aatagagata acacattttc ttttaagtgc ctcatagcac attcacccaa
665341 tttatgatgg gtcactggac atcgacttca gtatgctaga ccgaatagaa gagtccaaat
665401 gtttgaaaat atttatggtc tagtaccact ttaaaagcca aaccattgtg cagccgctgt
665461 taaaacatta aaatatttga caggatcata tttatcatcg tcagtggata aataccgtac
665521 atttgattta cagtctaatc ttttatcttc atcttttctt aaatttaaac aattctctgt
665581 tgactgtata ttctgttcaa ggctggtctg atacaactga tgcagactgg tattgagttt
665641 tattacagac aggtggattc agtttatacc cccccccccc cccccccaca cacacacacg
665701 tcatattaac atttaaaatg attgtgggtt ttttgttgtc atgcattcat tgcagcaata
665761 attcacatct gcaagtctgc aattttaatc cctattgact gtcagattta attgctcatt
665821 attatttgat ggttttattt tcgggcaatc tttgagccca ttactcaacg atttataaac
665881 cgctccaaag caaatccggc attaatcatc tacatttcag gctgtcagta agttagcctc

665941 ctctgccccc ggcttccctg aacagctcag ccttcagtgt tttgcttcac cttcctgtcc
666001 agcatcacaa atgctgccag ttgatgttag tcccaccgct ttagcggtat cctgcagaga
666061 catctcagga cagaaaacac atcctattaa acactgttga gtacacaaaa accttatgag
666121 gacaagagtg ctttgattct cctccttggg atttagcttt aggtaaacaa acactgtaaa
666181 catcacatta acctttagta cccctccaat atttacacca gaattgctaa atctggtgtg
666241 ttgtggtggg ggttcacaaa tgaaacaaga gagctgctga gagatgttcc cacgctgctc
666301 tttaaatagt cttattgata tatctcatgt attgtgccag gatatcttta attaacattc
666361 atagcaccac aggacattga attcaaactt ctccagggtg gacgagcacc aacgagcctc
666421 agaaatatca cgaaggcagc tgtggatgca ggtccttgca gggggacata tgtgtgtctg
666481 tgtgtcaaag gttttcacac tcatcgtgtc ttgccaccat ctgtcccagt actggcatca
666541 atgagcatac agacgggcac tcactaataa gctcaaacag gcagcagtgg gggggggggt
666601 cctgtgagga cattggctga catgctcacc attatcatca ctcactgagt gcaaataagg
666661 ccagagacaa ctggagatgc cagcagaccc cgaagaaaat ctctctgaga tcttgttact
666721 gtttggggaa gggggggtgg ggggcgactg gaaaggaaaa catatttaga gaactgcagt
666781 gtgggggga aaggggtggt ggaatcatga gtaagtggca tgttgcaaag attggaattt
666841 ttcagggcac ttttgcctcc gttgtgtgtg tgtatgtgtg tgtgtgtgtg tgtgtgtgtg
666901 cgcgtgcatg cgtgtgtgtg tgtgtgtgaa aaaggttcaa acagcaacag cagcagaggc
666961 aaaacctaaa agatggagga gaagaaacag actcggctct gaggacaaaa taaactgcat
667021 gaaaatccac catctgcaaa caatctgatt ccacgctgga ggctgtgcca tcaatcaaac
667081 acacacacac gtttgttcta gttgtctcat agcgagaaag ttctgggttc acttccagac
667141 tctttctaag gtgagtttac atgctgtccc cgtgcctgtg agggtcctct ccaggtaaac
667201 cagtttcctc ctacctcagc ttgaccggag gctaattaaa tgtgcgcttg gccagtttcc
667261 actcataaat acattcaaca tatattcctt aatctgatgg cacaaatctt ccttaaatgg
667321 tgcagtaact ttaaaataaa ccacacattt tgtctcgcgg cccaattttc ccattcaggc
667381 atgcacactc ccagtgcagc acgctccctg gtgttcgctc atgtgaccgc tgcacattcc
667441 agaatgaacc caccatttgg atggatggat ggatggatgg atggatggat ggatggatgg
667501 atggatggat ggatggatgg atggatggat ggtgcccaaa gctcctaaca ttgccttcaa

667561 tgaaattcat gcttgctcct aacatccacc caccccccgg cggtctgcac caccacgaca
667621 gacgcgccct cctcctctgt ttctcctttg catcagctgt ctgacctctg tatacgtaca
667681 tatttctaaa tggagcttgt cgtaacacat gatctcattc cgacttgtct ttgaatcagc
667741 agtcgatgag atagaagcca cacacagagc tggcgacggc agctcattca agttgagagt
667801 gtttttctcc tttcaaacgg ggtcacaaag gtgaagcaga gaagagggag gacactattt
667861 cttggccttg tttctatgca gcgcgaggag agagccgtcg aagaactgaa tgtgaatgaa
667921 gccgcgttaa cattcatcat aaatccgttt tgtggttccc cctatgatat ttcatccctc
667981 gtggtttctg gaagagcgcc acctcaataa gtaagcagct gaaaaaggac tgttttccca
668041 gagctgccgt gtcctcagat gaacccgcag ccgctgccag gattccagta aagtttaatg
668101 gaggaggaaa tattcaggtt tgaaaacatc acagaaggag accctcaccc cttcttatgc
668161 aaagcatttc tgttttcctt cggttcggat tgtagagcgt taacgtttca gaggaaggag
668221 gggagaggat ggagagagac tgcttcaaag tatctgaagg acaaaaggat aacaaactca
668281 ttaaaatgtc tccctccaga aaaaaaaaaa aaaaagccag agagagggtg gcagaatgag
668341 gactgatgtg tcagctggtg aactcatcct cctgcctccg gatgtaaatg agctcacgac
668401 acaacagcca caacacggca gcgggaattt accccggcca aagtcacaca ttaggacgcc
668461 gttgggctgc acagagggct ttttatcaca cagaagctgc agtaatacac ctacttatta
668521 atggaaattg gtccactttg gtgtgcactc cataaaaatc tgagtgtacg gcaactgaaa
668581 ccaggttgct ttccagcgag ggcttcaaca catttacaca tggcttttgc caccagctat
668641 attaaacact atagacacac cgttcgactc caggtcgtct gcgattagga ctgagttagc
668701 gtaacgtgtg ctcatcagac ctggggtgtt tgtattgaac gagcacacca gagcaacttt
668761 ttgatgcacg tgtctatgtg gtgactccct gcagagggat ggacagatgc aggatgatca
668821 gcagcggtac taagggctcc ctgtctctac actgcaaaaa gactgtatgc aatcatattc
668881 tgtgtatttt aggatgaagt aatctgttga ttttgatcac agaaactgta tgagatccaa
668941 cggattttat ttttggacct ctcttgtttc catcctgacc aaaaagttaa aacaaaaaag
669001 acttcaaagt tgataaaacc agtttatcag cctgcagcaa cttgtggtag atttgtttta
669061 tctcaccaga atttcagcgc aattgtatga ggagagttta gccactgaga atatcaatgt
669121 atcgttagca atgatgagac gctgtaaacc ttttgcttct gaaaaattga tcaatacagc

669181 tgtgggcgaa gggggttttc ttgaggataa aaggacgacc gcaaaccttc
tgaaataatc
669241 cttcaaaagt gaacaacaat atctagggaa ctatatcata ctgtatgaca
taaaactacc
669301 accgatctgt aaaaacaatg ctcctgtaca atgttttata gtgtgtttgc
tatggctgat
669361 gtcagaagtt gttatttatt attctgtaca gagacagttc tgtaaccatg
gagattattt
669421 actttgcagc aaatgttctg tgtaatcccc aaattgccca agtttcacaa
tacaccgcca
669481 caacactgcg gtttcctgca aaaaaattca acaatacacc tcctctagct
tgatttatct
669541 gcttctgata tattgtcgtt tctataatca tcatctgcct gcatgtcttt
gtgctgctga
669601 agcaaaaaga atgctgacag cctgaataca gtaggagctg ctatcatcca
gtttcttgcc
669661 ttcagctctt ttccctgttg cgtacacgcg ccttccttgt aaagtcttcc
ttgtttccgt
669721 gggtggatgg attctcaccg atgttgtcct tcggctccct ggtgagctct
gcgctttcga
669781 aagggttttc aaaagtgtct tgcagtcacc aaagctcgat atttctcaaa
ggttttgctg
669841 cctgtggaat gttctggtac cttcaagtca gagcaggtgg ctgagaaaga
tccacaacta
669901 cagtgggaaa ggtgtcgaac aaagagaatc actgtgatca atattaacta
ggctattctt
669961 ctgcagctct tttctactca tttcttacct tttggaactc ttgtggtcac
atttcaacat
670021 cacagagacg accggctggg gagcacagca gcagaaataa ccaatcaggc
attatttatg
670081 cagcaatgtt gcattcggaa tggcaacgga gtggcatcga acaacaagct
ttgtccttag
670141 atgacaccgt tttctcaaga aaatgattca tttgactttg cccaaaacac
ttttgtcccc
670201 ttttacttgt acatgcaaga aatggattta gaaaagaaaa aaaattgtag
tccttaacaa
670261 agttcttcct gtttcaggat acctcctgga aattgatgat ttctcaactg
ttgcccaact
670321 ttatatcgct gtgatgacac tttaccctcc cccccagcca tcaaaaaaga
aagaaaaggc
670381 aacagtattc gaacaaatgt ccacctgaca tgcgcttctg ggcgttcagg
tgaacgatgt
670441 cagatgtttt ggggactgta acaggtccag gcaggataaa gaagcagact
tattttgggc
670501 cgcaccctct cctgcactct tggctttttt ggccgggact caaaactgcg
aagttgacag
670561 aaggtaatcc ccgccatgtg aggcctcggc ccccgtttgg aggcggggtg
tgattcattc
670621 agatctgccg ttggtataaa agaaaaggga gtcgccagta acccaacttc
aacccacagg
670681 aatcgtggcg cagcgcagga ccacctgggt ggttatctaa gctgatgatg
aatacaatta
670741 aatctattaa aaatgcttta gtctttttcg tcctgctgcc ccgtttcctg
gtggcagcgg

670801 tcatgttttg gctgcttgac tttctatgca taaggaaaag ggtctttttc aggatgaagg
670861 agcaggagga cgatgccgtc gatcctccac tgtgcatatc ggattccaat cgcctgttca
670921 ccgtggagtc cctgaaagcg gtgtggcacg gccacaaact ggactttctg aaagcagcgc
670981 atctcggaca gggagcgccc aacaccgaag ttgtccagct ggaggatcag aggcgcagcc
671041 gcatccttga ctacgcgaag gacaagagac cgctcatcct caactttggc agctgcacct
671101 gaccgccgtt catggcgcgt ctgaaggctt tccagggggt cgtggagcag aacgcagaca
671161 tagcagactc tgtagttgtg tatattgagg aagcgcatcc ttccgacggc tgggtgagca
671221 ccgacgcgcc ctatcagatc cccaaacacc ggtgtctggc ggaccggctc ggcgcggcgc
671281 agctgatgcg cctggaggtg cccggctgcc tgatcgttgt ggacagcatg gaaaactcct
671341 ccaacgtcgc gtacggagca tatttcgaca gactttatat attacaagag gggaagatag
671401 tttaccaggg gggcagagga ccggaggggt accggattac agagctcaga gactggctgg
671461 tcgaatacag agaaagcctg aaatcgtcca atgatttagt cattcatttg tagacaaatc
671521 gttacatttt agagctgctg tgactggaaa tgctgccggc tgtccaactg ggatttattc
671581 agaatcaaat gttcaagtgt atttattttt ctttatggtt gcatcagtga agtagaaact
671641 attggctcct ctatgtcttt agaatataat tgccggaacc acctgccgaa aggtcatttt
671701 gtaccaacct tgtttgtctg tgaagaaatg tattttgtgt tttgatatat tttttctttc
671761 ctttaaacgc gcctggttcc gaaacgttcg cgtctctctg gtcctcagta tttctatgag
671821 gttcggtctt taaacggagc agatccagca gaccgacact gatgttccga tactgcagat
671881 cagggtggac gcgtcgcgcc ccgaatggtt ctaaccgctg ttgttttatg gattttcttt
671941 cgtaataaaa tgtcgattca ttgatgaccc gtttgattga gatccaactc gccttggatt
672001 atgaaggaat ttttcccata agaaaacaac gatgtatttt aaaaaaaaaa cacaacaaca
672061 aaatagaaaa agaaaaccgg aaaatgtaaa gcacgtccag aaaaaactta ttcttaaggt
672121 caagacattt atttttgaaa ggcagtctcg gatttacatg aaaaatcaag agtcatcaga
672181 gagaaatatc tagtcaggga tttagaacct cgacgaacgt taaaagtagg tccggagttt
672241 ataaaagccg aaaaaagcca cgttttgtgc cccttctaaa gccactaacc tctcacaatc
672301 aaatgaggat aatcacgctt gatcctatta atggtgaaag atacatccat ctataaggga
672361 ctaaacacac aaacgttgtc aaggcttaga cactgatacc agagccgaag cttcagctgg

672421 gccactctgc aagacaatct gtcttctgct tctgttgcat tctgtcctcc tcagaaatgc
672481 tgcttttgcg tggcgcagcc ctgcggtccg aatttcaaca caatcatcgg tgcaaagagg
672541 gacaggaggg aggtgaagcc ttgcagtctt agggaaatcc ctgaagggtc acaacacaat
672601 gcaaacacag gagggaactt agttctggcg acccataaac acacggcaac actggcaaca
672661 acacggggct tttggaggcc cggtgtggtg taaatatcag tgatctctgg gttcatcatc
672721 agacagatcg gagaacattt atggactttc actcaatctc caaaccctca aaataacaaa
672781 gccaaccctc agactgcaag gatagcacag cacagattaa aatctgactc cattttggat
672841 ttaaaactct tttgtcccct ttcacgcgcc cttgtgagtc aagaccatca accccggttg
672901 aactgagatc tacgaaaaac aaaaacaaga cagaagaaca cgatgctgct ctgtagcagc
672961 tcatctggaa tgcatttcac gcacaaacac gtacggtaaa tgaaatataa cttgaagtgt
673021 atcctgcgag gacccctgaa aggaaccctc ttggaattcc tcgactctga tctggattct
673081 ttccatgtgt gagtttccat gggagtcggg ctccatccgt cagagacgac aggcaggcgg
673141 gaacaaaggg ttaagggtct cgggctggtg gtttacgtgt gctgctctgt gttgctttta
673201 tgatccgggg ggagtgctgc agatctcctg aggtgatttt ttttcccctt cttctgtggt
673261 acactataag gtgagggccc ctcggtccct ggggtcataa ctccacatgt gaatggctgg
673321 tagtgaggag ccggggcccc acacaaaaag gtctctgttg aacctttgtt cctgcttgtt
673381 taactcctga ccatttgtcc atactttaaa cagtttttat aatccttttc ccagacatgt
673441 tttccacaga ttgatagaga actaaaggtg ctgttatgct cttaactttt attgggattt
673501 tttacagcct ggggtgcagc actgatgaag gacctgacgg aatttgctgc agaaagacgc
673561 gttggagtag gcagctgagg agaattttca aatgctttaa ctgatatggt tttacttttc
673621 agggacagaa caactcctgg accttcagcg atgagcgagt aggtaaatac tggaagaaaa
673681 tgcagcaaag gtgttgactc agaatctgag ctacaaagtg gaaaataatg tcttctgcag
673741 cctcgctcat aacttgataa acttgatgtt tagaaaatca caacctgctt acacaatatt
673801 acctcacgca ttctagcttt tcttccaccc caaacagata ttgtggtaat attaaggata
673861 ttgcatacgt agataactta caatattttc agatatgaga gaggagaaaa caccacctca
673921 gcctggattt ggacctgtga tcagcatccc actgtttcct tttcgatggt tgacacctag
673981 cggtcacatc ttggtactac agatttttat ttttcagttc cttttaacaa aaaatgacaa

674041 atattcaatt tattatttaa taagtaaaga ggtgctatac catctagatg gtccattctt
674101 ttaaataaaa gaatgtctaa tcaagaagac cagaatatac agaatcattt tcttgttgaa
674161 aaatccccca attggggtaa cagcatcact gtcaaatccg accctctaag gactgaatca
674221 ccggtaactg atggagctgc cacggtaatg gatgtgcagc tcaaatgtaa cacaaaggaa
674281 ccctgagagg caaagatgtt gaatattctt taatggcggg atcagtcacc tgagctcaac
674341 cttgacaaaa tgacacactg aaaaacactg aagccagaaa gagccacagt tgacaactgg
674401 ggatgctgtc tgcgacgaag acccctccaa aatatccagc aaaatccttt gtcctgaatg
674461 acattcgctc agtgcttcag aaatatcaga gaaacatggc ctgcttgacc tgatgtcctt
674521 gtgctcttcc accgttgtcc tgttggtaga ttttatctaa tctgacatct tcatcattcc
674581 ttttacacct ccatcaggga tgacagaaat ggtgactaag tttcccgaca ggccccatac
674641 gccaactact gttactgtaa tttccaggga caccgagaca agcgaatgca gcctgtctac
674701 atttgcaccg atttgtatta tatttactca aagaaaacaa ttaaacataa atatgaataa
674761 ttgaaacatc tgagtgagtt tcctctattc agttgcatca ttttagatag ttttctggat
674821 gaagctgatt agtttagaaa acagacattt tctatatcgt gatgttgcgg cctttggcca
674881 caagatggag acaaatacta gctgaactag ccggtggagg gctcttgaca gagacctttg
674941 gcagttactg aaactttgcc agagggtaaa aatgtctgca gcatcagcag aggaggtttt
675001 tcactatata tacggaccaa gatgaatggg cccaaacact gtcttggtac ctgctcttgt
675061 ccttagatgc ctggggtgtc agttaccaac agctcagtga gtccggatca tataatttat
675121 tcaaaataac aagcatgaaa atacaacaac aaatacacac ttacagcaat tgctctttct
675181 gtagaattgt cacgtagtct atcataataa taaaaatatg aatcataaca aatcattcct
675241 ctgcataaca aaacagatgc agagcatgaa ctgaagaggc aaccagagga attaatttcc
675301 ctaatatgca gaatagaggt ccatatttct aaccgactgt tgcgtttgaa aggatgtgtg
675361 gatgtgccat gcagacttag aaaaactctg aaaatatcca acattggtaa tacattagaa
675421 atatatagac actaatgagg gtaaaaccac aaaaaagtgt tgaaggaacg acaaagttaa
675481 tgacaataga gaaaactgga caacttttaa atcaacactg ttcagcaata cttgaattta
675541 aaaagcagaa attaaccaag acataattac aaaaaaatca acatcacaaa ctacacaaac
675601 cttatctgtt tatcatccat gatatgtagg gctgcaaagc actttaaaca tttaaaaaat

675661 acttcaaatt ccagcaccca cattgaggat attctgatta cttcatgcgt tatttattct
675721 caccgaatga tgtcatcaaa tcttacacac tggtcctttg acagtgatta gtggttagag
675781 attatcagtt taatgcagta gaatgcaaag cttgcttaca attttttttt agtaagagcc
675841 tcattttcct ggtacgttac acccaaagtg aaacattttc acatgtttac ttgccattgt
675901 ttggttgaag tttttttttt ttttaacgac attcatttta aatacattta aagcacagcg
675961 tcacacgtgt ccacgtcatc agataatgga gacgtacaga tagattccag gttagtccgt
676021 tatgtaaaga cagctgtccc acagtctgag tggttgcttt aagctgtctt taaacattca
676081 aactgagctc ctctaagagc gggagacgag gaaatgcagc catgttgtaa tcagatggag
676141 gtatagtgtc tcccaaagct ggtgagtcta atcttttctg gcagaatgat gtttacagag
676201 tgctccggct cctccagggt cacgcctcct gcctcactca gtaggtgttc cctttgttgg
676261 cgaacaagcc tgctgtattt aacgttagcc agccatgtct cacagcggcc gaagaacaca
676321 atgccggtgg ttcccaggat aactaagcat gtaattagag ccagcatacc gaagcaaacc
676381 agctgaccgt gggtgacgag gatgccgcgg gagtggaccg tctcctttag ggtgatctta
676441 agcaggtctc tctcaggagg tctgagaagc ttgtggaagg tgtgagcggg gagggagtaa
676501 tgctgcgggg tcagtgcgaa cactccgtga tccactggcc tggagatgac ctttgctctc
676561 tgctgcacag cacacgtggg ccctttgaac cacttggggc agatgcaacg gaaggtcccg
676621 tcaggctggc caacgcacgt gcccctgttg ctgcacggcg ttccagcaca gggcgagagg
676681 ctggcggtgt cgttgcacgt gaaaccggtg aatccgcgct gacaggtgca tgcgaaggcc
676741 aggccgcggc tcgtgcagtt cccgccgttg aggcaggggt tcggctgaca gctgtcgacc
676801 aacatctcac aaaagtctcc agaaaatcca gggggacaca agcaggaaga gtaagctgag
676861 gggccgtcgc cgtctatgca tgtgcctccg ttctgacaag gagagctgga ggacagagag
676921 acgggtggta aacaaagagc agcactgata acgtttgctg tacggtgcaa tatttaatcc
676981 tgtgattata aagccgcggt tcatgaggcg gctttgtttg ctcagcaaac tgggctgggc
677041 tgctcacggt tgctgagcat tgctgggagg aaaaaataat ataaatcggt ccacagcaat
677101 ccttcagctg ggttagcaa gcaaacacaa gatttattct ctacataaac cagacagcat
677161 ggacggtgga atccactagg atcgtttcat tggtttcaaa atcagtgctt tgacttttca
677221 actacaaggt cttaagtcat tgaaatgaat tcccatagtc gtgcagtcca atcacacatg

677281 tgcgttatat agaaaaccgc tcctcaatat ttctctgggt tctacatgca aagcaatgat
677341 ccagtaaatc atgtttctct atctgcgctg cttgatgtct tttatttctc aaaaaaaaaa
677401 aaacctaaaa actacaaatg aaatgaagct tcaatggtgc atcattgaat tccttcacct
677461 ctgcaattcg aggcacattc aaaaccagat aaccgagata aacctgatca atatttcaat
677521 tgtgtaatta ccgtacacaa atctctgaca tccctggcaa gagataagtt taataatagc
677581 tgccagacac tttacataca tcgctactgt ctagaagagc ggaataatga cactttatgc
677641 agacttctgt tttaattggc aagttttaaa agaagaattc ccctgacaag gaagtgaagt
677701 tcctcttggt ctcattttaa tattacaacg gtttttgcct cccagtggag gagtagagct
677761 gtcaattcac tggcattgag cagcagtatt ggattttcgg tccatgtgca tcaataacaa
677821 gtccggctga gggagaatga gcaccatggt ggtcattgtg tgtctgtgtg taggggctgc
677881 agaggcagca ggaggatgtc tcctcaggat cagccatggc tgtaatccag ctctgggaac
677941 ggctgacgtt gaggtgatct gcaaagcttg gctgatcttg aaacttccta gaaagggaac
678001 ttcaagagtc gtgtaagggg aggtgtgtcc tacaaatgac ctgaatacct ggccgacgca
678061 tatataatta atgacgcatc ccaggaatag ctgcaatcac agataatgat ggcacgtgcg
678121 gctgattaat tgccacattg attggacaaa ctgcgccggc gtctgatgtg ttagggacac
678181 aaagaacgtc ttcctgtcct actgagctgt ggccccgaga ggacgggacg gcagaatgaa
678241 gcaagctcct gatcctgtag atcaccgaca tgctgggcga tattaatgtg ctggagtttg
678301 aaattcggga agcagcctgt tgtgtcaaaa acacattttg atttaaattc taagcagagt
678361 cacactttgg cttaatggat ctgctgttgt tgtgacccag gagaggcttt gcacttaatt
678421 tcggaggaaa acttcttgag tcccagccac agacagccat gaacagatta taaaatactt
678481 cataagaagt ttaacctttt tacacaagct ttggtatcat catagggatt ctgggatgtg
678541 cccccccatg aacacatatt tacagtgatg gatttctgcg gtctattatg tcaaaagatg
678601 agctggtcat acatattaaa gcagaagcat tctcatttat ctttttgatg acattaatcc
678661 tcacgccgcg tgttgctgga atagcttaca tatcaggcca acatctttcc tgactgtctg
678721 accccacact tcatcatagt ctcactcctg gcttaccagc aggaaagcag gaagtttttc
678781 attgacaagc acccctgcga tgtcccttgc cttagttagc gttgcttaac cttttcaata
678841 tttgatggct tgggagtgaa catcggcttc tcagctgcga ctgctcttta tatcagtcac

678901 agtctaacac agcacgccac tgaacaagat acggccacgt ggacaaggac aaaaatgcca
679061 tttctcctgt agcgaggcaa cgtggcacat ggcaggtgga ccccgaagaa ctggcgaagc
679021 gatcagcatt tgaatgtctt gatttccttc acagcagaga cccaacgctg gagtggaata
679081 tatgaatatc agagctgtat ttgccagaca acaacaggaa actgctttac agccatctag
679141 aagtgttggt gtaggttctc aacacctttg gggtacatcc aataatcacg cagatgcttt
679201 ttggggggaa gggggtgtc attgtcatca cattttcaat cagatggtaa aatgggtgag
679261 aaatcagcac ctttccatca taacactgcc tgcaaatgtg tctgtcaaaa accacaccgc
679321 accagtctca ccgtgcgcta atgaataaag aacactcaat ccaatttctt ctcaacagcc
679381 acggagttcc tattgactgc ctccggtcga cagagaatgg cagcgctccc tgtattgatc
679441 attgatgcta atgataaaac agaggagatg acagcacaga agctgttacc tgtttgagaa
679501 gcagagtcct ggcttcagct ggcaatgttg ccccacgtag ccccgaggac agatgcacat
679561 gtacccgccc tgtccggtct cgatgcaggt ggcgttgcct gcacagggct tagacgagca
679621 cagccgtgta tctgttgtga agcaggaaag gaaacatttt ccaaaagggt tttagtggcc
679681 atgtgttctc ccgcctcctc ctttcatgtt tctgtgccta ttttctcccc ctgcctccgt
679741 ttccacattc tcgctcgtgt tcacaccaca gcatcttgtc accgagaaca aaactgtgaa
679801 ctcggcgtgc acaggaaagc agctcgtcta gaagaaggac gcggtgcaag tcttcacgcg
679861 gaggtcaaca cgaaagcaca aacaggtttt gtttgatggg acacagcagc ttgttgtttt
679921 ttgttttttt aaaaaatgga taaggtctat gtaaagacgc agctaacatc tttgatgctc
679981 aggatatgaa atttgttaga aaatacacac acattcacac tatgacactg tactgtatgt
680041 actgatcaca gaataatggt taattatttg cgtattatga tgggcaatta aatttgaagt
680101 acaacatttc aatgaatcat ctctggggag aataatgtgt tattttgcag acaaatgacc
680161 aattaatcac caattagcca ccaagtgact gtacattgca gttccccaat gtgacctcta
680221 ggggcagaca gaggaacgtt agcagacgtg ctttgactgt tggatgattc tgaagcgtta
680281 gtcagcctgt tcttgttcaa ttcgggttaa aatctttatt gaaatacagg cacgagtgcc
680341 tgtcggttaa tgatagcacg ttgcatgaac attcaaaaag actcgaatat gggaaaagtt
680401 ccatattcag atttaatttt gcccagtaga tgctgccaaa tctgacacat cagacctttg
680461 aatggaaaaa gatggataga tagatagata gatagataga tagatagata gatagataga

680521 tagatagata gatagatagg cctgttgatt tagactcctg gggggggggg
gttattatgc
680581 agactccggc ccatgagtgg gagtttaatg aggctgacct tgatcgcaca
ggctgcccac
680641 ccatccctcc tcgcagacac actgccatgc cttctcacat ttgccatgca
ggcagccggg
680701 gaagggcacg cactggtcac agttctctcc ttgccacccc ggtttacacc
taaaagggaa
680761 agaaaatgga acattcctga actattaata aaccaccaac agtgcagtac
tgctcacgcc
680821 tgcataatgt ctgcccggtt cgtttggctc cttttaatcc agctccagca
aactggataa
680881 tttccttaaa agtaaaaata aaggacaaca gcaatccctt ctccttagtg
aatctagcag
680941 caataatatt tgtaaagtgg gccaggatcg tctcttaaaa gctttgggcc
gtctttggct
681001 ggattaccat tcaatttctt tctagtgagt cacatcagtc tatggacaca
acgttgcgta
681061 atcccgcagg ggagagacgc ggccaccatt taggaaaagg aatcacagat
ggtttgggta
681121 cctgcacttg ccaggcttct cacaaaaccc attttctatg ttgcactcgg
cgctgcattc
681181 ccagcctttg agacagaaag aaaaagcgcg tttgcctcca aaatatatcc
gtaaaaaaga
681241 gacacaagtg tattttaaac acttagagcg attaaatatt tatatgttat
actttttttt
681301 tttgcatgcg cgaagattcg actttgtgga agtcaaatct ctttgtctgg
tgagctctcc
681361 agattggtga aattgtcaaa ttacctttgg cgatgcctgt cgcgaacaga
attaagacca
681421 ccgctgtgag atgcatcgta attcgtaaaa gacacatttc cctcctgcaa
aacagagcgg
681481 aggagacgcc aaatttctct ttgtatgagg agagaaagaa caacacgcga
tcgtgtatct
681541 gttctggtga tgttctggtg ctcaaaagtt caatcgagat aaagaaaatc
aacatcaaat
681601 ggaattttta aacaaattag cgggatgacg cgaatatgtc atgctgtttt
gatgttaatg
681661 atgagggtga gaatcgtggt ggaattgtga gtattgctgg aataatctct
ccgtcttttc
681721 cgtctcttgg tgattgacgc acctgaagcc agacgcgtct gttccaagcg
gtatccatta
681781 cgcacaggcg gaaagaagag agacgcgttt cacttcaaaa tcaacacatt
tcccgacaaa
681841 tgtaacaact taaacgagct ccccccggtg tgaacgcact cacctatgtc
gccctgatgc
681901 cccaattcca ggaggtcata tttgacagga caaatccaag ctccgagcct
ctgggagtga
681961 cactacaggt gcacagaggg tcggaagctg gtgaagactg gaggtacgga
tgggactgct
682021 gcatcgtgcc tcgtcccggc gcaatacgct cctccctcct gaaagagagt
attatcatgt
682081 gctcatcagc ctgacccccc aggagagagc caaagccata aaccaataga
aaaactgatg

682141 ttgatattac tgaatacaca acagagccta tagacttgtt tttcatgatc agaatataaa
682201 caggaaaaaa actgatgtta atttggcact aaaggatgga gatgagcttc tttgcttaaa
682261 gtaagagctg tacaaaatct gcttcttcag tagtttaggt ctgcactcaa atctataaag
682321 cataaagtaa accatttttc aaataaatct ttacaatcta taaggtgctc tacatgtgca
682381 tttgccaatg ataaagttac aactgcacag gatttagctt tattgctcct gccagagaga
682441 aaacagagac caatcgcagc tcttaactta tattacatct ttgattcgct attctgggga
682501 gaataagact acaggcaaca gatgcaggtg tttgctctct aaactgtagc tggtccactt
682561 tcaaaaggct tcattcataa ccatcactta aaatggagat cacaaacagg aacacaccag
682621 atgctgaaaa tgcagccagc aggagtaagg acagctaggg cgggcatcca tccatccatc
682681 catccatcca tccatccatc catccatcca tccatccatc catccatcag ccgctccctc
682741 ctaattgggg taagagttga tctcagcact atacaggccg gacatgtcgc cggtccatca
682801 caaaccctct tgttatgagc tgatggtgct aactgacagc gccagacagc aaaatactga
682861 cttcaaatcg atagcggagt ataagaatga caaacaagtg tggaggagga aaggagggta
682921 ctttcaaatc ccaaatattg aaaggatatt tacattcaca gttctccacc agtctctgct
682981 gaatgtgatc ataattacag tatgaatata catctgtaag aagcaaatgc cctcaaaggc
683041 tcctcggact gcacccccac accaccttaa tgaaataact gaatgacgtc tttcaggcag
683101 cgcccaccta tcatacataa taaggccact tctattctta ggcagcttat tgctgtccgc
683161 agtctccctt cgtcttcccc aagggcacag aatggtattt tccttcccac aatgcacaag
683221 gtgagcagtt taggcatcta attatgagtt tcaatccatt ctgacattat tggattttta
683281 gcaggggctg aaaatggtgg ctgagcaata ggcacatgtg ggtctttgag gtgcaacctt
683341 tggcttgaat atcagtgaga agagcagatg aatccacaat attgcagata acatcactgc
683401 catgctttgg tagatgagga gctagccggt gtaaacacag cagtcgtgag ccggtgtcac
683461 aagaggagaa ttgatttgac ataattttct gtgaggttgc tcatattccc cggctcattc
683521 ccccggcttc atgtgtttat ttccaaatct ccagagacgg agctgatagc tgtagcacgg
683581 cattagagtc acggtgatgg tctccctgcc acagacggcg ccttttattg ggatggctga
683641 ggtttgtgtg cacgttaaaa tgatggaatc gcccagagat ttattttta cccggctttg
683701 acctcatctt ctctgatatc tgagctttaa tctgctccgg tttggggtgc ttcaggtata

683761 aattggatat catggatttc agagcgtgtg ttttgtgact ttgtgtcacg
gtcgaaggtt
683821 acactgaaca aacccagact ctctgaagga aggggggggg gggtccacat
gtttctggca
683881 gctgcgtagg ttctgcttca tgcttgcaaa tgaaaagcac attttcctcc
caggtgtgcg
683941 cctcacacat catccagaca cccacgatac ccttacaact gcggtcaaca
cgataagcat
684001 cctttaatgc gtaaagctta cctttctgtg gctgcacctc gggtatcatg
gggagacaaa
684061 caggttaggt ttagaggagg atgcgcttat gtaggcatca tcttgaaaag
aacaagtgat
684121 ctcactaaag gagaggaaac caaaacaact gaggcaagag agacactttg
ataaaatcac
684181 agcttctcca aaggctccac ttcaaggcat tgtgaatgta gtccatagta
acaaaccctc
684241 taatcaacac agcgacagaa cgcagtaaat aatagcccag gatgacgaca
caattttgtt
684301 atcctacgcc aaaagattgc gcttggcttt tgagctgcag cctgattgta
tagagtgaag
684361 ctagctgagt gctaaaattc attacaacgc tctgcaggtt ccagcgcacc
gagccacggt
684421 aagtggcaaa cttaattctt ccgaattagt ctcaggaagg tgaacatttt
tcgctccctt
684481 cgctcccttc attgaaatcg tcatccagct tcctgtcgct ggatctgcac
ttggctttaa
684541 aagtgagcac caggtgacca caggtgtgtt agcctcaggt aaatattacc
gccgggtcct
684601 gtgctgcccg tttgtaaata gtgtctcgtg tctgagcctt ttcttgggga
gcacaagcac
684661 ggctggagcg ccggtgcagc cagctgtgat gattaaggag catcccttta
acaccaggca
684721 gatgctttct gccagttttt agtggaggaa tggaggaaag tgatatattt
tcgcatcatg
684781 ctttattcac ctcaccatta tttacagaca gaaagattta tttttttgc
cagctctcct
684841 gagattgact cttggctctt ctgtcgtctc acttccacct atccagagag
gcatctcctg
684901 aaatgtattt atgatgttta tgttctctct gcttaacctc cccccgagtt
ctctcaaggt
684961 ttggagcatg tttatgtggg catgtacgta cagtaatatg cgtgaatgtg
catatttgta
685021 tcctttcacc agaggacgta ggaaatatcc tgatttcgaa ggagaagaat
cttttggtaa
685081 ggaatttgct tgctggttgc ttttggtccc tgtgctcgct ccttcaactc
cccatcgttt
685141 ggttttgcct gagtgggcgt ggcctcggtc tcctggtctt tgcacacctg
ttctgcctca
685201 gctcatccac atcgatcagc tgctacctaa aagtctggtc ctcctctcaa
gtcttcacca
685261 ttttggtgtc aatcctacat ggtaacttgg tagtgggcac ttaaacttta
cgtgtacgat
685321 ttatgaatag gattttgtcc cttcgtccaa ccagtcacac cctgcctcct
gcctccatcc

685381 cagttctgtt cacccatctg caacctttca ggccctgata tttgggttta
caacgataag
685441 cattgcacta acaacatatg ctttttctaa cttccactga actcatctat
atttatacag
685501 cgtgcattct caggaacttc agagaaaccc agaacctgag ccaccaacat
gcaacagtag
685561 caagaaaaaa aatccctttt agcaggaaga aacaggctca ggctgcagag
ccaaaactca
685621 tggataaagg acggtcagac agacagacat ctttaaccag gaacagtttt
ttttctctgt
685681 tactaaaata aagggatcaa aacgagcagt tttttctgtt tgtcccgtca
tttgtttatg
685741 aatataatgg gggattgctt caccaaagtg gaaattaatg agtgggcggc
agctccttct
685801 atgtttgtga actgatattg caggtaaatg tttctcgctg tgaacagacc
tcatttcccc
685861 ttaattatgc atctgcagca tgagggcact tcagtctgat ctcagtaatt
cagcagccct
685921 gcttctaatt gcatgaaggt tattcccacc ctgactccca cctctccatg
caggtgagga
685981 ataattacat gcaatggaga cacattacca gctgtggatt gtttcacttc
tggagttttg
686041 gagtgaattc atctgaattc caggcacacg gaggcaaagg ggctctgtcg
gtacctcatt
686101 tagtcaaaaa cacaagccac agtgcagatc acctcgtcgc agctctcaga
tggtacgtgt
686161 tggtgcagga cattaatccg gaacatgcaa tcccgaacac tattaactgg
ttattagcgt
686221 gtgggtcggc tgcacagttc gttcctgtct ggtggttttt gagcacatca
ttaggaagca
686281 tcattagaca gtcacaccca gcggggctta gtgaggctcg cttaacagtg
acagtgcaca
686341 atggtcaatc tgccccaccg caggcacacc acgcgtacat cactcggaca
aatgcgcatg
686401 tcattagaat aattagcttt ttaaaaaaaa atggttagag catctggcaa
cacattgcat
686461 tccaataaaa tataattatg atctggccat atgagccatg ttaatgtaag
aaacgatgta
686521 gtgaggaggt tgtaaaacat tctatatttc tcaatgtact aaaagcatct
ctgagaggag
686581 gaatattttt agctgtactg tatttttaac tggtgaaatg cctgcgcttc
atttaaagat
686641 taccccctgag cacttcaaaa cgtgtaaatc tattgtctca gctcccaaca
tatgctcagg
686701 ctgtatggag gatgttccta caaggaactc tacttgctga gcctgccatg
cacaggcaga
686761 tataacagcg caccctatta gctaacacca gtatgatttt aagcgtggct
ctgacattgg
686821 actgcagttg ccctgctttt gtcattttcg acaatcctgc accgcaagcg
ttgtgtagcg
686881 tgcactcagg atgcacatcc ctgtgttgcc tgcctgcctg cctgccagct
cctgcctgtg
686941 ctcttttatt tcctgcatgt ggtttgctct ttcttcacca tgatccgttt
taagccttcc

687001 agggaataat ctcctcatct tctcttgttt tgctgagttt tgtttctgta gtaattgtaa
687061 tatcatcatg ttggagtgtg gccgtcgata cttcaaaagc acattaaact aatttttgat
687121 gttccacaca gacacttgcc ttttgttcgt ttgcagctcc actgctaaat tttaaggctt
687181 acatatttcc cctatttccc aatcccagca ggcagtcaca agctccaact gttcagtttt
687241 aaagacattt caggcatttt aataatgtag acatgtctgt gattgtgaca gtcaaaataa
687301 ggactgggga gggaaatcat gactccatga ggatttcatg atggagccgc aacaccagct
687361 cctagctatg tggttaactc tcaacttcca aatacttccg agtcattaat tattaaacgg
687421 gaaagaagtc tttgcctaga aggggaaatg gttatttgac agcaaagata agagaaaagg
687481 gtgaagataa acccttcctt agtgatacag gcccctgttt ttgttgcttt ggaactctga
687541 ctacattaac gtcagggtct gtttttccag ctctgttcgc cagataggct acagaacata
687601 tggcgtgtcc ttcaattaga ccctaaggta gtctgataaa tactacatta aggctcacag
687661 ctgctgtttt actgccaaat gacaatggta gcattcaccc cgcagcccag gtgcagtcgg
687721 ataatatatc actatcctgt tgtcaacatg ccagggagac aagagaatta ccttctcctt
687781 ttagcgtgat tgctactgca gaaatctcag ccgaacagcc ggagacatca gcacatttct
687841 ctctttcgta tataccaatt aaatgagagg tggggaagag acgatccatc tccaactgac
687901 tggtgccatt taatggatca tccatgctct atccccaagg gcgactgccc actaagcatc
687961 ggaagaactt caaacagcct cagatttctt ggtttccatg ccgcagaatc ccctcctcta
688021 atgaaaaaac ctgaagttta aagactaaat attttgctca ttatagagtg cggccattta
688081 agactacaaa caaagagcac acggccccga gggtgatttg gctggaacgt ttgaaggcaa
688141 tgagtcattt aattgttaga tatcgacctc acggttccca agtatgtcac ttcctccgcc
688201 tgctgtttga ttgtcggtta tgtcctctga aatcaataac acacacacag actgaaacat
688261 attccctctg ccccgttagc ctgcattcct tcattattca gacaaaactt aatggtgctc
688321 ggccccgttt ctcactgata ttaattcatc aaatcttttt ttattcagcg ggagcatgta
688381 atgggttgat gattgaacct ggcattactt caggcagaaa ctcattttgt cagttttaat
688441 catatttaat ctaaaataac agctacacgt tgtccaaaag gttgtgacta actgaagcta
688501 catgatgttt aggacccgcc aggtgcaaga gacagaacct taatgatgat atctgcggaa
688561 tgtgccatct gctcggatgt gagagatctc agctaaattt cattaatgga gatagcacca

688621 tcgttatctc catagcaacg gctagatctg tttgcttgga aaatgctgtg gtagtagggg
688681 aggcagattg acaagactga tagtgcgcgg gagcgttgga gagaaaagcg agaactgcga
688741 ggaacgagtg caggatgtct tcacctgatc tcccgctcac ctgatgccag ttttgtccgg
688801 gcttgttgcc atggcaacag atgctatcta ataaagctgc tgcaaccttc tgtctgcatc
688861 accaagatct acgaaataga tcactttaat taaaagaaaa acaatcattg attttcatca
688921 ccgaccctga cgaaggtcat tgagttgaag cctttttttt aatttatcag gtcagtgaat
688981 cagtgtagtg caggtgaggg catgaccgac cctggctaaa actttattac ttttatacag
689041 ctgtgtcttc tttgactgtt ttattaatgt gaggcccgtt ctctactcct ggaggagctc
689101 tgctgtcatt tttgttccag cgtgtgcttt agaacataat agagattaat ggtgttagcg
689161 tagcaccccc cccacacaca cacacacaca ccccacccgc ccttgatctt caccttctat
689221 tattaccaac ctgtctgcta tcaatcaaca cacacagacg ggtaaaagac acagcgaaag
689281 tgggaggaca ggagatttga gggagctcaa caagactaaa ttctgtctgt ctcccacata
689341 gattgtcttg atcaatgtag caactcctta taaacagata atatactgtc gttttcacac
689401 gggtgtgatg ttcttaagtg ctctgcgcca cagcgcatca atgacaaaaa tgcacactct
689461 acgcaccaac acaataccat ggggatgtga gagaagcatg ggcattaagc ttggatggga
689521 aagtggtttt acaaaactct acatttttta gacatttcat aaagcctctt tttcattttc
689581 aggggcccga gtgtttgaac agtgtttgtt ggcatcaatc actgcgtaaa caggaggtta
689641 ggaggtgatc tgcgtttacc cagtatttac acacacacac acgcacgcac acacacacac
689701 acacacccac acacacacac acacacacac tcgaccaggg ttatgtctat cacaggaaaa
689761 caggaatttg tcgacgctca ttgagatgaa cttttagagt cctgttctaa atgcttctac
689821 accctaaaaa cagatgaaaa atgtctgtat tttggttctg gaggcaaacc aaaaaacaaa
689881 aaacagagca cctggtttga aggcaaacaa tgcttgattt ctcatttgca gacggtgatg
689941 cagaaagatc aataactgct ccagggtcag ttgttctcag acagaagccc gtgtgaacca
690001 ctttaaagta taaacaattg cacaatctag aaaacaggaa gatgtgaagg gaatgcatgt
690061 gtacccccttt aacaaataag agaaatagcg ttttcaaaat ggctgctgaa ctgaactctg
690121 ttgcaatcac agtttaatta gttctttgca caaatgagac tctcagcagg ggtcctggag
690181 tgttagaaac acagcaataa cagatttata acagatttaa tctcctatga aatccagagg

690241 ggtgggttaa acatctagtt gctttaaaac agcgccatca ttaatgtttt tcacagatca
690301 gacaaacatg tgagctacat ccagatgtgg tcatctatag actcattttt ctaattatcc
690361 tggttacagg acatttatgg gaggggcaag ccttcagcag catgtgtgta gctgccatta
690421 gacaacaggg atagaccatc acaacccggt tgcaaccagg caggtactca ggctgtgatg
690481 gttacagacg cggcaacaaa gaaatgcaaa tactgtgagg aggaaaacag ccgggcaaag
690541 ttattcaaag atcagagaga gctgaagtgc gctccgacgg agagaagagg gatgagagca
690601 atattccaac agctgaaaat gccagtgaag gatacacccg cgtgattctg gtgtctatga
690661 gaaggggaaa tggacagaag cgtgtgctgg tgcccccca tcgagagacc gaacggccca
690721 aaggacgtcc tttgcatggg tcatcttcac ctcccatctt tgtgtgtcat gctgcagttt
690781 aagactcaga gtaaatcaag acctacatca tctctccgcg ccaacaagtg tttctctttt
690841 atttgcagac aaatgtcaga tgtagaataa atgtgagctc aagatataat gctcatttta
690901 tacagtaaac atgatgaagc gtgatgacga acccagccac tgtgaaatat cgacaccgct
690961 gcacgttcac acgttgtgtc tccatatttg ctgatgacat attgctgagc acgctcatct
691021 tgtattattt atcatgagcg gtcactgtaa tcctctcctc cttcagccct tgagctggcg
691081 agaaggggag aataaggggg cggatcagat attcccgtcc tgatttaatt gaatctcgac
691141 gttcagcggg aacctgtggc tgccggtaca caaaaaagac caaggtttag gacatgttga
691201 tattgcctct ctgctcgtcc ccattcgccc actttatgaa ttattaacac gttcacagag
691261 gcctggagat gcagagacat gctcaacctt taggttcaac ccaccccgag ttataatgag
691321 tctggaaaca cgcgtcggct gcagctctgc tgttgttgtt ccgttcgccg actcgcccac
691381 caaagccaga cggtttctgg gtcagtccct ttgaataaaa gcgctgcttt gttgtccttt
691441 catgggattt ttttctcctc gcaggctctg cacccagatt tttacttcca ccgcagccaa
691501 cacacacgac ggacctaatg aaccctgttg ttttctcccc atgatgcaac gggagggcaa
691561 ttctctcaga aaaggagaag cacggaatga cgcatcttcc atggtgaaga cgctcgacaa
691621 agagaattaa agtggaaaca aaattagaga agaaaatgta ggttgattta tacaactttt
691681 aagagtagat ggatgcaaaa atgaacgtat cactgagtca taatatgcca ggcacaggct
691741 gaccttgctg tacttgttgt gtttgggtct ctccctccta cacctctgtt ttctttcacc
691801 tccactctga ataggagcag actaagtcta aaaacagagc atggacccga tgacaaatga

691861 aaaggagggg aaaagggtaa ggacagagtg tttgtgtcag aacaaagcag agtaggaagg
691921 tgatgctgag aaataatgag gatggagaga aagagatgaa tgtgggggac agattccggt
691981 ttgtttagtt gaaggtaacg gctgacaaag aaaagggaat agcgagaaac aagagttact
692041 tcaagaaaaa aaatccaaac acaaagaaac tgcagttaaa gtagctcctt ttgcagcttc
692101 ctgagctgca ttatatttcc aattcaaaac aatattgcac atattttaat atataaaaaa
692161 aacgaagtca ccaactgatg gtgtcttagg gtcctaactc cttttatatt gtatatttat
692221 attaccggta tattcaaaag tcggaaacta atgaagagaa ttacaaaaat ggcatttaag
692281 aatgtggaat ttaaaagaaa aacatcaaaa acagacacca tggaatctga actgtgaaat
692341 atgctccgat ttctgtttgg tggaatacct ttttcctatg aaagctcaga catgggacag
692401 ttgaaacaag gactcacgct gggcttcata atagagtcct cccactggca cggaggtcct
692461 tttaaagcag tcttctttct aaacgatagc ccccgtgttc caaaaggcca tcaactctct
692521 gaaccagggc gctctacttg ctttcatcct gtaccggcag aagcctcggt gcggaccagg
692581 gtcggactct cacacgagca tccattaggt gcacgatgag cttgttggtt attattcccg
692641 accctgtgat cacattgtcc tcgaagcgac gcatgtttgg cacagcgtcc actgagtggc
692701 ctttgaatgc tcccccgtgg taatcagaaa aatcacacac atccaatgtg tgtgtgtcga
692761 tttacatcac aaaacatgga aaacacatct tcaattagtc gagaacaatt aacgcccatc
692821 agagcggcca gacacagatg tgtcttaatt aaatctctgc attaatcgct tgatctgctc
692881 aggtattatt atatttatgc ctttgcctga aacaagtgat gaaagagcag aaaatgataa
692941 tgatcttcat tgagtgtgtc actttctttg ttgagaccag aatggaaact ggaattgatt
693001 gacatgcagt ctaattatag tctctgtaaa gttgaaatca ttttactttg ttgcctggaa
693061 acagatggtt ttatccatca agctgccttc tcagctcctg tgtacacttg gagagattat
693121 ggcgtaggtt ctaatctctc ccatggaagc caaatgtgtc acagataaat cagtcgggat
693181 gttactcacc gtgaacccca gtcatcttaa tatacactgt gctgccacta cgtcacagct
693241 ggctaagaga actgtaggac aggctctggc taccatggtt agcgttagaa tatatacaac
693301 taaacatcaa tatttggcat ggcatcacaa acctgactag aaagatcagc tcaaattgtt
693361 aaagtatgat atagtaagta tctcaatcat taaacaacga aaccacagtc atcattgtgg
693421 tattttatgt ctttcattac aaaatcgaga catcagtttc ctggtatata tatatatata

693481 taaaaaacat tgtttgcaga gaaacaacgg tggaaagtag aaacatggcg atccatctca
693541 gctgagtgtt ctgtgtgttc acgtgtttcc tctgagggtt tgtaaaagta ggtgcgcacg
693601 tggttgtggc ctctattcag gctgttaact gacgggcctc agtgggtctg tggccaaagt
693661 gggactgtgc atctccctcg gagtcgtgcc gttcaatcac agaggggagt cgcctctttg
693721 aaacggccgt acctgcaccg acacacaacc cgggcggaac gctgcaaacc ctgcaacgct
693781 gtaccggctg cacgcatctc tgacatatgc gcaggggttc tgtgccaaac tcctttggga
693841 gaaatgtaaa tgaaaggtac agcagatggt tcattcacaa cgctggcccc caaatgtcca
693901 aattaccttg ttatggatgc ctcagcctca gggtggggag aattcagaac tccaccatgt
693961 ggaggggaga taattaggac tcagaagaac atttctcttt tatatacggt acactcttct
694021 tcttcggcag agagtgattc aggtttttgg agaccaggaa aatcggctaa agaggccctt
694081 ctgtgtggag tgtgaatttt attttgcagt tgtgactcac tcattctgaa ccgtccagac
694141 tggattgatc gaaagtcaac tcgcattttc ctttttcag tcaggaagta agaaatcctc
694201 cactttgcaa gacaaatcaa gcagaaaatc tcagattcta atcaagcccg acgacatcag
694261 accacccagg cacagcagac aaccaccttc agtgtctctg gaaccacccg aagacccgag
694321 gagccgcagg agctgtccgc ctctgaacta gagaactctg tatttaattt ttttggacat
694381 tttcagccac acagcagcag acagctttga tgtcaaacgc ttgcgcaact ttaatctgcg
694441 gtgccagtca tgcagcagga ggtccggtcg tcagacttca cctgcaagag ctaaatcctt
694501 catttaatta gcttcatcgc tcattaactg tcaagagagg ccatttcagg ggacagcagg
694561 gatgggctct gaattttaca ttttatcaga gggccgagct gtttcagatt gttttatcat
694621 gtattattaa gcgatatacc tggtcctggt tacacatata gggaaaaaag tgtcatgtta
694681 ttactgacct gcatttaaat tcccaacaaa aatatttctc gttgccacaa aaaagttgtc
694741 agaaagacca agaacagaac aacgctgacc cagttcatca taaaccctga ggattccgtg
694801 gattataatt ccagacacat cctcataatc ggcacaatta caagctatgg acttctatat
694861 gcacagcgga tcaagtcaga aacagataat tggtccctca gtggctgcac atcagccgcc
694921 atgctgctgt atacgcacgg agagatttct actgtatcca tggtaacggt tttgcatcga
694981 tatcccaatt tttaccggag caaaaagctg cacccatcag cgaagttaat cgctctctct
695041 ttacagtcgt ctctccgtcg ccatgaatgt ctgcagttgt aaaaatatgc atttaatctg

695101 catgtttggt gcacagatgt gagaatatgc tcagatttga tctctgcagg cggcagcaac
695161 agcgagtctg acctttacat ccttcactcc agcaccgagg tttaattaaa gttggagcaa
695221 acaacgtggc gagctagccc cccgtccccg tctgcctgca gcaattttaa tgccactgtt
695281 tctgcggcct gggcttcatt tactgtcgcc gtgatcgtga taattagacg gaatattagt
695341 ggaaatccaa tccgctgtca tccctgacca caaatgttgg aacgctgcaa ggcttgttgc
695401 ctgcaatcaa acaagttttc cagctgaaat tagcacttta aacttcttga aaagacagtt
695461 attaataacg tacaaaatac gccgtttttg cggcaacaaa ttgtgcgacc atgagaaatt
695521 tgaaactcag gatgctccct tttcattttt aaactgtttt tttctctttt cattttcccc
695581 ctcaggatgc cacatctgaa gccacaaaac tgccatttaa aaagagatta aaaaaagaca
695641 gaccaaaacc cttaatgcat aattgtggta atcttgtgat ctttgaacat aagagggtcc
695701 tggaaagccg ctcagaatgt taaacacgct ccactcagga tgaataagta tgcagctgct
695761 ggtaccatcg cagagtttag cccaataatg gacagaatgg attgcattaa tttccttgga
695821 agaataatgg taacataatt attattaaat gaatggtggg ggagggctgg gtgacttcat
695881 agattctgca ggctaatgat gcacattttt aatctgacac aacgtttttg tgccagtgat
695941 ttctgcttct gtcttttttt tatcatgtga gagtcctctg tgtgctattt ccttttttgaa
696001 attcatgcag catgaatata aacccaccac tatttatgca gatatacatt atttagggct
696061 acgttgtgtt tttgggatgc gcccccccac tagcaatcta cagtttcatc agaaagaccc
696121 aacaagacgg aaaataacat ttgatttgaa tgtggcgtct aacattaata ctgcaaaaat
696181 cagcgaggcg agagaagaaa gattatttgg ggggggcagt tatcttccta cagtgccttc
696241 taatacaaga cttcacacca aacgtgtcgg tgaataaatt ttactcattc catgtttgaa
696301 taacggcagg attaaaatat aggaacatta aattaataga ccacagttca atatctgcgt
696361 gttgtcaatg gatgtaaaaa caccacttat aagtggatac atgcagcgtg atttgtcttt
696421 tatgttattc tcgctgtaat ctttacagac tcaattattc aaaggctcaa ttccatttca
696481 tgaagaattg gtgcgaaagc aaggcaacaa actctgataa aaaccccact tacattcttt
696541 agtttcatgg ctacatctgc tgcgtgataa tgacgtcatc tgctgaaaac cagcaaccgt
696601 tcaacagttt gaccactaga tggcggcggt gcacgcataa ataaaaaata caaagttggg
696661 cgggttcttt ctctgattta ttcccttgag agccttgcat attgatacgt ttcagtatat

696721 gtgtaaaact aaattgtttc tattatggta acgtcgctca ggacctcttg cacgaagatt
696781 tcagtgctat ttgtatcaat ttgctgtcac ttaactgtct cctgtcaggg aatggaaaag
696841 attcaccagc ccaaagcacc ccttgacatg gaaataaaaa catattgctt ctgatggctg
696901 cgggagcccc taggtttgca ccgctgacat ttcctctatg tgtgtgtgtt attgctttgg
696961 catttaggag gaaagctata cccatctatc tggcgtatac ctggacagaa ataaaatgga
697021 ttacagatta gaatcagtgc tcaggtcatt gacagcatca ctaatggcag aaaaccgttt
697081 gggtgagatt aatggtaaaa ctaagccagc tgtagcactg gggggaggcc aatttaaaat
697141 tcctttttat cgcacagaat aggctggaag aataaattca ctgattatgc atctgccatg
697201 ggaacgctca cgttaaatct tgatgcagtt tcatttccgg aagcttcagt gatatcagat
697261 ctgttgtgag gagcataaga tctttttatt gttgtctttt tattagctct tatcaacagc
697321 acatcagtag catttagcgt gccgacgcaa cacttttgct gcttcatctg tttatttttt
697381 ctataaagga atccaaaact ttaacttgaa cataaattgc agaagatcta caaaaagaaa
697441 aatttaggaa actcgataag tttgttttgt tactgcaacc tgccacaaga gggcaattaa
697501 ggtatattgt cctcacttca cacatctctc atgtccccccg tgtttctaaa gcttgctcta
697561 attgtgggct gcgagtgact gaatgcgcat ccgaatataa aaaagtagtg caaattcagt
697621 gcactgttag cccagaagca tcaagctata gcatcagaat gctaacatag agatttgact
697681 ttgttatctc cttcgttgag gactgaggac tgcaattgtg acaacacgcg gaccaatcat
697741 cttattaact tttactggtc agggagtcac tcctcagtct tcttagacca ctctggctgc
697801 ctcattcctc tctgcaccac cttcacattc tcctttgtat aaattcttaa agagcgaaca
697861 gcacttggga atgtttccac cttctgccta aagccaattt cccactgaca atattccgcc
697921 ctgcctaaat catccctgtg cagtttgtgg agaaaaaggg cagcagacag gggaaaaatg
697981 agaggtaatt agcaattttt aaaaaaaagt tgtttgatca ttttattgta aggtaaacat
698041 aaaaaaacct gcattggcac ctccattaaa agagctcatc cacccactcg actaatattt
698101 gcctggttgt tttaatggaa ttgattcaag ggaagagatt atttctctaa tttattgtgt
698161 gctgcttgca ctcaggcaca cctactgtat gcagtctaat gataaccagt ctatgtgttc
698221 tgctgtaata tctgttgttt gttgggggtc aggccctggg tttctgcaaa gtgccgagag
698281 acaattttgt ttgtaacaga cactatacac gtaaaaagaa ttgaatatag tgggcgtgct

698341 gcacagcatt aatgtaaaag ggctatgttt cttcttgact ctgctgtcat tttatcaaag
698401 gaaatcagct acggggtgga tggaactatg ctgcaaatgc tgtaaatata tataaagttc
698461 ttttcatttt aagtctttca catcctgttt gatggtcaag gatgagacag aaaacttgct
698521 ctgtctttac tggagcccaa ataagcaatt caaagagcaa aaaatacagt cacttgtttc
698581 tatgtactca gctgtggagc cgacgcctct gataaagttg cgtctttaaa cgccagatgt
698641 ttggaccatt taacatctgc ttgggttttt cttctctcat cctggcaagt caccagatgc
698701 tacctcggca accacaccac acctgtggag atgctttacg atcagacaaa ccagaccgtg
698761 ggagcgacaa attattcaga attagagtgt caagtgcatc tactgggatt gtactgatga
698821 aggaaccctt tagacattca actcgatgcc tcgctggaac aaactccact aaatgtaccg
698881 agtgtgactg attgatctgt ggcctgtttg tggcccacaa agcagatctc gtggtagccg
698941 gcgctcaata tctttgacct acatatccaa ctgatttcca acaccatcct tgtcaataaa
699001 acaagttcat tactgggaat cagagaggaa atactggtga tgtgctattg ctgtggttta
699061 atttctaaag ccaaaacacg gagaagctaa cgcctgctgt tggtttcact tttgtatacc
699121 caaataaaca cgtctcacac ggggctgtag gtcactttga tcagcacaca cacatgtcca
699181 tgggacatga tttacatgca tcaatgctta cagagacctc gaagggatta agttggtttg
699241 ttgacaaatg ggatcagagt cgaacttttt tacatgccat tgtgcactga gctgccgcgg
699301 tgaaagtgtg tcacactgca acagtttgag tgcaccgttg gtgggctgga caagtagggc
699361 agacgattga aagtgggcaa agccagcaga cagaaaacaa ttggaaatct gttctactcc
699421 ccctgacatg gttcacttgt gcttgtccat ttcggatgat ggtgtcaggc tcgtgtcggt
699481 gcaaaaagga tgcccaaata ccccacaaaa taggaagaaa aagctaattg atatggctac
699541 gctgcggcgc cagatgagat ccaggacagg gtcttctcga caggatcggg gttcctgttc
699601 ttccacagca tcttgcccag tgttctccgg accttacagt gaccacgaag aagacccacc
699661 gctaaggcga cagtcctctc ctttactcaa aggatctctt tccagtttac aaaactctgc
699721 tagcgaaagc cttaaaggat ctgtgtctag cctgcagggt tctgatccct gccttccagg
699781 atccctgcct agcctaagag gatctctggc cagctttaag gaacctttaa gcaacctgaa
699841 aggctcaatt tctcggctga agaggcggtc gctgggaagc ctggacaact cgagccccat
699901 tctgagtgaa aggagtatca gccccagcct acagagtcgc aacggcagct ccagtgatga

699961 cgataacagc tgggacacta acagctggag ttcaggggcc acctgcctac
gtcggactcc
700021 tgtgaagcaa gatccacctg aggaatcggg ggaatcaggt gaaaagtctt
acaataccaa
700081 ccaggagcct gataacagca tggatgaacc tgagatcatt taccaaaacc
ttatcttcac
700141 aaggcccgca gctaaaaccg acagtaaaga aggcctaaag aggacttttg
ttccactgaa
700201 aaaagatgca gaaacccaaa gttttgcctt atctagcctg agcaagaacc
cggcatcagc
700261 ctcatcgcaa caccgggaca agaacacgga gcacaggcgc aggttttcac
agtttctgaa
700321 tgaggtgagc ttcagggtgt tgaaaactaa caacggacct cctcaacaat
acatgacgct
700381 tcgacaccgt taccсttcgc ctccaccgcc accgccatca actccatccc
actcgtcgct
700441 aacaaccgcg ctgcattcgc cacccccaca ccgaccaccg gtaccagcag
caacaaattt
700501 gtggtacagt ccaacaaact ccaacctcca aaccatcaag gagtgtgcct
caaaggagat
700561 taacaactcc atccaccatt ggagcaaaac attacctagc tgtaagatct
tggagcctgg
700621 ggatgtgcta caaaaagtaa ccgtggaaag tctcgcacac ccagagcatg
acctggagtt
700681 atgtacaaag ctccaggccc agtcctccac cgggaggctt tatctagaga
cagacattga
700741 cagggtgcga caacttgatg agctggtatc taatggctcc ctggggaaag
agagtaataa
700801 gaaagaactg gaaaagcctg tggagagaga gaaagagagg gagaagagaa
ggggaaccct
700861 gtgggagaga gaccgaggag ctggaagggg gacgaaacac gaaaaaagga
gtgaaaagcc
700921 ctgctgggaa aaggacaaaa ggaaggaaag gggcagagag aaagagaaga
aagtagtcct
700981 gaacagttat gaacattctg ctcctcctct tccacctcct cctcaaatca
tctcgagcgg
701041 acagagtaac ccctgcccca ccttcagctg ggctgaggga ttccccagat
tgtcctacaa
701101 attcacctca ttaccccgcc ctgttaatgt gactgtgagt acaactgtga
ccacacgatc
701161 gtcaatgtaa ctgcagtatc gatcctgtta ggacagttgt ggaggtcagg
gtttacgtgt
701221 acaagatgtt ccaaggcgtt aattctgagt tgcactaaac tctaaactgc
tttcctctat
701281 cgctcctcct ttcaacactt ctgttggcaa cagcaaggtg taaacaacca
tctgcagaat
701341 ccactaaacc atcagacgcg cgtttggcat cgttactctc tccatatcgt
ttttgtgatg
701401 agaagtgaga atattgcgaa gtagacggta gagatgtggc tctggaaaca
ggccttggca
701461 acaaccttct gaagccaaaa agccagacgg cttctattta aaagtcaaag
gaacctttcg
701521 ctccaggaac ttaaacctac acgtggttta aagtggatcc agagcagatg
caggctgcag

701581 caaataacaa gcgtcagagg gtttcactca gtctttattc accattttga cggtgcattg
701641 catcatattt atcctgacaa ctctgaatag gtgctggatg cttcggttaa ttatccactt
701701 gtgagaacca aaacaaaatc agtttcaatt atttaccatt aatgcaatct gactggagag
701761 cctgaagggt ttaattcata gaaatatcaa cattcactca ctgacatgca ctcaaatcat
701821 agagaaccga catctgcgtt attacttttt cacattaaac atttctataa ccaggattaa
701881 cctcgtaaag agtggtattc tggtttattt gctacttgtt tgtgtggcag tatctttctt
701941 tcctttacaa catctgtgcc tatggcattt aggcttccag acaaccgtct ctacccctcc
702001 catttgtctg tgtgaaggta acaacaacat taatagttaa acgctttcct ggactacatt
702061 gacccgtacg ctgcaccatt ttacgaaact ttagcagcag accttggtta atgtggtagg
702121 gaaaaagcag gcagacgttt gagacacggt ttcatgatta gaatgagtag tctgggtggc
702181 agatcctggc aacagtttag ctgatgcgtg ggtgctgaaa ggagctcact gctgcatgag
702241 agctttgaac agaggggtaa ttatcgcaaa aacaggcaaa atgaaggtgt aaagatgtta
702301 gcattgatgg aacatatgat taaaggtcca ttgtctaaga cttttaacac tgcattatca
702361 gaaactgaac atatatgcgg acatgaatgt ggttcaataa ctctcaaatt ccatcactaa
702421 ctggtaaacg tcagctgcag caatgagcag ctggactgaa ggctgatgca gattgtaaaa
702481 atccgttttg ctaccctgac agccatcgta tgcgagtgaa tgttttcata tgcgtttgct
702541 atgaagtagg ttagattttg agggaactac cccgcttcag tagaaaaaca cattcccacc
702601 tcataacagg caacccggag aaaattcaca aagggagaaa tttctggact gtctctaaaa
702661 taaacccaca gtctctgaaa ggaaaagtac aggatgatca gctgttttcc tgtcccaagg
702721 cagtccatct tacaattaga gtcctattgt gagaaggcca tgacagaggc gagggacacc
702781 tcagggttat gaacaacgat acactaactt tccctaattg tgaactgatg tcagctgatg
702841 tgtgctggtc agatcagcat catttgttgg tgtgtttcct gcctcctctg aacaccaaaa
702901 cagcggtaat agggcatatt tttcactgaa tgaatctgct gttgctccag taggagataa
702961 ttcaccaaca ccctccagga ataccgatcc attgctccac ctcctccccc tccctgggct
703021 tgccctcccc tcccgggata gcagggatgg atggaggaga ttactgaacg ctgtctcatt
703081 cagcggttgt atgagtcatc ctgctgcatt actcgtgccg tttggaaccc cccccacccc
703141 aaccaaccac ccacacacac atacacacac atgcaacacc acagagccca gcacaacaaa

703201 cacaagctgc cctcaccacc tgtgtgctga agcttctatc ccgtctgctg tttgggacgt
703261 gtgggctcgg gtcaacatca gccgtcctcc tgcagcttta acgcatgcac aaaatcagca
703321 gaacaggtga gagctgggag tccatcacgt tgcacgccgg catctgtccg gcggatgcag
703381 ggagccgtcg gtcctgcacc aagaaatgaa gttaaatctc aaatggcaaa tgcttcatcg
703441 gatgcttgtt atgcataatt catgaggtcc tcgtgcccat tcatctcgtt tcacttcagg
703501 acatccaggg acaagtgaat ggagcgaggt gacactttga aactatattt gggtacttcc
703561 gctacttctt ttatttcagg cttgtgtgat gaatctcatg ttaatagtgg aagctaatta
703621 gcagtccgga aatggcagct gtaatgaaca ctgcaaacgc tctcccaaaa agatttaaag
703681 cttacaggtg cagttaaagt ttaattttgt taactctaat cagtggcaca ctcactcctt
703741 ccccccttgac gctggtgtaa acccaatttt agaacctctt atcggacgta taacatagca
703801 aaagtaaata ttttcagtga ttgtgggtta tttttatctt ggctgctccc aggcaccatt
703861 tctttcatct cttttgtacc ccgccttctt catttctctc agtatctccc tctctcatca
703921 cttcttttct ctcagtgata tgtatgggtg gttgtaaaat aatatgttta tttgctgaga
703981 cggagagctt gttaaacacc agataagagc agctccatga agtgctgtgt cagccgctcc
704041 agataaatct gcttgaaaaa tgaatctact gtaatcattc tacactcttt tttttctccc
704101 aacaggattt cgacggggag atgcaacatg acgcgaggtt agcctcttcc actctcacac
704161 cctctctctg cctcattctg ccactttttc catcactccc ataatccctc ctttctccct
704221 caaacacctc ctcctgctcc attcagttct ctgctccctc tctctctccc tctctctctg
704281 cacgctccct cgctctgccg accagcctgt ctccagtcag aattagagga ggcttttaaa
704341 tgcatgctgc tttgaggggg gatgcacagc cacttgaatg ttgtgagctc atctgcagaa
704401 aatgaggcga aaggcattta aacggtaaga ctgtggggca ggtggtggct ttcatttgct
704461 gttatttatt tctactgctt ggtcacactt agacatacag agacatgtga gatgatgctg
704521 tcagtgctct tctgtagtgt tccatgtatt tctgcccatg tgttgcatcg cgtgaagcct
704581 cggtgagttg acagctgcct cttagctacg cgcttggaga atacaagtgg tgctttctgg
704641 catagtaaaa ttgcagcaga gcgaggttta gtgaaattaa accagtcttc aggggtattt
704701 tttccatttg tcattgtcat tcactgagaa gttggacaga cagaattgag tctggataga
704761 gggtttatat tgatattgga ccaagaagga cgcctccatg tttgcctttg agtcccagaa

704821 gtaaatcagg aagttttcca gcataggttg cctttagggt catccacagt cgtcgcatca
704881 gcgtatcatg accgcatcat cgagaagtga gttgctcttt tctccagggt taacctgcag
704941 ccagtgtgga cacattgatt tctgcaaatg atcgcatctt cttgttacat aattaaaacc
705001 aacagctcct cagataaggg gccttttaga ggggaaactt aacactttga agtgagtttg
705061 aaactcgccg ctcaatgagc tttaattgat ctgagggaat aaatacaact gattttgcct
705121 tttgccacag cgatgttgtg tttttaatgt ggctgtatgt atcattccct cagggtgaaa
705181 cggggtgctg cactcagcac aggtgatatt aatttaaaaa tgctaaacac aatggaccag
705241 aattaatata aaatcgcttt agtaacaaaa taccaccaaa aaacttaagc aaatgtgacc
705301 ttgctttatc gctttttctt gagatacaaa tctatatcaa tggaacccct gaatgttatc
705361 cacctgcaac ccaaattaaa cctgcattta actttctatt taatttagat tgtctttcac
705421 tgaaatacta gaaaatcgaa atctttatgc aagaagagcc ttgaaaggaa agaaatcggg
705481 agtgtgcgca ggatatatgg acctgaataa actttttttag aagcactttt ttagaagttt
705541 gaaaaacatc cagggtttgt ttgaccactt taaacacgca gccagagcag cagagcttta
705601 atttgtgtga cccaagtgca cagagcgaat gtacgggttt attaatgagc gcgaaaacag
705661 ccgtactctc ttcatttcac tctctgcgac ttttaattag ctctctacaa atgtttcagc
705721 ttacctccct cagctctacg ctgtgatcct gcagcagcct gggggataaa acactcaccc
705781 actgagagtt acccggtctt tggggaatat ccccaaagag gctttgtcag ttcatcttaa
705841 gctgtgcacg tatatcatcc aaaagcacat gcagcactgt aaggaggttt gcttcaggtt
705901 agcctggagc agattgccac ccctgtgcgc tctcatcctc ctgcctaatt atatttgaga
705961 ttctgaatca tgcagaagcc agttgtttag aaggatcccg tagccacttg aagtccttca
706021 tggggaaacg cctggcagcg tatacaacaa tttgatttgc acgtgcatac atcatttctg
706081 attctaaatc agacccctgg agccttgaga ccactgtctc cttgtccaat ctctcagcac
706141 ctcaggacca taaatggcac acctgtccat cacccagcac atacaataca tggttctgtc
706201 aaggccaact agacaggcca agcagcttaa gcccgtgctg tagacccatg gggtccattt
706261 actgcatgca aaactatcaa cgtggtgttt gaatgccaca cacagaccac ggagttttag
706321 ctcgtgactc agttgtgttt ctattggcca caataaatta ttttcagggt ttttttgata
706381 aagccgcttc ctcatattag tctatttacc aacatgtctg ttttgtgtta agaggcagag

706441 gcctcgtcta aatacacagc aatcttacag actgcaacaa actctagtta ccgcactcat
706501 tccatcttag taaatacttg attgtgcaaa ggaaaggccg tgcacgctac cctatcttca
706561 gcctacacat gcaaagggct cacccacctg tcctgaggct taatgcatgg tggatcttca
706621 gatgctcaat ttgcgctgtg atgataacga gaaaaaggct caaaggggaa aagtgaagga
706681 gtctgtcttc aaaggtgaaa tttagttgtc atgctctttg tagctctttg tactccggcg
706741 cggtcgggct gagctactgt acagcctgtc cgcgctttaa aaggagcagg tctctttcat
706801 ttggattcaa tagcttgcag taacacaaag tgaaaaacaa ttcagatatt gatccaaacc
706861 ctgcaatgtt ggcaaaatta tacacaaggt agccctgtac catccctttc tgcatcacat
706921 ttgctgatat tttacaataa ttgcccccac tacatgtaat tttacccaac agaatttccc
706981 tcatgattaa tttatgatga cctcacacat aatgctggcc tctaacctgg accctgacct
707041 ttctccacac aaacacactc actagcttcc actgtattaa taacctcttt ccaccacggt
707101 tgcttagata aagacataga gcatatgcac atatagataa agatcagctc tcaggagatc
707161 aacaccagat ttaacatgat catttccaat ctggaagaaa aagtaatcac gcttattctc
707221 tctcattaaa taagaatata catagactct caacaagcaa aaaccactgg gagactaaat
707281 caggccgaga gtataaaaga taacgtacat cggcgtgtct gtttcttttc acttgatatt
707341 cttacatcca gcaggagggt cgcccacatg accctggccc tgctcaaggt ttcttcctgg
707401 tgaaaggaag gctttttta gccaccattt catgactgga ggtcaggctc tagatttctc
707461 taaagcgcca ggagacaatt ctgattgtaa cagatgctaa ataactaatg tttgaatgta
707521 attgagctga attcaaatgc caagttgcca gggtgacata gtaatatgag aacacatatt
707581 gaatttcaca atatatctgt agctttctat ttgttatcta tcgtcattag tttagctggt
707641 ttataatcat aatccaattt gaaatgatat tgattatcca tccatgttgt gatgttatga
707701 gtaaagctac ttaagaaatc cataacccag aatgccatag taaggaggca tatgtgcagg
707761 agccccgttt tagttcaggg aatcatatac agcaattagc acaaggttat ctatgtgcaa
707821 actctgcaat aaaatataaa aatccagcaa acacgtattg tatacacgtt gcaataactt
707881 gaaagaaaac acacatgtct catggtggag atttcatgtg tgtgtaaata tgtattttag
707941 cctgtttcaa aggttgtgca gagatgaaaa agaagccata atgtcgttta tgtcagaata
708001 gatgttctgc tctgagtcct ttagaccaga cagcaggtcc attttaaagg gacttgtcaa

708061 gtaaatggat ggatttttga ctgctagtgt gaaaagagct tgtttggagc tgtcagtaaa
708121 tttgagtttt gtcattgttt cgtaggattt taatgtataa ggacagaaaa gtgggggaaa
708181 aagtatttcc agaatacaac taaaatggtc ttttttcaaa cttgcagaaa cagaattctt
708241 tactcaactc agtgaattaa aaaggaaaca ctgatttgga gccacgtctc aggggcataa
708301 aaaacatccc aaaaaagatt cctggtgggt ttagttttag atttggatag caattgttct
708361 tggactttaa ttgccgaact gtcattgatg gaataaaaga acagaagcaa gacaaagtca
708421 gtgtttctgc ctccggcaaa tctcatggtg tcacagtgta tgagttggat ttcctcacca
708481 aaaagaatga aaaaacctga gcccatccca gaacacgctg gagtttatta aaccacacca
708541 tccacaaagt cgcatttgac atgcaaactc agcagatttc ccttgaataa atgcagtgtt
708601 tccactgcac acttgtctcc ggccattaca gacctgttag ccccgtgtta cgtgcttgtc
708661 ggtgccaggt gctaatgcag tcggcccaaa ggtgcgctgc ataaagagct ttcgattgga
708721 ctgtgagtag ctgtgagctg ccagtgctgc tgggaagtca gcctacgttg acaacagtgg
708781 gtcaccgaca aggcagcgat cgacttgaaa tgtaaagtgc gtcctaaagc ttccccggtg
708841 ttttagctcg gcccgttacc gcgtgacatc gagataaaat gggttttatg acacttatct
708901 taaacgcagt cagaagtgga acggcggctc gctcgctgtc aactttaatt ggtgcaagaa
708961 ctcacatcat gttttatgtt ttgaatggaa tctggtgtgt gctttacatc gacgatactg
709021 tttattcatt cctgactaat gtgctgtgat tgattctgac ggggccggtc tgcttctgag
709081 ccgtgaatta tttaaatcaa gctatcttca aactgcattt aaacacctgt agcagccgtc
709141 tcattacatt aaggacttca ctgtcactga tacgctcagc atgtgtaatg taagcgcgtc
709201 ctcccctgtg ggagttctat gccagaccca ctgatgtgat ctcgtgtgat caggactggt
709261 gcaaattatt tctacagagt ctcaaagccg agtgaacagc gatgtttccg atgctaaata
709321 tctaagcaaa tgcggccact tttccgccgg tgctaacaaa atcgcactga tatgagtgac
709381 gtgccattta aaacacctag aatacataaa ggaaccggag ggattcatag cgcagcagga
709441 ggaagctact cattatcagt taatttatcc ccaggagctt ctctttgtga aaacagaaga
709501 aagccgggca ggaaattcaa gaaccagaag ctctgtttca actgaacaaa ttcccgatta
709561 cagctccacg tgcgacgctc tcataatatt tgattcgcaa aataaaaccc caacaggttt
709621 caaaggtctg ctttagtttg aggataaaga gaaagttctc ccagcttctg atatgtaaat

709681 ggaaaatccg gcgagtgata aacagcacag cgcggttgca gagtttttct
cggggctgca
709741 gaatgtgtcg actcgtcacc atcggtgctc tggtggttta atccacagag
ctgttcttct
709801 ggccgccggt actggagcgt gcacaggaga ccgctgtgtt tctggagtct
tgcgtcagaa
709861 gaaacacgag ctgcaaccca agtgggagag atcttgccag agaaaactac
tttcactagg
709921 aaagattgtt gtggagtgat cgctgctcgc gtttggtgtc acagctgcgg
ttaccggcgt
709981 tcactgaagg caccattgca gcgcctgcat ccaggccaag tttcatggtc
tgaaccagat
710041 gagtcatctg tccaagtcac catttgttag cacgggatcc tcattattag
caccattacg
710101 gacccacata ctggttttgt gggccggagc aacaggttat tttaatttgt
ttagacttct
710161 tgcattgcca gggtaatggc aacaaacgtg acggcggcta aaacaataga
agcagcccct
710221 ccagaccaat gaaggcgtga tggtttgttc ctcggcaaaa tgcacttaaa
tcacaggagg
710281 gatgtgctgc ggccgttaga gcttaatatc ctctttttgt tactgttaat
cgctctacct
710341 tgctcacctc tcgcggctca ttaccagtaa atcaacctgc aggcaccata
aaagctcccc
710401 atatagattc aagataaatg cttggtagtt gtgaccttta gttggtttta
cattaggcct
710461 ctaatctaaa gtacggtggc tgttggagcc attagcggga aatgactggg
gggcagattt
710521 aaagatggaa actgcaggga caaccatctg ctgtttgcat tgtctgatca
ttatgagatg
710581 atgcccctcg aggaattatt tagttgatca ctgtcagaga gcactccgag
gaggaaagtg
710641 tgcttgattt atatatcttg gctattttga gattccctgg cagcagcgag
catttcacaa
710701 tattctccga ggacttgaga actgaggata cttccttcct tcatgtggat
ggtgtttact
710761 ttgccaaaca acacctaagg caccaagatc tgttgtttgt tcccccccgg
ggggggtgga
710821 atgatgcagt gagggtagta gcacttcatt tgctctgcag ggaagtttgt
gtttgtgtga
710881 attcattcta taacgcgtgc atttccgaac caattcagaa actatttctg
ccgccttttg
710941 tcacttcctc gctcccactg ttacgcgctg ataagaaggt ggcggcggcc
gtagtttcca
711001 gaaaccgcca ttgttttctc aaccactgtt gagggagatt aaaaataata
agtagtgtcg
711061 ctattagaaa tgtcgccggg caacaaagga ttcattctag ttagttgctg
aactgctagc
711121 ctggtaatct gctgcaccca ataaaagcct tggaaacctc tacttcattt
atgctacaaa
711181 taaattctaa attgggcatt aaaggtgtca catgatgttg tgtgaggtct
tcattatagt
711241 tacagggaaa actgcagtac atgcagctgc ttacattcac aaaaaaatgc
actgcctctc

711301 cagtggaggg cttttagaag aattaaactg caggcaaaca ttcattaatt
aaacatgcga
711361 tgttatgagg tcaacgatat acatatggac tgtagtgagt acagtagaac
ctaattggga
711421 agatggacga gcgctcactg gctgcagatt tatgcttgtg cataggcttt
acgctgagcc
711481 atatgttgtc gctgatgcag cacgtcctgg taaaagcctt tcccgccgct
atgtccgtgc
711541 accctgactt taggggtgac ctgaacaagc caaccaatca gagaggtcaa
tgttgccgcc
711601 cccataatta catttttgac attagcccca gggtgggttg ggagagttgc
aggtgcgtca
711661 tggccgcagt ttaggttata agttagctgg caaagcaaag ctaattacta
tgattgcccc
711721 ctggtggtgg tctaggtcta gacctttggc actcagatgg gatcatactt
ggtttgatct
711781 aaaagtgtaa gcagctcata gactaaagtc tagattctat tgtaaatatc
agctgatatt
711841 tgcaccgaaa cacaaccttc cgagtgttga aatatgtttg tcaagaagca
gagaaagcta
711901 agtatgacag actgggctag cggctgtgat tatagcaaat actcagttgt
ttgttttctt
711961 aatagcccat ccatttttca ggatcaccca gtttctcggt ttatgtcact
tcgctctgat
712021 tgttacctaa tggccacttg atatcaacca ccgtaatgtg gctgcacgag
caaagacgta
712081 aaaatggaaa ggtttattct gaaatttcat cttcgctggt ggcaggttat
taacttctta
712141 cagcgcacga gacgtgagtt cagtccgccg gactgtagag caacgatgaa
ggatggaaat
712201 ggaaacacac acatttatca taacactgat ttattgaggc ataacctctt
tggtcagacg
712261 gtcaatagct ggctattcca caggggcccg gcactttatg gtggctacat
tcagttgccc
712321 ccaacaagca ggaggaatgt ttgatggaaa ataccgtttg aatgaaatga
aattcatctt
712381 tatacagcca gagttgatgg caggggttta tttgaatcac tcataggggc
acacagttac
712441 aaatgtacat atttcaggtt acagaattac agcaaagaca tttctagctc
cttgtttctc
712501 tgtttttccg cttctgccgc ttggacaccg tgatcagcac ttgctaaatc
agtgggactc
712561 tttttctctg ctctcttgcc agggtcatca cagatagcca actcctatct
gtctccccag
712621 gcaagactta acgctttgac tgtgggtttg tggttcgagt tcagactcgg
cttccacgtt
712681 cagccgaccg gcagcagcaa ctgtagacac atgtgagcca aggcagagag
aaaaatatga
712741 aaaagatcta tattggcaaa actgccctca aaacacagag aaacggttgc
aaacacccca
712801 aaagaaggta agaaaaggag ggtctttgca catttgtgtc agcctcatta
cgggaggatc
712861 agtttcatac atattaatac atgggaatga aatctctcca ggatttcaat
tccattaaaa

712921 ccccattaaa acatccattc attcccattt atcatggtca catttctgac cacgcatcag
712981 cagacagcgc tgcattttac caacatctga atgctgagag aacattggaa aactttgaac
713041 actaatcact ggctgtaaaa tcaaatttaa cgttaaataa taatgaaccc aacacagatt
713101 tcacgcagct tactgggcaa gacacagaaa gactggtcag attttgtcac aatggggcaa
713161 cactgaaaag aaatctggtt gaactgccca agactaaagc ggaaattcga accttcatta
713221 cacacgtgga cgctgcgaga ttagcaacat gccacttgag aacatataga acatactgct
713281 gataagaact gcttatacca aagctttaac atagaacttg cttgatgaag gtttaatctt
713341 tggagatctt tagccaagtc tccagatgtt aaaggtagtc tgttgccctg gctgtgttaa
713401 agcagcgcta tctttaagcc tttctgaggg tgccacctct tttcctcttc cttaactgcc
713461 agttccctaa ttgaagctct cagccatgtg aactcggagc tggactcatt ccgcctgctg
713521 gttggcagcg cggagccatc gaatctctcc gagagccaag catccatcag tctggcgaga
713581 tgctaaatcc cccggagcgc acgctgcccc tgctcctcat ttattaccgc atccattccc
713641 tcggtttgtg ctgtcactgt gttttcatct tccgcactcg cagcagccca gaaactgctc
713701 tgcattgggc caagtcgctg ccgttgggtg gtgctgaagg aattctcacg ttcttttagc
713761 ctctttcaaa gcacaacgtc acggctcatt ctatattttc agctctattt ttcccgtgga
713821 ggagggcaat catttcctta tgctttcagg gattcagtat gaatgatgga atttaccatc
713881 acattgatgg gagctacttt cccatcaatg cttgaatccc tgtgaatgga aaatgattag
713941 tattcctatt ccatctgtct gctcttccat cagacacgcc acctcccacc ctgcctgact
714001 tgtctcactt cttacacctc acccttcctc ctcctcccac acataaacac atatacaatc
714061 cttgctttct tctgcctttc catgaatcag agaaactttg acattgtgtc tgaccaggtc
714121 tcctcaaatc ttgttttctg aaccctcccc ctctccgcac gttcagctct ttccacgacc
714181 acaaggatga cctccgtaag cgcctctcct acaccaccca caagctagag atggtggaga
714241 tggagttcga ttccactcgg caatacctgg agacagagct gcgccgggcc caggaggaac
714301 tggagaaatt cacagagaaa ctacgcaggt gtgtcacgcg ccactcgaca cctgcaaatc
714361 ttttttgcct cgactcgcac aaatgcaaca ttaccctgtg gtgatcacgt agtggtgtga
714421 atcatctgca gctgaggtga ctctggggtt aactcagtct tacaaagctg tatgtgcttc
714481 ttactagggg catgttgcca tggtaacata ggttatctgt ccaggaggag gttaaaatag

714541 gagaaaaaag aatttctctt tctgatcttt cattcttggg caatcaatat gacgtcctgt
714601 acatctgctt ttaggtctct ttttcagcct tcactcctcc atcgattggg ttaaaggggc
714661 cataaatgca ctcttaccaa tgcgcgcaca atgctaatac aaaatctgat tctgaaagga
714721 aacacgagca gcaggtaaac aaatagtccc cctgaacctc actaatgcac ccaaagatcc
714781 atcaacgagc agcactgaca ggatgaagtg aagggtatga ggaaataaaa caaaagacag
714841 acaggagata taaatcaata aactgcctta tgttagtatt cagctcagac tcgaggcgct
714901 gggtctgcgt ttgcatggtt acttgtgttt tatcggcttt caaggtttat tttccttgga
714961 acaactcgct tgggctgatt gaatgtatca ccgactcatt ttgatgcttt caaccagcct
715021 ttttcccaca aatctgacga ttctccacca gcactccaag ctcagagttc gctacctctc
715081 gtctacatgc acgtggagac ggccccgctg cacgtcagac accatcaaat gttcgctcac
715141 gctgatctgt atgttgtgca ggatccagag cagttatgcg gctcttcaga ggatcaacca
715201 ggatctggag gacaaaatgc acagaacggt aagaagcaag caacatttca gcctaaagac
715261 aaaggacttg ttttgataac cttcacatga attatgaata atgggggggg ggtgcaaaat
715321 cctgatagcg tctgcagggt tgaagagtct ttctataaag atgcgtgaag gtcgacaggg
715381 tttccgaaat gctcgtgctc gtacacatat gcacacacac acacacacac acacacacac
715441 tcggggtgtc ccgcggtcga aggagtaatg aaggttatga tccaaattaa acagatgtgg
715501 gagaagaccc gaacggatgc gacagccaga agtcagagca gatgaatttg aagtgcttct
715561 gatgatattc tcttgaatct ctaaaacctc ttcaacataa gcgctccgga ggttctcctg
715621 agactcgtgt cacaagttat cttcacattt gcctgctcgc acatgcagcg cgcagcaggg
715681 aagcgcgggc gtgggcagag cagtgtcaac caaatctgca gccgttcctc agaactattt
715741 cactgccaga ggagctcgag aatacagatt attataaaag cattaattat ccaaatgttt
715801 taaagaaaaa acacatttgt ggctcagcat caaaaactag ttttgaacat aattttaagg
715861 gggcggaatt gcatatcaat aaactcatta atgaataggc aagccgtctg aagcgtttct
715921 aatgcagcat taatcctaaa tgagctgatg ttgttatgga cgacaaaaaa gccttaaaaa
715981 gaattgttcc aagttaacgt agaatgacat cacgtctcag gagatgacgc gggagcgatg
716041 gcagcattta cactgctccc actgtgtcgt gtctggacag aagaattcta ctgtaatcga
716101 tgcgaaggcg cctgttcagc ggcacaactg ctctcatctc tctcaaagca attacgtgat

716161 caaagacagc cagagcaatc aatgtccaca gtgattggtc ttacctgcca cggatcccag
716221 ttagaaaacc attatagctg ctggagtcac ctcggcggca gctgtccagt cgcttcagtc
716281 tgaatagtgg ttttctccct cgcatccttc agaaccttca tgattcatca gggaatcagg
716341 cagtgtatca taacagcatc agaggtttgg tctgaaaaca cccaaagtag ctgcaatatt
716401 ggacaaaaag tccatattga cccgtgtcag actgaatatt gagaaatcgc catcgctcca
716461 ctcgaactcc tttaaagttc tcacatgatg gttccgtcta caaggtcacc atcgtggttt
716521 aacagacgag tgactcaatc ggattcaagc acagttcaaa aatggtcgag aaacctttgg
716581 atttgtgctg ctgtgctcag gaattagatc tctcgcaaac ttttattcga tttcctacca
716641 accctgcgtg gctacagatg acacgttgca gtcaacaact tagattatct gggcgaatcg
716701 ttccgttgtc ttgtgtgtgc gactgggcca catctcggta gccctggatt accgacaccc
716761 actgacatct gtacatctgt tgggcctgct tacggcgggt caagccatca taccccagcg
716821 ggtcgctcct cttctacagt cacacccatc acataaaaaa aagaatgtca tttttaagga
716881 tttgtctgtc gagtggaggg cttcggtgta acaattatgt ctccctgaca ggagacattt
716941 ttgtcccatt ggaattgccg tttcttttga tgtcatgtca tgccaagaga tttcaagagg
717001 gaggtgtgtt tcttttgtaa aagacagttc aggcaggcgg caaccgaggt ggcgccaatg
717061 gaaacagaca cgtaattaaa agttcggagc ggcccgatct gacacggtca agtggaaagc
717121 gtcggcgcat aaaactactt cataaagagc caggaaagag cagctggaag gactggcaga
717181 tgcacattgt gatgacagtg gagtaattgt gctcggtacg cacaccagaa acggaagatt
717241 gctttaatat gcgcaccgcc tgcaattgaa agtgtttcag cggatgagat tacacatcgt
717301 ccctctgtag aatgtggaaa tttacaggag aagatgtgtt aaaaacattt ttaagaccaa
717361 acctaacttg acaccgctgg caggtttaag gatattgtat aggtattttc cttatatata
717421 gtccatagtt ttaaaggagc tgtctatggt gctgaacgtc tgtcgtcagc gttcctcagg
717481 gtcgattttc agccaccctg aaactgtctt gaagtcaaaa agctttactc aaatgccccc
717541 cctcactagg tgaatcttac ctctaagtgc tagacctttt gctgatcttg gatgtgtccc
717601 agctggcaaa tgtaaagaaa cacaatcttt gttaatgtaa cacgatcagg ggggaacaca
717661 aagctgcaaa tccagaacac tttaagatac attttgactg ataatcctaa ttacctttca
717721 tatgtacatg atagtttgct gaaaggtttg atcaatagaa tgatcgatga cgaaagcttc

717781 tgtcccaacg gctgcgtttt tgtctcctgt gtcctaaatc catatatatt cattatcatc
717841 tgtcacggcc attcagagtt atttatgtgt ttattaatga aacacaggct cagactaatc
717901 tctggggtct ctaatcacct ccttgttggc aggatagttt agcatgactg atgcaatcaa
717961 attgtttatt gcaaagagag gaaacaatgg acactgatgt cagtatggag agaatgaaca
718021 ttagcgctaa tcaatgtgtt tgttttagtc gcctgcggtt gatcactcaa aactgagagc
718081 aattattttg tttcgagcgc tcagattccc cccaaagaac atgcaaacag caacagcctg
718141 gcttcgccta tgcttgaaaa tgctcagtag gaacaacaat tgttttgctt ctgctcatat
718201 ttgcctggca aatccatttt gatcatccta aaagttgatt tgatgaaaga ctttcaatat
718261 gatatttctt ttgaaacctg ccttttttggt acatttttgc agctgatccg cagttctagt
718321 ggaggtttag tcttcagtca taaacaggct tgaggatagc tcagacagga catgagtggg
718381 aggcgagcga cctcacatat acagctttat taaacacgcg gctttaaaaa gagaacaaaa
718441 aaacaggaaa aaccctttac tccactctca cttctttctt ttgtccctcg gctgaaaaat
718501 aagatgttat ttcaaccgga gcaaggtctg ttgcaccggt ctccggtaat agtgcccgtc
718561 gtgggatttg cgctttgttg gttggtcggc aaacataact aaaggaggta agagaacatt
718621 cagctaaaat ccttcctctg gacagaaaac taggctagtc acaggaaaat gacagtttgc
718681 acactgtaca tttaaacggc tcaaataagc tcagatgatt gggtctggtt tatttggtcc
718741 tttacttccg actgtcagac cagtagattt ttcgttacta ctagaatcca catattttat
718801 ttaaggatga tgaggaggag gccagctggc agctcctgac ctgcactgtg ctgaaaaata
718861 taatccagtg gtccaacata caatattgag cactattggt accgagcaat ggtgacagct
718921 tgtacagtaa acacccaact gatcaccgtg cacagccgtg acctgctgac ctcaaccttt
718981 gaatttagag ctatgatcct gtgtcccaca ctgcaaacat aataatctat cagatgacac
719041 cacacccccc tctatcattt atctacctcc aatgtacaag tgagactaat gttagtagaa
719101 tctgtcaaca ctgagggtga gcaaagatat ttatttagaa gtaacaagta agatttgggc
719161 tagattgcaa caggctagca cttcagatgg caaaactgac agaacctgtt gctaaaaaac
719221 tgaatttcat accttcatgt ggtataaaag tttcacaaaa cctttttgaa aaaccttatt
719281 tttagttttg accttgactc ctacaattaa tgaacatgga tgacgtttaa aaagggccaa
719341 gctcctctga aatgtaaact aaaaaagctg aaaatcaaca catttcccaa gaattagagt

719401 agcagacacc aaacctgcgc tatgaaacac taatagttct actgcacatt catcaatgtg
719461 actgtatgat tgctgttatt atttagattg ttttaatgtg gaactattgc atatccaggt
719521 ttaaaagggc tctaatttgt tcaccaaagg catctgcatt tttgaaatat taacagaaat
719581 ccggagtaat gagaaagtct gatccggaca gcagattaat gagaccctga cgaaattgaa
719641 tttcgatatc aactgtggcc ccagtgctta agctgacatt aattgccacc tattagattg
719701 gctgtgcacc cctgtatttc tgaacaatca ccagcattga gagattaaaa gggagaaggg
719761 tgggaggcca agtgagtgct cagagtgtat catttgtcag ctggtgcggt gtgacagatt
719821 tgcaattact tttgtgcgtg tgtttctgtc tgtgtgtgtg tgtgtgtgtg tgtgtgtgtg
719881 tgtgtgtgtg tgtgtgtgtc catgcatggt agagaaaagc caacaaagag aggagcaata
719941 ttagaatact gacaacacaa cagcggcgtc gatatccaat gaagacgccg gggctatgaa
720001 atgaagcggt agcatcggcg caacagtcgg gttcctgtat tctcatcaca cttgcgcaca
720061 cagatgtgtt taatgaaacg ccacgggaac gtgataagaa acagcacaga acacatggag
720121 aacagagtgg tttaatcctc acgtccccat tcccaactgg tcaaataatg aagattgatt
720181 agactggaaa gcagttattt tgattttctt ttttcttttt ttgacagcgt tcgctgagga
720241 tagattgggt ctgtctttgc ccgctattat ttttcctcct gtttttagct cccagagtaa
720301 ttgatatgat atgaatgctg gcaactttaa tcttctttta attaagacct gctgttggca
720361 ctggctgcat actgacggca gcccgggcca ctcgtgctct caattttcct cctctccctg
720421 tgtcatcttg ctctcttccc tccaactttg cctccctcat attcctctct acgttttcct
720481 ataacccttt acaagattgt cctcttttct gtatgacagt cccaccacca tgaagaggaa
720541 aagagagccc tccggggaga aatcatcgtc ctcaacaacc acctcatgga ggcgaagatc
720601 accatcaata agctcagaga ggacaacgtg agtgtccaca actgcctggt ctggtacaga
720661 ctgccacaaa tctgaacttt cacagtgtga ggagacatcc tcggatgctt tcagaccagt
720721 aaattaaaga ttagatattc tgggtgtgag aggagtctcg gggttgctgc tcccactcct
720781 tcaactgttc aaatttttag attctggttt gtttaagcgc ttcggtaaac caccgcccaa
720841 aaagtcctgg atatctgggc gccagtggga cacggcagag ttcatgctca gccaccgggt
720901 attttaggac gattataccg aagtgcacag gcaacctcag gggcttctga aatttcgggg
720961 ccagggctgt gtggagcagg gggaaattat gcaatgaaat gtctcctttt tctttgaagt

721021 gatttcactg atttgtctgc aggggagcct ccgggatggg ttcgattttt ctctctttgg
721081 tctgtggcag gttctatcac tgataagcta aagagcattt tgccaatcct ctcagatttg
721141 ggcgagcaga tcggactcag ggttctttta ggaggggtg gagatttggt ccctgcacag
721201 agagcagcca ggcacggacg tccttccaag aatcaaagct tctaatacat gctgaaatat
721261 gaatcccttt tatataaaat gagcagagtc cagaataaag acaaaacagc tatttaaaga
721321 acttttgagt cataaatatg gaattttcat gtaaatcaac ataaagagca aagtctcttt
721381 cagtatttat acaataaatt tactccataa ccttcagcac atactaacat aacacagggc
721441 taaagggttt agtcaaacta cggggtgccc acataaccct tggcaacaaa gaatctttta
721501 aagctttcgc tggtaactgg tgggggggtg ggggccatca aagaaaaatc ctttgatgct
721561 gtgcgaagca ttgtcagggt ttgtgagggg aggatgcggc gcagccacga ctgaccttta
721621 tgcgcagaat gattgcacac aggcaggagg tgagaacggg agcttaacca tcacacacgc
721681 cttgagaaac aaacgtgcca gtagaatacc ctcactctgc cctgaagttt aagtaataac
721741 cgaaacacaa ttcatcatag agaggccaga aagagagcgg ataaataatg cacacggtcc
721801 ccaggctgga aggtgcagat aactccatgc cgcattcatg cactcgaata aaattaggcc
721861 gttctgagaa gataaaaagc tgtattacaa atgaaaaatg tgtcaactct gtcaaaatga
721921 aaaatgcatg ctaactaatt aaaacctaga tgagctttaa tagatgaatt tctttcttag
721981 atctgatggg tcactaaatc agaagcaaaa ggcaaatttt ccagtagact gcagcgaata
722041 gaataaatgc cctttaatgt ataattcatg ttattaaatg tttaagacag tttgttactg
722101 tgtgaacaat aactgcttta taaatgccat attttacatt tgtgctatat tgcaacatta
722161 cataacttct cttcttaccg attctcaaaa taatgtaaat atataacaaa gatgaaactg
722221 tgttgaggct gctgcttatt gtgattcctg ctttttccag gacctttaca ggaaagactg
722281 caacctggca gcacaactgc tgcagtgctc caaatctcac tacagagcac acaagatgtc
722341 tgaggtaagc gctgaaatac tactgacctg cccactgtga cctttactca ttcagcagaa
722401 cgtgcacagt gaagactgga ggagtactgt tagacaggtc atataaaaga gccaaaaata
722461 ctccatggat tatacttaat agagtttgaa tacttaaaaa tttaaaccct gtcaataact
722521 gagcaaaccg ctacaaaatg ttgcatcaaa cgcaacaaac gcttctctgt tttttattca
722581 ccagtccaat gttagctgca tacactactt gccaccatgg gctgtggtta attaactctg

722641 cccagcagca cgtgaacaat gctgcgcttt ccttggctct tgaactaact ggcttcagct
722701 tcattctgct gctgttatct aatcgaggcc tgaaagactg cacaattaca gaacgttaaa
722761 tccgcccttt ttataattgg aaactgtata tgtgctgggc acaaaagcaa agctgaacat
722821 cctctgtggg gagactcgac atgtgagagg aggcacatta ctaacagagc cttaattaaa
722881 atattgaaag aaaaaaaaaa cccaggaaaa tccactctaa atgtggaacc ttggtggaaa
722941 agtagctttt agagagcaga tagaaattgc tggaaagact aaaataatag gtgctagttc
723001 cttcctgcag cagtaaacta cttattctaa taatatgagc aagatctttt taccgtcatg
723061 gaataaagtg gggaaaaagt gtgtctgtta attaagattc gcctagatat atgccactac
723121 tgagctagct tactcacagc acagtgtgta cattatcttg ggccccccac ctcatcctca
723181 cctactttcc atcactattc cccctgggag aggcagtaga cgctaaaaat agacctactt
723241 taaaaatgca ggttatttta tgccgaggca agcttctatt tcttaacaaa gccaagtcgg
723301 tatttttatc tttttttcta tccccagctg gaagcccagg gcggcggttt aatttgaatc
723361 taagccgtcc cctgtggaac tgatatgtgt ttgatgatag acagaataaa gaggctgcga
723421 gttaaaagac agagggcgag ggaatgagag atttttttc agtgcaaagg cacaacagaa
723481 agatgaaaca aagaagaaag agaggagaaa agggagaact ctgagcagaa agaacagttc
723541 ctgctgaccc tgaccgcttt taataggcac aaagttcctg tctgaaagta acggggaaca
723601 gtcatattag gagctgacaa ttctctgtgt gatgggatga tagaatgaga ccaagagaaa
723661 aaaaagccaa tgtcgtctac ccaatcaaac ttcctttttt actacctcct caagagattt
723721 taaaaaataa acactcatag ggaaaaaaaa cagacttctt tttggatcaa aaatggtaag
723781 caacattttt gtgacaaaat atgaagccct ttggtaagaa gaagcaaatc taaagccagt
723841 gaagtctggg aaacgatcaa ttccattttg atgcccccctt gacatcgttt agaggcaggt
723901 tccctgcagt ataagtcaca ttatcgattt cctgctgccc gtagcatcga ggagatctca
723961 ccatctttga gctctaactc atctttgaag ttaaattggc aggaaggtgg gattttctgc
724021 cattttatgc ttctgcactg gaagcctgtg gagatcctca aaaaaggtgt taaaataaaa
724081 gcattttagc agatttaaca gtctttcttg gtgtacttat tttggatctg ccttggtgtc
724141 tctcctctag ctaccaaggg attttcagga acgccttagt tgccacatgg agaagcaggg
724201 taggggaagt gggaccacca tggccatgtg ccactccaac tacgctgacg ctataccaac

724261 ggccataatt gcaaaagtcc tggaaaagcc tgaacccgga agcagttgcc ccgtgacacg
724321 ctccccaagc ccgctgccac aggacagtga ctttcttgcg gagacaggaa attccgatct
724381 gaaccgccgc tcttcgtaca agacgtctga cctgtactgc agtgatacgg cgctttactg
724441 ccccgagcgt tggcaggact cggagcgcag gcaaagtgtc gatttccacg ggaccagtct
724501 actccagctg cacgcacaaa actccactga cagcaaccca gaagaagagg cctaccactc
724561 ggctgctttt tcccacgagt ctccatccac cttccaccag caggatgagt ttggcactgg
724621 gagcctccgt gcctccagct cctactccag ctttagcctc gcttcggatg acaagggggg
724681 agttggcgcc ggttgtggtc gcactgccag cggcaatttg tcttcttcgc accagtgtct
724741 ctatatggat tggagagatg agggatgcgc ggacaacgga aacaaaaaca tcccttataa
724801 taaagagagc ctaaactttc ccaaatctct cagcatccag cacatggtcc caccccggag
724861 cccgcagaac agcagctcac cagcgtacac aaggacagct tcaagcttca gcgaatcata
724921 ccagtccggc acacctcgtc tgtcctcctc tcgcagcatg ggctccacga ccgggctgag
724981 ccaggcccac agaggaattc cagaaaggcc aagtgacaac caagtctcag aggatgacct
725041 aagtagccgc tggagacagc tgagcgtgga agatgtcaac acgttctcat cttcctaccg
725101 cgacatcgcg gggcgagtct ctccctacag tttctctgag tgccacttcg ccatggcccc
725161 atcccagaag gtcaaaagtt ccctctaccg cagcttacaa gaaggagatg acgtcttcca
725221 cagccacatg ctggaccagc gcttcgtggg ttccgtttct cctggccaca tttcaaaacc
725281 tcttgagcat cggaagcaag aggcaacttc tgtgttgtat cgagccaaga acgacagtca
725341 agactccgag tgcagtctgt tcctctcagg gagctctaaa gagaaggaca gcggtgggat
725401 cgcagctgcg gcaggcgact ccatgaggga ctacgtaaac ctgagcgtcg acagctctgc
725461 tgagtcctta caccaaagct cactagaagc ctcaagtctt cagcactacc ccagtccaag
725521 gccggctgtg cggcctcgcc cgagctccag ctctgctctc aacgttggcc ccgcactccc
725581 caagaagact tccgcaagat accaaaaatt tggcagcacg ggactgacaa gaaaggacag
725641 cctgacaaag gcacagctgt acggcaccct gctgaactga gccgacggac agagatactt
725701 ttacgttatg catggtgaca attactactg ttaagtacct aattgtatgt cttaaaagtt
725761 ccctggagca cattctttat gtgatggggg tgttgtttta tgtatctctt agccaaggca
725821 tgtttaatgc agaccgcaac actttttcta tgatatgtta cacatcacgg tgtagctaca

725881 gtacccaagg ccagctattc agactattaa tggcagtaat gacactcata aacatactgt
725941 gatatttcct tattttgtca tcaagcactg ttagtaaaac cagcaaaagc ccattaacta
726001 cagctctatt gtacagaaaa gacactgtat gtaaaggaca ctaatgggct gatgtatggt
726061 tccgtattta tatgaatcta agctgatggg aattcaatgt ttatttgtgt gggggggaa
726121 agctgtttta gagggctaac cacatttaga caggacaaat atacccaaca ctgctcacaa
726181 aacaagtggt tttattattg ttgacaactg gccatctatt atatataaaa atataagtat
726241 tttaatcaca tggtatagaa taggtatgag atcatactat aacagtgcca acagatatga
726301 tttttaggta cctataaaac atataattca acacaagcag actttttaaa aacttagctt
726361 ggatttttct tatttttata tcagaagcag atttgggaca tgacgtgaca ccacagcatt
726421 tttaatttgg ggtggggggg tgggggattg ccttaaacta gttgagagct ctgctttaac
726481 gcatggatgt tttctctgct ctgttgttgt aaactgtaag ccgtagtctc cgtcgcttca
726541 catgtaacac acagcagttt cttagacgac ggggttaatg ccgcttgatt tccagcatcg
726601 accttcattg aaataaatta ggatgatgta ttaggtgcat taatcatgtg gaaaaatatc
726661 cattcttcta gttttgtagc aacaaagatc actaggaaat tctccaggat ggctggaaca
726721 gaaccttgta tgcaccaaac aactcactga gatgaaggta gacgtttctc ttgacagcca
726781 ctgtgttctt ctgaaaccag catgaccaat taagctattt tagaagaaat tgtgaaattt
726841 gactgtctgc gtaaatatgt tggtgaaaag accttgtaga taagagggtt ttttttctgc
726901 tcccctccta taatacaatg gtttacgctc acaagtacac atgaggacaa agcgacagcg
726961 ctcagatgtg tgcatgacct cttccatgat acattttgaa ggctttcttc ctgcctctgt
727021 gggtgagtcg atctgaaaag gctggtgaat aagttacatc tccatttgtc tggaaggtcg
727081 cgctgatgcc tttcatattc ctgcatattt ctggtttcag gctgtttttt taagatttgc
727141 aagctgctct tctcccctct ggccttgtca tgaaggtaga actgcaccca cacaggccgc
727201 aaaagatttt tcaatcgtaa tcttttgtac agtgtgtcta aattgattta gaatgaaatt
727261 tatccactgt atatacgacc gagaaaatgt agaattgttt tgttgtgggc aaaatatgta
727321 gagacatgaa gataggtagg cactgcgtac aagtgatatt acactatgac tattttttat
727381 ccgctttact ttatatttat ggaatgtttt gaccctggat gaaaatgctg aagcgtttct
727441 gtcctgtcct acctccaggt ctcttccagg tacaggccgg cagtccaaat gtcaacatgc

727501 ttgtctctgg tgcatggtta tatatttttg ctgtatgttt atttatttaa taaagacgtg
727561 tgttgggaaa tatgccaagg tttatttacg tcatttattt caaggatatt tccaaaatgt
727621 cacagcttcc tccgctggtg gatccagttg attgtaattt tttttgtctt ttctgtcatt
727681 tggtatctct attgtttcat gtttgtatta aggtattaga gttttcttct tgggttttta
727741 agatctgttg cactggttga taagcatctg tgtgcacttc agtgtcaagg ttttgaaaga
727801 acctggcaat gccttgacag atgtctacat attttggttt agtttccacc attcagcctc
727861 actctttgat tctctaagct gtcagcgtga aagaaaatct ttcatcggcg ccagaaagaa
727921 caaaaaggca tggagaaaaa cgatgtgcat gcagaaactg gaggggaaaa tataaaaaaa
727981 tataaaatat aaaaatccca aaagacatat gatttctttt ttcattttgt tatcgcaaaa
728041 aaaaaaactt tatgttggag ggcaaagctg aatataatgc acagggagaa acactgccat
728101 tacaccgccc accgctttag ataaaactca taacaatatg tcccacttat tcaaaactgc
728161 caataaagct actttactct cgggcagcaa aactttgcga tttcaacaac tatttaagtt
728221 aacatgctta gagggaatgc caaatccgag tgtgcaggaa gggatctgtg atcagttgac
728281 ttccagcatc tctgccgagg gtttacggtc ttatgctaag agattatctg agatagtctt
728341 gaatatgcac cagtttttgc agtgcaaatc tgcttattcg gcgagggcgg ttagtgccag
728401 accttgatct ctccggccca gtcgcaggtg gctgcagtgg cggggaggac gggatgctgt
728461 gacacacaca agcaggcctg tctgtgggcg tggagggtgt gcagcaaatg agcattgtgg
728521 taattataga agtaagcaga gccggtggag cttcctgagg ccaggatgcc tccatccagg
728581 gaaaactggc accgtaccgc atatccctcc acctggaaca aggttacagg ctttagtcac
728641 gacgcattaa ccttccgccg agagtgatgt tagcattagc ataataaaat gtcagcgcaa
728701 taaccaaatt gccgtttagt tgattgcaga cctctggagc ctaaatgttg tgggcatgtg
728761 atcacaaaga agctgagtca gtcattgaga aagcgtgatg tcggcatatc tgtcgagttt
728821 actctgcatt ttggggtgat ggtctaaatt tgggtcgaaa gaatagggga aattctcttt
728881 ggactttttg gttttagtca ttttaaaaac cgtctagttc tggaagtact gaagcacaga
728941 catccacaaa ggtcatttaa actgcaactt agacgtgtaa atccaaataa taataatttt
729001 gcatcaaccg ggcctgtcaa aacattccat ttcatctttg ctgtttctca ccttgtgacc
729061 ttcatatcgc ctcctcttgt tcatcttata aggcttctgg gaggagaatg ccgccatgta

729121 gtccccattg gtttgagcga caaaggcctc ttccagtggg tgaagagcaa ggctgggaca
729181 tgtgtatcgt tcctggattt agatgaatgg ccgaaagaat ccagagagag aaattaattc
729241 atgatttgac ctttcctttt tgtcccccaa caggaagtga gctgcatgtt aacgaggagc
729301 attaattaga tttactgaag ggtgtgttgt ttcatttttt taaccaacgg aaaatgtgca
729361 caggcgtttc agactcgagt gaatttttc agtgccaaaa cccacaacag ttaacgaaac
729421 cgcgtttgaa acaggtgggc cacgtatcac acatcataaa gcacagttgg ctctgcactg
729481 cataactgtc aaaatgcgcc ggtttcacaa ggctcattac tcagtctgtt tcaaatccaa
729541 ctttttcagg gatgtactgt aagaaaacct caattacttt atatagacat ttaagaggaa
729601 tagaaaagga gatccttacg tggtaaatct ggttggagag tttggctgtg gtttgaaagt
729661 cccaggcaat cagggtacgg tctgctgagt ccctgctcac acagtcggag ctggttatga
729721 actccacacc acctctgaga aacaggatgt ccaaggtctg ctggatactc gctttgtaca
729781 atttcaccac ctggacggca gcataaacac aggcatgcaa attcagaaat gctaataaat
729841 catgtgcgct aacattttct attaattgct cgtgtcaaag atacctgaat cccatttact
729901 ggtactggga ttgtacagtg cttaacagta aaaagctgcc atttttctaa acctagtaag
729961 aagtaaaaat aaatttcacc aagctaataa ccactcttgt ttgattggtg gatgatattt
730021 atagctcaga aacctgcgat gtagtgtagc tgtagcataa cccttaatag ctggccttat
730081 aaaaatgaat atatatttgc attggctctg aaatagaagt cgacccctga cacatgggtg
730141 ttgggatgga tacggcctgg acacactgat ccttgcacct ggtattttct gcataattca
730201 gttagttgct gtttgagttg acaccaccca aaaaactttt tgtcctctct aactgaccta
730261 tttacagacc tcagaagagc aggcaacagt tctgtctctt tcagtgtttt gctgatggaa
730321 attaatcctt gttttgaggc gaaatacaaa gttcagacaa gattctcttg aaaagattat
730381 aatcagccaa taatgaatga tgaaaagaat taaatcaata cttagaaaat acaaaggcga
730441 aagcccgagt ttttagactt cattggggtt tttttttttt ggtttttttt aaaaattcta
730501 atcagtttgt tcacaggatg tttgagaaca tcctgaaccc agatttgagc tttgttcaca
730561 taaaaaaaac ataggatgct gaaacaatct tgcaactgaa cagcgagccc atttagaaaa
730621 acctcaggct aatgggaaaa tgctgtgtct ttcagagcgg cggccctttt ttgtctaatg
730681 gggtaatttc accccagagc gagaaccgca cagcccaagg ctaacgctga cacgcccgca

730741 gagtgctcca gccaatttgc tttgggctgg gctacactca tactgacacc tactggcaga
730801 agtgatccct gctgtattaa ttaaaaaaag ccctgcagag aaaggactgt ttccctctgc
730861 ttgctttgct gatgggagaa aaatcaatgt gggggctcag cttcagagag agaaaacagg
730921 cttatcacat aaaagagtcc attaacctta ataacaatat caggctgcag cttcagggga
730981 aagaatcatg aatgctgaat ttaattttgc tttaaaatga ggtgactatc tacagttttc
731041 tttcctaaat atctggcagc atggtggtgg cctgcatctc gtctgtggat gaaaggcatg
731101 cgctcaaagc ttagctgctt tcgatttagg aaaggccaaa tttggtgaag ccatgtcaat
731161 aaaacccaat aaaccgtctc caaggagttt gataagtttg accttgctgc agcgagaatc
731221 ccaggccttg accaccgaac tgtaaccccc acacaggaac gtctctggaa tgctgggatg
731281 caagcccaga cacatcacct tgaactggtt gtcaaatctc cccacctgct gccctgcaga
731341 tgaaaagaca ggaaaggcag atcgagagga caagatcaat gagtcctttt gaatatcgaa
731401 acgccgcctg gctcacaaaa tgctattata cgaagagaaa ttgcacactt tattctccaa
731461 ctcttcttca aatacaaaat tcatcacaag gaagcaaact agttcttcca caactattaa
731521 aattccaaaa ataataaaac cattgaccta ttgttgtaaa agactacagc agaaacaaat
731581 tgactttgtt tcacttaaat aaaggcttta gagtactaaa ctaatgaatt aggacaaact
731641 gctgcagttt gttttatttg ggaagcttga ttggattttt tttctgtatt aaaggttgtc
731701 aagactaaac taaatagata catttacata ttgtttcctc tttggtttgg taattgtgca
731761 cactgtgagg agccaatccc ccttgtgaag cagccattag acatctgtgc agtaaagact
731821 gtgtaattaa accgagaccc ctcctttgat ggtaacaagt gaattgagga ctgatgagag
731881 ctgcagctaa acgggccgtc aaaaattcaa gaggaaaaga tgtgtggagt tgattgtttg
731941 aagtgtgtgt gtgtgtgtgt gtgtgtgtgt gtgtgtgtgt gcactttggt gatattctga
732001 ctaaatatgc ctcacgggtc tatagacact ttctagctat cagataaaag ctctgttttt
732061 tctgtctgtc tctcattcat ctttgacaaa gatcttgcag actgaatctt tccttgtgtg
732121 cgtgggaaca tgtgactaca ttgctccaca tctgctgcca agcaggtagg taatttgatt
732181 cacgttcagt tccaattaat atggcagcta tagtgcaaaa agaaaaagcg gcatataaaa
732241 cactgttatg gctttagggt gttttatcaa aatcaattaa aagcgtaaaa aaaaaatggt
732301 gggtttaaat gtgattaaaa gtgacaggct agtttccttg gaaaacaatg aggagggcta

732361 caaaaggaac cagatgaaca cgaaccagcg gctcaggcga tcgctatgca aagtagagca
732421 gctcggggct gactggttcc ccattaattt agccaacaac cccctccaaa ataataaaaa
732481 gaaaaaagtc acagacagcc cacaacaaac tgcactgcac tgcacaccac atactctttt
732541 acggggggt cacgggctgt aaactgcacc gaaaaaggtc catatttgac gacagggtgg
732601 gggaacacgc tcctgacttt gaactgcaat aatccaaagt tttaacaacc aatttctctg
732661 ggctacccct taattagctt actcagggtg atgtgcagga gatctgtcac tgagatttaa
732721 aactcatgtt tggcaaggcg atgagaacat cagccttccc ctcacagtaa gtagggccct
732781 gtcatatttt aattaagcaa gattaattag gctcctttgg agggaagata aggggaaggc
732841 tgcagtgatc ggtccctgct tcaccatagt ttactttgca gcccagaaag tgacacgaac
732901 tgacattaaa gtgacagaga cgtggatgag cctggtttga cagcagaggg ggacctggga
732961 cgttgtagat tttctcctca ttaactgacc cacccatgta ggttgcaaag cttgctttaa
733021 acaggcatct caaagtagat attgggaggg agaagtcatg ataaacaact aaaaaacgtg
733081 aaaagcacgc cttgttcaaa gggaacataa tggtttggtt tatatgattc tcaaagaagg
733141 tgaatgcgct aaagcagata tttgataaat attaaaaatg ttcatcatgt ttaggtaaat
733201 aatgataata gaaaagtctc taggaggagg agagcaaaaa tcaaaaccga cttgattaac
733261 aacaatcacc atggcaactg cacacctcta catgttacgg aagtttgtgt tcagtttcaa
733321 gagacaaata ctgtatttat gagataaaaa cctcccaagt ttaggaagtg gttatgtcct
733381 gtaacagcac attgaacttg gtgaagagat ctcggatcga ttgataagct agaagtggaa
733441 agaatgccaa atcatcacac tgtattgaat tattatcact tcgctgctcc tctgtgtcat
733501 taagggctgc tgacgctgac aaacgttgga gaatatgctt cacacatgac agatattcaa
733561 cccatgatga cgtttgtttc atttctctca gaggagagac gcctctcaaa agtgtcagtt
733621 taccgagact cattcagagg ctgcagtgca ggtaagtgct tccagttaag atcctgcagt
733681 tcctgtccta ggatgtgcag tgcttcagcc tttagctcac atatattctt aacattcatt
733741 agcacgttga tacatcacat cctttgttga gataaagtcc tgcatgtgat gttacaaaat
733801 cagtgttctg ctgctcagaa tgacccacag ccactcagcc acagttcttt ttctctctgc
733861 tccccactgg actggcacag gctccaaatt gcctgtaggt gcaacggcga gagtgaacgg
733921 ctgtgcctct ctgtcggggc tttgattaat tgtctacggt gtgctcctgc ctgcaggaag

733981 tcagctggga tcagctccag cgcctgtggt cctctaaagg ataagcaggt gtagtaaatg
734041 gatggacgtg cacagtgctg gtgcacacag cttgcaggtc attcgtttaa aaaaaatgaa
734101 ttcatgacaa tagcgtgtgt tattctgcaa agtacgaggt ggacgtaata tgtgctgatt
734161 ctacatgagt gctggttctt cctttgtggg ttttttttct tttctgttgg aaaccttcca
734221 ttccaacagt gccggaaggg atttggacag agaagccaag acctgctcac acaagcacac
734281 gctgacatgt ccacctgtag cagcccgtca tgggcatatc ttattctgcc aatcagccag
734341 ggaagacgct gacaacagct gccacttcac actagagccc tccagatgaa attcatttat
734401 tcctctcacc cactcctgct ctccccgttg gtcttcatct gaattcatcc tgcctctttc
734461 cattcctttt tgctgcgctc tccctttcc ttctcctgca atatgcttct ctcgcccgat
734521 acactcctct ccctctcccg attgcttctc agcagtgaca caccgtttgc gctccgctgg
734581 gagcgggtgg aggattttac aaattaaatt ggacgcggaa acaactgaaa acgccggcaa
734641 agaaaatgga cccgcacgct gttccggatg cttggcctcc tctcgaaatc cattttctgt
734701 aaacacggcc gctgccgcag catttggtta tgtgactctg cggtcaagca tggggatcaa
734761 tggctcctct tcccggcaga gagcataaag atgacattcc ccctcattag agagccacat
734821 cagcgtccca tcagtctctg tagatatact gtaccaacag ggtctgctgc agagatgttg
734881 taatctgtgc tagtttttag aaattactgc acttcagaca ttgttagttt tgtgaaagca
734941 aacaatgaat catattttag tgatataaat cccttagtaa cttagctttc attatggatg
735001 ccacttgggt ttcatgtctt ggtaaactga atattcctgt atgtgattag aaatgtcaga
735061 cacttcagac tacttgtatt aataagtaag aacccctctg cacagcctga tcctcagata
735121 tgatgaacct acagataata aagagagtgt tttaagagag ggaaaaaagc acagaagaaa
735181 attgagggta caaattgctc ccacaattta tttgtgtatt tattagaccc gtttttttgg
735241 acgttccttt ttgaccatta ggtcctgcag agcagccagc gctcatatgc agagatatga
735301 agatatgtgt ttgtcatcca ggttgtcggg gatccaccct gttagctgtg tgttgcatct
735361 ttctcttctg ctggagtttc atcaactttt ctctcagctt ttgtttttac tttcaaggtg
735421 gacgtcttca tcggtagatt ccatttccct actgtgacag ctgactttgt atttctgttt
735481 catctgaagt ggaagactgt gtgggaagta aaggaaagcg agatttctta cacttcactc
735541 tgctgcgctc cctcaaatct ctcctccagc tgttctcatg tacgtctctc ctgccggcca

735601 cctctgccag cttgcctcca cctcatctaa gttttcccag gactttcatt agtactcttt
735661 ctttttcagc tctgcatctc ctggatgttt ttttgagcca ttttctggtc ttcgagcgac
735721 ccactggttc tcctaaaggt ccattccaga aaccacaact caacaacaat cccattttga
735781 agtctctccc tctcttaagc agtagttcaa atacctcgag tatagaccca tctgtgactc
735841 atcttgctct gtctgtccaa gacatttcca ctggtggacg acagtttgca gaagcattca
735901 agagacttat tttcaaagcc tcatttcttt cttacatctt tactatgatg ccagagtctt
735961 tcgatcaatt aaatatagat tttaaaccaa cttttataat tgaagatgga ctccccctcct
736021 ccatgaagaa accgaggacc tatctcaaca gcagccccgt gccacctcct gccattttga
736081 gataggtgga aggaataact ctgttctctc tctctctccc cctctctttc tctcggtctc
736141 actcactcca tcccgtgttt attattgacc cccttcctgc agctgcctgt gcatttaatg
736201 ggttggccat gaaattatct gtaggcttta tcggcagtat tgactgacat gctgagagcg
736261 aaagtgcaaa gagcagcacg tcatcacagc catgtgttga cccaaaagat gttgttgcca
736321 caggggcttt aaaacaaggt ttgtggtcca tgtgcttatc ctggtggtct gtttgtagcc
736381 cattctggaa tgaacaacgt gctacatttg tttcatgttc tataagtgga caagacgaac
736441 agatccaatt ataatgcgta ttaagtgtaa tgcaccgaga caatctgcgc tgtaacaatg
736501 tacaccatca atcaaatgcc agcccataat ttaacagcct gatggcacaa ctgtagattt
736561 cttagagttc actttgaatc aaactttgtt aaatacctct gaactatttc aaaggtattt
736621 gaactaagac aaatgcacat tttccaggtc aagtagggtc aagcgtaaat aaagtacaat
736681 ttatacagtt atgagggttt ttggtccata ccaatttaga ctcactccag gcatgaatta
736741 ccgactgcag tgcctactga aaatttgtct tgttttttcc ccaaatacga acaatatagt
736801 ttaaaaaaaa cttgcaaaaa tcagcacaga agaaatgaga attagtttgg tgcggtggta
736861 ttttgagatt attgttttca tcagatcagt ctgaaatgtt gctgctcacc cgtctccacg
736921 tctgtaatca cagccgtgtt gtcgaacgag cctgtaagaa gacgccgccc gcagggggtc
736981 cagcaggcat ctcgcacggc tcccgtatgg caggtgtaag ctctcaggca tcggccgctc
737041 tctgctccat cccagacctg ccacgcatgg agacttcacc tcaatccaag caacagcaat
737101 tgattttaaa tgcacaaccg tcagaccaca caggttttaa tgcatttagt tcaaactgag
737161 actaaaataa aagagcaaca ttttatctta cacatttaga caaactcccc acacctttg

737221 tgactgaaag agatagaaag tggtgttttt ctttctagag gaagaatgcg
ctgtatcctc
737281 tggccctgaa agtccttctg cgagaatgag gcactgcgag tgacctctgc
ctccctttca
737341 catagaatcc tagctttctt cacctgaact caattattat gaggtgaaat
caaatgacta
737401 gcgaatgctg ctccattttt gccattagat ctacacaatc aggcctattt
gttgaatttg
737461 caagggaaaa aaatctttga agatgaatca atccaccttt gagtctgcct
gtgaatcagt
737521 tctattgttt aggtgttcta ttgtcacaga tcagtctgct gattatggag
acagcagtgt
737581 acaaagcttc tctttcatca gatgtgattg ttgcaagtgc gccaaaatca
gaccctacag
737641 aaaaatcggt tatgaatgaa ttgttaaaac taatattgat tttattttat
tttttttgg
737701 cactggttga tgttatggtg tcttaaacca gcatttggct cacagatgag
tgggagtaga
737761 attctcagag aacaagaaag aaaactttcc ataaaactcc tgcagagaaa
aatggctggt
737821 ggtcctctgc ctgctgacta aggttcatta cagtaatgac aaagtcatct
tttgtttaaa
737881 atccttggtc gcgactgggc cacagaggaa ctttgaagac tagtgtgagg
ctctgcagat
737941 ccctccacga gcctctctta gtgtttcatt attaaagctg tttccatcac
acagcataag
738001 aaagtcagcc caagccatct ctcctacaag agccaggctg gggaggggc
tgctgccgca
738061 tggaggggg ttgtcgtatt aatgtggcag gcgatgcttt aacaaacaga
tgccgcagga
738121 gccaccgggg agagcaaaga gaagagggaa gagctgcact gttaattgca
caaagacaag
738181 agtgaaagtg aagaactgat tacgtgcatt tctaaacatt caccaacaga
ttggggaaac
738241 actgccatct caaggtaaat ggagaatccg ctctctaagt cactcacaca
tccacacaga
738301 ctgaaaatgt tgaacattgg ttcagccttt tcttcaaatc actacagcac
aacttgaaaa
738361 caagcccctg taataaccct gagcaatgtt gggcttctgt caatgaacac
ttgtttgttt
738421 gcatcccctc caatttcacc attcacttgg cagacacaca gaatggatat
gtgcaaatga
738481 ctcagtgggc taatcagctt cctaaatgac tggtggtaat taaagctaaa
ttggtattcg
738541 tgttatcaac ttagaacctc tgcctcacac ctgctgtagc catggtgacg
acagctgcaa
738601 acacgctgga ataataatag cgtgactctc gctgttcctg cgagggagtg
acagagagga
738661 tgaaggtggg gacagtggcg ctgggggga cagttgtgaa gatacacata
gactggagca
738721 ttaagacttc acagagcagg gctcctgtct cttcacacac agaacaaaga
acaccacaat
738781 gcatgcagcc tcttaataat tatgcaatca ggtttgctca ttcagaatgg
acaatgttca

738841 aagaaaacat gtatttcaat ttacatatgt ttaaaatgaa aggaatgaac
actgcaaaat
738901 aaccaatcca acaataacaa tgaataaaca cataggtgac acggtatttc
aaagtataaa
738961 aacacattag cattgccttt ttcttactgg tggcagtagc agaagtgtta
ctaatacaaa
739021 tagcagcatt gcattttagg tacttgtagt tttttttag ttcattattc
tccagaatga
739081 ttaatatctc agttgaagag agatatttgc atttttggaa tgaatattta
acttcattca
739141 actttaacta ataagtaatc ctgataagga gctaatccta ttctctgcca
tttaaccaga
739201 aatctctagt gcgtgattag agtataataa agtggtgcag cagggctgaa
tatgttttaa
739261 taaacacagt aagggcgcgt gtgtgtatgt gtgttttaaa gttgctttga
tattgttcca
739321 tttgataaac cctgtaaatt gccatgaaat caaggagatt tcggataatg
aagatatctt
739381 cagggtttaa atgaagtgca ggtcctccgg tctgcatcgc tacagctagc
ttggtaaact
739441 ggagaagagc acaaacataa ctgaaatctg ctcaccttat ctgcaccatc
ttacacaaac
739501 tgccaaacca ggcagcctta gtgctgcaat tgtataatca tgcaaataaa
ctcatgcatt
739561 aattattctg agccctcttg ggatgctggt agaatgcatg ttgtccttct
ggtatggtaa
739621 catttagctg ctggcgaggc acccacatga cctgaccttt aggataggga
aatcaggggt
739681 tgtttccctg ctatgaaata aaaaactgct actgaatgaa tatttatatg
tgggggggaa
739741 aacctgttat gggaggcaca agaaaaaaac ctgatcacag ctttcagctg
agtggatgaa
739801 acatacctta aatgttttgt ccatggaagc agacaacagc agatgactaa
gatgaggtat
739861 tggactccat tgcaaagtgt tcacaggacc ctggtgacct cccaggctca
tgagaagttt
739921 tcttggtatc ccagctacgc caagtttttc atcaatatag tgtctaattc
gctctgaaac
739981 gccagacaaa atcgggcttt ctgttgtttg attgcttttg gtttgctctc
ctgtaaattt
740041 ggagtcctcc atggctgcga cggtgcccag tctctgcctt ttaggcatgt
aaggtctgac
740101 accagataag tcactgcaaa gtcgtttgga tgatttcaac ctattatcag
atctttgtat
740161 gatgtgagga agcctctgca gggatgacag ccttccctca gtttcaccta
cattcagagg
740221 tgccctagaa tcactacaaa gatgagttgc tgctgtgttg tcacagcaat
atggtgacgc
740281 taagttcaaa ggctgtgaat ggcaatatag tgaagatgtg tttccttttg
ggatagtttt
740341 atgaaagtcc tgcgctgatc tagctgcctg aggtatgaag ctgtgaagac
ttggttctac
740401 gccagaggac ttccacgctg tgacttgctg cttttcataa caagattcag
tttgagaaga

740461 cgtcctggcc atgtttagtt caaaattctc atcttctgat tctgaatctt cataggcaac
740521 tagcgaagac attttcttcc tgttgaaaat tagagggtaa aaattagttt acttcgtaaa
740581 attgcgctgc tagagtgaca ctcagtggtt atttagtgtg ttgcaccgat gtgttagtta
740641 ctttagaaaa gcgacttcag ccatgaagtg caccgatagc gaattttggg aaggtattag
740701 gaattcttga agtttgattc attaaggtga gaaaatagtt caacagcatt ttcacaagat
740761 gatttaaatg tccctaaata ctaatcagtt tttgtctcaa acagtatctc atccaacgtc
740821 tctttaattt cataggaaac aaagtaataa ataagattta aaaacaacaa caaaaaaaaa
740881 ccaattagat cagcatccag gcatcttagc catactccta ttttatagtt tatttttctg
740941 acgtctttaa agccattcag acagttcaca aagagaactt ggaactactg gttatcattt
741001 gcttcctacg attaagccat tacacctcaa ttataccttt aaacacaagc tggcatacga
741061 ataatgaaca aaacatgcat ctagctacaa ccaattctaa gaaaaggaca gagaaaaaaa
741121 caagaagatg taaatttcag ttgactaaac catgaaagca atcttgttta atggttgtac
741181 ctgtcttttg gtgaggggtt ccacgccgga agtgtttcaa aaacacaaca atctcaaaat
741241 cccgacaaag catttcagat taaaagcatc gtaccaataa gtacaattac tccacgatgt
741301 gtttaagact gtaatctttt attatgaaat tttcagcgga aaacagagcg agttccacag
741361 tcaatggtcc gatgtttcgg aatattttat ttaatgtaac cactttatga ctacgtcaca
741421 aagcctgtat gtataaaatt aaattaaacg taatatcgtg tgtatcaagt ttactatagg
741481 aaatatacca cgctgcccct ttgcgttaat agacaacacc agaacaccgg accattagaa
741541 caattttgtt ttcggggaat acattgggct aaacactgcc tcagataggc tgatgtaagt
741601 tgaatgagaa agctgtcagc tgtgtgtgta caatactgtt tggtatgcgt gttctttaat
741661 atttctttaa aaatcgcatc cgcgtaaagc gattcgcgat caacgtatgt tgcatccctc
741721 cacaaaaagc aggacaacgc gtgtcgcttg agctgtattt cgtgattagc ttgataagta
741781 gccaagttag ctctggtatg ttcacatatg gttctcacaa ctgctgttga aggagctgca
741841 atgtatcgtt atagcctcca taatcccgtt ttatccttcc ttgacggtct tctttcatat
741901 gcaacttcta tatgaattgt attatgtgtt aaatctttaa attgttgaac attttgtgtc
741961 attcagccaa tgtaaagagg atggccgaca gtccagactt tgaaagaacc atgagtcccc
742021 tggaactgta cgagaaactg acagcacaag gagaccaagt cagggctttg aaaacagcca

742081 aatctgacaa agtaagtact actgaattaa acaaaatatt tatttgtctt cttacctctt
742141 taattccaag aacaacctac ctaactcatc attttcatta tttgtgatgt cccgttttca
742201 attctagcat tcatatcatg aagttacaaa gtaattcact tttattatac actgaagaga
742261 aatattaatg caacactctt tttttttgg attcatgagc tgatccaaag gtctaagact
742321 tttttatgtg tgtcaatgtg ctgggtcgac agcaggttaa ttgcacaggt gtacctcaca
742381 ttgcccacag taaaatgcct tttttttaaa aaaaaaaaaa aagaagaaag atttgtgaac
742441 agtatttggg aaatagaatg agaatagttt tgaatccgct ccctgcttct gttgaaattc
742501 ctccgagtga tatctactag cattgttttt gtttcctagg ctgagattga tgctgccgtc
742561 cagttgctgc taaagatgaa actggaatac aaacaagtga ccggtcagga ttataaagca
742621 ggcagtcccc cctcagaaaa ctcagctcca ttcaatgggg caacagcaga tggcagtaat
742681 gatgaagaca cagtcgaccc gtggaacgtt tccaccacca atgccaaagg agtggattat
742741 gacaaactca ttggtgagat gggattattt tataagtaat gcatagtgta ataatatgac
742801 atggattata tacagaaggt atgaatgtta tattctcctg tttttgatta gtaaggtttg
742861 gaagcagcaa gattgaccaa caactagtgg acagaataga gaaagtctca ggtcaaaaac
742921 cccaccgctt cctacgaaga ggaatcttct tttcacacag gtcagctgat gttagattag
742981 aacaatgttt tctagattca tatttatgtt ccatagttgg atactcacca aatatttatt
743041 gtgtttatga aagcaaattg tgaaaatact ctaactcatt ccaaatgctc cgctttccac
743101 tgacagagac atgcaccagg tcttggatgc atacgagaag cagaagtcct tctatctcta
743161 caccggcaga ggtccatcat cggaggccat gcatgtcggt catctcatcc cattcatctt
743221 taccaagtgg gttatttgct ctgtaaacgt atttattgtg aggatatgag tgaaggatct
743281 gtcatgtgga tacatgatga gccttgcaga cttctgcaat gttcataaat gtaactcatt
743341 tttccctctc aggtggcttc aggatgtttt tgacatccct ctggtgattc aactgaccga
743401 cgatgaaaag tacctgtgga aggatctaac aatagaggag tgccaccgct ttgccgtgga
743461 gaacgccaaa gacatcattg catgtggttt cgatgtcaac aagacgttta tcttctctga
743521 ccttgactac atggggtaat ccgatcttct aagcaggctc cacatcttat tttatttagc
743581 tttcattgtg ttttatctat gttttaattt gtgcagggca tcacctgagt tctacaggaa
743641 tgtggtgaag attgagaagc atgtaacgtt taaccaggtc aaaggcattt ttggatttac

743701 agacagcgac tgcattggta agtcaaaact agcaaaaaaa aaaaagcttg aaggttattt
743761 gctgtatttt gtgattccaa gtggaatatt ttggctcttc atcttttttta tttttgctgt
743821 cattccagga aagatcagct tcccagccat ccaggcagca ccgtccttca gtaactcttt
743881 cccacagatt tttggaagta gaaaagatat ccaatgtctt atcccctgtg ccatcgacca
743941 ggttaggaaa atatttctgt gacaagctga tacagcctgc tggtgtcatg tttaaccgtc
744001 gtgctcatct ccttcatgca ggatccttac ttcaggatga cccgagatgt tgctccgagg
744061 attggttacc ccaaaccagc gctgctgcac tccaccttct tccctgccct gcagggggca
744121 caaaccaaga tgagcgccag cgacgcaaac tcctcaatct tcctcacgga cacacccaaa
744181 caaatcaaaa ccaaggtgcg gcttttactg gtcagattgt gtcgcgtgtg accgcagacc
744241 aataattgtg cctcgcgaaa ctgttctttg caggtcaaca agcatgcgtt ttcgggagga
744301 aaggacacag tggaagagca caggaagcac ggcggaaacc ctgacgttga tgtctccttc
744361 atgtacttga ctttcttcct ggaggatgat gaacagctgg aaaagatcag acaggtgagg
744421 gcagatcaga ggaagggaca aaatgcttta tcttctgttt ttcctctgca cctcaccaac
744481 attgactgtg tttctgttaa tttattatcc ctgtttatca ttccctcacc tcactttcat
744541 tcttactctg tctctaggat tatgccagtg gtgctctcct aacaggagaa ctgaagaaga
744601 tgttgattga caccctccaa ccaatgattg cccagcacca agagcggcgc aaacaggtta
744661 cggacgaaac ggtcaagcag ttcatgacac ccaggccatt aaattttaaa cactgacttc
744721 aaaccgacgc cgatgttgcc cccgcctctg atctatgtac cagaagaaac gtgcagtttt
744781 ccttcttctg ttgcttctta tgttaccgtg aaagtggaag ctggacattt tacatccaga
744841 ttggacctac gtgcgagcag catgtacatc tctatcttat agagtttatt gtgtctacag
744901 cccgagtgta tgtgtcatga gcctgactgc tggtctcagg gtgaatgttg attaaagctc
744961 tgcattggag tccatatctg ctcagacaaa aatgccttca agccttgttt gtttacttgg
745021 aaaacatcct gtaaaccaaa aattagtctt tgtagttgtc tacacatggg accttactcg
745081 ttagataatc ttgccctagt taccctgagg gatcgggcac ttctggcaca gacatgagat
745141 atctggttaa aaaaaataat aaaaagcatc actctgaaga aaattggagc tatcaagatt
745201 gcaaacagta gactacttaa gattaaaatc cattatgtaa ctgtttgctc ttcgtaacaa
745261 catggaacac tagatgtttg tgcactttat gtagctttca tttttgtctg gaatgcacct

745321 ccatattaga caacaaaacc actaaagtta ctgagtaatt tgttggaaac ttattgaaaa
745381 tgtgttaaat ataattatat cattttatta aaccaaatga aatgtgaagt catgtttttt
745441 tattttctgt gatcagtaga attggacaca aggttaatgc ctgtggttct actgtttcac
745501 tttttaatcc ttgaatatca gtaaatattt tctatttttg agggaaactc tatttccttc
745561 ctcatgtgac ctttacccag aatttgatgt tttgttctag catttcaata ctttgccaca
745621 ccaataaaga tacaaaggtt tatcacatta tacagtagtt tattcatatt aattgcatgc
745681 acactgataa gtaacatcgc tgcttatata aaactgtaaa ctagaacggc acatcaaata
745741 cataaaccct gacaatgcaa actacaaaaa caccttaaaa catttaaagc ttttaaaaat
745801 gtttcttcag taaattgggc aactctgtgt gctcagtttg cacaaacatt ttgaaatgcg
745861 cttttaatgc gaggagggaa caactgcgtg tcacgtgttc ttgtaattta ctgtctcgtg
745921 ctgcgtaaac gactctaggg cgcgtggggt tttttcatat gtattttcag cagaagcaac
745981 gcaaataaaa ctttattgac aaagtcacac gttacacaca ctataactgc aagactgaag
746041 gcgaaataac gacacaacat atcggtgtag tcatcaaact tcggtacacg ccgacgacat
746101 ccagctcgat aatcctgttg gttcaggaga acccgcaccg atccttccac aatttgtccc
746161 gcaccgacca atgggtgcga ggggcggacg gtcgtcttta ggggagttgt cctcctccag
746221 gtttcccgat tgggctgcgg cggagaaaaa ggcaaacatt cgtcttctct ttttcccccct
746281 tttaaccccc gcctcccagt ccggtgcaga aatagaactg ctctggctga acgaggctat
746341 agagtcggac acatcatcac aaaagtcggt cttccagttc accgttattc gcgattctct
746401 tctcgctcag catcagcaac ctcgaaggaa gagctaacct gcttggatcg ataccaagcc
746461 gggggttttg atttagcact aaatattccg gattcttccc ccctctccca acaaacgacc
746521 cacctgagga tttgtaaaga ctcacattcg tcgagttagc ctgactttta aaaaaaaaaa
746581 taataataat aatttctgag ggaatggcaa tggacttcgt tgcgggctgt atgggaggta
746641 agctaagggc ttagcccaaa tgttgatgtt agccaactag ccacgttaac cagttacctg
746701 ttttttcctg cctaattatt cagccgacgc ccggatattt aagggcttaa attaggttat
746761 tttcccctta tcagctgttg ttatcattag gtttaaatgc agcagtgtga tgcaaatgaa
746821 agctgtagct gttacatttt ctttcatgaa atgtggggga aaaaaaaccg gatgtaagtt
746881 ttttctttca agttctagtg cacctccaca ggagatagcg gtcttaaact ctgctatcct

746941 gggacataag gtcaaggtct tttctaccca actcgcaccc aatattttct aaggataacg
747001 tgatccttaa aggaaaacca atagacaatt tagagtacgt tttgcatttt atacgccact
747061 aatgaatgca tgcttcaccc ccgattattg taaaatctct gctcgtcctc gtctgtcgtt
747121 tttgtcccga gatgacgttg tccattcctc atcagggagt gaccgtgcag acaaaaagcc
747181 tacacatgga gcccatgcct tcctctttga atagatgtct tttcatgctc atattttccc
747241 ccaggaaacg ccccagtcta accttttag gaagctggtt tatctcagat accagaacat
747301 gtccaagatc cacaacagat ccgtggcgct gatatcttct gacttagact gttctggtat
747361 ttgttcagtg attcatacat gcccagccct gctacctcaa ccacaggggc cttaatctaa
747421 ttagaatctc tgaatttcag cagcagcaac tgttgaccca ggaaacttaa atatctttcg
747481 tgctttgcat gatccataat gacgcacaac atatcggccg gcatgttgct acaccagata
747541 ttaatttgcc atcctagttg ccgcctctgc cttttgaata cgacacacgg gctgtggcag
747601 caggattgtt ttgggtcgtt ggcataagtt aaaggccagg gccactgtac tgtacgtctg
747661 acacgggtat agtactgacg ggctgtttgc ttttgcagga cgttctcctg gtcctcagac
747721 accatgtttg cactgtgtgt gctccgctgg gtacacgctt tgtgcataaa gcgctaatta
747781 gcagatgagt gatttcctat ttttcacgct caatttgaaa cccttgtttt ccaggtgcgg
747841 ctggtgtttt ggtgggacat ccatttgaca ctgtaaaggt gggtgtgtac gtgttccacc
747901 gtcttttact cggcgttaac atcaggcagt cgcaccatcc tccaatttgc tgtcattagc
747961 tcatttggca acgcttgact ctggataatg gttgaaattg tcactgatca agttgcccat
748021 caaatgcaaa tttttatcag aatagagtta ctcattttat ttcacatttt cacattttat
748081 tttccgctag ttgtggcctt tgattgctgc aactagatga tccctacgtt ccaagagggg
748141 tttatcgaac acgcgttcta aaataatgca gaacgtttga atccatgtaa cgtcacatgt
748201 tttcctatca tcgcgatgcc ttcaggtccc cggcggctgc ttctgtttgt taccgccgtt
748261 gctagccttt gcacgccacg ctgacatgca cagagatggt gaatggtttg gttgtagctc
748321 ttgtgtactt gtgcttttac tgtcgtctct ccctgagctg gtgtctgggg gaggctgtct
748381 ttaacagcag cagtgtcact gtgttgtaga tgagagagag acataaacaa cctctccatg
748441 gaaatgtgac ctagtaagta tggtcaaggt cttttcttcc ccactctcac ccaatatttt
748501 gtcttgatgt atagagatac aaagcacaga ggacagtttg ctcttgttta acttcattct

748561 ccgtagatgt cccccccctat ttttttttcta attttgcacc cccttaacta
tctttcttta
748621 caccctgacg agaacattta ctgtctgaaa tcttgatgcc tgtttatata
cgtttaagcc
748681 tgcaatgcaa cctgtcgttt acacctgctg cgtccatctt tgctctcttt
ggtccgtcgg
748741 gttgctaacg taactcttgt tgtctgcagg tgaggcttca ggttcaaaat
gtggacagac
748801 ccctgtaccg cgggaccttt cactgcttcc agtcaatcgt gcggcaggag
tcggtaagat
748861 tcagcccccc agtggacccc ccccgttcag atttttctta gattacgctt
gcaaatgtca
748921 cagctgtgga ataatacgtg tgttttattt tctgccatgt gctgctgact
gccagtgtga
748981 atctcctcac tgtacagtgt ccggttacgg caccgtctcc gagtcccgtg
tgggctaaaa
749041 aaagctttag acgtgcgcag tatatttggt ggctgccagg agagaaaatg
tctcgcattt
749101 ctggcggctt tccagcatgt ctgacatcac aaacccccagc caagatcaat
aattattata
749161 gaactggttt gttctcagca ctttatatat caagtcatgg aattaaaaaa
aaaaacaact
749221 gctgttcctc aaacaaattt ccttttcgt atgaagatgt gcggttttaa
ggtgtttttt
749281 cagcgtgtgc tgcagccttc agccattcag tctgcttctc atgtgaattt
ttgaaatgtg
749341 tttgtgtacc ttctttatcg tccacgtgtg tcgagctgac cacgtcagag
tagaaatcga
749401 cccgtgaggc cggcactgcc tccagagctc agaaacagga ttttgtggag
gcacagatct
749461 gaaacgggtg gcttccaagc gcacagtggc tgcaactttg tgaagtggaa
gaacaaccag
749521 gactctgtcc cacagctggc tgtccacacg cacggcaaat gtggggaaag
cgtcctgttg
749581 agacatgact caggggtcac tggcagcgct gcactaatct gagctggagt
ttgtacaaat
749641 ggtccgatgt atatcaaata cttcaaggga acctgagcca gagctgtcag
gacctcagag
749701 ttcaccttcc aacagataca ttttcattgt tatgctgatg acatccagct
ttatttatcc
749761 atgaaaccag aggagacaga gaagttagtg aagcttcaga cctgccttaa
agacataaag
749821 tcctggatgt cttcaaattt cctcctcctt aacacaggaa aaactgaggt
catggtgttt
749881 ggtcctgaac ctctcaggga tagattagat cacatgatca ctctagatgg
tatctcatta
749941 gcatctagtc tctctgtgag gaatctagga gtaacttttg atcaaaatct
ctccttcaac
750001 tcacacatta aattagtctc tagaagtgcc ttttttcacc tgaggaacat
cacaaagatc
750061 aggaagctgc tgacgcggca tggtgctgaa aagttagtcc atgcatttgt
tacttccagg
750121 ctggactact gtaactcttt attattaggg tgtccaaaca actctttaag
aagcctccag

750181 gtgatcaaaa atcagatcag acagagttct tcatctaatc gttaggtcac atcttagcta
750241 tgctgttata ggccgaggct gccggggtcc ggaaacatga tcacctgaca ggcctctgtc
750301 accccactgg gtcatggtct cctttcctcc tcctcatcaa gcagactaga tatgctgatt
750361 cctgtgtagt ttttctgctc cccccccct attcatttac aggtatctcc tctccctctc
750421 cctctccctc tccctctcct ctcctctcct ctcgatttga acaggaccct aaacacacag
750481 ctaaggtcca tgagtggacg gtaatccagt ctttcttctc tctgtagatg ctcgggctct
750541 acaagggcat cggctctccg atgatgggct tgaccttcat aaacgctata gtttttggtg
750601 tccagggcaa cgctatgcgc cggcttggct gcgacacacc tctcaatcag ttcctggccg
750661 gggcctccgc gggagccatc cagtgcgtca tctgctgccc gatggagctc gccaagacac
750721 gcatgcagct ccaggggacc ggtgagaaga agtccaagag gaagttgtac aagaactccc
750781 tggactgtct ggtcaggatc tataggaagg agggcttccg aggcatcaac cgcggcatgg
750841 tgaccacctt gatgcgcgag acgcccggct tcggcgtgta ctttctcgca tacgacttac
750901 tgacgcggtc gctcagctgc gaaccggaag acccctacat gattccaaag ctgctgttcg
750961 ccggcggaat gtccggcatc gcctcctgga tctcaactta ccccgtggac gtcatcaagt
751021 cccgcctcca agcagacggg gtcggcggcg tcaaccagta cagcggcatc atggactgcg
751081 tgagacagag cctgcgcaag gagggctgga gggtgttctc ccgcggactc gtgtccaccc
751141 tgctccgagc gttcccggtg aacgccgcca ccttcgccac ggtgactctg tttctgttgt
751201 acatgcgcga gggacaagag tgcagcattc cgaactctga gctgcagccg gcgcagctgc
751261 agccggcgca gctgcagccg gcgcagctgc agcctctgca gccgccttcg gagaacatgc
751321 aggccatcgc gcagcaatga ggccaagggc ttcacacagc tgtccacact gagcgagggc
751381 aactgctgtc tgtatctgca cctgcagaca cgtgtagtat ttaactaata tatatgtatg
751441 ttgagataaa cgctcacttt tgtacacttt tccagtcatt taggcgactc gacactacca
751501 gagttttgca gacgtctcga tccaaataat ctctggccac ttaagagtat gtgtgaaata
751561 tatactagca aaacaaacag catgggcttc tggcaactgc tctggtggtc gttaatgatt
751621 agcataaagg tctacaaaaa gaattctggc agccatttat ctctgcacgg tctcttttac
751681 gtcagggttt ttttgggggga gctggattgg tcaacctctc catcgattaa tcaggttttc
751741 ttcttttgcc acaaatgtgt cggaacacaa taaaccctat gaaaatacac acacgttctt

751801 ttcatctgat ggcctccgtt tgttgtccct gtttaaactg taattaaagc ttcaaacttg
751861 aattcactcc tgtcactgaa ctcatgaaca tgtttttctt ttgaattgtg agcaaaaagg
751921 aagcaaactt ctttgtttgc cctcagatgc aaaagtaaac gcaacacgcg tttgcatcac
751981 gagtttagtg tcagcataag ctgatggcaa ggacatcctg gcagacataa aacgaatgtt
752041 tcaaatcaaa tctccattta taaggaaagg ctggctgcac ccagcaggta tatacatata
752101 tatatatggt tatacaccac atcttatcac agattttctg atgttatcta tgtaaaactg
752161 gtcattggtg ctgtaattga ggttatgttt ggtgtctgga agtgtgaaac cataacacta
752221 gccttaaaaa gtttcaaact tcaaaggatc ggcttctgta ctgctcgatt ctgtctgact
752281 tctgaccttt atgaagttaa gtaatacgtt cgccatgcag gcaggtttag ggggaactac
752341 accgtttcaa aaagagaagt taaactctaa tgtgtgtgca agggctgttg aaatattgac
752401 ttctattcta ttgaagtcta tgtacatagt cttttatgta ttgaggtatt tgtaaataca
752461 ggtgctcaga tcttcagaat aatcatgttt ttgatgcctt gttttcctag atgtttgcct
752521 aaaatgattt ttttgcctgt aatacagctg catgcatact gtaaagtttg acataataaa
752581 acttacaact aaagctgttt acacgttttc tgagaaaaca ttgaaaaaaa tacaaaaata
752641 atgtttttta tttaaggagc ttgtcacaaa attcacctct ttgactataa ttttgaaaat
752701 atatctttca tcatgaaaat aagttattta atcatatact gattgatttc ctctgccaag
752761 tcttttcatg cagaagttat aaaatgggga tattttaaac aaaagagtgt tgcctcagat
752821 caaacactgt atttatacat catgtaaact ctgttctctc tgaatttgtg caaaattgac
752881 cctggggctc ttctccatgg agagaacaaa attgactacc acaatgtagc attagactgt
752941 tcagagagag caaaaataaa gtgtacttca ttctctttcc tatctctttt ttgctgactt
753001 cggtgtgttc atgaagaagg agtcacagga atgacgcagg aagcggtaaa cctcctccgc
753061 tctggatcga ctcatgcagc ctcccttctg tagggcctca atggagctgc agggaaaatg
753121 gggcacaggt gatgtcttct tgttcccttt ctttaccgtg aaacacctgc aatcttacct
753181 gttcataagt tgagcaacag agctgaagct gtggctcata ttgagagcat ggacgtagtt
753241 gactgatggc agggtgaggt acagctgcag ggcctgctcc ctgtgttgcc ctttgacctc
753301 cacaggaacc gagatgccct ggcccttacg ctgctctaat cgtgccagat cagccagcaa
753361 gcaggcgctg tcttcaaccc cgttgctcca cagcaaacgc acgccggtcc gcaccagcga

753421 gatcagggtt ctgtcatagc aacgtgtccg ctggaaggga cgtgatgcct
cccctgtaac
753481 agaggggctg cattattaag ctaatctacc tgtcaactat aaaatactat
ttgtgcccac
753541 tgaccagatt tggacctctc cttctccaca atcagacaaa tgcgctcaaa
caaaccctgc
753601 aggctcttta ctctctcgct cagtcgcttc cggttttgta aggcagccat
gtctgactgg
753661 ctatacctct ccactgccat gcggttgcta acgatgaagt agccgccgtc
gagtgagcaa
753721 acgtggaccg tgacggcgtg gcgctggcgc aggctggtca ccagctccac
actgctgctg
753781 aggcagcgac tgtccaccac gatgcaaaca ggtccgggcg cagccggaga
ctcagacttg
753841 tggggttctg aggaggtggc tggggccatc tggaaaaaga ttttttactt
tgacttggcc
753901 tggaatcaaa agctttctgt tctttgacta aaaatattgt acctccgttt
gcttcttgga
753961 gagtaactct gggtcaggct ccacaaagtc cagttcatcg gagagtttat
gttgactttc
754021 acacctttgg cgacacctgc aggtgaaaaa agggttaaaa agattttatt
tgcacttgtg
754081 tttaattaac ccaccaaaac ttaaaacatc attgtggaat ctaaatggat
gaaagctcat
754141 actcttattt taacctttct ttttgctggt tctcaggtac tctccgggtt
ttaggcagta
754201 gtgagacttc agatgaaggt gccgatgtgt cggagtcgcc ctgacacagc
tgcggcctgc
754261 tgaactctgc ttgtacctcc ttcggccaca agggggcaga aacaccactt
tctgccgcga
754321 gactcttttc acaaacttcc tccgtctcct cctcgctgga gtcgttaggg
cgtatgatgc
754381 ggcggctctt ggtttctccg ccagctctct gctctggcag aacttcagct
gcttccctgg
754441 agcctgcaat cttggccttg tgcaggaaaa cacgccgtct ggtggcatac
tgcctcttcc
754501 cgtctacaaa ggagacctcg ggcaaaagat caacatcttc agcctcctct
tcactgctcg
754561 ttagctcctc cgcctcactg cccaccacaa agctgtcctc tgcatatgtt
tcatccatct
754621 caggcacctg agaccaacgg ttcagaacac ggaagctggt tagataaata
tgtaatgctg
754681 ttaaaatgaa aagactatat tgagcgtata cctgggaaaa tatgtccacg
ttgtgatgat
754741 ttctgtaaga cattttgaat ttgccctgga ctgcagggct tctcactgac
ttcaaataaa
754801 cacagtgcat ctctgaatct gcaggacagg aggacaaact ttgagtgcaa
cagtccccca
754861 tgcaaattta ctgtcccatt ttgacccacc attcaggccc tgtgagcagt
gtgtgttgtt
754921 gaccacaaag ccttcgagag agtgattcag ctcttctccg tcttcctcat
ccgatgacac
754981 atgctgccct ccgtcttcct ctgacagctc tgcctcttcg tccaggaact
gacgtacctt

755041 ttggcgaact gggccctaaa aaagatggga aaaggtcagg tcatgctaca
tcaagctaaa
755101 tttaaatatg tttccgaagt gaaaccaaaa accaaacatc caaacaggtt
ttccaaatgc
755161 tgtgctcaca catgcagtaa acatgttatt cactcacacg ttgataaaaa
caacagaata
755221 aatgacaaat atgtattcag tgtcagatca gtggtttctt cagacacctg
ttgccacaga
755281 ccttgaattt ctcccaatga actggttcac ttgcagctgc cggctttctg
tgcgtgaaca
755341 tggattcatc ctggaaatca tcatcatcag agagagcgcc acccgccacc
tcttcaggct
755401 ggattagaac gggagcatta aaaaacatgt gctgttaacg tcttggcttg
tggtacatga
755461 ttcatttacg acgagctggc agaaacaatg catagacaac gggtcatgtt
ataatcaaat
755521 cagatgcgct tataggtccc tgtgccgtaa cattccgcta atgaagaaca
ccttactgta
755581 tggaaagcag ccaaacgctt cctgttccca accactgggg agtccacatc
gctctccacc
755641 tgacagagac aacatacgtt tttccatctg cactttcctt tcagacgcat
gggtcaatac
755701 ggcttttgtc taataaatat gttcgtgaga aatttcaagg tctaattgga
aagtgctttt
755761 tcttttgccg cttgcaaccc agttgccatg acaactgcag atggtttata
aatgaaggtc
755821 gaaggcagca caagctgtca acacacgtgg accccaaact ttgttgtttc
tgtatcacgt
755881 gtttttctgt ttttatacca aaattgatgt caatatatat tagtttatgt
atataggtct
755941 ctttagacac aaggctacac aaagtgtgct atacggcata taattaaata
catatatata
756001 aaaacatcac attaaaagaa aaagcagaga gcagacttca attgttggaa
gttacttgca
756061 caaggaaaag atttctggct tgttcattgt cttaaacgac acggagtcaa
ggtgataact
756121 gacttctaac cgctcctttt tggggccgaa aacaattatc tcagtctttt
ctgggttaag
756181 ttgcaaaaag ttctgacaca tccagtcatt gatttcattt atgcatttcc
tcggcaaata
756241 taagtgcctg cagtaattag gtgaaagtgg tataaagtgt tgtgtgtcat
ctgtagaatt
756301 atgatagcag atgttacatt attctataac ctgagccaga gagagaatgt
agatattaat
756361 aaaagaggtc caataaagga cccacgggga actccacaag tcataccctc
agtccataag
756421 tccattcttt tattgctaca gtatgtaaac acaatcttgc cctgtcctga
gatgtacttt
756481 gtgcacttag tgaataaaga tcacaatcct ataatgtaat aaaagctttg
cctcggtctg
756541 ttaaaattcc attttctact tgtataactc ttgagtcatg aaaaagattt
cctgaccttt
756601 tacagagcaa atctttaact cgattttttt gtgacaaacc ttatttaccc
tttgggatgc

756661 cagcgggtcg gccctctttg gtcctcttcg tttatggact gcgacttcat cctcactgtc
756721 actgctgcac tctgctggga aagagatgaa gttcagactt caggagccgc cgcaaagcaa
756781 ataccataaa gtcaggaaat ttcttgatgg ggaaaaccta cgtggatgag gaggcaaagg
756841 ttctgcacag acagactcct ggttcacttg atcactcaga cgcctccgtt ttaagctgga
756901 gagcacagac ggggtgcggg gactgctagg actgggcaag atgatccgtc ttgctcgtgt
756961 tgatgtgatc ggggatggga aagcatcgcc tttaggtggg gaagcaaaga aagaggacac
757021 tgacctcctc ctgtgctctg acgacatttg tggtgtcgat aacttgggtt catttggctg
757081 tattggcttt ttgtgaaagt aactgcatgg tgtggaagag tctgcctgct ttagaggcag
757141 tgcggaggca gaggcccaag ggccaggatc gtcctctctg tcttcatcgg agtcatccag
757201 tgaataaccc aggtcaaagt tgacagagaa gaggtcgtga gacttgtcaa atgaactccg
757261 ctgcccctgt agatctgatg tgaccgcgtt gtctgtggtg ttctgggctg tctgagccct
757321 cacctgcaga gcgtcagctg taggattagt tgccgcatcg tctccgtgca tttctgttga
757381 tctgcaggga ttttgagctg cctgttttgc aggggacatc ctaggactga ctccaggagt
757441 ggggacatct gggattgtca tctgcaggaa ggcttcctcg tccccaaaca ggtccaggct
757501 gttgtccatc tgtgggtcat ctttcacgcc atctgtccac tcccacactt cctcgtctct
757561 catgtcgtcc ccacagcttg ggttttcctc ggccatacgt gcgattttta tttcttcatc
757621 catctgatcc acttgtttgc tatcatcgca gtcattttct tcttcattat cttcatcatc
757681 aaaaattgca tcccaggtca gaccccctgc tggctttttg cccttgctgg cgctctgctc
757741 cacgtgagct tgactttggg ggatttctct gttctcctga cccacagaac acttggctga
757801 gtctgacgtc ttgggatcat gctcaggatc actcgccatc acgtcatcgt caacatcttc
757861 aaggtccagc gtaaagctaa ccggaaaagg agggagacaa aacagtggag gagcggtcac
757921 gtccgggtct aagtgttcaa tcagtgatgg aggagattga gacaggagcg cctcaacatt
757981 ggccaggatg actttcagat tcttgtctcc ccctgggttg attttagccg agggggggcga
758041 agatgctgat ctccatttag gaaggtagaa cattgtctga agatcaaaat cttcatctga
758101 acgtacaggc tcgaccatcg tcccgtccgt ttttgtgtca tgctcattga tgataacatc
758161 atcagacaaa tcgacctctg gaaaaccttc cacaggtcct gggccgtgtg tgaggtcatc
758221 ataggagtcc tttgaatctt gaggatgtga aggtctttgt tgtaatgcac cagaaagcct

758281 ttcagggata cctttgagat ctgacgacac ggagcgaggt ggttcacatt tgttgatgat
758341 caatttgggg caattctttg agttcttctt catcttctgt ccctttttaa tatggtcatc
758401 gggttggtgg agataaggaa gaaggtcttg ctcataaagg cattcgccct aaacaaagga
758461 aacatggaaa aagataccgg tgtgttatag tcaacattaa tatccatcaa aagcacaaat
758521 gaaggcacat agttacacaa agtttcatca taaatattag ccaaaacaat attctctcca
758581 tttgaaccca atacacacaa ctctgtcaaa tttgcaaagc acatgacatg gaaatgattc
758641 aacaagagtg gtatcctgca gggactgcca gggtgccatc aaacctgtgg tcagcaggct
758701 caagatgttc tgtgaagcag aaagcccaca taatcctacc agcacgtaaa accctccaca
758761 tcattgtatt ccagtcacac ttctaagtaa ttaaactgat gctaaagtgt aaaaatgccc
758821 gatttcattc cttgatttta tgatcaagca tgaagagagt atgttattcc agtcttccca
758881 cctcctggtg ataaatgctg cgggtcaaca tttatacact cctggcaggg gtacagtgct
758941 gacaagtgga gcgtgttgtt agtggacaac atgctgtttc tctctgaccc acaaatttcc
759001 ctcaaatcaa atgcacttca aaagtcttgc ttttgtttgg aaagtacggc taagccagcc
759061 tggacaaaga catgcagagg cagaaatcgg actttaatgg gtcaaactct gcagaggtag
759121 acatctttca aatataagtg gaaatagctg taccagagtt cagctctcaa tatgtacgac
759181 aattactctt ttaacaatat gatgacacac gttcataaaa ttcatttact gcatatcagt
759241 gttgtttaat gaacaactaa ttcaaatttg tagccagcaa tatgtttaaa cgatctacca
759301 tgcatctgaa tctaactaaa cttgaaaatt aatatgtcaa gtgatttatt ttaacctaaa
759361 ataactttaa aaagagatac atacgtcttt acagaagatc acaaaatgaa aacaaaatca
759421 atgacaactt tgttctcttc tcttttctca tgagacccca tttcacttta aggcactctc
759481 gaacagttgc tgtaggatta accttaaccc ctggcccaca tttaattaat aatcagatta
759541 aggtcctgga tcctgacatg ctgcagtctg aaataaactt aatcacgagg atgcagagaa
759601 aaggcgagtg tggtggaaag gttagatatt ctcccccaaa gtgcaggatg agctgactga
759661 attagagatc tgaacttcct ttgataaaca tccaacactg tgaaagagga ccttgactga
759721 gcccaaagtg atggctaaaa acaggttacc tgcatgtgaa ataacctgtt aaaggaagcc
759781 agtatctctc tattcacagc agttaaagct aaaagtgagt gattggttcc tactatgcca
759841 gctctggaca ttaccctcag agacaaactc tcacagacat cgacagcggt tggcagtgga

759901 tgacacaggt cttggaagtt ctcatgcgtc tcatgagctg agcaattttg cagccgggcc
759961 tctttggcag aaaccaaggt tttcacctgg ttcctacatc ttaatccaga ttctgattta
760021 atagactgga gcccatgaaa gcaccatccc ggaagaaaca aagaacccat taaagaatat
760081 gccgttgcat acggtttcat cacttaagaa attagattga tttgtctttg tcatcttctc
760141 cgtcagcacg ctgcgcatgt aggccaacaa aacctgacct acttttagct tcctggtttt
760201 atgtaatggc tcagagaagc ctttcgtaat cacagcagag gaggccgccc gcttgtacgt
760261 gtgctctgtg tcctgacata aagtgaaaag caagacggtg aaaaacaggg tggttgtgaa
760321 cgcaacctgg gagaaaagtg cctcgaattt gaaggatggc ttcgggcaaa cagaggctga
760381 agtgaagggg tatgaccggg ccaaaaaaaa aaattgaatt gaatttaagt gctttattgt
760441 aaatctgttt tgttcgccct taatggcctc tttgcgaatt caggtcaggt gagtaagctg
760501 ttgcaggtta tgcttctcaa agtgtacaag caagaagcag cttcattatc cacattttgt
760561 acacatttgg aaattctgtg ctgtatggtg ctttttttcg cagtatattt aagtaaaaca
760621 gaaatcacaa atttaaactt atttactgca taaaatataa tcagcagagg cttgacgata
760681 tcatctttgg gcaggagctt aattatgacc tacctgtgtc agataacctg agaaacctga
760741 gaaaatacac aatggtgaga tcattcttgc tcgattagat tctggagaaa ccgaataaga
760801 ttccccagct cctgctgacc ccctgacctg gctgacatta ccgatccaac attgccgcta
760861 taacatgtag cagccgagtc cagtaaaata cggtcagtaa aatgacagta ccgttaaacc
760921 agaattatga gcgggataat caaggtcatc caactttaca aatattttag catcagctcc
760981 acaacacagc cctcgtgttt catctatatt agaccctggc tgctgcgtaa tgtggtttct
761041 gttttaaaag aagagctttt gctttttatt ctatatttat ttctgttatt ttttaacttt
761101 gtttgagaaa atcagataaa gagcaatagc ctgaaaaaat cctgctaaag ataaaaaaaa
761161 tgtaaaaact atgacgacaa caacatcgtg gagcctgttt atcacattcg gaatgtgcct
761221 tcgaggccta attgatgaat gtagtaagac acagttgagt gttcacatga ggctcagctg
761281 actctttgta gcctgcggag cttataaatc tgtttttgtt tcataaatgc agttacattt
761341 tgctgtgaga gtgggactgt taatagccat ttctactctt tttcgatcca agaatgtgat
761401 cacatggtat aaacatttga cccttacctc attttcctct ttcatattgt caatgagctc
761461 catcacctcg atgaagtgct ggcagcgggc tgaatgatta atcacatggg ttggaagtga

761521 tttgtgctgc cagtgtctcc attcagacag agacagctct ctggaaaccg acctctgagt
761581 cttagagagg ttgtcctttg ggggatcagc aggcagggac acgaactgtg actgttttag
761641 gactggatga ggttcatctt cccgcagtct caccgtggaa gcccacttgg aaaattcagc
761701 actgctcaga aatccatctg atgctgtgct ctcctggtga actggcacat aggacagaca
761761 cagtttgaga aattccatgt tcggctgctc caataagaac atgtattaac cggaattcac
761821 tcgccacctc actcactcaa gttttgaggg tggatgagag acgcccgtcc ctcggaccgc
761881 gaccttcttc ctctgacgga tcgcctgctg ctctcctggt ggtcaaactg gccacaggtg
761941 atgtgcatct tgtgaagggt tgggatcagg ccctggggca gcatgcgggg actactaggg
762001 tacatgtgga acctgcattt gttgccagtg attgacttgt acacgttgcg cttgttgctc
762061 tggctctggt tgtacgtctg acaaaggtaa acagtttgtt ggaagtagct ctgatgtggc
762121 aagttaatca tgtcgaggtg catcaaacct ttggtttcca ctcacccttt cttcacgccc
762181 ctcagacaga atgacaacaa ttcgcccctg ccgcttacga ccagtgcggc ccattcgctg
762241 cacgaggcga gtggggttct tctgtgcatc aaagcaaacg atgaggtcga cctctccgat
762301 atctagcccc tcttctccga cacaggtcga caccagcgtg ttgaagcctc cctgacgaaa
762361 cctgtgcacc accttaaaca caagaataga cacacaaaaa aagacaggat ttttttaagg
762421 caaaatgttt atcaatattt accatttcca taaaatcccc tagtcatttc tggtttccag
762481 atatacgtcc taactttgtc atctgaagcc ttctaataga taaaactccc cacatgtatt
762541 caggtgaggg ccttttacac aagcatcaat ttttgcaagt tctgtgaaaa tgtcactggc
762601 tgcaaagatg cattgctaag caaccagcag gaaaattatc agaaaagcgg ccacagtaag
762661 tagttttaca agaacacacg tccacagcac gattaccaca ctgtggctta catgattgcc
762721 aattacattt gctgtaattc tcagtggatc aatgagtgtt tctcaaacaa gcatttctac
762781 ctccagctgc tccttctggg tgaagccttt gactcccttc ccagccgatg cttgacccat
762841 aaaagtcatg acccttatca gcggagcatg gcggttcagc atagctgcaa tttcctgtac
762901 gctctcacgg aacgatgaaa agatcatcac gcgcgtgctc acctcctgcg tgccacaatc
762961 tggaaatgca taagaagaat ggggaaagat ttattgcagt actgtagaca cagcttagct
763021 ctccaaatac aatataatag tttaaacata atttagatat gaactacctg cggctataaa
763081 gatcgcgtta atatgttgca ctataagcac catgcagttt cctccttgtt gttttagaac

763141 actaagcgaa ctatagatct cagctttgaa tttgaactcc tcctgcatga ctgagtcata
763201 tcccttctcc tgctccaaca atacctcacc ggttttgtct gcggagcttc cagcccgcag
763261 tctgaaatgc tgcagcacca catcctccag cttctgcagt ttggggtgac tgtatatgaa
763321 tggctcgtct gattctacaa gtattgggag gagagaatgt tattcaaaca tatattgttt
763381 ccaaggacct tgtttttatg ttttaattgg atgccccaga tatggatatc tgatgtgtca
763441 cataccagca gactgcttta caaacattgc ttccatctct tggtaaagat ccataaatac
763501 aggagttctc tgcagctcat ttctggccct ggacatttct gtgtcccacc aagtgcaggg
763561 aacatgtgtt acaaaatctg aaatatgcta cataggttgt tattaaaaga tcgaaacact
763621 gcactgatga aagaagcgtg caaatgtggg ggaagaaaca cgctaataca gcctgaaggt
763681 aaggcgactg acctctggat ccatccatga tcccctggaa ataaaggaag agggatcgga
763741 gccccatctg catcaggagc tcatagccat ggtacaggct gatgcaaagg gcaaaatccc
763801 cctctagaac tccctgctga ggaccctgtg gagagaagaa ctctcaacta tcaaccccac
763861 tatccactga aaaaatataa gcaaacaaga atagttagat tatagcataa acttcaagct
763921 cagtttaaca agtacagtgc aactcccttc aaaaccacca caggatttac agtaattcac
763981 atttctccaa ctatgcagga acaatgagag aggtttaaat tgaggtaaaa ctgtttcaca
764041 atccactttg agaagactgg cagcttaaca cacaactcaa tcaattgtaa tttgtggcag
764101 aagcaaatcc actgtgcaca aaaaatctat atagttataa aatatcagcc agtgcagctg
764161 atatcaataa tctgcatact tcaaaatgcc tcactgcaaa aacctggtga atctttttat
764221 gctgacaagc tcatgatacc aaattgattg aataatgggt gcttagagtc catgccttga
764281 tgtgcggcag cgggttcttg cggaactgat ccctggcgag gatcagctga tatttgctga
764341 gagttcgcag gtccttgtgc gccatcaccc tgctttgaac caggcgagat gtgaatttct
764401 cgagcacctg caggaggcac agacaggagt caggtcataa ctaaagagcc atttcctgag
764461 ttcatgaggc atgaaaccga catattgaga aatgcttttt tttttaaaag agtccaacta
764521 tgaataaata tcaaatcaaa tccctattgt gacattggca gccaaacctt gaagcttaat
764581 ccctcctgaa actgtgactg attttgatta aacaaatgaa ggatgtattg ctacattaaa
764641 acaagtttta gtccttcata ttttattttg gtcctcatat tgatagaata tcagagacag
764701 gatcatcttt attatacaca ataatgtgaa tacttcttaa acagaatgga tttaatagcc

764761 tcactttgat caacagccta aaaatgagtg ggcctattct tttttccagc
ctcatctact
764821 aatacatgga ggagagatgg aagccatttt tgtggcacac tgctgtaaca
tctattcatc
764881 cacaaggcta gatgtaatgt gagtaagtgg gcttacattt tgtgatgcaa
tttagttttt
764941 aaatggtgga ggagagaaaa cagagcattg ttgtgtggca tcgtgtttgt
tcttgaaatt
765001 tacaaaatgc ttttttaatt tcattgttca tttgcaatat aactcaggtg
caataacact
765061 gattgtttct gatcatacag tacataaact aggacacgct ttcactccta
aattaaaaaa
765121 agtacgtgaa accagctgtc tgcccccatt cttctattca ttcaaaagat
gggagaaatt
765181 tgattctaca ccaagcttat gaagataaag gcaaagggtt aaataacagc
ctttaattcc
765241 tttagccttt aaaacaatgt tcagcagaat tcgacaggta catcaaccag
ctcaaaacta
765301 tccatctata gaagataaag aggtcagtcc attattaggg ctttgagtca
gctcatatga
765361 aaggtttttc attcaaaacc gtacgtaaag gacggttgcc taaccaaatc
tgttgacaaa
765421 ccacttgtaa atcaaaggat aaaatcaaaa tatattgtgt atatgatgca
tgatggagct
765481 ggacctgagg agcaacaagt atttttaaaa gaggacatca catacatttc
tgtttacagg
765541 acgagagaaa aggttactct agctatattt ttaatgactg aataagaact
atgactacac
765601 aagtatcctc tctcatgagt gccagtgatg taaggccaaa cgcaatcagc
atgttgtggt
765661 gtgtgtgtgg gggtggatta atattgaggt ccactgtttg gcagggggct
attaggtcaa
765721 tggctcccca tagagtgttg tgtgtgtttt tcgatacatg ttaaagcttt
acatccacgc
765781 tcacccgcag actagatagt tgcgtaatct ctttgctacg tgcacatcga
tgggtgcaag
765841 atggtggctg gctgctggag cgtcaatgat gacatacaaa attttgttta
gtgccctcaa
765901 gcgaacaagt tcattatctc tacaagggaa ctgtttgtct tccacaacta
acattaaaaa
765961 aatatatcaa tagaacaaaa catgaaaaaa attgacccaa agcaaaaaac
acaaaattaa
766021 ctgatggcac aaatagaaat agtttatcct gttttgtttt tttttacact
atactgccaa
766081 attattatta ctaagatatt catttcttgt tcagttcaat aacagtaatg
ttaccctgca
766141 gcagggcatt tcagatgagc taaagctttt aaaaggtata caatgctaca
tcaattcaca
766201 ctgtgtgcat ggcaaaaatg tctgatattc acagtaatta aatgatcata
agaattaata
766261 ctaatctaag aaaaataaag atgggtatca cagtgagttt tcatgttaag
atactaaggt
766321 tttaaattaa ggggttctgt tttggtatgt tttatctgtt tgactgtggt
gtcctttaaa

766381 tctgtgactg ctgcctttaa atctacaatc ctagtctgtt taaactgcac
cgcgggaaga
766441 aataaagtgt tttagaattg atttcaattg aattaatttc attgtggttt
ctggttatca
766501 tatattttga ttactattta atcctttgac ctacagttgg tttgtggata
gattgtttct
766561 ccaggtaaaa atgttaaact cataaaacaa tagtatttgt tgttaacgtg
gtcctatcaa
766621 cacagttctt atttttgtgg ccctataaac caccacaaaa gattttaaat
gaaacaacag
766681 tgtatgtgtg tgtgcgtgtg gggggggggg ggggggggtg ttgggacaaa
cataaacaat
766741 cataaatcaa actttcactt tttaatacat ggctgcaaat catttgcgtt
cagcaagtca
766801 aacccatgat tcgggtcact gtaatgactg taaattaatt acggctgaat
gacttatttt
766861 tcgctccgac cgaccggaag aggcggagtc agagtggcct gatcacgtgg
tctatcgtcg
766921 ctcctcgtaa acaactcaga tcgccgttca ggcctcaaaa acagtctcgc
caggctcctc
766981 cgcacagtcg acgcaggcga tttttttgcc gcctttaaaa agagaattag
gtgcaccttc
767041 aattggggtc agtgtgcgtt aaggtagtct aaagcgttag aaacgcgact
atggcgagct
767101 ttttcgagaa aagcgtcgta ataattggtt gctacagcag aaagtagcgg
cactccaatc
767161 ggaaaaagtc gcggaggtcc ggagcgcaac acatcggcgt tcaaaagacg
cgaaattaag
767221 tgagcgctga aaggctgttt ctttcaaact caacatcaaa tatcacaatc
ctttttttcca
767281 aagcgaagtg tcagatcacc gctgcgacga gtaaaaatgg accgcggtgg
ctgtcaaatg
767341 agcggcgggg gtagcggtgg ccgcatcgag cccaaccaac ccgagatgat
cacgctggag
767401 gaccttgagt ctttctccga agaccagctg tcggactcgg gcaacggcag
cctggaagaa
767461 gaggcagaga ccatggagga agacgacgaa cggctgctgc gctactggca
ggacgtagca
767521 agaggacacc aagtagaagt ttctccaggt aaaaatgtta aaaacgacga
ggaactcatg
767581 cgtaaaaacc cgttcgtgtc gatgaataat ttactgttct gtttagcgca
gccattaatt
767641 aacaacgtat aatgtgatga gtgtaaaatt aaaggaggca atcacaattt
ggcatgtctg
767701 acatacaatc cgctgtgcaa ggctgcgcac ggtgacctaa atacaccgcg
gagcagcaca
767761 atccttccgt ctcctgtaaa cacgagttaa agcttcgaaa aatccaatca
aattactttt
767821 gcagcattcg aggacagcac accgacacta aagaaagtaa aaaggaagtc
aggactgtca
767881 gtctccgggt ttgttgtgaa ttgcacgtaa tgtttgagtt catttaattg
catctaatta
767941 atgcaattaa taacataaaa caacgagttt acacgcacgt tttgtacagg
taaatcttat

768001 tggacgtttc agcgaaaacg tatttccata aatgaactat atatgtattt
cctggttttg
768061 ttggggcagg tatagctata caatgattta attatatata taagagaagt
tttctttaat
768121 ggtatctttg ttttgcttcg atggccaatc tattatttct aatatgcaat
gacgtgtact
768181 ggtgttctat atgtttttc ttcacttcac agtgtgttta atctatgtaa
gctgagtgga
768241 atgcgcttgg ttccgacact gagcagctgc tcagtctatt cttttagcct
aacacctgca
768301 tggcctcaat aagtctgttt tctttcgtaa atgtactgta ttaacagcct
attgtcagaa
768361 atgttaagaa ttgaaagtcc ccatactctg ttgatgttga ctgcactatt
tttctctcta
768421 agtcttttca attatattta ccacaccttt taaaatgggc tattttttt
gtacatctgc
768481 aatcacactg tgtcccccag cttcttagaa tcaccacagc taaagcagcc
tttctgttga
768541 cggtggatct cagcacggtg cgatagccac gtgccaaacg tccaagctgg
acgccaatta
768601 attcctcagc tccccaagta gtgtgacgtc agtggggcga cagtggcgct
cactaattaa
768661 ttaaatgaag atgagaaggt gacgcaggaa ggtttgcatc tcttagatga
aagaggctga
768721 cacggaagtg atctctggca aacagcaggc tcacaatata ctttaattgt
agccagaata
768781 aaggggtaaa atggggcatt tcataagctc ttcatcacag tttctttctt
tctttttttt
768841 ttaaatccat gaaccacgtg tcccaaacag tgaggtgtag gacaacccgt
tcttccagga
768901 ttttgttaca ctcctacata caagtgccat ttaatcacca atactggatt
cacaaacctg
768961 aacaacgtgt ccatttaaac tagcaaacct catcatatta atttttgctt
tgcaggaata
769021 caaaatcttg ttgtgtcttg taaagctcta aactaatagg tgaaaaacca
aaaccccctgt
769081 cttaaatgga cctgtattgt attattttat ttttttgaa aatagaaata
gtatgaaggt
769141 attcaagaaa agttgtacaa gtctgaagaa gaaaaccttt ttttcttttg
ccgaggcatt
769201 gggagggcag gactgagccc attgtgctgt cttatgtttc aatcacactt
tttaaacttt
769261 agtttaaact ttagtttaaa aagtttgagg caatatcgca tcaagctctg
tggtcaggaa
769321 cactggcgga ctaaccctac ccacacaaac cgaagcaatt aaaatattta
gccacttttg
769381 tgttgctcct ttacagttga tttatcatca actttgatat cttgactata
tttgatcaga
769441 gaagccggcc attgtaagta aagagaatgc acagaggcct cataggttat
taaaaaaaag
769501 cgtaaataat gtgtttcata gtagcaaata tgtttctaca ccacctctgg
ttgttcgtct
769561 ggggtttatg acatgaaaat cagatctgta tatattttcc ccagttaaga
cacataatac

769621 agaaattaca tcgataggac gtgccttgat atgtactaca gccgtgcttg
tgttgtgtca
769681 tgttttggtc tcctctgtta aaatgttcct ttcaagtgtg tattgtgcac
ggccctgatc
769741 gccctgagtc gtttatccta agcagacatg aacagagcct caaccgtgtg
aaaactttgg
769801 atacccgaca gcagctttgt taatcatccc atgattgatc atagagcaaa
cggcacagat
769861 gtctctgacc ccattgtcct ccatcatgtc ggctaaagaa ctgcgcttga
gcagggttca
769921 cttggagccc cacatgttta ataattcagc aatataacag actttgttaa
tgccgcatgc
769981 ttcagtattt tgaacggcac agactgccgc tctggtcgat ctttgctaat
acatctattc
770041 ctggtcttag gcagctttag tctattagag gtttagctca ctgcaacatc
ctggcactgc
770101 actcggccca tctggcaatc tttctacaca caaacactgc ctgctgcaga
ttagtgaaag
770161 cagagggcat tctgcatgga ttataagagt gctgttaaaa aaaaatcaaa
ccacggtgta
770221 actgtaataa gcgcttcaga atatgtgtat atctggactg tcaaacaccg
tttaacctat
770281 tgacatgtgt ttgggacaga tatggcagag ccgattcaac agctcaccac
caacaaccag
770341 gggcagagtc agagagagcc cgtccccttc tctctcctcg cacgcaaaga
gaaggtatcc
770401 cctgccgaga tcaccccaac atctttaagg atttaaaatg aaaatagacg
tttctaattg
770461 ggaagggacg gatgggtctt taatggtgag aggggaacgt ctatgcatgt
cagcgggcac
770521 ttatgatgat caggtcagcc acctctaaaa gccattcaag ctccttaagg
agatttataa
770581 ataaagagga ctaattggct taacttgagg tatctggttc attttatctt
ggctggagcc
770641 tgcagttgaa ccaaagacaa tcatggaaga ctattcttag tgcaggttcc
agagctgtgg
770701 taaagctggt gtgatgacag cagatttcaa actgcactga aggtcgttca
cctgagctgc
770761 agatgcatgt gagaaattaa aaagtgattt tttttttaa aagagagaga
attctagttc
770821 taaaagctta gacttgtttt tctatattta atgataactt cgtacctgtc
ttcctttttc
770881 ccgccagtgt gggcagcttc tgtatgagaa acgcacgtat gaaaagggcc
actgggcttg
770941 cataatgatg cgtgaggaca cttatgaaca gagcatctgc tacggtttta
tgaggataat
771001 gaaatacatc tgccagcaga attccctagg taagtttaaa gcatgtttaa
agcatagtgc
771061 acagtccctt ttccctgcta tgtatttaat ttgcctggga ttatcttcag
cctgtacatt
771121 tcatggaaat aataacaatt tcatttgtaa tgaaagaact gaagaggcct
cttggatgag
771181 aggcgaaacg tcttccagtt gctttcaaca gtccagttga cgcttcgctc
gacctttatg

771241 gaatttcctt tgtaggagat tatctaggca tgacactccc catcatcaca gtggtgcgca
771301 cggacgagaa ccactccgtt atatcccgcg acgttactgt tgcatatttt cttccaactg
771361 agcatcaggc ccagccacca cgacctaccg acagcgagat cctcatagag atctggcccg
771421 ccacaactgt gtacacaagg tgggcgaata aagaaaaatg ctggtactct tctgttgtca
771481 acaagctaca atgctcataa caatgcacca tgccttcgtc caccagggcc tttaccggtg
771541 ccactaatga ggtcaccatc attgaccaga tcaataccat ggcggagctc ctagactctc
771601 ctggcctgtg ctttaacgac tctttcatca tcgctggcta caccaaccca gcccacagca
771661 atcgggccaa tgaaatctgg tttctagagc gaccctgagg tgggggggt gggggggggg
771721 ggctttagca ttatcaactg tacgaccctc aagcctaatt aagactccaa tagcactgcc
771781 gcaaaaccag tacagtttca ttcaccgcct atacctgaaa cgaccaccag ccgagcacac
771841 tacacgtcag attatccgtg gcctataact gcagacaggt ttcctcccta ttgtgcagcg
771901 ctctacatac atgttgacca cagagtgctg ccagagacat tcaacaacct cataatggca
771961 gaaagggttc ccagtagaca tccagcctta tctatacgtt cggtgtgcgc gttgcagcta
772021 cgctgtcctt tcgtgttggg gtaaaggtca gtgtacaaca gaaatattgt tttgctggca
772081 ttcttcatgc ttgcttctgc tgtcgtctct gcactatgtt aggattctac gtgatgtcac
772141 attgcatatt tatatttaag aataagtgca atatatacca gatggcagat ccagcagcat
772201 gttagtacat taatggatgg ataaaattgc acagaggagg tcatgtgcat cacagatgaa
772261 catgcctata atgattgttc agaaggggta taatgagtga atgtatgagg taggagaagc
772321 tgtaaaaaat atcccggtaa tacaaaccgc tggaatctca acgattttac tgcttaatga
772381 caatcaggtt acttgcattc gaaacacatt atcttcaggt aatagttgct aagggtaacc
772441 atatgtacag aaaatacctc tcttcccttt tctcatgctt tctctacctg ctactgtgtg
772501 aatgttggca aacgtgtgca tttactcttt cctgctgctg cataaaagca agagaagacg
772561 gaccacacac ttaagctgta cagtatacgc tgactattct ctcaaacatg tgatggaaat
772621 gtgcatattt tagtttatta taaaaaataa ttataaaata aaaatgattg tttaacattc
772681 tattaccaat caaaatagtc tatgaattca tggtgaacat atttttctca tatgcagcat
772741 gtataaagtg atatatgtct gaccagcaac agcattaatg ttaaaatcct tatttactgg
772801 tttcaaaatc aatatctgag gtttttgggt tttgtttttt ttcggggggg gatcataata

772861 aagtggacaa taaaggatgt ctattcaaag tgtaatttta tgcgtaataa acatgtaaag
772921 gaaatatata tatatataaa ttaaagagtt aaagctagtg ttgggaagag gcaacaatag
772981 tagctggttg acgtcgagac aacaaatcca aagaatttct tttgcattaa gcattttgta
773041 gagaccgtaa agatattctt attacactta caaagccatg atgtgtgctg atttttcctt
773101 ttcagaattg tctcaatttc ccctttcctg tctcgctgct tttttttag tgttttgcaa
773161 atgcgtgctg tgcaaatgtg aaagttcaag tgcaacaaca tgacctgtcg tggacgtctt
773221 tgttcaataa aatattgtct cttgtctttc aaaatgtata cctacacagt gttgtcgtca
773281 tctatatgaa tccttacaga agggcaggtt tcaataaatc agggtgcaga tatagtgtcg
773341 attgagaaca catcaaacag caaggaaaat tttacttagt ttctgccaga ctttaagtaa
773401 aagcagtatt atatagttaa tgagccaaac ataacacata gccaagtata gtttcaaata
773461 aacttaataa aatcggaata aatgcttgca tccatcattg ttcacacttt tcacttattt
773521 gcttttctat aaagttcaga taatacaaca aactgcaata aataaagcta tatgcccaac
773581 aaggtgtgaa atcaatgtga ataattaata aacatacaat agcacgaaac tttcccttgc
773641 tgttgtcaca ttccagctgt ccagccagat taaaaccaca gaagaagcag ctgagtatag
773701 acacttgaca agcagagctt gttaacgttc cagagctgaa taccagaccg ccaattgcga
773761 caggacgctg ctttcaagaa aagttaattc aagtaatgtt aactgaaccg gtcgaaccga
773821 caagaggaaa aaacctcagg ctaaaaatgg gagctgatga tttccaaatg atttgtaaat
773881 gaccacaagg tgtcagtaaa gagtcaaaat acaggatacg ctttaatgcg ttgtcacact
773941 tcaactaaac ttgccctgtg agctcgactg taaatgcaaa ctgtattcag atcataagtc
774001 agagtgattt taatattctt taaactctga tggtgaaatt aattttcttg ggccgaatag
774061 aattttcaaa ttacaaggtg caacttggat cgtgcctacc tggaggtagc gtgcctggtg
774121 tgcagaaagg atctctccca gaggaaccac cactttttct acactgcgct ggtgagaata
774181 ggccctaata tctgggcttt catctgagcg tagctcaata tgggagatga gcaggttcga
774241 caccactgac tgcactgact gcagaaaatg aagcgaaaga aaaaacaaac agcatgaatg
774301 tcagtcttag aatgcacctt ctgtaagaaa caacatagat aaaagtcacc tttgcatctc
774361 ctcctggcgt agcagttaga gccaggacgc ggaactgcag catctggctg cagagttgcc
774421 tgatcacctt agagatagaa agaggagtaa ttacagggta ttatcaatgg ggcaatttgc

774481 ctgaagttaa aaaatgctgg ttgtgggatg gatggtgttc ctgagttcct gggtgtgggg
774541 tacgacagat aaacagcttt cactgataac aacggcaaca tagctcagag atacagcata
774601 gagtgggata tatatgataa atgatacaat aaatgatgtt tgaagaatta tttgagtgtc
774661 aaaatttaag aacacgtgag agcaaattta catatttgtc tctccttttc tttttgggcg
774721 agtgagggcc tagtacagta tagctcgtgc attagctcct gacttgctag aaacagcctt
774781 tgcaaaggta gtctctgatc ggtatatgac agatctgttc acctgacaat aggcatggtt
774841 gccgagcgcc ttgtgcgctt cgtcgatgac cacgcacttg acctgttggg cagggcacgt
774901 ctctcttgac aagtcgttca ccatgacctg aggggtgagg aagaagacac gcttggccct
774961 ccaaatctcc tgccgttgct ttgctcctgt gctgcctaca tgtcacaaaa tggacctctg
775021 taaagacggt ttaatgagaa tatatgatgc tagtgtcatg atcggctcac ctgtcagctc
775081 agccatgtgt gcctgaggga tgcccatcac tttataacag gcctcaatct gttgggccac
775141 cagaggtttg gtgggggcca tgaatacaat ctttcctgac ggataccagc gataaaagtt
775201 gtacataacc acagaggcta taaaggtctt tcccagtcca gtggggagac acaccagtgt
775261 attctgaaat agtgcaactt ctgacatctt cagctggtac tccctgattg gatagttagt
775321 ggggtatatc caaactttag ctgaagagct gtcaaatcca ggaaggtctt ggtacgtctc
775381 agttgaaggt gaggctttgc tccctgtccc tgcagagtcg tcacgctgaa gtgaatgtcc
775441 acgatcatgt tgcaagctct tttcggcctc ataaacagcg accaccatga gatcgtcgtc
775501 gtcgtcttca aaagcaggat aagcttcctc tgtcctcact tgatcttcag atgcgtgtgc
775561 ggacctctgg cttatttcag gccaaagagg gtttccagta ggttttgctt tagccgcctc
775621 tgttgtgttg ctctgggtag tttgaggtcc tccggcaggt ttcctgccat cctttgtcgg
775681 ttgaacaact ttactttgat tattggatcc accccaagtc tggaataaag ttctttgatt
775741 caaaccttta ctcatttttg ttatttgctg tggaaaaagg ttgagaaaag gtcccatgtg
775801 acgagtagca gcatttcaac aagaacacaa acacagacca cttccgctct ggttctacca
775861 aagcaaaaca ccgtttacat aaaatgttct ttttcaactt ttttcaacat atattttatt
775921 tttaaaacat tattaatgta tatttatgca aatttattga aattacagaa gaaaaaaaca
775981 acattacgtg gttaggtttt gcaatgaagc ttttaatgtt ttgagtaaga caccggaagc
776041 aaatattttt ttagttcctc tcccgctcag ctgtcataat gttaccatag taatggacag

776101 aagcaatata gagcacatac ttacttcttt ccaggtacgg gcggtagtta ctacatctgt
776161 tttaatttat aaatattcgt gttcattctc acagtagaac tcgggttatt gtgttgttat
776221 acttcggcta ggatcactgt ttttgctaac ccttgaaagc tgctagttaa catcgctagt
776281 atccccagtt aatctgtccc cctctttagg catatgcccg tggatacttg gtgagaaaag
776341 aagttcaacg tgcaagagag gactttgaag ccgtcgtgaa agagattgat ggcggtttga
776401 cgcatttacg ttggaagaag tcaattatcc ccgtccccca cttcaccgac acagtaagtc
776461 aggatgatgt ggtttaggcc ccctttgtgc atttttaacg cgtttgttta tccgtcatca
776521 ggatggccct ataataaaga ccaacagagc tgcaagcaag cctccaaatt ctggaccagc
776581 tgtctttcac cctctgcgga tctcatcaga aagagcgcca gcatctgcaa agggagagaa
776641 tcgtgcacac aggacggagg cagaacgaga tgattcacgg acaaaagaac ctcctcaacc
776701 gacgaatacc tgcagtgtca cagagggttt ggaaggacag aaagaaaccg gtttggactt
776761 taaatgtggc agaatgattg atagtgcagg aaattccact actgtgtgga gcagtttgga
776821 gctggacgtg aactgtggtc gcaaacaaaa aggttagagt tctggacatg gtttgttttt
776881 ctgtcttgcg tcagagacat ttgacatttt gaatttgaat atttaaggct ctcggcagta
776941 ttgcttggcg gaggacgtcc ccggcaaccc agaggctctg cgtctgcaca gaaacgccct
777001 gatcatggag ctgctgtggc tccaacaggc cattgacagc aggaagaagg tacaaaaagg
777061 gccatcatta ccgctaaagt ttacactagt taaatcaaag ctgtacacgg aaaagcttaa
777121 attcctttac actgactgag gtgtgtagaa taatttttta tgatcatgta ctgtttcttt
777181 ttgcccaatg agtcttagca tcctttccat ggagagtaca ggggaccaac ttaagcatgc
777241 atgtcgtgat ctgtgttttt tttacagtac ttgtcgctga agaacaggct cagtgtttca
777301 tgacagtcga cgtattcgag agtggacttg atgacgtgag acaaagacca gaagcagctg
777361 atctcattca actcacacct gtctgtttta tttgcaaatg tggaccgtaa cataattgct
777421 actctcgtaa cattttgcac attgtaattt ttttctaatg atgtgacaat aacaataaat
777481 tactattttt tatttttttt ttaaaactct tgtctgcttt tgatgatctt ctcttttctc
777541 aaagtcgctg ttttccattc agtgtaatta aatgacagtc cccagcccta cgtaagcgtg
777601 catggcaatc cagaagatca aatgtcccat caggatctga ataaatgaag gatcacttgt
777661 gcgtaagagg ttctgcctgg aagctcacac actattaagc gtctgcttta ctgtttactg

777721 agctgcaata accttgttct ttgtgttttt gcgttagtta aaatgcatat atgagtgcta
777781 atgttattaa ctagttctaa cacttgtttg ggtgattttt ccataaagat ctattgtgcc
777841 tcttaattat tatgtgtaga aacacagatg tgatgtaaag gttatcttat tgatttagct
777901 tacaacactg aagtgcccaa tccccccccc cccctctatc tgcctctttt gttgtggttg
777961 ctgatggttt ggcccacaaa gccagtcatt gctgtcatgc tgccatagca actaggatat
778021 actcaccacc gtcagtggag tagcgtgtgt tttaaggggg tttgacggag cagaaggtgt
778081 cgagctggag tccaggcaca gacggcaaga aaaccgactg aattttatat tttatagttc
778141 accaggtcgg tgagtcgctg agcagtgtgt gtgtgtgtgt gtgtatggat cagatctttg
778201 gtgtgtgtgg ttgttaaaaa agtatgagtc aaggaatgtg gctggtaagt attaaggtgt
778261 gtgttcacag gtggctcatt gatggcttca gtgctgcctc cagtgaagag cgtggtggtg
778321 tacaaaaatg gggacccctt ctacaccggg cggaggttcg tggtcaacca gagacaggtg
778381 gcaaccatgg aggcctttct caacgaagta acccaaagca tcagggctcc gctcgccatt
778441 aggaccctgt acacccccag acaaggtcac agggtctctg acctgcagca tctccagaca
778501 ggagctcagt acgtcgccgc cggctttgaa aagttcaaaa aaatggagtg agtaaatgtc
778561 agatggcgca gtcacgagga cctggggatg gagaaaaaac actttattgt ctttgtgtgc
778621 agttacctaa acatgggggt taaaaagcat ccaacggctc gtgaagaaac acaggtatgc
778681 ttctgttttc acacacaaag tttgatatta agagcagaaa accatcactt attaatctgg
778741 ctaaccagga ggaatgcagc caatctgaaa tgctaaaagc gacgtgagag atgtgatgtg
778801 tctgggctca cgctgtcatt ttggcccaaa tctgcaattt gtacacaaaa tagcacaaaa
778861 tgcttgtact gcacatatca acaatggcca ctatgcactg agcactttca gtagtcatgt
778921 gacttgaggt caaatttact cttctttgtc atcacagtgt gatatttccc aaggggttat
778981 tcctgtaaaa atgaaggtta actcattact ggataaaatt attttagaat ctcttttact
779041 catgatatat ttggccttta tctcattatc acaaattctt attttaatta tcattgtttg
779101 attgttttca aagtcataat aatgagttta aatccctcat tttcacatca taccttatgc
779161 ctttgtcact gtttttcaca taattgatat tttattcata aattaatttg gaccttttta
779221 atatgatgga tttttaactt tttatcaaaa tgctgacatt tatttttttt aaatacaatt
779281 gggcttccag ttctaaacga cagtctgatg atacagtgca cgtcagcaga tgcaccggtg

779341 ctttgtactt tgagcaggtg acgggcatct catggttctg ccctatccat ctatgacatc
779401 ttcccctctt ttaatgtcaa acattaaaag aggggctgga acatgtgtgc cttgggcctc
779461 tgccatttcg gagagctgtg catctggttc taaactctgg ccttgtgttg tggttgccgt
779521 gcagacgtga ctcatattta attccattct agaaacagac ttaatctccc acgcagactt
779581 atcaggggtg tgggcgtgcc tgcagagacc taatcaaagc ctccgtgaac tttgtcctcc
779641 tcagcataag aataataaga aaagacatct gattgattcg gctctgtgga gtcctgcaga
779701 gacctcagca caagctttta atgatatggg acaacatgtg taagagctct gagaggagcc
779761 ctcggatcag acaagatgtg tgaaagtact accagagccg tcagtttcca tggtggcagt
779821 acttgagctg acatgagcag aatggttagt tagtcattac actgctggaa tgaacgcttc
779881 ctctgcgagg gacgtgcagc agaactgatg tgcaggatat ctgtgatggg aaacagaccg
779941 ggagaatttc aagcatcact gtacaattga caacattatg gcttaacggt gccccgaatc
780001 tgacccgtgc aatgtggtct gcaggccaaa gtgatcccaa ggccaaacgt ctcagcgaag
780061 tggaggaagt ttatacctgt accctgcatt ttacagtgag tgttgcaaat gcagacatac
780121 ccttagaatc tgtaagccca caaatctgac ctttctattt aaaaatgtct tcttttagtg
780181 ttttcagaaa cggagacctc ctgtgcccgc cattccgatt catcattcct cggagtatgc
780241 gtcaagacct ggagcaaatc ttgggtctga tcacggacaa ggtcagctta cgaaccggag
780301 ccgtgcgcag gtcggtgaca ccggaggaga gacacagaga gctgagaatg aagcatcatc
780361 cagaactgcc tgtgttcctc caggctgtgc acgctggaag gagcgaccgt gtcctcggcc
780421 gctgagctgg aaaccggcca ctactacgtt gctgtgggaa ccgagaggct gaagaaactt
780481 ccatatgtgg agctgctgct ctccaaagac actgagaggt attgttatat caggacaata
780541 acaggaccac aatttacact ttgaggtggt tttaaatttc caacttttgc agatatcacc
780601 cagataagag aaggctttta aggagaaatg aggtaaaatt ataactcatt acaaaaataa
780661 aaaaaatcag gaagatgccg atgtggctct aaaagatatc aatatcatgt gttagaacag
780721 gaaagcggaa agcagaccag aagaccagta cagcgattca gctctcctcg actccccaga
780781 ggtacagatc tgagagaaac atcctccact ttgtaaatca cagcacacaa gatatgctaa
780841 atgttggtgt gtgttaccca tgggcgcaac cttgtttgac ttttgattct agtccgacgg
780901 tcggaggata aagtcgacgg gcgacaacac tgcacgtcct gcagtggcac cgcagaggag

780961 caggagacag aaggtcgatg agtcctctgt gttcttcgcc aagccggcca agatcggtca
781021 cagcagaagg ccaagaccgc cgatgggcaa cggttccagt gaggctcagt cagttatatg
781081 gcatcacact caaattataa tgctgataat cctgatctga tcatcatagt ccagcccaac
781141 atgtttaaga cagcagcaca gaagagggga gaggaggcac caggcgcaga ggaggtgcac
781201 gaggatgaaa acacagctgt agagattccc gtcgaccagg ttaaacctcc ctgtaacaag
781261 ctgtgtaaat tctatgttac acaatctaaa cccccctctg ctctctaact cagagagttg
781321 cagaaatagt agaagatgag gaaataaaca cccgaggtgc tcctacagac aactcccagc
781381 aggtaggatg aggaaactgc agttcagcag catcaaattg aagtcgttca catttcgttt
781441 tttctctacg tctccagagg cacaaacaga ttttaacacc tgagcagcac aacgacggca
781501 gatgccaaca cacacaaaat ggagagcagg tatgacaggt ccatatcaca tgtaatacag
781561 gtcctgatga agatcgtctt ttatttatgc cctgcagatg gagcttccct cagagcaggg
781621 ccaaccaaag caaacagaaa atgcgcccgc atctcagcag acctctgctg aaactttggt
781681 acgctccgca cacctgaaga gacagatttc agtcaaaaat cagggcagta atcatgtctg
781741 ttattccaaa aggagtctga gctgaaccac acatcttcat cgtcgctgcc ctcgtcatca
781801 accatcaggg atgatgagga aaaggcaagc gtgtccaggc tacatccaaa taaaatgaag
781861 attatttatt cagtcttaat caaaatattg gttgcttgtt tctacccttt tctgttcttt
781921 atttgactag gaagcccaga taatgacggg gcaacagaag caccatcaga ttacatcacc
781981 gtaaccatcc cataataagg ccacgggaga tgaaaagaat ctttgtagta ctatagatgc
782041 agtaatgagc caagcaacat tcatttgtgt taaaaaaaat gtaacagttg tcaataagtt
782101 ctataattcc tctaaatagg agatttggaa gaaactgtaa gacattaatg taaatctgct
782161 gtatgctata ttattatagt actataatct atctatgcat atacgtaatc acataaccac
782221 aggtttccca aaagtagcgt gtttgtaaag acgttgttca gttccaatag acatactgtg
782281 attatgttca gtggtactct gttccatgtg taacgtttat attaaaaatg gctaacttga
782341 tgacttcact gcgtcctgat ttgctgacaa aatcgcagca tcaatttaga aaccaaaaaa
782401 ggagttctaa aaattttgac ccatcaaact atccactgtc cacaccagtt gaacccactg
782461 attaaaccca cagcagaatc atcatttagc ctattcagcg gcatgcaaag cgccagcatt
782521 atgaatccaa gatgtcacag tttaattcgt cggtccacag cagcatctcc cagtcagaag

782581 tcacctgttg gctcagtcct ggccacccag acaagcctca ttttcttatt
ttgcctcctt
782641 tgcgatggtt tcctccacct gtcaaaccta cttcccagtc ttctgttgac
agctgaagat
782701 gtgagcacct cttgagtaca gttgtctgtc agaagctttt cttccaactc
tgttgtggcc
782761 ttgagtcagc cagatcatcc tgacagtttc ccccagtttc ctagtgtctt
ttaatggcaa
782821 agaaaactgt aatcactaac accttgcctt tcttgaccat ttctttgtag
gacagacctt
782881 cttttttgt gtttttacga ggtatgacag gtccatatca catgtaatgt
ttttacgagg
782941 tctctctcaa atagtttctc accatttcta taggaacacc ctactttctg
tagtataatc
783001 ctttttgatt aatgatacca cagtgcgctc caaccctact tttattcact
tttagatatt
783061 tcaatacaaa agtgggaaaa ttggactgtt ctcaaacgta aatttaagtt
taacacgtat
783121 ttcctccatc gtgttcatct taaaacatgc cacagtagac aaaaaccagt
ctgaattaat
783181 ccccgacacc agtcacatgg tgggacagca gagggcttta aattcaagct
taacagcagc
783241 taactttcat agcagcgcca taaatccagt ttagccttga ggggaactct
gggaaaacac
783301 tttaatttta gagctgaaaa agtttccaga caggtcacca aggtctctgc
tccgagaggt
783361 caaccgctgc ttctgaaatg ccctctataa tcctcctctg ttctttattt
cctccatgga
783421 atccaaaaag gtgttggaaa attcacttta atcccaaatt ccattagtgt
atgatctgcg
783481 tcacgttctg ctccctggga tcctgctcat cgatggagct gatgacgcac
agggtgatgc
783541 cgaccatggt gaggaccatt ccaacgacag cctctgcagc agtggggggg
gggggggggg
783601 gacatcagct aaaaccatac aatttttctc aaggttggtc accacccttg
caggcaaagt
783661 ttagtacagg ggcaaagtaa agtactagta attaaacaat ggttattact
tactcttgcc
783721 tccaaactct tcacccagta acttcccagt gagcagcgtg cacaggaagc
tgagagcatt
783781 cgccacagga acagcaaagg acagctctga atgaagacaa gaagatcgtg
cagatccaaa
783841 cacaaacact gttggatcag ttcaatgttc aggtagcccg ttgagtagat
atttcatggg
783901 aattcattgc aaaacaaata tccttaaata aattcactgt gtgtggaatc
ttggcatgat
783961 aaactgcata aatgtgagca ttacattcta taaacagagg catgacaatt
ttgaaattgg
784021 actacattaa tattgcatga ttggtaaaag ggcataaaca gagaagacgc
tatttatgtc
784081 gctcacctgt tgtggaaagc gtataatagt agaccaaaga accagactgg
tttagaagaa
784141 atggaatcag atactggggc aacaacacaa gaggggtgcg gtgattaatt
atgagatgta

784201 atgattgttg gaaataatct acgagataac ataaagagga cagccagatt tcgagcttta
784261 ccttaaagtt taggaaaaga aacttgactt cggcgagtag ctgtgagact ttattggtct
784321 tggtcacctt ctctatcccc tgggcgcccc tcttcaggag agggttggtg cagccccaca
784381 acacagacac cagcagcaga ctcaggagct ccgctgcaac acaagacatg gtcccccccc
784441 caattttata tccacgatac atcgctaaca cttttctgcc tcctttcttt ttaccactga
784501 gctcaccact atgtaaaaaa gtcaactttt agcagcggta gggacattcg gctagcttac
784561 ttccttagca ttcgctgaaa gatactatcc ctgtgcgtaa tactctcttt tgacactaga
784621 aaggaattat agagttatgg ctatatattc ttctttgcaa tgaaaattct atttcaaaca
784681 taagtacaat tattggagtt ttacttgata aattggcacg ttagctactc acctacggtc
784741 accatcgtat gacgttatgg cgtcccgttc aagaacgtaa atcaggttgt tgttgttttc
784801 ttccctttcc ccaaagaaac accaaaaggt gaattgacaa attactggac agcaaaatat
784861 tttattatga cacgtgaacg cagcttaaaa taatcacaaa agaattatat caatgacgat
784921 ggcctaaatc gtatacaaac acattttgca agattttttc ttttgaatgc agcgctggac
784981 acttaaaaaa tgttgaacca aaatccacca gctgatggcg ctagtacttg ttcaacataa
785041 cacggttaaa cacgtttttt gtggatcctc ggtagatgtg tgcataactt aatcacatat
785101 tattatcatc atcattagca atgctcaaat ttattgccat ttcatttaaa caatatacaa
785161 acacgaagaa caatacaaat aaagtgctat aaccgttggt ttcaaagcaa aacacattca
785221 cattagtctt ttaggcaaac gatcaggtgt ctttgcaagg gtaaaaacaa atagggaaag
785281 gctttttttt caaataaaaa attaactatc aacttattga ggggaaacat ttctaccccc
785341 atcaaacgtt cttttacata acattggtta agctataggt atagcaatat ttaggaagaa
785401 tgctgaccag catctacagt cttcgcggtc caatttcttt attttattct gattaatgag
785461 aaactacact aaagcagaaa gggacacata gcatgtttac tctgaacact gactttagtt
785521 taaaaaaaag tgttttcgaa gggttttctt tttccatcaa ggaagatcct tcttgagatc
785581 catttgtcta gtaccatctt gttcacattg tgtaaatttt ccatgccaaa atggcagaag
785641 ccatattagc tcaggtggca aacatcattg atggaacccg tacaaggatt cagtcaccac
785701 acagttggtg gtaatggagg aaaaacagca cagtcgtagt ctttgaacat agtctttggc
785761 tgctatgtgt tttccattga gggaatgtca tgtatggtga ctgcggtgga cccactggtg

785821 gtgctgaagt gctctgggcg gttctcctcc acactgttca gctcgacccg
aggtagcctg
785881 aacctgctct ccaaggctgg gtggcttgaa ttggcaatgc tgcctgatac
agaggtgatt
785941 cccatggtat taagtggcat ggtactctgg gtcctcttct gacgagattt
ggaaccacta
786001 ctgggtccta ggactctgga cacatgagta cgcctggaag aattcaccgc
atgatgttag
786061 ttatccttcc accacagaaa cagaaattat agagttaatg tagctgatta
acgaacgtcc
786121 cagtgtcgag tgtttgtctc acctagtggt gagaatcaat attgcaagat
tgcagttttt
786181 tttctacaat ttcatctaga aatatttcct atcctgtaat tttacctgat
ttctttaatt
786241 ttgtaaatgt gataaatacc acatgatagt gaaatctcta aagaaggagc
aacattgtag
786301 cacacacaga catcattatc tatttaaata ccctaatctt aagtaagcaa
atcagaatgg
786361 ttaaggtatt agatactata aaaggcattt atgtgtggta tttaatttct
gataacagat
786421 ttgtacatat ttacacagtt tgtaaaaggc acaggcacac atccccctga
gaacatatta
786481 atatcaaata aaaataacag gactgataga taaaggggcc tctaatttac
cattttaaac
786541 atatgaaact agtttatgtc tttgcagatc ctgtcattcc tttattatct
cataagcagt
786601 atatgttgta tcagttctac aatactggtg tgttctttgt ttacacttct
catttttgca
786661 gcattgtgca ggattttatt taagttttga ttgaaagatt aaaagtagcg
tgcccttaaa
786721 caacgtggcc tgactgtgct ctccaaggct gctgacattt acaatactga
aatcgtgcca
786781 tcaaacggga atgcggcgtt acctgttata cgtcttcatg gcgatcagca
aaatggcgag
786841 gatgacaatg ataccgatga caatgagagc gatcattgcc ccagaacctg
aggcagatgt
786901 agaggagaca gtggctgtca ttttaattgc ttgtttttct gttctagctt
caaatttgca
786961 tgtcacgttt cacagattcc catgtcgtac aggttatctc atgtcctgtg
ggaatctgca
787021 gtagcttagc aacaaatttg ctcacaaaaa aacaatctgt ccctgcaact
gctctggata
787081 tcaagctgat tactctgaac agacttgaca tttataagcc ctggtctaaa
tctctcgact
787141 gcattgacgc taaattgaaa catgaagtca gatgagacgt gcgaccacac
tcactctgga
787201 tgagtatttg ttctttagac ttggtgacgg tcgtggtgtt cgagggtact
gtcgagttgc
787261 tcacaggcga ggcggttgag gcttgcatct tcacctaggc acaaacatga
gggggaaaa
787321 ccatcaacgc catcaactga aaccgtaaag aaatggggct gcatcgacaa
gaaaataaca
787381 agttaataat gtgggactga aagctaaaaa acagcagttt tttcctgttc
aacagccgac

787441 ggcggcacag atgtcaggtt ttaatacagg ctggtaaaag aattggttta aagctcagtt
787501 aatgtgtaac cttaatgacc tggcgatcat caaagctttc aaagagcaga tcttattaga
787561 atcttttatg aaacatcagt gcgtctgtgt acataactcg tagcctcata aagctaaata
787621 catacacata aatacatagc ctaaagactt ccgctcccta catacaacct caaaccacac
787681 agagacctct gaccccagca ccagtttcag catgaaacac tgaacatttc cagtctttca
787741 acagaagctt tacatttttc aggcattcca tacacgtgtc aaaactggaa tcccgatagg
787801 catcgcgcag gaatacagaa tgtgtgccgt cagtcacaaa aggtgtcagg ttgcttcaag
787861 tgccctcgga gcagcatcat gtacggtttc acctgcactg tacttgtgtg tcaagaggaa
787921 accctcggtg acactgtctc gtctcgtctt cttaaccgtt ccagggtcac agggagggta
787981 cacaatgggc aaatgcagga catacacctg gatgagtcgc cagttcatca cggtttggta
788041 ccttgatcaa gggtacctgg gcaatgccct gaaagtgttc tggtaccttc ccctactaga
788101 acatggatgg aacagacaaa gcatagaagg tccatgtttt gtctgccctg ggagtcaaac
788161 caggaggctg ccgcttctcg cccagttccc aacagactga gctcccaccg cccactgatt
788221 agtgtttaac aatgagtctc cctgctgcag acacgtcctt cgaacgaacc ccctcggcac
788281 aatctgaggc cagaacacct cattcttcaa ttaactacag aacaaaaccc ttccgtcgca
788341 tgatttgcag cattatttac agccacagag gcgtcacccg tcgtctcctg ctgcagacgt
788401 cacggcagag gttcctcatc ctcttttctc ataacccaca tagaacaaag agtcgccatg
788461 actcaggagt catgtgcgct cttgttctga atgcatcctc atctccatcg tcactatgtt
788521 gctaaatagc gctaacgatt tgttgcacgc ctctttccct ttttctaact tcatcaactt
788581 ttcttcccat cataaaacaa agaacagagc catcacattc agtgttttca ttaaaggtca
788641 aacatgcccc aagcgatcct cggaccctgc atatctttcg catttgagga caaaacgatg
788701 ccatttgcag aactgacatc gttgttgcgt cgttgggcct cgatagttgt gtgtactgta
788761 catcttatcc tgcttccttc tcaacggcaa acattgcgca gatatatcaa gttagctgtt
788821 tgaacagccg acagcttctg ataagacctc tagacggtcg gcattgcttt ggcaacaaaa
788881 gaatacagtc tgcgcccgac gctggccgaa acctcagcta ttcacacgtc caccattttg
788941 tgctgacttt tcatttgtta gcttgactct aacttagcta cttccttagc aaaacaccaa
789001 aaaaaacttt tgcttctgtg caacatttga acagagtggc acagagagat tatcaggccc

789061 gtttttgccg tcccttttt catgcccaga aacactcctt atttacatgg acactcccaa
789121 aattgtttgg tatctggaga atgtgggcgt caggtataga atctcttttg tgttagtggc
789181 gaggtatttt tgaattgcag cgctgacaat ggcccagaca cctgacgctg ggtggaagat
789241 ggagatgatc tgaacacggt gaaacaccaa aaacagccac tcaattctta ttacaggatc
789301 aatctttcag cgggagcatg agagctacct gtgccgtttg aggctgacag tattggcaca
789361 caaaggttgc atgtccaagt acctgcggct ttacagaact agaagtattt cttcttatta
789421 tttttaactc atgctcttgt cctttcttat ttagatccaa tttcctctcc caaattccct
789481 ttaatattat tgatattaga agtggcgtaa aaaaagaaac gattctttga agaacaaagc
789541 gtctcttagt tctccacaga acaaagctct taaacctgtg atgatgtaat cttctgtata
789601 aatggatcac cacacacaat acaaggaagg ctgatggtat atatatttag ctcctccctg
789661 tcaaaaagaa caatattgat gtccagagtg acatcgttgc tgctctttca ccatttatgg
789721 gcatcattca atatagttca gtccgaacaa gcttcccgta tttatgtact gtatttctat
789781 atattggcca gtcgactcct tgatgtaata atactttgtt tgttgttcca caaattgctg
789841 ctatttgctc atgcagtcaa tttaatatcc tttcttccgc ctttgtcaaa caaaccgttt
789901 tgtcctgtgc tggtaaaatt tgatcctatt tttttgttgt aaacccacaa agcttcaaga
789961 ctcggtcatt tcccctctgc acagcttcac ccacacgccg aagcccctgg tcacgcattt
790021 acaaacacat tcaaagtggg accttcgtca gcccagagtc aatatttgag ctgccaattt
790081 gttgcaggtt aatatggcgc aggtcgccat tagtcctgaa ggtcacgctt atatcaacag
790141 caggaacgtc atcatctgtg tcagttttcc accttggtgt cctggatgct gaggaaagat
790201 ttgaggattt ttaaaaaaag tttttttttac attacaggtt tttgccacct gtatctgatt
790261 tgattgtaaa attagtactg aataacattc aaataggccc gactcttctt tcatttttca
790321 ttcatttgta ttaaatctgc aattctaatt caataatcat aattcataat gtgatgcctt
790381 tgatataata atgccatctc aaatgttgat acctttactg tgaaatcaca gagcctgtat
790441 tcttttgatg cgatatctga ctgaatcttc caacacaaat aacttggaga aaaaagtagc
790501 cactcatgtt gggtggactg aacacacctt gcaataaaca cataaacatc cctgttgata
790561 tcagaaagag cactgttaca gttggatggc aacacatctc acactgaata aagaatgaaa
790621 agaattaaga gggaatttat agaccgtgga gtccaaaaga gcaaactcaa tgtcctccta

790681 aagtgtccaa tcagtgtcca gtttatctaa gttttcatcc caataatggg
tgggagggtt
790741 tataaaaagt tacaacatgg tttctgtcat ctcattaatg ttttaattat
taaaacaaag
790801 caacattttg tgtaactagt tacagaaact gttgagaaaa tgagtgtccc
tcatcagaaa
790861 actagcgcag gtgaatcatt gtgaatcagc ggctgcgggc tccttcgctc
atttcccagc
790921 cgctgacagg tagccagcac tggaaaacct tggttgtcat agcacagaca
taaaaacatg
790981 tcatcacact agttccttgc tgtggaatta agaaaactct ctggaatgcc
agctattccc
791041 tctcgcctca gactcacctg ccgtcccacc agagcgtctg agagaacccc
gtccctctca
791101 aaatcacaca ccaaagtaaa cttacccaac gcgcctctct ttgatttcag
ctcaagcttt
791161 ctccagacgg gagcctgagc cgcccacgag ccccctccct cagcaggagg
ggggcatagt
791221 ccacaggtgg actgacctcc aagagcgtta gaagtcccag gtgctgctgc
cgtagccgtc
791281 ccacaggtac aaacagatgg gacagaggag gaagatagtc tgaaaacaag
cagaagtgac
791341 tttgtgtgag tgtgtgtgtg tgtgtgtgtg tgtgtgtgtg tgtgtgtgtg
tgtgtgtgtg
791401 tgtgtgtgtg tgtgtgaatc acaggtaaac tgcaggagga tggcacagag
gcggagactc
791461 tgctcaattg tccctcccta agtctgttgt cattgaaagt ccaaagcagc
tggtttttca
791521 ggtccatctt tcgggccctc taggcgtgcc atggcactga tcaaaaagag
cgcggacgcc
791581 tctgattctg aagaggatgg agctctgtgc gttttgtcaa aagaaatgaa
aacaacacaa
791641 agtttcgtgt gtttaaggat tgaatgtctg catagaacgt gctgatcaca
aacttgtcaa
791701 cctttgcaat gagatgtgtg tggttgaaaa taaacaggaa attacaaaac
ttgctcaaag
791761 gacatcatct aaactgtgag aagaaccttt tttgggtgtt attacaggtg
attagcacac
791821 aggtttatgg tgagaatagc agtgaagttg ctagtgaagg gttagcatca
ggtttattgt
791881 tttttttcac acgcgtgtac agtgtcaggg cctgatctca ccacgtgagc
gctcgtcttc
791941 tgtttcctct tctgaagagt ctttgtagct tcttaagttc ctgtgtgctc
tggatcaatt
792001 gtggcagaga tggacattgt aaatatatta tgaaaaattc aaaggttttt
ttttttaact
792061 tgtgagtaca gtgaggaaag cctagtttac agtgcagatg aacaaatgag
agcagatcta
792121 aactaaagaa gataaaaaag aaagcaaaat aaataagtga tcacagaaaa
atgatttaaa
792181 aaaataaata aaacagcaat aaaaaaaatg caataaaaca tggaaatgaa
aaaaagagga
792241 aacaagtaca aatggaagcc aggaaatcga tggttattta tttgaattga
ctgggagatg

792301 caaatgagtt tagtttaaga attaactgaa gatcagtatg gaattcgtgg
cttttattg
792361 taacaagctg cgtgaacggg cgctataagt attcatggac tgaacaagta
tatgaagaga
792421 cgtaaatggg aagccactga gggccaaata aatgaataaa agcaaataga
agttgtgtct
792481 gtgagctgag gaggaccacc ccaccaggct gcacaatgat gtgccaccat
aaatctaagg
792541 gtggaatggt aaatggtatc tcgaggtttg ggattgaaac cagaggcatg
tgcataaaat
792601 acatcaccat aatctaaaac agacataaaa acagcttcaa ctaatctctt
tctacaagct
792661 aaaggaatat ttaatttgtt gtggtaaaga aaaccaatct tttgaccgag
cctttttag
792721 tatctgatca atgttagagt tgaatgtaag tgtgtcatca atatttgtac
acagaactct
792781 tttctatttg tgatccattt atagtggaga agataagagc tataatattg
ctagcagttg
792841 aaaacaacat ccatttatca gttttttgtt tgttcaaaaa tgaaatgaat
aggcatgcta
792901 attaaaacat aaggttgata ataagctagt ggatgctaaa tgaacattaa
attgaaacca
792961 gaacagaggc cattgtcaag atcgcattgc tacctaactc atttttctaa
tattaacctg
793021 catgtctcct tctttaacat attaaaagat ttaaattgac ctctttccaa
tttatttcat
793081 ttggggctga acttttctcc atagtgactt taaaaacttc taatacctca
cttaaagttc
793141 ttgcttacga tggagccatt tatgcaaaac tatttggaga cactgggtcc
attaacagca
793201 gccagggcca cgttaacctg atccttactt cttcgctgca caggcatctt
tgcattcaac
793261 agtcaaggtc attgtggagg aattcctcac tcctgaacat gaagagaggg
tttttgtggc
793321 ctggagagga tgactggcca acacaaagtg ggctttatct ctcagctgct
ggatgattta
793381 gatgctgacc cccttttatg gtggtaaaat agttaatgag attacattaa
cattatagtc
793441 tggtttggtt gcagaatttc ccttgatcgc acacggataa cctttgcaga
catgttagtg
793501 ccctccaact ccacatttgg taaaggtgct gacagtcggg aacctttttt
ctcgcttttc
793561 ctcctgtttc tgagggaact gcacgagagt gagctgccgc ttccttcagg
agcagccacc
793621 cagcgcagct tcacggctga gcagcagcag ctgcagaact caatgagctg
ttgtgctgaa
793681 ggccggcgcc gcacaatagc ttcacctttt ctgtgacaaa ttcacataca
gtcgcagcaa
793741 aggaatgatt aactcggccc tgatgttgct gttgtgtcgg atagataaac
gcctgtcatt
793801 aggtcatgtg gaaatagaag aacaaaggtc aaccgacgat tctatgttga
acgcaagcgt
793861 gtcattctct attttcacag tcaaatgtca gatatgtgct aaatattctt
tgaattaact

793921 taactggccc acagcaacag tgttgacatc tgtttagttg ctttggattg ttttgtagtt
793981 atgtttatga acatatttga aatatttact ttaaccattt cctactcaca tcaatacctg
794041 agtgaaccct gaagacattc tagccatccg agtgtgactc cctagttaca cactgacccc
794101 tgctgacctc tgaccccact gccctgctca ggttttgttc tgaatcttca gtataactaa
794161 tgtatttaca tgcacgatgt atgtgtctgt tcctctccct ttttccatcc tcccccccctg
794221 ccccatctcc tcttctcctc ttttcctcag gcaactatca gcaagagggc cccccccca
794281 tatgagtccg gtccagctca aggtttcttc ctgttgaaag ggagcttttc ctgccaacac
794341 cttgaattta cagcgtaaaa tccttcagtt ttgcaggaaa attgtgataa tttgtgagca
794401 accttgttaa catatgccta taaatgaaat agcaattgtg tttatagatg aatcggtctc
794461 atcggctgca ttttcagcct ttaaaattag aaacttgcca gataaaccac agggaacaaa
794521 aggtttagga agaatttttt ttatgtatgt gaaacgaatg gctgcatact acacaaatag
794581 aagcagaaag ttgatgagac agagaccatt tctgagaaat cagtacagaa cgtgaaaata
794641 cacataccgg taactggaaa ttattgcttt ttttttcctt ttcttttttg ctatgttgtt
794701 ttctggattc caccaacaag acacaatatt catattcaac agaaatggaa caacagtgac
794761 aaaactgtgt ctttgaccct tttttttgta gacccagaag taaaataaac attcagagtc
794821 catgttgaac acaacacgat gactttttttg tattttcttt cccttgattg agcattttca
794881 ggacgtataa catgtttttt tttaaatata tataattttt ttccctgtta caacattgcc
794941 aagagaagct gtaacaaact gcacactggt ctcagcaaaa caaaacaaaa aacccaaaaa
795001 ataacaggta aaataaagtt ggcatatgta cataaatata cattttcatt tgtattttga
795061 gctccatgac cacagagagt ataaaatctt tacaggtatt cctcctgctg ctcaacacac
795121 cgtctcctca gattcttctg acagctttgc tgcagcgctg atacaaaaaa aaaggaacct
795181 tttctcagaa gatgagatgg acacagaaaa acaaagattg aggccgtctc cctttcttct
795241 gcagatccag aaacgtaaaa agaaagaaaa aaacccacca acagaaagct tcggctgcaa
795301 aaaacgaagt ggagcgtttc actcgctgca atatgctggt gaaaaaaggc taacaaggtg
795361 ttcagctcaa gacaagtaca cgtcaaagaa atgcgcttcc aagtttgcct tgatacaaag
795421 aacaaaaaca acaataaagt gctcacatgc agacagaact gtcttttgct tattgttaca
795481 tcgcaaaaca tgtgtcggat tttgtgctgg cgaatcacaa atgaggttca gactcggcca

795541 aaagccctct gtgtactcta gtacaaccat cgtatgatat gagtggagag aatcaaacaa
795601 aaaaaagggg ggaactattc aagtaagtca tgattttcat tggacgtctg ttccagatct
795661 ggctgaactg cgcaaatgct tgtagaaaat aaaaccaaaa agagcaagag tcgcaataga
795721 gcaaatgaat gctttggttg acatcccagg gaccgtagca aaatgtttta aatttgaaaa
795781 tctaaaaggt ttaacaaacc cacagctgag atatttacag tcaggagcag cgagggagcc
795841 ccaggaaact gctatgagat gtggggagag tgcaaatcaa gcagtaggac tcagaagaac
795901 agaacgctcg aacgagaggt tcgacacgac agaaatcgac gtccccaaac ccttaaaaac
795961 ccagacgacc tgcgctgaat tggacttgct gtcgaattca acgctcagaa acggatccgt
796021 tctctgtccg catgaaagag tttcaacaaa atggaaaaaa aacactttag tttcttatcc
796081 acagaaataa ctaagcatgc aaaaaggtgt tgggatatca aatacacaga aggaatacac
796141 ggctacaatt tagcaaaaga gtaaaaaaaa gaaaaacgac cccgaaaaat gcaagaacaa
796201 aaagcccacc accacaactt tgttagtcac aaatggcatt tcgtgctgca cgtgatgcta
796261 agtagcttcg ttttcttgat atactttgtt tgcattaatg tttcttttat tgtagtacag
796321 tatatacatg cagacaagag tgagcacacg ctcgatccgc agatatgaaa acagatggac
796381 caaagagacg gatctgagta aaataccagg ggggtgagaa atgtctttgt cgagaggtac
796441 cagcgaatca actgggagac tggagcggga ccgatgagag ctgacagtgt gttcggtggc
796501 gggcgcctgc gttgggacag ttatctccac ccgtctgtgt cagcagggtg gtgagatgga
796561 ggcccagctt gcccgccgct ccccgccgcg ccccgcaaaa tgtgatgggt gcgtttgtgg
796621 atcgcatcca gccgcctcat tccaacgcca tgctcgccac ccacgccgtc tctcagcaaa
796681 tgcttgtcgt tttttgtttt ttttttgctt atagttgaac tgaaatgatg ggcgtagatg
796741 ctctccgtga gcgcagagac tggagagaga gagagagaga gagacataga gagagagaga
796801 gagggcgggt gtgtcacagg ttcaaaaggg cggtcgagaa ggccgcgcca ctgtcgtgga
796861 ggtgagtggg ggggtcttca gttgaagacc ttcgggggtc catcgggacg tctggtctgg
796921 acgatctgct gctttggccg caccgcctcg tcctcttccg tctcgctctc gcacacgtgg
796981 acgaccacgc tgggcgtgga ctctgtcccc gcgtggagtt catatttctc acctgaaagg
797041 tggaggatgg gtggaaagag gtcacagagg aggccatacc aaattttaaa acagatcaga
797101 cagacaattt ctaacttttc cgctaacttc tttggacaga agagggtatt tgaggacgtc

797161 atcttggtcc caaatcaacc cagccatttc cggacacttt ataaagccgt caacgaaacc
797221 attaatcaag ataatcatca gcagtttta aaaaatgtat ataatgataa gaggaaatta
797281 aggttagttg cagtcatact acgtttttag ctgcaggtgt tggtgctgca aaacaaaatg
797341 aaagatattc cttatttcca gcgtcaaatc gcaattaaca cacattttta ttaaagcatc
797401 actaaggtgt gatctgtgac actgtgacac ttcagtaagg tatattaaaa cctttaaata
797461 ttcagcagca gacgtgtcgc cgctgtggat ggtccactag cgagttttaa gatataatgc
797521 tgaatagttt gagccgaact caacaaaaaa tgaaatgctc cggttaaatg cgcctgctaa
797581 tgcaaagtct ggcctgtttc cagatagatg gaggatattt ctgccacaca aatttctctc
797641 ctgtgtgtcc ctccgttgtg ctgaccaaca gcgcccgtct gtaaagatcc tgacttaaaa
797701 cagattggac agactttccc ttcatttgtc caaatgccaa aagcagctga taagacgcgc
797761 tagatgttat cgtggttgcc acagagatga gaatcagacg gaacaaggac ggggaggaaa
797821 ttggtcagct tgcgtttcaa cgtggtgcca aaatagatag acaagcaatg aatggagtcc
797881 tgctgttgct gcggcgcgct gacagaggga gtcagcacgc cgcgctgcag cgctgcacca
797941 ctctgacctc agacgccagg cggagaacaa agccgcttcc tgatgcatcg tgttgctagg
798001 gaacagaagc tgaacaacaa gagcgtgcca ctctgtgtgt gtctgtgcgt gagtgtgtgg
798061 gtgtacgtga gccaaaccat gtttctgttg atattaggaa aagattattg atgtttgagt
798121 ggagaggcgc tgttttccca cctcatcaac aaccactgca gacacgacac gcatttcagc
798181 gtgtgtgcgt gcccgtgtgt ctgtgtgcgt gtgtgtgcct gagtgtgtgt gcgaggtggc
798241 attttagaaa ggttgctgta atctaaaatc ttgccaaagt cgatggcggt aatctcgatg
798301 cagagaagct gaaagtactc ttcactttaa atttaaatcc aactgactaa tttaagtatg
798361 atgaatgaat gaatcgaacg acttgtctca tttaaagtat tttggttctt ccaacagatt
798421 gtacaaccat ttatgtttaa agatggcctt catgaaaagg ctctaaaagc acctctatcg
798481 ccggtgataa gatatggatg ataaaccttt gctcattatt ggatgggcag acaatgaata
798541 cagatgtgac agaattatta aatataagtt ggttaacaat tgcagttatt taatggtgcc
798601 tcccaatgaa tatgcaatgc aagcatgctg cgtactgacc tctgtaccga ctttgcaggg
798661 ctcacataga aacagtaaaa tgtctatccg aaataaaaat gcatccatga actttgggaa
798721 aattcaagaa taaaagtatt gtgagttctt cagataaatt gcctgcctgc tgaggagcag

798781 aatcatttcc tgtgctgcgt gccactcaag gatgactcac tcaagatctt gtttttttta
798841 cgttttccaa tgttttcctt ctctgggcat ctgcatagac tttgatggtc tgtgtctgag
798901 agcaggttaa actgtatgta acgtggcaaa tacgtctttt ccgagtactg accaggaccg
798961 agcttggcga cggcgcacag caggtcgtag ttgatgactg gcgtggcatc ctcgctttga
799021 ctccagccca caggcggcga ggcaggtggc gagatcagaa actgcttgac gggctgcgga
799081 ggggccaggt acgacttgtc aatgtcctcg tctccattct ggatctgtgg agagaccgtg
799141 aggctgtaac gcgacatgaa gacttactgc ctttacactc cttttcacaa tgagattcgg
799201 gggttttgat agctgctaat tgtcataatt actaaatcaa catgtgccat ctgtgtgagg
799261 gtgaaatccg taatcctctt acatcaacgt gctgtgatgt gcagcaggag tcatgatggg
799321 tgatatttgg gataaattaa gcctccgtca gagttgactc attacagcat gtgggaatgt
799381 gaccttttgg atcctgagtc tgaagctgca gcagccagtg acggacgttc tacttactta
799441 ggtgtgctca aatctcacct gagcaaagta cagtttgagc ttcctgccgt tgaactctga
799501 ctcgtgcagc tcgatgcggg cgcgggcggc ggcctccggg gtgctgaagt ttatccggac
799561 tctacggaag cttttgaaaa gctggaaggt ggtgtgctcg tcgtagatcc gaaacagggc
799621 ctcgaacctc tcctgtacag gttaaaaaca ggaaataaga tgagaaacat ttgacaaaca
799681 tttgaacagc ctgcatcttt ttcttatcac caacaaacag cacattttga atgcgttatt
799741 tagcaaattc ccacacaaca ttatctattt taagatttat tgatgacacg tggagacatt
799801 acaaataaca ggcttcattt cttccactct cgtttcaaaa acactgtccc tttatcatat
799861 acccctatcc cccctgtctc cacaggcatc aaattggccc gtctcagctg tgagcacaat
799921 caaacagctc agcaggagca ttgacggaac aatgaagatg ctttcggaaa aatcggtggt
799981 tgtttgtctc ccggccgacc acctcacagc acgagacaaa acgcgactct gggaagatct
800041 tggcagcagc ggagacggag cggatcagcc atctcgagga ggcgactgtc agaaaccgtg
800101 ggagatcaag ttttacagcc ccgtgagact tattttatcc accccgtggc aacaattcca
800161 gcctgtctaa gctatttatt gtcctctatc agtgggaaac gtgcggcagg gaggctcccg
800221 caacccctcc catggttggt tgacacagca agtctcgacg gggtggaaag agcggctatg
800281 acacctgtct ctgctctcat aatggtgaaa cctgcagggt cagcactgtc agaggagcga
800341 aaacaccaat tcaaagatgg gggcacacct gttatacaat attttggctg gacggaaaca

800401 aactaggttt tttttttaa atgtttataa cagtgtttta tacagaaata
aagtgaaaac
800461 acagaggtga atggtgggtg ctctgggggg ttcatatctt cactccacct
aatgcaaagg
800521 gtggttaatg accgagctag tggtgaccca cagtcgctgc agcgcggtgc
tggccatggc
800581 caattcacac tgtaagaaca agtaggtctc tgcccagttc agcctctccc
acacatcctt
800641 tcacgtgcac tctgcccctt ggagcagctg cctggactcc accctcagac
tttcaggcat
800701 tccattttta gttgggcacc ttgaaatgtg cactgaaacg aggcagcagg
ggaaagaggg
800761 caggtcgacc cactgcagat ttaactagaa tgccaaaaaa tgctgtgaat
gatgtgggat
800821 agcagaggag gctcatgttg ggatgctttg ctcctacata gatggcgtaa
atgttctgtt
800881 ttactgtttt aagtgaccac tacaggggtg ctgttgatgc aaacgagaag
ctgatggatt
800941 ctgacagaac tcagcgtttc tggaaaactc tgagatttgc cgctcaaatc
gtgatgtgat
801001 gggcctccgt tgggaccagc gataaatatg tcacgtgacc ttttaatggc
attaaaacca
801061 gacacaatgt tctggcattt ttattcaaaa atttcaacat gttgttttcc
aacttgaact
801121 gctgggaaac tttgtggcat tttttttag aagactttat agtttatctg
gcctccatga
801181 tgctggggtt agcacccaca acctcaaagc ctttttcac aagttgccat
taagagttct
801241 tcatttcata acagacactt tgtttatgca aaatgaaaaa tgaatcagta
gatatttatt
801301 tttcattttg aatccctcat gaccttatgg atgacaggcc tacaagttca
gctatgtgaa
801361 attaataatc tctctctcat acacacacac ccttatattt ctaaccttct
gaggactttc
801421 atagacatta taaccccca gctctgtacc ataaccatcg caactaaccc
cctaacccta
801481 accctaaccc taaccctaac cctaacccta accctaaccc taaccctaac
ctaaacccaa
801541 ctgaaagctc accctaaaaa ccaagcccaa ccattaaaca gtcctttgaa
gttttgagga
801601 ccagcaacaa tttcctcact tcccaaaaat gtcctcactt tgctggtaga
atgcacgttt
801661 cagtactcaa tatgtagcaa gtacaacaca cacacacaca cacacacaca
caccattcag
801721 caatatagac acgaggagca gtgtaataaa ggttcccaga tcaaatccca
tattaacacc
801781 ttatgctttt ttgctgatag gttcacatct cgttggcatg taacagctct
ccaggagcga
801841 gcgttggggg ggaaggaggt ggagtcgagg ggtgtttggt tacgcagagc
tgccattgca
801901 gttttctctt cagcgagcaa ttatttttgt tcccttcatg tgtgcaagag
ttacataaat
801961 gaatgtgtgt catgctgtat gtgcataata gattcagagt tcttcatata
tatattagtg

802021 tcacaatact gtcacagatt tgaggctacg tgctaattaa aaggcacaaa
tactgcagct
802081 gacaaaataa gctaccttta ttaagctcag atgttacaat aaataaggta
atgagtgttt
802141 gactttatct tctatcagtc ctcagagata aaacacacta gtggaatgtg
actttaatgg
802201 tcggctgcat tttttttttt ttttttaatt tctaaaggag gtttattagc
ctggttggct
802261 gcagaggtgg ttatgtaaat tctgctctat ttcactgacc ccacaaacat
caaccaatga
802321 ttaagtaaaa gtaaaacaca ggattgttga ctgttggaat aatctgttaa
actcacatcg
802381 ggtgcatgct gggtcatatc gatcagtcaa ttatttattc gttttaggtg
aactggagaa
802441 ggtgtagagc cccctattgt tttacaagag acattacaaa taagggacat
gaaaatgtct
802501 acacatcaag gcgatgttgt ttcacaaaca tgtacagcat ttctttcccc
tcaatcttac
802561 ttgtatattt ttcacagaaa acctctaaca gaggtttaca ttaggctaca
atatgaactc
802621 tatatggcag ctgctcgctg taaaagctct cagtaatggt ctgggaactg
gtactgctgc
802681 cagccacgag gccaaggtcc ataatttaca aacattttaa cctttgagag
atgaaatgtg
802741 gacacaaatc ctctccaaag tgtagctgga agtcagaagg aacgcttagg
tactttcttt
802801 cagtccggcg ttgtcagttt taactaacat agagtaagag tggaggtttc
tgttgatgcc
802861 aaggagcgag aatgtacagc agccgtcccg acggcctggg accgcacaca
cacacacgtc
802921 tgtgtaatgg tatgagtcag gtaatctccc agtccctatt aatgttgaac
agaatgtgag
802981 cacaactgaa ggacagtggt tcatctgtcc agggacacac acacacacaa
acacacacac
803041 cctgcacctg ctctgactgt atctgcagcg agccaggaca agcgagaaag
aacaccgtga
803101 tcttcctttc caacagtcca aataacttct gacgacgggc gaaaatggga
cacaaaacat
803161 ttgacacgca acagaaaatg tggaaactga atgtgcattt caatcaaaac
atccatcttt
803221 cccaaccgct aacaatcatc tacctctcca cagtttcact cgacccttgt
gtctaaaccc
803281 cgatgacagt gacgtgtttt agggtggaca cgcttgtgaa gaggtgccat
gattgaagca
803341 ccaagtgctg ccattaatgc tggccgcctg ttcgcccctc tcaccgtgtc
acctttacat
803401 aacagccagg aggagcctct gcaggttgag ttcccttcca ctgtctcatg
acgcctgtat
803461 gcctgctccc ccctccaccc aacccaaatt agatttgaca cattatatta
atcaaagcag
803521 tttgttttat ccgtggggaa ataaagacag actcttccac cgggggggaaa
gaaagtcaat
803581 ttcagattat ttcctcattc gtagcgtgta aaagatgttg ccggttccat
ttgttcacac

803641 aaaaattaga tatgaagtaa tagctgaaac tgtgtacatg cttttccatt
tctaagcgtt
803701 tgtgcatcct ttagtcaaaa tatggacaga cgctctttaa atttggatag
aatgggcatt
803761 taactttgaa taatattata tttttgactt ttttagcatt gctgctgcta
ttattcatcc
803821 atcctccttg gtggtgctga cagaaagtag taaaataaat ccacctttcc
ttctggtgac
803881 agatgcgaca aagctgaagg ggaaactata gacataaaga agagcggagc
agtaaaaccc
803941 tgccgtgagc gatgtaaacg ctgctaccag tgcagcagtg actgtaatgt
catctttcag
804001 ctggctggat gaagcagtta tgttcttgac actaataaaa agagcatggc
tgcggcggca
804061 gcgggacatg tctgcggaca tgattggagg cagcgagcga gtggtgagcc
atcctgacaa
804121 gatcatcaac aatcaggaag ataaggttct gaggtgcagg gctgcagctt
gggagaatgc
804181 atgtttattc aggtaatgaa tttgctaagt gctggacctg agtgttactc
cgtgagagat
804241 gaccgccact taagtgcttc tccgtgttat gtaaagacgg aattgtgttt
cagagcaacg
804301 cgcagagatc ggtggcagtg tttcaggaca gaccagaatc tatatctgct
gtatcctcat
804361 ccattacgga agaagaagaa aaaaagaaaa aaaaggaaat atccacaaac
acacactcag
804421 aaaacaccat tagcattata atgatttgtc ctcagaaata ccggattaat
taaaatagat
804481 tggtagacat atcgcagggt acaaagttaa ttgattggaa tattcataaa
tttttgcagg
804541 taaattaagg taacgactcc tgttgatgga tgaataaatg ctgatttgtg
atggaaacac
804601 accacttgtc tcctccacac cgactgaccg actcacaatg ctttattttt
gctgcggctt
804661 tgcatttaag ctagtgttta aatcagattt gcacttggat actcaaatcc
atgagagtaa
804721 tccgcacgct agttcaattc tacactgaat tacaccattt ggatcaaaag
gaaaagatta
804781 acccagccta attgggatgt aaaataaaac ggtcattggg cgaggttttg
ggagtttttt
804841 tctctgaagg tctcgcgatt cgctcagctt tccttacaac acgtgtgcgg
ctttagcacg
804901 ctctaatcta gacccctcgt gcttgaatgg gatttgggtg aaaaaccatc
aaactgggcc
804961 acggagcctc cgggtgagac actgaaagca ttacagtaaa aatagaccgc
gttgctgtaa
805021 ttaagaaaat taaattcttt aagtgtgagg acggtcattt ttaattatcc
gtctccctaa
805081 agggccaaac tggaaccagt ctaatgaggt caaatgagag cgaaggatta
cagtgataac
805141 agacagagct ggatgatgaa tcaaaccccg gtgcacgttt gttccagcac
atttaggctt
805201 atcacattcc acgatttttc cataaaacgg agccaacttt ccgatgcagt
cacagcaatg

805261 gtgggatgcg tttaaggaca acagaggtcc tgtgtgaaga tgcttcactc gcagccgcgg
805321 acgccagaaa ccagaaaaat caaaaggcag aggagagata agccctttt aaaataatgc
805381 ttatcgactg cgttcgtcat catttggtga cccggcaaca gggacggaga ataaatagat
805441 gaccccaggg caaggtatag aaattaaact ttaaattaaa ttaaatcaag ctaattaaac
805501 aatgcactga ggagcacatt gtgggccggt acaatgagaa cactgtcacg ggtcaatcat
805561 cagccacgat ggtgcatttc aagtaccgat gttcaagact tctaagcaaa aaaatcaaga
805621 ttcaaaccgt gtttcttgaa aagcaggtga acgtgctgac cagggagtcc ctcctcgggc
805681 caaatggtca aacaatgttc cattatgacg tttataagat gctgcccaaa cataaaataa
805741 taataataca gtaggtaggg taaggacatc caaaatccag caacaacacc aacaattaca
805801 agttgtggta aaaatggctc ttcctcctct agggttagag ttagtcacaa actaggatgg
805861 catgattaag gataaaataa ttaataatga atcaattgcc agaccggtga actaccggtg
805921 aacctccact ctcactcaac caatagcaat tgtgcatgcg gcgtgcgaaa aacctctatg
805981 ctccgcctct tttccagtcc tcctcagctt attcaagtca tcagcaaatg ctcatcacat
806041 gcgtgaaaat gtcagatatt gtgtttttcc aatgttgccc gtgaaaacgc tgcagccacc
806101 tcactgatct ttctagctgt ggaccagcgg ttcagcacta taatgactgt aagctgatga
806161 agtaattatg ggttgcctca gagaatcctg ctcattacca gtggataaac tctgaaggtt
806221 gtgtgtggtg acccggcact ctccctctgc ctcgcgcaaa gcagccatta gcattcgcaa
806281 atcctcatta cctcaaaact caaaaacaga acatgatcgg gagcttttta acagatcacc
806341 agggccgtat aaccgacggg aagccgtgcg gacgggtaat tgttgggggt gggatcaata
806401 acttgagcgc agagtggtat cgacgggggc cgactgaaag gcgctacatc gcggctccct
806461 ccgggacagt ttgtccgcgg cacatagatt taaatcagtt tacagcatgg ggacacatta
806521 atcctattac cgccaagctg gtctttatgg tttttattga agcaggagct tttgctggtc
806581 aacagcactt aggccacttc tgctcgcaac cacaacgtct cccacgcaga tttagacact
806641 ttagggacga caacatttca gcgagcaagt caatattaca taaggaagct gaaaggatgc
806701 cgcggctctt cctggaggtc atgggcccat atgcacagat gagatcgctc ttcagccggt
806761 gatgcgcaac tcttgcgtca cccgctgagt tgaccgatga acgcgcatta gcgcagcaaa
806821 ggtcgagggt cgcgcgggta agtcacaaaa tccagcgggc gccgcacgca gtttggggcg

806881 ggagccaccc tttttgcacg ccggcatttc acaggtcggc ttctgcggat gatcgcgaaa
806941 tctccttcca aaaattcagt cggagattga aatcgaatgg atggcgtcaa atgaaacgag
807001 gcgtatttga ttaatagtcc tgcttttgta ccatatgaat gtcagctccc agtcggccga
807061 agttctgccg tatcgtttta aaggtcgctg gtgtgccatt actatttcag gcgccgcatt
807121 gttttgttaa gagttctgct tggctgaaaa ttgcgctgga aattctgcgg gtgaaattgt
807181 gttccaacag caaatccgac gacaaaggtc ctttattgcg tgacagtgtt tctgcacctt
807241 tcctcttgcc aagaaaactg cgagtggcat tcttttctcg tgttgtttct aatcttatca
807301 ccacacggcc ctggtccatg tggtaaccag acacccccgc ctgggagacg gctgtccttt
807361 gttgtgcgtc aggcaaatgg acacagtgtt aacctttagc tgacacctga gcccgccgcc
807421 tctctaaggt ttcaggccaa tgtgagctgc ccatctcccc ctgcttagtg cccagattgg
807481 gggggggggg gcaggcacac ctgagcccaa tgctcgggcc tggagccgct caaccttgcc
807541 aggatgtaga tctagtttct gtccagctca gaacctgata ctgtccccgt cctggtcctg
807601 gtcctgcctc tgctcctctg cccctcgcct tgtccccaac agctggtcag agttcatcac
807661 catcatattg tctaaaaatg ctgagtcagg tgaaagttca aacgcctgat taaactttaa
807721 tgagaatgga ctaaataata aaacacagac aatgagtcac gatagcgaac tgtaaacttt
807781 gcctgcttgc ctgaaaatca aaagcgttgg aagctctgag aaccgtttac cagaaggaac
807841 gttctcgtct gtattcacaa cgcaggaacg accattttcg ccacggcatt gcattgcagg
807901 gcgccgtaac tgcgacaatg ggaaccgctt gtggcgtaag gcacgctgcc tgttctaaca
807961 cacaaagaaa aatggcgttt ttaaagatga aatactaaaa cgatatgctg ataaacatct
808021 aaatcgtgaa ggagagccaa ggccaacttt ctggcaatga taaatgtaac ttctgcagag
808081 gaagacactg agaaaaaatg ggaaagttct gttgccgcac agctaataga ttcagcagtg
808141 aggccctgaa aaacccttca gcttcactgt gacctgctct tgttgtcgct acgccttatt
808201 tagcggctga caatcatgac gccactgtgt acggtggcat taaagaataa ggaaacagct
808261 aaatgagtcc tcataaccgg cgccgtttct gagccgctaa gtcctgggag gagacgtgac
808321 accggtgacg gctaaacatc gaggcggaaa cttttccatc ggctcgccga cggaggtgtt
808381 tgcttatctg ctgactcaca gctgcggctg gaacgtgtcg gtgcggcgtc tcggagtgag
808441 agggcgaaat taagttgaaa aatatcattt tgcggcttct ggcgcggtct cagaggctct

808501 ataattaagt atgaatcgca acatcctgtt tatttccaca cgtttcttgg acagaaatag
808561 cacaagagag cctgaatttt tttttaaagt atatatatta aagcaaataa ccaaacagcc
808621 ggtaaagacg ggaggcgtgc gaaattatag gcgaaggcct taaatcaagg tgagcgcaaa
808681 gccttgaatc atgatctgtg aatgtattca ctgcctctgt gaccttcagc gtgtgtcctg
808741 tgctgtcatc tgctgttgcc ggtagttctt ccaggtaatt actgtgacac aacattcccg
808801 ctccgcggcg cctgcccacg ccggaacctt tcaaaccaaa aaatgacggc gcctctcttt
808861 tctggttaga aagcggtttt ctgtgaaagc ttcgaacagc gttcgaacaa cctgcctgct
808921 tggctcatta atgatgtaac cagctgggac gaagccaagg cgtgggccag ccatttaaac
808981 agtgtcaaaa caacagctcc gacagaacgg ctgcggcatc tcctttaatt aaaaacccac
809041 caaacactga ttatctccgc gttgcccatc caagcaccac agccattacc taatctttcc
809101 tcggaagggt ctataaatag cagcaatcca catgttatct cctccccaaa aaatgcttta
809161 gagggaaata aggatgtcgc gattgttgtt tctcatcttc agatttgaga gatattatta
809221 gaaaatgatt aagatcatgt tcaatgatat aagggggggaa aaggaatggg ggctgctggg
809281 gaggtgagct cagtccgagc ctatccccca cgtgccttcc tttaatcttg tgctctcagt
809341 gatctctgtt gccttcggtg ggccacaaac cgggattagt ggtgttctct gtgtcattcc
809401 aaagtctaat gtccttctgc tcttttaatt atgcacacag acacacaagg agatcatatt
809461 ctactttttt aaagggaatg tgtgaaaact ggcctgtctg gtgtctgacg accgtcacgt
809521 gtgacctacg aagcccaatt taacagtaca atattggaaa tcatcaataa gcatcatgtt
809581 tgtggtaaca gagcacataa atgtattgta gttttattta gtcttgcaca tgtaagcaaa
809641 gacctgggtg gaattgcaag aaactgtgtg ttggatcaat attacatcgt tgctggggtg
809701 aaatctctgc atttttgggt gcagcggttt ggtctgcaga gcacaccagc agagagggag
809761 acaggccagg gtggcgtgcg tacaccccaa ctgtgatgaa cacctactct gattataatt
809821 actggatgat tcaactcgtg tcgagctcct gcgtcgctct gttgtgccct ccctctctgt
809881 tctgctgcct cgctttggct ctttggtgat gagtcaaata aaacaagcag caggtgaagg
809941 tgagtcaagc tctgccagga tatctgcgca gctttcaagc ccccaaagca gtgcaaaggg
810001 aaagaggagt ctacgctgtc ctgccccttg gacaaacact tgaaaccttc aggagaacta
810061 aaaaaagact ggtgtttaga ataatcacaa agagtctcat cagagcgtgt catacatgtt

810121 atggtgcttc catctttcct gcacccacag tgctgatggg atgaggagag agctgtaatg
810181 aagaaagtga ctgtccacac tgcgtctccc tcactttctc tgtatgtgct tcgcatggac
810241 aatgccacac atcccaccct tgcttgcgca tcatctcgac accccccaca caaagagccc
810301 ctctcctcct aactttccct gaaaaatgca tacatgccag ctccaacctc tgcctgctgc
810361 acttcgggga tttaaccaag cgtatctcat ctccagatct gcagtttccc tttggcgttg
810421 cgcgcgtcgc gggacgtgcg tcttcgcgat ccggcggctc ccattgataa aaaccctgca
810481 ttcaacgtaa cgcagccatg catgcgcttg tgtgaagcat ggcatgcagc cgcggcatgg
810541 gtcggatcca tgcggcttaa acgtgcactg gcatcgtctg gaggaaagag cgtacctgca
810601 ttttgtctga cgtaaatacc tcggtgtcta cttcacaagc gatgatggtc accatctcca
810661 gtttcatatt tatcgatggc attccccctc gcggtcggaa acgatggggt tgattaaaag
810721 tctctgtcgg gtaaagattc tgtacagaaa ggcgacgtta ggtccagttc agttcggttt
810781 gtatcagagg agagagttgc tgccggactc ttcgctgcgc cgacaagagt gctgccgcag
810841 cctctctccc tctccctctc gccctctctc tctcttgctc tccctccccc tgtggctcca
810901 cattgacgtc atttctatct tcacgttcat gttttacgcc actacagggc cagatgaagc
810961 gcgcactgca gaattcgcct gattccagtt cattccgctt catatgattt atttctggag
811021 tcactcttca acttcaagcc tcagctgcgc caccgcgttt cttgtaggaa gctggtcggg
811081 tctccaggga ttaatgcaat taggagtttt tgccaagaaa tatctattgt gatgaacgga
811141 cgccagctca tttatcaatc atcgggggta aaaggtcatc tggatgaacc tgcggaggct
811201 gtcattcgtg aatcacagct gtgaccatgt aaaacagacc cctgacagat cccatcattg
811261 tattttggtc ctgcagtaat tgcgtaacag cgtcggtcag acatgtgcag gcttatttga
811321 ctccagtggc ctctgaggtg cagacatggt gaaacgacag tcattctgat gcgctcaccg
811381 ccaaagttgg cagatgcgga gatgaatatc taatttaggc aatggattct ttgggcaggg
811441 tgctttgttg atactggaga gagctacccc cccgaaaaag ttctaagcca gctgcagcct
811501 gtaatgtaac ttttaatgct gactagttga ttggagatta agtgtaccgt ggctctttat
811561 cgcaacaaaa ccggagctaa atgcagccgc ggctcggtct ctcttaaaca cgaagctgcg
811621 cttaaggtct ccatttcagc aggagctaaa ttaaaatgaa atttccacag gcttcgtggg
811681 tcattgcgca acattcgaat cacacagtgt cttcctccaa tcaatctttt ctctctcagt

811741 ttcataacga taattgcctg atgggctgag acagctaagt cccccataag
ccattaatat
811801 tgaggctcta ggcggcggaa gctatttctg tctgcgtgtc agtcattgtc
cgtgctgtgc
811861 agatggagat atttttgaca ttcctccatc agatagcatt ggtcaggctg
ttccctgtct
811921 ggaagaatct acatctgact gccaaaaacg ccatgtccag gttgctgaaa
cctgcccgcc
811981 accatcagct gcttcatctg ttaaacagac gggatgcaga ggcaccgcca
gagatggccc
812041 ggctagatct gaggtttttc caaaacaacc ctaaaaatga gggatgcacc
gagtgcatcc
812101 acctttctt actgctgtct gatgtagctg ttgctgggtt aaaaacaaac
aaaccatgat
812161 gatttattta tgctccgctc aaaaattctc tgttcacttg cccctattta
caaagtcttg
812221 gtcagcagga gagtcagcgt cctgttgagg gaaaactcag ctcagcacct
tctgttatca
812281 cacgtttatt gaacccatct gaacctgatc catcactacc tctgaggcga
tgtttactca
812341 ttgttcagga ttgaaaatgg ggtaaaaatg atacaaaatc acccgatgaa
gctgtttttc
812401 atcagcagcg cggaggctgc gcccactctg attcaaatcc cgagtccaga
ctgaagcaca
812461 cattcagcag gttcgtttgc tgatagtgat caacgggttt ttgtttttgt
tcttgctttt
812521 ttccataatg tcccaacttt ttttgttgac tggcttgtca gctcaaccct
gacttggacc
812581 cagtgggctg gatacacacc agaggagcag cagatggatg atttaacaga
tgaatggcaa
812641 ataaattaat aacaaaatat aatgaaaagt ataaagtaaa ttcatttaac
tgcctcagct
812701 gcccgccagt tctaagaatc taaactacta taattattaa acatgatatg
agttgaaaac
812761 tatgaaatat gtcagatctt tcatttgagc cattaaaact agaaagaatt
tgacagtact
812821 tcaggtattc tgctgcagag atacagtctt gatatgatcc agtatttatc
actgtaagat
812881 agaaagagat actgcatttg atgttttcca aaataatctt tttcctactc
cacatttttc
812941 atcaatgatt gctaatatta aaagaaaatc taccgtactt tgaagaggtc
agtaataaga
813001 gctacagctg ttactgtagc taagttactg ctcgttgtaa gaacagggtc
gtctgaaatt
813061 taggatcatc ttggttggtt ggggttaggg agttttgggg tcagactctg
ggattctgcg
813121 aggcgcctag agacaatctc gactgtaaca agcattttat gaataaagtt
caaattcaat
813181 tgaacctttg ctacaaaaac accaaagttc ccacaggaag gacagctgaa
acctataaac
813241 atgctaatat gctacactat ccctgcaaca tccacagaga tattagatca
tttcagatta
813301 agctcctctc atgattgttt tatatgttat acattaacaa gccctttaaa
gaatcgtcac

813361 aatgttttag tctgttgatc tccttacgcc tctctgctgc agccgctgac aaacatcgga
813421 cgtttgtcat tcgcgcgtgt tgccgatgcg ttcctctgat cgcctttgcc tcatctgatc
813481 tgacagagat ggatggctgt gcatgatgaa acacttgtct tctcctctgt cctcattctg
813541 cagccctccg cctccccctg ctgctgctca tccacctcat tctcaattct ccttttctca
813601 tttacagttc cctgcgttag atctcgccta acccaccagc gcaggactct acggagggga
813661 atgtgacgca ggtcttttat tgattcgaag cgtgaaggcg cagccgagaa gacttccatc
813721 gtgaagtttc aaattctgcc ctccatgtgt cgaaagctat ttcactagtt tacatttgag
813781 atagtagcag tataaagcct ggacgacccc catctgtcac atttccaaaa caaactactt
813841 catcgatgac tcactgtgat gaaacaacat ggtgtttcat agacgaagca aaatatagtg
813901 ttgttttatt aaaaacagag atgaggagca atatggcggc atgtctccag attagtgatg
813961 agcggacttt gtattctgaa gcagtgctct gcagagaagc tgaacagagg gaatcagggc
814021 agtggatcat cgtggtcggc tcaaaagcac acgtccgtgt cggttagcgt ccagccggag
814081 caaataccaa cagacacgtg accaaatatg caaatctgct gcctgtgtgc gtttgtacag
814141 tcagtccaca aactaaattc agtctgaaca cacacacaca tgcacacaca catacagata
814201 tttattcact ttacacaaat ttgaccaaaa tagaaatgac gggaataact ccaaagcgcc
814261 tgcactgcag gcatgctgca gctgcattat catcatgatc aggtttgttc cctcactccc
814321 tctctctctc tcccccccca tctttctctc tcatgcacac acacacacac acacacaaac
814381 acacacaatc catcccttca acctcatgct acctcagttg gcccagccaa agccaaccct
814441 ggtgccagcg ccaatgcagc tggtgtaatc taggccaagc tatttataaa agcaagcagc
814501 caagtacagg cacagggagc caggggaaga cggagggtgt gggtggggta gggggggataa
814561 aagaacgtct acaagctgag gagatagacc ctcagagtga gggtttgcaa aaacaaaaaa
814621 agatggctgg attgatggat ggatgtgtcc agggcataga aagtagtaag aggatgagtg
814681 ggacatggag gaggcgtggt ctcggtaggt cacgtggcct gagtgtgcac taaagatcat
814741 cacaaatgtt ttacatgact tctggcaact aagctgtaat taattgcaat agacaattcc
814801 ctgagaccat ctacaacagc taccctttc ctcctaatga acagaaacaa gcctgttgtt
814861 acgtgggcac gctgttgcac aagcggaatg agcaataaaa ccaatcatga aaatcatgcc
814921 attattatga tgcgggggtc tcaatgtagt cgagggagct caggtgaaaa ggtgatcgga

814981 tgcatgaaca gttagctcta cttgctagct gaggctgcag attctctgtt cctgagagaa
815041 ttcatgcagc agaataatcc caaattgtta aaaagttttt tatgcaaact ttatgtatat
815101 atgagaaaaa cattgagcta caatattact acaattacta ctacaatatt atcagtttat
815161 ttaaatagtg tcatgagtgc atcagaaatg tgaaagtagg cctttttta atgaactaca
815221 atgtttcatt acatctagta aatgcacatg cacacatggc ctctcaaaga cattgtttgg
815281 gatggcccac atctggtggg acagcagcag ttgttgggtt ttttaagcac atccagcagc
815341 ttagaggctc ctgtatggag ggatggacgg ggaggcaccg agtgacacgt gaccatcatc
815401 tccctcagcc aaaggcttta aaatcaggtc agatcttggg acgggttata ggcccacact
815461 gcagacacaa caaagggggt taaaggcgtg agatgttctt aatatctcca gtacagttat
815521 ttaaataaaa ggaaactaga gcagcagata atcaccagaa tgatcactac agtagtttgc
815581 ttcactcagc tctagcagtc agcatgttgc ccaggcaacg ttattaatcc cctattattg
815641 tggggaaatt ccagtatgta atcggcgttt tgccatttaa aatgattgtt ttcagtcagc
815701 tgcttccttg ttgatcgtca aacttggtca tattgctgac gtggagagtt taggctgttc
815761 gaatctgcct gatgtgtgtt ctgattccct ggtcgtgtcc ccccaccctc ctccagcaaa
815821 cctaattatg ctcatgtgtc tcacctagac ctaatcatac ccgctcccct tcctgggtgc
815881 taaaacctgg cgaggggagc gggtaaaccc tctttctcac ccatcttgtg aatctcactt
815941 gtgttgacga tgcctttcta gtatggttgc tccgttcaac taaactgaat ttaaatattc
816001 agcattacaa acataacatg tttgtatata tttcattatt aaatattttt gtagtgaact
816061 gtttggacca tgtgagaaag tagattcaaa taataagttg ggttttgtca tctctgagtg
816121 tgtgaatgca gcgacggctc actgcaatga agccggaact gctgcatttg aaagtggcca
816181 catttctatt tttaggctga actggataat tgtgatttaa atagaatagt cagacaacaa
816241 ataacatcct gctgtggatt ctgaagcgtt ttgtaatcag ctgctgtgag gagagagagt
816301 gagacgtctc ttttatcgtt aaacaaccat ttctgagtcc aatcgggctc gcatacagat
816361 gagggaagct gtatctctgt gaagccctaa cagaaaatgc acagcagcac tgtcatggta
816421 gtacggttgt tttcaaatct gaaacattgc aaattaattt taaaatagtg ggtaacagga
816481 gacaaaactg aaaatcctga aatcagcaat aaatccacaa gtcaataaga gagctcagtg
816541 ggatttttat ggacatcaag gatgatatta tttaatattc tgagtggcac atcgaaacat

816601 atggtagaaa atacacacat tctagagaca cacacataca ttggaaagcc aaaatccacc
816661 ggttgattca acagctgctt cacgcacatc accgccactc tggagtccaa cagcacccac
816721 ccaaggctaa ttagctgaaa catactggtg catttaacac ctctatgtgc tccacaaacc
816781 aagcagagct gctgtcaacc tgttcacagt cccatccaca gcaacagcaa actcttcttc
816841 tcctcttcag tgaactttca gtgtggacca gagaacagag agatgaggtg accgcggcac
816901 tgcttggcca cccacagtgc aaccaggcct atattgttgc tcgcagcaga tttctcagat
816961 gaatctgttt ctgcttcaaa cctgcaacct tgttgccaga atatttctgg gagcagctgc
817021 gtgtgaatag agtagcagat ctgacctaat acatcctcat atcagagcca gagctgtttg
817081 gtccacttta gatgcccaga aatttgtttt tctttttttg gatcgtttcg atcaacgtcg
817141 ccccaaatgg acaactgtgg taactgcagg atgtccgagc actttcaaaa ggacagcatg
817201 aaagtgaagg tgtgggattt tttcagcatt gctcagccga tcaagctgag ttcccagact
817261 atcttgttgt aaaagtgccc ttatatggtg tggatgtaaa aaagaaccct cctctgttgt
817321 gcaacagaca gcaaagaggt cacgctttag tccaaaacta tctgcatgat gttttgctcc
817381 aggactcaat gtttcctccg gtcaaatgct gccagacatc atctgcagca atcgtcaaag
817441 tattagacct attttaacat gcaaaggatg acaaagtgat attggaatgt tctgcttccc
817501 agactgaaat caaatgtgga ccactggcag attccccctt gacttcagca tcaaaacaca
817561 tcagaatcac tttgccgttc gttactgaat atctgtacct caacagctcg acgcgacaaa
817621 attgcacatt ttattgctgt cggctgcttt ccactatttc agcttgatat aggaaaaaca
817681 agcacagcag gtcggcgaga cgaccctgct cggcgcacca gaataatatc caggccacag
817741 caggagcacg agcccacggc gctcattcac cgcaacgctg catcatgtct gaccttcagc
817801 accagctcct tcacgttcac cacccagcag tggtgcccaa aaatagacgg tctgacacac
817861 gcgtgcatgc aagtgcgctc tatgtacacg cccggccgct tgcagtcact tctcggctcg
817921 gttttcacat cctctcttac actaccttta gtccccatgc agttaatgct gctctctgct
817981 ctgggcctgt cttacactat ggcccgagac atgtgagtca caccattaga cgtgttagac
818041 agaagagggg cagcccttca gatgttgtcc tccgccgtta aaattgctta ttcatgaaag
818101 cgtcagctat atatctgttt cttttattca ttccggatcc atcaatcttt tcggcttctg
818161 ttagtgatct ttttttaact ccattgcctc ctgctacctc tgctcattac cctggtctca

818221 ttgtctcttc tctctttctc tcatctgttt ccacctcttc tctattctat ctagtcggtg
818281 gcttgtgtgg attgacgact tttcctcaca cggtaggaga ccactgcttg ggagagctat
818341 ggtcgtgaac ccaacagctg gattgtggga agaggaagtg cactattgga gacctacaaa
818401 acaaccccca gaattagtaa ctattagtct ccttgaaagt gacaagaggt ctgaacctcc
818461 tccggtggga tgtttgctca tggctgtgca gtttgactct gcatttcgga taaggcacca
818521 ggccttgagc tcttcgtagt gagcagaagt gcttcggtta tcgttttccc caacctgcag
818581 ctgcagcaac gtatcaccag tgctcctcca acagcaacag ttagatgggt tcaatcattc
818641 ggatgtggag ttgtccatgg aaacggagtt cgctcttagg tcagggtcat tattcatgca
818701 tatgtatgat aagtgcaggc aggtgcagaa ttgtgggtaa agatggtttt attaaaggca
818761 gcactggatt gtcataagaa aggtgcaaga ttctagtttg aattattaca gtaacagaaa
818821 aattgtgcat acagtcataa cttgttgtgc tttaggtttc aaatgtagtt atttgtcttc
818881 acctaaagga tcatccaggt tgctttggtc atttagctga attgcctctg gtcatttcat
818941 ttttgtgata attatgtctt ctaaaactct aaaacactgc cattatagct gcataatgca
819001 gttgttaaat acgatgtggt gttgctttaa aacttcaata aaggtagcat agagtgcttg
819061 tttggtccac catcaatgtg cattctgttt catttttgga tgttctgatc tcaccagaag
819121 aaatattcat ttgattccag tggcaaatct ataatggtga cagttgggca aaaataaact
819181 aaaacattag taaaaagagc ttgtttgatc agggttattg tctggttctg gactgaacgc
819241 agcttcatca gacggcagca ggagcagtaa taaggtttgc tctgtggccc aactcagatg
819301 ggccaacaga ggccccgccg accccactcg tctccatctc ctcaccagct aacaaacttc
819361 cttagaaagg ggttagataa taaatcatgt cacaattaaa agccgcccgg gctgatgccc
819421 cgctccccaa cacgcagtcg attgtgctct ggatgaatgg gagaaccatt cacacgtcag
819481 cagtagggtt gactcagcac ttttaaagtt caaaccatga tgattcagca cagattcagt
819541 gcaggttttt ttatgaaacc taatacaagt attcaggtgc aacacttgaa cctggaggca
819601 tataatagca cgtttaaaac aaagcatgca cgctgtcacg gcgttttaaa ccaatgcaac
819661 agtttccaga agagcctcgt ttccttaaga cattccattc taatcactca acttaattta
819721 cgctccatat cacctaaaat ctggtggttg gggggagagc aaagcatttc atatgagata
819781 ataaaatgtt ccacttccct ttcactgatg tagaataaag gggaaggctt ctacagctca

819841 ctaagagctt cttataaata cgtcttcctc tctccgcaag caggtctttg aaaacactaa
819901 cagaaggtaa aagacggtga tgagggggag cccggagaca gaaaaatgta ggttgccatg
819961 tccctggtct gtacagccta aaatgctagg atgtgtaata gcacgtggaa acaacaagca
820021 ccgctacaag cccagaaaaa caacctcagt cccgtttgtg attccacaac gttacacaca
820081 ttaaaaaaag tttcatctct cttctcttcc tgtcctcatt tggcaaaact ttatctgaat
820141 gtcaaaagtt atttttagcg cggcagctcg tcttaattga accatatgga gtctactgac
820201 gtgactgaag ggagcgtggg aggcatttcc acttaaaagc tgcagcgtgc atggtaatga
820261 agtgaggaag tggggggtgg gggaaagggg ggggggtgc gcggtccaca gcgacgagca
820321 acgggtgttc cgacagcagc ctgacaacgc gtgagcgatc cgtttaaagg gatttggaat
820381 acgtgccgca tttagcccct ccacatacac gaaaggaaca aatgtaaaca catacaaggg
820441 caacgcttac agctgaacgt gatgcagtat tataaaaaga gctgtttcat aaaggatgga
820501 tggatggatc caaacacaaa ccaaaagccc gcatggccac gagaaacaaa aggcagcgtc
820561 gatttgtgca ggtcgggagc cggcgctgca gatgacatca tgcacttgtt gctcattcaa
820621 tcctggatgc ggtgacacat tcagcaggat gactcaacaa aaagacatca aaccacagag
820681 ctcactgcag cagagcgtga ccccgtgagt gccccccac cccagctccc agctccccac
820741 cctgtgtgcg tcctcttcga acgcagcctc gtggacgttg catgcaaaca gtgccgtggg
820801 aaggtcgctc aggtccatca tctcgtctgc catgccatcc tggtctggct cgccaaacag
820861 catgtcctcc tcctcctctt catcggggtc gctgctgtag gcctcggagc cgttgctcca
820921 ggctttggag ctctctcgga gcatgctgaa gcctttcagc ggacagttgt ggtggtaggt
820981 ggtgtaaaaa tagtaggagg gagaaaaatc aagttttctt ttatggctgc aggatacaga
821041 ttttggtgac acttggaaat cattcaaaat gtcattttga attactggga taaaccttta
821101 gatttagaac aaaaaggccc tgttattcat tagatttgaa ctagattatt ataatgttac
821161 aatacagtac aatgtccaca cagactgcat gctttaaaca tattttgttc acacttagat
821221 cttatgattt acaatatatt tttgaacgtg gcttcgctcg tcactgtcac tttagtggaa
821281 tcagcgcgcc atctagtgga cgatacgaaa aagaactctg actaatccct ttaacagaaa
821341 tgcagtatac ttgtcgacaa taatcccaac atttgactgt caaatttaat tccacacaat
821401 taaacacaac acgccatagc attaataatg ggtgcgtgat atatttcgac atcattattt

821461 gcgtcttgga aacagctgga tgttacgtga tggaaccgtg aacgcaataa taataataaa
821521 tcacatgagt ttgtggtcag atcatggtta tttatggaaa cactgctaaa ttcacgtcaa
821581 atagctgtga cgaagacaga attgctgaca caaggagcga ctggcggttt caatctgcgt
821641 gaaagcgatg acagtcgttt tactcacctc ccaagtttca gcgaaggcgg aagtgaccca
821701 tcacgaatgc acaaaaagcg ggatttttct tttgatttcg cttaatcccg tttattggac
821761 ctgttgttat tttagaaaat gacgtcacac gcgtcggtat tgcagagagc gtgaaggtga
821821 cgtcactcgc gttgcattgt ggggccacga cgccatcttt agagcaaggc gaatgtaaac
821881 aaaattgtcg aacacacgcc tgaggtgctc aaagaagctt caaatgcata gcactgtcga
821941 tctgaatcat ttgatgcaaa ttttatgcag aaaaataaaa acccagaagg ctcaattcgt
822001 cctcttgtat tatttaacct ctactcatca caaattcgac acagacatct gtctggctgc
822061 accagattgg gatcatctgg aacatataaa agggaaccaa aggatgaaag gtctcacttg
822121 tgtttttaca tgacaaggtc gtgttttaca gtatcagtct gtccagaaag ccatctgaca
822181 tgtaacagat ttaaattata tatattttaa atgtctgcat acacctgttg cagaaataca
822241 ccaaagtgcc aaaaacaata tgaatttatg atatatgccg acatagaaac ttcagtggca
822301 ccatttctca atccttagag taaagaatat gattttggcc cacggtctaa ctgtaattca
822361 tcccaaaggt gttctattgg tttaaattca ggactctgtg caccacagtc aagttctccc
822421 ataccaactc cctcatccat gtctttatgg accctgcttt tggtactggt gcacaatcat
822481 gttgaaacgg ggaggagcca tcctcaaact gtcgggagtt tgaggatgcc ttggtataat
822541 gaagcattaa gcctcccttt cactggaact gagcggccga gccaaaatcc caacaccaca
822601 ccataacccc cccccaaact ttacactcgg cacaatagtg atacaagcgg tacagatcac
822661 tccagagaac acttcttcac cgcagcacac caccattgca tcctacgctt tgcattgtgc
822721 ttggtgatgg aaggttacga cacagctgtt cagccatgga aacccattct ccgacactct
822781 ctatgcactg tccttgatcc gaaggccaca tgaatgtgga ggtctgtagt tactgactct
822841 gcaggacgct cgcgacctct gtgcattcaa gttggtcgtt caagtggctc ctatcctgag
822901 ggtgaccgag aagcagtctg catgcaaagc tgcgtgatgt tattcctgtg gcaatggaac
822961 tgattggaac acccaaattt aatgatttgg acaggtgagc agatactttt ggcaatatag
823021 tgtacataat agaggaattt agtttgttcc aaaattatat atacagtata tatactgtat

823081 gagttagttg gagcgtgtat tgtgccatag aataactgtc atgtgatgaa
tgtgaatatt
823141 taatattatt tggtgcggat tgattgttga ggaatccact tagaaccaaa
actgttttta
823201 aactatatat aaaaataatg caaggtttac acacattaag gttaaaaaag
caaagaaaaa
823261 agtcaagatt gggctcaaga catgacaatt accataaact gcgtttattt
tatcaccaag
823321 ctggactaaa tttacagtac agcaaacatt gctccgtttt cattctgcag
cagttttgat
823381 ttcacataca cacattagtt gtgaagagaa tctgattctc gtggcctttg
attcagcaga
823441 aaataaaata aaatatcctc agtagcaaac actgaactag ttctacagtc
agtgatatca
823501 agaaacgagc aggattcctc atagattatc cagtgtaaag tgaattaaaa
ttaatttaaa
823561 ttcataaata caataaataa aatcaggtgc acccgttgtt tggtcacttt
aagaagagca
823621 atgacaagaa taataaccca gttcatactt gttgaaaagg ccccttggaa
gctccgataa
823681 gagaaaaatg tacaacagtc atcatttaag tctaaaaata caaacaggta
gtaagtgaag
823741 gcacagcaca tcactgctcc cagctctctg cagccatgat tatggatcca
cctaagccca
823801 cagcagcttc tgttgtcata ataaacagtc tgacatggtt gcattctgct
ttccaagggg
823861 aaaagtatgg agaacatatt aaatgaggtg gaagctccat ctcagtggtt
cacaaaaaaa
823921 gtgaagagga tttcaattag tcttaaagtc aagtgggccg cgagctgcat
cagtggatct
823981 cgacctgatt ggtggctggt ccagtttcac tggtaacgtg gtccgagcca
ctccatccgc
824041 gtttaccaga gttccgtagg agaaggagcc gttgaaatcg accaggcctt
tgtcgtggat
824101 agttgggaca ccttttgttg aaaaggggaa gttggttggt tcatatgtga
cagtgctgtt
824161 agtgcaattc caaaaactgg ccaaacagaa caactaaaaa ttacaacaag
aagggactga
824221 aactaaagcc agtctaactt aatatccaag atatacaggc atttacgtac
cactggccat
824281 atccatagtg acaaaaggtt caaaggtctg agggtttttc cggtttttgg
taatgaggaa
824341 aaccccgagg aagcagagag cagatctgta gaaaaagcac aatagttcca
atgaaattcc
824401 tctgtttata atcattttct gaggaaagaa aatcatcaga atcagaatca
gaatcagaat
824461 cagaatcaga agaaggttta ttgccattgt tcatgtaata cacagtatta
cacaaactag
824521 gaatttgtca tggtgtgccg ctgcatgttg tcctatgtaa atgatgtctc
actcacccca
824581 gcaaaaacaa acagatgtgt aggacatctt cacttttaaa ctccaggtaa
aacacagcgc
824641 ctggaaaaac aaattcaaca acaggatgtt tacagtttag agcctcactt
tcgatttact

824701 ctgcgaggca gcatctgact actcaccagc tacaatagca aagaacgtgg agaggatgta
824761 gttgacgctg gcgatcagag agggctcaaa cagcttacag gcctgggaaa ggaatctgac
824821 aacagggacg tggatattta taaggtaact tcagatgtaa ggtggtgatg tgctgctgtt
824881 ctgacttacc gagcctggaa cacaattgag gccaccatgc acactaacat gacgctgaaa
824941 atggggtagt tgagctgcat ggtgcccctg acggtgagga ccagcatgct cgagagtgcc
825001 ttgactgtaa tgactgtgac agagcctgag cacaaacaca cgtttaatca caggatgtca
825061 atatttttgg cagcagcagc ttgacgttat tgaaaatctg aagaggttca ggctttaatc
825121 gacagactca ctgagtagag agaccagcag cacaataacg atgaggtagt ttgcccgatg
825181 ctgtttgtag aagtataaaa gcaagcagaa tgtgatgatc tccactagct gtcaaattaa
825241 atcataaata ggggacacgg tcagtaaaaa gctgcatgtt tcatttgact aaacacaata
825301 gaatggacgt aaattatcaa atcagaaatt ctggctccag ctgagacttt actcatttta
825361 ctacttgcag ggttataaat atcacaacaa ttttaatgat accaccagca taaaacaagg
825421 agtttgtgat gcccagccat agcacactca taaatgctca gcaaatgcct ctcagaggac
825481 tcgtttgtga acaatcctgt gacagatcag gcacttggca cacacccctg acagacagac
825541 atgtaaagga gtcataaatg tgccttttc accaccagag tgaacagcat aacagcttta
825601 tatcagttct ccttcgacat acagcaactc tgttccacag gacacttcct aaaagagcag
825661 atgttcttac caggtacagg agaacaggcc atgctacaac gtgtttgacg atgttctctg
825721 ctttaagttg ttcatgggag tttggtccaa atgtcacaaa gagataaatg gcccctatgg
825781 ttaacacgca gccaaagaaa gacagtccgt agcgctctga aatgacagtg gtttcaggat
825841 tcaagatttt tatagtcacc atcgctgtgc atgcacaaag cagattagca tggttcatgg
825901 agactgttag aatctgacag tggatttaaa taattgtact ttaacaaaca tcgacagaca
825961 tgtattaaga tcataagccc agggaaacta atttctagaa ataaaagttc ctggaattgt
826021 tggtgaaaaa ggggctatat atattttttt tttattgtag tcctaagatt cagttatttt
826081 aaaatgtgat ctatcagaag actcacatac caactgaaag tcccaacacc agccaagtac
826141 caaaatagaa gagaaataat aacagtttcc tacagttgtg agtgtttgat gaacctgaag
826201 ttgctgtaat tcaggtatca gtgtccacag cccagagtgt tttgtcacta tgagctgagg
826261 aagcgtctgg gaagtcttaa ataaaccagc aagatgcttt gagtggtttt caaagtaaaa

826321 aaaaaaaaaa aagccttaaa ctatactatc gaatggccat aggaattgag atggtttact
826381 caatttttag tccttttaca tcttcttggg tttgggtact acttcctggg tttggtgggg
826441 ttaagtatag ttcaaaagga gcctggggtt acaactacat cccacataac accagagcca
826501 gaacacttgg gattactttg acattatgat acagggacag gaaacatgca gggacaatat
826561 atcttaaagg gaaaacctat gtgtaacaag caatagatag aacaggagtc atgtggacct
826621 tttttgagga caatttcaaa gcaaaatctg acatctggct gtgtattttc tcaagtgtct
826681 tgaagaaatc agcaaaaact gtgaatgatg tgatgtgtta cttgtgagcc acagtaagaa
826741 acgtacagtt acacggtcat gctgaactta cttaaaaagt cctttgtctt tgacttctca
826801 agcaaaaaaa gaaggcctaa aattgagctt gctggagaca caaagaatat atttagattt
826861 ggagaaaaat aaagtttaca tttttaatat tcatgcaaca gtctagaatt atatcaataa
826921 tactaacggt tcaatactca ccaactatag acaccgcatt aagaggagct atgagagaaa
826981 ggggagcgaa ggcataagaa acaaagttag caccttctcc taagcaggta agaataaagc
827041 cgcaccacca ggtcttagtg cggtagaacg tccgggggtc cttggctcct gccaacttca
827101 agtggctata tttctgtgta ggtaagagaa aatgtcacga gtaaatgttc agtgcagagt
827161 atcatattaa aactcataca aacataaaag cacctaatgc atgcagcggt agttggtttc
827221 atagtaaaca tgctgttaga aagacaacag agccaaatac aatgacccga gtattaaacc
827281 aaactctaaa tcaggctgat ttaataaaga taaatgcagg tctccaatgc aaaactcagt
827341 gtagttattg taatgaaatg taaacacacc tattctaatg aagggaccct gaagctacac
827401 tgacagtaca actacatcag atgaaatgat gagcagaaat taaaaaatgc aaaatatttt
827461 aaaatttaaa gagagcttat tgtgtacctg aatacagaga gagatgctga caagcacatt
827521 cccaaaaata gcaagtaaag ttccaataag gttatcctga aagaagggaa aaaaagaaag
827581 aaagaggaag aaaaaagtta gtttatgtta aaaaccagaa aaatacaaat cactataatg
827641 tttacgatta tgaaacatct ttcattttat tctattgtca gcagagcctt gaaacctccc
827701 ggatatctta tagacacgag cagaaatcta tattatactt ttgcaggcgt cgtccataaa
827761 atataaagtt gttgtttttt aatctttttg gaaaagaaaa caaacaattt tccggagaaa
827821 gcgtggacca aagtgcctca ccgtgtatga acctgcgtcg ctgtggctgc tgtccatccc
827881 gattgtcttc cggcgctatt ttaattgtaa attgtagcca tgagttcaca gaccaggtaa

827941 ttcacctcgc cgctaatgta aagaatgtgt aacgttcact tcatggtaat tatttacgct
828001 gcttcatgaa gagaagcgcc tcacttccgg gttgtcctcg ctttgatatt gcgtggtgac
828061 gcaatacgtt agaatgacgt aagcgtcact gtctttccga aaagaaagta taaaatatat
828121 cacaaaataa taagacgcat ggatcgaaat ataggtcatt gcggtttgat aacagtcacg
828181 caagcgtgtt aattcgtttt attcaattta tgtttaattt tcatttctat tttgaaatgc
828241 agaaccactt aaaaacagca ggttgggcta tcgagccata ttttacagat tttaagcggc
828301 tcagaccgaa tcgtcaagtt gaaacctttg agctgtgcat gtatatgtac aaaggtatat
828361 aaatatcatc ataaaattaa atttcgtgtg aagtccctat ggtgccataa taattctatg
828421 ccaacatcac attgatatta tgacgaacgg gaatggaatg tttttctcct attatttgtg
828481 ccattacgac agtaaaggag tgacacagta cgtcaacaac attgtttcta ttaacaggag
828541 ccaagcaaca attgttcacc gaaggtcagt gttgtgtgtt ggcacagctc tctctttttt
828601 tattaccaat gagtcatgaa ctcttaacag caaataaggg cacagtcacg gtgagtccag
828661 cgacccgtag attaatacag cctcagtaac aaatgattaa gattggtaga attgataatt
828721 aaccacctct tcaaattcca tcaaattaaa aatagataaa tgggcattta ttgaaggatt
828781 ggtttactat tcatatttta taaaagaaaa taaacaaata aacgttcact ccatctcagt
828841 gatttacaaa ttgcatctga ctaaagtagc ttgttaaaat tgacatttta aacgttctac
828901 tagcgtagac cactagagaa cgccgaattt ttctacttta gttccttata tattgttttt
828961 aaaatcttaa cttaaccagg cacttttgaa ggttttttgg atttttaata tgctgtcatg
829021 gacggatgct ctgagcactt ctattattct atttcatagc aattatctta aagtaattaa
829081 aaacatcaca gctgaggtca gcctctctga agtatgaggg tggatcattg atctaagatg
829141 taaaaaaaag aaacaacaaa aaaaactgtt aaagatgact aattctattc acagtaactc
829201 aaggaaaaaa tattctttag tctgttcagt catatcaggt gagtttaccc aaaacaaaca
829261 ccccataaac ttcacctcat gagaaatagt tgtcttattt aatgtctgtg ttaatctgtg
829321 ttaatgtgct gtaatacagt attttaggga agtaatcttt cagggaatga gcagcccagc
829381 tgggcctaac agatgataag atgctttatc tctggtcctt tgtttccttt caacggactt
829441 tcctaatttt tttttttttt tttttttaa ttatttaccg gataaacttt ctgcttggcc
829501 tgatacgttt gacctgatga agctggatca agttgtcagt gaaacatttg tggcttggag

829561 tcatatgcac tgaaatacca atcctgaagg actgtaaaag tccagaatga cccaccatta
829621 ttatttacat gcgtgacgat aggggagaag ctgctcagaa agggttatca tttaaacatc
829681 atctccctct tgacctcaga gctttggccc cgcctgacaa ccttcgtgac atgttggaat
829741 gaaaccctgt ggtacagcct gtagctagtt atcagacata ctctgattat aacgtagtag
829801 cttctgctgt aaaagtgtca aacaagggct gttgtcacac tgttaactgt tgtatttaga
829861 ggcatgacag taaaaacacc tccaatgaac aaatgaaaaa gaaaaaaggc ctttcaatta
829921 gaattgttac tgttgtcaaa gactataaat gatttaattc aacgtagtta ggtttaggtt
829981 taatttgctt ggttttattc aggtttaaat tgtttttttt acagttcact aagtgtaaaa
830041 cttcctaata atgttgaagt gctgatgaga tgatggaatt ttatatggag catttcaaga
830101 acaaatgcac ctctttcctt ttacagttat aagtaattgt attgttctgt gggaaggtgt
830161 gtcccatccc agggcctcac tgcaggacat ccagacctcc ttcatagtgg acagacctgt
830221 ttacagccac cgctgcactt gcgggcattt ttgtgtgttt ttgtgtcagg atgagaagag
830281 agttgtctcc cagatattgt caaaatgcca catgaatttc cagccaacac ccaaaaaaca
830341 tgattttgca aatgtgtacc aaattgtcac atttcttcaa atgcaacatt gtacagtaat
830401 ttaaaccaca gcagtgaatt taatttaaac aatttaaatt tacaaagtaa atttacaaac
830461 aaaaactact gaatgttttc cattatttgc ttcaaagatg acataattaa ccatatcaaa
830521 gatgtagatt atcttctgat cgcctgactc actcaccatt tccccagatc tgattgcagt
830581 aattatcagc cgcttttaga tttctctggc accaaactga aaacgatgaa tcaggggggg
830641 caagcttata cagccaacaa atcctgtaat ttgcatctgt aataattatc tttgttgata
830701 gaaaggtgaa tttttcagtg aaaaatatca cgcattttct actcgctcaa tatcacacaa
830761 caaaattggc tttctcctcg tgggcgccac ccgccatgga ggaatgtgca gacgttagta
830821 aaggatgtgg aagtctattt aagaaacagc taggcctgtt ttaaaaaacc aaacagacca
830881 gcagccgttt gtaatgactg aagaatgtgt gtacgttaga ttggagcagg gattgaaggg
830941 gacacattcg ttattatatg gtgctgcgca acaggaagtc atcagagcca tgaggtgata
831001 ccgcatctac aggaatgtta tgacaggtta tgaaaaggag gtggaagagc aggcttttcc
831061 taaaccctct gcaggcccag acttgttggg aacattggga ccgagtgaca atgacaccgg
831121 tctacattaa tgctgagatt aatgaatggg cgctcacaac tgtgggtgtg atagtgactg

831181 atggcgaatc agctcgaaac caaagcttct ggctgtatag gaagataacc agagaccgat
831241 gtcattttcc tgtctcccag gagcacccac agtctcgcta atggaacgct gatttgaaac
831301 tgtgacattt catatttcat ctcagctctc acatgtttta gaatttgaat tgcttgttgc
831361 tgctaaataa atgaatttaa atgaaataaa agaatggatg gattttatct caaccactga
831421 cagacaaatc ttatatgtgc tgcacagagc ggttccaata accttgttac agcagccctt
831481 ggcctctaat atggaagctg ttatttgttt tcataaatgt cacagtgatc attttcttta
831541 gcagccgaga ccttgcactg ccttctgcct ttagatatat attgcacaga tgtaaattgc
831601 atttaagctc cttcaaaatg agattttgga caataaaatc atgaaaacat gattagtggc
831661 atagggtttt tggttcaagc cccagttgga aaatagactg gtcgcaggag aggttccagg
831721 acactttcaa ggcactgcca agatactctt ggtcaaggga ctgaaccctc aaatgctcca
831781 tggcagctta atccctctgt cacctttccc ttagtgagcg tgcatgcatt caatgtacgt
831841 tcacgtgaaa cagcaagtcg atggggatta aaatgctcca ggagatgcga acatgtcaac
831901 aggaactgaa ttgttatgca gttcacgtaa tgttaaagtg ttttaaccat gcattgctgt
831961 aatgtgactt catagatcct gatgtgattg accccccgac cccagtatgt tgtgagaacc
832021 cacattcagc ttttctcacg gtctgcggct ctctttagtt ttaaacccgc agttacgaag
832081 cgagtaaact aaaactttgt ctcaactctc gaccccggtg aactgtattt attacacatc
832141 tcagtgtcat ggtgtaagat acattttctg agcttgtgtc aggcaaggta gacaattttc
832201 tttgtttcag ttttagatta ttactcagac gctgttgcag tctttatttg ggatcacctg
832261 ctgctcatgc attaataata atccatttag accctgtgga gggtggttta gaatgctccc
832321 tccacagggc gtagtgataa gtgtccctat atgtactgcg ttacgaccaa gggactcaat
832381 cctgccagtg agagacagag agagcttggc tgtctgtgaa atctgaggag agagagggag
832441 agagagcgcg aggagatcag agggaacaaa agactgtgtt taattttctt ttccccctcc
832501 tctgttgtct ttagtagttc cttcatattt tcagcaccct gcaatcacgg catcttgtta
832561 gctttggatt caggttagac ttgttgcagg agacaaatcc cctcccaccc ttcctccctc
832621 cttctcctca catcttcctc cgtcttccat tcacgcaccc actttcatat gcgttctctc
832681 tttttaacag gtgacaggcc gcatttgtct tgcaggcgag actgtcacgt ccattcactc
832741 catcgtcgcc tctcctcctc tcctcccacg ttcctttggc agcaggtgaa ttctggttga

832801 gaggagacgt gtcggcaggt gcaccaccgc agaattacag tggagatcga ctgatttaag
832861 cccgagcatc tgacttttgc atctttaatg caaataaggc ctgaataaga tgtacatttt
832921 tgatttattg aatatgtttc tgttgtgact gtatttcaca gcagcactcc tgttttattc
832981 ctgctactgc tcaaattgtt ggagcagttc tgacatttca ctcacttact tcctgcttcg
833041 agctatctgg tttgaggcaa gtgttgcaca agatcttatt cggataatta cagctcaaat
833101 gtatcataaa taccttctta ttgtcatgtc aacaccactc acctgaagca ttaaaaaaac
833161 acgagagacc cacagagcat ctgcaggtgc ataaaaatat ttaagatcgt gtcacaaatc
833221 tgtgctaatg aaaaaatctg gaggctgtga ttgatgtaaa aagagcttat aataatcgta
833281 acactattaa aaaggacaaa aatttctaaa tattttctac tatcaaacaa atgagcagat
833341 ttaaaggaac atctttaatg atgctgaact tggttacttt ggtggattaa tcctagaggc
833401 gatgaggatt tggcaggatt atcattcacc ggccctccat gaggcgcacc ctgtcgcggc
833461 ccttgagcac tgcatgccgt gactgttccg gaggactgaa gagaaacaga gtgcgtggtt
833521 cctctaaatc tcgggcgagc tgagacacgt agactgaaac tgggcacaag ctggtgaaag
833581 gaacagggag ggagagcgtt gtggggaagt atcagaggtg aaatttgttt tcctttcatt
833641 cacgcttcac tgggctgagc ccagaacctt ttttccttat atattgtcag gttttttttt
833701 tttaatgtat tccttttttt tctttttgat atgaaggcta ctggttaaat aataaactgt
833761 agaaccatcc aaataaaaag ttgaatggca aagcggcgtc atgatttatt tttgtaaata
833821 gacatgatga attaggagct gatgaagagc cgcagtgaaa aattgggaaa tatcaaatat
833881 tcattttaaa tgaagaaaag aacgtgttgc ctagaagcaa gagcctggtt tctgtgtgag
833941 ggattcatcg ctgcacctaa tgtgaggtgg tctgaacatg ttcctttatt tggcatgatg
834001 aagcaacatc tcctggaaag ttctcctcct caactatccc acgggaatat caccactcat
834061 ccattgaaaa gagggaaagc gagggatgaa ggctggatga actgaaggag gaggagagtg
834121 gtgatgggat gttggatggg aaaggaaggg agatgtttat cgtttgtgaa gggatgttta
834181 agtgtcatat tctcgccctc atgttcttca gtttccactt tgtcacacag ctgaagtctc
834241 cagccaaaga gttgcgcaga catctgaaac atttaagctg atgctcacgt tgtcgtctgt
834301 ggaatttgag ctgtagtgta tctgcctcag cttcacggcc cgcgtgtgtt gtgttaccgc
834361 ctgcaggaac tcttcagtcc ccgattctct ccagggtaca gagggcttga ggctcctgtg

834421 acagactgat tgtgtgtcat ccaagttatt acaactccct gaggaacaag aacagagcat
834481 gacctcctct gagttatagt tcccgatgca gctttaaaga acaggtccta cgcagtgaaa
834541 ttgatgcaca aatttcacct ccactggaaa cgtgcacact ccactttcct tcttcagtca
834601 ataataactc cccaatcctg aaacccagtg tgcccatgtg gatatgcagc ctcagctgtg
834661 aagctgttgc ttgttgagct tagcgtaaag acctctttaa tatgtttctg gaggattgaa
834721 gacaccttac tgacctttta ttatgactga caatcaagca acatcagcac tcatttaagc
834781 ctttcaatca tttttattct ggttacaaag gcagcgtttg tgacatggct aacccaaagt
834841 ccttcagagg tgaagctaaa caatgttctc agtgaacatt aaatcttctt cccatgaata
834901 ttttcaccat aacaaatgtt ttatctggag ttctatcatg ctccttcgcc ctctctgaaa
834961 atgttttttt tatctgctca ggacgatcat cttgcctgat ttgcttttga ctgtggtgaa
835021 tagagcattt atttcttgtg aattgtgcat gctttgaaga gaatagctct ggttatcaat
835081 caaaacagag caaacctttt tgtggcattt tcatggtatg tgagttaaat attgcaaaat
835141 gcacaaagat ggtgaaatga cctcaatgtc cacacagaca tactggcagt ggttcatcaa
835201 tcccggtcgc agtgcagtca agcacatttc tctcccatta ggtgtgaaaa gattgggatt
835261 ttgggtttct ggactgccgg cgttattcta taactttatc tttgatgcag atcattcgtc
835321 gatctgagac agagataaaa tggtgacacc tccagatctg cagtattagt ttgcatttgt
835381 gtctcgctgt aagcatgtgt aggtttccac ttgttgaaga tgtgcttctt tttttgtgcc
835441 tgcagcattt acatgtcatc atttgttatt ttcatacctg ctctgttgtt catagtcatt
835501 ccataccaaa ctgtttgaca aagaaattaa agagccatcg tcatgaggac caaagcaggt
835561 agcggatgga catttgctgc ttcagaaata acccaggcag ctgtaaatag ataaatagtc
835621 cattttatat tcaggacaca ccagaagatt cttcattttg ttttgtttct tcaaattccc
835681 ctctaactac tggctcagga cacaaggctc tcaggctaaa ctaggcaaga gaattgcagg
835741 ttccccttgc cccaggtaag tgccgtcctg agttcttcca ttatgcaaat ataaaatcac
835801 agaaactctg acaatggggg cagagctcca aaagtcttca tgctctctct gcctctattc
835861 acattaatat caatgtgact gctgttcttc tttcaagcag accccaccta cgccctccaa
835921 tttacctttc cttccaacac cccttttcct aaaccacctg cactcctccc cctccagcct
835981 gatcctttac tctgggctat gttcagtttc tgggagtggc tgctgttggc tgatatgagg

836041 tgaatttaaa aaaagtgagt ggaggtaaag gaggtgttgg tgttattggc gggggtgtcg
836101 gagagtccct ccagactctt ggagcctctt ctagtctatg ccaaacaaga gtgactctac
836161 cagagaggtc tccccctcca cagcagtatc gtggctccgg tgaggtcaaa gatccaggaa
836221 gaggtcaaca aataatgcaa gcgcacatgc accaacagca gctgtgcgca ctgtttgcac
836281 acgtgaatgc taacggcact cttgcacact tttccatttt tcaaatttca ggttttcatg
836341 ctcaaaactc tggtgctgag acttcatacg aataagttaa atctgtattt cgcagtatca
836401 gtaaaatgtc taaaacccgc aaaatacctg actcaaagtc cgatgtctgt ctttattcat
836461 atttgtcatt tttatgctgt ttattgcatt tcaagagaac tgtgatcagg aataggaaaa
836521 cagtttttga ccggccaata gtttcctttc tgtgcctttt cgctcaacaa ttcagatttt
836581 tgtaataagg tggttcaggc gtgaatcatt gttttacaat gccgacgaca gtgttgaaaa
836641 aggagatatt acggaaagaa aacggagcaa actgaaaggg gaagcacggc agctttacat
836701 aactgtggcg gagccttatg gaaacaggtg catccgtgga ggattttcag aatgcaatgc
836761 actgtaggta gagatcaacg tgccttgggg cccataaact atgtcaaaat catgacacat
836821 tcctccatga cacaccagga agatttagag ataaagaaag atgagcagat aggccttctt
836881 aaaaagagct gtagccgctt tatttcttca actcctttca catgtttact tgagttcaac
836941 tgtttatact tgcagcctcc tggagattga ggcttgatca aaatgtcctt gagttgttaa
837001 ctctggggtg agactctagc cataaagaca caaagcaagg aaccagacaa gatggtgaaa
837061 ggagccagga gaaggagaaa gagaaagaga aacacagtgg gaggaaaaag gtgacgagag
837121 acgttcttct gagcagcagt ggtgcgtgga gcagaagctg gtgcctcttt agccaccatg
837181 catggcgcac acacacacac acacacggag agatcagtca cagacaacaa cccaaacaca
837241 tgacactggg gtttgggttt ttttatccag tctgagtgag tttcagggca tgaaaggcaa
837301 tcgtttgaac atctatcctc ctttcgccgt cgttcccaca ctgttggatt gactcagaat
837361 ttctttgcat actcgtgcat cagcgggttt ggaaagacat aagactgagg agagaacaaa
837421 ggagctggat gttttcaagc gttagctttc acaccactgc caggtaaaat gtcagaaagt
837481 cgaagagagc aaagatacct caggtgagac ggagcattca cgccctaaac gagccaccag
837541 aagagtctcg gatctgccgc atctgatcta aatagttgaa gaccaacggt ggaaatctgg
837601 ttccatttag ccacatttgg aaagtttaag agacactttt tttactcatt cttgtgagga

837661 tacacagtga ttggtgcaca cacacacaca cacacatggg cacacacaca
gacacacaca
837721 cttttctttg agttcgtgca aacttgcaaa tgcatgcgtg ttagttgcag
agggtgtgtg
837781 agtgtgtgtg ggcaaactac tatgatcatg cacttttggg agataccaca
ccagatcgcc
837841 tcaggacaga cagacagaca gacagagctt aatttggatt tattttaaaa
tgttatatat
837901 tcttatagta tactatagta ttgttttaat tatgttgcat ctgaatcatt
tgtacaacat
837961 taacagtgaa aaaattattt aattgatgaa ttacatttct ttacattaga
atttgacatt
838021 ttggggggaa atttcagggt gaaaatattt gtggcagaaa ttggaatgaa
ttaaatagga
838081 actttcattg aaaattcatc ataagaattt acaggttata taattcactg
atttaaaact
838141 ctacatggtt gtcaacaact gtgaaaccaa ctaaacattt taatgtcaat
cccaattcct
838201 gctggtctca gctggtctca ctaaaatatt cacatttcca tctttaagtg
aagataaaaa
838261 gaccaaaacc tttgcatgtc atggaaatcg agaggtagag tgaaaaatga
tactggaacc
838321 cattaacata ttaaagctga gttcctggtc attagtaaac cattgacacc
tcttagattc
838381 ccgatctcac tcttttatgc aggctgataa tgagattcaa aaccctcctg
ccttggattg
838441 tcttgcaaag gatgatcacc ctcacgcaca tgtcagccag tttctctcca
gcctgggaag
838501 agttaatgat tctacacctg tcctatccga cacctgtttc agtcagccaa
tcaggtgaga
838561 gaggaaaact ttgctggcat gaaataaatc tgggccgtat ctgaggaatc
acaacacaac
838621 tcatctgcgt gtgtgtgagt gtgtgtcagc gctctcttct gccaaaattg
acggctgact
838681 gacactcagg agaagaagct gaaattaaag gaaattattc atctcacctg
gacgagagag
838741 atgaccaagg agactgagta agtttgagga attctaattt attttgcctt
gaaaatctga
838801 tcctttctca tcaaatggtg cttataacca catgagaact tagactttta
tattgctttg
838861 tctgacatcc atattttggt cccatttaat agtctttata gtctagtctc
ttggagagaa
838921 actcccatcc ttcttagctg caatgagacc cccatgtgaa aaatccaggt
ttttaagaaa
838981 ggggtgtttt gcagaggaaa ctgatacgga tagctgaaac acacacatac
actcggtcag
839041 gtgacggcgg tggttgcggg tctgtttcgg cgggtgttat cactaaccta
gaggccacac
839101 agcacaaaag gggacttggg aaataagaat ctgctcatga ggagacaatc
agggggcaat
839161 gacagcatgt attgcattga attacataat ggtgaaattc tatacatgca
gccaggtggt
839221 tttaggggga gatggggatt tgttggtggg acggagtggt gctgacttcc
tgtagctgat

839281 gtaatgttaa ggtagagaca ggtgtatttg aaagtaagtc tatgaaggtt acttggctca
839341 tgtcccaaat gtggtgattc tagtttcccc gtcttattaa atgcaacctt aattaccttt
839401 tatttatccc tgtatgaaat tgagacattt tgtcacatgt tctttctctc cctgtcgaca
839461 cttttaatta gctgttgacc cagagtaagc caaaagctat ttgttcatta gctgccacgg
839521 cctacgtttc aaaaggggtc ttatcctggt gggcaggata tttacaatgc aatgtcaacg
839581 tctctgcaaa cagatctcca gtctcaaatc cagactttcc tccgattttc tttcatttct
839641 gacatgtgat ctgaagatcc gcactgatgt ttgaaatcag cttcttaaag atcagggcaa
839701 acatgagagt ttatacagca ctgcagctgt gcctctgctg gggggccctg ttgagggggt
839761 tagcagtggg tggccatttg aagtgatttg acacaacagc ttctgctggg tgttagtcag
839821 ccctgtgatt ttgcaatcga aaccttcctt gctcaggggc aagtgtgtgc agtgtactgg
839881 ggtctattta caattactga gaaatattta cagttgaatg atttatttcc tatttatgcc
839941 tccacacact tgacttgtat aatcccacat ttctaaatct gtctgtccct gtcatttaac
840001 tctgtccttt ctgtaggact ctgggactgg tttttcagag cgagaacttc aactttcacc
840061 gttatagtaa cctcatcatg gacccctggt cctacataga cagccctgtt ccagagcaga
840121 accactccaa acccagactt cagccaggag acgacctggc agccttaacc atgctctatg
840181 aacagtgcaa ggtatataaa gttcaataac tcatatttaa aaagtgaatt attatttttt
840241 taacaagctg ttaatatctt ttctacttta ttatttcaga gccagaaaga ccagaagctt
840301 gttgcatgca accgcatagg caacatctgc aaaacagaaa agtacgattt ttttattttt
840361 ttaaattgta actagaaata tacagtaggt gtcattgcga ggaaaataaa cttgaattaa
840421 cctttacaag gaacaacatt cctgtgtttg aaaagtgaca aattagtaaa aaaaaagaaa
840481 aaaagtagaa ttcaattaat aatcactaag caattagaaa gagaagggct ggcctttccg
840541 aggtcataaa cgggtgattg ggtaatcctg gagccaaaca tgcttccatc catctatctt
840601 ccctccatca tcacccctct gccgcggttt taaagagttt cccaacagac agacactcgt
840661 ttttccctcc ctctgagcac actcttctct tcgcccctgt ctgcttaccg ggtgggtgag
840721 agatggccat tgttgggggg tgcagtgttt ccccttcaaa catttgaagt ggagaaactc
840781 aaacacggct gagaaaggaa ggtggattag taaacaaata aaagccaccc acactcggcc
840841 ccagtctttc tgtgagttca cccttgcagt agccgtgtgt gccgaaagtt tatagaccag

840901 tagtactctt acgcaggtgg tgtgtttgcg ctggcagccc gcaggcttct gtaccgctta
841021 gtctatttca tcatttgatc agctgttctc ccgcctgata ttttaataac tttccccacc
841081 tcgacaggac ttccaccagc accaatgact ttgcgcattt ggagacgtcc cccggcgtca
841141 tgaagatcct ctctgaaaac attcccatga gtcaccctca ggaggtcttg ggttccaaac
841201 tacacacatc actgcccagc actgttcctg ccacctcgga cacctatgcc accctgacta
841261 gtgttgtaac agacagctac gacaagcagg agctgaacat catctttgac tccttgctgc
841321 aaaagtggcc ggacacgggt gctttttctg atcccaattc agaggtctgt ttggtgccac
841381 ctgttctgtg ctctctggtt ttttcactgg tcttttttga tgacccctgt tctcattttg
841441 tgcatttcag gtgctctcct acagtaatcc tttccctgag caccagtcca gcccggttta
841501 ctctggctcg tactctggct ccccaccgga aagtttcagg aatgagttcc agttctccct
841561 gggagctccc atggcttcac cttacaagtc caatgaggtg cccatggtct atctgaacaa
841621 gggtcagttc taccccatca ctctccatgg agtggacagc agtgtgtgcc tcaatgtcac
841681 caaagtcaag gtaagtttag acatttgggg ttaatggtaa aagattccat ctattcatct
841741 cacagttgta tttacaaagg tcaccagttg catcaattct ttgtgtgtga ccaagccagc
841801 ccttcccaaa tatctgtccc tcccttggca ccagctaaga aacactgact aattccacgt
841861 tgacacccta cacgcagggc cacagccatt gaatcaacag tcttgcgagc acaaattatt
841921 cctacagtgg tgtaatgagg ccagagtttg acaagactca gtcaaactcc atttgtctga
841981 cacgagcaca tggaaagaag agaggagtaa ggagtggact gctgcgcagt tttaaaatgt
842041 gacaatccgt ttctttgtta gacatatcaa ataggaatac acacgacaca cctactcact
842101 gcaaataacc tatgacactg tgaggagaaa agggggattt ggagtgataa atgaaatgtg
842161 acggctctta aaggagtcac agaatttaac gaatgccttt cagctcagtc ttctttcgtt
842221 gcctcctgca gacggtgatc atggccgttt ttgaaaatga aaagagtcca gaaatgcagc
842281 tccgcttctg gaatcactgg catgcgcgcc agccgactgt gaaacagagg gtcatcgaca
842341 taggtgcatg cttcgtaaat ggaggaggtc tttgctcacc tgttcggcag atagagagtt
842401 aaaatgtttt attttctctt acagctgact acaaagaagt atacagtggc atcagcaata
842461 ttgaggaggt ggcgttcaat gccctctctt ttatctggaa ccccactgag gaagccaagg

842521 taaacagatc actgtcagtc tatgttgggg aatttttctg taaggacgtt
cctctgaagg
842581 aggaaggaac acaacaggtg gaggatttca aagaagtagt gaggaaaggc
agaaaaggga
842641 ggtggtggca agtaagggag tggtggcaga gggactggaa gaatcccaga
aatgtgttta
842701 gccccgaggc tcctgtcacc aacagcagga gtgagctatg atggcttatt
catgtggctc
842761 aagggctccg aggtggggcc accaaacttg ttgcaggtgg aggagcaggg
agagaagaaa
842821 ttctcaaaag aatgtgagga caggaaggcc caaggctgag tcttaattta
gtaaaaaaaa
842881 aaaaatgtag aaagaaacgc acctaaaagc tgagtgaaaa gggatgaatt
ggccttcaaa
842941 tgtctctaca aaatgtctgt gtaaattctc agtcatccag gtcattgtat
ccaaggttga
843001 cagagtctct acaaaaacgt gagtgaccat tttccccttc ctccaggtgt
acattggcat
843061 caactccctg agcacggact tctcctctca gaagggagtg aaaggtctcc
ctctgaactt
843121 acagatcgac acctacgata tcagctctgg aaccaaccag ctcatacaca
gagccgcctg
843181 ccaggtcaag attttctgcg ataaggtaag tctctttacc gtatcctatg
cacaaaacta
843241 aaaatctggc aggtgcaaaa tgtaaccaca cttttgtttt cttctcaggg
tgcagagagg
843301 aagatgcgag atgaggacag gaagagaagc aaaaggaggg gaaagaatga
tgctggtaag
843361 tttacatcgg tgatgaattc ctgaagagta gcctttggag agaatagttt
aacactttgg
843421 gccatcctgt gtatcttccc agtacacaag tccatcgtga gcagctctat
cagcagcgaa
843481 tgcaccttct tccaaactct ggatgaccat atgacccagc cggttctctt
catcccagaa
843541 acacacatct caagcctgca acgcatggta ggtcccaatt tacacaactt
ttctctcctg
843601 agtcggccgt gattccgact cctgcatccc cacaggcatg agacaggcat
gggggaaaac
843661 cggtgggaaa gtgggctggg aaggggagga tatggtgtca gcagatttaa
gcaacaaaga
843721 tcagatgagc taattggagt cgaaaccagc cgcatgtgca tgccgctaca
gctactgaca
843781 ccaagcttcg gctatggggg cttgtgtgaa tgcagacaca cacctggtga
acacagatac
843841 ctgacacaca ctaatatgcg ccaccctcac cccccccat tactcaaaca
tgcacgtgag
843901 ggcaaaaaca gccaggtggg agtgctgttc agacaggcca gtgttcccac
agagtgaagt
843961 gggacatctc tgagaaagtg tggaatagac gccccaagcc ttcccattta
gtacacgtaa
844021 agacaaaaca ataaacaata aactaataaa acaccagttc actgtgtggc
tgcagcccta
844081 actttaaccc ttccacgtta ggtcagtaag attaagtaag agattcttct
atcagcatct

844141 gtccatcagc ctgcaattct ttacgaggtt atgggaaaac gcaacttaaa gtaatgataa
844201 cattccttct ttgtacgcgt agacacggtg ggaaggtggt ggagacagag gtgggggtgg
844261 agggaccatt aggcaatcct gttatgaggg caaaggtgtt taatcagatc ttattttccc
844321 ttctgtctct gcaggccacc cccatggacg acactgatag gtaagcctgg atgattacta
844381 aaaaaaaaaa agaccaaaaa attggatttg tttattccac atattgatga cgagcttcct
844441 gtgcttacgt aaatgtctga ctgtttttaa caggagtgct cacaagagac tgtatccgga
844501 cagagaacag agcagctctc cacccagcaa acaggcccgc agagacgaca cccaaagagg
844561 tggattaaaa aacataaaag agcaaatgat cgcctctttt tttctatgct tctgttgttc
844621 tcctatgcta aataagttgt ttcttgtcct cattcagttc tcctgtacgt gagatcatgc
844681 tctgaagagg tttttgatgc actcatgctc agcgcgccga cactttcaag cctccgtgaa
844741 gctgtaagtt aacgttcaga gattcaagta tcggcacgag tatcgttttc ccttgaagcc
844801 gaggttaact cgtgtgtcat tcctctcctc ccaaaggttt cagaaaaata cggaatgcac
844861 aaagacacaa ttgggaaaat ctacaagaga tgcaaaagag ggtgagttgt gttttcggcc
844921 tgcctgcaga atcactcgta cagagctgca ccatctgaaa actgggttaa atctgcaaag
844981 tctaccacca gataacacgt taactgtaaa tttagggctt agtttgtggc tttaatctcg
845041 tactcctttg gggaagctaa tgttgactga ctgggttggt aggttgagga atgcgttaat
845101 cgtagctcct ccatgacagg tgactgaagc aggatggaaa gatttcaaaa gtgctgccga
845161 tgcgtcacag acgtcttttt gttcaggttt ttgttttcat tgagacatga tagagatata
845221 tcttgttttg tcctttcaag ccaaccaaaa cttttccatg ggctaaaagt gcaggtgtca
845281 catgagattg ttccattttc gagtgctgat ataaacgatc gtctccttgt ccttgctgat
845341 tcataaaaga cctgcttgta aagtgatcat tatcgcagcc tgtattaatt aaaatatact
845401 gcctctactg aatgtcacgc agtaggtact gtcacattga tgatggcctg gatcagaact
845461 tggaatagaa ataacagagg caatccaagg gtagatgtta aggagaactt tgtcagggac
845521 tgtagcacct ttgggctttt tttttttcc tccactgctg ctcgctggca gccagggctt
845581 caggggggtgt agctgagcta acaggttgag ttacattctc ccagcagttc cctcctctgt
845641 agcttttgtc ctctgtcagc tttgtcactg cagacatgta cggtggttgg ttaggtcatt
845701 aaatacatga atgcctggag attagtgacc ttatttgctt ccccggctaa gagatacagt

845761 cgcagattag gaacctggca cgctgctgcg tttcttatcc tcgagtgaaa atgacaaata
845821 cacagtggaa cttcgcttga cttgatgcca cattctccat cttcatgatt ttttgtttct
845881 ttttttaaaa tcaggattct cgtcaacatg gacgacaaca tcattgaaca ctacaccaac
845941 cagtcggcct tcctaataga gatctctgag cccgtcggcg gccagttcca ggtcactctg
846001 gctgaagtat gagagtctcg tccgtctccg gctgccagtt tgaagcatca tctaagagca
846061 ctgcccagtc gctacagcag caactgtaga tggttaaaac caaagagaac tgaagggagt
846121 ctttggctca aaaaagttgg cttctcaatt ctgtaaatac cactatcact ggttctgaat
846181 ttatttgaat ttaatattgt aacatacata tagagcaatt atagcctgta catacataat
846241 gtaaatagat tttaataaca cagtgtttgt gaagtcgaca tgttataacc tggttctccc
846301 cataaaaagc tctgtatttc aagtttgttt ttattttatt ttatttttaa ctttacagag
846361 ctctgtgcac attgtgtgca ttatttccag aggttgctac tcttgtaact ttcagcgtat
846421 tcgtcatttc ttctgcaagg ctcgtttgca ttaatccatc cacaacctgc aaacattcgt
846481 tttgttttca aactcctttt gcgtttcata cgagtcggtt cctgcttgtt tcagaagtaa
846541 aacagcccaa aaatgggtta aattcacaac caaagtcaga catttccacc gtttaaaccg
846601 caacaggagt gagtattggc catcatttag ccctatctgg acatttcctg actacatggt
846661 gtaaccacag agttttcctg gaaacatcac caaatcccga ggcccgccag aggtttttca
846721 tagtataaat gtgtgactgt atgtatgtgt gtaggatttt cttctcattt caaaacaacc
846781 ctgtaatatt tcatgaatat gctatgacaa gtctggctgt attaacatgt cttccctttt
846841 tgtaagaaaa taaagaaatt gtattggaga ttgtggggtt tttacttatt tctgccaatc
846901 attcacatct gttagcaggt gtgcatcagg ggacaaaaac aatagtggat aaattagact
846961 tttgaatgaa agtgcctgtg gaaaatgcag acatcccaca cattctgcgt ctcttatatg
847021 cggtaagcgt acatttttaa taaaagaagc ggagaaaaaa aagccaaaac gcacttcagc
847081 atcaaaaata gagcgcgcac acacccgtgc acgcagcttg atgcatttcc ctatttggtg
847141 acaaactcag gggtaaaaa aggcagggag gcactctgtc ctcgacaaaa cacaggcatc
847201 gtccaatacg tcaaataaag aggcacgtag ggagatgtgg cggcaaggct agcaggcgga
847261 gaaagggaca gacaaaagga cagagacaaa aagggaggca gagagatctg ttaaggaggg
847321 actgatgagg gggagaggaa taatgaagag gtggctgcac catttgcagc tactgagaga

847381 aacacgctgt cgccattcag atgggacgcg agagccagcc attggtaagc accagtcccc
847441 cgagtgctga ttacatggac tctttgtgtg gggggggtgc gtgtgtgtat gtttgtagtt
847501 gatggattca ttaatgacct agaatgattc acagtgcgtc cagccgccac cgggctcatt
847561 ctccaccggg cacgttctct tcttccacac cttcacagct gaaagaatct gctcgttgtt
847621 gtcgccgtca gtgaaacatc gtgcaaatgc aataagatgc tttcaaaaag cgtgacttcg
847681 gcatgcaggt gactgcagag aggtctctgt tctccatcac gtcagtcaca tcaaaacatt
847741 tcagagcaga agaggaacag atggatgctg cgagtggagc cggtgtgata acacacatgt
847801 gagacagaat gcaatagacg agagacatga gagttgtggt ggtgaagcaa acaggcaaat
847861 atcagtgttc cagctaaaac tgtttaatcc atttcttcat tttttacttt tttgaagctt
847921 ttgtttgact ttattgccat atgcagtatg ttttatcaaa gactgttgtg tgaccaagaa
847981 aacacccctt gaaacaatat tgtttataca tccttgggtt ttcttgtttc catccctgag
848041 ggcttttttt cctcgtcacc ctcctcctga aacacaggag gctgctagtt ggacttttaa
848101 cattcagaac ggagactgtg gttcagacgc agcccttacg taattactac cccaacagaa
848161 tctgttcaag gtcacagttg actattgatt gtcaggcgtc ggcacaataa aacatcagcc
848221 agactacacc taattagcct acaccagagt ctggtgcatg agcctaactg ggtctcatga
848281 tgggtgaaca cacacatgcg ctgttgtggg gagactggcc aagttttggt ttcctagcaa
848341 cagagtaagg cactgactgt tcagtctttt catgtctagc gcacgagctg tgaagtcaga
848401 cgtcctcctt ctcatcgctg ctcctcatct tgggccgctc catcactccg acgccaacag
848461 aatctccccc ttttctgagc cagtcggact ggagatgctt aataaagagg taaggaataa
848521 actgcacagg aaagggagtg tttatgtctt atgtggtcca gatctttgcc cattaatttc
848581 ttatttttac agaaaaccaa ctgactgccc cccaagaaaa ggcatcaggc ttgtacccct
848641 tatgatgcac acccccgcct ccatggtgat ggggattgtc tttgcccctc tgggactggt
848701 ccttgttttc actgccgcca tcactcctca gtggagagag ggacgggcac gcctgggcac
848761 ggcagccccg ggtttgcttc ttcgatcggg agtgaaaagt cacgggacgg gggttcgtcc
848821 tgggtcggcg gaggccctgc tgttgcgccg ctctgacgga ctttgggaga gctgcctgca
848881 ggtggagcac ttggagctga agcagtgctg gcctgtggcg ggtccgtatc agaaagattc
848941 gagagttcgt ctagcacaag gcttagtcct cacttctttg ttcctctgcg gcaccggtat

849001 tgtacttgca tgcattggag tccgctgctg gacggatata cccctgagag gggtggcagc
849061 tacaggtggt ctcctggtgg tgatgtcagg gttgctcagc ttgacagcac tcggggtgta
849121 cacccacaac cttgataaac tgggggtggc ggcttcgaat cagggcatca ataaccccag
849181 attcccccat ctcagcctcc acgcagccgg atcgctctac tttggctggc tgggctcgtg
849241 cttacagatg ctgggaggag gcgctcttct cttcagtttc aaacagccac gatgctcaat
849301 ctgcccctcc tgcccaggcc agcacgccca ccctgcctgt cgttcatgtc cagagtttac
849361 caaaaagtcc gacacggatg aatacgaggt cagctgttga aagtcagatc catgaggtga
849421 attccaactc aagaaatgat gctgactcac aggaaaacac ctgatttagg ggcttttaca
849481 ttatccagat gagggatgta ctttttatca ttaaagggta gagcagaggc ccagattagt
849541 tcagaagaag gaacacttgc tgcaacgtgt ggtgaaaccg tccttttgtg ttgcctttgt
849601 agccatgcct ttgtactgtt attttatatc ctggcaaaaa acagaattgt tacccatgag
849661 gccggtcgag acatatatta aaacagtgta tttttgagtg aatgagaacg tatacgtttc
849721 cgagcgtgtc cgggtgaatc gtggtacgac ttaacttggc gatgtacagc gggggtcttg
849781 tttctcattt atttactgtt tatgtgtgtt gacacttgat ggtatattga ataaaataaa
849841 gtaaacacca ctcttgttta agtgctgcat tcaggtcgta taggagaaac gggcaccggt
849901 gtagagctgt aaataggctc tgctgcgtaa tacataaaag atttaaataa gaactaaatt
849961 ttaggcaaat cttctgccta atacttcaaa agtataccct tgttacggta ttgagaatgg
850021 ctctgtattt acatgtacat atattacaac agactttaat taatctaaga aaagtttgat
850081 atttcatcca caattcagac taaaaatcaa tataaatata cattgatgtc acagtgacac
850141 ttagcaaatt gttgtttgca tcttttccgt gttgtaaacc agttaaatgt ctttgtttca
850201 agggctaaat ttgcgataca cagtgtgatt aaagtgcagt caaggaaaat cgatcaaata
850261 ttttctagct aacactatca agaacaattt cccatttgca tgattgtaaa accacagttg
850321 ctcagataaa gaaataattt tcggtcgagt ttccgctcac ggccatgtca ctcagctcct
850381 tgtttatgga ctcctgcagc gaccctagtg acagttccag catgggactg gtctctaaac
850441 ctggagaacc catttccaga agaacgtcag acggttctgc atccactaac tgctccccgt
850501 cgctgaggct ccacctgtag gcgccttctg tccctgcctc gcccggttct gagggcggct
850561 cctctgtggg cgagctctct gctgttgggt ctccatcttt ggaggctttg agtgaggtcg

850621 ttcgttcaat ttcttgaacc agttcctcat tttgttcttt ccactgacga aactttgatg
850681 ccgtcacttc tgaatcgtct aagatcttgt tgatctgcag cagctccgcc tctttcgcct
850741 gaagagaaat ggcagttggg gggggggggt tattgaccga acgcgctttg ccaaacatgg
850801 atgttcgatt ggctgtatga atattcaatg gctgtttaat caagtcattt ccttctactg
850861 tgtcacagga ggcctgacat tttctctcaa ggaacatcac gtaattatcg ggctggtact
850921 tctctgcaaa tcaggggtta atggttttgt cacaccgtaa gcagcacatt aaatctacag
850981 tgagacattg cttcaataag gaccagcagc aatgcttgtt taaaaataag acagcataaa
851041 ccatttttat ttgatgtaga gggaatattg gagttggaca attatcttca ggtatccgct
851101 acagaccctg tggcacagtg gttgaccttt gacctgctct gtttgtgctc accttcaggg
851161 tgctctgcag ggtccggaga gtgtgaacct tctcattgat gaccgggttc ttgcagcgtc
851221 gaatgaaaga tctcctgaag tggtcatgag tgaacggttg ctcctgtccc gtcagagaca
851281 cgcctccttc ctttatgctg ttcatcatca cacccatctc atccactggt tgataaagat
851341 ggcgcggaca gacgagaacg ccgttaatga ggcaaacatc acgtaacagc accaagcaca
851401 aacaaaacat tgggctgatg tcacacatgt gtttatccat gtgatcacaa atgagtgtca
851461 tgggggaaaa ttaagaaaca aatttccaat tttggatgaa ccacatgtca gaaggaccat
851521 gagtaaagaa atatgtcttg tgttgaatga aacagcagaa atagaactgg taaagtttta
851581 cctgttcctt tgctgctccc tgtcagtgga cttggtaacg gcgccctgtt cttccacttt
851641 tgccgaggta aagtgttgga ctgaactttc tgagagacac aattgcacac gtaataatct
851701 tgatttactt ccaacgcttc caaatcttaa cagatttctc aatgtgtgaa ctttgcagtg
851761 accttttttc ggcggggtga gggtgatcgc tcgctttcag attcagtctc gctgtaacat
851821 tctgtcagat ggaaaggaat acactggata tacaaataca ctgcaaatgc atgatctgta
851881 agaatgtttt tgatgaaccc attttgtaga atgaatatga tcatttatag taaatgaaga
851941 cagttttatt aacaaggtaa tggagagcaa atgattgacg taccttcttc actatcgggc
852001 cctttctgct tcttgtgttt ttgaatcgtt gtaatcaaat aattaatcca cctaaagaag
852061 ttacaaacca aaaagtatca attatgtgtc tgcaatggag aacgttaaac tgcacacaaa
852121 ccggaccgca aatctaccgg cagggggcag tgttgtatag agaagaacga tcacattgca
852181 tttatgtccc tgagcctccg gatggacttt agaacttgac gtttttctgc actaacgttt

852241 ttgctaacag atagaaggga ttggaccgaa ctcacttgct catgtcgttg acattgtcag
852301 cagcaaaaaa gaagttctga aaccgctggt ggcacatttt aaaaacactg gtgagagaga
852361 aaaacagacc aacatgagtt gaaaacaaat gacaagagaa gaagcataat aaaacactgc
852421 aaattattta ttttactctg cagcttgatt cttattcata agatttttcc aaagagcaaa
852481 cccaattagg ttgagtaatc caccattcat ggagtaataa agcttctctt tacaaaacaa
852541 cagctacacc actaggtttt tatgtattta gtcattagtt taaattacag acgactgtag
852601 aagcgaacca caaaagaccc ttcttactat ttcctcttgt gttctccagc actctctatg
852661 ctgtagctgg atatattgac aaaaccttct gccttctcat cctgcaaaga aaagttgatt
852721 aggcaaactc ggtgaatcat ggtcattaat ttgggggcgt cagagacact gacagtccca
852781 cctgttggct ggagtaccaa tacagagccg gccccttcag gacaaaccag aaacgctgcc
852841 acttctgggc gaggaagacg ctgctttctt ttctcttctt ccacagccag ccgtcacagt
852901 cgggcttgcc cagctcgcgg cacgacaccc ggcgtcggct catggccgtt gtctgtcctg
852961 caaagcccag aatgtgtgaa taggaaagca gtgatgattt tatagaggag tccaaaacaa
853021 atgtgctgtg gcttagtgct tcaaccaatc cttaatttat gaagcacgca gcttctggag
853081 cgctttggag gtgaactatc ttcctcacca atgctttaaa taattataca ggggacgggg
853141 aattgaacca cctgaaactg cttggaggga ctttggtaaa cctgatcatt tttcccacag
853201 ctgctacact cagccttgca caggctactc aatgacataa ataactgggt gcaataataa
853261 caaaagcaac ttcatatcag cagcgtggtc ggcctgtggt gtttgagact aaagccctaa
853321 aaacaaagaa gtcacacaca aagcaaacct ttcaaagatg tcttctctct gtctttcttc
853381 ccctgaaaac agaaagaaaa gcatctttgt ttcaagtttg tggtcagaac atcagctcat
853441 gagttaaaat atttcacttc tcttcttctc tctaccatgt gccccaccat ctcggggcag
853501 gaacttatgc tcgcccggtc ctggttgagt tcctgcagag aaggacttcg gagtggccgg
853561 aagggagacg gacttctggg cgacggcgac ggggaccggc cttctcctgg tctcggagag
853621 attcttaggc tgtcaaacac gcctgaggga atgaagtcca aatcaattgt cgcaccatgt
853681 tcaggacgtt taagaggaaa gcagctttgg gttctctcac ctgaggctga tagaggtgac
853741 acccccccct ggtggtcctg gtatgacgta agagttagac tttcctctgc atcgtagtca
853801 gaggccaact ctgactcctc ctgtcccata ggctgctgct gtgcctctgg cccctggatc

853861 gctctcctgt ggtgcagagc acaaacagac ataaatgagc acaaactgaa ataaatgagc
853921 tcctgaaact gactatttga aaggaaatga ccgataaaaa gatgaaatag gacttttata
853981 ccaggattct acacagactt aattcagcct cccacctact cctaccattt gttttactcc
854041 cacaatgcaa cgccattact gttctgcttc tttcgttgct gtgttctttt ttcattgtct
854101 ttttcatctc caacaaccca ttcatctcct cctcgtggcc ctcctgttct ctcatttcca
854161 gtcagtcgtg agttgcggct gcccatcgtg gaatttgagg cattcgcaat gtgacctcat
854221 ccaaaacttg gcggtggatt aatgaggtca gcggggttct tgggaggttc tgctctaaag
854281 ccttgacagt ttattggatc agagtcgagt ccagttccct gtgataaatg tttttacgct
854341 tcctgagcaa cagcagccca atgtttgctg atgcatggac tcaaaaatca gagtttattt
854401 gagtgatggt gggacaagtg gacactcatt tgcatagatc cctggtacct gcagtcagcg
854461 cctaaccaca aatgcaaatt tacaactcat aatgttgtgt atttgtgtat tgagttcctc
854521 tcagctgctg ccttccttta agtttagtcc tcccctcctt gacctgtctc atcacccccc
854581 ccccctttc ccaaaaannn nnnnnnnnnn nnnnnnnnnn nnnnnnnnnn nnnnnnnnnn
854641 nnnnnnnccc cccccccccc ccccccacac accgacctgg atgccaatgg ctcgcccctt
854701 tcccaatgta aataatgtgt agttcttgtt tccatggttc cgttgccaga ttgttgtctt
854761 attttagatg attcagcact cctttcttgt gtttgacctt acttttagtg aggttttgtc
854821 acagtctgca catttttata actcttacct aaaagacagc gacctgacag aagccgcgac
854881 cctctcaaat aaggagtgcc tcagattctc ttcatccttt tcctcttctt cctctgaact
854941 ctctgccttc ctctgcacca gaaacattag acatcaataa aggcacgttt ggagcgcaca
855001 aaaggatgag caaacatatc acaaggactg caatgaacgc cttccgtgtt ttcttgggtt
855061 taaggagatt agacaagaca aaagaaacaa caaaaaacat agtatataca cacatttaat
855121 ggagtgatgc atggctgaca cagtttagaa atgtttgaag ttttagactc attttggcaa
855181 ttctgggaaa aagcgtgtaa aagcgatcag ccaccagagg gcgctctcgg ttctccagct
855241 ttatttttgg ggagccctct gtcctccaga ttcttacacc atccctgaag attttcctta
855301 ttaaattttc ttattctgtg caaacttgta tagtgctgac attgatggaa taaatgcttg
855361 tgtgtgcgcg cgtgctcccc tgcagtttgt aaccacacag ttcaaaagga aaacagctat
855421 caccatagcg acatatactg tcagcatgca gtgtttgtgg agacagatcc caggtgatat

855481 taaatagaaa gcaggtgtag atactttaga cataaacctt atcggctgcc cagtttctat
855541 tattgttttc tgtctggaca ctggatacca ctgtttgggt tttcctttaa atcagcctga
855601 taatattagc tacaaaactt aaaaaccaat ccttaccaac aggttaaatc taatttaaag
855661 tgcctgacct gtgtggatga aacctggagt ggatgcgtac gtcggaccga ctctgggatc
855721 ttcttcagga ccagagttac tccgctggga ttctcctgca gcttctttat aaggtttgct
855781 ctgctccacc ccacctgatg aagaaagaaa gcgataccgg gatctgatct gaaggttttc
855841 caacactgta aaatgtaaaa gcagcacgaa catgagcagc cattctcacc acaatctggt
855901 cgttgacctg aatcacttca tcgccagcca aaatcttcag gtagacatcg gaggatggct
855961 gcgaaggtaa acaggacgca aaaattgtac ttttcccatg tgaattacat tgattgagca
856021 gctttagcgt ttacctagag caggttaatt atacatctgt tacggtgaca tttaggattg
856081 ggtgaaaaaa aaaaaaatgt gaacaactta attattgaat tacttattcc catgattaaa
856141 ggcaatcatg agttacacat tttttacagg attcaggaat atgcataatt aaaacgaccc
856201 agtaatttaa tgcatgaatg atgatgaaca cgtataatgg ttatagagcg ttgatgggtt
856261 gacattgatt tcccataaag ctcatttaaa cgacacaatt gggcccctcg gcatttagtg
856321 aagtcgtctg gtttttcttt ggaagttgct gttcagagga aaatgtggcc cggagcaaat
856381 ggatgtttcc attgtacaac tacaaaactg ttcttttctt catatagcaa cattaatctg
856441 ttcagcacca acaattttca ggacaaccca cagacatgtt gcaatgtgat gaaacaattt
856501 tcccaaacag caattttacg tgaacccgac aaagaaagaa atgtgatttt ggtagatttc
856561 gtgactgcag cttaaaatag cgcccccagt ggatggaatt aaagtggcaa aaagtacacg
856621 ttctagcagt gtttgcaact taacatcatg atgactcccc ataatatctg aggagttcac
856681 tcctcagtga acggggccac accggagggt tatcagagga gtttggatcc tgacccgtaa
856741 cgtctaagat aagctccaca atcatcagaa caatctcagc gatgcctgtg cagtagcaga
856801 ggagtcgggg atgctcctct gttgacatca tggaaaaata acgtctcccc cttacagtcc
856861 tggagggagc tggagcttcc ctccctgctt aacacgcatg atgaacactc gtccaactga
856921 tgaacactcg tccagctgat gaacactcgt ccagctcatt ttacatcctc cagaactgat
856981 tagcacttct taggcccatc atgtcaaaga atactggact ggaccactgc tacttgacgt
857041 tagcctgact tctttagcca gttcaccttc tgcagaatca caaaatgtcc tttttttgcc

857101 aaatctcagt gggactgacc caaaaagccc cagtgagcaa ataaagttga ggaccacagt
857161 cgtgactgat catctgtggg gcttgggttt tgtgaagcag cactaacaaa gaatgtaaca
857221 gggtcggcct ttgaagccga gctaaaggtg gccgcgggag ggtaagtgag cgcgccttac
857281 ctcagcggca gctccagtca cgtagtgatt actggagtca gtcgatgcta tctcaatccc
857341 ctgaaacagg ggggaaaaaa cagagatgtt aaccacgttt ccaggccgac aaagctgcaa
857401 acgcacaatg tgacagcgct gtctcgttat cgtcagcaca gagactggcg cctgtctgaa
857461 catcaatggg tcaggcttca attgcgatca atactggtca aaaaaagctg aacgggcccc
857521 gacacaccaa taaatccagg gggaaaagcc agtaatagct gacagtgtat cttacaaaga
857581 gttgatcgaa ggtcttttta gaattttcaa ttaatgacag gtcttgtgca tgaatgcgct
857641 gataaactgt ggactttcct gcggtggctg caacttgaat gataggataa ttaagaggga
857701 agaggaaatg agggcatcta ttgtgacatt cctctcttta cttcctattt ctttgaatca
857761 ttcagaagaa ttcagggatc cggtcagttt ttctctttac tgcttcgtgt tcagcaaatc
857821 tagaataaac gcttccctgt ataaacatta ttttttggcg aagcactgca cttacactcc
857881 tgcaaccaac cacagttgct ttgtgcatcg ctgcaaagca tcaaatgttg gagaacattc
857941 aaggggagaa gaaaatgtcc acacctttat gaaataactg agaattataa cgaatacacc
858001 aagatgggag ctggagatgc agcctgtttc atagaaatca aatcaaaagg acgcagtgat
858061 gatgcacatg aagagggcca taattgctgg actgaatgta ccttgtgaag aggagagaga
858121 tgagaaagaa ggggtagggg gtggtctgca aataacaaag aaaaaagaga gcgaaaatgg
858181 aggaccttga attagactga agaggaggtg agtggaacaa agcggcaatg gaaacgtccc
858241 agcaaagatg ggggcaggcc gagctgaaaa agatggagga gtaagaaaat aggaggaaat
858301 tggtaaaatg acacaaagga gagagaatga gagaggaaaa gcttcaataa agagtaagta
858361 gcccttaaat aaatgcagag taaatggaaa gtttcatgtt cccacaagaa aaaaagacta
858421 taatccccag atagtccaga ttaggtccaa actgatacgt caaccataga gcgacagaat
858481 aaagagcttc accagcttgt cgccgggcga cacgggcacc aaatcgacgc tctccagctg
858541 agcggtgtgg gtcagaatag cctcggggct ggtggtcaag atgtcgtcgc acgccgccgt
858601 cacctgccga cactgcggaa gaagacaatt acacacgagt gtactggaaa acaagcgcac
858661 gggtgacgga agagagagca ctcaccacgg agatgatgtc cttctccttt tcatagacag

858721 tggagtcctg ctggatgagc gaaagatgac agaatttaaa cagaactggg cgcctgtgac
858781 acagtttaac tttgctattg attattttct tagaaatggt gacgacctca ttgtcctccc
858841 cacatacatc ctatttcctc ttcgcaggaa atggaagaca actatcttcg aatctctact
858901 tgtgcgtcac tccaggccac agacatgtga cgaagactct taaaaataca catttacact
858961 taatttagcg ggtgatgtca aacaaagata ggaagtgctt atcagtgtca cacgcacaat
859021 aaccaacagg ctggagctaa gatgcttcat ctgtgagagg gggagagggt gataaagatc
859081 aaacagactt gagagcagca gatggaacaa ctgaagctca tttaggtggc tggtcagtgt
859141 gcagagtaaa acaaagtgtt aatccctttg aaatgtaaca agaaatcaca gctagtgaaa
859201 ggttctggca tgaggaagca gggaggcgtg tggttggggg cggttggtgt ttgttgtgtg
859261 tgactgtgct gccatcttcc cctctgtgat ggatgtagat ttaaaacaga tctcgtcgca
859321 ttagcccaaa acaaagctta gccaagcaat ggcgctcaac ctggaaaaaa acacttggag
859381 tgatggtggg gctaattata agccagccag ctgatctccc tgacccgact gctaacattg
859441 ccatcgacat gttatgattt cacagcaacg tctaaaaaaa gattttgcca ttaggaagag
859501 tctgtgccac atgatagtag cacaacagat tcgtcagcat gacacagagt catgctgagg
859561 ctttgacgct aatgtcagcg ggcctgttgt gacagcgcta cgatgctaac tgatatcacg
859621 ctaattagca tttgacacca accacgttgt tgataatgct gaagttatta tagcgttgtc
859681 acagtaaaac tgagcggctc tgctaccaag ccatcttttt tgggttcttc accacagcag
859741 cactaccggt tttacatttc ttcaaaactt ctacagaaca ggaaataaga cattctctgt
859801 gtccttcctt gtaaacaagg tcacatcagg acatcttcag gtgcggtgtg ttcaaatact
859861 gaagtaccca aggccttcaa acaattctaa atatggctgt catatgtttt caaagattaa
859921 agccacatta cgactttcac gatcgtgctt ttatatcaag caagaggagt atagaagcca
859981 ataaaagtgt gaaacccatg ctcgaagacg cccatacaga aattctgcgg aaaagatttc
860041 catctacttt gctgaatgag aagaaagctc ttgtgtgctg tgttgtgaag gactggtgtg
860101 tggctcgatc aggcggtgca ctaattctcc tgcagcggcc tgtacccgaa agctgttttc
860161 tcaatagttg tcttggaaac aaaggacacg ctgtgacgtg ggcgtaacgt tctttaaaga
860221 aatgcgaatg gacgtgtgca tactggacaa acgcacactg gtgcagaatc gtacggacat
860281 gtgaacgcac cgtacccctt tctatcgtga tccgcaggtt ctggtgcgac agtctactgt

860341 cctcgtgtca cagcctgttt ataaacctgg actccccagc tagctttcta tttaccccca
860401 ggtcaaattc tggctataaa aagcctggaa gacacatcgg atctctctga cacccgctgt
860461 ccatctgatc tccatgtttt ttcctccaaa tagaatattt cttccgaccc tttgtgccat
860521 cattgtcttt cagcacacaa gcgctctatt atttcactga tctgtcatca gtacatgtct
860581 tatttagctt cagccatttt attttctgtt gcattcttca tgcttcaagg gcttgcattt
860641 gtcacagtag ctgcacttcc ttctaacgca acgattaatc aaggaataaa atgggcaacc
860701 gaacatctat tatgtctaga ttgaacaata atgtctccca gaggcctctg attactctgc
860761 ctcttaaaag tgaacaaagc tcagaacaat cacagcaaca taactgactg cgccgagcaa
860821 ttttttattt ctgcgagtgg atgtgaagtc acacgtttgt gaaaccgctt gttccgcctc
860881 tgggcccggg gctttgtgat gacggtccgg tggagggagg ccggacatat ttctcattct
860941 ctctcatcag tcaggcaggt gtaaaacctt ccgcacccat caaaactgac ctttcggccg
861001 cccccctga tgacatcgag cgcacctgca aaaacagata ctgcgcaggg ttcgatgaaa
861061 gaaaagggtc ccgtctgttt ctggggagag gggtgggggg tcattgagtt gctggcggtg
861121 aatcattcaa ttcaattcaa tgattcacca ggtggactat gagccaatca cagtctggcc
861181 tttttgcctt cacggtcaaa atgatcaaaa cacaagtctt tgaaatacag tacaagcagg
861241 aacggagcga gcggcagcca catcaaaaaa tgatcagcct cagagagagg ctgacaacga
861301 aggccgcgag ggctttatct cgctaatcgc acggtcagcg ccacgctgct gtgaataaaa
861361 acggacctga ttcatttgga agcggtaaaa acatcctgcc gggctcaggt gtgatgccaa
861421 aaatccaaac agtgcatctg ttccctgcag tcaaaaggaa cgaggccaca aggttcaagt
861481 gatttgacag aaaagtttct caccaaaagt gtctcaaact tgctgataca cctcaaataa
861541 tgtcaataat atcaccaaca tattaggatt tttatagcgt ttaaagtgaa ggtaaagtac
861601 agcgctaagc cggcagtgag gtgaggactt tacctgtcgt gctgggcagg tgtccagagc
861661 tgctatattt aaccgcctct cactgagcgt cctctttctc tcacagcttc cccatgacgg
861721 gctctctacc tccctttaat cgtctcttat ctcagacttc tttgtcccca tgaggatgtt
861781 tatctccggc ttcacaacgg tgccacaata aggtgcctgt tgctccccca ctgtctgttc
861841 gtgcactccc ccaggagtca caagccatta aagagtcatg gtacaatccc tcctactcct
861901 ccttctttct cctccctcct ccctcactcc ctccgcccct ccttcaggcg ctacaacata

861961 tccaggcctg tcccttcttc tacctgctaa cgcaatttcc ccccagttca cacttcacct
862021 agtgacaaaa attgcttcat ctttccactt gcagaaagtt tcccgcgcct ccacagcgac
862081 gtagaggtaa agaaacccaa accatcgtct ccggcgaacc caaagcttcc ctgagctcct
862141 ccagctatgc caggaatgta aagataaaga tgttacttaa gctgagcagg gaagcactgt
862201 gtgtgtgtgt gtgtgagtgt gtgtctgtgt ctttacctgt tgtattttaa caggagttaa
862261 aaacaaaccg ctaactgcct aaaaggtcgt agttcctctt tgagagttaa atatgcgtga
862321 cacccaaaat aaaaacactc ctcacatatg cttccacatt acattcaaaa caatggacca
862381 accttgttga caatgtctcc cagctctttg cagtagctga atatggtctt actgatagcg
862441 cccccactca gctggaaaaa ctgatatctg gaaagacaac aatgactcca gttagaaact
862501 gagcgctgtg ttttaacagc atgtcagtgc taaaaagtgc ggtgctactt ttggaggttt
862561 gaccaacccc ccaaaacaat cccacgctat tcttttcaca ctttaaacct gcagcaacac
862621 aagcacccgc ggctattttc gtgaagaatc acttcctgac atcaacacaa ctgcggagaa
862681 aatagaagct ggagtggtgc acatgtaacg gttagataac tccctctccg atcacctgtt
862741 gagcaaagag aagagtccct tggcggaggt gatgagctcc accaccagct gcaggacgcc
862801 ggtcggcagc ttggtggtgc tgccgccgtc gtagccattg atgcgccagc ggctctggat
862861 gcccatctgg agggactggg acacggcacg gagcttttcc gttacactgc gcaggctctc
862921 gccgcctatg ccgtacatct ggggagcaca gacacagaga caggtcaaag gacaagcccc
862981 gaaaccaccg ttactgttgc atagcgacgc catctctgta aatgctccac tttggccggt
863041 ggcctcggaa tcaaaggcag cgagggttat tcaagcagct gaaaaccatg caaaatgaat
863101 gaattagcat ttattatcta ctttccgcgt ctttacatcc tgttctgttg tagcctctga
863161 aatgaacgca ggaatgaggc gcaaacaggc gctgcactaa aatcacctct cacgtcacat
863221 ctcattagca cttccactag tttcctgttt tgatcaggtt ggaaacctaa tccagagcta
863281 cagtcccgat taaaatccag atcagcttgt ccgcgcaaca caacacatgc ttttcgcatc
863341 cagccaccca ttttctgtca gtaattctta agtacataat tcgaagtcgt acgtccttca
863401 tctccttatt tacactctgc tgcctcttta ttatgtacaa ataaaatgtt tcaaaggttt
863461 cgcaaccttg ataggcctca agtgttgcat tacacaggaa agtggtttta atatttaatt
863521 tatagcagct aaataccaca tattacgcac atcgctccct tgtgccgatg gcgacactac

863581 acagaacgtg gcccactcgg cggtggtcaa gaggttctag tcccggcagg cgttgttatg
863641 ctcatcaaac tgcgcgtcaa catgcgatca tgaatatttc atggtcactt gcagaggtgg
863701 tagatgttag ttgcgtaagc aacgtgaact cagacgtgtt cctttcttcc atgtgagtgt
863761 ctgatggttg ttttctatga atgaactcag tgttggccct tcatggtgca ccaaggcagg
863821 cagacacaaa cactgaatac aaatatgcct ctatatttgg ctgggggga gttattttct
863881 tacacaactc gatgctcctg gcttttattt atgcctccgc actaagtgtt aaaccttctc
863941 tgagcgtttc tgtccatatt ggtttctaaa tttgctaccg ttcttctttt aaaaacagga
864001 aaggaaacca acaagcagtc taagcaccct gagctgttag ccttatcctc acagactgtt
864061 gcaccgccga cgctggaacc ctgcctggtt ctgtcctttt caattagaaa caaaagtcat
864121 ccactctgga gcaacacaca gacatatgca gctacttaaa gtaagtgctc taaaactctg
864181 tttcaatgtt ggaacgaaga aaatcttcaa tgcagcggag gaaaaacaga gcagagcatt
864241 caaggagagg cttttcaaaa agacatttgc atgtaaagtg tatcaggctg acgtttttaa
864301 ctgtaaatcg gctgcagcaa aattatatta ctagacatgg ttcaagtgca accaaagcaa
864361 agagtgatcc tgctgtaaaa gtggaggaaa atgaagtgat aaagttgaag tggttgtgtg
864421 ctctggctgg acctgttgtc ctctgcaaga gagcaacaac ccaacaacat tagtgtcggg
864481 gcggaagacg caaacatgac gcgcaggtat aggtacaccg gaggatgcac acgaccgcgc
864541 cacgactcac cagaaagcag agcttctcca cagcttccag tatgagctct tggtgcccga
864601 tcttattgac tcccagattc tccaggtcct tggacgtcag ttgcagcaga tccatgcccg
864661 ttaaatgcca cgcggaggtg gggtactgct ggaggggggc gtccaaacct ggcagatcat
864721 aggctggatt taataacaaa gaaggttaaa cacacacaca cacacacaca catgaacaca
864781 cgtgtacagg tggagacatg cagcgggagt agaggggatc agtggtaaac aaaaattagg
864841 ttacataaat atgcatgtaa cttgaaaggt ctaattgcaa ttaaataaat cagcaaccca
864901 taattctgcc atgtcattaa tatttcagtg ttaatatgaa tgtggaaccc attggtatat
864961 caattaattc actttggata cacggtgata aagtgtcctg atttacaatg cagggcagat
865021 tctattctgt agagtgactg gtaattgcta aactaaatca agttaatacc aaagtgtcta
865081 aatccagata taactaatcc agatatatat cctctataac taagtttggc cttttctcta
865141 tatgctccat atgctgccgc atgaggacat gtctaatgtt cgggccttga acgcaacatg

865201 tcgaaacgga gaaaattctg cctgcatttg cgttgttgcc actaccgctg
caccttactt
865261 aaccactcga ctgttgtggc tcctctcttc aacaacacgg gccacttcct
cattccacct
865321 tcacctttca cttccaagag gatttgtgcg gcgtgtctat ttgtgtcctc
tccagtccga
865381 tcaaaaaggc cccgtgagac gatcaggctg ggtggggaga aaacgcgcag
agcgttcctt
865441 accttggagc cactggctca ctctctcctc gctccacgca gtgataggct
ccatgctcgg
865501 agtcctgcta cagtcgctcc ttcagttccc agccgtccta gccgactttt
acctctccgg
865561 gctggtggag gaagaaaaaa ttgacgctga cttgggatcg gacgatgacg
aatcgttctt
865621 ccacccacag gtacaggtaa gagtctccct gctgtcagcc aatggcgcgg
ggatgtcagg
865681 ctcggccggg agcaaagacc cctccctcac gctctccccc accacctccc
ccaatccctc
865741 acctgcagga gttttgctgg tggtgcttaa taacttcctt cataattccg
tgcgtaatga
865801 attcaaaggc ggcgcaagac gacaagcgca acatgtggag gcagaagggg
tggggtcagg
865861 caacacctga acgcggtagt aactgcagtc tgctgtagtc aacatgtggt
aataacgctg
865921 taatcaccac ggtgatccgt ggtgcgtttg tgcgcatttc ttgaaagtcc
attccttcca
865981 gggaaggaaa tcagcttgtg cctgtgtgcg cataatcctc tttagccctg
aaactctctg
866041 gaaatggctc cgtgtttcac aaacaaactc tcttattgtg tataatacac
aggcatttaa
866101 aattaataat gggctagaca gtacttatca atttaagaat atagcaattg
ggtccaaaag
866161 cgtatcatat ttatatttct tcattagggt ctttgatcac tttattcagg
taaaaagaca
866221 tgaatcaaaa acatgaataa cagcggcaga cgtcatctgc tgatggtgtg
aaatgttgag
866281 ctaatccttg tcaggggttt atagtaaatc gggaggcaga gggcagcagc
tgatagagag
866341 cttagagcca cggaagagct gccctctgat atctggcacc ccctgctgtt
atcctctttg
866401 aaccagcaga aagagggcga aacgccaggt cagggttggc agaaaacacc
agcgtgcgtg
866461 ttgcgtatgc gtgctacctg ctatttttgt caataatcag agaacatcca
tggcaacaca
866521 gcagggatat gtgggtgaac tgtgtagaac tctttctggg cctccatgat
gtgatgattc
866581 cgcgtcaaat cacctaatat aatctgcatc ttgtgtgctg gagtggtcca
accctctgga
866641 ttaatccatc ccaacgaaac gatcggtgac agcagattac tttctgaaat
tctcagcagc
866701 ccagcccaaa tgtcagatac cagagaaaca gcctcaagtc ccaacatgct
gttttgccgc
866761 cgcagatgta ggattatgag gtttcctgcg aaacaaagtg tcaacagggg
gatgaggctg

866821 aatttgacaa gcttgggtgg aaaacacatg tcagctgcca tcctcctgct cctctgctct
866881 ctttaacagt ggctaatctc ggcccagtcc ttcagcattt aatctgtgtg acaaacgata
866941 gcgccactaa tgtctaggat acaagaatcc agattgctcc ttgtctccgt gcggcacaga
867001 ggcgggcttg agctaattca tttgttgcac agtgcagccc caccgaaatt ttcatgcact
867061 gcagacgtgt gctcggatat tatgactgac gctacagtat gtatggaaat tccatgagga
867121 tgcacttaag tggaatcagt gctcagcggc catgctgcct cagcgcttta atcccctgca
867181 ttaaataaac tttatttaaa gcacactcgc ggatctaatc tgctttacgc acaatttgta
867241 catttgccgt agcgtcaggg aagcaaaaac acaccgaggt tatgcaactc atccaacgcc
867301 atgatggcac attgcttctt tggtgagtgt ttattcatag agatctccag ttaaccttct
867361 gtcagacact aaaaatcaat atagagatgt gcaaatgcag gtgtgaacct tcttttcgtt
867421 taaatatcta aatgaataaa agcagggaaa catctaaacg ctggattatt tgaaggtatt
867481 tcaaaatgta taaaacagtg gcagtattta cactatgtgt gtgtgtgcgt gtgtgtgcgt
867541 gtgtgtgtgt gtgtgtgtgt gtgtgcccag cacggcatgg cactatgtat tgcatgattc
867601 attgcttcct cttttgcaca ctcagcatcc ctctcctccc cctgctgcaa agtcaaactg
867661 ccacacagcc gtatcgtcat ttcatttctg ccctgtcctt tgcgcctttt taggtgctgc
867721 acggtgcagt ctcgcctcac tgagtcgctg cccaggatgc tttctgtgta gctcggtgtg
867781 tgtgtgtgtg tgtgtgtgtg tgtgtgtgtg tgtgtgtgtg tgcgtgtgtg cgtgcgtgcg
867841 tgttgggggа gatgtgttag ggtatccctg aggtctctgt cacatgggag acacctctga
867901 gtcatccctg ttgcctgtga cgtcaccgga gggtcctttg atgatgcaga ggtccccctg
867961 ctgttggagg agggtggggg gggcgactta atgtttccag cagcaaaata tcagtattcc
868021 ttcgctttta actttttaaa caagttgttt tcttatgttg acctgtcgta tagaacagga
868081 agaatgtatt actgtctaaa agagctggag gatctcctgc catgagatat tgcctgcatt
868141 acagcctttg atgggcccca ctgtggccca gcacgtacat atttatgagc tcccactgga
868201 ggagccataa accatcagga acaatctgca gcttctgtta aaaaaaatag agaggatgag
868261 agatgaggca gcgcatcggg gggcttccaa tggaagctgc ttatccatca tcaagcagtt
868321 atccggtcat cgtgggctca gggatggtgg cgactttaaa tatcacgagt gagggacgaa
868381 tggcagtgat gtcatctcac cttcctgcca ccctccattg gctcgtctgt atggaaatga

868441 gctgacttct gtctccattg gcttatttgt cctgcatttg aagagacttg tgcaaaaaaa
868501 gggggattat aaatatgtta ctttacactc gaccaaggca cggtctaaac caacatgtca
868561 ccgggtttct actgcagata tgctcccaag cctcgaggac acagagtcca aaagtcacct
868621 gttcacgtgt ccaggcaggg ctccatagct gcgctgtcag tcacacccac gttcagtgtc
868681 ctcaagggct gtctgccggc ccgggctgag gcagtttgac acactccagg tttcattact
868741 gagccaacca aaccagttcc ctgcgaaatc cactcggaat tgaatgaagc atgaagaggg
868801 ccatgaaggc tggaggggaa cattatattg aggactggaa gcagcagcac aagggctgtt
868861 gtctgaatgg cacggtccaa attctaacct tctattcttt acgttgtgtt tattgtgtaa
868921 tgtgttatgt aatattgaaa aaaacaaaga catctagtgc ctgagagggg gctctggtgg
868981 caaggcgctg gtatattccc atgaaattcc acacagaaag gacaaatcac tctagttgat
869041 acctatatat ttggcaacca tatatcttgt ttttcatttt caccggccag tgcgcttgtc
869101 cagcgtgcgc atctcagtcc cagttgcatc tgttgaatgg cagagagcag ctggctcccg
869161 agcagcagcc tccacctctc tgcattacgt agttgcagct cgcctctccg ccggatgatg
869221 gcgcgattgg acctgggcgg aattggcaat ggggaaaaaa aaggggggt ggtcggataa
869281 aattcagctt gagtctgcca ataatagccc gagttggagc gtgctcgtac tacggaaccg
869341 cggtccctgc gcgcatcgca cactgtcgga agtgaggagt gaagaagtcc tgcctgtcca
869401 ccagggaggg aagagcaagc gagcacggag ggaggtctga atccgtcgtc ggttgcaaag
869461 acagcgcgga ctctggtaat accttgaggt ggtattagac cgaggcgcaa ccccattaaa
869521 ggatcacact ggtgtgcacg gcgcttcgtt cctctgcgtg cttgtcaaac ccaaattccg
869581 agtgtccaac gcgcgtacct gggaggacaa ggggggttct tgctgctttc ggtcggacaa
869641 tcactttgag gggaaacaag caaggtggtc agccggcgct gaggtccagt caggggatta
869701 caattgtttg gaaagcttgc ggcgtctgcg tgcgctcctt ctccgtctcc cccaaacgga
869761 gcgctgatcg ttcttctcca gctgcacttc ggacccgaga gaaggaggtt tccaaccagc
869821 ggcacttgac aggagctcca ccaaaaggga aaaaaaggaa tctcaggaga gggaatttga
869881 tccgctgaag gggaaagaga aaatccttcg aaaatgcctc tggcacagat tgcggagcca
869941 tggcccacga tggaactagt gcagctcgaa acggaggtaa tgagacttga tatgtgttga
870001 gcgtgtcttt cttaatcagt ttatcactta ttctgctttg atttttattt ataggcgata

870061 aaatgaccaa cgtgcctctc gtttggcccc gttaagtgtt aacactgaag ggggaaagtg
870121 atctagacgt atacatttca gtcaatttct ttttaaactg caagattgga gagaacaata
870181 acaactaaat atacggccgt gactttaatg gatttgttaa aaaaaaaaga aatcaggaca
870241 atcaatatat atgatgactt ggcatttttc aaggccattt ttgtcccata cttcatattc
870301 ttgaggagca gtaggggcca ttttaaccaa tgttcaatta ctcacactta tttttaaggg
870361 tctcggatag tatttattta ttcagagtac tttaccaagc ttgggcagct cagatttact
870421 gctgaaccag aaaagagaag gcgagaggca tagaactgtg actgatctct gcagatttgt
870481 ctctggtgtc cacccatcca tccactgtct gttgacttat tccttacctg ggtcagaggc
870541 tgctgttgtt ttttaaaccc actgtgcctc aagaagggag cattttaaac attcagatat
870601 tcaaccttac atttttgtga tttttagctg cagggttgca ggggtggggc gtttttttac
870661 tctctgttgt acctcattta gccctaacta agaacagtaa atgtatattt ttaccgcgcc
870721 agtctgatgc tttatgatca atgacaggag atgatggtgg tttgaggcaa ggctgcatgt
870781 ttaatggagg aaaaataagg agatctggtt ctctgatccg gtgcagctga tgagctactt
870841 gcactcggac tttcgcgcat gcaacctgtg gcacgtcagg cttctgccct ccccccttcaa
870901 ttagccctca gcaggtgaat taggctggtg ccgctgtgct tcagaacctt tatcacacgc
870961 tgacctcgag aggctcattc agatcagaaa ccaggctcat ttccctgtct cacagtctgg
871021 gttgcctgtc tctttccagg tacccgaaaa atggaggcgt ttattgaata tcaatctgct
871081 cattaggtgg atagcaaaga caagttaatg agactcggtg cactgaggtg cttattacaa
871141 ccgtattttg agagtattga ttgtgcagat cccattgtcg gggttaatt ggatgggaga
871201 cagaagtgat aattgctaaa acatcatttt ttcttgtccc tcaggctgct atttaccacg
871261 aagcatgtta gagcataaca gatggagctg cacaacctgt gaaatataac tctataacgc
871321 taaaaaaaaa actactatgg cttcaaacag ctacggctat tcagagccaa gatcatcatc
871381 gctaacacta cagagtgcat ggagattttc atgcattaaa catcttattt gtcctcgtgg
871441 gtccgcgtta gctgttagaa cccagcgtct gtaagtgatt tcacagcagg agctgtgcca
871501 tttttcttct caccaaccta ccagccagct gcacactctg gttcctgatc agcctgactg
871561 agcagaaaga gggaaactgg aggccatatc agaaatgttt tggaagatgc acaacagtgg
871621 gaagtctgct ctaccctgtg gacagatggg acgagtggag caactctgct tttttgtta

871681 aattcgggga aatgaaacac accaattctg ctcagatcgc tgcacgctgt aagggtgtaa
871741 tgaaggggaa tcgggttaag gccttttgtc ttgacaaagc gggtgaggag taaatgtgtc
871801 aagagagctg ctcggcaaat atttgtttga ttgatcactt taacgcatcc ctgaatcagg
871861 tctgttataa attaacctct cggtgaatca cctctgataa atggcagaca ggctgtctac
871921 atcctgttcc tggcgggtcg ctgccacctg gagaaccctt gccagcttgg atagtgaaaa
871981 tcctcaaaga atgcggagga attcttccct ctgggaattt atacaccctc atccctcatt
872041 tgctatttct gagaatcatt ccaagctcag atgtttagcg ttctctgctc ctaagaggct
872101 gtgttaaagt cactttttat atccttctaa aattctttct ccacctattt ggctccaaaa
872161 aatgaataat gaggtcatcc taaacatgtt tacacgggga gagtttagga gcaaaacccc
872221 gtgattccag atgctaaaga gtctttaact caccacatac ttgtccctgc agaggggaaa
872281 gtagggttaa cccatggaat gtgtatcgtg tttgagggcc aggcgtggtc aagccagtgt
872341 aaactccacc agccttgtga gtgggctcaa attcagacga gtctgcccgt gcatgaaagt
872401 gtgcgcttgc ccatgggctt cgcccctgct aatctgggcg tgacgtgcgg gaacacctga
872461 ggagggctgg gtgagcgtgt gataacacaa ccctggccca gcgagtgact ctttcgcagc
872521 tcatctcccc ccccgaaaag aagctccgaa acaacttgtt gtccattttc tggcaatgat
872581 ctgctctctg ttcttattga attttgcaat gacaaggaag cagtccggta ggattgcacg
872641 gacaagtgaa tggcgattgt atttcctagt tgcctagtta caaggcagcg ctgcttgttg
872701 ctgttaaagc acagattgat ctatctcaaa cagtgcctat gtagtgaaag cgctgtcgcc
872761 tatatttgca tagcagggtc cttgctgggc cccagcatgg gtccacatgt aaatccaggc
872821 aagtttggcc atatttatgt ggctgtttgt gaatgcttgc atgcctgtgc tctcattgag
872881 gaagcccagg ggagggaaca cgcaattaac tgggccactt ggatcgacca cgcagagcta
872941 acgaggttga aatgtgtcga gctgcatcgc gaggaatgcc atgccgacca cgtgttagct
873001 tttacgatca ttgccaaaca tttccagttt atcgggctac gtccatcctt gtgagttttg
873061 gttttagaat gggggagaaa cagcaccgtt tagataggtt acagtacatt ggatatgctc
873121 agccagctcc tattctttcc ggattaggca ggttgtggtc atatgacctt tactggaaga
873181 gaccagtgaa tagaccagtt ttgacaaagg tgtgtagact gtggagacag aacattaaga
873241 tcttacagga agcaaacact tctaatttcc aacagtttgc ttcctgtaag agcttgtttt

873301 ctcacgtgat tagcatgtgt agcaatgtag catggacggc catcttttgt gtaaggatgg
873361 ggaaatgcta gtgaggagcg ctggaataaa gacatttacc tcaccatcat acactcttgg
873421 agaaagcagc tccacataag atgaccatgc agtggtccac cagagtagtg tgcccactgc
873481 tggtcttcat catgtagctt taaacaccaa caatatggag gcatcagggg ttgattattg
873541 gtctgcaaaa atgcccatct cattatatct attactgaca taaaggtccc caaaaaggga
873601 gcccactgca ttgctagaca gattgctgtt ggcacaaatg cagaatgtaa cagtaggttt
873661 tactcgtcct cagttctttc agttaaaatg ctttttttcat ttgttcgcca catacagcct
873721 gagctgcttg atggttaagg gcagcgcttt ccattgtgct gtcagccctc acagtctaaa
873781 agaacaggaa gaggggaaga gcgcgggcac agaggaagta aacatcctca gggtcgcctc
873841 tctgtagtgg aaaaccaaag caaagcgcgg aaaaagagag gctgcgtcgc tgagattgtt
873901 ctcacactcg tgtggaactc ttggaaggtg ctggctatca taccataggc tgaagttgat
873961 ggtcaaactg tagaatcctc acgataacat tgaaatatgt ggaggtttta caaacgtgca
874021 ggacaaaaat tatattccgg ggagtcaaag tagctttgaa ttatattctc agccctactt
874081 atactctgaa tgatgtcatt gtactggcat gcgtctcttt gaaaaccatg tctagtttac
874141 actctgcttc aaatcgccat tgtttcgcaa ataacaatca ttttcctctt tgggcagact
874201 cgctttagtg gagaaagggc ggtggcaggg ttttgtgtca gcctgagatt attaggttta
874261 ctgaggagca aagtgttcga gaaaatggac cgctgccgcc cacgcggcct taccgatgtt
874321 tgctctatcg gcgtctccca gccgcacgca agcatgagaa atgcactgtt gcacgttctt
874381 actatgtctc tcctgtgctc ctctcttgac agaacgggca gagcaccact gaggatggag
874441 tcacgcctgc tgttaaggta gcgtgcacgg catctccacc acacacacac acacacacac
874501 acacacacac acacacacac acacacacac acacacactt gttcatacac tttaaatgag
874561 accccagcga ttattcatgc caacggcaga aaagcaaaat aacacggaaa ggacaaaatt
874621 attctacact tttagcagtt tccacagcag agacggtgca catgcacgac agtgtgactt
874681 actgttaaat ttaattctct ttcaattgaa tcgaaagccc tcagaggaca ttagccggct
874741 ggagtttaat gtgttgaatg ctgtctgagt gttgctgatg gtcaccctgt ttggccctgt
874801 gtttcacctg catcaaggta aaaggctccg ataattatgc cgcacgcctt cacatgtgct
874861 catagcgcac acagctagca gcacatgcac cattaatcgt cgagtcggct cctttcctgc

874921 ctcattgtcc ggcaccgaaa cccgatcccc ccccgtaagc gattatgctg cagccgtgta
874981 gctgaggaga ctgaatcatt cccccgcatt tagctccttt tttttttttct gtcctcgatc
875041 tcacgggagt ctggggagcg tgtcgtcatt acactcagct gaggacaatg tgagtaagaa
875101 ctgagcgttc tgctgtgaag tcaaggaagt ctttggggtg catagtggtc aaaggcatta
875161 atgtttgatg gggaaagtga atgaggacaa tgtctatcag atttatttat ttttagatgt
875221 gtgcagtttc agaataatgt gagcctaatt taagaaaaac tggtactttc agatgtgctg
875281 tatacatcac catagactcc ttcctccaac gttatctcat atattatcac cagatttgat
875341 atttgatgcc agatagtttg aatcctgaga ttacacataa taatgaagaa aactgctctg
875401 aaactgaaac ccaagataat ccgctcacat caatgcagat attgtctggg tctggtacct
875461 tgctaatcac atttttgcaa tctgactcga ttcaattatc agtctggccg ggttggtgcc
875521 gtgttacctg ttgacctaat tgattgccga gtaatttggt aattagatga cttatgtaat
875581 gctgttaatc aagatgtcct cagacagctt gcgtcgcttc tccccacgcg gctgcaggaa
875641 cgacacgcag acattggctg atacgataaa tattctcctg ccggcggagc gctaggtggc
875701 actcgctgca gatgcagttg ggctccggca ccgttgaaac ctgtcgctgg agcgatcgat
875761 gcggagctgc tgtgtaaatg ctgggaatcc gataatggat gctgttatct actccagcca
875821 tgggagttac ctggctccaa aaacctaata gtgggagggg gggggggtgg tggacatcat
875881 gcttgaggaa gggaaaagag cggctgctcc tgcgaaaata tcacacacgt ttgatgagaa
875941 agcatgtcga cttctttgga ttcaaggagg gtttaatgtg acagaatgga ttcggaatat
876001 taccgggatc aactgtggaa tctgtctggt tcaaacacgc tttgctgaat gatgccagtg
876061 ttggaacttg ccgctgtgct gcctctgatg gtagatcaga agaaggtgtc aaaacaaacc
876121 ggatggcagt tcggtgcaga gctcaattcg tgacagtgtc tcactaacga aagcccaacg
876181 tctttacttc tgtttgcttt gcagttcaac agagattgaa cttgaacttg tgttgttgtt
876241 gtcgttggct tctgatggcc tctggtgagg cgtattgacc gtgttcgtgc gttcacctaa
876301 gaggctgctc gaatgacccc tctgcagacg gggatgata ttaccctgct ccttattccc
876361 tgcccttctg gccaggctga caatcacaca agtgagagaa ctggtccatt tcagccaacg
876421 atttcatcat ccccattgcc cttgcgacct gctagattta ctctaaaggt tgggggcata
876481 tcgacccggt ccagacaaaa gtgctccgag ctttcttgtt ttgtttatga ccatttgtac

876541 actggtgaat ttcccgcagc ctcagcagtg caacacatct tcgcattccc ctcgttttgt
876601 cttcattcat gcaggaaatt acgtttactc ctcagctgct ggacggacat gatgagggca
876661 gcatctctag gttcactcct gcataggagg tgatggggaa actgtggcaa atgagctgtt
876721 ttatagggaa tgggatggcg agctgttcga gctctggagg caggtagccg gagttagtgg
876781 ctaactggga ggacagcagc agcagctgga ggcgttaacg ttgtatggac agctgagggt
876841 tcaggagtta cggttaaata tgtaatctta ttccattgtt tacaaagcgc tgcttgtgac
876901 gcacaagtaa catcaaacta aactagatga tgttgtcttt ctcatccgtt ttttctttat
876961 ttacaggaca ctcgcttaca atgtaagaag ggtattgtta tgatgacgtt actctgttct
877021 ctttaattca agaacgaggt tttcattatg gcagaattgt gcaattcctg cacatatctg
877081 tagatgtttt aaaggtttcc ttgatgtttc cctcacagac tagtcaaacc tagcagggct
877141 gctgcacttc tttattaaac tcatacttgt gtagcattta acatctaaaa aataagagat
877201 aaacacatat ttgtcccaag agcttcacgt tccattgact gtcgttaaga cacatttaag
877261 aaaagcagca aagcttcaca gtaaataatc cccgaacaat tcagattcag atcctttatt
877321 gtcccacacg gggaaatcgc agaaatgagt aaagtggaga gtttccgcgg gtttcatcca
877381 cttcattatc tactaccact cgtggtgccc aagggccaaa agggttaaac atttttacca
877441 gtagggtcag tcacatttct ctgtgttgat tgtggctctt tcctggagtt acagtagctg
877501 cagtgtgcac tgacccgggg gggggggggg ggcggtgagt ggtgggaaca gaaggagcgg
877561 agcaaaaggt tgaggggaac tgtaggagaa cattaggggt ccccgtgtgt cactgctgtc
877621 caatccttgt tgtacttgag acaaagaaga ggcggaataa agtggcactg cagggatatc
877681 tgtggtcatt cgatagttcg tcggcgttca cgcccgctac ggcgttttgt tcgtgttgat
877741 cagagggcag gctggtcgca gccttggtgt tgagtgtttc caggagcgat taaatattta
877801 ccagtgatga gagtgcgtac gccggctcgc aaacgagctt gttgacctct gggctgccga
877861 atcaggaagt ggtatagctg acgcgagtgc aaccttgtgg cccgtgtaac cgcgtgtgca
877921 tacatggtgt tggcatagca actgcatttt gttttgcggt cacgtgctgg aagtcgattt
877981 atgcagaagc acttgcattt aaatacacac acatacactg agctgtcctc atttatcagc
878041 ttatttatgg tttagctcac agtttcacaa aggttacaca aagatgatgt tgatgatgat
878101 gatgatgaag gtgactgcat ctgaacgctt tgtcagctga gctttcttct catttcttga

878161 gcttggagac gatgctttca atgtcactct ggtgtgacct cgacctcagc ttgcaaccgc
878221 tccattaaga cctaatgtgg ggaaggttgt tggcgcattg tttccacatg atttacataa
878281 agacactgga atacagagga ggataaagga acaaatagca ctcaatggac tttgctccac
878341 tttagaaatc tttcagtctt ttgacctgac tgaactctgc ttcaaggcag aataatgtac
878401 ctacctcttt acgtagtgtc tgcagattcc ttttctcttt tctctttgga gactctttga
878461 agatgagatg catctgactg tgcttctttg tccctttggg tttgctaata ctacaatttt
878521 tctgccaaaa catttggaat tgagagttga ttcctgcctg gagctgttcc catcagcaga
878581 gcgatattta cttgtttgca cacctctgtg gacacgagct gcattcgctg tgccgtttgt
878641 aggcactctc tatgtagttg gagttcaggt caaaccagca gctgacgaaa attaactgtg
878701 aatttgacgt tgtttcttat aaaagggctg tcgtccaaca taatcttttg actcgcagct
878761 ctgtgactga ataaaaacta ttaaaagata atgcgtcaca cgggcgtctg ctgggccttg
878821 ccccctcgac agggcctgta ataacaacta tggacgtgcc tcggagcttc actgagctaa
878881 acattgcaca ttccactgca gcttgtaact tcggcagagg gacaggaagt gaagagtgaa
878941 tccagatcca tctgaagaag acttcagctc tctcatttgc cgccgctttt tgtccaaatt
879001 cgatcgatag gtacagtttc cattgagttt atctaaccac aggagatttt agcagaaatc
879061 tcacaaatgg cgtgtgacag atgagatgaa atcattgcca gagactgttg tctgaaagta
879121 aagataaata taagcagcac aacgttacag cagtctcaca ccaccaggct gccacgtgag
879181 tgttttcctt agaaaatgaa aaaaaaatct gcctttgatt gatgccacag gagcagaaat
879241 caaaactgct agcttgcgct gatgccagca cagcggcaca gactctgagg tggctttgac
879301 agcatcagag atggttggtg ttacacaact acatcctgtg acggcgtatg ggagtcatcc
879361 caaatccgca ctcgtcgcag gtcctagatg tcgcagagcg gcgggctgtc ttcaggacgt
879421 ctgcctccaa tctataagaa tgttcattgc tgtcaggctg ctcattcttc ctcaagtcct
879481 ccattttctg gctcaaaaat gactcaggtg gtgagcagag aggcactccg aggttgacat
879541 ttgggtgctc tttgcagtac cctcaaatta tgcctcactg ttccgtcttg gcctcctttg
879601 ctttcctggg caacagtgcc attaatatct cagcacttcc cttaatttcc tgcgtgtgtt
879661 acgctcctag atagatatga cactaattag ctgagggaga tatttaggga tccactttgg
879721 gaagcataca tctgttactc ttaaatattt aatcactgtt tcatcacttc ttttgattaa

879781 acattacgcc tcttttgaaa tcatattttg ttgtgtggaa tatcggatct
cggtgtctga
879841 acatcctgta tttggagtca tcatgaaatc ctttgcacga actgagtcac
gaaggcgtct
879901 cgtatagtgt gcctgctgcg ctccgctttc atcagaacca gtggccatgc
tagcatgtaa
879961 tcacaataaa tgtcaaaaca accctgttta atctgatttc cacacgaatg
ctatctacaa
880021 aatgtactgt aattattctc cagcagttgt ctaatggcag taattagtta
tcctggaatt
880081 agaccagtca gaacaactgt gcatgtcaaa aaaagcccat tttctggcca
gctgtttgtt
880141 tgttggtcac ttcttctcag cattagcggt cggcctgtgt cacctgaggc
cacgggagcc
880201 atgttggtgt caggattggt taatagaagg gaaagtgttc aaaaaaaccc
caccaggtgt
880261 ggagaagaga atagcaataa gtgtgtttag gatagggaac acttctagga
tggcgccgct
880321 actgtttatg cctttgtatt gctgttttgt ttcatgtttg gtttttcagt
ggtttttttt
880381 tttttttttt ttaaggaaat ctccaagtct caccttaaca taatgttacc
gagtcagaat
880441 gactggacat tttctgcaat gacaccctca catcaacgct aatagtatga
ggtcatttgt
880501 aacctcttca gacctgactt aacctgtatg aagcggacag aaaatttaag
gttctttctg
880561 cctgcacaga aacaagatgc tgattttagg gactatttaa ttgattctta
atggcaagaa
880621 ggctacagga gccgacctaa tgtctgcagt tttcatcaaa gaagctccag
gttttgagaa
880681 ggaattctgc ttctttttaa ataagtgtct tttatatggg ggataggttg
acactgctca
880741 ggtgagctca agaaccctgc ttaagtattt actctcattg tttcataccc
atataagtgc
880801 tgaagctaaa agctgcccaa atgctccttt ctttttgtgt ggagccgtgt
gtggcagagc
880861 caccgtcgat ctgaagtgtt tgccactcag gaagttaatt acagtcctcg
ctcacagcat
880921 ctgctacagc agaagacatc aagtagtctt ttatttgatg actggcaggg
tctacaggac
880981 tgtccacctc gttttgaggc tatatttgag ttctggttgc tgtagaagat
catttacaac
881041 aggcccagaa gactcactct ctgtgctttt agataaatat ttgttttcta
tcaaaatgaa
881101 atttccgagt gtctgtttca gtgtcactgt ggctcactgt aggaaacaac
tgaagctgga
881161 agttactgcc catttgatca caccacagag gcgctgcatt gaaaggcctg
cagtggtcca
881221 gcagcgcttc tgacgaggac cacgagagaa ccgcttcccc gtgcacactc
gcacacatgc
881281 ggattctaaa tgaggagaag cagcgaggct tgaagatgcc ctctctcctc
tggttgccca
881341 ccatcatttc accccaacat tccattaaat catcctgttc tcgtcttcag
ctgcttcctt

881401 aatcttccat cactaccttt cctagaaatc taatttatca ttctgtcttg caggcctgat
881461 aaaggccagc tatttttttt cctttaatac catttgttaa actctgttgg ttcgagcgcc
881521 cttgactgtc ctaccacatt tcaccatctt ccaccttttg tactctcaaa gtttctgaag
881581 caaactttca cttttctgcc gtgtaaatca tttaaaaagc tggagttctg tctaaatgtc
881641 acccaaccgg tccagtcagt gagcaagagc ggcagatggg aaattagctt tggtactcaa
881701 gccggctgga ctttgctctg acaaatgtac ggaggtgcac gccccaatca tgagcttctg
881761 gttacagccc aaagaagaaa atgacattat ggtggcttat ctgtacgcgg acacgtgcaa
881821 taaaagcgca taaatgcttg atcgcatgta gtgtgagtca gggggttgtg tttctgcacg
881881 ttaattgagc ctctccagaa gggttttagc acgtatcgca actgttcgtg gtcgccagcc
881941 gaccttgtta caaagcgata acgtctgcag acccaggagg tgtgtttttt ttaagggaga
882001 tggttgtttg agtgaagtgc ctgccaagtg agggtcccct tatctgggtt tatcatcgac
882061 gttaacggcg gcagttggta gtttggaggc ttgggggtga tggctgcctt ccaaacacgc
882121 atgcaccaca cgcagcaagg cggggacata gtttacagcc tatttcccag cctgcctctg
882181 tgtgtaaagg gtgctgtaaa tcttgtgctt ctgccttttt tgtgctgtgt ttacaaatct
882241 gccatctgca gctgattcgc ttctttcctt ttgtaggtta aatcatcagg agtctgacta
882301 gaagaatgta cagagttggt ccatctgtag attcacttca ttcccttcct ccatccattc
882361 atgtctgctg tggtcccttg ttgtcatggt tactgtgccc ttgctagtgt tgctaagctg
882421 tgctaagact gtgacttgtg ctgttgagag cactaaggta tttggtgaag actgtgcacc
882481 tacatggttt tcattaataa tttaaagatt ttttttaaaa ttcaatctgt ttcccaacac
882541 tgtcacacat gagcagggct actccgacaa atcagcgtcg ttaatcatgg cgcgcgcatg
882601 catgcgctct gcctcttatc tctgcattct gaaatgatgt tagtccggct ccatgattta
882661 aaggccacaa tgatgtagta catcattatt acttcctagg agaaatacaa atagacccgt
882721 gcacggtggt gactctggca tgcactcaca tgctgtactg gtgtttcaaa ggttaaaaaa
882781 aataaaaaat aaaaaaaccc tcctattgtt aaaatgaagg ccggagaaat gttacctgga
882841 aacatcatgc aaataatgca tcccgtattc ttgtgcgcgt ccttgccacg ggtgcttgat
882901 tttgtctcat aaaaggatgt tgggagacgg agattaagaa acacagttgc catagtttca
882961 ctctttcctg tctcggtttt gatcaaggag agacgttcaa gcacggcttc attgacatgt

883021 ttgatcaatt cagattcaca tttccacttg gaggtttctc tttttctaca
tttgttggtt
883081 tatttttttc attttcaccc cccccttcc tttttttcct atacagtagg
aacatgtaac
883141 ctttgaccgc tgttgccatg gtagcactag gatgcagtac agggaacgta
aatgcaaatt
883201 acaacatttt ctggaacatt cacaaatgac agataatgct gcagatctgt
tgaaagttta
883261 aaaaatgtgc attttcttag taatttaaat aatatataga tagatatcaa
tatataaacc
883321 cgcgacccga cccggataag cggtagagaa tggatggatg gatggatata
taaaactgtt
883381 tttttctgca gaacagcagg ttgaaatcca tttaagataa ctattcatta
tatacatcag
883441 tgtgatgcta tgttactgat ggtggcaggt gcctcatctt gcccttttta
tgtaaagtat
883501 gcaacctcac cactaggtgt cactagtgat ggtttcgggt caaagcttgg
aatgggattt
883561 tttttttcc cgacctctct ggttacaagt cagaaaagca ggatgggcat
ctgatgtcaa
883621 gcagtgttga aatgggtacg agtgaatatc aacgcatggt gatgctctac
tacggtaacg
883681 gttgtcataa caacataatg agccatgata catccacgct gactaattag
tgacacagaa
883741 atactaagtt agtagttata ttggtttaaa aaagaagaat attatgtggc
tttaccatga
883801 gtccatatgt aaacaacagt cagacgagtc gcgtggtccg actcctggag
gattacaacg
883861 ttgttttgag tcacgaatgt tgtagtttgg atatttatac gaagtgcaaa
gccacaggag
883921 ggagaaagcg ggaagctgac taagacaaag ctgctatttt aaagtctctc
cggctggtgt
883981 aaagagtttg agagtgggag cgattacgtg atgatggaaa aataaacggc
gagagaaatg
884041 atgctccagc gtagcttttg tatgcgtgtt tatgacctga aaacctccac
gtacgctgca
884101 gacctgcact tttccccagc agacggccag cttagcttca cagcgatgac
ctcatgttgt
884161 acattagcgg catctttctt ccctccctct ctcatccatc tctcccactt
attctccttc
884221 cctttagctt cttcctgaca tttgggtgac ccctctgacc gtctccgtca
ctgcagataa
884281 tacacaaccc catcaaggcc tcctgtcaac cggtgcaacg cagagctgcc
tatcatctta
884341 ctactgctgc tattaatatt acagatgcca acatgggatt tcctgtagca
tccacgctaa
884401 tcctgtgcta cgtctaaatc taacctcctg aactattgtc taaagcggtc
gtgtctcttt
884461 acacttcctt gtgtcatttg gtgattttca gcataccaaa ggctgagtta
ttggtcatta
884521 tctgttttta atgcattgaa cgtgcgcctg tgtatcccat tgctgtcaca
gaggagccag
884581 atggcagcgt ctgctaaaga acgggtatta accttggtca gaggtcaaaa
gacatgatgg

884641 tcacctctcg accctgccgg atgacttggc actaatcacg aactgctaaa ctaatccctt
884701 aaagctcatc ccgtcatctt ttcatgggat agaaccaaac gtggggtaat gccgttcagc
884761 agtgtgaggt ttagggttac aatcctttac aaagacttga acgcaccgcg atggcgcagc
884821 cgcgagggcg aacatctgcc gggaaacccc gacacgtctg tgatggaagg taaggtgaag
884881 gggtagaagg agtcaaagga aatgtgctaa atgttggaca gaggtgtgtg gctgatgtgg
884941 agagctaagg actgaagcaa aagtgtgaat gactcagccc tgctcctgta aaagatgagc
885001 agggtgccgg gaagacagaa acaatgagag atgatgaaag acgaagggtc caaaactgct
885061 gccctgtgca cgctgcagtg ctgagagact gatgaatgtc agttgttgga ctctgaagga
885121 tagaacagtc ataactccaa agccttaaca ctttatcacc taacagctta cggtcattta
885181 ggtcaaaggc aaggctaaag tccaacaatg gcccctgaca acgcccatat cctttaacca
885241 gccctgagtc acttttgatc gtgttagaca ggtcacctgt cgttctctct gagatcagac
885301 tgcatttcct attgtggact agctttagta gctggatagt tataatctac attttatgtt
885361 agcaatatta ctgtagatat aactcgatgg ctcggcaacc aggaatattg tcttaactgc
885421 agaacactgg atgagtttgt tttcctgggc gagtttcgaa actgtaaaag tttttctttt
885481 taaatgcccg cagaactgag agcacaattg tgccttcaaa agaaaggaag catttagctc
885541 actggctttg cttttatcac catgcaatac ctgtttatta ttctcttatt cccaggtgtg
885601 tgtgtgtgtg tgtgtgtgtg tgtgtgtgtg tgtgtgtgtg tgtgtgtgtg tgtgtgtgtg
885661 tgtgtgtgtg tgtgtgtgtg tgtgtgtgtg tgtgtgtgtg tgcacactca tgcaaatgca
885721 gataaactct gttacagtaa taagactcca ccatgcttgg ttagatgatg gagctgctgt
885781 ttctctctgt acatcctact tatttatgtc atcttccact gcaccccccc tcctaccgtc
885841 tctctctctg tctctctgtc tctctctgtc tctctctgtc tctctctgtc tctctgtctc
885901 tctctctgtc tctctctctc tgtctctgtc tctctctctc tgtctctgtc tctgtctctc
885961 tctctctgtc tctctctgtc tctctgtctg tctctctctg tctctctgtc tctctctgtc
886021 tctctgtctc tctctctgtc tctctctctc tctctctctc tctctctctc tctctctctc
886081 tctctctctc tctcactgtc ttactggagc cattttccag tgaaacaggt ggacacagac
886141 tgaaatcctt ctttcattca tgccatttta agagctctct ctctttctcc tcccttttta
886201 gtacatccat tcattttatg gctccttgtt ctaaaccttc tgcctttacc cacagctttc

886261 tgtggcagct tctgctctgc tttgctgtaa ttggtcacag aaccaacaca aaggtttaca
886321 gtagagccgc gctgacaccg ggaacatgtc cttacttggc cacagcttct atcaatcatg
886381 tacactggtc tgtctgcaca cctttactgg ccccgtgtgg ttaccactga gcattacatg
886441 gactgctttt cacctgttga cttctcagac cttcggaacc gagttatata tttgcacctg
886501 atcaatcaga tcgcattttg cacaatctga taacttgtca gaaatggact aatgattgga
886561 tatttacgtt cctttgaaca cgccagctga tggatgagcc agagcaccac agctcacatg
886621 tattccctgt gttctctaaa cattaatcag ataattaatg tgagttggtt ctctaaggtt
886681 tgaaaggtat atgctctctt attaaataga gatggatggt ttttagctgt cttcccatat
886741 tactagcgct ctggatgaat tccatttact agatggcagg tattgaatat gtgacagaag
886801 cagatttatg ttcggctgca cttaaaaacc tgccggactg actgtcgcag ggggacggag
886861 cctcgtatat ttccagcctc attaatttag atatactgga actccagcca gcccgtcgtg
886921 atgaaattgc ctttgggccg cccctattca catgcgcttg tgcccacctt cctccgatgt
886981 gtctcgttcc agtttccaga tgtgaatccc ccccctccat ccccccaacc gggtcgtttt
887041 gaactctcca gaaaactcac aaagagcagc ttcctggtgc gacacagccg cgtccgtctt
887101 ttcgaaacca ccctttactt cccatttgag tgcgcgcgct gtagtttccc caccgtgcgc
887161 tcgctcagac tcggacgctg tctttctgtg gcaccgtgag tcacctgttg gcacaggtca
887221 catggaagca tcggggcaaa gagatgtgga ggttctattt cgcaccaaaa cccagacagg
887281 agaggcagtg gacgctctgt ttcgtgcgcg tgtgtgtcga taaccgtgtg tgtggccctg
887341 atgcttcagg aggctgctgt ggtcttttgg ggaggagaca aggggctttt ctttgtgtgg
887401 gcggatgcga gaccctgatc ctagattgtg gaggatgtgt tttaacttcc atatccttcc
887461 gtcctgtgac acacagcatg tggttacttt tcaggactta aaagaaagtt gcagttctag
887521 atttcatttc tacattagcc gccagcctat gaatcagtaa ctcagttcag tgatttcagt
887581 ttagaggctg gagtaaagat ggtgctgttt tgggtttgtt tttttaaatt tttgacagtt
887641 tatattttta gatgggggtt aaagctcccc ctgacatatg tttgattgct aaaagcgaca
887701 aggaagtgag ctcagcatga gagaaaaaag gaagtaagaa gtaggaggaa agagaggttt
887761 cctgtcaccc gtgtggcaac cataaccacc ttgagttttt tattttcttt ttcatccccc
887821 cctcccaccc ctgtgagagt gtgtgcttct gtccagagga aagtaaatgc aaatatcagc

887881 ccctgtgtct gtaaatgaat tgagactcca tgcccccccc atcctcacag
cggcacgcac
887941 agatcacctg gatagagcgg gacttttcca gtattagagc aaaggagagc
tccggccgct
888001 ctactcttca tcaggtcagt ccaccccccc cccccctcc ttcaacggtt
nnnnnnnnnn
888061 nnnnnnnnnn nnnnnnnnnn nnnnnnnnnn nnnnnnnnnn aggggggtc
cccccccccc
888121 cccccccctc ctccaccgct cagccagtgc ttcagcttac gattgctcaa
tcacaccgct
888181 gcctttgtaa cttccagcta aagcggtgtt gcatttctct tctgtctaat
ttgcacgttg
888241 ctcataagga cgtgttaaat ctgcagattg ggagatgaac ttggccagga
atagactgtt
888301 atgggaagga agtcgggaaa atctctgtca tcggctgatg attgtcttaa
tcatctgtca
888361 gaggtctctg ggagacctca tcctccccat ctgcctccct ccttcctctc
actgctcgaa
888421 tcgtgcgtga ggaagcggga gcgaggtgcg gtcctgcatc agaaatgttg
tgacatgtgc
888481 actcgggtgt ttgcttatgt ttgaaaaggg atctgcacgc tagttgggat
agttataggg
888541 tataacaggg gtcaggggtc gagtgagtca tcagaaagtg ttttgaatca
ttgtttcaaa
888601 ctttagattt tgttccgttt cagttctctc tctgtctctc tctctctgtc
tctcgctcgc
888661 tctctgtctc tctctctctc tgccactggt gtgtacgtgt gtgacttgtt
gggtgcagga
888721 ctgagaataa gtgcgtactc ttccttcctg ctctgctcag gggttctggg
ttcagctgca
888781 gtcagtgtct gaggacacgc aggaccgatc agacctgctc agagaggaat
ggagagacgc
888841 caggcgagat aacggaggag aaaatgaaga tgaagagtta ataaaaacat
tatagcagag
888901 gggcccctgc tgaaggcctc tgcagaggga tagataaaga gaacaggccg
agaggttttt
888961 ccatatttac gtgctttgct cagagtcgat actagcgcga gtggacaaat
cggggcagcg
889021 cgacagcgat gaggtgaagt tcgagaggat ttatccgagg catcgcccgg
gtcgccgctc
889081 cgtggttggg atgaagaatc agaagtctgg gcgatgttga gatggagaaa
gagaagaaaa
889141 agtttaacct aagcagcttg ctgagaatct acctgggcaa aaataagaaa
agccttcact
889201 gtcacagctg cagaccgccg ccagcatcag acaccagcac cgaacccgca
ccgccccccg
889261 gcagggactg gacggtaaag ttgtcacaac ttatttacat cggcgtcgcg
ggcgttggcc
889321 tcaggagagg ttgtggtgga ctcgtggacg gcacttgcca agtgaacaag
cccagaatct
889381 gcagctgatt agcgctgttg tggcttgttt tgctggggga aaaaggtttc
ctgttgtcaa
889441 attaaatctg cagtgcaggt ttgtctctgg gcatcgcgct cacctaatga
tgaattagtc

889501 tcttcttttg agtgatcgtt taacgctgac ataggttcct tattttggat gtttgggaca
889561 aagaggagtg taataattac taaaaaaaaa caactctcat tttacgctct tctcagagca
889621 gataagctgt attttactat ttttgccttt tatagtcaaa tcttttgctg atttgtgata
889681 attgacaaat aatgaccgtt taagaacctt ttttattatt ttgggcagca ggataaaaca
889741 tgttgattaa agtgctttga tccatctgaa gacaaaaata aggacaatga aaatagggga
889801 acagttcatc tggaaacggc ctgaagaaaa caacggtgat tattgtgccg gtgctggtga
889861 attaatggag cagagagaag cacttcttgg ttcctgttga tgctcagctg tagcgatctg
889921 ttgcggctgt cacaaaacca gcagcagctt gcgggtagaa cactggagag gcgttcagca
889981 gcaccttctt tagaactgac agtttacggt cattagttcg ctgccatcac ttcctggaac
890041 tgcattcaat cattcttcag cgtgtttccc ctttttctgg tgaagtgtta atgatgctag
890101 cgacgttttc tccggcgtcc tgtacaaacc tgtttccttg ggtttgcgac tctctcacat
890161 ctactgtttt gctctcttta ggaagatgga gacatcaagg agatcaacat tacccacgtg
890221 gtcaaggagg gctcggagaa ggctgatgct tctcagtttg agctcctcaa agttttgggt
890281 caggggtcct ttggcaaagt aagtcgctta tccgagccca gtgggttaaa ctacttccta
890341 cagattaagt ttatgcaaat tctcaccaca cttttgctcc attttactgt aagtaattcc
890401 cagaagactt cagaagagcc ccagttacct aaaaaaaaga accatcattt ctcacaaaag
890461 aactcttcgt agttttcatt tcgaaatgaa gagctttgct gttgccacct acagagaatc
890521 tgcagctctg cagatcaaaa ccaattcagg acggaggaga cggggtcaga gttggtgtca
890581 cctggttaca attaagaaac aaccagatcc gtcatcttga gtcatctcac attgagtcca
890641 gaacattgtt atttgatgag ctgtgaaacc cagaagtcac gtggagaccc tggaacaaac
890701 agtgcacaga agtcacttct tagatgtgtg cgcggggagt tgtgtggagt gtgtgtgtgt
890761 ggagggcggg ggggggaggg gtaggggtgt tccactgtga gaaaatcatc ttgtttttct
890821 gtcgtcttgc aggtgttcct ggtacgaaag gtgacgcctc cagatgccaa ccaactctac
890881 gccatgaagg tcctgaagaa ggcgacgctg aaaggtacag cggcctggcc tgaggcgaca
890941 ggttgtcgcc acgggaacaa aactggcgat ccaacgctgg ggttgatgct aacgtggata
891001 ttccagatga acagatgtcc tgtgtttgtc gccggcgcag agctgaaacc tgctttttca
891061 gttttgtggc gatgttttga aaacgctgga gggtgtgtcc ctttcctgtg tgtgtttgta

891121 agaccaccaa aaagccacag tgccggtgtg tttttggtaa atgtgctgtg attatgcaca
891181 actctgcgtt ttctttgtct ctcttgaagc agctgctctg agattctccc gtgacgctcc
891241 ttttttattt ttacaaactc gcttgtcctc cagtgaggga ccgcgtgagg accaagatgg
891301 agagggacat ccttgccgac gtcaaccatc cgtttgtggt caagctgcac tacggtgagt
891361 cagcctcccg tcccgagcct gggatgaccg atttcccgaa tgccagaaaa tgccagaaaa
891421 tgccagaaca tgcttgctgt tcccgcctta ttgaacgttt gtgtttgcag cgttccagac
891481 cgaagggaag ctctacttga tcctggactt cctcagagga ggggatctct tcaccaggct
891541 ttccaaagag gtgaacacga ttgcgttgct gaaataattc cgtcacgtat gaggcgctac
891601 catcatctcc ttttcccccc tcacaggtga tgtttacaga agaggatgta aagttctatc
891661 tggcagagct ggcattagga ctggaccacc tccacagcct gggcatcata tacagagacc
891721 tcaaacctga gaagtaagga ccgacagaat ccccagactt tatacatatg gataaatgca
891781 tctttttaat aatggatgct ccagtttgga cctggttgct gttaaaaaaa tgcatttgtc
891841 agtgttgcag ccacctctga agtctttagt ttagcgtttt gcaccgcaga ggttcgaagc
891901 ccctcctgaa accgtgagaa ctatcttttc taaacaaaac caaccgcccc cttcttcatt
891961 tccgacccct cgtcactttt gattggcgct cccctttttgg ttggtggcat atcgctgccc
892021 atagttgcgt gaagtctctg gcctccacgc tctcgccgtg gtttcaatgg ttaggagatc
892081 cagcaagact cttccaaaat aacaaaaaaa aagcagttta tgttgtgctg cacataaaca
892141 aaagagatcc ttgtttagcc ttcagcagaa caggatgtgt ggtgcaaggt tttgtgaagc
892201 tcaaaagaat ccatccttta aatatttgaa tttattttat tgttgtttcg tagcattctc
892261 ctcgatgaag agggccacat caaactcacg ggtgagtctc ccacacttga ccttcattcc
892321 tctcagcggt gaaacatctt ccaggatatt gattacacgt ttcagtttct ccctgcctgc
892381 gtcagtgatt gccaccattt atcttcccta accagccttg aatttaatgg ggcatcaggt
892441 gactcacaga agaggcgctg atgatctttt attcataaaa gtgttctgat gaggtctggt
892501 tttgttttcc agattttggc ttgtgtaaag aagccatcga ccacgagaaa aaagcctatt
892561 ccttctgcgg cactgtggag tacatggccc cagaggtggt caacaggcag ggacacacgc
892621 acagcgccga ctggtggtcg tttggagtac taatggtaag caggaggtaa cggtttgttt
892681 atttcagaca cggcttgtgt acgcataaat gatccgtaac aagtgttaat tctatttact

892741 ttgtctgttt cccagtttga gatgctgacc ggctctctgc cctttcaagg
aaaggaccgt
892801 aaagaaacca tgaacctgat tctcaagtgg gtgttgagaa aatgcagtta
gggcgtgctg
892861 cccctgcaca aagtgaccaa aaactctgtc cctccttttc cccctgcagg
gcacgtctgg
892921 gaatgcctca gttcttgagc gccgaggccc agtcactgct cagggctttg
ttcaagagga
892981 acccggccaa caggctcggt atggaccgtt ttcatatcca gcatcagtct
ttgggggggg
893041 ggcagaataa cgctgctaac ctacttttta tcttttttta aatgcaggat
ctggagctga
893101 tggtgccgag gagatcaagc gacatggctt cttctccacc atagattgga
atgtatgcat
893161 gcaacactta aatataggca aaatctttca ttatgtgtta cattatttct
ctatatatat
893221 ttaaaactgc tttattttgt cagaaactct tcaggagaga aatgaagcct
cctttcaggc
893281 cagcagtggc acgtccggat gataccttct actttgattc tgagttcaca
tctcgcaccc
893341 ctaaaggtca ggagagagat acagaggtgt tttcttctgt gaagccatca
aagcattaaa
893401 aaaaaaacaa aaaaaacctc tgccttatcc ccagactccc cgggtgtacc
ccccagcgcc
893461 ggagcccacc agctcttcag aggcttcagc tttatagcat caactctgct
ggaggaggaa
893521 ggttcggagg aacaagccaa gcctcagcca cacccagtgg tgcaggtgag
tccgcgcagg
893581 ggcgcaggtc gggtgcagcc gagcggcagt gcagcagcac tgaaacatct
gtctccacag
893641 caactgcacg ggaagaacct gctgttcagc gacggctacg tgctgaagga
agacatcggc
893701 atgggctcct tctccatctg caaacgctgc atccacaaag ccaccaacac
cgaatacgcc
893761 gtcaaggtat gaacgcaagc ggcacggaag gatgtttgag aatggaaaag
gttccgtttt
893821 tcaaggccgg ggtcacgacc tttccttttg ggctccacag atgatcgata
agaccagcac
893881 ggatccttct gaagagatcg agattctgct tcggtatgga cagcacccga
acatcataac
893941 gctgaaggac gtgagtgtac agaagcgcgg cgtgacttgc gtaactatcg
tgacgtttag
894001 gctaatgcga ccgtttgcgt ctcaggtgta cgacaacggc aagcaggtgt
tcctggtgac
894061 ggagctgatg cgaggagggg agctgctgga tcggatcctc aaacagaagt
tcttttcgga
894121 gagagaggcc agcgctgttc ttcacaccat caccaggacc gtggagtacc
tgcactcgca
894181 gggggtgagg gtcgcccaaa tgtcatcaac aacactcatg catgctaaaa
ataacgagct
894241 cttctcaaga ttaaatgcaa agaaacataa atctaaaaga tgaaaatcaa
aatacctgaa
894301 ttgggttgct ggtacttatt tcccatttga tctatttatt taagaatcat
ctctctccaa

894361 agcgatcgat gcccttatcc cccttatttt ctgacttcca ggtggtgcac agagacctga
894421 agcctagcaa catcctctat gtagacgatt ctggaaatcc ggagtccatc cggatctgtg
894481 acttcggctt cgctaagcag ctgcgtgcta acaacggcct gctgatgacg ccgtgctaca
894541 ctgctaactt tgtggctccg gaggttggcg tcgtgaaatt ccctgaacgt tattcccacc
894601 aaagtcggta tggacagaaa taacggttgc gtttgctgga ttttaggtgc tgaagcgcca
894661 aggctacgac gagggctgcg acatctggag cctgggggtt ctgctctaca ccatgctggc
894721 tgggtgagac ccaaggctgg agctgctctc ttctctcccg cctacgcttt ttgctcactt
894781 tgtcgctcgt ttccttcgtc agctttacac cgttcgccaa cgggcccgag gacaccccgg
894841 acgagatcct gaacaggatc ggcaacggtc acttcagcct gggcgggggc aactgggaaa
894901 ctatgtcaga cgccgccaag gtttgggaaa cgtcatgtgt gtctgtgcca atagaagctg
894961 acttcagacg gctcctcttg tgacatcgtg tgaaattcat ggtgtgttcc cgcaggacct
895021 ggtgtctaag atgctccatg tggaccccca ccaaagactg acggcccggc aggtcctcag
895081 acatccctgg atcgtccata gggacaaact gcccaacagc cagctcccgc accacgatcc
895141 caaactggtt aaggtgacag agctgcacca gcaaatcact tcaaagtaaa actgcaaatt
895201 gcgtaacgaa atgtgatcct gtctctcctc agggtgcgat ggcagccacc tactccgccc
895261 tgaagaactc ccaacccct cccgagctga agcccatcga gagctccttc ctggcccaaa
895321 gacgtgtgag gaagcttcct tccacgtccc tgtagagact cgaaaccacc gacctgctta
895381 ctggagactg acccaaaccg ctccatcgcc cggtcagctt gcacggggac ctcgcgtccg
895441 tctcgcagcc aagaaacact gactgtgacc ccgccccacc cccatcttgt ggcaccagtg
895501 gtcctgctct ccttgtcccg actcgtcagc caaccaacgg tgaaacggcg aaagatggcg
895561 aaggtcgatg ggagggacga gggctgatcc gagggtgcag gtttttgggt gtatcggcac
895621 tgtgtggctg cttgaatgtg tgtgtgagcc ggaactacca caacgccgtt tttgatcgtg
895681 gcgatgaccg tttttctctc cgaatcctcg cagtggttgg tctgtcattc agcttgctgc
895741 acgcccgtct gcctgtccgt gtcgtccatc tttgctgttc tttttgtaga gttgatgagc
895801 tttatatgac caggtgtgcc gccgagcgga gctgtgccat cggctccacc tctgtcccac
895861 tgctgttaag tgtttttgaa cgtttcttta tgagtagtag acatttcagc cataactgcc
895921 tctttggaac aaccagaaca ggtggattcc aggaagtgtt gatgctaatt gatgctaacg

895981 tttcggttcc ggttgacctc ctgctttgtg ttttgtttga cagcgtgcac
gtgggcgtgt
896041 ccgtgtgtgt tctactgtct tttgagacgc tgcaggggtc atatgtgcat
gatccggtga
896101 tgtgatgatg tcacgtggtt ttcttgtttt tcttcctctt tttttcctct
ttgcttggtt
896161 gatctgctgg caaccagatg agacgtctgt tatttttatt actacaaatc
tagtaacata
896221 ggtggctttc tgtgaaagga aacttgccca aggaacaatg tggcatgtta
gaaattctgt
896281 attggctttg tttcttgttt tttttttttc actgttcctt gaataatgtg
tttggaatgc
896341 tgtttcctgt aatggctgct ttcgttgttg atttacggta gttattttta
tttgtttcga
896401 aagaaaatcg tgcttgaaaa taatggaaga ttggctgagc acacgcatca
gtctgtggac
896461 ttcagttgca caggtccccc cctgcttcca taagcaaagg aagggaaaca
tccttcagcc
896521 tggcatattc tgtgctcatt tcatttctgt tctatttgga gatccactga
cgttgatgat
896581 tcgaaaactc atcttttgaa tttaagtcga tagatataaa tgtgcttcag
aggaggcaga
896641 cctcatataa actgcgtgga ggggaagacc tgtgtgactg aaccatcaag
gaaccagctg
896701 tcttacctca aaagggatca aacttatatt ctaattcttg tttcatggca
aaattctccc
896761 ttgttaaaaa attcatggta agatgttgta taataaagaa gatttttata
tcttatgaat
896821 attaaaaaaa cagttgctat gatgacctgc ttcttcgatc tttatgtttg
tcttagacta
896881 atatatatcc tctaatggat aattgcgtct taagaatcta cctgaccagc
atttactggt
896941 tggcctatga acggatgcgt ttttccaaag gtgtccgctc gggggagaca
aagatcaata
897001 tttaatatcg ttgatcataa aacctgtagc tgaccaagag caacaccctc
tatctttttt
897061 aaaaaaaaaa acactgtaat gcagcggtgt atttcttact tatatcacag
ctgactgatt
897121 tactcgcacc tgaatttaaa atgtcactcc ctccctgcct ctccatctgc
gtcttcttgc
897181 ctgaagcagc tccccttgt tctccatcat ctttttactg tgttgtcttg
acgtggacct
897241 caatctgcct tatagtatca agaagtcact taacggcact gaacgctgca
gcataatctt
897301 aatcagacac tgcttcagaa ctatttgtct tttaatcatt tgagacttgc
atatatttcc
897361 ctttggcata ctgctttgta ttttcgtcat ccccttccag tcattcacat
taaaattaat
897421 gacttcctgt attttcaaag acgcgtgcag caactgatat tgaagacaac
gcaaatgaag
897481 agtcccatcg tgggcgttat ttgtccccga ggaggacaag agaaacagtt
cagtcgatgt
897541 ctctgtcttc actgatgtca tcgctccagt gaaggcgtca ttttcttttt
cttacatgtc

897601 agagtggatg tgattgcagc tgcggtgtcg tcagatgctt cattcttgac atgtagattt
897661 aaaaaccaaa caaacatccc tgtgcgttta ttaaaattgc aaatgcatac ataaaatgcc
897721 cccatttatc ttttatcaac ccgctggtaa taaaaaaatg tcacccataa ttcaccttaa
897781 attagcacaa gtgtacacac aaattagatg ttggtgatta tgtacggcct ccaatgtata
897841 tgtaactaat atagtcttat tgcttgatca tacacacaca cgtacatgtg tgtgaacgag
897901 ggacattttt atacaggaca aggtgaaggc acagatgtgc aggcagcaaa taccttcata
897961 caacccagat tatctttaat ctgaggtcaa gagattgagt gctgccttcc agttaattaa
898021 gatatgacta taaacagata agcagtaaat atataaaaaa tttaagcaaa aattgattca
898081 ttgcattatg tagatattaa atcgtgcttg aaatgaagtc tgcatattca acaaagcctg
898141 cacattttgt ggtgtttgtg attaaaaacg tgttcagtta catctgtttg cagcaaagcc
898201 gagcagggca gctcagtgac gatgatctgt gcatgcggat ccgtgcacag cgtactggat
898261 tagtctaaag tttaaaggtt gtgcaactgc aggaatcttc cagatttggg catcatgagg
898321 aaaaaaaaca accctgatct gaggcctgtt tgatctctaa aatctattac acaattggag
898381 actaaacatc tgaaaaacag agctgctgca cacatcagag tttatgctaa agaaatgatg
898441 tgattgtctt cgagttttga cctttcaggg acgggcacat ggatcagtga ttaatcaatg
898501 agcttcactc atctgcagcc ctaattcttc tgtctcggtg attccgctca gatctacgga
898561 gctaaaagcc gaggaaccgt tggtgcaatt cagccaagag caaagtgaag aataaaaaat
898621 tcaaaggtct gtattgatgc acgcacacgc atacacacac acacacacac acacacacac
898681 acacacacac ctctgtccta aaagccttat tcacgtatga ccaccttgtg agctaatgag
898741 ttagcatcag tcacaaacag tctgcgaatc ttaaagcttt gttgaaggaa cggaaacttt
898801 aaatgggtca gaaccagaat gaatgtggga gagcatgcac accaaagaag ggtgaggatg
898861 ctaaagagca acagtgacaa atgggagagg gaatttggag catcaaccct ccctcctgtg
898921 tgcgccatct gatgcagaga gggcgttgct aggggcaacc gcagacgaga agaggagaaa
898981 gatggaaatt tagcagctgg tggaaggaga gagggggcag aaaatgagtt aagagtggga
899041 gggagagggg ggcagatgag tgtcgggcgt gtttaggcct aaaatgaaga tgacgacaac
899101 aaaacaagag acgacagcag catcagcagc aacgaggcga gctgcccaat catctgcaga
899161 catgcaggcc cctccctggc tccgcacaag cactcagccg gctcagacct cttattaatg

899221 aatggagctg gttcctgtcg gaccgctgca catcgccgct ctgacgcagg agcagcagga
899281 ggggaacatt tccaccgaca gccgggacga ccgagagcag gaggagggcg agcagggcca
899341 tgaagcggct gcagagaaac acctgcggtg aggacacgag cggcgacatc gcgtcagccg
899401 cggctatcga aaacaaggac ccgcatccta acctccaccc acctggctca cctactccat
899461 caccgccgtc ccctcctctg attccgtcgg ccatgtcgcc gtccacgtcc tccaacgccc
899521 tcctcgccct ggcctctccg gccaccagac gctccatttc gatgggagac ttccgccgag
899581 tgtcgggggc tctgctggcg cgtcccgacc ccgcgtccgc ctccgccacg gcgccctcat
899641 ccaagctcgt cactcccagc tccagcatgg agttcgaggc ggctcgtcgg cgcctcctgg
899701 aagtggagga gcgccagcgc gtcatccggg agatggagcg ccggctggag gagctccggg
899761 aggtgttcgt gcgctccgag caggaggtgg tggttcacgg ggagctggtg tcgcggatat
899821 ccggcagtgt ccagcagggg gaaacgtacg tggccgagaa cagccagcgg ctgaagaaag
899881 gcctgaggtt caaaaagcat cgacccacca tcgtcttctc ctccatgctg ggactccgca
899941 catgcctccc gtggcctgtg aaactcaaat agatcacggg ggagggggtt gcattttaac
900001 tttttgtgct ttataataac ataaggacac atctctgttc tctagctttt gcttttcaac
900061 atggctgctt tcagattctg ctctcatatc ctgaggctgg tcctccacat ctttagagtg
900121 cctataatca tcacacacgc tggcagagat tccggtgtgt tcctgcagga aggttctgtt
900181 cagcacggcc atcgcgccag tggaagcgtg caggatgtct cccaggtttg cagagacgct
900241 cggctgtttg cagcgtagag atcgttctct tcgtggtgca tggatgagtc atttcaggtg
900301 gcgtgggagg acaactctta tgttcagagg cagcatgcag agtgatagaa tgtcaagctc
900361 tggtagactg ccgcatcctt acataaaagc aagggtgcgg ggacgcaggg gtagcaaaaa
900421 atgtgccttg gcgtctttga gcgttcgctc tggcgccacg accgctgctg ttgggcagcc
900481 cagcagtggg gcagcgaggg agccatggag gcttttgtgc cgggatgatc ccacctgtgg
900541 cctcccttga tctagtcacc tagcaaccgc tcatctctgc cctaaatctg cctccaccaa
900601 accagagagt gtttagcgga gcatgaaact gctgtgctga tagcacaaca cagcccttta
900661 caactaattg gctgcatgtg tggggaaagt attgattccg gaagatagca ttaagccatg
900721 catgcgttag agaaaaacac tgctggatgg aatgtcacag atatggtgtc caaaatgcac
900781 ataagcagct tcattagcat cgtacttgtt gtggaatact gagaagttca aataagcgtg

900841 aggctgttgc acattatctg tcacttatgc agtaggtgcc tgaagactgt gtatagattt
900901 caatttttttg acctaatttc gcttcctttc ttcgttcatt ttgtatttag atctgtggtg
900961 gttgattgta tctgcccatt ccagcagttc agtgctgcag acgcatacgt gcgtgcgtag
901021 aaaagctcag ccaaaacacc agggatagct atttttggct catgaacatg gagatggacg
901081 agggacgcgt ccaatcctgc tgcaggtttt aatgaacaaa ggcacgtgtg gcatcagcag
901141 tgtgggttct ctgcagtgca acagttactg ggccctctgg ctgcacaacc tcaggctggc
901201 gcttctttta ctctttttat tggctctcca ctttatttta aagtgatttg agatttctct
901261 gacaaggctg aaagggaaat tgaggctttg ctgcttgatt agaaagtaaa aaaacaaaac
901321 agaggcgaat ctaggactaa atgtgggtca gaataaagct tcagttgctt aatcctgttt
901381 tgacaataag ggcttcggcg atcagtttcg acgatcacta tgattttttt tttttttttt
901441 aaacgctact gtcgaaatgt cctcatgact aatagccatg gtaacctgaa tgtccataac
901501 ctcgactgta tacaagcatt ccacatggcc aaggaaagca ctgctcgccg tctttgtgca
901561 catctcaaac atgctcagct aagctagaaa gcctcgaagg ccctcctgtt ggtcccaggt
901621 tagtgatagt gttagagttt agcctgtaca cacttgtaat gtacctgatt atacagccag
901681 ccaggctgct ctctgtaggc cttatacggc atttgagcaa tggaagtaac aaaaccacgg
901741 aaggccaagg ttatcgcacc aatatctgag caggagaact cgcgcaccgc cgctggactg
901801 cacattattg ttgttttcca tgaatgaaaa agacaatccc aaagatttct gggcccactt
901861 gcagaatgaa tgtaatcaat gttgcttatt ctttcaaaac acacactgca gctgcatcgt
901921 taaacccacg ttagatccag ttttatccgt cgtttctgga gtttgctaat agatttaagg
901981 tcacatgacc gatagaaagt atctaaaaat gcttgagtgc attctcggcc ctgcactgac
902041 aggaatgttg tctcgtccct gttgatgtgt tgttacgtat gatgtatatt tctgatggac
902101 accaaatgtt tatgtacttt attcaccaca catttttgat taagtcgtgc atttttctgc
902161 tattatggat gttaatttta tttgtggaat attacaccca ttcacagggt tactttaata
902221 tgagttattt gtatcattcc ctagccttaa tcctaatttg caacatgcca ttaggctgtt
902281 tttcaggatt agaaactaaa actgaaacca tctgaactga aagtcagtgc ttttagaaac
902341 agctcaggct tttctttctg ctgctcagta caaaagtgaa tgttctgctg tccggtgcat
902401 acattttaaa cccatgaccc tccgatttcc agagctacaa cacaccagta aatgtctact

902461 aatagatctc cttgttaacg cgatcaggtt ttgtatgata gaccaattta aaataaagga
902521 acagaattac tagagagtct cagctccttt ctcagcgggt tattttgggt tagataacag
902581 tgtttaggtg gaaaatgcag aattactgga aaatcatctc acttccaagt gctcctaatt
902641 gcagggaggg tttgtgaagt tgcattaggc tgatgcttct gctgctgccc acttaacgaa
902701 aagcattaaa cctgcagaaa gcttttagac gggcacatca gacggctctt agagcagcct
902761 ccattatatc cagcctgagc tattctatca gcagatgcat cacaacctga catggttaat
902821 aagtaccctc tgaaatggga aacatgtcat tgtgaagatt attccaggat tacctttgtt
902881 ttttaataga ttatccgtat gttactataa attacgcaac tgggaatata actaaagtgg
902941 acgacatcat cggtgggttg tattggccgt tgtgcaccgg cctgacgagg cggcttgtta
903001 tgtaatcgac cggctggaat ttcatcgttc tggaatgggg ctgagcaacg tccagaccag
903061 gagaagcagg gacagagaaa gatatggaca aatgcctaaa gggacagaga gtgcatacag
903121 agcacataca agtcactcta aataaaaaca tcaaatgatt taagactata aaaaaatgac
903181 tgccacttta ttgaacaggt ttatgtgtat gttacaaata tacttactga aattaattta
903241 atatttacat tttacagtgg ctactggtta tcattgtccc ccgttggggt cttccggttg
903301 aattatcagt gaaaatataa gcagaaacag tgaccagcaa acaaccatta caaagagacg
903361 ctcaccttca ctaaaaaaaa ctctgagctg attatgcagc cattctttac tcttttacga
903421 ccctctaact gtcctttgga ctggaataac catattacca ggctgtcgtg tgcacgtttt
903481 tctatccatt aggtctttaa aatgactaat ggatagaaaa acgtggatag aaaaacgtgc
903541 acacgacagc ctggttgcgt tattgagcgc attaacataa aattaggaac agccacgctt
903601 gtccgaactc acttccctaa ataatcctga agactaaatt taagtacaat tgcgcttgtg
903661 aatgacggtg tgagtgagtc cacagtaagc gtgtgttcac tggactcatt attgctgagg
903721 ggtgaccaac aggctcactt taagtgccat ttcctggtag aacagagggg tttctttttt
903781 taaaaaacaa cccacagagt cattattgaa ggttctctat agacagatgc ctggagccaa
903841 cagaaattag atccttcgct gttgttgcag ccaggaagct gtgcccggag ccactccagt
903901 tacagattca ctggggacgt ctgaacttca ggtcaaaatc ttgacgagac cagggaacac
903961 tgcagtaacc caaacctctc tctgctcctt tctgcatgct tcgtctcaca caataactaa
904021 aatgcatcat attgcaggtc gggagggctt tcgggtattg atctgctgtc ctgcaactgt

904081 gacccaaaac cactgcagcg ccagagtcaa atgcgatggt ctatatgtgc ccataggctg
904141 ctgacgagac ttggctcctg cacctcagtg atggtcctcg gggtggggtt catctttaac
904201 aatcttttca tcagagatct gggtataggc acctacagtc cgacatcttt ttactataaa
904261 aaaaagcttc ttccttttaa aggagcttgc tacatacatt tttacagttc ttagagattt
904321 taaagtgcct tagacatttt cccatttttg ataccaggta aagagctaat attagaatga
904381 taaattatat agttggctat atgatattat aggcttgatt tgaactgcgt ttagagctaa
904441 agtgtagtcc accggagaga gcaagcaagc ttgaagtcgt gtagccaaga aagccttcag
904501 ttcccccgtt gtttgtcagc atagtgctgt accattactg tggtattgtt ccaaactcat
904561 gttccatggg gaatatccag agtttccagt tggtatattg ttatgatcct attgcaatag
904621 aattattttc ctctttagtg tcctttcagt ctgtcagttt gtgcacgtaa ttgacaggaa
904681 agcttccgct cctgctcggg tccccctcaa tgtgaccaac ctgtcatgaa agaagggtgc
904741 aatgatcacg tgacgataaa cgtactgagt gttgttttct tactttataa gactgcagga
904801 agcagagtta gggtatcctg acgctgcagt tacacttacc caccagtggg ggtcactagt
904861 ggccgtaacc acgatgatct catcctttga aaaggagagc tgctcagaac tgactgctct
904921 gcagtccacc agagccttga ccagcttgtg atgaggcctg ctgccagaaa cctggagcac
904981 acacaatttc aattttctga ttattcatag aaaaacaaaa cagactatac aagaggaaaa
905041 aaaagtttgc gtgtgtgcgt gtaccattgc attcctgcgg ggtcgcggga tgcaggcctg
905101 gacaggtgag gatggctgtg cgggtgccaa aaccggtgct tgagtgaagc cacttcccac
905161 taagctgcag tagagctcct cctgactgcc cacacagctt ggagacggtg tgctacattt
905221 accctggctg cctccgccgc tactggtgcg gcgatacgag gtcgtcttct ccgaactgca
905281 gaaattaaac acaaaaatca caagccgtca aattgtctgc agacttgacc acgacctgct
905341 gccagatctc tgcactgctg tctcacctga caggcccgat gtggttctga gcgatacagc
905401 tttgagaacc agtgtggctc tcacagctct gcctctgtcc cctgaacaga cctccttgtc
905461 catgcgggga cttgggtggt tttcccctt ggccttggct ttctgtgctg gttgaagatg
905521 tgtttctggt ttgtgtttgt ggactagagt gggagggcgg gttagaggac cgccgtggat
905581 ttggagtgaa ttcctgtgct gtgaccgaaa tctgggggtc tgaacgacct gtggaagaga
905641 cccagtcaaa gagtgacatt gatatcaggt ttacactaca ctgaatttaa gataatttga

905701 cttaaaacag atttcttttt aaatttcaac ccacctctct gaatgatttt agctggcaga
905761 ggcggaggtg ctgatgctcc agacagggcg ctgctggctg gagggtttct atacaggaac
905821 tctagattct cataggtttg acaaggtctg cttcctgctc caccattggg acaattgttg
905881 gtgctccagc aggcaaagga gcttccacca cctccgttag atgaaagcgg ggagcggttt
905941 ttgggcaatg ggctaacctg ctccaaagag gaaagctgta aatgtggaat tacttaaaat
906001 ctccagatat ttctggcagc aaggcagaaa cctaccctct catccagatc atcctcgctg
906061 tcataaaact cctgagaatg tgtctcccac ataaactcca cgtggatctg agagttgaat
906121 ttcccactgt gagcaagctc caactaaaac acacacacac acttaaaaat aagtttctct
906181 tttaaaacaa aatgttacaa aatttctatt ctatgtgaaa ttttgaggta aatggacgag
906241 attcctgttc ttttcctcta cacacaaaaa ccataaagac aggatatgat agaagacagg
906301 tctgattcag agaggcagat ggctggtaag aaaataaaac ttgtttatga atgatgcaca
906361 ccggggcctg atgaggatag agtttcttat cctgggagtg acttcagctg cacagggaag
906421 gatcacaagg aatcctatcc aatcgtggaa gtactccccc ccttccccct ctaagttgcc
906481 caagagattg aagatgactt taaactaaag tgtacagatg attaaaaagc gcctcaccag
906541 gtcgacacac tgcaagtgct gaagcctccg agcagcatcc agggccgttt ctcctgcaga
906601 gttcactgtt gtagaggaga aagatgggat tgtttggtga actagcacca agcaaaaaaa
906661 gttctttaac attttttaaa attgcacctg tgtgcaggga tgccttcgat ttgagcagca
906721 actttagaga ctctggcttg tggtgcagga cactgtagtg gagtgcagtg tttccttctg
906781 ctgtcctctt ctccagacag ttgctgtgat gtgaacatac agacggtgat gagcatcagc
906841 agaactcctg agcaagttga atgatccatg tgactgcttg tgcagggtct gacctgttct
906901 gacagaggaa atctaccagt gccagagagc tcctttcctc aaggcgcacg gcaaggtgga
906961 gagccgtctc tttcaccccc ttcaatggga aaaaagaggg caagttgagc cctgtcatct
907021 acggtacatt gaagataatt tgatttggct aaatgcacat ccaaatattt gcttttgcac
907081 agcaaaacgg gtatgtttat gggacgtttc tgtgtaattc aatgagcctg gctcacttgt
907141 ccactagaga catgagctct gacctaaaag ctgaaataat aaaccaagga caactagcac
907201 aaacaaatac acacacaagc acacacggtt gagtcagcac tgtcagatgt gcagctcatt
907261 ctgagccaga agagggaccg ggagaaaaga tggagcttaa tggaaatgag cagaggaaag

907321 agggaagtat gatgagggaa aaattaaaaa cagacatcaa agggggccgt
ttttcatgtc
907381 ccatgttaag aaatgtttat tctttacctg accgtcaggc aacactagtg
gtttgctgag
907441 gtctgctccc tcagcgtaga gctgtaggag agaggtgagg tcacgactcc
tcacagtgtc
907501 atacagccgg ctcgggtctg cttcttctct acgcatgacg taacgacgct
ccgcgtattt
907561 cgccacaatg tactcttttc tggcattcct tgttgttcag tgtgcaagga
agaagagatt
907621 aacatttgac aagtcttatt tgctaaacaa tcacaattcc tgccagactc
acatgtcact
907681 ttgtggcagg ggcttgattg agtcatttgg aaggcatgcc tccattatgt
cattgaatct
907741 ggagttgccg atgcttacag ccaactgcag tgacatacaa agagagggtt
ccagacaaaa
907801 gcagttcaat gaacatcaaa tataatgttt ggtgcatgta ttaccaaaag
ctcagaggtg
907861 ctcaggagat ccagagtcag tgactggatc ctcgagtagt ggacgccaag
ctctcggtgg
907921 atgccagagc actctatgca ggtcaggatg cccaaattgg tcgacagcca
tgttggatct
907981 gaggtgcaca ggtcagaaag gagacatgag tccacctgcc tgagcttatg
aatgtttaca
908041 gttgtgaaaa ttggtcgtaa aagagattca attcaataga tttatctttt
tttctaacct
908101 ggagctccac agtcagcgca ggagtcattg ccaggcatct gtcgaagttc
acttatgata
908161 gcccgagaga gttcctggag cccactgtcc tggaggtgaa gctggtctcc
aaccaaggct
908221 gtgttcaagg cctcatcctt gctgttctgc agcacggaca cccagctaat
atacacacac
908281 atacagtaca aaagcacttc aataaatatt aaaagttcat cattcccgag
aggatcagaa
908341 ggaatcccac atgtgttttg aatttgtatc cggagataat tcagctttct
ttctctgtct
908401 gttttccagc ctcatactgg acctctgtca tctccagaac cctgaacttt
ttttcctttc
908461 aactctttcc atctctctgt ccccaaactc agagcacttc acatcaaaag
cgctgctcgg
908521 aggcccacac gcaggggaag ggcaggaaga gaaagacatt cgtctacttc
cctcaatccc
908581 cacaacacac aatcaagctc tgctgctggc tgtcagtggc tagaaaaagt
tccacccaac
908641 tccagctggt cagaggggc gcctggagga catcctcagc ctttcttcac
cgcaataaca
908701 ctgcagccat tgtgtacatt cccttttaaa accaagcact aaaaaactag
gggaatgacg
908761 tgtaacaaca tgcttaaaac agcctgaaac cagaagataa ggcagtgtaa
attataaacg
908821 acagttgttt caacacttac atcagacatt cctgctcatc ctctgcctgg
aaatggtatg
908881 ttcgattatc tgtggagaag caaaaaaaag ggtgttgtaa tgcagccgct
gcagtttgag

908941 agagcaattt accccattag gggtgaaaac atgaacagcg ggaagaaaaa catacatctg
909001 gaccccagat gacacttgag tgatgttatg cagagtggaa agtgtcatgg gtcagacatt
909061 agaaatgaac ctacttgtga ccaggtcaaa ggtcttcttg tcctcagggt tcggtcgcac
909121 ctgacaggtc agtaggttca acttggccgg gggtcgattg atctgaaaat cgagcgcatg
909181 ttttggaaaa tataaataga tctctgctga cttttatcag aatttacatg cctggtgaaa
909241 agtacaggcc cgcacatcct caccgtgctg tgagaaatag tcaagcagcc aaacttcact
909301 ccgcattttc tcttctgcca cactttcctg atcctgcagc acaacaaaca agaaaaggga
909361 gggaaaaaat catttgaggg agaccttggg cggttcagga ctaagtgatg atagtgtaaa
909421 tgtccaaata atggtgatca gcactgaaac gacccagaac atgcgtcagt ggcctcttcc
909481 atctccaatt ccccgctcac aaccacatcc tctccctgtg tcgcactgta atctaactcc
909541 gtgtgtgcgt ctgtgtgtgt gtgtgtgtgt gtgtgtgtgt gcttgtgtgt gtgtgtatct
909601 acatgacaca aaacttggaa ccactcagtt gtggagtctg ccaaaaagtt tgaaaggagg
909661 ggggcactgc ctgagttttc atttcacaat aatgaagacc aggtggagtt cacatagagc
909721 tgcatgttct ttatctttac caggtacaac tgcgctagat tcgatgttca tgtcctcagt
909781 tttagcaaaa ctactttccc gagttacatc actttataat aatggaatgc aagtttgaac
909841 ctgtaagctg taattctgac tcagcattca ctcacccgtc gctcttctta agcaggaaac
909901 cagatttctc agttccgtac ttcttgttgc cctgtggttg atggatgctg tagccattgc
909961 cggagttctt cctgttgaga tactcctgga atacaggatc aaactcttta ttccagttct
910021 gaagctcatg aaaatgtgaa acggttgtaa tccgattgcc acaactagca agctaacaag
910081 aaaagataat gtttccgatg acccctgcta tgcctaagaa agctgtgaaa acagttactg
910141 ataacacgtc gattgtatta tatttggaaa attttggatg ccatgtaagc agattatgtc
910201 tgtaaacata tttctggaaa ctttgcttta actagattct gaactgtaat tggactacgt
910261 tgcgaatgta aacagggctt gtttgttatg aatgagcgta cctctttccc ctcaacttgc
910321 agcagtaatc tgagagaatc cctcaactgt gtgagctttt taacttcctc gtcctggtcc
910381 aggcgcacct aaaacaaaga cacacacaca aaccaggttt ccataaaggt ctaacagagg
910441 tggggggcg tgcttttctg agagaaggta cggtttggat taaagcagcc cagtgagcag
910501 ctctgaatgg aacataaatc agttaacctc aagcattaca gaacttttct gtgtgaattt

910561 tctcctcaga ggctgaggtc tggcatgcct gaacaaatca ctacgctgtc catgcagaac
910621 cagaaaaacc tctaacgctt ccataacatc aaggtttcca aaactccctc cagaaagagt
910681 taggagaagt atctgctgac tgggaaccag tcaagagtgg tgtgcaatgg agaaaggatg
910741 gaaatggggg atatttttaa tattaacttt tggaaacaga gttgatttat taaccctgac
910801 taatattctg cctgaagtta ctaaataaca acactgtata cagacatggg atgactaaga
910861 tttttacata aacagactga cagaaggcag gtagtcagcg aaccaccagc agctcctggg
910921 aactcaccgt atgaacagat gcagccagct tctcgataaa gggagtgaga ttctcagccg
910981 ctttcagtcc atcctgaaag aaactgaacc agaaaaaaaa cagcatttgc tgagtcactg
911041 tggcactcta cagcctggtc cgagcaaact agaacaccac atgcttgaac tgtagaaaca
911101 gctcagtcca acaggaaacg tctgatttaa caggtaaaca acagtaataa tagcttgtaa
911161 aaaagaagtc aaaccacaga agttcagtga aaatgtttag gtagaactcc gtaaacaaag
911221 gtgcaggaaa gaaaaggggg aataaaaaag gtttgggact gacctgagct gagcttggaa
911281 gtatttaatg aggctctgga gaagatctgg gccctgacgc actttgagct cctggatctt
911341 cagcagatac tggaagcata aacatggacc tggagtttaa caaaaccact ctgcccaaaa
911401 acatgcaaca ggattagcaa agacaggaaa caaacaaatg gtttttcgct ctggttttta
911461 tatgattcca aagatcatat cccttattta attacagatg acagtgggtg aactttgcca
911521 actgaagatt attccctgca tggatgtcat agctacacgc tcactgctcc ataaaacaaa
911581 ggtctgcatc catctggctg cgtgcagcag agcatttctg tttggtgtat atttgtgttt
911641 tcatgcacgc agaggcaggg tggaacagtc tggagcaagt ggaaattcca ttttccctgc
911701 ctctcctctt tgtctcagca gctgggctca gtcggggtta gatgaaggtt ttttcagagc
911761 tttgtccaga tccttgtgaa ttcaacacct gtatgcaaaa gcatagctca acagtcttcc
911821 ctgcacttca ccgtcgttct cgcgtgcttt gtagctaata taagctaata tatataatat
911881 attttaaaga ccacccatgc cacacaaatc aagcccacct cgcctgtgta aaaagggtgt
911941 ctgggtttgg tgtttagact ggcactgatc ccccacctca cacatttgca gctgaaaggt
912001 cctcctctct ctctccatgt cctcccctcc gtccgatccc tccgtcctga tcagacccag
912061 ctgtctgctc ttctcctttc tctccttttc caactttcct ctgatggggg agggaaggac
912121 gagcaaaaat ggggaaaaga tttagtttat catttgttcc ctttttctgg gtgattttaa

912181 atgacatgca tcagttttat tgaaggccat ttaatggaga gtggccaaga gggaagctat
912241 aattagatta aactgctgtt acaccaagta ctggggccca gttaaaggcc agaccttgct
912301 ataaaatttc aatttggttc tctgtatgca ataacttcat caaagttaca ttacattttt
912361 atgtcgtact ccttccaact cttctccatc tgtttttga gctcctgtag acaagaaaga
912421 caacataacc tgcatcaatt aagagtcacc acacccaaac acacatgacc tggttttggc
912481 agaggccttc accgatgatc accgaatcct cacattttgc tccgactgag caagatctga
912541 gcagacattg gcactttctt cacagttcga gtgaagcaac actcgcctct cagatgggct
912601 gtttcagaga cggggctggg gctggagctt acagaggcat tagcctctat ttactcaata
912661 gtctggtgtc acggccaatt ctcggcaaga ctgtggtttt aggctttagg acattacttt
912721 gcacattatt ctgcataaac agatgcgtct tatctgaaat ctttctaaat ctttctaaga
912781 tcagctgcat ctacagccta gacagatagg cattatgcat tcaaggggga atcctctctg
912841 atacacatgt ttatttattt atttttacag ctgcaaaaaa attataaaca cccatagtca
912901 ctggaacatc atgtttgaca tggtcttaca ggcaccaccc ttagagatgc atcatcaaaa
912961 gctataaagc ttataaatcg caatatcaac ttcagcagct atgactgtat atgcagtttt
913021 cataaatata cattcaatat aattctaaaa atgtatatat gtatagtttt ttttgtcttt
913081 tttacaatat tgacaggtat agtgtaaatg tcaatatctg aatacattgt tacacaccag
913141 caggtatagt ggtgtttaat gatcaaagtg tagactcaca agtcggctgt ctcggagttc
913201 tgacttcagc acattttcca aaggaaacgc catgatgttg ttgaggtttt gcacctgaaa
913261 caagaaaaag agcaaattac aaaagcaaag aaaatgtgtt ccattattag ccacagatgg
913321 ctcttatcac ttgcagcttg agcaaatatt tatgcacgcg cactctgcca tacacccgtt
913381 tccccccaca gactcttgtt tctgggaagc ccaaaaaatc ctgcgatttt gtcattttga
913441 gaagtggtga gtcactgaga gaaaaaacgg gaaaataagc ggcaccaaca ggaatttttt
913501 tgatccaagt cgactgtgac ataatgagtg tttgtgcaca cttaagtgga acctgagatt
913561 aaattgaggt ttggatggaa atgagatgaa ctgattttat catgtgagtc acggcacaat
913621 cagcaaggcc ccggagaggc tgcagcttta cgattagcac aacttccctc acagaaatac
913681 ggccgtatgc agcctggtag cgctctgcct tttaacgtca tcacctcttt acacatctga
913741 actttaaacc cagaatatgc tcaggcaact tttttaaacc cctgttggac tggaaccaac

913801 ctaaatgtta acataaataa catttcttct gagctgacct atttatattg acacaaagac
913861 ccagagtcct aacgctgctt tggttggcac ccgaaacaac ccgagtattg acccaggaaa
913921 ataccctaca accaaattat cagcatgcgt atcgccatca cggatgaacg tgcgcgacct
913981 acaaatgcag gggggtgcga gcgcgtgcgg tggtttttgc accaccaccc acctcttccc
914041 atctgcacgg cccagtcccc cccctgtgag ggtctgactg actcgcagga gggagcgcat
914101 gactgtgtgt tgtgttgtgt tatgcatgga ggtctgggat gaattatcag cgccataact
914161 gtcagtggga gggttccggc ctgccccaga tttcagattg gagctccacg agcgcacagc
914221 tgctgaaaag ccatttccac aatgcgtgta ataagggtgc tcacgcaata tactcaacat
914281 gttgggagag tgctttgtga acattctgct tagtacatct aaatacttta gagctcttat
914341 ttccatcttt ttggggttgt ggattttcaa ccgttcgagc tcctctttcc tcaccagatt
914401 tttaaagagc gctgtgacct ctctggtgaa cacggccaga ttgaggaagc cggtggagac
914461 ctcattatta tcctgggtca ggtggctgtt ccccaagttc tccagaacct cgatatactg
914521 ttctttgttt tccacatggg ctgaaaaaaa acaaacaaac acacacacac aggcaaaatg
914581 agcataagca gaaaagtcac ttgaaatgag atgtgcagat agtttaaagg agacgtgctc
914641 tggaggagcg ggattagttt aactagacag gcatgtggga taaattactg ggttccaccc
914701 attgtggagc ccacaactct ctgctgctta gaggtcagtt ccccaggtgt cgtcatgggc
914761 tttgttgaat ttaccgtagt tggaaaaaga tgacggaaac taactgactg gaattccctc
914821 cgtttatttt gacatctaaa tgagatttat gcaaaaatag atatttcaca catgaggaca
914881 attaacttaa tatgacttaa ggaattagag aacagatgtt gaagttgaga cgaagaatga
914941 aaaatgagtt gtggtggaaa ataatttaaa ggttttagaa ttccatggca atctagagcc
915001 aagaactcac ctttccctta aattcaaatt cctggaattt agctcaacat aaaatttata
915061 gctgctgtgt cattccacac tctcccacca gaaaatctaa gcagcgcttc tgcagcttaa
915121 acatccactc acaaagccac aaatggtgtg aaaacactat cagcagtaca ggacggatat
915181 tttgttcata aaaatacatg cacagcacat gtcaatgtta accttcattt acgacctcat
915241 tacagacgct ttgagtgata aatcaattat aattatctta tattgtgcta ctgatgtggg
915301 aactccaagg atgatttcac agctgacctc tcactaccag ataccagctg agagcctcga
915361 ccctgctcac tgatctctgc cagaacacac actggtctta taggccacaa cagggttttg

915421 gcagaaaagt tggactgagg tttaagggaa catgcagtga aagcagagaa catttctctg
915481 cgtgtgtttt tacatgtgca acacagtaat agagcatcag accatgtggg gggagggggg
915541 cactcagttc tgggtccatg ttgagtcagt ctgttaaaat tcatgagggc tgattaaggg
915601 gttcaagagc acaacggtag agggaggcag ctttactgga accaacccac atgattttgc
915661 ttaaacgcag gagaaaacgt atagggagat ttgtcacaga gcactaacgg tcttgggcct
915721 gacggtatct ggttgccaaa ttctgggttt ctggctttct ctgactcact tttactaaaa
915781 actgtcaatg cctcaaaatg ggaagcaggc ttacaagtgt gcactgaaaa aagaaaagaa
915841 aaacaacttc aggatgtctg ctcctctggc acgctcacat ctcatgtgtg aaacagccac
915901 aaaatcaggt ttgtagtcct tattttaata cacaccacgg aaaaacttgt acacaatacg
915961 gatataaaca caatcagcct gcctcccaca gaggctggtc ccataacaag gggcctcact
916021 cataaaaacc actgcccaca tgagctggat gaggtcatcg taagaagacc ttatggtgaa
916081 ttatgctgag ccccccaccc cccacccccc agcactgaac agctcatcct tccataacag
916141 aggaagacct ttgcttagaa catcacatgc ctcatatttt tggagccaaa catccataat
916201 cttctacaac agcttttcct tgtgcttatt caaagtagac ctgaaagttt ggaaataagg
916261 aagatacagg ctggaggcag ggggttgggt gtgcatgaga gtgtgcacgt taaaggagaa
916321 cttttgagat gggcagaact ttacccagag gattattaga gggagactta cacagcccag
916381 acgtgtgtat gcttttaatc atcttcttca tcttctgtag cgtcaggtgg tccgtctcca
916441 gagactgcaa tgaaagcacc acctttaaga aactttggtt ccagatcaca ggaagtgcaa
916501 tttcaagcat gtttggaaaa tacaagtgag aaactgaaga tatttccatt cttccgtctt
916561 atgcaagaac actcaaacgt ctacactctg tctgtgtgtc tgtcgtaact ggtttaaaac
916621 agacactggt gtgagctggc aagtgtgttt cagctcggca gcattttcac acgccaggtg
916681 acagaggtga accagtgagt caccacggtc gttctgcagt cacgtgcaac ccctgagtgg
916741 tgtcactgca cctgtctgac agctactgtc attttcctgg ctcgagcggc ccgaatctct
916801 caatgaaacc aggctttaat gggttgggtt tttttgtggg gaaaacaaga cttccagccc
916861 caggacatgg aaaaatctag attgtttgta catttctttg gtcactcgcc tcttctgatc
916921 caaggctgga aactcacaca caccttgttt attcttgtct aacaggccgc ttgtttcatc
916981 aggctgggct ccacctttgc tccaccagaa attttttttt aatgagccag taagtaacag

917041 aggctccagt tagatggtgc ctcccagtct ttccaagtca actccctaag tactagttcc
917101 ctaattaaaa ggcttcacat gtggttgatc tgactggaag gtcatttctt tgcttaataa
917161 attgcttcta ggttaaggaa cacacacaca ctggaaacac aaacagagaa tggacacaaa
917221 catcttcgac aaacaggtga aagtcatcac caccgtcaga catccgatcc ttcatttatc
917281 tgagcacaat gacaggtttc actgttagaa aagcggtaat cagtagtgtt ttatttaagc
917341 ccatttagtt ggggggaaga agtgcctgtg caataattaa acccattctg ggtggcagct
917401 gaaggaaatg aaaccgttgc ttatctttcc tttttttct cctttatctt tgctttaatc
917461 gggagtaata agtaacaagc ggttgagcca agctggtaaa tggagttgct gtctgcgtcc
917521 atgaaccgtc cacacatgcc cacacactgc ccaggccatt catcacgccg ggtccagcaa
917581 accagagccg acagaagaac caaagattga gagaggaaca ttcgggttta actatgtgag
917641 ggattccaac gcggccctcc ccaaagtagc tatttcagct atgcatttct gagtatgtca
917701 tggccatatt taggtgatta agccaagata aatgcatctt ccagctgctg tctccatccc
917761 tgctcagcaa tctgcaacat ggcagctttt ggaatgtgtt ttcacacatc cgggtaatgg
917821 atcaatcaga tcatcacaga ctaaacttag aagatttatt tctcagatag atctgagaaa
917881 ttatccagaa gatttgcact gtttaaaact gtaactggac ccaaatcttt gactttacag
917941 ttacgtcaca agacagacaa gaaggaagga ctgtttgctc agatgttttg ttttatggcg
918001 gcctttcttt tccctccttt gtctgcggga gctttcggaa gagcaacttt taaccagcat
918061 gcatatacac acacacacac acacacacac actcagatgt gtgacaggtc caaagtgcga
918121 gttcgaaagg ccttaaagag ataggttgca caaaaataaa aaggccatta tcttttgaac
918181 actttgagtc actagaccac acacagcttc aatcattctc ccttagtcct ctgcaagcac
918241 gttcaagtgt tgctgcgccc cctttctgtg gtgtgcggta tgagagcaca caaacacaca
918301 aacacagcaa tagttgggaa aacagcagtg cctaaaagct taaaaccaca taaatcaggg
918361 catctatatt ttatttggct gcattaaaaa ccctcaatgg ccttcagttg ttttcagatc
918421 tggcaaagct gattaaatct ggacaaagac agtaacaaca cagttgtgct aaagtagata
918481 ataggttaaa ataattacaa ttataatgaa tacactcaac taaaatctgg taaagccaga
918541 gtgcagattg atatctagat tcagatttgt atttatttta aatctcaaac caaaaatatg
918601 aagtaattga ttaaagttat taagttcgct gcgtgtctgc aatatgtgtc gagtaatgct

918661 aatagaagca tgctacacga acaggtaaac aacagagaaa ggtgtgcgtg cgtgtgcgtg
918721 tgcgccgtgc gtccaaaatc tcacctcttc caccaccgaa accgtgttcc gacagtcgct
918781 cattttggac tgaaagcccg acgtcccggg ggacgacagg tcctcgtttg tcatgaccac
918841 aaactccgaa acgctgattc tctccggcat gtttggagcg cgaacagaac cgaaactcac
918901 tgaggagaaa cgtgacccaa agtttccgac gcgcgcgcaa ttcccatcgg agggaagagg
918961 tttcactcat ccattgtgct cccagaagtt tgggtcagaa gcgcaggaag ctccgccggg
919021 tcgcctttca ctgaaataaa gttggccgaa gtcggtgccg cgtcctctcc cactcgacgc
919081 cgccccgagt ctgtgtgcgc gtggaggtgc gcctcccctg cgcgcgattg tccgcccacc
919141 gagggagggc aaaacttcgc gtcacgtggg aatgtttgcg cgagaggaga gagtgctcgt
919201 gagcctctct ccgggagtct tttcattttc tcgtttctat gcagagctcc gagggcgcgc
919261 tgcggtcgcc atggttacag aatgcctcct tcacacactc ggcaccagga gaggaatttg
919321 aatagatgag cagattagcc ttgcgttgag gaggcgttcg ctgagcgtcg aaagttacag
919381 gtttaaatgc cgttaataaa atggaatgtg acgctcagaa atgttgtggg ccgcaggtgc
919441 ttgcagagaa tctcttttaa gtcgtttgga cctctggctt gatcctccaa aggctttctt
919501 cttttccctt ttcatgtcat gagatatttt ccctccccat tctctccctc tcctgacatc
919561 acagttaaca aaaaaagaga acacgcactg gagaggattt cttttaaaaa aaaaaaaaaa
919621 aaaaagctac tagagttgca gacggaacat cacttgcatg cttggcattg ttgtgtctct
919681 cagcacagag gaaagaggtg tatcactgag ctttgcctcc ggccatgata caatacacag
919741 tgtggataca gtgtagatgg gtgatgttag ggcaccacat caaatgcagc ggctcctctt
919801 tggttttacc acaaacccac agatttgtcc acatttgaat taaaaggaaa actggtcttc
919861 gctctatccg catgttgttt gaatcatatc catccgtgga aaaaacagac tcactttgtg
919921 gatttatttg ctctctgtaa ttacctaatg ccaccagtcc ctgaatgcgt cttgtgttgc
919981 agattcacag tcagacagtg tttaataatc acagacggac aaactggttt ttgtgtcggg
920041 aggcttttca cttaacaaat aagtctgacg tgccaacatg tacgcgcacg gcttgtttgg
920101 agcgagtgca cgcgcacgca aggcctgtca accaaagatt aaggaggaat atttaaacat
920161 gttcctctcg aaacacagag aaacaaacaa aaaaaatcac tggaagagca aacccacttt
920221 tatctgcaca agctgaggaa acacaccatt gaaatggcag aagaataaat gggaaagtct

920281 ccgtatccaa gtaagaaaaa aagcatcctc tcttctgagg aagttggggc acacgacaaa
920341 ctccaaacaa tgcacggcca aagaaagagc tcctctgacc cactttatcg gcttcccatt
920401 cttttactgg ttctatcgga ggcctatgtg tgaaagtgtc gacagggaga acgtgaagat
920461 ggagataaag atatgaatgt gcgcagaagt gcaacaaatg tgtcattgca tgcttttttg
920521 cattgatgca ttcattattt tgcctgcaca ggctgatgta atgcaggggc tttctgcgtg
920581 caacaatacc ctccttcagt ttcctttgca gcctgcagta aagaagcaag ggctttgtac
920641 gtcctccagc cttccagcct ggctcctttt gtgaaagtga acaaactggt ctgctcaggc
920701 actgcactcg catgtggagg agagcacaag tccagggagg aggaaaaaga tcattagaag
920761 ttggcaggaa gtgggattgg gggaaggttc gacggtgaag gtggagtgac agaggatggg
920821 agaatgatga actctgctgt ctgagcttgg gtgctccatt gtccttttcg tcacccccca
920881 cccttttcct tcctcagcag cacctcgtta tcgccattct tctctccttt ctgatggtcg
920941 ctgcacgttg cacaagcctt ttggctgcag aggggttaga agcaccgaat gctctgacgc
921001 acactcagag ttctgtaagt gacatcttcc aggaaagagt acagctgcca gtatttcctc
921061 tccgtttccc agcacccaaa tggagctgac cccccctta aaatctccct ccgctctctg
921121 gttgttgact cagctccgct gcttctgcta tcaatttact acccctgtca agccaggagg
921181 cccaggctta ccctcttctg gatgatgcgg gtcagggtgg ggagagtaag agagggagtg
921241 gatttggggt tttttttccc tttagatttc agtttcaggt tgaaataaac atgagaaaat
921301 gtcttgactg aggtgaggcc cccacagaga gcaagtggag gatctgtata atggcaccaa
921361 tgagccagca gaacaatctc ttgttgacat ctatcaaaac caggtaacac agactttcca
921421 gactggcggc acctacccaa gcttgcttgt gatatatatt tagctgataa ttgcatcaag
921481 aggttcatgg ataaatgact tcagttaatg gctccaaacc gctctgagga gttttatgtc
921541 tgcgacatgt tgccttcttt cacgttcaca cacttttcac cttacctttc tccaccagaa
921601 aagagcagct ctaaataaac gtgtgtgtgg tcctaaacac accaaccatt ccttctaaac
921661 tgcgccacgc ttgtggaaaa taacaccacc attatgagct aaaacaaagg cggcttagcc
921721 gcaggttgat gccattcatc cacgatctgt cagctggctc ctaccgggcc tgcagacgtc
921781 tcccagctaa agctcaggta attatatttg cttaaatctg ttgttcgagt acccggaggt
921841 gcgatgcaac ggagctggcg agcgcacgta tgtacgtggc gtctgaggtt ttcgtttacg

921901 gcctcttgtt aaacacacga gctgttgtgt tgcatctgtt aatagacact tcatgctaat
921961 cggcaccaga tgttcgggag gaaaactggt ctctgggaga cgacatgatg gtttgtatgt
922021 gacccactga actttgacct cctgcttgtc tgagctcttt tgtgagctag tctccgaggc
922081 aaccgagccc caaggaatga ggggtggttt ttgtctacct gtccggtttt attaattgct
922141 aattcagcga cattgttact cttcagatca accaaatttt gcctcttcag gcgacgtcac
922201 ttctcattag atcacagccg tcctgctttt aggcaccaca ctctgcgctc atggggcctt
922261 tcatagaatt tggaggaaaa tatatttaaa aaaaaaaaga tttaaattgc aatgacaagg
922321 aatgaacata aaaatgaagc agcgccccaa gagtaaaaca tggctccaat atttattcaa
922381 tcatttccaa acagaccaca caatattaca aaatattaag tgctgtatgt catacaaaac
922441 aattatcaga cttcaaaaat gggtataaat acaaattatg tacaaatacc atcaaaaaaa
922501 taatcctcaa agaaccctag aaactctgaa ttcacatcta attgaaacaa acatgcacgc
922561 aaaaacaaaa ggttcaagtt ttgttaggac aggacactta gcttatctct gtttagaggg
922621 aaaagccaaa aataaaaaaa aataaaataa accccaacaa tcgaagcaat atttcaaaga
922681 tgaacacaac ataagccaca actgacttga gctcaaacag tttcaaaatt caaattggaa
922741 caataaatct aaaatttaaa aaaaaacaaa catctgttgc aataacagat tcagttttgg
922801 gacttctact ctaaatcttt attacattga ttcttcccca gcattgctgc atttcatgtg
922861 ttttaggggt tatttcctca gaaatacaat agtagtttca cacaaccaca ttaaaatcag
922921 caaactcaca gcgaaagaag agcacctgga tttgaagtaa agctgccaaa ccaatgagag
922981 taaaactccc atcctgctca ttttcatatc aactaagggt taagagcacc agcggaggcc
923041 cacagcttag agcagatgac ctgagcacct ttaaaaagca gatacagtat gtttaaacat
923101 tagatgcttg cgaatctaca tgaactggtc actcacagct tcatttggtg tctttttgta
923161 tccagtgatt gtatcctgaa aagccgcgtg gcagttaaag ctgacataat acacggctgc
923221 cccctacagg ccaaagagcg gcacgaacgt taaaacttta gaaaagagga aataaagaaa
923281 cattaatcct gagttgtcac gctgtgagca cagcctgctg cccgtccatc agattttagc
923341 tgctgctgac gggacgagca actttttctt ttcttttttt ttccaaattg tcatattttc
923401 agtgaaatct gagaacattt ccaagtttaa aaaaaataga aaaatcgaca ttttcattag
923461 taactgcaaa cagttggaga tcacacatca gtgcaatctc agagccactt caatcttttc

923521 tttagtcaac atatttacaa attaatttca tcatattaaa atgaacaaaa cagttgacaa
923581 ttttactgtt aaatcacaaa atagtaaaca atgtaatggc tttatagtgt aaatttccta
923641 cctcctgaag gcattagatt aaatacccac ctcccctgtt taccacccct ctgtccctta
923701 gttcgacaac agatccccga gatcataggt gtcgaagaag tccgacaccc cctcgccgtc
923761 atccagactc cagaggtagt cgtcgtggtc taaaggtgga gagaagctaa cgaagggggt
923821 gttggggtct ggggtgaagg tcgccgatgg gagctggtcc tctgtgatct ggagaaggct
923881 ggggggcagg cccaggagac cttcgacatc aagaatggag gaactggacg ctgcggctgc
923941 tgcaggaatg tgagctgaaa tgaggagaaa agcaagaggt gctttttatt cacaattaaa
924001 atggatacat gttaaattcc agcaatattt cctcacaaac ctggaattct aatagtaagt
924061 aaacaagaat gctacagtga gctcagctta cattcaacag gctcctcctt gatgctgcag
924121 ctaggaggcg ctgtagaggt cgtatgcgaa gtacagacag tgggaggaaa ctccttttta
924181 ggagtagaga cacttttaac cggactggca tcttccagcc cgtcctctgg acacaggtag
924241 acttcaatgg ggccgttttt actcttcaaa tagatctgta gtgactcctg cggcagcaaa
924301 atcataacat cagaattagt cattactttt ttaggtgttc aaggcagtcg tgcagaggtc
924361 cagatgtgcc gtctcacccc ttctgtgtct ggaacctcca gtttggtgtc ggctggagcc
924421 ttgacagcaa tgactgtttg gtcctggaga ctgccgatgg agcggatgtc gtgataggtg
924481 acatagccca gcgtggagac tggggtcaag ggtcagaacg gcaccggaga ttaggatctt
924541 tcacgcacaa ctgccccaag aagagcgcca aagggccatc aagacagcgc tgcgtgtaaa
924601 ggatatctct ggttgtctcc gtgttcggtg agttgtttga gctgcccggt actcgagagg
924661 atcagctcat ccagacattt ttctgcttgc tccagatctc caatctcctt cctaagagtg
924721 cacgccttct gtccccctcc tgttccaccc tcaaacacat cgccgaccct accaggagac
924781 acatgagcat aagttatcaa atacaaaaag ctttatttct tactataatt taaaaaaaat
924841 tccagagttg tcacaaaaat atctttcaaa taaaggctcc tctatttaga ggtaacttaa
924901 tattaccacc aacatgttaa ccagactgac aaagctacaa gcccaaactg ctcctatgga
924961 ggaagacacc cagtaagatg aaaaatatta gcctcagtgt ctgcagagtt cagggttcat
925021 ggggcctcta cagagacgag aagctccagg ctgccaccac tggccatggc tgagaatcca
925081 tgcttcttgt gtactttgtt tgcattattt acagtaatag ggcaaccttt atattcagtt

925141 acaaaagtag atatcactat tggggatctc agttcagttc ctgctgctgg tttgctactt
925201 ggtgtgaaag cgaaaattgc atctttactt ttgagagtgt ttatgatgtc agtgttggtt
925261 gttgctgcca acagctgcta actgctgctg ggtgctctaa atgtcctggt tgctaggggt
925321 ggattgctag gggtggacat cactgtcatt ctacttacac agaacagtaa acccttcaag
925381 tgcaccatga tccacttact gcacatgtta gtgattgttt attgtatacc tagtgcagta
925441 acaactaaca accaataggg ggaaacaaca cgttcacaag cttaaacgca ttacttcttt
925501 tgcacatgtc acatttattc tactcacagc cactgaatgt ggttcttgga ctttttacgg
925561 atgagctgca ctccctccag gacgttggtg atatcataga tgcgtctctt ctggacctcc
925621 aggacctcgg tggcccagtt cagatcaaga accccatcgg gcgactcggc aatcaggccc
925681 acaaatttct tagtcagcag gcccaaggaa gtgtcgtagc gtgttcgctc gcctggagac
925741 ttgggagctg cgggacgaca ggttaacaga tgaagctcgt ggcaacattc caggcttata
925801 agaagccaga cctgccagtc aacattaaat catgacacag aatttccagg gaactctttc
925861 actcactcct cggacttgtt aaccgggctg caatgttgca tttgccttta ggggtgcgga
925921 actctggcat ataaagaggg tcctctaggt ccagcttcct tttggcctta aaaaaaacaa
925981 catttacatc aattaatagg agtttaaaag gagttgaaaa gggtaaataa agcaccacaa
926041 tgatcaactc cttgaattct gcgaatctat agatttatag tgtctgcagg gatctttcat
926101 ccattcagtt cagataatgg gcagttaaag actgattgat atgaatgagg tgcgtaggac
926161 aagtccggcc cttctgcgga actgtgacct tgctaaagag cagcctggaa taaagtgtcg
926221 taaatgtgcc gacagcccct cagtgtagga acaatggacc tgaatggggg aggctgcgtg
926281 catgcatgcg agagttgatc ttgagggttc ctcaatgacc tctctttccc tttcccacct
926341 ccctttgtgt ttctccccca cacacacaca cactttggtc tacactataa aacaatttgc
926401 gtgattttta tggactgacc taaatatctc cacacacacg taagagtgaa actaaaactg
926461 tcaatgatat ctagttgcac catcatcgtg attccgtcca gtcggtgacc tcatcacctc
926521 ctgtgctgtt gtctgtcatc ccttcatcta ttttccccat cacaaaccat attcaacaac
926581 agcaacactt tctttccctc tccaacactc aaggtaaatg agttcctcca caagaactct
926641 caaatgtgac gagccaacca cgtgcggacg tcgccgtgac gggatggtga aggccgtcat
926701 tgtcccgggg tgactcactg acacgtccac ctgttggccg cgtacatctg gagcggcgtg

926761 gcgagtgtac gtgctgttag tgaggctggt gtaatcttga aggcacacat gctgctgcag
926821 atttattgag acgccttgtg agccctcaac accaggtggc gcccagtgaa accagttaat
926881 agagtgaaag agcatctggc tggagacttt tacaggcttc acatctgaat ggagacccca
926941 gcgtcggtga cctcagcatt cagagcgagc tgcacctgtc tgagcgcagc ggcaacagct
927001 tcatcgtgca gctgtattgt agagccgggg tggtgggggg gtggggggaa atcgctctgc
927061 tgactcgacc acagcatgca aacaacgttc tgtttgttgg acagatgctg cagcctggtt
927121 ttccccccaa tcgtagtcag catttttttt tttttttttt ttttaaatgc ttgagcgaac
927181 aagctacgtg aggaaatggg agaggccggg ggcttaacac actcccagtt gtcgttttat
927241 ctcaaagact cgcagtatcg caccgctcac gtttgcagtg acctcgccct gcctgtcgcc
927301 aacacccggg acccgcacgg ggaacattta tgattgttgt acattctttg gctcgttccc
927361 gccactgcaa tacttacaca cacacacaca cacacacaca cacacacaca gaggcacgca
927421 aaaggcgttc gtagacgaga cctcggctaa gaaacaacat cacattcaaa ggcatgttac
927481 agtatgcctg caaacatctc cgtacaaaac ccactcgcta ccacaccctc catagtctga
927541 ggacaaacac ggcctgcagt gttgccaaga ctgtcgcagg tagaaggtgg tggacatggg
927601 ggagcaacaa gccgctttga aatgcaaaac tgtgctccag gccagcggta aacaagggat
927661 agaatgcata cagttgaccg agtgacaaac gtcacaccag aggcgctcgg agagggacgc
927721 agattcacac aatgcgtctc ccggccacca tgtaaatcag aaagctgatg ccagactgcg
927781 tgtttgatga aaagcgcgga atgcaccccg aataagcagg acgctgtttc agacccgcat
927841 tacgtgcatt cgagtcactg tttaaaatgc caccagctct ggctgtatgg gaaacagaag
927901 acatctttca tctgcggcgg cgccgtcaca gggaggacga tgtggcctgg actcttaacc
927961 ttcaccaaga gggtcgagtt gaccacgaaa ctcgtgagct ggtgatcacc ttcgtaccat
928021 ttttgaataa attctcgtag atcagaaaag cacgtttgtt atcaaaaatt aaaggaagtg
928081 agcagtacac aaaggagcag tacttcctgt ctgtacacac aaacacccct gaaatgctcc
928141 atttgcagca tcagcaaagt gcaaggggaa aaaaaaaaac agtctggctt taagtgcggt
928201 gataaacaga aataataatc tctgattaaa cacacaagat gtatcaatga gtcatttgac
928261 agctagaatg gaagaaggct ttcagaagag gattcagctc ctttagttgc tggggaataa
928321 gacttaactg ggcgttccca cggaatcaat tacttcattt attgtaaact gctgaggaag

928381 tttcacaatt gatgtaactt gtgaaaccag gcctgtgact tcaggcgttt
ttgtaaatga
928441 cagtttttga ctcaccacca ggttctcacc agcactgcta cgtgtaggtg
ttctttgaga
928501 cacctaaccc ccccaccccc acttcctgag atcagtgaat gtcccgtcca
ccttgccatg
928561 ataagctagt tggcagcctg ttcctcactt tgcttacagc taactaatcc
tcttgagggg
928621 cctattagtg aatatagaat cccagggcag ggggtcagtg tgagatttgg
acacaccctg
928681 taacccctaa cccatttagc catattgtat ctcaagtgaa aagaccgcat
ttggaagttc
928741 caaaatagaa ccgtcgcagc cggcgtggag ttctaaacac atgcgctgta
cgacctgatc
928801 aaatgttttg tttatttctg tcccaacggg atgtcagaca gctttaatat
ggcaggaatg
928861 cagcacgtcc accctgctta caggatggtg gtgaacaggt tgcaacgata
acaagtccag
928921 gtccattcca gcctgatttc tgtgcataca gtcgttaaca tacagcacag
aagcaggaca
928981 gtacacatat taatgcccct cagctgccta ctcagcaggc acaccagcta
ccaccccaa
929041 ctgtgagcgt cactcatacc ccctttcttg gcatctgatg ggtctgcaat
attaaccact
929101 tcccagcccg cacacacaca tcacaaaact attgcagaga catggtgacc
aggctccagt
929161 gactccagct gggctgcggg ataaaaggtg tcagaccagt caaaatggct
gtaaacacac
929221 agcagcacgc acatccctgc ggctttgttt gcagatccat aagtggctca
aagggttccg
929281 gacaaacata ccgtgagcta ggattcaacc cgcatgctaa agagtgcccc
catccccaac
929341 atttatgtca aagtgtgtgt gtttgggtct ttaccggcag acgtccggag
gatgctctga
929401 tgggtttagc ctctggaccg tggggggtcg agtacaggct gttggctctt
tgttcggctg
929461 cggtcgtgtt gcagatctgg gtgaagtaac cggccggtac cgcgttcatc
agcgggctgc
929521 acagcccggc actgaagaac tcggtcttga ctccggcgga taggagcttc
attttgtgct
929581 gagagcagga gagacccgct accggccgac ccgacgccgg agagacgcct
ttaggcatcc
929641 gcatcatttt cttgagtaaa cgaaattcaa ccgaacccaa ctaaagcgag
ccaagtgagg
929701 tggctaatat gggctgctaa cgggggctag tcgctactgc taaaatacaa
tgcagtggaa
929761 tctattttaa aatgtaaaat gatagaggag cgaggagaga ttcatacaga
tatcatcctg
929821 ttaaaaccaa cacgtatgca agaaagagga agcattagcg ctgtttgtca
taagttatca
929881 gcagactggt cctttgtcct gcaacaaagt gcaaagtata tacttgcctg
actctaaaaa
929941 atacagtaaa taatagagtg ctaaaggtcc cgcagtctct tttaaaatcc
ggtatcagga

930001 gagttctggg tctcattgag atttacagct ttattgcgtg cctctgatgc ggtatccgac
930061 cgggtctgcg gctccagcgc gtccagctcg gcgctcctcg ctccatacaa gaggagcaga
930121 agaagaagaa cagcttttta gcgccaaact tggtcccgcg aaatttagat ctcccgcggt
930181 tgaagtcggc ctctgattgg ctgccggcac gctcggcttg tcatatttgc ataaaacagt
930241 tgtttgatga cggaaggggt cctcttaaag agacagcggc aacctttgtc ctttcagttt
930301 tatactgcag gagacaatgt gacctctttt tcttcgcgaa tggcattaaa accacttttt
930361 gtgatcctat ttgcatctgc atgcaatgta agcggtcgaa cacacatttt tgtatatgtt
930421 attcaccaat aacaagaaca aaatctattt tggttcctat ttatttgcct gtttaaaaat
930481 acagtaaagg gtgtttagaa atgtaaagtg ttgaataacg tcacaaaata gagtttagca
930541 gtttatttga tatcttttgt ttttgatatc ttaatatata gatgtgtcta tcagtaacct
930601 agggagggga acccccgcaa aagttaaaat ctgtgaaaat ataaagaatg ttgaccaatt
930661 taaacagtat gtgggattac tggatctttt tctttttaat gttacttaag tcttactgca
930721 catgttgaaa gtttactgtg gcctccactg aggcgctaat ggggatagtt tgtagatgat
930781 aattggattc agtctgaaca tactcttgct tattggctca gaggagatat tgaaagaaaa
930841 caacccctcc ccccccacac acaatgcttt cagacccaag ccatatactt tgaccccac
930901 tggccaatgc aaatattccc aacaatcaca ggcctgttgt cagacttcca ggaattcaga
930961 cattgcatag aattcaaata ctgttagaaa ttttaataga acactttttg ctttgtttaa
931021 tttatttcaa atatatcggt tacacacacc aaaggccgga agaaaagatg aaatttgctt
931081 atcaaactag ataaaatcac aacctttaat agtgcagtac aaaaaaaagc cataacacac
931141 atttaattgc aaaccaatgg ttataataga acgataacat agaaacagtt tctttcactg
931201 tcttcaatac cttaaaaaac acaatttctt gtcttgatta ccggtacatc cttgtttttt
931261 tcattattat ccaagcaact gaacatttac tgaaaataaa acacagcaca tgcatccttg
931321 cattatatga acaccaccac tctcataaac cagtctatat actgatcctg tctggattga
931381 ttacacgtca cgacatcatc tgacccggaa attgtttctg acaaactgga gtagatgttt
931441 tttcttgtct agtagttaaa tgatcaggtc aagtttgtgg cgcacactga attccgagcc
931501 aacgatgcca tcttgtggta agaattgaga tgtgcatttt ctctcgggac acacacacac
931561 aaacacacac acacacacac atacagacat acatatataa acacacacac acacacacac

931621 acacacacac acacacacat acacacacac acacacacac atacatacat
acatacatac
931681 atacatgtga taggccagta gggaaaagaa aaatgaaaca aaaagaaaca
aaacaattac
931741 tgtaaaactg aaaaaaagcc tctacaagtt gtttaccaaa tggtatttct
tttggtgcag
931801 ctgactttta agtacggtac tctgaaaata tacggagttg acacaagtaa
tcatcatttt
931861 aacccttctg tggtccagaa tcaacaaaac ctttcttcaa ctttcttata
aatctcgcaa
931921 acccgagaag gaacgttggt gtcaaatcgt gtgcaatatt aaaatataga
aaaatgaacg
931981 taaacgtgtg atgttaaaac aatcagattc atcattttgt gcccagtata
gacgggagtg
932041 gggggggggg gggattcaga aataccgatt ctgaatgcgt taaataatat
gaccactagg
932101 tagcgctata acatcagtag gctctctcac taggatgaac agaagctgaa
attcagatct
932161 tcacattaag attttttaaa ttggggttac tgtgttaaat gaacttctca
ggaactgtat
932221 aacggagttc ttcacagatt taagggattc gggaaaatgg ttctttaaag
gtgccacttt
932281 attaccactg gannnnnnnn nnnnnnnnnn nnnnnnnnnn nnnnnnnnnn
nnnnnnnnnn
932341 nncctgtcgc atctccagct cgccctcatc cagggccgga aagtcgcatc
agcacctctt
932401 cgtttgtgag ggccgtgccg tcggagccct tgtgtttcca atcaaacgga
ctggaggccg
932461 agcggtttca catacagatt cttttatttc attacaagtg aacatacatt
ggaaaacaca
932521 cttcatgata aagagtttat gaaactcacc acacgtctgt agaaataaca
caatccctac
932581 aaacatttac aattaactct agtatagagt tttgattaca tctgggaaaa
ttcaatgtca
932641 ttcaaaaaaa aaggtccaca gaaacttttc ttcacacaca cacacacaca
cacacacaca
932701 cagcagatat gtacacactg atgttccaaa gtctccgaat cattggtaat
cagactaact
932761 gaagggaaga ggtgagatga gttcatccca caaacacact ttttttggaa
agaaaatgtt
932821 agcatttaaa aatgtaccag taatgtacat attgggggtt cagaggagga
gggctgatct
932881 tctgctttgg tgtgctgatt tgtctgtcca tgggccaaag actgagtctc
tgtttccttg
932941 ctcgtccact actgttgatc cttgttcatc taggcaacag tccacttttc
tttttaaaaa
933001 cttttccttt tttctctcct caatccctcc tcttctttca aatacctatc
ccaccttctt
933061 ctgcccctct tgtcctcttg cccggagtga agtggagaca tgtgtttccc
tgcacctgca
933121 tggaagagaa aggaagaaaa gtgaagtcag atctctcccc cctcctttcc
ttcgtcctcc
933181 cctcccttcc ctccccgcag aggagagcag acagactggc gccgagcatg
gaactttcat

933241 taaccccagg cagatagcga agaatccgct acacactcgc tggctctgtc tccagcgtgg
933301 tgaatgtggc cggtaaatga cacttttgaa tcggcgcgtc agtgattttc tcccctctgg
933361 tgcattagaa attccgtgtt tttaaatatt gtctttgatt agtgtcagac gcgggacgca
933421 gatgtttttc ctgccccatg atgcttctgg gcacttccgt catatgatcc cactgatttt
933481 aaagccatgg ctaactacga ccgggaggta tgcttaccga aacatgtcct cctaatggta
933541 caggcgctcc ccttctttgg agaaattgtg ggtgagatct ttagtctgga cgcagagcag
933601 agaggcgcag ttataacact cataagcaca gatgaaatga agactctttc tcaaacaggt
933661 tatccatgca cgcactgtat aaccccatag gacacacacc aattgaatcg atgcttatgt
933721 ctggagacac gaacgcaccg tgcacagtct ctcctcacct ttatagacag aaccatcttc
933781 ggcgcggcag cctcctccgc ctgcagcgcg atctggaggt cgaatatgta gtcgatgacg
933841 tgctgcagga tctccacctg gctcaccgac tcgttctgcg ggatgctggg caccagctgc
933901 ttcagccggg agtagcagtc gttcatatcg tgcagggcgc tcaccggctc ctccagacac
933961 gagtgtttat tccggccgat gaccagactc tgctccgaga tgcaacacac ggccttatag
934021 cagctcctcg ccgagcggac gggactgatg gctttcatgg cggaaaagaa aacacgagaa
934081 gcgagcaacg acgctgaata tcgggcctgg ctgtgagttt agtggtgctt cgcgtattct
934141 agcggactcc agtaaagctc ctgaaacgct acagtgtcct gccgcagacg tacagttagg
934201 gttatatacg cggaaactca cgtccacgcc caccttttcg catcgcgttc tgattggctg
934261 tcgggaagag ttcgccgctt ctcattggct gtcatcacct tggaaagaat gaggaaatta
934321 agacgagttg ccttgaaggg agtgtcaatc gattaattac ctctcaggtg gcggcgttta
934381 tttgaaatgg aaaaagtcag tgagacgttg atgaaattaa gctgttaaat gtcacattca
934441 agctcacacc tggcgactct gataacagaa atttgcgtaa atttgactaa aacttctatt
934501 tttgtttaaa ctaacagctg ctgttgaggt gaatgcagct ggtgccactt tggtctaaaa
934561 tacagagaga acattcgcag ttataaactt tctaataata ataaagggct gcggttctgg
934621 cagttcttta gaatacacgt aacacatttt aaatgggata ttgtaaatgt gttgattccc
934681 cttagaataa tgtgtcatat aggtaaaagg tcagccatct acgttatctt aatgattttc
934741 tataatgtgg aacggattat tgatcactat aaagtgagcc atgtcaagct tctaaaggtt
934801 tgatttaagt cttcaggcct ataaaagaat taatgtttca atttgtacag cgtattaaaa

934861 gttgcatgtg aaggctcgtg ttcaaaccgg accgcttttg ggttccatgg tcaaaggaaa
934921 ttacatttca acaatcccaa atgttgttcc ctttcagttt acatcaaagc acgtcccgtt
934981 ctgctcctat ttgggtctta ttttcccctt ttctcacaat cctgttttgt ggaggaatgc
935041 aggcctgagc cagctgtgtg tcctatccca gcccagctgt gttgatctaa cgcgccagtc
935101 ccagacatgc tggcgccagc ctcccgacgt catccattca ccccgacgcg cactcccctc
935161 tttggcagac agcccgccct cccccgtcca ccagcagagg ggagagctgc ggagacgctc
935221 ggagatctga ttggcggatc ggttctgtca ctcaacaacg tggcctattc cctcctcccc
935281 tgcgtggtta gaaaaaataa aggaaaacct ttctaaatac ccggtaacgc gcatgatgag
935341 cacggaaaca tgagcaggac aactcatgac aggaaaaaca tcagggaaac tatctcactg
935401 cctattaacg gcaaacaagt ggctatttat gaaaacgcaa acgaccaaat acttcacact
935461 gttcgctaaa ttagatcggt ttaattaata tatttatctt tcaaaatgga ttaaacgaac
935521 acctgagtgc tatacagttt tacaaactct ggttgaattg gacatacagt aatatccaag
935581 atcccagggt gatgcgctga acgcgtccgt gcgtaaatac gcacaggctt ctacccatca
935641 tgtagaggtt caccagatgt tccaatcact ggactctctt gacatcccac cctcccacag
935701 tcaatgaccc ggtattacaa tgtcactcag cttaggggaa accacgatac tgaaccgtga
935761 gacggaactt ctgctctaat gttgcgcttc tctgcgggag cgatccaaag tctccaaaca
935821 gaactttgta cttaaatatc tttagtggag ggtttaatgc gaagacaaat attcaatttt
935881 cagctatgtt gggaatacaa tagtatctat ttgctgactg agcaaactga ttaaactgtt
935941 aaatagatta gagatgtcga ggcctccccg cagaactgac gtcatcagac gatctaatca
936001 cccgcggagg tttttagaca gttttaggat ctaagaggta tttcaaggat ataaaacaca
936061 ttcactccct ttctgtagat tccagagcat acatctgccc ggcaaacgcg tgtgagcgtg
936121 tgtgtacgtg tgcgtgcgtg tgtgtgtctg ggtagggatc aggtgcttgt cggaaaggac
936181 ccggacacaa tcgggtctct ctttcaatgg atgggagaat tccgagcctg gcgtcagtga
936241 gtctgcctct ctccccgccc tccctctccc tctccccgct ccaccgtgca gctggacgca
936301 gacaatcaag ccatcaacaa tcgccatgca taattaccag cgcctcctct ccagttatct
936361 ctctcaatta aatttaaacc cctccacatc ctattgacgg aactctttaa gaaaaaatct
936421 ctagtgtgcc caaatctcta gtgtgtgtat gtttgtatac ctgtaagatc ccccсttccc

936481 cctccacatt catctttaac ctctcaaagc aaagtgtctc ttttttactc
cacaacagag
936541 gagagtgctg agtaatgagg gtcatcttgt gagttgagat gggaggaggg
agggtgggag
936601 gtgttggggg ggcacccacc ataatgtcat gaggagaggc agtgttgtat
acagaggggt
936661 atctttttca tccacaagtg cgggcctatc ttgggggagg gagaataatt
aagtggcaag
936721 aaacatactg aaacttaatt ctttgtatcc ctgtatatgt gtgtgtgtgt
gtgcatgtgt
936781 gtgttttgct agattcctct gctcctcctc tgctccctgt tttcaccctg
gccttcgtca
936841 aagacgccac agtcttcctc tcggctttga acagcccaca gttcccagcc
ctttgtcccc
936901 cggtgacccg gccagtgaca tggcatttta tgcacattgt tgattcgtac
tggtcgctgg
936961 tgtcgcatgt gtgagtctct tgttccagct tgtgcgtgat gaccctgttt
ggttatttcg
937021 cgcgactgtt ctgaatgcat taaggatgcg ccgcgctgat gttcgtcaat
tcattcactg
937081 cacataattc gcgtctcaag acaaattatg taaatcaacg aggaggcgtg
gatggatgat
937141 ttccatcgca gcagagtgca ggaaccattg acctgatgct tgtttggctg
caggagctgt
937201 tgggtttctt cctccctcta cggtctctct tctgtgaggt ccacaggccc
aggaacttta
937261 ccgacgacct tctctttcag gtgccggcaa gcatggaaag ccgaatttaa
aatatcagca
937321 catctggctg ttgtgtgacg tgggaatctc gctgttgtcg ctccgatgtt
gtcaggtggc
937381 cccgtttctg tccttggagt acaaatattg catagtccca aaacaaaaca
aaaaaaagca
937441 gagggcccat ggaggtctgg caggtggaca ccagtgactg aaggccgttg
agcccatggc
937501 cgccgccaac cgcagcaaaa acaagccgcc tgtgaggctc atcgtgcctg
aacgggccga
937561 gacagtgcca gctgtgggtc gcacgattgt tttggggctt tcactgtccc
tgactggaat
937621 ggcccatttg tcctgcggta ccttttaggc catttcattc ctcactgtcc
cctggatgaa
937681 ggacaggcgg tcagacttgg gatgatccac agcaaagccg ctctggcagt
tgaccattaa
937741 tggaaacagc cgaggctccg gcatttcttc ttaaagcgtt cctgctttta
aacctgctaa
937801 taaacagcag aatgagtgtg ttcaaatgcc gcggagactc tgcgagttgg
gaagagtcca
937861 attcaaatct gctctttact gtagctgaga cgcatttgtt actattaatc
gatccaaaat
937921 atgatcttga tactaaaaca taccctgata tgctgctgtg tgctgcatat
tcaagctgct
937981 tatacactgt gatgggcttt tggtatcagg tgcttccttt ccctggtgca
aataagtggt
938041 tgtcatagtg atgccatgag aaactgctgt aaagcaacag acacattgac
tgatgacagt

938101 cacaagtgaa caccaaagaa cagagtcgtc cctcattttt ggactgtcgc
tgttgcttct
938161 ttttttttcc aggagtgaga agagacttgt ctgctctctt ttgcaaaatg
ctaatgctag
938221 catgactgag cgtatagaag gaattaaatc agctttgctt tgtttgatca
gatatttgct
938281 agcatgccgt gaaagtaaat tggttatagt tatataatag ctccagttta
caataaatca
938341 gaccatcaca aggtggtata aaaatggatg gactggttct gagctgctag
tgccagtgtg
938401 tgtgtgtgtg tgtgtgtgtg tgtgtgtgtg tgtgtgtggg gggggggggg
tgcaaactgg
938461 aaaatgtgca cagagagtcc catgaacaga acaaagtgtg cagaacgtca
ggacagaagg
938521 agggaaaaac gatggcgaga gaaggaaagt cagagacacg atccgctgct
gcaggggttc
938581 agctggtcag gcttcacaga gacacacaaa caaaggccat caggtccctg
gtgggtatgt
938641 ctgcacctgt gtgtgtgtgt gtgtgtgtgt gtgtgtgtgt gtgtgtgtgt
gtgtgtgatg
938701 tatttctagg tcaagtgtgt cccagtgtca tctgaaatct aagatgagat
gcagcattga
938761 tttttttcctt cagtcactga catcnnnnnn nnnnnnnnnn nnnnnnnnnn
nnnnnnnnnn
938821 nnnnnnnnnn nnnncacttt tcctttctta catcctcccc ccaccccata
cacaccctca
938881 cgggtgcatg agcactcaca cacaaacacg cgcgcacact taaatgtgca
cacacttgcc
938941 actcctcctc atctcttgtc tcctccttga gctactaatg aattaaaaca
gtggggaatg
939001 agacgcagca cacagctgat tcccactgtt tccgcacaca cacacgcaca
cacacacaca
939061 cacacacaca cacactcaga cacacacaca cactcagaca cacacagcct
tccctaatct
939121 gggtcagcgg tgcccctctt cggtcagccc tggcggagcc ctccagacgg
aggggcagag
939181 tgggagcaga gaggtctgga ctctcggtga cctgattaaa aggctcttac
aaagacctct
939241 gagcactgta acacagacga gacacacacc agagcacctc tcgctccgtc
tgaaaacgct
939301 acatttcatc ttatgcttcg atatccatta aaaatgatta ataccatctt
gcaagccatt
939361 tgaggttctt ctttcacccc gagttgcatc aatctccccc aaaatatcaa
caacctcttc
939421 catcgagggg ctttaatcct ggatttatga catgtccaac atcatgaggg
gatggaggga
939481 aatggaacag tctgccagct ggtcccgtgt gcacatgatc gggttcaaat
ttgtaatggt
939541 tcagtgtgtt gcctcatggt tttctgtttg tttgaattgg tgttctggtt
gcagatcacc
939601 tacggtttaa acagcatgcc tttgtgacac acgctacatc aaattcaatc
ttgcgccact
939661 aattatgaag caaaactaaa agctgatcta atacaactgt gactgaccct
taaagagtaa

939721 gaccagcagg aaatgtccat atctgtgaag ccccatctgc tgtgcgtgtt
atatcatgga
939781 tatttcctgc taacattctc cggctactct gtaatttact gaaaatactc
tcacagcact
939841 aagaatctgg ccaaagttaa gggtcagaac cactcaagac tgctaaaaat
gtcacttcct
939901 aagaatctca gaggaaggga ccaacatggc ttcctttccc agacataagt
gaggaaatag
939961 atttttatga gaaaaagagg gagagaaaat gtctggaatt attacttatg
atacaaatag
940021 ctaaaaatac tatttgtttc tgtcatagca tgcaaaaaca tgtggtctcg
cttgacaagt
940081 tagcaagatt agaatcaggg acaaggcttc actcctgagg ctcagaactt
ttatttagat
940141 taaaactgta ggaactgatt ttatattaga cattaagtat ttctaatggg
aaaatgaaga
940201 gttgtggtag ccaggcagct tgtgagctta aaaatagagt gtgttcagct
gcagttccaa
940261 ggttttcagg gagaaatgga cggtgctgga acaggcgcaa agggaaaata
aatatagaag
940321 gcagagatca gccgaaacct tgttaaaatg gcagcaggta gtgtaaggta
ctgcttcaga
940381 cattagcagg actgctagca gtaacaatgc tgacatctaa gacgcggcac
gttgtcaaaa
940441 atgcccattt tcggggtcaa atcgatcatt tatttgacag agttgtcttt
accccttaat
940501 aaacagacat tatcaggata ggcaggattc cactgaaaca gcgtagtcag
agacagagac
940561 cagtcaggct gacgacctca atacggtgcg ctcagataaa gacaagcaca
tcaaacaccg
940621 ctgataacgg cggcagtgaa ggacacaggg cagctcctct gggcaaaaca
agagatatgc
940681 ctgacaaagc ccggtttata agaagtggga attgctgtta aacagacaca
aatcaccaag
940741 tctttgtgtc cacaaaaagc cgcgaaggtg aagccacact aaggaggagg
aggaggagga
940801 ttaaaggcca gacgggagat cacctttatc tgatcattgt taagagctaa
cggtggcgtt
940861 ctggggctga atgggactct ttctgatgtg acagacgagc tctgacggtg
tcaggggtca
940921 atcaaatttg cgtgctcgtg cacgtcgagg cagccccata atgactgttt
aacaacctga
940981 gccgggctca cctgtgtcaa cgtgttaagt gactcagtta attaatgtcc
cgcggacctc
941041 ctgaaggcct cggagcgatg ggatcaaccc ggagagctgc tcttttcctt
ccacatgcgg
941101 gactaattgg tgtcaagaat caagccgcca tcttgtagtt taactcttgc
agataagcat
941161 ctgtgccgtt atggggctca ttcggcgtaa tgctcataaa taagttgctg
cgtccaggcc
941221 agatgtgcgc ccccttctcg tcagagcggc gccctcccct accaacaaca
acccagtgtt
941281 gggctaacct cctggtgact ttaccacagc atctggcaaa tgttaggctt
cacctgaacc

941341 cggcgtttac gctgtaatcg gagcggcgtg cttacagatg tgccctcagc aggcgaggcc
941401 tgcgcatgcg tgcacggccc cgcgcctgcg tgtgtgatca tgtatgtaat cgcgcatctg
941461 cgcagcttta aaaagacaat ccggttcagg atgggaacca gaggagccgg tgcaggcaca
941521 gcaggggagc cgcctttcac ggcgcacaca ataaccaagc aggcccaggg aacacggtgc
941581 ctggctgcct tgtgagagct cagcagccac ggcgtgccac gcacgtggtc gggacgggtc
941641 tatgtgtccc tttgttgatg gcggctgaag tcgaaagggg aggtgggggg ggggcaaaaa
941701 cagacaagac ctgtaagaca ataataccac actccccgca cagctggcat attttctgag
941761 aagccgtttc ctgtcaaaat tcttacacag gaataatgag ggctttgatt gagccccagt
941821 taacggaagg cacttcctgt tcctttgtat tggtttcatt gtgagggagc gtggggcgag
941881 gcagggtttg agggaaacag ctgttggaga tgacacagtc aagaacatcc ctgtgacccc
941941 ctccctatca cccccacaca cacactcaca cacacaccct tagagataaa cctgtgagcg
942001 ttaagctgat ttcactaccg aatcaactca caacctcatg attctgctta aatgattcgt
942061 tagcaaaagt tcggtttttt ttcggctctg gcaccttgag tcaccacggc aacacggtgg
942121 gtctccagtc atagccaact taaatcaaca cggcttagaa gaaaggcgat tcaaactgaa
942181 agtcagagaa tactcctcag tgtgccagga acacctgaag ggatatttat gagcagcgcc
942241 gtgcagcctt tgcccaactt cctgtcgcgc tcttctacag tttagctcaa gctgtgcaca
942301 ttttccaacg ctgtttttgt gtgtggctgt aatctaagat tgaacaaata acgcgatgaa
942361 attctgaaaa atcaaaagag atgagcggct cttgtcgaca gctgggacaa taaaagctca
942421 aattctgggg ggggggcggg ggacaaagac taaaaaacag tggcacgtta aagttgttct
942481 cattggttag ctgtttgcag atttctgagg gaagtcacac gtcttatctg tggaggaaag
942541 tctgcggttc tcgagctaat tgctgcagat gcagatgtgc gctgcactta tctgtcggga
942601 cgagtgcacg ctggtttgtg aaggcttgaa taattgtaag tttagatgct tacaactgac
942661 attcaggtat tcaaatgcgc gcgcgcgcac acacacacac acacacacac atacacacct
942721 ctgtgcaagt atttgagtga ggttttccat tagtgttttt gcagagatca cagttgtttt
942781 gtttcctgcc gcgcagtgtt tcgccacaga ggctaaccag tgctgctcgc gctctctctg
942841 ctgtgcatca gagacgattc gctggaaagc gatgagcacg ctcgcgcacg cgggcgcatg
942901 cacaatgcta ctattataag cgtctaacta taaaggagaa actatcaaaa acactgattt

942961 ccatcggatt taaaaggaag tctgtcctcc ccctccattc aaaacaaata
tccctcccat
943021 gagagctttg tgttttgggt caaaggtcat agataccatc tccctgtatg
ggaacatcca
943081 tcgttgccac cttcctcagc tggctccatc ggtttttccc actcagccct
gcatgttatc
943141 aatgcggggg cctgtattcg ctcctccagc cccctcctgt tcctgcttaa
aggacatatt
943201 gatcagcagt aaacactgat agacccttga aggagttgca gtgtgagtgc
gtctgcgcag
943261 cagcaggggt cacactgtga ttttaggctg catgccgggg ctaattgaac
tcaggggtcg
943321 ctaacatgca tcgctgaccg cgtgcgttag ccaacttagc atatcttaag
ctactgataa
943381 gatcaaatgt gtgtttgcgc aatttaaagc aaaactaaca cactaaaaag
ccgtaaaata
943441 agtagaggaa agatgagggt taagtgttat tgcaggtttg gcaatttgct
gcaataaaaa
943501 tcagccaagc atgccggttc tgtttattgg agaaatgttt gattggccag
ctgttatggt
943561 tgagttttat gcaaaggagg tttagatgtg aatggatgtg aagctgtagc
tctgcagctt
943621 attgtgctac aggaacacaa accaggaaga ctcgccgacc atccagggcg
attctccgct
943681 attttcctcc acagagatgt gagaaacaag tgcataaaat ggtcaactat
taaagcgaaa
943741 cagcacaata aagcgattat caagaagaag cagctatttt gcatttcctg
tgagtcttat
943801 aaagcatgaa aacaaaggca gagcttacat tttctccttg tcgcatttga
caaatgaagt
943861 ggataaaaga ttttgaattc ttttgtatct attttaatca ttgctaacat
tttagaaaac
943921 agtgccattt catgtttttc atgctaaaaa agtgttccgt aaggaatcag
aacataagac
943981 gtgatctgct ctgtgcaccg tggcaacggt agcattgttt tccttcaagg
tttaaaattc
944041 tatcaatctg ctgactttct aaaatggatg tgatgctcag ctattcacat
aaatagataa
944101 ataaggacat tgtaatgaga ataaaaaagc atgaaagtgg aggaaacctt
tcacaataaa
944161 agtgtgtttg cagtcgttgg cagtgatcgc gaacgtgttg tctcagctac
attttgggac
944221 ggcggacatt aaatcatgtg ataaaattac aaattcggaa gaaaaaaaga
cgagaaaggt
944281 tttttttct gttcatacgt cattcagttc aaaagaataa aagaaaacta
ccaggtacca
944341 gtctgctgtg ttggtgttaa cacatgtttt cctgcctgcg tcatcatgtg
ataacacaac
944401 tgaatcagtg tctctctgtg tgtgtgtgtg tgtgtgtgtg tgtgtgttta
cggtattgct
944461 ggactttaat aaatgtgtgc ggacagtgag tttaacgcca ttgctttctg
tgacccgacc
944521 tttgacctgg tcatctgacc ttcctgttac cgcttcttgg tcacccagct
gtaaaattgg

944581 ctgcgtgcgt gtgcacgagc acatctgtgc tgtctctaca tccccagaac
ggctctgcac
944641 gtttcaaaac tgtccgtcct catcgtgtgc gttccatgtg cggtttcctt
gaatctttgt
944701 ttgcacacgc ttgaaaaaaa aaaaaaatct gctctgttga aatctcatct
ccactgctcc
944761 attaggacgt tcccagattc cttgccccct ctgcagaaag aagcggagaa
ctgcagccag
944821 ctgtggaatt acaggcagca gcgatgaaaa ccgagcaatg ctgctaaaaa
gacgtttagc
944881 aacagaagaa cctcgaagtt cttggatcct ttcctttgtt taaacttcag
gcaacataaa
944941 aggaagacaa aagaacatcc cagcttacag agacacatgt tttctgagag
ccaggggctg
945001 aataaagagg acacaaaccc accaaaacgc accagactct atggacacat
cagaaacaca
945061 ctcgtcaggg acggatcaca gcgcgtatgg agacgcggag gggacggaga
gattattgca
945121 gacttgtggg aacgggatta agaactatga cctgcgaggc aggagcctgg
aggacaccaa
945181 tcaaaagcca cgcaggaacg gtggaaggaa attggtgaaa tcaaaagctc
agacttcaat
945241 ccaggagaat caggggccgt ctgacctcac cgtgacccga caggagacac
tcaccggcca
945301 ctttattagg cacagcttgc tagtcaaaag ttggaccccc ttttcagaac
tctcagaatt
945361 cttcatctca aatgttcaac gaggtgttgg aaacattcct cagaggtttt
ggtccatagt
945421 gacatggtgg cttcaggcag ctgctgcaca tctatgatgc gactctctgg
ttccaccacc
945481 gatctgagct ttgtgacacg gtgccttatc ctgctagaag cagccagctg
gaatcagtcc
945541 cctagaccct agaccctaga ccctaagccc taaaccctag accctagacc
ctgatgtcca
945601 gttgtagtga gcctgtgtga cctgtagcct caccttcctg ttcttagctg
ccaggagtgg
945661 caccgctgtg gtcttctgag tgttcagaga tgctattctt cattcctggg
ttgtaccgag
945721 tggttgtttg agctacagtt gagctagaga tggttgtgca tgaagatccc
agcagatcag
945781 cagtttctga actactcaga cgagcccgtc tggcagcaac aacaggccaa
agtcccttaa
945841 accccttttaa aatcctcttt cttcccatcc tgttgctggt ttgaacttca
gcaggtcgtc
945901 tcgaccacgt ttaggtgtat aaaagcattt agaggcggcc gtgggattgg
ctgattggtt
945961 gctggcgcta ataagcattt gaacaggtag acctaataac gtgtataatt
aaaaacgtgt
946021 gtgtggtggg gggtcaaaaa tgtgggtgtt gtagtgggag agagcaaaaa
agaaagcagc
946081 aaccttcgac aacaacctcc cttcagcctc ctcgtgctgc tcgacaatca
acatttctca
946141 atgggctgat gctaattcgc cacgttcaaa ccttcacgct cccctaaatc
tgtttccgtc

946201 ctcctgtttg aaccgatcat gtccccagta cagcacacag ggccccctt tcgtctcctc
946261 ccatccctct ttcctctatc tctctctttc tctctctccc ccctgctagg atccccacag
946321 ctgcgcggac caggtgcgtt ccgccccaac acacacacac acacacacac acacacacac
946381 acacacacac acacacacac acacacacac acactcacac ccgccaacag acgcagcagg
946441 aaatgcctaa taccagtttt agaatgatgc cactatgcca gcctgacggg gcggctgcag
946501 gatctgcaaa agtgggagaa aaccagactt taagagtgga tgggaatcaa aaagatcacg
946561 tttaatagtt gaaaataagt ttctttgggg gcggctgaaa gcctgcaact cactctaaca
946621 atcctgaagt gcgaggggcc gccatgcaca tgttcgcctg tgagtgtggg tggatgcaca
946681 tgtgtctttt ttaaaaaaaa gtaccagtta ttttggtctg tggttgaccg caactcacac
946741 tctcccctgt cttgtactca tctcgcacgg ccccaagaga aaggggaaag agagcaagag
946801 acagagcagt ggatgagcaa gacgacgaca gaaaagtaaa agcagaagcc tgagatgaag
946861 atttacaaag tctggggaag gagcagtgac tcagtctgct ctgttagccg tggacggcta
946921 cggttctgta cgcacgtgca caaaatccaa tgcacggcaa aatccctgcc tattcttcac
946981 acgcgtctaa atataactga taatcccaca aaaccgactt tccgaagcga atccttcaac
947041 atttcagagg aaatcttctc aattaaaaaa ccctccaatt tcctctgata atgtaatgta
947101 atcatgttgt cctaatgtaa tcctctgttg tgtagtttgg gcgccgagca cgtgcgttcg
947161 cccccgtcat gtcagcagat gtgcagcacg ccgtcacgtc gcccggcagc acgagcaacg
947221 atgcgaaaga ggaacttcca gttgtcacca tctcccatga gcagttgagg ctttggcacg
947281 gccgaggtga gaaaaccaag caccccttta gactctttaa tgaattactt cgcttttaat
947341 ttaaaggaag gcgcattagc ataacccccca cgagaccgat atggctgcag ctaacccaga
947401 actttgttgt ggtctgagga gaaaaatggg acgctctggc ctcattgtca tggaaatgtc
947461 acctctgtac acgcacacat tagcgtgtgc acacactgtc gcaaggatca cacacccagc
947521 tgacacccta gtgtggccac accacattct cacctcaaac ctttccactt gctgactctc
947581 aggccagcgt ttccaccggc ggatgttttt tgaggccact gattctttga aaagatgttg
947641 ggcgcctcag acctacttca gctatttcag atcaggtctt caggacctca gggtgagcgc
947701 acgtgcgtgt ctgagacgag tgtggtccac ttcctcgaac acacagagag atcgacacga
947761 ctgacacgga ttcctcttcc tcgtgcggta ccggggcgtc agatctgcgc tcagacggag

947821 cacacacact ccaacaggaa gagtgtgggt gtttgggagc agctgcatgg agaagcatca
947881 cacccggctt cctccctctc tcctctcatc tgctatcagc cctcccttca tcagctcatt
947941 ccctccctaa caggaccact gagactccgc cgtggccttt ttggttccag atgtagcgac
948001 ggaaaccgcg ccaacgtgtc ctggctctgt ctcttgtgac acaccagcgc atcagcacct
948061 gcgttgcaca aacctcccgt gccctaattt ggccgtcacg cctgcacgag gtgaggcccc
948121 gatgactcaa tgcgcagttg gacggagcgg ttaaacaccg atctgctgca ccctaaggtg
948181 aggtgcagca tctttcattc ctctgcaaac atacttgtcg cggcttgatt agaaaagcca
948241 ggccatagcg gaggtataac tgggtaagcc gccgtagacg gtgcacatga acccagcatc
948301 tttcactttc ctctttcact ttcctctatc accggctatc acaggcttct actgtgatcg
948361 gtattttggt tttttaaccc tgccctgtca gcagcaacac tgatggttag caggctgcag
948421 acagtcagca gttatttctg tgtttcctgt ctgtcacgcg gtactagcta gaactagcta
948481 gcacggctga caacacactg gctcccaggt gcacagcgtc acagtgtggg ccctcggcca
948541 gagtgcttga gggccattta gggagccaac taggccctgt ggcatgtgct gccctgtgcc
948601 ctcctctgtt aacccattgc tatccccaat tgaaaggaga gacaggcttg tgacatttgt
948661 gctttgggaa aaaaatatca catgtctgtt tatgggcatg tgtgagcttg ctttcttttc
948721 aaggtacaac taattgacct gtttttttg ttttttgata aatggacaaa tctcgacacg
948781 tcattaacag ctgcctgaca ggagcatccc tgcccataga acaacacgcc tactcttcaa
948841 cgtccgtgat cttttagcgg tggtaacata ttagcgccat cgtcgctgga taaaattgct
948901 ccatggcctg ttgaggagga gcaggtctga ggtcagcccg aagaagacct tcagtgaaac
948961 ctgcaagcag gtttgccctc ttcccccgga ctcggccctg ggccgcagat gcactgaccc
949021 acacaaggcc acgcttctct gggtagcaca gaggggctgc tgtgctccgg ttcctggagc
949081 cctgacgctc tgattgagac cctgccgccc atgaaggaac cagagggagg ggaagggagg
949141 ggatgtaaac acatctggca tccgcgaaac cctgcagact ctcataacat cacgggagga
949201 acgcacaggc cgtgtagaag agagagcgct gtgttcctct cctgccatcg cacatgcagc
949261 ggaagacaca cacacccacg caattgtgcg gctgctacct ctgccatgtc gaaaagtgat
949321 tatgtgggga cccgtaagaa ggctcaacga ttctccagct ggtgggacat cagcatgctc
949381 gccgccattg ttctatttat accacctgac aaacaagccc atgttggtgc tgagaggcca

949441 ttatgcagca gcagaggaag cgcttaaaac aatgccgtta ttgcagatgt
gtgcaaacag
949501 acaggggggа catacatcac gccgaccccc ctgcgtgggt tcacctgtct
tctgtggcgc
949561 atttctgcgt tttgcgtggg cgccggctgc tcgcattagg cgtctctaca
ggacggaggc
949621 ggcgatggtt gggtggcgcg ttcgcgtctg cgtgggggtg agagtgcaga
tgttttgaag
949681 tttaatgcag ctttgactgt gggggatgag gagtgcaggt ttgtgcacgg
caaagtaggg
949741 caatcgctcc cgtcttgatg gcgactgagt ctgttccaga gaggcctgga
agcaagcgga
949801 gagtgctgtg ctctttgtgg ccagctgtgg gaccattaga ggcccatctg
cacacaatag
949861 cctctgtctg aagggcgtcc acagcctggc tccccgtcct tcctcaccgt
gtctgtgcac
949921 ggaggccggt accacgcatt tccatgcccc attttgtctc atcgcagact
tctacacgcc
949981 agtcatcatt atctgcctcc tttcagcagt tccgatgcca tgtctgcctt
tctcgtccct
950041 gcaaaacaaa cagaatgacg ccgctcctct cgttgccatc ggatacggca
gaaactgaat
950101 ttaccccggt gttaaagctc ccctgtcacc tctcttttta aagctgagga
aatgggtacc
950161 gattggatca aaaggttatc tgccctcgaa cgctcgataa gacacaactg
catgcaaaaa
950221 taaaggcgga tgcctgaaaa cagcgagctg gtccaaccac atgccactcc
acatttgatt
950281 gagcctcaca cgtcctccct ctctctctct cacacacaca cacacatgca
gggttgcagc
950341 atgcaggcac atgcaaaaaa aatatctgtc ccaatactaa aataagccct
cagaggttgc
950401 acctgcacct gtgccctggt ccacacctac acacaggtgt caggatgtga
cacacgcaca
950461 cgcaaagata gcagagggag ggggaaaaac aagagcgggg tgaatcacac
aactgcggtc
950521 gaccaaaaag ccttctcttg atattatcgc tgtgttttgg agtgcaatga
tgggtgtgat
950581 tttcattttt agacaggcct taatcagaag tactacacac ttaaataatt
agctttacat
950641 accgtgcggg tgtgtgacgc atcattggat tttgaacatt aaacgttcgt
acaagtgaat
950701 ggttcaaata cctcctgctc tgtatcagcg tatcacaatg agtcacacag
ggttttcctc
950761 tggtaacctc atcactgggg cagaaattta ccattcatta taacacgcat
gcttgcatga
950821 cacacattgg acatgacaga tgcattgcaa caggaaggat gtgagtttta
tagtttgaga
950881 tgaagacagt ggggtcacat ttatttcata attgtgctgc tgtcaaagtc
agaaatatgt
950941 caaagtttga caaatatggc tattttatta atcaggcttt cagaattgga
aattcgctgg
951001 tagttggtaa acatcgccac ctggtggacg ctgagcgacg tgtcgtgacg
tcactatagc

951061 ttctgtttca aattaatttc ttgcccatta aaatcctttt atgcgcattt cgttttcccc
951121 acataaaata ccttcccccc tatcttacta tttgccaaaa aaagtattag tgatcaatca
951181 aaagtgttca gcgtagagac agaacttgaa gtgtatgaga caaaacctga acccgttgtt
951241 ccaccgtctc tttttcacca ctctgtgtca atgttcatcc tccctgtctt caataggaca
951301 agagatgccg gtgcccctga gagcacagta gcccttggga acctttttca ggtactgctg
951361 atgatggtcc tcggcatagt agaagtgctg cccctccagg atctccgtgg taatgcggcc
951421 gtggccttct ttatccaact cctagtgaaa gaagagcaga atatgcgttt atgtcaaagg
951481 aacagaaaac agtagtgaaa atgtttatgg tgtttagtag gcaccacttt ctccctttta
951541 accaaccacc acttgttttt ctgcagcgga tgtgaacttt cagtggctgc aatgttttgc
951601 gatgcagtgc aacaacaaat ttctgctatt taaggagcac acaggtgatt gattgacaga
951661 agaaaagagc ttttcctctt ttttctgtag tttagccccg ttgctctctg tgggacgagg
951721 catcaaagcg gatccgatct ctttgtgaaa tcctcacttg tgcactctcg agagacgatt
951781 tcaggcgctg gaaaaccaaa acaaacactc attgactcta aacaacactg aagaatacct
951841 gctgtgcagg aggggttttc tctgtctaat aatacacctc cagttgtctg tagacctgtg
951901 cgactgagag aaaaccacag cttgatttgt tctgggtaaa ccttcatgtg gattttcaaa
951961 ggtcacatca aggtgttgaa ctcagaggag agggaacacg agaaggttcc ccagcatcca
952021 gatcacctcc attagttccc ttattgtctt acagtgtgtt attttcctgg actctgatat
952081 agacgctaaa ttagctctat gctaaccaca tttattggct gttttttttt aaaattgtct
952141 tgtgcagctg gatttaggat tttggagcct tgtgattgac ttcatgtgga atttaggatg
952201 tggatattta aatacagaag aataaaataa acacataaga acagcatcat ctgtttttct
952261 acagttaaac tataatgcat gttacattcc aaaaatatta gactcatggt gggtaaaaat
952321 attagactca tggtgggtaa aaatattaga ctcatggtgg gtaaaaatat tagactcatg
952381 gtgggtaaaa tattagactc atggtgggtg agcccttgta aaacggacat gaactggcct
952441 atttttcttc tatctggcaa cacaagaaag gttccagctt tcctttttgg tctcctggtt
952501 tcttcagaaa cagaacatca cctttatgac tctgctcctt aaccaaatat taaacattac
952561 atttgtacaa ccttggaact gatccgcccg tggaagtcct ggctgcgcac gttggggttg
952621 catgtgcttg attttacaag atctggcttg ataaaagagg ctgtgcagtt tctttctgct

952681 gttgaaataa aaaggttcac tatgcatggt aatataataa tgtccacggg gcggcggtgt
952741 ggtaccggct ctgtgcgtgc ttgtgtgtgt gtgtgcgtgt gtactggagg taatatggag
952801 gagtcagtaa atcaaagcac tctgctgaca gtgaggctga ggcagcagct gacctccgag
952861 tcagtagccg ccgccgccat cgctacaaag atggctgcag tcaccctaca gaggattctg
952921 aagtccacaa accgagctac agcgggacgg cgtcacctca agactggctg ccgtgctccc
952981 tgtctaaaat caatgtaaac catctgacac aagcacggcg ctttgcacgc cctggcctaa
953041 ttaatcaatc atcgtctccg cgccactgta ggagcgaacc ctccggggct ccttctgcga
953101 ggaggacaca atgtccgagg aaacagtctg ctctctgggc aaacacaccc cacccgtcct
953161 gatgcctacc gatgactccc ccatgctcca ttcagctctg taaggcagtc acaacatgga
953221 ggccatttta gatctataaa tcagaatgca tggcgtcacg ccactctgta taaccttaca
953281 tggctttgct ctctctcttt ccctctctct cccacgctct ctcgctccct ctctctcact
953341 gcagcaccgg cagaggtata gagaaggaac acggaaggta aaacaccaac aatggaggga
953401 ggacaattga tccatccacc ccccagctga ccgttttgac cattttgatt gtttaggcga
953461 agccgcccgc tgggagacga gcacgtgcat cagcgtttgt gcgcaatgtt gctggagtga
953521 gacgcacacg tgaaacacgg tcgtgccagc gtgtctttat accacgacac tgggacccaa
953581 caagctaaaa cttgatgaga aagatgttgc tttggattac aaatagtgct tcagcgtaac
953641 atcaggtgaa cacacgccag gtcctcccca gtgacacaaa acatgcgcat gtgtctgcgt
953701 gtgtgcgtgt gtgcgtgcgt gtgcgtgcac gcgtgttaag gtctgagctc actcagccca
953761 gtcagccctt ccttccttca cgcctgtcgg aatgactccc atgtcaaaga cggggctgag
953821 cgggtgcagc tctctcctcc ttttggactc tctctcggtc tctgtccatc accgaccctc
953881 gctctccctc gccggctccc atgtgagcac agaccactgc tccgcactcc cgagtcctcc
953941 ctgctgtcca gccaagcgag agagagagag agagagagag agaagggggg agcgagagag
954001 accctctcgc catggccgct gccagagaga cggccccggg acgcaaatag gacgtgacac
954061 cccgagagag ggtggaggtg gaggagagcg gagggaaaga aaagcactcg ctcccatctt
954121 cccaccccac tgtcttgcct ctctctgttt tttcgtcctc cagaaagcct acggctgcag
954181 ggcggtgggg gagagacacg cactgacata gagtttgtga cacacgatga ctcatggtgg
954241 agacatttga ggagggatac gaccttaata cgcccattta tgtaatcact ggtcgactcc

954301 cattagagct tcacacgatt tagcatttat ctgaagacac ggccatcgtg tcattttgaa
954361 ccatttttat tttacagaaa agtaatatta agataaaaat ttgaaccgtg aatgacataa
954421 atgtgtctga aaggcaccaa actagtgaaa agacactgtt tcttcccctg catttaaagt
954481 ctccatgagg ctaaatcctg aggaattata atctacgata cttcacggta aaatattgtt
954541 tcaaatccaa actataacag cctgtcagat aattgtgcga gtcagaaaag acaagatttt
954601 agaaaattca gaaccttcaa atttcaggct gcaacttttt tgtcggatct gaaatgaaaa
954661 tcagccctga ccgctgtggc gcggctacat ttggaaaaaa gttcaaaaaa acaaaaaaca
954721 aaaaaacccc gacaaaaaaa tgaaacgata taagatgcac ccgtcagata ttcagatccg
954781 gtcgcggcac tggagcgggg cagctcgccg gtgcggcgct gggatgtgac agatttggac
954841 tctgcctgaa aacacgtgaa atggcctgac atgatactta tcgactggtc ggcggcggcc
954901 gcgcttgccc ccgccgaccc cgaggagaga gccggagcac aagtgatgca gggaggatga
954961 agtagagtga cagaggaaga gacagggaga cggcgcagag gcgggcgccg acggaagaag
955021 aagaagaaga agaaggtaga gtggagtggg ggggggtaat aaagacttta aacggtaatg
955081 tgttattatt taaaaaacac gtatgcaaaa cttgtgtatt gtcagtatga atctgtttat
955141 acgaggagga tgaaggggat tattcccccg ttcccgtctc cgctcggggt caggcatcca
955201 ttttagtgaa cgctcctaca ttttgtgagt ctgggaggat acaaacgctt cacagtgttc
955261 cgatttgttt gttctttctc cttttttttg ctgcttttaa tcccaaagga gacaacaagt
955321 aggtaaacac gaagaggtag aacgcgggag atgatcccag catcgcggtt gggaacgcca
955381 cggatccgtc gcgttttagc cgctctctgc acttgacaac gtctcatcga agggatgagc
955441 atttacctcc cggcattaaa aagtcatttg cgcgcgacgt gctgcaaaac aaccttcgcc
955501 gcagcccgtc ttatggcaga actccgccgc ggagccgcgc taacgcagag gcgccgggtt
955561 aaagcggcgg cagctggcga ggagttagaa atgcaaatcg gacatttttt tggagatttc
955621 cataaaaaaa gagtccgtgt gacactctgt cctgctttaa caaccctcca gcttctcctc
955681 aggacttcta gcctcgaatg accccccctc gccccttttc cgacactcat ccaaaccccc
955741 gtgcacccca cttccccccc tttcaagctc attaagttgg gatgccttat cagagggcac
955801 agtcggccct ccaaggcccg ccgcacacgc acgtacgaat gtgtgcgtac acgtgcaaca
955861 ttcaagctgg ctcgataaaa cctattgttc atacgcgcgc gtgcacacat gggggggggg

955921 tccacgttcc aacccccccct cccccaacct ataccctgca aactcttcct
aatcaactct
955981 tcccatcctt ttacttaccc ctgccgcacc ctctcgcccg agactggtga
gacatttcga
956041 cggagggagc gatggtgggg aagagtgtgt gtgtgtgtat gtgtgtgtgt
gtgtgggggg
956101 ggggacagag ggtaagagat gcactcctcc tctttgctct gctgccacag
cggcttggat
956161 acagaaccct ttgatgttct gcaaatttca aatttcatta tagagaaccc
ccttctcgtc
956221 tccccccgct cccttccccc tcctctcctc cccttctgtg ctcttgcgct
taaacaagct
956281 tttctacccc ccccaccccc agtgctgaag ttaagaaact gaaagccctg
cagaagagac
956341 tcttcttatg ggttaattat tcattgggct gcgcgtatgt gccaaatgta
tgttgtgtgt
956401 gtctctgtgt gtgtgtgtgt gtgtgtgtgt gtgtgtgcac gtgcatgcat
gtctgtgtga
956461 gactgaataa gagagtgcgt gagtcacggg ctggcggtcg agtggaatgt
ggatttcgca
956521 acgcccgttg taactttgag cgaggtgcct cgcccccgct gaccctctcg
ccactcggtt
956581 ggccttttag cacggggccc ggcgctcacg cagcccaacc acacacgcac
gccgcaacgc
956641 gcgcacagcg actgagccag tgcttcctcc tccgagatgc cgcttacgtg
ccagacttcc
956701 ttactcagcg gcagtgcagc gaggcgacca tttcagggcg agatgctgcc
attttggttc
956761 agagcgcgtc agctgctttg agaagacacg cgtcccagtg gcccctgcga
tgaaacgggg
956821 gtgccactga aaagaatgac agatcagtgg tgtcagcagc gtggcggaga
gagaggaggc
956881 gaggacgaga gtcggaccgc ggcgaaacta tcgaagccta agatggagca
aagatgagct
956941 cgttgttaga gacgctgctg ctgttgtgca aaaccccacc gttaaatcag
gctgaatgat
957001 ggcagacttt caaaagcatt attaataaag gtttagctgg tcattaatct
caccaactat
957061 accctcagtt gaagatattt tccttcacgt tgctggtttc agatgatatc
cggtcttagc
957121 ccctctgatc tgatagcaac cgtccccccc accccctcc tttcaccatg
atcatggcct
957181 cagctgtcac ttctctctga cccctgaccc cagcccaagg ccaaggttgg
aggttgttcc
957241 tgtggcaaca aggaagccgc ccccatgcac cctgtgaccc ccccctgacc
ccagatcatg
957301 atttgctcca tccctgccag cgcgtccatg aataccattt aagaaataga
aggtggagaa
957361 caaatgggac ggatgtgggg atgggggggg gatcaacggt gaaaaggcta
attgatgggc
957421 gaatagatgg cagaacaaaa gggggacgcg tttgtcagtg aagagtcgac
tttctggaat
957481 agagtccgat gacctttggg ttgactttct cttggctatc cagcatgagc
cctcagaggt

957541 cacagagaca gaggctgttt ttgtccctta attacatctg ccctggtgtc gaggggaggg
957601 agggggaaca gtgaagggaa cagcgctggg cggaggacat cctacctgct ggtaggccac
957661 ttgactcttc aacgcgacct cctgctgagc gggactggac gtgtaaacgg ctgaacgata
957721 ctgggttcca tgatcattct gctgcctcat acctgtattc acacacatac acaagccccc
957781 cccccccccc aacacaatca gcacaatggc acgtttagcg tttgcacaat catgtcatca
957841 ccctgcatct tgaatacatg aacctaaatg agtttaaaga tcagagcaga tttcaccggt
957901 ggaaatgtga tgtgtgggtg ggcaggctgc agtatgggta ggggtgtgtg tgggtgtgtg
957961 tgtgtgttgt catccgtgtg ccactgtcta atttttcgtc agctttacat cagtttgtac
958021 cttgattagg aagccaaaca gttgctgatg ctcccatccc gagcagctct ggcatccgac
958081 agtacgcgtc ggtggctttc tctcgctggc gagggcaaac tttgagcctt tacgacgtcg
958141 gtgcaaattc ataaatatat acaatatccg aagcaaacgc agcgtacgag cagattcaat
958201 cagtgttttt ttttcacacg acgatacctc ggtatcactc ggtggagctg ggggtttaca
958261 taccctttat ttatttatgt cgatctcaca aaattggatt tgcatgccct cctcctttaa
958321 cacggttttc ctctatggaa actctgcagg gaaatatcgg cgctacgtgg ttagcgctgc
958381 aaatgcctcg cgcgtctgtg ccccactgca aattcacagc taatccgcga aaaataaaat
958441 cacgttgtta tctgatcaat tgtgagttaa gggacaaatg acgcattaag agacgcaact
958501 ggcgtgtcat aaaaagttta cgggcgataa tctcaatcag aaattattag cagataataa
958561 atgtgcaaag gagccgcagt cacagggagc tggcgcctgc ctgacacagt gacctgcata
958621 tcctgtttgt tctagagagc tggcgcggga gagggacgga gggacgacaa ggatcctgat
958681 gtttcgtgct tggtggagtt ctgagcatca ggctttcaaa gtttgggtga tttattcata
958741 aaccatgcat atttgaagat cccagaggag aataaaagca gtaggggaaa aaatgacaat
958801 caagagaagc acaacactca ttacacatgc tcaaggagaa tctttatagt ccccatccag
958861 agcatgaaaa agtattatta tcttagcatt agtgatgcaa tagagtactg ttggtatata
958921 taaaaaccga ccgcctcaga gggctaatct ttgttaataa cattaatact taacttgcct
958981 tgatcagtag atgctgatca tacctgtcag tcaaacctag tgaaacctgg atattacttg
959041 gcactttgat ccaagattga tttgtctgca tttagttaat gtgaatgggt tgaaatattg
959101 ccaaaaatac agatgctaac gacagttcct tcataatctg agtcttaact gtgctgtaac

959161 gtttctctga tttcaaaatg ttctctttat gctgcatatg tttatggttc
tgtcagcctg
959221 tttccatttt gtctttattg tactcgtttg tctccctctg cttctgcatc
cctctccacc
959281 gtcagcctct tcctctccaa ctctgcagtg ctggatgttg gccatcttgc
ccccagcgct
959341 gttgttcaat ggggaagcta atggtggaac ctccatcaaa ggccacagag
ccactctgct
959401 attggccctc tgccggagcc tttactctga ttggcggctt tcgggggtct
tgtagccttt
959461 gccgtttccg ctgaccttcc tttctgaccc caagagtctg accagtctat
cgacgtaaag
959521 ctgcagctgt tctttacaga tgtgtaaaag taggtctggc tacaatctgc
ccattaattg
959581 ttaaattaga tttttttatt aaaaagaaaa caagacaaag gtttttcctc
acaaagaaac
959641 gggtaatgat atggaatgta tcacacacgc atgcgtgcat gtgcgcaggc
acacacacat
959701 cagccttgag tggtagtggc tgtctccaac acaaagacaa ttaagattca
ttacatcttc
959761 agagatccct ccaccatcta aaggggagct gctctccaat caaatgggaa
cacgttccaa
959821 aacagcagcc cgggttctga ttattcccgg tccaaacgct taaaccctct
atccttaagg
959881 aactacagtg gcaacaaata ccaaaaaaaa aagaaaataa ctttgattct
cgcctcccgc
959941 ctctgccgtc tctctcctgc tctccatttt taacagcccc cttcgttatt
ttctccatcc
960001 atcaaaggca gcagtaatta ttaatgagag cctccctgtt ctttctgcat
tatgcatgag
960061 agccttgcgt ctgaaagcag agggtaatgg ggggtggaca aaaaggggag
gggccacagg
960121 cgacaccctc ccacgcgcac agcctcggag gggtcactga cctctacccg
aggagccaaa
960181 aatattaatc agttccgccc tccccccttt ttcaggaaat tcaactgtag
ccgattaaca
960241 caaacggggc caaaatcaga caaaacgcga ggatgctttg acattttgag
tctcggtggg
960301 ggcagctcct tttttttttc cttcttccca gcagtttgtc ttgcacaaac
ctttggacat
960361 aagtggacat ttttctccct ccttcgctaa aaatctgctc cagagtttgt
cctctggagt
960421 ttatcctcaa atgaaaattt taaaaaggag aagagaaacc agagaaaaag
caccactcag
960481 ctggtccctg ctggcttctg gggataaaag cttcctattt cttgcacttc
caacctaacc
960541 tgcactgctt ccctctgcag actttggtac cttttctttt gtttttagct
gttcgctaat
960601 tcaaatgtga gcaaacaaca acaacaacaa taaagctgta aatttattcc
agggtggcca
960661 atctgttgtt ccaggttcgg tggtctgacc tcatcgtatt ctcctctaga
ttcttcagac
960721 gcctcatgag tgaacaagtg taagatctca gattagcttc cccacttctc
ctgcgctaat

960781 cctcctcttt ctgcattaag gatgctgctc agcgcttgcg aagatgtaag
atgagaaaag
960841 atttcaaacg aggagaggaa caatttcatc acagacaaaa gaataaacat
ttcaaccatt
960901 aagctgttgt cagcgcctgc aattaagaaa tgtttctgca taattggcga
cttttcgcat
960961 ccttctgagc gcagagcgaa aacatttgaa gtggatttgg aatcattgca
gttgtcagcg
961021 aggaccaatt aggctgcagc cgagagcgcg ctaatgaaca atctaacagt
ttcccttttc
961081 tctgcctttc tggtttacct ctctatccca cccccacctg ccttccccgc
taccgtgtcg
961141 ctactcggtc acccacgggt ctggagcgag tcgaaacatc aaccaggatt
atttggtatt
961201 ttccacgctg ataaactctg accagatctc attatttcta ctattctgtc
agaaaaaggt
961261 gaaccgcgta cacacaaaga gacataatgc ctgctaactg cagtcttccc
tctgtgtgtg
961321 cgtgatagcg ccgagcgctg tgccgtgccc cacggtcccg atacgtctgt
gtgcgtgcca
961381 gcagagccct ctcctcctct cagcagctat ttttgccctg ttcttatgta
agccgctcct
961441 gacgcgctgg agacaggggc agagtacaga gatgggatgt cgagagcatc
cccaacagat
961501 acacattcgc tagcacatgc gagaacgcaa atcgcaattc ggcgtacagt
acacgctaac
961561 gggagtgtgt gacatatgca aacatacgaa gtggagcaga gcgagatgct
ctgttggcgg
961621 tcaggtggtg caagtacaca cagatgcagt cctgaaccga taacccagct
atgacgacag
961681 gacatgtttt tacataggtc atataatcat ttacacagat aaagagggag
agagagagtg
961741 tgcgtgtgtg agtgtgtgtg tgtgtgtgtg tgtgtgtgag caagaaacat
tgtatttctg
961801 gtacccaaaa atctgcctat tcaggttcca aatgatcatc tccgctgctt
tccaacatga
961861 tcgcttcatg gttctgggca tgttgtcaga gcaccgtggt tctggccctt
gtgctgtttt
961921 atttttagcc gcttgtgcat ctgacaacca gaacttgttt tcgtgttgct
ccggtattta
961981 actttaaatc ctgcctttgc tgccttaact aagctcaaga caaatgatgc
aaatgatgca
962041 gggtaaagaa aatctcattc tataaaaggt tcaaatttaa aggcttttgt
aaatagcaac
962101 ggaagggaag gccacattat aaagccattt aatttcacca tatacaaaga
gacgccgcgt
962161 ggcaactccc agcgtgcaca cacgtgccat aatctccata aattaggcct
gcctgtgtgc
962221 gctagggtgt aacaactgac acctggtaat gagctcgcca ctttcagcca
accgctgcag
962281 agagaagggg tggagttcga agattcaggg ctgcaggggt cacaggtcag
gggtcacagt
962341 cacctcctgc gggggtttgc cgttagagcg ttgggggatc cctaaattga
gggatgaggt

962401 gacagggggg gcatgtggag ggtgccctac agggaattgg cgtgtgtcgc agccagctgg
962461 gctgaagata ttaaataaaa agaatattca tgctcatctt aattgtcaaa atgtaactgc
962521 cacctgcatg acttggcaga tggaggacag cttgatttat gtctatcgtg tgacctgacc
962581 ttctctggag gggggtgact ttttggtgca ggttaatgct agttatgcag ggaatacaca
962641 atatttcaca tttctttatc tatttgaagc tttggtgcac ctaaacaggg tacgtaagta
962701 aagaaggaat gtgagggtgg catcaaatga gggctgctgc ctcggccatc ttagctgcat
962761 attcgtgttt tcgtgtctat tcatgcatgc ctgtgatgcc ggttcactga cgcagcatca
962821 gcagcccaca ccataaatac tgcatgggct aaaaaaatgg agagaacgca ggcaacaagg
962881 gaagaggaag ggaaaggaga ggaaaagagg ggctcttggc tcttaaaagg aagggtggcg
962941 agcagaggaa ggagagggat ggagcaagag atgggagatt ggggaaggag gtggaggagg
963001 atgaagagaa aggagaagaa agtgactaag agataaaagt ggagtgagcg gatgcgatgt
963061 ttgtgatgag cgggtggcct cggggagcag tagactcaac gcgctgctct gtggatgtgc
963121 tggcgggctc tgggctgctg ctcacttgcc tcttcgctcg ctacataaag ggagagggtc
963181 tggcggaggg ggatgcaggt gagacaagtg agggagagag ggggtaagga agctggaaag
963241 gtggcagaag gaacagatgt aaccttacat ttgaaaagaa caaggagaaa agtgcattat
963301 ggaagcatga atatctagaa tgagacggca ggtcggtacg ataaagctgc tcctccaatg
963361 gcagctgcag gaggagaggc ctagatccct ttaaccaaag tacccaactg acgcgcctcc
963421 ttgtttagct gtgcgcactc accatgtgca cacgcacttg tgtcttctgc ctatttttat
963481 tcgcgcacac atggattaat gctatctacg aacagacata atgagcgctg gcgcataatg
963541 ccaagcgaac ggcgcgcttg tgaatggtaa tgcgtgcaca attgatccac agtgtgtaaa
963601 ccacaagatg tgtgtaatca ttgaaaggca tcaaaccacg tttgcactct cgctgatgaa
963661 gcagaaaatg tcaccctctg acacgcagat gtcaggatgg ttgccgaaaa gttcacgtac
963721 agcgccgcgc gtgcatctga gggcgacgct gctgaagagt tttgcctaac ccagacataa
963781 gaaacttata aatttggagc gtgttgaatt ggggggg tgg gggtggtggt gttgggggt
963841 tggcacggca gtggaggggg ggagggttta tgcaacatcc cagggaagcc agcagattga
963901 gaatagtgtt gctgaatatg gaaagagggc acacagcaaa ggctgctggg agtttctgat
963961 tggctgctaa tgttgctatc caggctggga ttggtggagc tgacgcagcc cgacatcacg

964021 ttgtcgggac taaacccttt tgaactgagg caaagcagag aggcacagat ctcgaaactc
964081 acacgcgcac gcacacacac acatgcacgc acacacgaac ggattttttt tttttttaaa
964141 gtatgcgtgc agttggatat aggtggatga tggttgtcga tgcgctccga catccacgct
964201 gatcgtgtcc tgatgttccc gggtttacct cctcgtttga tgacttgtcg caactctgca
964261 ttgtcaatag cggaaaaaag atcacacttt cttaatttta acgttctcat ttaagcccgg
964321 gcctaacttg gccaggaaaa aatttgagcc aaagcatgcg tgaaggtgca gccggcaggt
964381 ccctttccac cgctgcatgg cacatttgtc agatcagagg aatttctcct gcacatgaac
964441 ggggaaccgg agaagacatg gattcacatt tgagtcacga acgacgagca tccgtcaaga
964501 aaaataagcc tatttgagct tgtggttatt ttccaaacat gtcacctggt gttaaaaaag
964561 aaaagagtgt atagtcaata agatgtgtgc tgttctgaaa tcaccggatg cgacaaggca
964621 gtgactcagg ggaaatgcat gactacactt ttggcccaac tgtttttgcc ccgtggtgag
964681 ttgctttgac tctgcttgtg gtgagatgag gcttcagtgc attggtgagg aatatcagct
964741 gatcggtgct ctgagacaaa tgtcaccaaa ggcgtttccc tctttttcg ccctcttctt
964801 tgtcatccgg cttacttcag cggggagaag cgaagacacg gcggacgtgc cgactcatct
964861 gtaagtcagc tggacaaata tggccgacct tcgagtgaag gagggaacag ccatgacaga
964921 tgcagcagag acggaggagc tcacgtgtat gtgaaaagcc gaaggttccg ctcatgaatc
964981 tttgatccat ctcttccaaa cttgtcacgc tcattcaata tgagtgtgag gagcaccttt
965041 ttgcctctct cttgctctct ctcacacaca cacgcacgtg ccctcatagt gcattaatgt
965101 tcttacatca cctcctccat gagcagagac aacaagaaac atgcagcgat ggagagacga
965161 gtttggggga agggagcctt gtcgctgtta gggagtgagg gggttgaacg acgggagggg
965221 aagaaacgtg tgtcattaaa gaagagtggt gggtagccaa ggatttaaat gctccaaagt
965281 caaacatgtg catcgtagtg cagggtgtgt gtacgataag gctctgaatc ttctgttaga
965341 agtttaccct ttcacgttaa cagcttcatc gatgtcgccg gattacagtc gtacacgaaa
965401 cacactctgc ataattaaag acttggagat attcagccca gaccagatta aatgtattac
965461 caccaccatc atttgtaaca gcagataata acataccctg tatactatat gatggagggt
965521 tttttccagt tagggcttga actcgcccca ctggtgtgcc tctccatgga tggctggagc
965581 tcagacacat taacactgtc tggcattgac ttacacccat gagatccacc tcccccacat

965641 acagcacaca aaggcgcacg cctgggcatc ttccttctgc tctaatacac cttgtgccct
965701 tcgggacagg tgttatttga gggcgacatt tgcttttttt gtgtgtgtgt gtacaagttg
965761 ctaccttgag taggatcatg ccgttcccag aagtgtttga gcagcgtctc aagactgacg
965821 tctttggggg agaagaccac tctgaccacc tcggtgtgac cagttaggcc taaaggagaa
965881 acaaaaagat gcattaacta atgacatttc aaataaaggc atctgctttg cctccatgat
965941 cccatcttcc tcctcatccc tctatatctt ctttaaacag ccaggcccag tcttgtttgc
966001 cttgtgagtg ccactacggg cacgcgtcag ccgcaggtag aaggcaggtc aaatcaacat
966061 ggagccatgt tattgcttat gacacaacgg aagagaacga caaacagagt gggggattgt
966121 ggggggtaat caaggtaggg gtatgttgat aacatcgcca atggaggcag tctactgcat
966181 ctgctgctgc caaagccccg aagattcgac aaactgaagg aaaaacttca gccctaacat
966241 gcaaagaaca cgcacacaca cgtcagtata gtgggcttaa gtctggtctg gtttctactg
966301 gggtgcgccg cctgatcctc tgtcatggca gtgagtcacg agaaaagatt gtgacacgga
966361 gaaaggtctg cagtagttta ttataataga aacaagaacg atgccaaatc tcccctctca
966421 acacagagct ggtgcacgtt gttggccgtg ggtcatcaat catcagcctg cagcgtagct
966481 tgcttcacct gtactccatc atgtcacagc tgatctcatc agtggggtac caaaaaataa
966541 gatggaatta tttataaatg tacaaaactg gtttaaactt ggagtcatct tggattaggc
966601 cgagaaggac aaggacgacg cccaaatttt atatggtata ctgtatatat ttaaaaaagg
966661 tcatcagatg caaaagcaca cagatggatg gacaagtgtg aacattcaat tgctcaggtg
966721 agcgtgatca gctgtgcatc tcacatggaa tgaccccaa gagaggccgg agggtcacca
966781 cacgacaaac gaggtttcac tatatgcatg aatattactg tagtcattta taacaatgtg
966841 attggctgta aataagggga gacggcgtcc acagggggat agacggtggc agagagctga
966901 aaaattgaac ctgcactaca atctttcaca cacgtcccat gtgaaaaagg tcactggaag
966961 aggagtgaat gcggctgtta ccgagtcaat atgccaaaga acacttgtgc ggggtgaaaa
967021 atggccgtgc tgtcagcgac cctcctgtca ctgtcagata cctattccag tgggggcctg
967081 cgtttctcct gcccacagct ttttctgagc agaagacaat gaatgatatc atcagacttc
967141 cttttatgat gtttattatt gtcactgagt gatgaggatc gttagcttta acctcaacct
967201 gctgctaaag tgcaggaatg ctcagaatca acccctgcag gacaaaagat ctggtgtcaa

967261 tcaattccac ctctcaaaaa tcggactgct gactgactgt ccgtcaaccc ggctgtcctt
967321 ccaccctttc ccagttaagc tgacacttcc atcagctgct cctctctgtc cagctgtctg
967381 tcctgtcctg gctgtgttga cagctcctcg gtctcacgcc atggcggtgt tctgctccgt
967441 catggccaat ggagcggtgc ctgcgccctc tcagccgatg gacaggcggc tattctttct
967501 cacgctggtt tgcgttctaa attttctttc accacaaaag tttattctac ctgctacccg
967561 acttgctgta gctctcactt tctcttttcc tgtgatccat ttctcatccc cacctccctg
967621 cattgctgac caccttgctg gagagccgac agaaaagccg acagatagga aacacacgca
967681 gccctccctt gattggttct gctcagaaag cctgccaacc aagcccagag cgccccaaag
967741 cgtctttgct tttctctcca tgtctcccat gatgtggctg cacctagact ccatgcccgc
967801 ctgtgaggat gtgccctcat gcatgaataa gacagcccta aaaacacgcg agaggctcac
967861 aggatgagac agagctagac atcttgattt taattataca tcaacgatct acatgaatgt
967921 attcacatcg cgcatggaaa gaaaccagcg gaaacaggta tttagaatat tttgggggct
967981 tctcatgcaa atacaggaat cggcattgga tcaacaaaat cctgttctgt aaatcagcga
968041 cttcacacaa tgctttggtt tttaattcac gttaacctct gttgctaaaa acgccgcaat
968101 acagcataga acagaaccca atccttacaa gtaggtcatg gggaggtggg gagggggggt
968161 acatcccatc tggagcagtg cattctgagt agttgactgc agatgaaggg aaggcggtga
968221 gaggatgagt gtgctgtatt taaaaatgcc ctttccggct cactaccgcc tgatttttca
968281 tctctcattg cagcactggg tctcttgaac cccgactgat ccgacgaaga tccatctcgg
968341 ccctccggtt caccaccccg caggggaggt gcgccagcgt ctgaaatata tccaacaaaa
968401 tgtactattt acctgagcag acttcgtggt agtttgggtt cggtgtgtaa ccgccggcgt
968461 agcccacctg tgtggagaag acccccggaa gtctccagaa aagtctctct gcaccccaaa
968521 aacagcccat acctgcagag gatgacagca tgaaccaatg agcacagact gataagggtg
968581 ggccccacgc tgggtcaaat ttgaatcata aatttgtgac tgtgctgcct taccaaacat
968641 gatggtctcc attccttctg gaaaaggctc cactgtggga ttcccgttga caacatgctt
968701 gactgtaagc aggaaatatg tacaaatgtg aaatcagcac aattgtttaa ttatagtaca
968761 aattaaaaga aagccatcgg gagcctcgat aggctaatag gcctgtgctt ggataagggg
968821 ttgacccggg ccagcggatc agaaagagca gctcctgtgc tagagtacct ttatgaggtt

968881 acgcaagagc gactgccatt ctgtccctct tgcgctctca tgcagtcatt tctgatctcg
969941 ttctcgtctt ctcagggttc accaagcgag tcggtgagag caatccaggg gcatgggtag
969001 atatgtgagc ggagacctca aaaacagacg cgacggcgct aatcttcgca atgctacaaa
969061 cgcctcagca tatactgata tgcaccagtg tgccgagagg tggcaggggg aggcccggta
969121 atgccaactg tacagtacag aattagagca ctaaaaagga agtgtgtgta tgtttagtcc
969181 acagatgtgc aaggcggatt agggtttaag aagactgagg tgacttatgt gtaaatcgcc
969241 tttaagcaag gctggtttgt actgcaacca ctaatattag tttacagata aacaggccac
969301 caggaatgct aagggcttag gtgcagacag ataactgatg cttgctgtat gtgcaatgca
969361 caactaccat tctgatgttt ggggatgtgc gacaatgacc ggaatacaca gaattgctca
969421 tctgataaaa attctggttc agttcctaga caggcaacaa cagaccgaag atttcagggt
969481 atcttagata tcaggtggca gccatgaagc cagacacact ataaagagtc gctgcagagt
969541 ttcagctgat gccacatgcc tcagtgtgcc acctcaaggc cacgtcttga aaaccgacca
969601 gcctgtttgg tttgtctgtg cagtaacaga ttgcaggagc acaaaggcct ccaaaagtgt
969661 cacagaggcc cggctgcctt caggccctgt tgtgccatcc caccgtgcac cgattctgtc
969721 tccaagcagc aggttgtttg gcaccaccag gtcaggctcg tgcacacagt cgtctgaaat
969781 caaagtgtat ctgtgtcagg gagcgaacag accaaatagt cttgaacaag gggggtggg
969841 gtgggggta agcaaggtgg tgggagggcg gttacgaaaa aatcctgtga agttttctcc
969901 agcctgtgct tgatttcacc gtcttttttc ctggccaagg atgaaaagca acaaagttgc
969961 caaatgacag tgagggagaa aaggagagag taagggagaa gcaagtgtgt gattgagaag
970021 gaggaggagg aagtggagga gtgggaggag gagagttcag cctgccaaaa caatgcaaga
970081 agtaaatagt cagtgagaag caacagatga tggtagcaga aagacggtga aggattcttt
970141 tggcgttcgg accagtgagc ctctaggcgc taaatcaaag gaccgagctc tttctccaaa
970201 agtaaataca ggaacaataa accagcaaac aaataaacaa tgttttaaaa tgaagtcgtc
970261 cctgtgcaca ccgacgtgtc accacaatca attaaatttc tctcatgctt ttctgctgac
970321 catcttccaa aacatccctc attccctgct caacgatgcc cacgtggagt catggatgca
970381 ttcaggcgca caatcacacg ctctccccaa cctctgctgt gggtatttct cccatggagg
970441 acctggcaga ggtccagggg ggtgccgggc tgaatccttt cagcccagcg tcacccctca

970501 caggcagaaa tggacgaata ctctgggtac gctcactcag tacactggca aaagaaagga
970561 cgacaacaag cagggagggt aactgaccgg gcttacagta gactggagcg gggggcatt
970621 tttttgaaca actaagacgg gcattattta tttagttttg cctttgtttt ccatggatgt
970681 caaagcagca cagaaataga ttcaacatac agctgtattg atgctgaatt ctgcactttt
970741 gttgctatta gcatggtagc tataggcggc tggacccgaa cgaaaccatc agaaccagca
970801 gcaaccttgc atcattccta tctagggtgc agcaataaat ctgttaagca tgtatgtgtc
970861 cggttttact agtttaagtg agtattaagt ctttatttac cctttgatgg gttgcttttc
970921 aacccatgct cccatgtttc ttatccggca caacgaggtc caagcactgc ttccccttca
970981 cgcagggaga cggatgccaa cactggtacc agagaggtcg attttcaggc tgcccagttt
971041 gtttctttcc aaaaatccat tctatttccc cctctccccc cacctcccag cttccctccc
971101 tttgcaagtc ctttctagcc tcacacaggt tggcgaggag tcaggcacga gtcgtcggtg
971161 gagcgggagg ctttatactg gtgccaaata ccctctgcta tactggagga tgggtaggca
971221 cacacacaca aaaagagtgg ggcaaaaggg ggaggggagg atgggcaaag acttgtggaa
971281 agtggtcata aaacaaaaat agctttatgt tttaatttct ttttaaattt atagttcaaa
971341 aagtggctat tctaatgcct gaactgaact ttaaaaaaaa atctgctttc ccaatgggtc
971401 tgtataagaa gatctttagt ttcatctatt agcaagacaa gacaaacttc ataaagcaat
971461 aatagcgtga atagaagcta ctgtaaggtt caaataacta ctgacaatgt atcccaataa
971521 agcttctcca ttcaagtgtt gaacatgtgc aaacggtttt atcagcctat taacttaaac
971581 aaatgctgag agctatcatg atgaaacatc aaattaaact ttaaagtttg tacaaagtaa
971641 aaagcaaact actctcactc ttacactcta ttttcctgac atgaagggaa gataacagca
971701 aagtttttca aacaaaactg ctcctgatgt cctcagatgc aacaaaggag aggcatcact
971761 cattcactaa ttgtgaatta caaaagaaag cgaatcaaat aaacacctaa ttgtgcaaca
971821 cagacataaa atgttggtcc cacatgttcg gaatagggta cttattggac aataacacct
971881 tttaggacac cgtgatctaa ttttagtgtg ctgccgttgg ctctttttga ctaaaattaa
971941 gtcccaaatg gtctctgaca gctacagtga agtttccgat ggctgtgatg acacctacat
972001 tgtgtgggaa tatttcatca gaggctacat gcttgcacac acatgtcagt cagaaccgtg
972061 ttacaatgga aatctgagtc agcaatactg atagtgctga acagaacact cacagtccac

972121 atccatgggg ttggcatggc ttgttttggt tgcattaatt ttgtaggcct cttgagttca
972181 ttgagaaaaa tggcagaagc aggtcaagtt aagcacaggg tcagcatcgt actaggaaac
972241 aggataaatt agcaaactgt ctttgttttg aattgcatat ttatgttttt tacctacata
972301 ttttcctgcc cttgcagccc ttctgaagca ggttgcctgc tagcatcaga aaaaggaaag
972361 caaaatacat ctttgccatg aatctgctct gaagaattac agtgaggcat gtaacggaga
972421 ttgattttgg tcagctgacc tcccccaccc tgttcggaag aaatattggc agtctatcct
972481 gcgccgactc acaataatag ccgctgtacc cctaccatgt agcaatctgg cactggatca
972541 tcctcgccaa ggtcacaggg tacgtacaga caggcttgag ggaggcaacg cagcacaaac
972601 aaccaaggat cttcttcagg accagctaaa ggggtgggcg tcacttttaa aatgagtgct
972661 cttcattatg ggagtgaata tgtgtaacac gtacactcta ttttggacct ggaccgacat
972721 atgtggaaat actagtgctt actggcccat accaccaaaa acactggaac ctattccatc
972781 acacaggaga acaaacccat ccccacctaa gctgaccccc ccacagtgaa aggggaggaa
972841 cagatttctg ccatgcggtg tctccgagct gcttcgacag acggagcagg aacttacgat
972901 ggggcggagc cttcatgcgc ataataccca gatgggcccc tggatgctgc aacacctctt
972961 atccacccct acccagagtc catggccaga gtaggaacca ggttttaaca atcccaacat
973021 gggccagcgt atcacagaat tttacaacct gagattgtag gtcattataa ccaactacat
973081 aagagtaaga tgtgggcagc ggtagcgccg acattgagct gggaagtgga gggcctcaag
973141 gcccgatgca gacaaaacat ggaaggtgtt ctggtagtcg gggcggggga gggggtccgg
973201 tgagctaggt gtagatcccc caaaagctaa cacggggccc tgcgaggagc aggcggcaca
973261 ttcacggatg cactctgcct ttgcccacat gcagctggga tgggctccag caccctccct
973321 gtgaccccaa aagagacaaa gtggttaaga tttttttttt aaaagaaaga aaatgtataa
973381 tagtaagaac tggattaaga ggttaaaagg ttggaaaact aaactaaaaa gcttgtagct
973441 aaaaaaaaaa ggatagagga gtgtattggc agtaatgtgc actataatac cacatgacat
973501 gcaaaggtgc agctgaaata acagctgata aagggttcag ttgtcaggcg agttggttaa
973561 agtggtttct agtccacttc agattgacca gaggctggtt ggttcaaata tcattgttct
973621 cttcatatta aagacctttg aaaccaccct gaccttactt tctgagttgg gactaatgtt
973681 gaccgtattt cgtctccaca ttttagttct aatgctctct gctgtggaac catgttccca

973741 acctctagtc ctgagagtaa aacgacccta aaaccatcat taaatctaca taaaatgttg
973801 atttcaaact caactctgat caaccaatca gcgtcactga ttattgatgg ttattgaatg
973861 aactaaacca ggagttttat ttcatggcgg gaatattcct gcgctttgat gtcgcgttcg
973921 ctcgcgcttt gccatctgcc gtagacgtta atgcaattct ttgcacgtgc gttcgtttac
973981 ctgccaccac catcgccacg tgcctccccc ttagcgcgcg gtctctgctt ggcagctcgc
974041 ttttatgtgc catccgagtc agcggcgcga gcgacgcgga aacaggaagc aaccaaacgg
974101 cgcgcggttg cgcacgcgag gatggcggag aaaagagcac aacagtctgt ctccaccgtg
974161 cacgccgaca ggacttgggc ggggacgggg aaaaaaggca actcacttat gaatattcat
974221 cacgcgtggg caacgtcgtg cgccgcatat caactaatca aggggttaat atgactttgg
974281 cacaaaagcc tacagtgcgg gaaagaaatt aaatttaaat aaatatccgc gccgttatgc
974341 atgcaaatca gcttgcgcca attatcagct ctcattctgt gggaggtcct tctggtaaag
974401 cgtggacgta tggagcagct cctgttagac agtggtgggc ctatacagag cgaaacgcct
974461 gtttgtgttt gcgtgtgtta tcgaggcctt tgtcacaaat gtatcatgaa aaaaaacaga
974521 tagtggaaat aaatgaaaac gttcatttca atttcactac cagggtggtt gctcggcctt
974581 aatgtacgtg caccatcatt gcttgggcta attcacaaca gaagtggtcg gttggctaca
974641 gaggttattc agccatttta tttacagttg catttttctg tttctactaa tactcttatt
974701 tttgaattat tctcacacac tggtggttag tttgagcaac aaaaatgtgg ctgaacatgg
974761 cgaccgtgtg tgctgttcca ccattttccg caggttagat gtggataaag ttgtaactta
974821 gcagcaattt cagagaaatg agaaggcaga ttttgcaacg gtcagctcac attcatgagt
974881 aatttccttt gttgttttat tccatttttc agccagagca gaaatgatgg tgattattta
974941 catgtgaaag cttaaagcat ttctgcactt gctctaaacc agccaattta accagtaaga
975001 aagaaaccag gccattgttc ctgtatcctg caagccagaa taacatcaca ccccactgca
975061 atccctctct ccactcattc ctttcctctc tctctctctc tctcttattt gccggggaaa
975121 tgaagctcaa tcacctgcgg aggagctgag ggcggcaaag gggactttgc aatgcaaatg
975181 ccaggtaatt acccgaaagc ttggagagtg gaagcacaca ggatgaaaat agagcaggca
975241 gggagagaga cggagatgaa ccaaatgctg ggttcacatg tctggtgatg caggatgatt
975301 ggggtgtttt aggggcgtgt gtttatgtgg atgtgggaag aagaaagggg agaagagaca

975361 aaaaggtgat gagaggcaca gacatggatc gaaccacata cacactgaga gagggcaccg
975421 agtctctgct aatttcttcc ctggtatatg ccccccaacc ccaccccccc acctccaaca
975481 aatgatcaat aaagctcagt agcacaggac ggaaggctgt gcttttaatt tgggagaata
975541 tttgtggtga acaaacccct ttttttgca cgagtgctgc tgcagtttga gtagaagtgt
975601 ttgcgcctgg ttgcaccgtc gtttgcgtgc tctttaatgg gtacttgcgg gtgcgtgagt
975661 gtgtgtcgct tgacgtgcgt gtgtgacttg tgtgttgcac tggaggattt agtgagtgtg
975721 ccagcttgca gctgtttgat tgtcggtgag tgagagtgaa gagatgtaca gatgtgagcg
975781 agtatcaaag agattatgaa atgacctgga atgaaacgcc gcgctggatt tcacccggct
975841 accgtggcac gttaagccca ctgtttgatc aagtagaatt tttaggttac tccacgcaat
975901 tccgttttag cgccgtagcc caaatgccga tttgcaattc agaataactc gagtgtttgt
975961 ttggttgttg tcatgtacct cgactccagc gctgcctctg aacagcacct gaacagaaca
976021 ggagccatcc aaacagaaaa actgtttggc aatcgagtgt aattgtgcct ttgctaaagt
976081 ctcactttgc ctaattagaa taataaatca tgccgcctcg ttgttttcaa agagccctcc
976141 atcgtctcgc cgaagcaggt gggattgtgg gatggtgcga aaaacaaaag ctctgattac
976201 cctgctgcgg ctacagtcgg ttaaaagcac aacacggcgc tctccgggga ggccgagtca
976261 aaaagcgagc tgcacgggat gataaaccag agtggggaaa ggcaacttga caaattttgc
976321 atcatatggt cgcctgcact tgacttctct tgacataaat cacttggctc attggaagat
976381 ggcagacagc gatggtggtg gggtcggggt cggggtcggg gtgggggggt cttttccagg
976441 atggttgttg tgttgtttgg tagatgtgga aacctttttaa attaaaggca caacttcagg
976501 ttaaaacaat agtgagactt cagattccca tcctaatttg tgtttacaat aagaagcttt
976561 tactccatcc acaatgtaga aatgctgcat ttactgttta gtttagttca gtttagtgtc
976621 agctctttca aaaagacaca taaacagaac acatttcttg aatatttgat tatcttatta
976681 tcagccaaat gtggtataac cattacacca ttttctaatt aaaactctaa atgtcatact
976741 gatgtcaaat ccaatcagtg tgggcaggtt ttcagccaca cgcgtcccca cacgatgcta
976801 aatcaattaa cgtaaaaaaa atttgaggcc aaatgttctg ctttaacttt ttatactgag
976861 aaaagatata gaaataaata ttctgttgta aaacagtaat caggccgagc tggtaatgtg
976921 ataatggtgt aaactggaat ctgtagagtg gcattaaatg aaaatttaat gaaacttttt

976981 gcacacacgc ccagtttgag ctccgctacg tcatttgcat acttttgtta cagccgtctt
977041 agctgaatgt gctgattttt atgatgaatg tctccttctg gacctaagtt gctgtacagc
977101 ctgattatcc ccttgatatc aaaccacagg atgttaaatg cagataaact gacagagaca
977161 gacgaatgcg gcagttctgt gcagtacttc ctcttcctct tctgctccct gctaagagcg
977221 gcggggctaa gccactcttg ccctacatac agcctaaaca gccgattacc gaatgtccct
977281 ctaatcccat gcaccagggc atgtgccact gtcatcatgt accaaccacc caatcgccct
977341 gtagccaccg tcactattcc tgtccccacc aagccccccc ctaaaaccca accctgccac
977401 ccatggctca gcttcctcca ttttctactt ttatccactc ttccgattta cgttgttttt
977461 ttgtcagaag ctgcgatacc gaaacccccct ttcagagctg tctctctcta tctttcccca
977521 gcaacacccc atcccatcct catctctccc tctccacccc accccaaccc gtttccagtt
977581 ttaacctcct cttgaccacc tggcgtggga tagcttcctt tagtcccccc cgcccccccc
977641 actgcctcac ctaaaccacc agacctgaga ttagggttaa tctggacgtt gtgtcatcct
977701 gtatccatcc acctccccca cagcctcatt cagtcctcct ccctccctct ttaccaaaga
977761 ggctttggtt taaaatcctt tattgtgtac accacactcc cctcgctgtg tctttccgcc
977821 tgctgccaac tgcaactttt ccccccgcac ctcagcatca gccaatccca aaacggacgc
977881 tatccccaag cgacgttggt ttgaaaaaaa ggaaccaaaa atgccagaaa aagatttcca
977941 cactattgct gtctttggaa atctaggata ggaagtaaag gaggactcaa aatggatccg
978001 cggcccgtct gtgcgcgcgc gagcgtgcgc taatgcatga ctgtacttag cgagttagca
978061 tacagggcga tgacttgtca taatggtgtg catgcaacac tggcaggctg tgggtatgtg
978121 tgtgtatctt tgtgtaattg ttggagggtg gctgcacaag tcacaggatt tagatgagag
978181 tctggagaat tcatcctcca ctccactgtc ctagtcgcac cccctccctt ctcaagtgca
978241 tttatccagc cgagctcttg cagccctctc accccgagga cagtctaaag gcgcggaggc
978301 ccgagctgga tgaggagacg ccacaatgga gctggctagg atgtgtatag aagggagaca
978361 aaaaaaaatg gggctaaaaa gacacacagc gctaaagccc cttttcatgt ttttgtcttc
978421 acaaataaat ggtggattgt ccctcaagca aaagtcttct ttcttgagtt gaaaagccgg
978481 gggaaaagca cttggcagag tggtgggatg gagagtgaga ggtagagagg cagcgagaga
978541 tgtaggacaa gaataaagtc tcttatcaca agggggcaga aagaaagacc cttgagcagg

978601 agaacctgca cgagttgggt gttttaatga gagaactggc aagaaggagc ctcagaaaag
978661 gatcgcaaga tgcttcaaac gccatcatac cataattacc ctcaaccagg actgctaatt
978721 tgcttgatag cggcgatgtg ttcacttact gcagatcaag aatatcagtg agtattcctg
978781 tctaaatcca ggcaaacacg cagcgaggtg caggaccgtc cgctggagcg gcgctaacgg
978841 gcgactggat gtggcgaaat gtttcagaaa agggagagcg gaaggaaaga aactcgttca
978901 aagtgtcggc gcgcgggagc ggagcgtccg cagaggccgc ggcgaagaga gcgaagccgt
978961 gtagacagtg gatgtaaatt aattgctgtc atgtggattc tggtcccagc aaatcagggt
979021 cacgtcgctc ttctgtggtg cctcggcgtg attggaaaaa aaagggtcca ggtcgcctat
979081 gtagatatat atgtaaaacc taaaaaaaaa atatccaaca gctttaaaat ttctgatcct
979141 tgcacaattt atacatttgt tgtaggtgga attctgattg aggataatgt gacaaagcaa
979201 taaaactgcg taaaaacctg cagataaaat gaaattataa tacaaatgag cagtaatgtg
979261 gaataaaagt cagtgcagca ctgcaacact ggctcttgta taatctgcct acagtacacg
979321 tgatggccgt gccgtgccat aaaatcctct tcatcaccag gagcttttca gagagtcacc
979381 tcaggtccaa acagaacact ggatgccaac tagtgcagac ctaacagggt tataaatgca
979441 ctgtggtccc atgaattagc catgcttatt actgaaaatt acgaccgcgc tttctgctta
979501 actaggctgc aaaattgatt tacctttttc tccagtctgc cacgatccca aagacacccc
979561 cttaatatga aagataccag tattatttta tgtgctaatc agaaaatatg tgaagcatgg
979621 actttaatta caaataaaac aaagacatgc ttttattaaa aataaaaaac acctaaaaag
979681 tcaccagcct gcaaatcaca ccaaaaatat gctttttcct attttgtttt gttttttgt
979741 ttaaaaaaaa aaaaaaaaaa aaatcagtag actttacagt ggagtatggc tcttattata
979801 tacatgtttt ctttgatact gtatgcccca aagcaaatca ttcatgttca aacagtaata
979861 tctgaacatg acagaaatgg tgcaaaatgg gaagtaggtc acatagcaat ttatttgaca
979921 aaaagggatg ggggtgggga accggggcgt acgcagaggc agcagcgaaa gggaaggttt
979981 ttggaaaccc agatcagata gtgtgtgtgt gtgtgtgtgt ggcttattgt atggctgacc
980041 taaaaacaga aaagttacat ggtgttactg atcgttggag aagtgctcga tcagtttgga
980101 atctcgacgc tgacgcaggg ctttgtatag tttcccccct ttgattttgg tgaatgaaca
980161 cacacacaca cacacacaca cacacacaca cacacacaca cacacacaca cacacacaca

980221 cacagtggta gagaaaccga aactgatatc acggtcgccc ttccatgttg gtagattcga
980281 cacaagcgag ccagcagcat tgatcagttg tccctgaact gcactctgcc cccccccac
980341 acacacgcac acgcacacgc acacgcacac acacacacac acacacacac acacacacag
980401 atcctcaaac cccctacatc ctcctaaccc ccccccctcc cccttacagc actaaatgtg
980461 cgtgcaccca tatgcagagt tcacccgtat ttacagagta aatatgcaga atccacagat
980521 gacaccgtga acgtgtgtat tccagcgcag acacccacgc gcactcataa gctctccgaa
980581 gagctccgga tcccgcttaa atcccgtcat caccgaacac ctcagctata cgatcggagg
980641 ccatacatcc cagaggtctc ctccgacctg cgatgctaaa cactacagcc gagagtctcg
980701 gcggcagatt caagatcacg agcgccaact tctccagaaa atatgaaata gagaaatagg
980761 aacgccgtta gatgaagaga ggggattagc tgcactatta gatgaaaaca gcacttacgc
980821 ataaatctaa actctaaacc tattgtgctg ctactttaaa aggttaaaag ctgggtgtca
980881 cagataatga tttgtcctgt ttttacaacc aaacacacac tgtgtgcttt aaaaaaaaaa
980941 atcaatccgg gggtcaaaga gtttatgcat tcttatttgc atactgacag acgatctaca
981001 ggatctactc tccgcacaat cgagacaatc tgtgcagact gcagcgagga tccgcaaagc
981061 cacattaaca cataagagag gctctggagc accagtcttt tctaatgact ccataatccg
981121 acccaaatgc tgcctgcttg ctaatctctc catcggatta ctatactggg gacgagcact
981181 gtctacttgt ctccctgtcg tgggcctgtc tgtctcaccc ggctcttctc tttcatgtat
981241 tttgctttat attcctctgt aaaataattt tttcaaaaaa gcctctgtga tcctcgtgga
981301 tgtgctggtg gggctgcgcg gttctttctc tcccgccagg ttcttcacat ctgcaacctg
981361 ttcaaacgga ggagatagcg ggatgtcaaa atgtgctctc taagatcatt cttttctctt
981421 gatgatgtgc gcgtgagcaa accccagtac aggaacacac cctgcacgtc ccaacacggc
981481 ctaaatttat gccccgtgtg atgttgtagc gagaatttta aataaacaca cacttgacac
981541 acacacacac acacacacac acacacacac acacacacac acacacacac acgtacggga
981601 gcagattcaa tgtaattcgg attattttgt tcctctacac attttaagaa acaggctgaa
981661 atatgtgcaa aactggtttt gtgaatttac aggaatcgtg ctgtgtgtgt gtgtgcgtct
981721 gtgtgtgaga gagatacaca gatgaatgtg tctgtttggc ctcagctgca tctttaatgt
981781 caacggttaa gaaaaaagaa aaaaaatctc agaaaacaga actgtaaaat agcactaatg

981841 ctagctgatc tctgaaagcc cctcctgaga ttgtccctgt ctgcagactc accgacagct
982901 gagagacgga tcacatttgc catgaaatgt gatatatcct agttctggaa tatttgaaat
981961 ccttccaggt ttgcaataat ggcacgaatt tgaaatagtt tggggttttt ttttgttttg
982021 ttttttcgtg ccttcgcccg atgagatgaa aggatctgga gcgaacgtgt gtcaaaaggc
982081 ttctggaaag atgcgcagac gccgaggctg ttttgggtaa agcctcacga atatcacacc
982141 ggccggggcg gaagagcgga aaatgtttca taactgcact tttgtaatta gaaaggcttt
982201 ctttaattat gacaatatta tttggataag taggacacag atataacagg gcagatgtca
982261 taatatttca ataaaaatca catcaatcag agattttggc acacactcga ctacatgcag
982321 ctcagttcta atcctgcttc tgcacgaatg tggcggttgt ggttgtcggc gtattttgga
982381 ataacgagag aggttaggag cgactcgctg cgatgagcga agtgaagaag cgagccgtga
982441 cacatgagag aaggccatgg aggaagcgat ggagggctgg aggcgggaga gatggaggga
982501 gaggtgaggc tggtccagct cgtttcagct ctgcgtatgt gtgtgtgtgt gtgtgtgtgt
982561 gtgtgtgtgt gtgcgagaga ggcaggaaaa agaggggggg tgggggcact ctgctgtcca
982621 cttccaggga tcagctgatg gctatggatg atggagcaga gaggggtcag gggtcaaagc
982681 cttgcattgt cagccaggca ttgctctccc atggggctgc cttcttcgct tgcctccccc
982741 caccctccac gacacaccac tctgcgctct gccatcaatc ccccgctgcc tcgtgtacat
982801 ttacatacat ttatcaacgt ggtgcgttcg cgggcggctc gcctgcgcgg gccgggggac
982861 gcgctgcatg tgttacgggc cagtcgcagc gcgtccgtcc acccaaagag gggaaaaagc
982921 gcgtgcgtta caccgtgatc gggctctgac acgtgatgcg ttcgtgtgcc tgggtgaatt
982981 ttcacagatg tgtttttttt atgcacatct gtgtctttgc ctgtgtacgt gtgtgcggga
983041 cgaccccccc caactcaccc cagaattaga agtacccttc tcaacccttg tcctcccatc
983101 caacacacac acacacatgc acacccccca ccactttgcc tgtgtaatcc ttgccagctg
983161 gccctgttgg ctaatccctc tgcactgatc agctgggcca cgcatccagt acgggacagg
983221 acagggacag gggcagagag gggcgggatg ctctgtgtga ccgtggtgtg tgtgtgtgtg
983281 tgtgtgtgtg tgtgtgtgag agagagagag agagagagag agagctgggg ggtggtgtcg
983341 accagaaagc ataaattgaa gcccgtgcct ttgcctcagc gcagtgtgtc tgtgtgtgag
983401 ccagttgcct gttcctcctc gttctgtcca tcccttatct ttgtccttct cctcctcctc

983461 ctcccttgga tcctgataca tatatatacc tatctggcgt atatatgtag
attttacagc
983521 ttgggctttt ttttttcttc ctgtttttgg ttttgtcttg agcagatcca
cttggatttt
983581 tttctttaaa tggtggtgac ttgggtagaa ttaagatgag tcttttttttc
cttccttcta
983641 ccctctctcc tgttttccct gactgcatgc gtgacacctg cctgcctccc
atccctgcat
983701 cccaccactg acccccccctg tgtgcctgca tccacacgct catcatcccg
agcccgcgag
983761 gaccttggcg ggatggagcc gcgcggtctc acatctacaa ctacagcgtc
aacacaaacg
983821 caagcgggga ccgaggaccc agaggaggaa ataccgggca cctccgccgc
tgtttttctg
983881 cagcgtcttt agaaaggaag gatatatttg atctgagcta ttctgcttgt
acaagtgtag
983941 catcctaaat agggacgcag tgacgagtgt aataacaaag cagcttcaaa
cagtcgcagt
984001 tgttctggac agacattcgt gttcatttta gcagctaaaa tgtgcttttc
tgacagatat
984061 ctgctaagtg attcctgtgc gtacctagct atgaaccaaa ggatggagac
atgtcattta
984121 gatcttgttt ttaagtctgt acattgatat aggtacatga gcagggtgca
gagagagaga
984181 gagagagaga gggagagaga gagcgagcga gagagagtag gagggtgggc
gagagggagg
984241 gtaaggggag ataacgacac aggctttacc aagtgtaatt gaagaactgc
tcggtctgat
984301 taaacctcac caagttcttt aaccaggacc ggcagaagct tggcttgcat
cgaggcgatg
984361 ggtcaggcgc cgctgcgcat ctgcagcgcg tgctcataag acatcctgat
gccatccttt
984421 gttttgctgc gaggggggaa gatggtctgc aggaatggta gcagccaggt
agccgaggct
984481 gctgctcctc tccgtgatgc cacaaaggta gccgtgctcg ctccatctcg
ttctctctct
984541 ctgtggtgcc tttttaatga tgctgctata gaacgtagac agataaacgc
ctcagtgctg
984601 catctgctgc tgtgatcaat cgcctttttt ttttctctct ctcgtccttt
tgttcacatt
984661 ttgctgctgg agagacagga atgcatgcct gctctggttc tgaacccaga
ggaatcacaa
984721 tgtcatttgt cttgtatttt aactgttctc cccccaacag cctcccatta
ctgtctgcat
984781 attaacctga tttccaaaga gaatacaagc cgacgcgggg gggttagcat
gcattgcact
984841 ttgaggtgtg aaaggaccgc ataggaagac cacaggtgag gagaaggaca
atgtcaaata
984901 ctaatgggcg gaaaaatatt caataataag gtctattcat attaacaagg
cggctttatt
984961 aataaatgaa caccaaaggg gggaaagtgg ttctaaaaag tggggtgatc
aaataaatcc
985021 aagtgcatgt tggcgttgga gttttgggtt tgagctcttt gtgttcacag
tggaccttga

985081 tttaatttca atacaattaa ggcacgcggt gaatgccaag agagaagcca cagtcttcgc
985141 tttggaggat tagaattaaa aaaaagaaag aaaaaaagag tggcaacgac acagacggac
985201 gttggccaca atcctgcaag attaaccagc ggtgacgcta atgggatctg acaggaagcg
985261 ttaggcagag aggttacaga gcgctgactt cagatcagca ggcacaaggc aggaaagccc
985321 agcagaggcg ctgatatcag gcagaaaacg cagagagagc gagagggtca gacacgctga
985381 gccggtcccg tagccaaacg gacagagggg atcacaccgg ggggatctgc tgtgtggtga
985441 cagagggcca cttcaaaggt ctgtgtgttt acacctcagc ataccctcgg ccggatccgg
985501 gtggacgacg gcaacgcggc gtgctgtcga aggacaatca cacctttacc tttatttaca
985561 gattcacagg agatttgttc tctctttagt ttttttaaca ctgcttgctg ccaaaaggtc
985621 tcaataaaga tgtcaccaca tccctgtttg ccctctctgg tattcttttc taattaaaaa
985681 aaagccaaaa aaaaaagaaa aaaaggcata aagtcattta aatcatgagc ttcagctccg
985741 ataatcacag agctgcgtga agtgactcca gctctgaatg gctgcaggct acagcgcgct
985801 gacctacatc tggcttcatg gagacacagc aagcagaaga attacagaga ttcagctgcc
985861 tttattttaa ttactgctgt ggctttatta acatgttaga agtataaaaa aaaagcagaa
985921 gtctgggcag aaaacccttt attttctata aaaggtgaac attaaatgat ataaaggagt
985981 cccagcccca cacagggatg cagaggccac atatgcccac tccaaggtca tcaacaacac
986041 tggggccccc tcgcctgtct ggccattatg cattttccaa actctcagta caaaaggagc
986101 acacggggag gggtgggggg gtcatattca atatgtcaac agcgctggac ccctggagat
986161 gatgagcagg tgactgcgta tggacgtccc atcacagcca gaaaacacta atcagctgca
986221 cagattctta tgtttgtgct gcttttggtg gggggaatta aaataaacta gaaaaagtga
986281 cccccgacag gtgcaggatg atagaaaaag accaccaaag gtttaaactt gaaagaatgt
986341 tgaggatctg ataatattat gcttgtgaaa ggcaacttaa tacagctgga aatatgactt
986401 ttttaaaaat caagcagtcg agcctctttc ggtgactttg gctctaatct tcctgcagtt
986461 tggatcatca gaaaccgtct ttcagaaaca aaacgcacac acagaaagag agaagaaatc
986521 taatttagtc atgtaaatag aggtcgcgcc ggcgacatgg cgaagcgtca ctaatggaag
986581 aaagagatgg atgccttatg ataatgagga gaaaataaag cagaaccatg accttgcaga
986641 gcgagatgaa cccaacggca caatgaacct aacgcacggc cccggcagca gaatacagaa

986701 ggcacatttt gaaatatcac acacactcac agagcggcat agaaaggtgt gtgaaataaa
986761 aatagaatca ctgaaggttt ccttttttcaa gtgaattgtt gaaaccaggg tggtgcttca
986821 tttttagata atgttaatgg tgctcaaatt ctagaattca ataagacgtt aataaatgtc
986881 ttatatagga tgaagacaca cacgcacgca cacacgcagg aattttcagg gcaactcatc
986941 ctgtttttct atgcattaac ttgatgtgca ctccaggata cacattctga cctcctccac
987001 acacacttcc acgccattaa cacctcccca ctccccccac cacctctctc ctcctccatc
987061 gtctgactct gcttgtcttt tatggccgtt catttgcatc ccaggagggg ctaacaggcg
987121 tcctcaggga ggctcggatg agggtcacaa gaaatcccct tgaggcggcg tgtttgccac
987181 gcaccctcgt ctcagacgcg acacgtgccg tccaacccca ccccccactt ttcctttcaa
987241 ttgtgccccc tttttctaac ctttacccca tccctaatct cagcacaacc ttaattcaca
987301 aattcagcct caatctcacc ctgacattta aggataaagc tggagggcca ggaatcttgg
987361 ctccaaaaag ctgcccaggc ttcacaatgg gaaattctga aaattaggaa tgcaccaaga
987421 cacgcaggat ggagggcata ttgccagatt tggggggggg gggtgttctc ctttttctaa
987481 tattaacata cacgcagcct gtgtaaaagg tttcttccat tttaaccgct ctcattgctg
987541 attttagtgt tgtgcaaata aaccaaaata tcataaaaca tcccgctgtc atctattaac
987601 tattttaaag agactgacat cttaaatgtc tttgttggca aggcctttgt ttttgcaaga
987661 ttttatagta gttttagtta tttcaggaca acatatacag tatctgtact gacatatggt
987721 ttgtaagggc tcctgagagt ctatattgta ggtttaaaca gcaatagacg tgctggccaa
987781 taattaaggg gaactaaagc acgttaaaat aaaaatttca ccagtacatt aatttatcgt
987841 gccgagacca gacatgtaat aaaacacaag acaggaaacg ccaacattgg gcttcagctc
987901 aacgtggtta gcagagaaaa taatgttgtg tcaccctctt gtggccacag cgggtattac
987961 agtaacccca agaagaaaat ggcggacaac agataactcg cgccctgcac gtccagcggg
988021 gacatttatc ttaaacaatt gacagaaatc aggaaaagaa gcaaacaccg tggtaaagga
988081 gttaagccca gacacctgtt tgtggagcat ctttattacg ccacaaataa aagccagtag
988141 ttctgtaaga ggatcttgcg ttttgattca acaacattct gaaagtgatg gaagctgggt
988201 gtgatgtaaa gagccatggt caagcaggtt taggaaacgg gagcgtggag gacgagaaga
988261 gcttggaaag caaagagggt gtggtggtga aactctaaaa ccagtgtcag gtgggaagaa

988321 aggcggcgaa acattcatga acccagttcg ggtgggggag aacgcgtatg aaaaaaacaa
988381 acccaaactg ctttgcgtaa tcaatgggaa tgcacacaag agctgctgaa tataccacat
988441 ttattacttt tgaactgatt aatagaaaga gtgggggcaa ggccactcgc attgctgtcc
988501 ttagaaaact gagggaggaa aaaaaaacag ccccaagcat gttttcattt atccaagata
988561 aagaaaaaga gcgtaaataa agtccaatga ttgtaaccag ggaaggggaa gggatggggg
988621 gggtcccgat ttttttgaaa ccataaaatt agacactgat gcaactcaac atttgaaact
988681 gtgctcttgc tctccagatc ctcccaacac gacactccct ccagcacagg aagacggagc
988741 tggggggcg ttcaggagcc cccaccctcc acacacacag agcacgacag attctcgagt
988801 gggcctgaag atgaaaatcc actgaagagg gcagcgagag aagtgagcat ccttggaagg
988861 tgaggagaga gggcctacaa agaaaaggag acaaactgcc catttgtcac ttcgggacac
988921 taaaggaaac actccaaatt cctacggtaa cttgtgtttg gggggggggc tacgctcaat
988981 cttggaccat aaaaacaaga ggatcatcct ttccattaaa aaagggaaga aagcagcata
989041 caaaggggaa aagcatatgt agtggaatgg ggcgggacaa aaaaaaaaaa gatttcacct
989101 aagggtgcta tgcgtttagt tgtagtccta tgaataatag tttccttttc tttttactac
989161 aagttcgaag taagtcttta aagtcaaaag aaatcaagag agtcttcatt ttagtttttt
989221 ttttgtcctt ggttaattct tcacccctgt tagcatgttt agagatctgg gtgtaatgat
989281 gcgaggggcg gacagggctt tgggggcggg gccacagcag tctctgacca aagcacccac
989341 tgttacttga ctcgtccttg gcgctcctgc aacgcaaaaa aaacagcagg ttacacacaa
989401 acacacccca gtggacagaa aggactactt ctacggtgtt gctaacccta acgggtttct
989461 ggctgcacag agctgaattc agccccgact gcgacagaat aaaacccctc ttccgtggcc
989521 aatcggagcg cggcatcatt gttgacgggt aaacacgtta cctgaagcct gtaatgactc
989581 ctgcttgggt tgctgggata tggctcgctg ccctggccct ccacctgtca cacagaacgc
989641 aaaactgagc aaagcaccac ccaaacccat ggaaacacca aacacacaca caaacacaca
989701 cacgccaggg ccacacacaa acatctggaa aagggaaaca ctccagtacg gagcacgtgg
989761 agacactgat gacacgtctg ctctacagtc atgactataa cggtactttc cccctttaaa
989821 ataataaata aaaaataaac ggggcaacag gaaccagtta caagtcagtt cagaggaagc
989881 ttcttttttt tttttaaagt aaccctcaaa gttttatatt ttacatctca ccacaaaacc

989941 caatactggg cagctcagtt aaaaaatgct tcaaatgtac acgtagaagc tcgttttttg
990001 ggggtatttg agaacagctg taatcaaaca ggccaccaga tgtttcgaga ggagcaaagc
990061 aacaacgtga gctaaaaacc ccagcggctg gataagcagc agctttacag gctgctgcta
990121 aagccagcgt ggacctcaca gcaacatttc acttagttca agataaagga gtcctccagc
990181 atcgccggcg tgtttcatgg cgtcggggag cagctacgat ccgtcacagc catctgaaag
990241 ctgcaggagt ggcgccgagc cgtcctccat gctgctcaga cgaatcactt atctgctgca
990301 cggctctggg gtttggttct ggtacagatg gcagtttaca agccattatt taaaaataag
990361 attaaaaaaa ataaataaat agctggaatc cccccaaaaa aacacactta aatgggccca
990421 caattagatt caggatgaag tggaacataa aaatggagcg gggagcattc ctagcggtca
990481 catgtgggac gtccacactg gtgccggtgg acaccagcac cagaggtgag gacgactgct
990541 cagtgagctc aggtggttta ggagccacca gaaacgcaga gcccgtctca tactagatac
990601 tatggaagga tactatagca tagatacttc tgccaaactc aagcctccac gcacagattc
990661 tgggctccag actgagacat aaacaggata caagaggaca gaaacaggca ccacagtaca
990721 cagctgagat tctggtatgt tcttggagta tctggatcca gtcgcctcgc aaatttgttt
990781 tatttcaatc aatacaagta gaacgattca ttccttcacc actggaacca agagagtgtc
990841 gagagaacag aaaacagagc aaacggagca ggctggagcg ggacccacag gcgcccagat
990901 ccctccggag cagctcaaca ggctgcacgt gcatccaaac cagcgtcagt gcacccactc
990961 gtgtatcatt accttttttta cacactcctc ctcctcctga cgcttctcat agtgagaaaa
991021 gtcatcgaag atggaagtgg tgtgtttata tccggcgatg atcttcagca cctgccgggc
991081 cttgtccaga ggtacctcct gtgtgtcccg agagttggtc actggcttgt tctcgttgtt
991141 ctccagtcga atgtgccgta gctggctgtt gggaacgtcc tttacaaaga tccagcgcac
991201 gtcgaagcgg cccttccact tgtcctgcga ccacacgccg gcagacgtgt tgtagtccac
991261 gggcgagcgc atttccgcca cgccacagaa gtggccgctc ccgttgacgc tgaacaggag
991321 ataaagcggc cccttggcgc ccagagagcg gtacgccgcg tccagccgct tgttgccatg
991381 ctccgtgctg caccagatgt tgtatttgat ggagcggtgg atgtcgtcct cagagtagct
991441 cttgatgata aataccctgc cctgtttggg attccagtca aagtccttgg ggttgtaatt
991501 gttgaccatg cgcagcttct ccaggactgg gtggggctca gaggggaccc cagggagcac

991561 cgccacaccg gaggaggtgg gaggcgactg ccctgccccg ttcccgctgg ggtccccgaa
991621 gccgttagcc cggttccgcg gcgccaccca gcgagacggt tgaggagctt gttgttggct
991681 cggtggtgga ggaccctggg agagaggaac ctgggggatc tgaaccatcc ctggttgccc
991741 gggtggaagc tgatgcgggc ccagctggga aacagttggt accaatggtc cgttgctgag
991801 gggcatctgc gggttcccta cagcagtagc cccaggctgg ggcgacacct gaatgggtgg
991861 ctgcccattg ctgggattga gaggaacctg ctgaggggct gcagctttcg gcatggtgcc
991921 cttgttgtcc cacgtgccaa tgtccatgtt gtgtttgatg ggcggaggag gcaagttggc
991981 gccggccatc ccacccttgg tttttagctt gggttgaggc ttggccggct tgctggcaat
992041 atcagcccag gaggctggct tggcagtggc aattgagacc ggaggcatac tgggggttcc
992101 tacagatgac actgggccga gaggacccgc accaggtaag ccagaaccaa ccactttagg
992161 agccatgtcc ccgttaccac ccggagcggc gccgcccagt ttaagacccg ccatgccctg
992221 gtccaggctg ttcattccag gtgccttgtt aagaggctcg ttggcaccgg gcgcaaacgg
992281 ggactgtcca tcgatcatgg caccgccgag tgagctgggg gcgtaggcgt agctgctgct
992341 gtagccagaa ctctgcggcg tccccgactg tccctgagag ctgctattgc cccacgccga
992401 gaaatcaatt ccaccgggga agaagttgaa accgtgctgg ttgaggaatg ggttgctccc
992461 cagagggcct ggctgtccaa acatagcgtc cgggaggtag tggggttccc cattgctcag
992521 ctgtccatag gaggccaggt aaggcatagg agggtcacca cccgtggacc acgctgcctc
992581 gttcaaggaa tacgaaaatc ctacggaggg gctgtagtag ctgggcatgt aggagtccga
992641 catggccgta taggcattgc tctggacaca gaaacaactg ggttttagtc acattacaat
992701 acattaacaa acaaatcttt cctcagacac aaattgatag ttttaagatt agaagaacat
992761 tttaacttat agaagcagag atcagactgg tatggactga taccagctcc tcctcagctc
992821 ttcttactca taaaaacagt ttaattaagc ttcctgtatc aatttagacc tcctgagtta
992881 aattaatcgg agttctaaaa caacattgaa gctgcaggtc catattaaca gtagcacgtt
992941 gctcagcagc tactggtgag gagacatttt aggtatttct ccaacagact gtaaataatg
993001 cagacacact gcttggcaac acaggtccgt ttctcctttt gaacaactgg ctggtttttg
993061 aatgttgatt ggctgacaat caggaagctt aatgcatcaa ttaatcagca aatattttgt
993121 tacagcgtgg taaataaatc ctgaagccca tcacagccag gaccaggtta ctttctttcc

993181 tctccacggg aagaataaga gaatcacagc agttcagaaa acatccaaat gtgcttttaa
993241 taccaaatac tgcaacctaa ataataaatt gttagtctcg taattgccga tttgctaaag
993301 caagtaggta tcaggtttat tctgatttat tcaacgaaga aagtggccca cattggtcgc
993361 ctagtggaaa actcacctgt cttgcctgag tactcaggta aggctcaaac tcatcatcgt
993421 tcaacgtatc cttttgggtc acagctccgt tttgcactgg gaagagcaaa tctgccgtta
993481 acacaaagtc atcgtgactg tgagcatcaa catgaaacat cagcattctg aatcaatacc
993541 attcaaacat ggatcaactg tcacaactgg tgtgagagcc aaaaatgtgt acacgggtat
993601 agatcacatc agggaataat tagcatctgg ctttgggatt atgatctagc catatactga
993661 tgatcagaat ctaaaccatc tcaccgactc cttccaagtt tccgcgcgtt ggttcttcca
993721 agcgatacgc agggattttt aagacaaagc atcaataaag acgctggcaa aatgtaccaa
993781 tgctacgacc tgaagagtgc ggagatgggg aaacgtgagg cccgttaaga catggaaaaa
993841 gcatgtcgga agacgtgtca tttcgggtta acctagcaac aggctaacgt agcgtgctag
993901 caatgtggtt agctgcgcac agtgcgcagc gtctccatgt taacgagcgc caagggtccg
993961 ttcaacatga cggacttggc caatattatc cgagcagcca gaacggaata cagcgacgtg
994021 gggaagcgag gcgctttgta gtgacacata cggtggcaca ttcgtgacta aaaatgtatc
994081 ctcgcacctg tcgaggcgac aaaacgcgtt ccaaatgcaa ccaggaaaag gatgctgatg
994141 tgtatcgcat tgctatttaa cgttagccag agaggcaata gcaatgctaa ccgtgaggag
994201 cctcggatgc tcactttcag ggcgcccag caaaattgcg acggaggaag accgagagag
994261 caaagtatag aatttccgga ttccaaagtg aacttaaagt aaagacacgt tttaacatgg
994321 gccagttatg cgcatccaag gtatcgcatc cgtgcacctt ggattttcaa agcgcaccgc
994381 acgcaccgtt aaaagacctg cacagacgca ccgcgatgtt caaatgttta tccttacctt
994441 tgttagcttg gcctttcggt ctctggattg ggtccaggtg attcatgaag gtttgattta
994501 tgagaagaga ggaaggggaa aaatagatga agagatattt tttcagcagt cagatccttt
994561 agcaggtggt caggcctact gcactactga gccgctgcct ccgagctaag ctatcctagc
994621 tagccgtcgc gatgctgaga aatttggccc tcgtaaatgt ttagaaaatt gccctcttta
994681 cctgttcaag aaggctgctt gctgacatgt ctttccaccc cggagcctgt gtaagtgtat
994741 ttactcctaa caaaagcacc tgggtgtaga attgatctaa ctatcgcaga cttttcagcc

994801 aagcacccct atctctctac ggagcctact ttctacacaa tggcgggcag
caatttgctt
994861 gacagactac tcaacatggc tgggcggccc tcgtcagccc gcgatggcgt
ttatttaaag
994921 cagacgtgcg ccccctgccg gcgcggagga tcccagcgtc tgcagcggtg
gagggtttgt
994981 tcgtggagta aatgacaaga aatgcaatta tcacgcccaa ttaaatcaat
tctgtaagaa
995041 ataaataagg atattggcct cgattaaagt cagatactga tctgaaaggt
gggtgtttag
995101 gaaatatgta acgatttaaa aaaaatagat ttatttaaga gggtactaga
gcctgagggt
995161 cagcatgcga cacgtagggg aaaaaaacac tgaagtcgtg tataaaatgc
agcaccgatt
995221 tcccactctg tttgtaactt tatccagcaa tccagaccat ggtttgcttt
aagatcaaag
995281 ccatttatgc tcaaaagcat tggcattgat tttaatgggc tttgagggat
tcgaggtccc
995341 tccggaatcc agaacgtcgc taaaatttaa cgagatcttc ctcgaaccat
gatttcctga
995401 aaatttaatc agattcctta aattttattg catgtatgca ttttgacgtt
attttgttga
995461 caaatgcgag ctgctatata acctccttag tcgattaggt ttagtcgatt
aggtttaaaa
995521 aaaaacccaa agatttatta attcttggaa cattatatct gaaaccttgt
cagtagcagt
995581 attttttcc cctggctttt atttgcttcc attcgactta aaacgccatt
aaggaacagg
995641 aaacatatat ttaagatcgc gatttcatgc ccccgccaca aggaggagga
ctcgttgcag
995701 cgatcgacat gccgcccatc agtcacggtc aaaaaccagc ataaatggga
cactattcgg
995761 gattatattc tttcacacag aaatcacaac aattgcaaaa aagattaaac
acccctccac
995821 atttaaacgt gttgcattca gttcatacgg tttaaatttt gggttgagtg
tccaacacac
995881 tgcttattgc tgggcctcgt gttcagcact attcttttct atgtcaatgt
tgaaatggtc
995941 tttacaaatg ctgaataaat aaaataactt taaaaaaaaa acccaaaaca
aggtttgatt
996001 aactgtggtc catcttcaga gtgacaggac tatctatttt aaaaagaatg
ctgcaggaaa
996061 cggaacgagt atctgatggt gagtgcatga atgacggcag tagcagcacg
atatttgact
996121 tatgaaacac gcctaggaca gtagcatggc acgcacggat cacatttcac
ctttcacctc
996181 tcaatcattt ttgggttgtt gacgtttttt ttccccaggg ggttgttgac
tgctgcagat
996241 tggacaggtg gcatagcaca aacaggagct ggacgtctac cagccaataa
aaatagctgt
996301 gggctccatg atgtcactgc tgagtaaatc atggctgctg catatagagc
gagaggagaa
996361 aagatgggtt ctcttaaaaa agaaacagaa acagcttctg tgcgcagttc
tgtgcgtttc

996421 taatcattta aacgcatcta cttctccttt atgtctgatc taatctctcc gtgcctctca
996481 gcacggtccc tgtaacaatt aaatgcctca tgtactcatc cagccagtca cagtctgccg
996541 gcaatgccag aaatgatgct taaactaaat cgggagcctg atctcactcc catacctgac
996601 attctggagc cccgacacca tgctgctgat gtggaacccg atcagtcata aatcagctct
996661 gtttgaccat gtgacatgcc acatttgtga ttctgccctc tgtccaccac atctctttcc
996721 tccccctgtt gccaccgaca cgacccctct ggaccggagc ctccgcagca gagttaagca
996781 ggaggtgaca tgcgtccttt tattcagttg ctttttcatt cttttgtagc atattttat
996841 atcaaacatg caaatgtatt tatatcaagt gaaaatgtga ttgttgctga cctagaggaa
996901 gtgagggtgt cagcgtggcc tcactgacag gttggtagga gcgtcgaacc tgtcgggctc
996961 cctgaagatg caattaatcc actctcattt aataaagtca tttaaattta atggtgataa
997021 tattaataaa ataatatcgt atttgcgtgt agaattcccc tgtctctgta tgttggagtt
997081 cactggagtc cgcaaagtgg ttgtagtttt tcttcatagc agataatcaa ataggggaga
997141 ttctaataaa atatatttag attcattttt tttaaaaagc accttttctg cagactcgtg
997201 ccttagttgc acagcctgac actgtctctc tttccctcag cacaacttag ttgagatatt
997261 tccgtttctt caagtattta tatgttttta ggacagtgga gaagtatatg tgcattcagc
997321 acaatgattt gatgatcaaa caaaaagatt taaaaaataa ttttgagacc attaactgta
997381 aaaacatctc tccaattatg actttggttc agaagtttgg gggttcttta cattaaaaag
997441 atgcgctttg aatttcagat gtaatttaat ccacaaggta aactccggca cgcatcatgt
997501 ttgttttaat tattcatttc atttttaatt ttggaaaaaa ggacctctaa tgtggattat
997561 atattaatta ccgggaaaag acttgagttt ctttgtttag aaatacaact ttagacctaa
997621 acatgttgag actgacatgg gcatctattc acattagcag ttattgctat ctttaaacaa
997681 acattaaagg cacattcatt aatggcatga cttgcattcc atttaatggt cttaaaacac
997741 aggatggccc tttaacttgt tccgctggtg tccagacttg cggctctctg ggctcgggtg
997801 gtccaacagg acaatgagag ggttgttgtt agagagaaaa tagccgcaca cttgtacaat
997861 ctatcaaagc agatttatca ccaacatttg tgtggaagga tgaatgagcc aaacctgccc
997921 atatgtggcc ctttgtgtgt gtgtgtgtgt gagagagaga gagagaaaga gagagagaga
997981 gaggggtgac tctgtttccg atgacacccg ctgatgattg ggaacagctg agatgggtct

998041 gtgtggagct ggacaacggg gcagctgtac atctctgtgt ccgacgtaca cacaggcgca
998101 caaacacaca agtgcataag caacagtgga atcctgaggc ctgacccctt gggaccgagc
998161 cagagtgaaa ccttaataag gaccttgttg tttcaggagg cagcctttat ggatcaggtt
998221 catatctcaa tagcccccat tttctagaca acgaccatgt ggttgcagat cacattccct
998281 tgatttgcat acagtcctca actgttttgt tgtaataatt atatggttat gtaaaggaat
998341 gcaataggta tgtactcatt actttactgc accaaagtat ttagagtgtg gagtataaac
998401 tattaatggt ggtgttgcaa tgtgaatagt cgtctaaata aaaccaggtt gccattacta
998461 cttgtcctgg ctctgcaaca tctcagccaa tcagagactt ccccgcaacg gacccctgca
998521 cttgggggcg gggcctagat gtgctgcagg gtcacaagga gtgatgtaac ccgtgtttat
998581 tttcagcaca tgcacgtaaa tgtagacaca gagacacctc acagacacgc acgcacatgc
998641 gaggaaacaa atgcaccctc gcgcacggac ggtgcagcga gaaaccgagc gtgcacgctt
998701 caacggcgct gacagaaaaa aaacttactt tcgaggtttg ggacctgcgt ttctgcacga
998761 tgctctcgcc gtcttctact tcttgccttc gtctgcaggc gtgtacaaga aggtgcgcgc
998821 gtcgagggga agtcacgctg gagggaagga gagagaccgg cgagacatgc agtcacggcc
998881 gctgatagta atacggggcc tcagttctct tctgtcagct gcagctgttg atcacactgg
998941 tgcaacgttc accatcaacc tgaatgctca aaatcattaa aatggtcgtc agtaaacagc
999001 actggaaaag agtcataagt tggttacttt gaatttggtg cggtatattt agtatggtgt
999061 agaccagggg tcggcaaccc gcggctctgg agccgcatgc ggctctttgg cgccgcccta
999121 gtggctccct ggagcttttt caaaaatatg aaaatggaaa ttgttttaat ataattttg
999181 taggagaaaa acgtgacaaa cattcttaaa gttttcaaca tcccacgata atggaagtta
999241 aacttaaagc accatcgtac agcagagtgg tcacatgggg cgtcattctc tccaggatgc
999301 gcggcaggaa aataaacatt tcatcattgt aactgtctga gagacatgtt aattgaaaaa
999361 ttcagtcaat atttatttta catttttaaa atggtcataa tttcatttaa tgtctcaatt
999421 cgttattgtt gaaatggctt aaaaatgtat tgttctttat gtatgtttcc tttgataggt
999481 cacttgtagg gttcttttca aaagacatct gtatgatgcg tggccattat ttcattggac
999541 cgttgtagct acaatcatga atgctcatta tgtatttgtg gcctacttac catttggaaa
999601 gtaggctaac acagctaata tagacactta ccgcctgtgt tgccttcatt ataaggcttt

999661 taattttttg cggctccaga cagattggtt tcttgttttt ttggtccaat atggctcttt
999721 caacattttg ggttgccgac ccctggtgta gacagatacg cctgtcagaa tatccgtcat
999781 cacctcccaa tccttccaca caacgcttca gtctttgtaa gcaacaggca gctcgtcgag
999841 cactttgtaa aatgatggtt ggaaaagggc tttggtgttc ctgcagaggt tcctcccatc
999901 gccttaaaaa gctgcagcct cgctgtgccg catttcttgt ccaaaccctg gctgcattta
999961 tgctcgttgg ctttccattc attagcaggc acttttaaat ggttattccg tttaaggtgg
1000021 gcggcaaaaa gttttctttc ttctcctaca catcacattt taatcattca ctattatttg
1000081 atttgtcttt atttctgttt ttccacaaac tcttttgaat ttacttactt ttaacaacat
1000141 ttaactttaa aataaaaatc aaaagtttct aaaatgcatg aagcatttga agtaacatat
1000201 ccttaatcac aagcagggag ggaatgaagg ggaataaagg tcattatgaa ctttgtatgg
1000261 ttcaaatatc tcaacagtgg atcaggagtc aagcagaggc gtcagcttgt cggtgatgat
1000321 gatgctggaa cagatgctgc agagcagtca ggaagtattg aaaacccgag gaagaaaatg
1000381 aagagatctt taacagttat agggggaaaa gtcaagagat gccggcaaat acagagaggt
1000441 ccatttcaac catccagcag ctgaagaaag acaatcgaga cgtgcaagaa aaggtgaagg
1000501 aaaactctga ggcgggattg cagggaacag ctccgcagaa taaaaactgt ggaactatca
1000561 tgaaacacct ggaggaccgc tatgacaagt taccatttct ctgtgacaag agtcaaacag
1000621 ctggagcacc acagccaaaa tctggccgag aaacatgaaa cctcgctcac aaaagtcaca
1000681 cagccggacg gaaataatca agacccgctt aaatattccc agagaacatc cttaaagatt
1000741 taaatgcagg aaaaatttaa ggaaggattt ttgcaaacat attgatgaaa atgtttgatg
1000801 aactctaaac actttctcaa caagatcaaa aactgtgcta taataaacat gcatttaata
1000861 taaaacaatc tgctctaaag taccaacgca cattttattc agaggtcgct ggctgagtta
1000921 tcagattgtt ttggaatcac tttctcgcgg tgttttatgc tctttctcca tgacttctgt
1000981 ctcccattac agcacatgtg cactgaccta acagcccgta tcagctgcgt agcagagcta
1001041 aaacaatcca ttacgattca gttggactca actgtgttga tcagttagtt tagcaagctt
1001101 aagaccacaa cagcaacaca ctttgatggc aatattaccc tagagggctc tacacttcat
1001161 ctataaactt gttattccag cgggagccgt tggttaatta tcatttaagc tacaccgacc
1001221 acgtgtgaca tatgcggagc acgatgtcga tcatgttctc catccagaga acacagcaga

1001281 gggaccacgt aacttcctga gaagctcgtc tgctcacata catcagagac attccacctc
1001341 atctgacact cgtccatgga attctgcaaa ttttcctcac tattctccca agcgcatatg
1001401 caagtgcctg tcagaaagac tggcatgccc aaacattaga cacctgtaaa tccccacaac
1001461 aagaaaacta aagcgcgccg gctggggacg cgtttgccct gggaaatgaa gagtttgtcc
1001521 tttgccacga gatccaccat aacctctgct cccctgtcgt gtttgcgttt gacatctagt
1001581 gtgtgaaaat atcctgtgtt tggctactga aaggtgataa tttgttatag ttttgtgcag
1001641 agtccctttc tcctgtgtgt tggtatgacc agcgtatgca acctatggtt gacacctacg
1001701 tgtcattctg ggagcacact aaatacttca gatgacttgg gggtttactt ggcaaccaat
1001761 ggctgtttta agaccatgga tacacttcca gtttggagtg agcgcagttt tcaagtaacc
1001821 accgtacagg gagataaatg catttctgtt tttatatgtt gatggttgag gcttcataaa
1001881 tcacggtggt tttgtcaaat tagattgtga tagtgttgtt agtgtgttaa gatcattagt
1001941 tatgctagtt tatatcagta cttatgacat atagtgtaac tttttattga taaccaaatg
1002001 ctttgacgca atcggttgtg caatccataa ctcgcctgag atgtgtcatc atgggactgc
1002061 tggtggtgat gtgggggatg gagaacagct tagtaaaggt gggaggggtc gacacggaga
1002121 tgtacaggac aggacactca tgcttgctcc ataaacctcg ccgcattttg ccgctgcagc
1002181 tgtcgtggtg cgcagtagtg cgctccgtct ggggtgacca tgtcaaatct gacaaggggc
1002241 ttttaaacac tgagcagggc gaaactcccg ggatggtatc actcgttctg tcagcgtggt
1002301 cggttagcac ggagcagtga agaccccgct catttaccat gagctgtttg gatgttatgt
1002361 acccagccta tggacattac gcaccctacg cgccgactgc tccagctttt atcaacagtt
1002421 tacaggtatg gattttgcaa gcgtttagcc tttttggaag ttacagttat ccagcgtgtt
1002481 agcggggaga agagagatct ttacctaaac ctagcagccg gatcgtaata gtttgtgcgt
1002541 aaaattgaaa acgcatggcg gtgttttcta cgctcgtggc gtttgtaaac tgaatatgtt
1002601 ctgccaaata tgttgcatgt gttgctttat tgttccgccc gcaaaatacc ttgtgaaact
1002661 ctctgtcccc cgccgtcaga ctcccgcagg tgtgagcaga gcttcgactc actgccggga
1002721 ttttatggac actcccaggg gtcccgaggg gatgtctggg gtcccgggca ccggaggatc
1002781 aacttcctca tcgtcttcct ccagcacttc atcgtcgtcc tacacgtcca tgaggccaga
1002841 ggagggcccc aaggagaaac aggaggcccc cgaggcagag tatctgtctt ctcgctgtgt

1002901 cctcttcacc tattaccagg gagacatcag cagcgtggtg gatgagcatt tctccagagc
1002961 ccttagcagc tacatggatg gagagggcaa acggcggcca acagaacaag gcacaggtag
1003021 aaatttctcc cttttaaagt agccatttgt gagaggaatg agcatttact gtgcagttgt
1003081 cgtgtgcatg catctatttt agtttagtac ttttcccccc aacaagattc aacaagatag
1003141 attttatgaa atgctctagt gctacgggaa aatatccctt aaaccgataa gacaagattt
1003201 aagtaaaatt agcaggaaaa ctgagctaaa cgtgcacatg taaaagtgat ttagtgaacc
1003261 tcaaactctg ttttacaaga gatgacatca ccagagggtt aaattgcatt cgggctgtct
1003321 ggcgctctgc tccatccctg aagattcgta accgccgtgt atttttgtga gcacaggtgc
1003381 ctcagttgtc tgcgtggtgt ctggctttgg ggggggggcca cacagggtgt cgataaatct
1003441 gcagagcaca gcagagcatc agacaggtga tgtgatcagg tgacagggtt taagggctgc
1003501 atgttgtggc tgttgtctca gcagaggtca aaggacaggg ttcagaggtc atgggggtca
1003561 ggagcaggca acaggggccc ttctgattcc aaattaaaga aaaatgcagg gagacagaca
1003621 tacgtatata tgtgcgggta aaagttttac atgaaactct cagaaatccc tttttatcca
1003681 caacctggga gcatccatta gatatccatg tagggcctgc agccacgact ctgcccctct
1003741 ctgccgctcc ccctctctct gacactcttt ccttgttcag tccgacgtct tcattacagc
1003801 atgcacacct cctgcctcac aacagatgct atgtttatac atggccgtaa gcgcacagcc
1003861 tcgccggcgt atccccttca aacgtctcgg cggctgccat tctgactctc gctccagctc
1003921 ctgccccaca ttggtcgctc agtcagttgc ccgactgtcc gtcaggttta tatacagtac
1003981 gctgtggtct gtgtgattgg ggctagaagg aaacactagc tctcggggtg ccgcgtggtg
1004041 tctgtttcca gaggaatatc taaatataaa agtatagcgc ttaaaacata aaatggctcg
1004101 ctgacattct gtcaagggcg aggagagggg ggagcagaga aaacaacagg tgggggtgca
1004161 ggagacagaa cagaacagag gggtggttaa tactgtgtgt gtgagagaaa gagagagaga
1004221 gagagatagg gagggaggta gagagacctt tagaaattct ggactgacat gagagtgaga
1004281 agtgtcacca caaccagtga tttaggagcc tgtggtccat aaaccccctc cagggcggtg
1004341 cgtccaccta tatagactca gatgatgata aagggtcttt ttcctacctc tcggtccact
1004401 ctgcactcct gcagggtgtg tttatttctg tgtcttgttg gggttaggac tgtgtgacag
1004461 tatgatccaa tggggaagat atgtgggaca gagggtggaa gagtcatgtg agtgacgtct

1004521 gctttgtagc tcaggttgct cttcttatac acgtattcag ctctcaatgc ctcttcatcc
1004581 gaagccactc gctacagagt aaagctcatg tgtgcttaac gccattccct ccttcctgtg
1004641 cccctctccc cgcagaaaat ccttcccccca gcagccgacg aagcttcccg ccttccttct
1004701 gggatagtaa ctactcctca cctcagagtc gctcccactc cgagatgagc gctccttcct
1004761 attccatgga cccgtacgca tcagggatgc acccaggcct gccgcatccg cacgctcacg
1004821 ctcacgccca cccacacacg catcctcact cccaccctca cccctcagaa agctgggcat
1004881 atcaacaggc ccaggcctat ggccccccgc ggcctctcca tgaactctac tcgccatcag
1004941 ctctggagcc acactacggg cccttgctca tgcccgcggt gaggccaccc cacctcccca
1005001 cattgccaag ccactatgaa gtgagcaaac tggaagctcc tgcctcctgg cccagcctgc
1005061 tttcccctgg agacgtcagc cagacgctgg cactaaacat ggatgcaggt aaccaactga
1005121 ctgtggcaac ctgggaccac ctgttgctcc aaacatcacc agcacatcac catcatcagt
1005181 tgagccttag gcaaaagaga taaagcaaaa acccatcttt aagcattttg tcaagccagc
1005241 aacacatgcg aatcagagtg aatatatttt cagcaggttg tctgcagggt tgtgcaacaa
1005301 atcagcaact aattgtaacg cagatgcacc tgatttttac ccgtccttga gatattgatc
1005361 cggtataata gatggcacac gaacccccttg gacttacatt caaatcacga tatcacgagc
1005421 atcagagaag tccttttacc tcccaagcag gatggctcca tgacaacctg cgctgggttg
1005481 tccacccttg ttctcattgt ttcctcgcct tctttttct ttgcaggcct ccagcagcac
1005541 aagaaaggca aagagctgta ctggttctaa caagccccct cagtcagtca cattgaacag
1005601 tcattaccag atccaattag tccctggacc tgctctgcta ttgaagcctc gcatcccttt
1005661 atccccccccc cctcacacac tccgtcctca tccgtccacc cccctcctcc gtccgcgctc
1005721 tcggcctccc ctgccagtat gcagccttcc aggtcgacgt gtgtgcacca tggagataaa
1005781 gcggagcgag ggaggaagag cgtccctcta atctgcagcg ccgctgcatc tctctctccc
1005841 cccctccctc ctgtgcgctg tgccgcgcgg gccttggcga ggcgcgggca acggtgacct
1005901 gtttggaccg ggaaagacgg gattgcggca gacttttgcg gtggacagag cgatagaggg
1005961 atgaatggag gagcggaagg gcgaattttg cgagcaggga ttccttccag tgcctctgct
1006021 tatctccgcc tcttgttcct catccgcggc aaacaggagc agcaatggcc gcttacgcct
1006081 cgccgttgtc ctcacgaggc cttactggtt tgttaccgta tcaagtcttt ctatttattg

1006141 gtgttcatat gcattttttt tttaaaaaaa gtgtggttta ggccagtgtt
ctaaaacgac
1006201 tgtgactcag acaggaggca ggaatgactg tacggaacga cctcgccggc
gtgctaaagg
1006261 tcattggagg aaaaatacat atcagaaaga tgttggagta tccgcttgct
atgaacagat
1006321 taaaagattg cgtttttttt ttttcaagtt tgctcatcat gttgtgtttt
atctccgtgt
1006381 cgttctgaat tttgactttt tttctgggtg tcattttgca tttgcaggat
tttaatgtca
1006441 ataaatattt ttatggtaaa tcctcgcctc tcgtgttatt gcactatatg
tcattgttat
1006501 ggccatcacc atgtaatccc tctggtaata ataacaaaat acctgcaagt
aatttactca
1006561 ggaggcacca acacttgtta gcaccatatt attgcaaaat gaataaaatg
tgggtggtga
1006621 aaggcaactg gtgttatctg gtttaccatt cagatgaata aataatgatt
attatttata
1006681 ttagggctct acttgttgat ctagtaaggc aatgatgatg tgactcagct
cattaattat
1006741 tcagttgctc tgaagcagca ggggttttct tatttttagc agcgatatga
atccagagac
1006801 gcagcagcac ggctcgacgt gttttactca tcttatttga cagaccttcc
attttgaatt
1006861 cctgctagca ttcacacttt gtcacctata aagtgggaat ggctggtgaa
tgccagcaat
1006921 gtgtaagcag agagcagcgt ggccactcca gctatgcacc gaatgctgct
gcgttattga
1006981 tccgacaatc agattacgtg ctaaggtatg ccagcatggg ccccgctgtc
acaatttagc
1007041 ggcagtgatg tgtgaccccc cctctccaca cacacacaca cacacacaca
cacacacaca
1007101 cacacacaca cacacacaca cacacacaca cacacacaca cacacagcag
acagcagcca
1007161 actatgtggc tggctttaag ctgagggaat acacagagta atgaaattag
aagagtttac
1007221 cggggatagt gtcccctgca ggggcccatg ccagacatcg tgttgtcggt
tcacactggt
1007281 attttcacgg cggcttctct tttcacctca gatcctccag gatcttctcc
atagtcgacg
1007341 tgcctgccac tattgtcgcc ttacattgct gaaatggcac aaaactccct
gaagcatcaa
1007401 tgaaagcccc acagtctgct caacattggt cacctattga tctctgctta
tttgatctga
1007461 ttgatttacg agtgtaaaaa atttagcatt tcaaataggt tcctgcacat
taatgagcag
1007521 ctatgaagtg aaagagttat gtatataagt gtaaagcagt tatggatgtt
taaggacgga
1007581 gaaaatatat ctaatgaaga aattctgccc tcttgtggcg tatttgttca
taacacttga
1007641 ccgtgtataa ctctactatt aactaataat actaataact atcagaagaa
tataaacaca
1007701 caaaataatg ttcagttgag attttaaatg acagaaaaac accttagatg
ccaagtctaa

1007761 atgtttgtgt acattgatct tgacatgtga tttactcatg tgacgcaaaa actcctgttt
1007821 atgcatgaaa ccagctttta cagtaagaca aaccactggt gtcattgacc cataatgaca
1007881 ccagcgacac cagaggaaac aggtcagtcc tgcctaagga ataaatacat tcatgatcgc
1007941 aagaggaaca aactcaaaac ttattaaatg ttgctcttcc agcatgattt ttcttcctca
1008001 aaagtcatgc aacccaaaat ctgttttttt aaagcaattt taaacatgtg aagcaaaaca
1008061 aatataggac acattacata attgattatc tgtccctaac ccgagttagt taatgtgtca
1008121 tttctgagtc tgacactgtc acatgtaatc tgaactaaag tggaattatg cagcaactca
1008181 tgtagaaatg gtaggaaaat gtcccattag ctgctcttga gaccttcctg ccttgttaga
1008241 tccattagaa aatgtgaatt tgcttctgaa ttcaaggatc tgggttggca aaggtcacca
1008301 caacagcagc agccttgaac tctgaagatt ttccgttgga atttacattc tgctgacgcg
1008361 gcagggagtg gaaacgaatc acagactcat cagtatcagg tctttttttt aagaggaaca
1008421 aaaaaaaagt cttaacttga atcatctttc tggaactaca gaaatgctgc agaaatctgt
1008481 actgtagata ctgctccctg aaccagaatg cagcatcttt acggttggaa acatcgtccg
1008541 tagcttggct ggcaagtaga gttaataaaa cataaagtag ttcctgtttc agttgcgtgt
1008601 gtaatgtctg ctcaatgttc ccgacaatga gatgagatca aacacttgaa ctgaaaatga
1008661 aaacaggatg agggcatgga aacaaaaaaa gaagaaaatc ttctgaaaca aacaaaggaa
1008721 acgttcatga ttaatatgat tcacaagtgg tgtgtgggtt cctgtcgaat gttagcgtgt
1008781 gtcagcacac acttttactg taaatcgcaa agtctgtgct cccctttatg atacgcacgc
1008841 tgacttccta tcacaatgac ggagcggctt tttttatgag ctgctgacag tctcctgact
1008901 ggacatgctg caggtcataa cggaggctgc gtataaaaga atagctttgc gtcgacatcc
1008961 tgtatccccg gcacacacac agaggcaggt aagatcagtc acacacacac ggaggcagag
1009021 gtgtgcgcac ttcctgtgct gtgggaagag aagaagaaaa ggctgcaata gaaagacata
1009081 aatagcttag agaacgcctt gagctcacag ctgaagccaa acgcagcttc acctctgacc
1009141 tgtctgtctc tgacctgtct gcctctgacc tgtctgtctc tgacctgtct gtctctgacc
1009201 tgtctgtctc tgacctgtct gcctctgacc tgtctttctc tgacctgtct gcctctgacc
1009261 tgtctgcctc tgacctgtct ttctctgacc tgtctgcctc tgacctgtct ttctctgacc
1009321 tgtctgtctc tgacctgtct gtctctgacc tgtctgcctc tgacctgtct gcctctgacc

1009381 tgtctgcctc tgacctgtct gtctctgacc tgtctgcctc tgacctgtct gtctgcctct
1009441 tctctgcacg cgttgtcagt caggatgaag ttgctgctgg ttgctgctgc tgctcttctg
1009501 gctgtggcca gctgtgacgg catgtccctg gaagaaatgg agttccacgc ctggaaacta
1009561 aagtttggtg agtctcctct gcccgtctgc gactccctac tgtcctcaca agagtaacga
1009621 ttcctccctt tataggaagg tcctacagaa ccccatcaga ggaggtccag cgcatgcaaa
1009681 tctggctcaa caaccgcaaa ctagtgctgg tgcacaacat cctggccgac caaggcatca
1009741 agtcctatcg tctgggcatg actcagtttg cagacatggt acgcgcacgt ggatgtttct
1009801 gccatctttg tgactcacca attggctctg taataagtga caccacctcc ttttgaccag
1009861 gacaatgagg agtacaaaag cctcatttct ctaggctgcc tgagagcctt caacacttcc
1009921 gcgcctcgcc ggggctccgc tttcttccga ttggctgagg gcactcacct gcccaccact
1009981 gttgactgga gggataaggg atacgtcact ggtgtcaagg atcagaagca atgcggctcc
1010041 tgctgggcct tcagcgcagt ccgtccaaca caaagagcct tttttttgt tcttgcagga
1010101 tgggttttaa cattgaaata tctcctttag accggatcgc ttgaaggcca gaacttcagg
1010161 aagactggga agctggtgtc tctgagtgag cagcagctgg ttgactgttc cggagactac
1010221 ggcaacatgg ggtgtaatgg aggtctgatg gactacgcct tcaagtacat tcaggagaat
1010281 ggagggatcg acaccgagaa gtcctaccct tatgaggctg aggtaatgat cgagatgact
1010341 tcagagtcac aatcaaaagt ggagacagca acccacgtcc tctttcagag ctgtgcaaca
1010401 cctccacctt gtggatgcgt gacaatttga cgcagcctga aatataaatt gggatttctt
1010461 ttctctttaa ccaggatggc cagtgccgtt tcaagcccga aaacgttggc gccaaatgca
1010521 ccggctacgt tgatgtgacg gtaggcgatg aagatgccct gaaggaggct gtggccacca
1010581 tcggacccgt gtctgtgggc atcgatgcct cacattcttc tttccagctc tacgactcag
1010641 gtcaggagac gcagcctcgc cgtacagtct gagttttctt ttagaatgca gcaacagacc
1010701 cttctgacct ctgtctcctt ctctataggc gtgtatgacg agcaagactg cagcagtcag
1010761 gatctggatc atggcgtctt ggctgtgggc tacggcactg acaacggtca ggactactgg
1010821 ctggttaaga acaggtacac cttttgataa aaaactctca gtaatagcat taaatatcaa
1010881 tattagggct aaagaaaagt gacactgttg ctgtaagtgt taacgggaat gtgtgtttgt
1010941 gtagttgggg tctaggatgg ggtcaggagg gctacatcat gatgtccagg aacaaagaca

1011001 accagtgcgg cattgctacg gcagccagct accctctggt ctgagacggt gagccgaaaa
1011061 cctgccctct tttccatttt ctgccttttc actctatttc tgtgcattca ctggtttttg
1011121 tccatgtctt ctggttcaga agattcacca agatggagaa gaagagtcca cgaacaaatt
1011181 cttaataatt ttgcagattt cttgcacaac agattatttt cagggtgaat gtttttgata
1011241 attactttta tataaatgtc ttatcgaaat gtagacaaaa taaaaatgct aaacaatgaa
1011301 aaagtgaaaa cttgaattta ttcgcaaact cagatattca gtcaccttat ttaaaaacat
1011361 ctgtatcaac gttgatgttg tctactttaa aacatgatat cagcgtttta tcttacaata
1011421 actcaaacct gatgaaagga tcagtcgatg aactcgagac tgcggatgca gcaatcaggt
1011481 cctggaactg ttccctgctc ataatcatca taatggacca tcccaacatg gccagttatc
1011541 catctcatta gtatgtaaac agctctgcag gtttttttag cccgactcaa aacagcaaaa
1011601 tgggttttc ccacgcctgc atccacggct ccagtctgaa agttagttat gctgtaaaaa
1011661 gtaaaaatta cagccattaa atgccaacgg gtgatttaaa atccaacaca atggacccag
1011721 tagtgaattt gtgggattta tttgaattca ttaaaggttc ctgtgactgt aaatgacagc
1011781 tgcctcacat ttgggcatga aaaaagttcc catttaacag attaacttca ctaaaatggt
1011841 atctgttgga aaaagcactg agcagaatac tcacaaatgg gcttttacac tcacggcgac
1011901 ctttgtcttc ccgcagttac cgacagacct tttcttgttc cgttgagggc agatagtgtc
1011961 ttcacccacc cgaatctgaa aaaaaacaag agttttaaga tggtctgact gggttcaagc
1012021 cagtcaaaga ggtggatatt tccatttttc tcctgattgc tggtgttctc tgcctctgct
1012081 tggatgtgtg tgtgtgtgtg tgtgtgtgtg tgtgtgtgtg tgtgtgtgtg tgggcgtgtg
1012141 tgtgtgtgtg tgatcttcct ggataaaatg ctttcagaag cagtgttgtg gtttgttaaa
1012201 ggttatcttg aggaaatgaa agcggatctc ggtggagaac gtgaacctgc tgaagaacat
1012261 cagagccgaa gaacgagtga aatcagacac ttttgtggtc cctcgcgcct ctgatgcggc
1012321 cagtaaaaac tccattatgg atttgggcga cgacatctaa tgaaagtgtt ctgacctctg
1012381 ttccagtgga tacgttgggc cctcctcttc ttgggcgccg cctgattcag ccgctccttc
1012441 agcgtggccc tcggggccga tgaccctggc tgatactgct gcagaaagag aggagcatgg
1012501 gaaatggtgc ttcacttgtt ttcatgtgga ttacttgaca ctgcggggaa caaagaataa
1012561 attcccataa acagtggggt aattaaatgc cgcgtgctgt aactacattt tctgaaccat

1012621 ttagtggctg gaaaacaacc aggatgcagt ttcagctccc tctgacacaa agtggccttt
1012681 attttctact tgatattaat gtgctgctaa aggtgcttca gtgcagttca gagctgattt
1012741 tgctgtttgt ataagctaca ggagccgatt ccacaaaaag aagacacatt ctaagttagt
1012801 gcacgtccgt ttctgcccgc taacaaggct ggatcgcaga ctaaagaaaa cggctgctct
1012861 cttcccagac aacagtcgcc gccatttaat tagaaaagca aaatcacatt aagtgattgt
1012921 cattcttccc cattctaaat gccacattag gtttgatcgt gctaatattt aatgagaagt
1012981 ggaaaacaat gacgcaggga cactgagagc agggctgctg ctggtggggg ggaggcttat
1013041 taaggcttta atcagcttgt atgacaaagt gtgaccttga ctcttcattc tttcctcctc
1013101 agcagctctg caaactggca tagcccgtaa ttgaaaaaga gatttatatt atctttattc
1013161 agatgactct gcagacctgg aagagagaga gagaaaaaaa catttaatct gtatgggatg
1013221 acagcagtca gccaattagg cctgaatgac tgttatccca agacattatc gccatctcgc
1013281 ctttcatttt tgtaaatatc aaggagtgtg ttccaacctg gggagagcag agatgaaaaa
1013341 tctcccagaa agagacaaag ggaagcggaa ggagggtgcg caagaggtga ggcaaattaa
1013401 ttggttttgc taaatcctcc tcaaacagca gaagccaatg tggtttcaca aaaaggccat
1013461 taccagctat caatttaaat ctgttttacc cttttcaagg aaaccaatta agcaatttaa
1013521 tttggccttt ttgattcacg gtgttgccca ctacggagga tgggacatcc gattggcgtc
1013581 ctgcagggta ggacaagtga aaacactggg aggtgttatt gcaacaagcc aagctattga
1013641 ttgagatcca gggagggcag gaaggagaag gccatagact cgggattgga gatttagtca
1013701 gcaagacaaa agtagccctg ctgtgtccat ttaaaacggt ctcaatcact gaagtgtaat
1013761 gcaggtttat tgtcttctaa tctgaattta gagccgttgt atttgtttaa aaggtgtcat
1013821 tccctgttct gccactagat ggcactacta gcctgcttat atttgtcgcg agactggaat
1013881 catgcaaacc tctgttggat atgagtcgtt ttcttttcat tcttcctctc gaatgatctt
1013941 gaaggaaagc atgatggata caagggacac gttttcacgt atgtacacac tttccccaca
1014001 ccagtggacc cattaagctc tgacaatgca cacatcatgt actgggcgtc tgtctaatca
1014061 acaatatcat ggcgcagttg tgtagatggg agaccagtca ggctggaggg aagtgtcagt
1014121 cagctgtgtc acctatagca gagagcaaac aggctgtctc gagggataca gatctgggcc
1014181 gcggatgcct ctccacccat gcattatcct attctcccat catgctttac aggtgcatgc

1014241 atctctttgt ttagtaagga catgtttagg aagtgaggac aaatatttgg ctggtcgtca
1014301 catcttcaga ctctctgagg gttaagacgt gattctaatg ttaggatgag ggtaaggtta
1014361 agggttaagg ggtcagttag gatgttaggc taaggagctg gggactgcat gaagatgcaa
1014421 gtcctcagaa acatagaagt acaagatgtg tgtgtgtgtg tgtgtgtgtg tgtgtgtgtg
1014481 tgtgtgtgtg tgtgtgtgtg tgtgcacaat actgaggcag taatacatgc agaaagcatc
1014541 taagatcaaa gctaaacaaa gaatttgctt tgatatttta taaatacatc atatttcttc
1014601 agaggaagag catgttctat atgacctcgc tgtctgcctg tgttgaaacc tgtgtgtgtc
1014661 cctcgtccct cttttctaca tcctccttca accagctgcg attcttggaa gggagagagg
1014721 ccagagaagc tatccggtcg aagaaaacgg ctctgcttgg gccaattacg cccagacaaa
1014781 gcactttgca tgttattgtg tgtgggattc atatttaaat gactgaatgt gttctaatga
1014841 ggtttgggag caatgtgcaa ggaaggaaaa aaagcgctcc cccatttccc ctgacagact
1014901 gatccacccg cgtgctcatg taggtcaaca catgcaggag agacgcacag atgtgcgatc
1014961 acatcagctg cgagcggcgc gttaccctca cacacaagtc aacagcagca cacatgtctt
1015021 gcaaacagtg ggcggcagat gaagggatgg caggaaggga agttgctgag ggtccgatat
1015081 gaaagcccca ctcccaccgc cattctttta aagcgacaga tggattgata aagctgccag
1015141 cgtcgataaa aactgtcagc tgttattcct tttgggtctt ggctttttct aggcgctgca
1015201 ctcaggcttc acggctactt gtagccacag gctgttgggg ggaaaagcaa cacatttagg
1015261 attaaaacaa ggacagaatg caaatataaa atataaaatg aacaactgac gacaaaaggc
1015321 tgcacgtcta tagcacgtta gcttcctcgt aaagtttcct ctgagtgaac gttaccatta
1015381 acccgctcag aaaggccccc tagtgtgtca cagttttccc tattcctctt ttgctcaggt
1015441 cttcctcttt cctccctctc attcatcatt tcttgttcta cccactcatc ctccccccgt
1015501 cttcacctct gcctcatcct cctcctgtgc ggtgccgagg tgagggtgag gggtcaattg
1015561 cctgggctca ctgccgagcc ctgctctgtc ttatcttttt ttgtgtgtgt tattttgcct
1015621 gacaactctg tcccaaagga gcaggattca ggttcagcct ctgcagcacg ctccgtgcac
1015681 acggactgtg tgtttcctgc atgcaggacc gtctcgcgct tccacagatc cttgtttatg
1015741 cgccctctcg cctggcttct ttagctctct tcaatcgctg accttattcc ttgctctgcc
1015801 tttccctctc catcaatagc aggagctaat aaggcaatta aagcctaatt aaaggagtca

1015861 ggagagagag tgaagggctc aggtaacttc atgagggcga gtgattgctg tgtttacttg
1015921 ggtttctggc gagcgctgcc ttctgaccaa cacaacaaaa tctggaaagt gcaaagctaa
1015981 caaaggctcg agggaccgta catttaatcc catcaggatt ctgcatcttt agactttctg
1016041 tgtgtattta ggaggcatcg atacaagggc gccatgaggt aacaagctaa tctgtttagc
1016101 tcagagcgca cttatcagcc cactttttgt tgctagcacg gggtgatttt gcagcacgtc
1016161 agccaggtta cctccgctcg ctgctctgaa tcaaacccgg ggattaatgg gggaggggggg
1016221 gggggtgcag cgatgcgtga cagatgatca tcctctcctc gggacgggag cgggtcacca
1016281 gggtctcggg tcagcgttgt ttatccacaa acatcccaaa gcttccatta cgcctcctcg
1016341 ctcctcgatc actacaggca cttgtagtct gaaacaaaat ctttggccct gatttagaag
1016401 gcacttacaa tgcattagct ttgtggtaat tactagaaac tcaattagaa gaagccaccg
1016461 aagaccaaga ccaaacggct taggctgcaa cgctggtaaa tggatttgga gttaatttag
1016521 tgagtcagag ggtgaaacag ccatcagtga tgttttaagc cacagccact gacctgcgca
1016581 cggagacgtg ctcgacattc gggatccctc tgagtgttgc actaaactgc agtgggactt
1016641 tggttcacac gcacgtgcgg cctccgtcat tcatcattca tgcaacggtg cattatttct
1016701 tagtgatgtt tacgcaagtg tagaggtcac gttctcttca gagtgaactc acatgtttta
1016761 tttatagatc tttctatgtt cttgaaagcc cttctgggca cgggtatgac cactggcgct
1016821 gcacactgcc gtctactgta ccgctgtgta tgtaatgttt gtaaatttgg tgcatttgac
1016881 agcttataaa tatgtttttg aataattgta gaactgctac atggaaaaaa acacacagaa
1016941 agaaaataat atgaacagaa aggcaaatgc tgcgaccacg cacagctagc aacaagagct
1017001 gatgtttgtg agggacatta ataagtgatg gacgtctggg acgggctcgt cctgactgtc
1017061 cacaattata catcactcat cagtcgcggg gaaaaataaa ctaaagtgtt gccaaagttt
1017121 tgtctcgctt gaatctttat tgatgcaact gcagaatttg tatatttgtg tgattttcag
1017181 ggtagattaa cctgttttaa cttgatttca tgtgatatag atcatgtggt gttgatcatg
1017241 tggtatatat agatcatgtc ctatagctca catagtgttg atcatgtggt atatatagat
1017301 tatgtggtat atgtgaatct tgaagttcca cccttgttgg tattttactc cctggtgaaa
1017361 acatgcactc acatgcactg aatttaacca aacaaagaca aacaaacaaa atgatgataa
1017421 ttacgttaca cttctgaaag ctacattaag ctttggatta gattggcagt gtctgttcag

1017481 cattaataat aatgctagta gctccagcga cgtgtctacc tgaagctaac
cggcacagag
1017541 aaccagtaag tcgtatcact tattagtttc tgccacccga acctgcagag
aaactggtgt
1017601 agcgagcaga tggcgcgcac gctgccgaaa agagtgggcg ggaagtctgc
atgtgcgctc
1017661 gtgaatgctc ctatccgtgc cagcgtgtgt gttctttggc gtatgtgtga
tcatgtaggt
1017721 gtgtgtgtgt ttgtgtgggt ctgtgtgtgg gtctgtgtgt gggtctgtgt
gtgggtgggg
1017781 tgcatagcgt gagtggctcg ctgtgcctcc ttctcctcct ggggaccaga
tggcgatgga
1017841 gtcggctgct ccaaaaccaa tgacatcata gcttaaagac aatgagggaa
agatccataa
1017901 actctcacgg gccgtgttgt gatcgtctct cagaactcca accctcatta
attgtacagt
1017961 tttgccaaag cagctcagga tatcagctaa agccagattt tacccgcctg
acatgttctt
1018021 ccagtgaaac caatgggtga catcattaag gttctgtcca ggtttcgtac
attaaccaca
1018081 tacgtctttt acggctgctc atattatttg gtgaactgta gtaaaaacca
acagaaacct
1018141 gaaagcggac ccaaaggaac ctctaagggt ggtagaaggt ggtagaaacc
accttcacag
1018201 gatcctcctg tctttgtttt aagtattttg gatgaagatc tggagcccac
ggctgaaaat
1018261 atccgataac gctgctattt ttggctcatt tatccacagc attcattaga
aatgtttggt
1018321 ttcacggtgg aggagaaccc gtatccttct gggtctgtta gtccaccgct
gctcacttac
1018381 gcgccatcgt tttaacgttg tttttccgcc tctgaaatga caaaagttca
acgcagatgg
1018441 aattaaatga tgcgtaaaaa acacagattt catttcggat aagtcagaaa
atgaatggga
1018501 cgttattccc gtgccctcgg aggggactgt aatgaaggtt ttgcttttgt
caccgacgca
1018561 gtgacatttg aagctaattg aagtttagga tgctgtcgaa tctgcggtgt
ctgtggttta
1018621 aatgcaataa ttaatatccc tccgtgctga tgtgggtaat atggaaggta
aacaggaata
1018681 attaccgccg ctgtgaggag agaagatact actgagacgt ccaaaagtcc
agtttctctt
1018741 ctaatgatgc taacagggac cacggctcca tctgctgctt tttcttgtgt
ttcctatgtt
1018801 accgattaaa gataagaaca aaagtccatt tactctaaat gcaattacag
ctctcagatt
1018861 agcgagaaaa cataaatgta attatctgtg agatcattac gccactgttc
aaggacagga
1018921 tttgccatat taccacgtca ttcagggctc ccgacattcc acaacacaca
attaaatctc
1018981 cttatgctga tttctgagct ggtgccagga aaaaaaagag ctgtgctttt
ctgtgagatc
1019041 taaaatttta taagatggtc gcaaatattg ttttttttccc caatcaatca
ataaatataa

1019101 aacactcaaa tatttcttct tcatactatt gaaagcagaa atatttcgtc
tccacgggca
1019161 gaaacagctg gcggaggccc agacaaacag ctgcagacag gaagttccat
tagaaactcg
1019221 accaccactt cggtttttc tcttttaaag gctcaaactg tgtaattaga
ccacgaccgc
1019281 ccgctccgac attcaaaagc acacaaacgg agtcttttc gtgggtttgg
actggtccaa
1019341 tctttgctgc tgttgtggct tcaacgtccg cgtcagtgtg gccacgttcc
tccaatcatc
1019401 tccggtttcc ggtagattgc cagtcagctc acgtgtaaag gtcgtacgtg
acggttaaca
1019461 agcgtcgttg ttctggcctg cagctctgag agttgctaat tgagcaacat
cctgcaggag
1019521 acgttccttc ctgtgactaa tggagcaggt agctgcaggt aggggccgac
gcagggagag
1019581 acgcttcagc gtggccacgg ctaaatgtga cctgtggtgg acgcctcctc
aaacgggagc
1019641 gccccagccc atcgcctcac ctcccttccc cggctgatgc gcggcgctac
ggccaatcag
1019701 ccgctcacac aaaggaggaa ttagtatatt ctctggcaca aatgtgctcc
cctaattgcg
1019761 tctgcgccgc gcttgtgttc tccacacctc cgcgctgtct cacctttttt
tttctctccc
1019821 ctctctctct gctgtcatca gcctcttcaa gtgtgtgttt ttcctgttat
tcttgcacat
1019881 ttcactccgc tccccttcca atcattcggt cttcatcagg cggctattgt
gtgagctcag
1019941 ggggagaagc gggaggattg acgggggccg ggtcctcatt tggcaccctg
tgctgtagga
1020001 acaaggttgt cagcctcaaa tgatgctctc ataatcagca cgttggagcc
ggcgcgccat
1020061 tctgtggatt tctgtggctt cccttttggc ctgtcaatct ttctttatct
ccctctgaat
1020121 agacatctac tgtttgcatc ctggtggttt tggtttcctt tgtgctcttc
tctcttttcc
1020181 ctccattcct ggcgttccct ctattgtttg ccacttccac cttctttaac
actccctcca
1020241 acactgtatc ttcatctctc ctggcacgcc gtcacgtttc cgaagtgctc
cgtctctctg
1020301 tgatgccgcg tttagctcgt ctcccagcgt cgctgttgtg gcggcggcgt
tcaatctgcc
1020361 agtcaacgcc gtggcagaga cagcgtcact cagcgctttg ttgagaaaag
aaaagaaaag
1020421 aaaagaaaag aaaagaaaag aaaagaaaag aaaagaaaag aaaagaaaag
aaaagaaaag
1020481 aaaagaaaag aaaagaaaag tagtagtaac gggaaatggc agtcacattc
caggttgtaa
1020541 ccagtcccag gtggtttcca cggcagcggg tctgggagaa gccgcggcct
ctgcgtctgt
1020601 tctgcaggga tggggggatc ccggcgatat gatgccagcg tggctcaagt
gcttccacat
1020661 caaatattaa aagcatatta ggacgtttta ggctctgcga tgaactgtgt
cgagcttaaa

1020721 aaatgactta ctacaatatt taaaacggaa gaatgaatac tgcagaggac gctatctgct
1020781 gtaaccctgc atgtggagcg cgttctcata ttttacagac accaggcaaa catgtgtgaa
1020841 agtgcaacct tctgtaggaa actaaggaga cacaagaacc tggtttgatt aaataacagc
1020901 aatatttcct tggtccaggt cacaggacca ccgaacggat gctaaactgg tgtttaccct
1020961 gttagcatca gcgaatggca cgtgtgaata tataaatgat attcacacac atcttttgtg
1021021 atttcagacg gtttccagcc aggataatta aatcctaaat atattcttct ctctgtcata
1021081 ttaagagaaa acagctcctt tgttgtcacg tttccaccgg cacttaaatg gtgtcaggcg
1021141 attgttggag gtcgccagaa caatttgaaa ataaatgtaa aagatcatga aaaggtgtca
1021201 aacaaaagaa gtttgaggac acattaaagg gagaacattg aaaatagtca taaaagatgg
1021261 aagcgatgga tctcaaggca cgttcccctt ctctggccag tttgcccagt aaggttgcag
1021321 ataaatcttc gtaacaatgg ttctgctaca tgagccggca gcacaaacag cctctttatt
1021381 aaaaatgctc gacaggtctg gaagctaaag gccgattagg gcacaataac gggtccctgg
1021441 tagcttggac gcatggctgc tgctgcgctc taataaagta cgagctaatt ggctaattac
1021501 acttaacaga aactgctcac cagtgctttg cttatgggcc tcatctcaaa ggcatcactg
1021561 gggtcactcc tatggaagct agctccacga tgatcacatg accgctcagt gaccacctga
1021621 ccactccgta gtggctgcag gaccactgtg attggtcaca gctaagcggc taacttacta
1021681 aaagtgagat ttgtttcccc tgaatgtccc ccttgacccg cttccgtctc tttcttctgc
1021741 tcatgatgca gacggagagc aagagagtgg gcagaagcag ctgacccaac aggaagtctt
1021801 tgacagggag cctcccagca ggtagcaggg gtgcaggact ttgacacaga aacagagcgc
1021861 gtagtcatcc atcaccgtgg caaccgcgcc tgtcagggag gcaagcgcta gggtacaaaa
1021921 ccagcgctgg ggcagatggg aagggagaat gtggcccgaa gttctctgcc taaggagcct
1021981 cggtcagaga caggacagca gccagaatgt cacatgcaca cgtgcacacg tgcacacgtg
1022041 catgcacgca cgcacgcacg cacgcacgca cgcacgcaca tacacgggga aaagtgtgca
1022101 ttgaccgata gccttccctg tccgtctttc catagtgcag accaactgtc tggaggaagt
1022161 agatagaaca cattcatcat cacacactcc tgtcttggag acctctaaat gagtgtgtgc
1022221 ttaagcaagc atacgtgtgt gtgtgtgtgt gtgtgtgtgt gtgtggtagc cctctcctcc
1022281 tcccgtgtga agacagaggg agcaccacgg tggtgtgttc gagagcttct ctcgccttcg

1022341 tgtttaccag cactcacaag tctttgagtg tgtgaaaaca tccagacgag gcctgttgac
1022401 ggcgtgcgcc cgtgcgtgga aggttgctga ttaacggctc ctccaaagag agcgtctttg
1022461 ccgtctctgt gtgtttgacg cgagtgtcgc ccaatctcgc acggcgccgt cacacaatgc
1022521 tcgaagctaa tcaataacaa tcagacgtat tcaaaggtgg ccggagcttt gagcctttgc
1022581 cccccccaca cacacacaca ctctgccatt ttaatcaaaa aaaaaaatcc catcaaactc
1022641 acaattaagg ctgattacca tcatcgtctt cggcttgttg tcagaagatt agggcaacac
1022701 aaacaaaccg ttagtagcca gattagcgac agagcaaaca acgcggcgtt gtgatgctat
1022761 gttcattttg ataactgcct ctgatatcgt ctgtggcgtt cagacacggt ccctcagtgg
1022821 caaaattgtc agcaattgtc aactgctgtt gccctgtcaa ccgtcgttgc ccccgggggc
1022881 caaaacggcg gagtaattct tcagtgattg tattttattt acacgtggca cacagggaag
1022941 agcttccatt tcccacaaat gactcctccg ccggatccgt ttcaccgtgt cagttttgca
1023001 ggagtcgacc gcttcattta atttatacga tattgttttt tttacagtgt aaaccccggt
1023061 gcttagagtt gacagaagat taaaacctaa ctcagtttgg agccttgggg ggagggaaga
1023121 ggcaattatg agagcctctt gtttcttctt aaattcccgg agagcaatcg acagtcacct
1023181 gatcctctgt gatcacctga ggtattactg gggggggggg gatcttcctc acctttgaca
1023241 gtataagaaa gcaaaatctt ctgaatacaa taaatccctt ctccaataat ctgtcaccag
1023301 actggaagag atctttgatt tctgccattt attggttgtg acggagcagc ggatgaagct
1023361 gagatgattc cagacttgtg ggaataatct cagggcggcg ccctctgatc gatgaactcg
1023421 tcaatacacg aggctggctg tatgtgtgtg tgtgtgtgtc cctgtgtgtg tgagatgtag
1023481 acagttataa gttgatttta gtgatttcct tttcacaggt ttgctcgcca agatcagctg
1023541 attagttctt ccaccatcat cacccaacag aactcttcca ccagaatcgc tcgcttccgg
1023601 aatctcgtcc gtaccactaa atgggctgcg cctcatctgg ccacgcctac tcacatggta
1023661 ccggagcgtg tgtgtatata tatatatata cagtaagtgc caaacagact ccagcagcag
1023721 aggcgctctt atctgcggcc ctctagtcca ttaaagctct taaaccatca actccacaga
1023781 cgccgccgtg gcggctccat taccggagcg cgagggcatc gcggctacat cgcagtaatc
1023841 gcagcagctt gtcactccag ctgtttgagc gatgctccct tgagtgtgtg aagcttgtgt
1023901 ggagaaacgc gtcggcccaa acggctcgag tgcgtacgat tgctcaggaa tgaccccaca

1023961 gaggttccac agggacactt gaggacgccg aagaacctcc agactcccat tgttgcagct
1024021 aactagctca gaatgctact ctgtctcgta tcccctcaca tcttcgttaa ttacagcccc
1024081 agctgaagca ccaaaaggat gttggactca ttgctaaaag cttctgagtg tctggactga
1024141 ggtgtaaaga gtcaaagagg aggagaacgt ctgcaggcat ccttgcagtc cagtggattg
1024201 caggacggat gtcccagagg taaaccttca gatccagata tcccagcaga tccaaatgcg
1024261 actgagcctc tccagaaagt tcccgaatat taataatcca gaggcagatc acacaaaatc
1024321 cgctggattc cttcaagaac aatcctctca cgtcacccgg tttaagcttg ggagccgccc
1024381 actttctgga gttttcgttt aacgaagcag ccaaattacg aacttccagg tttgatccag
1024441 cgagcacatg tggctccgtc taaaaatagg cagctattag cacctgagtc tagagagaga
1024501 gaacagcaga cagattcctc gtatacgttt acacgcttgg gcactaatgc tgatcctgct
1024561 agaacacgag gggccagaca gaagatctat gcagtgttgc tcagtgttgc tgaggtcacc
1024621 cgttcagcac cggtcaaagc gatgggctcc atttctgcct cgtatggctc tgaacaccaa
1024681 caaaccttaa aggaggtcat tagcctgtct gctagctgct aatggcaaat ctgacagcta
1024741 tagctttgca tttagacaga gaactgagtt ttaattgaac acatctgcac taaatgatga
1024801 gaaattaaag caaagccacg tttggcatga tctggggggg gggggggggg ggnnnnnnnn
1024861 nnnnnnnnnn nnnnnnnnnn nnnnnnnnnn nnnnnnnnnn nnnnnnnnnn nnnnnnnnnn
1024921 nnnnnnnnnn nnnnnnnnnn nnnnnnnnnn nnnnnnnnnn nnnnnnnnnn nnnnnnnnnn
1024981 nnngcagccg cggtgtttct gctgaaacaa caaatcccgt cctgtcatta ttcctgcgca
1025041 tagcaaagca gccgtccaca cagaagagcg cagccgccgt ctacctgact aatattctca
1025101 tttatacgtc ctggaaggaa tctagcaggg cgccatctgt gagcggcggc ttccactgct
1025161 gcgagacgct gacgccaaat gagcagcaca gctataaata ctgcacgcca tgcagggacg
1025221 aggcgccgcc gactggacaa agaagctaat gagattgatt gattggatgc tctcgttttt
1025281 aattgcctta atgaagaatt aataccgccg ctgaatattt gtgggtgtga taataagtta
1025341 gcgttgcgcc attacaccaa aggatatgtg actggtggat gagcctgttg tcagctacct
1025401 gctgggcatc gaaacgccgc taacaacgtt gcttttatta gcgtggaggt cacacttctg
1025461 gcggcacgga gctaattagc ctggagcgcc gcgcgcgcag cgctccaatt attagctgat
1025521 gtggcctttt ccagagcagc acagacgttc tcctggctgc tgcctgagcg ctgctccgtc

1025581 tgagcggcgg tgacaggccg gccgtcggcg gaacatcggc cgcacggtga ttgatggttc
1025641 ccaggtgctg agtggaacag gcacatttca aatctgtctg cgacgccaga tcgggggact
1025701 gacagcggcg gttgccttgc acgctaaaac cctttataag gaataaagta aaatgttatt
1025761 tggctcaaat gtttccctta gtcgggggat aaaaggtcag ttctgaatga gaacaggctc
1025821 cctcttttag cgatgcagca ccagctgctc tgacatctgt ctgctgatcc tggaccagca
1025881 gaaagcatct cccttcataa aagaagacac cgttactggt tgaaaccttt tcaaatcact
1025941 tgttgtgatt cttctttggg agatcaaact aattaatgag tgtaccattt agatgcagca
1026001 cgcttgagca aatgattgag tttgatccct cactaaaccc cccgaaacgc tccgctcgca
1026061 tctcttttg gaggaaacgg aaacaatggg caggtcgtgg acctcacgaa tgctctccct
1026121 gtgtcctcat ttatgaagaa ttccatctgt aaacgctgat accgtgctgc atcctgctga
1026181 ccgatatatg acacaagccc ttttaaggcc aaagctttgt tagttttccc ctctcttctc
1026241 tgtcttcccc tctgtaggtc aactcattca tctccggtcc tgctgagccg tcagtgcagg
1026301 ccgcggctca atcgatcagg gggtcctcct ggcctctgtg tgtgtgtgtg tgtgtgtgtg
1026361 tatgcgcccc cccccgcaca catgcgtgta tgagaggtcc gactggagcg cttttattcg
1026421 cgacggcctt tgccttcacc gtctccttcc tctctgggct catatctatg cttctcacct
1026481 gcatttatgc agcctccaat cctcctgcct cctaatccct cccaatccct tcagctgttc
1026541 ctcgtccttc cgccgctctt cgctctcctc ctgacccccg tacctaaatc tacctcatct
1026601 gcctctcttt aaatccctct cctctcccgc caggagccgg agagctgaca gctttatagc
1026661 tcggccgtat ctgctctgcg attgcccttc gtttcccgcg gtgacaccta attagtcagt
1026721 tgtcacgaga ggcgtttggg gtgtcgcagg aggcacaatg tggcaggcgc gtttcacaga
1026781 taacgggcgg ctgttgttgg agaggcggcg aggatgccca cgcagagcgg aaggctggag
1026841 agcaggcgcg gaggaggagg ggagcatcgg cgacaggcgg attaaggatg cggggggggg
1026901 ggcagagagg caggtgtgat gtggaggaga tgaaacagga gcaggagacg gaggagagga
1026961 gggtcggctt ctgcggcgcc gcagtcaagt tctcgggtga gactctcgca ggtcccccag
1027021 caccagctaa taaaatctgc catcatctcg gtgtcaggga gacggcctta aatagatcgc
1027081 cccccccccc caacacacac tcgccgtgtg atctaggaac aaacgggagc agcaaattca
1027141 agcgaaagat cacacggaat gctggaacta gtggcgtctc agtcaccctt tagctgacac

1027201 ttccggcgct ctgcatgtca acgtgcgtgc gtgagcgtgt tttaggcgtg cgcacgtgca
1027261 ttagcatgca tgcgagagcg aagacacttg acagcagctg cttccttctg cttcgtattg
1027321 ataaagcctg aatggcgata attgggctgg cggcggcggc taatagctgc tgcgggcaga
1027381 gatgctactg taggcgagtt agattctccg ttggaaaaag ttattgaaga cctttgactt
1027441 gaagtatcgg cggcacaaat gcactaatta cacagaagcc gtgtaacgct cacacgtgct
1027501 ggtctgacgg aggagacgaa gcccttcagg ctgtcacagc tgcacgtgaa gtcgtgctaa
1027561 tagcgtcata tggaatgagt aattagggat gctagcacgc tcgtgcctgt gtcattttac
1027621 cctcaatcaa tcagagcaga tgccgacgga ggagctgcat ctctggacag tgaagctccg
1027681 ttaaaggatg gacaggaacc acgagtcaag tttcaaaaag agcccacgtg tttcattttt
1027741 cagggatttt taaccccccc ccccccaaaa tatgcacatg agactgtcta gagaccattc
1027801 gggggagaca cttcctgtca catgaccgac catcgtcact ccgggatgtt cagtacatgt
1027861 tgcgaagaga cttactgcgt tgaggtccaa cataaagcgg cataaggctg agttttgtcc
1027921 atttaaagtg tttgtgctgc tcctgtcgcc gtgggttact gaaactcatc atcagcaata
1027981 gaaatgtcaa atttaactga agtgaatgga attatgatgg tattctctga tagcgcttca
1028041 tcttaaacat ccccccaagg ttgaaaacgc aggctacaga gtcgagcctg tgtgagatct
1028101 ggccaacatt cattttcaaa cagatcgatg ctggatccag cacctcctcc tggacccctg
1028161 ctcccgagcg gctctgctat ccagggctga atggaggatg agcggcttgt ccagctcact
1028221 aaagctccac ggagtctcaa gtggatgatc aaacacgctg acattcctcg gcaacccccc
1028281 cagaaggtct ctggtgaatc tttgaacaca tgaaaccaca ttacaaaccc catcaagtgg
1028341 tccacaggga ccgactgtct ccatgtgaga cgacggtgcg tctgcgaaag acaccgggtt
1028401 aaacaggaac cgcgcggctg ttatttgacc gatcggcgcc ggcctcggca gctcttacga
1028461 ttcgaagatt taaaccacat agggcgacct gtgcggggcg tttgacagct aaatccacta
1028521 tttcatggct gatgggaaga tgtggaagcc tttcccccga catagcagcg agcagatcat
1028581 gactccagag gcagatggct cgaagcgagc agatagcggg ctgtctgggg tctgctggag
1028641 acaaaatgac gcgtcttcgg cttggttttg acaacacatg ctgcaaaaca ggcacggaaa
1028701 caccttcctg aaaacaccag atgtacccag caatgggtct ttccaatgac gccaacgcta
1028761 atgctgagct catttagcct gacagcgaca caactcatca tttccattta acacaaaggc

1028821 aaacctgcct aattttctgc tgatcgttgc tcactctttt gtgttgctac aaggctggaa
1028881 acatggagat agttatcaga ccactgttat ttttaaagaa aatattggca gcaaatgaca
1028941 gaatgttcag cttcctgata ttctcacaac agctttagca tttatgctag ctctttgatc
1029001 catttttaga tgctaatggc catgacctgc aaaatggtcc ttaggcctct gctattgccc
1029061 catgtttggt gacagagtcc ataaagcaga agttatcttc gctgcagatg gtgcgtttgg
1029121 aggattgatg atgatagcgg ccatgaattt tcctgtttat ggctctttta aattttgatt
1029181 cgtgtgtgct attctggaac atccaggagg aaaaaggaaa gtacgctaat gactagcgcg
1029241 cacacacaca cgcacacaca cacacacaca cacacacaga cacacacaca cacagacaca
1029301 cacacactca gctgtttcct caccctctcc ttttaacttg tccacactgt aattctcagt
1029361 cctcctaccc cttttcttgc cttccttccc ttcctcttct atcgctgtgg gaggttatga
1029421 atatggatgc aggctgcagc atcggggaga ggaataggct ggggtacaca cacacacaca
1029481 cacacacaca cacacacaca cacacacaca cacacacaca cacacactca cacacacaca
1029541 cacacttttg cctcttaatg cccattttcc tgtgttctgc ctccagacag cataaatctg
1029601 tgacttcagc attttgatgt ttttgtgtct gttgcgtgtc accacagaca ttgaagccat
1029661 ttctgccatt tacctgcccc cctccacccc cccacccccc tccacctccc aacagttcca
1029721 ggccagatat gtggtgttca acactggggc tctaaatcat gtttcacagg ttgtgaatca
1029781 ataataagtc aacaatttca cgtaaaaaag aggtgttttc tggaaataga tgggagtgtg
1029841 agtgtgtgtc tggcaggagc aacagtcgcc tgtctgcctc cgtaatgttg ctcatgtgcc
1029901 gctcagatcc aggcggctcc tgattgaaaa ttaccttgaa atgtcaacct gtgcgtcggc
1029961 agctgctgcc tgtctgactg cagatcggcg gaaacattag aaccacgttt ttattccaaa
1030021 aagcagctga gatctgcgag gctgacctgc caaaagtgcc tccgcggggt gacggtggag
1030081 gcccccgatc tgcccgacgt tcattgtagt tgcatcaccg ctgggtcgca cagggatcgg
1030141 aggagtttgg acgtaagtga cggaagaata aagcgcatca aaaaatagga attcaggaaa
1030201 attgcaaata gtctcataaa tattgcgtca gcggctgaga cgactgacaa agttgtgagc
1030261 ggatagcgct gaaccgcagg atgaaacaag ctgaaataaa cccgagatta ggtatttatg
1030321 tcatcacgcc tgcgcgtctc tgcagcagca ctgatggaac ctggaacctg acgcagatcc
1030381 agaggattct gcaggaccct tcggtttagc tgaggctttg ggcagcagct gttgatccag

1030441 tcagcctgga aatcagtgtg aaatgatacc agcacataac ccccccaatg aaggtttaca
1030501 atacaatgag agagagagag acagagagac agagagagag agagagagag agagagagaa
1030561 agagagagag agagaaagag agagagagag agacagagag agagagtaat aatctgctca
1030621 ggcctttgaa ttctgctcct gattgtgttc attttaccgc ctcccggtat cccggtcttc
1030681 ttctctggcg ttccagccaa accggagcca acatcttcat cttcttctca aagccgataa
1030741 cactcgcggc cgagcagcac ggccaacttc ctgccaatca cgctggcagc ccatctaacg
1030801 cgattggctc gccgctaaga tggctgtggc aaccacgcgg cggttctaaa ctttgaggag
1030861 gacacgattt ggacatctgt tattgtcaat gggacagggg ggctgagggg gggcagaacc
1030921 gtcttccctc cacgaatctg cgtgcaaatc ggttcttcag ctctgtcgta gtttcaccct
1030981 ctcgtgacgg attcccgctg attctcccgg gcttttccgc gcccgctgac tctcatcctc
1031041 ggcttttatt agtgtgactg aggcagagga gcaaaaggag ggaggaacaa cagttcctga
1031101 cagggaaccc tggttctcga cgctcctcga cagtaaaaac aagggaggca gcgctgttgc
1031161 tgtgacggag aaatgagaat caaatctatt gttttctgca cctgactggg gccgatgagc
1031221 ctgggagcag cagcgctgac agaactgggc cgactgaagg ctggggaaat aacacttctt
1031281 gggaagtctg caaagagctc cgaaacgagc gggcggaata gaatgctgtg aaagcgccgc
1031341 gccactcgga tcccaacaga ggcgctcgtg aatttacaaa ggtggcgttt gtgctccgaa
1031401 agctcctgtc gggaacctca cggtggagat gcagatgggt tatttggggc ggggcggtcg
1031461 ctcctcaacg acggagaaat gaaattctca attaacctct tggaaaacct tgatatatac
1031521 gattatgtga tattatacat gggttgctgg catcattcac tcctgctgca ttcaaaatcg
1031581 gaatattatt tgcttttttg agggtgaaat aagcctctag aagtttatgt ttaagacggt
1031641 ttgcctttaa aactgctatt cttgtcttct ggatgttctg gtagcggaca gacacgcgat
1031701 actttcatga tgacttccat caaaaaaccc agcacggaag gacgcggcgg gatgacataa
1031761 tctctgggaa cggtgggagg tggggagatt tatcgggact ctcagcgggt cgagcagatg
1031821 acgggcagat ggagccgggc tcgccggcct gctgaccagc tggcgcgttg ctgcggggat
1031881 gccgcgctct ccctccgcag ggccgctaat cctctcctac cacttccagt cacaagccgt
1031941 gaccccgtcc tcacgcgctg gcccgacagt ctccggctcg tgacaacaca gcgattcctc
1032001 acagagaaat gtcatagctg cggcgggtgg ggaagtgggg ggtggtgaag gtgtggaaat

1032061 acaggctagc agaaggttgg tgcttcgggc tcacctggga tttgctcagt gagatgtggg
1032121 ctgacacgag tggcctcctg ccgcgtcgac accgagctcc tgcagcttca gagcaacgcg
1032181 ccggcccccg cctgggctct gcgttcctcc ccctgctctt aacaacacgc ttcaccacca
1032241 cagttcacct ccgtagttcc taaaaccacc taaaatgttg ctcttaattg tcctcttagc
1032301 agagacgctg ctcatgactg cacagtggct cgttaaagcc aaattgaggt ggtgtctggg
1032361 gagtctggag gccaaaataa tgaacttgaa tcacagcagg tttgttatgg aggaccagcg
1032421 ttcctgggct ccgtgttgga agtgttgccg tgacggtttt ggatggatgt taagcaccaa
1032481 agtagcatcc gcaacaaacg actgaacccg gcttttacct cccttctgag agggtttaat
1032541 gttgtggctg actaggaaat gtgcacaggt gacacgttaa tgggtaacgt agctgtagaa
1032601 gaggggacaa gaagactgaa gaagaaataa agcactcaga gaagcggccg cactctcagg
1032661 gttgatgaat gtctccacgt acctggaagg tgacagagcc gtatctcatg caagtgcacg
1032721 ccgggaaatt cttgcaaagt aaacgcaacc cagagatttg tggcatctat tttgggggcc
1032781 agtaaaattt gtttggctga agcgataact cagcccaaag caaggtcctg ctttaggtgc
1032841 ccgacactga agtcgctgcc cgttttctaa tgtgtgtcgg taaattagcg ataaatcctc
1032901 cttctccctt tgtcttctgc cacgctcaca tcattttctc tcctcatcca cctcactctg
1032961 gttacagtac actctcaggg tggtagcatc attagcatca ttagcatcat tagcacaaag
1033021 gggtgttaag gggtctaacc atcaaatcca attagccatc atacctaaat gcatcccgat
1033081 gctaaacgaa caaaatgagc tgttttagct aacatccaag aatggagtat tttataataa
1033141 actctccaat ttaccaacct aaagtttttt cctgcgtgca ttacagcacg cttttctttg
1033201 tctgctgtgc cagatggaga tgacgatttt tttcaaaaca gatcagagaa agtcatgaga
1033261 cttttagact gtttccatcc ccgtctctca gtaagcggga ggctgctgcc tccaaaatta
1033321 atgctaatta agagacctat gacattcgat gtatgtcatg acatggattc atctactctt
1033381 gtgtgttttt tgcactaatt tcagtaagat ttcagtaaaa agaagcgtag atttttgggt
1033441 gccgtttttg tcatccggct cacagtcaat accgctgacg agggccaatc aacaaaacga
1033501 ttagcacgtc tgatcaagtc gcggcgctac tgtaacgcta atgagacgag ggtggaaact
1033561 tgttagccac aagctcctgt cgctgaaagc aaggggggac gcgactggga gggacgcgtc
1033621 gggcgaccag tctcgtccag ccgaccgtga atgacgagcc atcagcgcgg gcccggagac

1033681 tgacagctgc acgttctaat tttagacatt aatgatgtcg atgtgcatcc acacattcaa
1033741 agacagtcag ttgaaaacac ccaaggggct ccggcggtcc ggtgccagcc actcattagt
1033801 ttctctggta acagctaaaa tgtcccaggc acggactctg aggggacaaa caaaccctgt
1033861 cagctgggaa taattcagtg cctttatgga taaaaagtct gctagcttgg ctcagaaaaa
1033921 gcctctttcc tgataaatat tgggtcactc cggtcgggtc cttgatgcag acgcgaggcc
1033981 ttggagggga aattaaggac ggtttctgat ccgccagttc aggctaatcg tggttaaatc
1034041 acgaaattca atagagacgt ttaggattga tggttgtgta caattcttta taaaaatgaa
1034101 caggaatggg gctaatgctc gccagctgcg ggcctggtac ggcccctctg aagcagcttc
1034161 tctggagcgg ctgcatctat aattcaccgg gagcaaatac cgtctcaagg gcaaaaactc
1034221 cgccgcctta ccttcaacaa cagcaaaagg cgaacttgta caactcaatt tatagagttc
1034281 gcacaggcac ggcaccgttc accttcaagg tagagagagc ctgaataaag gggtgctcaa
1034341 ccatggcagc gcgcacctcc gcccatgaag aaccccatct taattcataa cacaagcgaa
1034401 caaaaatgca cttctattaa attggaaatg gcatcgcgga aaaaacaaaa tcacactttg
1034461 ccacgagcca ggagagagat tagggaaact aagagaggaa gacggcagga caatttaatt
1034521 gaaatctctc agggaattgg acggcaaaat aggaacgtga gggagagggg gaggggacac
1034581 aagtaatcgg gggggggggt attacaattt cacagttttc agacgttttc cagccggttc
1034641 aaaggaaaag gccgcgcgga ctcctgcagg attaatgcgc cgccatttgt gattgattct
1034701 caatcattca ttaaaagtta gcatgctccg aggcacaagg ctgtgattga gaaacagttc
1034761 tcactgtggc cacctccgct cctgctttag gctcacttgg ccattctgtt tcattattta
1034821 gtttttaatt aaagcacctg cagtcctggg ccgctgtgca ttcaccgaag ttgttctgca
1034881 caacagctca ttggagcggc gagctggctc ctctgcagcc gcgttgtttc tgtagcgctc
1034941 agctgagccg cggccccctg acactgtcac ccaccaatca ggctctcaaa tcttccatca
1035001 gacacacccg cagtgggagc atttatgaag atatgcagca ttttgattct ttatttgctg
1035061 tagatgattg aacgcaggcg aaaatgttcc ttcattactc tagaatagaa gagctcatcc
1035121 tcatctcccc tgggaattga tggaaaccag tgctgaaaac accatgacag gctaaaagga
1035181 gtcaaaatgg ttcattattg tagatagata gcgccggaat actgtgttta acttttccgc
1035241 cataaaataa ttgattataa cgtagctgcc gaggaatacc agcaaacctt cacgtcaaca

1035301 tttgcagaaa tactatttat gtagttatat aaacaaagat acagaagcta atattctcgt
1035361 gattacacac tcatgctgcc acagcgagct gttatggatg aagtagctgc ttatctatgg
1035421 aaacggacaa atcggggttg gtccaagcta ctgcgagggt taatcctcgt ccagctgtca
1035481 caggcattta ctgacatcca caaactactt taatcactgg gtttattttg catttttgtc
1035541 ccgtcgtggc gacgcaattt gctgtgtcgt cacgtcgcct tattaatgaa cctccagcag
1035601 ggacctgagc aaacgacagt cggcaggaaa tgaggtgggc gtgactgctg acaggtgaga
1035661 aggtcgcgct ccgtcctcgg cgcctgctaa tgatgtcgct gattgccaga ataaagctgg
1035721 ctgcggagag tgctgacgcg gggtaaaaca caaaaaaaaa accagaagaa cacaaacaaa
1035781 ccttccacat ctgtctaatt tcatactgac attcacttct cactcccacc ctcgatgatc
1035841 agcgttacac acgtcaaatt agagcctaat tacacggaac ttcatctttc tttgtcacgc
1035901 gtgtgcgcct ctttgcacgt ggacgtgcgg ttgctggcgg tgatacgcag cctgataaca
1035961 gatttatgtt tctttggctt cacaggctta ttaacattca catgctggtc caattatctc
1036021 ctctgattaa gctgccaatc gattgaattt aataattggt gttatggaac aatgctgcgt
1036081 attaatcctc aaacagcagg agctctggat tagtcagaca agtgcgtcac aaacactcag
1036141 ggggggggtga aagggaggag cgcttgatgg gaaaactgtt caactgggag atatggagac
1036201 ggggaacatc agtaatatgt gagtgtttgt tcaaaacgtg gcaaacgtgt ctgtgattgc
1036261 aaaggtagac ttcaatttat gcacccggct gcagctctgc acctccacca gtccatcatc
1036321 ccatctctga gtcagggtca cggggagggg ggggtctgca gtgatgnnnn nnnnnnnnnn
1036381 nnnnnnnnnn nnnnnnnnnn nnnnnnnnnn nnnnnnnnnn nnnnnnnnnn nnnnnnnnnn
1036441 nnnnnnnnnn nnnnttattg tctttactat caatcatctg tgactgacga tgagaggttg
1036501 gaatttaaga atatctgcag tgttctgagg tacatttgtg gcaaatcaac cacaaatgta
1036561 atggcaatta gtaaaaattc aagttaaaat atataaatta atatgaaata agaaatatga
1036621 aattagcttc tttagctaat cggacagata attccagcgt ccacatcatt tccatttcca
1036681 caggctcccc tcagcccttt gactcgcaca gttggctgac tgctcaattc tgaggcaaat
1036741 cattcgttta aaaaacaaaa aacgccaccc aggattcctc caccatcaca aaacggaagc
1036801 cagaagttca aagaaacagg aaactcaatc cgacgccgcg atctgccgtt agaaatcaga
1036861 agaaaaggat gtgatgtcat taatcaacat gctgcttggt gcgagaaggg aagccagaaa

1036921 agcttcaagt cctcagtaac agcacgcaca aatagccctc tggccatgtt cttgattaga
1036981 aatcttgctg aagagtgtgt ttttaatcat atgtgcatac acacatttat ctagctggga
1037041 tgtctgttgc tcacaggctc ccgctctcag gtagcgcctg tctgccagcc cccccgcaga
1037101 gccctgctgg gctgcgctga aagtctaaat aagagcactc agagataagg atggccgcaa
1037161 actgaattct tgagctgcta acaggagttg gctggttgtg cgtgaagaac aacaggattt
1037221 acggagcgcc ggaactccgt aatgactcac tttgatgggt gcagaggagc agcagcccag
1037281 tggaagggag gattgcacgg aaacaccgag gcaatcaaga attcttgaat ttttgcagcc
1037341 tcgcccttcc cggggttgat ccaaactgac tcagaggata aacaccaggt ttctacgtgt
1037401 ggctgctctc cggcgagact cagatcctcc ggactgaagc gtcatcgggg gagctgtgct
1037461 gagtcagcct ctaaacagcc accaggtttc cagtggtttc atcatttcag tgaccttcat
1037521 taattcagtt tcaaaattaa gaagtttcag actaatttaa tctgatttaa ggtgaaacga
1037581 ggctcatctc tggtttataa tgacagtcaa tttaaaaaaa aaaaaaactt cacttcttca
1037641 cagaccttca acgcaccctc tttttcacaa ccttgaggga tttaagtgac tttattctcg
1037701 aaaattggtc caaactttgc actcaggtcc aggttataat ccagtttaat atttaagggg
1037761 taatatctta gtaggagaac cagccaaacg ccaacactca accatagtgt gacgataatg
1037821 aggacccgct gcagttttca ccatgaaaac aatgacgtgt agaaatgctg atctgatgca
1037881 gaaatatttt ggtctttgcc tccagaaaag gttagaagct ggttatgatt caatttagct
1037941 gtaacccatc aacagaaaat agatgtatta tgaaaggctg atcttcctca gcccacgggg
1038001 acggcttacc atccaacaaa aggaggatat tgttaaaaag gccattttgc ttcctcccct
1038061 ggtgtcgtat tcccctctca aaaccaatgt ttttgcccgc acagacatgc atccattacc
1038121 ctgatatcat ttcctcccct gcgcatccat ccatccctct cccattcttc ctcttggctc
1038181 ggacggcgag ctgtggcaag aaaagcctcg acaccctggt gatctgcagg aagagcgacc
1038241 cggtgtgatg ggaccggatg cctcgccagc acactgtggg gggggggggg tgtatagctc
1038301 ctcggatgtg aaaaacagaa agacagaccc tccattaaca gcagtcctct gtattcaaac
1038361 acatccttag agaggacggt gccagtcatg gccacctgcc cgtacacaca cacacacaca
1038421 cacacacaca cacacacaca cacacacaca cacacacacg tacgtcaaga cactctacta
1038481 aatccaaact gggaaagttc atttaactgt caggctccca tgaaaggttt ccgtagcaac

1038541 gctaaacgtc ccgcgtgacg caacccttgt catcgattgg aagagttcgg cgatcagccc
1038601 ccgcagagcc catatattca taaacatgtc atcgcgaatt gtgggaggaa gcataacccc
1038661 cccctcagtt gcgccgctgc gattacctgc agcactttcc catctattca caccgttttc
1038721 attcaccgcc aaatgtcaag ttgtttgtgt gtctgaagcc cggaatcacg tggaaatgcg
1038781 cccgctgtct cgcttccgcc tgctcacgcg cctgcaccca tgcggagtgc acgtggacgc
1038841 ggtcatcacc agacgagttc ctgtgacggt tcggcttccg tcaccaacgc ctcagatgga
1038901 ttctgtgtct ttcaggtgcg atgcgttagc cgctagcagg agcggggaga ttgatggggt
1038961 tacggtttta aaatgccgga ttcatcacca cttgacaaat ccatacccga cacaaactgc
1039021 atcagcagcg gctgcacctt tcctcgtgta tcacccgcga tggaatcttt gaggtgatgc
1039081 gtgtgtgtgt gtgtgtgtgt gtgagggcat ccatgtgtga ggagaggacc agggggtggg
1039141 atggggtggg ggggttctcc gtttgaaggc agaagggggg atttgacggt gtgggattga
1039201 acaacattta tccaggacaa agccgaggaa aataggtttg tccctgatga catttccatc
1039261 gattgaaaca tgtaccgcta aaaaatgact aatctcggga cttgtgctgg cagttttgtt
1039321 cttgggaggt caagctaccg tgtctggaca acatgttgga ctcgggctgc gtttattgtg
1039381 tgggatttcg cagcgtcgag cctgagggaa cggcaaaaat ataaacctcg gaatttcacc
1039441 tgtttgtctt tcaaaaagca gctgataaca tgtgtcagcg acacaaaagg aggcagattt
1039501 atgcatcaag aggcagttta aaatgtcacc aactaagaaa tatgtgacac ccgctcacat
1039561 tcctttgaaa agttacattt attctgctgt ttgaagctcc ccgatgaagc agggtgccgt
1039621 caaagacgcc gcatctgtgt ttggagaaac gtgtttgacg gtgccgacac gtgaatggag
1039681 agccggtgtc aaagtgccca ttctggtatc gcagacaaac agcgaaggca ccacaggtta
1039741 catctggtgc cataaaatgc cctcatgctc cgatgccgtt atatggccca gtagagcaat
1039801 caatggtcct aatggccgct cgaacatcgc gggcggcgcc catttggagc cctcatgttt
1039861 ggcagatggc acgagacctg gctcgcctgg tcacttgttg gattcccaac atgaactcgt
1039921 cgcggtccga aagaaatact gtcagaatca tgaatcaaac caattctcgt gtgccaacaa
1039981 acagattagg attcgttaga gccccaaata gagccttgac cccaccaaat ggacattttt
1040041 aagggcccct tcggtccagc agcacctcct ctctccttta agtttatatt tgggagaaat
1040101 atgccgatga tactgttctt tctcacgctc aggcccgggt ccacaccttg ttttctcatc

1040161 aggcgttccc tccgacacat gtgctggctc acgttccccc ggagacccag
gctgaacagc
1040221 aacagcctcc ccgctgaatt cagggtgaat tccaaccaga tgtggtcgga
gcagcggttg
1040281 gtttatgctg ataaaaggtg catcgacatc attagtattt ttctaataag
cgtgtcagct
1040341 gtgtgggttt gaattttctg tcttctattc actccaaatt gagtttccac
tgaaattcag
1040401 ttaaaactcg gaggcatttg agcccgtctc tgccaaaaac ccaatgtttc
tcttgtatct
1040461 gggggattct cttgtcatta gatgatgagt ttggaggagt tgctcaaata
aacacttcct
1040521 ggatgtaaaa aaaacaaaac aaaacagagg gatcagtgtt gtttttttgt
cctgttacca
1040581 ggggacttcc agtgctgctg cagggcccat ctgttgtaga tcgaagccat
atcacagaac
1040641 cactgacccg cactgcagct gccctgtcac aatattgttt ttaaaaggta
cactgcagca
1040701 gccatctgca ccttattgcc agtgggactt aaccaagata ttccaattgt
aaatagagag
1040761 aattcctgaa gcttaaagct ggactggggg ctcatcattt acttgttatt
gatcagctcg
1040821 gctccctggt gggcgcactt ggtttacaac tgtgtgggag gtaccagcgg
ccctcaaagc
1040881 gtggagccac agctgccttt caagtcaaca cattcattaa tatgatctct
gcaaatttgc
1040941 atagttccaa atgcaagtct gacacgggtg cccacgagtg ccacgaagcc
tagaatctgt
1041001 tatgctgatg tagcagacac actgacagtt taatctgatg tcgaggtcat
acctcgaaac
1041061 tctcatccgt cactttattt aatgtgcggc agcaatgcaa gagaaattct
tacatgatcg
1041121 gacagacaaa caggcgcatc aggcgccccc agtggccact gctggaacag
ttctcttgtt
1041181 tgcagggaga ggccttcatc caccagcagc aacattactc cccacttatt
tgtcatattt
1041241 atagttaatt taggacctat tttatatgca gataagcaac taggtgaact
ttctcttcca
1041301 taaaaggtag ttaagttaca cttaaacttt tctttatttt atttcgtttt
ctccgctgcg
1041361 accacgtctc ttcgtgctgg ccgctgcctg accacctcag tccatttccc
cggcgtcttt
1041421 tgtgtaattt tagaaataaa atgaccttaa aactctcctg cattcctcct
ctttcaaccc
1041481 ggtgtaatcg agcgccggct gacggctgca gacagatgca cacatgctgt
ctgagaccag
1041541 agaacacaca aagtgtgtgt gtgatgtttt ggcccgaggg catgtgtgtg
tttcctccgc
1041601 tcattgttga gtttttttaa agctctcgga gcctgtggtt gtagcgtatt
gacgtgctgg
1041661 ctgtggcgag gcccgactaa aaggctctct gggcttttag ccgtggcctc
gtttagacag
1041721 ctttgtaaag aggagcttgg cctctgtggt tatcgcgtgg cacaacagag
ggagtgaata

1041781 tagattttat ccgtttctct cagcccgtcc tcgcaataat gacagcaagt cttatcgagc
1041841 ctctctgttg aggcgcaggc agggaggggg gccgttgcga cgcccccgac agaggaggtt
1041901 gtggtggggt cttttgaaga caagagagag gtgacccaaa gggtgcggag aggcaggaac
1041961 attcatctgc agcgattctg ataaggtcag gaaaccagaa tcctgggaga ggagttggag
1042021 gaatgatggg ggggtcagaa aacctccagg gaggagagga ttggaagtga gagggggaaa
1042081 taaatgggat caggtccctg tatgggtttt acagcccact gatctgctgt aggctacgtg
1042141 tacagtgaaa aagtgcacgt gttgttctgt tgcacccaga agagtgctgc tggtgaaaag
1042201 ggggggctga gaatctgact cttctgcatg agctgctctc cagggctcat cagaaacctg
1042261 caaccttctc ccctcagctc ttttgtgcaa tttatcgtct ttatttggca aaatgataca
1042321 tccccccccn nnnnnnnnnn nnnnnnnnnn nnnnnnnnnn nnnnnnnnnn nnnnnnnnnc
1042381 cccccccccc cccccccttc cccaccatgg ggactcctcc ctcttacact tgtgatttat
1042441 aagcatgtgc tggaggaact gatgttcaaa gagagaaaag ctgcttctgg ggccaaccaa
1042501 gacaacttcc tggttgcaga gtcacggggc gagtgtgcat tcacgtgtgc ccccccgaga
1042561 gggatgggag gacccgaact gtgccgattg tttcaaacga gcatccttct caggacagat
1042621 gcacaaacag gtcgtacgga caagcaaaac aacgaatact tctagttttt agcagttcaa
1042681 actctctgat ctgagtgtgc agacgaggga atttgctttc cccaacaatc aaacgagcaa
1042741 tttcataagc agacgtgttt gtgaggattg tccagtcgag tgtttgcgtg gaaagatacc
1042801 atcaccgccg tgtttgacat gaaaaaaaac cccaacagat ttgatttttg aatccaaatg
1042861 agcgtttttt tatctcaatt gaacagaaaa ttgaagctaa tgtaattgca ttttcattaa
1042921 aactgcccca ttttgtttcc ctgctccagg ggaatctgct gtgctacaaa atgcaattct
1042981 gcttatcaat tttgctttgt gccaatataa catgaataag aatgaagtaa caatcttata
1043041 tttaatcacc aaagttttcc tttccctttc caaacagttg cattaaagaa acaaagccaa
1043101 ctcactcaac aaggaacacg tcacaaccac agaggttaga aggcagcttc cctacctgag
1043161 cctgaacaca agcaggccca gtggtgccgg ccgtgtgcac cggtgctcag ctgtggagcc
1043221 gtgtagctca ttagtgggta attaggtcat ctcgtagcgc cggacagtca gcaacgcctt
1043281 atcaccccat aattctttgc agaaaatggc aagcgatggt tccaactacc aggctgtgag
1043341 ctcaagaccg acgcagtttt gttcttcagt cccacgttta aaagctacgg tttcaccatt

1043401 gacacttgat ggacccagaa cctcgactgc tggtctccac agacatgcag caagtggggg
1043461 gtgggcagga aaccttccct gcaggctgaa ctcttccttt taaaaccatc acagttgttc
1043521 atgtttatcc taataagagt aatattctta ttctgggaag tgcctggttc tctgtccgca
1043581 gccagaaccc tcaacaataa ccactggagg actttacgga cacttcagct ccccccctcat
1043641 tgatgtgtcc tcacctctgc tccccgtctc tccttcagcg agagctgtca taatgacttg
1043701 ggccgagcac tttaggataa ttaatttatg gagcagctga caccctaaag tcagctctga
1043761 gcaattaaag cttcatctcg gtcccctcct gtcagaggag aacctttaaa cgcccttaat
1043821 tccagctgaa aagtgggttt atggggacca tccgacagga ttcttaaaca ggtcctaccg
1043881 ccacccaaca accgctccga gattcatcac gcacatggaa attaatgagg aatacaagcg
1043941 gctacagctt ggttaaatat ttaatcttgt atttattagc gcagtaaaca catccttacc
1044001 ttaagaggct cctttcaagc ttcctgaaca gcattaatta gcgctccttt cctctgatag
1044061 tgttttcttt ttaatgtttt ctccctgcca gcttagctaa ggcagccatg tggctcctct
1044121 gaagccatgc cccttcactc tacagggttt tgtaggcctg atttcagcct gcacgttgac
1044181 ctttgtcccc ggctgactag ctttgtgtgt tttatcctgt gtggaataaa agagcgaaca
1044241 gctcacacat ccatcaccgc ggctgtggca gatcacacaa tatgatagag agaagtgtgg
1044301 caacagcaag tgagccggag cagaagctta atggagccgc acgcagcctc taattaaaga
1044361 cgtctttaaa ccgccgctgc tattcccacc tggagctctt taaaacctgt tcagcacatg
1044421 tcggtagatc tgagcccggc gtccaagtac cggaaaactt ggatgaaact ggctgcgaaa
1044481 tgctgttaaa tcaccccggc gcctctctct ctgtctgcgg accaaatccg agcttccgcc
1044541 gtgccgagac aaattcactt tggcttcaga agaggggagc cggttctgcc gcatgtggcg
1044601 ggcccgcgct gggcacacat gataagattc tgtatttgca ctgcagcaaa tccaggccac
1044661 cgtcaccttc aggcagcggc gctgttaaaa cacaatcacg gtggcgggtg tcaggccgaa
1044721 cgtgagccgc atctgtcctc ttgtccttag gagggtgaca tcgcccggac gcgccccccc
1044781 tcctctgttt gcaggatcac agatgagggg ctttcgccat gtttgttctg cagagtggcg
1044841 ggggcgccga gcgagtttgt gagtttgtga taattaaagc tggatagagc cctatcaagt
1044901 aaagctgctt atgtttaata cagtcagctg tgcagacacg ggccagcggc gcacggcctg
1044961 gcgcagcggg ggaccgcacg ccgcatagcc cgcgcgcata atccccccaa tgcatcagca

1045021 cgacagagga tcggaaaaga gagggggga gggcagctnn nnnnnnnnnn
nnnnnnnnnn
1045081 nnnnnnnnnn nnnnnnnnnn nnnnnnnnnn nnnnnnnnnn nnnnnnnnnn
nnnnnnnnnn
1045141 nnnnnnnnnn nnnnnnnnnn nnnnnnnnnn nnnnnnnnnn nnnnnnnnnn
nnnnnnnnnn
1045201 nnnnnnnnnn nnnnnnnnnn nnnnnnnnnn nnnnnnnnnn nnnnnnnnnn
nnnnnnnnnn
1045261 nnnnnnnnnn nnnnnnnnnn nnnnnnnnnn nnnnnnnnnn nnnnnnnnnn
nnnnnnnnnn
1045321 nnnnnnnnnn nnnnnnnnnn nnnnnnnnnn nnnnnnnnnn nnnnnnnnnn
nnnnnnnnnn
1045381 nnnnnnnnnn nnnnnnnnnn nnnnnnnnnn nnnnnnnnnn nnnnnnnnnn
nnnnnnnnnn
1045441 nnnnnnnnnn nnnnnnnnnn nnnnnnnnnn nnnnnnnnnn nnnnnnnnnn
nnnnnnnnnn
1045501 nnnnnnnnnn nnnnnnnnnn nnnnnnnnnn nnnnnnnnnn nnnnnnnnnn
nnnnnngggg
1045561 gggggccccc cccccccccc cccccgcgcc cccccсttaa atctgctgcc
atctccagtc
1045621 aagcagtcag tccgagtgca gctcagcccg tcaacgctcc agaggcctcg
tatacaaaca
1045681 ggccgacctg gacaccgact ccaggattga ctcgaggaat tcatccagcg
cgagcgtgga
1045741 ggacgtgttg ccgctcgctc ccaacctggc aacccgctcc accatggccg
atgccccccc
1045801 cccatatgtg aagatctgtg tgtctcatga aggactgtgt caacagagac
cgtccccccct
1045861 ctgaacagtg tgtgataaac cggggaaatt tcgcaatgaa aacgtttgac
gcgagtggca
1045921 atttcagtca agagaatttg actctcgtag aagttattta gtcacatact
gcagccggcc
1045981 agcaggggga cgcagatgtt caggtgactc ttttatcgtc tagcgctcgc
attccttaaa
1046041 tacatgagaa gaaagttatc tttgtcaact tcagggtctg tgtgtgtgtg
tgtgtgtgcg
1046101 cgcgcgcgcg cgtgtgtgcg tgtgtgtgtg cgtgtgtgtt ttaacaagca
ctttagcatc
1046161 gagctgacct gattttactt tcttgacact aacaatcaaa ccaatcacag
aatttaacat
1046221 gtgaaaaggt gtaaatgatg agtttttgtg tggtttgttt gcttgatggt
aataacagcc
1046281 tgcacgtcca caaactgggg gggggcgcag acaatcagag caggagaaca
gaggcctcgt
1046341 cgcgtcggct cacgtgcgcg cgtgtggagg gagcctcaat caacaggtga
agagagcaat
1046401 tactgctccg accttcccga gccgataaag ccgctgattt gtgtcaaaaa
tggaacgctt
1046461 gacctcaaac gtgacctcag gcagcagcga cagcaacgtg ctgcacgtgt
gcgtgtggag
1046521 catgttttgg attaaatttg tcaactgata attgaattct tctgctgcgc
tttcattgat
1046581 gatgtaattg cccacggttg cccgtttaca ctgctgggag agagagagag
agagagacag

1046641 agagacacag agagagaggg agagagacac agagagagag ggagagagag
agagaggaag
1046701 agagagagag agagagggag agagacacag agagagaggg agagagacac
gagggagaga
1046761 gacacagaga gagggagaga gacacagaga gagagggaga gagacacaga
gagagaggga
1046821 gagagacaca gagggagagg gagagagaca cagagagaga gggagagaga
cacagagaga
1046881 gagggagaga gacacagaga gagagggaga gagacacaga gagagaggga
gagagacaca
1046941 gagagagagg gagagagaca cagagagaga gagagatgtc cggctcacac
tcctaaatgg
1047001 gaggcataca gttctgtaat ttcacagctg taatgtggca tcaaattagc
ctttcagatc
1047061 aatgttaatc taacctcggc ccagaaaaaa agatctgagc gatccagtct
ggaagccggc
1047121 tgccaaatcc cgcagcctcc tcagcacggg tgccatgacg acgctttaat
aagccgtaag
1047181 ctcctcttct gatttgagac aacctgaaat ataacactaa ttccctcgat
gaaagcgctg
1047241 tgatttctat aaatgtgaca gatgtaacac aagccgaggc gtgagcggag
ggatggagcc
1047301 gcggatttgc tccctttcat atttgctgaa gcggcctcgg ccagtgtctt
cggaggtgac
1047361 ctcctcccgc tccttccgtc tgatatccct tcccaccttt tctcggctcg
tttgatccct
1047421 cacctccgag gagggtatta gacttcacag agcgggcggc tgatacctca
gggatcaata
1047481 cggcccggct ggtcaattca ttagagctgg agtgtgatta attaagctgc
cccggccgtc
1047541 gggaacagac gacactgaca gtgaggcagg acggaggcga agagctcgac
gcgggaggtg
1047601 tgcactcggg tccttcacct ccgtcctcgg agctaaaaga gcctttttcc
accacaggat
1047661 ttactccaga acatgaagaa agttccgagt acaggggtcg aacctgatcc
ctggaactga
1047721 aggtcgcggc tgaggtcgag gacctctcgt tgttggaact gtctttattt
caattcttaa
1047781 agaaggaaac agaaggaggt ggttcaggac ttgaaaaagc ttattgcagc
agagttctaa
1047841 ccagtccaag gacctggtca gtaccagcac atcgtcagcc tgatttcaat
acattttggg
1047901 gacctttgag atgctgcaga atcacagatt tccatgaatc tgctgattgt
ctgcagtccg
1047961 aaacaggaag ctgctaagat gccctcgaga gggaaaaacg agaagagaag
tacaacaaaa
1048021 gccaacgcct gaatcttccc acagatgcgc ttctttgaaa ggtgaataca
aaaggtgaag
1048081 cagtcggcgc cgcggtggcg gcgcattagc ataaaagcag aagggaatcg
aattacagct
1048141 cagctgaagc cttctgaggt aaattggtca aaattacaac taaagtgggc
ggctggtgcc
1048201 ggggctgatg ctgacataat tacagcgctg cctttcacag agaggaggcc
ctgatgaatt

1048261 tgctgtgatt gccactattt agcgagcggc gtcctgattg cgcacacgcg ggatgggtgg
1048321 caggtagctg ttaatccttc cagggctttt tttagtgttt gtttgctccg gctctgactt
1048381 cccatctgtc ccaggtcccc tgtcgtgtat ttagagcctc tcccgcagcg ctgcgtctgt
1048441 tcggggacgc ggagcgacgg cagagaggca gcgttcccgc caacggaggc tcggctagcg
1048501 aggtgcgaat gtggctgcaa aggcgggcct gtcagagaga gcagcaggtc tgacccagga
1048561 aaccgtggct gcaggtctgg aaacggttcc tgtgaggtga ggacggccac gtgcgcacct
1048621 ctacagcaac gtccgtgcat cacagcctga cgttccaggc agttgatcct ggacatgtat
1048681 aatgatatca agcagagctg agatggaagg aaagagagag gaagtggggg agaaagcccc
1048741 taatccacca gtgggactcg gatgcctgtg gaatccagta ttccattcag tcaggcccct
1048801 gcggccccat tttagataat cagcatttca caaataggac tattttttgc ttctgtggaa
1048861 atggctcctg agatgatggc ctgacaatga gagcggatgg agctgggaga atgctttaaa
1048921 taataaaggg cacgctgcct catgaataga gtattatagg gctggggatg ggggggccgc
1048981 ccacagcctt tcaccatcac ttctcatgcc aacactttgc ccagagtcca gctcctccct
1049041 ctgggaggat ggggtggggg gtgggggggg cttcaaataa aagattttga aggtcagtga
1049101 ttttgtaatc cccccccccg actgtaagct gtcaacatta aacatgcttt gattgaaaat
1049161 tgagatgatt gagtcagttc gtcctcaaat gatccaaagt gtctatcgag agaaatcagc
1049221 taagtgtgcc gggcaacaga gaactttgca gccaacatga gggcaaagag cggccgggtg
1049281 ctcgatcacg tggacctgac ttcagatagc cgctaagtta gccgctaact ttccaagcta
1049341 gcatccagag catcacctca cggtcagtct ttctcccctg gttattaggg gacacattcc
1049401 cgcccccgtc ttccctggac agtctggagt cacgggggcg tttggtggga caacatggag
1049461 aaaatctgac attgtttcgt ggttcagtca gtgtgtaaaa agaccagaac cagaggatgt
1049521 ggccccccctc ctctgcacat agtttcaatt tctctatctc caatttgtca aaatgtcttt
1049581 tggatcacga gactgagact atttacacat ctgctctgca tgtgtgtgtg tgtgtgtgct
1049641 gagcagaaca tgacagcctc ctgacagcct ctgtgcacag ccagcccccg cccctctcac
1049701 acagactgta gactctcaaa gttcttcaga agtgtgtgtg tgtgtgtgtg tgtgtgtngg
1049761 gggtgggggg ctgaagagga accgcacatg aataaataga tntcaaaaat gggggtgcgt
1049821 cgggttttgg cattttgggt tganangggg gggttggggg ggtttaaatg nnnnnnnnnn

1049881 nnnnnnnnnn nnnnnnnnnn nnnnnnnnnn nnnnnnnnnn nnnnnnnnnn nnnnnnnnnn
1049941 nnnnnnnnnn nnnnnnnnnn nnnnnnnnnn nnnnnnnnnn nnnnnnnnnn nnnnnnnnnn
1050001 nnnnnnnnnn nnnnnnnnnn nnnnnnnnnn nnnnnnnnnn nnnnnnnnnn nnnnnnnnnn
1050061 nnnnnnnnnn nnnnnnnnnn nnnnnnnnnn nnnnnnnnnn nnnnnnnnnn nnnnnnnnnn
1050121 nnnnnnnnnn nnnnnnnnnn nnnnnnnnnn nnnnnnnnnn nnnnnnnnnn nnnnnnnnnn
1050181 nnnnnnnnnn nnnnnttcac acacacacac acacacgcgc gcgcgcgcgc acacacacac
1050241 acacacacac acacacacac acaaatataa attcagcatt aatgtttgcc agcgggtttc
1050301 tgtacacgct cgtctggaaa tgaacctttg gcagctgaat cggagacaaa taatgtggat
1050361 cttgactatt aaacactgaa ttttaggata aactaaccat tctatcatat ttcagtcgtt
1050421 cttgtagcat cagatctttg tgtcgtgcta aaatctgtcg aaaatctctg ctttctgttc
1050481 atattccaga aatattccag ccatttgagg attccgtttc ctccccagaa gcaacttctg
1050541 cctttattta tctgcttagc ctcattttat ccattttcaa tcaaataggt aaatcaacaa
1050601 cggtgagact catctctcgc tccagcgccc cctgcagcgt gggtggtcaa ccgtccagga
1050661 agtgtgacat cataatgcta actctaaagc caggtcggct tacttcccct ctgttactcc
1050721 atcaaagttt tcagctaatc agccgtctct ctggtttcat cctctctgct ggtgctgcta
1050781 atcagccgtt tatcaggatt tctgtgcacc atgaatgatt cacggccaat cagccgacca
1050841 gacaacacga tcagcctctt aatcactcgt tcatatgtcc gacaacatcc ctcagtcttt
1050901 actgctgcgg cagagtcgga gtggaggctc agaacgctct ccacgcgatg gatttaatgt
1050961 ttcactctgc gagttgttat tttggttact ataatcacaa agcaaaggct tttcatgacc
1051021 taaaataaat tgaaaataat ccccccttgat tggattaggt gagtctttct tgcttttgat
1051081 gtgtctgtaa aggtaaaagc agcttaaaaa acaaattaaa agttacattt ttcttccatg
1051141 aagtgaaaca cgttgccagt tcaatattta acgctctcgg tgttgttttc tgacaagtac
1051201 ggaagccctg aagggacatc ccatgttgta attttagcat cttgctctta tgatagttgc
1051261 tattgtgcca aacaccagaa aacatcatta tcataaagac gtgaaatcag caggtagatt
1051321 ctgcagcaag aacacgcgcg cacgtacgtg cacacttctc aaaagacact ggatggaggt
1051381 agcggacgag atgaggacag acgttttagg acacacagca ttaggttttt gctgagggag
1051441 atttttaatg ttgtaattac gcttgttttc catgcagaaa gcaggacggg cagatattta

1051501 tggaagcgag aaggatcttc aatatcggtg cctcacactt tattgactga cagttttatc
1051561 gatgtgcgcc agcacactga ctccagatta ggctgcagca acaagaaaaa gatcatcgtg
1051621 tataaattag tgttcaatgt agaaagaaag ggaatcgttt ggggaaaaat cccacaaccg
1051681 ttttggattt tgtgaacaga tgcatcagaa ggggaaactg ggaggatcac agtatccacg
1051741 gcctggatta tcaactctga tgctgaggta accatggcaa ccactgtgca aacacaagaa
1051801 tagtccaaac cgacgctaaa ccagccggct tcaacctgac aggagcgcat cagcgtcaac
1051861 aagttagcat cagcaagaat aaacaatgaa atattatgat taatgttatt ggcaaaccaa
1051921 cgaaaagatg acgtcaggtc agaatgttac tgttctcatg aaatatctca gcccaaacat
1051981 caaattccac caagaaaaca cgcgtgaaca tgtgcgttac aaacggccgc ctagcagtta
1052041 gtgtgatcct ggctaaaagc gtctcgctaa attaagctaa tctgctttta tcttagatta
1052101 aagccattta aagcagccaa tatggctccg tctcactgtc ctccgtatat tttgtttgtg
1052161 taattcccgg cgcattggag gtttaaattg actccgtttc agttggcttg tcgccgacat
1052221 gattaagttt ggttttgccg gccgaggtat gaaaacaggt cggcgaggca gcagcgagcc
1052281 cgtgttgtgc ccggggttat gctgttgagg tgatttaaat aacactgtct gtctctcctt
1052341 tcattaatac gctccccctc tctggcggag cctcggggca tacagcccga gcagcgactt
1052401 gaatgaaatt ctgtttttca aaatgagccg cgcgctccaa agcgatggta atctcagatg
1052461 tagaaattgg aaaagtcagc gcagcaaatg catcagcagc tttcccgcag acaaacatgc
1052521 aatattgatt ctctttcaga acaggtcata ttttatgtat ttattttaaa gacttggcga
1052581 ttgggcgccc ggcgctcgtc gggagctggt ggatgaacgt gaaggaggag cgaggcggcg
1052641 acgctggtta acatcgttta cctgctgtca catcagcagc atcttcctcc aagaaattag
1052701 ctcaacgaca gccagacacg ataaactcgg tgttttccta aagtaaacaa gcgggaaaaa
1052761 gctgcctgcg acggcgccga cgtccaggag acagaaaaac cgcacgggga agcgacgcgg
1052821 acgcttctga gatggtgaag ttctctcttt tgcttcatca gacgaccccg ggaaacgttc
1052881 cggagcctct gcaggttcat ttgcgccgcc tcttatggcc tcgtttgtta tgcgccgctc
1052941 ctgcggggct ctgcagcgcc gataccgccg cattaaatat taaggattgt tttgttttac
1053001 acctgctcaa caaacttagc cacgatggag aggccggttc cttgaggaat gatggcgccg
1053061 actgaaatca gcctcgcgcg gtcgctcctc ggttacacac actccgtccg tgacacccgg

1053121 gagcgacctt tgacctcaga cacccaaacg cgatgggttc attctgaagt aagaattttt
1053181 aaagttgttt ttttttcctg ccccaaacgg catttctctt taaaaataaa tatttgttta
1053241 ttgaaaattc agcaaatagt tcatgtcgtg cggagaggac ggagcagaat ccggagcacc
1053301 gaaaggtgat ttagggattg ttggtgggga gggtgaggct cataaatgtc cttgcacaca
1053361 caagcacaga tctattagcc tgaatttctt atttcccaga aggccacgct gataaaacac
1053421 cccccaaact ctcagttttc tgtctgaaac tcaagaatta cagtcagatt tggataaacg
1053481 atgaatcagg agggtgtgtg tgtgtgtgtg tgtccacgtg tgcagaggtg tgagtgtcgg
1053541 ggggtccggt tgctgtaaat tagacggcgc tttgattttt attaagagtt tattaagaat
1053601 ctcctgcgtt aagctgctcc gatcagagta atccccatcc aaacaacaga taatgaaggt
1053661 tctaagagta tttggtgagc tggaggaaag caggacgggg gggggggggg tgcacccgtg
1053721 attggattta gattaggaaa ggcctctttt aatgggtttg gagtaatact gggatgccga
1053781 cgactggggg ctgtttgggg gttatgactg gctttagtgg gttaaactgg ttaaagagag
1053841 agagcagatc gggcgataag aactgggtgg aaccacccgg ctgttgccag gggacgcgac
1053901 tgcccgatga tgctgtggtt gttggcgcgt tgtcatgcta cagtcgcgtg ttttcacttg
1053961 tacgcctggt tgtgttatga gaacatacag ttacagaggg aatcccggca gcacccagcc
1054021 ggcttctgta tccaaacaga gcaggcggcg gctggcgtca cccggaacgg caaaaaaaag
1054081 aaaagaaaat ggtgctttcg ccagcaaata cagaagaact ggttaaagtg tgggacgggc
1054141 tggagggcag actgttgctg aggattattg tgaacagcaa gaacaaggtt gactcatcct
1054201 tttcaaatcc ccgagcggcg ccctcgtctc cgtaatgtaa agtcagccaa gctcggtccc
1054261 taaagaccag tttacaagca gcgctcttcg tcagggtgaa gaggagcggc tcaaatgaat
1054321 cagagcccaa atatctgagt cacagcgagg aacggggggg gctctccttt caattagata
1054381 gagtgatgtg gggggagggg gggagaggag ggagggaggg agggagggag gaggaggata
1054441 ggggagggag ggaggggggag aggaaggggg aggagaaggg ggagagggag gaggagagag
1054501 ggaggaggag gagagaggga ggaggaggat aggggaggga gggaggaggt agattagcaa
1054561 aatgtgagac tgataaaaca tttatacaag ttttctggga tctgcctccc acataaacca
1054621 accaatatta ataaggaagt gtgtgtgtgt gtgtgcgtgt tcaaatccca ctctgatttc
1054681 ctatgtgtgc atgttccccc ccttcttccc cacctgggtc tcctccacct gcaccccccc

1054741 cccccataca cacacacagc aatggcggct aacaacagtt agccagctta gctcagtgca
1054801 gcatctgttt gggattaata ttgaaaggag acaaaatctt ctgacacagc agttttgaac
1054861 ttcttcccct cgggaagcca cgtgcacacg cctggcggag gagcgcccgt agcatcggag
1054921 gcgaggtagt cacatcccca caattctgaa gctgacttca ttaaagacac ttggaactgt
1054981 ttaccctccc tcattgtgtg tcttcatgct tggccctcgt gccctttagc cgaagctcac
1055041 gtggcacaga ggtgttgcac atctgcccag acggtaaaca aaaacaaaac ctcgcagcag
1055101 cgcctcctcg taccgacaca gcgagagcga aactcccaaa tccctcgttt ggctaattag
1055161 cgaactcttt atttcctcct cctgtgacaa ttacagccgt aatggcggaa accgcaggtg
1055221 tggcgtcgca ataattgatg actgacgcgc tgtcggctca cgcgccagtt tcacctgcgt
1055281 ggcgccattg cgcaacgccg acaggctgta ttttacccac catttaaaaa taaaaataaa
1055341 tcaaatctgc cgctgttgcg cacatataaa tgtctgggag ggagactcag acacactggc
1055401 aacataaaaa gacaaactct ttttcccggt atgattgtgt gtaattgtaa aagctcgagc
1055461 gacgtcgagg acgcgggggg aggaagagga acgaggaaga agcgatgatg gagcctgact
1055521 cttcctctcg cggggtggct gtaaacagcg cttttgtcgg gctgccactc accgttaaac
1055581 ggcgccactc gaggaaaatc agccctcttt agtcccaccg tgactcagca caaagggcac
1055641 gcgagtcacg cgctcactga acagctctga cgctgttgtg cgcgtgcaaa ttcagacgga
1055701 acctgctgaa tattaatgaa atcttgactt ctgacactgg agaacgtgtg tgtgtgtgtg
1055761 cgtgcgtgtg tgtcgtcagg tcagcgtgtt gagattaagt gcatgagtag atctgacggg
1055821 agagattaat gtgagaaatg gaaaaaaagc aacaaataaa tgtttggatc tcatgcgttt
1055881 ccataatgct tcactttagc gatgtgaata acactgtgtg tgtgtgtgtg tgtgtgttga
1055941 aggagcttca ttactcactt tcctctcagt gggagaccac ccagtctaca gcagctcatc
1056001 gggacctgaa cgacacactg ggagcccatc ccaaaggcaa acacacccag gccaccagca
1056061 tccacactca taaaagttaa aaaaaagtaa taaaatttcc tagttataaa gccttctgtc
1056121 tcacatcttt attattttct gaaaccgaag agaaaaaaaa cccatctatg tttgaagtgg
1056181 ctgacctgat ctgtatatgt gcatctcaga ggaagcggat cagattatgt ggtgttgaag
1056241 agtgtgtgtg tgtgtgtgtg tgtgtgtgtg tgtgtgtgat taagtgttag nnnnnnnnnn
1056301 nnnnnnnnnn nnnnnnnnnn nnnnnnnnnn nnnnnnnnnn tgggtcagca ttttcatttc

1056361 ccaggctgca gacacatata catgcataca cacaccacga gaactgtcgt gaaaaagccc
1056421 cccctcgcac acacacacac acacacacac acattccgta actgtactga ctcctatcgc
1056481 acacgtgcat gcacagatgc acaaatcctt gccagataaa caggattaag agatttttag
1056541 ccaaagtcag agacaagaaa attgagtgaa gttatatttt acagatccct ttgtgaagtg
1056601 tgtgtgtgtg cgcgcatgtg tgtgtgtgtt ttgcatgatc ataatggtct catcgtgcag
1056661 agtttgcacc cacgggggat gagggtcaca gggaggtcgt ctctctttaa acaaagtctg
1056721 gccagctccg tgtcctccac cgtcctgatg tcctgatgtc cattgtgagt gttgcgtgga
1056781 gaccttgaga gctgctctct tccagtggcg aggacgcgcc gccaagtgga gttcagcacg
1056841 caaatgaaaa cctgcgtgcc agtaaatctg aaggacgctg gtttagtcat cgttccttag
1056901 atccatttgt cagaaaggta aagcgctaca tgaaggacac gagatgggag gaaaagaaag
1056961 acaagtttcg ggtggaggcc agattcagct tctgatctcc agagcggcta aagttcctcc
1057021 ctcggctcgt ctcggcacat ttaatcaacc tgaattaacc ttgaaggaat taaacttgaa
1057081 cgtttgaccc ttaacctatt caaaaccttc ttctcattta cgtcctgcaa aagctgaatt
1057141 ttcctgacga accccgaacg cacctcagct gtatcgtgtt cgaggcggcc aagcgtgcag
1057201 gtgtgcaatg ctccctccac ccttcctcac gattcatgga aatctgacca taaattgatg
1057261 tgacccccac cctgacctcc agggaagcgg tcagttctcc ccagacaccc gtgagacgcc
1057321 tcaggtgact caccagtctt catcgctgcc tgacaggtga ggaagactca tcgctcggtc
1057381 caggctgctg cgatgtgaca gttcttttag ggcgtcaccg agcccgggaa catcctcgca
1057441 gctacctgag agaacgtctg aaaggatgac atcatctgcc agacgagaaa ctaccaggaa
1057501 aacgactccc agaacgaccg acgctccccg gaggatagaa gcgcctgtgc tggcggagag
1057561 tgatgaactt cagaagcagc cgcgcgatga ttcctggcga ataaacgtcc cctttctaac
1057621 gcgtctgtct ggttctttca ctcacaatca gcagcagcag cggaaaatcc agctgctgcc
1057681 agtggacaca ggtgcccaga gagctgcttt cattctgaga acctcaatat cacacacaca
1057741 cacacacaca cacacacaca cacacacaca gcaagacatc gagaaacagc tgcagtctga
1057801 tgaatgccct atattcattc cacaggaagc tggttcatcc gtcactactg atgcaaccct
1057861 tccgctctaa cacccccccc aacacacaca cacacacaca cacacacaca cacacaaaga
1057921 gtctctctct ctctgattag tctcccagct ggtctttgcg acaaatgctc ctttgatgca

1057981 tctttccggg ggccagcgcc accatcagca ccctccttct cctcctcttc ctcactttct
1058041 ccccagttgg cattaaagtc acttccagcg gcccccgcat gagccgttct ccccatcgtc
1058101 ccattgagcc gcggaagcat cagaagctgt gtcctccagc aggaggatga gggggggaga
1058161 gacttgagcc tgttgtgcag attcttttat ttttgttttc cctgcagagc cgctggtctg
1058221 cagcataata acacattctc ttttaagtct gttctgtcag tgttaaataa aaggagcagg
1058281 gggggccatt gttcacggtc acagttcacc agggagcctc tcaggatttg ctgccgctgc
1058341 ttcgagagca tcactaactc atcactttca cacgcagggg cgcgatggtc ctccccagca
1058401 gcaggccgac aagatggatg tgataagaaa tgtgattatc gtaccgacag cgaacgcccc
1058461 gtaatctcac aacaaatccc ttttaaaata taaattacat ccgtcttatt ttatttattc
1058521 taacgagcgc cgcagccgca ctggcaacaa ctggcaggat gtgactaaaa catgcgagac
1058581 gcaagtttaa cacatgtgga ggtggctgcg gctgtaaatc cggtttgatt gcttcaccag
1058641 atgttatttt ggaagatgga agcgctaaat gcttcatgaa gtaaaggttc atctgaggtc
1058701 acctgagcct ggaagtcatg ttcaggctgt gtgtgtgtgt gtgtgtgtgt gtgtgcgaga
1058761 cggaccagag gagtcaccgt tttgccatcc aagagggatt aatcaaggga tttcactatt
1058821 tggctttgat ctttcactga agtggcacac ctacctttga tcttcccccc aaaaaatccc
1058881 agcaagggcc acagtgatgg cggcgataaa gagcagtcca ggtagagcac tgagaggaag
1058941 cttggaaata tggctcttta ttgcacacat aacagttatt ttcctttatt tgtttttttt
1059001 ttttgggggg ggggtgataa acaagcactg gcattacatg gttttgtttt acaccctatt
1059061 cagatctctt gattctaccc ggcgaccaaa caaggtgaca caaacacaga acaaaggcta
1059121 cttcacataa aacctcacga ctccacaatc aaaggaagga aaataaaaca ggttgtagtg
1059181 caccgctaaa tggttgaaaa cctaacatac gagtataatt ttagaaaata ttaacgtata
1059241 atacttttat cacagtccaa aaaaaaaaat atatatatat atataaacca aacaaaacaa
1059301 aaattccagt ggggctaaaa acataaaagg atcctcattg ggtgttttgg gagcgtttgc
1059361 cctttaacca cttgagtgct ggatcaccaa tagtgattgg ttaacctgcc gtttaactca
1059421 ttggaggaat gttttgttaa acagaaggaa aagaacagac tgacaggaag gacgttagta
1059481 gatcagtaca ctgaatgccc cgggacagat tctgaaaggc actgagagcg atccgccaca
1059541 ctgtggtccg atcaaaacgg gaaatgaggc caccaagttt tggtcaaacg ttccccaaaa

1059601 ggcaggcgtt tagtatattt gtgcttcaca aaaagagtaa agggagacgg aagattagcc
1059661 gatgttcggg gaggggaaag gcactcctgt ttagagtttt tatttaggac ggattttagc
1059721 tcaactcgac gattatggtg caaaatagtt cctacgttaa taaataattt gatacgacta
1059781 ccacaaatcc agtctgtccc acatttgctg gtgaggacgc aggcggaacc cccccccccc
1059841 aaaaccccaa acaccctgtt tttccagatg aaagtcacga agtgttgatc ccagagagaa
1059901 atgttaagtt tgatgctgta attcctcgtt tctttgcatt gctgaaatgc tttgttgtgc
1059961 gatttcattg acatttattt atttaaaaaa aaaaaaaaaa agaggttaaa ctaatctgga
1060021 ttaatgaaac tcttccgctt gtgtgtcagt gtcgatgttg aattagtgga ggaagaaaga
1060081 aaaacgaaac aaaaagatga tcaaagcttc aaagccacat gggcgtttta cactgcgagg
1060141 aacagcgtgg ctgttctgat cccaaagtac ttgatactgg caccttaact ggtgtgtgtg
1060201 cgcgtcccga acggagccgc gctcgttatc aaccagccga acggggagaa gcttccacca
1060261 accgcacggc gacagctggt gctaactgta gcggcgccgt ccaggcaggg aagccagggg
1060321 agcggggacg gcgtgcgcgt cagcctccca gtttccgcgg caacaatgag atgtcctgct
1060381 cggcttattc acagttcaaa acaacagatt ccaggtgaga tgagcaacat ttcggcacag
1060441 attaacccag acggcgacgg aggggtcttt aatggccatg tgcagttaga cgcggcagtt
1060501 agaccagcga ccccgtgtta gcgtgcagct agcaccagca cccccagctc ttcctgtgtg
1060561 ggtggggtca gtggcacaca caagggcgac caagaagaga gggaggggag agagtgagag
1060621 agggagggag cagggctgtg aagggagcga gggagtgtga aagagtgtga cagcagggga
1060681 ggtcgtgtgc cgagcggccg cgccggtgcg aggcgagcgt ggttttgtgg cggtcggccg
1060741 gcgccacacc ggtgttttta atcgtgttag tctcgtgaac cagaccaggg taatacattc
1060801 atgaaactgg ttaaaaacac aagacaaaaa tatcagcaag ttagtaaaat tcttactaag
1060861 aatgttttat taaaataaat gccaagaggt taataagaaa aataacagca tacaccatga
1060921 aaactaaatg caagcatgta aaaatactta tgacaaggaa actaataaag tctaatgagt
1060981 taaacaagcc gggcattggc aagaaaaaca aaatataaca gggagaaagg tgacaccccc
1061041 caccatcgac cgctttcaag atgtcaaaca aaagagggtg ggggctgttg tggggcgggg
1061101 gtgcagtaga tttcatttaa aaatcaaaaa aacactcaga aaaatgatta atatacatat
1061161 aaaatgagat tagaaaaata cattataaac ctgtaacaat ggctgctgtt aattacatta

1061221 tcatacatgt agtaggcagg ccagagaaca cggtagggtt gacctttgac ctgatggatt
1061281 gatccagcct ttcacaatga cacagcaggt tgtaacattg cagaggggat tttttttttc
1061341 tcttaatact gttgaacgct caatagcagt tgattctaaa ctttaatatt gcatttcaat
1061401 tgaaagtaat aacactgaaa aagaaaaaag ggatataatc aatgacctct cggtgtaact
1061461 gcgcaatgaa agagctctga aacgtcctgc cggggggggg acccggctcc tcgcccacct
1061521 ccgttcgtcg gagccggatc gacagaaccg tcgcttgttt caagagccgg aaaccgctgg
1061581 gctgcgccgc cgtctcattt gttcgttaaa aagctcgtga ttgtttcggt agtttgacat
1061641 ccagaggcgc gagccgggtt tgcagctgtt gaaacaagcg tgatgccggg ggggatgggg
1061701 gggcgcggag gggcgacccc tccggcgctg ccccgctctc cttatctgcc tttatgttgg
1061761 cgctctgtgg gccgctcttc tcagctcatg attaaatggc ggtttggcgg actgttgcat
1061821 cgctattggc agttcaattt gttcttggaa attagtcaca gtcaccaaaa gacgagaaaa
1061881 aaaggacaat atcacatgat cgaatggaaa cccccccccct acacacacac acacacacac
1061941 ctgtgtgtag agcagagtaa agggccatcg agggagaagc taaattaaaa aataaaagaa
1062001 tctcaggaag acaaaaccat ggcacatatt agctggtcgg caacgactgc tattcttttt
1062061 gcatagagta actcgggaac gccgcgctgc ggcgggggga gggggggcta ccctgctcga
1062121 ctggcgtgag cgcgtaatgc tagccgtccg tccctctctg cgccaccacg gtgggtgctg
1062181 ggtgtcgctg gacacaactg ggacgctcgg aaacacagaa accacagctg ataaggcact
1062241 tttcgtttct gatccttcgc tctgtcaggc atccgtctct cgtggtcgtt ggcgttcctc
1062301 tccaatcacg tccaaaaagg taacagcact ttgtcccctc gcccgctcgc ttttactcgt
1062361 ttttattttg cgtttttttt ttctgatttt gtaactgcaa gtcgtagtat caaaaggtaa
1062421 aattgccaaa aatagatttc tcagtttttt ttctcttctc cagcaccctg taaaattagc
1062481 tatgttttgt tctcttctgt atttgtatat gcattacaga tatctacagt attctatatt
1062541 tatatatatt tatataagac gtatgtataa agcaatcgga acaggcaggc atggctttca
1062601 catcctattt ttttctttag agttgaacaa agggttgtgg gatcagagcc cgtctggtgg
1062661 gcctgacaca ttgtggatgc gcttctggat ggttccaaca gttcaccggt gaaatcaaac
1062721 acgcccacgg tcaggggagc gggcctctgc cggccgacgc tcctcagatt aaaacactca
1062781 acgtagagat gcggcaaaat gataagaaag cacaaaagaa cctcgactta gtgttaaagc

1062841 cgcgtggggg gtgctggggg gggggggggg aggggcnnnn nnnnnnnnnn nnnnnnnnnn
1062901 nnnnnnnnnn nnnnnnnnnn nnnnnntttt gggggggggg gggggggga ggggcagatc
1062961 cacacggctg ctaaagctaa aatggcgaaa aaagagccct ggatgagcct gatgcataac
1063021 tcagtgaaac cctttggtta aatatgcaca cttactagaa tactgttttt tttgttgttt
1063081 ttgttgggtt tttgatcaca gctcagccta ctgatagagg ttcttctaaa aacatcaggc
1063141 actgtatgat gatgtagtgt ggatgtgggg ggggggggt agccaacggg agcggccacc
1063201 tggagccact ctgatgtccc caaagcctta acacacccta ctttcatcct agaattaatt
1063261 aaaaacgcac gtgcgcgcgc acacctgtcg gcaaccttct cgtcaagctt gccctccgtc
1063321 ccgtcccgtc cctccgtcct gtcctacttc ctgcatcgca cattagattg catcgagtgc
1063381 ggccgctcgg atcacgcgaa ggtaattagt caaatgtgaa cagaaaccaa aaatatcaag
1063441 cggaggagag aagaaaaaaa aaaaaagact ccaaactaaa atcaaaataa tcacaacatg
1063501 aaaacaaaaa caggacagga gaaaccaaca catgaatcgt ataaagctcc tctgcaatcg
1063561 cttcagccat gcgtccaaat attaaaacca aaagaaagta aaaacaggca gagaagctgt
1063621 tgggaggggc acacccagaa tgtgtcggct cacaattaca accaccacat gtggaaaatg
1063681 acagaaatta aacacgttaa tgtcagcacg tctcctcttt ccttctgttt ctttttctcc
1063741 ccatgtaggc gggtggtggt ggtggtgtgt ggaggggggg ggctgctggg ggagcctctc
1063801 acttttgacg gggtgatttc tctgaaacca ggcggtttgc gtcaaataga gactgaactg
1063861 tggtttgttt agagggaggg tgtcaaattg gagggggggg gggttattgc tatagttcag
1063921 tgcaggcttg tgagcaatta cggctccacc cagcatgtga ctcatgaaga aaaagaaaga
1063981 gaaagaatgg gaatcgaact caatgaacag atgggaaaaa tggatattac acaactttgt
1064041 cgttgggttt tgttgcgttt ctgaagattc tgcgttggtt tcctactctt tttttgtgtt
1064101 agggtgacag tttgggcagg cgcccctctc tctctgtctc acttgcgtcc gagaggctag
1064161 caaaaggcaa ttaccagtta actgatcagc aggcaggctt ggggcggctc gtcattgggt
1064221 gaagagttca gaggtcagaa ggtcatgtgt tagttgccgg tggcggctag gtggttagaa
1064281 acaaaaacaa aacaaacaga aaaaaaagat agaaaggata ttagtgtcag caggaagaga
1064341 ctggaatgac atcattttaa caataaaatg aatgcaatct tatgtcgccc cctcccctcc
1064401 cgctcctaca cccctcaccc gcctaatctt gttaggataa tgacctcacg tcacgactgc

1064461 taaccctttg gacgcaaacg gcctcgcagg gaaaaaaagg gagggggggg gggagtgtcg
1064521 gacaaagaca cagacaagaa aacaaaactc caaaatctag tgttttagtg tctgtcaggt
1064581 cacaaacagt gtgattagtg gtcagaaggc tgaggaacta atgttacgtg atggcagctg
1064641 cgctagcggc aaatgtgcaa tgaattgatc aatcgctgtt ttccgattct ctctctttat
1064701 ttagcagtat atttttatat gatataaata tacacatgaa ccataaatac atttgatata
1064761 aaatgctcaa atagaaaatg acagtacctc atgtacaact gaaatatcaa aaatgtgttt
1064821 tagctttttt gcgatcggtt ctgcgggaca ctgggggggg gtgcgagcta cctcggctta
1064881 tgtctgtgcg tgtcggccgt ccgtagaaaa ataaaaatac aaaataaatt caaatctcaa
1064941 ggtgaaatgg cgccgggtgg cggcacggcc actttcctcc tgccttccac gccgctcgct
1065001 tacagtaaaa tacgcctctt ttcttttttt tttgctcatg agcggagcag ataaatgatg
1065061 tatgggaaca agcctgctcc attttagggc tctaatctca catcagtgga atgtcgtgag
1065121 gctctttcaa cgctacctgc cctcgcgtct gcctgaggtt tgacttatgc ggaagagtaa
1065181 atgtaaacaa aatgtgcgga ccacaggcga ccatttagct ggtgtgtgga tgtgtggggg
1065241 tggggggtt gggtgtagtg gcgaaggagg acggggaaac aatgacgaca acgaaaatac
1065301 agttcggaac aatatagaaa atgtcaacta aggcatcgag gcatataggc ataacattga
1065361 aattcatcag tcaatagcaa agtgtagctg agagactaaa ctgacctctg tcagcggtca
1065421 caatcctttg gtaaggatgg acccgcgtgt catgccgtct cactttagta gccattgcac
1065481 ctagattgta acaggtccac aaggttagaa accattcact ttgagcaggg gaaaacagtc
1065541 attttctttt tacagtgata acattcacat ttttaactgg tagcgtggag gctaatgctg
1065601 ggctaataca acaatacaca gaatagtaac agaatacaca tgggagtaca tgtccacacg
1065661 ccacaggtat ctctttaaat gtagaaaaac gcagtagaaa aaaatacatc tttttggagt
1065721 agaacttagc atttaaaaat gcataactta aggaatatat ttccacagtg ctcaggttta
1065781 tgcaaggctc tttgaacgga ggggcgtggg agagtaaagg atggaggctc ctgggccggc
1065841 tgtggaaaaa aaggagcgct tcctccctct gggagccaga gggggcttta gagaaatttg
1065901 tctccttatt ctgaagggtt gggggaatct aatatggctc agatggtaat tgaatagtgc
1065961 cagacaccac ctgcttctcc cagtctcttc ccctcattca ccacttctac ccgggtcgtt
1066021 ttccctccta tatctgtcac gtctgatccc ttcccgcctc agcacccgac accatccgtc

1066081 cgcagggcac aagctaactc tccgtgtgtc actttgtgtt caagtatttt tttaaaaatg
1066141 tgcatgaatt tggacaccgg cgcgttaagt atgataataa aagcgggaat tttggctccc
1066201 gactacaggg ccacctacgc tgttcagagg aggcagatgg cctggcgctc ctctactcac
1066261 agacaaacca atggtggtta gttgctagca cgggtaagtg tgtgtgtctg tgtgtgtgtg
1066321 tcccatgggg ccctgtgctg ctgaaggcac cgatcgatat ggtgagtaaa taaaatgcag
1066381 ttggctggtg taacgctacg agttacagag agaaactaag tcagctgccc ctgcctgttg
1066441 ctgccccgcc tgctgtacac gccgctccct gaaggctccc aaaacacaca gctcagctaa
1066501 cagggacgcc aggtacggcg ctacgcaact cccaataaaa gcctggattc tgaatagtgt
1066561 tgttttgttt ccattaaacg cgtgtccgct ataagcttcc gtttatatct cagttctttg
1066621 gtaacagata tgttaaacgc tgacgtgtgg aaaaaaaggg gacgtggggg ataagcgctc
1066681 aggttcaagg gggtagaagc taaaacggag acaagagaaa ataatgaaca cccccccact
1066741 gggagtcggg ggggggacta tctccaggca tcaaggcaga gagcagcaca gaggaatgat
1066801 ttcaatagct gcagaaatcc gaggggatct gctgccagac tgcatctgcc ttcaggccgg
1066861 tgcacgagag ggttaggctg cgcgtccctg tcggcaggcc tccctttaca gaggctaggc
1066921 ggcgacgccg accttacaga acagaggcca cctaaacctg agagcacggc gccagagtga
1066981 agcataaaaa aatcccaaac agtgaacggt gtcaaaacta ggaggagcct ggccctggcg
1067041 gggtcccttc tgcccacgtt tctacgcagt gtccatacga gccgcattat tggtcgtccc
1067101 gttacctctc attctccact ctgtgagttt gtggtcctca ggtggacacg acatatttac
1067161 ctttaaaaaa gtcctgactg tggatcaagg atgctgcgtc cagcatcgca gctccagggt
1067221 aaaacaagct gccctcctgt ccaggatggc taaagctggc cgctgtacct gctgcaccca
1067281 cgctccccca ggctcgctta cctagaaccc cactgtgctc gagggggtat tccagcaggg
1067341 aggtaggtgc cagggcgtac gggtactcgt acggggtggt gtagatcagc ccgccgtctg
1067401 gtgtcggggg caccaaggtc ggggtgccat tgggcagcat ggcagccatc tgagttggcc
1067461 tgatgaggtt catgatgggc gccccggctg gtgttggggg tcggagcccc gggggaggca
1067521 gaacctgacc cgtgggggcc gctataagac ggggcccctg agcggctgct gctgccgctg
1067581 caagagagaa tgcaaggttg gctgccataa gcgagagagc aaaggacagg agtagaaaga
1067641 gagcgggggg tggtggccat caacacagat gaagagtgga gggggaaaga gaggaggaag

1067701 agaagaagaa aacaaagtgg caggaggaga gggggagagg gaggggcaaa agggagaatt
1067761 agtccatgca atgatcatgg tgatgaacca caaaagccac aatattgcat aaaataaaat
1067821 aaatcaaaat agccaaacta aaggagaaat aaacaataac ccatcacaca aagagcacga
1067881 aatgctgccg tttccccgat gcattcaaag catacaaatc accatggcgg cgacacattc
1067941 agctacccga tatggtacag gtgacgacaa aacagcgccc gcaccgactg aaatggaccc
1068001 gtgggtgaga cccgagttga gggcggattt aagggctggg ctctgcaccc agcggtcggt
1068061 ccatttgagt caagtacgta caaataaaca gacaataatg gcttctggtg ttagcatgtg
1068121 ctgcgagcgg tgccacacaa gcgtccccac aggcaggtcg attaaatcaa gctggaagta
1068181 cagttaggtg gtggcccggt tcatcacggc tcacatttga agaaaagctg ggatcaatca
1068241 cgctgagctt aagtgttcac ttgtctggag caaatatgaa tcgccgtatg aagctgacgc
1068301 ttaattatgc actgatggat ggaatctggt gctgcaggag caaacagagt aaaacgggtg
1068361 gccaacatgc agattggagc gtgtggaacc aggttggaag ttcagagcgc cgacagcaag
1068421 agcgatcgcg tttggaactg gggaggaggg ggggcgttta gggggagaga tgaagatgag
1068481 gaggaggatg gaggacagga tggaagttgt cctacaaaac tctctggaga gcctggaata
1068541 gaaagtctgc cggtggtcac ggttatgatg ggatgtagag agatgtgatg atggagcaga
1068601 cctgggttga gctgagaact ggatcctatt gttgctctgg attttaaaga acaggaagga
1068661 cctcctggag gtctcggtct ggaccgttat tctgaactca acccaggtgt gtttcagact
1068721 gcacattaat ctaatcaaag ttaatctgtt aacaagatat tatattggat atattgtgcg
1068781 gcttactgtg tagctaaaga ttagtgatgt catggtgcgg atgagagcgc gcgcgcacgt
1068841 gtgtgtgtgt gtgtgtgtgt gtgtgttcca gcagctcact tacgtgcttt gatgttgttg
1068901 tctctgtacg tcccgttgag gatcgccagc tccatcagct gcatcttttt caaattgtct
1068961 tctccctccg cctgcagacg cagaaacaaa ccccccccaaa tgtgactcta ttcttcgcga
1069021 ctccactgaa tgatcggcca atttcgggcc gacgggtctg aacaatgggg cggctgtttc
1069081 cgttgacagc gtgcctgggt ggcgctacaa tcactgcgtg tcatctgttt ttcacgagcc
1069141 ccagctggac ttggctttag gggaccggag atggagataa gctctcgcca ccgtcgtggc
1069201 agaatgcctt attgttacta aagtgctgac acagacatta agtatgggga tgcatattca
1069261 taatctctat cagaatgata attactgtaa tttttctcgc tcccttgtgc gcaatcaaat

1069321 atgcaatagc cgccctcgct aatgtcagtc ctgtcaatta atgtttggat aaataaatac
1069381 acaagcagca gccactgtca ttattgccaa attaggggac ccaaattatt caaggaaagc
1069441 aattaagtga tcttcctcaa actcgtggcg cctcctcccc gcacctccac cccaaaactc
1069501 tgttgtctct ggtgctacta gcatgcagcg accagccttt acgggtcagg cctcctgctt
1069561 taagctccct ggggtgcagg ctgcaaggac aggtatgcaa agcaccgctg gcctcaaaac
1069621 ctcctcaggt gacctattga ttttgcaccg tttcctgttc tgtagaagac agaagctcct
1069681 gtatctgcac gcagcagcgc taaaaacacg cagcagagcg aggcggctta ttagagatgg
1069741 atggaggcag gtgggctcaa aatgaagcca cgttcccgtc attatctcag ccgctgtgca
1069801 gcctgtctgc taaagtgaac aacagagtgt ggctctattt aaggggcaat aaggaggaag
1069861 acgcagtggg gagggaagaa aaaggggggg ggggaaagac cgaggtccag agttaaaccc
1069921 tcccacgaga ttcctcctca gcagagagag aaactgaggt caggacagag gaaaagtgac
1069981 ggaggctggt ctctcgtgac tgcgtatgag tccgtgcgcc gtgtatgtgc gacacacgca
1070041 atatttagtg gagcacagag ctgccaatta accaagcgag gaaattcact ctgctgcttt
1070101 cccctccgct acctccagcg tgctccgtct tgctctgcct ataatgggga agtcttgaag
1070161 cacagaaatc atgccaacta ttttccaggc tgcaacaaag ccagaaaaaa ggacagtaaa
1070221 gcagccttga aaatgccaca tattttatga tgtcaccgat tgctcattat ttatttcctc
1070281 cacaaagatt tttgctcatg cccggtcgga atatcctttc tgacaaatcc atccaaaatg
1070341 tacagagaca ggagcataaa aatgtccttt ttaagcggtt gatgcttcct gctccagcct
1070401 tgaggcaaac cacatcccat ttgatgtgca gggtcggcta agataaccac gctgagctaa
1070461 ctgatcgggg catcttggtg tcataaacac gtctacagac atgaataaac cctggatcag
1070521 ggcagcttta gcggtgctac ttaaacgttt gctttttgtt gttaccgtca cttttattta
1070581 tatcagatgg agtcggcctc ctctaagaaa agagagaaag ccataaacga gctgtacaca
1070641 atttgctaat gactcacaca aatcaaaaac aacagcgtgt gtgtgtgtgt gttcaccgtg
1070701 ccttaaaaaa gtccctggtt cttctttggc agagtgccaa atgaacttgc gagacaaaat
1070761 agagccgtga aaggagcata aacaaggaga gcagccaaaa cgggattcag cagatgtaaa
1070821 tcgtggcagc acagtttacc acattagtca caacaataaa ttaaacatcc atctttccgt
1070881 gcatctgaac aggcagctgc gtaaccgcag acacttgttg aaagctacca taagcctgct

1070941 attcatttac aagtttttag tgacaagtga ctcccaaatg aatcgtgtga cggaggatca
1071001 ccagttgaaa caatttaccg ctcgtcctat aaataaaatg agacggaacg tgagtgagaa
1071061 ggctccgcat gaggaggaag taaacagatt ctcccactcc gtgagcctgg atacgagtgc
1071121 tgctggtgtg tcgtgtgtgc ttggcggtga tggatctgta tgtctggaga ggttaaacgg
1071181 gtgcgaggca ggacgattct gcaatccatc caactttgca taggtggcac ggagcctgtt
1071241 gagggaagag agacataata gagggcatcg acaggggaac agcacagatg ctgcggctga
1071301 gctgaggtgt gtgggacggg tgtatcccca gctgagtgct gcagatacag atccatcttc
1071361 cccattatag gggggggggg ttccccctgt tttcaagcta attatctctg caaaatatga
1071421 atcctttcat gacgcatctt ctctaatttc tctgtaaccg tgatctggtt taacctcacc
1071481 gaacggcccg atggtagcag caaaatggtg gcaggcagga gccgtgagat caaggaaaac
1071541 taaattacac tgctggaata gagtggaata aatcatgcgg cgcagagcgt acggagccgg
1071601 cttcccacta cagaagcaca gcttagatga atgtttgatg aagagcaccg gggagagcag
1071661 tttaatgttg ccttgctcaa ggtgttgcca caattaacag actcctctgt cagcatgcaa
1071721 cactggtaat ggaacattcg ccgaggcgct ggagtgtgtg tcgtgcactg caggtgtgtg
1071781 agggagcaga gggagagttg ggtttcagaa tgagaggaac gaggactgga cgcttcccct
1071841 tatcctctgt gcagccgccg gcacccacaa ccccccctgt ccatcagcag agaataaaga
1071901 cgcagcgtct ccaagaatct gatccaatca atgaggctta tttgtgaaat cagcagagcg
1071961 ataaatgcta ccagggagga gcaggatggg gggggtgagt ctcacgatga ggtcactaga
1072021 tgcatgctaa cgcgccgtca tgctcccccc tcatcaaggc tgtgtgtttg caagatgctg
1072081 aacacaaact gcagctaatt tggactgata cagacaagaa tgatgcgtgc ctgtgtgtag
1072141 gggggaatgg ctctctgaca catgcactcc aggctctttt cctctgcctc acccacagat
1072201 aaccacaacg gcacctggac tgggatggat ggagctgctc acatctctgg ctgcggctga
1072261 aggtgggggc tttccctcat atctggcatc tggccattgt tagcactcag agaaatgaaa
1072321 tgatgggcgc accaaaagaa cactgcgggg tggaaaagtg ctacagagcg actacaagag
1072381 aaagtgtgaa tgattgtttt ccgacgtgtg cggagacggc ggagatatgc gggaagaaga
1072441 gaggaattgc tgaagaatca gacgagaaaa gacagacagc gcgctgaaaa ccaatttgtc
1072501 tgttccccag tgggctgaca ctggctaagg tttgctttcc tttcctggcc ttactgccag

1072561 tcatcgccaa cagatgtgca cacacacaca cacactctct ctgcgtgcac
acatatctgg
1072621 ggcctttgca gggccggggt cagactctcg ccgaactact ccaattacct
cagcagtaaa
1072681 cacaggctgc cccagctgcc tcctgggatg cagggagagg ctggatgtgt
agaggaggga
1072741 aacataagcc ggggtgggga tggagggggc gggcgggtct ctatcggaat
ggaggaggtg
1072801 gtgctgcata ctacataaag ggaggggtag ataactccaa gtgcagcgct
ggaaatggtg
1072861 gcggcagcag atgtttttcc tcctcgttcc cctcctcctc tcaactccct
ttccaacatc
1072921 tggaggaaaa tgattactgt tgccacgccg ccacggcaac ttatcgacag
ttccacactc
1072981 tccgatgtgg acatcaagag gcgggaagag aaatgaaagc gttaaaagtc
cgtgctgcgc
1073041 caagcaaaca cacagtttga tcacttcctg ttatttggaa ccttctgtac
agaaacacca
1073101 caagagcaaa catggctgcc agacacacaa agccaagact actaatcaaa
gcaggagttc
1073161 aatccagaag cccagaatca tctccactgg ctaattcacc catcactgat
cagcaaaaac
1073221 cctcgctgcg tgttcaaagg ttattttctc tgccatgttt tgctgctgaa
cctctgtcac
1073281 accatcagcc atgggaatca ggcgctcctc agaactgtct cacaccttta
ctagaaccca
1073341 caaagcctct ttatatctct cccgctcccc tcctgactcc cagacagctc
tgttagcgag
1073401 acgcgccatt agccatgcta aaccaccagc tcattagcat aggagtaaac
ctgactacag
1073461 tcagattata acaaacacat cacggcggct ccgccccatt actgtcactc
acagagctta
1073521 tggaggtaca cttgtggcaa atgaagtcag gaacttctgt tgatgtttaa
aaccacctta
1073581 aaccatcaaa gcagaggttt taaccgcctg agggcaaaag tgtggtaact
gtggtcgata
1073641 atgctgcact ggattctcat aaatatgcga gcatctgcca tatttagcca
tacagcttgt
1073701 ttgagctact tttctgctgt cctatcttcg tcatcagcat catcagctgg
cctatgaatt
1073761 agctttcctc ccattatact ccgctcacaa ccaaaactgc accatttcca
cctccacttt
1073821 tatgtcccgt agaagcgcct gccagtgtat tgttccgtct gccgtccttt
aatgccatcc
1073881 aatctagggc ggaatatctt gaacggattt ggccagggat atttgggagc
cttaaatact
1073941 gccggggggg atttgcatcg ttgagagatc ctgactcaga gcagatctct
gaaaacctca
1074001 cacataacag ggatatgaag acctcgtgca gagtgtggat tcaaacacag
aaccttcaga
1074061 acgggtcaac gtggacgtgc actcaaaaag caactccatt agcatattta
gcatattagt
1074121 gctgcctcag tcagcaaaga gcaccgtcca ctcacaaaaa tgagtaattt
ttacatgacc

1074181 ccatctagtg tgattccttt aatgatatgg atcattgacg tgctcgtgga ttcaattcag
1074241 ctctgaataa atccgctacc agggtttaaa gggtcctttt tgctataaag ctagaggacg
1074301 tttctataga aaccaaattg ggttttaaaa ggagaaaagt cttgtttata gcttgaagat
1074361 tcagaggaag ggaaatgctg ccgtggcagc aaagaaactt tatgaaagag aaaatgagcc
1074421 tggaataaac aaagcaaatg agatatgaaa agacacaacg ggggaggaga agctatgggt
1074481 ccagatttgg ctggtgggat agagagagtg aaggcgagca ccgcagcctc taggagccca
1074541 acttgaccag cggcctctgg actcctaacg ctgagctcta cacaatccag tttgcctgct
1074601 gaacaaagtg acaccgtcac cggagaggcc agatgaaagc gtgcgctgtg cgttcccgta
1074661 tgtaagtgtt gaaagccggc cttgctacgc ccccggaggg agcttagaga ccgccgcggg
1074721 cggacgctgt aaaaagtgcg tgagcgtcaa aaagtttggc agataaacaa tgatcctagt
1074781 cacagtcggg acatgttggg tccacagatt aaacatccat acatacactg gatgcacaca
1074841 gctggttctc ctcgcctctc tataagggga ttaaggagac cttcaaagag aagcagagat
1074901 tggctggagg cttgcagggt ccctctcctc tgcactgggc gactgtggca gattaaaact
1074961 gcctggactg gttctcagcc tgcctctttc cttcctcatt tacctccatg ctggattagg
1075021 acactccaga ctgggcgacc aatacaagca ggtgggttag gaaggacggc ctgagtgctg
1075081 tttaaacggc tcatcagagg cctcctgcac atgcagcaac aatgtgatgt gggaaacgaa
1075141 gtaaaatata gtctgaatta tcttataaac gatgcagcca ctgaagagaa ctttaatggt
1075201 gtgatgtgtg tcactcgtag gaatggcagt aataaatgca gtggggaagc aggcgtgtgg
1075261 cagctgccgt tgtgcacaaa ggttactgcc atcccaggct atgatcggga ttattgctga
1075321 gattccaggg atgcaaagcc aacacccatg cagggcgttc cgattcttcc agtgactcac
1075381 acgtgttgtt tgtgagatat cactgaagac gtctgcctga ctatcaatgc caggaacagg
1075441 gaatgaaagg ggacagggag ggaaaaatag ggaaatggcc aggctgggca ggttgttccc
1075501 tgcaggggga ccgtttcttt aggagtaaaa ccacctgtcc actaccaaac agagccgtgg
1075561 agctggctga ggaggagtat gggcagatgg aaggaacagg caggggagaa gccctccggc
1075621 cagtaccgct cggagaggcc acagctgcac agttatcaga agtaacattc caaaagttga
1075681 gatttgaaga atgagggcaa aggacgtaac cagatacatt tgtggtcgtc accaagttgg
1075741 ttccatgaca aaacctctgg ctaaaacgta ctgtatatat gtctgcagac ggggctgaac

1075801 aaagcgagcc tgacctgacg tgactcacta ctcaaaagaa attaatggca cggggcagta
1075861 atcattattg gcattcgtgt caggaaagcc acatctgttt cctgtggtga aacagtttaa
1075921 gtggtcaata gcaattaccc acttatcact tagagatcat taatctgaac tgatttatac
1075981 agcacgcgct ttaaccattt acccgcccac agagggagtt taataggtgt ctgaagtgca
1076041 caactagaag ggcactttag gaccaaacag ctggataaat gagtaaatga aacgctctaa
1076101 agataatgac caaagctggg tcacatgatc taggtgagcc cattcaggtc agccgggtgc
1076161 cagggtggca aacggaggtg aaaagactcc aaccacacac gctgtcgcca atgtgacaag
1076221 agcagagcag cagtgaagga gtgcacccac ggtagtcctg ccatcgctga atgaggtcga
1076281 cgagtcaagg aagccaatta aaaagatgat taacattcat gtgggagatg cagcgtctat
1076341 gtgcacgtcg gccagcgcgc accactgcgt ctctcacaca cgcacacaca aacggtgccg
1076401 tccagcttat cgcgagtgcg gtgtcctggg aggtgcagat caggaggtta atatgatcca
1076461 atcctgaacc ttcattagct ggatgggggt aataacagcc tacagggagg gcagaagtta
1076521 cactcctact cctggcctgc aacaatagca atatacgtga caagagcaca ttagagtctt
1076581 tatcttccag ggtggacagt gagtttcagc catgttctac tgtagtttag gggaattgtg
1076641 ggagcacaag ctggttcatg actctcaatt aacccattta ttctttgcat gaacccaaga
1076701 tgcataattc tgcaatgtag atagaattaa gctgttttaa atcacataat agagggcatg
1076761 ttgatctgtg gaagcaccaa aggtggagac gtgcactcac agaggtaggg aatcattcca
1076821 gagtgaacag gagagagaga gagagggaca gattgagaga aaataagagg gaaatgatag
1076881 cgataggtag gcttaacgga gaataaacat cattaattgg gaaggcacac atggatggat
1076941 gcaaccagat ggtgttttgc acccatttgt cagacgttgc tgacaggggt gtgcgtgtgt
1077001 gtttgtgtgc gcaattctaa acaattgcac aaaaaactta aaggaaaaat gggatcacag
1077061 ggtctatctg tgcagcgctg tctaagtcaa gcctcttccc atcgcctcgc tcccctggct
1077121 gaacggttct gatggcgctg ccactggagc aatctgccaa gccggatgca tactggtgag
1077181 tacagaccta acgcccctgc agtttgcctt ggcatccatt aaactccatc tgtctggctt
1077241 ttacagaatc aacagctact ctgtgttctt caaatggggt ccgaagcaga aagaaaagga
1077301 ggcaacacac gacagagcaa cagaaggcta aaagagatgg cgacatgatt ttacacaaaa
1077361 gaaacacccg tcttagccct ggagggtaca gtgatggagg ggacggaaca tggctgacct

1077421 cattgccaag gtgctgcatt ctgcacagta aaaaatgtct atgcattttt
aataatacga
1077481 cgtacaggaa tgtagaaagg acagagaatc agaagattgc cacgcagctc
gtggcggcac
1077541 cagtccctgt gcactaaaga cgacctcact gactcaaaac acatgtggtc
gccgacacaa
1077601 agaaacgggc ggtagtaggt gaagagccta cgcgacctgt tccttctatt
ttccggacac
1077661 cctcgacagg aaagggtccc tcaatgtcaa ggtcttagtc tggtctcagg
tggtttggtt
1077721 ccagtggctc gttttgctcc aaactgggct gtgacagcac tagaacaatg
tcagttaatt
1077781 atatctattc aatgacagga cacagaccaa taaatatgtc tgtattcaaa
tgattactgg
1077841 cagattttct ctcctctttt cccttaaaga gccgagcggt tgctggaaaa
tgagtgaaag
1077901 cgcggcctga agggttccga tggactgagg tgtttgcaca tccccactgt
ttagatgaag
1077961 gttacctaca gtgcagtagg gctctgcctc cagctcagtt taagggcctt
agctgaagac
1078021 ccgggagatg cagagcattt ttgctaatgt ggatgtggat gcacgtgcag
ggctgtgcat
1078081 gtgtgtgcaa aggcaagatc agccgagtaa atccacactt gaaagagtag
ctcattcaag
1078141 ctctcccacc catctctgtc tccaggccgc ctctctcatc acactcgtgt
tgtgctgctc
1078201 ccagttcagg aggagttgaa gagttcacag aactgggaag cagccgctgg
ctcccactgc
1078261 cccaaagagc aaacctttaa cccggtagct ttaaaagttt ggagcagcca
agctcacacg
1078321 tccatgtatc tggtagaaaa catcgacaat cccatgaatt cagaggttca
atgtgtgaat
1078381 caatcacagc tgaagggatg tataaaggtg tcattggtat cgctacagta
taaaaaggtt
1078441 ggatgtctgg caaacaaagc ttctacaagt gatcactgaa acactgccgc
acttagtgtc
1078501 atctatcaaa tgaagaatct tttcactctg ttaattaaag atatcttgct
tgaagagaaa
1078561 aaaatgcagc aagtctttaa ttataactct aaaaatgtct cacttaaaga
ggataaaaag
1078621 tccttatagg caagatttcg ttatagtcta gaagatttca gaagttctat
ttttcgcctt
1078681 tttagaaaac tgctaactcg aaggcaaaaa ttgattagca ctactatgag
gtgtgataac
1078741 attttaagct atttatacag cattccatcc agatgttaac caccaagagc
tgccactgac
1078801 attcacggat gccattcgtc cgtcctccag catctctatt cagagcagcg
acacatccac
1078861 gacaaatctc cacgtttatt aatgtgtcag ctgaaatgtc aaatgtcccg
cagaagaggc
1078921 aaaggagagg ctcaggaggg cgataaagga gttgtttcta tgcagttaga
tggcgagaga
1078981 ggagggctca cttcaaaggc tttctcactt gaaagtgctt cccatctgta
cattagcata

1079041 tatagaacac agttctgcaa cagagaacta tcgataccac ggccaactgg ctccagctct
1079101 gcaggaagcc aggcactgtg catcgctgcc tgattacaaa actgggactt ctgtcccgtc
1079161 cacatggtcg ccgcagcgac caggaaacat gtgcacagca tgccaaaagc atgctagcag
1079221 gcatgatcgg tggtacagtg tatgtagaaa ctggggcaga aggaaggtgg ggagggaatg
1079281 tggcagtcac agtagggaaa gccttccact ccaccaggtg cacggctaag ctaatattcc
1079341 agtcggtata cggatgtaaa caggatgctg tcggagcagc agggggactc ttccatatgt
1079401 cgcgttgtta tgcctttgac tgaaggacaa atgcgggtgt actcacggca ggtacgagga
1079461 gcttcttgac ctcatccact gctctcctca tcttgatctc ggctctggat tgtgtgtctt
1079521 ccactgtgag caggacgtga aggtcctcgt ttaggtgctc ccagtttggc tttcctctgt
1079581 tctgctcttc ctggaagtgg caagagaata aaaaaacaaa aaaaacgtga caaatctgtt
1079641 agaacacgag cttacaaaga gaagccagat gacaggagtg gccctttaat cagctatttc
1079701 acttcaactt tgcaaaggac accttttaa atagttctct cacatcagag gtcagagatg
1079761 acagctagtc ctgcaccaat taatatcact gcataatgaa tggatggaga gatcagaaaa
1079821 atgatgggcc atgtactttt acaggctctt ttggatgact caaagcgcgg gtaatgcatt
1079881 tctagggaac gctttgctct acatgcttga gcgaatgcta atattctgta tccaaccttc
1079941 tcaaatgaga ctcagcacca cttctacagt gttgctataa tgcactaaac gcatttacct
1080001 ccgtctgatt ttttttaaat gtatttattt tcccaggcaa cgctgtctgg ttgccaataa
1080061 ccgagagtgg gagccactcc cctgctggaa gagcagttcc aaagctttca gggggcgcca
1080121 aattgtagca aaagcagcgc ctgaaaggca gaatctgctg tattcaaaac cgacatcact
1080181 tcacaccatt aaatagggag ccatttcagg atttgaattg ggagcctgag gttggataag
1080241 agtgtgccgc attttacgtt tttcagaggc ccccgtgaaa aggccagtta caagtgagga
1080301 cttactcttt tgtggagctg gcacaaatac tgtatggaga agccttgcaa ttgcaatcaa
1080361 gcatgcttct ccctttagaa gggctgttac accggctggc agccctgagc agacacccag
1080421 accactcatt ttttatttta tcagtaaagt gattacagct gattgcagcc ccccaccccc
1080481 gcaaacattt aaaaaaaaaa aaaaagccaa ttaattcaaa actgttttgt atgatttgtg
1080541 aatgaaactt gaagtgttct ggtggatctg aatgttctcg ctcctgttgt gagtacagga
1080601 atgttaagac tggccccccc gccacaccca aagggaaaca ccccggcttc aggccgctgc

1080661 ggagatgtcc aattacgtcg tcgtgttgca gcgctctgtg gggaggccca cctgacatgg
1080721 tttccacgca cgcgttgagc attacaaagg gaacacgcag cttaaagatt caccacccac
1080781 ctgctttctc ctccaaagag acttgttgcc atggcactgc actttacaac acacattaaa
1080841 tattcctcct cacatttccc tcattctctt tttacacatg ggggggctaa ttgctttatt
1080901 ttgctaagac cgtttactta tgggctcctg gacaacactg ggattaaaag ctgactaagt
1080961 caaagtcaaa gatgatacac acatgtcaca gagggtcttt gcggtgctta aatgctgatc
1081021 tttttgtggc agcgggaccc gccaagctcc atttctcatg tgtctgtgcg tgctgacaga
1081081 ttttcctcag cgacaattgt ggtcaaaggg atttagggag agaaataaga gaacacacac
1081141 ttgcatccgc acatacttca caccagcatg actctgtgaa caaggtcagc tcgtgccctc
1081201 tgagaggaaa gtgacagcat gacaaacgtg tggaaggatg aaggcagggt gaggaaagag
1081261 gagggaggca aggctggagg gaatactggg aaggcctaaa acggagccac ggaatgctaa
1081321 agcagtgccc acatatcagg gaggattcag ttttagtaaa tgtggagcat gaatgaacaa
1081381 cacacgcaga tcatccggaa tcctctaaga atagcaacag tggaggctgg ttgtcagtta
1081441 ccacctgttt gcctgctggc tcagcctaaa cccaataaac tttgaagcct ccatgaacga
1081501 caccacatgg aaaaggttat aaaattagac gcgtgtttct ccagaccgcc cttccagttc
1081561 tgtgctacat acaaaaggtc actgtgtttg aaatagtggt gtcattactg acgctgcaga
1081621 ggccttcagc tggagcaaat gtagtggacg tactccctaa gagatgtgtg cacccccccа
1081681 aagctgtaca tacggtgttg ggactcaagc tcgttcttag gaaatatatt acactgcttc
1081741 ctggctgaga tttagggctt ttgttccagc actggtgcat ttcaataccc agaaattaaa
1081801 gcagttcatg gcaccagtca gactcatcaa aaagccctgg tttcctttaa aataagataa
1081861 agatgtattt tttttaaact gtccatgtgg aaagattaaa tgacgagatt cagaggattc
1081921 tgcacacaaa ataatagagt aactgaagga gtagctcttc cctcacctgc tgcttacctc
1081981 ctttatttca aatcgcatcg tcttttttcc acttgccttt gtccgactcc ctctctcatt
1082041 gcacagctcc actgagccga ctccccgcca ggcagagcgg cccaggggaa gcaaccagct
1082101 gaattattga gagcataaaa atgctgccac taatggatcc tgaataaaca cactggctaa
1082161 gctgctgctg tggcttcctc tgctgccccg gggcatcagg ctggaaatat tgaggctaag
1082221 gacgccgtgt ctggaggata cggttgagat ttttgtctgt ggtggaggct cggttatgac

1082281 aagtccaagc cattgaaaaa atttcggggt gcggggactt gacctgaaaa ccactccagt
1082341 gacttgctga gagcaggaca gactgggatg aaaggattcc agccaatact ggacagttaa
1082401 acattgttga tataataata ccacttgata gtaaccccga aatctaatct tgtccttgat
1082461 aacacactct aagaggaggt ggaaattaaa atctgaatgg aggagacatg aaaaagggaa
1082521 aagggaagga aatgttggcc cggcagcaaa ctggagcttc taagattatg agttagaggc
1082581 aatctacaca tctgtgcgta ggaatgccag atctctccat ttctgcgagt gtgcagaagg
1082641 tgatccccac gcgaaggatt taacactctc attactgact tgtcggttca ctcttacaca
1082701 gcaacggtgt ctgccatatt agggcagcta agatgtcata acaactcaaa caatagcagc
1082761 ctttatttgc gcgtggagcg tgcacagatg cttgaaacag tgctgtttta aaaaatgtca
1082821 gtacaacctc acagtaactt caaaaaccat caaatcggga tagaaagggg cacatgcaca
1082881 tcggtcttta agggaaaatg tagtttctgt gtgtttagac agccatagag ctgctctgtt
1082941 agtgaggaca gcactgagat attcacttaa aacagaataa gacagtcagt cagtcagagg
1083001 tttttcatta ataatgcctg ggatgggatg gtttccaggg ccagagtata acaccacaca
1083061 tatacggtac atacatcctg ctgacgagtg actgcaggta aaaatgacat gaaacgggtc
1083121 ttttaagttc accgctgggg tcttgagggg tatacaggca cgcaatattc gccttttaag
1083181 tccccccaa tctgcacgat agatccagaa atagctgctg cccctggcct acagctgtct
1083241 gtactttccg tggcatgagg acagaactat tggatgtcag agcaggcatt tacagcgggg
1083301 gtctgctgca aggtttccag acacagtcac atataaatat atctgaatca tacagtatgt
1083361 gtggcattaa tgctgtgcaa tgtctaaaga cacgataaat ctttgtatta atggactctt
1083421 ctttttcatt ctggccggta caaaataaaa ccacacgtca catttaaaac ataaggccat
1083481 ggttttagtc ccacagtgat gagctcacct tctttttgtc tctcatggaa ctcttgcctc
1083541 gcaccatgat tttgcatcct gtttctgctt ccaactgttt ggccgtgagt ccccgtgggc
1083601 ccagaattct ccccacaaag ttatactaga aatacagaaa gagaaaaggt aaatgtacat
1083661 gtcatatatg aggttgagaa ctgtacagtt atagaataat gatattgccc tgaattgcat
1083721 ctactacata aacatgaaca gagatttaat aataacatta aatgaagtga tgtaaaatgg
1083781 tgaacaatga aaacgaacta cgggagatta aactcagtaa gcgtgatgtg gaagaggcat
1083841 aggatgatga tgatgatgat gatgatggag aggtgaagta aggcgtcgca cttcatacac

1083901 tttcattatc aaggcccctt ttttcttcac tggtgtgtgg cattggagct ttgttccaaa
1083961 aagaaatgta tttaacgagc taatcccctt caactgaaac aggcttccaa caggataaag
1084021 ggaaaaatat gctggaataa tccaggacaa ttttaggccc aacaaaaaca gatgggaagg
1084081 aacagagtga gggaaaaaca aactcaaccg ttagagtgcc atgagtggga gcagggcatg
1084141 atgggtaaga gggcctgtgt acggagttga aacatttaaa tactccctgg cataatggga
1084201 ggttttccat ggcaatgacg agcacaagtg ttcccatctt gagcagatac aacgagccgt
1084261 gatgccccta cattaaattc ttaagcatag tcacatctgt ttacacactt tggcgcacgg
1084321 ctgcatctgt gacttccaaa aggccatcag aggagtcacg tttaattaca aaggctccac
1084381 aaatggaatg tgatgtcgca gtatttccct cagaccgtgc aaacaaaaac tgacgaggac
1084441 agtttaaacc tgcgggctgt cccccccct cccttcttct gtcctgatgt tgtgactgcc
1084501 tgcaactatc tgctctccct gttccagtct ggcaggagaa ggacgcccga gagggtcccc
1084561 acggatggtg atagcatcaa cagctgcaca aagatccagc atgctttgct gctcttcgtg
1084621 taacgtggaa accagtgggc aaatgtgagc ccggtgacct ctggaggcca aggtcagcca
1084681 ccaacaatgc gcggtggcct cccaccttgc actaacccac acctttacac acacaagcag
1084741 ccatgtctgt ctgcatcaca gcacccgctg cagacgagac ggagccccca gccctggaca
1084801 tagtggcggc gctctgacat atagaaacag acacagcctc taaagcaata gagaggctac
1084861 ggtgacagtt tcctgggtgt tattagacag ctaaccctgc tggcagctta ggcgtcatat
1084921 tatatctagt ttatacaagt ggggtttttt taatatatct tggtttctgt gcttacaagt
1084981 caccagtacg cttcagcttt taccttcagc tcatattaca cttacagcca atgaattaca
1085041 gaaccacaag tcaataatac aggaattgtg catttaaaat acaaggatag atgaagggga
1085101 tttaatattt aactgtgctt tctctttttt gcttctacaa acatcctcct aacaggaaac
1085161 tgtacgtgtc tgtgggtatt aatatgccaa tgatcacatg acagcgaata ggaccaaaga
1085221 agcgaaaact gatgagtagt agtgcgagaa aaggcttgtg acagaaataa agacagagga
1085281 agctttacac gctcgcaaaa attaaagagg tgacacggtg ggtgctgcca ttctaaattt
1085341 aaaagggtta tttttgttct gtgctttaaa tgtcacacgg cgtaacaagg cctccacggt
1085401 gtaaatcacc tttacaattg aatgaaacct cataattaga agtataaccc tacagtcaat
1085461 aaaacgcctg ccaaaaacat ctcattaatt caatacagct tgatgttaat ggtgacggat

1085521 cgctctcccc ctcgctgctc cctggatcgc tgctctgctt cagcatttct gcagatgttg
1085581 atgtgctgtc ggggaggacg gctcccattt tcagccgcag atggagggca aagtggacga
1085641 gttatcaaaa gtgcgcaggg aaattcaacc tgtatggaac tgcactcgta atggcgggat
1085701 aatcagtaca gggcgccatc aatgctacat agactgggga catgtcaccg cctgttgaca
1085761 atggagccgc ctctggatcc acaaaaagat accgtaattt tcagactata agccgctact
1085821 tttttcctac actattctac ggtgtggctt atttatggtt ttttccgtgg cgctctatca
1085881 caactattgt gaatcagtca tttttactaa ccctcatcag catggaaaat actagaagaa
1085941 aaacatatga tgcgactttt aagttaaaag cgatcactct ggcggttgaa gaaggaactc
1086001 gagctgcgta atcttggtgt acattatggt tatcaatggc gagaaggtgg agacgcccgc
1086061 ctgaagaact gatcgaaagc aaaaagacga caaaagcttt tagacgtaaa catcgcaggt
1086121 ggcccgagct tgagaacgtt ctggaagact gggtcaacac acagagagca ggcggccgcg
1086181 gtgtttccgc tgtacaaatc agactgaagg caaacgcaat cgtcaccgtg atgaaaatag
1086241 aacattttag aggtgggcca tcgggagttt tagattgttc attgtttgag taaaaaaagc
1086301 ataaacaatg taatttgtgt gtagctgata caaacgtata tttcaaggta gctgcattac
1086361 agacaccgtt agaaaaaaag catttaccgg tacaatatat ttgtttatgt tgctaagcgg
1086421 attaaattaa aagaaaagtt aaaaaatcct gacgtgataa aatttggatt taaggtctct
1086481 gtataaacgt acaagtgaaa agagtggctg ttgtttctta cctgtctgtc actcgtcagt
1086541 gactcgtctc ttacggtaat aaaaacatac tgaatgtttt caatcaaatt tttcatatta
1086601 ctacgtggga tacctgcggc ttaaaaacca gtgcggcttg tataagtaca aaattgattt
1086661 tctttctaaa attagattat gcggcttata atcaggtgcg ctctgaagtc cagaaattac
1086721 ggtagttttc ttgcttttgt gccattcgat caacattcgc tctgcgggct ggaggcgagt
1086781 aaaagccatc agtgtttgat ttgtctggtt tgaaacaaaa aaataaataa aataaaaata
1086841 aaaatcaatg attaaatcaa tgattcaaat ttgttcttgc ttttgattcc tgcgcaaatg
1086901 atcgcaatgg agctggaatt cacaatagta acaaagataa tacctgaact ccgccagctt
1086961 ccaacagtct gggtttctct cttttctttg ctcattgaaa ggctgagagg ataaaagaca
1087021 ctgaggccct ttgaaagagc aactatttca cccatttttc agttcatctg agttttccca
1087081 agttttccca aacaaacaca gatatggata ggtgcaagcg agtgcgcatt cagacccaga

1087141 ccccttcagg acgggggctt acgctgacat taaagggcct ttgtttaacc acagttatcg
1087201 ctactccaca ggctcgccat gctgcctgtg catctgtgcc tgcgtctcta tgcgagtgtg
1087261 tgtttgtgta tttaatgcgt gatgtgaact agcgcagacg ctcagtcgtg accgtcacat
1087321 gggtttgttt atgtgtgaac aacagcgctt tgaccgacaa agctcgggga aacagaacaa
1087381 ttgtgccttt cacgccgttt gtttgtggcg gtgggggaga caggaaatga gatcatgctg
1087441 agggccctgg ctctgcgtgc acggagctta tcacaaagcg ggaccagacc gggggctgaa
1087501 tatgaatcgc ttattagctg gccccaccga gcttatggaa atgaggaaca gttcattcag
1087561 tgtaagctgg aatgtgtggc gacgccaggc aatgtcgttt tatccacgct gttcatctcc
1087621 atggttttaa cctaaacctg ccccaaatgc tacagaacat atgcccttta gagctccagc
1087681 tgacacactc tgcaccacta cagagcttca actgtttgct ccaaaaaccc gaatccctca
1087741 gaggatctca tcattcctct aacacaacaa aaatcaatgt tcctgtgatg atacacactc
1087801 attcatgttt agggagagaa tctccacccc tccacttcca ttcacctgtt tcgcggctcg
1087861 atgctcaaaa acaatgtgct attaatgcac agcggcgttt taacatattt cacatgataa
1087921 aaggtcaaga gggaagaaaa acacgagatg gatatcacac agatgcctca cctgcagcgg
1087981 caaggaaggg aaattaccct cagatttctc tggtgacatt ttcgcctcaa agccctgcag
1088041 acttcaaagc tgcctattct acacagcagg cgtttagcgc ctaatccaac actctgttga
1088101 taattctgtt tgggtttact ttagggttgc aggcagggta gaattgcagg agcggagaga
1088161 cagacggaag gcataacgat cgatggcggc tggaaaaaaa gtctgaatca gagtttgtga
1088221 acttttcgat aaacacaatt taaataagat gtgtccttta gcggtcagag ttttgctcat
1088281 cacaatctgg agtaaactgt gagctgtaaa tacacatcca tcaaccctca gttcctctct
1088341 aaacaccttc atgtaaacag tgtgtgtctt tcaacaaatg ggagcctaat agagatttat
1088401 ggtcttttta gaatgcacaa ataattgtag atgcagagag aagtatactg agatttaagt
1088461 tatcattaga atttataatg tttgaacctg gatgttcaac gggaagcctc catccagacc
1088521 tttattttga aaggaagcat tggacaggaa gcctgagatg gagggcagca accatgagat
1088581 ctctaaaaac acaccaccag agcaaaacca agccaagcaa aagcggagac gtttctgctg
1088641 ttttgcattt ctgctcgtcc attggaaatt aaggataagc ttccgtcaca gaggttagtg
1088701 accacacttt tagctgtagt taatggctct tttattttcc tgctgggtca ctttaaatgt

1088761 catgaatata ccaacagtag ggggcagcat tgatacaaac gacaatagtc ctcccattta
1088821 aactttttaa tgtcatgaat cacgtgaagt gaatgagagg atgttgtttt cctacagcct
1088881 gaatggagac taatggaatc ttggaacatc tcagcactat gccagtaaaa agtaatccca
1088941 gtgccatggt tccatttctg ttcggcaaaa aatgatgaag ccaaaacgta cacgtggctc
1089001 aggagttatc ccctccttaa aagtcaaatg tcaccgaggg ttttgtcttg aatgaaaaag
1089061 gcaaaacttc tggcaaatac aaaaacagag ggaataaagt ggggacagag ccaaacttaa
1089121 aagacatgtc acatcttatc agatgcctga aggtcagcgg aaatgacatg ctgagctgtc
1089181 agtgagctgc ctatcgatgt gtgagtgcgt gtgtgtgtgt gtgtcgtgtg cgtttagaag
1089241 aaaagcagag ctgcagatag cgtttaattg aaggttgaag ttatcagata gccagtgact
1089301 cggctgccag tcaaagctca cagagctttg cgacactgac aaaaccaggg aacggctgac
1089361 tttgggaaaa gagtgaggac agacggagag acagactgac ccaggccagc gttttataga
1089421 gaggcgagag gtgcacatag agaccagggg agggtcagag gaagtagaac gcgatggcga
1089481 gtaatttctg tagaatcgac atgcagcaac gtgtccagac tggacaagtt aaggttgcag
1089541 attaaaagtg gggaacggaa gacaatagga tgaagacaaa agttcggaaa acaaacacaa
1089601 atggcggatg gaaggcgaga taaagccaga ctatcagacc ctgctctcct gggaggcctc
1089661 aaactgcatc acagacgcat ttcaatgtgg gaaatattct ccagcccagt aatcaccatc
1089721 attatatttt cctcttcttt caccaatggg cagagagaga ttggaagtga catttccccc
1089781 ctaatcccca tcatttgttt cctttccctt cccttcaaaa gatctagcat ttattccaca
1089841 ctaaagatca tcctgcagaa tacaggcaac acacacacac acacacacac acacaccaat
1089901 ctggatccac agtagttcca cggtaacctc ggatcagtaa cactttcaaa taattacatc
1089961 acggcatcca aaaaggctga acattaaaca gtggctcagc gcattaaggt aaaagagtta
1090021 catgtggcat ttgcccctaa agctacttta aaacaagtac accgagtcct ggttggttgc
1090081 tttagcctga gctcagagga tgggtctggt ctaaaggtgg gacttaagct gatcccaacg
1090141 aatcccccag tgccctttat caatcctgtc agggagaaaa tgcaatccac gcctgactag
1090201 accttgcccc gctccacggg cctcttatcc cgcgtcgccc tccccccatt catttcctca
1090261 ctccaaacag ctgggattat gaagagaaat gttgctcgac aagacgtgac acatcctttc
1090321 acgtggttgc ccgcagatta ccatgagaaa ggtgaccgac tgtgacagtg gtcacaaccc

1090381 tacaaataca cacaggaccg ggctgttaat gtagtggacg cagacatgga tgcaggcgta
1090441 acccgtgagc aatcacccat gctgctctaa cacccatcta tcatctggaa tgataaattg
1090501 ccctacatct aacccccaat tgtccttatg caaaggaaag gagtgaggat ggacacacaa
1090561 cgcaccctga atgcgaacat cactcgaatg attgtgccat tacaaaatct gtgacagctg
1090621 atggagtaat ggtgtgtttg ggaggaacag aggagtccag agataacaat aattaggcag
1090681 aatgctctgc tgttatttgc acttacgtca gggtactctt tgacaggcac aaacagtttt
1090741 tcctgcaggt ggacgatggc tcctacaggc tcgggcagct ccaggggggg tttgtccaca
1090801 aggccattca ctgtgtcgct gtacatgtcc ttacggactc tgttgatttc tgaaaaacac
1090861 aaatcggacg acgtcagtat ctgaatacac catttctctg gtttcgttcc agatataaca
1090921 agggtcttat attgggagca cattaccgta actgcaattt aatttaattg tggcgtagaa
1090981 aaaaaacttg tgcattatga agaggtcaaa gatggtctct ttttttaccc agcactccct
1091041 gcagcagtcg gctaatgcag cagcccatca agcagctact agccaggaca gaagcgctgc
1091101 tgagaatcac tcaggctcag atacatgcct tcagatctga ttctgctgtc agataaaact
1091161 aatggacctc agtctcaccc ccacacatac acacacacac atcacagtca cacactctac
1091221 ccacaaacaa agaacagcac gtttctccca aatgtgcaca tggaaatgct gcactgcttc
1091281 cattaggggg taaaagtgtt tgtgcgtcct ccaaagttct tacggctctg ataattgtgt
1091341 gtcaagcctc ccctgaacaa aatattaatc tacaattcta cgaaaggaag agcagggaga
1091401 aacgggttgt gtttaactac ccagatctga gtgtaacaaa gtgtgccgct tggcaggacc
1091461 accaggaatt atgtgctgtg tgcagccagt gggcgttatt tccacgtctc atgagttgag
1091521 tgagcagttg gacattctat attaaacccc agcgagggaa cctgatgctt aactcagatg
1091581 ccccgagcac agcacgtctg aaatctcagc ccgaaccttc aactgaggct aaattggaac
1091641 acgggcatcc ttctgtaaat agatctggtg agttgattta cttttccagg cagaccgacc
1091701 gacgtgtgat ggatccactc ataggggcta acgaggatcc acgtcgacat ctggcctgca
1091761 gctgcactca cagaacccgc ggaactcatc cgaaatatac aaacaagcca cggaaccttg
1091821 cccggagcca ttctatccgg caccagccgg gtccaaacaa cggcaagcag ctctgaaggt
1091881 ggattcatcc aaagttgtgt aaatgtctag gtcgacaaat tgagtcaatc ctgaacagca
1091941 gcagcagctt taggtcgcgt gaaacagaat aaataaagca gagaggtcgg gaaacaacaa

1092001 gatactgagc aaagatgggt ggcggatcac aaatgcctgc acagttaagc gaaacatctc
1092061 aaatctgatc aagatagggc aagggctaag catacggtac atctaaagca ctacagcacg
1092121 tgtatacctg gcattttctg gttttaatct ggttggaaat gtacaaaggg gactgttcat
1092181 cacaggccac ttcactgctc agcacgtgta ggaggtccac cggtggcaaa aataaggtat
1092241 tgttgtttta aatggaaaga aaatcaggac tcggaataaa gcatgcaaca gaagcaacgt
1092301 aatctgtgcc atttacccca aagaacgggg tccagccttc aaataacaag agaaaataat
1092361 tataagtgtg cgcacgtgac gagtgcacag agccaccgcg ggcagccccc cgcctccttc
1092421 caatttcctc ctgctgaaat gccacattaa cataaagggg ctgtgcagag ccatcactgg
1092481 agacacgggg catttgacag cctctaattt cagcgctgca aatcttttct gaagcgctgc
1092541 gctcacctaa aaattagccc ctgccttgcg tggccaacaa aatcatttgt ctagctgtcc
1092601 accctggcag gaagtgctga ttttgttaga aagacgaaag agactgaagg ttaccgtctc
1092661 gcaacaccag gcaatcgcac aaggatgtgc tttcaaagag aacgtttgat gcgctgtggc
1092721 attagattag agaaagatct gaagtatgta catgtttaac agctgaggga ggaggcagag
1092781 agaggtcccg tttctcccct tcagccatgg ttgggcccag cgtaagccat gcacggcctg
1092841 actcagcaaa accacacaca cagccaacac tttctatcgc agctcgcgtt tatttgtgag
1092901 ggggaagtgg aaggtgtccg gaggaaaagg gaaggcttta aaccagaacg gctgacttct
1092961 ctcccacctc tcagctctcc cactgcctgc cctttcttct aaaatctgca attaaaacag
1093021 tttaaaaaaa agagtcaaac tctggggttc catctacaag agatgctttt cacttcattt
1093081 cccttctgct cgtctctgta atcgtgagat atggacacca aaaggaagga tagaagcttt
1093141 gaattagctg aacagcagta ggtgggggtg acttccagag tcccagtgat accaaaccaa
1093201 agagctgtgg actacagaga cagtctgatg ttcttgaaag cagcagggag atgatggctg
1093261 ctccttcagc accctgaccc tccctgacat gcctaaacag taataagctg aggacggcat
1093321 tagcccatga tcacggtgga ggaactgaga gcagcatcta aactgaacgg agacacgcag
1093381 ctcgaggcag cgtgaactct ggaccatagg ctgatgtgct ttcatttatt tacccagtct
1093441 gaggctaaaa tcctgacctg acgcaagcac gagagggtga cgtggtgaga aaggccacct
1093501 aaaatggcct cctgactcac aatttttaag aacaacctcc caggaaggaa aatccagaac
1093561 tcagaacaac aatcagctcg gtggcaaaaa tgagacagaa gaccagtcag ccagaaccgc

1093621 tgctcctata gcaaatggga acaagtccat taagagccca aaaatagacc tgctccataa
1093681 atgctttgtc ctccctctgc agctctgagc attgtgttct gaccacagag cacatcccgc
1093741 tatttatgtt ccacgtacgt gcgcaacaca gaccaagaga aagaaatgta actggtgagt
1093801 aatggctgat cagctttggc tgagcaaagc agctttagac gccttgtgat gcctggagtt
1093861 agcctgataa tatctgccac ctgaaacaga ttgagtctaa caccgattaa tcttctagcc
1093921 aaaacgatca gctctgcaga gacacttaac gagttctcca tctttaaagg gatgtgcagt
1093981 ttaaaggaag gtggggggt tcaaagcccg acaccaccgt ccttctgtgg tagcatatag
1094041 atgtatgctt agcatagttt aagaggggaa aatggcagct gggaagcaga gataaatata
1094101 gaaatctggg attcgtgtcc aaaaaactac agtaatcaca gatactccta aacgcaccga
1094161 tgccgaagcg aggaacaagc attttcactt tgaagctgcg cacgttagca caatcacaaa
1094221 gcccggctac ccgtcgcaag tgaaaggatt tggctgttta atgcattcct gccctttatt
1094281 cacggtgcag acgactttat ccgaagagac gctccacttc aacagtttaa gggtttggcg
1094341 ggccgagccc atgaaagacc gaggcttctt tgaaagtatg gagggggagt gtacgtgcac
1094401 cggtgcactg acggagctgt ggaaacatat gcactgctgg gtcttatcac cagctaacag
1094461 ctgtaaccgc caccgctgtc gctgccacac ccctcgggag gggggcatt cccatggtgc
1094521 catctttaaa ctaccatctg atgctaagtc cccgtgttca tctatcaatg tcagggtttt
1094581 catcttaggt aattcaataa aataattcca gtcaatagga gggatgcggc agctcacaaa
1094641 tttacacata aatacccga agattacctg cggtgtgttg aggacagagc agaatgtagt
1094701 gcccccatta tccgatcggg tcaaaacact aattcataaa aaataccggc agcttttctt
1094761 cgtagcagcc gtctaaaacc gcatttctgt tctaaaaaca cacgcttgta caatatttag
1094821 ctggtgtcca tgaaatatat ataacctgac acaaatgcaa gtacatgtgc atgtataaac
1094881 atgcccaaaa caaccacgag ctaatgagct cgtgtgtttt taggctacgg tctcccgtgg
1094941 gagaggaaac ccacagaaat atcgctgggc atggatggag gaggaagttt tgcctcgcag
1095001 caaaattctg ttgaacaaat acagatggaa agtagcgagt ccccgtttta ctccgattaa
1095061 ccagccgctg acacctgtgt attcccccga caaccacacg gtagaaatga ggcaaatcat
1095121 tttgtttcct cctccgacgc agccgaactt aatctctatg tctcacagca gtggaacctg
1095181 gcaccagaca cagggacgcc ttcaactgat acggaaagat tggatgaagg tgcgatgcta

1095241 aaaaaaaagg cagtaattaa tgtaatgtaa cattggactg acattaaact aatatttctg
1095301 ccaacgtgtg aagatgcacg gtttatctcc tcctctactc ccgaacccgg gagaatattt
1095361 acgccaaatg cagaatgtgt aggtgtgacg gcggccaaga acagcaatca gcaaagtcat
1095421 taatcataac cggtggcaaa tgtgaggctt tctggggttg tcagtgcgag cgcacgcagg
1095481 ccggggaagt gatgccaggc tgaatgtggc tttctggggc tgctggcagc ggcgacgacg
1095541 gtggactggg ggaacatgtc gttgcatgtt ggtttggtga ttaaacaaaa caggtagaca
1095601 cagcagacag ggagagaggg gccaaggcgg gaagtggggt aggtagccag cggaatgcca
1095661 agaaagaaaa caacaacaac aaacagagca atttgcttat tcagattcag attcagaggg
1095721 gaggactgga tctgaaatca tctatgaatg ttgcacaggt gcatttattc agaaaaataa
1095781 agggaggagt cgtctgtgtt taaaaggagc tggtattctg gatcatctag aaagatctgc
1095841 agcaaaagct cctccactcc tggctctccc acacagcatc cagcgggcta ttaaaactta
1095901 atagctaaaa actttacact aaaattagcc cgggcgatca tcgcgtttga tgacgtggaa
1095961 ctggcttatg ggactgcgag ctgcgccgca tgctggcagc gcgatgagcc gtggggccgc
1096021 cagctttgtg ctctcgatgg ggtccatcgt agcgagaggt ggggctggtt caatactgag
1096081 ccgtaatggc ttgcttttaa tggctgccgc aagggcccgg cattcattac tctcagtaca
1096141 gtgggagatc ctgcaggatc ccggggtgag aagtgtgata acatacacac taatatatat
1096201 ttgaacttga ggaaatcact ccagagagag aaacggagga acagacagag cagagcagcg
1096261 gggagtgtta gcagggcact taatggaacg tttaaggcct ctcctctgga cagatttgct
1096321 aatcgctgat tgcacaaata ttctaatgca gctctgcata caacagatcc actttcttca
1096381 gcccactgaa cataaagtgc gagcaaacag actgttgctt ctgcatctca tgggagcact
1096441 tttcgcaaac atcgctcgac aatgagatca gacagtgtaa caaaagagag gccttgtatg
1096501 gtgccgctca acaatcccgt tctcaccgta ctttaacctg ttggactcaa ctttcagcat
1096561 tttttgtggc tgtctgcaca ccaaacacgc cgacggcctt ataaaaacat ggccgtttta
1096621 tcttgcaagg cctccggcgt gcacgcacga acctatatcc ttcctctctc ctcccccccc
1096681 cacacacaca cacacacaca cacactgaca acaatacaga ggatgcaatg taaaatgaag
1096741 cacaggatac atgctgagag ctccgcaaga tgtgagaata ggagataagg agcgggaaga
1096801 ataacaggga tgaagagtca ggatacatgc tgaccaggtc cactcaaatg cagtgcttgc

1096861 ccccccacaa aagggggagc aagttcccat ggcctgtaaa aataaaaacc ctttttatgc
1096921 agcaaaaact caaatggtct gcaagggtgg acagctccag ccagtcgttg caacaggaaa
1096981 tgctgtgaca ggacaaatct gggtttaaag gaggcctaaa aagcagagca ttcatctatt
1097041 aaacccgcgg gggggggggg ggtcgttcct catgggtgta cctcgggttc cacacagttg
1097101 ccctgcccat cctgcaacaa tgccaggaag agatccagct gtcacaaggg gaagctatag
1097161 aggaaggaaa ggctgtcgct gccggcgtga ctgtgcgaga taacatgcac tgggcttcct
1097221 gtccaccagt atagctaaag agtcactcct cacaaccagc agggaggacg agagccagaa
1097281 taaacattca gataacggca caacttcaca actttaacgc tatggtggcc gtagatctgg
1097341 agcttctccc tgcaccgctg cttctacaga cacgtttcca gcgccaaagt tcctggtaat
1097401 ataccggggg tgggaacttc tcaagaactc ttcctctcac cccttgtcga cgtcaccagc
1097461 ttcgtgcttc ggcaaccagc gtgcgccagt tctgacaggc ttcactcaga gttctttcac
1097521 caaaaatcac ccaaggtttt ggtgatttcg gacggcatca catcttgctg tcagtcaggg
1097581 acgaggaaag ctcaaacctc actcatttga tccttttcag gagattattg tgacgtgggt
1097641 gttgcactct tgcaaactga cctcttgatt attttgtgac caagggtgtg gccccaacac
1097701 agaaggcctt attttaagag gagcaggtta ttttcctcgg taaaaacatg tcactcaaat
1097761 cccaaacccc attacacctg ccacgatcac atgaagtgga cttctgggct caaatgtgct
1097821 cagatctggg ccaggcccct gatgcgaggc cccccaggag cgtgaagtag ttagcttttt
1097881 atagaggagt gtcagaaagg ttcgctgcag gtcagagtag ggaaaaagtc cgactttaaa
1097941 tcactgcttg gataaaaagg ttatgtttgt ttacatcagc gtcaggcacc ggaccagccg
1098001 ggacatgtcc acacacttgt aaggtgcaac acacaggaat ggctttgttt ccagttttac
1098061 cagtctaggc tcctgtcacg agaagagttc cggtatgact aaactaaaat ctgtgaggtg
1098121 gagacaaatg tgccggaggc ccagaataat cgacaatccc gttgcgacgc cggtgtcggg
1098181 aggtcaactg aagccagcac actgggggaa aacacggacc gccgttcccc cgcacttcag
1098241 ttggcagaga cgcgcacgca cgggcggctg ctttgagagc gagcaggtgc gggcatgttg
1098301 ctcgctaccc gacagtgttc ccggcctgtg agggatcggg cggaacgcca cgctcatact
1098361 ggcgtcctga gcgcagacgg tgggagctga gatgatgcga gctgacactc cacgtagcag
1098421 cctttaatgt caccactggt cctcacatga aagaggccgt gtagccccaa ggctgaatgt

1098481 tttgtcagac aaactcactt atgagtggag gagaagtaga agggaaaaaa acaaattaag
1098541 gagacgaggg gagggaggaa gatttaaagg aatcctggag tcactctgct aaaataaatt
1098601 ggtttaaaga aagctaagcc aggaaaaatg actcactccc tcctgggaga agtgatccta
1098661 attttttctt ttggaaaaca cggcgttggt gattgaatct caaatgtaaa cagcagaggg
1098721 gccgtcgctg gtggcgggga ggggggtgat gagggaggag gtggagcaga gatacacctg
1098781 agagtgctgc catcacaaac accctgaatc atgtcaaatg aaagcagagt ccaggtggaa
1098841 ccgtgagagc aggaggagga tttgaggaga cagagacaag caagtccatc gcctggtgcg
1098901 ctggtccgca cctcaagctg tcagccccat ttggcacctt tcctgaagct gtgcccgtcc
1098961 tcccagtagc aggagctgga acactgcagg caccatttgt gtttgcaatc aattttacaa
1099021 cagaatggtc ctaattccgc cccaaaacgg agcatatcgc ccgtaaaaac agcgcttgtg
1099081 tccagaaacg ttagaaacat tagccagata tctgcagtcc tccagagttc aagtcagctg
1099141 cttttcccac ttccacagga gcccagaatc cttgttgctg ttaaatttct cctctaaaga
1099201 gaaaatactt gataaaatat cacatttgct caaatgccct taaactactc tgcaataagc
1099261 tgtttggcat ccaatgtgca aaatgcgatg tactgtattt acaaacagca cttgtgcgta
1099321 cttttacata aaccggctgg aagaacccag tgtgtgtgtg tgattaaagc acaaaaagcc
1099381 attgtgttat tattaactgg gggggaggaa gggtgagacg gagtgtggaa ttcagggagg
1099441 ggtcagcgac cttcctttac agccactcag atgacctaat tcgcagcaca ttgaaatcct
1099501 ctgttcctcc tcatccagca ccacccgccc ctcatttgac agcatcgcct gccttctccc
1099561 tttcactgga ttctgcacag cgtctgagcg gggacattcc ttccagtcag ctatctgggt
1099621 ttttaaatgc cagcttgacg cgcgcagcaa agaggctctt tgttcaccaa taaaagccaa
1099681 atagcacaaa acggcgcgac agactttgtg ataaacaaca acagaagaca attttacaag
1099741 tctttgcttt tttaaatctg tgaaagataa ttgattgtga ttataccctg acactcccag
1099801 acagtctgaa ctggtgctcg ctggtcatgt taggactgaa actgcattcc gtgttcaccg
1099861 aggagggtcg ggacggtgca tgaaggcagc acaacacact gcgattttac tacctcactt
1099921 cctgtggtcc tcggggaagg ctgcggcagc accgcctgct cttgtcaagc ctcgcagaaa
1099981 caacacgggt agctgaggga caggtaaatt attcatcttt gatttatcgg cgggctgcca
1100041 ggtagcaaaa ccaacggcga aagcgacaaa ccaagaggca actgatctgc tttgttcctc

1100101 cgagggaggc tggcgtatta tgacctgggc tttagggttt aaaccacgtc agcggcctga
1100161 cacggcctaa ttttaccttc tctgatcact gttacaggtt gcagcagttt tcctctgcaa
1100221 ggaatttatt cttgtagcag gggtttgaag gtgacactgg ctgcatacag gaagtcaagg
1100281 aaatgtactg tggcgtggcc acgggaagga aaacaggccc aaacaggaag gggaaaggaa
1100341 agactgtgtg gcagctaacc agtgtcgcct aaaatgtata aaacgggata aaaatatgtc
1100401 ttattatgca caaaaattac tattattatg catggaaatg tgggaaaagg ctgttcagca
1100461 gtcctgacaa gaaacagctg ttttactgag ctggaaagta actttactca tttgcactat
1100521 tgcacaatca ccgttttcct gacacccttg tatcaagatt cgcacatcct tccgaggcaa
1100581 ctgtaaatcg gctgccaaaa tagcattttc cgatgcttcc cgcttcagac gtctgcgcct
1100641 aaaactgtta ttttattacc atctcaagcg gtgaggtgaa atccagagcg taggaaaagc
1100701 ccagctcggt cacagacccg ctccggctta tttaaaagct ccgtttattt tattttattt
1100761 tttgaagcac aatgaaataa gacagagagc aaagcagccc ttctaatgcc cgactgaaag
1100821 caacaacacg ccatgtgagc gagtgtgcgg ggaaaacaaa catcaggctg gaggttaagt
1100881 ggtctgactg tgctcccggt gaaattcctg agctgcctgc caacgctcgg ttgaaaataa
1100941 atgcagggag gtgggcgagc cagagcggat cggagcggac ttttgggtgc ccccctcccc
1101001 cttttcttta ggatgacgta aacaaggaga gaaaagctgt gacaaatgct gcgggtgcca
1101061 tgacttggct tcctgtccat ctagctgtag tatttcgact taaatcggac cacagcacgc
1101121 taaagagcgc agcagggggg ggggtctgct gggactgagg caagtgtgat agctatttaa
1101181 agcactccaa gaattttaca gggtgcgacc ccgcgcacac aaactaactc gaggatacgc
1101241 tgagaaatac catgtggacg ggaacaagga ttctttgtgt cagatggaat cagggtgacg
1101301 ggcccacctc tgtgggagag ggcaggagag gagcaggcct ctcttgacat tgcagtgaga
1101361 aaacagcccc ccccccgctc ccctctgtct gtctgaagca aaaacacacc gctgattcac
1101421 ttccccatag tcccgttttt gcctggaatg ggtgttactt cgcgcagatg cgtttgcata
1101481 atacagaaga aacggggggg aagccacgag ggcagctgtg acatcaccaa acatgtctgc
1101541 accgctttaa ggaatatcaa agagaggaaa ataaatttcc cccaggactg tgttgtctaa
1101601 cagccggcgg aaatcaaagg gacggcgacg gcgtgcgaag gaggcagcca gcctgtccgg
1101661 gtagggcacg aagacagaag gcaaatggag tagcgataag cagacccccg cgctggcggg

1101721 attgaggtcc gagataagag ccccgagctg tctcgggatg actttatcgt tccgtgcctg
1101781 catggtggcc gtgtttgctg tggttagagt gggactccga gagcaagatg aagtacggat
1101841 gcatcggcgc taaggggagc gcggcgcggc gggggggggg cgtgtttgga aaacgggagc
1101901 gtcggacaca aacaggaaac ctctgagtcg acttcctcat cggcgataat ggcccagcaa
1101961 accggctcac gagataactt gtggttaatt aaattccagc ttatgtgccg cttcggggga
1102021 aaggaagggg cagtggcagg gggattaact ggttttccca ctggtctgac acccacaatt
1102081 ctctctccct gtcaatctct ggccaggctc gctcacgtct cgctggccaa tctcgttagc
1102141 tgctaaagtg attgtttgcg gaggttagtg caggtccaga ggagcgccga gggtgaacgc
1102201 taccgggaga cagcagggtg tcagaaaagg caggacaatg ggcataaaac caacacggta
1102261 ccccatttat caaaatatgt tctccaaaca gccgctcccg aaggagcgtt cccatcacag
1102321 gcgccacagt ggacagtttg gtctcctggg aaaaccttgt caaggcacac cgagacaaac
1102381 gggtggacaa aaaagaaatc agcagtggca gtgggggggc atcatggcat gaagctgcca
1102441 cttggggcac agcgcaacac agtacgactc aatctacgac agcgcctttt aaaaggcagc
1102501 tttccagaag ctctgattaa atatctgcaa acaggctcac cattctgttg cccacagtaa
1102561 aattccccat atatcatgac aaataacccc gcccctcctt tctttcccac ctccctttgt
1102621 cagcgctaca catccctgga tgttatatag gctgtctcct actgccaagg aaaaatatag
1102681 caatttgaag cagcttgttg gctggcgtat cgtaaagaga aataaaaaaa acctatagca
1102741 ggtgtcacct cggggagatg aacaactgtg acgcaggggg ggggcttcaa agggggcctg
1102801 tcactcggcc ggagtgacct tgagggactg cgcacatata tggagacgta acaggaagat
1102861 gtatgccact ggaaaggatg gggcagttg tcacattcac gcgtggtgtc agctttccac
1102921 tgccgaactg ccaaggaaac aactgcgcag atccgcagct gtcgaggggg ggggactcgc
1102981 taccaacgtt cccacaattg ggaattttca aaatgaaatg ggggggctt tttttcttga
1103041 gatctggcgt gcaacgtgtg ggattttaag cagaattctg atgacgtacc acaaaaacgc
1103101 ttgttccaac ggtttacatt tgtaccccaa atgcagcacc tgctcttcct catagggga
1103161 acgtgacacc cacccggaat gagacaactg gctgagaatt ccacagtgtc cgcagcagac
1103221 ggaaaggaaa acaacccgca gacagaaaga taagttgaaa attcccttcg gccgtgccct
1103281 gtttgacaga aagttacagt ggagtgtgat atttactgcc agtaaacacc cacctcggat

1103341 caaacaaaca ctaacgctaa aaaataaact ctcctcggtt tcaggaaatg
tgattatctg
1103401 acaggacaga acagctacag taccaaatca tcaataaccc atgtcagggg
gccgtgttag
1103461 ggtcgaactt tatacaaata ttctgcgaaa aaccgcatgt ttacttttgt
atttaatatt
1103521 aactgcacat tcatgaataa acactgggct ctaggtggga tcggcttaaa
ccggtgcggg
1103581 acacagggtg ggaggaggtc ggacttaaag aggcgcagct tgtccaaagc
agaagagcaa
1103641 accaaacagc ccataacagg aatcaggcgg ccgctgcacg ggggtgacgt
ttggcacaat
1103701 ttctggtggt tcgtgtgatt tctggaagta acgttgagtt tgggctgctg
ctgaggcaac
1103761 gctggtaaac catagactgg cgagggcgaa ggtcacacgt tctcaggttc
aaaagagaaa
1103821 tacaaaaagg gatcacaatc ttcacaacta acacaagttt ttttgtgtgt
cagttaaaca
1103881 caaggaagtt agtttagttt taagatgact ggtgaccagg tctccggacc
cattacttcc
1103941 cgctggtgcc gacccacctc tcttgtggtc acagctgtag gtaccgggag
aagtcaaccc
1104001 attttaaaga ctccatatat gctgattgtt ccatgggtaa cttacgtcaa
ccctccaatc
1104061 aggtgtgatt tgtgggtggg tgggggcaaa atacttcact tcttgcctat
ccaggcatct
1104121 acaccgacca gtttagcaac tacattccac tggggctacc cgtgaaaaca
tgcaacaggg
1104181 agggggagt gtgttctgct acacacgtcc tatgtgaccc gaaacttgga
tcaggacgcc
1104241 tacagtgttt tcgcctgcgg cgcgtccggc tctgcgttcc ccccccaggc
cgacccgcta
1104301 agggggctgt ggcgctctct agtgtcacat gctcggttac atgtacaata
agtccaacct
1104361 tccgcttagg ccagcttatg ttattaatac ctatcaacat taacggccgc
taaaccccct
1104421 gcgccgcgac acgatccgcg gcggcggcgc ggggagacgg atctcccggc
ggcgccgacg
1104481 tgctttcgcg ggcaccgagc acgcttggcc ggcggaacaa cgacggcggc
gggagcaaac
1104541 acgaccgacg cgggcattcc gtcgaacaaa cgacgggcca ctaaccacga
acgtgcagcc
1104601 gtttatgaaa aaagaaaagt tcgggtaatc ttggcgcccc cctcctccca
gcagcaactc
1104661 gctggccatt gtttgggcaa caagcacgca cattccaacg ctcggccgct
gggcaactac
1104721 taacccgacc ctgcggagaa ccaaaaaaaa gcggaaatca acttttctca
tacttggcgt
1104781 ccgtcttgcg gacgttcgtg ttcgccccgt ccgtgtgttt gccccggagc
gagtttaaca
1104841 gcctcccctc tatttccgaa gtgtgcagcg gaacgcagac ggtcgggcag
gctagcttga
1104901 aatgtggcta acccgagcta aaactgccgt ggaacgtgga cccgggcccc
gtcggaaatt

1104961 atagcaaatc cgcgcttggt gcagccaaag tttgctttag aactccacag tcagtcgccc
1105021 tgattgaccc gctcatggtg gccgccgggt ttggtttatt tacaccagtc agaaagtttc
1105081 tttccagtca ccaaaaactc gacagcggtg caacaaactc tggaagccca gcgttagcaa
1105141 cgagaccctc agaccccgat ttgctctctg tactcgggtt aaggctgccg tgctctcgct
1105201 gtcaggcacc agcccgggga gttacaagtg gtaccgagcc tgccacggtc gcttatagga
1105261 ccgtttgccc tggcgccgag cagcgagtcg ccctgcgaag tcgtgccagg accggactgc
1105321 gccttggtca tgcccccccc gccccacaca cacacactcc tcatttcccc ctgacgcctc
1105381 gcgtcgcccc gcagcgccgc ttaccttcgt cgagaagcct ttccaggtgt gtgaagatgc
1105441 cgcagagatt tggcaaactg gtcatgagct tcttctcgtt taacaattgc atcaaatagt
1105501 cgggactcgg cctcggtcgc tccttcactt cgacctctcc gaccatcatc tttgcggtgg
1105561 gagcggagag caaaaccacg gcgaaacaca aaatcccctt tcagtaaaat aaaaaaaaaa
1105621 gaaaagaaaa gaggggaaaa aaacttgcgg ctccaatccc tctgcttccc tcctccttgc
1105681 gatgttaaaa ctcagtaacc ccccctcaaa attcggattt ggtttgttcg cggtggtgaa
1105741 gccgagggtc caaaaaaaga aaccgttatc ttgctcccca acactcgcgc gccatacact
1105801 cctctacgcc gtgtgcgctc ggggacgttc gattccacca ctgtaggcgc gagagaccgt
1105861 tccgcggcga aaccccgccc tgtcgactcc tattggccga cggggcttcc tgggactaat
1105921 ttgataacca atgagcgccc acacacgagt cagaagggca gggcggagct ggtgagcgga
1105981 gtgattgaca gatatttgaa ccaatggaag gcgagtgcct agaaaagcgt ccaagtgccc
1106041 tgaagtgtcg ccggcactca tttgccttct tcgcagtaaa ggcaacagta caaaaaaaac
1106101 ccaaacattt tcagattcct ctatttttgt tactaaatga aggtaacagt gccctcctaa
1106161 ttcaaaaaga atacaaagaa ttgatattgc cggacaggaa aaaaaaacca gttttctgtt
1106221 ataagagaaa ttcctcaaag ctctgtttag atattggctg ctacaaagca acaaactact
1106281 aactaactta actactatta cacaaatcaa gaacggttaa agggggtgaa aacagttgca
1106341 ttcgtcaatc agcacgaacc tgatcctttt tctaaaataa cccccaaaac attccaatac
1106401 agctctttga gagcacagaa gttaaattca agataagaaa atccctttt taaccccaga
1106461 gctgaaatct ttttgagtga gaattgacaa ttttttttgtt gaaaatgtaa tgttcgcaga
1106521 gacaaagtct gtgtatatag aagtttaaac ctttcaccaa aatatatatt taaaaataac

1106581 actgcaaaaa atagccttat tgctcatttg tgtccccaac gtgatagttg tggttgtacc
1106641 gtctaacagc agcgttaata agactttcag gggcacttca cacactttgg aagaagcaga
1106701 ccgccattga cgaggcattc caggtagaat gttatccaac ggttgataat tatcgttgga
1106761 ggacttgaat aactagtttg gaaggtttca acaatggtga tcagggttaa gatgcaaatt
1106821 tcatgtgccc agaaactgtg gtatatataa ccttggcaaa tcaacacgtg agagtaatgc
1106881 tatacataaa aaagctaata tcagcataac ttgagcagtc cacagacaac aacatgcttt
1106941 agttgctaga cttgtacatc ttgttatctg tcttgtgacc gattgatcga cattcttgta
1107001 atcaatggag tctttttgtt gtcctgtcct tgggaggacc tttctacata ttctctgaag
1107061 attaacaccc ctgatcaatg gagcgcagag atcatccacc cccgacttct cacttccagt
1107121 tttgtgtgag aaggaaccac aactggttgg gtttgattgg aacagctgtc cactcagctg
1107181 tcggctgagt ggggcctgac taaaacctgg ctctgggggc tctctccaca tatctcaagt
1107241 cgaaggccac cacaccgaag gcagatgaac tatgaccttc cggccgcaac tggaaggtca
1107301 tagttcagct ttcaaaccac gccattattt taacgagtag caggctgcac aaactcttgg
1107361 tgctattgtg acacattcaa taaaatgatc gagcagaatt ggatctggtg gcgctcttcg
1107421 ataaactgcc actggcacaa gagacacgca gcgtttccac gtcgcagagt aactctaaaa
1107481 cctgccaggg aacatcacca acaccccatc aaaatgtttg ctatcagtcc tgaggctcca
1107541 gataagggaa cacagcagat ggctggagcc cctgcatcta cataccagca caggaatgta
1107601 ggtgtgacgc atccactgga tctgaaaccc ctgtaacacg gccagcatcg caggaggcca
1107661 gcgactcatt tcattattcg gccaagtctc cgacaaatcg agtccggtgc agatgcgaga
1107721 cgtcgctgat cttaaagctt tcatgaaggt ctcggcagct ctgataagcc tggatctatt
1107781 aatcaagccc actgaggaat gatacaaaaa caatggtatc aagctacaga tacagtgtga
1107841 ttactctcta tgtccctcat cttctctgcc ataaaatgtt tgcaaggact atttaaaaga
1107901 cgcaggctgt cattcctggg attgtctttg ttaaagaggc ctacagaaaa gtgccacaga
1107961 gggttgcgtt tgagagctgc catcttattt cgattaaaca agctgttgtc taaaataagc
1108021 agttcattgg ttttacgccg ctgtttgatt gttggtctga ttgttattat ttccaacagc
1108081 aaagcgctga tggttcttat ttgcataatg gcccgtcatt tgcaccttca cctccatata
1108141 ttattcggcc cgtcgaccca acggtctgca atatcaatgt gcatcgtggg gccgcggtga

1108201 taaagcgtcc cttgaagtca aactctgata agtgatttcg gcggatagga tcaagcaaag
1108261 gcagagcgaa ttggaatcgc cacgtttcat gtgacttaga gatagctaaa ttgtccacgg
1108321 tgctgacttt atgtccctct tttatttctg cccacaatct ttaaataaaa acggcggtaa
1108381 aagtcacagg ctcctggcat caaaaagaat ctgtcattta tttttcagct ttgaaaacag
1108441 agtttcagtt ttgggttttt ttttcctcaa cgggaagcgc ggtaatttgc acctcgcctg
1108501 acgctccttc ctcgggtggc agacggcctc tccctgcgtg tgtgtgcaaa acagagacac
1108561 agattaagag acgtggtgat acggcgcaga aagggcgtgc gagagcccga gagaacgcag
1108621 aaactacatt aatccctcac atttctcccc ttgcccttgt gtttttgtca tttctaataa
1108681 catggtttct attcagcaat taatccaatt aacagggatc cagataatta atgaacatgc
1108741 atttagtccc ttttgaagag gggctgagcg ctcgtcacaa tgggctcagg gagcaccctg
1108801 ggtttcccgc tccaagtgca gcatgttgac gggacaatgg cgtcctcagt ggggacccgg
1108861 tggctggatg tagcctggct tccagggctg cagcctgagg gaagcggctg ggagacagcg
1108921 gttctgtcga ggcaaggctg cctgagataa gggctgctgc tccagaggag gccaacgggg
1108981 gcccacgggc tccgataaca cccctgcacg caaacatcac tctcttttc tttttgactt
1109041 cctcgtctgg gggctgaggg ggaagaaaac agcggaggaa tctgggtcat ttacattttg
1109101 aggaaggtgc gaaataaatg tccttgtagc aaacacggtg tacgcagagc gccgcataac
1109161 gccgccattg ttcagatcaa attgtatcac atgcacggcg tgaggagtgc acgctttgtg
1109221 ctcgcgtact tgtttgtgtt tgtgttacgt attcaacagc ggatgctcat ttgtaactgt
1109281 cggcagtcga tctttatttg tgttgaagtc aggggcatgg acagcacgcg gcgagcagac
1109341 ggcgccgcgg cgtgatgcgg gcctgctgcc agccataggt ggaggagcag aggtagtcaa
1109401 acgccattta aatgtcgata cgtcgatagc tctccgagac cgtcgccaca taacagcatt
1109461 tattttgatg actttgcaca cttaaaatcc agagattaaa gccaacaagc acggcagcag
1109521 agggatgaca ctttctggcg gcttacaggt agcgtttatg agatgagagt aaatcccagt
1109581 tttatgcttg ttcaactgta aatgaaaact atggcgacac gggcagcatt cacttgagtc
1109641 acggcgccgg cgatgatcac agggatcgca tttgagctgt atttacatga cacagatttc
1109701 taatcctgtt gtttagacct aacacccatt taaatcctat atcttgactt cagccggtct
1109761 aatgtctggt gcacattctt ttgacgtggg ttcggtctag tgtcaggggt gggcatctcc

1109821 ccatcgtgtt acatgtcaca cccatgtgct gacgggggta cggggttgtt
acagtccagc
1109881 tccagccgct gatttcagtt cttttgttat tcctaatgtg gtggaaagag
aacacagact
1109941 ttaaatagca ttatcccgcg cctctttctc tatcagtggg ggatctattt
tgggctcact
1110001 cccctccctg tccccttgtc ttcatttaga aatttctctc ctatcttaag
gtatgacttg
1110061 acttttaatt tagcccttca cactacagag tcaactgatt caacagcaga
caggcgcctt
1110121 aaaaaaagag gaagtttatt catccagatt tgactgtttt cctcttttct
tcgactcctc
1110181 ccgtctgcaa gaattggttc ctcttccaag ggctaatgga cgccatcctg
ttgggctaac
1110241 tgaccctctt ttttagaaac tgaattttat cataaatctc catgcttatt
tctatcattt
1110301 attggacttt tgatcattta taatgcgatc caaccaatcg tgcgctccta
taggtgtggc
1110361 attaaaacag gacggacaaa ttaagcttta cttgtttttg ccagttagca
taagagagcc
1110421 tcctttttat tcgtctctac ttcaatttca gaccagagaa gcagtgcttg
gtgcattggt
1110481 ctgaagcttc cattaaagag cccattgttc atttgttact attcagagag
ccagctgagg
1110541 ggctacaaca cagtctgaag ctgcttagtt cccacagttc agagacaacc
tctctctccg
1110601 ctgctgctat tcgccagtta tgttttgtta aaggtcacct tgtgtgttgt
cgttgagatg
1110661 cttcagtgcg ttttggcaat ctggtgatgg aaatgcctgt tggtctgtgt
gttgttgccc
1110721 agacaggatg ggggtgtgca cgcggggcgg ggaggggagg ggaggggtgg
gggttcgtgt
1110781 tgaaggagct gtgacagctg gggccacttg tgatagaggg ctgacgggaa
aacattgcgt
1110841 ccaaactcca gcctcgaacc tgctcgtcat caatcaaatg gagaaaatgc
cccacaaaac
1110901 agtggcacgt tttacagcgc gggggcactt aagcaggaaa aacagcacac
ccaccctaca
1110961 caccggtgtc gatacggctt aaaggagccc tgccgatgct acatctgtcc
ctgccaattg
1111021 ctaacagcgt ggatccattc ctactccgag cgaagctttg cctttttctg
tgcacgcctg
1111081 ccagccacat agcaggcgaa gaaatgagtg ttcgcgagcg ataagaggag
gacagagaac
1111141 agagcgcggc gaatgacgga gaagaatgct cattgatcac gtctgtttag
aaaaggtatt
1111201 gatcctgaga tgtgggatag gaacaggagc aagaagagat aagagaaggt
aaccagttaa
1111261 tcgggaggag attggagtca actgtaataa caacttaaag gggaggtttc
aagcaggagg
1111321 agcagagcgt gacccttcc ttcaaagtgt ttccattccc cctgcacttg
aattaagtct
1111381 gtaaaaacag acgcctggct tatggagagg cagacagatg gatctccact
gcctcacaca

1111441 tgctcagtag ctccctggat gtgcctgcag gccgccgggc ggacctgaat aagacaaatc
1111501 attctcgggg gggcatttat ttaagttgcg ccgcaccatc ccctcattgc tggtcctaac
1111561 aatctgcctt ttgagacccc gacatcggaa taagtgagac agctcagcaa aagaagggac
1111621 aagagaaagg aaggagggca ggaagcgata aaggggaaga cagatgaaga cgtccccgct
1111681 gcttaataaa tcctccagaa tacgcagggt tgcaacacgc gccgccggaa ctttgatcac
1111741 gttaagtgtg accagcgcgg acgagttctt gaatttcaac acatgtgagt gtttgttctt
1111801 gttctctttc agaaaggggg gggtgctgat gttgctaaat gcctgttgtt gccatcctct
1111861 tcgtaaacat ggggggaata aacagatagg cgaggctctt ctttagtctg cagccggctg
1111921 ctcttaaatc tcggctcttt cttgatttag acagctctct ccaccaggtg acacaacatc
1111981 ccgctccctc ctctccatcc atcttggcga ggcccacaca gcctctcttt ccatcatccc
1112041 tctcagtgct ggatgcatct cggaccgccg gtgcgatctg ttacactgtc ttcatccatc
1112101 cgacatcctc tcggtcccaa acgcaaacgc cgcaaacctt tcacggtgcc ttttcctcct
1112161 ccataactcc attttcccgc cttctctggt ctccgccagt actctgcttt tcccaatctc
1112221 ttttcccccc tcgtcaacag aacgttatcg gcacgcttgc gtagattttc ttggagaaag
1112281 ttgagctcag agtagcgctt tcgcacagcc accgtcctct ctgagataac tgctccacct
1112341 gaggggcaga gccgccgtct cggtacaagc cagacacttg tccacgtgct ccgtgctgct
1112401 gatctgtgag cccgcgtgca ttctgcgcat aaatcgtgca gaggcgagtg ttttcatcag
1112461 ggcaacggct tgcatgtgtt cctcagtggg agttattgct ggctgcgggg cagtggcagc
1112521 tgtcgtgagt ggctgagctg gctaagctaa taataacagc aggagctgag tgagtgctca
1112581 cgggcccacc tcatccaagt ttgacccttg gagtccagtt gcagccgact ctgagggccc
1112641 agcaaggttg gaggtgcact cggtgggcac ccctgatgtt gggagacccc taatgagcca
1112701 ctttcacagc ctgtggaaga cattttcgct gtgaaaatac acgcagcagt tttggaggct
1112761 gtccgttaga atacagctgg caagagattt gacaaaatgg ctgaatgaga cgttacagcg
1112821 acacatgtga catcagaggc agatctgtca cagcagaggg ttcatcaaaa tagaagaaac
1112881 ctgatgcgtc aatgatgcgt ctccgtctgt gacaaataga atctgcagaa gcgaatgaga
1112941 gcacggtgct ccaacctggg ctccatgact cgaaagaaag acccttcatt taaccggggt
1113001 caccgggggc gtaaaatgat cagttgctca ttatgagcaa ttcaggaccc ttttttttggt

1113061 acatccgagc acagaggaaa aggagggaga aagcgatgtc atgcttggtc
ttcccagcct
1113121 gcgcgtctgg accagccatg ctccgatgcc tttgttagaa caaataacct
cactggctgc
1113181 ctccattccc gtccatttca cacctcgctc tattacttgc acgagcaatt
accgcattgc
1113241 acagttgttt gttactgcat cctttaccgt gtgcgagagt gtgcacgcct
ggagaccgcg
1113301 gcgttcacca gcgttccttt taagactagt cggggttcat tatgaggttt
gtgaagaggt
1113361 cgaaacggac aggatttagc atttaacagc cattagtgtc tgtgatgtga
ctgaagcgaa
1113421 ccaaagcaat cacactacct ggtgatttaa ggacgatgga gtgaactctg
ggtaatttct
1113481 gcgtattatg gctcgtatga cattgtatgc tagctggagg cgaaaggctt
taatgagcag
1113541 atggagaact gaaagtttct aacgatgaaa attctaacga gctcatgtgc
tgagaaataa
1113601 tgcgcgaagc ctagcgacgt ttccttccct actctgctca ctctgctggc
tggctgctag
1113661 cctgtggctc agtaagattg gacaggtgga gtgggacagc agggcatcgc
tgatacagtc
1113721 tgacagtgct gcgggccgcg ccgagtacga tcggctcatc aaaccacatt
tttcaccgct
1113781 tttctttttg ggaaaatatc ttttgcagcc ccggctggtt ttgtttcatt
aggcggctgt
1113841 agttgttaaa aagatcaatg tgttttgtgt taaaagggaa ccactctgca
cctcctcttc
1113901 ctgtcagcgg gcagagcgcg gcccggttca gcagggtgga acagctattc
agtaacccag
1113961 gttgcaacag gagcactatg aacctgtctt ctggatgaga acagaatcct
ccacaggttt
1114021 gtaggagaca cgacttacaa gagatggtgc aagagtcttt gtgagtgtca
gaaatcaatt
1114081 acgctgtttc cccccgtgtc ccgcagagtc ggatatgctc aaatgtgtgt
ctctgcgcac
1114141 ggggagagta cagttgcgag cgcctgcaca gtgtctggga aggaaatccc
actcttctgc
1114201 atagacctgt ttcaaagcca gaacataaag tttctgactg gcagagaaag
aaaacacgcc
1114261 cgctgtcatg tacaaagcat gtcacaaaca agcacgcagg caccggcgtt
tctcacccca
1114321 aacgctgagc tcactccggc actccagttc aaaggatgca tgtttgtgtg
gaggaatgct
1114381 gtcggtcgtc attccctgct tgtttgggca ggccctcgct ccagctgagc
tcgtgacaga
1114441 atgggtacag tgacaaaaca caatgcaaca gcaaaaaaag agagagaacg
cagagaagtg
1114501 agtgtgaaga gcagcagtac agcaggacag aacacacaga tgtccaacac
acactttatg
1114561 gtctgacgta taaaactact caccggaaat ctcccacccc cccccaggtt
tcattcacac
1114621 ctccttaatt aaacgctttg ctatttactg aggtttaaag actgaatgaa
attttaacag

1114681 gaaagattta ttattaagga actaaattta agatgacaag gaacatcaat acaacttctg
1114741 atccttttta tatgacacaa aacaaccccc ccaccctcct ctcctttggt gcatagcagt
1114801 gtttgaaatc aaatgtgcgg cgttgaaaat tccctttaaa gtagcagtaa ttccccacag
1114861 atgcacacag caacattgat aggcacaagt tccagctgcc acaaaacgga cacagatgat
1114921 gacaataact cttgatttcc ttcgtctgga ggtaattagc ataggctagc tagcgctgct
1114981 ggaaaataca atatcacaat attcgagatg atgagaagtg aactcagcca agtcgagccc
1115041 cccgtctcct tcagttgagc aggcaggact cgtaggtggg caccatgtat ttgatattta
1115101 tgaaggcatc ctctcctccg tagcgctcga acatctccag agtctcctgg atcagatcgc
1115161 cgatgttctg cctcttgtgc tggctgtagt cgatgccgtc cccggtgtta actgaggagg
1115221 agagaacaca gggtgactca gcggcgctct gtgggggcgg catcagggct cgccgggcgc
1115281 cacctgtggc ctcgccgcga cgccccgctg caaattgagt cggcaaatgt catctttccg
1115341 tctgatttca aacccaatgg aaggaccgaa ttgaatttac tgctctcatg ccagggccag
1115401 ctgggactga gcagcagaag agtttttaac tgttccgctg aaagtttaat gaggcggcct
1115461 gcagagagta gtgcagcgta aaggacagct agctacggct gcgctcttgc acaacgctgc
1115521 gctggtgcac atcaggcacg ctcactgacc tgcacactct cacaccacag gcaggtgagg
1115581 cccgcatgcg gactgttcaa gaatgcagga agaactcaaa ataaagccgt cgcgctcatc
1115641 ccgagctgat cataatttag aacaacaccg tcccgctctg ctgtggcgcc cttttggatg
1115701 tttttttgaa caaagggaaa agaccctggg tgaaatgttg cacccattgc gattgcagac
1115761 atcctaccgg tgcgccacaa tcaacaggtg tgaagcatcc tgtcgtcttg ctcacgttac
1115821 acttcgattt ggctccatta cagccctcac tgctgagcta acggcagaac acccgtcccc
1115881 cattcggtgc tcatacagtc cccttttaga gagaacgctg aggcagaata acaacacatt
1115941 cctccgtcaa acgagaattc tcacacaaca attgcactgt aggcagtctg ggggactcgg
1116001 gggtaagagg gaacattcct gagttatcta gaccgtcact cctttcttct ctttcaccgg
1116061 ctaaagattt tattttcggg gtcagacctc gttgcgtaat gtttacacac actaactttg
1116121 catttttttt tagaaacatc ccgaagcctt ctgctcaaaa gtgtacctgc gggatgagga
1116181 atgaacagca gcactttgag gaggaacgtc ggccccgtct ttcattccta attaggcctg
1116241 aggagacagg atgcattaaa ctgctcttct ccgtccttta ttctccctca gccttttcta

1116301 atctctcatc ctccgtgaca gactgaacca cccctctctc tcctctctct ctccgcccgt
1116361 ccgtggcctc ctcattaaaa cacagagcga gccggtccca taaagcgggc tgttaagcag
1116421 caacatgtct atgatctcag tcggctccac tcactttttg ctcaggagtc actctcctgt
1116481 ttaacattaa atcttccagc caagctctga ccgctgctcg gcctgtgcaa tgcaactcac
1116541 agctttagct gtacctggca gctacttagc aaccccctttg caccccccccc aacccccccct
1116601 cccctcgaca acaatagctg catgttctca ggaatgttgc cttcaggaac cagctctcct
1116661 ggtgcatcac agttccggct ggttcaatct tggaaacgct acaagggtta tctgcaccct
1116721 gctgctcagg tcggtccgga agtgccaaaa tagtttgttg ttcgctctgc caacaacaac
1116781 tgagaaaaaa aagatgaatc aataaaataa caatatgtca gagagtcatg agattaagag
1116841 tacaaaacaa ttattggtaa ccttaattcc tgaaacactt ctaataagtt tggctgtgac
1116901 atctgcttgt caggaatgtt gtctgttgtc agggtgagtt ttattttgta tgggataaat
1116961 gttcacaggc tttcatttgt ggtttcactt ctctgcagca acaaaataat aaaaagtgga
1117021 aaagaatatg taagattttg catggataat aactgatttt atgattcaga aaagttctca
1117081 caagaggttg atgaatttgg tgaaattttc tgtggtgact agaaaattat ggtttcgcac
1117141 gatcacatta agagtcgtgg ttttcaagtc atcgatttga gcaataagag ccacggcaac
1117201 gccatcgggg agaatgggcg ccctggttct gtgatgtggc cgttgtttgg cgtgctggtg
1117261 aaaagaccag attgcttttc tagggtctga aagctgaaat gtgcatgtgt gtggtgtgtg
1117321 tgtgtgtgtg tgtgtgtgga ggcatgatgg cgtctctgga ggggcgcccc ctgtcagaga
1117381 gaaaatgagc agccaaagac agcccagggg gagagctcgg ccttcagact gtggaacaca
1117441 gggagccgtg aaagaaggga gaagcaaagg gaggaacaag gaggtccagc gcgggggtgg
1117501 ggggtgggat gggggggggt ggtgttactg ttgcaacact gggagtttca gtccttcaaa
1117561 gtgcatttct attgctgcgc tgctgggagg ccaggagcat caaaaggaag gggctggggg
1117621 tggggctggg ggcagaggaa tttgcaataa ttctcaatat ttaaagagca caaaagtgag
1117681 gagacggttt ggggggtgag ggcggggcat tcaaagctgc ctctgcaaat gtccttcagc
1117741 aaaataactt ctctttttcg ctcttctcct ttttcctcca tttcctccct ctttatttcg
1117801 ccagctttag gcaatatccg caactctgag cacagcgagg ggtgtgtggg gggtaaaaat
1117861 ctttaaaaac aatcataata aaaactctca tcctgtgaac gggggggggg gggggggggg

1117921 ccagctgttt gttcctctga ggaagaggaa gggtcaatgc agaggcagag gaaggaagga
1117981 agcagcagca actgcgatag tgaaggtaaa ctgcatcaac acaccacatc aactggcgtc
1118041 catattgtgt agggagccct ttcacacatg caggcgtaca gcgcagcgac gcgtgcgtcg
1118101 ccccgcatgt cccacagctc taaacaaccc gtcgtcccac atccagccaa gtcaaggtcc
1118161 cacagaagct tgtgagaggc tctgctcaac cactttcaca catgtggacg gcagatttga
1118221 gaaggggata tttagcggct gctgatgggt ggttatcaga ccacaacaga gctgtactgt
1118281 aagcacaatc ttaagtttcc tgtcgggttt ggcatgagtt atgtgtgcgc cccctgactg
1118341 taatcccctc ctccaccagg gacagggtga caccgactca cacactgcag cggctttggg
1118401 aaatttagcg ctgaattcca ggcccgattc gacagaaaat caacacacct gccattgttt
1118461 ctaatcacgc cgaccttcgg gctggaagtg cctcatgaaa gcagcttaaa gcgcagcccc
1118521 ccccacaaca caatagctgt ttgatccaag cagccagatg cagagcagaa ttgatgcgtg
1118581 cgaggccggg tctggccttg tttataaagg gaagtgtaaa aactcgggtt aaaagcggtt
1118641 tgtgtcagag gaatctaacc aaagctgcac agccgggaag gggggggggg ctggaggggg
1118701 ttcattcctg cctttaacca catactttcc ccttcctgct tttatctaaa aacccaattt
1118761 tctgttccag atcagtagca gactagtaga cagagacgca tgacagcgag gctgcaacct
1118821 taaaaatccc tgatgttcag gcagttgcag caaacacatt ggtgcatcag aggaacttca
1118881 aactgctgtc acggctgtct gcgttacgtc acttctgttg gcgtgtgaaa actttaggag
1118941 caacggagac gtcccccct cccatggagg tctgcctgga ttatgatctg tgtagaaaat
1119001 tagcagcggc cactcggttt gagcttctgt ccttccttcg gcattacgca tcaagacgtc
1119061 aacgctgccg ccaccagtgg tttattgcaa cacatggctg cgcgttggag gcggcaggaa
1119121 tgtatagaaa ggagtgggac attctgtgca tacgtgtgag aaggtgtttg gagtggatgg
1119181 ggccgccgga ggaggtctac gcctaacagc agggctctaa ttaaagggcc tcacagtgag
1119241 acggcaaaca gacctctcat tagaagacgc ttggcggcga ggtgggtgag ccaaggtgtg
1119301 tgtgtgacgt ttgcacgcgt gtgtttgtac tgaaccagcg gtgtctgctg cgatagggca
1119361 ataagcttaa gtgggacaca acatccaaga cagggtcaaa aggccaaacg ggggaaaaca
1119421 atctgcctgt caggtatgta tgagagactc gtggttatga acaccgactg atcgtttcat
1119481 ggttatcaga gtccacaacc cccctttac cttaatggat cagccagaac ctgacgggca

1119541 gaacagctgc cccccaccta caccatcatg cacacaccca aatctggagc
tgggcccatt
1119601 ggcactctgt tgcttctgtt tgcacttcag ttcaacactt ttagcagcaa
taagtgcaac
1119661 tttagcagcg ccgctcaaac gcatgcgagc acaagcagaa cgggagtcca
caggccgaca
1119721 ttttcctgtt gcccttgctc actgagggct aacctttcct gtaggtcagc
gatctcattt
1119781 tccacgtgtt tatcgggtcg agctgtgctg tctctacaga cccgctgcag
ctcgctgcca
1119841 ttttggcccg gagaaagttg ggcctcagcg aagcggcaca ggtaggagcg
aggcgtcaag
1119901 gtcagactgc agcaagaacg caaagttaag atgcaacctg ctgaggaggc
agccccagaa
1119961 ctaatagatt tcaatcgatc tgagttcctg gtctacatca gtacatagat
ttaaaggaat
1120021 tgaactcaat tggacacttc cgtgccgcag ctttgtctca tcattccttg
gaaatccccg
1120081 gcggtggaag ggattcagcg gaacactcag acagtggtga aatcactctc
ccatgtctca
1120141 caaaactcct ttcttgccca tttcttttcc aattagccat caagaccgag
ccgtgaccgg
1120201 gaacaatatc agtttgcagc atttagtcat agcattaatg tacaaagagg
gcacgtctta
1120261 ctgtggatga tggatcaatg ctattttcca agctgttaac atgtccatta
ttgtttcaat
1120321 taccggcgct cagataatgg ttatccctgc gccatcacgt ccaatcgcag
tactgcaaat
1120381 ttgctttagt atgctcatca cagctctaaa agtgtcgtat cacggcgacc
gctcgttgcc
1120441 tggaggttca gccgggctgc aactttttag ttatttccca attaggaaac
atgttcttgg
1120501 tcgcaaaggg cttctgcggc ctcaattctc atcccagctg ttcagaactg
agcaccgatc
1120561 caaaatctta acttccagtt caactgtgct gcacccgcag cagctgcagc
tgtctgcatc
1120621 cagcaccctg aaaggctgga gtccatttac tgaaacccag taaatgctct
cctcacagag
1120681 gacctagcct ttaacgctct gcccgtcttt gtctctcccc tgctgggagg
agagtggcgg
1120741 tgctgtctgg cgcgagtcga tgccgtcaga tgcgatctca ttacgccgcg
tgcctcaagg
1120801 ttagtggtgg gctgctggct aggcacaaaa tcgatccgca ccagggacac
acgagaaagg
1120861 ccacacaggc caagcaagcg cgccactttg aaatgcaaag tcggccgtac
acgtgcgagc
1120921 acgccgctcc tggcgctgaa ccaggagacg gacacagtta caggaggtct
cactagtgtc
1120981 ttatggagag gtgcaaaccc acctcctggc gcacgcctac gctgacccac
atcgcatcaa
1121041 gctatttcaa tcacctagct tgtttcttgt gacacgcggg ttacgtcagc
acccattatt
1121101 gtcgggtctt attacacgga gctgatgaat gtttactcgg aactgacatc
agaaacgcgc

1121161 cttttgattt ccgacgtgtt tgacagaaat ccgataagca tcatctcttt cctgcccgcg
1121221 cgcgcctgaa aatgtcaggg cgccattagc tcttcagatc tttgcatatg gagatatcaa
1121281 attaatggct gtcaggaata ataaataaaa gcaggggata ttttattccc tttctgggtg
1121341 gcggacgacc ttaagaacac gagaagagat gaatattcag aggaaacagc ttggctggtg
1121401 cacctttgct ctggcagcag gtgccatgct tgtggaattc agcaccttat taaagctacg
1121461 cctctaattt acacatttta atggatacgg aggccatatg tttaggtttg gtaattactg
1121521 agctggtttt tgttgcagtt tttcttcagt tttactttcc ttggaaatca gaagctactg
1121581 aatgaaacat tagagagcag attggacagt gtcgttattg tgtttcatta acgccttcgt
1121641 ttatgcatac gccaccactt tacagagagg ataatcacag cgtgcagata gattaatgga
1121701 acgctgaatt tatgtcctgt gaaactactg tggaggcagc ttaagttttt gctttaatat
1121761 taaaaaaaag ttttttttata aaagttttcg ccaaactatc cttcaacaca gcagacctag
1121821 catcgatgcc agttttaaaa tttgaggacc ttttgtgttt atcctgtatt acagtttgct
1121881 gtgcatgtcc atgttgaata actctgagtt tatgtgtttt tattcacgca cagaggacct
1121941 tttgaaaagg cctcagcaac ggtaaaatag agagttggaa gggccgcgcg gaggaatcga
1122001 agaggcttaa ggagtgacaa agccaggccc aagtagacct tctctggaaa aaaacctaaa
1122061 gaattagatt aaacaggttg tcaaaggtgg gacgggcgac aacgggagga gtggacccgg
1122121 acgaatcgta aggacagccc acctccggac ggcagatctg acggtagatg gagaaatgag
1122181 aggaggccag gcgctgcagc agatccaaag agcgccccgt tcgtcctctt cgctcaacgc
1122241 aagaggaaca tttgaagtga cgcgctaatt taagtcagcg acaaatcaaa gcctatcact
1122301 gatattaccc taaagcgtgg cgagtagcct ctcgccgctc cacatttttg aagtaaaaaa
1122361 actgacggcg gttgaaacat caaaggaatt aggtgcgctt gagttcctta catgaacgca
1122421 cagcaaaaac agtgtcatga ccaaacgccg tggaagtctc gacatgctcc cgctcaagac
1122481 acaagcgctc gcgtccacag gcggcgatgg gcctgaaaac taagatgatt tgagtcttca
1122541 taaccacttt agccttggaa gctgccatca atggaaggac acacacccat ccccgcacac
1122601 acacacgtac aggagcgagc ggtgaacggt ccagcccttc cattagccta atgaagtggc
1122661 tctctaagat ggatgacatg acatcacctc caccaccaat cacgatataa cccctaaaaa
1122721 accttccact aaaagcccca cgcctccttc ggctcacccc atctattgct tactgcacca

1122781 aatggacgat gagcgtccca gggccaattc ggcctactca cggcactttt attggccgcg
1122841 ttatttatct ctttaatgca atcggctgga gtggatgcct atggattcaa agggctcgta
1122901 aatcacagtg agtagggacg gtggtatgat taaaagaact taggctcaac aatgacaatg
1122961 gcaccgagag gacggcgtgt caggacaggc cagcgctaag tctgaggcca gcaggagaag
1123021 acgatggttc cttatgacgg aaataagaaa aacagctgca ttaataaaca caaaggctaa
1123081 caactgaaaa caaagagaaa agagacggcg tttgcctttt tattgatttt ggctttcagc
1123141 tcgctaaagg acgtgacgtg aaatacatct ttcttgatgt taccgtctca gtttcctcgt
1123201 ctatttgagt ccagaaggac gaagggaagg ttgtacggct gacaggacgc ggcggagaca
1123261 cccattaaca gtagagttta caagcagcgg cacagcctcg ggtctgtcaa gctttacgag
1123321 ggcctgagcc ctttaaagca ggagtgttta agcgcggtat ttacataaaa cagacagagg
1123381 tgtgtgtgta tgtgtgtgtg gagccagctt cctcgttctc aggtggtaaa actggagcct
1123441 ttaagttatc agcaacagaa gcgtgcacgc aaacaatgaa atagtgctgc acgccacgaa
1123501 tatgcggccc ttccaacggc gacgccagtg ccccgtgcac gcgccgggtc aggctcggcc
1123561 ccaagaatga cgcagaggca gagaaggagg aaggagaggg tggatactga gatgtgctgg
1123621 gacagcagca ccaccccgac gtgagtcatc ggacacgctg tcagcagaca tccaacatca
1123681 cacactgact cactacgctg gaggagacga gatgacaaca gtcgccacga tggaggggag
1123741 cgggagagga cgtcacaggg agacgcagtc agcagtcatg gcgacgccgc gctgctgcca
1123801 gatatacagt gtacggcggc gtggacgcca ccctcctgag ctgggtggcc tctgagggaa
1123861 aacctctata gatgatggtt tcctgtgcat tctgctcccg gtgtagctgt tttttaattg
1123921 gctgctccaa tatgaatccc attattagcc cggcaccttc gatgagcccg tcaacaagtc
1123981 cgattatgtt taaattgaaa ttctactgcc cactaatacg gaaacatgtt tatttgaagt
1124041 gttgaccgtc aaatagcagc tgatggcgct ctgtcttgtc caccctttaa ttaaacactc
1124101 gtgttgcatc atcacttgta atttgctgtc gcagaaaacg cgctaatgaa actacatcat
1124161 cgttaacgtc aattcatttt taaatttgtt gttcttatcc cgcactgagc ctgcttatga
1124221 aacacagacg ggaacggaca caggtgaatg aaaagctacc ctccgtgctg ctttcagacc
1124281 cggcctccga aaggaccccg acgtgcaacc tgcccatcaa aagagggttt ttgcattgac
1124341 agaaatgtca ctgaattgaa aagggaatct gacgcgataa agacacgctg tccaaaatac

1124401 cacggctgct tcaaacaaac tcacggatga aatcctgaaa aatgccattt ggggagtgga
1124461 aagcgaaatg ccaccgtgcc gattcatgcg gttcctcggc gcacgctaca ggactggcag
1124521 agacccgttt taattatcgt ttcctctcct gcagaacaaa accggctcgt gttacagctg
1124581 ctaccgtttc cgtgggatct gattggggat ttgaaatgtg attaactgtt ttgtactcca
1124641 ttatgtcaca gtcacatacc gtgtacgcac aatttgaaca ggcgtctctt cggctcaaaa
1124701 aaaaaatccc gtgtcgctgg ggcgagcgtg tcgtgttcac tcgagactta taggtcgtcg
1124761 tctttcgagg ctgatggttg tgttcctggg tcgagctttg tctcctggac aagcgggagg
1124821 caggaccatt aagtcagcac agaggggacc atttgtctgg ctcttttctg gctgcggctg
1124881 tattatctgg cgtgatgcta tttgtctgag accagccaag ctgcggatgt gacaggatgg
1124941 atgaatgatg ggctcccaga cgcagccggt tttgcttcgc cgccaatgtg gggcaagaag
1125001 ccagagttaa gcagcaggga gggcgaggag gaagagcgac gcgaaggtgg cggcgggggg
1125061 agggggcctg gaaaagaagg caaatggagg acgccgcggt agccgatggc caggggacac
1125121 aatttacggc acctgttggc cgtgactgat caaagcacgg ggaggtaatg gcgtgtgtgt
1125181 ggtctgacac cagatgtagc gatggctggg aagagtcaca cacacacaca cacacacagc
1125241 agaaaagacg tgtgagtgaa acgaaaaaga aagacgggag aataatagag gcgggagaca
1125301 aagggctgca cggagagcaa agagaagggg agtgagggac gaataaagag aatgaattgt
1125361 tggtgcaggg atgtgaggaa aatagacaga gggagaatat atttgtcccc tgatgggagt
1125421 gtgttaggag tccatctgtg gagaaatgag atcagtggca gacttagtgg cagaggaagc
1125481 ctcggctgcc ccgcggctta cactctgact caacatcccg cttgttcagt ctgctctcac
1125541 acaacctgca gcgcatttaa tctgccggcc gctcagtgcg tgcgttcgcg cccgtgtgca
1125601 cagaaacaga ggtttataag cagggctcct gtctcacgcc acctctccat caaaggaccg
1125661 gggcagcgtc tcctgctcat ctctaccttg ctggctcttt ggctccttcc tggctggcac
1125721 cacggaggaa tctcctccta gaatcagcca ctaaatcatc tgtccgccac aattaaccct
1125781 ctcacccgga gtttttactt caagtctctg tgtatgtgta gttctaagat gtccttcacg
1125841 gtcctcggat gaccacctag accagccata ccagacccac agagaggcct cacccaacca
1125901 caatggttac cgtagttaga cctctagttc agttgtcctg atgggagcaa tttagctatt
1125961 gcaataatgt caaaaaacac aagcaatgag cacgtgatga cacacacaca cgtcagtcag

1126021 atggtgcagc agcatacgtg actattacca ggacacacac gtaatcttaa gaagcaatca
1126081 acaacaggtg cacgcacgct aactgtgtgg gtgatcatta tctgcacaca cccccattac
1126141 atggtcgtag taaagccgag gctattaaac atgctaggcc acccaacaac tgattaatgg
1126201 tctgttcaac tcattacata caaggtcaat gggaaaacta caaatcaatg taaatttgca
1126261 taataaacat gaataaagct aatgtgtaca gtgattatgg tgcatatacc accagacaga
1126321 cctccccccc ccccgggaga tacctgagag gagcccagct ggctgtggtg atggtgccag
1126381 gtgagagtgg atggtagaag cctgagggag gaggtagatt agagcgtcag gaggccgtcg
1126441 ggggaaagaa gcagaaggaa aaggatgaag tttgatgaaa gaatagcaga ggagaggaga
1126501 tgagttcctg tgcgacagag atgaatctgc tagcttctct gattaaagga agcaggggtt
1126561 tgcatggtct gatgcaaaca taacaactaa aaagggctca agagtggagc acagagcgaa
1126621 agtggcactt tttaagaatt ctagatacat ttggacatat tcatttgcat gatataaggc
1126681 atgggcacat caagaggatg aaatgcatca taaatattcc ccaaagccat tcctactcct
1126741 caccatttct ctggtttcac agcaatcaac cacaggccga cataatacac cctcccgctg
1126801 caactttcct cttgatctat gtttcatgcc ttcactgggc tgaacgagag caaacccttg
1126861 cacggaggcg cgggattatt ccggccttgt gaggatgagg agtagttaat taatcacgag
1126921 tgtctcattt cctctttata acactcattt actttatggc tcatttttgc ttattgctct
1126981 tctcttgccg gctattaaga gtttgatgag agtggctgtg attgttgatg gtttaaattt
1127041 tttaatatcc attttttaaa gccttatagg gacatctgaa aaaagggagt ttatgtccca
1127101 aactttgaac tgagtgttgg ccgaatggct agctggaaag aattattaat tcacggaatc
1127161 tctaaccgtg tcctaaataa atctctattt cccgaagtaa ataataaatc aaataatctg
1127221 gagttaatga cacccctgcc ttaaagagct gcaccgctgc tgcagccctg cactgtaaat
1127281 caatggtgga tacgtctgat ccctagatac ggtcattttg tgggattatt acttttccaa
1127341 ctatgctaat cttcaggggg aagagaattt gacatgctag cacaggagtc gattagtctt
1127401 ttgcagtgaa gatcatattt attaataacc agtgagagtg tgcctgcagc tgtgcctcgg
1127461 ctcgcgtccc tgtgatgaat gctttatcgc tcagtgtcac atcgttttct tgagtactcg
1127521 ttattagtat acatagatct ggcgcaggct cgtgctgcct tccgtgcgcc ttcatgcata
1127581 caggaatggc acaaataatc aaatgctcgc aagttaacac aatcactgcg tgtgagtcaa

1127641 tacataagtg tgatttgtcg ctgcgagcat tatgcgactg aaaagaacat aaacctcatc
1127701 aactctgatg ggagaaacag tcggatgtga ctgtttgcct ttgttctaag ggaaaataag
1127761 gattctgttc gcagcagatc ctctctgtct tcatatccgt gcctctcggt cacgcctggg
1127821 cactttaagc agctccccga ggagaaaggt tgcctggaac gacaccgccc acgggcctcc
1127881 ttcagacgca tcctgcaggg cgagggcgga ggggttcgca gcagaagtgg gaggtgacga
1127941 gtcaaaagtt atgaaatatg ccccctgaat aatcaacacc caaatcaatt tgttcagagt
1128001 gcgtgagaag tgggcggact acagaggaac gggcagagtg ggtggtgacc cggtccatat
1128061 ctcagacaaa cacacaaaag cccgaaggcc cgagagaaga gatggctggc agggacggga
1128121 cacaacagag ggacataata ttgtgcttta atcaattatt ctgctttgac tgcatcttcc
1128181 aaccctgctg ttttcacaac atccaagtca tcagaatgta aaaacaggag cacgcgaagc
1128241 gcagcgtcgc taacgaagga tacatgtttt cacactttga gcggggccac agcgcaacgc
1128301 cgttgtctct cggtacatgt tggtatgggg acaggagtgc actgacagcc tgacctgacg
1128361 accatcgagt aggctataaa tacaagaacc tctcccccac aatgccacac aatttattca
1128421 cacttcattt tggattctga gtgcaagttt ccccctaaaa ccatcacatg ggtattaaaa
1128481 cccagccagg cagagcgtcg cttctgcagc aatgagcgcc tttttgcagc agtgtgctcc
1128541 agcatgggaa ctatcctctg agtgggttat gattaagacc gtctgagtgc ttccattctt
1128601 acaagggctc taaagcctgc attaggaaaa aaaatacaaa aatatttctg ggtcccagac
1128661 attttgattc agactcgctc atctttccta tttcttgttg tcttcattgg tcacatcatt
1128721 ctcctttaga gagtgttgcc tgaaagtaca gagacgtgac cagaaggttc ctggtctgaa
1128781 ccccgtagaa gcccctgtat gctccctggg ggctgaggtc gaggaagtgc atctttatgt
1128841 gtgatctcat cttgacgcta ttgtaaaaag gcggcgctct tgtcatttgt ctcctctatt
1128901 ctgctcaaag gcgtaaagca gagtgacggt gggcctatcg cggcgccggc cacaagctgg
1128961 ccgaaccaga ttagcgagca tcctatttct cacattgaca aattaatttc cacccaaatt
1129021 aaatgaaggg accctcccgt gatgattccc ccggaaatta aaaatgtttt tgaagaacga
1129081 aataaaacag caacaactcc ccaagaggag aaaaaaaaat ctaattacaa gtaggatagt
1129141 aaattaggga taaatgaagc tggggagaag gaatcctata tggtcaatga aagtgagtaa
1129201 tgcgtgacgg aggggatgtg tggattcagg ttcagtggag atgctgagaa atgaagagga

1129261 aaaaagaatg aaccatatag cctaacctag ccttgactat taggggatgt gctattttcc
1129321 cccaggcgcc ggtgaggtgg aggtaaaaga caataataga ggggtgaaag agtgcaggac
1129381 acagaggatg gggggaagag atgagaaaat aaacagcagg atggagtgag tggcaggggg
1129441 aggcacgagg ctgtcggggc tggcgccgct cggattcact gacatttaca gagtggccga
1129501 tcgttcctca ccttcctctc cctgaaaagt tcaaaacgcg cacggctcca cagaggagat
1129561 gctgtggcgt gtggattaac aggtcctctc atctctcctc ccttccatct tcagacgcta
1129621 cggctgccac tctggctgtt tgcccatcgg cctggctacc tgcctgtctc ccacctgccc
1129681 gagactcttc cttaaccctg attaaaccta ctttttaggc tgtgttgcaa gtttgaattt
1129741 gctgatctgt cacccggcca gggtatcccg caaaccaggc tgcacacagg aggctgaaac
1129801 actgcattca gcagccaccc agtttggggc ccagcaggga agattgctgc cccccccttа
1129861 aggccatctg ccccccactg cttcatatca cacacagcat atggttgcac gtgggttcgg
1129921 ccagctcgct cgatgggaag agatggcaaa catgcttggt ctgaccttgt catctctctt
1129981 taaattcttt ttattactct ctaaccttta gtggctaatt gagctgctgg acagagagga
1130041 taatggtgag ggcttaaaga gaggccactt aaacaccagc ctcctcgact ccaactctgt
1130101 ctcttagagc agcatctttg taggcaatgg gaggaaaata ataacaatca aaacatgcac
1130161 caaggtcaaa agcaagggca tttattttca attacctctt caaaagtgcc tccagcttca
1130221 tccccataca gaaacctgtt gcagcctgtt gtccacaagc cgtgagggtc attaaagcaa
1130281 cctggtgcat taaaacagca aaagcagctg tgctggttga taatagtgct ttcccccaac
1130341 gctgctcccc tcacggttaa tttatttaga aagttaccca ttaaaatgga ggcagagggg
1130401 ggcacacaca gcaggggact cctttagcct ggcaactctg ataaaagacc tgacgacttc
1130461 actcgtaacg cggcagaaat actggcgact gaaaggccca ggctggtgtg aacgtgatta
1130521 tgacatcaag tgttgaaaca gatcaacggt agcctcccct cgtcatcgga gatctggcag
1130581 cggctacccc gaactggctc gaccttgctg aaccccgctg ctccctctcc tccagaggtt
1130641 agtctcacat cagtgcgcgg tgtctgaagc agcgcagcac agtcttaaat cttatttctc
1130701 catctcaatc cttcctgtct tcccttttt atcttcatct ctcttgcggc ttgctgtatt
1130761 aacccgagcg tgatctttgg cagctctgac ccgagctgcc gcatctttct ttcttcatcc
1130821 tgagcagctt taggtaatag cagacattac gtccaggtgg gatttttctt cctctttgtg

1130881 tgtatgtgag cgtgtccctc caccaatact tatgggacat gattaacggg accaatagct
1130941 gtcttagaca caggtaaact aaacagagaa gcacatttgc cttgattgag ttcgattaca
1131001 ggatagctgt ggcataaaca atgctgatat tatctaacac aatactttat tatagtttaa
1131061 aatagcttat ttcagccctc tgagggtggg agatcaacat caatcagaaa ataaatgtaa
1131121 taaacccact tcagctgcct ggcataactt ttattcatgt ctttcagggt caatttctta
1131181 accctaaccc taacttggtt tctcatacgt tagaccagaa tttacgatgt acagcagtat
1131241 aatgcggacc agacatgcat acataatatc tataatatct aataagtcca atacttaaat
1131301 gcatctttcg atggaaatac atgtttctgt caactttttc ctgtaataat tgcccatatt
1131361 tcagctgatt ttaatgttgc ttccaaattt aaccaccctc aattggattt ctttcttttt
1131421 aattacattt agaataacac acactgaaaa acacatctga accgcatgca cacacaggct
1131481 cacacagcct attccccaat tttcacaatt aagccacatt caatatgttt cgtgatgctt
1131541 ttgtcctgaa taacaccctt cccaggtatg taagctctct attaaagctg aggccatctg
1131601 aggagaaaac gtgctgcttc tcgtaagcag aatagatttc ttccccatct ttcacaggag
1131661 gctgtttgct ggagtcgacg ctctcctcca ttttcattag cagcttgacc accatacatt
1131721 ctccatataa ttgaggttga agccttctgc actgtaatgg ggctttctat aacagatgaa
1131781 agcctgtcat ctttgggaac cgcgtgcatg tgtgtgtgtg tgtgtgtgtg tgtgtgtgtg
1131841 tgtgtgtgtg tgtttgaaac caatcacttc atctgtgacc acctttcaaa agtgactttc
1131901 aagaagtcca tctttttttc ttaaactgat gtctaataaa atcaaagcca gacagatgaa
1131961 acattgaaca tctttcttgt aggaagctgc agagacgagg tccatctgag gctctgctgc
1132021 tctaattaat gtgattaaac agactgaaac agtttttttg ttggcttatt tacgagttaa
1132081 aactcctctg gttgttgtgt gagattatga cttgaatggt gccacctata tcagatccat
1132141 tcatttcccc ctctaatctt ctgtgttcct tacatggaca ggcagatttc attcaggtgg
1132201 attttgtctt taatatgcag actttaaaag caacacaggc cctgcgtttc cctgtcatca
1132261 tcattcacct cagttcctca gtcaaacgca acatcagaaa agcctacatc tgagggagac
1132321 tttagctgat gaaaggtagc tatcgggatt cagacttcaa gtaaagtcaa gatggccagc
1132381 gtttaaaggg agggaaatca cttaaaacag attaggaact gttaccgtat taaagaagca
1132441 acatttaggg cttattttta atcttactgg cagggcgctg ctatgagtct gccctgtttt

1132501 gtgctgtaga gaaggaaaaa taaagagaaa tgaaagcgca atattaagat ttgggtaata
1132561 tttgcattgt tgatggaatc tgccttgtag atagagtcaa acaaccgcct ggaagccgcc
1132621 atggttgctc ggggtttaac taatctcaaa ccatcaaacc tgcaaacact gttcgttcag
1132681 ctccctaaaa ttactgagag tcacggaggt gcttaaattg gaagctgcag ctgagtcaaa
1132741 gaagggctgt aaataaaaaa gaacaacggc actcatccag agtgtgtgtg tgtgtgtgtg
1132801 tgtgtgtgtg tgtgtgtgtg tggagggggg gtgttggaca gatggtgaca cacttgtgat
1132861 ttttttagct gtaaattttc aacggtggtt aatgcgaatg aaaatctgaa cattaaaagt
1132921 gctgaatatg ttcaagaggc tgctctacac aggcctcgga gcaggaggag tagcttcaca
1132981 tgttgtcttt aaacagtctg tcgtcagaga tattgaaaag acaatgaggc taaacctata
1133041 aaaatttaaa gaagcctaaa aagaacttcc tgaaccttcc attttctttc tgagcagcat
1133101 ggatgaatgt caaacgatca atgtcacaaa tctttggccc accaaattct ttaatatttt
1133161 ccctccttgg ttctgggcac ctgaccttcc agctctgagc aaaaactgca ccgtcattcc
1133221 cgcggaggaa atgtcagctg tgcacataat ctgctgaatc agagcaggtt cccccagctc
1133281 gtcacgctgc gtctgtttgc ttaaattaac gtgcatctgt gactccatct tgctccggcc
1133341 tgtatgcatc cataatgtat gacgcctgtg ttattgtgag ctacatgtgg ccgggtttgc
1133401 acgctcttcc gttgtgctgt gataatgcat cagtgcctga catcagcgcg ttagccgcgg
1133461 catgacgcga ccctccgggg gcccatcagc gtgagtgttc cagagaataa taaataggag
1133521 gagattggag ctgagaggat gagaccgccg atcgattcct ttgctgtgtc caaattagga
1133581 gggacggttg agtgtgtgat gggtggacgg agagagagca cagacagaag gacagcgggt
1133641 aggccaggta ggtggaagca gcgcttcagt aggggaaggg gggggggggg gttagtgaaa
1133701 cagccaggct aattaagctc catcccagtg gaggaaatgg tcctgctcct gtccttgcaa
1133761 ggacacgctg ctcatcacat gtcgcacaca ttccagaaag accacagaca cttaaacaat
1133821 ttgattagaa tgcaaaatcg cacaagtggc atcgcggttt cgcctgagaa acaggcgttc
1133881 acacacatct gccatcacgt ctcctgaggg gctcagcctt tagaggaggg tggactaatc
1133941 agtgccaaag actgctggcg ccagccgcta caggtcccag ggtgcaggcg tgtgccacgg
1134001 gccagcccac tcatctctgt tctgcacacc cactggcagc acacacacaa acacacactt
1134061 gaggcgctcg tgttatgtct ccattcacaa caaacacata caaatgagaa cacgagcaca

1134121 caccgataca ataagagacg tgtaaacgtt ccagaatcac acacgcagcc cttggctcca
1134181 cggacgtctg cctagaaaag cttggctaat gaacaaggcc tctggctctg gtggctttag
1134241 acaatggtgg cgtggagtta ctcagtgcct caccacggct ctgcagcggg attagaggga
1134301 tgtttcatat tgacacagag aacaaatcga ggtacgctgg taattccatt tcacactcac
1134361 cagcagtgtg aggggagatg ggaaacagga gggagtgatg gaggttgcag cccacgggtg
1134421 ccctgtgagt gcttttgacc tccccggggg gactgatcct ctcacagaca gcacgttaac
1134481 gcaaacgtcc gtatgcaagc cccagtggaa atacacagac taaacacaca tgctgaaagg
1134541 aagcgtgcat atttctgagg acagtggcac tgaggccagt ttcatggaac aaatgagaga
1134601 actgaaaggg cagaaaacaa gagtctgatg gtgggtgaaa aggagggtga acagggagct
1134661 gggctggtct gcaacatatg ataaaagagg agagacctgt tacctttgtt gtagaaatat
1134721 gtctgagatt taatctaagc agtgatgcaa acaacacagt gctgtgtgtg tgtgtgtgtg
1134781 tgtgtgtgtg tgtgcctgtg tgtgtgtggg cagctgaagg attggttgcc agggagattc
1134841 tgacatattg ggccccactc cctgctgccc tcagtttagc cccgacaagt cacagaaaat
1134901 gttcagcagc aggctaattc acaatctctc cctctctttt atacacacac acacttgcgt
1134961 gcagccactg cttaggtgat catcgggtcc atcgttgatg gtgtagcgtc cctctgttgt
1135021 gatgggttgg gtgtgtgagc cccttgtgga ataaagtgtg ccacagatat tccacagata
1135081 ctccaggagt ctctttcagg tggtctttgt agtgcagctc tggtccacca ggccttctac
1135141 tggctgatgg cagttgtcca taatgcacct gatggggcaa caggtggatg tggccgaccc
1135201 tttgaagttg cctgtgcacc agggtagctt tcattccaat agagtcagca tgttggagga
1135261 cgtcgctgtg gggaatgtga ttctaccagc tcctaccagg ggatggcgag gatgataaag
1135321 gatgatggcg aggatgataa accaggcctc tgctctacaa cagcgtgctc acacagactg
1135381 gcagaacgcg acttcggttt ttgttctcag gtggctcttt ttgcgagagt gcagcctaaa
1135441 ggtagagacg aggttgacgc gggcccgaat gtcttcatgg aagtgtgtgt cagtataccg
1135501 ccaaggtaac agaactgtct gacagcttta ggtggtgcaa cattgacact gaccacggac
1135561 gggacagtgg gtggacacct aatattcatc accagctcag atttggggtg gtgaaaacta
1135621 gctttaatga ccggtaaaca acaaccaggc tatgtagcat tgcacctgtg ctgtgtcggg
1135681 agtatacgat catctgcaca ttgccgctca cggatgttac tgacttgtag accattagtg

1135741 tacacacctt gtgtacacca ctgtcttcct tgatgactgg aaaagtagtg tggctgccat
1135801 caggaaaagg atgaagatca caggggcgag tacaacgccc tgctttacac taacactcac
1135861 tgggagagag ggagattgct ggccagcaac gagtacacag gcctgcacgc cactgtgaag
1135921 gatgctgagc aatttctttc gtatgaccca gaggatttcc caaaactcta ccaaaggcct
1135981 tgctcgggtc agtgaaggtg atgcagaggt ccttgtgttg ctccctacac ttgtcgcaca
1136041 gtgaaaatca agtccagagt gctacacaag tccggagtca cactgtgtct ctgcgaggat
1136101 gtcctctgtg aagtgggtta tgaggtgaaa cagcatgagc cctgcaagct ctgagcccgt
1136161 catagagagg agacacgcac acaaactcct tttgctctgt tgtctttcat tttaagctag
1136221 tatgctccta cctgtaagta ggttaacggc accgcagaac cgcatgttcc ctcttattcc
1136281 tccctttcaa gagtgctttg ttcatccact cagtcatgct agtcagtctc tctcactgac
1136341 aggcatcttc ctaccctcct ctcctcctcc agctgctcct gggacctgtt gttctcatct
1136401 actgtcagca aaacttggcc tgttgtaatt agtgtagaat tttaataagc ttatcaggtc
1136461 aaaagcagct tagtccttga ggaccctcgc agggttgaaa ttagtctcac acccacattt
1136521 aaaatgtcac atttcacatg gcaaagaaac aagcatgatt cattagcaaa cacttagtga
1136581 caaattcagc cccagaatcc cccccataaa gagattaacc cactttaaat caaccagtta
1136641 acaaaatagt aaattatttc tgcgaggggc aactcgaaga agaacaaaag gcaacagccg
1136701 tcatatgtca ggctgccatt gtgctaaata aaccaatagc tacggggagg atgtcaactg
1136761 gtgagggaga cagaggggga gagagtgata aacaagcagc agctaatgca tgaagacagc
1136821 gaaaggaaga ataagcagaa aggaccagac agaagctgaa agaaaataaa aaataagcca
1136881 taaaataagc aagaacagca atcttgctag acgagcatcc tcgtcgctaa agcccgggcc
1136941 atagaccttg ggacttaatg aagcctttct ttcctgatat cgcagtctgc atcctcaaca
1137001 gcacgagctg tcctccgcag atcatcctcc gcctctcttt cagtgctccc tgctgcactg
1137061 aaagcgaatc cagctcacag tgcgcgctgt aaaccagctc tgctccagac agcaccgtaa
1137121 atctgtcctc gtttccactt aatcgtaaaa cgtccgagca ccagagagtt ggaaagggtg
1137181 agagaacagg gagctaaatg ttagccacag gggtgaaggg agtaaaagtg cttcctgact
1137241 aatgaaataa acaatggatt catgaagctc aggctgtgat gtcatgatca tgagcacaaa
1137301 agagaaaacg cactggcgtc atgaggggaa gcaaatgtgt tattgtcact ccaagaaaga

1137361 ttagatgtac tctaatcttt aaatgttata ttcatgaagc tacgaaatag ttcttgagtg
1137421 acaataacac aatttagaca gtcatcactt ttgaaaagta aaaataagac gcgcaggagg
1137481 tcaaaatgga caactggatg cctatttttt ctccaattgt gacggcaaaa cgtcccggtc
1137541 agagagtaat gcggtctggg caaatttaga acgtaatttt ggaagattcc gaagtgattg
1137601 agacaagtat tgccttgttg ctgcagctcc caggggagga cgggcttctg cagaacctca
1137661 cacgctgcat caggacaaaa aaaactccac tgataccaat attgtatgac ttgaacggtt
1137721 gtcgcgtctc atttttctct gtttcatcag caaatggggc ctgatgaatt gcatttcatg
1137781 tgagatgaca gcactctccc ctgaatatat ctgatctggt ttgttttgtc cctcacactt
1137841 atccacccag tgaaagtggg actttgtggc ggctgattta tgtcagcccg gccgcgctcc
1137901 gcgctcattt gtagcgtatc tgtttgttaa agcaaagatt tccttactta aactgtcttt
1137961 ttacctcatc agctacaagc tggttgaaga aaaataactt atccgaataa aaatgtcaaa
1138021 ctgcaggtta ttgtgtattg aagcacagtt aaacactgac tcgtatgaca ggataataca
1138081 ggagagatgg tggacacgtg gctgttagtg cagttaattt gatgagctag cctgcagtga
1138141 gtgcatgtgt gtgtgcgtgt gcgtgtgtgt gtgtgtgtgt ggtaataagc tccatttcct
1138201 ctacagcctg acagaatccc atctcatgct gtgacgcctg tctgtcattt cacagcctcg
1138261 tgtgagatat gctccagatc aatatttaat gtcgctaatg ttggagagag agaggagaaa
1138321 ggagacaaag tgggagaaag tgtgtgtgtg tgtgtgtgtg tgtgtgacac agacaccagg
1138381 acgtagcaga cgatagacaa aggcgaaaga gaaaaactac agcgatattc ttgggcggga
1138441 tgtgaggccc cgaaagcgtg agagtctgtg gggaggagcg aaatgtcacg gccaatctct
1138501 aatgaagggc cttgcgggaa ctatagttag ataaattatt taaaggcttt catttaaaaa
1138561 tgtccaaaaa actgtgctgc gatgatttgc gcaggggctg gggaaaggtc agccttgtgt
1138621 tgggagtgct tcattaacct cccaccacct ccctacctca attatttctg tcaaagagca
1138681 cctgggggga gggaagggag ataggggggg ggagtgtggc atggctgcgg tccagctgat
1138741 accatgctgc tgctaccgct gctgccgaga gctgaggggc tcccactaca tccgccttat
1138801 ctcacaagct ggtggcagcc tatttcaggt aggatgtttg ggtgacttca gtgttgtaaa
1138861 atccctggag atatgcagga aagaggctgg gcgaactggt ctctgtgtgt gtgtgtgtgt
1138921 gtgtgtgtgt gtgtgtgtgt gggagagaga gagagagaga gagagggtga gtgagtggct

1138981 cggctcgtct ctggtcttca gtagtgttta gcactttgat cttgcaaaat gttttctaca
1139041 accgtggaac tcacagagct gcgaggaagc gtgcacacgt gcgtgcacga acaaacccat
1139101 acacacttct gtctcccggg caggaaaagc agcttggaac ttttcatccc tgttttccca
1139161 cctcgtctct gcctcctctc tgctgtcaac aggagccact gtagcttttg acgtgagaac
1139221 aaaaaaagca caattaattc aaactccctt atatctgcat aaatatccct cacatgtctc
1139281 ggatttgttg tgttcggtta tttaacgctt atattttgtt ctcacacagt ggtaacgaaa
1139341 tgttcaatat tttgacaaga acaaatctcc cagtcattaa aatgcagcca gtgattaaac
1139401 cttgagaaag cacttgtgtc tctaatagca tttattttc tctctcactg aaaggagaaa
1139461 ccgccaaact tggcatgtat tcaggtgaat gggactcaga atgagataaa ctaaaggtat
1139521 tttgtcgcag cctttcctgt ctgtgcaggt tatgttctgt gaaatgtgga tggcacgcgt
1139581 gttcagtgtt gtgacgcaac gtgcactgga gccattttga caactttaca agagtaagat
1139641 gtgaactatg cttttgccca tcatcgttca tatttaatcc atttatgaga cctttgagtg
1139701 ctgttgtaca tgactgagat catgaaaaaa tacagaaaat accagagata agaaaggaag
1139761 agaaatatct ttcgtcatat tttctaaaat cccaaatttc tgcagcttcc aagcgagcag
1139821 caggtctgag ctgcagatca gagagatttt aaatcaaacc ttatttgact ggcaagtgtc
1139881 ttccacaaaa cagccataat agatgctgct acatcaaaga gaagactaat caggctgaag
1139941 gaccgaggtg tcacagagca tcagccctca ggggccgctg cctgtctccc tgggagacgc
1140001 ttcatcacat ttgacctcaa cacactcgcg cactgaatgc ctttagcatg tgcaaaaaca
1140061 tgctaggctc atggcggcta acccacgcta cacgtcgcag agggaatgcg tgtgagaggt
1140121 gagtcggttc attgacaggc ctgtcgatga aggctgggaa aaagcagtaa aaaaaaaaga
1140181 aacctcatct catcttgatg tatttgggtc ggtttcacat ccccggtcac cgctcgttca
1140241 gacggtgtgg ctaaacaatg aaatcacaca gtacatttaa cttaatgtaa atgcacatca
1140301 ctccccatga aagcagctgc atcgttctgc atgaacaaag agcatataca acaaccagga
1140361 caaaaacggc ctggtactta acataaaact caataaatcg gttccagcaa acaacacgtt
1140421 gtatccattt atggctgcct ggttttgccc gagagctcac tgtatgatca agatgctctt
1140481 atgataaaga gagaaaatgc agagtgccta gctctcctcc cataagaggg tgaacgctta
1140541 tttgcattga aatgaactgt cagagacctc cacgcgcgtg tatgtgtgca atgccgcgtc

1140601 gacctgcacg gatcaccggt gatgaggtga ctcctcaatc tcagcaacaa ccctccagcc
1140661 tcgtgggaac ctctgaacct ggctgtactt atgcatgcta tatgttttct gtccgctccc
1140721 tatggtgctg cggcgctatc gtgttcccat ctatggttta tggtgctccc aggaatagca
1140781 tgtatggagg caatatgtga gaaaattcca atttgactaa atcgatcaaa acacggacgt
1140841 attctgatca gggcacacgt ttcgcattag tgttagccac aaattagctg tcagaaaatg
1140901 ctgaaaatca atagctctac tgtatataat gtatggggcg tattgtctga gcaagtgaga
1140961 catgattgat aggccccgtc agcggtgtgc tgagtgcacc ttgatgctca gactaaaacg
1141021 ccattttatt accactccag tattggagct gatcgtctcc ttgatagctg gtttcagtgg
1141081 tgagggctgt gtttccaaaa cactaaataa aaggaaagtc tgtctattta actacatttt
1141141 cagtcagtag ccacaaagaa tgctctttcc ggtgtgtatt actgcactac tgcattgttt
1141201 catttacccc aatgatacat tattgaccat tattgagcat atatgtggag aatacccaac
1141261 tctcctacat ctcatttcaa agaacagtgc tttatttgag ataggtgagc taagctgaga
1141321 ttccatatgc agggcattac ctatagatca ggagacatct ctactgagtg gcttttatag
1141381 gtaatcaata ggttggctgg gtcacggtga ctcttactca ttataactct ttaataaaag
1141441 ccaatcctgt gctgattaca gctcagtctg gaacaaacac gtgaccattt atggatctgt
1141501 ttgcacagga aaaaaaatct gacaagcttt aggtgggaac agccagctta atcctcatca
1141561 tttaaggtta ttagacattt atgatcagga atgtaaacca ttatctcatc aaaagggact
1141621 taaaatagga atcaatacca gcatcagagt gctgcagtgg aaatatcagg aagctctcga
1141681 gtgaaggtga cctggtctta aacgttccca acgatgttcc gaggcattta tcaacggctc
1141741 aaacccgacg ctcaggctga gagcttgagg agacacactc gagatggtgc tgtcaccagt
1141801 aaccttcact gacccgtagt tcccttccct gtctcggctc aggccgtagt ggtagttggt
1141861 tccatctctc caaacaccag gttaggtggt gaaagctgcc ctcatgaaag aatttccagg
1141921 aaatggcaac aacatggggt cagtgtgtca catgtgcggg ccgagaggcc acagatgtta
1141981 tgcagcaggg gtcgggctcc atgtgagccg ctcgccagct cgccttcata tgtcaggggg
1142041 atttggagac ctaaacacag acaggggcac aggaaaacac aggccagtgc actgggaggc
1142101 ccccccgcct gacacgactg agcggacagc taattgcaca gcggccctgc acaaaatgga
1142161 cctcacaaca gtgggaaaag tcagaggaag aaagtttcta tcgaacgttg tcacggtgac

1142221 aaaatctgct gttttgcagt cggatgtgtg tgtggatgtg gaacacatcc agccgtatac
1142281 acacactccc cgttatcctc cacatcatcc tgaatgacaa gcatatcgac caccgacagg
1142341 acgctgatac acaaaacact cagacgcaga gtgtgtcata tttcagccct ttgtgcttcc
1142401 cttgctggga gcaaatatgt gatgagagac cccgctggtc ctgcaggggc cagagggtct
1142461 gcccctgtga cccgtccaga gaggacaggg tctggacaac gtcaataagc cataagacac
1142521 aggggaaaga aggaggatcc gattagattt cttgttgcac ctcagcagag tgggttttgg
1142581 tattttatta acattttaaa gcttgagctt gggatcgtgt ttcagcttcc acagcagcag
1142641 gtcggcagtg aagctctcgt ccctgtgatg atctgagccc tctgacaagg cgttttagag
1142701 cccccccca accccccca tctatctcct gctcgtttct tatgtgtaaa gacagctcag
1142761 acgtgcggag catgaggtag acacctcagt gactgtgaca gtgactgaaa cccacgaccc
1142821 tgctcctcca gccactgtgg ctcaatattt gatgggtgga ggtggtgctg gggagacagg
1142881 aaatcctggg tgaaactcct gcaggagtgt ataaaatgtc ccaggtgggc ggggaagagg
1142941 aggtgggtca ggacagaaca aggattggta cagtgaatta ttcatgctgc tgaggttaac
1143001 agtagaacag cattatgggt ggcgcagagg agaacaaagg gaggagggca aatgggccac
1143061 gctgaatgaa aagtgaaagt acgcaggcca agccaagcgg agatgaaaga gtgctttata
1143121 ttcgtataga ttgtacagtt ctttactgtc tattaacccc aaccaaggtc agatgggtgt
1143181 cacctggcaa gaggcaggaa tcttcctgga atattagtgg ctcgtcacag gtgacggtcc
1143241 tttctgttgg tttgaaaggc cacggagaag gtgtgggttt gtgtactggt gacttgcttt
1143301 ggacagaacc tgagagcaca taagaactca gcgtacgcag tgacggtgtg aagaaataca
1143361 tttcaccttg atctcttact ttattctctt ttgtggtttt tcttttgata gttctctctc
1143421 acagaagatt tttatctgca gatgacataa aggggacacg atgtgttcca gcacggttca
1143481 gaactgcgga tgtctatctg ggctgttctg gatattcccc tgaaaacctc tgacagacca
1143541 tctggcaaaa cacacgagcc tttatcctcc gtgaccagag ttttgcccca caactgggtg
1143601 gtccaacatc tgcctggctg ggttgtttac atatttgtca catacaggat gctgacacac
1143661 agaacaaaat gatttaaatc taaaataatc attaccctga ctggaagaga accaattaca
1143721 gtatttttct agcaattgaa aatatttgca tcatttggac tggcagaatt atataaaacc
1143781 tcctaattaa acctcaagta ctgatttatt taattcaaag gcagcgacac tttgtctgta

1143841 cccatctcct tcatcaaaca catttggtgt ttaactctag atttaacata ttgcgttgca
1143901 tatggcgaca tgagccgctg agccagatgg gaacattaag agatagaaga aactgtcaac
1143961 tggacgtgag acaagacttg gctggaacta catttggggg atttgcctga atacagtgga
1144021 atgttggggg ggggggtctt gagaaatggt gtgcattaaa atgctagaaa gggatccaat
1144081 gacagccatt agagagtgtg aggggctgcg actgctgcgc gctgacatgc ctgggtgaga
1144141 acgtaaggga tggggttacc tctgtctgaa agaggacaat aatgggagga gcttcagcgg
1144201 cagacagacg ggagacaggt gtgagggctt tacaaccaac tggcatccca atcagcccga
1144261 ttagtcaaaa tgtgtctggt ttacaagctt tcttagtcac cggacagaag cttttgattt
1144321 tgccgatgca gcggtgggta cattagcttc aactccactg aacaaacaag ggaaagatca
1144381 aaaatggcaa ttatttgtgt tgtttattat gtccttactg ggagctcagc ttgaggagac
1144441 agtgggaatt ctacttgtca tttgttctgt gtgtttagtc actttcgtgc agcaggccag
1144501 ctgctgaagg tgtggctgtt tatggaagct gacaggcgac ctggcgcagg gtgtgaatgc
1144561 tgatccaaag ctggctggac aaacacgccg gcgttcatcg tgggctgccc gtggagatgt
1144621 caaacacaat gatgtagtga gcggctgcag gagggttttt gcaggcgggg acgcgcacac
1144681 agacgagcat gcctttgctt ttttcccccc tgtttttgat cagaatcgag gaacacgcca
1144741 ccggtggtac caaacaaagg aacagcgaag gatcaccttt agcaatcaaa gctatggctt
1144801 agagctcata aacgtgcctg gagtttggaa aactgtctca gactgttttg aattcattaa
1144861 ttacagcgac gctaatctca tggagacagg gagggaggaa gagtaatctc ataactcatc
1144921 caattaaata attgtatgtt acatggatta ggacagagca gccccgctga tacagaactt
1144981 ctgattctat agcatgatga aaggcatttt cctacaccga ttagtgtgct aaacctgatc
1145041 atcctcatct aggctaacct atccatttaa aactgtaacg tctgctggga aaaccaaaaa
1145101 tgggtaggtg aagccaggca atctgaaaca aagcctaaat actgtgggtt cgtgacagct
1145161 ataaactgtt tcagtccacc tgctgcagtc cacaatttca cagcattcat ctttttttctt
1145221 aaaatatgaa acgcagagag acgtacagag acacgttcac accagcggac gtgacattgg
1145281 aaatgacgct gacgcaataa taggcaataa tgcaccaaag ctacaatgtt gacttgactt
1145341 tgagcaattc gagttctgcc gtgtctgcac tgcaacaatc aaagtggcgt tttcataagc
1145401 tgcggtcttt aagtgaagat gccaaattct atttcatttt cagcaacctg ccaaactcag

1145461 gctctgagcc atttacagac tggcaccttt cattcccatt agtgcttcac gttacaaaag
1145521 acactcgatt atttaagaaa ctaaccaaaa atcgtgatgc gtcacctgat gaacccaaag
1145581 ggaagaaaaa caatttgtta aagcaaaatc agaagtatta gaagctcgat ccgggcaaaa
1145641 gtggtgcaaa tgttatgttt agcattctgc ttgtctgtgt tcagagccgc cgagattatg
1145701 catgaatcta accgcttatc agctgacctt taaattggag ccgcagagtg gctgcaggcc
1145761 cgagggccga cactcaaacc tgattggctg ctctgcctca gacctgctga caatagaaat
1145821 taacacatcc aggtgggcat aactgcgggc gacagcagta agaaatggag ggagatggag
1145881 actgcctaaa tatatttcca ctatttccca ctacttctct taagcttcag gagagtaaga
1145941 aaaagtcatt atgtgctgtg aagcatgcta acaggagagc aggtaagaac agacaagcac
1146001 ggtattacct ggattttatc tctcacttag tcatctctcc ttctatcgct cacctttttc
1146061 tctctgttcc ttccattcat tccctgtttt attggtagag aggtcttggc aggctgtgat
1146121 tgcctgatag atagatatct cctgtaggaa aggggacatg caagaggaga tgggctgagg
1146181 atgagctgtg cattctgtcg tccatgggga caagagctct gtgtgtctgt ggaggatgat
1146241 ggattacacc cagggccaag ggtgtgtgcg tgtgggtgtg tgtgtttgtg tgggaaaggt
1146301 cccctcgtgc cgtgtatgta tgcatacatg gagtgaatta atttttcaca agagcagcta
1146361 gaaaacataa ttaaataaaa caaatgtttg agaagcggaa tcaacttgga caccatatag
1146421 gcaacagcgc agctctctgg cagtaaacaa aagcatcatg ttgtgctgta gaaactagca
1146481 caggtggtgg actacaacgg catacgtttg gccttctgtc acataaaatg agttccagga
1146541 gaacaagctt tggaactgat tcgcctgcgc gtgtctgcgt gtgtccacgg cggccgtttg
1146601 atatttcagt gtttgaacgt catgatgtac atccacagtt gatgaagtgt ccttggagcc
1146661 ctccctgtta caccctgtcc ctggcagagg agtcatgggg tcaacccact cttcctgttg
1146721 gatttgtgtg tgtgtgtgtg tgcgtgtgtg tgtgtgtgtg tgtgtgtgta gagtgatgca
1146781 ctgacaaagc cggacaaaga aacacagcag caaatttgcc caactcaaaa agctcaccgg
1146841 ctcaccagga atgatagata gcgaggcact atcacaactt atacttctct ccctctctct
1146901 ctgcataggt gtgtgtgtgt gtgtgtgtgt gtgtgtacat ttctgtgggc tcaacaatgt
1146961 atgtggggga gaacttatca ttgctgagat gaaggcctca ttcttacagg aagataaata
1147021 gaccaaatga accagatgac cacagtgttc atgtttctgt gtgtgtgtgt gtgtgtgtgt

1147081 gtgtgcgtgt gcgtgtgtgt gtgtgtgtgt gtgtgaaaga gaaagagaaa gagaaagaga
1147141 gagtctgtca gtgatgaata ggggtttaaa gacctatatc atacagataa gcaaaaaaaa
1147201 atcaatgcta caattcaagc tgagtaaaat ctccagaaga atataaaaac catataaact
1147261 gcagtcaaga gttgcctaat tcactaagaa aaatagaagc tacagtgtcc acgcagatta
1147321 cctgccatct cacaaaaccg tgcagggata gctgctgtct tcctcgccaa aaatctcctt
1147381 tcttcccatc gagcaggatt ccctctggag ctggaaagag agatttgcta caggagtgcg
1147441 tctctccttc acggggaatg ttgctgcatt tcattaggga tgagattcat cacggcgagc
1147501 aaacaagaga gcgctgcaaa tcacgctgga ggacacgcgt acacggctcg tgcacaagga
1147561 gccacaaagc aaacaaagta aagagagctg attcaggaga caaaggggta cgtcaagcac
1147621 gttgatgcta ggtgattgct cgttattgtg gacttcaaaa tgacgcatgg ctgtgtcagt
1147681 ggtgggaaca gaggctgaca aaagggttca atcttcagaa gcacgggttc cagcatggca
1147741 tgagtcattt ggtccaggat ctgaaaccct gtctgagtaa aacagacacc ccagggtcca
1147801 gtttagagat gacaagctct gaccctgaaa aaaggctgag atagggccca tttcaacggg
1147861 tcacggccct gtctcactgg gacccacgct gtggaagtga gtgaacgcag cctggaggat
1147921 ccgtcccgtg atcggagtgt gatatcttag caatactcaa gaacggcagc accaactggg
1147981 aaagtagagg acagactcag ggcgtggagc actgatcata cggaatccac ggggaggggg
1148041 gcgggcgtca tgtggccaag gctggaacaa accaacaatg gtcaatatag agaggaagag
1148101 gcacaaactg gaggctgatt tcacactgaa gtcaacagct gcaaacggga cacaactgat
1148161 ccattcacac caaaggcaca caagtctcta cacacacgca cagactggac agtggtccgg
1148221 ttgtggctgg ggagataaga ggaatgccac gtaagcgcaa tgggttacag cgtgggagag
1148281 ccagagggag atgttaccat tcaatgcaag acagtcccct gtgtcttcca gcgtatgaat
1148341 acaatgttct gtctgctcca ggcggtgctc tggctctgct tgagtgagag ctgaaccagc
1148401 gtgggccgga gctggatgtg tatataagag atattagata atggaatggc ttcaaccaaa
1148461 gctttgatat tgattgaaac aagcaacata aaacagatgg gggacaaagg aacacagaga
1148521 gcgagaggga aaatattgtg aaacacacag acagaacaga gacttataac ttacattggt
1148581 ttttttgtac cagtatgaaa aatatttgtt ctgtctgaag ttctacccac tgcattgaca
1148641 gagagaatga gcgataatga ccagccgact tcagagttta cgatataaac cgttaaatgc

1148701 aagcaaacat tagaaaggca atggctcctc ttggttgggt gcggagtgga cacctctcta
1148761 gctcacactg gtctccccgg gacaggtcaa tagcggggac gtctgccagg tgcgaggttg
1148821 gctcaggtat aatcaatgcc cgccagagga cgcagaagac ggcaggtagg cagacgagcc
1148881 ggcacggtcg cccccaggca gagataaaac cgctctggga ggctgaggct gcgcgctggt
1148941 ggaaactcgc acctcaaaat ggtcttgggg attgatttga cagctaattt agggaggaga
1149001 tttgcataca gccacgcgct aataaggcgg gcttttcttt tccgtccttt gtctttttcc
1149061 tccacctctc tcggcgttct cctcctttgt tagacctcag caaacagtaa aagataatgt
1149121 aagcgtatta cagagacaac agattcaaat tggatattga ttaagagact caagccttgt
1149181 gtttcctccc gttaaagcct gtgtttaaat gattgatttg cagcgtcact ctctgcatcc
1149241 tcctggccgc aggctgagct gtgtacccaa tgccttcgat gtgctcaaca tgtcacgccg
1149301 cgctaagtgc tttcacacga gcacacaaag agacctgcct atctgatgcc aatctccaca
1149361 ggtcctggtg tcacccaggt ggaggcgttc aaagagatca aggacagagt ctcatcagca
1149421 gccctgcacg gtgcagccgg actgacaggg acgggaaaaa atagagctcc aatgtcaaat
1149481 atagtgaaag gggggggggg ggaaggcgca gcgacaggtg taccgcgctg gatgccagca
1149541 tacctcacaa gacctttcac cggtttagaa atgctggcag ggtagcagag agatttctaa
1149601 aatgagcaaa cccacaagga aaatgggggc cgcttgacac cctctgtctg actcactgtt
1149661 catgttcttg aagatgttga aaatggggag gatctgcctg tagtacggca ccagcgcctc
1149721 tcccaccatg tcggcagacg tcaccaggtg ctgcaggacc ttcagggtgg agcagatcac
1149781 ctgacggttt ctggtgttca gggcgtctgg aaggtaaagg tcagggaagg tcatcgaaag
1149841 ctgtaaagtg agcgtttgat aaataaatga acacaactgt caaagatgaa tgagcaacac
1149901 gagcaaacgt gagtgaatcc atggagttat taattcagtg atttccaaaa caatcttctt
1149961 tattctttat tggatcaacc accagaattg tgtggtttca tattgatgat gcagtcagtg
1150021 aaggcgccac actttccgac tgggtgatgg catcaaaatg agcacgccga tgatgagaaa
1150081 aagagcagcg agggaggatt atcatcccca cagcactcag agcagcagac tgcagcgcca
1150141 tgtgtgaaca acagctcata cgcaaatcct gagctgctgg gtcacacaac agtcgcacac
1150201 gtgagcgctg cgacctaatg tcagcgcggt tacgggccct ggcggcagtc accgctgcaa
1150261 cagaggaaca cgtaggaagc cgcacaatgt ggaagggtca taaaaacaag aggctaatgt

1150321 tttttacacc ctcgtcggag tgcatttgtg caaccgaatg catggtgtgc gtgggacgcc
1150381 tccactttgg tgacctttcg agagccagtt tgctgggaaa tgcctctgtg tgcgtgtgtg
1150441 tgagggagcg agagtgggtg agagacagcc ccggtggtga tgatgctcaa tcaggaaatg
1150501 tattgtcctt tattgccatc tctccgtgtc atccgtcact gcttccccag acatgcgctg
1150561 agatatgtgc tgccgcccag agaagaattt caaacctaca taacaaacaa tgaaattcta
1150621 attaccagca gagcaaacac ttttttccct gctgtgacat ttggattgct ggactgttca
1150681 attattcatt gctagtcttg ccttactaaa aaaaaaaaaa aatggggaaa aaaaattaga
1150741 acaaggagag agaagtggag ggtctgatgt attaacgctg gtatgaaacc tgtgttggga
1150801 caaatctgaa taagggaata atccgtgcag acctgtttca tattcagcaa gaaagtgaaa
1150861 caatgatgac cttgtcttcc atttcctctg aagtgtcaca tgacatgggg ggggggg acg
1150921 cctgtgtttg gcatcaacct gttgttctgt gtcaggtaat gtgggggcca ccacaacaca
1150981 ctggactgtg tgttccatac aatgaacgtc tcacatgtgt tgagctgaac aaatcaaccc
1151041 aagctcaacg caggcatgac gctgaatcct cctcctgatt taaggaagcg gaatgaaagc
1151101 ctcgatgata cagtcacagt gacatttccc ttaaagtcac tgtgacacct gcacaggtaa
1151161 atcagtgttt tatgtgccat cgccttcatc ctgcacgttc actgttaaag gctgaagata
1151221 actcatcaca tagaagtgtt ttttttattc ccttgtttgc ttgttgttgt tgtctttgat
1151281 tatttttcat cctgcttgtt agaggacctt caaggttatg tcagccggga ccttccttat
1151341 tgtgtattct cgctgggtga cccgcagccg tcatcagaat gcaccggggc ctgacatgac
1151401 tcagtcgctt tgcagataag aggctgccag ccacattgaa gatgtattaa catcagagga
1151461 gggaaaatat ataccttgcc atttcagaag gcagactttc tctccctcac aatcgcttcc
1151521 tttccccctc tgcaacatat aaaaataatt aatctccaat agagatatgc gctggcggct
1151581 ctgccttgtg gatagcaaaa caggcctggt ttttaataaa gcaggaagtt aataagaagc
1151641 aggccattta aaattccatt atcagtgcaa gtatgtattt tcatgaggag cagaaccatc
1151701 acctttgatt tccctccagg aaaatgacca aaactgctga aagctttgga tttttaagca
1151761 tgtggcctca gtgtcagtga gtagcagggt tatttcgtcc aattagtcca ccaatttcag
1151821 atatgaattt agcaatccgt gcacttaaga gacaaacatt acacagcact cgtgccacta
1151881 aatcaatgct tcaattatgg ctaagagaat taaaaggtaa gtagttaaga gtgaaatggc

1151941 acaactccct gagtccctga ttctcccgca gccctgccag gtgttggccg ttccggcacg
1152001 tgcctgccca gacatccaac acccacacac aaacagcaag caaactccag attcacatgc
1152061 acaccgccag ccaactgccc atgacatgaa cactatccca ggctcaccag gcgccactta
1152121 aaggctcccg ctttgaaact gacactccac ttggcagcac tagcaaacaa aagagagagt
1152181 gtgagggaaa cagagggaga gacgggggg gggggctgat atttcccaac aaaagctctc
1152241 tgcacactga acgtgcactg agatgctgta aaatgtggct gcccgattca ctggcgacta
1152301 tttttgaacc cccacccttt ggcctgagcc atgccctttc ccaccacctc tctctccgcc
1152361 aaatggcacg agcacgtgcg tgcacgtgag tccgtgtcat cgcggtgaag gtaaggagac
1152421 gctgacacag ttaaatgtag catgtcacca ggctgcatgt ggcagattag caggtacttc
1152481 cagagacgct tcttctggca atcaggaaaa caaggacact ttggcctgaa ggttaaagta
1152541 ataataggaa cagtgcgaca ccaaggccat attaggacct gtggcaggcc cactcgtctg
1152601 gtgagggtag agtgtgacag agcaagtatg taggacaggt gagtttattt ctggggctgt
1152661 ttatttttat aggacgttat tatttctgca attgtcacct cttggtaaat tctttttatt
1152721 ggccctctaa atttgccctc ctagcgtatt tttgctgctt tcaaatgcac ctgtgtcttt
1152781 taataaccgg gagacaattg tcttgctttt tagctgtagt ttttgaggct gcaaattaag
1152841 aatttacttt tcaaaacagt aaataatgaa acagcaagca aacattgagt tgattgagga
1152901 taaaaataca cattaaatga ggaaaacctg tcaatcattg atatattaat aatatctcta
1152961 cagactgcct gcaagaaaat gagacggacg ctcttcaaac tctaaagtgc tgtgaaaatg
1153021 ctgtgatgat gtccgtccca gtgactaaac acgcaagcag aattcagaga aaatgactta
1153081 catacatttt aaatccacaa acttaattaa gtagccttga gataattgga tgatcctttc
1153141 aggggcagaa aaacacggtg aaggtgaact gacattagga gacgtagggc ctcctggggg
1153201 aagggttgtg gtgtgtgtgg gttgtaaagc ctgagaggaa gacacttact cttgatgggc
1153261 atgatgagct gggggataac aggcaggatc ctgggccctc cgtgctccag catgtcatgg
1153321 accccctggc gggcgagcac cgcgtacgga tgagtggtct cgcttaggcc gtcgaagaac
1153381 aagggcagga agtgatggta gtctagcttg tcgatctcca cctgaggaca gaaatgtaca
1153441 ttcactttaa aatatggcaa aaagagcacc tttaatccag aggggccgac acagtgaggg
1153501 agcaggtcga ccttcacaca agggcgtttg atagagtgag cagccactga gggaagcgct

1153561 agctaacagg gacaacgttt tgacgctggc gctggtttta cgcctacaaa catagaaaaa
1153621 gttctgcaaa taattcaaac gattgctgag atttaagtct tcgttgtttt gtccttgact
1153681 ttgagcttgt ggcctccatt aataacccct ccagtcagca ccttctcaca tgcgcatgct
1153741 ttgcccacac aatggccgag gacagctggt caggagaaca ggataccggt gcctgcgctc
1153801 tggtacggac acacgtgtta acacctgtta ccagagatcg gcgcaggcgc gcgcacgtgt
1153861 aattgggggg ggggatggag ccgtgcgtcc ggctgtaggt caatagcagg ctttgatgtt
1153921 tccttggctg agcgtgagcc gctgtgtgac aaattaaagt gaagcctttc atgtctgtgg
1153981 agccatcaga ggaaagccac tccatcacat ggggccgcag gctgtccttg agccttctgt
1154041 gttcagacaa tcatatcaca ttttacatgt cagctcagtg ccgtgtggct acatttgagt
1154101 ccgctctgaa ggtacttgta ctttactcga gtatttccat ttcctgctgc ctatttttca
1154161 taacgtttgt ttgaacaagg gtgtctattt ttacgtgttt atgaaagggc caccggtcgt
1154221 gggggagcct ccctgtggca ctattgttga acaaaggatg agaggtcgaa taggcgatcc
1154281 tgttcgtcat cttatgaaag gtgatccctt tggaaagcag ctccaagcaa ccagcttgtc
1154341 cttgaggaca gctgaagccc taaggacggc ctcactctac gtctataaac aatcacatat
1154401 agagggcacg catgccctca cagaccctcc ccccgttgct ggtccacaat ggtccgtgct
1154461 aaaatgtcac ccgggcgctg atgtttggtg ctttcaagtc accggcaagc agaaccttca
1154521 aagcgagaca gacaagcgct ggctgccctc gccgcttaca tcccatcctt cttgatgaac
1154581 gtgtctcaga gcggccgcac acaaagagag gggaaatgac agcgagatgg agagacgggg
1154641 ccagggagag aacgctccca ctgttaacac agagagacaa ggctgatcgc tttgaggcaa
1154701 tttgtcggcc aaccttgacc ttttctccca cctcggtcaa gatcgtcgtt gtgagtcagg
1154761 cgcggcgagg gaggagagcg aacgcaaaca tgcagtcaca tgggcggaaa cgaaagggtg
1154821 tgaggtcgat gctagccagt gccgggcaat aaaatgcaca aattcaatag aatgttctgc
1154881 acttctttca tcggagagat ggagagacaa atctacttta atacaagacg tttccatctt
1154941 cacaattaaa tttggatcct ccaaaatgga tataatgtta ttctcaaaga cttaaagttc
1155001 ctttaaattg acaggacagt aaaattaaaa gcctaaggaa tacatgaaat cactcaataa
1155061 aaatgtaaac gtaaactcgc aataccaacc aatacttcaa gctattatgg agtgtagaca
1155121 cacacgcata cacacacaca cagacacaca cacacacaca cacagacaca catattctca

1155181 aaaacacaat cccgtggaat cttaatcagc agctgggctg agcggtaagt
cttgcatccg
1155241 ttatcgattc tccgcccagg tttgtttgtc tttgggtgcc tgtgggaatt
ccccccggag
1155301 cccggggagt caatgcgagt ggagctaaga aagactatca gcaaagttgc
agcaattagc
1155361 cttgggacac agagccgcac gtctactctg gtcagcgccg cgatagcaga
cacacagaag
1155421 cagacaagac caggaacaag tctggggaga gcctctggtc attaaacaac
agtatcagca
1155481 gccccccccc ccacccatga cactcaggag accagcagga gcggaaactg
caggctgtcg
1155541 cattaaatac acactactgg acgtgaccga ctgtaggtgt aggtgtgcgg
gtgggactga
1155601 ggacaagtgg cctttagctg cagccctact atctcctaca gagctggacc
acctccgaca
1155661 ctaaacattc catccaaaga tacagcagct tatttaaagg caatgaaaac
agaatagatg
1155721 ggctgaaaat ataccgtgac ccgaccattt gtgattctaa cctttggcat
ctcacaaaaa
1155781 gcgcggctca taaatcacca ggcagctcgc catctgtttg acaatgatgc
tattttaatt
1155841 gatattgtgg taatgagttg acctggtttc tgttaccacg agcatggaac
gagctggcag
1155901 atgagcggct gtcggttcgg cttggagagg ggaacagtga gtcattcatt
acagtcagcg
1155961 tggcccaaca agcacaagga agtgtgcaaa agaatatcaa aatggagaac
cagtcaagac
1156021 aaaactggca ttagcattag cttttccctg ctacaggaaa actaacataa
ctttccaaac
1156081 atcttgacct tgtcatcaat agcagttccc tgctcatact attcatttat
ttatccaagc
1156141 ctgccccaat tttaatgaca aaactcctgt actgtgcaac tctgtacact
tactgctaaa
1156201 ggaaacacca gatccataga aagaaaataa tattcatttt ttctcggaga
aagaagaccg
1156261 gaaccggcaa atgaatgggg ctcgagttac tcccgttatc tcacttgaca
gctgtttcca
1156321 tctaacaagc atgcaagtat gcttaattca aggcaatacc agtaacgtgc
acggagtcac
1156381 gggaggtgat agcaaacaca agctggatag gatttctgtg gcgtcaaaga
tagttcaaaa
1156441 gaaggtcaga aagtgttatc agtttttgtg aaaacacaca caaacacaca
cacacacaca
1156501 cacacaaaga taaagagaaa tgtattgcca agagtcccaa atgttatatt
ttgagcaaca
1156561 aaagagccag aataattaaa ctggtgacaa tagcaaacgc actgcttttg
ttggtgttgc
1156621 tattaagaaa attgagaaag tgcattttct cttcccattt aaaaagaggg
gacattttca
1156681 ttcaggagct tgttatgctg gcaacggtag tgaaatccaa tgtgtttatg
ggcagttcca
1156741 aatggtcaaa ttatggtcaa acagttaaac acaatcaacc tgatttgctc
ctttcctttt

1156801 ttctgcagca ggttcctgag tctagtctgg tgtggagcgg gtccaagagg aggagacgtt
1156861 gaggggcagg ggagaagctg tctgaagcct gctccacttc taaaggtgaa gaagctaaat
1156921 aaagcaaaga cgctgctccc tgtgatggaa gtaggccaga atggtgagta aatgagaaaa
1156981 ggtgggaagg aagaagacag aaacactatc caaatgaact ttgccttata ttcacgtgcc
1157041 gttagcaaat attctgtgtc attctggaca ttcacgtgcc ccggccgtgg tcgcaaaagt
1157101 cacagtttgc cggttaatgg tctgattgca tctttgtctt gtctgtacgg cccctgctct
1157161 tagctctcag tgattacagc tgagcagccg agcttgacaa cacgcaggca gagcctaatc
1157221 aagtcggaga gagggaggtg taatcagcat ttcccctcca atcagcccca aaatctgtct
1157281 ctaatctgta gccagccatt gatggctttg attgatagaa agcattggtt atctgtctat
1157341 gggggctcgc agggagcagc gcagcaagcg ggagcgagag acagattcgg cttatcagcc
1157401 atcgctacat ttgttgctac ttccctcgtt gagcgagcac tttttcctca caggctacaa
1157461 ccactgctct cctctcattt atcataccga agaattaaat gtggacactt caaagtactt
1157521 ccacacacac gggtccctgg ggcatgtgtt ttctccctgt ttaaaatgga atggcccaat
1157581 ccatcactgg ctgtttgcga cggaaacgga aactataagg agtatttgtg tatatgcgtg
1157641 tttatgtgaa cgcacaggca tgtatctgtc atcatacggg cccaggatga ctgtgttaca
1157701 ctgctaaaaa tgtctctcaa acgtgcttgt ttacaccgag cacgcgatgc attgatttta
1157761 ccaacaacgc accgaaacaa tagggaccgt gacaaatgtt ggttctcata acagggtggt
1157821 tttagcagac tgtctgccat cacttatgca cgtggatcaa tagaataaga aaacaaagat
1157881 cagatattga ctgcacacat aggcacattt gtttccatta gctatgctgg aacgcctcat
1157941 ttccctcaca tttagttcaa ccttagttaa taatatctct ctaggtttca ggttcaattc
1158001 caggcttgag ccttatctct gtacagtttt gcatgggttt ccccggagat tccaatgtcc
1158061 tccgtttaga gctatcagtc tgaagaggtt ttggattact tggtttgagt tattgcaatc
1158121 tgcaaactca aactcaataa aatgcaacac gggagggaaa ctagccctga agaacatgaa
1158181 gagcatttgt ttaatgaaca tccctgtggt accgtgttcc gtgcggtcgg ccgggccggc
1158241 cggccaaaac atcgggagca gcaccagtga ctaagtaaaa aactattttc aacataacaa
1158301 attcaacaaa tgaatccagc tgcgaatgct ggtgccaaat gtgctgagtc agtaatcccc
1158361 tccctaagcc gtggagaggc acacaggatc tgcagcatca gtagcaatcc gctgtgtaca

1158421 gtactctcag cagcgagcag cagaggcagg acgcctgtca aagcttggcc cgctgcccaa
1158481 cgggacctgc ttgccagtgg aaagcccacg ttctctggac ggcagatgtt aagtttggac
1158541 tcacggagat gcactggaca caaaactgca gagctgtgac cagcagggtt tatttaaagg
1158601 tcttaaagga tgtgtttaga gtctccttcc aatgaagcct ggagaggaga ctcagacagg
1158661 taaccagagg tctggttcat ccagacatgg ctgcagcaga gcggtttttc ctggatcacc
1158721 cgcagatctg acgggtttcc aggcgatgct gccacaaagg tgacgattga tctgataaat
1158781 cagaggatga tcagctgttt ttcttttttt cccctgctgg aatgatgtga ttgtacacgg
1158841 ataaagccaa ctgctgccaa gaggacggag acaccagagc gggatcagaa agcgtacacg
1158901 gaaaactaat aaagagagtg tgacgccgca ataagtggca gatgcaccat ggatgcactg
1158961 gaggttcagt agaatgaatt aaaacacctg acaatgggaa gaaagtggta gaaatattct
1159021 tttgaagaaa tatccttttg atcctaacaa ggagatcaat ctgtcatcct taaaggagaa
1159081 agctaactgt gagagaaagc taagacagcg gcgctccatt ttaacctgag tgggagaaat
1159141 gaagagatga gtgtttgctg ggcagactga tgcttatgtg catccagggt tgggtccctc
1159201 tcatctgagg gaaaagcttt ggtgatcaat ggggatcgac tgtttgagtg gcagctgtcc
1159261 aaatgatggg tcggatgaga taatggctaa aaagaatgaa gctttattca ccaggaggcc
1159321 cacttacaca gacggcccca tcatccatct gcaggcagag ttctgccgtt tcttcacatt
1159381 gatgatagca tcttcggtcc catggttcaa ctctttcact ggaggccttt gagaggactt
1159441 tgagggaatc taaaaggaaa ggattgtttc agggaggctg actaatgtgc tcgacaagcg
1159501 gggccttatg agtgggatga aagcgagctt ttgaaatgtc gctggaaatg tagtgccgcg
1159561 tgaatcggag cgctcccctg ttcagaactg tgactacttc tgcaggattc ctgcttcgga
1159621 gtctggctct gatcatttgt taatcatatg aacgcaccac ggcgcatgct gtagccccag
1159681 tcatatccga gtcctcctgc tggaaacaaa cagcaccaaa acagcacaaa aagggtgtaa
1159741 atgtaacggg gagactaaaa ggatttgagg tgaaacacac ccacatgcac ccaccaccag
1159801 cggctgaggt agcaggcgct cccatgaccc ttcagggtga tgacacgtcg ggaaaatgat
1159861 ggtgcagttt gagctctaag cccggatgga acagtccttt ccacactatg agctcagagt
1159921 tacaccgggg tgggtcaagg gtcacagaag gggatacttc tggtgatggg acagcgtgag
1159981 cacggcagat taaattctgt tgttacgtgg cggcctggtg tctgtcggtc accctgcaga

1160041 cataaatctc tattgttgta tgctcaggtc gttttttttg ggggggtggg ggggttcatc
1160101 cggtgtggac cttttactgg cagcaaccga tgaaaaggtg agcagctacg ggatgaagtg
1160161 ctgccacttt atccttttg catttgatgc aagcaaacac aggcgcgtgg cccctgggcg
1160221 accctctgcc tccttccgtt ttgaatatct acctcttcag agtaattaat ccattttgtc
1160281 tctccatcct tctcgagtgt ttttgcatga taccgtagct acgagcctca ctgaagagaa
1160341 gcagacttga gttctgaaca ttaatcaagt cactgcaact tctttgccac tattttctga
1160401 tatccctgca cacaagaaag tggagcagct gaaggtctat tcgaattcaa attctgtgta
1160461 tacttttagg cagaattaat tttctgcatt aataccatgt gcagcaaata ataacaacaa
1160521 aaacacgcgt tctcctttcg gtctcctcct tttcgaactt aatgtctaaa tttgaatgtg
1160581 cacgtctgca gtagctgacc tatttacatt ataaccagga gacattccat taaaatcaaa
1160641 gttcacttgt ttagacggaa taatcactta tgccgcgaca gtttttttaga cttcctgaac
1160701 atattgcgtg gattccaaat tcatttttga ctagtaaata atgcgaagca tttagaaaac
1160761 aagtgaaaaa catctgcgtc cggcaggctg tgctttatta tctctgctga tgcactggtg
1160821 gtaatcaccg gcgtggctca ggagaaatca gggaaggctg aagtgcctct gaaacccaaa
1160881 gagtggggat ccagagagag gcattttgaa tgaaatatgg cgtgtattac tcctttctgc
1160941 tctccagaca gggtcgtctg tcatagccag gaagaggcca gagaaagtgg gctaagtggt
1161001 caagccattt cttgtgcctt tccaagtggc tgaatagata ttaggatttt acaggctatt
1161061 acacaccata attgcacagc atattgaatt tcaagcgatt ttcttaaact gactccctct
1161121 tccccgtttc tataaataaa cattttacag catgtaaagt atgcaacacg tatggggtgt
1161181 gatttacagg ctgcgcgtga cacacactct gtggcgctca cataaattca ataacagcac
1161241 tctactaatt gcatggctta aacccagctt tggctcttta gatattcatc taagccaatt
1161301 agacgagagg cgggatggag acggggcgta atcctgactg cagagcaatg tcactggagg
1161361 acctgctgga tgcaatttac tcctaaataa acacccctga agggtttagt cacaaacaca
1161421 aaggaaatat aacttttca aagatataat aagctgtaca ggtgaattaa tagcctcctt
1161481 atggttcaga agaggagtgg ctttatttgg aggtgattct atatgagatc cattttctaa
1161541 aggtgcgctc aggtttggtg ctttgcttga tgtgtgacac acacaatgac aatcatgaca
1161601 aaaccccaga atcatcgata aaatattaaa gcagaaattg tgacgtaaat taagatggag

1161661 aaggcatcgc aacttcctcc ccctgtacaa aaatgaaccc atttgacatt gaaatgggct
1161721 ctgccagtct gtaattgtga tagattttgg accgagagtc ttcacagtat attgagctgg
1161781 gagcagaatc catatatcaa gaactaaata accaatacct aaatggcatc ttacaattaa
1161841 gaatgctctc ctcttagtgg gttcctgaaa gatctccgct cttgagaatc ctgtctaatt
1161901 cagagctcac ttcatatatg catcggagct tcatggacag caccatctga gctttgttat
1161961 gataaagagg agagagagaa aagattatga aatgctgatg gagcaccctc agcgttgcca
1162021 ttcatcaaca taattaacca taatcagcac catccgcaga ggtggatcac ggagagagaa
1162081 tctgttaaat atatggaatg aagcagagct gcaaaatcat gtagcctcct atttaaaaag
1162141 gcccattaaa tggctgcaga ccagggagac atgcagcatc acagccacca ctctaatgct
1162201 gttaactacc caaagttctc acaattcctt cgtgacaaaa attggtaaag atgaacagtg
1162261 tttctgtcta aacaagtctc tgggcttttt tatatcacta ttctaatcat gaaataaact
1162321 gttgcagata tggtcttatg ctattattgt tgaatgtaaa gcactaaacg gcagaggctc
1162381 ctcatgcacc acaccattca actgtttttg tccattcctg cataattcca ctgctgatcg
1162441 gggtcagcgg aggttcctgt ctggaatagc ccgtcttcag cccagttccc attacaaatg
1162501 tttatctgtt ccacctcatc cattattaaa aggcgaatgc agcaggggcc atgtagacta
1162561 ctttatgtgg gtttaaaatt taaatacttc aaaatttctg ggttattgga acaaagacct
1162621 gacagcacat aggtatatta atgcacgggt tcacacagac atttttagct aatgctgcat
1162681 tttactcaag aggctgtgat agctgtctta ccattaacta tattagaagc ctaaaatact
1162741 gattaggata taattaatat gaaaaaaggc caaggtaaat tacttactgt catgtaataa
1162801 cgtctactag agtttaaatc agtgattcca caaagcacga atgcaaaaga gctcagacct
1162861 ggatctctgg atctggtttg tgcactcagt aacaaataac ttttatattt caaatgctac
1162921 accttgcaca actgactgaa tgctgctgtt ttgtcatttc aattgtttcc ctcaaaggtc
1162981 acaaagaaag ttccaacaca gcccttctaa atggcttgta cgttgaaaag ggatccagtg
1163041 ctccgtgatt cacaagctgg actgccagga gccaatcgcc tctgaccctg accttttcag
1163101 gaactaatag aattacagca cattgaatgc caattaagta aacacataag tagaaaacaa
1163161 aggcggcaga aagagcacag gagtttaatt attataaaac ccctaaaggt aaacacatgt
1163221 ttctgcagca gccagcctcg cagagatata ggtcaatgag gatagaatgg atgagcgtgt

1163281 gtgtgtgtgc atgtgtgtgt gtgagtgtgt agaccgaggt gcaagaaaat aaaagccagg
1163341 aggaggcttt caaacgagcg aggcaggccg gcttacgaaa gagttgttaa tttagcctcg
1163401 ttttaattag ctcttctgcc agagctgagc aaagctatcg gggagccctg aagatctgag
1163461 caggctgaaa gaggcaacca gcttggcagg cctaacacag tttatctgac caacagctgg
1163521 ccacacagca cccaccacgc ctggaaccac tcagaggttg aaaaaacaga cagatattcc
1163581 tggaactggg tcgaccatta tggacgacac cgacttcaac atgttttatt aaattttgtt
1163641 tttattttta ccactcccca tgcctaaatt aattcagaat ttggtattta ttacctctta
1163701 aaaatgcaat gacaacatct gaaagttgct tctgctcctg actcatggtc tctagggacg
1163761 aacctttaca cagagcatgc ctaaactata ttgaaccctg tgtagatatc ctctctgtag
1163821 atatgagggc agctgatatt taaagagttt gactatttta tgagtttcac tcctataacc
1163881 aggcaaaaat aagcaaggag gcgattcctt gtgagcagca aacagtgcat gccctgtcag
1163941 aatgagcaaa tatgagttta agggccttag tcttcggaga ggatttatga catggggtct
1164001 tattatctgt aggtcggatt actgccatac gctcccatcc aaaaagagag attccacgcc
1164061 acataaagcc gagagtgttg acagagctgt gccatactct cacttccagc ccttattttt
1164121 tggatgttcc acttctttgg ctgataatga tgggttagtc acccaaagcc tgatggatcc
1164181 actcacactt cacttgttgg ccgtagcatc ggaaaaatcc aaaaggtcaa gaggacctgg
1164241 gtgtttttct ttggtgacgc caaactgatg aagcctccac ttgaaagaga gagaaattag
1164301 tagatgaaat agataccttg atggagatac agcaaagtct gcgcagtaac gttggggaag
1164361 aggttattgt acttgacttt gaagacagag gaagcggcgg gagtgctcca gacagaggag
1164421 tgtgattaac atgctgcgga cagccagtta gactctgtct ggggaaatat acagcgactg
1164481 ctttaataag ctttaataag atgcagcctg tttattgtcc tgttaattag caaaagatgt
1164541 aaacagaatc cttaaagatg ctcagtgtga gaaacaagac acaaatagca tttcacaaaa
1164601 tccactagat tcaaacaaac atcataaaga ctttgttcaa aagtccccca acagtaaagc
1164661 acacagattt tacggtccaa ttattctgga caacgaccac caaaaaaact tgtgtatttc
1164721 ctcattgtaa acacattacc tatagagtag aacctgcagg aacatgctca ctgtacacac
1164781 acctgcacac aatagcacgg cccagaccag tactcaacaa ctgctccagg cctctaattg
1164841 gccccgttac tcctcctcat cactctctct ttttatgctc atgaaaggag agggtattat

1164901 gtctgccaca ttcctctaac aagggcctgg cactgcaact ggcacttacc aaagtacaat
1164961 aaaactctcc aaatgaaacc tggaggaaaa aacacccaac aagagagaga gagagagcat
1165021 gtgtgtgtgt gtgtgtgtgt gtgtgtgtgt gtgtgtgtgt gtgtgtgtgt gttgacagag
1165081 actgacagaa tggggatgag atggagatca gagccataca ccgaggtggt gtgctacaaa
1165141 ttgggagaca aaaaggatgc accctggttt cctgtagagg acccagaggt actgagaaga
1165201 gctcattcag gagcatctaa atatgctcag agaatgtgct actccaaccc ctgaaggtcc
1165261 caaccaatca ccacaatgtc agatataatg aagaccactg aatggttctt gctcaggctc
1165321 ctctggactc tcggttcacg cagttcacgt acgaggagga gaaagtggtg gtcgatgaag
1165381 ctatcctcta tctgctgcaa aggcctgatg ctcaactggt tatataaggg attggaactg
1165441 tcaggatcat attctctgac ttcatcagcg ccttcaacac catttagcac ctcctgctaa
1165501 gatgacatat gggggtgaaa tccaacctgt tggcccatat tacagaatca ctgactgaga
1165561 gacataatct cgcctgacac tgtggtccac agcaacgggg aatcatgagg gccagttctc
1165621 caccctgtct gttcacccgt acacgcctga caattacaac tcagagtcaa agcatatgct
1165681 gaagaactga catttaatgg tggtgtgcat cagggctaga cgggaggggg ggtagaaatg
1165741 gagggtggag gagaaagggt gaagtggtgc aggaactaca ggtgtttggg acagccgttg
1165801 gaagtaacag gacgggtggt gctgtacagg aggcggccgc ggggaccttc agcattacag
1165861 gctggtgttc ttctctatgc tagagtggcg ctatgcatct agaaagactg gtgaagatga
1165921 cattgctcct ttacaacagg gacagacgga gagactccga acaaaaagct ctccatcgtg
1165981 gacgaccctg atccccctca gtaaatagtt tttgcacact caaagctttc ttcacatgtg
1166041 cacacagtgg tcctttgtaa catagacctt tatatatgta tatataaatc tgagtcaaag
1166101 atccttatgg tgtaatggac agcctgtctg tgtgtgtgtg tgtgggtgtg tgtctgctgc
1166161 agtgtctgag gctcttaaac cgttactgct gattatgaaa cacggctgcc gcagactctc
1166221 gctctcacta tttattctcc catctactgc agagccaatt tgctaatgga tgcagcaacc
1166281 ttgtgattta ttgttgtcaa agatgtgctt gaagtaaaat gcactaaatg atttaagttt
1166341 gcaagtaaaa tttacatttg aaagcctgcc cactctggag agtgcttcac atcaaatatg
1166401 caaagttgat tatgtggacc atctatttag cgcttctaaa acattgccca ggcccactca
1166461 gatatattag actctatatg gattgtccaa ggcttaggtc tattcttgga ttcagttacc

1166521 aagatggaaa ttaaaagagc gatagaatgg cagaatgcaa aaactggaag tgtctggcaa
1166581 catttctatt aaagctacag gaaaggtgac tccagacgtc tgcagaacgc agcacagaac
1166641 ataacaacct attctccttt cacttttttc attggaggta aaaccactgt ctctactcac
1166701 ttgtgcaggc tcgccaggtc tttgtttcct ctgcctctcc agcctctttt aactcatatc
1166761 agtactatat cacagataga gaccagttgg aatgcctgag agctccccag ggtgtctggc
1166821 agcggctaaa accagctgtg gctcgcggag ctaagtgtcc atccataaag gactaagaca
1166881 tgcatgtgct tatcattttt tatttcagtt tctggatttc tactcaactt catctcccct
1166941 ttgtgtgtct ggccctccct gcacccattc tgaaagaaat aagaaataag agacacaaac
1167001 ggaagggaca gatgcccagc tcatggcagg gaccgcaatt cactctgtgc ttcagcccag
1167061 aactttctaa tattcccagc gacaaaaaaa gatagtgttc atatcagcac tcacaatcag
1167121 tgcacataaa cccaagcgtc tgctccattc tgcaattttg aatgtcaaaa atgcaatgta
1167181 acacccaaag ttgctgtctc acgtgtgaat gaaccacctt actgtctgaa gcagatttca
1167241 ccacagcctc agaatcaaat attgcctttt tctgttatta aagcacttta gctgcatggt
1167301 gtcttgaccc ctgtggagca tttcagcacc gggagacaca tgtgaacctt tgctttgccc
1167361 tgcttttaaa atcgctgctg tgaagacaaa ataacatact ttttaataag aaatgtgcca
1167421 aaacagaact aaagctctag aagaggcccc ctattatatt gtatatctga ggcatctttg
1167481 gcagttcctt ccacgctgtt cagctgtgat ttactgagac acagcctgca tgtgtgatca
1167541 cagattggca aactgactct cttttcttcc cttcatattg tgccatgtca ttaaaacata
1167601 gggttggaga gacagtatca gatgcaattt tccgaaaaga aaaaagaaaa tttaacatag
1167661 agcagaacac ggtcccccac aacctctgtg ctgtgatcta taaagatggg cgcgagctaa
1167721 tgagagtctg acctgcaacg tagctgacga ggagccgtgt aaagaggcat tatggggaag
1167781 gaaagaaaag gagcgtatgg gagccgaaca agaaagaaac acagggggag acggaagata
1167841 aagagagcta aagatgggga ggaagaattg gcttgaaatt ttctgacaat gaaacgtctc
1167901 cagtaattgt tcctgcggtg ataaatggcg tcccgtcgta cagcaggtct ccttgtcctt
1167961 gggaggaaaa gagatgagga caggggagga gggaggcgat acctagatgg actggcgacg
1168021 cagtacttaa cccggctcca gcagcaggca cctagctgaa ggtgactgtg tatgtgtgta
1168081 taatgtcaca tattctactc cacacttgtg ccttttgtgt ctgttttgta aaaacgtaca

1168141 gtacgtcgaa gcgcttcctg ccaaagagaa gcagaaggca acgcgcttgt gaatgtggtt
1168201 ctgccctgag ggatgaggat gacagaataa aaacacatcg tgacacttta gtaatattcc
1168261 ctccaccaca cgctcacagc gctcggtgcc ccacagatat tttctgcagc catccaaggg
1168321 agaaagataa aaccttatta tgttgtcttt cttcaggaaa cgattgtttc aaacacattt
1168381 agagaacaat tccgtcatga tttccgatgc ctttatgatg ggtttgtatg ataagtctac
1168441 tgctacgaga ttaaggttaa ttaaacctgt caggctgcca acaccagcct ttactcacgc
1168501 cttttatgtt aatctcattg ttttccacac agtctagact tttcattacc ctggtcataa
1168561 taatacatca tttaaaacta ttagtcacaa agtgtgttat tcacttctag aattgtttta
1168621 tccctgttat ctataaacca caaagcaata aaaaaacagc agcttgtgac atagccattt
1168681 tgaattattt aaaaaatatt atttataaat aaatattatt tgagcatatt gtacatctga
1168741 gtcaaatgtc aatcaatata atcccaactg cttaatgccc ataaatacca ttatgacaga
1168801 gaattgtgct gtggattggc acccacaact ttcaacttct ttttccacag cagcatcatt
1168861 tttgcatgaa tgttggctcc aaaagtgaac aatgtgtttt taaaaggttc ggcttcacgg
1168921 acttcaaaac aagtctgttc ttcatagata agcatattaa ttacgttgac gggcatctgt
1168981 gtacatggtg gagaagaagg aaggtgataa tcatagtgga gaattggccc gatcgtgacg
1169041 aaggaagaaa aagaaatcca ccacactctc tgatgctaca tctatcactt tagctctgaa
1169101 aagaaaccgt tccttctaca gcaggacata aagactggct gaagaagttg ttcaagctgg
1169161 aaaaggattg tgaaggcgag gaagaggaag acacactata aatgctaaag agtgcagagc
1169221 cctgagttag ccatgacaca cattaatcat ctgtgtcctc tctgcgctgc atacagatgc
1169281 ctgcagctgc agctgctgct gctgctgacg atgatagtga tgatgatagt gatgatgatg
1169341 atgatgatag tgatgatgat gatgatgagg gtccaaccct gcgtgtagct acatacagct
1169401 tcgacactaa cacaggtgag ataaactcct cactactcct tcagttttcc atctcaatgt
1169461 tctctatacg ttttctcctc acccacacca catcaccctt cctcccacat acgtgcacac
1169521 tctgcgtgca ggcgggcaca cttatctgcc ccccttcctg tgtttggtcc ctgcaggcca
1169581 cgggtaggtc catgtcaggc gaggagcagg gggttgaaag caaaaggttg cgctcattat
1169641 acagattaaa cctgtaaatg ccctgccgtt ggtgtaatcg atgtgccact tcattgcagt
1169701 gacttattga tcaatcttct ctggggggtc gcacacagtg catcgttata gcttcaatcc

1169761 agtgaatgcc tttacttttc aaccctttgt attccttaga cctgcaccct aacagctcag
1169821 agccttgaaa gcggtgtgct ctcgtatgca taaggttcac cactgctgtc atacagcagc
1169881 aatactgtct actgtgcacg tctgcaaact tgcaaacatc tgagccagct gagcagtgga
1169941 gagagacttc atgtcaccat tagagaaaga tattttatta cttccatgtc ctacctctgc
1170001 tctatcacac ctggttaacc tggttacttt aagcccataa atagaataaa aggtgaaaaa
1170061 aatgtgggaa aaagacaaat tacaagaaac tcaatagaaa tcccaccacc agtctgtatt
1170121 gaagacctcc aatgaactgc agggactcta tgtttaaggc tgcaaagact tttttgaaaa
1170181 gaaatgaaga gaagaaaggc tgagagacac ttttcttcaa atgaggccgt cctctgcgaa
1170241 tccatagata atgacttcaa attacaaata acagaggtcc ttattgtccc atatggagaa
1170301 gcccagccca aacgggcctt ttttcctctc ataagttgca ggtgcatctg tctgaaatgg
1170361 aaggatctga aatcacacaa tctctctcag ttagtcgcac caaaagcttc cttttttga
1170421 accactgtac agtttctgtg tcctgtattc aggccggtca catgtgagaa tgtggcctta
1170481 aaaatgttgc tctggtgtgc tgttgctctg gttacctgct gtagaaactg caggtaatgg
1170541 aaaaatcctg gaatttctca cccgttacaa ggaggcagat ctttcaagtt ggagcgctcg
1170601 aggttgaacc tgacagcccc tcccagaggc acagtatcct ctctccatct gcagactaca
1170661 gacctgctaa cagaaccagc agacccccac attactgaga aatgcgtcac ttgtggcggc
1170721 tgcgagcgca ctgctgtggt atttgccacg agctcgagtg taggattctg acccgtcact
1170781 gcggggactc ctgtcctacc tggcgcggta ttaaagtgtc tattgttcat cagttccatt
1170841 ctcaccttca tttctgggaa attgcaccct gcctgacaaa tggcaggatg gcatggctcc
1170901 acgggctgcc caccagcaca ctgccgctac tggcgggaag gacgggaaga aattgcctcc
1170961 cgttgcttga attactgagc aagctcattc cgcagaggag ccgcagtcaa ctccacagga
1171021 gggggatgga acaacatccg ctcatgtgag cttacatgat gatggggtca agggtaaagc
1171081 gacagcttat tccaatcagc tatcagcctt gatttgttat ttcaccaaag tggaagccca
1171141 gcgcgcaggt gagcattaat gtgtgctgct gcataattca aatgctgagc tggaaatgga
1171201 acatgcattt tacattttaa aacatccaaa ttgctaaata tcccgttagc gttggatccg
1171261 tagcacagcc aagggcattc ttttttccgg ataggacttc atatacttga gttgcaccct
1171321 ttgaattggc ttttcgctaa agtgttcttc aaagaaatga gtcacaagtt ttaagaataa

1171381 aatcttcaca ttggaggggg gggggggggg gggggggggtg agagctcttt gtcaaattta
1171441 aatgccgcac tatgttctct ctgacggcct ggtagttatt tattgccata gccattacgg
1171501 ttattagaaa tctcactcag gaagacaaat ggcagcttgg ctggtcattg gtcctctgtc
1171561 atggctgaca agggagacgc agcactctcg cctattgact tttttttgta gctaaagcca
1171621 atctctgcca gcacaacctt gcaattcaaa nnnnnnnnnn nnnnnnnnnn nnnnnnnnnn
1171681 nnnnnnnnnn nnnnnnnnnn nnnnnnnnnn nnnnnnnnnn nnnnnnnnnn nnnnnnnnnn
1171741 nnnnnnnnnn nnnnnnnnnn nnnnnnnnnn nnnnnnnnnn nnnnnnnnnn nnnnnnnnnn
1171801 nnnnnnnnnn nnnnnnnnnn nnnnnnnnnn nnnnnnnnnn nnnnnnnnnn nnnnnnnnnn
1171861 nnnnnnnnnn nnnnnnnnnn nnnnnnnnnn nnnnnnnnnn nnnnnnnnnn nnnntcatct
1171921 ctgatcttga actttttttcc catttcctta gaaatcggct gagcatcctg gcaggggcca
1171981 gcagagaaga ggcttttagt agggccattt cataagcaga ctttgtcttt cacagactga
1172041 cagattatca ttaatccttc tgtaaaagca caaagagcca ttaagagaaa acgctgggaa
1172101 agtctaaatg ttgtgtgtag ggctgcgagt gggaaagaac gacatttcga attgagacga
1172161 gacagaggtg tgtgtgtgtc gtgtgctttc tgaggcctgt gagacatatt tcactcagtg
1172221 gcccagaaag aatgacggat gattccagtt gtttcctcac agcggaaagt gaaaggtgac
1172281 acagtgatca gtgaggactg tggtcgaaca acaatatctc tgttgctgca aaaacaaggg
1172341 aaaacaaaga aaaaaaatag atgacaatgg gtgtcggatg aaaaatgcta ctgaggtccg
1172401 gacgtggaag gaagcgactt ctccgaccag gaattacacg taacaacacc aagactttcc
1172461 tggtcacaca aggtaaatga ctgaattctg tgattagaag gcaaggtaag acgagagaca
1172521 gaggtccacg aatcatgacg tgacaatttt ccacgctaga tctttattga ggatatgtat
1172581 tggtttagca ggggccaggg ttcggggcgg gcccgggtta actgctgttc cctgacagcg
1172641 cagatgagaa agtgatgtgc acagtggcag gtcactgtgt gtcagagcgc cagctaacac
1172701 tggctcattg ggaaaccagc acgctccatt taacttcaca gggcatccag tcactttccc
1172761 acatttctcg ccctccctct cggatctccc cttgcatggt ccttctccca cacccttcct
1172821 tcctaagtag cgtcactctg actcattagg gtaattgaga caggtcctcc tgacactaaa
1172881 gagctgttgg attgccaccc atttacgcgt ttaaaagagc gcttcatcaa gcctccgtgt
1172941 gtattctcct ctttgtggcg atacttctta acttcttttg ccttcacttt ccctttatcg

1173001 ggtcaatgtg gggaagtgta tgtgctgcta attggagtaa gcgactgatc tagaataccg
1173061 tctcttagtg ttgggaaagc cctcaaacta ttggagtgtg actgcgtgac atagaaaggg
1173121 taggggggtg aaaactgctg atctgagtat gctagggtag acgcaggcag gggtgaagat
1173181 tgagcgaagg gaatccacca cctcagcgat tctcggggct gtgctgttcc aagctgacaa
1173241 agacacccag attgcagcca gaggttgtct cggcgaggcg agatgagcca cggcgcccgt
1173301 catgaaagga gtcatcctta gcgatactgc tgggtcaggt tggactgatt gtcagatttt
1173361 tagccagtga gggcaccaca gcgctcggct cctcccgctc atctctgaac acaaatgatc
1173421 cacagctgcg attatacatc actaactgac gtctgtggag gttctcagtc acccaggtca
1173481 tgataacgac ctgaattact tactgacaca attagaaaca aaggtgtcag tccatcaatg
1173541 ctaaatgtct cgtatggcta gaaggaaaac agaaagctaa agtccagata gaaaggccca
1173601 aaaggaacca cccattttgc taccagtctt ttaaacgttg ccttgatttt cccccagccc
1173661 cgtacattca cctcccactc catctttctt atcagcgacg ggttggaaac aggagggaga
1173721 taaggaaacg ccagaaccaa tacaagatgt ggtggtcgaa caccggagca gattggccat
1173781 tgacaccgtc gtcggctaac aatggcgttt gagcagagga aaatggtcga cgggaataga
1173841 aacactgaaa taaaatttct cctagtgatg cattaagtgc cccgcggtgt taaccgttag
1173901 atctatgggt ttacggtcca ttccctgagg tgtggcggca ccgattagct cagtgcggtg
1173961 tagctgctgc cgagtacctt cattagttcc tgtcagtcaa tcactaccac aaatttgcac
1174021 agcacggaag cataacaata aataaatgga acagggtctt ctctcccctt tgggatgtcg
1174081 ataacaggtt tttgtgcatt cattgcaaca caaatcaggt tagcaataat acttctgtca
1174141 atcaattcat ctgtcagact gcttgattta tgcaggtgca acatgaaaac aaataggaac
1174201 aactcgaaca aaatgagtga ttttctgcac attgtaagaa caggaatcaa ctacacttgc
1174261 tcttcatgta ggtaagctac atctgcactt tatttttctt cagtatgatt atatgaagaa
1174321 caggttttgt gattataata aagttacagc ccagttttta gcacagccga tgccaagaac
1174381 tgaagagtgt gaaatcccac tgtgactggc ggcctgctga ttcacaacac atcagaatgt
1174441 tacaatgttc tctatttata agccaacaga atttgcagct gatgataact gaaactattt
1174501 tattttctgt tgttccatta taaagtattg ggcctaggtg acgtcaggcc ttctaatcta
1174561 tggtcaaatc tctgttgccg acacatatta catcttttgt gtgccgctct ctaataaatt

1174621 gctgcatttc tgtctgctat gatgacactg acgcaccttc tgctgatgtc tccactcagt
1174681 tattttcttt taagtctttt gtttaaccat ggccataaca taaaaccaca ctttctgcag
1174741 tgtaaacaca gccctaaagc tgttattcct cattgctaag gttattatta ttattataaa
1174801 aataaataat aacgaaagtt tgtttgtatg aaactcgttt atatcagttt cactgttttc
1174861 actgagtgtt ctgtatccat gagaggccac atatttttgt tcgcaagcac attttccaga
1174921 ataaaggacg attttgaagg ttgagctaag taaacacaca aaagtgcagg aaaacaaata
1174981 tagcagtatt acataagacc gacataaaat gtaatattat gtaacaaata aaaacacaag
1175041 aatgtttaat agaatattgt tgagtcttgc gtgctaccag aatattaacc acggcatcac
1175101 tgtcatacat cgatacagta tgtatttcac caatctgcaa gttcaatcgt acactagaaa
1175161 aaaggaaact ttaaatgagt cctggtgagt ctgaagactc cctcccttgg ttctgcaaag
1175221 tttgtgtttt tctccccgac ggtttccgcc ggctcaatta actattgatc agatttgtgg
1175281 aactgaatgt aaataattaa ccaaagtgca gaaagcgtct gcagctctgc aaacagcttc
1175341 aatacagagg cctccctgta tgtaatcaat agggcgtgga ctgcatgcac atcggggagt
1175401 atgaggacct gtgtgaggta gccacagaaa cagagaaagc gcagttataa ccggctctgc
1175461 agctgtgtca gcaaaaaaat taacatccag atctcggtaa aaatcaggca gcagctgttt
1175521 ccttggagat accagattgt gggggaaaa catctcattc tgagacatag aggcaagcta
1175581 attaatttgc cgataaggta tcctcaagta aattaaatcc gagaaattcc tgaactacga
1175641 tgtgtgagtt ttccaactct ggtgagatct gctccatcat cccataagca gctctgaggg
1175701 aaccgataac ctggttgtgt ccaagaaagc cggtgtaagt aaagatgtaa cacggcgagg
1175761 atagaaaagc tcaaagctct gaggggctgg aagtgtgggg tggcaccagt gggggaactg
1175821 agagagtaac cccatttgaa tatgaatgtc tcccatgatc cacatcaagg gcactgacag
1175881 gggcacctcc atctacaggc accttcagca ggtggagtcg cacaggtgtg tgtgtgtgtg
1175941 tgtgtgtgtg tgtgactgct tcctctgtgc agccatcagc cacggcattt ttccaaggta
1176001 ctttgcacag tgcattaatc acagagaccc ttgagcactt ctacggatcc aaatattccg
1176061 accttgaggt tagaagaaag acattagtcc cgatctattc aaagtattgg cgacagagag
1176121 gggagaggag ggaggggca gaaaagcttt caaggtaatg aaattctgct ataatttaaa
1176181 ggaaaagact tgtggggaca agcctaggcc agaggaagaa aaaaaaagat taaattggat

1176241 tcttccccct ttcttttaaa taaggcttaa gtgcatgtgg ggctagattt tctttcttta
1176301 tttcttttct tttttttaa caaaagcatg ataacagcat taacggccag aaagaatcaa
1176361 atcatttcca aaccaattaa cctttgacag aacggatgag tggactggaa tgagaatggt
1176421 gagccgggaa ggagagacag agaggcggta gcatgcagac ccagacgttc agtggggaga
1176481 aagctaccag ggtattctag cagctattag tgactgaatg actggcgtga agtctgcaat
1176541 ctctcacaca cgccgcttaa agcaaacaca cctgattccc ctgcgttcgc tttctagaaa
1176601 tgcttttgct ttgctgttta tttcgtctta ataacagtaa agttcacttt gactttgact
1176661 tggcctttga aaatctgcgg ccgcccgaca tcgttcccat ctgcagcacg tgaggtcccg
1176721 gtgaaggaaa gagcaaaggg cgaaagaacg ggaggaacag aaaagaccgc cctccatttc
1176781 tgtccatcct gactgatagc agaggtggtc tgcagttaac cccacttatg ctttataccg
1176841 ctcctcactc tctgtctccc tctatatatc tatttctaac cctccctcac tggctcacac
1176901 acatctgcag ccggtccatt aagttaatta aaggctccat tttgagctgg agcccgctag
1176961 caaatacgag tgccaatcaa tagatctctg gcttattttg tgggagggag ggggtgacag
1177021 tgcgggtact gcattctgac atgatttgtt gctttggcag atgagccatg tcccctccta
1177081 tcttgtcctc tgtctctgtc taatttggta tccactctgc cccccccgcc acaaactgat
1177141 tcccactctt cggtgtgatg tcatacatca cattcttctg ccttctaccc ggagctactc
1177201 atggattttc ccgtcatcct acgaggagcc gcagtcccgc gtcctctaca aatgtggtga
1177261 aggctgttgt gcgtttgtgt cagtaaggat ctgcattggt gcgccagctg tgggcggctg
1177321 cagctggagc ctcagcaggt ggccagatgg acgttcaaca ggatgtgacc tcagcggccc
1177381 aaacaggaag tcagaattat tacaggaaat gtggcactgc cgtgagcaat gctgaggcat
1177441 ccgccagtat gtcattatca tcatcatcct cctgctcatc atcatcctca tcatcaacat
1177501 catcatcatc atcctcatca tcatcaagtc ctgcctgaca gcagctatta gcaaagacag
1177561 caaacaaaaa ccagtcaaaa atggaaaggt gaacaaactt tcagagcaga tacaatagat
1177621 ataagtgaat aatttctttt tttgtttttt ggggtaggat aagagctgca cacaatctgg
1177681 gtgatactga caaaagtggg gtgttcattt gtcaacaggg cttcaagtca ttagtagatt
1177741 tatagcattt acatttccta tcatgttgag aagtaaaaga gtcatcaacc atgatccatt
1177801 tcccattttt tacaactgcc ttgtcgtagc gcagcaaggg ttgaaggccc acaggctaaa

1177861 caaccatcat cagcttcgtg tggcgttcct ctactatgaa taatggctgg gggagcagga
1177921 ggtgcagcag gaggtgcagc agttccacat ctgcagacaa gctctctctg ctggatgcct
1177981 ccgttcccaa agtccaaccg ctcctgcagt attccccagc agtattgcac cagcgcagct
1178041 catccgcctg cttcccagga gtctttccac aattctttcc ccccagggaa tcagccatat
1178101 gggccttggt ggttagccag tgttgttacc tttccacatc cttcctcccc ccttttctct
1178161 attcatttct ttttttcagt gagaaatgcc ttgtactttt tccccccact actctaatct
1178221 aaacaccctc cctctttctt ctccttccac tcagacaaag cccagggggg cttgttcata
1178281 ttctctcttc ttattgtgtt tcatctttta ccttatgaca cagaactatc ccctcagagt
1178341 atccagcttg cagccacgga tcgggagaaa aggcagggag ctggagagaa cgcatggtta
1178401 accccatttt attctgtcta ttcaaaagtt ccctgacaag gctccgctgc tcgcatcaga
1178461 taaaagaaaa tcattcacag tgagtggaaa atacgggtga gcagaggcag accattttct
1178521 gcaaaagtgt gcaacagaaa gtggagttca tgatgaagca acatgcaaac aatggctttt
1178581 gaagaagaaa gcgtgtgcgt gtgtatgctg ggcataaaac taactgccaa aatggtatag
1178641 gtcatgtgat aaccagcatg gatctgtgtt tattgaaact ttctccaatc cggatctttg
1178701 tgcgactttg acctttgctc ttccaaggat caaggccaag gggcttgaca gcctaggatg
1178761 ttacgcaaca ttgtgttata agtggcaggg tacattctga actatggagg taattcacat
1178821 tatgtggggg aatcagctgt ttggcagggg tctgcgctcc gggagcttat agcttatagt
1178881 gtatagtgta ttttaggtga aacaaatact aacatgcact ttaatactac gtgtctcaat
1178941 ggcaagaaaa gcaccatagt ctgggttatg catcctctcc aaaaccgctc tggcaatgaa
1179001 ctcatctctc gctctggcta taatttcatg tctgcctcta tatttcacac tcaagcatac
1179061 acacctcctg tgtttctcaa gcacgtgcac cccccccccc cccccctcag tcacccaaac
1179121 cacctcattt ctgcagccct ccctcctctc caactctcac tctgtgcgta attcaatcat
1179181 cccaaggaga tggatatcca tcccccagga gcgcttatgc tctccatttc ctaaagcgct
1179241 tgatcagatt aattgattgt ccccctccct gcctttgtgc cccttctgcc gactcagtct
1179301 gaggacggcg gcggagggag gggtgcggaa atcaggtttg cattgatatt tttataaaat
1179361 ggcttttcct tctgccccca gacagaccct gtaaaaggac aaatgggagt agataaaaca
1179421 catgatctgg gagactgggg agggcgtggc atcgccctgc agaagcagac agcaggaatt

1179481 tacaaaaagg acaacaaccc gaagcccaca ggagaggcag cacagaaccg
gagctccagg
1179541 gacgagacaa agcatatagg tgatcaatgg aattacataa acaaggatgt
ttaggaggca
1179601 gacagtacac gtgtgcgtgc atgtatccct aacgtgtttg gctgctggcc
tgagcgcagc
1179661 agagggtaaa gatgggagag tcaaagggag gcccgcaggg aatgcaggaa
gacctggact
1179721 catccattat aagtgagagc caagcagctg ccatgtctcc tgattactgg
ttccctctgg
1179781 atccctctct cgctccatct ctctatcttt ttcttcctct ccctcttgta
gattatggta
1179841 cggttgcagg cctttggctt caacgttatg gcaggggcca aatacaaaca
cgcccacacg
1179901 ccttcgtgca cacagatgca ccgttcgagg cacgcacgcg agcacacaat
aacacgcaga
1179961 cggggattgg aaccagccca tctcttatct gtctgctgca catgttctcc
acacgtgtaa
1180021 agattttacc tgagcgcaac gctaaagccg aagcttttac ttcgggagag
ctaaattacc
1180081 tctcagatgc aacctttcc ttttcaacat gaccttgacc agcatccgcg
cctgcacgga
1180141 aacgctgcag agcatcactg aaccgtacag acgcaacacg gcggttttgg
tgggaaacac
1180201 attcctctaa gtgtcctcat ccttcactgc gaattaatgg agtaaaatgt
tttgatgccg
1180261 aacaaagctc cagcactttt cacgctgtca ggaaagtctt gcccgattta
gttttaatga
1180321 ggagcggcgt ctatcagaga aactgaattt aaatcactta ttcaaatgtg
caacgttcaa
1180381 tttcaatgac tgcattgttc aaccaaatgt caattaaatc attagaggaa
attataagag
1180441 ctcataattc atattcatat tcagatttca catttcgaat tcagatgttt
aacttgggtc
1180501 gtgcatccac ttaggaagaa agaagagtca gatgtgaatc agtatttatt
ttagaacagt
1180561 cagcgactgg ctcagcacga tggctcaaca taacatggca actttacaca
gttgccaata
1180621 aaaagaaatc tgaaagtgtt gcttatggtg atgcgtcaga gcaggttgct
gctcacagtg
1180681 gggcgtcgtc tctttcatct gcagaacaaa gcactttgca atcatttgca
cagtgactgc
1180741 tcagagtttc tttaaaagtc ctgtttcaaa tgcaacagtt ctgtcattta
atcaaggccg
1180801 tttaggctct ttctgtatcc actgtttgct gtctgatgac agttttacag
aagggaggcg
1180861 aagccctgtc tcaaataaac atacacttga atagagactc acagctacag
tatagcacaa
1180921 acgtacacac acatacacac actcgcacac acacactttc actttatgct
tgagctctca
1180981 gccaacaacc caaccgacac ataagttatt tttctttcca gaagcctctc
cagatttgta
1181041 tttgctgagc tctcagactg ggattgatcc tgggttactg tcttgaaccc
gtgccccagg

1181101 gcagctctgt gcaaacacac agcagctctt ctccttttac tccactcaaa actacaccct
1181161 gttacactgc gtctctaaag acacatgcag ggagtgacga ctgcctccac actggctaac
1181221 gctctaaaca aacccaacaa gactgaagaa tccagaagag ttgtaataaa tctgaacaat
1181281 aaacatttat gaataaatta ttcatagaca gagagagaga gagagaacat ttgcagtatt
1181341 gtcacctgca gacaaataac tcttgagaga cgccaactgg ttttccttct gtctttgaag
1181401 tgaggaaagg gggaggaaag ggtataattt gtttcattca tttatcattg cttacgctga
1181461 ataaatgaca ttatgagtgc ttgcaaggcg gtggagaagt tacaggatag ctggcagcac
1181521 agtggggaca agaagcagga aacatgctga ttggaaggct gcatccatcc tgcacccgca
1181581 gttagagtca gaagcaatgg tgtgtggaag cctgactgct acaacacaca tatagcacaa
1181641 tcacaattac ggttgctcct tgtgctgcaa ctgcccccag ttcttacgga gtatttgatt
1181701 gatggatgca atggtcggtg ctgtgagaca actggagttg gaaaggagcc tggggtgtgc
1181761 taatctgcac tgcctcctac aggatgacag cccacactgg accgctcctt cggggagaga
1181821 ctgacaccgc cctgcccctg ccaagctgat aatgatcagg gtcatttgtt tgcacctaag
1181881 gtctttggat tcacaaagat ttaatagaaa tcaaggacat ctcaaagatt tcttttcttt
1181941 tttttaaatg cagggtgtag ttatatatta cactagaggg ctattgacca ggtgatttta
1182001 tttacaaccc aaagaatgaa acactaggag atacacaagt gacatgatgc ctagaaacag
1182061 gcaaggactg atgggagaac acagccttta cacagcaaag agacatggac acaatgggac
1182121 acaggcggga caacaggtgg aatcggtaag gacgatggga ggaggcaaag gtggatcccg
1182181 atacaggcta cggttctttc atgatcaaag gaaacccctg agattaaact aatctctggc
1182241 tatgttgctg ccagatttcc attaaaatgc tcagatttca cttttcaaca gcatcctttg
1182301 tatcctaagt ctgagtatat tgcacttatt tgcatctacc tgaatccaaa ataaatatgt
1182361 atatttttta ataataaggt tgagggtaat ccacctgtga ctaaggctgc tgagtggaaa
1182421 gccagcacct gtaactgtga caactgggat ggagagtgta attaaaacaa aatcattaga
1182481 ctccagggaa ttttgccatt aatggttgtc tcatggtgct cagatgcagt gctatcattc
1182541 catcacatta cctaatggcg tgtgaagagc acgatcagca aacagcagca cacctacctt
1182601 ccaagcgatt tgctgcccct tggtgtcatg caacagagca accgggagct cccggcgttc
1182661 ataggacttg cggaagctgg ttggtccgga aggccgctcg cggtatgccc ctgcagccgg

1182721 ggggcccacc acctgttcgg acaagaaagg aaaacaactc tgaggagagg tgatgaacac
1182781 gagcccaaat ccacatgaag cttgttttct gaaatcagga ctgattttgt tttctcaaaa
1182841 actcttccgc tgtttttaaa aaaaaaaaaa gataatcaag ggtgacgtgt gtgtctgctc
1182901 ctcggtgttt ctagggaagg acaaaagcac agacagtgtg cacttgtgtg tgtgcatgtc
1182961 ggtcctgcag ccagaggcgc tgccggaggc attgttagga tcatcttttg tctgcctcca
1183021 tattagacat gctattactg ggccaccaaa agccaatggg acttctagca catcagcctc
1183081 agtgtgagcc accaatgcta gtactgttac tgtggtagct attacacaga ttcatgaaag
1183141 aaaattaggc caaaaacatt ttggagaatg actaaaagcc tcactgagtt gggatgcatg
1183201 agggaattgg aggtatctga ttgaaaagat tagcatgttg tagtggacaa atggcaggct
1183261 gtcagtttcc ctgtctggtc ccctgcgcgt atctgacgcc atctgtgttg cgttaccaca
1183321 cgactggcag atgtgttgct gtcattaatt ctcagattac cgcccgctgg aagaagaatt
1183381 tagccgcggc ggcccgggga aatgcgcggg tgaaagtggg atgcattatc tttcgcgcgg
1183441 ctgttgcgag acttcaaaag atgtccgcgg tgcttgtgtg gctctgttct ctcgacaata
1183501 cgcggcggtg gctcggcttt tttaagttgc atcattcgct actgttgcct atttgttgca
1183561 taattcaaaa acaaacaaga aaagaatgca agaatactgg aatcagagat gtgtgttgct
1183621 tcaactgtca aagagtgatg gaacatggag gaaaagctag aggagaggag agagtcctca
1183681 gctgtaagcc atttcagcat agcaacttca aatggatcca tctaatgcat ttgttcaaca
1183741 taccaggaaa taagggaggg aactgtgtaa tattggatta atttggtttg taatcttccc
1183801 aaagcaagaa ttatgtgtga ctaataatgc ataaaaagac aataagaggc atcgtttttg
1183861 gactagagaa gtctgaaaat aatcatgcac ctgctactgc cagtcctctg ctttaacacg
1183921 gaaatagaat agctgttcct ttatttattt agaacctgcg acaccaccaa atgctccttt
1183981 gcattccctc tctttgctca ttcccatcac agcaggaaat gttttgcagc tacaggaaga
1184041 tgtaaagctc gccagactcc tgctacccca gcctgcagca gtgacaaagc caaaatatcc
1184101 aggtaactac gaggccggtc gagccgcgct gcacaaggag gttaaaaata gccccgactg
1184161 ggaagaagga gggagagaat ggacgcaggc tgcactgact ggctgagaag cttttgtgct
1184221 ggggaacgat gatgctgcag tcactcctgc accgtcttca tgcctgagat gctgcgaggt
1184281 tcatagattt tccctgtttt ccttccaatt atcatcgctg ccgggagaac tcagcggagc

1184341 gcgtggacaa aggaaacgcg tgcacaatgg tgtcatttca gcaaacagtg gaacgcagtt
1184401 ttccacgtga aaatctaaga gctgtagcgg gaagagaata acaaatgtta ccttcacaca
1184461 cgtctgatgt gcctttcatg gtgttccggt tgaaagcttg tgtattctcc aatcgctaat
1184521 ctacaacaat ctagctcggt agcattattt gtgtttttta caacactggc ataaacacaa
1184581 aaggacaatg gaaaagctga gaaaaggaag ctatataagg agacgcctct atggaggcca
1184641 taaagtgagt ctctgtgagg cgacaggctg cagaacagag gtggaggtgg tgtgaggcaa
1184701 taaagaaagg acgtgtctgc tttgtgtgtc cctggtgcac acgtcaggga gtcataacaa
1184761 ggcaatactg gtcagatgag ctccggggtg tcatttctct ccttcgactt cactctgcct
1184821 ctagatccgt ttgctccgta atgccgttgt ccactccaac tagccccgcc ataaacccag
1184881 ctgcaagaac cgcagacaca ataacacgtg gaaaagcgcc cattccggtg cacatacata
1184941 catgctgagc tccccattgg caatccctgc caggtttggc cttggtaaaa acgtcacggc
1185001 gaggaacaca gacgctcact ctattgttat tggcgtccca cttccaagcg ttctggtttg
1185061 actttgtcca aaggtggcag caaaatggat acagaaacac tgaagtgaga ctgaggtgag
1185121 acaaagataa aggcagaatg tctcagtaat ggccgcttta ctgcatcaca ggcgaatgtg
1185181 cctcctagct tctaatattt gcatttctgt caattaaaag gttggtaaaa tacaactttt
1185241 gcacttcctg agggaggaag caagacggca ggggagtcag agaggacaga atagactgag
1185301 ggtctcttga taaagtcagc atgcacaaca agcaacagaa ttagcttttt atgcacagac
1185361 cagtttgtaa ccgtattgct ttttgctgcc gctgtgcaca ggagcaggag aggcagcaag
1185421 attacagagg tctgttttat tcccaggctg cattaacctc ggtgaaaaag acacccgtaa
1185481 aataaaggct gcgttcaagc tccaagggaa tctctgcaac ggtcaaattg ctccgacagg
1185541 ctatttttgc ttactcattg cctctgcagg gcaaaacact cactgatagg aggctgatat
1185601 ctttcagatg aatatatctg caatatttgt gcagcacctc attaatttct tatgagcaaa
1185661 cagcaagagg tgcaatcagt ctgtggggga gtttcattat gtgtagaaat tagccctgtt
1185721 ctgataaaca aggttggatg agaacaaatg agcaaaaaaa actcacaaac agggaaaaat
1185781 cccagcctga cggaaggtaa cggagacaga cacagtgtca caggcttcgg cacgaaccgc
1185841 agtctgcgca cgtgggcacg agtgcagaga tgtttgcaac aaaaaaacca ccacctacag
1185901 acagagacga cgccacacac ttcaagggcg aaccatgcgc ttattgaaac gagcacatgc

1185961 ggggatgatt tgaaaagcga ctgcattaaa atctgacacg ccgacgagcg caacaccgtg
1186021 tggttgaagc ccaactctgg gagtcgcgta aggaagtagc aaacggagac agggccaggg
1186081 acgggcagag agtgagatgt ctggagtgtt ttccatcctc tgcatgcggc acacttatca
1186141 cactggcagc agggcagcct gatgctcaca gatcccaatt ggctgattca tatctagtcc
1186201 tgagcgcctg aatgccgggg gcagcatatt tcaggtttta ctgctcgtca agtcaacaga
1186261 aagccatcaa ctttcaactt tgagatttac cacgatggct ctgggttaca ctgatttaaa
1186321 aggttaattt catccatgaa gtgccagttt gctctaagcc taattgctgt gagcatcttt
1186381 gaaaccttca tgctcttgtt ttttgtttgt tttctagaaa accagagctt tttatagctc
1186441 cagatgagct tttagaggtg cactggtaca gaccgtgtag acaaaccggg accgatgggt
1186501 cgggaggtga aagtgttggc gactgcacat ttaagtgtga aaatattgcg ggcaccttga
1186561 tttggcagca tcgagcccac ggggggactg agcaggagac agacacaccc agagcagcgg
1186621 gggggcatcg atttcaccac aaacctgcag cccatcacaa tgatgggaga caagagagca
1186681 ggtcagccac acacacacac acacacacac acacacacac acatacagga aaaggtgtgt
1186741 gagataacca aactaaacat agcaggagat actggtccca gctctggtat gggaacactg
1186801 tttaacacca gtgaatatta tctaggtgca ggcatgtgtg gggaaaagga accccagtcc
1186861 ctctctctta tcacaccctg tgacttgcta ttctgcccaa tcagctggta atgctatcag
1186921 ttaaaaatta agttgggggg gtttgtttac ggggactcag agcagagacc aaccaactgg
1186981 cagagtctgt gaatttgacc tttacctgat ttatactaag taaaatctga aggccgcatt
1187041 tttcttttct ttctttcttt ctttctttca ttattgacca gagtaattca aacatcttca
1187101 aacgagccct cgtttatcta agagaatctt cccactttca cctcttttag tattctcaca
1187161 taaaacctct ccttccacaa ttccccatga attcaattta aatattttg agaagcctgt
1187221 ctcagcagaa tgctgtcaac ctaatgtgat gatggccctt agactcctca ttaaatcgct
1187281 cagcatcaga gcattaacaa aagtgctaaa ttaaagcttg ggtaggccaa caaacgagag
1187341 acgaccttga gaactgccta tctgtatcca tcaaaggtct tgatgacctt ggtctattaa
1187401 catcagcgcg gccaaccctc caaacagaat cagcgtggct ctctagcacc aaacagacgg
1187461 ttgccagata taaagttgaa tatccagaaa tgaaagggag atgacggcct tcagaggacg
1187521 gacaatgccg tcacagacca gacgtgagct ttgaatgcaa gagcaaaagg ttggattgct

1187581 tttcgtctct ctgactcttt gttctccttc tgtcttggtg ggggggggct cagatcatag
1187641 aaccaggagg gaggcagaca gaagcagcga gaggttgatg ggcgggttga ctcatgaaca
1187701 ggctgcctgt tttcttttcc cacacgacca cgtgttggcc aaacagcccg ctgcagaatt
1187761 tgctctccgc tctttgaggt ttaatgatca aatgtctctg cctcctctcg cctgctgcag
1187821 gacggtcaca gcctccagcc gcctgcgact tggcgacatt gatgggaaga taacacaaca
1187881 gaattccgaa aggaagttcc agactttaac attgtaaaag gtaaagcagt tacgtttgaa
1187941 gaacatggtc ctgaaacatg tttgaacgtg tctttttaa ttgaattgtt ttgtagaagc
1188001 ccaaacgtcc tgcaggaccc agtaaacatc ttataacctg gtgcgttgtc tacgctgagc
1188061 actcttttgt atcacaggtt tgcatgctag cattcttgca aggtcacact tgcatcattg
1188121 tccttgacat ataacagaca tacagggcag ttaattttcc agtctctgtg ctggtcagca
1188181 aatttcaaaa ggtcagacta gagctactgc attgatagtg gttttaaaag gtttaaaaaa
1188241 aaaaaaaaag gagcagggaa aagcaggcct tattggtagg gaataacatt gttgtcaaag
1188301 ccattgattt tcctgtgctg ttcctattat gtggatgtaa gcagtatgtg agccggcaca
1188361 cgtgcccctg tggaaaggtg gatgggacac actaactgag gtggattata aaaacgaggg
1188421 cctcgccgct aacaaggaca agtcagtgtc aaaggtgata atggcgggga gtcgctgatc
1188481 aaatgccaca tcagcatgta cagacagtat ggacgcaaag gcagcgtttc accggaagaa
1188541 caggaacatt tggaggcggg cgcccataaa accagaacta cttgttgtga aacgcctcca
1188601 gaaagcagga aaatcaaaat gccttcacat gtgtaatatt aaaataatcc ttttaccagc
1188661 tctccgttaa aacatctgtt aatcatgcag catcagctga cagccagcgc attatgcaaa
1188721 ttggttttat agtcattcct aatcattaac agcaaaagtg gcatgtaata gattaattaa
1188781 atgacgcctt taatgtgtct gaactgaagt gggtgtcagt ccagccaata ttccggtatc
1188841 cctgattctg aagccaggac aggagttgcg agtctggccg tcctctgcag cggctatcac
1188901 atgacatggg ggctaattaa cttccgcgca gaaaatagaa ccttccaaga ggagcaataa
1188961 acattcccca ttaaaagcca cctaattata attcaggcta ggagcccttg aaggagacca
1189021 caacaataga gggtagtata cagtaattgt caagccagct cagaggaggt cacacgtaat
1189081 catctgagca cttcctctaa ggctgccgcc acgcgacgcc gctgaaatat gtctccatat
1189141 ctcccggaaa aaaatccagc gggtgttttc ctccgccgca aaggtcacag acgtttcggc

1189201 agcggcgcac ttcgcatagc gcgccgccag ctacagccaa atctatctgc cggtgtgagg
1189261 aggcaccgag caagtctctc tgccaaacgt gtacacacaa tcacacgtgc cctgttgtgg
1189321 cgagatcaaa gctggggaag gatttcacgg acatccaagc aaaatattaa acatgtgttt
1189381 ctatgtaggt cagctgttgg tgtaaggcct ttaaagtaaa gagccaggca gccagaatca
1189441 acaaagttcc tcagaagatg tccgagtcag cctgtgcttc atccactcgg ggcctttagg
1189501 ctgcgtggtg gatccatagt actggaaacc tcggcgagcc tccggagacc gctgccaatt
1189561 gaacctgttt tactgggact cggttccacc ttgaattaca cagctgctta ccaggatggg
1189621 ctacaggctt ctggtgttgg tgggaccagc gtgcgctgca gagggaactg gaaggcgggt
1189681 tgatttagcg cccacagagg tttattttct tcatttccac aaagaattcc aaggctgagg
1189741 cagaaaggct gagttattat acagcacggc ttaggaaact cactctgggt aggtgagctt
1189801 cacactcgac acgcactcaa cgccccagac ggcctcatgc acaatttatg tatcatgcag
1189861 ggaacattat gaggtcctct gtcaggtaaa tttgttcggc agcgatgttt ggagtgattt
1189921 tcactgccag ggaaaatatc aaatagactg attcatctcc ataacacttt aaagtattgg
1189981 tcaacaacgg cctgcaatgc acagaactgc cagtgggttt atttcaacgc cagccttttt
1190041 atctttctag tgttctttgg ggtttttcct gcactgcact ttagaatagc cggaaaagag
1190101 aggttttcac agcctaatga acacgcatca agactgctgg aaaaacatgt gataactctc
1190161 cagcaaattg aattcaagac gtcatattaa gcaggcttgc gtttgcaatt aaaagtgaga
1190221 ttgtgtctaa tgaggttgta ctcatatgta gactcattta aggcctaatc aaggatcaat
1190281 tctgcgccgt ctattgttta atttgatcag atggcttcat ttgttagatt agcatctgct
1190341 cggatgcaaa acacacctga caacaaagtg acacggaggg aatacgaaaa tccaataaat
1190401 ccaacacatc cgtgtccttt tcctgctctt tatttgctac tgattgttct atcggtgatg
1190461 tatttctgcc tggtcaaata cggagcgcgc agcagataaa agcagcagag agtataatgg
1190521 tgctccgcgg ggaggagtgg aactagatgt gggcttctcc gcctgtccct gtgggtgagg
1190581 aaaggtgttg tcaggttgtg ccgaggaccg cggcggtaaa cggatctctc ctgtccaaca
1190641 gtccggggca ggaaacagga ctgttaacac cacagtgcat gctgggaaat gctgcagttt
1190701 ctctgataaa tggcaacaca gtcaacaaac atttatgaaa ttccaatttt gccacattta
1190761 cgggtttgtg gagctgaaac caactctaac ccaagtattc cttcactgtg acgttgacat

1190821 ttctgtacat tttcccatca taacccttta cgatgatact ctgggtgcag agcacatttt
1190881 ttgtttttca atttaatgtt tgggaattaa ttcttagggg gcggtaaatg tggtttaagt
1190941 cgcaggggcc tgtgcaattg attcgatata tacgactgct aatacttgag agcgtaataa
1191001 cgctatgctt caagtccccg taccctttcc aggcttgaaa tggccttgtc gttacctaca
1191061 aatcacagcg ctcagcaaag ctgccctcac tggcgtgata tgtcagtgac cacaggggag
1191121 gtgggggggg acagaaggta acagacacga gcaagcctgt gaaaaatgta atggtttttg
1191181 ttaaagattt agccagcgct gtggcggcgg tggagcatca ttcatggatc agatgcttcg
1191241 gatcctctgg ggagatggtg aatattgaag ggctttgcag gagccttcct tttcgaaggc
1191301 tcttgacagg agatttgttt atgaccttca gcaaaggagg aggaatacat gatggttctt
1191361 tttttgagtc gcaagacaat ccctgccacg ggggtggggg gggggggggg gggaacccgc
1191421 acacagtttc aggtttacaa atttactgtt gtcatcagag ctgtgcagcc gcctctattc
1191481 atcatacagg cccgtttatt tgtgggctgg gatgtaataa ggccgtctta gctcagataa
1191541 caagaaaagg acccatcaaa gcagccaaaa aatggaaagt caacacttga gaagcaaaga
1191601 agacgtaaga gtctgactgc agacgtttca atccatccaa aagagaattg ctgactgtga
1191661 aggcacaagc ctcctgtcac accatatccc cctaaacatg gcattttctt tactatatct
1191721 gacatatagg atatataccc tctgagctgg ctgtaacacc agccatgatc tgtttatatg
1191781 catctggggc acaaatacac acaagtacag acaacagcaa agagttgtgt tgcttttgtg
1191841 ttgcacaaag ggtagtgttt aaaagtgaca cgcaagagaa acacagctga tttaaacgtt
1191901 ctgggagaga aacactcacc accgagttct tcatggcggc cttcacggtg aagccgctcg
1191961 ccgtcagcat cgtcttcctc ttcagcatgt cgacaagttt gaactgtttt aaaaagccgt
1192021 caataatgtc ccctttctgg agagcgtgac gcacttgctt ctacggtgga agcttgagtt
1192081 tcatcgccgt ggagacgcgt gtttccccgt tgccaaggtg atgccatgac gctgctgaaa
1192141 acggcgaaga gtgtcgtgat tggtatcggc gtaggactgg ggtctggaac tagaattaag
1192201 aaaaagtaat gtcgaaaaaa atgttttaaa aaaagaaaag aattaaatgc tcatctatta
1192261 tatgcctttc caaaggtttc catgccttat tgtgtctatc attttaaatt tgtgagcata
1192321 aaatcttacc atcagggcaa aaggatgatc atatagggag aatagattct aacacttgat
1192381 agtatcacat aaaaccaaaa atcttgtttt gtttgccgct agttcaacaa tcaagaatca

1192441 acaaaaaaat gatgttttaa tggttaatta aacataaaga aatgaaaaat gtacataccc
1192501 actgtagtga gttagcaccc cgagtttgat tgaggtgact gttgcaaacg gttgcatcta
1192561 cactgattgc taatgttggt aattgtgcag ccaccagact tggataacta atacgcacca
1192621 agcactcttt tatgcgcgca cagctccaga tccatcagac agaggatcgg ttgcaccctc
1192681 agctcatctt tatactcctg cagcagaccc ctgttcagac ggaaatactt aatgatgctc
1192741 aatttcaggg agtccaagga tttcgcagtc aataagccgg tcggagtctg gctccagctt
1192801 tgaatcactc caatttaggc atgcgacgtt gttgattttc caatctttta ccccctctct
1192861 tttttcttga gtgaactggt gtaactgtta ttaatcctca ctgcagaggc aatttctcta
1192921 aaagccccca tttgtttttt atggctcctt tgagagaggc aggtatcgca gccttgcagg
1192981 gagactgtaa acaacgtata tgtagggaag ttatgtccct gtcagcatcc aatgctgctc
1193041 cctccctctc tctgcttcct ctgagaggat tttaaaaaag gaaaggggtg tttgcacgga
1193101 gctgactgaa caaactggag tgtcagcagg aattccacga ttgttcaaac ggccctgcgc
1193161 tatctgtgac ggcgcagtgt ggaaggatgg aggcccgtcc ttgtgattcg tggaagctaa
1193221 aattgccaag ctaacgtggg gaacatgaaa taaatctttc aagccctgct gttctgctga
1193281 ctcctccgtg attatactat gggtgtgaat atctgggtcc atcaggctgc cttcaaggac
1193341 acgtgtgcaa attaatttgc agagtgaaga ttaattagca ttttctgaca ccaatgataa
1193401 actgtcgatg agcaaccggt ttggtccaaa agggacaaaa aaacatggag tgggtaaaat
1193461 aaacagcttc aaaatggcaa catagtaata caagaggccc tcatggattt ctgtcccatt
1193521 ttttcacctc tgaattgtgt cttcttcggg ttagtgtcac tctttcatga gcagctcatt
1193581 tgttgggcca ccttcggggt tgtgtgacct ctgtctacac tgagggaaaa taacaattac
1193641 cattagagca caaactggga gctaagaccc agaggggacc ggcagaagaa caacagcaga
1193701 gggaaaagac ggcagctacc tttctggaca ctatccccaa ttattatgaa accagagaaa
1193761 tggccattag atgggaagga gagctgtcag tgggttggtg ggttttgccg ggttgtgaac
1193821 gtggcaacgc tgtagggtac aaaatccatg gcaacccatg tagattgcaa tcaaggtggc
1193881 tacacaggca gcctatagga aaggcattaa tagataaaaa ataactccat cttggccctg
1193941 ttataacttc cattcattgt tttatgctaa tgctacgtaa attctctttt gtagagcgat
1194001 ttgagaaaac atgtgggaac accatctggt gctcctcaga tgagatcata acacggcagg

1194061 tactggagga taatccgagg aaacgtcccc agatgccatg tgtaagtgtt gttcggttag
1194121 ccaaatttga aatccatcgg cggagacgtt cagtcactga aggatcagaa atttactctg
1194181 acaagcgtca atatttgtgc acatcttgct gtgctaaagg tggatggata aaatgttccc
1194241 tctaacagaa gcagtcagga aaccaggaga ctttcacagt tttcaaactg cacccctgtg
1194301 gaaatcagtg aatctcggag cattttacaa tgacagtcga taaaatgcac ggctgttttg
1194361 cattccagtg tattgtaacc attgttgctg agttcacaca tgtaaattat ccctgttgtg
1194421 atttcctaat acaaacaaaa gaacaaggag ggccctacag atgcttcaga aatctgatgt
1194481 gtcattacat acattgtaat catgctataa ttatgggtgt aactgccctg gaggaatttc
1194541 accgtcagtt accgtggtta gccacttgta attattaata atagtaacac aagaatttca
1194601 gagcagaact gaatcacaaa ctacacaaga aaatgttgtt agtgatctga aacaggagca
1194661 agaaaactgg gacttgcaat taaaatcgct gtattaacac tcttacttta tgaatatgtg
1194721 tttctgtaat tatggagtta ctatgaagac tttgctttta cgtgcatgaa cgagctcgtg
1194781 taaaaattgg cctctgtgtg cctgtatgca ccccccactc ctgaacgtgc tcctgaggcc
1194841 ccctacaggg tcttcccccc tggtcctgcg atgctgtttg cttcacgagt ggcgccaact
1194901 ctggagacgt gttatcccaa ggagaagtag atgatttgct ggatgctctg atagatctga
1194961 cgactcagat ctcttcaaat ggcagactgg tcagcagcaa tacctgatat ggtcccagag
1195021 cggcgagacc tcgcacggaa aagccacagc gtatcgatct tcatgcatta tagaagcagc
1195081 gctgatattg ggaggcacga cttgctcttt gtgtgtccat gcgcaccaac atgcccactg
1195141 gaccgaagca gagacaatca gacgcgggtc cagatgataa atgtacagat gatatatctt
1195201 atcatggctt gattgtttac acgattgtat ttgttattgc ccctttatgt gtagcgtgtg
1195261 cgtctcgctt aatcattcag ggtaaatagt accccgtgaa tgcagagtgc ctccaaccat
1195321 gaagcccccc gccccccccc acagcagctc tggaaacacc taactctcca aagaatcgag
1195381 cttccccttc acccgtcggt caatgttaca cgaccctgaa tgtgccaaca gtgacaccag
1195441 tgatcccatc cgggacacag ccggcctcct ttgtgcacaa tcctcctgtg gtcatggtgt
1195501 cagggctggc gaggaagcgg agagggtgag gggggtaatg gcgctttcgg attcacggcg
1195561 tttgttatga gagctctgcc gagagcccgt cagcgatcaa aagagcaact tggtgtgacg
1195621 aggaggcagg ctgggaccag ccgcaaggac gaggacgaca gtaaggacgt caggctgacg

1195681 tggaaacact cacaaattct tgaaaaagaa tggaaaaatg tcataaagcc ataattcaca
1195741 cactatgtgg acaagaatca caatttagtg gtgataaaat acagtccaac atcgcaaaac
1195801 aaaacgcatc gaagctgatt tccacattac tgtgtattac tgcatgattc aagcataaca
1195861 ggaaaaatgt caagcagctt ttctgatgtt aatttgccct tttatttatg ccttttggac
1195921 attcaattaa aagacggagg aaagcagctc aaccctgtca gagataccaa tctaaaaaca
1195981 ctcctgtgtg ctaaaataac actttccaca cataaattac attttgaaca tcggttctag
1196041 tcgtctgtaa ttccagtgtc tgaattcccc tcttcctcgc agaatcaaag acaaatgcaa
1196101 acaacatccc cgcagctaca aactgtttat ggcagggaaa atgcaatttg ctttcattcc
1196161 actggaaaaa aaagaaaaaa aggggggggg gggcgctcgt tcacctgact gattctcatc
1196221 tgctctgcgc gtaaaacatt caaatcaaga gagacaggca tttataaagc caatacaaca
1196281 gagcgggttc ccctggtacc aggggtgctg tcaggtacag gcagagtgag ggagggagag
1196341 aaaggacaag agggctgttt aggagttttc ttttaaccct aagagcagag aagtcatctc
1196401 agctcacatt ccagctgcct cctttcacct gcagtgtgca gctagcctcc ggggtcttca
1196461 ggagcccccg gggtcttcag gagcctccgg ggtcttcagg agcctccgga gtcttcagga
1196521 gcccccggag tcttcaggag cccccggagt ctttgggggg cttcagagtc ttcaggggcc
1196581 ttcggagtct ttgggggcct ttggagtctt caggagcctt caggagtctt tgggagcctt
1196641 cggagtcttc gggagaattc ggagtcttcg ggagcctccg gagtcttcgg gggccttcgg
1196701 agtcttcagg agcctccgga gtcttcgggg gccttcggag tcttcgggag ccttcagagt
1196761 cttcgggggt ctctagagtc ttcgggggcc ttcggagtct tcaggagcct tcagagtctt
1196821 caggagtctt caggagcctt cagagtcttc gggggccttc ggagtcttca ggagtcttca
1196881 ggagccttcg gagtcttcag gagccttcgg agtcttcggg agccttcgga gtcttcagag
1196941 tcttcggggg ccttcggagt ctttgggggc cttcgcagtc ttcaggagcc ttcaggagtc
1197001 tttgggagcc ttcggagtct tcgggagaat tcggagtctt cgggagcctc cggagtcttc
1197061 gggggccttc ggagtcttca ggagccttcg gagtcttcgg gggccttcgg agtcttcagg
1197121 agccttcgga gtcttcggga gcctccggag tcttcggggg ccttcggagt cttcaggagc
1197181 cttcggagtc ttcaggagtc ttcaggagcc ttcggagtct ttgggggcct tcggagtctt
1197241 cgggagcctt cggagtcttc gggggccttc ggagtcttca ggagccttcg gagtcttcgg

1197301 gggccttcgg agtcttcctc ctcctgacat cttggctgca atagttgcag catttgccgg
1197361 ctgtattttt attcacgtca gccctcatga acagatggcg tttacgtttg taatattatt
1197421 aacacttcta agatcctgga ggggtaggtg ggtgaagggg tggggggggg gttacaggga
1197481 tgagcagacg cgcaggtgaa acacacacgg ttcccctaat tgtgctaaag gcagcgatgc
1197541 taattaggtg tttgtatgta aattatgtaa gaatatttgc tccagaatgg gctttcctgt
1197601 ggtgcaagtt gttttttct ccaaagtgtc agtgatttta cctgcttatc gggtgcatcc
1197661 acatgtcaga aacacatcac acataattca ttctagtaaa ttggatttaa taattccatt
1197721 ttcatgcatg catgatggag taattacact tgattttgtg ctttttgaaa tatcataagc
1197781 aataaaaaaa aaaactagga aaatgcaaag caaatggctt attgacagac tttgggggta
1197841 attacagaaa caaatggaaa taaaatgacc ctaagtgcat gttattaaac acaaatccaa
1197901 ctgaagagtc aaaacctgct tctgaagaga agtttttcc catctgcaaa tgtgaattca
1197961 ctctccctga aagagtgttt gaaatatata tatgacagga tatctgactg gtgctgcaaa
1198021 tgcactctcc tgttagatgg tgggagaggt gttggagagg tggaatgacg tgaaatgact
1198081 ggaaagcacc aggaaggaga ggtggcgtga gctgaaagag aaggagaaaa ctgagagcag
1198141 gggagcagga gcaggctgtt acgttccccc ggtgttggaa caggaaccta ccttttatag
1198201 cctcgggggc agaagtgcat atttgtgttt ctgacttctt taatgtctaa gaaaactaaa
1198261 gggatttgtg tgtgtgcatg tgtgtgtgtg tgtgtgtatt tgttacatac tgagtaccaa
1198321 tatactcatc cgagtgacgg catttttgca aagtcggtcc attttagcac aacttcaaag
1198381 tattgtttga gggctaagac atggcttcaa ggatgatgtt agcattgggc taaggttatg
1198441 gttgcggtta gggctttagt tgggatggtt agggttaggg tagggagctg ggccatttat
1198501 gatgtgccta tatgtcccca caaagataga aatatctgtg cgtgcatatg cgtgcatgtg
1198561 tgtgtgtgtg tgtctgtgtg tgtctgtgtc tgtgtgtctg tgtctgtgtg tctgtgtgtg
1198621 tgtttcctgt gaaagaagat ggggggtaaa gcagacaatg ttctcaggca tacttcgaca
1198681 tgtcactctg tatttgtcag aacggggggg aagcacaccc ccggtctctg gcgtcctgac
1198741 cctgcagctg ggtagtgcca tctttgtgta ctgccacatc tctgggatta tggggccgtg
1198801 gattactgac cctcagcaac cccactggtg actactagag cgcccagcca ctacctcctg
1198861 ctcatcgcga acagactgaa cttttgcccg taccatgggg aggcctgata gtgcatgtgc

1198921 acatctgcac gtaaggacat attgcaagat aagattacaa ggtacccctc
cattcaacag
1198981 caggatgaaa tctgagtgca cggctgcgca aacgcactga ggatctctgc
aaaacagctt
1199041 ccaagcaatc ctccagaacc agaaaaggtc attagtctct gaaaggagct
gattgtaggc
1199101 tggattcaag cgaatctttt tttgtctcta ttcagtccct aacatgtgca
ggaggttttc
1199161 tccggttgcc gtggagacag tgaaacgggt gtctgctcct ggatcagttt
aagaaaaagg
1199221 atacattgta aaaaaagaaa acagcattta tggacacgct ggggtttctt
ctccttttgt
1199281 acattaatca tagttggctt tactggcaga cttggctttc acggcttctt
tcatgtgctg
1199341 catttttaaa aagcataatg tacaacctgg tttttttttt tttaaaagca
ctatatttca
1199401 ttcataaaaa gccgtaaatt cctcccctgt attgtccaac ttcactgtct
ttaaacacat
1199461 catatgtcca ctgcaccatg tctttataag cagcataata gcaacatttt
gctataaaat
1199521 agtaataatt atactactac tcctactact acttctacta ctactactac
taataataat
1199581 aataataata ataataataa taacaataat aataatcaag ttttacagaa
aatctcaact
1199641 tgccacccag tttgtgagct attttccttg ttacgattta atctaacatt
actgttgttg
1199701 ttccaggagg agagagaata atacaacgaa accaaagggc aagaaagaaa
aactacaacc
1199761 ctaaataaat gctacatgac aaaataaagt gcagttttgt ggcctctgct
gtgagttgtg
1199821 gcagctggat cgactggtgt tttgctgggc tcacctctgg gcactgccca
gccaaagcaa
1199881 ttaaaaacaa acctctgcag ttcccgcctg tgtgacacga gcagaagttg
accgattctt
1199941 tctcagtttt aacaagagaa aaacgtcttt ctttggacca gaacctcgaa
agagcaccgc
1200001 agcgttgcag gtgagctttg ctgttaattc cagctgttgc tttgtgattt
gttaaaacat
1200061 ctcatgactt attacgaatg atttgctctc tttataaaat aaatgggctt
gccaatccca
1200121 cggccgcctc taaatgcttt tatacaccta aacgtggtcg agacgacctg
ctgaagttca
1200181 aaccagcaac aggatgggaa gaaagaggat tttaaagggg tttaagggac
tttggcctgt
1200241 tgttgctgcc agacgggctt gtctgagtag tttagaaact gctgatctgc
tgggatcttc
1200301 atgcacaacc atctctagct caactgtagc tcaaacaacc actcggtaca
acccaggaat
1200361 gaagaatagc atctctgaac actcagaaga ccacagcggt gccactcctg
gcagctaaaa
1200421 acaggaaggt gaggctacag gtcacacagg ctcactacaa ctggacatca
gggtctaggg
1200481 tcgagggtct agggtctagg gtctagggtc gagggtttag ggtctagggt
ctagggttta

1200541 gggcagaggt gtcaaactca aatacccagt gggccaaaat tcaaaactgg gacaaagtca
1200601 cgggccaaca ttgatattta ttgaaaaaaa tcttcttcca ggtataaaag ggaacctttt
1200661 gatatggacc caaactagtt ttgctcaaca actgaacatg gaacaagcaa agcttaacac
1200721 aatacaatat ataatttatt attgcacaca tgcaaaatca aaattcaaat aaaaaaacac
1200781 atcactggca ttcattgttt ctcttttaaa tttcaacagc aatcttttgc tattttaagt
1200841 taatgtaatg taaatgtaat gtcttttata gtgtcatctg ttgttgttct ccttgtaatg
1200901 cacattggct ccacatacaa gacagacagg tccgtctttt acatatctaa acagatattc
1200961 tgcctcctac ctgttcagaa aggtcctatt ttctgccttt cctttaatca tttttcagag
1201021 gggtactgcc agaattagca ttagcatata aggtccctat gaatatttcc tctctcttgg
1201081 tggttggcaa gccacaaatt ggtgcattac caccaccagc tgatgtagag tgtggatttt
1201141 cacaccaaaa caccagcaga acattctggt atttatagta ttagcacatg cgctttaagc
1201201 tataataccg gcgggccagc tctaatagtc aattggtatg gcttcgccgt ccaaatataa
1201261 ttacattaca ggccaaattt ggcccacggg ccagagtttg acacctatgg tttagggtgt
1201321 aggatctagg gtctagggtg tagggtgtag ggtgtagggt ccaggattta gggtttaggg
1201381 tctagggttt agggtttagg gtctagggtc tagggtttag ggtgtagggt ttagggtgta
1201441 gagtgtcggg cttagggtgt agggtttagg gtgtagggtc tagggtttag ggtgtaggat
1201501 ctagggttta aggtgtacgg tttagggtct aggatctagg gtttagaatc tagggtgtag
1201561 gatctagggt ttagagtcta gggtgtaggg tttagggtct aggatctagg gtttagagtc
1201621 tagggtgtag ggtttaaggt ttagggttta gagtctaggg tgtagggttt aatgtttagg
1201681 gtctagggtg tagggtttaa ggtttagggt ttagagtcta gggtgtaggg tttaaggttt
1201741 agggtctagg gtttagggtc gcatcataga cgtgcagcag ctgcctgaag ccatgtcaat
1201801 atggaccaaa acctctgagg aatgtttcca acaccttgtt gaacatttga catgaagaat
1201861 tctgaggaaa aagacaaagg caaaaggggg gccaacctta atgaagttgc aggggagggt
1201921 gactgtggta ggggagagtt tgtacagaat ataacaaatt gaatctttat tttcaaataa
1201981 agattttgca agactcggta tgcaaatcaa atcaaagaaa caggcaggtt ggactcccag
1202041 gactccaaac tgctctctaa aatggtgcat gtgtttctca gaacatgcac ccactcctct
1202101 cctccaggtc ggtgatttgc cttcttgtgt gtcactctct ggttttgaga ctcactcttc

1202161 ctcctctccc gctcctccgg gcattcatct tcctgtcagc ctgtcctgtc ccaccgtgtt
1202221 ttctccatag ccggcagaac tacaaatgct gttttgaggc cctggggccg gggggagaca
1202281 cctccatcac tatcatccac atcctcctac tgacagactc gacagtcttc ccttctcctt
1202341 tacaatttta ggatcagatc ctcggaattg tgcagagata agcctgcgca tgaaggcagt
1202401 tttgagtatc tgtcatttgt cccgtcaata attcttccag cgagccacaa tccaaattcc
1202461 cccaaggatg attgaattgt aatcgtagcc gtgactctta atgtgtccct ctgtttagca
1202521 cctggctgaa taattgatgg gtaaaacgat cacttctgaa gcttttagaa tcacgggtta
1202581 tttccatttc tgcagcatcc accttcaaag gtcgcgactt ctgcctcgtt tcagtggtag
1202641 agagagaaac atcacacgtc aggaagagca tgtaaagagg aagagcagcg aaaaacgatg
1202701 ctttttgtgc tgaatcggtg gctggtctcc gttaccccga tcagcgggta ttaggggggg
1202761 attagaggac cgcttcctgt ttcagattaa acaagcagtg ttcctgaggg ataaccagac
1202821 ccagcgggat ggagtgagtg ataaaaagtg gctggagggg aggacggtgc taaaattttc
1202881 tgctaaaaaa ttttgctaaa ataaaaaact gctaaaattt tcatgcaaat ccaacttttt
1202941 cacttaatct ggcatgcatg tgaatatgtc aacatgtggg caggtcctca gttatttaaa
1203001 acactagcag tacaacagaa agccattttc tgtacctaat caccaccacc actcgttccc
1203061 ttagcctttg caggcctcca aaagaagcct gtcagcggtg attgccgaca tttccattac
1203121 ttcatttagt tttgtcagca cgttacgaac aggagagaac tgtgccctca atcactgcaa
1203181 aaaaattaca ggcaagccgg cgcagagaaa taaataggca tgttagcatg cagaatgcgt
1203241 gactgcacat tctctgcagt gacatggagg atcgctattg gccatattaa cagcataaac
1203301 gtctcatttg tttatccagt gacttcacct cggaaggagg gagatttaat ggtttgctca
1203361 caatccaggt caggtgttgg aaatagaatg atgattctga tcaataacgc catccaatct
1203421 ccctgaaaac acggagctgt cattattgta aaacacatat acagtaaaaa tccttacggt
1203481 gtagtcaata gtgttaatgg gggaaattga tcgaaaggga atgtgtgttt gcctcaagag
1203541 caggtggggg gggggggggt tgtagatcat gtcatttata ttcttaaaac actgacgtcc
1203601 acacagaacg cagtttccaa accctccgat tgatgcagta cttttgatag gtgtctctca
1203661 ataatgcctg ctaaaaatgc caattgacaa ttagccaaga aaccactatc cacatgccat
1203721 gtgactcaga gcctgttctc acacagtttg cctctctgag tctcttaaga tgacgcgaga

1203781 caacaacctg acgactgcaa gaactgagat tctagaaaaa aaactctgtc tcctgaagtg
1203841 gaaatgaacc caaatgctgt ggaaggtaac ctccatggat catttgtatg tggttccacc
1203901 atcagctcac ctttttctga ttatttgcct ccttcaggtg acacacaaag gaataacaac
1203961 aagaaagacc taaatagctt agctacactg gtaatgaaca ggcactgctg ctcgctatat
1204021 atagttcaac acacttagtt tagcaagtag tacattacaa acccatatag gaaaaataag
1204081 gttgtctttt cagtctccca ccaagctgaa tcatttgaag tgttagttgc aaatttagaa
1204141 atttccatcc tagaatctgg gacagacttc aagagacaga aaaatgagac accaactagc
1204201 aggtccctat gtgaatgtct taattgttaa aggtgagggt cctgttctca tctgtgtcta
1204261 cgatccttcc tttcctgcgt ggcacattta tctgactttg tcaggaaagt gcacagttct
1204321 aaaaacctgc agggccaatt tactaagaga cctgtgctaa aactctacct tcagcacccg
1204381 cgacatcaaa cggcgggcgt cgactcgtct ctcctcattt gtgtttgtct agaagtgctg
1204441 gcagcctggc ataacaagga gaatctgatc ctatgaggtg attgtgctct tccgtcagca
1204501 cacaaccacg ttttattgtg cttgagactg agtgctaaga accggcttca aaagagctct
1204561 ttcattttgc cacatctgta aatgatggca tctctcacca gccttccggt aacttttaaa
1204621 cacctccgca catttttttt cccttcattc agaatgaact gactcagaat agatggtgtt
1204681 catttttttt ggcagggtgg ggagtggggg aatgaggacg gcggtagcta ttactcaaaa
1204741 tcgcttccat cagtcgggtg agctctctgg ctcctcgccg tgacagctgg atgtgtgtgg
1204801 gcgacagtac aaacaaaggt gaatcgatgc gtttccccga ggaaaagaca aaaggagagg
1204861 ttgggaggga gacggggggg cgagagagaa ggaaagactg agagatgaag gtggcgacgc
1204921 ggcagggagt gtgacggtgt catgctagct ggttttgccc ccccccaccc cccccacccc
1204981 gtgccctagg gagatcatta gctcacaata tggagtctat caatcctcac agaatacccc
1205041 tctctgctct ggatatacac cctgtctgtc tgtctgtctg tctgtctgtc tgtctgtctg
1205101 tctgtctgtc tgtctgtctg tctgtcggtc tgtctgtctg tcggtcagtc ggtcggtcta
1205161 gctgtcagtc tgtctgtctg tctgtctgtc ggtcagtcgg tcggtctagc tgtcagtctg
1205221 tctgtcggtt tgtctgtctg tcggtcagtc ggtcggtcgg tcggtcggtc tggctggctg
1205281 gctgtctgtc tgtcggtcgg tcggtcggtc ggtctgtctg tctgtcgtcg gtcggtcggt
1205341 cgtctggctg tatgtatgtc tgtctgtctg tctgtctgtc ggtcggtcag tcggtcggtc

1205401 gtctggctgt ctgtctggct gtctgtctgt ctgtcggtcg gtcggtctgt ctgtcggttg
1205461 gtcagattat atgaaatgat tgatgtcaat taaaacccta gcagcagaga gtgtgacgca
1205521 tgttcatccc tcaggggtct ttgatggggg gggggggggg gggtcacaca aacaataaaa
1205581 aggtagcaaa cagatcatcc attgtgggaa tgtttagcat cagaaacctg aggctgtgca
1205641 gtgcaacaga caacctggca cacgagtggg tttgtacggt ggcgtgcggc taaagtcctc
1205701 ctctgagcaa ctgcggcaca tgcaaataac attagagatg ctttattggc cgtattaatg
1205761 cgtaattgta cctctctgtg ggcttcgtct gtgcatctga gaacctgtaa tctgcaggca
1205821 aactcgtaga tttggcgacc gcaacatccc gaagtctctg gctcgtcaaa tagcagagat
1205881 caggcttcaa gcggtgacac aatggttccc gctggcgcat cactgagcag ctgtttgttc
1205941 ctgaaacgct catcacgtaa agcgcgtggg ctagtgaggt agagtttagg ctgttaatta
1206001 ctccaaaaga agaagactcc actaatgttt tctcaaggtg gcgagaaaat aagctttgtt
1206061 gagactggga gacttcatac ggcagaaggc cagaactcct gggaagctct cctaaggccc
1206121 tccaagggac ctcctaaggc tttccaaagg ccctccaagg gccctccagg gccctccaag
1206181 ggccctccta aggccctcct aaagcgctcc aagggccctc ctaaggccct ccagggccct
1206241 ccaagggccc tcctaaggcc ctcctaaagc gctccaaggg ccctcctaag gccctccagg
1206301 gccctccaag ggccctccta aggccctcca gggccctcca agggccctcc taaggccctc
1206361 ctaaagcgct ccaagggccc tcctaaggcc ctccaagggc cctcctaagg ccctccaggg
1206421 ccctcctaag gccctccaag ggcatcgttc tacagctgtt tggtctttgc aaatcttctc
1206481 tgatcttctg tccaatgttt agcctaccat tttgcctcca gttgtcatcc atgtgcacgt
1206541 ctttatactt cctctccacc tcgaccagct gttccttcaa cttttgcggg gtggaattta
1206601 gatggcggcg agaagagagt gctcagtttt ccctgtgcag catcaccatt ttatggtgct
1206661 ttttgtctta tttaccctca ccccgacctc actaaagacc catgtttaaa gcctgaggtt
1206721 tagaggagga tggcacccat gaccctattg gtaaaaaggg ggatgaagcc caataacaaa
1206781 aggagggagg gaaagacagc gagggaggga gacacacacc ctgtgggagg taatcgctct
1206841 tcctgttctg gctatgcctg gccagccaag cggagctgcc acctcattgt gtccttctgt
1206901 accgaggctc tctctggtcc tacgaggagg gaggaagagg agggacaggg agggcgagga
1206961 agagcggggt aaagatccac catgacgcca acacatggat cctccatgag gattaatcca

1207021 acacagtaca aagatctggg tctgaagctg attggctgca ttggaggtgt gtgtgtgtgt
1207081 gtgtgtgtgt gtgtgtgtgt gtttgctaca cctggaacaa actactctta acagaggaca
1207141 acagagacaa gaggaagtgg actggactga aggggatgat gggaaaatta gattaaaaat
1207201 caaagcgagc gatttatggg ataatccgta ttcttctggc gaagaaaaat agaaaagaga
1207261 caatgaatct gaggtagagg tttgattaat tctgttatcc tgaacccaat cagcaatgtc
1207321 caacatcccc gtggtccctg acccaacctg tcctgtctac gtgtgtccta gaaacagggt
1207381 cgacagccaa cacgggtggt atttaacacg ttactatgta aaccggatgg tttccaaacc
1207441 aacaactttc actttctgat gggatttatg tggcatgttt ttagaccagg accaggctac
1207501 acgcttgggg ctgagattgg ggctggttcc ccacctctgt ggacacccca aaccctgttt
1207561 gatttctgta aaacagggtt tgaaactgac taatcgtcct gagtaaatga gcacaaatta
1207621 gcttaattta ttgtgaaaac atgtgacttc cgaaaatgtc acgttacaaa aaaaacccga
1207681 cagaggcggg gggggggaga cgaggggggc aaatgcgttt atttctcctt cggttcctcc
1207741 ggcacctgca ggtcaggacg gaggaaatga ggcaggcaaa tggaacacag actctggatg
1207801 cgtccacaga atatatttaa cacatctcat caagcccata aatataaatt ataacgatgc
1207861 agtgtatgga caacagtaaa tatgatgttt aatggctttg acctccagta aacccagcct
1207921 gcccccacaa atacgcctcg ttgatgaaca catctgccaa actttataaa cgtcggcacc
1207981 gtttcaccta taggcgtccg aaaactcgtt aacgttttga aagaaactct tctgggagcc
1208041 tcgtattgat ttcttattaa tagattgttt gtcgcaacaa atgatgtttt tttctttagg
1208101 cgccgaatat gcagagcggg gaggagaacg gccccgggcg ctgcgtctcg caccggggga
1208161 atatccgaat gtctttgttt gatgctaaat gacaccgggg ccatcgttag ccgctgctca
1208221 cagctcgtaa atgcatgggg ggggtgccgg ggttcgctcc cccggtgctt cctttctgat
1208281 caatatgcgt aactgtgtgt gtgcacacat gaatcatcta taataacaca acaacaaaca
1208341 cttgacggcc tctattatga tcacattgaa ttttttgtag aaatggctgc ttagtaacga
1208401 cgtcaaatag caacacgtat tccgtctgct gcacactatt gattattgat atcgatgttg
1208461 acgatacatc tgtagacctt ttcatgcaat tatgagagac tttgctaaca tgtacctaca
1208521 cgggcatgaa aatcgaccct tttcctatcg gctggtttca aggcatcgcg acaaccagca
1208581 gcatttttc ttttttttt ggttttctgt caaaatttca tttatatcac attaataaca

1208641 tccaccgtgt ggcaccaacc accacaacac ctggacaaag caaacttcat gagttaaatg
1208701 taaagaacaa gtgggcgtgg tttgcttttg gaccatacag gtaattttaa ttccatttta
1208761 tttaaaaatt ccagagcagg gaacagcatg agctggtcca ggctaaagtg ggggccaccc
1208821 ctgctcgtgg ctggctgacg aaaggacaca gcatactgta cgttcaaacg catcagtcat
1208881 ggtttgttgt cctggagacg agtcagtgag gtcaggggtc agcgtgcacc tatggagcaa
1208941 acggctgcag atcaatgcag agagagacga acagcgagac acaaagctaa agaaaaagac
1209001 tggtaatagc gcaatgaaca ttagcagtag ctatagcggt gatcagttta cgcaccagag
1209061 cgtgtccgga ctacagaaat aacggacttt taaatgtgct gcgtgcgcag ccaaccgata
1209121 ctaaatgtca ctgctacgct gatacgtcct cattgtgagt tagcttttag cattttatcc
1209181 tcctgcacgc atgtgctgca tgcacacgtg tgaagtcaga cattattgct gtttaagtgg
1209241 cttcatgctg cacgtccgcg ctcccctttt tattcatgct agctcgtctt tcaggctcct
1209301 catccctgtt tgtgcgttat tggggccgtc tccctgattc actcccttta tcgtcgtgtc
1209361 cgggcttatt tacagcagcc tgtaactcat cccagcagca gacagaccgt tcctccggcg
1209421 tgtgtgcgtg taaaactttt acaggtctcg cagactgcag ccgtcgaccg ggagggggt
1209481 agagacaggg atggggggg nnnnnnnnnn nnnnnnnnnn nnnnnnnnnn nnnnnnnnnn
1209541 nnnnnnnnnn gggggcattt acagcagctt gtaagcgccc tttcacgctt ctcagctcag
1209601 aatggaacaa tgtttacttt tgggaacgta acgcgttaaa gaaaggactc taaagctgct
1209661 gaagacacgt ctggagagtc acgggaagga cgcgtgagag gtaacgcaga taaggcgtga
1209721 atttattcaa aactcacctt ttatgcggca aaatcctatt caggagcttc gcgcgtcggc
1209781 atcattgggg aacaggagtg tgccggcggc ctgctcgatt tgttaggcca gtgagtgatt
1209841 attgtttatt gcctcaataa aaagcatccc gtcctccgcc ttcctacccc catctggagc
1209901 aatgacaact taaatattca gcgccgcgca gccgtgaaat aaaaacaaaa cgagcgggaa
1209961 aataaagcgt gtcgccggcg acctcccctt caggagacgc ttttcttttt ctttggagga
1210021 aaaactgccg cttgtctctt aaacaatgat gcacgagtgc taacgttgtt ttatttacct
1210081 gcgcgccgcg gctcgcgcgc gcccgcgcgc cgctacgcac tgaaacgcgt gcccagaaga
1210141 cacgacataa tgagataaac cgccacaatt tcgcatgcac aacagttgca ccaaagtgtc
1210201 ttctgaaagc cgcaccaatt aagctaacac aattaactcc aggcccgcaa aggcgctggg

1210261 agattttgca catcttatga ggatttgtag caggtcccag tcctcatttc acataatgct
1210321 tccaaccatc gtggagcacc tgtggagtat taggagggcc attggctctg attacattct
1210381 ccgtctccgc tgggcatcgg ggtttgttgt ctctgccaaa ctgcttttgc ttttgcggga
1210441 gcactttcat ctcagccgtc tctttgccag attcctctct tttctacccc cccccccca
1210501 aaannnnnnn nnnnnnnnnn nnnnnnnnnn nnnnnnnnnn nnnnnnnnnn nnnnnnnnnn
1210561 nnnnnnnnnn nnnnnnnnnn nnnnnnnnnn nnnnnnnnnn nnnnnnnnnn nnnnnnnnnn
1210621 nnnnnnnnnn nnnnnnnnnn nnnnnnnnnn nnnnnnnnnn nnnnnnnnnn nnnnnnnnnn
1210681 nnnnnnnnnn nnnnnnnnnn nnnnnnnnnn nnnnnnnnnn nnnnnnnnnn tttcaacccc
1210741 ttttgggctt taacgaacnc cccctgtagg cgatcgatac cngacaangg ggngaaatca
1210801 aaaatttttt tcgcccccaa aactngcncg gcgaattcaa tttggtcgtt aacattccag
1210861 tcgctttaat tagatgcctc agtagcaaat tgcaatttac atttttatag tttttcgagt
1210921 taattagttt gctcactcag ccagacccaa cttgtttatg ccccccacct ccatgttgcc
1210981 tccttttctg agtcttgcgt tgtgttctta agaagaaagg gacttaatgt tacaccattt
1211041 cctttctttc tttcaagcta ataaagcgct ctgctaattc agcgttcagt tgataggttc
1211101 gtgcactcca aacaagtttc ctgctcaaaa acacttttac cgatttaagt tataagaagc
1211161 tcaacctgca aaaaggccgt tatttcaaaa aaactcaaac gccaacttcg actctgcgct
1211221 caagaacgtg aaacaaaaat ggcggtaaaa tgtgagtgct gcatctctaa agaaaagcag
1211281 cgagatggta gaggtgaagg atggaacgga gggctggaca gagagaggaa gagcctgtcg
1211341 ctgcaacaat cagccaagca gacaggcgag tcacctctac tgtcactcac cgcccccccg
1211401 tgaccacggg actaaataaa acaaggcctt ggaggtcctc cgtgttgtct tcggttttat
1211461 ctcctcccac catcactctt catcttctga acagacagca ggcatttact cgcacggacc
1211521 gacgccgctg acagcacaga cactgtgtaa gcactgacct ccattacgcc acaattagac
1211581 gccacgtgtc ccccttcaag gtaggacgca actcgttgtc cactgctaag gtccctttca
1211641 agacttagtt ttatcagtga acatgttgaa gctattctcc gttgtcccta cacaggcaaa
1211701 gctgtgcatg ttggctatgt tagcatgtgc gcatgctagg cattaacgat caactctttc
1211761 ccacaatcca aagcatggcg ccatctggga ttcagactcc tatggaaggt cttctgggat
1211821 gtctctccca tccacagact ccctcattat ggatgaatct ggtgggaaac atgttttggg

1211881 agacccgtct ggctcccagc agttttcaca cagaatcctt gagagcgaat ttccctcttt
1211941 ccgagtttcc gaaagtgttt tttttaaagc cccttcttcg cacaacatct tcagaattct
1212001 ccttttgtct aattttagca ttttcagaag ctttgctttc tcttccaatt gttccagcct
1212061 tcacatggcc acgtaatttc gctaaaaggt ttaaccgaga caccgacgag cagcgggatc
1212121 tgtgcctggg aagtgaggag ggttagaggg gagagcgtgg agtcacgggg gcagatgtga
1212181 ggagagggcc tccgacctga tgttccagcc tctatctgct aattagccct cctcgctaac
1212241 cagatgttct taattaaatt tacatgcagc ggtgaataac tgcataaaaa gaagggttcc
1212301 atcaggaggc cacggaggag gcattcggcc gccaaggcgc agcgcttaaa gtacacagga
1212361 acataaaaag ctcgttaagc cagacagtaa aggggagaga ccccctggag ctctttaaac
1212421 accatcttga gctgtgccct gcatgcctgc acccacctcc atctctttac cctcctctgt
1212481 ctcccatctc ctgtctttcc agtggcactt tgggatccca ggtggagacg ctttgagtta
1212541 gctgcaggtt agctccctct gtcataccct gtaactaatt acttcctggc gcggctgaaa
1212601 gacagctaat tacacattac ttggtggaca cctcagaagg ctctatggtc atcagctgct
1212661 cgataatttg ccaggagcct ggccaaaaga ggataaaccc gctcggaaaa gggcattaac
1212721 gctgtcctgt cacttttaaa aaggcaaaca acggtttaat ttcaagctag agcagagcga
1212781 ctagcgagct gtgagctaat caaacaaagt ccagagggta gatgatcaaa gtaaatatca
1212841 aaggacttga cagccagagg tctgcctgct tttattttcc ataatgtctg acagacctcc
1212901 gcctctgttc tatagataac tagaacagag acgaaaaggc aggcggaggt ggtgtagcaa
1212961 acacctatca cagatgtggg gaatctggaa ttaatcattt taacatcttc tacatcatat
1213021 tacagtcatc tccacgagat gacgcgcatc ctcgcactta tttgatactt ttaaggtcac
1213081 ttttggggtt taatgggaaa taagttgaac cattgtcggg tattagctgg gcttttaccg
1213141 cccatgagtc aaaatgactg ctgtgaaaaa tgccccattt gttgctttaa cgactgcgtc
1213201 taatgctttt ataggccagc gcatgtaaaa gatttcctgg caggattact gcactttcct
1213261 tccatcgcag tctcatattt ttctgctggt ttgcaatgtt gtattcaatt tgtttgcgtc
1213321 ttttagccaa gagtaggcag cactcagcac atttagctag caagttagca atccagaggg
1213381 gtttatccat ccaagacaaa caccagtttt tgccgtgttg ttcttcagct aaagctcctg
1213441 tttacctttt tacatctggg tcaggatttg atgcagagct agagaacggt ctgtatcgtg

1213501 catctgcttt caatcccatc atctcggcgc gtcagcccga cttttgaaat ttcaattttt
1213561 cacttcacaa gtcttgtttt catcagacta atgtgatggt ttggttttta agttagttat
1213621 catcctgaat ctatgaacca gtatgaggga tatttttgta aatcacacac aaagctaccc
1213681 tctagtagct ttaaaattgc ctagcgactg tgctaagaat aagtaaacag cataacctga
1213741 cctagaaatt cgccacctga gagggtttta tgtccattag aagctctgtt gatttcatca
1213801 agagcacact gagctaaaat gctacacgtg tctgtaaaga tgtgatttca gtcacaaagg
1213861 tgtgcctgtt ggtctttggt tgttagcaag tatggtttta gtgtggatct ccttgggata
1213921 aaaacgggac tgatgacaac atcggggaca ttgcaatgaa gttatactag cataattagc
1213981 atcatggtgg agagccacat aatatagcac aaacacccag gcttcaacta cccacgatcc
1214041 aacacagtct caaatgcaaa acacacaccc acatgcaaat tatgtttgcg cacgcgggga
1214101 ggtaacaaac agctttccac ttttgccaga gtctgtcctt ggacttggtt gccgctgatc
1214161 aatgctattg ctactctaat actccccgtt agcacaacac cggaagaaga aacgcatttt
1214221 gatgttgacg tcctgagaat ttttgcttac tgtcggcaga gactcaagcc aagaacaagg
1214281 aaacagcgag agagtggcgt tgaaaggaca ccagataatt agcaattgcg attattcgcg
1214341 cctaaaagcc gcttcatgca aatgaacagc aatggagaga gacaagattt tttttttttt
1214401 tgattcccgt ccatcattgt ttgtcagtca tgttcgctta catgtgcgcc gaataatcat
1214461 cacctgcagc agccgcagtg tccctgcaat tagcgtgaaa taatgcctgg ctgcatgttt
1214521 taccacatat tcaattagag cgcctgttag ctgtcctccc accgggaaga tacacaacaa
1214581 taccaacaag agcaggaatc gtgcacggtg catgcgggcc ggtctggcgg ataaaagagg
1214641 agcatacatt tcaaaatggt actagcagag gacgcaggct tggtctgccg gtctccaaat
1214701 gtggtcataa attttcataa ctggtgggtg gatgtagctg gcagagaaag tgagcaaatg
1214761 cctccgatgc tgtgatgaat gtcggcgctt gggggaacgt ggatattcca gtgaggcagg
1214821 aatcgagcag aatatgtagg tataagatca ctcccattat ggttcatcgt gctaacctgt
1214881 tagcttgaca aaccagtaca acggtagtgc gctccttctt cccgaacatc accagcagcg
1214941 tgctagcttt agggcctttt ggttttgatg gaagcctgaa ggtttgtttc actgcacgag
1215001 gcagactgca cttcccccgc cctttttgtt ctcccttcaa aacgaagctg ggggaatatc
1215061 tcccgccagc agatggtgga agaagtgaac gcctcccgag gaacttcctc gcatggtcga

1215121 gcctgtttat tcacatcaca gggaggagaa gcgcgtcccc cccgcaatca gacaggaaaa
1215181 agcaaaaatc aaattcacct gttggcttcg gaacacagga gctctggttc agaaatcgat
1215241 ggcgagtcga tggcggccgc gggattatga gggggacgga tatcggtacc tactcatcca
1215301 cgtgtccgtc tacacgggga ggaatttgat taaaaggctc cttgattcat tctttcagtc
1215361 ctattgatct ctttatgtaa accattactg gagcttcaaa tgcataccac acacacacac
1215421 acacacacac acacacacac acacacacac acacacacgc atgcatacag agagaggaga
1215481 tctacaggtc atatctataa ataccagcct gagtcggact gagtgacggc cctatttcct
1215541 ctctctctct cgctgctcgc tcacgctctc ttacacacac acacacacac acacatagac
1215601 acacacacac tagacaaccc agacagctag tttgctgcaa atttaaaggc aataagctga
1215661 ataatggtga gcagcactgt cgctattcct ggacattagg tgctcggcca ccggcggaag
1215721 atttatctcc ccctctgaaa tttcatttga ggcaggaata ataaggcgta ggaaatgaaa
1215781 gttaatcacg agcccactcg cgcgttcttc ctctcctctc cccgtctatt ctccctctct
1215841 ccgtcttcga cgctggctgg gagggccgag ccgagccgag ccgagctgag gcgtatttta
1215901 tttagccggg agccttttgt cttggcccct gtgacctttt aaacacttca taaatcagca
1215961 acaagactcg cagttgtgat gcagaactaa ttacaatagc agggccttcc agaggggcca
1216021 ccaaacaaaa tggctcccca tcaaacctac ccggtaaagg tgtgcaggtt agagaaggtt
1216081 ggggatggat taaagatgct tctgtcattt ccaaaagcat ctcgacaatc gctaaaatct
1216141 gtacattgac cagtcagggt ccaaaccgcc aggccaagga tttcaaggat ttcaagggtt
1216201 tcaagggttt caaggcaacg tcagtcacga ccgctgcctg cttgagacgg tgtggaacga
1216261 aagtagcgct gaagaacagc aggagaaaga acgccgggct cctcgattag cctcgactac
1216321 aatctggaat tacaccacat taaagagaat agttattctt tgcgtttaaa aagtaaagtc
1216381 aaaaagatgg aataacgtat agattataat ctcatgtgga ggacagtgga aaatcctgtt
1216441 gttttccatc tgatgcttaa ccaggctgca gcggcgtcag cgccccctag aggccggagg
1216501 ccactgtggt aaatagagtg aaatcgaaat gaggctgata tttctataga aatatataca
1216561 aataacgaat ttaataccac tgcgctgtca tttataatgt gtgaaagcaa attctttaat
1216621 ctgagatctt actttcagga atgaattagt tgcatgacca tttctttatt atgttcctga
1216681 gtctgtactt cccaacagtt acagcacgct tgacttcgac ggcaccacag aatacataaa

1216741 taacaaatcg ctgaggaaaa actgagaaaa gagctcatca gttgaaggga gttttagctg
1216801 cctttcatat ctgtgcccct tcaacaacca acagagcagc taaatccaaa ttacctgtga
1216861 aaatgacccc aggatgcacc gctgcgtcct gtcgtctccc gtaaattatt taaatgcaac
1216921 acttgcagaa agcgttccta ttagcataga gcatgcagcg ctgtttatgg cggggaaccc
1216981 agtcattttt aaaaggcacg ggaacataaa accttcagct ttcatgcttg ttgagcgctg
1217041 acagccaaac gctggctccc ttttattaag atcaaaaggt ctccggcact tctgtgcgtg
1217101 tacatgtgag caaacacacc tttaaacaca ccacaaacac ataacgcctc agatggagtg
1217161 tgttcagaga ctgggatgca gccattaagc agctcctgat gaatccaatt ttccttgaat
1217221 aaaggaaagc agccgtctgt catcctctca gcgagtcact taacctggat cgtctgtgta
1217281 aacggccagc gggacggagg ggtattatct cagccgaagc ccaaagaaga ggggaaaact
1217341 ggtgggaaaa ccaagggaaa agcagctgtc cttcctacgt ttctgtgtgt gggcaaagag
1217401 aggagaacgg gaagacaaaa acaacctgtg tgatgatgtt tctgggtgct gatgtctcag
1217461 acctgtgccc ctggaatcga tggggtttgt cttgggttag ttccccttga aggtcagttt
1217521 gctttattgc ccttttcgac ctcaaggagg aggaaaaaca cacctccgga cattcaaatg
1217581 tgcaccgaga ctcgttccgt tgtgctaaaa ggatgcacct ctcactctcc tcccagaatc
1217641 aaatctctgt aatggcaatt aatggccttt tggtgcataa tgaagatggt ttccgtttca
1217701 ttaaaactgc ctctcagccg gcattagcgg gtgtgtcagg gaggatctga cgacaacgga
1217761 cgacggagca tcttcactca tttatgctcc tcactaagaa cataatagac tattcttata
1217821 gacacaaagg tgattcattt gtaacatatc caataaataa gatggaaaag atgaataagg
1217881 ttcattatta gcagcaggac tcatctgttc aagtcatcag tccacagaac tgcctgattt
1217941 gtcctgagat tctgtccaag aattgatgcg atgtggctcc atctcaaccc tgcggaggat
1218001 ctatttcaca ggtttgctcc tctttttccc gagcattcct catcctgccc gcctgttgct
1218061 ccacagtcca tgcgtctctt tttacctgtc acttgccccc ttcctccccc tttatctctc
1218121 ccctcccctg ggggccgggc ctcccgccat atggacgagc ttgtctgccg gcgggggcta
1218181 acctggcact accccgtcac tcggcgtgac cccgtggagc cgcggcgctg aggcgtgcat
1218241 catcctcctg ccagacgtgc agaggtgcac gacagctctc gcatcacggc cctgtcggca
1218301 tccatccgtc aatcaacgcc gccgcgcggg agctctcgcc acatgttgaa ataatccgaa

1218361 ataatgtcgc acggcgagag gccggtcgag ggccggagga atttggcgcg acgcctcagt
1218421 aggattttaa atcaagcggc ggctggctgg tttctgccca ccgccccccg tttccgcagg
1218481 tcgatgggtg gaattttgcc ccatttttga agagagatgc gaccggagct gctgcctaga
1218541 ggggagggag cgagggggag agggcacgga gggagagatg gaactgacag atggagaatc
1218601 cggcccttta aggcccgtct gctggtacca atctcatcct tgagtgagct gtcatttttc
1218661 ttaaaataat ttgaagcaga atctgccagt cagccatnnn nnnnnnnnnn nnnnnnnnnn
1218721 nnnnnnnnnn nnnnnnnnnn nnnnnnnnnn nnnnnnnnnn nnnnnnnnnn nnnnnnnnnn
1218781 nnnnnnnnnn nnnnnnnnnn nnnnnnnnnn nnnnnnnnnn nnnnnnnnnn nnnnnnnnnn
1218841 nnnnnnnnnn nnnnnnnnnn nnnnnnnnnn nnnnnnnnnn nnnnnnnnnn nnnnnnnnnn
1218901 nnnnnnnnnn nnnnnnnnnn nnnnnnnnnn nnnnnnnnnn nnnnnnnnnn nnnnnnnnnn
1218961 nnnnnnnnnn nnnnnnnnnn nnnnnnnnnn nnnnnnnnnn nnnnnnnnnn nnnnnnnnnn
1219021 nnnnnnnnnn nnnnnnnnnn nnnnnnnnnn nnnnnnnnnn nnnnnnnnnn nnnnnnnnnn
1219081 nnnnnnnnnn nnnnnnnnnn nnnnnnnnnn nnnnnnnnnn nnnnnnnnnn nnnnnnnnnn
1219141 nnnnnnnnnn nnnnnnnnnn nnnnnnnnnn nnnnnnnnnn nnnnnnnnnn nnnnnnnnnn
1219201 nnnnnnnnnn nnnnnnnnnn nnnnnnnnnn nnnnnnnnnn nnnnnnnnnn nnnnnnnnnn
1219261 nnnnnnnnnn nnnnnnnnnn nnnnnnnnnn nnnnnnnnnn nnnnnnnnnn nnnnnnnnnn
1219321 nnnnnnnnnn nnnnnnnnnn nnnnnnnnnn nnnnnnnnnn nnnnnnnnnn nnnnnnnnnn
1219381 nnnnnnnnnn nnnnnnnnnn nnnnnnnnnn nnnnnnnnnn nnnnnnnnnn nnnnnnnnnn
1219441 nnnnnnnnnn nnnnnnnnnn nnnnnnnnnn nnnnnnnnnn nnnnnnnnnn nnnnnnnnnn
1219501 nnnnnnnnnn nnnnnnnnnn nnnnnnnnnn nnnnnnnnnn nnnnnnnnnn nnnnnnnnnn
1219561 nnnnnnnnnn nnnnnnnnnn nnnnnnnnnn nnnnnnnnnt cggacccggg gattgaatca
1219621 acaaccttta tctcacgcac tcaaagcgtg aaggttatga ttccagttct gcaggcacag
1219681 tcacatataa acaggcgtta ggtggactgg agactaaagg gtgtgtgtgt gtgtgtgtgt
1219741 gtgtgtgtgt gtgtgtgtgt gtgtgtgtgt tctgtgatga actgatgacc tttctagggt
1219801 ggattcattg aagcgggggt taagaagggt ttacttcctg ttgcgttgcc tccagtctga
1219861 ggcggtgccg ggacagatcg ggggggtcct gagagaacag attcaccctg atcttcagtt
1219921 tgatgtctgc gcgttgttgt tctgttggct ttaactgtga ttagacggca gattggagac

1219981 atttattgtt ggcggcgctg cggattcctg acagacaggg tgactcagaa acacaccatc
1220041 catccgaggg gggtgggggg gggggttctg cagagatatg ccaaatgtgt cgtttgtcaa
1220101 caagactcaa gagaaatttc accctcaact acacctcatc tactgtactt cctgtgtgtg
1220161 tacgtgtgtg tgtgtgtgtg tgtgtgtgtg tgtgtgtgtg tgtctatata ctagcatgaa
1220221 acgagcaaat cttgaataat aacaggaaaa tatgtaaaac gcggacgtgc tcggtgttcc
1220281 aaatcagagg aaaaatggcg tcacggttga aagagtcact catgaaatct atcataaagt
1220341 tcagcaaagg tgtattatgg gatggcagac tatgcaaacc atggctaatg acatgggact
1220401 tacgagttcc taccgtgtta ctgtgcaaat atgaattaaa gtgtgtagtg tgtgtgtgtt
1220461 ggtgtgtgtg tgtgtgtgtg tgaagggggg ggggcacaat agatgtgacg gtcacatgtg
1220521 cgtgtccatg tccccagaca cacaggtgtg tgctttcctc cccttgcctg gtcctctaac
1220581 aacgtgtcct ggattaagtg atatgaatgg cgagcctctc ttcacgcagg ctccacctac
1220641 agggggctaa ataaaaaagg ccctgaggac aaatgatgca ccgcgggtcc aaaccgctct
1220701 cactggccct cccttgtgtg ccgtgtggtg ttcagggaca ccaaggatca gctatgagac
1220761 aaatgcagaa gcagaggaaa tcagcaattc aaatcagccc tggtggaaag agatgtggct
1220821 cataacatgc acgtgtgaca cggtggcggc atgagcgacc tggaaggagc gcacgtgtag
1220881 gccagggctg attgatcatg aagctaaatc aactcaaagg ggaactgatg catcagacat
1220941 gaacctgggg tgttgacgct tccgtcaaag agtttctgcc atttaagaaa tgtgaaggat
1221001 ttataaaagg tacttaattt ccctcattca ctgacctttg accccaccca attatatttc
1221061 ttcatcttca gggtgagata atgacaatat gtttgcattt ctcttctctt ttctggcccc
1221121 actcacggtt tcttcctgtt agaagggagt ttatcccttt tgtagatcag cctcaaacgc
1221181 agcgtgcact ttcagatttg ttgttgttta ttgtggacaa taacgacttc tggttcctgc
1221241 tcctcccgtg tctggtggct ctgctgctcc tccgcccgtg attaattagc tttattagac
1221301 gtgttaaagg tcacttacag cgtgaaaaag ctcacttcct ctgccgcacc gcacctgttg
1221361 tttggcaatg aaacaggctg aatacaaatt tcatctcgga acctgaaaag aatttgcatc
1221421 tcaaaaatct ccgcttcact cttccctcct cctcctcctc ctcctcttcc tcttcctcct
1221481 cctcctcgtc tgacttcctc ctgcctccct ccatcacctc cacaccttcg ctcacctcgc
1221541 tagcacacat cagtaacaat ctcatttacc gcctcattaa ctgtagaatc ctagacgctn

```
  1221601 nnnnnnnnnn nnnnnnnnnn nnnnnnnnnn nnnnnnnnnn nnnnnnnnnn
nnnnnnnnnn
  1221661 nnnnnnnnnn nnnnnnnnnn nnnnnnnnnn nnnnnnnnnn nnnnnnnnnn
nnnnnnnnnn
  1221721 nnnnnnnnnn nnnnnnnnnn nnnnnnnnnn nnnnnnnnnn nnnnnnnnnn
nnnnnnnnnn
  1221781 nnnnnnnnnn nnnnnnnnnn nnnnnnnnnn nnnnnnnnnn nnnnnnnnnn
nnnnnnnnnn
  1221841 nnnnnnnnnn nnnnnnnnnn nnnnnnnnnn nnnnnnnnnn nnnnnnnnnn
nnnnnnnnnn
  1221901 nnnnnnnnnn nnnnnnnnnn nnnnnnnnnn nnnnnnnnnn nnnnnnnnnn
nnnnnnnnnn
  1221961 nnnnnnnnnn nnnnnnnnnn nnnnnnnnnn nnnnnnnnnn nnnnnnnnnn
nnnnnnnnnn
  1222021 nnnnnnnnnn nnnnnnnnnn nnnnnnnnnn nnnnnnnnnn nnnnnnnnnn
nnnnnnnnnn
  1222081 nnnnnnnnnn nnnnnnnnnn nnnnnnnnnn nnnnnnnnnn nnnnnnnnnn
nnnnnnnnnn
  1222141 nnnnnnnnnn nnnnnnnnnn nnnnnnnnnn nnnnnnnnnn nnnnnnnnnn
nnnnnnnnnn
  1222201 nnnnnnnnnn nnnnnnnnnn nnnnnnnnnn nnnnnnnnnn nnnnnnnnnn
nnnnnnnnnn
  1222261 nnnnnnnnnn nnnnnnnnnn nnnnnnnnnn nnnnnnnnnn nnnnnnnnnn
nnnnnnnnnn
  1222321 nnnnnnnnnn nnnnnnnnnn nnnnnnnnnn nnnnnnnnnn nnnnnnnnnn
nnnnnnnnnn
  1222381 nnnnnnnnnn nnnnnnnnnn nnnnnnnnnn nnnnnnnnnn nnnnnnnnnn
nnnnnnnnnn
  1222441 nnnnnnnnnn nnnnnnnnnn nnnnnnnnnn nnnnnnnnnn nnnnnnnnnn
nnnnnnnnnn
  1222501 nnnnnnnnnn nnnnnnnnnn nnnnnnnnnn nnnnnnnnnn nnnnnnnnnn
nnnnnnnnnn
  1222561 nnnnnnnnnn nnnnnnnnnn nnnnnnnnnn nnnnnnnnnn nnnnnnnnnn
nnnnnnnnnn
  1222621 nnnnnnnnnn nnnnnnnnnn nnnnnnnnnn nnnnnnnnnn nnnnnnnnnn
nnnnnnnnnn
  1222681 nnnnnnnnnn nnnnnnnnnn nnnnnnnnnn nnnnnnnnnn nnnnnnnnnn
nnnnnnnnnn
  1222741 nnnnnnnnnn nnnnnnnnnn nnnnnnnnnn nnnnnnnnnn nnnnnnnnnn
nnnnnnnnnn
  1222801 nnnnnnnnnn nnnnnnnnnn nnnnnnnnnn nnnnnnnnnn nnnnnnnnnn
nnnnnnnnnn
  1222861 nnnnnnnnnn nnnnnnnnnn nnnnnnnnnn nnnnnnnnnn nnnnnnnnnn
nnnnnnnnnn
  1222921 nnnnnnnnnn nnnnnnnnnn nnnnnnnnnn nnnnnnnnnn nnnnnnnnnn
nnnnnnnnnn
  1222981 nnnnnnnnnn nnnnnnnnnn nnnnnnnnnn nnnnnnnnnn nnnnnnnnnn
nnnnnnnnnn
  1223041 nnnnnnnnnn nnnnnnnnnn nnnnnnnnnn nnnnnnnnnn nnnnnnnnnn
nnnnnnnnnn
  1223101 nnnnnnnnnn nnnnnnnnnn nnnnnnnnnn nnnnnnnnnn nnnnnnnnnn
nnnnnnnnnn
  1223161 nnnnnnnnnn nnnnnnnnnn nnnnnnnnnn nnnnnnnnnn nnnnnnnnnn
nnnnnnnnnn
```

1223221 nnnnnnnnnn nnnnnnnnnn nnnnnnnnnn nnnnnnnnnn nnnnnnnnnn
nnnnnnnnnn
1223281 nnnnnnnnnn nnnnnnnnnn nnnnnnnnnn nnnnnnnnnn nnnnnnnnnn
nnnnnnnnnn
1223341 nnnnnnnnnn nnnnnnnnnn nnnnnnnnnn nnnnnnnnnn nnnnnnnnnn
nnnnnnnnnn
1223401 nnnnnnnnnn nnnnnnnnnn nnnnnnnnnn nnnnnnnnnn nnnnnnnnnn
nnnnnnnnnn
1223461 nnnnnnnnnn nnnnnnnnnn nnnnnnnnnn nnnnnnnnnn nnnnnnnnnn
nnnnnnnnnn
1223521 nnnnnnnnnn nnnnnnnnnn nnnnnnnnnn nnnnnnnnnn nnnnnnnnnn
nnnnnnnnnn
1223581 nnnnnnnnnn nnnnnnnnnn nnnnnnnnnn nnnnnnnnnn nnnnnnnnnn
nnnnnnnnnn
1223641 nnnnnnnnnn nnnnnnnnnn nnnnnnnnnn nnnnnnnnnn nnnnnnnnnn
nnnnnnnnnn
1223701 nnnnnnnnnn nnnnnnnnnn nnnnnnnnnn nnnnnnnnnn nnnnnnnnnn
nnnnnnnnnn
1223761 nnnnnnnnnn nnnnnnnnnn nnnnnnnnnn nnnnnnnnnn nnnnnnnnnn
nnnnnnnnnn
1223821 nnnnnnnnnn nnnnnnnnnn nnnnnnnnnn nnnnnnnnnn nnnnnnnnnn
nnnnnnnnnn
1223881 nnnnnnnnnn nnnnnnnnnn nnnnnnnnnn nnnnnnnnnn nnnnnnnnnn
nnnnnnnnnn
1223941 nnnnnnnnnn nnnnnnnnnn nnnnnnnnnn nnnnnnnnnn nnnnnnnnnn
nnnnnnnnnn
1224001 nnnnnnnnnn nnnnnnnnnn nnnnnnnnnn nnnnnnnnnn nnnnnnnnnn
nnnnnnnnnn
1224061 nnnnnnnnnn nnnnnnnnnn nnnnnnnnnn nnnnnnnnnn nnnnnnnnnn
nnnnnnnnnn
1224121 nnnnnnnnnn nnnnnnnnnn nnnnnnnnnn nnnnnnnnnn nnnnnnnnnn
nnnnnnnnnn
1224181 nnnnnnnnnn nnnnnnnnnn nnnnnnnnnn nnnnnnnnnn nnnnnnnnnn
nnnnnnnnnn
1224241 nnnnnnnnnn nnnnnnnnnn nnnnnnnnnn nnnnnnnnnn nnnnnnnnnn
nnnnnnnnnn
1224301 nnnnnnnnnn nnnnnnnnnn nnnnnnnnnn nnnnnnnnnn nnnnnnnnnn
nnnnnnnnnn
1224361 nnnnnnnnnn nnnnnnnnnn nnnnnnnnnn nnnnnnnnnn nnnnnnnnnn
nnnnnnnnnn
1224421 nnnnnnnnnn nnnnnnnnnn nnnnnnnnnn nnnnnnnnnn nnnnnnnnnn
nnnnnnnnnn
1224481 nnnnnnnnnn nnnnnnnnnn nnnnnnnnnn nnnnnnnnnn nnnnnnnnnn
nnnnnnnnnn
1224541 nnnnnnnnnn nnnnnnnnnn nnnnnnnnnn nnnnnnnnnn nnnnnnnnnn
nnnnnnnnnn
1224601 nnnnnnnnnn nnnnnnnnnn nnnnnnnnnn nnnnnnnnnn nnnnnnnnnn
nnnnnnnnnn
1224661 nnnnnnnnnn nnnnnnnnnn nnnnnnnnnn nnnnnnnnnn nnnnnnnnnn
nnnnnnnnnn
1224721 nnnnnnnnnn nnnnnnnnnn nnnnnnnnnn nnnnnnnnnn nnnnnnnnnn
nnnnnnnnnn
1224781 nnnnnnnnnn nnnnnnnnnn nnnnnnnnnn nnnnnnnnnn nnnnnnnnnn
nnnnnnnnnn

1224841 nnnnnnnnnn nnnnnnnnnn nnnnnnnnnn nnnnnnnnnn nnnnnnnnnn
nnnnnnnnnn
1224901 nnnnnnnnnn nnnnnnnnnn nnnnnnnnnn nnnnnnnnnn nnnnnnnnnn
nnnnnnnnnn
1224961 nnnnnnnnnn nnnnnnnnnn nnnnnnnnnn nnnnnnnnnn nnnnnnnnnn
nnnnnnnnnn
1225021 nnnnnnnnnn nnnnnnnnnn nnnnnnnnnn nnnnnnnnnn nnnnnnnnnn
nnnnnnnnnn
1225081 nnnnnnnnnn nnnnnnnnnn nnnnnnnnnn nnnnnnnnnn nnnnnnnnnn
nnnnnnnnnn
1225141 nnnnnnnnnn nnnnnnnnnn nnnnnnnnnn nnnnnnnnnn nnnnnnnnnn
nnnnnnnnnn
1225201 nnnnnnnnnn nnnnnnnnnn nnnnnnnnnn nnnnnnnnnn nnnnnnnnnn
nnnnnnnnnn
1225261 nnnnnnnnnn nnnnnnnnnn nnnnnnnnnn nnnnnnnnnn nnnnnnnnnn
nnnnnnnnnn
1225321 nnnnnnnnnn nnnnnnnnnn nnnnnnnnnn nnnnnnnnnn nnnnnnnnnn
nnnnnnnnnn
1225381 nnnnnnnnnn nnnnnnnnnn nnnnnnnnnn nnnnnnnnnn nnnnnnnnnn
nnnnnnnnnn
1225441 nnnnnnnnnn nnnnnnnnnn nnnnnnnnnn nnnnnnnnnn nnnnnnnnnn
nnnnnnnnnn
1225501 nnnnnnnnnn nnnnnnnnnn nnnnnnnnnn nnnnnnnnnn nnnnnnnnnn
nnnnnnnnnn
1225561 nnnnnnnnnn nnnnnnnnnn nnnnnnnnnn nnnnnnnnnn nnnnnnnnnn
nnnnnnnnnn
1225621 nnnnnnnnnn nnnnnnnnnn nnnnnnnnnn nnnnnnnnnn nnnnnnnnnn
nnnnnnnnnn
1225681 nnnnnnnnnn nnnnnnnnnn nnnnnnnnnn nnnnnnnnnn nnnnnnnnnn
nnnnnnnnnn
1225741 nnnnnnnnnn nnnnnnnnnn nnnnnnnnnn nnnnnnnnnn nnnnnnnnnn
nnnnnnnnnn
1225801 nnnnnnnnnn nnnnnnnnnn nnnnnnnnnn nnnnnnnnnn nnnnnnnnnn
nnnnnnnnnn
1225861 nnnnnnnnnn nnnnnnnnnn nnnnnnnnnn nnnnnnnnnn nnnnnnnnnn
nnnnnnnnnn
1225921 nnnnnnnnnn nnnnnnnnnn nnnnnnnnnn nnnnnnnnnn nnnnnnnnnn
nnnnnnnnnn
1225981 nnnnnnnnnn nnnnnnnnnn nnnnnnnnnn nnnnnnnnnn nnnnnnnnnn
nnnnnnnnnn
1226041 nnnnnnnnnn nnnnnnnnnn nnnnnnnnnn nnnnnnnnnn nnnnnnnnnn
nnnnnnnnnn
1226101 nnnnnnnnnn nnnnnnnnnn nnnnnnnnnn nnnnnnnnnn nnnnnnnnnn
nnnnnnnnnn
1226161 nnnnnnnnnn nnnnnnnnnn nnnnnnnnnn nnnnnnnnnn nnnnnnnnnn
nnnnnnnnnn
1226221 nnnnnnnnnn nnnnnnnnnn nnnnnnnnnn nnnnnnnnnn nnnnnnnnnn
nnnnnnnnnn
1226281 nnnnnnnnnn nnnnnnnnnn nnnnnnnnnn nnnnnnnnnn nnnnnnnnnn
nnnnnnnnnn
1226341 nnnnnnnnnn nnnnnnnnnn nnnnnnnnnn nnnnnnnnnn nnnnnnnnnn
nnnnnnnnnn
1226401 nnnnnnnnnn nnnnnnnnnn nnnnnnnnnn nnnnnnnnnn nnnnnnnnnn
nnnnnnnnnn

1226461 nnnnnnnnnn nnnnnnnnnn nnnnnnnnnn nnnnnnnnnn nnnnnnnnnn
nnnnnnnnnn
1226521 nnnnnnnnnn nnnnnnnnnn nnnnnnnnnn nnnnnnnnnn nnnnnnnnnn
nnnnnnnnnn
1226581 nnnnnnnnnn nnnnnnnnnn nnnnnnnnnn nnnnnnnnnn nnnnnnnnnn
nnnnnnnnnn
1226641 nnnnnnnnnn nnnnnnnnnn nnnnnnnnnn nnnnnnnnnn nnnnnnnnnn
nnnnnnnnnn
1226701 nnnnnnnnnn nnnnnnnnnn nnnnnnnnnn nnnnnnnnnn nnnnnnnnnn
nnnnnnnnnn
1226761 nnnnnnnnnn nnnnnnnnnn nnnnnnnnnn nnnnnnnnnn nnnnnnnnnn
nnnnnnnnnn
1226821 nnnnnnnnnn nnnnnnnnnn nnnnnnnnnn nnnnnnnnnn nnnnnnnnnn
nnnnnnnnnn
1226881 nnnnnnnnnn nnnnnnnnnn nnnnnnnnnn nnnnnnnnnn nnnnnnnnnn
nnnnnnnnnn
1226941 nnnnnnnnnn nnnnnnnnnn nnnnnnnnnn nnnnnnnnnn nnnnnnnnnn
nnnnnnnnnn
1227001 nnnnnnnnnn nnnnnnnnnn nnnnnnnnnn nnnnnnnnnn nnnnnnnnnn
nnnnnnnnnn
1227061 nnnnnnnnnn nnnnnnnnnn nnnnnnnnnn nnnnnnnnnn nnnnnnnnnn
nnnnnnnnnn
1227121 nnnnnnnnnn nnnnnnnnnn nnnnnnnnnn nnnnnnnnnn nnnnnnnnnn
nnnnnnnnnn
1227181 nnnnnnnnnn nnnnnnnnnn nnnnnnnnnn nnnnnnnnnn nnnnnnnnnn
nnnnnnnnnn
1227241 nnnnnnnnnn nnnnnnnnnn nnnnnnnnnn nnnnnnnnnn nnnnnnnnnn
nnnnnnnnnn
1227301 nnnnnnnnnn nnnnnnnnnn nnnnnnnnnn nnnnnnnnnn nnnnnnnnnn
nnnnnnnnnn
1227361 nnnnnnnnnn nnnnnnnnnn nnnnnnnnnn nnnnnnnnnn nnnnnnnnnn
nnnnnnnnnn
1227421 nnnnnnnnnn nnnnnnnnnn nnnnnnnnnn nnnnnnnnnn nnnnnnnnnn
nnnnnnnnnn
1227481 nnnnnnnnnn nnnnnnnnnn nnnnnnnnnn nnnnnnnnnn nnnnnnnnnn
nnnnnnnnnn
1227541 nnnnnnnnnn nnnnnnnnnn nnnnnnnnnn nnnnnnnnnn nnnnnnnnnn
nnnnnnnnnn
1227601 nnnnnnnnnn nnnnnnnnnn nnnnnnnnnn nnnnnnnnnn nnnnnnnnnn
nnnnnnnnnn
1227661 nnnnnnnnnn nnnnnnnnnn nnnnnnnnnn nnnnnnnnnn nnnnnnnnnn
nnnnnnnnnn
1227721 nnnnnnnnnn nnnnnnnnnn nnnnnnnnnn nnnnnnnnnn nnnnnnnnnn
nnnnnnnnnn
1227781 nnnnnnnnnn nnnnnnnnnn nnnnnnnnnn nnnnnnnnnn nnnnnnnnnn
nnnnnnnnnn
1227841 nnnnnnnnnn nnnnnnnnnn nnnnnnnnnn nnnnnnnnnn nnnnnnnnnn
nnnnnnnnnn
1227901 nnnnnnnnnn nnnnnnnnnn nnnnnnnnnn nnnnnnnnnn nnnnnnnnnn
nnnnnnnnnn
1227961 nnnnnnnnnn nnnnnnnnnn nnnnnnnnnn nnnnnnnnnn nnnnnnnnnn
nnnnnnnnnn
1228021 nnnnnnnnnn nnnnnnnnnn nnnnnnnnnn nnnnnnnnnn nnnnnnnnnn
nnnnnnnnnn

1228081 nnnnnnnnnn nnnnnnnnnn nnnnnnnnnn nnnnnnnnnn nnnnnnnnnn nnnnnnnnnn
1228141 nnnnnnnnnn nnnnnnnnnn nnnnnnnnnn nnnnnnnnnn nnnnnnnnnn nnnnnnnnnn
1228201 nnnnnnnnnn nnnnnnnnnn nnnnnnnnnn nnnnnnnnnn nnnnnnnnnn nnnnnnnnnn
1228261 nnnnnnnnnn nnnnnnnnnn nnnnnnnnnn nnnnnnnnnn nnnnnnnnnn nnnnnnnnnn
1228321 nnnnnnnnnn nnnnnnnnnn nnnnnnnnnn nnnnnnnnnn nnnnnnnnnn nnnnnnnnnn
1228381 nnnnnnnnnn nnnnnnnnnn nnnnnnnnnn nnnnnnnnnn nnnnnnnnnn nnnnnnnnnn
1228441 nnnnnnnnnn nnnnnnnnnn nnnnnnnnnn nnnnnnnnnn nnnnnnnnnn nnnnnnnnnn
1228501 nnnnnnnnnn nnnnnnnnnn nnnnnnnnnn nnnnnnnnnn nnnnnnnnnn nnnnnnnnnn
1228561 nnnnnnnnnn nnnnnnnnnn nnnnnnnnnn nnnnnnnnnn nnnnnnnnnn nnnnnnnnnn
1228621 nnnnnnnnnn nnnnnnnnnn nnnnnnnnnn nnnnnnnnnn nnnnnnnnnn nnnnnnnnnn
1228681 nnnnnnnnnn nnnnnnnnnn nnnnnnnnnn nnnnnnnnnn nnnnnnnnnn nnnnnnnnnn
1228741 nnnnnnnnnn nnnnnnnnnn nnnnnnnnnn nnnnnnnnnn nnnnnnnnnn nnnnnnnnnn
1228801 nnnnnnnnnn nnnnnnnnnn nnnnnnnnnn nnnnnnnnnn nnnnnnnnnn nnnnnnnnnn
1228861 nnnnnnnnnn nnnnnnnnnn nnnnnnnnnn nnnnnnnnnn nnnnnnnnnn nnnnnnnnnn
1228921 nnnnnnnnnn nnnnnnnnnn nnnnnnnnnn nnnnnnnnnn nnnnnnnnnn nnnnnnnnnn
1228981 nnnnnnnnnn nnnnnnnnnn nnnnnnnnnn nnnnnnnnnn nnnnnnnnnn nnnnnnnnnn
1229041 nnnnnnnnnn nnnnnnnnnn nnnnnnnnnn nnnnnnnnnn nnnnnnnnnn nnnnnnnnnn
1229101 nnnnnnnnnn nnnnnnnnnn nnnnnnnnnn nnnnnnnnnn nnnnnnnnnn nnnnnnnnnn
1229161 nnnnnnnnnn nnnnnnnnnn nnnnnnnnnn nnnnnnnnnn nnnnnnnnnn nnnnnnnnnn
1229221 nnnnnnnnnn nnnnnnnnnn nnnnnnnnnn nnnnnnnnnn nnnnnnnnnn nnnnnnnnnn
1229281 nnnnnnnnnn nnnnnnnnnn nnnnnnnnnn nnnnnnnnnn nnnnnnnnnn nnnnnnnnnn
1229341 nnnnnnnnnn nnnnnnnnnn nnnnnnnnnn nnnnnnnnnn nnnnnnnnnn nnnnnnnnnn
1229401 nnnnnnnnnn nnnnnnnnnn nnnnnnnnnn nnnnnnnnnn nnnnnnnnnn nnnnnnnnnn
1229461 nnnnnnnnnn nnnnnnnnnn nnnnnnnnnn nnnnnnnnnn nnnnnnnnnn nnnnnnnnnn
1229521 nnnnnnnnnn nnnnnnnnnn nnnnnnnnnn nnnnnnnnnn nnnnnnnnnn nnnnnnnnnn
1229581 nnnnnnnnnn nnnnnnnnnn nnnnnnnnnn nnnnnnnnnn nnnnnnnnnn nnnnnnnnnn
1229641 nnnnnnnnnn nnnnnnnnnn nnnnnnnnnn nnnnnnnnnn nnnnnnnnnn nnnnnnnnnn

1229701 nnnnnnnnnn nnnnnnnnnn nnnnnnnnnn nnnnnnnnnn nnnnnnnnnn
nnnnnnnnnn
1229761 nnnnnnnnnn nnnnnnnnnn nnnnnnnnnn nnnnnnnnnn nnnnnnnnnn
nnnnnnnnnn
1229821 nnnnnnnnnn nnnnnnnnnn nnnnnnnnnn nnnnnnnnnn nnnnnnnnnn
nnnnnnnnnn
1229881 nnnnnnnnnn nnnnnnnnnn nnnnnnnnnn nnnnnnnnnn nnnnnnnnnn
nnnnnnnnnn
1229941 nnnnnnnnnn nnnnnnnnnn nnnnnnnnnn nnnnnnnnnn nnnnnnnnnn
nnnnnnnnnn
1230001 nnnnnnnnnn nnnnnnnnnn nnnnnnnnnn nnnnnnnnnn nnnnnnnnnn
nnnnnnnnnn
1230061 nnnnnnnnnn nnnnnnnnnn nnnnnnnnnn nnnnnnnnnn nnnnnnnnnn
nnnnnnnnnn
1230121 nnnnnnnnnn nnnnnnnnnn nnnnnnnnnn nnnnnnnnnn nnnnnnnnnn
nnnnnnnnnn
1230181 nnnnnnnnnn nnnnnnnnnn nnnnnnnnnn nnnnnnnnnn nnnnnnnnnn
nnnnnnnnnn
1230241 nnnnnnnnnn nnnnnnnnnn nnnnnnnnnn nnnnnnnnnn nnnnnnnnnn
nnnnnnnnnn
1230301 nnnnnnnnnn nnnnnnnnnn nnnnnnnnnn nnnnnnnnnn nnnnnnnnnn
nnnnnnnnnn
1230361 nnnnnnnnnn nnnnnnnnnn nnnnnnnnnn nnnnnnnnnn nnnnnnnnnn
nnnnnnnnnn
1230421 nnnnnnnnnn nnnnnnnnnn nnnnnnnnnn nnnnnnnnnn nnnnnnnnnn
nnnnnnnnnn
1230481 nnnnnnnnnn nnnnnnnnnn nnnnnnnnnn nnnnnnnnnn nnnnnnnnnn
nnnnnnnnnn
1230541 nnnnnnnnnn nnnnnnnnnn nnnnnnnnnn nnnnnnnnnn nnnnnnnnnn
nnnnnnnnnn
1230601 nnnnnnnnnn nnnnnnnnnn nnnnnnnnnn nnnnnnnnnn nnnnnnnnnn
nnnnnnnnnn
1230661 nnnnnnnnnn nnnnnnnnnn nnnnnnnnnn nnnnnnnnnn nnnnnnnnnn
nnnnnnnnnn
1230721 nnnnnnnnnn nnnnnnnnnn nnnnnnnnnn nnnnnnnnnn nnnnnnnnnn
nnnnnnnnnn
1230781 nnnnnnnnnn nnnnnnnnnn nnnnnnnnnn nnnnnnnnnn nnnnnnnnnn
nnnnnnnnnn
1230841 nnnnnnnnnn nnnnnnnnnn nnnnnnnnnn nnnnnnnnnn nnnnnnnnnn
nnnnnnnnnn
1230901 nnnnnnnnnn nnnnnnnnnn nnnnnnnnnn nnnnnnnnnn nnnnnnnnnn
nnnnnnnnnn
1230961 nnnnnnnnnn nnnnnnnnnn nnnnnnnnnn nnnnnnnnnn nnnnnnnnnn
nnnnnnnnnn
1231021 nnnnnnnnnn nnnnnnnnnn nnnnnnnnnn nnnnnnnnnn nnnnnnnnnn
nnnnnnnnnn
1231081 nnnnnnnnnn nnnnnnnnnn nnnnnnnnnn nnnnnnnnnn nnnnnnnnnn
nnnnnnnnnn
1231141 nnnnnnnnnn nnnnnnnnnn nnnnnnnnnn nnnnnnnnnn nnnnnnnnnn
nnnnnnnnnn
1231201 nnnnnnnnnn nnnnnnnnnn nnnnnnnnnn nnnnnnnnnn nnnnnnnnnn
nnnnnnnnnn
1231261 nnnnnnnnnn nnnnnnnnnn nnnnnnnnnn nnnnnnnnnn nnnnnnnnnn
nnnnnnnnnn

1231321 nnnnnnnnnn nnnnnnnnnn nnnnnnnnnn nnnnnnnnnn nnnnnnnnnn nnnnnnnnnn
1231381 nnnnnnnnnn nnnnnnnnnn nnnnnnnnnn nnnnnnnnnn nnnnnnnnnn nnnnnnnnnn
1231441 nnnnnnnnnn nnnnnnnnnn nnnnnnnnnn nnnnnnnnnn nnnnnnnnnn nnnnnnnnnn
1231501 nnnnnnnnnn nnnnnnnnnn nnnnnnnnnn nnnnnnnnnn nnnnnnnnnn nnnnnnnnnn
1231561 nnnnnnnnnn nnnnnnnnnn nnnnnnnnnn nnnnnnnnnn nnnnnnnnnn nnnnnnnnnn
1231621 nnnnnnnnnn nnnnnnnnnn nnnnnnnnnn nnnnnnnnnn nnnnnnnnnn nnnnnnnnnn
1231681 nnnnnnnnnn nnnnnnnnnn nnnnnnnnnn nnnnnnnnnn nnnnnnnnnn nnnnnnnnnn
1231741 nnnnnnnnnn nnnnnnnnnn nnnnnnnnnn nnnnnnnnnn nnnnnnnnnn nnnnnnnnnn
1231801 nnnnnnnnnn nnnnnnnnnn nnnnnnnnnn nnnnnnnnnn nnnnnnnnnn nnnnnnnnnn
1231861 nnnnnnnnnn nnnnnnnnnn nnnnnnnnnn nnnnnnnnnn nnnnnnnnnn nnnnnnnnnn
1231921 nnnnnnnnnn nnnnnnnnnn nnnnnnnnnn nnnnnnnnnn nnnnnnnnnn nnnnnnnnnn
1231981 nnnnnnnnnn nnnnnnnnnn nnnnnnnnnn nnnnnnnnnn nnnnnnnnnn nnnnnnnnnn
1232041 nnnnnnnnnn nnnnnnnnnn nnnnnnnnnn nnnnnnnnnn nnnnnnnnnn nnnnnnnnnn
1232101 nnnnnnnnnn nnnnnnnnnn nnnnnnnnnn nnnnnnnnnn nnnnnnnnnn nnnnnnnnnn
1232161 nnnnnnnnnn nnnnnnnnnn nnnnnnnnnn nnnnnnnnnn nnnnnnnnnn nnnnnnnnnn
1232221 nnnnnnnnnn nnnnnnnnnn nnnnnnnnnn nnnnnnnnnn nnnnnnnnnn nnnnnnnnnn
1232281 nnnnnnnnnn nnnnnnnnnn nnnnnnnnnn nnnnnnnnnn nnnnnnnnnn nnnnnnnnnn
1232341 nnnnnnnnnn nnnnnnnnnn nnnnnnnnnn nnnnnnnnnn nnnnnnnnnn nnnnnnnnnn
1232401 nnnnnnnnnn nnnnnnnnnn nnnnnnnnnn nnnnnnnnnn nnnnnnnnnn nnnnnnnnnn
1232461 nnnnnnnnnn nnnnnnnnnn nnnnnnnnnn nnnnnnnnnn nnnnnnnnnn nnnnnnnnnn
1232521 nnnnnnnnnn nnnnnnnnnn nnnnnnnnnn nnnnnnnnnn nnnnnnnnnn nnnnnnnnnn
1232581 nnnnnnnnnn nnnnnnnnnn nnnnnnnnnn nnnnnnnnnn nnnnnnnnnn nnnnnnnnnn
1232641 nnnnnnnnnn nnnnnnnnnn nnnnnnnnnn nnnnnnnnnn nnnnnnnnnn nnnnnnnnnn
1232701 nnnnnnnnnn nnnnnnnnnn nnnnnnnnnn nnnnnnnnnn nnnnnnnnnn nnnnnnnnnn
1232761 nnnnnnnnnn nnnnnnnnnn nnnnnnnnnn nnnnnnnnnn nnnnnnnnnn nnnnnnnnnn
1232821 nnnnnnnnnn nnnnnnnnnn nnnnnnnnnn nnnnnnnnnn nnnnnnnnnn nnnnnnnnnn
1232881 nnnnnnnnnn nnnnnnnnnn nnnnnnnnnn nnnnnnnnnn nnnnnnnnnn nnnnnnnnnn

1232941 nnnnnnnnnn nnnnnnnnnn nnnnnnnnnn nnnnnnnnnn nnnnnnnnnn nnnnnnnnnn
1233001 nnnnnnnnnn nnnnnnnnnn nnnnnnnnnn nnnnnnnnnn nnnnnnnnnn nnnnnnnnnn
1233061 nnnnnnnnnn nnnnnnnnnn nnnnnnnnnn nnnnnnnnnn nnnnnnnnnn nnnnnnnnnn
1233121 nnnnnnnnnn nnnnnnnnnn nnnnnnnnnn nnnnnnnnnn nnnnnnnnnn nnnnnnnnnn
1233181 nnnnnnnnnn nnnnnnnnnn nnnnnnnnnn nnnnnnnnnn nnnnnnnnnn nnnnnnnnnn
1233241 nnnnnnnnnn nnnnnnnnnn nnnnnnnnnn nnnnnnnnnn nnnnnnnnnn nnnnnnnnnn
1233301 nnnnnnnnnn nnnnnnnnnn nnnnnnnnnn nnnnnnnnnn nnnnnnnnnn nnnnnnnnnn
1233361 nnnnnnnnnn nnnnnnnnnn nnnnnnnnnn nnnnnnnnnn nnnnnnnnnn nnnnnnnnnn
1233421 nnnnnnnnnn nnnnnnnnnn nnnnnnnnnn nnnnnnnnnn nnnnnnnnnn nnnnnnnnnn
1233481 nnnnnnnnnn nnnnnnnnnn nnnnnnnnnn nnnnnnnnnn nnnnnnnnnn nnnnnnnnnn
1233541 nnnnnnnnnn nnnnnnnnnn nnnnnnnnnn nnnnnnnnnn nnnnnnnnnn nnnnnnnnnn
1233601 nnnnnnnnnn nnnnnnnnnn nnnnnnnnnn nnnnnnnnnn nnnnnnnnnn nnnnnnnnnn
1233661 nnnnnnnnnn nnnnnnnnnn nnnnnnnnnn nnnnnnnnnn nnnnnnnnnn nnnnnnnnnn
1233721 nnnnnnnnnn nnnnnnnnnn nnnnnnnnnn nnnnnnnnnn nnnnnnnnnn nnnnnnnnnn
1233781 nnnnnnnnnn nnnnnnnnnn nnnnnnnnnn nnnnnnnnnn nnnnnnnnnn nnnnnnnnnn
1233841 nnnnnnnnnn nnnnnnnnnn nnnnnnnnnn nnnnnnnnnn nnnnnnnnnn nnnnnnnnnn
1233901 nnnnnnnnnn nnnnnnnnnn nnnnnnnnnn nnnnnnnnnn nnnnnnnnnn nnnnnnnnnn
1233961 nnnnnnnnnn nnnnnnnnnn nnnnnnnnnn nnnnnnnnnn nnnnnnnnnn nnnnnnnnnn
1234021 nnnnnnnnnn nnnnnnnnnn nnnnnnnnnn nnnnnnnnnn nnnnnnnnnn nnnnnnnnnn
1234081 nnnnnnnnnn nnnnnnnnnn nnnnnnnnnn nnnnnnnnnn nnnnnnnnnn nnnnnnnnnn
1234141 nnnnnnnnnn nnnnnnnnnn nnnnnnnnnn nnnnnnnnnn nnnnnnnnnn nnnnnnnnnn
1234201 nnnnnnnnnn nnnnnnnnnn nnnnnnnnnn nnnnnnnnnn nnnnnnnnnn nnnnnnnnnn
1234261 nnnnnnnnnn nnnnnnnnnn nnnnnnnnnn nnnnnnnnnn nnnnnnnnnn nnnnnnnnnn
1234321 nnnnnnnnnn nnnnnnnnnn nnnnnnnnnn nnnnnnnnnn nnnnnnnnnn nnnnnnnnnn
1234381 nnnnnnnnnn nnnnnnnnnn nnnnnnnnnn nnnnnnnnnn nnnnnnnnnn nnnnnnnnnn
1234441 nnnnnnnnnn nnnnnnnnnn nnnnnnnnnn nnnnnnnnnn nnnnnnnnnn nnnnnnnnnn
1234501 nnnnnnnnnn nnnnnnnnnn nnnnnnnnnn nnnnnnnnnn nnnnnnnnnn nnnnnnnnnn

1234561 nnnnnnnnnn nnnnnnnnnn nnnnnnnnnn nnnnnnnnnn nnnnnnnnnn
nnnnnnnnnn
1234621 nnnnnnnnnn nnnnnnnnnn nnnnnnnnnn nnnnnnnnnn nnnnnnnnnn
nnnnnnnnnn
1234681 nnnnnnnnnn nnnnnnnnnn nnnnnnnnnn nnnnnnnnnn nnnnnnnnnn
nnnnnnnnnn
1234741 nnnnnnnnnn nnnnnnnnnn nnnnnnnnnn nnnnnnnnnn nnnnnnnnnn
nnnnnnnnnn
1234801 nnnnnnnnnn nnnnnnnnnn nnnnnnnnnn nnnnnnnnnn nnnnnnnnnn
nnnnnnnnnn
1234861 nnnnnnnnnn nnnnnnnnnn nnnnnnnnnn nnnnnnnnnn nnnnnnnnnn
nnnnnnnnnn
1234921 nnnnnnnnnn nnnnnnnnnn nnnnnnnnnn nnnnnnnnnn nnnnnnnnnn
nnnnnnnnnn
1234981 nnnnnnnnnn nnnnnnnnnn nnnnnnnnnn nnnnnnnnnn nnnnnnnnnn
nnnnnnnnnn
1235041 nnnnnnnnnn nnnnnnnnnn nnnnnnnnnn nnnnnnnnnn nnnnnnnnnn
nnnnnnnnnn
1235101 nnnnnnnnnn nnnnnnnnnn nnnnnnnnnn nnnnnnnnnn nnnnnnnnnn
nnnnnnnnnn
1235161 nnnnnnnnnn nnnnnnnnnn nnnnnnnnnn nnnnnnnnnn nnnnnnnnnn
nnnnnnnnnn
1235221 nnnnnnnnnn nnnnnnnnnn nnnnnnnnnn nnnnnnnnnn nnnnnnnnnn
nnnnnnnnnn
1235281 nnnnnnnnnn nnnnnnnnnn nnnnnnnnnn nnnnnnnnnn nnnnnnnnnn
nnnnnnnnnn
1235341 nnnnnnnnnn nnnnnnnnnn nnnnnnnnnn nnnnnnnnnn nnnnnnnnnn
nnnnnnnnnn
1235401 nnnnnnnnnn nnnnnnnnnn nnnnnnnnnn nnnnnnnnnn nnnnnnnnnn
nnnnnnnnnn
1235461 nnnnnnnnnn nnnnnnnnnn nnnnnnnnnn nnnnnnnnnn nnnnnnnnnn
nnnnnnnnnn
1235521 nnnnnnnnnn nnnnnnnnnn nnnnnnnnnn nnnnnnnnnn nnnnnnnnnn
nnnnnnnnnn
1235581 nnnnnnnnnn nnnnnnnnnn nnnnnnnnnn nnnnnnnnnn nnnnnnnnnn
nnnnnnnnnn
1235641 nnnnnnnnnn nnnnnnnnnn nnnnnnnnnn nnnnnnnnnn nnnnnnnnnn
nnnnnnnnnn
1235701 nnnnnnnnnn nnnnnnnnnn nnnnnnnnnn nnnnnnnnnn nnnnnnnnnn
nnnnnnnnnn
1235761 nnnnnnnnnn nnnnnnnnnn nnnnnnnnnn nnnnnnnnnn nnnnnnnnnn
nnnnnnnnnn
1235821 nnnnnnnnnn nnnnnnnnnn nnnnnnnnnn nnnnnnnnnn nnnnnnnnnn
nnnnnnnnnn
1235881 nnnnnnnnnn nnnnnnnnnn nnnnnnnnnn nnnnnnnnnn nnnnnnnnnn
nnnnnnnnnn
1235941 nnnnnnnnnn nnnnnnnnnn nnnnnnnnnn nnnnnnnnnn nnnnnnnnnn
nnnnnnnnnn
1236001 nnnnnnnnnn nnnnnnnnnn nnnnnnnnnn nnnnnnnnnn nnnnnnnnnn
nnnnnnnnnn
1236061 nnnnnnnnnn nnnnnnnnnn nnnnnnnnnn nnnnnnnnnn nnnnnnnnnn
nnnnnnnnnn
1236121 nnnnnnnnnn nnnnnnnnnn nnnnnnnnnn nnnnnnnnnn nnnnnnnnnn
nnnnnnnnnn

1236181 nnnnnnnnnn nnnnnnnnnn nnnnnnnnnn nnnnnnnnnn nnnnnnnnnn
nnnnnnnnnn
1236241 nnnnnnnnnn nnnnnnnnnn nnnnnnnnnn nnnnnnnnnn nnnnnnnnnn
nnnnnnnnnn
1236301 nnnnnnnnnn nnnnnnnnnn nnnnnnnnnn nnnnnnnnnn nnnnnnnnnn
nnnnnnnnnn
1236361 nnnnnnnnnn nnnnnnnnnn nnnnnnnnnn nnnnnnnnnn nnnnnnnnnn
nnnnnnnnnn
1236421 nnnnnnnnnn nnnnnnnnnn nnnnnnnnnn nnnnnnnnnn nnnnnnnnnn
nnnnnnnnnn
1236481 nnnnnnnnnn nnnnnnnnnn nnnnnnnnnn nnnnnnnnnn nnnnnnnnnn
nnnnnnnnnn
1236541 nnnnnnnnnn nnnnnnnnnn nnnnnnnnnn nnnnnnnnnn nnnnnnnnnn
nnnnnnnnnn
1236601 nnnnnnnnnn nnnnnnnnnn nnnnnnnnnn nnnnnnnnnn nnnnnnnnnn
nnnnnnnnnn
1236661 nnnnnnnnnn nnnnnnnnnn nnnnnnnnnn nnnnnnnnnn nnnnnnnnnn
nnnnnnnnnn
1236721 nnnnnnnnnn nnnnnnnnnn nnnnnnnnnn nnnnnnnnnn nnnnnnnnnn
nnnnnnnnnn
1236781 nnnnnnnnnn nnnnnnnnnn nnnnnnnnnn nnnnnnnnnn nnnnnnnnnn
nnnnnnnnnn
1236841 nnnnnnnnnn nnnnnnnnnn nnnnnnnnnn nnnnnnnnnn nnnnnnnnnn
nnnnnnnnnn
1236901 nnnnnnnnnn nnnnnnnnnn nnnnnnnnnn nnnnnnnnnn nnnnnnnnnn
nnnnnnnnnn
1236961 nnnnnnnnnn nnnnnnnnnn nnnnnnnnnn nnnnnnnnnn nnnnnnnnnn
nnnnnnnnnn
1237021 nnnnnnnnnn nnnnnnnnnn nnnnnnnnnn nnnnnnnnnn nnnnnnnnnn
nnnnnnnnnn
1237081 nnnnnnnnnn nnnnnnnnnn nnnnnnnnnn nnnnnnnnnn nnnnnnnnnn
nnnnnnnnnn
1237141 nnnnnnnnnn nnnnnnnnnn nnnnnnnnnn nnnnnnnnnn nnnnnnnnnn
nnnnnnnnnn
1237201 nnnnnnnnnn nnnnnnnnnn nnnnnnnnnn nnnnnnnnnn nnnnnnnnnn
nnnnnnnnnn
1237261 nnnnnnnnnn nnnnnnnnnn nnnnnnnnnn nnnnnnnnnn nnnnnnnnnn
nnnnnnnnnn
1237321 nnnnnnnnnn nnnnnnnnnn nnnnnnnnnn nnnnnnnnnn nnnnnnnnnn
nnnnnnnnnn
1237381 nnnnnnnnnn nnnnnnnnnn nnnnnnnnnn nnnnnnnnnn nnnnnnnnnn
nnnnnnnnnn
1237441 nnnnnnnnnn nnnnnnnnnn nnnnnnnnnn nnnnnnnnnn nnnnnnnnnn
nnnnnnnnnn
1237501 nnnnnnnnnn nnnnnnnnnn nnnnnnnnnn nnnnnnnnnn nnnnnnnnnn
nnnnnnnnnn
1237561 nnnnnnnnnn nnnnnnnnnn nnnnnnnnnn nnnnnnnnnn nnnnnnnnnn
nnnnnnnnnn
1237621 nnnnnnnnnn nnnnnnnnnn nnnnnnnnnn nnnnnnnnnn nnnnnnnnnn
nnnnnnnnnn
1237681 nnnnnnnnnn nnnnnnnnnn nnnnnnnnnn nnnnnnnnnn nnnnnnnnnn
nnnnnnnnnn
1237741 nnnnnnnnnn nnnnnnnnnn nnnnnnnnnn nnnnnnnnnn nnnnnnnnnn
nnnnnnnnnn

1237801 nnnnnnnnnn nnnnnnnnnn nnnnnnnnnn nnnnnnnnnn nnnnnnnnnn nnnnnnnnnn
1237861 nnnnnnnnnn nnnnnnnnnn nnnnnnnnnn nnnnnnnnnn nnnnnnnnnn nnnnnnnnnn
1237921 nnnnnnnnnn nnnnnnnnnn nnnnnnnnnn nnnnnnnnnn nnnnnnnnnn nnnnnnnnnn
1237981 nnnnnnnnnn nnnnnnnnnn nnnnnnnnnn nnnnnnnnnn nnnnnnnnnn nnnnnnnnnn
1238041 nnnnnnnnnn nntgcaagct tgccaaggag ctggcagact attgcatccc tagctatgtg
1238101 tcgtaatagt atggccctcg gaggacttta ttaaaattga aatggccctc gatatgaaaa
1238161 aggttcccca cccctggtct atgctctgca gggccagcaa gtggccattc acctcagggg
1238221 agcgcctgtg caccacagat caagccctga gcaataaccc cccaccaaca ccccccaaca
1238281 gtttaaagta aacaaacatt tttcttttgc aggggaagaa gtttnattac aggtgccttc
1238341 tatngcaaaa ataatttnaa caacaaattc tatgcgtgca aantttccat tctgtacttc
1238401 ctggtctcac acttcctcct gtctcacctg agctctcatt ggaacacctg atccactcac
1238461 cctgtcaccc atctccaaca tgctcctcag ggtttgcatc ctcttcatgt ggttccacac
1238521 aggtaagacc gtgcatccaa atgtaatcca gagtcttaat gagctctttt ctcttctctc
1238581 catgacagtg aagacctgga catcctctgc atcccccaga tgtgtcacag atgttgagag
1238641 gagctcctca tcagcagaag gtatgaggta ttatttttat ctgattttac attcagaaca
1238701 gtgatggttc ctattatttt agatctttag acgtggttgc aggtggcctc tgagacagaa
1238761 tgagagttca ggaatgtatg gaaattccca gttactcatt tgaactcatt tacttctact
1238821 ttttactatg attttatgac aacattagca tagaccgcac aaatgcagtg cagcctgcca
1238881 ccttgttatt gggttaaatt gaaattaact tagacactaa agttctttaa agtgtgtgta
1238941 gatgttgccc tgtgatgaac caatgcacaa acaaacaaag cttcagccag catgtttgct
1239001 cataaaaggg aagcaaaaag ggtggatgct ttaatcatgg ttcacaatag tttttacgga
1239061 cactctcccg ctgtccattg ttgccatggt gaaggaacca gtggctcttg ccattgaaaa
1239121 ccgaatactg agacattgtg ggttcctttt aatcccttta attttgattg tcagtgttga
1239181 ggttcaggtg ccgaaacatg taataatcaa ggctgtgggt aaacggaggg attaagagtc
1239241 atccgtgcgc tgataaagaa cacttcccat tctgtcactt cctggtccca cttcctcctg
1239301 tctctcctga gctctcatta aagcaccatg agccgctctc aaacatcagc ctgagtgttg
1239361 gtgccctctt catgtgctgt gagagcacag gtcggaacaa cccactgagg gtacaggcgg

1239421 gtctagaagc cacagaacct ccactgctgg agatgttagc agcatctcag ctgcagcaga
1239481 tcgaagcttc caaaccgatt ttatccaaac acatattaca tgtgcaaaaa ggatgttggg
1239541 ataatgttct gtgagccaag aggtctaatt tcaccacaag cagcttaagg tttgtcagac
1239601 aggacgttaa gtcagacgtg tgagacagaa tgaaagctga aggcaagaga agaagatctg
1239661 aaagtgtttg gtttgtttta ttgtctccca gcaacggggg tgattttggg aacagccgcc
1239721 ttgaccttct ctatcctggc cttggtcctc atcctggtca ttctcctctg gctggctctg
1239781 catgaacccc cccccagctc tctggctagt ctggctgttg gtaacagcca cagctcagca
1239841 cctccccgat cacacttcag gtacatgtga agtcaaatct tcaacccccc cccaaataaa
1239901 aagtttagga ttttcacttt ttttccaaac tttgagggga gacccggaga aggttggcgt
1239961 tgaaacggac gtgtacatga attacaccaa ggagtcctac accaacctgg accccaccag
1240021 tatggagagt gtatactcca gcatgtcctg atcatctaaa tacagcaatg taataaagtc
1240081 atcaagtatt ggtctggttt tgtctcattt cccctaaatg aatgtgaata tgagcagcac
1240141 ctgtagcaac acccttagta ttaagtgcta taatactggt tatggatgtg cgctggctcg
1240201 tgatgtcttt aggtgttaga aagcagtaat gaataggata aggataaaag actttattga
1240261 tcccacatcg gggaaattgc acattacagc ggtagcccgt ggtgtacaat acagaaaaga
1240321 aaagactctt atattatgta cgttgctatg tacattgtac agtatataca gaaattaaaa
1240381 ttatgtacag aatagactta acaaaacaaa atgtttaacc acagtggttt tttcttcttc
1240441 tttttcttct tcttgtatct gaagggtcac atgttttata gaaagccact ataagattct
1240501 ctttgcaaac ctgttattat ttaactggtg actaaatttg ccagagatga atttatttta
1240561 caatatcaat caataaccgg ctcagctctg ttagacacgc caccacacaa gtttagacag
1240621 ctgtatcagc acagaggccg gcagaacatc cagaatttgc cgcacatcct aggaaccagc
1240681 cagatccgcc tccaggttac agaatcacct ctgcagaact gtgggtgcag acaactgaga
1240741 tatacccgtg gtgacagctg cggggttcta aagcgaagga cacaaacgtc ctctgacagt
1240801 catcggggaa aacgtcccgg agtctccgac tccattttcg tcttccagcg aggccaaagt
1240861 cgacagattc atcctcccat ttatggtgga agagatgttg caataaactg gagaacacac
1240921 gcgctgcgag tagaacagga gttaaatagc agtgttcaac atgaaatcta aatgacccgt
1240981 ataggaaatg aacaaaaatg gtttgtcaca gtgaatcatg ggattttcaa cagtatgtca

1241041 caacagtgac gtccgggggc cttggttcac tgctgctgcc tgttgttgca atcacactaa
1241101 gagggggagg aaacggtggc tcaacatggc acaggaagag gctgcaggcg aggagctcat
1241161 catttcggtc aggttgtgga caccagctga ttctgcaaca gtttatggta cagtctgtaa
1241221 ggttcctcac acgtctctat gtgttggcgc ccgcttgtgt ctgcatctcc acctcctggt
1241281 gccccggcag cacctggctc tggttcctgc cgttcacgga atgctgcaag gtgatcgtgg
1241341 ttgtgcagcc ctctcctttc ttcagcttcc tgatgtgggc caggatgtac ctccggaact
1241401 tcctggtggc gaaacagtac accacggggt ccagaatgca gttcacgccc atgaggagca
1241461 gcgtgatctg atgcgcgtcg ttgatcgcct ggcgggtctc ctggctccag tccacgtggc
1241521 cccagccctg ctcgatctgc agcaccccca gcacccaggc gccctgggtg acatggtggg
1241581 gcaggaagca caccacaaag acccccacca ccgccaccag catctgcagg gccctgcgct
1241641 tgagcccccct gggtctctta gaggacaggg agtcgctgcg cagatggaca gatttggcgc
1241701 tggatgaaag cagcgcctta gcgatgagga gattacacac caagaccagg cagaagacca
1241761 cgaagaacaa tgcgatgatg atgaaatggg tggtggccac cgtcttcttc tgcgagtccg
1241821 tctggttctg gtagccctcg aagcagcgga tgacctggtt ctcgtctgta ttggtcccgg
1241881 gagccaccaa atgcgggatc gccatggaga tgttcagtat ccagatgaag acactcacaa
1241941 tgatgccgcg aagtctgtgg tccgaggagg cggcgtccag gggtcgggtc accgcccagt
1242001 accggttcac gctgatggcc ccaaggaaga ggatggagca gtaggtgttg atgaagaaga
1242061 gcgagcccgt gagccggcac atgaagtcgc ggtagaccca gtttccgagg cgatggtagt
1242121 agccgatcca gaaagggagg gcgcacacaa acaggaggtc ggcgatggtg aggttggtca
1242181 tgtagatgcg gatctcgccc atggccttgg cctcgcgcat gcagcgcagg atgaagagca
1242241 cgtagatgtt ggccaggagg ccgaggacga agatgatgcc gtaggcgggg gggaagagct
1242301 tgtaccggaa ctcagagtcc aggaagtcct ggctgtgtga cgccatatct gtccgtctgt
1242361 ccgtccgctg ttcatggtgg aacacggcag aaaacagcgg agactttttt agttccatca
1242421 ttcccaaacc tcgttattcg tacgcttcct ataatgttgc ttatgcgata tgtgtcaagt
1242481 tcgccacctt ctctcttttc tcatcttctg catctcctcc acaacatttt tgctcacaga
1242541 caaatctaac ctcattcaca atttcagtaa cgtttgtact aaagctagtc aatagaattt
1242601 gaagttacat taggtaaaat cttaagagac acaaggaata aaaacttacc agagcgcttc

1242661 ctcttctcta ccctgtgtga agagttgagg catcctgctg atgtcattcc tacagccagt
1242721 tcacaggaaa acctgtaatg ttgcaacgtg tctcgccacg cctactgcag cctctgatga
1242781 gggctggacc tgttgtaacc tggaagcctt tcctcactga agaagaggga aaagagggtc
1242841 taggaaaatt aatgatgatc ggttgtccaa catgattttt catgctttta ctggattatt
1242901 atattaatta ttattgtatt aataccatat gtccaaacca agtaaggtgt gaggtgttaa
1242961 gcgaccgcag aggaatgcag gcaggaaata agaaagaaac cattttgatt tacacaactt
1243021 ccgcctgcac tggggtaact acagctcgac ctgattgcgt aactgaccct caggaaacgt
1243081 cactctgcag gttcagtaaa tttacagcac accttctgat gtgtgaatcg tgaagagcgc
1243141 aacattattc aacagtttat attaagatgt catgtatgtc caaatttaaa ggaacttcca
1243201 tctgaagtga tacatgttat actatcgcag tgcttctcaa ttattttctg ttacgccctc
1243261 cctaagaaga aaacattttg cgcccccccg ccgtgactat aattagtata atttgtctat
1243321 gaaattgtta taagtacacc tctgcataac attgtatcct tattaatatt ttgaaaacaa
1243381 aaaaagaaaa aaatatagac caaagcggga tgattgttgc caatattcgg cacgttttgg
1243441 ccgaaaaaac tcaagcggct gatcagcgtg actggggtgt aatgtcttta agtgagggtt
1243501 agggttagtg ttagggttaa ccagggttag ggttaaccag ggttagggtt atcaacgtga
1243561 caggggtgta atgtctttaa gtgacgcctt catttatttg gcttcatgct gtcctgccaa
1243621 catttttaga cagtaaacac accggtcttt cctcgtctcc caccgtagtc gcagtgaagc
1243681 caagcgctat aaatgcttcg tcatatttcc tcgtcttagc tttcgggaga ctttcgtatg
1243741 tctcattatc tccgtctctc tccgcctttc ttttcatcac tgttaagtat ttttttcatg
1243801 ctgtctcttg gtggtttgtt cactgcactt cctatctctt gctctgtggt gtgtgagcgc
1243861 gggatgtgcg attacacttt attctagtac ggcaaaaaaa ataaataaat aacatgttct
1243921 ccggggtcac acgcgccccc cctggcatcg caccactatt tgagaagcgc tgtactatcg
1243981 gatgagggtg taatacaaaa gtcttaccac aaggtggcgc aacacgctta taaaccagtg
1244041 ttgggggttg aaaacgctgc ctctgtgatt tgaggccagg tgaggcgcca gacagcttca
1244101 catctgccct tgcatccctt gcagcttcgg ctacttatgt ggtgaatgtg acatacaggc
1244161 agaaaacaca gcacaggctc ctctccctgc tgccacagtg taattccaag atgaaggtta
1244221 acgagcctcc acaaccttat tgctgttatt atggtctggc agtgaaacaa gagctccttg

1244281 gcggttatgg cctgcaggat gtgccagaac tgccgtgcca ttgctgcata tacagtctga
1244341 cactattaat ttctgtttgt tttgtttttt gtcttcacga tgtgactctt tttttttttt
1244401 tgctttgggg tgtggatttg aaagattcca cagcaagtga gtaagatgga tctcagtgag
1244461 aagcctatgt cagacgtgat gtgctccaaa gcaaagcatc tgtaatcgtt ttctaatata
1244521 atacaggcag aagcaatcct gtagttttta tattcccaga cgatccaacc tcagtgaggg
1244581 aactatgtta catatgaaag caagacctta gatgaatata gaatgagaca aattagccac
1244641 agttgtgagt ttattagatt agcgtgaatg cattttatgg ttccatttca tcataaatag
1244701 aggaatatat catggaacag aatatgtcaa agcaaaacat tcgtaaatct gtataagtga
1244761 ctcttgctgc acaggtcagt ccgaagaatc cttccaccga ggtgtggctg tgccttctgt
1244821 gcaggtgtgc agccagaaat ggaatggggc cagaatttat tctttaatag gaatggcggc
1244881 tctcatccca gaacatctgt agacaaaaaa acagacttgt gcattcctcc gggagggaga
1244941 aagggtggac tctgcccgtc tacagctctc cgtgtccaat taacgcctat ttacagttgc
1245001 tccttcgcct caccttgatt cattgcactt tgcctccggc agatactgta tggaatgctg
1245061 tggtgggaag cgttacagga gggcaggaca tggcagcagg cctacagtga gcagaaagta
1245121 tgaagcagag ggagacaagt acctggtgga ggggtagggc tggagcgtgg ggcactctga
1245181 caggtttact acatgccaga ggcggagatt tgaggaatgc cctcctgcag tgagtgagct
1245241 cggcggcggg gggggggggg ggctaaacag gcccagtcat ttatacctct ctggatggcg
1245301 aaggaccagg atgattttag ctgaaaatat ggccagtttt taagcattaa acggcagtag
1245361 ggacaccata aatcctcctc ctgcatcact gattgttaaa atgatgagct gatgctgcag
1245421 caaaagcacg gaggggccat gttaagttgc tttctgtgtg tctttaaaag gggtcgctct
1245481 gagcattagc atgcctcaca atctgcccca cattctaatc catcagggtt acatctgttt
1245541 gaacactggc cgtagcccac tctgcaggga cagtgaatta agcgcattaa ccctcatcct
1245601 tgctgtaaaa gggacaaaaa gtaggaaaat tacagcgccg gccacacaga ggaacttaat
1245661 cctcctactc ttgtctaatt gtatttctaa agtcctcgtt tcgatgaggg gaatggcgtc
1245721 aggtggctgc gtcgacctga ctggtgtctg tacacgatgg gttttttttg gtgacctggg
1245781 ccagattatt agagattagc ccctctggag aagataatgt gctcagaaat ttgtaataca
1245841 cacagtgaaa ttgcagctgc tcccaatccc agagagtatt tgataatgat ttgcggcgtt

1245901 tataaacccg gtgggacaaa cggtggcgaa ggatgtttgt gaggaggaag gatgttgggg
1245961 ctcttcctgg atcagcctgc ctcggggtgt atttgcagca tttgtccccg cagccttgtc
1246021 cttataccgt agtttcctgg cattttgcat gtgcttgcat gtgattttta tgttctcaaa
1246081 aagacaaaga tggcctcacc ttcacagaat tggtcgatat gcatggattt ctggacttga
1246141 acctctctga taaggccctg aaaccatatt tccccctcaa actgtgcctt catccaaact
1246201 ccccccaaaa acattagtaa cataaagaca gacatgtaat ttgtacagaa tgtatgcaga
1246261 cagattactc acacacacca gggcttggtt ttcctgctgt ggctttcagg aatctgggct
1246321 tgcctggcta tgccttgact ttcttttatt actttccccc atccagaaag aacaccgtgg
1246381 ctttgtttac ctcgccatgc tcgcgccatg ccattattag agagggaatg gcagcgctgc
1246441 tggaatggct gcaggtcatg ctcctgaatt gagcccagcc gatgtcattg atagtggtat
1246501 tatctctttt caggggggaaa attatatgtg ttgtgatttt ataatagtag gcttgcattg
1246561 atgtcaacac aacagagctt aaaaatgtcc attttgctga ttccctacta ctagttccta
1246621 ccctaaacag gttaaactgg tcaggtttag agtcctgttt aagagttacg gttagaagga
1246681 ggcccacaca gtgtaagact gcatgagtgt gcatgttctt caggggttaa aagccatttt
1246741 atattatgac tccattcatt tacccgttat gcaactgaga gcaataacca aagatgaaat
1246801 cactttcacg ctataatcat gtgctgtgtg attgcagccg gctccatagt ttcaataaaa
1246861 agagtggcac cgcagattga taagcgtttc tggtgggatc agcattctga gtggctcttg
1246921 gcttgaaaag cagagagaga gagagcgaga tctgagtcct gcaacctgct cggcaacatc
1246981 agagatgacc cgggtggaaa ggataggatt aaataaagcg tcaacccgtt tgccacgaca
1247041 atggtgcagt tttcccccac atacaacact gaaacactca ttaatcacct attatgcaca
1247101 catttaatgg ttaatgcggt gcacataaat taataaaaat acagtacaca taaattcttt
1247161 tgggtcatat ttttgaaatg tgtgcaacat gtatatattt taaacggcta tcatgcagtg
1247221 atttattgcg tatcactaag tgctcttagt ttacgctgtt gcaaaaccct ttttaatacc
1247281 tggagaatag aaaatattca gcgggagtta aaatgttaca ttgtctgatg aaggccgagc
1247341 gctccgcggt gccgcagctt gcaccgagtc ctccccgccg gccggtggat gtggtggcgg
1247401 gaggcgggac ctgtgggccg ggaagggcgt tctctccgcg gtcagactgg gcagagtcat
1247461 cccgaggggt gggtcctccg gcggcccaaa aaaaggaaaa cccacctttt tttcattgtt

1247521 aaggacgcgt tggaatgtga tggtttgatc tgaaacgagc cagggaggtg gatatccgaa
1247581 aggaaaagag ccgacgaaag tgaggagaag gacgggagcg gggaccgggc gcagctccgc
1247641 agggcctccg gagcggatga acttgtcctc ggcttcagcg agtcctgccg ccccgcatga
1247701 gctcggagcg gggaaagttg tggatatacc gcggcgtttc ttcccctctc ccggcagcgg
1247761 aagagccgag gtaaagttag aattttcctg ttttcctccc cggtgttggg ctgtgtgtgt
1247821 gcggggggga tgaggcgaac aggcgggttc ggtcagcggc ttcctgagtc ggaccgagtc
1247881 tgaactgcgg caagtttttc accctcctcc cccttctgct tgtcctcctc ctctgctcgg
1247941 cgaagcatcc aggactgcgg cgaaagctcc agctggtgaa actggagacc cttgaaacat
1248001 ttcacagctt atctttttgc agacatttcg atgtttatgc tgaacatttc caacgcgctc
1248061 ggcctgtgcc aacgcgcttt atgacgcatt taatacaggt gcccatcgcc gcttatttcg
1248121 tccgtaatgt gtgttttaat tgagatgata cctgaaatgt gtgtgttgtt cccccgctgt
1248181 cacccacgtc atctagtttt atcaaacgtt aaaggaaata cccttaaatt cctcgccaaa
1248241 gatgccaaaa tctttcggag tcttccatga ttagccatga cacaagctag ctagatcccc
1248301 aaattcactt gtttctttac cttcaggtgc aaagaagatt aaatattgtg tatgtacata
1248361 tttgagcagt gaaatgggct ttaaattcaa cattttgaag ctcaggaaga gcgaaaatga
1248421 ggtcatgcag aacagggtaa aagtgatttt catactaaac atggagctgg aagatcagct
1248481 gttatttaag ctattttagc agcctggaac tgcaaacaga tgccttttct tgtttttttt
1248541 tttttttttt tttttaaaaa tccctttaga gtatctgcca gctgattaca cgtaaatcta
1248601 gtgctcaagg tcagtgagga aacctgcgat ggcagaacat ccgaggagaa aatgttgtgt
1248661 tctgaacaaa cctcttgctt ttctcccttt acgcaagtgt aatgtgattt tccttctgtc
1248721 gcggagacgt cagccgatga atgagcggtc tgaagatgct gcctgcaact tctggaaaca
1248781 ctgacaggca tatttttcca caaatgccaa ttgattgtag cccctcttaa ctccagaagg
1248841 ataacttcat tagctcaaag ggggagtttc ccccttcctt cccaatggcc agagcataaa
1248901 aatgcaaagt ttctggctcg ctgtgcaata tttgcacaaa acatcttata ttaagaggcc
1248961 aaacctgctc tcctactttg ttgatttgtt caaccaactc cggagaaagg cgtttgtctt
1249021 gcgtcacgtg agctgacata cgttgcgcca cgcggtcaac agacgagaga gagcgaaacc
1249081 ccgactgaac aatgtgcccc tgtgctgcct cgccgcaaca cagctgactg tgcaggtaga

1249141 aaggctggcg gtagtggcag aacactgctc tgccaggtgg ggctcgtaaa
gaacagcact
1249201 gggagggaga gcgatggagg ggacctgcct tgacctgttt cagacacgaa
ggagacgcac
1249261 aaaacaacaa aaaaaaaaag cctctgatga ctttccaggc tggctcccag
ccctctgtgg
1249321 actacaccag cttagctacc aaaccggtgc tcctgtgaaa ccacttttcc
cctggagcct
1249381 gaacaggcac ttgtgtggca gctgaaaagg ggtccctgcg tcatatttag
gcgaggttgt
1249441 gacaccgcat gtggagggaa tgtctgcatt ttatctgaca gtttgggatt
ctcggggggg
1249501 gggggggggg gaatttaacg agaataccag cgaaggactg ggcgagaggc
tgctggatcc
1249561 gtgctccccc cgacgttact cttactgaac atgatggctc agcgctgcga
gaccaggtgt
1249621 taacgttagc ccaccattat gggtcttttt gtgctcgcta gagtgatgcc
gagctgtgca
1249681 aatctcacct gcaagccaag ctaacaatca cgcatccatc agtgcagctt
tggatgcaac
1249741 caaaatcatc attacgggtg tccaataagg gtaattatca gactaagcgc
tggacctggg
1249801 ggattagcga gcagctaaat gactgccccg gggattaaat taagaaaaaa
aattgaggtt
1249861 ttctgcctga gggaatcaaa ccgacaggaa tgttggacct agcccgctgg
cggaggtctc
1249921 accaacaccc ctcgcaggga gggttgtgca cagaggcccc tctgctggtg
taggactgtc
1249981 acatcgggat ggaattacgc tctataaaca gtgatgtcat gtatttttat
gatattgctg
1250041 cattttcaac tgttcaaacc tcccataaaa gtagcacttt agcttctgtc
actggcgttt
1250101 ctgcctcggt ccagtcgtcg gttaattgat gatttaatcg tagcctggat
tagtttcagt
1250161 cccagcgagg cctacacttc ctcggagctg taacagatag ctcagatagg
atttgaagcg
1250221 gatggtgttg gtttgacatg gttattatta gtggtatact cggggcctcc
ttgaaatagt
1250281 acagatgaat caggttctcc gagagcatga agcccacaca cagccgccgt
ccccttttgt
1250341 gagtgagcgc gagtgtgtgt gttttgtgat cgtgcaataa taggctcggt
atccgggaca
1250401 gaggctgctg ttgacaatac gcaggccagc tgtagctgcg accagacacg
gcgccgtgcg
1250461 cacgccctac aaatagttat ttaaaaaaaa aaagagacat tctgctcatc
atttgtggag
1250521 gtcggcgtgt cctcgctcgt ctttttcctc cacgttgttg ccactcgtgt
caatctgtct
1250581 ccacgtcatt taaaaggcac atccgggagc gttcgcgccg ctcgccgagg
ttctcgttaa
1250641 ctcgcatatg ggccgttgtg ggtgttaacg ctttaacacc tctgcgtcat
cacaaaaatg
1250701 gatccttttc ctacgagctc cctcacaatc gcaccgctaa ctaatacact
tctccaccat

1250761 tacagtatgt gtaccctttt taactcgcac ctccggtttt tcctgctaat cctctggctt
1250821 ttaagttcat ttccagggca gttatcgaac ctcgtaggtg atggggaggc cgggaagaaa
1250881 ggctgatgtt taatgataaa cccggtccat ttaatgtttg gtgagcaggg atgtggagtt
1250941 tgagctgcat tttcatgtct ttgctgatgt caggactgca aagattccga ttcaggcctg
1251001 agctcttaca tgttgtggtt ctggttccca cgcttcaccc tgtgctgagc ccttgtttat
1251061 ttagccaaac actcctccct gaagtgtatt tgtggctgtc agatagcttc aggtgttctc
1251121 tgcaggtttg taatgtttaa tcggcagcag agccgggccc ggcgccccac gcggctggag
1251181 tttggaccat aatccgggtc accgtgctac tgtgctgtca ggtttcatcc ttctgttttt
1251241 ctttcccctc gccggttcta gcactgtttt tttttttttt ttgggagggg ggggtttcag
1251301 gctccaataa gttgttttac aaaaggttcc ctgtgcgtgt gttctgttcc ttcaccggag
1251361 ggaacggctc cctcatcaat cctaaaaaga aaagagagag tgaggtgtga gggaaaacaa
1251421 gccggagaga gtgagggaaa aaggagagga gacatgttta atggatggaa gcaaatgtaa
1251481 aacattcctc tgaaattcct caaccacact tattgtttta gctcccttcc tcagtatttc
1251541 ccaccttcgg agtgttgcct tatctccttt ctcaccatta ggcctgtaac attcctaaat
1251601 atcctctgct tcacacgtat gttcaggact taatgaattt ggaaatccta aacggttccc
1251661 ctcaacatgt ctgggtcagc tgcgtggaga ccgctgttgt tactgtcttc tgccagttca
1251721 tgggctcagc gacgtgtaca gcgcaggaat tcggacgggt gggggaataa caagcagtcc
1251781 ttcgtcttca ggaaagggga ggaggaggag gaggggggt ctcaggggca tatttggcat
1251841 agttcagcac cattccagtc tggttcaaga tgcattcttc tctgaggtct ggccggcccg
1251901 ccagaccgat cattgtcaag gcaaagacga tcaccacaca caccgtgagg tggcagcgag
1251961 ggcagaaaaa ggggttttct tgccacctgc agactcgcgg ggacgacctg ccacccagcc
1252021 tgcaaccacc ttaataccta taaatggacg cagtcggtaa atattgaagc ttcagccagc
1252081 agcagggttt gtgtgtcttg ggcaccatca cgatggttta atgagtgttt atgtgcccag
1252141 agctataata cgtctctctc tgcctttctt ctgtagaatt atagttacag cttacgctcc
1252201 gtggagcttc tgaactcccg ggctgttatt tgcagctgga cagctgctga tatcggtgcg
1252261 gcagaggttg ggttcagcag gaggtcttca gagtgtgagt ccagatgacg ctgaactggt
1252321 ctaacagcag acaagcgcct ggcacttcct gtcttgcctg tgatgaggcc ggttcagggt

1252381 ggcgcccaga caacatcaga cgtgccggcg gcttcgctgg cgctttgatg
cgtctggtgt
1252441 gttttcatct cactgctgtg gatgaggaaa ccacctcaaa tgggggaaat
aaaaggagct
1252501 gccagtacca atcagcgcag gacagatgct tccaaagcgg agcgtttgag
gagcaagaaa
1252561 gtgacgcaat cctctggaat ggagttgacg gactttggac tcgtgaaagt
tgtgtcaaca
1252621 tgatctgaac tcatccccgt acgttgacgt ctgcgtttgg atcctcacac
gctccttctc
1252681 ggcgctcaca ccttgagtac acggaggacg tgttctcgca tctccacact
cctgactcta
1252741 atccgaagat ctccggggtg gctctggaaa tacagttcct tctactgatt
atttttcttg
1252801 tgatcagaaa atatacacgg tctcagagta ggagtgctca gacaccacgg
cggctttggg
1252861 aatgctcact tcccctcgta tatttctcac aggctctctt gttttgctcc
ctgttctcct
1252921 tttttgactt tatgttgcct cattctatca gtagtcttga tgacatttta
gaaactgaac
1252981 agttcccagt tggctattat ctgagcaggt ttgtgtccca ttggtgtgtt
tgttcaccca
1253041 tccctctgtg tttgtgtaac tcttgtaatc ctggggtcaa tgcatggaga
ccctgactgc
1253101 ggggctattt tcagcctcct tttccttcct ctgaatccct gttcttcttt
atgttgcgtt
1253161 tcttattgga agggattttg gccggagggt ctttcgtgga agttggcaga
cagtaggttt
1253221 gactcctgaa taccaacctt tactggtggc gtttcatctg gggctaaaag
tcggaatcac
1253281 gtggcgttag gcgacaaagt ccacaaaatg ggaggaaaaa ttgacagtat
ttccacagct
1253341 attattcaaa atgcctgtca aacacccaca gaatctccga atgtgacatc
tgcttcctcc
1253401 ctatttctca ttaaagtaat agttaattaa aaggcgagct ttaattgcta
ctggttcaga
1253461 gccggtcgcc tggctcggct acagtcagac gtatgtttgg ctccatacga
gctgtattca
1253521 gacgcaggag cctcccagga atcctctaac acccctgagc ccctgagtgg
ctcgtttgtg
1253581 cactgggaaa cattgtgaaa taccaggcat ggtttatggt cctgcctgta
ggaccagcac
1253641 cccctttttc tcccagcatc tggggctttt tttgggcggc ccggccgggt
tcacccctca
1253701 accagtcccc attaatttac acgtttgata tattcatcaa cacaggttgg
tttttactct
1253761 gagatgccaa agtcactaca ggaagatgtc cgtcaaaact gtccatctat
tcagaaatac
1253821 ttagtgggat taattattgt tattttactg cataattagt gtaatcagtg
ctatttatag
1253881 ggacttgtgt agtgacagac tggccaccag tatatcaatg aaggacctcg
gccccactct
1253941 gaagaaagct aggtttataa aatgataaac tgtccatgtt tactgtcaca
ggtgggttat

1254001 ctttggcatg taaatgtaaa ttacaggtct gaaaacaaat tagtgccaaa ctgttttgaa
1254061 tttataattt gtggcacctc ttctagttgg tgtaaatgtg gtactttaca tgcgccctgt
1254121 aatgttttaa ttcagtgttt catcggcacc attttgcctc tctgatggcg ccggtacttg
1254181 tcctaaatag cctctctctg cgatggcatc tccgtctttg cctccttttc catctctagg
1254241 tgcgagctca ctgaagaatg cattaaatga gtgttttatc tctcctacag gcctgcttca
1254301 tctgcactcg cagccatgcc aaagcattcg agacacgatt gaccttaaaa ggaaacaata
1254361 gcggcctatc aattctgctc tgccagtcaa ggaatggcct gctgtggcct gggcagggtc
1254421 cagtacacac aacagaagga agtgtttgga aaatggagcc agcagggaaa gtttctcttg
1254481 tttttttat aggtgtatac tgcctgttca gggattgtag aaataccagg ataatgggtc
1254541 ctaacgcagc cctgcccacc ttttaacatt cctgatcaag tctttttatt cacaaggtct
1254601 acgtgtcctt ttactcacta aaggacaaaa cttgatgact aaaaaaaaaa ggttcgggag
1254661 ctttgtattg agctgtgtgt ccgtcagtga catcacaagc tgtattactg acagactgat
1254721 ccgagttatc aggagtcctg catgtgaacg tctcagctgc tgtttgcgga actgtgagga
1254781 cgtgcactgg aactggacac ctgtgcccag acaaccccac agctttcact ggttcccagt
1254841 gggcagttaa gagggtcgtg gagtcccacc tggcatctta gctaaagctg acaggcccgt
1254901 gttgtcattc agctgttgtg gggttcatgt gcttcactct ccttcttctg tcctttggtt
1254961 ctgtctccag ccgccctgct cagtgttttt gatctgggca tcccattttc ctcctcagta
1255021 gacaaaacat tgtccgaccc ctctgaacca taaccctcaa tccgcactca cgtacacaca
1255081 cacacacaca cacgcacaca cacacacaca cacacactct cttatactgc cttcttctaa
1255141 tcacactcag gaaatgggct ccatacagac gaggcccgcc tgggcagtcg aactctggat
1255201 gtagtttcag gctttaaaat attctccata gaggaaattg aacacgatcc cccccccaca
1255261 ttctctcttt ctttagcttc ccatttttc acgctacatt tatatgcact aattttaaat
1255321 gtccccctt agaccaacgg acagggagaa tctggtaccc aagggctcga cacacacaca
1255381 cacacacacg cacacacaca cacacacacc gagtaaaaac tttagattat tggaaacatt
1255441 catgtgtgtg tggacatttc tgtgtcagtt cacatatatt agaaaaacat gttgatgtaa
1255501 tggaagagga agtgtgggga ctgccccaaa acagattata attacaccaa acgctgacag
1255561 agtgtgtatg aatggaaact ttccacgtgg gaacggtgct cagtgtggcc atacatgtgt

1255621 attctgaatt ggaacagttc cgatcggatg agaccttcaa gtatgtgatt taccagtaac
1255681 gttccccaaa cttgacacgt tgaatggagg agagatgctt tctgtgtcat gaggccacag
1255741 acggacttaa tatctgaaca acagcgaaga cccgcgtgtt gatgtgagca acagcgttgg
1255801 gttggatttg aaggagaggg tgacctgcga gcagctgagt ggcaggagcg actgtctctg
1255861 cccagccata tctacagtat caaaggctgt ttcggcacat cactcacgtc tggtgtcaac
1255921 atttcattgc cagtgacatc agcccatttc agatttcatt cccagccgct cttgtcgctg
1255981 agctttcaga aatacccgga catggcgttc ttcgttaaaa gattagcttg tgcactcatt
1256041 cacacatgta tgtcttttgt actggtatgc ccattgggcg cttgctgttg acagaaatct
1256101 tctttcttct tcagccccct catccgtgtc tttccccccg gcttttactt ccctttcata
1256161 tcctttaaat tcactccaga ctccaatctt aaatctggat tctctgtgct taaccaaaca
1256221 tatggaatgc tactgtagtg gaatgctcca ggtctgggac gtgtgtgtgc gcgtgcgctt
1256281 gtgtgtttcg ccaggcacat gttttgaagc ggatgagagc ttctaagaag acgatgttta
1256341 tgataatggt gcgtgtgtgt gtgtttgtgt gcgcggtgcc tgtcctgaga actgaatagg
1256401 gccttctgtt gttgtggcca aacactgcct gtctgctcat gcatgagtct attgcaaagg
1256461 tcctgaatgt gttccatatc tgagcagtta gctcattctt agcttgttag cttgactgcg
1256521 aacgatcgcc cctacagtga gcgcattgtc agctcggcct caggctcggg gccttgccaa
1256581 gtgccaaaca taacaatggt gaaagatgat gatgtgtgtg tgtgtgtgtg tgtgtgtgtg
1256641 tccactttga caatcctcat ggcttgattg tgtgttttca cctacagaat ggagactttg
1256701 gaaggacgag attaatgtgt gaccgataca aggatgcatc tcaaatgtaa gtagcctttt
1256761 tgcctcagtt aaccctgact ttttcctctt ttccagtttt tgttggaaac aaagagtcta
1256821 tcaaaattaa atttgatgat ttaatatatt aaagtcccgc aaaatccagt aataagacac
1256881 tttttttgt tacaaatggc caaaatatat attttggaga cggatgtaaa attgtgttgt
1256941 ggacttggtt tgagctgatt aaagaactag agggtttggg aaataagtct tagcttaatt
1257001 ttctctgaga ggctaagata agaacactga aatcacattt gtgcatgagg aaaatcatta
1257061 acctgttatt tactaaagtg caaaagtcca gctacgggct tctctaaagc atcgtcctct
1257121 ggttttgttt tcgacagcga ctgatttctt gacaatgata ctgatgaagt tctggtattt
1257181 ttgtaattct tttatagcac cgacactaga ggaagggttg atattgcaca gaaataagta

1257241 gaataaagaa ggagtccaga ttctaataaa tcactttaag atatgtcctt cccccgctgc
1257301 tttacccacc ttttgtccct ctttctcttc cgctcctctg gtctttgtga gtttatttcc
1257361 cgttgggcac aaacgccggc gccgcacttc cttcccagca gcctgagctc acgcaggagt
1257421 ggcaggcatt cgttgcactt tatgtatgtg gaacacagtc atattcataa atattcgcac
1257481 tcctcgtgct cagggacaag ttgacctttg cgtgtgttag tttatcttat ccggccttag
1257541 cgagcgctga cagaacgtgt cgctgtctcg ccaacatcta acagaacagc ttataatcaa
1257601 tatgtggcga aggtgtgttc ggtgcatcct cactgcattt atctgcattt attattccgg
1257661 atgctggcat ggctcacatc ctgttcaagc tgaatattgt gaaacccaaa acggggaatg
1257721 gatccagcta ataaagacac ccctgattca gccatgtata tggctgttaa agccaatcaa
1257781 ttggtccttg gactctaaac tattaggcaa cgattaggaa aatgctggtt tatctcatac
1257841 tttacggaaa gaattaggat taggactacc ctaaccagtt ggaagatttt cactatacct
1257901 tccttctaca ccctccactt ttgttgttac accaaatgta gacagaaatt ttgaaaagac
1257961 tccttttgcg gagctcaaaa gtgtagcttc tctgctgtga ggccactctc agtataagcc
1258021 tatgaggaaa ccagttcttc gggcccctgc gtcacgtgac ataattacac tgtttggcca
1258081 ctatgttaac ttggcttcaa aagcctggca ctcttcctgg gggctttgtt tccccccaagc
1258141 taaatgtttt ctggccatag cttcatattt acataaatac acgagcatca attttccccg
1258201 agaagactga ttagagagcg ttttgttaaa cctgtttacg tgcgacagcc ttattatctg
1258261 taaaacacgt ctgcatgcta acatcagctg cgttggcctt tacccccttat gcaaacccct
1258321 ggatgcttgt tgggattcca acaaaagcac gatgaatttg gtatccttgc tggtaaaatg
1258381 cacctaagat ttcctttccg aatgccaaaa tgaggtaaat gcaggtactt ccctcctgct
1258441 agcgctgatg ctctccctcc ttcctgtagc tagtgctgca ttcctgctgc agtaatctct
1258501 ggcagctacc tgctctgcat tctgcagagt cacgccacac gtctcgctcc tgcctcgttt
1258561 tgctgacgct gacgcaggca ctttcttctc cgctccctgc tgagagagcc accgaggtct
1258621 ccgttcatcg ccaagtctca ggctggtgtt gcattaccag actctgccag cagccagacg
1258681 gcgggcgggg tgaagagcca tgcgctcaaa ccccctgacg tgtccacgaa ggagctgttc
1258741 agatagaaaa gtcgtatatt ttgctgtcat ctcatctcct caaccattag aataacagta
1258801 agtcaatcgc gccagcgctg atacatttaa atcaattcat taatttaaat gcagaggcag

1258861 gatttccttt ctattattta tataattaca gagttatgtg cctgaggccg tggcttgatt
1258921 taagcccata cactctataa cccagtccat ctgtgatgag taaataatgg atccgaggta
1258981 acattttttg ccataaatct cctctcaccg ctttgttctg tctgctaatg catttgctcg
1259041 ctccatggca cctggtttat agttgtattt ctcacaatct ccgtgtcagt ctgcagaaac
1259101 cctccttctg taacatttcg cagacgccac gaccctattt cgaactggca cacggcataa
1259161 aacagttatt cagcaagtca aagggtttat ttgcgtgatt cccatttctg gggctttgat
1259221 tatatcattt taaatggtaa ctgatgacaa taagcaagca atttgtctga ggaacccact
1259281 gagtgtttgt gtgtgcagag cctttgtcca actccagggt gttcttggtc aaaatgaaac
1259341 ctttgtattc tgtcagcaag cagtaaaagt agtgatttct ttgggacttt acatttaatg
1259401 tgcacacaaa aacacaggga caacgtgatc gttccgctgc aagcaataaa aatgaaattc
1259461 agttggaatt acagtgataa cgtggctgtc tcactacttg ggtgccttca tcaacaacaa
1259521 tcaatgtaga taattaacat tgcatggttc ataaagggct gtaggaccat ccccaacttt
1259581 ttccactgga gaaataaata accacagcat agcaaagtct acaagtatta tttttattta
1259641 ttcattaagc gagtctagtg ataatctagc ttttacagtt ttatgattag atataaagcc
1259701 gacagcagaa tttatgtgct aaggctgcac attcctcaag gttatgccat atcttaactt
1259761 tatttattcg ttaaagaaca tgtccataat tcctaataca tatactgtac ctgactcaat
1259821 ttatgttgct gtgtgtgagc catcactcta actccagacc caaaagcctg tctgttcttt
1259881 atgacccgtc gctgacgctg ctgacctttg tctgtctacc acaccctcgt tctgtctgac
1259941 tcgccctgta tcacagtctg cagctcacca gggcttaatc tacgtgtcat caaattgtga
1260001 tggcttcata ttctcttatc agttggacgc agcgaaacat gcacatcccc gctcaaggtg
1260061 ttcatggcgt tgcgcggctc ctcccaagtg tgcggacgct gacgtctcct gacacctgag
1260121 agctccacaa cagctgttga gcttagcttt aaataggaag tcctgggaga agctcctggt
1260181 aatggacggt cagagagttc ctcgtaattc tgctcatttc tttattatct gtcgccagag
1260241 ggtttgtcct gttggtttgg actttctaaa aggtggacag tgagcacaca caatgagcgg
1260301 ctctagtgac tttcattgac acgaccgttg ctcttttccc agcaaacaca tctggaatcc
1260361 tgcttttatt ctatttgagt cctgttcctg ttttatcatg caacactaaa ataactcctc
1260421 ttcaatttcc tgaccctgca tgtgaatgtg tgactggcag agatacgttt acaggagggc

1260481 ggcgggctga gctcgtgcta gcgtagaggc agttgtacaa accaaaaatg atgctttcct
1260541 ttattcatta ttaacattca gtgtaaatat tcactctgat tgcagtttct cccctaagaa
1260601 catccggaat aactggatgt gtttttgtg gggttgtttt tggggaggaa cttacatccc
1260661 ctcccgctct gtttctactt tagagcagct gtggccgctg tgtataatcc ttcatgtagc
1260721 gctgatatct gatcaacaac atgctctaat ttagcgttta gcgaccaagc caagcttaaa
1260781 agctccgagg gattcgacag ttacatctgc aaaagttaaa cagcctcgca cagaaatttg
1260841 gtctaatgtg tacaaccatc atacttgcat tagatccaac ggggcagaca acataagtct
1260901 tgtggaaatt cctccttcct gctatgtctg ccccaccatt gcattaaacc tctggtagtt
1260961 tccctttcaa cccctctggt attttcccct ctcgtcttgt aacgctgctc ctttacatat
1261021 attgtacagg ctgtgggttt aaagctgagc caggagaccc tttatgtagc cagacagagg
1261081 aaacggagag acgaggcctt gaaaagccat aagaagaagg ttagtgggct aggacatgtt
1261141 ttgaaaggct ttctgtaaaa ttccatttga aattcccgat ggctgccaaa ctgaagcatc
1261201 agcacttaat gacccattaa tgagacaaca gccgactatg tttgacactt gtggtaaact
1261261 ccagcaagaa tatgtgtttg atccacagtt ctgtttgttc taataaagcc gagctatgac
1261321 agtgcagcag acaacaaaag gaacattgtg ggatgtcctt gttctcgttc tgagttagaa
1261381 aaaacaaaac atgaaggcca tgcttcagat cttaaccatc ttgtggcttt cattgctaca
1261441 cacacacata gacacacaca cacagacgcg cacacacaca cacggtctta aatgactaca
1261501 ttattggggc ctttacattg actccatttt ccattcattt ctgcagccta atccaaaccc
1261561 tcacctcaac tccaaccaag cttttcctca aaccttcacg aaacctcaat tcatgtgttt
1261621 atcttaaact ccactgtcat catgaaatat aatgattaaa atttttgacc caaaattacg
1261681 ggccctttac cgtggttgga ccccacaggg tgacaaattc agtctggcac acacacacct
1261741 ttatatggac acaagctggt aatgacacaa aggtgtgatt taagcatttt gatgccactt
1261801 ggggcccttt attaaccatt tacagggcag aatagcttca gaaccacaag taccaaccta
1261861 aagtcaagtg caatggagat gggatttttc ccccatttct tccctctctc tttcatagag
1261921 ggaacagaat gtactaaagg gagcagtcag acaggaagag agtggctaaa gcttaatgat
1261981 atggtgtatg attgtaatgt tgctgcttgt gagactagaa agagagagac ctttttcttt
1262041 gtgtttgttg ttgcaacggc ctcagtttgg tgttgttggt ttaagacaaa atgaatgcgt

1262101 ctctggttac tttcctctcc ctttgactgg ttggctaatt aagtttcacg
tgattatggt
1262161 taatttgtat gattctgtta atgttcattt caagccaaga atatgccagc
agccgcagtc
1262221 tcgtagcatc gtctttttgg ccacatttac ttgcaccagc tctatttaat
agggtaccga
1262281 ggtctccttt tcagcccctc tcccctctga agtgatgtgc atgaagctgt
gctggtacct
1262341 gtgcttctgc atgaacaggg gcttcaaatg gagccatgtg taagaacagt
actgagtgca
1262401 cccacgggtc acataaggtg gggtcaaaca aacgtgagac gtgaacttcc
ctagtctttt
1262461 ttaacaaagg ctgtgagcct ctgcagaagg tcaatttctg tcaaaatttc
aggaagtgaa
1262521 agccgaaaaa agagcccggc tcaatggatg agtgagcaga tgtttaccac
accgctggga
1262581 cagtgaaagc aatgcaaaca gttgtttcaa atagaaacca tattactgga
attcttgggc
1262641 tacacaacac agctccaagt agctctgttt taagtaaagc cctgaggaca
ctcgggggaa
1262701 aaaagtagaa atatttttat attctctcaa gggtctggct ataaactcca
gacaatgccc
1262761 tgtcaggcgt gggcttggaa caagctgtca atgacctggt taagagcagg
cagccccat
1262821 gttaggcagt tgtgaggaat gtgtttgcat tgtagaggtc ggtttacatg
cctaaaaacg
1262881 ttacccacct ccaggttttc ctcttgcctt gtcattcaat actagtgttt
gtgtcagtgc
1262941 cctttaagga acaaagatta ttgatagata ttggccttaa tcgtacctgt
aagaaattcc
1263001 tgacctgctg cattccagcc tttttggacg gccctcataa ttctcccctt
tcttgtccag
1263061 cgtgttgtcg gtgataagtg aatcaggtat gttgattagt ttgggagagg
ttggctcttg
1263121 ccagagtcac tttgcacgca ccgccgccca ttaaaagcac ccacttggct
gcgctggtgt
1263181 gcgtgtctaa gagagatgga ggggagagat gagggaggga ggtagagagc
tggcaaagaa
1263241 ataaagaaag aatgaaagga gtggtaggca gtcggccaag gcaggagtag
ctggacctaa
1263301 gcaaacacca tgggctgccc tcagaattgt tgaatttacc gaaatcagtg
aaatttacgc
1263361 gaatcgaccc ttcggcgatc ggagaataat caagaccaaa cgtgaagtac
ttcattatta
1263421 aattaaagaa gtggctcttg agtcttggtt aaatttcctc taagttgcat
ggtgttgtgt
1263481 gaagagctgt cacaaagaat tatacccgga gtgtgccagt gagcgcactc
cggtttctct
1263541 gaatttttgg gaatttgtca cagtgctttt actctaccga aacaagatca
gtcaaggcac
1263601 tgttggatct gttcatccac ttgatcgcaa cagaggaaag aaatggcctc
acctagacat
1263661 agatgggtat aaatgatgcc tagagcttct ttcttcttcg cacgttacca
cactttcttc

1263721 tctttaagtt aatttacatg aggtatttcc atacaggcct gtattctgct gagattaccc
1263781 aaccaccaga cctcatgaaa gagtaaatta aatgttacag caaaaagatt taagtttaag
1263841 atttttttca ttaggtaatt gttgccacat tgaccttcaa agattgttgc ctcggtcaag
1263901 ctgttaaatg aatcgtggga cggcggcgtc aaatgaaatg atagaaaatg taaaactcct
1263961 ctgaagtgaa ctgtaatggc aagttgtgtc ttgttttatc tccgacatga aacgagggag
1264021 cacatttaat aaattggtgt ggtgagcagt taaagtgatc taaagcaaga atatgatgtg
1264081 catgtaaagc taaagcctta aaacagcatt tgtcaagcgg agcgaaaagt gatcatgcgt
1264141 gcaaatgtga agtcctgccc tcgggtcgat gtctgccgcc gagtcatgag ggttgaatga
1264201 tgaatccttc agcagaatga tggccaaagc agagacgcag gatttagaaa gagcatttat
1264261 cccctttatc tttcattttt atggaagata tacgacatgc tgtggaatga caagtccccg
1264321 tgtccacacg cagctggaaa catgtacaac accctggcta acagtgcttc acaccactac
1264381 attatgatat taggttttta taacacccag gagcaagcgt gaatgatgag gtgtttgtaa
1264441 ggcaatgtgt aacggttgcc acagcgacgg ttgcgtgtgt gcagtctgtg tagatatctg
1264501 agagatgctg ctgtcagcag aactggcaag ctagaggaaa tcgataggga atacatcagc
1264561 agggcgtgcg gcagtgcaag cctgtgcagg catggtgaaa ccctcatact catgcataca
1264621 tgtaagcagg gccacccccca tctcctttaa aggtgaagca aggagaaaca aacagtggct
1264681 caatggcaaa tggaggatgg gtgcaaaaat gcaaacaagt agaaaactga aatctctttc
1264741 tggaatggat ttgttttcaa gtgtgcctat accgtagagt agacggagtg gtgtgtggcc
1264801 ctatttagtc tccttctata atttgggtta aagcaattag atggaagtgt gaaatcacaa
1264861 ggtgcagaat gctgcgtgta cactgtgctt tctttttttt tttttccctc actcatcaag
1264921 cgaacctttg agaatgcaaa tgactgtcat tgagaaatca cggctaacta agtgttattt
1264981 cctctctccc tcctccagat gacagatttg tccgctaatg acccctctga aggaaaacca
1265041 gccttaaagc cacagtcacc aaaccaacaa actgcgtaga taattaagga cttcccttta
1265101 aacctaaagg aattcatgtc acttttacac aaatgcggac ctcctgaaac gtcatcatat
1265161 agtctaaaca cagcaaacac gggatataac ttttaaactt ctgcagccat gtctgatagt
1265221 aaagacatga cacaccgtca cctgcggcac aagctgcaga gtctcagccg acggctggat
1265281 gaactggaag aagcgaccat taaacttcaa aagtccgagg atgaacttct tgatttacag

1265341 gtcagtattg agtgcacgta tttttaatat gcagtgggga aggctacggc aagcttaatt
1265401 acatattgtc ttttccacag gacaaaatca tccaagcaga aggcagcaac tcatctttac
1265461 ttggtgaagt ggacattctg aggaaaaacc tcttgaaaat ccagggcaaa gatgaggagg
1265521 tacgcaaagc tgaagacctc tgtcgtactg tcagagaaaa actggaaaga gaggaaagtg
1265581 tcacgaagga actgagggca gaaattgaac gcctccagcg gcggatgact gaactggaaa
1265641 aactggaaga agcttttggg aaaagcaagt ccgactgcag ccagctctgt ctaagcctca
1265701 atgaggagaa gaacttgacc aagaagctct cgtcggagtt ggatgccctc aaagcgcgct
1265761 tgaaggagat ggaggggtcc gagaccaaac tggaaaaggc agagcaagct ttatctgtag
1265821 agctggaaaa acttaaaaca ttcactcaga ccttcgtgaa cgagcgcaaa cggctgttgg
1265881 agaaacagag agaggatgag aagatcatct tgagactgac agacaaattg gagctccaga
1265941 aaaaacgcct gggctcatca gcagaccctg gccacactga tgtcatcagg tcacgaattg
1266001 aggacgagtt gtcttcgaag ggattcctta caagtaaact ggcgggacgc aagaagagcc
1266061 tcgactacat gaagctgact gacgacaacg tggtcctggt caacaaatcg gagaacgaga
1266121 agaacagcgg ccgggaaggc tcacgggagg aggacaacaa agtgaaggag ctgacacagg
1266181 agatagagag gctcaagaac cgccttaaac agctggagat agtggaggga gacctgaagt
1266241 gctccgagtc cagaaacgga gaactccatg agaaattcca gatggagcaa attcgaactc
1266301 gccagctaag tgagcaggtg gaacaactcc gaacgcagct gtgtggaaaa ggaatcggaa
1266361 gaaacggaac tagtttggat aagcacggaa gcactattat ttgccccaac agccccgcaa
1266421 aggtcctgga aaatggcaaa gctgagggtg aagaaatgat tgtgaaagat ttcaggaaag
1266481 aaaagcccag atacaggagt tctgcaacag tctcggaacc gagttccccc aaacacaaga
1266541 acagagatca gtctcctaca tacaggcggg aaaccaagtt aaggagcaga gagctgagcc
1266601 actcgggcga gagctccccc aaacctgcta gaagagccct gagtcctgct cacaagagta
1266661 ggaggacacc gaaaagtact gcaattacat ctgattatgg cgcccgacaa gggacggatg
1266721 agaagacacg agcggcggtg ctctcttctg caaacacaag ctctggtgat attaaaaaag
1266781 catctgttct cagtcgatac cctccagccg tcactgacca gaagcctctg aggaccactc
1266841 ataagcgatt tgatgtgata aacagaggga gcgacagcga gtccaacaac tctgatggga
1266901 tggcggacac gtccaccgtc agtggtgttt ctgcctcaga gaaggacacg ccttttgcct

1266961 cagaccagga atctgcagat aaggtccaag aagcgatctc tgtatctgtt ccatccactc
1267021 tttccaaagc aaatggctcg tacaccgcgt accgttccca cgtctcccca ctgctgacca
1267081 gtgaccaggg atcagagggt cactcctctg cctccgaaac tgagtccact ggctcaaggc
1267141 cgtccgagcc ggagaccgca acagagacgg gcccaacttt aagcagcagg agtgcaccaa
1267201 gcagatactc cagatacgct cacatccatg attcacattc agaggactct tctaccagaa
1267261 gctcatttga cgaggagctt cacagccgaa caccagtcga tgatggaggt caccagggct
1267321 gcacctcatc agctattgag atccagcgat tgtacagccc acgtgaggcg ctgaggtcaa
1267381 aggccgtcat caagcctgcc attgttgaaa ccgacaggaa ggagttgatg atttcagaac
1267441 ctttgtctac aaatgggaaa cccaaaatct ccaccaaacc tgttgttacc accaccagta
1267501 aaatgaccag cagtataacc ttctacccca atgacccaag ctcttccaga accagtagtc
1267561 gcagcagcag cgtgtccagt gagcccttac ctgtcaaaga acgacataca tctaccagta
1267621 acatcctcat tggtaagttc atagggttgg gttgcaataa aaccatttat tttcatgtat
1267681 ttttaaattt cacaagtgtt gtcaattcca ggccccaaca ttgaacacca tggtagcgtc
1267741 tctgtcccat atgagatatc aattcccaag agtgacataa cctcatggcc ctgccaagac
1267801 caggactgtg gtggtgagga ctacaacaaa cctgcatcca gaaccaagcc ccacagcagt
1267861 tccagagtgg agaccaccac caccagccac ctgtgcagcc agcgcagcag catcagcctc
1267921 cagtccccgt acaccggctc tgcggacctc aacgacacag agtcgggctt cgaaagcagc
1267981 agcagcacca ccgtcaccag ctggaggcac caaagacaaa gctcacatcc ccatgaagac
1268041 ggttacggtt taacggacca gaagaacatg actgtacgga gcacctggaa aacccgaggc
1268101 gttgtgtctg tggaggattc agttcaggga cgagggaata caagagcagt gaccgatggg
1268161 ggcttggagg atgatgcaga aaactcaaca acgtggaggg cgtaccgggc aaccaccgtc
1268221 gatgcagaag aaatgaacag tggaccaggt cctggcagga accctgatgc ccagaaagga
1268281 gccaagccat ctcctgcaga ggtgagatga atgaatcgga atattgcttt gacaaaaata
1268341 gacgttacag ctgattgtta aagccgcttc cgcctacctg tcccaggtgt acatgcgcaa
1268401 gatcaacagc gtaacaaatg caagagaatt ggaagaacca gggttccgac ggggaaagcg
1268461 ctcccagtct ccctccgtag aggcgggggg cttaggaaga atcatacctc actcgcctgt
1268521 aacctctcag tcctggggac gatcacacac acaaccacag gtacgacagc aaacctgccc

1268581 gttcttacac aacgtttaga aaaagatggc cccaccagtg ttattttacc atgaaaaatg
1268641 tgcatattga agtctcggtc ttttaatatt acaaagctct ggaaaaacct ctgtgctgga
1268701 atgtttagcc agtcgatcaa ctacataaat acacacatga acatggcctt ggagctataa
1268761 ttgccaacag cgcgctccct ccaatttagc ctccatgggg taaattaaaa ttctccagcg
1268821 ttgcttctta acgccccttt tcaccaccag gataaaccag gagaagcatc actgggggac
1268881 aaacacctct tttgctcagc ttaggagtca cctgctctcc tttttctgac ctttcatcag
1268941 ctgatttgta agccgtgtcc agcccgatca gttagatcca gttaattctg ctcctcccat
1269001 gttctctttg aacatcagat tttccatctc ctgcctgcag gactggtaat gagagtagag
1269061 tgggtgaaga aaaaaaacaa aaaaaaatac cctgctgacc accaagtcac aatcaatcaa
1269121 caatcaagga gagagcacgg ttttcccttt aagagattat tgattggttt acgtttaaaa
1269181 ttggcccgta aaaattattg aagccttaat tgtctaaagg cctttgggga cacatggtgt
1269241 cacctttatc ctgaggagga ggggccgggt agtcaaaagc ttgtggtagc aaggcagcat
1269301 gtggggtcag acctgttcca ctgttcccaa tttgcctgat gtgaaccaca cacaataatg
1269361 tgtggaataa cagtctgcac agcagctgga atttcactgg gaatgttttc aatcccagga
1269421 catcagacac cctgcactga ctcatgaaag caatgaaaga atagttttga gcatcaatga
1269481 gcatctgggt aagtggaaga caaatttcac ctcaagtgtt cagagtattg tctcgcttga
1269541 gagattattg ggagcgggaa gacaacacca atccctaaat ttgactgaca gaccaagggc
1269601 agacagattc aaaacagggg tataattagt tctgcagaag ctctgacacg gtacttatgt
1269661 taccacttaa atctgtaggt tttaaaatct ctcctggggg gggggattgt gcacacagcc
1269721 tctgcttttt tttgtggaat ctgtcgtaaa tctgtactga cacagttgta gaaacaggct
1269781 gtacgcgtag ggtaaaagag tctaaaaaaa gtggttagca ttttccaagc taaagtaatc
1269841 tgatgtcatc tgctgcagca cgtatgtagc ctggtttcca ttcaaatgca gcatttaaat
1269901 atacagtata caagatcttt aagaaatata tttgccaata tatgaggctt gttaaaacac
1269961 gccatggttg gcacataatc tcaaatcctg caaccacaga agaaggatag gaaggtgcgg
1270021 ctgaagccgt tgataaatat ttgcatttca ccgctgcttc ccatcaatta tattaatatt
1270081 tgtttctttc ccatcgtcca ggaatgtcgt ggtttcctca tcagcccacg catgcatgtg
1270141 ctgccttcac cattttattt ttttattctg acttcattca tttttgtgtg aaccttgtgc

1270201 ataaataaat gaaaacagtg gaatttcctt tccatatatt ccgactcacg gcatttaaca
1270261 ctttctcaca tttatcttgc ccaaacacag cctgtgcagc cattactgtg caaacacgca
1270321 gtatattctt accattttag gtctgtaaca actctcagac ctgcgtggag cagcaggaat
1270381 gaggtgaagg cattacaaca catggcaacc ttctgaaagc ccccagccct gcagcagtag
1270441 cttaccatag aaggacaata tgccacactg ctgtttcctc cccacggtga ctgaagcagg
1270501 aagtgaccgc acttaaacac gcccacttgg cagggttcac tggtgactga tttcctgtct
1270561 gttacccctg gagattaagg gaggcagtcc agcagggctc cattaaagcc aattattgag
1270621 cacgcattct aaaactgcat cggaagccaa ctccaaccca ccaaatgtca ctaattatgg
1270681 tgttttttaa ggctaaaatt acaggcgcgg ggtgcttttt gtgtatgcct taggaactgc
1270741 atcgtcatcg aacttttcgg agcggggtta ctgagggtat gagagctgtt gtagccgcca
1270801 caggctagta atgggtttgt tttctagtca acccaccaaa ttataaatct ctcagcttgg
1270861 ggtatgtttg gggtttttgg ttggggattt ttcccttgct agaggtccag cccatgtcac
1270921 acaccagtta acacaggcag caagtattta attcccacat catagaaaag attagggttt
1270981 ggaaccccag acaatgggcc tgagtacata tcagtgtttc tccagaaaca ggaactcttc
1271041 ctaatcttca tgtccaacta attccaggct gctgaaacga cagagcccag ctcaaactcc
1271101 ccccacagcc cggcgacctg gaggagacag ctacccacca gcgattctca catcctgggg
1271161 agctccaaga ccactggagc cacctccaga ggggagctgt gggccagtcg gggtcatggg
1271221 tcagggacca gagtggaagg gagaagcggt gcgagtggca ggctgtggag ccatcgtcag
1271281 gcagaacatt agaccaacgg aggacaggta tgaaagaaga gttcatctaa ttgatcaagg
1271341 ctgcagaagg ataaaggcct gattgatgct ccgagcacat tattggatca tatatgcaac
1271401 aaagggaatt aaatattggg attttaattg gagatgtacc aactgacccc gaattctccc
1271461 cgacattaac accaaattac cgtttggccg tctctctaaa gcaactacct ggacgttatg
1271521 gcccaatttg tgatggaaaa cgtaaaccag cgcgtgtttt cacaagtatg tttcagagca
1271581 agctgcctac acatagaatt tgagaacttt ctctctttct tttcataaca gcactcaatt
1271641 aatctctcaa tggcagaagg aggtccgagg atcagcctcc tatcatttag tggctgcagc
1271701 tttcctgcca tcttcaaaag ttaacaacaa aagcaaaaca gcagttgcag ccatgcagaa
1271761 caaaaccaca gacatccgga caccgagacg cgatcagctc ttccaatccg gaactttccc

1271821 tgtcccgaaa gggccacaga cagagagaac agcctgtgca gcagccctca tatcctcttc
1271881 tgcttctggt gttggatgta ttatagttaa atcccaagaa cttgacaagg gagaggctgg
1271941 catgctgggg gcggctggag attttcttgg atgccaggga gggtttacag agagctgacg
1272001 gaagtgagac tccaatcttt tttttttttt tctccccctc agaggaccat tacatcgacc
1272061 tcactggagc ctgacaagct gctccagcat cagaatttta actggatcac tgaaaacagc
1272121 ccgtctcgat attcaatcat agggattttc atttatttaa aatgtgacca aaacaggaag
1272181 tgaattaccg ccacagctga cttctaccag ttattctgct tttgctcctt tctgacacac
1272241 acatttgttc ccaaaaccac atcagagcca tcaggaaaac tagaaatctg cacaacttag
1272301 aatgacggat cagtttcagc ccgtgtcagc acgttttggc atgtagcctc acgttgggtc
1272361 gaatttgagg atgttataac aaaccttgca tgttaaataa ccgaagagca cttggagacc
1272421 gtggctcagc atgcacgggg gcttgaagga ttgcaggttc cctgcagtta aggaacacat
1272481 gcgcacgcgt gatttctggg atgttggttg cgctttttgc cccggtcatc ttccactaac
1272541 tcagatcttg attcacttcc tgttgacaga aatccttgtt ttcttgcaca tgtagtaaaa
1272601 aaaaaaaaga aaaaaatctc aaaaccggtg cttcatcgta cagctgtcgc agtttttgtt
1272661 tgtgcatttt gagagagcca tagctaaaaa gaatcctgag ctggcggggg tggacatact
1272721 gtatgtcact gctgctgtaa agactgtgca cataccggcc agccacagga ggacacacac
1272781 cttccaacta gaagtatttg acagtccagt tcgtgtgtgt gctgggttcg ccgtgcgaac
1272841 agccccagct tgacgccgtt tttggttttt tttgtttgtt tgtttctatt tctgtagagg
1272901 tgaattattt tgttgcgtcg tacgatgttg tcgtagttgc tgtaaccgag tgacttctgg
1272961 ggccatcaca gcagctgaga acgatcgtgg gaggccagta attaagtatt ctttaggtaa
1273021 atggacactg acgcgtgtct ctgccgcgct ggggtccttt caccgggctc ctgctgcatc
1273081 agagatgaca cacatgtcgt tagcttcagc gctaaattta ggaacgttac tgtcgaaccg
1273141 catctgtgaa accgacttgt tgcatcagtt gaaacacaac tccgtctctg acaccataac
1273201 tgtaactgtg acaacaactt gggacacaaa tcagtggaag agctgatgtt ctgtgtgtta
1273261 gcatccgtct cgtagctgtt ctatttatta gactccacgt atgtacaggt tcttttactg
1273321 taaaggcatt tccctgggtt gggggaggag cccagagtgc ctctgctttg gcctcttatt
1273381 gtttataaaa gcaggagaat atatgcaatg tctgtatcaa tgtcagtaat catgctgtag

1273441 atgacttcct ttcatttgac cttttgctag aactatctac agcgcagtcg gtgccttgag
1273501 atgcattaag agggttcgat tgtcccaact actttacact catgctgtct ttttccactt
1273561 gctgtgcagg aaaaaacaca tgcttgctgt cgagcccttt tttaaactgt atatatttta
1273621 caccaggaga gtttatgcat aaagagttaa aacaaatttc cctatcccag cttcagagtg
1273681 ctattttaga gaagccagcc tgcaccgccc agaaagctat attctcccca tgtgtctgtc
1273741 agcgtgcagc cgtgtggcct gtgcggccaa aacaggctca tttctgtgtc aaaggccggt
1273801 tcttctcatc ggggaacgcc gtgtctctcc ttcgttaggt tgctcttcct cccctcctca
1273861 tttcagtctg aaaggtcaga ggttagggtc acatctgaca ccgtcgggtc tcggggcttc
1273921 taaatacagg agcaggggaa gatgcagagt cccctttagg gatcgtcaga gctgaccgag
1273981 gtatcgaatg tcccaccccg gctgcgctca acgctgcccc gcatgcacgt ggagcgatca
1274041 ccgcggagac cagatccctg agggaggggg gggggggggg gcgccgaatg cttggtgctg
1274101 cacgtgctgc cggtcagagt tgtagttttg gagagtcaga gctgacgtcg gcgctggtgg
1274161 ggcgaccgta tgttagcagc agggtgacgc gagggtgctt ctcagcctaa atcctgcctt
1274221 acaccatctg acctggaagt ctcgccgtcc tcgggttcac tgcacaacat ctatcactcc
1274281 aatgaggaat tctcttcagg ctccctgatg gccgtgagca gggtctcaag aggaccccgg
1274341 ctgtctgaaa atggacagct ttatgatgtt ctgggggcct ttccttcacc tcgggccgca
1274401 tccgctccgt cctttcatag ctactgtaca gcacctgcag cctgagctgc ctctagtgcg
1274461 cacggtggcg cgcacatggc cgaatgtgat gacgggcccc gtgggactcg gagtgaagac
1274521 tttacagggt tccagaatat taaggacttg accatgagtg gcagacagga agcggttaag
1274581 cagacgtttc cttttcaaat cccagcaaga gttaacagaa atcctcttaa gcgccgctcg
1274641 ctgctgcgta aacatctgtt attaacaagt cattgttctt tctgaagcag actagaatgt
1274701 acttaatgga gattggcgcg gtgctgcaga aatggctccg gggcagcgag ccggccctca
1274761 aaggcctcca ttgtgaagcc ggggtcacgg tgtcattggg tagcattggc tctaatctgc
1274821 acttaaccag cacttaactt taagacctca acaactaatg ccccctcaat gccctttgta
1274881 aaaaaataaa taaataaaag agatccgttt ctgtcccaga gttgtacttt aaaggttccg
1274941 gtagcctccc gatgttcccg tgttgtcgcg cccgccacct tcggagcgtt ttagcgtcgt
1275001 ctcaccagct gaggatggaa gatcgtgtcc ggccgctctg atcgtgagct acgtctccgg

1275061 tctcagcctt ctcatgccgt gccaaaatca ttccgcagta ttcccagttt
acagacgggc
1275121 cggcggcagc cccgtctccg acgtactgaa tttgttttaa aggatccgtt
tgatgagatt
1275181 tcttctgcga aatgtgtgtt tttatttctt tgtcaaacaa aaagatgtct
tattttggaa
1275241 gctatatcgc atagatttta tttaaggaaa atcttgtaat tttgtaaagt
gggccttatt
1275301 atggaaatat attaagtatt tgaccattgg aattttaaga gctgtttttt
tttttttcaa
1275361 gataatgtat cattgtttat attacactcc tggagagttt gtcttcctat
tttctttcct
1275421 cacatcacct ccatgaatca aaggccatat aagattgggg gcgggggggg
gggggggtcc
1275481 acagttacgt aagtaatgat ggagcgctgc ttccttttat cagagctggg
agggtccgct
1275541 ccagcctgga tcggctcgcc acacgtggtc ccggcttttt atcgtggacc
agccagcttc
1275601 ttcgctgctc tcgtctccct cgctcataaa ttcagggttt cgcacatcat
ctgggctgaa
1275661 ttgttggaaa gagctgccaa agaaagaccc cagacacact cgtaccgtac
acaacacaca
1275721 tgcaatctaa acacggaacg cttgacgtca ggcaacagtg ttaaaaccaa
tcagctctga
1275781 ttgacagaca aatatttgga ttataaaatg acacaaaaag gtcattttct
ccatgttaaa
1275841 gtctcttcat ccccagtggt ctgattggag ggagagaggc tgcgctgttg
ttttcctttt
1275901 tggtttattt ctccagtttt aggcataatc catcttgcat gttttaacat
aatgtggccg
1275961 gcgcactctg catgtaggtc tctcgaagct gccaactgta cagcataccg
tatgtattcc
1276021 aataaaaata aaaaccaacc tgtttgtaat acgtctccat gttggaacct
gcgtccgggt
1276081 gcgtctgcac gctgctgaat gcgtgtaaaa tgcaaccacc tatggcaatg
ctgctctttt
1276141 aatggttttt gctgatcaag ctcagcaggt tttatattca tagtcgtgtg
tgtgtgtgtg
1276201 tgtgtgtgtg tgttgggggg ggcagctggc attgcaaaat aagataaatg
aggcaaattg
1276261 ttcaggtaca gaggaaaacc acaagctgct gtggaatgca acgtcagcac
acacactcct
1276321 ctaaaagcgc tacgcctcgc tgtgggaagc ggccgcctcc ttcgcaggtc
ggatttgaat
1276381 cccgtcaacg taacaaaagt gaaattgaac ttttttttcc tgacctgacc
ttaatcgagg
1276441 gtcagaggtg cctccggtct aagctgcccc gcgttcacat gcaggagagg
gcggtggttt
1276501 ggtccctggt actcctgagc ctgacctgaa ttagtgctgt gaagccaacc
gtggccacgg
1276561 cccagtagcc ctccaggcca ccactggtgt cttccacccc ggagcagatc
tcagccctgc
1276621 cacagtcgtg gtaataacga ctgaattaat gctgacacca acagcatctg
gaaggttttt

1276681 gtgcccatgt acgaggggct gcggtctccc atcggcaacc ctttcgccat caaacaatgt
1276741 tgctattgtg aaaaacaacc tccccagtaa ctcggggctc cgagcacatg gctgaaaata
1276801 ccagaacgct gccgtccaaa acccagctca aattattgtt gttacatcaa gcgtctcctt
1276861 tggtcacgat aataataacc acatctctgg gctttttggg gggggcgggg gggtgtaaca
1276921 ctcaagagtg cctggaaaca acagtcctga aatacaaccc agaccgttga aatcagcccc
1276981 ccccccaggc tgctgccgcg gacctccggc accataggca tggacgcagc cacacgcttc
1277041 aaagacctgt cggtagaaga tcaaagcaaa gttgctgata atgtaaacgc agctactaat
1277101 gaaagagcca ggacgtaatt atgtgtctct ttttaataac aaatgacaat atatatatat
1277161 atatttataa aaaaaaggat ggggagaaaa accatattaa ccatcgtaaa acactggaac
1277221 tgctatggaa acaaaaattc acaattcaat cgccacccgt ccgtcaggct tctaacacat
1277281 gcactgcctc tagtctctgc cagctaaatg acagcaaatt atgaacgaca aaataagacg
1277341 gagaaagatg tgccaacgta aaacttcaaa aatggagtta atttttcagc agatgttgga
1277401 gttaaatagg ccgagtacaa aggtaatgcc atacgtcttc agtttaagac cttaaataac
1277461 atatttatag tcttaaggag ttattttatc agcattataa ataaaagcag aattacagtc
1277521 ctcccccttc cactagtctt tgtaccaatc tgtccaaaac aaagtctgca caactgaaat
1277581 gtttcagata aggaatctga aaatgtggcc agccagataa taaatccttt aaatgtcacg
1277641 tgactgaagg caaagttgtg gtttttcaaa agtagaaaga ttcatttggg tttttttttaa
1277701 ggggggaaat atttcagaca gtggcggcag gctgggcctg ctgagggttg ctggtggctg
1277761 tgggcgtggc ctggcgagga agtgtgtaca tggcgcccag gaactgatcc gcgatgcggc
1277821 cggcctcacg cagcccgctg agcagggcgc cgtggactgt ggcggggtaa tttctgatgg
1277881 tgtgctcccc ggcgaagaag aggcgtggga cgggctgaag gagagaaaga agaaagatga
1277941 agcagaacct gccagactag ttcacatcaa acccgcatca gttcaactgg atcccagtca
1278001 cactcacctg cgaggctccc ggtatcgccg gtccaggggt gataggctgc gccatgaggt
1278061 cgtaatcgtt cccagaagaa cctgccgcta catatgagta cgagcctcgt gcccaggggt
1278121 ctgcacgcca acgagtgacc acagtctcct ttggctgcca tgacaacgaa caacaacaac
1278181 gcatactttc agtggagttg ctgggaggat ttttaaacaa ccgaacaaac gttaaattgg
1278241 gtttcctgca gacgtaatcc acccggttaa aacatttggt gtgaaaatca cggtttgctg

1278301 ctggtcacag ttcaggaatg ctgcgtacct gcggcacagc gctgcctcca aatattcctt
1278361 tgaggatagc caggcagcgc ccaacgatca cgtcgtcgct gatgttctcc ataatgccag
1278421 cggcctcacc ggccatcagg gcgagcagga tgggcgctgc ggggtggttg aacacatcgc
1278481 agttagcagc tgtcacatga ctaaactggg actctcttga accgaaacgg ggtgtcttca
1278541 tacctttgta gaggttccag aacaggaaga gttcgccccg gctggccgtg gtggagccga
1278601 cgtgaccgaa cagattgacg ctcgggtccc agaacacgcg gtcgaagcac agcaccacct
1278661 tcagacaggg agggatgcga catttacagc tgtgcaacag tgaagctgct gattgtgagc
1278721 aacaggctca gcgcctctcc tgtggcgttt ggctctgcga gttcaaacac cgccccctgc
1278781 tggagatgca gcgttagcag cagtatcggt tgcgtaacaa tgcagtatcc cttctgcagt
1278841 aaaatgaggc cgatacccag cgacacacgg gcagcaggag ctggtgctga cgtacacaca
1278901 gcgctaaaca caagcagcag aggcaagaca tcagcgcgtc accttgttga ggttgccgaa
1278961 gcccatcctc tgtatcgccg acgtcttcca ttcaggcagc gggggacaa actgcacggc
1279021 ggacggctgc tgcttcaaca cccccagcgg cagcgtgcac agcacagcgt cgcacttgta
1279081 tatgaaagtc tgggtcgtgg agcgggtgtt gaccgctatc acttcgcagc ctgcagaaca
1279141 cgcttgtggt cacgaaggcg acatgaccga tatgtttgaa cgggatcggg cggtccgtgc
1279201 gcacgtacct gaggccgtgt agcggacctg tcgcactgcc gtgttcagtt tgatgtccaa
1279261 gccctcggcc agcgccacgg gaacgcagga gtagccgttc ctcacagtca ggtggctccc
1279321 cgtgaactca aagtcatcgt cctgtgcaga aaaccaacgg tcaacaagtc tgctatctca
1279381 gcgtcgcgat gaaaagcttg cagttgtgcg tgtacgtgcc tgatcccagt gcttgagaga
1279441 aagggtggag aggggtgtag cgttggcaaa ctccaagttg gcaaagtgcc agtccaagat
1279501 ctgccgatcc ctggatgaca ggtaaacatc gctgtgggaa aaaacgggaa ataaagagac
1279561 accttgatat acggtgacag ctattcagta gcaggggaac atgtctgctc acgcacgggc
1279621 taataaagcc tgatgctgaa agactaaaca cacaggatac cagcatgtta gctgcaggac
1279681 accagctcat aacagaactg gtttactaga tatttgaata agttgggtgc gttccataca
1279741 gtttggactg atgttgacag cagacagccc gcaggaggat atgaaagcag aagttacctc
1279801 ggtggattgg cctccagctc ctgcagcttc tcctctagct tggcctgcaa ctccaccaat
1279861 tcatcatact cctgcacaga aacaagcctg gagtaagatg catgaccact tcacagggtt

1279921 ctactgctgg aggacatctt ttcaaagatt agtaatgagg aaatgaattt gaacttgacg
1279981 atccaagtaa aacaatatat tcaaacactg aaagctcact caaatacgta gaggggtgtg
1280041 cagccatcta tgcggcccaa gtttgagtaa aagtataaac agtgtggaca gaaagggctg
1280101 accgacgctg tgcatcagct caccacaaca gaaccaacac cagcggctgg gcctagtctg
1280161 gtaacaccac ctacaacacc acgacccaga cggcgacaga gtcagacgtg ctaacaaccg
1280221 ctcaacgata gcaggatgtc ctgagtacct gtaaataata aagactgaaa gcgccaacac
1280281 gttgccttta cagccttaaa aacacatcta tgtggtgcag tcaggcttaa tgtgagcgca
1280341 caaacatgct cttaatatga tggtcagaca gtgattcaag gcctaaacgc ggacagacac
1280401 cagctgtgtt taaaccaact caaacctctt tttaaaatac ggcagcgggg cagatttatc
1280461 tggaccaaaa ataaacgggc atccctcgct ctgtgagtaa gccataatct gacatttaac
1280521 cacttaaaca cgggctgctt tgatttacag caacagcggg atcgtctgac ccgtccagaa
1280581 aatatcattt tacaaagcag acaagctcat ttgcttctgc tttggccttt tgacctgcag
1280641 ctccacttca aatcaaagcg ggggaaatct aatattgctt caaagtcgtg tgaattacaa
1280701 acatgatcgc tacaccagca ccggcaaacc acgactggaa aaggtttaga agtgttactt
1280761 cctctggtat tttaagactt ttagccagat cagagttctc ataactttgt tccagacagg
1280821 aagttgtact ggatggaaga gaaaagagga agagagctca ccttgcagag ggcagtcaag
1280881 tcccggtgct tgctcttcac caagaactca gctgtgatat ctctgggtgg tttgacctcg
1280941 ctggcttctt tatactgctg gtgcagctct ttgactcgct ctttggtgtt caccatctac
1281001 aaggaatggg agagaaggaa gcgtacagag atacttgtgt actccatagt ccagaaaaac
1281061 cgccatcctt cttgtgccca ccttgttgag gagatctctg agctcttcct gagtcttaac
1281121 aatcttcttc caatgttcta tctgctcgtc tttgacgtgc ttctcctgca gcctgagcac
1281181 atgcagatac cagattggga cctggattgg gacactggtc attttataaa tttattctga
1281241 acaacactca ctgaatgacc acttccaggg cctgtcctag agaaactggc ttgttgttca
1281301 ggaagttaaa gtccagctga tggctgagga acgaggtggc ctccagcaat cggttgaatt
1281361 cctgctcaac catctcatct ttttcttttg gcacctagaa ccacacaaaa cagggatagt
1281421 gtacacacca atgcaagaaa agagagcaac aataagaaaa aggtacgggc taatctcacc
1281481 acgagaaact gcaaaattat ttattacatt tacgcaggga caaattaaaa cgaggatcat

1281541 agggagtaaa gttaaagttc cttacgcttg tgcatcgttc acccttcaag tgtgccaaaa
1281601 gatgggtgtg gaagaggttg acaagaggag agagagagag aacattagca cacaaaatca
1281661 tttacaaaat tgttaatctt tgtgcagaaa agcaaatggt ttatcatgtt gggtgaatct
1281721 ggctaaacat ccttcgactc aaccaaaagg ggaaaaccct gtttatttaa tttgatgaga
1281781 ttaggcgtaa tctggtccta tttggggagg aacaatgaga cgctgtttcc caaagagacg
1281841 ctaaactaat ttgccgctcg ttatgtgtcc gatttgtagc tcaagtaaaa ccagatctct
1281901 gacccagcac atcactcggg catgtaccaa catgaaattg tgcatctgta aacacttttt
1281961 acaggccgag aaagtcaaag atttgagcta atgcaatagt ttaaggcagc ttccggaata
1282021 aacctgctaa atgtggttag ctatagcaat gaaattggat catactcaca gcctgcccat
1282081 ttgcttcata cagaggacac ttctgcttga tcttggccag ctccatgttg acctgcttac
1282141 taatcacagc catcgggttt ccgcctaaca aaggcacagt aacactccat caaagcacct
1282201 cttacaccca gatgtctgaa ttaagagcca gattttataa agcatgcgag ttcatgtata
1282261 tcgggggtaa gctttattta ttattttatt ttatttaata atcagttgac ttacccagcc
1282321 ctgtcaccac catggccccg agatcagcaa catagtttcc ctttctgaat gtagccactc
1282381 tgcccccaac acgatcctac atgtaacaag tgaggcgtgt tttatctaac cgagaacatg
1282441 ctgtggtcac tcaactaaat cccaataaac cctgttataa ctctacttta gtatttaccc
1282501 tgttccgagg gtagtgctaa tgttaaaatc tgctgagttc cacctaccct cgactctaat
1282561 actgtaacat ccatcccaaa gctctgcagc tgcctcgctg cagccagacc agagacacca
1282621 ccgccgatta ttataacctt cccagtcttc ttagctgcaa aaccaaaagc agtcaattaa
1282681 agagatgtac ttccatttaa tgactatttt cccccctagt agaacgtatg aaaagaactc
1282741 actgggtaaa ggcttgaccc tcttgtaaat gccaaaatta atgagcccgt gcctttccag
1282801 gtagctgtgt atcctgtgga ccagcacagc atcgcctgat acagagcaaa ttcaagaaaa
1282861 tccactttag ctctaaaaat aacaccatat cttcttcata tgaaaacaga ttctcactgt
1282921 tgtacggcgc ttccagctgc tgcaccgtag cctcaaaggt cagctggatt ttggggttgt
1282981 ccaaccacag ctggagctga atgagccata ggcgtgtaag tgctcactga atatttcaat
1283041 cccagcaggt tttagatttc aaaagtgttt gcatgacgct tacagtgcgg tttcggatgt
1283101 ataagaagac cttctgtgtc tgctgaggac cactgatgat gtcagggaag caggcagcct

1283161 cttgtgatgt catacggtcg tgaggaagac ggctctgaaa agcagctcct tctacgccta
1283221 accagaaaca tacacgtcaa aacagcgtcg acagtgcagg aaaagcgaac aaaatgttta
1283281 gtaatttccc caacaagtgt tctttctagt gaatatgctc ctaaatcttg caccaggcct
1283341 caggctcacc cgacggctcc tctggttcac tgtcattttc ttcttctacc ggtggagccg
1283401 gtggagggat cacctgcttc ctctctttct ctgccttggc gtttctctct tcctcagagt
1283461 aatactcatc ctcagagaga ttggccaggc tctcgtccat ctcgcggtac tccacctgca
1283521 acaagtatgc acaataaaag ctaaattaac atcagctttg agatgcggat tcgctccggt
1283581 gatcttcagc aacctttgag cctttgcaga tttgtaagag cgcaaacaag attcaagcaa
1283641 atgtcctcat aatgttggga attttcactc tccagcggcg ggaaagtcaa ttttaaattg
1283701 attacttttg ctcgcttgcg ccgacttgtt ctgcgaactt ctgctgaatc tgggtccacg
1283761 cccccagggc ccacttggtg cggcaaaggg ggccctccgg cctccccgct gggagaggac
1283821 cgctcttttt tcttgacgtc catggggacg gcagacgagg ccgaaggacc ggacatcgca
1283881 tcagtaacta gcactctctc agccccagtg gctccggcag aggaggagga ggaggaggag
1283941 gaagaggaag aggagccagc atcagacttc ttgctagaga gcatcatgga ggatgttgga
1284001 ggcctctcct ggtcaaaaga ggtacagagt aaataaaaga cagcgaactg aaagaatcta
1284061 tagtgaaatg accaaaatgc aaatactcaa ccgaaaactc ctaaaacaga aaacctcaga
1284121 tgcttgtaat aatttggaca ttcacatatt taaaaaatca ttttacctct aaatagcaaa
1284181 agatttgtat tgtttttttcc tgttaaacct ggaaatgaaa ctggtgctcc ttaagggaaa
1284241 aacaaaggta ttctatataa aatggctaca acatattaac tttttattcc atatccttga
1284301 gaaaccctta tattaaacct ctatcattac atattggata ggctttatca tttcaagcct
1284361 atattgttgg catgaagtca aaatcagact gtccaaatag ttacagagcc accgttatat
1284421 ggagaagatg atgcagctta tgtgtcaaaa aaacaacgaa aaacatgagg taacaaccac
1284481 agaaaaatag taacacgttt aagttgttgg cgtttttctt tttactccca ttactttgca
1284541 taaatgacct gaactgatca cttctattgt ggcaaaccaa tgaaaggaaa ccaaaacccc
1284601 cattagtttt agatatattc agactatcat ctatacatct agatggtttc agtaacacaa
1284661 ctgtgacatg gatttcatgg cttatgaaat gagggctcca tggttaggaa aactggtctt
1284721 gttttaccca tttctccgtg catctggtaa tatccattca aacactaaga aacgaagaca

1284781 gaaccagaaa tagtgattac caacaggatc acctgagtaa aggcgttggc cttacaaaaa
1284841 catccgtgga gacttagtga tagatattaa cgagaataaa catgaatgcc catgtgaata
1284901 ttaggactaa ttaaccaagc aatttaaaca agcgcaagtg ttttattcaa atacgagaac
1284961 ttgcttcgaa atacattcaa cgtactggtt tgtttggcgc attattaatc gttaatagcc
1285021 agtttattga ccgaatcaat agcggatcgc tagcaacttt actagaaagc taataacgac
1285081 acaaccatat ggctatcact gtttaaatac ggtggctgct ggtagctagg tgccggagag
1285141 aaacctacaa agagcaaata ttaattatga tttttatttt ttacaagtct agactctata
1285201 aaaaatctca tacataacaa gcacggatta aactctgcag cgagccaatt tgatgaccat
1285261 gctacattag ctaccgccgc taacaatgaa ccttagctag caccatcact gagacagcag
1285321 caactgctat cgagtataat ccacacggaa ataatcgtct gtcaactttt gagtgccaga
1285381 aatcgttgtt tcaagcaata aaacgaggcc acggaattat agaaatattg aaattacctc
1285441 ttacacctcc gatgtcaaca caaagacggc agactagcag aacccacaca atgtagccgt
1285501 ttgtgattgg ttattggaag aggttaccca agagcactga gtgatctgat tggcccgtag
1285561 agctgtcaat taaaaagagg cggaaactgt cggcggcgac gatgtttcct tgtccaattg
1285621 tgtttaacaa ttaacgtcgc acaatgcatt taaaacatag atcgcggtga atcgttttat
1285681 tcattaagtg gcatttaatc acttgttgtt aaatatacag tacaactgat cccaaccccg
1285741 ccatcatcat gacgtcactg tgagaggatg ggtggggaca gacaggtaaa ggtgtcgccg
1285801 agcgtctccg aaaactcagg gaaataaaac gacaacttca cttgcttgtt tcttcatttt
1285861 agaccgtgcg taagtaacgt cgaaattcag atctaaatca tgaacacagt ttttaggttt
1285921 gcaccgagcc tctcgttgtt tcagctaaga ctttaaattt gacgtaaacg ctctcccaaa
1285981 attagttttc tgcgtagctt ttctgtaaat accaggcggc gagctctaaa aagttggtct
1286041 gagagggcta ctcttttaaa aaaccaagtt ctatcgcgct ctcccagctg ctcaaaatga
1286101 gatttcactt ttgaatagta atctggtgag aactcattga aagagcgtcg gttttagtgc
1286161 tatagtgggt tttaaagtaa aatccacttg aattccaggg tataaactgt gtaacgacag
1286221 ggctgatacg gaatctatcc agcaggtttg aaacaatggt gaatgaagag ttggccaacc
1286281 ggccagacaa cactgccttc actcagcaga ggcttccggc atggcagccc atgctgtctg
1286341 cgggtatcgt catcccgggc tttttactca tcggcctggc cttcattggc atcggtgtcg

1286401 ccctgttcat cacctcacag agcatccaag tgctggaggt atgagaagtg aagccactgt
1286461 ctttgcttca tgctaaatcc tcccccgact tgtcaccctg aacctgcaac cctttattga
1286521 cagaggtgtg gcgtcagtaa caaaggcaga ggtgttgttt ccatcagtaa tggaggctct
1286581 tgtcctttct tcaccagttg gattacacgg gggaacagcc gccctcccct tgctacaagt
1286641 gctctgatcc aactgtcaag aactgtgttt gcaatctgga attcagcatt actaccctgt
1286701 tcaaggttgg atccaaatgc aggtgggaca cttcatgatg ttgggatctg ctgacgctac
1286761 gttttcttca acaggggccc gtgttctttt attatggttt gtccaactac ttccagaact
1286821 acaggaaata tggcgtgtcc agagatgacg agcagctgta tggagacttg gataacttca
1286881 aagtgagcac atgtatccat ctattatata ttactttcct gtctttcttt ccattctcct
1286941 gacaacggac gcatgcacca ctgcttttat tcattcatgt tgtggtacat tccagtttag
1287001 acatgtgtat ttcttttaaa gtgccattta agtccacatt gttggaacta tggacagtgt
1287061 ctaaccctta atttgacaca catgccattt tgtcttgtgt gtcccagagc cccagtgagt
1287121 actgcgctcc ctaccagtat gacagcaatg ataagcccat cgtaccatgt ggctcgatag
1287181 caaacagcat gttcaatggt aggtctgaaa atcttatggt ccttcatttg ctagtggatt
1287241 gggatacaaa tttattttct cattgtttaa agacacattc aagctgtatc aacgcgtcaa
1287301 tggaacaaag aagctggtgc cttttgatgg gaaagggatc gcctggtgga cggactacaa
1287361 catcaaatac agaaaccctt ccatctcccc tctgaagaat gcgttcaatg gtaccagctt
1287421 gaggaaatgt tgatgctaat tgttctgtca gaaatcgtaa acgtaaaggt ttgaattcca
1287481 ggtactgtga agcctttgat gtggcccaaa cccgcttatg aactcgaccc caacgaccct
1287541 gctaacaacg gtttcatcaa tcaagatttc ctggtttgga tgcgaacggc agcccttcca
1287601 gatttccgga agttgtaccg acgaatcaca gagggtgatt atgcggaagg tctcccggct
1287661 ggaacgtacg tccttgaaat tgcctacagt atccttacat gttctccagg ttctgggata
1287721 atgatgataa ttgctgaggc ataggtgggg gaaggatgtc atgtgatcca ttttgagtta
1287781 aggtctgtat gatgtcaacc tgtgtgccct ccggtaatat aatttagaac cttttgactt
1287841 ctcgttgatc tttttttaca tttgcatcca cgtttcttta gctgctagaa ataaatttgg
1287901 cttcctcatg ttttgacaac acgagtcttg ttctgcaagt caggtgtggt tggttttacc
1287961 tcagcagtgt gattgagcaa aacaaatgat ctgacggtct gtaagaaaat aaggtaggca

1288021 aataattgat taggttttcg atggccctca acaaagagcc tttgatggaa aatttaaata
1288081 ttttcttggg ctggaagtgg caaaattgat ttataaatac tgagaatatg ctgatttttc
1288141 tagctaccgg taactaggct gactaagatg attgctgtat ttctgtgtat aaattacacc
1288201 atataatgat gctataacac ctggcagtag ctgtagaaca tttgtatttt ctctttttgt
1288261 tgtataccct taacatgtta tcctcagatt atcctgtttt gagctttgaa ggaagaaaga
1288321 aggtggttct cagcaacgtg tcctggatgg ggggcaagaa cgagttcctg ggcattgctt
1288381 acctcgtgat tggttcgttg tgtatcgtta tgtccatagt catgctcatt gtctacgcca
1288441 aatttaagtt tcctgaagaa gaatgagttt gtagttgtga ttttacattt gttttagtct
1288501 tttaaagcgc attgctcaca tttcccagtg acattacttt ggaatgtgtg ggttgttgcc
1288561 tttataatgg gatgtgtcta gccattactg gtcatttcgc ctcaggtacg cctaaagggt
1288621 ttaagatttt agggaggaaa accattcact ttacaaggca aaatggactt tgcgtgtatt
1288681 gtatactgta tcagtttgat ttgtgttact ttaatatggt ggtttgtttt tcctcaggtt
1288741 ttatatgccg tatttacaag gctttaaaaa taactcttaa gagcttctca ctttaggtca
1288801 gaaataatat agaaacaaca tttgctttca atttcaaaag tgtattgttc tctaaataga
1288861 aatgccacta ttgaaactca gggatcaatt gtacataata tgcagatgta taccttgaat
1288921 ttactgattt ttgtttattt atcaataaaa taaaagcttc agattctctt tgtgtcattg
1288981 tgaaatctgt caacacagta atcagaatta tgcttcaata tgtatagtat tttaatatgc
1289041 aattatgtat agatgaagtt taatatgtat aagacaatta ttacaaaatt accaaacaca
1289101 attgctcatg ctccgattac atgattaaga taaacatata tagactatat gtaaacttaa
1289161 ctccctgtaa aaacaaattt agaaacctgg atgtcggatt tattgctgca tcatcggggc
1289221 aactcccaat ttccgtaaca cggctagaca aatagacgta tcatgccgtg gcatgtgtgg
1289281 atattgtagt cttttcaatg ctgttaacag cgcaacacac gccttttgaa catcaagtac
1289341 cataatccat tgcgcttata aacttgacga caccggtgcc gcagtttgac agcagcagcc
1289401 tgaccagggt tgttgttact gcagcttctc tgaggtcttc tgtcttgttt cgttgtccta
1289461 ttttacatcc agaccagtct tggatagcca ataaaaagta tgtctgacgg cggggggacg
1289521 gaggaaaact ccggcaccat ggaggtgtcg accgtccaga ccgtcgctga cacttcggtg
1289581 ctgcagaagc acattcggaa attggtgccg ttgctcctgg aagatggcgg cgaggccccg

1289641 tcctcactgg aaaccgccct ggaagagaag agcgctgtgg aacagatgaa aaaatttctg
1289701 gccgacccac aaattcacac catccttgtc gagagaagtg cgctaaaagg tgacgtttca
1289761 aacaacaatt agttaaattg acaaaaacag cattgcacgc ctggtccggc taaatgtagc
1289821 ctaacagtta acgctgtcat tcgctttatt ttaacgaatt attgaactgc atagtaaatc
1289881 tagaacacct tgcaatttgt ctttgtcaaa aggaaaatcc aattattaaa agggattaac
1289941 tttaccacct tagtgtttag actttccatc aagtaccatt tatttgtgca cacctccgag
1290001 tcgtgttaac aaagctaaca tctatgcagg acagaaataa aaatggaccg tcatgaataa
1290061 aggaatgttt gataactacc ccagtgatgc cttcctattt ctgtttttat catgtgaacc
1290121 agctgtcaat gatcctcttt gtttttgttt gactgccagt aaaatgtggc ctccaagaac
1290181 tgttcttgga gctacttgtg tgagtcttaa gcctagatcc ttatgatctt tcacatgatt
1290241 agtccctgcg agtcatcctc tcagatatta atcagttcca accacatctg ctgtgttaca
1290301 gatttcattt tttgtatctt gtgttgttat tctgctttaa gacgagtgta acaagatatg
1290361 ttctgcaata aagatgacat gctgcatgct tttgttaata gagaactaac attttggttt
1290421 tatttatgct ttgcagagga agttggagat gaaggggagg aggagaaaga gtgcatcact
1290481 tataacgtca gcattgacat acactatggg ttaaaatcca acaggtaaag gaggaatcca
1290541 atcccctcct cgatagtctt ttcttctgtc acagagcttg tcagctgtag tgataattgt
1290601 aaccttaaaa gtaacaattt aactgcaact ggctgtttcc tcctaaaata cataaatgta
1290661 aactctcaat gttttcatat tttcgcgcag cttggcattg atcaagagga caggtgtcat
1290721 tgatgcagac aagcccatat caacacaagt tcgagttctg accttgagcg aggattctcc
1290781 ctatgagacc ctgcattctt tcatcagcaa tgccgttgcc cctttcttca agtcctacat
1290841 ccgcgagtct ggcaaagcag acaggtaatc caccaaacgg gtgtcatgca tggaaaaagc
1290901 attaaatcta attatgtgac actaatttat ccaaatcttc cagagatggc gataagatgg
1290961 ctccatccgt ggagaaaaag atcgctgagt tggagatggg tctccttcac ttgcagcaga
1291021 acattgagat tcctgaaatc agcctgctca tccacgccac catcgccaac attacgaagc
1291081 agtgccacga gcgcggagag aagccaaagg tcacagactt tggagacaaa gtagaagacc
1291141 caaccttcct caatgggctt cagtctggag tcaaccgctg gatcagggaa attcaaaagg
1291201 ttgaaagcct tccaaagaaa aataaatata catacatata ataaatcctc atagtcagaa

1291261 caacatcaat ttcttttttg cacttgtatt gttattaggt gacaaagctt gatcgtgacc
1291321 cagcctcagg cacagccttg caggagatca gtttctggct gaatctggaa cgagccctca
1291381 accggattca ggagaagagg gaaagtccag aggttctgct gacactggac atcctcaaac
1291441 atggcaaacg ttttcacgcc actgtcagtt ttgacacaga cactggtaag tgacaagcag
1291501 gaagaccatt tactctctct tgtgtttttt ttctgtattt attaatattg ttttgaaaat
1291561 aaccattctg ctactggcag gtctgaagca ggcagtggaa acggtcaatg actacaatcc
1291621 tttgatgaag gatttccctc ttaatgacct gctctcagct tcagagctcg ataagatccg
1291681 ccaagctctg atggccattt tcactcacct aaaaaagatc aggaacacaa agtaccccat
1291741 tcagagggcc ctgcgtctgg tagaggccat ctccagggac ctaagctctc aacttctcaa
1291801 agtgttgggc accaggaaac tcatgcatgt tgcctatgaa gaatttgaga aggttggttc
1291861 tttagaactg tttattcaaa aatgattgtg cacattcttt atcatctttt acctcttatc
1291921 atctccgtgt gcaggtgatg gttgcatgct tcgaggtgtt ccagacctgg gaggatgagt
1291981 atgagaaact acaggtcctt ctgagggata ttgtgaagag aaagcgggag gagaatctga
1292041 agatggtgtg gcgtttaagt cctgctcacc ggaagcttca gtcacgtttg gatcatatga
1292101 gacgtttcag gcggcagcac gagcagctga gggccgtcat tgtccgtgtg ctgaggcctc
1292161 aggtatggtc ccagcaatca ctctttttat tctgaataat tctgatgtga tcagatcaca
1292221 ttagatttca acttgaaatg ttttccaggt gtctgccgtg ccacagcatg ctcctggaga
1292281 gacagttgag cctcaggaca tgaaggtcgc tacagttctc tttgatgctg ctgatgccaa
1292341 cgccattgag gaggtcaacc tggcttatga gaacgtcaaa gaggtcgatg gtctggatgt
1292401 ttctaaggag ggcatggaag cctgggaagc tgccatgaag cgctatgatg aacgcatcga
1292461 tcgcgtggag acgcgtatca ccgcccgcct gcgcgatcag ctgggaaccg caaagaacgc
1292521 caatgaaatg ttccgcattt tctcccgctt caacgccctc ttcgtgcgtc cccacatcag
1292581 gggggccatt cgagagtacc agacgcagct catacagcgc gtgaaagacg acatcgagtc
1292641 gctgcacgac aagttcaaag tgcagtatcc tcagagccag gcctgcaaga tgagccacgt
1292701 ccgagacctg ccaccagtgt ctggctccat catctgggcc aagcagattg accgccagct
1292761 gactgcatac atgaagagag tggaagatgt tctcggaaaa ggctgggaga atcatgtgga
1292821 ggggctgaag ctgaagcaag atggagacag cttcagagcc aaactcaata ctcaggagat

1292881 attcgatgac tgggctcgca aagtacgcaa caaatgtttg gtctttattt
ctttttcatt
1292941 cttgcatcac atttcttcaa atgtctgttt ttcttctctg taatccaggt
gcaacagcgt
1293001 aacctcggcg tgtccggacg catcttcaca atcgagagta ctcgtgcccg
tggacgctct
1293061 ggaaacatgc tcaagcttaa agtcaacttc ctcccagaga tcatcacctt
gtccaaagag
1293121 gtccgcaacc tcaaatggct gagcttccgt gtccctctgg ccattgtcaa
caaagcccac
1293181 caagctaacc agctttatcc atttgccatc tctcttattg agagtgtgcg
cacttatgag
1293241 cgcacttgtg agaaggtgga ggagcgatca tccatctctc tgcttgtagc
tgggcttaag
1293301 aaagaggttc aggttctggt cactgaaggt attactctgg tctgggagtc
ctacaagctg
1293361 gatccttatg ttcagaggct ggctgagact gtcttcaact tccaagagaa
ggtctgtaat
1293421 ggatggcatg gttgttttgt cttttcagag tgggaggaaa atggatttta
aatatgtttc
1293481 atgttatttt ttttaggtgg atgatcttct actgattgaa gagaaaattg
atcttgaggt
1293541 tcgttctctg gaaacctgca tgtttgatca caagaccttc tctgacatcc
tcaatcgagt
1293601 ccagaaggct gtggatgacc tcaatcttta ctcctattcc aacctgccca
tctgggtcaa
1293661 caagcttgac attgaggttg gctccacaaa acacaactga attattcttt
ccgtgttttc
1293721 tctgcatgta ttatttcaaa gattgatttt taatttttca tagattgaac
gtatcctcgg
1293781 agtacggctt caggctggtc tcaaagcctg gacccaggtg cttcgtggtc
aggtagaaga
1293841 taaagctgat gtggacatgg acactgaagc tcctcaagtc agccacaaac
ctggaggaga
1293901 gcccaaaatc aaggtaaaca ttatatctat agcgttgcga tattacattt
ttatattagt
1293961 ttctggatgc cactaaataa ccctcttttt aaccagaatg ttgtccatga
gttgaggatt
1294021 actaaccagg tgatctacct gaacccacca attgaagact gtcgctacaa
actctaccaa
1294081 gaaatgttct cctggaagat ggtcatactt tcacttccca gaatccagag
ccagagatat
1294141 caggtagaaa ttctacagtt tgtgtgataa ccattgctga agaaacattt
agaagaagga
1294201 aaatttgatc aaataaatgt atatattaaa ggtaatttgt ccctcctttc
caggtgggag
1294261 ttcattatga gttgtcagag gaggagaagt tttatagaaa tgctctaacc
cggatgcctg
1294321 atggcccagc agccctggag gaagcataca atgcagtgga ggacattgtt
ggtgaagtgg
1294381 aacaatatgt caaggttgga gtcatcatcc aagccccttt taccattcgt
attgcaactt
1294441 aaaggagctg aggtaacgaa attcaacatt ttctctcatt tccttttgct
gtgtctttcc

1294501 aggtgtggct gcagtaccag tgcctgtggg acatgcaggc tgagaacatc tacaaccgtc
1294561 tgggcgaaga cctcaccaag tggcaggcgc tgttggtcca gatccgcaaa gcacgtggaa
1294621 cctttgacaa tgcagagaca cgaaaagagt tcggacccgt tgtcattgac tatggaaagg
1294681 taaaaatctt gatgtctttt tgcataatga aaaaccattc aaattctata tgtttgtaag
1294741 aagaaaacag ttttttcttt attttattgt gcattttgaa tatcaatgtc agtacagtta
1294801 ttttactgaa gtgattgtat ctgtgcatca caacaggtcc agtccaaggt caacttgaaa
1294861 tatgactcct ggcacaaaga agtgctcagc aagtttggac agatgcttgg ccagaacatg
1294921 caggacttcc atgcccagat ctccaaggta ttacattata tccaaaagaa gttttgtttg
1294981 tagagttatt ttgtaaagta aatgaccttg cgctgattgt tctcctgtct ttaatagtcc
1295041 cgtcaagagc tggagcagca ctccgtggac acagcgagca cctctgacgc tgtcaatttc
1295101 ataacatatg tgcagaccct aaaacgcaag atcaaacagt ttgagaaaaa tgttgacgtg
1295161 agcaatcgtc gagaacagaa gcattcttct taaccacagc agatattaag gaaaatttta
1295221 atcagttatt ctttccttta atcttcagtt gttccgcaat ggacagcgtt tgctggagaa
1295281 gcaaagattc cagttccctc cttcttggct gtacattgac aacattgaag gtgaatgggg
1295341 tgccttcagt gacatcatga agcggaagga tactgccatt caacagcagg tggccaacct
1295401 ccagatgaaa attgtccagg aggacaaagc tgtggagaac cgtaccaccg atctgctcaa
1295461 tgactgggag aagaccaaac cagttgctgt aagaaattgg aactaattca attttaaaat
1295521 tgttttattg ttaatcgcca ttgttcacct tttcagaata ctattaaagt gttatgttca
1295581 gtctctactc atataatgtt taaggaaacc atcaaatgtt gtcttgactg gttttcatgt
1295641 taatgtttct gttctaaagg gtaacctgag accagaggag gctcttcagt ctcttaccat
1295701 ctatgagggc aacttcagcc gtctgaagga tgaaagggag aaatgtgcca gggccaaaga
1295761 agctctggaa ctcacagaca ctggggtgtt cagtggcagt gaagagaggg tgcaggtaat
1295821 ttcaacacaa gctgcatttt aaaatgttgg tcaacaaata tcacagaaac actcttgtaa
1295881 cattgtttaa ccaaatactg atgccatgat tctttgtcag gtggcactag aggagcttca
1295941 cgacctcaag gaggtttggt ctgagttgtc aaaggtttgg gagcagattg accagatgaa
1296001 agagcaacct tgggtttctg tccagcctcg caaggtagct aatcctcatc aactatttta
1296061 ccattttacg cttcgccttc ttgtgtgtgt agctggttca tgttggtgtt ctgtgtttct

1296121 agctgcgcca gagtctggat gctcttctga atcagctcaa gaacttccaa gcaagactga
1296181 gacagtatgc ctcctatgaa tatgtccaga ggctgcttaa aggatatctc aaggtctgaa
1296241 tgcaaccttt attgtaatca aagtttaccg atcccccccc cccccaaata aaattagtcc
1296301 ttattttaat ctttccattc aggtgaacat gttggtgatc gagctgaaat ccgaggctct
1296361 gaaggaccgc cactggaaac aactaatgaa gcgtctccat gttaactggg ttctgtcaga
1296421 gctgaccctg ggacaaatct gggatgttga tctgcagaag aatgaaatgg tggtgaagga
1296481 tgttctcctg gtagcccagg gagaaatggc tttggaggag ttcctcaaac aggtaataat
1296541 tgactcccac tccaaattat aaaaaaaata cctatgtagt gtaacaattt gccatttctg
1296601 gtctttagat ccgtgaggtg tggaactcat atgagcttga tctagtgaac taccagaaca
1296661 agtgtcgcct gatccgaggc tgggatgacc tcttcaacaa ggtcaaagag cacatcaaca
1296721 gtgtttctgc aatgaaactg tctccatact acaaggtaaa acaagtaact tattgttcac
1296781 tataaaacgg tttgtaaatt ttatggacct catcaacagt ttgctctttc ttttgttctt
1296841 gcaggtattt gaggaagatg ctctgagctg ggaggacaag ctgaatcgca tcatggctct
1296901 gtttgatgtt tggattgatg tccagcgtcg ctgggtctat ctggaaggca tctttactgg
1296961 cagtgctgac atcaaacatc tgctgcctgt agaaactcaa agattccaga ggttattttt
1297021 tttttattca gtagaccaaa tgctcacagt aactttgtat ttcttgctta atttcatgtc
1297081 ttttttgtga tgttttacac agtatcagca cagaattctt ggcattgatg aagaaggtgt
1297141 caaagtctcc tttagtgatg gatgttctga acattcaggg agttcagaga tctctggaac
1297201 gactggctga ccttctagga aagatccaga aagcactggg agagtacctt gaaagagaaa
1297261 gatcctcttt cccaaggtaa cagagtgtca ttgcatagca agagaccaac attttgccag
1297321 ttttcaaatc aaatatttaa aaaaatctta ttttttaaca ggttctactt tgtgggagat
1297381 gaggacctgc ttgaaatcat tggtaacagc aagaatgtgg ccaaactgca gaagcacttc
1297441 aaaaagatgt ttgctggtgt ctctagcatt ctgctgaatg aggacaacac tgtggtgctg
1297501 ggaatctcct cacgtgaagg agaagaaata ctctacaaaa caccggtgtc catcacggat
1297561 catccaaaga tcaatgagtg gctgactctg gtggagaagg agatgagagt gactctggcc
1297621 aagctgctgg ctgaatctgt cacagaagtc acgtctttca acaagggcac tgctgttgac
1297681 ctgagtcagt acatcacctg gatcgaccgc tatcaggtgt gtatttaact gtacacattt

1297741 acttatttat tatgcgggaa aaggcaggat gttacacaca cgtttttttgt tttcacaggc
1297801 ccagttagtg gtactgtcca ctcagattgc ctggtctgag aacattgagt ctgcactgac
1297861 caccatctct ggtggtggag acatgacact catgcaagga gtgctgtcaa atgtggaggc
1297921 cactctcaat attttggctg acactgtgct aatggaacaa cctcccctcc gcaggaggaa
1297981 actggagcac ctggtaagaa ggatttcggt tttgtcgcat aaaggtgttt gacaatgttg
1298041 gggagactaa aatcaatctt ttgcttcact ttaagatcac cgagcttgtg caccaacgtg
1298101 atgtgaccag gaccctgatc aaaaacaaga tcgacaaccc taagtccttt gagtggctga
1298161 gccagatgcg cttctatttt gaccccaagc agacagatgt tctccagcaa ctgtccattc
1298221 agatggccaa tgccaagttc aactatgggt ttgagtactt gggtgttcaa gataagcttg
1298281 tccagactcc actgacggat cgctgctatc tcaccatgac ccaggctctg gaagctcgcc
1298341 tgggaggatc accatttggt atagaagctc tttagttagg tcctatggtg actgtggttt
1298401 cataatgaag taaatgttta catagggggaa acttcttttt cattcaggcc ctgctggtac
1298461 aggaaagact gagtcagtga aagctcttgg tcatcagctg ggccgctttg ttctggtctt
1298521 caactgtgat gagacatttg atttccaggt atgtgaatgc atttgcatta ttctaaattg
1298581 taaatttctt tttgttttaa aacattaatc acatcttgaa caggccatgg gtcgcatctt
1298641 tgtgggtctg tgccaagtgg gtgcctgggg ctgctttgac gagttcaacc gtctggaaga
1298701 gagaatgctt tctgctgtct ctcagcaagt ccagtacatc caagtggcct tgaaggagca
1298761 cagcaacccc aacagagaca gaagtatgac taataattaa aaataaatga taaataatac
1298821 agatttagat ggaacactag ctgccaccaa atatgttaat ttaaaaaaaa tagtaaattt
1298881 atttcttaaa aattaaattt gtggacctag ttgtttttaa atagaagttt ccatgataaa
1298941 cacctttaat ctctataggt gtgccagtca tgacagagct gctgataagc aggtgaaggt
1299001 cagtccagac atggccatct ttatcaccat gaatcctggc tatgctggcc gctccaacct
1299061 tccagacaat ctgaagaagc tgttccgtag tttggccatg accaaaccag accgtcagct
1299121 cattgcccag gtcatgctgt actctcaggg ttttcgcaca gctgaggtcc ttgctaaaaa
1299181 gattgtgcca ttcttcaagt atgtagagtt gaaacaaata tctacatagc tttggggggg
1299241 tgctctttat ctaatgtgtg cttccttctg tgtcaggcta tgtgacgagc agctgtcgtc
1299301 acagagccat tatgattttg gcctccgagc tcttaagagc gtactcatca gtgctggtaa

1299361 tgtcaaacgt gaacggatcc agagaatcaa gaaggataag ctggagcgtg gggaggtcgt
1299421 tgatgagaat gatattgccg agaaccttcc tgaacaggag gtatgaaaat caatattgtg
1299481 tgatgttaat ttgttgctgt tactacactg gatatttgtt attgtagatc ctgatccaga
1299541 gtgtgtgtga gaccatggtt cctaagctgg tagcagaaga catccctctg ctgttcagcc
1299601 tgctgtcaga cgtcttccct gggatccagt accaccgtgg agagatgact gctctaagag
1299661 aggagctgaa gaaagtctgt ggggagatgt acctcaccta tggagatggt gatgatgtgg
1299721 gcagcatgtg ggtggagaag gtatggtgaa aaatgaagtc cagctatgtt gctgtggcta
1299781 aaatgttgat cttgtttttc tgaaacttgg acatttcttc tctctcaggt tcttcagctg
1299841 taccagatca cacagatcaa ccatggcctg atgatggttg ggccttcagg cagtggaaag
1299901 actatggcat ggagagtgct tctcaaagcc ctggagaggc tggagggtgt tgagggtgtg
1299961 gcccacataa ttgaccctaa ggccatcagt aaggaccacc tgtatggaac cctggacccc
1300021 aacactcgtg aatggactga tggattgttc acacacatcc tcagaaagta agtttggttt
1300081 tgaaacggga aaatatagag tttgagaaaa agcactgttg gaaaaacatt ttaaaaaatg
1300141 tttgtttttt tttattcaga ataattgaca atgttcgagg tgaactgcaa aagcgtcagt
1300201 ggatcatctt tgatggtgac gttgaccctg agtgggttga aaatctcaac tctgtcctgg
1300261 atgacaacaa gctcctaact ttgccaaatg gagagcgtct cagcttgcca ccaaatgtaa
1300321 gagtgcagta gcctaaacct aacttttttt tttgtttttt ttactcaaaa tattttcttt
1300381 ttctaggtgc gtgtgatgtt tgaagtacag gacctgaagt atgctaccct cgccaccgtg
1300441 tcccgctgtg gcatggtgtg gttcagtgag gatgtcctca gcactgatat gatcttcaac
1300501 aatttcctgg cacgcattcg cagtattcca ttggatgagg gagaagatga agctcagcgc
1300561 cagaggaagg gcacagagga tgaggaagag gttgcatcac ctatgctgca ggtgaacaaa
1300621 gaggggaaaa atgagtttga aacaaatgtc agttgaagca agggtttgtg gagctgtttt
1300681 ttcagcagaa ataaatggtt ggtgttctgt tgtgattttc aacagatcca gcgggatgca
1300741 gctggaatcc tgcagccata cttcacctcc acaggcttgg tgatcaaggc cttggaacat
1300801 gcctcaaagc tggagcacat tatggacttc acacgcctgc gctgcatggg ttccctgttc
1300861 tctatgctgc accaggcctg ccgtaatgtt gcgctctaca ataacaacca ccctgacttc
1300921 ccaatgccca ttgatcagct ggaaaaatac atgcaggtga tgtaattttc agtctctgaa

1300981 gacaatttta aaatggcata aaagcacata tattccagtc atacaagggt tattcacacg
1301041 cattattaat aatcatcttt tttttttttt tttttttaca gaaatatctg atctatgctg
1301101 tactgtggtc cttctctggt gatggacggc tgaagatgag ggctgagctt ggagaataca
1301161 tccggcggat caccactgtg cccctccctg ctgctcccaa tgtccccatc attgactatg
1301221 aggtacggtc ctcagagcca gcaagattaa tacttgtttt aagggaatcg agttgatgta
1301281 ttcttgcatt cagaaagtca cacaataatt attagtgtct ttaatttgat ttggccatta
1301341 tctgtatcag gtgtgcatct ctggtgagtg gcagtcctgg caaggcaagg tgccacagat
1301401 tgaggtggag actcacaaag tggcatcccc cgatgttgtg gttcccaccc tggacaccgt
1301461 tcgtcacgag gctttgcttt acacatggct ggcagagcac aaaccgctgg tactgtgtgg
1301521 gcctcctggc tctggaaaaa ccatgacttt gttcagcgca ctcagagcat tgcctgacat
1301581 ggaggtcaga ctctaaaaaa aaaaaatgac ttaagcacaa ataattcctg tctaaactga
1301641 attgatatgc atttgagttc ttcttcactc cgctaggttg ttggtctaaa cttctccagt
1301701 gccaccactc cagaattgtt gctgaaaact tttgatcatt attgcgaata ccgccgtaca
1301761 cccagtggtg tggtccttgc cccagtgcag ctcggcaagt ggctggtatt attctgcgat
1301821 gagatcaatt tgccagacat ggacaaatat ggcacacaga gagtcatctc ttttatcaga
1301881 caggtatagt aaattcttat gttgcacttg cagcactttt atggccctgc ggttaaattc
1301941 attgcctata tacgtatgtc ttcttgaaag atggtggaac atggaggatt ctaccgcacc
1302001 tcagaccaga cttgggtcaa attggagagg attcagtttg ttggagcctg taatcctcca
1302061 actgatccag gcagaaagcc tctcacccac aggtagacat ctcggtctta aatgcagaat
1302121 atcctgccat ttcacagcta taaaatatac cattgtgaag aaaaataata aaaaatgaca
1302181 gctacacaaa gctacacaaa ttcagagttc ctgcaaagag gaccaacact gtccggatgt
1302241 gctgtttcca ggttcctgcg ccatgttcct gtggtgtatg ttgactatcc aggtccagca
1302301 tctctaaccc agatttatgg aaccttcaat cgagccatgc tgcgcctcat cccatcgctc
1302361 cgtacttatg ccgagcctct tacagctgct atggttgaat tttacaccat gtctcaggta
1302421 aaacggtttc taaatattgc atgctagata aactgttcaa acccttttct gacatgtttc
1302481 gagttattct gttaacattg ccttttattg atgtgttcac attctggatc tgaatcttca
1302541 atttcaggag cggttcactc aggacacaca acctcactac atctactcac ccagagagat

1302601 gacccgctgg gtgagaggaa tttttgaggc tcttcggcca cttgagactc tgcctgtgga
1302661 gggactcatc cgcatttggg cccacgaagc tcttcgtctc ttccaggaca ggtacagctg
1302721 gactgtgatg ctcaaatgta ttgcttaaat gctttgtcac cagaattgtt ttaatcagtc
1302781 taacactttt tgtaggctgg tggatgatga agagagaagg tggacagatg agaacattga
1302841 catggttgct cttaaacact tccccaacat tgataaggat aaggccctga acagacccat
1302901 tctctacagc aactggctgt caaaggtaac ctggagatct gtttctttgg tgacaacggt
1302961 cttcaacaaa gaacaattta acagcagttt gtactgagag ttgtttttgg atgcaggact
1303021 acatcccagt ggaacaggag gagctgagag actacgtgaa ggcccggctg aaggtcttct
1303081 atgaggaaga gctggatgtt cctctagtac tcttcaatga ggtgctggac catgtcctca
1303141 gaattgaccg gtgagccaga aaataattaa aatgggactt tctgctcaga ggtaattatg
1303201 attgagtcac ttttaaaatt gtatcttcac agaatcttcc gtcagcctca gggccatctc
1303261 ctgttgatcg gtgtgagcgg agctggaaag acaactctgt ctcgctttgt agcctggatg
1303321 aatgggctca gtgtctatca gatcaaggtt agtctgccac atatagatgt tttagcaata
1303381 ttattacaac tgtttgtcat attaaaatac ttttgcacat cttcaggtgc acaggaaata
1303441 tacaggagag gactttgatg aggacttgcg cacagtgctg cgacgctcag gttgtaaaaa
1303501 tgagaagatt gccttcatta tggatgagtc caatgtgctt gactctgggt tccttgaaag
1303561 aatgaacaca ctattggcca acggagaggt aaatgatgat gtcatgactt gcaagcttta
1303621 cgacttctta acagaaaatg ctttttctta caaatgtttc attgctgtta ataggttcct
1303681 ggtctgtttg agggagatga gtatgctact ctgatgactc agtgtaaaga gggagcacag
1303741 aaagagggtc taatgcttga cacatatgag gagctctaca agtggttcac tagccaagtt
1303801 atcaggaacc tccatgttgt tttcaccatg aacccctcat cagagggcct gaaggacagg
1303861 gctgcgacct ctcctgccct cttcaacagg tatgactgaa caccaggcat gaatgtacag
1303921 ataccggtac aaatcctcag ttaacaagaa atatctttct tagatgtgtc ctgaattggt
1303981 ttggagactg gtccacacag gccctgtatc aggtgggtaa ggagtttacc agcaagatgg
1304041 atcttgagaa acccaactac aaggtgccag actacatgcc catcgtctat gacaaactgc
1304101 cccagccacc atctcaccgc gaggcaattg tgaacggatg tgtgtttgtg caccaaacac
1304161 ttcatcaggt acttgcttgt gttcaatgta ctgattgatg gaattattgt cctgtaaggt

1304221 ttaaccagta tttgactcag actgatgcat gactaatgtt gatgtcttct ctaaacaggc
1304281 gaacaaccgc ctggctaaga gaggtggtca cacggtggcc atcactcctc gccactacct
1304341 ggacttcatc aaccagtacg ccaacttgtt caatgagaaa cgtagtgagt tggaagagca
1304401 gcagatgcat ctcaatgttg gtctccgcaa gatcaaggag actgtcgatc aggtgctgca
1304461 gtttttgtat ttgtattttt ctcactgcat gctaaatggt ttttgttctt gtctaactat
1304521 atattttctt ggaaaaaaag gtagaggagc ttcgtcgtga cctaagaatt aagagtcaag
1304581 agttggaggc caagaatgct gctgccaatg acaaactgaa gaagatgttt aaagaccagc
1304641 aggaggcaga aaagaaaaag gtacacacat ggggtggatt tctaaggctg cgcctgagta
1304701 gaaaaaagtg aaccaggcaa ctattatatt gataataata aaacaattaa cagtaatacc
1304761 gataaagtgg tatatgattc tttatacaac attcaaacat atagtggtat aagtgagaga
1304821 accaatatac agccccaaca ttaaataatg catcagtttt aactgtgatt ccaggtgatg
1304881 agccaggaga tccaggaagc tgtatacaag cagcaagaag tgatcaagga caaacaactg
1304941 agtgtcaaac aggacttgga tcaagtggag cctgctgtca ttgaggccca gaatggtagg
1305001 gttgtacaat ttgtagcatg ttacttgttg ataaaaaaat aataatatta acgatacaat
1305061 gtctgttatt taagtctttt caaatttata atattaccac acagttgcaa aggcttcttg
1305121 ttcacgatgc aaccattaac ttcatcctcc cattattgcc tcccctgcct tgtaataatc
1305181 cacccсttgt tcatctgatg ctgatagctg ttaagtccat caagaagcag cacctggtag
1305241 aggtgcgttc tatggccaat ccacctgcag ctgtgaaact agctcttgag tccatctgct
1305301 tgctcctggg agaaagcacc actgattgga agcaaatccg ttccatcatc atgagggaga
1305361 acttcattcc cactattgtt aacttctctg ctgacgaaat caggtgaaac aaaacatttc
1305421 tggaatcaac tgatgttttc tcttcatgtc catctctgtc tctctatatg ccccccсaca
1305481 cggcatcacc tttcattgta aaaacaatgg tccttcctgt cgtagcggtg agttctatta
1305541 agcggcagca cctagtagaa gtgcgggcca tgaccaatcc cccagctgct gtcaagctgg
1305601 ccttggagtc tatctgcctt atgctgggcg aggagacggg tgactggaaa aaaatcagac
1305661 aagttattat cagggaaaat ttcatcagca gcatagtcag cttttcctct gaagaaatga
1305721 ggtacaggtg ttaactgcta gtcaccgata gaaatgtact aaatccagca atcagcacaa
1305781 caatcaagtg gcatctgttt gatttctaca ttttgtcaca cttttcaaat cagacaatga

1305841 actggcagta ttataaccac aacctctagt tattagattt actgcgattc tacattcctc
1305901 acactaaaac ctgttgaaat atctcaccca tttgcaatga tcatgtataa tttttcact
1305961 ccaattgaat atccagtatc aaccctctgt ataatcataa atttatgctt tactcaatcc
1306021 tgcttcctcc tccagtgatt ccatccgtga gaagatgaag aagaactacc tctccaatcc
1306081 aggctacaac tatgaacaaa tcaacagagc atcactggct tgtggtccta tggtgaaatg
1306141 ggcaattgct caggtaagac caaactatat taaaatgaat acagcactgg cttgaagaag
1306201 tgctccttgg atgaatacat ttattgcctt tttagcttaa ttatgccgac atgctgaagc
1306261 gcgtggagcc tctgcgcaat gaactgcaga agctcgagga tgatgccaca gataacaaga
1306321 caaaagcaga ggaggtggag cagatgatca gagacttgga gtctagcatt gctcgctaca
1306381 aggaggaata tgctgtcctc atttcagaag cccaggccat caaggctgac ctggcagctg
1306441 tagaagctaa ggtaaatgat cattaaagtg atgttcagtg gctttttttc cccgttgcat
1306501 caatcacgca gcgcttttta aacttgcatt gttttcttgt aggtgaatcg cagtacagcc
1306561 ctactgaaaa gtctgtcggc tgaacgggac cgttgggaga agaccagtga aaccttcaag
1306621 aaccagatgt ccaccattgc tggagactgt ctgctgtctg cagctttcat cacatatgct
1306681 ggctacttcg aacagcagat gagacagaat ctgttcacga cgtggtctca tcacctgcag
1306741 caggccaaca ttcaggtact taaagctgag tttctggctt cgttcgcatc actagttgat
1306801 gttttttgac aaatctactg tatgtggagg cacacgttgc gtttcctctc caccattgta
1306861 gcttccattt accccatttt ttctgttaca gttccgtaca gatattgcaa gaactgagta
1306921 tctgtccaat gcggatgaaa gacttcgctg gcaggccagc tctctcccag ctgatgacct
1306981 ctgcactgaa aacgcaatta tgctcaagag gttcaacagg tcagtttcat ctagcactgt
1307041 aacattttga ctttttaaaa tgacaagcat ttccttcttg gcctcgactc aattgtatct
1307101 tcgttctgtg caggtatcca ctaattattg acccgtcagg ccaggccaca gagttcatca
1307161 tgaacgagta caaagatcgc aagattacca gaaccagttt cctggatgat gccttcagga
1307221 agaacctgga gagtgctctg cgttttggaa accccctgtt ggtccaggta tgccttgctg
1307281 ctttacactg gtatcattgg gtcaggtgtc atcctgaatt tatctacaag aggatgtttt
1307341 tatgtttgct cttaaggatg tagaaagtta cgaccccatc ctaaacccag tgctgaacag
1307401 agaggtcaga agaactggag gacgtgtcct catcactctc ggagaccagg acattgatct

1307461 gtcaccttca tttgtcattt tcctttctac aagagatcca acagtaggtt aaagtctatt
1307521 cttaaaaccc tggtcttttc gaggtatgga tttgtacaaa gaaattggct tgtctaattt
1307581 gttgtcactt ttctgtgcag gttgagttcc caccagattt gtgttctcgt gttacatttg
1307641 tcaacttcac tgtgacacgc agcagccttc agagccagtg tctcaatgag gttctcaagg
1307701 ctgagaggcc tgatgtggat gagaaacgct ccgacctcct caaactgcag ggtgagatgg
1307761 caaatataca agtgcaaata gttggcaaaa ctggaaacat aatggctgtt tgcttgagat
1307821 aatcgagata attagaactc accagcatct gttccttctc ttgtaggcga gttccaactg
1307881 cgtctgcgac agctggaaaa atccctgctt caggccctca atgaagttaa agggcgcatt
1307941 cttgatgatg acaccatcat taccacactg gagaacctca agaaggaggc ggccgaggtg
1308001 accaggaagg tggaggaaac ggacatcgtc atggcggagg tggagacggt atcgcagcaa
1308061 tacctgacgc tgtcctctgc ctgtagcagc atatacttta caatggaggc tctcaatcag
1308121 gtctggacac aatcaccctc tccctccaga gctttttaa atcaacaaac taaactttag
1308181 aaataatatt tctgacttct aatatgagtc acataaacac actgctttgc ttttctccag
1308241 atgcacttcc tctaccaata ctccctgcac tttttcttgg atacctacca cactgtgctg
1308301 tatgaaaacc ccaaccttaa gagcatcagt gatcattcac agagacttga tgtcctcaca
1308361 aaggacctct tccaggttcg aaccgttcta tgaataaatg tagttgctgt gtttggaata
1308421 gtattaatag acataccggt aatttccttt ttaggtggta tttaacagag cagccagagg
1308481 catgctgcac caggatcaca tcacttttgc catgcttttg gcaaagatta gcctgaaggg
1308541 tattaccagg tacacacagg aaatacagga actctctaca gtgattttca gtgaccagga
1308601 tttctaatta tggtgtaatt ctccccagcg agccctcata tgactccgag ttccagcact
1308661 tcctgcgagg taaagagatc gtcctgactg gcatgtcact gcccaaagtg aaaggtctga
1308721 gcagtgagca gagtgaggcc atggtccgac tcagtcgtct gccagcattc agcgacttgg
1308781 tgaacaaagt cgaggctgat gaggtgagcg taattgctcc tagttctact gtgacatacg
1308841 atctgatcaa cttgcttggg acatttctaa atgtttttt aatttctttt tttttcccta
1308901 gcaattcttc atgtggattg agagcaacac tccagagctg gctgtgcctt atctgtggtc
1308961 agaagagaag cagtccagta agtctacgcg tctgtagggt ctcaaacaat gattcttctg
1309021 tcttcatatg tgaaatgctt tgctttaatc tgttcagccc ccattggcca ggcagtgcac

1309081 cagctgttgt tgatccaggc attccgtcca gaccgtctgc tcgctatgtc gcacattttt
1309141 gttgcaaagg tgctgggaga atccttcatg agcatcattg agcagcctct tgacctgggc
1309201 aatgttgtgg agaatgaggt agaatcactt cagtaaaagt atttaacctc tggaagtgtt
1309261 taccctcatc agtgttgaca cgttgctgac attctccatt ttgacacctg gctgcaggtg
1309321 aagcccagca ctccagtgct gatgtgttct gtgccaggtt atgatgccag cggcctggtc
1309381 agagatcttg ctgctgagga gaacaggcaa atcacctcca tctctattgg tatggaagca
1309441 ataactaaaa accatctgaa gtttagtgag ggaattgtag ttaaagcttt gtgcttttcc
1309501 actgttcaca ggttcggccg agggtttcaa taaagcagac aaagacatca acactgctgt
1309561 caagtctggc aggtaggtag catgttctgt gttttctgaa catctagact tgttatcgca
1309621 tcctcaccgt tgtggtctga caggtgggtg atgttggaga acgtccacct ggctcctgca
1309681 tggctcatgc atctggaaaa gaaacttcac tccatgcagc ctcatgcctg cttcaggctc
1309741 ttcttgacaa tggagatcaa ccccaaggta agcctcagaa tttaaatgtt tcctagtttt
1309801 gataaaatta gtgtgatcct tatattggct ttactttcag tcattgtgaa agtctaagta
1309861 ttgtattctc cttgtaggtg ccagtgaatt tacttcgtgc tggtcgcatc tttgtgttcg
1309921 agcctcctcc tggtctcaag gccaacatgt tgagaacctt cagcaacatc cctgtagctc
1309981 gcatgtgcaa ggtcacttat tttggaaaaa ccactatatt aatctctcaa tccaaaacca
1310041 tgtattctct atattaaata catgtatatt gtatgtacag gctcccaatg agcgtgctcg
1310101 tctctacttc ttgctggcct ggttccacgc agtcattcag gagcgtttgc gttacgcacc
1310161 gctcggatgg tccaagaaat acgagttcgg agagtcggat ctgcgttctg cctgcgatac
1310221 cattgacact tggctggatg acactgctaa ggtcagggtt cattttccaa aatgatgcct
1310281 ttgaattgtg ttaacactcc agaccggtat aagtttggtg acgcaaaaat atgtgacgtc
1310341 aaacatgtca acacgcaggg tcgccagaat atcgctcccg acaagatccc ctgggctgct
1310401 ctgaaaacac tgatggctca gtccatctat ggtggccgca tcgataatga gtttgaccag
1310461 cgcctcctca tcaccttcct ggaacgcatc ttcaccaagg gaagctttga cagtgaattc
1310521 aaactggctc tcaaggtcga cgggcacaag gatctgaaga tgcctgatgg cattcggtca
1310581 gtactggcaa actgaatggt ggtttttatt tgggaaatat ttatttgtgg tcattgttta
1310641 aatgacggtt ttcatttgtt gcagccgcga ggagttcatg cactgggtag agatgctgcc

1310701 tgacactcag actccatcct ggctggggtt gcctagcaat gctgagaagg tcttgctcac
1310761 cactcagggt atgccccgat catctgtact cagtcgctgt accaaaattc accacagttc
1310821 ttcctttaag gctttcatgg tcacacaagt gtcatcatta accatgtctc tctattacag
1310881 tagctcttta agtctagcaa ggtgcatcag cagcacagaa tgggtacttt atctgtttta
1310941 gatggaagca aacgaggggt ttcttttgct tccattaatg ttttgtttct tgagtagcta
1311001 ctaattgtac agtaggagaa ggaaggtgtg tatgtcctca ccctccagca gctgtatttc
1311061 acagccatca tgtctgctat gctaaatttt tatctcctcc ctaccgtcac ggtacagttt
1311121 tgaatgggaa tcccaccctg tttttgttag agctccttac cacctcctcc cgtccctgca
1311181 aaactggctg cagaagcacc caccttcaac cacccccctg ctccattatc cccattccca
1311241 tccccatttt tttgttgaat tcctatgtta attttgttta cggtggcatt cagtcaagaa
1311301 tgtggtttta ttttttccac ctgggaactc tgttgcttgt gttggattga aatggtgcct
1311361 gaagcctttt gtgttttgtt ttttcctacc cggtgaactg tagtcctgat ttgtttgtct
1311421 gtaataacat gacctcacgg taagtttctg tgtctgttat tctattaatt tcttttcccc
1311481 tccacctcct acaatggccc tttcttccac ctccttcccc tgctcacttg ctccccctgc
1311541 tgggctgcac aggcactgac atgatgggta agatgctgaa aatgcagatg ttggaggatg
1311601 aggacgacct cgcctatgag acggagaaga aggagcgcac ctcctccaca tcggatgctc
1311661 agccggcctg gatgaggacc ctgcacacca ccgcctgcaa ctggctgcag ctcatgccac
1311721 aaacagtgaa tccactcaaa cgcacagtgg agaacatcaa ggtaatgtca gacatgtgag
1311781 gagaggaggt tgggaagaag acttcacagg ataaggctga aatgatgtgt gtgctgttct
1311841 gtgatttgtt aatgtttcgc gcctcacaag gtgacgaggt aatttctgag aatttcattc
1311901 tcctgtggaa gttctgtcat tgatggaatg ccttgttatc caacacgcaa ccatcaggac
1311961 ccgctgttcc gattctttga gcgcgaggtg aagatgggcg gcaagatgct gcaggatgtc
1312021 cgtcaggatc tgtctgatgt ggtgcaggtg tgcgtgggca agaaaaagca gaccaactac
1312081 ctgcgcacgc tcatcagtga ccttgttaaa ggtacccatg ttattgtaat ctgttgatca
1312141 gcttatatcc gggttattta gtgagaggtc ctctggagca gacggcaaac ttgcgtttac
1312201 ttctcctcag gcatcctgcc tcgtagctgg tgtcgctaca ctgttccagc ctccatgacg
1312261 gtgatccagt gggtggcaga cttcagcgag cgcatcaagc agctgcagat gatctcccag

1312321 ggagctgcca ccggaggtgc aaaggaacta aaggtgctgt tcgacttggt gcatgttaaa
1312381 tgatgatcct ctgtgccaca tgccacactc atgcttcccc gcctcctctc ttctggggtc
1312441 tagaacatcc atgtttgtgt gggcagcctg tttgtgccag aggcttacat tactgccacc
1312501 cgccagtatg tggctcaggc taacagctgg tccctggagg agctgtgtct ggaggtcaac
1312561 gtcaccgctg cacagggggc ctcactggat gcctgcagct tcggcatcaa aggtcagacc
1312621 aggcggcaaa tctgctcctc ttggcgccgc cttcgaataa tagaaagaac aaatgtgtac
1312681 aggataaaaa caacttaagg ctgagaatca ttccaagcct tcatttatct tgtccttatt
1312741 taacccctga tgttgaacat catcgttgtt ctcccaggtc tgaaactaca aggagcaact
1312801 tgtgccaaca acaagctgtc tctgtccacc accatctcca ccgagctgcc cctcactcag
1312861 ctccgctgga tcaagcaaag caccaccgag aagagacaca cggtaaagga tcagcagcct
1312921 aaaaacaagc ccgaaaatcc accggacagt ttcggttaca aaaactctgc cgacgatacc
1312981 ttcgctgact tatttccatc tgtgtctcga tgcaggtgac tctgcccgtg tacctgaact
1313041 tcacccggtc tgacctcatc ttcaccgtcg attttgacat cgccaccaag gaggatccac
1313101 acagcttcta cgagcgtggt gtcgccgttt tgtgcaacga gtagaccatg gtcgctcttg
1313161 aagctccaga gctgcccgac gatagtcatg catgacgtag attcctgact cattaaccac
1313221 catgttagaa agtagtttgc agcctgtgtt ctaaaaataa tcagtagtta aacatatggc
1313281 agccttgtaa ctcgtctgtg gaaggtgttt ttagaaggtc aaggggaggt tgtcagatgg
1313341 tcactgaaga gaagatgaaa tgttggaaaa ggaaagttgg aagtttgatt ttatggaagt
1313401 agctaatgtg atttttagat ccagttaatt ctgttgcaat aaaaatgaac ccaatcaatc
1313461 accactgtca catccctctt tttccatcct ggtcttttct ctcaaacagc tgcaggctct
1313521 tttgaaagaa gagtgcttaa atttaaagct cggaagctct tttctgggaa gaatatccgc
1313581 ctttaaggaa gcgtttcacc tcaaacaata tttttccgac tcttcctcaa atgtggcttt
1313641 taagactttc caatgaaagt gaggatatta aatactgtaa caggcctggt ggttattaca
1313701 gagatgggga aactaatcca acaaaggaaa tgaatcagac tcattttgag gccctggtat
1313761 ttgtgtttta atatatataa accattcaac tagatcagta ttacacaata aacattctcc
1313821 tgccctgtgg aacgccaact cgccagtgaa cttatgagaa acatcaaccc agatctttaa
1313881 cctccattgg agatactggt ggacatcgcc gtttctgtga tgtgcacctg tgtcctcaat

1313941 ccagtgtagc tatgtagcaa cagttttcca catttaaagt tgtgttaaac tgatttggac
1314001 aagcagaaaa tcagacgtgt gcgagagacc aactacaggt gtataaaaca tagatcacca
1314061 aacaaatcaa tagccttccc ataaagtgcc tgagttcctt tctagttcta gaattccggg
1314121 tcagtccgtg ttgacgtggc tcagccctgg ttgtgacgcg gacgctcctt cctccatgct
1314181 ggggacgcgc tgcctcttcc tctgccccag ctccgacact tctgctccgg gaaagcacat
1314241 ctttctcttg taaaggcagg gactggggga cctctcagag gagctgtcgg gcaaattccc
1314301 aggtcccacc gtggaaggaa gaggagcgtg caccccggaa tcatccagca gctgcacatc
1314361 catggtttga gcacgcaccg ttgactcttc aaggtcgacg ccaccacctt gctcaccatc
1314421 tgagggcctg aggtcaatct cacagagcca cagttcagcc tcactctcac atgtcatctg
1314481 gacatccagg agaggctgct ccgatgctgg atccctgcag ccactcttca gcaggaggaa
1314541 cctcctgttc ttgcccagga acctgcagct tcctcctcct gcaccactgc tccgtctcgc
1314601 ccaccttctg ggagcagtca gatcggactc gtcagaatcc tcagagcttc catcatacgg
1314661 atccagaagc tgcaatcagg aatacatatg ggcatgaatg caaacacaca tagcgacaat
1314721 gcgtgcgtgg cagaaaatac ttcaataaat gagtaaaaat atgtgtttac caaggaacag
1314781 tcatgtcttc cacggggctt gtgcattaat ttaagtgttg gagtctctgg ggtgctgctc
1314841 atacatccct aaacaacagg aaagtcaagg attttactcc aacaagaaaa aaaaaaacca
1314901 tctatgtacc cagaaatgtg tgcattattt aataagtcga tataaattgt gcagaaatag
1314961 cgacaaaacc tataaaatac caaagtgctg gtgtctgacc atctcgggct gatctcagag
1315021 tcagagtctg tgctggattc cactttacac atttcctggg gaaagaaaat aaacctcaca
1315081 caaaaaaata tcccataata aatcaagtac ggaatttccc acaatgagtg atgagatgtg
1315141 gacacgtgaa cgcaccatgt atttgaaaga aagctgctcc acggtgctgg gaacatcact
1315201 gtggtctaag tgtgaaataa atgaccattt cagtagaata aatgtccctc ttccaggtta
1315261 aaccactaaa taaataatat tgtttcttga aggtgttcgt tacctgaatt gtttagctgt
1315321 cgtcttcttc tgtacgccat gacatttata ttctatgatc tttgggccat aaaaccctcc
1315381 gatatgtgga ggatagctgg taattaatga tcaggtgttt ttactctcaa acagcgtcga
1315441 aatcgagttt cccaaacttt ccccaaatac gtccggtgtt cggtcgtttt caaaataaag
1315501 ttcatacggt ccctgttcgc agagctttta aattagtcgt tttcaaatgg ttctatagcc

1315561 gacattaatg ttgcataaac cacatttttg tttttattat tttttcccta tttaacgaaa
1315621 taatctgtaa ttataggaca ggttttgggt catccctgaa gtcattgctc cacggcccct
1315681 tggctgtggg acttgtatca aaagacattt ttaaagggac tttattgctc cactcgggtc
1315741 cccctcgggc agttctccgg ggagccgccg gcccgggtcc tccccggccc gggtcctcct
1315801 cggcccgctc ccctccctgc gtaagatccg gccgggggga aagggatgaa tgctaatttc
1315861 aaagatggcg gcggagggag gagggaagga gatgaacgaa atcaaaactc agttcaccac
1315921 ccgggaaggc gtctacaaac tcctcacgca ctccgaatac agccgtccga acagggtgcc
1315981 tttcaactcg cagggctcca accccgtcaa ggtctccttc gtgaacgtca acgaccagtc
1316041 gggcaacggc gacaggatct gtttcaatgt gggccgagag ctctacttct acatctacaa
1316101 aggcgttcga aaggtagcgt gctagcgcgg ctagtgtcag cggggatgcg ggacgtcgca
1316161 tgtgtccagc ccgccgccgc tgcccgacct ggttgccgaa ctttgggctt gtttgggtcg
1316221 tatttaggaa ctctggcagg tgtttgactt tgaaattagc tgacagccag ttagctccga
1316281 gctagctcga aactagccca ccagtcggct aaccagcgga actagctaag gttagcacac
1316341 ttcgcagcta ctcccgaggc caggcactaa attctcttcc agggtggtca caatcttcgg
1316401 tcttgtcggt tggaaggtct catgaaatat tggatttggc gacccggttt caaattgacc
1316461 acttggttgt gtgttaaatt gcggagcttg acgtcgccgg tgtcacggct gtagttggtg
1316521 acggctgtga cacttgtggg tgctttgact gtcagtagtg gaggaattac actgtttatt
1316581 tagtacgggg ggggcagcag ctcccaacgt tgccctgcat tcgttccaaa ggttgaaccg
1316641 gacgaagcct atgtgtgcag cagacgagac gctgctaaag ctatgcctcg gttgctgctg
1316701 ccatcgctac acctccacgt tatagtcgcg gccaactctt ccgctgctaa ttacgaggag
1316761 atctccccag cttcgcggtg gtctttggtg gacggaagtc agctgacgtc acagctttgg
1316821 cccgctgcct gctcgtccaa tatgctgctg tgacatctgc agaaactcct gcaataatat
1316881 ttgagcaagc ccgatgctaa ttgattcaca gcgtggccct tctgcataaa tagcaccgga
1316941 gtcaacagct attaaatcca gctcgtctca tgtgcatgtg tgtccatatg ctcctctcct
1317001 ataggttatt gtccatcatg ggagccaacc cctggcatgt aaccgacttg ggaggcttgt
1317061 ttctgtcaga ttagcatgca atgtcacgtc cgccgacaat ggaactactg agtcatcatc
1317121 ttccactcgg ggttttccct tcaacaacag tgtgaactgt aaatcattca cgagaggaac

1317181 tttattcagg ttatattgac ggcattcacg acggatgtgg cgcccgcggc gccgcgtggg
1317241 tctccctggc attcgcgccg ctatttgtgc tctttgtttt ccttaaactt ggaattctca
1317301 ctgttccctg gatgctgagc tgcactgagg gtctgattga atttctctgt ccaggccgcc
1317361 gacctgagca agcccatcga caagcgcatc tacaagggaa cgcagccgac ctgccacgac
1317421 ttcaaccacc tcacggccac cgcggagagc gtgtccctgc tggtgggctt ctcggcagga
1317481 caggtgcagc tcatcgatcc catcaagaag gaaacgagca agctcttcaa cgaggaggta
1317541 cgtcatgcga aacccagcgt tttgattggc cgatgtgtga cgctgcgctc acgttttcct
1317601 tctccctctt ttgtctctcg aacctcgacg ctcttcgccc gccagagact catagacaag
1317661 tccagagtga catgtgtgaa atgggtgccg ggctctgaaa gcctgtttct ggtctctcat
1317721 gccagtggga gtatgtacct gtacaacgtg gagcacacgt gcggcaccac ggcgccgcac
1317781 taccagctcc tgaagcaggg cgagaactac tcggtgcaca cctgtaagag taaatccacg
1317841 cggaaccctc tcctgaaatg gacggtgggc gagggggcgt tgaacgagtt cgccttctct
1317901 ccagacggga agttcctagc ttgtgtgagc caagacggct tcctgcgcgt cttcaacttt
1317961 gacgcggtgg agctgcacgg caccatgaag agctacttcg gaggcctgct ctgcgtgtgc
1318021 tggagccccg acggcaaata catcgtggcg ggcggggagg acgacctggt gaccgtgtgg
1318081 tcgttcctgg actgcagggt catcgcccgg ggtcacggcc acaagtcctg ggtcagcgtg
1318141 gtggcgttcg accattacac caccagcgtg gaagagagcg acccgatgga gttcagcggc
1318201 agcgacgagg acttccagga tcagatgatc cacttcgggc gggaccgagc gaacagcacg
1318261 cagtcccgtc tctccaagcg caactccacg gacagtcggc ccgttagcgt cacgtaccgc
1318321 ttcggctcag tgggccagga cactcagctg tgcctgtggg acctcaccga ggacattctc
1318381 ttcccccacc tccctctctc tcggacacga acgcacacta acgtgatgaa cgcgaccagc
1318441 ccccacgctg gcgccacgat aatcaccaac acctctagta acgctaccaa cggcaacaac
1318501 agcggcgcca ataccccgc cgtcaactcc ctctccaccg ctttgccccg ctccaacagc
1318561 ctgccccacg ctgccgccac caccgccacc gccaacaaca ccaacaaagc cggcggcggc
1318621 ggtggcatcg tggacagcgc catcgccacg ggcgtcagca agttcgccac gctgtcgctc
1318681 cacgaccgaa aggagcggca ccacgagaag gaccacaaac gcaaccacag catgggccac
1318741 atcagcagca agagcagcga caagctcaac ctgctgacca aaaccaaatc ggaccccgct

1318801 aagactctgg gcactctgct gtgcccgcgc atggaggacg tgcccctcct ggagcccctc
1318861 atctgtaaaa agatagcaca cgagagactg actgtactca tatttttaga ggactgttta
1318921 gtgactgctt gtcaggaggg atttatttgc acatgggcga ggccaggcaa agtggtaagt
1318981 tccttttaat gcaggacacg aaataacgtc atttctgtcg agcttttaga agtgggagca
1319041 aacagccgag agcataagat gacccggtcg attcacattc ctagccttta tagacacgcc
1319101 ggctctgcag gaaggcttaa attcgcagcg ttgtcgctgc ctggttgggg agacgcgcct
1319161 gtataaagct gccaccggag gaggagaagc taatccctac gtctcggcgt ttaggagaac
1319221 gccaaaagag gccccgctct ccgtcgtgtg tgtccatctc gcactgttgt gtctttctcc
1319281 cctttcctgt gtgtcccaag agatttctcc cggaggctcg cctgattcaa ctccaaattc
1319341 tgttaaatgg gagcagaggg tggttgaagg ttagcagagg tgcaataacc atgttggcgt
1319401 ttcagctcca aagtgccttg atcgagcaaa gctccggccg agggcgtcgg tccaccccgc
1319461 gggctgcggg acggagaaga ttagccgcta gaggctgatc gtttaagagc tgttaattgc
1319521 ttgatttctt ttcttctttt tttcttcttt tttttttctt ttgcaacagc atatgaactt
1319581 tcccaacgca aatctccttg ttttcctttt tccagggttt actatcatcc caaaaccaag
1319641 ccagctctcc cagtgggaca gtagtatagc cacaatattt ctgctgctaa agaaccctgc
1319701 aacccagagt ccgatgctgc tctccaccct gcaagcattg caagttctcc tttttacttt
1319761 gacaccccc cccccatcc cctccctccc tctccttcga ttttgttgac ggcttctttc
1319821 cttttggccg ctcacacact cgtcctagca gagcgcggag gcaagccaag gagcgatctg
1319881 ggctcccctc acacccgccg ccgtggtggg gttttaacac tttttttttt tttaggtttt
1319941 aaaagcctcc ccccccaacc caatagcaaa ccaaacaata gtggcttgag atttaaagga
1320001 cggttccttt aattttcata atccagctgt tccccccccc cctttaacct tgtggtgacc
1320061 tagtcttggc tcagggttac tgatgactct tggatgaacc atctcccccc caccccccca
1320121 ccttctgact tccctccagt agaatctccc cagtatcact ttgcactgat tttgttcttt
1320181 agttctttag tttgtttgtt ttttataatt actctaatgg aacaaaaaca aaaaaagagg
1320241 gttgaggagc gccttgttgc acatggcttt aacatcctca gtctggaaat ctgaattcct
1320301 aaagctggac gttgcaatca tagtctttaa gagagagaga gatgtttgca cttaattagt
1320361 ttattagaag aaaaaggctt tacatgtttg ggtgtgctca gaactcacag atggctaata

1320421 cagtaggtga caaacgatta aacctttatc tattgagcat ttattttaat attatttcag
1320481 attcaaatct gctctgtatc atactgtgta tattgtaatt aattgatttg atggggctta
1320541 agcaaacgac cattactcca gtggaagttt tgtcattgat agttatattt ctaaagccta
1320601 aagtatacat attaataata ttaaaataat cttgaataca ctgtttgctg ccgttttctt
1320661 gtcccactca catgtagtaa acagaccgct cgatgccttt tttcatttca attagcttta
1320721 atcgttggtt ataacatcag ggtagtgtat tattacacag gctacatggt gtgtttatta
1320781 ccaggagaac tcgtggatgc attctttgtc attttcttaa tgtctgcgtc attaatcgct
1320841 ctttttaccg actctttgtg ccattattca cgagctcggc tcaaacacaa cttcagtgta
1320901 aatgaagatc tgatattcta gacgttttac atattaaaaa aacacattag tttgcgtgta
1320961 agtgctgttt tgttgctaaa tcactacaaa tcaatgttaa aaatgtgctt ccccatcacc
1321021 tgtgtgtgtt ttagacgagc gtgaagctgt gcatgtgagt gtttctgcag gtgaaaccct
1321081 gttttccagt gcgtcgctcc gcagcggatc ggcgacgtgt tggacttcct gtttgttctg
1321141 cagcgttctc gtcatttaac catctttgtt tcatcactca ttaaacgtca gcctgcaact
1321201 ggtgcagaca gtgaaaacgt gttttaccag ggttgaacat aaaatcgggt tgatgtcatg
1321261 gcggcggcgg ctggctgacg gccttcagac ccgtccagac attgtccttc cagcccgtct
1321321 ctctggtctc gggtcagagg tgagacaaaa gcgtcctcgt ctctcagaag cctccgtgtt
1321381 tcctctccag cggcggaatg tagaagctcc tgaaagctgc gtgacgagac tcctcctgag
1321441 gctgaaagaa gccaaaacgg ggatcagatg caggcgccgc cgcgtccaac ccccgtgtct
1321501 ggcgcttacc ttggccagcc gcgattggca cttcttggac gcgatggccg gcagcgtccc
1321561 ccggtgggtg aacggaggca cggtggaaat ggcgggggcg atctgcggcc ccggtaccac
1321621 caggttgagc ttgggcagct cggcgggata gtgggggatg ttcctcctgg ccagcggcgc
1321681 cggggcgtgc ttcgtgttgt gctacaggaa gacaacgcaa tgggtcaccg tcgccgacgt
1321741 ggcggtcggc tgtgtggaca tcagctcacg ttcagcaaag gcgtctgtct ggcgttgcgg
1321801 cccctcagct tgccgaccct ggccggccga caggcgcggt ttaggcagtt gtgcgagccg
1321861 ccgccctcca ggccgtgcag agtcccgggg aggcggtgga aggtctccgc tctcttttca
1321921 gccatcctga aagagaaagc gttggcgctc atccgttcag aaggcggcgt gtggctctgt
1321981 gtccagtggc gcacctgatt tccctgaagt aggtgtgccg cagggccgtg tccgcggtga

1322041 tgcgctcgtc cgggtcgtag gccagcatct ggtagagcag cgagagggct ggagccgggc
1322101 atttggggat caggcgagag atcccggtgc cttttttagg ggggaagttg aagtgcatgg
1322161 cccgggacct gccagagggt cagagcatga agttgtttta cctgcacgag agaaccggtg
1322221 ctcaccaaag gagaagcgcc tactgcttga acttctgcag gacgctttga tccggcgtcc
1322281 ctaggacgtc gtggatcttg gaggcctggt ccacctcgtt ggctccggga aacagagggt
1322341 tcaaactgaa aaaaggggtc tcatttagca gtctgaccgg gccaccgggt caacgtacgc
1322401 gcaggaacat tcatttagtc ccacgtctgc gcagacgtca cctcatgatc tcgaagaaga
1322461 cgcagccggc gctccacatg tccatcttaa agctgtagta gccgtcggtg aggaggcact
1322521 ccggggccct gtaccagcgg gtcgagatgt actccgtgtg cggcggcttg gagtagatgc
1322581 tccggcaaga accgaagtcg ccgagcttca ggcaattttg ctgcaaagat cgatgcgacg
1322641 tgagcagccg tgatgggaac atctcccccg cacagcaaat gatgggatgg cgctcacctt
1322701 gatcaggatg ttttcaggct tcacgtctct gtgaaagatc ccacagctgg aagacaagca
1322761 catacgtcag ccgctccaaa tatcacctcc aacctgcctt ccctgctcac ctgtgcatgt
1322821 gctccagtga cttgcaaagc tggtacatgt agttcttcac tgtgtggtca ggcaggggcg
1322881 ttcttcgtcc tgcaacccaa aattcatcca ggttttactt tttcttagtt gaatccacac
1322941 tgagttggac tgtcgtggtg tcgcaccttg gattagctcg tagatgttca tctccatcag
1323001 ctcgcagatc agagacacgg ttccagactc tttgtcactg aaacatcacc acacctcatg
1323061 aagctgaaca caagacaggc ccgggagctt cagcaacctg cggtactcac agaatgagct
1323121 cgtgaagctg gatgatattt gcatgtgggc tcagcctctt catggcctgg acctcccgca
1323181 ggttgttggc ctgctccagg ctgcaacaca gcacaattgc acatgtcatt ttctagaggt
1323241 gtagaaaaat gatgggtatc tctggactga aatggcagca caaggtgcaa acggctgcac
1323301 aagagcatta aaagccattg ttacaaggtc aaaggtcatg caacctgaca acagggtcag
1323361 ttgggatgta gagatccacg caaagcagag aaatgggggc aacgctgacg tgctgtacct
1323421 gtttatggtc tgtttcatag ttttgcacgc gtagaacttt ccatctttca ggctttgtgc
1323481 ttttaaaacc tctgaaaatg tcccctctcc aattttcttg atgactttgt agcctgcaca
1323541 aagagaaaag gggttttatc acaagttgcc cttagtcatt gtgtaatttg tgttgtggcc
1323601 atttggctca taaacctttg ggaaatgtcg aaaaataaag ctgcatggaa acaccaaacg

1323661 atcatggtaa ccacgttaaa ccgtggattt taactaaata caccagagta ctgatgcgaa
1323721 tcccccсttt taaaaaaaat aaattctcca aatgttgtgt ttcaggagtg acagagctga
1323781 ctaaacaggt caacagctgc aacaacttcc cacctgcggt caaattcatt cgatgaaaaa
1323841 ctcacagtgc atttctctgc atattaactc gctcaattac atcccataat acgaatatcc
1323901 ggggagaaca tcccgccgcg aagcttccaa accttcgtta tggggcagga aaggcgttag
1323961 cttagcaccg ctagttagcg agccctgctg ctgaccgtcg cctaggtaac ggagcacgcc
1324021 ttcgccctcc gctctgaccc ggggaggaag agactcctgc gcggctccgc taatcacgct
1324081 gccgctttgg cattaaaaga tgcggcgcag tgcattgcga ggggggtggg ggggcagtca
1324141 agcagaattg ttcttctagg cccccatatt taacccgata ttatctttgc gatcgcccca
1324201 gtttcccttc ttaccttttt tccagagttg gacggttacc accaattccg atattttctt
1324261 accgggaacc gggttgttac tctgcgcgtc cactgtgttt cctctattgt tacagtcgct
1324321 gttgggtggt gttttgcacg catttcgaag gcatgccctg tttttcacgc caatgagcca
1324381 gttggagtag tgcatcatga gtgcacctta cactccgtgg agccgaccat gagcttaata
1324441 agtgcacaga agctgctcga ctcagagcac aaggttaccg agcaccagtg cggctctcac
1324501 acacgccaga tcaaccgcac ttcgcccgtg aacagaagca gaggccagat tctccctgca
1324561 gatggcgctg ttgcagaggc ctttctcagc gtgactgaaa atgtttaaat tattcctact
1324621 tagttgaatt aaagtatcac attctttctg gaaacagcat agcatagcat agcatagcat
1324681 agcatagtat agtatagtat agtggctcaa gcagagttca tatgcatttg tatatgcatg
1324741 attccgtcac atcacaatat ttgtaagatt taagatttta aaagttggtt atatttcaac
1324801 tatgagtaaa tagtcacata ttctgattga gttacactca acatcagatc tttgctgctg
1324861 tttttcagtt attatagtat atggcatgta aatgcggaca ttttaagcca aaatgtattc
1324921 aaaggtatcc tcaatgccct ataatacact tatgtactgt gcataacatt gcatataaaa
1324981 tgaaatacca atatgtaaat aatttcacct ttactactgt attgactgat ttttcgattg
1325041 acaaattcga aatgactttt taccctcgtc ggcacccata acaataaacg ctactgcgca
1325101 tgtgtggtgt catgcaccta ttccttaaat ttcttagcca ctgatgtaag agaaatgtga
1325161 atatttttgt tcattggcca ctttccttgt ttgaatcgca gcgaatctct tgctaagatg
1325221 aagacgagat tcaccaccgt cgacattaag gcagttattg ccgaaatcaa tagcaagtaa

1325281 gtgcacgttg actttagcgt tctcgctaag ctaaccaatc gttgatcatg tgcaaatgtc
1325341 aacaaatgct gtcctgtcgt ttaaatacct ttgtttcacc atctcttcca gctacatggg
1325401 catgagggta aataacgtct atgatatcga taccaagacg tatctcatcc ggctccaaaa
1325461 gtaacttttc tttaacatgt gacattatga cataacagta aagaatccta caagcattgt
1325521 tttacataca aaggtcatac agttatttta atgagcaatt ctttgttata ttatagaccc
1325581 gacagcaaag ctatcctcct gatcgagtct gggacacgaa tccactccac ggactttgaa
1325641 tggcccaaaa acatgatgcc ctctggtttc gccatgaaag taagaccaca tgatttccct
1325701 tttctttgac agtattaatc tttgatttag aatcacaata aagcgttaag gtatcataca
1325761 agagcaagat gtgacaaaac agaactttgc agtgtcgaaa gcacctgaaa acaaggcggc
1325821 tgacccaggt taagcagctg gggaacgaca ggatcgtcga catccagttc ggttcagatg
1325881 aagctgccta ccacttgatt gttgaactgt atgaccgggt gagcaacaga acgtgtcgac
1325941 cgctaatttc taaagttaaa agtttgcgtc taacctgtcc atccacgtct gtcgtcaggg
1326001 taacgtcatt ctggcagacc acgagtacac catcctgaat ctgctgcggt tccgcacagc
1326061 agaggtggat gatgtcaaga ttgcagtgag ggagcggtac cctgtggaga gcgctcgacc
1326121 cccggagcct ctcatcagtt tacagcggta gtgatggacg tccatctgac ttcagtccaa
1326181 ccactttata cacctcatat aagtgaaatc tcgccgtttt gttctgaatg tagactgact
1326241 gaacttctca gcgcagcaca acaaggggat caaataaaaa gggtgcttaa ccctcacctt
1326301 tgtaagtaga tctttttctt tcctcatgtg ttaaatgttc tttgagatat tactttccga
1326361 ctttaataaa cacacttaac tatattttat acctcagcct acggagccac tctaattgaa
1326421 cacagtttga tagaagtggg tctgccgggc tctgccaaag ttgacagcca agccagtgtt
1326481 gcacaaggta catgacagat gggactaatt acacttacag aacactgaaa gtaggttcta
1326541 ttctttcact ccaaaatgta atacttaaat aaatgctttt tagtggcctc taaaatcctg
1326601 gaagccctga cagttgcaga agcctatatg gcaaaaacag agaacttcac tggcaaggta
1326661 agatctctat agatgtgtgt gttcaaatct gatttagcat tttatgatgt taaaaagtaa
1326721 gctgagcaca gctggacatt tcataccaaa cactttgttt aaatcaaggg ttacatcatt
1326781 caaaagagtg agaagaaacc aagtgtaaca cctggtaaac cctctgaaga actccttacg
1326841 tatgtctcct catgttttca ttgccctcgc caagaaatct gctgtttatt atatttgatt

1326901 tgtgctttct gctttcagat atgatgaatt ccacccattt ctctttgccc agcattcaaa
1326961 gagcccctat ttggagtttg atagttttga taaggtacat gtgtgcatga ccccttatgt
1327021 ggggatgagt attgaatctg cgatttcttt tcctgaaatc tgatgtgatc ttccctctca
1327081 ggctgtggat gagttcttct ctaagatgga gagccagaag attgacatga aggccttgca
1327141 gctggagaag catgctatga agaagctgga aaatgttaag aaggaccacg agcagagact
1327201 ggaggcgttg caccaagcac aggtccttag agtctccaca cccctgactt atccatataa
1327261 ccagattatg ttcacacttt ttgattacac gtgcccaaaa acccctctgt gtttcaggag
1327321 attgatagaa taaagggtga actgatagag atgaacctgg ctatagtgga gagggcgctg
1327381 caggtggtgt gcagtgcact ggccaaccag gtggactgga ctgagatcgg gattttagtc
1327441 aaagaagccc aagctgctgg ggaccccgtg gcatgtgcca tcaaggagct caagctgcag
1327501 gccaaccaca tcacgctgct tttaaagtga gagcgccccc cggccctact cagaaatcca
1327561 aacgctcagt ttttgcctta cagtgagctc gttgtgtgta ggaatccata tgtatccgag
1327621 gacgacgagc aggaagatga tgtggtcgag gagacggggc ggaaaaacaa aaacaagaag
1327681 agcaagaagt tccagaagaa taagcccatg ctagttgatg ttgaccttag cttgtctgct
1327741 tatgccaacg ccaaaaagta ggtttacatt tggtagagtt gacgcagaag aagcatcaca
1327801 gtcacctgat ttgtacgttt ctaggtatta tgacaacaaa cgctcggcta aaaggaaaga
1327861 atttaaaacc attgaagctg cagataaggt atgaatgatc ttgatggcca atcgggatga
1327921 aaatgtttgt gctaatctgc attttaagat gatgttctgt ttctatatca tccaaggcca
1327981 tgaagtcagc agagaaaaaa acccagaaaa cactgaaaga ggtccagacg gtgaccacca
1328041 tccagaaagc ccggaaagtc tattggtaag cctccaaaga aagcacctct ccgcccagaa
1328101 gtgttcgttc tcgccaccgt ttcttccccg gttgacaggt tcgagaagtt cttgtggttc
1328161 atcagcgcag aaaactacct ggtcatcgct ggaagggatc agcagcagaa cgagatgatt
1328221 gtcaaacgtt acctccgcgc gggtaagaca caaacgcagg cgcctcaaat gacctctgac
1328281 ctgttgtccc tttattcatc gttcctcact tgctcagggg atatctacgt gcacgctgat
1328341 cttcacggag caactagctg cgtcatcaaa aacccttcag gtaaatcctt cgctccgtca
1328401 tatccctgca ttttggaaac ttgtttccac gtatttttgc ttcctgttcc tcctgcaggt
1328461 gaccccgtcc cccctcgtac gctgacagaa gccggcacta tggctgtttg ttacagtgca

1328521 gcctgggagg ccaagatcgt caccagcgct tggtgggtcc atcatcatca ggtcggtttt
1328581 ccttcctcac agctaaattt gctagcatga actcctgata tagaaacaat ctgcttacag
1328641 gtttcaaaga cggctcccac tggagagtac ctgaccacag gaagtttcat gatcagaggt
1328701 gtgttgtcaa catcttagat tctgtaaagc cacaggtgcc aagtttgtga cctttgacct
1328761 gcctctcctc caggaaagaa aaactatctg ccaccttctt acttgatcat gggctttggg
1328821 ttcctcttta aggtaacggc tcgtgcgtgt gttaattctt ggctgccttc ggtcttcaga
1328881 tctcatgttg gtctctatgc aggtcgacga acacagcgtg tttcgccaca gaggcgagag
1328941 gaaggtaaag acggtggagg aagacgccga ggaggccgct tccaagaccg ctgagctgct
1329001 gaacgaggag ggggaggagc tgatgggtaa caaacacaga tggtttgacg tgtgccggct
1329061 gtgtgtgacg ttcaggcgtt gttgaacgac tctgccgccc gccgcaggtg acgacagcag
1329121 cgatggggat gagggtgagg acgagcacga tgacagcgag gtcaaagagg tcacgcccgg
1329181 tccggaagac gacgaagacg acacgaggga tgaagagagt gaagagatca gcttcccaga
1329241 cacgagcatt tccctctccc acttgcagcc caacaggtaa aagttcagtt catcgtcgaa
1329301 aacaaaaggt gcaaaactaa atcgataaaa tcctccttat tttacagcag tgctcagaag
1329361 cccggattca aacaggaagt aacacttcag gtaaagattc tgtatcacat cagttcaaat
1329421 catacataga aaaggtctta ttgtcttttt ctattaaggt tgaaagagat tcccaagtga
1329481 ggaaacacat gacggccaag cagagaaggt ctgagcatta atataacaaa ctaaaagcag
1329541 gtgaaggaaa ctatttaatc cttggatgtt tttgttttca attagggaag agaagaagaa
1329601 gcagaaacag gaagatactg aggagaagac agagattcca gcaggaggct caacaaacaa
1329661 tcagggctcc aaaagtggag gagactcttc ccagcagcca ctgaagagag gacagaaggt
1329721 acgaggaggt catctcatct gatggccagg actcttcaca cttgtctttc tcatctctgc
1329781 agaacaagtt gaagaaaatc aaagagaagt acaaagacca ggatgaggag gacagagagc
1329841 tgatgatgca gctgctggcg gtgaggatca gaacacacga ccccacgccg tcgctctggc
1329901 tctgcggggg aataacagat gaaccgcctt tcttctttag tctgctggtc ccaccaagga
1329961 ggagaaggag aaggggaaga aaggaaagaa ggggaagggg aaagaagagc ccgttagaaa
1330021 gcctccccct cagaaaccag cccagaaacc tcatcacctg gaggcgaaga aaccagagga
1330081 ggcggtagga aaggaagagg gggaaaaggg aggagaggaa agaggagctg ctgaacagga

1330141 ggagaaggta caacacgtct ggcggggcag cttctctcgc cttctccaac
cttcacatcc
1330201 actcgtgttt ttcaggagga cgaggcagac caggacaacc ccggcgcaga
ggtgagggtg
1330261 cttccaacaa cacaccatcg tttctggaaa gcatcgcctt taacccaccc
gttccgcttg
1330321 cctcgctgca ggaaaccgag gatctgctca cctccctgac cggccagcct
cattctgaag
1330381 acgtgctcct gttcgccgtc cccgtctgcg ctccgtacac agccctctcc
aactacaagt
1330441 acgtgcactt cacacgtgac gcacgtgggt tggggcaccg ctaacgagct
ttatttttgc
1330501 tgcaggcaca aggtgaaggt gacgccgggc tctcagaaga aaggaaaagg
taaaggtctg
1330561 acgtccttgc agctgctgag gcctttttct ttttttacaa ctattgttct
gtctctgcag
1330621 ccgcgcgcgt cgcagttttc agcttcatga aagcaaaaga tacgaccgcc
agagagagag
1330681 atctgtaccg cagcgtcaag gtggggagtc tgattcagta tggttgaatc
taccctgcag
1330741 aaatagttac acgcctcttt tctgtcgtca ttcaggatgc cgatctgtcc
aggaacctgc
1330801 ctggaaaagt caaactgtcg gctccaaacc tgcaggctgc caagaggaaa
tgagctgatt
1330861 tttttttttt tttttaaagg atgctgttcg acactttatt tgttttaaaa
taaatacaaa
1330921 tctgaacacg cagcgctatg atccatttat ctgcacactg aacacttttc
tacaatcaca
1330981 accaaaacac ccagggatca ttgtgcaaac aggcaaatac aaacacgccc
tcgactcctc
1331041 aaaacacgta tcgtatgtac agaaaatttt acaggagccc cttgcaaaca
aactgaagac
1331101 tttaaaagcc attttacccc aaaaaaccca aacataaatt aaaaagatat
aaataacaaa
1331161 tcaaacagat gacagccacg cgagacacca gattgagctg tgcgacctta
agtttgataa
1331221 acaaatgtgg gagaatccgg gcagtgttgc tgaagcctga acatttacat
ttatgttact
1331281 gtgtataact ttgtcttagc tggacggaca caacttgaac gcaccgggac
agaaacatgg
1331341 ccctctgtgg tgagggtggc caccaaactg agggaacccc ttcgtgctga
gaactgaaac
1331401 ctataacaaa cccgaacgac aggactgaga gtgaacacgt ataactaatt
taacaggatt
1331461 ttatacttgt ctccgtcaca caggtggtgt tctcccgtca tcctccggac
tagattgtca
1331521 ggttctcctc agactaggag cccagcgtgt tgacgtgcgt catcctcagc
ttgaggctgt
1331581 ggaagaggtg tttaggcaaa cagtcccagt actgagacag acgctctctg
tgctgaagca
1331641 cggcctccat gcagaaccta aacacgggga ggaggggtaa aaatgagctc
agctatcctt
1331701 acagagagaa gtgacaacgt tgccattcat ctgattccat tgatcacccc
ccccccgcac

1331761 actcaccgga ctagtgactt gggctgaaca ttgaagatca ataactcatt gctgtgtgcc
1331821 tgtcggcgga agcaaaagtt cacaattgat tcaaaacttt tcaccgatgc ataaagtctg
1331881 tgctccactg aggtagggag gtctcttaca gctctctgct gagctaacag gttgttagca
1331941 caccctccaa acacgaacac ttctccgtca ggtcctgagc atgctgtgtg ccacaacctg
1332001 aaacacacac acacatcatg ttgtaagtac acgcctgctg aaagtgaacc taaagcgcac
1332061 agccatgttg acactttaat gggacttgcc ttggtctccc tgtgtgactg tgcttaaagg
1332121 gcttccattc gtttttgcta acgtagtaca gccaagcgtc acctggcaaa agtcagaaca
1332181 gcgatcactc gttccagcga tcaccccatc ggaggaatgc tttcgctgca acagaacacc
1332241 gtgcgagtct tactcagcgt ctctcgctct gtggtgaaac ctccaaacag gaagatgtgg
1332301 tctgctgata cgggagtgaa ggagtgccac gacctgccca ccgggccgtg ctgggggacg
1332361 ctcctgccgc acagacggga cggttacacg ccgatcacgg atcaattcac ccctgatatt
1332421 caggtatact tacatttcgt gccacaccca cgtgtccagg tcaatgtagt acaggtcatt
1332481 taatcggtaa ttctacaaaa atcaacacaa ctaagctgga atatggttgc atttgaaaaa
1332541 aaaatacgaa gaatataaca ggaacgaaca agaacgaggc tgaggcacca cttgtcctga
1332601 ccttgtatcg tcctccaaac acataacccc tgttgccgac cgtggcgcag gcatgagcgg
1332661 ctctgggtaa agggcggctt ccctgcagca gaccacacaa ccagagagag acacatcaaa
1332721 tatgttttgt gcttgttttc ctgtgactaa tagtgcgaca gtagaaaaat ggccattatc
1332781 tggttaaagg gcggttcgcc acagaactcc aactcaaaac aagcacgtga gcgtggcgag
1332841 gtactacagc cttgtgctta ctgtggtgat gggctggctc catgttgatg tctccaaatc
1332901 caggatatga atgtggttgt tccagcctcg tccagggctg tcccactgca ggagtggtta
1332961 cagttacttt tatatgaatc gcttgtagta aaacattttt aaaaaaaaac aaccaattat
1333021 aatcattaga ttatggaaaa ataaatattc acacacccca aatgaggatg actcgtcaaa
1333081 ttcaaatgac ccttgatgag gtccttgagg agtgtagcca tagcctccaa agaatatgag
1333141 cctgtggaaa gagcagttca ccaatgttac aagttagtaa tcaaactggc tggtttataa
1333201 atgagacatt tcatcaaagt aaaactgggg agagagagaa caaagatcaa agtttatcta
1333261 ctatcaacag agatccctcc ctggctcata aatgtgtctc attacttttc caaatcagac
1333321 atgtaaacag ctgagaaatg aaaatacact aaatctgtgt acaaggattt caagactcac

1333381 ctgttcctgt gaacccagca ccctagcttg tccttactag agggtgggag acctttaga
1333441 tccctcattt cttcccaaat aagagtggag gctctcagag gaaggcggta gatctgcaaa
1333501 acgtagcaca aatcccaaga agacaattaa aggtgatcca accccttaac aaaccttccc
1333561 actttaaacg ctgagaactg tacccgattg gtgttccccc tggcgtgatg acctccgaag
1333621 agatacaaga cgccatcaac acacacccca cagctgccgg acatggacgt gtgcaggttg
1333681 cctccagcta tatgctttgt cctgtcagca gaaaattagc ttttaaaatt gaatttgcgc
1333741 ccaactgacg taaatgtcaa atagctaaag gaaccagtaa aggtcgcagc tattaccata
1333801 ctcccgactc gatgttgtag gcccagatct cagttctcgg caggtacaag tcaaagaatc
1333861 tgtggttctg agcgttcttt tacagaaaag gaaaagaaat tggtttcata tgcagaccga
1333921 gggtacattt ggtgcatgca cacgtcatgg cctgcttacc ttatatccgc cccacacata
1333981 cattgtgttt cggtcaatca ccgctacgtg accactgcgc tcagccggag tgtccaactc
1334041 aaatgactcg gaggattcgg tgtccagggg ctgctcatcc tccacctcct cctcctcctc
1334101 ctcttcatct tcctcacctt cgtgcatcag ctcaaacagt ctgtcaactt cgtcatccga
1334161 gtcattgtca tcctcttcgg gcggcagacg gtccatctct tccccgtcct ggtctattct
1334221 ttcagccatg tccccttctt tatgttgtct tctcaagtca taaggtgcag cccacaaaca
1334281 gattattaca gtcagtaatt ttgagccggc agcttcaaag gattggtcac aaatctgaaa
1334341 gacagtgaat aacacatcgg cactttaatg cttcaatgtt tttaaacaca ccgcagattg
1334401 tatgtttaaa tatgactact actgaaataa tattaaattg aaaacaactt ggggaaaaca
1334461 ggactaagta actggcttta ggaagcggaa ctacaaactg ggtcaaccaa aaaggcgtta
1334521 gtagttagca cgattaacgc tgacctacaa acgatactgg cttaaccagc tggtttctac
1334581 actgaacaat gatttggtct gaagatcgca tgcgattcat taaaaatgca caaactaagt
1334641 ccactcacca ccaatctaac gtcttgctgt cagccattgc tagcttagct aatgctaatt
1334701 tcttattctg gaaatgccca caaataaagg ctctagtgtt acactagcac cgtagctccg
1334761 gcgcttgcta ttgcttaatg atacgccaca tatcagctta ttaaaaaggg taagttgcgt
1334821 gtcattagct tactttgtgc gatgtttccc ctgcaaatta cactcatatc cagtattgtt
1334881 gacgttgtgg agtgacgtca caaagtaccg aagggcgaaa gggagttgtg ttttttttaa
1334941 acccaccaac acccaaacaa aattttgttc taatgcgcca caatttggcg ttttagttat

1335001 aaaacaggcg tgacagtcta aaaacggggt aaaactttat ttaacttgga
tcaccataag
1335061 gataatctac actgcatttt aaccgaacac aaggcataaa gttaaaattg
tcaacgacat
1335121 acatatgaac aaaaattgcg aaacattttt tcacaataac gcaacacaat
aataagcact
1335181 gcagttgagt tttcatgatc aaataacagt aaaaacgata aagtcttctg
tttcttcatg
1335241 cagttttttt cccttcagag tccctgtaat ttgctgagaa caagaggata
cacacacatt
1335301 aataatggta taaaactaca atcgtctatg ctgaacaagg ccgagaagag
caaaacctac
1335361 ctgtcttctg cagttctgct ggctggatag tataccttaa aggtaaagcc
acttcttgga
1335421 aatgagccct gcagatagta aatataattt aatcaaatgt tgggtcagct
ggaaagacca
1335481 aacggaaaag gttttctttc aggggaatct ctaactaccc tcacttgtgt
ttaggccttt
1335541 tcttaccggg ttaatgcaga gacagtctgt gctcgtgatg ctgaaagggt
cgtatttatc
1335601 tgcaaagaca atgacgtctg gaacagggta gacccgtagg gagtagtcat
acgcccagta
1335661 tacaggactg acatagaggg gcagaggagt caaatggccc tgggacagga
ttgtcttgac
1335721 aaactggagc gaatagaaaa aaatgcacat ttttcacaca acatgagcaa
aattgtccta
1335781 ttaggtgaca aactgaggct gtgatgtaaa aataattgct ttaatgtgag
caattttgca
1335841 cacgaaagag aagtatgaag ggttgctctg aggctgaaac gaacatggtt
tggaatgtca
1335901 agattgtcgt ggggcagtcg gacgcagttc ctgcacattt tgttgaccag
gtcttccctg
1335961 aaaatgagga tctcctggct gcagtactgg accctgaaaa aagacaaagc
gctttggtgg
1336021 tttttcaaag gtctggctac cagtgaaagc ctggtgattt acctgcacgg
gttcgtggtg
1336081 aacacagaaa agggcaccct ctgtctgaac tcctctgtga tgtgattggc
cagtggaggc
1336141 ctggggagga cagattcatc tacaactgtc ccaaacaagc gaatccggtg
tcgcggagca
1336201 gacttacctt ggcagaatgg cgcccggacc ggggtcttct gggccgggaa
caaagagaaa
1336261 gcgactgctg tagatttaag gaaaacgtgt tgaaaataaa gcagcgctaa
atattccagc
1336321 agcttcattg ggactgacct gttgtggatg ttggggtggg cacatataac
atcagcaagg
1336381 gcttttaagg aatctatgaa aagaacaact gatttaaaag gctgaaaagc
cctttatgta
1336441 catgagcttg tgttagacag agctctaaaa agtccaacct ttgagtgact
ttatccgtgc
1336501 gtttccataa ggagcgctgg agaagttgcc aataaaaata aaacaggtgg
gaggcatcga
1336561 cgagtaacct ggaagaaaaa gcaaagtgaa actaaactcc ggccaagtgt
cggaagtttt

1336621 gagttgtgat tccctaaccg acccgagaac atgaggttaa gcttttccag cacttctacg
1336681 ttgtccagcc acacatcaga aagaatcaca aacatggcat cctcgttttc ctcctccagc
1336741 tgcttgagct tggccgaggc cttcacagaa gtcgaggacg gacccccgaa aaagttcatg
1336801 ctcccgtagt acgccctgga agaagagcgc agggtcacag ggtgctgcca tacaagaata
1336861 aggctaaaag aaaaatggag acacagcctt gactcactca ccttgtggct gaggatggtt
1336921 ctgttggtgg aaacccaaag ccattgacat ggaaaacaga gtcctcgtac caacctgcag
1336981 gacaaaggca aacccacact aacgtctgca gtagtgcttt atggtgtgtg ccttcttata
1337041 cttttattct ttccgcatgc aaggttgtag cagtagccgc tgactcaccc tctgcaagca
1337101 cgaagcagga ttctgtgtac aggccattgt gaaactggtg ccaacgctat taaggattca
1337161 aaatatgtca acaagaaaca gaatattgta tgtttttctc ctgaaggagc ctcgacaaac
1337221 atttgaagga tatcgctttg gacatgtcta gttggactgt tccactcagg tcctccaggt
1337281 aaaatttacc ctttaaaaaa aaacaaagaa atgtttcaga aactagaagg aagtatacac
1337341 gtgttatatg taccgctctg taaaaacaaa atctatcatt cttcacctct ttgagctgtg
1337401 tgatcatgcc cagcacaatc acctctccca tcttagctgt gctaccaagc agagcctcaa
1337461 tggtcttgag ctgaaataaa tgacatacat acaggatatg tcagagaaaa gggttgtttc
1337521 ccttctgcaa ctataaaaag acccgcgcat taataatgcg gtctttggat aaagccaaac
1337581 ctgaaatttg ttttggccct cgtcaacagc tgttcctatt gcaggtgggg tgaagagttc
1337641 atgtcgatgt attctctgga aaaaaaaaaa aagggtcagc attcaaacag cggtcatgtg
1337701 acatttacag atcttttgtt cttgaacaaa gctcactgac ctgctgtagg atggtgtaac
1337761 gctcccggaa aagctcagct ttgtctttgg cccaaccgca caggctggga gcaggatgtg
1337821 atgtcatact gaggctggaa gaacgagcca acgatgacac aatgatcaga agaaaatatg
1337881 tcaataaatt tgtcctggca ataaaagatg agaataacag cctttctact tacggtataa
1337941 atttcttccg ctcaacactg taaatgtatc tgggcacatc aaaggctcca atgatattaa
1338001 aaacattatc tctggaagag agtttaaggt ttattggcaa aattatcaca cattttcttt
1338061 ttagaacatc acccatttgc aaatgttcaa agtatcgttc tactcttttg tgcttaataa
1338121 taatacaatt aacacatctc ttcatcttaa ttacttattt ggggggggttg acaatattct
1338181 aaacagtgat taaacaagtt tggtgagtac actcacatat tttcatcaca agactggctg

1338241 caatcctgca cggcactttc tgctacggac agctcgatca tgtttgacga cactgttgga
1338301 aaaacacagt caggcctggt acctaaacac acggtgatga ataaacatgc taaataggct
1338361 gaaaataaaa tattggtcat gtacatacat ggctgctttt ccactgcatc cagaaccttt
1338421 tccataacat cctccaggtc agaggaattg acagactcca tcacttccac tagatatttg
1338481 ctggcttcac tataaaatac atatgcaagt tacaaacaag cctttttttg ttattctccg
1338541 tttaaataac tgttaaaaga gacatttcaa gcttttaaaa tactctattt cacaaatatt
1338601 agtactgcaa atattaacgt tccacaatat gaatgtctta ctaaattttt aaaggatttc
1338661 acaagttgat gaaagtcagt agcaaaagat gaaacttgtt aacgggcaat atttgagttg
1338721 tagacagtat aaaaatagtt tttatgacct aaaacagaca gagcttgtag agaatgtaac
1338781 atttaacata accaggtatt ttttattaac ttcattccaa caactggcaa caatgttacc
1338841 acagcacatt tcaaaaaagt ttaactatta ttattcacat acggtcgcag cataagtccc
1338901 cgcatcttga aagcagttga cactttgttc tttattttcc gggcagcgtc catgatcgct
1338961 cttgtctgac tccagacttg gcgctaaata tcaccttccg gttacgtttc tcgacttccg
1339021 gttcgggatc gaagggaagg aggcgctttt acttcgtcag gagcacaaaa cggaagatgt
1339081 attttatttt gaaaaacact cggctcggtc gagttcttgg ctgtgctgtc aaaacatgag
1339141 aaaaaaaata agcgacgttt gaaattgcaa agcaatacgt gagtatgagt aaagcttgtt
1339201 tttttaagcg tatgttcatg acatgtcaac gttgctgaca tcgcaattca acctgcctgt
1339261 tctgtttaaa acctgaatgt atttaccagg taaataccat ataactgcat gcaatcttcg
1339321 gtctgttcag tcgaacaaca acatgaaata ataatcctta gtctccattt ttcattggta
1339381 agaatgcgat ttgggtgaca ggtgtgactt ttaagtttaa agactcgtct ccgttttcta
1339441 tgaatagcag ccagtcatgg attcaggctt ccagaggcac atctcggagc tggtggcccg
1339501 ggagaggagc gggcacacgg ctgtggtgga gggcagccta ctgtatgtgt gggggggtta
1339561 catggtgaga aacaccaaca atattgaggc tgatctctcc ccgatgttca aattctgcag
1339621 caaatactac agcacttact aatgtagcgt gaaaggtctc tttacgcaaa tcatattgtg
1339681 atgaacaacg taatcccatc aaattctgta attcagtaca atctagacat actgtgtttc
1339741 aaaacactgt cataattatt aatacaatgg catataaaat tactttctgt tattacaagt
1339801 tgcattggtt ttgtccacag tcggtcgctg atgacgaggt ttttctaccc aacaacgaaa

1339861 tctgggtgta cgacttagaa ggtggtgtgt ggtaagtata gtaaatagat ccttttctcg
1339921 ttgctttgtg aaacagagac agtaaattaa catcatcctg accatcattg gacctgggca
1339981 aatgtttcat tcacttaccc acattttttt aaaatgtatt tactttgaag ggaggttttt
1340041 catatgacag gggacgtacc gccgtccatg tctggggcct gcggctgctc gttggatgga
1340101 cacatgtaca tatttggagg ttgtgacgac aatggccaga ctaatatggt gagactgata
1340161 actgatgctc tgtttaatca atttaaaagg ttgctgtaca gttgtgggca ctctgactca
1340221 ctattgtatt gataacatgg cgctgtagca acagacttgc cttttcttgg tgaggatgat
1340281 aaatatgcac catcgccacc atctggctct caatagtatc tgataaatgt cccatcatca
1340341 caggaaaact aatataaaca atgaatgaac acagtcaaaa tttaggctga atgagggtgg
1340401 catgaatgag tgatcagtgt ttgacttcaa acagatctat tgtgtcaacc tgacagacgg
1340461 caaatacacg tggaggagga tcgggcaggg gctcggctcc gcaccgtcac ctcgagacaa
1340521 actctcctgc tgggtttaca agagaaggtg gttaagttta gcaacgcttt cagattttct
1340581 tgcacaccag catgcctgat cctcatacgc catcccacag cctcgtctac ttcggaggat
1340641 atggtcacaa actgctgaca gacgtggaca gcaggaacag aagcttcatt gtggatgaag
1340701 catcatgggt aatctgtccc tggtgctcca gcattttgct gtaattccct accttttagt
1340761 tatttgactc tctgtcagca ggttgaagat gtcttctggg gttggaacaa tgaggttcat
1340821 gtatttgacc caatgaattc cacttggact gaaccaaaaa caaacgtaag ccagtaaaaa
1340881 ttctataaaa taggaacggt cacttaaaag attctttcct gaaatcatgg gaggatgtga
1340941 cagtggcaga ttgtgtgtgc agggtcgagc tcccgccccc agggctgccc acgccagcgc
1341001 caccctgggc cacagaggtt acatttgtgg aggaagagtc atggtgagcg tcagttatgg
1341061 acttctaagc atatgaaagc agaattaaat gacaagaagt tgaaactgac tctttttaa
1341121 cgcaggaaac caggacgagc gacgttcact gcctggacct ggatgcgtgg acatggacag
1341181 aaatgtacat catcttaaac acccagtaaa tgttaaacag gtttcacttt gactcagttg
1341241 taaatatctt ttctagaacc ccagcatccc ccattccagt gggtagatcc tggcataccc
1341301 tcacagcagt atcagacacc tctctcttcc tgtttggagg tctcagtgta gactgcaagc
1341361 caatgagtaa gaaaacactg ccaccctttt atttgttatt gtttttaggt cctttttct
1341421 cattctgatt tgatttgaag gtgatgggtg gttgcttgat gttgagacaa agctatggag

1341481 agaagtggag catcctttta ataataagcc gaggtagaaa agtgggcgat ctgatggttt
1341541 acatcttaaa atactttaaa aagacatatt tcatctattt atatttagtt acatgttttc
1341601 cctctgtcaa aggttgtggc acactgcgtg tcagggcaac gacgctgatg tgattgtgtt
1341661 tgggggaagt tgtgactaca tccttctggt cgacactgta agttccttaa tttacattat
1341721 ctcttaaact atagctaaat ataacagatg aaatgatgaa ctattccctt gtggccttca
1341781 aagggtgtgc atgttgcata tttatgggga ctcggctgac tgtgttgctt tttcagggcc
1341841 actgcaacga cgcacttgtt ttccagacgc agccttatcc tctcttcagg taattaaaaa
1341901 agtttaaaga gcagcttctg ccttttttcaa cacacctcca gtacgccggc tgtatttaac
1341961 ttgttgcatt agaaatatct tcttcaacca gttgtgtctt atataaatcc ttgtttctca
1342021 ccacaggatt tgtgaggatt atattgccaa gaatgtgaag aactatgaag ccctcagaaa
1342081 tcagcttcca cgcctgcctt ccaaacttct gacagcggtg cagaaaagga tgtctttcta
1342141 caggccttca aagaagcagc gctagtcaga aaagtcttaa cagccatgac aaaacaatgc
1342201 attggccata tgtcacacgt tggggttttt tcttaatacc tggtatataa taatgtgtca
1342261 tcttgtggtt tattgtttaa cagattttat tttgtatgat gaaaataatg ttacaaaagt
1342321 gtttctgcga tgcctgagga ttaaatgaca caatgaacac atgcctgttg tttttggttt
1342381 catccaagga aattttcctc acaaatttct gccataaatt tctgcatatc taaaaataga
1342441 tgtgcttttt atttgctgta taaaacaata ttacaaaaca tccgtaaagc tgtgccaaaa
1342501 gcggtgcagg ctctcgcgtc cctgaagagt tccacatgcc atacggccat gcgaccactg
1342561 ggtggcggta tggtgttgaa ctcaccgtga gtcggactgg aaccagtgca gccacacatt
1342621 ccttttccac aagggcggag acgtgcagcg actgcggtca ggaaagagcc cttatataaa
1342681 cactcccgcc tctgccatct gaaaaactaa actctacacc cagctgctct aaagtgctgg
1342741 tgaaaaatgg caccctgagc tgtgctgtga gaaatgtgcc tcctaacaaa gccattacaa
1342801 ttctcttcta cttaaatatg gtggctccta tagcagaaac cctggttccc ttgtcattgt
1342861 gtgtgtgttt tttccaattc ctcctgcgac ccatgaggtg aggacaaatg aacaggacag
1342921 cgggaggtgc gctcagctga gtcaactgaa ctctctctcg atggtgtcca tcagctcttc
1342981 ttctgatcca tcatacagga ttatgggcat ggggaggggg gagctgtggc agccgccaaa
1343041 ggtcctgcag agagatgaga tgcatgtcaa caacatcacc gtgggagaca ttgtatggca

1343101 gcgtgactat ggtgtcagga gtggcaaagg tcaacgagtg ggatgatttc agcttgtttt
1343161 ccagccctag ccttcgtttt tccccgtgtc cccagcaaga tgacaacagt tttgcttcta
1343221 ttcagctctc cgtcagaatg atgagtcagt gttttgatcc cctcaaactg aatttgttct
1343281 gtctcttcga ctgcgcagaa aatcgactca ataatgcatc agatgtgtct acttacggtt
1343341 gtttgtgcgg ccgggcgaca tctttcagca aaacttctgt tgtcaaaaat ccccgagagg
1343401 aggactgata taatagagag ggtgggagga tgaaaaagac atatttcaag ctcagtagta
1343461 ttcccagagc tcgtctagac ggaaatgtat ttatttaaga tttaagatgt acttactttt
1343521 atctgatgtt taggaaaagt cgtgtttttc ctcttgtttg cgcctctcgg cgcatgcggc
1343581 tcctctggcc cggagtcctc ctcgccttcg cgcctggcgt gacacagtcc gtaggttttg
1343641 ctgctcttct tagcaggggt cgtcttctcc gaaacccacc ctcgcctctc aggaaccgcg
1343701 tccctttctc catcgttgtc ttccccagag gagccggaat cgtggccttc gcggtgacgg
1343761 tcccgctgcg cgcacttgcg cgcgttgcgt aaaatggcgt ggattttagg aggctggagc
1343821 ggacgcttga ccggcttgca tgaggctttg atagaggcca cctggccgac acacgcaggt
1343881 gccaccttct tccctcggct ggtcccgctg cccatgactg ccccctcaaa gtctaactgt
1343941 ccatatccaa gttcaccgag tgctgcccgg agttgaacat taaccgccag gtttgagttc
1344001 tggcagcgtt gcagggcagt tgtgcagtta gtttcacggt tgggagacag atctctggct
1344061 cagcagcggg cccagatgcc gactttacag aaacatttcc aagcttgtag aaacgatcca
1344121 cgggctgcgc gcacgtcgct gcgctgcacc agcgcaaact ttctctaact ccacatcagg
1344181 atgagaagtt tcggttacat aaccgcaact tggctcagga ccacaaggac aaaaccccgc
1344241 ctcctcaagg agacaagcac gaacccccaa gtttccagga tttgggcaaa aaataactct
1344301 ttgtctaaac atctctcgta aataaaccca gaaaaaacac gtcccgcatt taaatatcta
1344361 gccggttgta taacgactga gataattgac cctaaaagga gaacaggcaa gggctcaggg
1344421 ctggtttgat ttatctgcat cacattagca acaatgagcg gccattaacg cgtatgtgcg
1344481 agataaattt cattgaagca agtaagacca gtcggcttca cccacctagt ccgggggggg
1344541 gggggggggg gtaccagtac cagtactaga gagagagagg tattgaactc cccagagatt
1344601 tctttctaag cttttgtttg tgcagttgcg cgcacaaaga ccatcatctc tccaccagag
1344661 ctcaaccgag gaaggagctt gtttgctatc tttcattaac tgtctcattc tctctcgctc

1344721 tgagtgtgtg tgtgtgtgtg tgtgtgtgtg tgtgtgtgtg cgtgtgtgtg tgtgtgtgcg
1344781 tgtatgcgcg cgtgtctaag acagctgtgc aacctggtcc ctctggagga tggcccagtc
1344841 acaaacacct gctgttaaaa caactgtttt ctaaactgtg agaggggcca tgagagaact
1344901 gtttaaggat tgtttaatgt atattttaca cttcctgccc ctccaactac cccccccccc
1344961 acacacacac acaacaattt gagcttttaa aaaggtctaa tttccatttt gccccattgt
1345021 aaccacattt gtcgtgctgc actggtgcgc tgtcatctgc agagggggtc ggaccagggg
1345081 gaggatcagc aaccagacgg aggccagagc agcataactg tggtttccca gacttcctcc
1345141 acgtttaaga acattctcgg taaaagtttc actctggtca gcagcaggtt ttatccgttc
1345201 gatctcagtt ctggcacttt attttcagct actgaataca aatgtgctct gtcttctccc
1345261 aacagcagga gagcccgtgt gataaatgtt aataattgag actcggaggc gtctgagagc
1345321 caaaaaaata aacttgttgt cgccgaagac acatttctgc catatgtctg ctaaaaaggc
1345381 cctgatctag ggcgagaatt ttacaagcca gctgcccact gttaggcatt ccagtaaaac
1345441 cctctgggcc cctaccctgc caggtacaca cacatgcatg tgacagggtc aaaaacgaga
1345501 acaaccattc acacacacat acacacacac acacacacac acacacacac acacacacac
1345561 acacacacac aaatacacac acatctgcat tttgacattt tcaagaacag aaagtccaga
1345621 ttgaggcctg atcctgtgtg attggacaaa actacttttc agccatattt catctctgct
1345681 catgattctc tcagatgatt ctggatccaa acaatgaacc tcaagagacc tttagagagg
1345741 aggacacctt ccccgtcagg agctcctccg agacacagat cacacggagt attgtattgt
1345801 ctgacacagt gacataaggt tgtcagggca acagtcagcg ctgccaagag ggggttcagc
1345861 tgcagtgaaa gagccagggg cagccctgtt tgtctgcaag cgatccctct taatccctcc
1345921 attagtaaat gtaaatgaaa gccgccgctg ctgcgttgac gtcacacgga agacctattg
1345981 ccctctggag caggtcggtg ggaaaccgga catcgtgaca cgtttacagt ctttcgagtt
1346041 acagtccaac acgtgagact ccaactgcaa ccattgtgtt ggcctcatga aacatgctcg
1346101 aaaggctgcg tccagcaggt ggcgctatcc ttcaaggaaa acagagatgc ctggcagaag
1346161 aaaggaaggg taaaaaaaag aaaagcacat catacatttc ttttgaacaa aactgtaaat
1346221 cacgggttta atgtggggga ggaggtttga gaaacgtgga ggataaaaaa ccaaaacatc
1346281 ttcctctagg aaagttctgt tatgtcccaa tgaagcccgt tatgggcagc aggggacgat

1346341 tcaaacagcg ttccgactcc actgattatg cgtccaaaat tgtaatcacc ccaacagcag
1346401 atatgatcta cagcttttaa aaagcagagg tgaagcactt ctctactgaa ctgtactgtt
1346461 ttaattggtt taatattttt ttaactgtac cattatacaa atggaaaatg aataacatca
1346521 taacaacaca agacaaatga ctcttctatt ctgtgaacta ttactgaatt caatgtgcaa
1346581 caacatctaa atggctgcca aagtttcatt gtattcatgt gcctaattat atctgtttgt
1346641 tcatgatgtt tgttagcata gcagaaaatt aaaacttgtc tgtacctgca caggaaaacc
1346701 aaaacacaaa agagaggcgt ccatgaataa ggtgtcctga gaatcaaaag acagtgtgga
1346761 gtttccttga agtcatccag aagatgaacg tcagcagagg cagagaggag gacgctaatg
1346821 aggaagaaga gcagatatag tggaagatca aggaccggca tgggatgtat catggagatt
1346881 aagcagcgcc ttgagaagaa ggcacatcca gctttgatct tggcagcacg ggggcagcag
1346941 aaactctccg agcaaagcaa atctgtgctg aactcagatt agaagtcccc aaagttagag
1347001 atcccatcaa actcagcctg gcaactttct gacaggaagc tagtggctta gcaacctgac
1347061 gcggtggtag atgcacaaac caggggcggc cgaggagccg cgtcaaccaa acagcacagc
1347121 ggtgggatca caagttaaaa atagcccaat aacaaaatgt actcttaata tttactctgt
1347181 tttatctcta tttgggcctt cagctaaaaa ggctggtaca ggttaatttc aaaataaaag
1347241 cctgaaattc atttcccttt gttctttgtc atcaggtgca gcccggtccc gataataaag
1347301 ccgagccaag cgctgtcgag ggtgtggtgg tcagcaccgc ggtgacccac tttcctgtca
1347361 aaaggccatg taatcaacag aaaacaatta ctggctccct ccagctctcc aagcactcga
1347421 tacgagcatc tgctcacttc ctaattacct actaggtggg aagcttcatt tggaggagaa
1347481 ctcacggcaa tcaatcctta cgttatacct gacttgtttg gatgcgttgg gccccctttct
1347541 gtttgattaa cttctccctt cattccatgc ttttaattta tagacaaaca cacacacaca
1347601 cacacacaca cacaccagtt tgttcagttt ttcccctcaa cacatcactt tgaacagtgc
1347661 aatatcttaa taatatggca aaaatggaaa cggttctcac agtgactttc actttccctt
1347721 catccccaaa tggttcagca acttctcacc ccagctcctc cagacggctc cgtcggcagc
1347781 tcagacatca cacattagat cctgacgctt ttctcacccc cccacccccc ccgtgtcatc
1347841 aaatggtcat gcacagctcg ccatcctgat gaatgtccag attaatgtct gcataactaa
1347901 tgactgcgcc tgcaaacagg caaatacgat tatgctaaac cacatttgta ccatgctaac

1347961 acgcatgttc attagcattt taagctcatc ataagcgcta aagaagcaat caacaatctg
1348021 ggtgatcggt gagtcaccag tggatcgcta ttgagccatt cttctaaatt cccgtaaaga
1348081 tttattttat ccagtcatgc aaaacgtgcg cgcaaagaga ctttgagagt gtggaacaaa
1348141 cagatgcagg gatgctcccc cccccatcct tcgctccatc agaacaacac agagaatttt
1348201 aaatggatcc atccatccaa gaggcgtttt tgaggagccg ggagcagcaa caacaacagt
1348261 gtgatgccgt ttgtgtgctg acaggccgag aatgtcgagg agctttccta tctgtgctaa
1348321 aaatcctgct gccccataga atcgatctgg gttacacaga cgtgacataa ccggtgaatt
1348381 tggaggcatt ctgggccacg cttgactgca tcagatattc tttcaagtaa taaatagagg
1348441 tctagaagga gcctggatgt gctgcttgac tgatacagcg gtcaggtaca ggaaaacaag
1348501 acgttctagg tggacgcgcc tctgctgtct gggtggttgg ctggtctcct tatgagctca
1348561 aaatatggtt aattacatat ggagttcatg ttccatagga gaataggaga ggagcctctt
1348621 tctatgatat tccagaggca gagaggtcaa aaatagtgtg tgtgtgtgtg tgtgtgtact
1348681 cactctacta tcaaagtgag gtcatgtatg caagtgggac tatatatata taagtgtgtg
1348741 tgtgtactca ctctactatc aaagtgaggt catgtatgca agtgggacca tatatatacg
1348801 tgtgtgtgtg tatgtatata tatatgtgtg tgtgtgtgtg tgtttacagt atatatgctt
1348861 agtactgttt tatgcatgat gatatcactc actcactcat tttctactgc ttgttcctcc
1348921 actgagggtc gcgggggggc tggagcctat cccagctcaa tgggcggagg cagggtacac
1348981 cctggattgg tcgccagtca atcgcagggc taacacatac agacacacaa ccattcactc
1349041 tcacactcac acctacgggc aatttagagt ttccaattag cctaatcccc aatcatgcat
1349101 gtctttggac tgtgggagga agccggagta cccggagaga acccacgcag gcacagggag
1349161 aacatgcaaa ctccacacag aaagtcccca atcagtccca accggggatc gaacccgggg
1349221 ccttcttgct gtgaggcaac agtgctaacc aacacaccgt gccgccctgc atgatgatat
1349281 gttataaata atatttttca aggagattca acataattaa ctgatcacca tcctttgcct
1349341 tagttcttgt gacatcacat ttattttata cagtatttct tgatcataaa cggggaacgt
1349401 ttgctttcta ttctggtaaa tgtccgctcc gctcagtctt gtgacagcta aagttcactt
1349461 gcaaacagca gatgttggca cgccaccatc ctgttccgac aacgccggtt gccttctgac
1349521 cgatcagacg gaatctaccc aaacgtgatt acggctccat tattggttca acgctccctc

1349581 taatcaacac atggtgaaat aactggaaat cctcgccgag gttccatgca caactttaac
1349641 cctttgcaca caaccacaga gatggaacta tggaacctca agggctcatg ggggaataac
1349701 gtgcccagtt cttgacaaat gacacccagt aagcaagtct gccatatctg acctgaagat
1349761 ttatgtctgc gatggcttcc aacccacttg gatctgtcag cgggacattt ttttatttat
1349821 tcaattattg ctttggtgaa aatggatgcc ctccctccac cactcaaaag aggcagattt
1349881 taaccagctc tgcacataat aagacagcag caaacgtgct gacagatggc ggcggcgctg
1349941 atcaaaggct tcacgtgggg cttccggggc gagaggaggg gaaccagagt tcaacacgtc
1350001 ttccttcaat caccgttttc cttatcagac ttatcagaca gaccaaatca gcctgtaatg
1350061 gcgccgtgga ccaggacacc atacagcaga ggctgcgggg agattttatt ttgttttggc
1350121 cagatcctga atgaaagagt gaatgtgggc tgttcagtat gatcctccgg agccgacggg
1350181 cttcctctgg ctgttgttat cagagctcgg cggtccgggc tgaataattc agcgctcgca
1350241 gcagcacctc tccctctctg caccgaccta gtttacatca ggagtttaca ggttctcgcc
1350301 atcctgcagc ctcaactatc cagacgggct cgcaacgaga taagcttccc atggggtcgg
1350361 aaataaaggt agaataaaaa ggaaggaaat gttttcacct gataagtttg tattaggact
1350421 gaatgggtga taatgtctga taaaactccc ctgaacggcc ctggcagcat ctccttctgt
1350481 ctatttgctg ctcggtgcat cacgaccaga ataaacaagg aggatccaga agatccaaag
1350541 atattctcct attgttctct acggaagcca tcctggaatc ttggatgaaa acggccaaat
1350601 caaaatctct tgcagggatg ccggctggtt ttaaacagaa gatgaggtca ggcagaaggg
1350661 ccgagtcatg gaagcgcttc ttctgagcgg gagtgggggg gcaaatacct cagaggtcgt
1350721 ggagcagttt agtccaggtg aattatgggt attttcattg gaaaggacgg caaaactgaa
1350781 ctgcaacaaa aaggcagccg cacgtttatg tctgattttg tggcggcctc agtctctcta
1350841 acatagatta tgagcagcat ttacaacaac cacgacattc agagctactt tcaatcaacg
1350901 ttccacctgt tgacctgtta ccttgccaac ccgctcgagg atgctcacgg ttccccttcg
1350961 accctcgatc ccgaattagc ctcaatcccg gagcgagaga ggcggcggta ccaaacggtc
1351021 agtgacggcg aggcgttcag agggaaaacc agcgtgactg tgccgacaac aagggatgga
1351081 acaacagatg gcaaacacag gagttaagag ttagatgaaa gggagagaga ggagagaggg
1351141 agagagagag ggagagagag ggagagaggg agagagaggg agagagagag agagagagag

1351201 agagagagag agagagagag atgaagccca ccaaagaaaa attgaattga acagcctcgg
1351261 gttcacagca gccaaggtta agggaattca ccaaagcagc ggtgttttca taggaaatca
1351321 atagtgtgct tctcattaat gtccttatgc tgcttttgta ggagagccgt ctgattcctc
1351381 aacatgcaga aaagaagaat caaatctctg tccgccgtct gagaaggctc tcgatgctca
1351441 ggagaggacc aatttattct tggcctgaaa gctctgacga caagagaaac ctgcgatcgg
1351501 atacttggga taattcatca ttttccactt tgagtgtttt cacacttttc agacatttcg
1351561 gccacttcac cactctcact gctcgtgtgt gcagatgttc ccactgacct ctggccgacc
1351621 tctaagcatt aatcacacct cggagtccac cgtggacatg cttgccttgg gtcaatattc
1351681 agatgctcac gtggagccgc tgatccatcg tggaaggaaa aaaaaaaggt tttagaattg
1351741 ccagcaagaa caggaaaaac aggcgaaagc gtcccgtctg acagaagata cgggagattt
1351801 ttaaaacctc cgcctgtttt taataacaaa ctgttctccc aaaaagtttg tgctgctgtt
1351861 tttgcctgtt ttattaatgg gtcaaccttc tccagtcagg agggagagcc agaggacgag
1351921 tcattggtaa aaacaaagct cttaaaaata ttgaaaaata tcttataaat acaaaacccc
1351981 aacaggtttg tctttgtcca ggtttttatc tttgatgacg gtacatcctt ttgaactcct
1352041 ggacagatac agaatacctg tttgctgcct gggggtcatg ggtcaagttt ggtcaaacta
1352101 gcatacttag tggttgaatc cctccccagt tcttcctggt ggcgtgaaga gacctggacc
1352161 attggtccaa agggaaggag gtcctcaggg gagttccaga ctgctagttc ctgcaaccac
1352221 ctgatcctgc ttctacacct ggagctcatg ctgctcatgc taactggatt tgaaagaaaa
1352281 ttaaccctcc ttacgcctga atctgccccc ccacacacac acatccacac gatggtgttg
1352341 cctgatctgc tctgtcaatg acctcacttt gtcagtaagt gcagtcaggc aggacaatga
1352401 agccaacgag cccccggcca ctaaaggcca cccctgctta gctcctgatc cgcgctgccg
1352461 gagctgacat cagaggtgtt attaccatat aaatgagata aggttttagt aattgccggc
1352521 tgaaattaaa gccaaactgt cgtctgtgtc ggagacagtt ttttccctgc acgtggaggg
1352581 ggggaaaaaa aggggacgtc ttacaggaga ttgactggga attctggaat taatgcattt
1352641 ctaattcgct cacaaaggag agtagcatta gcttagttct atcttatatc tctgcccaag
1352701 cacactgtga tacagagacg ggaagaaggt ctattaaaag tagcttggcg gtgtatttac
1352761 ttaattataa gcccgttttt gaatacccat gaattgattt aatagccttg tcaccggaac

1352821 acagtggaac atgcttagaa gagcaaatgg agacaagcct tatttagaat tcctttaatc
1352881 cccttaaagc tgtctgattg taaagagcag gaaaacacca ccctcacagc gaattagcag
1352941 tcagaaaatt gcatcttgat aagcgccgct aattacgtct gcggtgaggt gtgtgtcacg
1353001 cgctgatatt tatgatatta tgccacgccg ctgtggcact gaggaagggg cactggcatt
1353061 tattagttga cggaatagcc cacctgggat gagaacagcc gtgacatttc agcggcggag
1353121 cagggcaatt accccgcatg tcagcgccga tgcagccgag ccggcctgct gttgcagtta
1353181 gtctcggcta ggatttgatt tctacggagg tttcaccagt cggaaataaa aggaatcttc
1353241 tgttatgtga atggcgacaa cattaacgcc actgatgtct ggtctttctt ttaaaggtgt
1353301 ttctggacac ccaagtctta aggttgcatc cccagtcgtt ggagctcggc gtcctctctt
1353361 tgctgtcagg gttccgtccc ccgcccagca ggagctggat aggggccccg gtgtgaatta
1353421 tcacccacca tgcttgatat ttaccaattt atttattttt ttctccctct ttctgactca
1353481 catggctgtt tctcagacga gacctggcag ctcaccagac gtcactgttt tcctcttggt
1353541 ttcacatatc tgtcccacat gattgatgat ctatatgtat tatcctgttc atatacgtga
1353601 ttcgttgtta aaacaacata cagtgtgcta aatttgttgc taattcaacc aatttcttaa
1353661 taaaattaat ttactcctat tcaatgttct ggttttatgc ggcgcctcct tgagaagcat
1353721 aaaaaacgcc ttcgcccgcc tctgcggagt ttttaatcca tctgattaat ggaagttcct
1353781 gcgacgtacg ttgaccttgt tttccgtctg ttgctctcgc ggggcgattg aatgataccc
1353841 ggagaatgac gggaaattat ctgacgataa agagggagcc gcgaactatt gatcctcaaa
1353901 aatctgacgc cacaaagggc aggagaacat ttgccaaacg tccggggatg attgatgact
1353961 ccctgcaact atatttggcc ctcattcata acaaacattt ttccgaaaaa tcaaccggaa
1354021 ggcgcgtccg gcttgggagc gttccgccgc cgttgacacg tgctctacat aatacatgag
1354081 caccggatca aacaaacgac cccggctctg ttgtgacctt tcactaacat gaagtcacaa
1354141 ataaaaccct ccactaatca cggcagcgtg tttcaattac cccccgatct cctcttccca
1354201 gaacgccggt gcctgaaggt catggcagcc tttcagtact gcatcctgtc caggatgctc
1354261 tgcagttgac ctttcgctgt cacttggcgg gatgcgacag atccgtatgc aaaaccttcg
1354321 tctgtcacgc tgaggaagga cacaaataca ctcacaggct gagaggaaga acttgtctca
1354381 tacacagagt attttatgat attttacatc atcaacagaa caaatccggg cttttatttt

1354441 gacaggacgc tcacaccttc atcttccccc aatcagctca agggaaacca gcttcaggtc
1354501 tggtcatcca cggagcattt cacaccagag ctgtttggtc caggaatggg tttttttccc
1354561 gctttggcag gtaggaagtc taatgggaca tctggtgcgg accaaacaag tgagtctttg
1354621 gtatcaattt gtctccaggt gaaacctgat gcaggaaata caggcgaaag cgtcccgcct
1354681 gatgtccttt aggtccagga ggtcctttag gtccaggaag tccaccaacg agacgtagga
1354741 cactaatatt tggtcaggct aaagcctaat ggggatgttt ttccattttc agttctggtc
1354801 tgctttcaga agtacactat taaagtaaac aacctaaatg agggtgtgaa atccctctgc
1354861 atttcctgct tcagcagaat cctgttctgg actattctgg catgaagtca agcttaattt
1354921 atagccagta cacattagca gtgcttacga aggccatgac agaggcacaa cctgccgtgt
1354981 tgcttccgat gtggccaaga acgcgttcct gtgttaccct gcctaaagac ggacggttca
1355041 agaacattgg gaagcttgtt ggcacccaca acaaacccac acaactggat taaagtcagt
1355101 gtttggacct gattagttaa gcacatcgac agaaacgact ctcgaaataa cacggttttc
1355161 ttcctggttt cgtgcagcca gattcagcaa acgtgaggga tctttttacc tttacgaggt
1355221 aaaagtcaga gtccctggga gccggctcca tcctaattac tggaaatagc gcctgtcagg
1355281 aattcagggc gagacccgca gccacgaggc ggacattaac atatggaacg ccgggaggaa
1355341 tttaatggga cttctcattt gaaaatgaca ggatgaaaag aggaaacgca gacgtgctgc
1355401 atttatcctg aacacaaaca gactctcaga cacgctgggc gcagttgcac aaacacatca
1355461 gacatttgca tccccacatg taaagcttcg tgactcttgt gcaggcaggc ggagcagccg
1355521 ctgggggttt aatcgctgaa tctgccccct cgtcattcac aaacaccact ttgtaatcat
1355581 cccctgacgg tatcggactc caaatgaagt cagccatcgt gtcaaagtaa aagatgcttt
1355641 gcatgatggc cagttttagg atgtttatgg ttcctttggc tctcctgtga ctccaaactc
1355701 cagctgatgc ccttcatgtt atgagatcca tagtgggggg ggggtccaac tgagtcattt
1355761 agtggacaga agtggcttaa aatagctcct caaaatactc gtaaagagca gaataaacat
1355821 ggcagtgatg aatataatca gctcacacgt acagattgcc agtgtttgtt cctggactca
1355881 gagctaatca tcaaatatta aaccacctca gtttgccgcc acgggctaaa actgttcaca
1355941 cagtcacggg gagactgttt acttatcctc atccttcatt gtttctctct cttctttgct
1356001 cacagaggtt tttgcagaaa gttgggcaga ttaattggaa aaccataaat cttctctcga

1356061 atcaattctg tggatagagg ctgcagcaga ctgccgaaat gtaaatttcc ctctcttcat
1356121 cagcatacag taacaagtat cgtatagttg tagccgcact ttatcctaat gagaaataag
1356181 gcagttgtca ttactctcta tgtagttata ttcatctgtg atctaaacta tgacgcaggc
1356241 tgcacagtcc cacacctgca gccgtaatgt atataaagga tttaaaagaa gacttcagct
1356301 tgtgtggaaa cgcctcgagg gttcatgtca aagagccctg gactgatacc ggatcggaaa
1356361 ttgctcttgc atcttcaggg tgaaggctga cgtttcaccc atccgaggac ttaacgattc
1356421 atcagtagat acggtgcatc cgacagacgt atggcttaca cagaggcagg taaacgccgc
1356481 ggaggctgca cagatgcggc ttcttcagga gaaagtggag aaaaagatgg gaatttcaac
1356541 tctggctgaa tctgtttgca gcgttgaaac ttcttccgtc tccacatctg ggaatttgcg
1356601 cagacggctg cgtgttccaa cgcctgtatg caggcggagc agtcgccaac gcttgtttgg
1356661 cagcacaaga tgctgaactg cgcttcaacc gtggggataa tagtggaata ccattgagca
1356721 gaggtggaaa tgagagcaaa gcgtgaggat tttcatgatg aagcactccc atgcgcagcc
1356781 gccggtttca cgacgcctct ttatccgtca ggaacgtcag gtgttgtttt ataacccggg
1356841 ccgccatctg ccagcgcgtc aaactcaggc accctgttct ggatccccag acggcagcgg
1356901 gagtgatcgc cgccagtcga gactccccag acgagatctg aattattaaa aagtctcaac
1356961 cgcaactttc tttcttttt taggggggtg aactcttaaa cttgggtgat ggagcagcag
1357021 gggcacctgc gtcatcctga aatatggctt tgattgcttt tattttgctg gcctgatgaa
1357081 agcagcgaga cgttttcggg tatttcaccg taaactgctg gaatttaatt tggaaaagca
1357141 aaagatgaag aggtccttct gccatattta tggctcctta aaagcactga tgcttttgtc
1357201 ccggtgtaaa gaattacagc tctgcgttat gagctccttc ttacataaat gtttatacat
1357261 cttatgcgcc ggtgccagcc tggattagcc tcatgtcgct aagtgctctt aatttaacct
1357321 tttttgtgca ggttatgtct gccatcgctg tctgcgtcct cagaacgcag cgctgagtgg
1357381 cacagacgga tgacgggtgc taaactccac aaactgcttt tagttattca agcggtgtcg
1357441 ctctgaaggt gaacccgaat tactcgggtt cagaaaagcg ataacttgtg atggcgcctt
1357501 tcgtgccgtt cgctctgctg agtctcctct cgctctgaag ctacaatgtc atatacggta
1357561 aacccccccc cccccccccg ggacctgaca atattagtgg gactgaaaaa aacgctgtta
1357621 taatgtttgt catgtgatct gcttataaga gcgctggtta taattcatta caacggtgta

1357681 ttagctctgg aacgctcgtg tgggtggctg aattgcgcgg ggctcataaa cgcaggttca
1357741 ttaaaatatt gcaacacact ttcatgactc cacttgagag ggaatcgctc cctcgaatgt
1357801 ttttcgcagc ggcgccgagg tctgctcccg ccttcccagg tggtttattg gcaccccagg
1357861 atccgccgcg cggctctgcc cacatggaaa agctaaaaat tgaaatcccc cccccccgct
1357921 tccgccctct gcgggtgttt tcgcagcctg aaaacaaata cagaaaagtg cgaagtgcaa
1357981 atttgtcaaa acccagctgt gttcgtgatt gaaggggtca tttcccgaaa aacagccccc
1358041 ccccccaccc cgttagcaaa gctctctgta tatattctct caacaatttg ctgttttata
1358101 gttaaaaaat tagcctgaaa acaactcatc cttcagccaa acgacgtaat aaggatcgta
1358161 gcaaggctgc catgcaggtt gttttgcaga ttgaacaagt tacagctcca ccctctggcc
1358221 cggcatgtgt actacagcca actacttctg caaatccaga tacattttct tgcgtttgtg
1358281 acgtttcttg cagcctttaa acatttctta agcgctgttt aatatttctg tcgcgcgcag
1358341 gttgtgtgtg aaagcataaa gggcccaaat aacattgtca gggtcaggag tctgccaaat
1358401 taacctttcc cctgtctgga agatagcagc ggtcagaaaa gaagagagaa taataaatgg
1358461 agacttttct ttgtcacaaa ctccttcatt cctgataaat actcagagga gatgttcaga
1358521 tgtgtggaca tacaagaatc ctcaaatggg atcagtttta cttctttttc atctaagact
1358581 gtattgaatt gtggatttaa gatggatttt gccattaaca agctagccag ctggctagtt
1358641 cccagttccc tttattaaga cggttttaag catttcagca gctgttccag gatttgaacc
1358701 tgtgcacagc tggacaatca cacggaccag aactgctgcg gtgtccaaac tcagaaaacc
1358761 cagaaatgac ctagaaagac gcgcctgact tgggtcagca cttcctcaac cccttaaaaa
1358821 gcacatccag gtcccacgcg gaaagattaa caagcattga tccgagcacg agttgctggg
1358881 gcggcgaacc accagtttcc tcacgggagg tgaaatcccc cattaacttc ttcaggtttc
1358941 ctctcggctc aactggatcc aaagcgccgg cgttcgacgg ctggcgtcgg aatcggagag
1359001 cgactagagt ttgtctgtgc accaacagaa agcacgaaga agccacagcg ataaaaacaa
1359061 atcaaaactg ctcagtcaca acaacagaag ctcacaaagg aagacgggac gcgactctgc
1359121 ctcctgtctt ttgtagatga atcagaaatc atccccgtgc tcagctaacg ccgtctccgc
1359181 cgtggctgct ttctgcgcga ccttgctaat cagtgtttca aaagcacaaa agtgcagcgg
1359241 ggtcgccgcc gtgacggcaa attatcgctc tgccggagtg cgcctgcggt aaactggccg

1359301 ttctggtccg tgggtaactg tctgacttcg accatctttg atgtggggag gaggtttgaa
1359361 gcgagatgga ttaaaatgca agattcaaga ggtgcttttt acctcttgaa aaacaaacaa
1359421 acacaaacga acccattttt ttagtggatt atgttggttt agtattgaaa tgagaggaga
1359481 gcggagcagc tctgcctcac agctattgac ccattcagct acctcatctt gatggaattt
1359541 agatataact gacctcccac gtcctccgtg gcggcatcag cagcaggtca gcgaggggcg
1359601 agcgcagcat tggacaatca ggtcaagcct ggttaaaaat atacatcctc tatacatcct
1359661 gcagccggct tcttgtccat ctgacagata tatgttggca ggtaaacgca cgcttctttt
1359721 agcaagctta aaaaaacagg ccacatcggc gggactaaag taagtggaga gcagttagca
1359781 cagttagcat ctgcccacct gaaatgaggg gggggggga tcatcctaat tgagatccca
1359841 acgagtgctt ccctcataca ataaaaaata caatcagagt ctctatttaa ataattgcat
1359901 ttgttcctct ttttgcccca agttaattag caaaaaattt aactcaggga gggagaaaaa
1359961 tcgacaacca gccgttatta agagggataa acacgggctg tcagcacatc tttacaagcc
1360021 ttatgcataa tactaataat cttggcggct ccgaggtgct tcggctctga aagggttttc
1360081 atccatagct gaacaatttt ctgctcctgt gggatccttt gcatcaattc aatttatttc
1360141 agtaaatgga ccaattcatc agggaaattt ggtatgtcat ttgtgttttt catcaagcgc
1360201 taccttcctc tactgttttt aaccaaaacc agtggattaa acatgcacac atcttcaacc
1360261 taaaacgccg ctacatgaaa ttttaatgcc tgccgctgac agcctgaatg aaaggagcgc
1360321 tcagtataga aatggatcag tgataattaa taatacagca gagttttacc agtctctcgc
1360381 tgccactgaa tattgattgt tgagttaaat tcaattcaat tcaatttatc tttatttaat
1360441 agcatctgtt acaatcataa ttgtctccag gcgctttaca aaagcccagg gcctgacttg
1360501 gagtgaaagt gttgcttctc tagcattgta tgatgaaata attggacttt aagtggaatt
1360561 taaattgatt caatcaataa cgaggcaaca tcggtatgaa accaagacct ggggagtgac
1360621 tgaagctagc agagaaggtg gaaaagtcaa gctaatccaa cccgggcagt tatcttggaa
1360681 taagatttga gataacagcc attttaataa ggtttttctc tttttttcgc tccgtcctac
1360741 cagatgtgcc atgagctcct ccctggcaga ccgcgctgac gggcccgtgt catcggcgtg
1360801 acatttcagg atccttcaca ttccatcagg atggtaatta atcatggtgg gaacagtgtg
1360861 gcagactgga aacaccattt aggagctgcc aggggggcgc tccttggcac attccagggc

1360921 aggaagggga gggaaagcgt cgtcatggcg acggctcagc agcaccgagg accgatcggg
1360981 gcatctgaga catgtgttgg ctgtgacccg agaacaggaa ttagataggc aggctgctcg
1361041 ctgtctgcag agatgagggg caacgagcac acgtggaagc tcggctgtgt cagtgaggta
1361101 agacgttcca ttaatgaaaa aattattata atgtcattat cacatagggt ttctaaatag
1361161 atgtaaagaa atctaactta cactctcatt tatcacagtt ggatcacaag ggttcatggg
1361221 agttagacta gtaaagcatt gtaaaaagtc tgaatccctc tttcgtcgtc tcccatctga
1361281 tgtctgatgc ctctttattt aaaataagtc ccaattttct ttcaagagca gagacatatg
1361341 acataaccta aatgatctat agctcaattg attttgactt tgtttgcaaa aaggctgcac
1361401 cagtgtcatg tcaccgttct taattccaaa caataaattc atcgtattat tactatttga
1361461 ttaaagacga ggacaggagt gtcataaaag caggagtaaa tgcacttgca gacctcctcg
1361521 tcggggctgt aaaacacagt gtaccaggcg gggtaaggaa acagcctgca ggcaggaatg
1361581 gaggctgaaa gagaaccaga tgtggcccag actttcacgg tgtattaagt gtaggagcag
1361641 aggtaaccta cttcaggtct ggcagctgta cacacgttct ctgaaatgcc acgatgaata
1361701 aatcagagga aataaaagta ccaccttctg gagcgtatca ttatttcatg aaagttctgt
1361761 ttaatgtaca cagccttgag gtacactttt caggcggctc ctgttttcgc ccgtattcac
1361821 acgagtcgcc ggcttgtcac cacgtcaagg tgacctttaa aggcattgtt accgcgtgtt
1361881 tgggtggctg ttcttcattg agggacggta atgaaaacag aggtgccacc cattgccagc
1361941 ttttaatgtt aattggcagt gatttgtgaa gaaggaaaaa aagaaaagaa aaatggcctc
1362001 tgtccaggac aaagcagctt cagaggcgac ggctttagag gaggaggagt gtcacaaaat
1362061 atccttctag aagggaaagg cagcgggatg aatactgact ctcagagcac gggatggaaa
1362121 tcacgactcc ctgagtcggc caaacaggtg aggtcagatt attatcaaag ctgctgaacc
1362181 agaaagttga tactgtgtgt tccaggagct ccagggggaa gacactgaca gttcaaggcc
1362241 ttccagtctg caagaaagca ggcgagctcg gtagaaatgc atctgcaagc aacggatgat
1362301 tagcatctaa gcttgcttac ccgacagtaa taaatcaacg taagcagaag ggaaaccgtg
1362361 atagtgcacg gacgttcacc tttttaattc acttattcac tccaattaat gcgcggtcac
1362421 aaacgccggc cgccgacctt ggtgagaggg tgaggtgatg tcaggagagg aggagaaaaa
1362481 atgttagcct tgcggctaat gcagctcatg gagcaactct ggagcttccc tcacacatgc

1362541 tctgcttcat gacctcacca ggaacacatc cagaatcatc ttccactggc cgcagcctcc
1362601 agaacatgac atcaccacat ctgtaatggt cacagctaac ggggctgtga tgtgctacag
1362661 agtccaaact ggaacgtgtg ttagaaagtg gagcacccat aggagatccg gccagtggct
1362721 gacagagatt aaaagaagac aagtgcatga tagcgaacac agataataga tcagaatcga
1362781 tctgaaggac tgagacgaat cacttgtttc actgctgtga catgatgtca ctgcttcccc
1362841 aaaagggtca ttctgaaaag aacaatgaca aaccggctgg aattaacttc attaattaga
1362901 gatgttaaaa atggagatgt gaaaaccaat atccactaaa atacagtctt gagcccggag
1362961 ccgcactctc catctattgt tctcaccgtt ctgaactggc ctggatgtct ttgacggcac
1363021 ggtagcattt tttttctctc tctctctctc ttgttttgtc tgtgcggctt cgcagtgaaa
1363081 tcaatacagc aatctctcag tcttgacggg ttttgtttgc tttaggacac agctcatgtc
1363141 ataggctgcc ttcagtccat gaaaagccgc cggtcgccgc ggcggccgtg gagcccggtc
1363201 aggccgaggg cccgcttgtt gggcgcttca aggcagtctg agggtctgat ggatggaaat
1363261 cgaaaggcag atccatagag atcacagctt aaatgaagag tgattttaat tggcttacag
1363321 ttattgatgt gcgtttgctc caggctcctt gcacttggct ccgagggcga cgccccgcat
1363381 cagcctggct cccagcgact gaagacattc taatcctgtt gaaatgaggg gtgacaatag
1363441 cagtttgtat cagcgtaatg tctggctgca tcatgtccct tttttctgtt gaccgataaa
1363501 ggaaccagtt gaaaacctgg tttcatgcta cgtgttctta gctttttatt tgcaacagca
1363561 tttgtccagg aaatggttcc tactctctaa tcgccgaggc gcctccagac tctggtctcc
1363621 ttcaggattt ttaccatggt cccagatatt tccttacaga taaacgcgag cagacagaac
1363681 tgaaagattc cctttttttc cccactgaaa tctttattaa aatttgcaag cgctgcacat
1363741 gattatgaaa taagacctgg aacacagcct ccaacttttg atacacgcac tgtcagcagc
1363801 agggaaatgc ctctgagatt aacacggagg acactttccc ttattgcact aaggctgcct
1363861 gaattggccg ttttaataat gcatattcac tcatgcatat tcgtagcact gcatcaagca
1363921 aagtgaaatt aaacaagttt atcatacaaa agaaaaatac gtttacgttc catatcgggc
1363981 ttcgtctcta aacgatcagc agatgtttca ctatcagaag atcctgaaat aagacggcta
1364041 ttggtggtta tcaagacttt ataattaatt atagttctgc ggccaatatc ttcggagcct
1364101 aataactaag gaagagtttg atactggggg agtttattag tggtggattc tgtggccaaa

1364161 gggaatggat acgttctcag atcatctctc cctgctaaag tgacaaaagt gggcaaaaaa
1364221 agtaaatata atcaaatctt ctgtacattt gaggcatatt cttcaaaaca tggatgcacg
1364281 gtggttatta atgttttaaa accaaagtga tggtgacagt ttctgctaca ctaaagacaa
1364341 gaaatcagtc caaatactga tattttaatg aaatgatttt gtcaaaatgt actaacttac
1364401 agtgactgac aagagaatta accagaaacg taaagagtag cacaatcgca ttcacacagc
1364461 cgatctaata caggggtcat ttctgaggag tgggggctgg agaactctga tgggtggaac
1364521 tctggcagtc tttagttttt aattgtattt ttaattgttc taatcgtttg gaaaagggag
1364581 caaaagagga ggacgagaac atccgggcgt atttgctgag ggagagtggg aaccttggag
1364641 cggagggggc taatgtgtat taattgaatg atgaacatgg gctctgttaa tcaccacacc
1364701 tcctggtgga tcacatggag actgttatcg atcagagtcg gtgctcagct caaggctgga
1364761 tctgagtcca aaggtcagac caagcgtcgc tcactttcaa cctggtttgt tgtgcggaaa
1364821 agaaaaaatg aggggggtg atggtcagag aggggattaa tctcaccacg ctattgcgac
1364881 actattgtta aacctcctac atcacctgtg gtgagaggaa cagaatggag cgaggctgca
1364941 agatttctta tttcattttt aatacctcca gatccgatcc atccaggacc ggcttgctgt
1365001 aatccctcgg ttcgatatcc ccccatgttc aatatgttaa tgcccaccac caaatggatg
1365061 aatcatcgtg gcacacaggc tcgcgaccat ttcaaaaaga acagatgtat caacactcct
1365121 gaaaatccct gtttgctctt ggacaatcaa cttagcatca atgggcccat caacaggtgt
1365181 gtttgcgctg agcggagaat cccttttcca acgacagatc atttgtccca cagctttaaa
1365241 aagacgcccc acgtttgagg atgcacaaat tgcatgctac cgccccagag gccgaacatt
1365301 aaagaggctt ttgtatgttt gatttgccac cagcgccgct gaacacactt ctttttcagc
1365361 cagataatga tctctttctc agctccacag tgtcctactt agctcactta tctaacttgg
1365421 agggaaaaat aattatttga tgtggtggat gctcctcagc cttctaatgc cgtgccgtcc
1365481 ttgattatta tcctcattat ataatggctc aaggccattt ttcttaagga cgggatttgc
1365541 agggtgcctc tgtgtgccag gattgagcct tagacaaaac atttgttgac ctgaaccaag
1365601 cgcaacggca gcgccgaggg aggaatccac ctgaatatta aatgggtgtt ttgctcaaac
1365661 aaaataacaa caaagagaaa aaggttttca gagggagaca acgaagtcgc tccacattct
1365721 gattcggctc tcgctgtctc aatattgaat aagcaaagag ctattatgat gcgtttgtat

1365781 gcagcacatg atttattact gcaccttctg ttgtaccttt agaagacaac acactattag
1365841 agaataaccc cccccatact ttcaataacc tcaagagaga tccatctaca ggctgtcttc
1365901 ctcactgcgg cggacagcaa aacggcattt tgtcagctcg atacagtcag ctattcagac
1365961 aggacacaag cgtccgccaa gccgttacat cgtatacatc tggatggtgt caagagatac
1366021 gtgtaatatt tcataccaag ggacatgaag aatcccacca agctaggtgt catttgctga
1366081 tggaacgacg ccaaaccatc aaatccgact gacaggaata gaaaaggcct cagtaacaga
1366141 cggacgagca tcaaagctaa tagcctggcg gctttttatc ccgaccacca gtctgattga
1366201 tgatgagatg ctattgatcc gctgcagaca ttcagacttt atttattctg tataccagca
1366261 aagagataaa tctgaaggga aacacacttt gggaagaaaa aaaaccaacc cagtctttca
1366321 ttaaatgcat atgcgctgac ccatggacgc ctcgatgggc taccaattca gctcctccgc
1366381 agactggctc cgaggataaa aattaaagct gtgtcgaatg ttccagtcgg acgactggcg
1366441 gctctctgaa aacaggcttt catgtgctca ggctgacagg cttttgatgt aagtagcaga
1366501 gtttgagaat aggctgcggg gatatcgggc tttagaggag gagagagccg aggtagagat
1366561 gaagagaaag gtgtgagaac atgggagtat aagtgcttat ctacagcatt atagattaaa
1366621 atatatatac atctatttac atcccataat ctgttacagt atgttaattc acgttaatgg
1366681 ccgttcccat gttataacta atgaacctat aaagacgagg ggatgatgaa tagcagctgt
1366741 catcactgtg gtaatatcat ctttttaacc aacagctaag ggttactaac gaattaagga
1366801 actatgaaat acaggagaag gagtaattac gttaaatatt cgtagaggat cgttacctgg
1366861 gattatttct tccttagctc tcagtgatag atggactgat gttggtcatt tgttattatt
1366921 tgttttaaag cctaatgttc tcccgtttga ctgcatgtag cagtaaaatg cagcagtacc
1366981 atatatcttt ttaatttatt ctttattttc aactggagac tatttataca ctagccaact
1367041 taggcattgc tgtgttcgga ataatattta tgaaaattac ttatgaaaca gcttctgcct
1367101 taggaagcga acgccgaccc ccccaatgct gtcaagtttc tgattattga cggcgttgta
1367161 tagttatcat acaacggcag ctctctgagc atcagaaatc ggattaaccc acttagattt
1367221 actccaatgt ctatgcgtgc gtatacctgt gaatttgatt tattttgttc tttaacgttt
1367281 gcacgagccc cgtctgtact gaagaggaga agaattccag gttaccgccc agcaagaaga
1367341 gaacagacgt tattattaac ctcaccacct acgaatttac tccaaaagaa gtccaattat

1367401 tgaagaagaa acatgtaatc cagggttttg agcgtaaaac agtcttaagt cttgatttaa
1367461 gactaaaagt agaatgagta tatgagatcc acactaacct acaaggttgg acactattaa
1367521 ttcattgggt ctaacttact atattcctaa tcacaagagg aacactggtt tgcatttgat
1367581 cttccatttc cagaggataa ttttccatcc acaggtggga ggctgcttaa aatctccagt
1367641 tagaggtcaa atcccgctcc cagcgaacat gtagtacctc atctgattat gaagctccac
1367701 aatgaaataa ttgaatgaaa atgccaacag gaagccaagt cagagaccgg ccagctgcag
1367761 actttgagca atcaagtcta aaagcattac tacaactgtt tctgatttat ttatttcctg
1367821 gaggtcattt catatgagct tccacaggag gccaaaaatg aaaacacatc acaaaacttc
1367881 tgtttgttgt tattttttga aaataatctt acccacccat tagtgtggga tgggcaggaa
1367941 cgcttttata tcgtccgccc tctgcctgcg ctccatccat ctctcctgga caaaatcagg
1368001 gagtaaaata tacctgtcga atttggaatt ttcatttccc tgatcctggc atcggtaagt
1368061 gacaggaaca ggattgggag agcagcatcc tgcttctctg actggagagc aacccacatg
1368121 atgctgtcat gttctttgat aactaagatt aatgccagcc aagacactgc cgtcttcctt
1368181 accctggtgt taccacttca ttctgggcca tgtggcttcg cctcccggaa agaaataatc
1368241 ctgtatggag agttaaataa acctgagcaa aacttttgtg aagagagata ttttgagaaa
1368301 aaaaaacttt aaggctcatt tttgtgagtc actctcttaa ttaggagttg aggggtgcta
1368361 ctgcacattg taccgacttg gcatcaattt acataaatta acttccattt ctgctctttc
1368421 catcttttac acgataacat gtctgggtga gcaaatgtca caaattcact tatttgttta
1368481 agctattcgt ggactactgc agcgtgcaat gctgcccaac atgtatgccc gtggtactgc
1368541 agcaggaagt tgcaccgagc tgtgcatcag ccaaaactaa atccaacgct tttaaccagc
1368601 ctcttcagta aagtttgccc atgtagaact ttttgtggcg tttaaacaat taatgagatg
1368661 aagctaccaa gcttcagcct ctgaaggaca aatgagtaaa tacaggataa atgggggaat
1368721 gcaaaagaaa agaggcattg agtgctgatt acctccactg atctggattg tggagacgca
1368781 ctgattccat tagctttacc cttgaatctg aaacaaaaca caaacacacc gttcacgcgg
1368841 gtgacatatt gttgcaacat ttcggaacaa agccggcgcg tcctcaggtc cacctcggaa
1368901 aaaaagagct agggcagcgt accctgtgaa attaattaaa gtgcgctcaa ggttttttga
1368961 ctttgaaatc tgacaccctc tcagcattct ttttagctta cctcacaaga aaggaatttc

1369021 gtaatgccct tcggttgaaa gtgcgctctc agaacagcca aacccattta
tgctttcttt
1369081 attctcagtt tatgttttgc cactctgaaa ttgcagcgta aacatcctgt
aatatcatct
1369141 tgctgcaggt tgcaagatgc tgaaaaagct gacatacaga aaacacagag
catcattagg
1369201 attgatttcc caccacctga cacgttagtc gcacattcgt cgcccatttc
tggtcctagg
1369261 cggcctcttt ttaaccatat tatcagcttc catgtcatgc tagtgacttt
tttttgtaca
1369321 tgttttcatg tacgaattaa ttctctttta attatttatg gcttttattc
tttcttcctc
1369381 cctacctgtg attagcaatg tctgaagttt tgttaactct gttgtctttg
tgctgctctg
1369441 ccgaatctgg tgagctcatc ttcaaaacat aatccaacat tcaactctgc
acacataaaa
1369501 cagtcataag ataaagaatt gtgcagtaat tactgcaaaa aagcagttgg
aagtaaggcc
1369561 gccaggatta aaggaccaca ttgctactga ataatgcaaa accactaaaa
aagggaatca
1369621 cagattaaaa aagaaggaac aaaaatcttg gtattaatcg gctgtaggtt
ttccattgtc
1369681 tggagcatca ttcctaaggg ttaattgaag atttttttta aggatgcctt
ggaagagact
1369741 gctttaaagg acaataaact gaaaagagga atcatttatt gaagaggcca
tcaaaggaat
1369801 ttggcgctaa ataaccaaaa atatgactgg cactgacatc ttttcatatg
gataaaataa
1369861 taaaaccatt cgtggaaaag gaataaagat gttaaagata gaagcagaac
acaaataaag
1369921 tgtgttttct gaaacttgca ccaaagctaa atggatcccg tccttcgccc
aataacgaac
1369981 cacctaatca accaagtttg agctggtctg gaggtaggaa caatattgat
tattgcagct
1370041 gcagccatgg gacgttggag tgagtgtaaa gacgagtgga gtcagttcac
acatccccta
1370101 ataacaattt agatgaagtg ccgcagctcg acgttgcctt gtttgaatag
aaattgatgt
1370161 gagctgaagg gtgatcacta tgctggagga acactcagag atgggagaag
aattccgaag
1370221 gatgccctga ttaattgttc taaatgtccg tgcatgcagc gacggtccga
gttagttaat
1370281 ctggagacag agagggatgg aacatcgcaa taaatggatg cacaccggtg
gtctcggctc
1370341 agcggcaacg tttcctttcc atttccattt ttattgctgt ccattccagt
tcatttcatg
1370401 tgctcagcaa cgtcatgcct aaactaattt gtcggcagct gtttctcatt
ctaaacattt
1370461 ccattagcgg ccgaacaaat atgaaatatt gctcagactg ttacgagctg
ggattttgct
1370521 tcctgtcttg acccgggaac attgttgttg ttgttgaagt tatttaacgg
gtcattcctc
1370581 atctttccat cctggttcat gcaaatttgg gctggggaga ttgttctgac
acgagaggtg

1370641 atatcaaagt tatcaaaagt gcccatttgt ccttggaaat aatgacacca cggagctaga
1370701 gctcaaaaac aatcatagca aactaagtta agaggcggat gcgtgtgcgt gtgtgttggg
1370761 ggtgggggcg gggggtagcg ctcatgtgaa gtgatgaaga tcggtcctga aacagctttt
1370821 ttaatcagga aattagcagt gcagcggagg gttgaagaaa accgaaagcg tgtatccttc
1370881 cctgtggctg agaggggaaa cgagatgtga ggaggggggg ctgggctatg ttggggaggg
1370941 gggggggggg ggtgatgggg gtccgtcctg ccgtcagacc gtgctgtaaa tgtcctgctg
1371001 gtcagtcacg tctgatcggt gcatcaatta cgacatccag tcaccgatga cttgggaggg
1371061 ggggtaagag cagcttcctg tgaggcatga agacatggag gtgagcattt atgaagggaa
1371121 caaacgggca catgcaaaca cgtgttttca cccgtcctgt ggaaatacat gacctgtgag
1371181 aatgcacttc cctgtcacga gtgcagccat accatatgca tggatacaca cagtaattgc
1371241 acacctgaag tgctcccccc cccacacaca cacagacaca cagacacaca cacacctcct
1371301 ttaactgtca actttccatt actgtacatg catttgacac acacgcaccc tcatcggaag
1371361 gctgaccaat cagaacattt tacagaaaaa aaacagagga tgtacatgaa aacaacacac
1371421 acacacacac gcacacacac acacacacac acacacacac acacacacac actcaaaacc
1371481 caggaggaga ccctgtcccc tcatcatggc ccctgtcaga cgtttcttgt ccccaggccc
1371541 tgcgcggctc ccatgagtcg gttgccaggc aacggaggct gcattaacat gcgcgggaga
1371601 gagggagcag gggatacctc ttgcccgtgg agtggtacga cagcatcagt gtgaggcgac
1371661 gggtccgctg gcagcgtctc ccccaccttc ctctccgtct gtctctctct tccagccatc
1371721 cagcgctgcc gctcagttag acggctcagc cgctggggcg gaggagcgga tatgttgggt
1371781 tgctgattgc gtggagcatt caggggcttc cacctcatca gccgaggtag tttttttttc
1371841 tttctttttt tgaacattga aaagacagga gagcagcaaa atgcgtctca ctgctgagga
1371901 gtatgaaagt ttggcagagg agatgagctt ttaatcgggg tcaatagggt ctcgccacag
1371961 aagaaaaggt caaaatcttg ttgaagtctg tttttttggg gagggggag cagatttgat
1372021 gaaaaggcga gggaaatgcg cacatgagac agctttgtta ccatcccgcc cccatctgcc
1372081 catcaactga ttccattctt caacttgaca tcatcccctc cacgcagacg agcgggagaa
1372141 gaaaggagcg agggggagaa gaagcagagg tggggaggcg agcaaaacag aggcagaggc
1372201 gccaagcgga ggcggctctc gcaaggctgc cggggagaaa gtgaaggcag tcagaacttg

1372261 gctacgagtg agatgaaatt cagggaggag gcgcaggagc gctgcgaggt accacacgtt
1372321 gaactggaaa cgcaccacca gcaagacgag ccgactgtct ctggatctcg cgcgcctcca
1372381 cggctgaagg atgagagggg ccgacttttg ccttagttgc cggggatgtg ggggcacgag
1372441 cgacaggcgt ccacctccag catgtccaat gctatattac tcctgatgct ctgcaaggta
1372501 ggtcgcaacc gcctccctca ccttcatgct cagcgccaaa acaagcggga cctggtcgac
1372561 cctggcgccg gtgggtgatg gtggatgatt ccagaccttt tggagagaca cttttgtagg
1372621 cttcagtgaa agattttagc aattgcggtg gttcgttgtc cagaaaatcc agtcggagtt
1372681 cctctgacat tggtgacaag tttaaaagct tcttatgtaa cagcgggacg gtctgaaatt
1372741 aaaggctcgt taagcattaa tggactcatt ttcggtgaca gagtaaaatg actttaagct
1372801 aattgtattt tttgaaacgg tttatttata gacaatccta tatttctgtc ttctaaatcc
1372861 tctgtaagac accccccccc tccctgtaat tcatccctct cattcacaga acaataagat
1372921 gtggaaatgg gtcacagcaa ctattggcag agtcgctctt tcaggttctc cggcttgatt
1372981 tgaaaactcc tcctctcctt ttcttccctt ttctttctct ccatacgttt tgttacgccg
1373041 ctcaaaacac ggttgagaga cgtcctgatc ctggtcaccg tgtccacagg cacactcact
1373101 gtcacacaca cttggcccac actgggaaga ggttgggaag cggctcgctt taattccatt
1373161 ttataacaac gccaaaagcg actctgcctg tcagaccctg ttagcatctg gactcgtctg
1373221 acatgttcag gatcattcaa gtggcaaaag gttttactct ctagtgatct ggtccctgga
1373281 acgttagctt ccgtaaagca tatccggatc ctcacgtccc aggtgtggcg ctcccctttc
1373341 agcacaatac agaaaataac ttcctatctg agtctcagga tcagtcgctg aagttaagac
1373401 attaaagtgt cggaatcggt gaaaaggagt tcgtgagtga caccaggaat caattgggag
1373461 ataaaggagt cctcagtatc ttaggacagc acacagtggc taatcaataa cagccggaaa
1373521 gaacgagctg ataatcctgc tatttgacat aaatgagctc tggtgtaagc aggagttcct
1373581 tttaatctgg gattgcatga acctggaaac ccggctctcc atcctggatg taaagcaaac
1373641 agggctgtga tgatgtcaaa tggcatggat gccacatgac cccccccccc ccccaacaaa
1373701 agaccgccaa ataaaaaaac cgctccctca ccactactag caccagctct ccacatcagt
1373761 gtgtgtgtat gtgtgtgtgt gtgtgtgtgt gtgtgtgtgt gggggggggg gggggtcatt
1373821 aagttagtga atacatgaat gtaaatgtgc tcaattacaa ataatgcatt taatatcctg

1373881 cttaagacga agatatatgt gacattgtta gactgttcat tatgattcaa gaagcatttt
1373941 attgttgctg ctcctcaagg cggagctggg tttaattact ttatatacat tttattatgt
1374001 tcttcattta caagataaat ctcacgccgt gtgtatcttg tgcaaaacag tacacgagct
1374061 ggtttttaaa tacgcaaaaa gcttttatgt gttagccctg ccagacgtta gcagggcgtg
1374121 gaagtttgaa tatcggggca ctaataaagg tgtctgggat gtctggttgg gtgacgatgc
1374181 gcggaatgtc acatacgaac ctcgttggaa catcttttga atttaacgat ataaaaaaca
1374241 aagcaaaaaa tgcctgcgtg attgatggca gatctcccgc agttgctaaa atttaccaca
1374301 gttccctcag tcgacattta ctgcagcgct gcccttgtgt tgaagtcatt aaaatgacat
1374361 ctcattgtaa cgtcctccct cagtttctcc ggatctcctt gagattctcc ctctctgtca
1374421 tccgtctgca cagaacaaca gctttggtgt aacgattcct ggaaacggct cccaacgatt
1374481 ctcctgcttt cgcaaaaaca caacacaaac agaaagaaag cagagaaaac tgccacaaat
1374541 ctccttcaac acacgcacac atgcgtccag ggttaaaggg gcagttcacc caactataac
1374601 aactttattg atatttctgt tgaactctcc acaaaattaa actcacacac acattcttgt
1374661 actaatgtat ttctgaggac tttcatggat gagccccgac agtaactatc acaaaaatat
1374721 tcctaaatat aatcctaatc tcaatctaaa cccaatttct aagattaacc ttaaaacgga
1374781 gtatggatcc tcaaagagac ctaggcagtt gtgaggccaa gcaaaaatac aatacaatac
1374841 atctataaga acactcacaa ataaataaag ctgctaaact actcaaggga gctcacccca
1374901 gagcccataa acctgacatc agcaagaact gcatgagagc tggtgcagct tcccacagat
1374961 ctcgccaaaa tgcgagcata gctcgaggct agtagattaa cagtggacat ttgggctgaa
1375021 tataggctct tttgttgttt tgtgttgaat ctgattgtta tacttccctt gatggttgtt
1375081 ttgcctctga gaaactgccc agcttttatt caagtagtga agtttgggat gtttttgatg
1375141 ccttgagcga attcccgttt tttggatgaa agagttcctt taggctcatg tccttttttct
1375201 aatcatgtcc atttcaaatc cacaaaccca aagtacgaag ctcgctctgt ttttgtctgg
1375261 ttagaagggt tatttttggt atccattagc ttacagactt tctgctgcta gtcagtgtat
1375321 aagacccagt gacctgtaaa aaggcgtgca tttctccaga gagttgttta gatgccaagt
1375381 tgcacaagct gctgcgcctg ctggaggcag gcgactagtt tttaatcccc cccccccac
1375441 cctgagcgaa acaaatcaat agatgcacac actgttgagc aaagtctggt gccggagttt

1375501 cagctgtctt tgccgtgttt tcctcttctg agcagtgcgt caatcaaccc atcagaacgg
1375561 gggttttgaa gtgtagcagc aaatgttcag gctttaaatt tgacgagtct gcaaaaatgt
1375621 ctttttggcc caaatgaacg tcgcatcaac tcagtttttt ccgtaattta aaaggaaaaa
1375681 gcaattaaag cgcttcattt cttcaaagag agagatgttt ttcctctccg agtatgagaa
1375741 ggtgtttata tttttgccat gtggaggcgt gtaatttaaa tcagccgcct gcttttgctt
1375801 taataagaaa gacatccaca ggtttacgat ccaaactcca aggaagactg gatttaattg
1375861 gagctgtgga gaaaaagtca tttagatttg atggaaaatc aatcccgtat gcgcagataa
1375921 atcctagcct caagctaaaa ggtgtaacat gaaaggaaca ggaggaaggt ggactcgcgc
1375981 tttaaaatat agaaggcttt tctttctatt ggagggacag tttgtccaga aatggagggt
1376041 ttgggaggtg gcggcagacg tgaagctccg tcgatgcagc gtgttgatgt ttatgttcaa
1376101 tttgaacacc gtggcgccac tttacagcag agctgtgagg tttggctgca gccctcaata
1376161 cgataaagga atatcgacac aaacagaaat gaaatccatt cacattttgc agcaactgtt
1376221 taaaacacaa acgagcaaaa caaatacctg aaaaaaaaga tagaaaaagc cagcaaatta
1376281 actgtgcttc tctctcccgc tcttatctgt tggctccaga tcggtttgag acgtaaagtg
1376341 cagacttttc ctgggaaaat ggtcaacact ctccacagtg atgaggaaaa gggaagccaa
1376401 gacaaggaaa gcaaggagga gagcatcctg gccatgctgg gcatcattgg gaccattctc
1376461 aacttgctgg tcatcatctt tgtctacatc tacaccacct tgtgactctt atgcacctgc
1376521 aggttgatcg cagctcattt ggacgtctcc atgggaaccg cgctggaaaa atccagcttg
1376581 actggcggcg gactggaatc aagcctctgg gatggccgcg aggcgcccac tcggcatggc
1376641 ttcagatggc tgagaccacc agaaactggc gggccgctct cctctgcaca ggtggactca
1376701 tccaggatac gcttatgctc gtaaccccga cacgtgcacg gtgacggaca cttgggaagc
1376761 tgcggcagac ccgcgaagga ctgcacagag tcgtttttca gacggaatta tctcctttac
1376821 agacagcgta cctgccacac gggtatctta tcagcttttc ccgacgtttc tttgtcagtg
1376881 tacatatttg tatcggattt aaagggacac attcgagtcg tttttttaa tatcgtattt
1376941 ctcagtgttt ttggagaaat agcgccagga tgtgttacaa tttactaaag tctatactgg
1377001 gtgacatttg tgcagttcac atatatttgc tttggtaaat actttataat ttagtctgac
1377061 taaagtacta aaatgcagat tttatgtttt ggttgtactt tagatttact ggtaaacctc

1377121 tacggatctt tgttcatact gtacataatt aagtcaaacc ttgtgcataa tgacaatatg
1377181 ccttaaacct gagcaaatgg attgataatg attattatta ttactttgtg aagtatttca
1377241 gtcgttgtgc ataatgtttg atgcttttga ggagagctca tgtggtggcc agcacgcttt
1377301 tgtctgaaac aaagtgaaaa ctcatttgac agcgttttca ttgaaaccac cctcatcagg
1377361 ttttttaaat tcagtttttt taatccccgt cacgttctgt cccttatttt tctctttgac
1377421 cgaactgaca ccgtttctcg cctcgatgtc ttacatctca tctctcgggg aaaacgagca
1377481 tctagacaac attttacttt acccaggact ggaaacaggg catatttttt gtacttgaag
1377541 gtaagatgta tgtaaaagaa aagcaaggtt aaaaaaacaa acaacaaaaa acattcagac
1377601 tttgagcaga atgtcttcaa acttttggat cagaatcatg tgggagttga atttatgagt
1377661 aacagtccca tattatcaac tcgctaatct tgagcttttt tttgtaaaaa ctttaaaaac
1377721 ctgatgccgt gtccataata aacttccgag gtgcttgtag gttgcatttt gtatctttat
1377781 tcaaacaaga agcgatatca aatagcattt atcgcgcgtt gagctattcc gacggagttt
1377841 gtggagcaat gacctcatca tttcagaaag tgcctgatca aaatgtctca gcctcaccag
1377901 tttgtgacag caaaccagtt tgggtatctc tacaaggccc cactaacaca cagctgaact
1377961 accccgatcc actccactga attccctccg tcaggtgaag tggggattaa aaacgcctgt
1378021 aagcacacgg acaaatcaca cgaatgtatt cttcttacat gctaatcctc cctgctcatg
1378081 ttgctttcgt tcctatctca gtgtcttctc atctttatgt catgctttgt ttttgtgtgc
1378141 tcaatctata tttaaaaccc tattttaatg gtgaaggtga caagtggagt tttttatttt
1378201 tccggcttgc agttcacgaa taaacatatt gacaagttcc atgtgggtgt gcaggactgc
1378261 taatctttat tggcttcgcc tgttttgctc atcaaattgt gttttgtgtc agcatggtga
1378321 attattgtgg ggagagaata agagcaggga ctgctctctc cactgtagcg gaatctgcaa
1378381 atatctatat tctcctcctc tcgacagctg agtgagatta caccagagcc ccggagaaga
1378441 tggatgaagc aaggaaacct taaggagtcc cagccattgg atcctctgag ccacatttct
1378501 taatatgact gcaaatcaaa ctgaaatatg tagcggacaa agctcaaagc tggcaggaca
1378561 aagcaaacca aatcacccta aacatattta tttctgtatc catcttcccc aggatgggat
1378621 cttttgggcc atgtgtgtgt caaaataatt tcccgctctg ttgtggctgc tgtgccactg
1378681 gtgccgtgct ggaataatcc tgttggagga ggaaaaatga gactaaatcg agcttgcctc

1378741 cctttttcc tctcgcttaa atacaataga ttaggcaacg cagcctaagg aacctcagct
1378801 gcgtgatttc acacgcgccc taaagcttgc tgcaagctcc cgttttcacc tggatccagg
1378861 gctcatccct caaaccattc cctcttttga tgctccacaa ctgaacaatg tgcaggaggt
1378921 agtagatgga tgattcaact gtgcggatgt tccgtctcgt cctattcctc ctaatggttt
1378981 tcacagaaaa gctgcacgag gctattaagg cccccacccc ccaccccca ccacgtcaac
1379041 caccgccact cctggctggc gatgtcaggc cgccgctgtt gttattcaac ggcaattcaa
1379101 cctgtggcag cggcgcatgt taaaatttaa tgctacgtta caaagagcgg tttccaggag
1379161 gacacagacg aaagtcacgg cagcggatct gtttcacgct cgatcaaata ttaacgtaaa
1379221 gacaattcac atctactgga aaatagatca tctgttccca tctgtagttt tttccctttt
1379281 cttacagaac agtgcgttga gaggaagact gagaaagtgg gcagtaagag cttttatatc
1379341 acctaaacac aatacgtaca gattaatgat cctgggttga tccagcaatg cttaaattag
1379401 aaaatgaaaa ccagttaaaa ttctttaatc gcatgaatat ttgctgactt tgctgtttga
1379461 aaaagaacca accaaagctg ctgcaatctc tcaggtagta aacacagaat agccatttag
1379521 ctttttctcc ctcatataat ctacactgtt ttctcttcct gagcacaaat gggatcatct
1379581 tcaagcaacc ctcctcttca tcaggggtca gaaatcagtc atttgtcttc aaatccttga
1379641 agacaattaa ggggcaggaa aacctctcgg tcctgacctg ggcaggttaa agttctgttg
1379701 aggtcaccga tgtcacggct tcaatgagtc ccggctcagt cggagatttg agaggatttt
1379761 cccaggacac gtgtcctgta tggatttcct gtcacaaagg ttgcacagat ccagcctttc
1379821 ttcacgtcta tccatgcaga gaaataaatc ctgtgatcaa aatccaggaa aaggcgtgaa
1379881 aggcggaaca aatcctgata caaatgtaaa acttcagtgt gaaagcgtca gcttcagctg
1379941 cgcagattaa acaatctctt ttttctgtct ttttggggaa atgtgtggtt gtattttctt
1380001 gcatttcctg atttaaagag gattttgnnn nnnnnnnnnn nnnnnnnnnn nnnnnnnnnn
1380061 nnnnnnnnnn nnnnnnngga aagacattaa atcagtaatt tcagcctgga ggaggtggtg
1380121 aggagcagaa actggtggta gttatggaca acgtctgtcg cggcgttgaa aaaaaaacac
1380181 tttagaagct caagagcagc tttactgaca ctccagctac tgttatcaaa ggacggcata
1380241 gaaaaccact ctgttgccat cagactctga aacacacctc cgctcagcaa acctgtcatc
1380301 accaaaacta gctcgaaagc ccagtgaatc catccatcca tcatccatcc atccatccat

1380361 ccatccatcc atcatccatc catccatcca tccatcatcc atccatccat
ccacccaccc
1380421 acccatccat ccatccatcc atcagccatc catccatcca tccatcatcc
atccatccat
1380481 ccatcatcca tccatccatc atccatccat ccatcatcca tccacccatc
catcatccat
1380541 catccatcca tccatccatc catccatcca tccatccatc catcatccat
ccatccatcc
1380601 atccatcatc catccatcca tcatccatcc atccatcatc catccatcca
tcatccatcc
1380661 atcatccatc cacccatcca tcatccatcc atccatcatc catccatcat
ccatccatcc
1380721 atccatctat catccatcca tccatccatc catccatcta gccatccatc
catccatcca
1380781 tccacccacc tatcatccat ccatccatcc atccatccat ctatccatcc
atccatccat
1380841 ccatcatcca tccatccatc atgcatctac ccatccatcc atccatccat
catccatcca
1380901 tccatccatc catccatcca tccatcatcc atcatcatcc atccatccat
ccatctcatc
1380961 catctatcca tccatccatc cacccatcca ccacccatca tccatccatc
catccatcat
1381021 gcatctatca tccatccatc catccatcat ccatccatcc atcatgcatc
tatcatccat
1381081 ccatccatcc atcatccatc catcatccat ccatccatcc atccatcatg
catctatcat
1381141 ccatccatcc atccatcatc catccatcca tccatccatc catccaccca
cccatcaccc
1381201 acccacccat catccatcca tccatccatc catccatcca tccatccatc
catccatcca
1381261 tccatccacc cacccaccca tccatccatc catccaccca accatccatc
catccatcca
1381321 tcaacacacc ccacccaccc acccacccac ccacccatcc atccatccat
catccatcca
1381381 tccatccacc cacctaccca tccatccatc catccatcca tccatccatc
catccatcca
1381441 tccatccatc aacacaccca cccacccacc catccatcat ccatccatcc
acccatccac
1381501 ccatccatcc atccgaaggt tttattggat aacagttctc ttgagatgat
gctgggggac
1381561 tcagcttcca gtatcagtct gggctggatg gccagccggg tgcataaaag
ggaaaacttc
1381621 agccaaggca tgactgaaaa cctcttttgc ctcattagca tttgcactcc
aacatttaac
1381681 agcaagagct gctggacaga aaccgtaaga tggatctgat ctgccgcgaa
atcgatgctc
1381741 actaactagc tcgggcgtag ctgataagct cagcagaatg gggttttcgc
tcctactgta
1381801 ctcctcactt tgagcccgat ctgtacccgg aggaggggaa tggatggctc
ctcggccaaa
1381861 tgattaaagc ccggcagaag tgaggtttaa gcaggggaaa aaagaaaaaa
agtgccgacc
1381921 agacgcagac gtatgcaggg agcgtaacac ctcagtgcac ttcaatcagt
ccgctgcaca

1381981 tgcggtgcac tgttggaatg cacattcatt cattcccgct gtctggtgca cctaacccga
1382041 gaatatctga tgtttttatg aattttacat tgtcctcact cgtgttagag tcacgacttg
1382101 ttatatcccc aaacacaagt tctgaaagta tgagtagttg ctatttcctg attctggaga
1382161 agctctggtc tgatgagatt gaggatgaag agatgtggct caattcaact caattattag
1382221 tatgatttta gatgaatggt ttggaattta aatagactaa gtgctgcaag aaaacacagt
1382281 tggcatgaca aaattatttc aaaccacaga aaagcagaag agcagcacat gttgccaggc
1382341 ctattttgtt ataagatagc aaatttctta ccagtacccc aagttctgca tatctcattc
1382401 ctcatttcct taatgcatct gcaaaaacat gcagggttta atgacataaa gaagcagaat
1382461 attgatcagt tacaatgttc cacattgttt ttgtgtttta tctttagggt tttccagacc
1382521 ttgcagcccc agagaggaac accagtccag tccagtccag ctctggcagg cagacacatt
1382581 ttaatggggt cacaatcatc gctggctctg ccagcttgtt ggaccggagc accttcctgt
1382641 ctcaggctgg aataaaaagg tgcagctgct ttgttgaaca ttaaattgtg ttgtaaggac
1382701 agacgtagga tacagaggca gacacaacaa gcatgcagat gactgccaag gaacagacga
1382761 gatgtcttca tttaatctgc cagggtcatt taacgccgcc aaaagatagg tggagaggga
1382821 atactgtgca aacactgccc tctgtcggtc aaacacgggc ggttcaaggc atttatctgc
1382881 accgacagat tccaaggacg tctttattaa atatgtttgc aataatcctt caaggtctgt
1382941 gcagcttaat caatgcagtg atagaatcaa taggcataag cagcacagag tagctaatca
1383001 atgtggcggt attgattgtg tgcaaagaca aacaatttgc tgtattgatt tgtacattgt
1383061 ttagtattta agcatgacgt gtcactgcag catgataagc aattagggtg attccacgca
1383121 gcagttgacc tcatcgaaat cagtcagctg ctgacatctg gctgacaccg gagttaacaa
1383181 caaacagaag agagcacaga agaggagacg caatgcattg tttggtcttc attacattag
1383241 gtgcctaacc tcattgattc atggctttac aagtgtgacg ccactgctga tctgtattca
1383301 gtccgctgca aatgacgctc actccacttg gcgctgacgt cgagtccatt gatattcaac
1383361 tcggtgggtg ttgaggttgt actctcctca gaacattaaa aaaataaaaa aggaacaaaa
1383421 accctcccca agatgggggg gggggggtg ttgaaaaatg atacacacgt gctgtgctgg
1383481 cacctgagca tcgctattca tgataggtgg taaacaacat tatcattgac ccttccgtga
1383541 atacacagcg ctgaagagag cgctcttgca cgtcattgct ggggatcatg tcaagacaca

1383601 acaaaaggta aacaaggttg tttttattcc acaggtgaca cgacaacagc gagacagagg
1383661 agagtgtgat tacagcctgc acaaacacat ttctggggag gggggactgg gcttggaagt
1383721 ggagggttct cgtttcaagc cctggtgcag acaaaagtgg atggtgttct gctagtaggg
1383781 gaaggtgcca gagcactgct gaagtaccct tgagcaacgt accaaaccca caaatgttca
1383841 aatagggccc tgtgatgagc tggcgaccca ccctctccat gagcccaaaa gggaaaaagt
1383901 ggttaagatg aaagatgatg tataagtttt tctggaaatc atgactggct caaacaaagc
1383961 taggctaagc taatgggtta gaaccgagcc atcaaacatt cattagcctg atttacagtt
1384021 caaatgggtt gtaaatcagc actgtctgtg aaacacctgg ccagcttctg cctggacctc
1384081 atgcccttgt ggtcctgcca ttttcccttt tgaaattcag ctcaaacaca attccagctt
1384141 gaaataaagc atgttcctaa ttttttcccc cgttgacttc actggctaca ttagaggaga
1384201 caattgttcc attagcctgg gccggatatg tgtcacctga cgaggattaa cggctaatgt
1384261 atcgcgtctc cttgtaaaca tgaataatgc atggtcatta attacacccg ctgcatgagg
1384321 agtcagccca ggaaaaataa cactcctgca caacatctac ccattcattc accgggacaa
1384381 aatgttcttt tgatcgtgac gacgatgatc gatagcgttt ttgggccgct gacatatgaa
1384441 tacatctgtt tcggccattt accaaacctt tgggagtcca tgaacatcgc ggtggtttga
1384501 tttttctttt tgctctttga cgtcttaatc ccgagctctt caccccgcca gtcgagccgt
1384561 ttatcggtct gcggtggtta tctttatctc tttcaacagt cctgtttcag cttattcagc
1384621 tgctccccac gcccgctcgc agcgtgatgt atgttcggct ttaccagcct ggctggcgcc
1384681 tgagtgcgag cagacccctc tccctgtggc cgtctccaga tgatgctggc tactgccgtg
1384741 cacataacgg cttaacgagg cctccagctg gtcgccggtc catctgtcca cggccaaaaa
1384801 caacagtaac actggaagtg ctgcctggtg tctgttgtat ttatgcctca gcgtatgttg
1384861 tgaccttgtt tgatgagaaa gggaagtgat gatcatcatc cactgcgtgt gagggtgcga
1384921 gagggtgctg accttttcat atcgctctct tttaatagac actgacagcc ttgtagaaat
1384981 cacttaccat tcgtctgtgc tactaaatta gaccctccag taaaactaaa cagctatcaa
1385041 atcacccatt cacccactca ctcactcacc cgttaacgat cagaaacggg gcgtcccaca
1385101 caacctttca tgctgacttt gtgtggaatt ggcttttctg tgttttactt tcacaccaaa
1385161 gtgttccaag cattaaagta gtaaaaagtg caccaagcat caaagtcaaa gcgaacctta

1385221 ttgttattca gacctgcttt ataaacagtg gaataaaagg gctataacaa tcattcacca
1385281 tctgcgttat gtggcggagg cttttgtcct ctgcttggag ggcaagcaag tccttctgat
1385341 acgtaaacac gttcgacgtg ctctcaattg taccagtgaa ataataaagc gttttgggct
1385401 tcacgcaact gctgtttctt aaaacaatat tctgtgtttt caaagtgaaa acagaaccta
1385461 tctgacatca tttgggctat tcaagacgtg aacgttgtgc ttctctctac aggttagatg
1385521 ctaaagctga aaacagggtg gagaagagtc aaaatctgaa aaggatcaac acccgaactt
1385581 taaagtttgt ctttggtcaa tattccaggc tcacttgatt agcatgctaa ccgagtgggc
1385641 gatgaggcct ttaacatttc atgagtaaca aaacaatttg catgactttt gtgcaccagt
1385701 gaggcggagt gaggggcacg ggcggcgttt catgcaccac tgccgcaaac atgagaagtg
1385761 catactggaa acaggccgac gaggatgaaa gcgagaagac aagcggacga cacgcagcct
1385821 ctgcctatgt gtgcgcctga catgtcccca ccctgccgca cgtttttgtt ctattaaaga
1385881 tgaagaaaat gagtcggccc caggagaagc tcaaagcttc ccctccttcc ctgtgttttc
1385941 accactgagc aaaaactgga gtaattggtg gggctggtgg aatcacatct cctccctccc
1386001 ccgccactcg cgcacgatga gcggcgggtt gatgaagatg ccctcggatt aataaattag
1386061 cccaccaact tgtaccagaa aacatttaca tctagatgag attacagccc tgacacacaa
1386121 cctcattacc ggcctctgtg ccccccaac cccgtttagc tgtcagccga tcgcgtcaca
1386181 ggcccctaac tcattcaacc aggccgtggc tgcaagaaca atatgtgctg gatctggttg
1386241 attgatgcca taaatcagat ggaggcaatg cagaagagcc accagcagca gattatgaaa
1386301 tcccagttca gctgcagcac taaagacacc ctgaggtccc aataaaaatg attcttgatt
1386361 aggagtgcac agcctctgac ggcgtgtgtg agaagtccct gatccctcaa atctagcatg
1386421 acatcatttt tccgagaagt tttatgacat acaaataaaa tactataagt gctctaaggg
1386481 aaagagatga atggaatcta cttactgtct tactttagaa catattagca tgtttgctgt
1386541 agcgaagacc agatcttaag acccaaggag agaccaaaga ggcactttaa ctgtagcaga
1386601 agacaaaaag accaagcaga atcgttagga atgctgaacc agaaccagat ttacgtcaca
1386661 actgtacctg ctttgaatgg gcacagacag tgaggagaac ccagatttat ccaggagaga
1386721 caacgaggga caggtgaggc tggacaggtg gcagagatca ggacaatcag caaggagaaa
1386781 gacatgcaga ggaaggacaa aagcaaaggt gaaggaaaga gacatgtcac caaacactgc

1386841 agagcaggaa gtatgaagtt ggttttaata tataatccaa aaacaaaaat cccaagtgaa
1386901 acaggatgca gacagtagga gggaggtcac cgatccagaa gtacggctag tttgatgtgt
1386961 cctctgtgtt tgctaccttc tcagcgatta agtaaatccg gcattcccaa cagccgatca
1387021 atgatacagg aataatttaa acattgggtc ccctccatcg gatcgtcaga atggacaata
1387081 acacttgagc tgaactgtcg ctaagcttca aatccacttg taaatacaca agtggaatac
1387141 cacaaaacct tttggaatcc acatggactt tgaagaatct tccaggctgc agttgtcggc
1387201 accgtctctg ctttcctgtt ggtgacagat gccgaggagg ccgcagaacc gctggaggtg
1387261 ctgaagccaa gggtcttctc cattaaaagg atggcattac gttgctgtct tccactcatt
1387321 agtattttcc accttcctcc caacctgcac gggttcaaat gctgcagatc ctgccgctcg
1387381 gtgttaaagg gaactgtgca gtcacacatg gaggtggaaa caacagctgg acaggtgact
1387441 ctatgggcaa atctgttaga gaggaagctt tggaggataa gacctctgtt gatccagaag
1387501 ctcacatcaa acacatttag gtcacagctt gaatccaaag gtgtgtgttt tgttttgttt
1387561 taaaaggaga agcattaaat gcatatattc ctgaatgact taataggaat ccccacccac
1387621 ataagcacat cttgacgggc ttttcatatc ttttctttta aagaatcaca actctaaaac
1387681 gatttcaggt ggcaaaaatt gatggggatg gttgaccctt gtacaattcc atgtcaagga
1387741 cactgtaccg agagtggtca tatcagcacc tatcacatct cgcctcttcc aggtgttcaa
1387801 tagacgcctt gagacgtaat gaaaacccca tatatcactc agcacaaagc cgtaaccttc
1387861 tgcttccccc gcgcaccctt gcaggcagca attcggaagt tgaaatccag agaggaggaa
1387921 gaggagcggc cttggtaatg agaaatcaaa gggtaagcag ggggctgtga cagcacagcg
1387981 ggacagcgtg gacgcaggtt ggcaggtaca cgttaaagct gtgtggaaac ggccgccctt
1388041 tacggcgagg gcgaggcagc gaggatggag gagcactgtc cagaacctca tgggtgcttt
1388101 ttgattgaca ggccgtgtta taggtcttaa actagagagg ataagtgtta ctccatggga
1388161 tgaactggct gtcgctctgg tggacgctgc cagaaaaagc cagcatcagg aaaaaaaaat
1388221 ctatcagtgt gtttctcgat agttagaaac agaaccatga agctggaaca ggctcacttc
1388281 attctttcta gcagaaggtg aagcacttta atgaacatgt atgcatattt tgatcctttt
1388341 cttaggtgat aaccctgaag agaacaaaac aaatatacta tactaatcct gagtaatata
1388401 aaagtctgaa cttcagggga tcttcatgga acggctacct aatcgatgtg gaagagttcc

1388461 ctgatgaccg ggagccgtgt tgtcggctcc tgttagcatc cgccaaagca
aacagtgcct
1388521 cgctgaggca tcaggcctgc gccatgtaca ttttggagtc ttatttagat
cttataagct
1388581 cctatgaggt gcatttgggt cctcagctgc agtgatgatc accattctca
gtgttccagt
1388641 aggttccttc tatcacaagg agttcaagga atagttaaaa aactaatctt
aatctttggt
1388701 caccagttta tatgcacata tagaaatagt tcaggtgtca ggaggatact
gatgataagg
1388761 actcctgaca tctgcagttt ctatgtcaga tcttcgataa aatgatcttg
aagcggtttc
1388821 atcttcccca cataatcaca caaattgatg ctaaaatgga tttcgtggaa
aaaaaaaaga
1388881 aaggcggacg acatttactg tgggagatta gatcagaggt agaaatgacc
catcgtcctt
1388941 ggctgcctgt tgatgctcag tgtgaccttt aacccgcctc agtaatgaca
ctgaattcca
1389001 catttcattc caggccaaga gggaagcttt tacccatgat gttagcggcc
cgagcctttc
1389061 agctgttcag ctggtagaga aagaagacag atctaaggtc agtgacgccc
caaacacctg
1389121 agccagctct gcttctctgg gatgtccagt gtttaattcc tcatgcacac
aaagatcagt
1389181 ccagcctgtc ctgtcctgca agcatgtttc aacagtccag cctgtggacg
tgagtgcagg
1389241 taaggcacat cagcagacgc ttgcttctta aattaatttt gatccacttc
catttggata
1389301 gatcaccaca aaggaacaaa gcaatcagtg ccaaatagtg tgccacgaaa
gacaagagga
1389361 cacctgaaag tcaattttat ttatagagct ggatggtgct gagctgagcc
tcaggctaag
1389421 atgaatattc caggcagaaa tatcctaaat taatcaggcg atcatggaag
tctttttttt
1389481 ttctaaactg cagttggtga aagcaattga ggagaaccgc caggaaaatg
acaacgtgta
1389541 ccgtaagtga gtgttgctgt tccctcaggg taacgatgat cacatgcatt
tagagtttca
1389601 cccaatattg attcatgggg aaagaaggtt gcatggagac ctgtgtggcg
ccggtgtgtt
1389661 gtatacattt gatgagccgt tcatttattt cctgccctgg aggaaaactg
ttcctcgcct
1389721 ttaagtcatt ttgaacaatt tggtttaggc ggctcgagca gggaaactta
acacccacac
1389781 aggaatcaag caaagctata actctcgtct gtcgctgtgt tgctgaaggt
gggtggtttc
1389841 agttatgggg gcaacatata tgagttgaag gtaaataatg aaggtgtctg
tttgccatga
1389901 ggcaggatga ttctctgtct gcagctcaca cggagagatg tgaggggacg
gtgagcatat
1389961 gtcatcatgt caacatcaag atatgatgac agatctgatc ctcatttgca
cacttttgtt
1390021 caggccctct ctgcaaatgc tgcatgggaa gagggtaaaa atggagacta
acacggaaca

1390081 aatgaggtgt ctggaacggt aaaagcttta aattaaatga atgaacacat ttccatggat
1390141 gcgctcaaaa ccgcacatta gacggtgtaa tggaggcagt ttgtagccag acagtttctc
1390201 attggcgtct ttctctgacc tttttagttt atgtgattta aagcaattcc tgacagctga
1390261 aaattccaca ttttcaaaca aatcaagagt gtgtgtgggt gagtctgtca catgtcgccc
1390321 ccccagctgt caaaggccat gattatatag caccagtgag accttgatac tgctgccgca
1390381 gtaaaagtag cgtttgatgc aataagagcc gaaggatgag cagtcagggg tcacattgtg
1390441 ctgctatctc ctccaatcac acacacacac acacacacac acacacacac acacacacac
1390501 acacacacac acacacacac acttgtagag ccaccttttt atggcctttt ccattcattt
1390561 ttcagttaac tgtaaaatca aaccaaaacc ttacttattt ttaccaaatc ttaaacttaa
1390621 tattacaatg atattaagga acgtaaggat ttgctctgca gctttactcc ccccaacaca
1390681 gttatacctt ccaaatcccg aaggaacccc acattgaaaa caagaaggca aataacaatt
1390741 ttgtaccagc acattatcca aagactataa acattgaacc acactgtaag ataaagttag
1390801 gttacataaa cgctacggta ttcctctcag gcattgtgga tgtcgaggat tacttcagga
1390861 acgagcgatg taaaagagag aaactctttt cttctcatca gtttgagatt gacagtttgg
1390921 tcttttgatg tgactggtaa tcttccctgt gctggaaagg ttgcctcaca gcatgaaagc
1390981 gcaacaaagc agccgtggca cccaaggccc tcggctcaaa gtgtcaccga gcctcatttt
1391041 ctctcagaca gacaggagct tgcaggataa ctgcaagnnn nnnnnnnnnn nnnnnnnnnn
1391101 nnnnnnnnnn nnnnnnnnnn nnnnnnnnnn nnnnnnnnnn nnnnnnnnnn nnnnnnnnnn
1391161 nnnnnnnnnn nnnnnnnnnn nnnnnnnnnn nnnnnnnnnn nnnnnnnnnn nnnnnnnnnn
1391221 nnnnnnnnnn nnnnnnnnnn nnnnnnnnnn nnnnnttgat ttgattccat tctagtgtgg
1391281 ttcaataatt gcgcaattta ctctttacct cttttattca aaccctggcc tttaaaggga
1391341 aattagattt acagtttcag gaggaaggag aacaggctgg ctatgctggc taggaaggag
1391401 gatcatcttt ctctctggcc tctagtggga ttgtttcatt tgcttttatt tgtaggaaat
1391461 aaagattatg gcgcaaagat tagtttcgat cagagctctc gtcaccaatg ggattgctct
1391521 aagcccgact cttccatcat tctctccttg accttttcct ggaccttctt tctggtgctc
1391581 ctgtcagctg gtgtttgcta attccatcac aagtgccttt tcttgcttca tgtcaaccac
1391641 aacgatgtct ggtttgcttg agggcatagc tgccggtcaa tctggagctt cctgtagccc

1391701 atctttccga tgtattcacg gtccttttt tatcctgaat tgttggtctc
atcttcgtac
1391761 atcaccaagt gagtgacttt tctccaggca gtgccagtgt ctgcctgtgt
tggtgtctct
1391821 acatcagtgg tgcccatctc cccctgaggc cagcttgctt ttgcagcagg
gctacatctt
1391881 gatgcctcgg gtctaatttc caaccacgca gatggctttt acggatttac
ctcctctacc
1391941 ggttttcttc tactgttgac ctcattgctt caacatgatt gttgtcacta
gtctgtgaca
1392001 gacccagggc cactgcatga ggtttccctt ttctgcctcc tggttggccc
accacctcag
1392061 tcttgaccaa cgttcagata aactttacat gaggtgacgt gactttacat
ctgaaaatat
1392121 atgcttttat cgcatgagat gtttacgaac acaccattaa acagcaatgg
caaaacatca
1392181 gatgagacca ggcattaggg agattgttgg tatgtcaaaa ctgtaagatg
aatgtggttt
1392241 attagacacg gttacaggga tatgtatgcc atgaagccat tattagctgt
atgacttgat
1392301 ttattctgtg caacgcttct ttctttatcc actcagtgtg tagctcgctg
tacctgagca
1392361 aaccaggagg tacccaagtt caaccgtgtc cttctgtgga aatgaaacat
aatttatgta
1392421 tctgccccat gactcggatc aactccaaac tttaatggat gctcccttgg
cttatgatac
1392481 cccttaccat caggtttcat taatatcagg aacatttttt taagtcccca
tactccaaaa
1392541 caatctggtc ttttaatgca tcccactacc ctaaccgcca caactaaacc
catccctaac
1392601 ctaaaactaa ttattgccat tgtcccaaaa cagtcctatt ggagtagtga
ggatgaaaga
1392661 ttaacatgtt ggaagaggtg aaagtattta caatagcaaa aatgatccaa
atgtgatgtg
1392721 aatgctgaat atctccctta atattgcatg cacacatgtg aacagagaga
gagagagaga
1392781 gaatgagcaa aatggtgaca gtgtgtttaa accacatctg actggtaaat
aacactggtt
1392841 aaaatatcca aataggcaat tttggactgc gatatggttg aaatgcagta
atttacctgc
1392901 agcagatgtg ctccagcagg tgcctgaggc agcctattat gttgcctttc
tccatctgta
1392961 ttgttggttc atcggagtaa gcagacttga ccgagtggaa acctttagga
aaaaaataaa
1393021 atggcacctc aatgaaagat cacccctgta acacgttagc tgtctgacca
gacagagatg
1393081 ggccggaata caatatgaag tcgcaggtgg caaataggag gagggaaagc
gatgttggga
1393141 ctacatctgt gcaggttacc catggtcaaa cacacacatg aggaatttga
atgtatccag
1393201 agtattaatt ataaatcacc tgtcttgttg gctatgcatc agttgagaag
aggcaaacga
1393261 agcaatactg taccttgagt gcacggaagt acgaaataaa agcttgtgca
gcacacgtgc

1393321 agtgcgagag cgcagccttt tgtttgtgtg tggaaagtgg aagatcatga ctgcaatcat
1393381 atctcagcct aataatgaac tcttgtttgg ttctctctgt gtgtgtgtgt gggggggggg
1393441 gttgctcttg ggcattttcc cttgtggtgc tgtctaaaag aaaaaaataa atcccccatt
1393501 ccacgcaggc tacgctgctc tttgattgat tatgcagaga ttttccacag cagtaatgca
1393561 attcatagat tctgacatgt ggctgtcaga gccaaaactg caccaaaacc gagattaagg
1393621 gtatttagga ttaataaaac atcctgatgg atctacaaat gcagcccgac atgcatttaa
1393681 gtattctaca gaaattcata aaaaccacac atgcaaaaca tgcctatttt gaggaatagg
1393741 ggccacggtg acagaccaca tcagtcacac cacaggtatt cataaacgtt gtgcagactg
1393801 acccagtagg tggcctgact ttattccaac tcattaccgt caacctgtaa tccggtaacc
1393861 tgacaaggct ctctttggtg cttgttaatg atctgtttca cagggattag agcagagacc
1393921 ggagtcattt tgataacaaa gagccacagg tcagactgca tcctcgcatc actggaatgg
1393981 acggaacgcg acattacaca agcgcaggag tggaaagatg tcatgggagg ctattaccca
1394041 agaaacatgc agacggtaat tagctaacaa cttaaacaat tcgcagatct tcgaccgcat
1394101 catgaatcac cgagtctaat ttctggctaa ttttgtcaag gaaaccagta gaaggtttgc
1394161 tgccccaata aatcaaagca tcagtacaaa aatggattca cgctgctcca ttaacataat
1394221 cacagagtca ctgggggcaa attgttgccc tgactggttt aacaaaatat gacagacgga
1394281 aaggaaagca gaagatttta gcatgaatgc ctcacaggag agctttgttt ttttccggct
1394341 ctgaccttaa attgagtttg gagaagtgct ttcagaacgc gagaggaaat tatttggatc
1394401 tttgtcatct attaaatata gtgggggcag cagacagaga ggctgatcta atctgcccgt
1394461 aaaaacggtc gtcatcctcc gcctggcaga ggctccgatt tggtgtcagt gtgaggaacc
1394521 ttccctttat tccgtggtaa aaccctgctg acacatttcc ctgtcttgtg agattcctgt
1394581 tgaccaaaat ttgataaatt gtaccccttc cggggttgtc gaggttgcgc ttttgtgaca
1394641 caagtacaag aacacacgtg tgactcagtg acgcactgga acaaatgtgt gtatgggctc
1394701 agcctttcat agccacatta aagcgtatca agccactgat atttattacc gcgcttcaga
1394761 cgagttaatg aagtcgtgga aagatttcat ctcagcggcg ggcatggaaa gaaatatctg
1394821 tattatctgt gccggctcag cagaaaagaa ggggacaagc ggaggaagtc gtaaactgct
1394881 gatcgaggag atctgcctcg tcttcttggg ggggggggtg aaggttaaat ccaactataa

1394941 aactacaaca gggggatcaa gagccggacg gcatgctgga acacatttct tgtgaaatga
1395001 cagagattgt tttaaacgct gtgaaatatg ggacgcggac taaatcgaat acagcgctgg
1395061 agtgggagcc gtctgcagct actttatacg catggacaca gttggactca agtgagggat
1395121 gaatttcagc acccccaata aaagccaggt cgcaataagt gaatcactct tcacactgta
1395181 agtagagctt gttaattggc tctgtgagga gtctctccag tggggaatgg cgacgccgtt
1395241 atcctcccag actgggttat ttcaacaatg tgtcattggt tttgggtcag ggacgctcag
1395301 tggcgaatcc ttggggttgg ggtgggggac tccggagcgc ttcctggctt tggtgtgcat
1395361 ggattcaatt aacctgccga gatgatgccg ccgcattctt tgtgttttat ttgccaacgc
1395421 ataaaattcc ctttgaaagt gcgctccgcc gttgctctaa agaatcagag ataaggcgca
1395481 atgaatcgta tcactttcat acaccgccgg caaaatgcaa ggctttataa accttggaga
1395541 aaacaaaggc acccctattt gtttagtaat gggtgttcag gtcgttgcat tacggtgtac
1395601 ttgtgtaatt aagcccagtg caatccacta atccaccagg aaagaggtta atagagttta
1395661 ttaggaagtg ctcacattat aggagcatta ggatcctttt gttggtgcac atttcaaaga
1395721 atatcaagtt ttgaccttgt cattatctga gagaatgcaa ttatttttga aaatgacatt
1395781 tgcttggatg tggactgagc acagtccctg attgatagat gtagattgta ttttgctttt
1395841 tactggtaga tattcagata aaagcaagac ttccatcaat gtctatttgt tctggagatg
1395901 ataataaata ccgctgatgc tgatggaacg atgtttgcac ctttaaagtt gaagaggagg
1395961 aagatcaagc tggagcggca cagaaagggg agagagagag ggagagagag agggagagag
1396021 aagggggggg tggagagaat tgcacctcct tcgttatatg tgacaataat ccagatcggg
1396081 cgtttctcct ctccctctct cttctctctc tctccgtcct ctcactctca tcccgactca
1396141 catcacttca tctctccatc tgcccgtgct tccgttcgct gctcctctgt tacatccatt
1396201 cctcctcctc gctctcctcc tccatcctca gcatgctcct tacatgaaag acgccaactt
1396261 gcagggaatt aattaaacag aaaaggagaa gaaagcaaat gatctgccga ggaataagag
1396321 gagaagcttc acacgtttgc atctcctgct gagtgcgttg gcttgaccgg agcagcatta
1396381 tggatctgtc attcatggcc gcgcaggtga gacgtttctc ctcctctctc ctttgtctca
1396441 actttaatct gcccggtaaa tttatgggcg tggccgagct acaagttatt taacatgcac
1396501 tgaataatat ttgctgcaga ttaatttaac atgctgccat tgcgttattg tctttcccag

1396561 cctccgctca acttgaaaaa ttagatgtta tgtccaaatc aacagagtgt tttaaaccgc
1396621 tttaatatga gagttggatt ttgccaactg agcctctatt tcttctgtta taataataat
1396681 aataataata ataataataa taataataat aataataata ataataaaac aagcctccta
1396741 atacacattc ttggacactc tgagctgagt gggggggtg aggggttgca gtaaaaacca
1396801 agccagttat tcactgctgc tgtgcaaact tttgggagac ttgtaatcac tccactggtg
1396861 acgctcccca agggcttaat ctgctgcttc taaaaacagt gcgcagcaga cgggctgagc
1396921 gacgcactga aggaggctga ctgctggcag cttactgact ggcggcggag ggctacgtac
1396981 tcttgtgtgt gagtgggagt cgttgaatgt cacaaagttt tatgctgcag ctgcagagtc
1397041 gggggcctca cagacaggaa acagcaggtg cttggtcgtt cgtataacct gctgctgctg
1397101 ctgctgctgc tgctgctgct gggagagctg aggtgtcact gctgtaactg ctatactctt
1397161 caaattctta catcacagag aatttaaact gttactagag ctaaataatc tttgcagcca
1397221 ggaaatccag tccactctgt tgtgtctctt agggggcaaa cagatgatca cagttcaaat
1397281 ccaccactgt ctgcccagag catccaagtt tttggcatcg tcatgccact gaccaaaaat
1397341 gtcaaaaaaa agacagaaat aatcctccag tgtgacaaag gagccttttg ataagaaaac
1397401 aaaaccggaa ggatctaata aaatctaaaa catttgtgtg gactgagata caaagaatgt
1397461 acacccagca tgagtcatac tacccccca ctgatgctga acggtccaca gtaatttaat
1397521 gagaatatgg atgaagaaga tcccattgat ttaaagatca aaactcatat aatatataaa
1397581 ataggccatt aagacaaatg agtatacccc cccccccgc agcagcaaac agctctctcc
1397641 gacggttaat ggccaatttc tctcatgtgc aaacgatgcc agttgcccga gtcgctctgc
1397701 aatgattgtt aatggagtcg ggtaaacatc tctgcaggtt cgagagcgga ctccacagtc
1397761 ttcacctgac tgccagcttg ggagaacatg cttgctggtc cctcggcagc agcggacgct
1397821 ggtccttatc gctgctcact gagctccctc ttgcatgtgt ggctaattgt tggcatttaa
1397881 atggctgatt tgaaacttcg ctggagcgaa agaggcggca ccccgtcgtc cagccgggga
1397941 gacggaagca gctgagtgct aatcagagcc cgtccgaaag ctcaattcca aagcaagaaa
1398001 tgggatatca ttagtttgac gtgggctcag tcccggctgc acactgcgag gacattttag
1398061 cgtgccgtgg cagatgtggg accatatgaa aggagccact tccatgtgtc gcagacagac
1398121 tgagactcag ccattctggg cattttgccc atctaacgct cgtttcagat gacagatctg

1398181 ccagagacag agagcccctg aaggctctat ctctcctgta catggcaccc
tgctgccctc
1398241 gcattctcac tttagcgcgt gcaaactcct ccaattcccc acgtgtgctt
cctttctatt
1398301 cattaattgc atttcagagc catcaaccac acgagacaca ctaggtcttt
ttccattctc
1398361 tcacgtcata cattttgtct tctcatgaca ggccatcagc ttctctcatc
actcaattac
1398421 ttatgcatcc aagccgcggc tctgagtcgt ttggtcctga cagggctgtg
catctcctct
1398481 tctgccagca aggacagacg agagggaacg gatgaggatg gagatgcggc
tgacagcatg
1398541 aacacaaaca gttgtctgta tttacccaca gcagcagcag cagcagctca
tgagggactc
1398601 gtccgagaca gttggaggca aattaaaggc aacaaggcta caataattaa
agccgatggc
1398661 tttaatgtga tgttttaagg gagttaattc attaatgttg gcagcacacc
ccgccctaca
1398721 ccatttgcaa aaggatcttg gggtcaaagt tgaggaagga atgaagagcg
gcaccagaaa
1398781 ctcctgataa gaaatgcgag gatcccattt ggattttccc gttacaaccg
tcttctgaag
1398841 caatccaaga gcgtctctgc tctgcataca aaaatgaaat tatgtaatca
cattatctca
1398901 ttgtggattc ttcatgccag acttaaaact aaaaagctcc tcgttctcca
ttgtgggggc
1398961 gaattggaag aatgtaataa cagctcccgg gcttgtttat ccccgacttc
atttcccctg
1399021 gtcctttatt tgtattctca acaatcggat ttatattaaa cccgatgttt
ttcagttgtt
1399081 cacttttatg acccggcgtg ttctaaaaaa gcctgcgaaa gtaatcgcct
tataccaatc
1399141 agacgacgct gcgcgattga atgatcgatg actccagtcg gcctttcctg
tcatctattt
1399201 tcagcaggga agcaggaagt gaggtcatct cattttgctg cagttgcact
tcgtttagct
1399261 tccaggcgag atctcattga agatgtcacc attgagttcg accccttcct
ggtgacaaaa
1399321 acagacacaa agttttaatt tgatagtgat gctttattat ttagagaggg
ttctcactgc
1399381 gagggtgaag cagaggtcag gctctctgta tccccccccc cccccccatc
ctccaggtcc
1399441 atctgggtca tgctgggaga tcacagcgag cagaagttgg agttagaaca
atggagctca
1399501 ctgctaagag gtcagtgttg ctgaagttaa gactcattca gaacaaaagt
ttgcaacctc
1399561 caatacaaat taaggacagt ccctgtcccc acaggtggct ctgcagatcc
cggatcagtt
1399621 tcaagcttaa gtgagaaaaa ttatcaccct cagtggggga agcagcggct
ctgtgttttt
1399681 ggctttgaca tctgttgctg gtgccaagca gccttgttgc ctaattcaag
gtggcccccc
1399741 cagaacagcg attatacaaa attagctgca gctctacggc accttagtga
ggtgacgtgc

1399801 actgtttatt ttaaaagggt gggtgtttta tccgtcctgt ctgagggaca tcacatttga
1399861 tgttggtcca aggtggctcc cagctgaagc ccctccatca cagtctctaa aatcttgctt
1399921 cctattagaa gttgatggac acttgcaata ttaccagtta gtaatcagca cactgtgtca
1399981 atattgacat ataagctgct ttgttagctt aaaaatgact aacagcacag gctgaataca
1400041 tctgtaatct taccaagact ccaagtgagg aaaactggta actgctgaca tcagttcctt
1400101 gggcggttag cgcggcgtgg cgtggcgtcc atcccttcgg ttcagtctga gggacagctg
1400161 atttttcaag gagcgcctca gcagtgacgg acagctccgg aaagggcgag gaagcacatg
1400221 acaaaaagaa tcactcatac atgtttaaca tgagaaggtc aaaaggcgat atgacctcac
1400281 cgacatcccg gccggcgtgt ttaccgaacg gtccggtgac acgtggagga aggatcagcg
1400341 cctcggtgat gggacgagat gtgacaggag cgtctcggtc aggaagtcca gctttagatt
1400401 ctctacagct gaaaagacac attaatgtga aagataacag gtttgaacct aaagcaacca
1400461 tcaagcgagt cctgccagtg tggaaatgag agctcagcaa agggtgccga tcgatacccg
1400521 ataggctgag acaaacacca aactgaggga aaacaacctg cccccgtcgc atgcgaaaca
1400581 aaacaatcac aatcaattat tgccaggcaa cctgagaata attaggttaa gtgccgattg
1400641 atgagaaagg agagaacaac ccctctccct gtcaatgtca aattcagtgt ttacaacact
1400701 gtcccagctt ctcgccgtaa ccacttttg catcgtcgac ggcgacgtct gtgcacctga
1400761 aatgatcgat tgcgtattga aggaagtccc ggggagaacg agagggaaac gctaatggcc
1400821 tgtgacagaa gtatgaagca ataacatatt tccctgatag acctaactgc acgatgccag
1400881 cagcccctt gatattcaga agcaatgaat tatgtattcc cacataaata atctatgccc
1400941 ccaacccgtc cccccacagt gcacggccct gttatcccct tgtttcgaca tgtttctgga
1401001 tgcggccctg ctattttgtg ccgaccgcag accagggctc acagaccagc catatttagc
1401061 ttcagctcat gcgcagagat tttaatatga aagggggga cttttctgga aatttgcggc
1401121 cgtggacgtg gagacgggca ggtgccgatg actcaccgtt gaatcattgc tgaattgtgg
1401181 atggagctta ataccgcggc agcagcactt tattgccata attatggagt ttgggtcttg
1401241 tgagggggct agaataacag aattatttga gtttttcct catttgaagt caattaaagt
1401301 ggttccactg gatggtttag acaagtcctg ttaaagagtc cttttgtggg ggtttagaca
1401361 tcattgaaac ctatttttct gtatatatta tttattagca tttagggaag agtttatttt

1401421 ccagctctgt ggtctcaaac aatgttcctt gaataccaac aaccgctctt ctcttgattt
1401481 gatttggtta agtgcgcctc gctccttgag actgaccttg tgtctgttcg taggaaattg
1401541 tcgggatgct aatgatgcta agcttttttt tctttttaa ctcgctctcc ttagacaatc
1401601 acgaacacag attccacagt gggaaaatgc tgctgcttcc aatgttctaa gcggctccgg
1401661 ctgtatctgt gtctgactgc atccctgcgg aacggtgtta tcccggatta cgtgactata
1401721 ttctggtctc atagcccatt ggtgtcgtct ggctcactct aaaaatgccc ttgttccagt
1401781 ttccccgttg atgaactcat caaaacacaa tttacacccc caccccaccc accccgcccc
1401841 ctgcattaaa ctgcaaaaca ttccaaccag atgttgattt tttccccccg catgatcaca
1401901 tttcgagcat ctattttggt cacgcggtgc caaggtcggc tgttctttgg acgctctgct
1401961 gagagctgat ttcactgtga ccttttttatt gtggacggat gcagctttaa tgaccagatg
1402021 acatatcggg ctcttacagt acagtcagtt atattactta tggaaatcat tcggaatgct
1402081 gctacacatg agtgaagtac actaaggtgg agttttctta aagcgctgaa taattgatgc
1402141 tgctcgcgag cggtcgtgga aaagagaatc acagctacga ttcttttctt ttctctttgc
1402201 tagtttgctt ttaacctttg ttcaccctga tcctgttggc agaagagcaa cagtttatta
1402261 tttttctttt tttttttttt aaccaagccc agcatttatt aatgagtatt gagcttctct
1402321 gtgttttgac cggtaccagc tggaactgtg gtgcagatca cgcatctgtt tgggtttaca
1402381 gctactctgt aggcatcaca ctgcctccac agcattgcca ctgtgtagaa ttatccttaa
1402441 cctgccacca cctaaaaaat ggaaaaaaaa ctgctatagc agcggaggct aacgatcttt
1402501 gcacacaccg taattgatta gtttaattcc tgcctcctga ctttgattta tatgatatct
1402561 atttatagat ggccgagtgc aagtgtatat tgaatctatt ctagatttgc aaatgaataa
1402621 ctggtatata tggtggttct tttgtggata tgaaccataa atataagcgt gcttgaaggg
1402681 aatcacagac agggagcaaa cacaaccact gccgggctgt tagtctgaca gtgttacagt
1402741 acttgaaaag gttgtaacta ggccaacatg gcactggcgt gacaggctca tcactccccc
1402801 acctccaccc acgccacccc cacctccacc atctcctaca ggagaatcac aaagacagaa
1402861 agcactgatg ctaacctgaa gaggagcacc agaaacccgg gcagcggcac acagattttc
1402921 attctagttg atacgctgaa ggcagttttc ccaccacaga aaaaaaaaat tacagttaat
1402981 tacagcccca cttgttatgc aaaaccagct tttgtataag tgagaaacct ttctggctcc

1403041 atcaggactg gtaggatgga agctggaggt ggcaggttga gtttaaagac gtataatcac
1403101 atatagcgag aaagaatcct atttttcttt cctaagaaag acttaagctt tattaccatg
1403161 gcaacaacaa ggggaaatca actgcacttg cgcttggatg tcttcgtgca tgcggggaaa
1403221 tcaagtcttc cactcacaaa acacagtcta aacacaaagt ttccactttg tgttttagac
1403281 ccttttagcc aagaacacaa catgctgtca aacccatttt gacagcatgt tgtggggctt
1403341 gggaacgtac gcaggagggg caattaatca catttttgta tgagattgac actttgtggc
1403401 gaaggcaaag acatgattac acaggagagg gaaggagcag gggaagtacc gtattggccc
1403461 gaatataaga cggtgttttt tgcgttgaaa taagactgaa aaagtggggg tcgtcttaca
1403521 ttcgcggtct agacattata cccattcaca acgctagatg gcgccatata tatttaaagc
1403581 gaatgctgaa cttaacgtcg caggccaaag cgaacccctg tcacgaagaa gaaaaataaa
1403641 aatagcggta gcacgaagaa gaaaaataaa aaaatagcga taagaaagaa aagagaagaa
1403701 aataagagaa gagataacag aaaatagaga aatgtagcga caatctggag aaaagtgggt
1403761 cgaagatata atcatttgtt tcagatgtac tgtaattatt ttctgtataa aaatgacatt
1403821 tggtgttcaa aaagtctttt ttcaaacttg agtcttgaaa aagagggggt cgtcttataa
1403881 tcagggtcgt cttatattcg ggccaatacg gtataattga aaggaggctt ttataagcaa
1403941 aatccagaag aaaaacagca gccaggggga ctcggaggaa ttcagtggtg atttatgttg
1404001 gaccgaaaga gcagttgttc aataggatct acaacattgg ctacaacacc ctgaaactgg
1404061 tcaaaggaga aacctattcc gtcacaacaa cctgtaacag ttagacacac ctcaaaaaac
1404121 cttgcatcag gctgctgcgt gtgcacacgg aggccatcag gatgtatgcc tgatatgcat
1404181 gtttcatgct tgcaacaccg aggcaaattt gctccatctg gctctgtcat tttaaaataa
1404241 cttctgctac cattcagtcc aatttctcag tggagttatc ctacaggatg acacaggcta
1404301 gcccgcccgc gtctgccacg ggttataaat gttaatatga atatatcatg tggcgtgaga
1404361 tgatgcagaa gacctttagc tgtagaatgt gccattaccg tgttaataca gaagaaaggt
1404421 gcagatatat ggacacacag attatacaag aggttaaaaa gtgttaaaca ctctgtgcag
1404481 gctgtacaag ctatagagtt tcagctaaca tgaggaaccc tagtttggaa gcacaaatct
1404541 ggatgtgcca acatgcaaat gtggcagttg aagctcctcc aagcagcctg gttttcattt
1404601 atttttttta aaaattgagt ttatgtttct ggcttggtgg ctgaaatcat tgtatcatga

1404661 taaaatgttt ccagacgcgc tctgcccgca gcaccatctg accgttcact ccgaccagct
1404721 ggtcagctct gggaaagggt tagcatcttg tcctcggggc cgctcttggc taatgtcaga
1404781 gggaaaaaga gatccatcaa aagtcaaaag ctgttgtgcc catgtcttca cggaggcccg
1404841 tcatcaagct gttgccgtcg ccggagcaaa acggggagaa gatgactgtc catttacacg
1404901 gacgctcgga ggtcaatgac ttacagttag ttggtcagtg tttcccaatt gctgaccttt
1404961 taatgtcgag gattattttt tccgccattc aaaagctaaa tccagtgaaa gggaacaagt
1405021 acatttcctc cattctgatc ttttcatgct tgcgttttga ggaactgaca tttgcctttg
1405081 tgggacagag gagcgactct gcagctctga aactgttgca gccttgggag ctggaaagaa
1405141 tgacgtctga ggccgttgat gacaaggtta gagcagacag agctaaatta ataaaagcgg
1405201 gggtgcattg agagcctcat aaccggagga atgacatggt ggctgggtgt gctgacctcc
1405261 tgagggggtg aggtgatggt cttaatgccc gctctcgact cagttcatgc cctcaggtct
1405321 gcatcattca gttcaagttt tccactgcaa cttaaatttc tctgcattgg ttgtgagtgt
1405381 gcacgcttgg acacagcagc acctggacat ggacaatgat ccacaacttt cacagcttta
1405441 caggaggaca aatgtccatt ttcttatcat taactcactt tgttttcctg aattgtaagt
1405501 tatgacaata caacaaatga agaccaaaac tttacagcaa tcattttttt tcaaagcaaa
1405561 taacccctt ttattacatt accttccttt aaacgaaggt gaattaaagt tgatagttgg
1405621 acattcgggg atgatgttta atactcacac tgaagattca ggttggtact gacgcttcag
1405681 cagcaacact ccacagagag gattttgtcc atgaaacatg aaggaggcaa agtgtgactc
1405741 ctctgggcgc cctttaaatg aaattgtccg caatgcaaaa taaatgagca ggcagcttga
1405801 tatattttga catgtgtgca cagagcccaa tgtgcccccc cccсttccaa ccagcgtcgc
1405861 gtatttagag attaaaattg gtcttttttg gtttcaagta cgtcaaacat ctgatttcaa
1405921 gaccccgtaa ataatgtatg gagttagaat tgatgatctt ggatgataaa ataatctcta
1405981 agacaggtta ttcacatatt aatacatgtt tctgtgtaaa acattattct aaaaagcccg
1406041 agtaaataat ttaaagcact ccgaaaaaga aaaaaaaata tcacagaagt gaaatgatct
1406101 gcatctaatt tgcaactgaa accattcttg tagtgttttg ggggcttttt aaatgtttgg
1406161 tatttaaatt tagcagaatc tcggggctga atgcacgggg gcaagtttat accccaaatc
1406221 cagccgaata gttggttcaa aacgccgaat gtgtcattga tgaataaagc atgagcagaa

1406281 attcatcccg ctggctttca ccaccagcgc catcatgaaa gaggaatata gaatattaaa
1406341 aaaatggcag gggtcactgt ttaatgtcta taaagtctct agcttagctg tttttgtggt
1406401 gcgatcacca ggaagatcag tgtttcttta aacttctttg ttagttgtta gtttcaggaa
1406461 cattttcatc acttcttccc tgagtgagca tcagtttcta aatgcaaaca tactttatct
1406521 ccttcttttc tcgtatttct tcaagagcct gccatgaccc aagtgatttt atgtcccaac
1406581 agctttattc accagcagat aatttacaca aacatcaccg tgaggtgcac aaaaccataa
1406641 atgaggtgcg tttattggtc acatcacaca gggtgttgag ccaccatcac tttgctcctt
1406701 actgtaagca gctgagtggc aagatttata caggagtgct gccggctgac tgagataagg
1406761 aaatcaatga agcagcagct gtaaatctgc gcagcttgtc ggaggcagac ctgaaaccgt
1406821 aggtcctttt gtgggtgaac aagcgtttgt aaatctctgc tgcagattta tggcatgatg
1406881 gcttgtgtat gtgcttcaga taaaatctat tgttagcctg gatggagcag ctactcttat
1406941 ctccatgctg ctcccctcag atgctgcctg ctgtaatttc ctgttattta ttttcctggc
1407001 ctgtggcgcg tcgtgtcgca caaggagcgg gttatagata acacccactc ccaaaacact
1407061 gacatttatt gcagctcctc cgggagagct gaagtgcggc gcacagctgg aacagaggtc
1407121 ggaagaaaac ccaggaagtg tcggatgtaa cagggatctg atccagaaca agctgcaaat
1407181 gcagactggg ggacacaact tcgggttgga cttgcacagc tcgtaacggg gctcctgagg
1407241 tgtgacttcc tgtgctaacc ccgccctctt ttactcacaa agagatggag tttggttgtc
1407301 aaaatgttgc ctcgttttag atgaacaaac caaacaacga ttctccgttg gtgcttttgg
1407361 gtttcttcaa cattgtctca gtttagtcta caaaacttat attcacattt tacagcattt
1407421 tgctgtttgg tcacttttca acagtgaaag tgaaaccaag ggatgaatac ttggcaccag
1407481 tatatatgca gtaaattgtg gattatcttc catctggatc gatggtagtg ctgcgcgtgt
1407541 aaacgagcgt tttcatcccg accgcgcagc agcccacacc tgtggcggca gcgattggat
1407601 tttgaggcgg cgagcagacc aacgagctcg tcagcacaac agcatctgtg tgcaattatg
1407661 tccgtgtgca ttaggtagct gctggttcag cttctgtagt gcacctctca gggcgctctg
1407721 ggggagaagc acaggtgcga acggcttctt ccatctcgct ccaggttctg gaccttggtg
1407781 ctggtcatgc aggcgttatg tcctttcctg gaaaaaacga gcagcgtgtt gtaaaagagg
1407841 aatcttacat atgtagcgta acacctatct attcaccttt tctgagtagt tgcatagttt

1407901 aatcatttag cagcactgat gttgccacac aaaagggtca gatttagatt
tgcatacaca
1407961 cacataatta aatagtaatt acataatgca gtgttctctg taatgcgtgt
gtgactcaga
1408021 gctcgttgtt aggggagggg agctcacacc tgtccaaaca gaggcgtacc
tgaaacacca
1408081 gagactgttg tgacagtttg aagccaacgg ctttgttttc attgatgggt
catgtgatat
1408141 cgagcgtaaa gcccattcct caaacagcag ccgtccgcct ccccactcgc
catcctctcg
1408201 cgtacggctt cgaagttccc tcctccagac ggacgcgctt atcgatcagc
tcggtgacct
1408261 ttttattatc ccctggagaa cagaactgac tttttctttc tgctttcagg
tatgcgttgc
1408321 ctttgctgtc gtcagaagag tttttcgggg tttggttgtt gcttttcgag
gagtcctttc
1408381 tgggagtgac atcccacggg aagcaattat ggttcacgat ggctgtgatc
acccatgttg
1408441 ggtggaacag ctctgggaac agctttgact aataaatcga agccttttgg
agtggagcag
1408501 gtggagagca tacaatgaac agggcttcac tgcaccacca cacagtcttt
cagcatctca
1408561 aataggtttt ttaaggattt caaactctac atttcaagtt tgtgactttg
aacatggaat
1408621 ctggagctta tttagtgctg ctcaaagaac taacagaaat ccacctgaaa
aatttagtgg
1408681 ttacaggcct gtcgagtaaa ccaatgtcct gtttagtttg aataatccac
agcctcactc
1408741 tgaacagctt cttttattca gaccagccta ctttccaccc tgcacaaccc
cttcctgtcc
1408801 tctctcaaac gactgtttgc aacgctgtcc tccaaggaaa aagaaaatca
gaaaaaaaat
1408861 tgctataaat tattcaattt ccatgtagga acacattact tttgcacagc
cttcgcatta
1408921 aaaacctgtc aatcaaagct gctcaggagg agaacgtcat catccttcct
ggtcagtggc
1408981 tacaacagat catttgcatt acggagagcg tcactacctc ctcagggcgt
gctgggctga
1409041 gatgtacggg gacacagact gggataaccg ttgtgttatt gcgaatggaa
ggaaagggat
1409101 tgtgggtagt ttcctgtgct gtagggtggt atgaggtttt aaacggtatt
ctttaaagtt
1409161 atgcagagat atatgcatta ttttatggct tatatgcagg ttgctgcact
atgtatgttg
1409221 ttacatttgg ggtctttttt aaattcttgt cttgtcaaat caccttttct
tgcaaattgc
1409281 tgtaaggaag tccagatgaa ttatgctcag ctccaaaatt tggccaacct
ggttgatcga
1409341 tatgtcagag agatattctg gctgaggaac taaatgatat gactcctgat
tcaaggacgt
1409401 cttcatgagt aagctatttt tcttttttcg cctcatcact gcgatgccat
tggacatgta
1409461 ggaatgcagc gtttgttgac attgtcagtt gtgcttcaac gtgaaatgaa
tttgcttgaa

1409521 tttgtgtaca tttgggctaa atgaggattt tggttagttc agtcagacca atgaggttct
1409581 agctggtcac gatgaggctg aaccagaaaa gtcaacggtc cagctcaaag caaactacct
1409641 gactcctgaa atccctcact ttgctttata gcatttggtg caagtacagg aaggggtggg
1409701 agggtgaaat gcattagaaa atataaactc tggagacagc atcataaagt gtgaacctct
1409761 gtcccagtag gtttaatcta aagtttcagg gaactatctt ataagtctgg gtaatgcgat
1409821 cacctccccc ttccaccacg ttgccagggg atatttgtgt tcaggacact caattctaac
1409881 acgtttacca gaaagctttt tgtctgggat gtggtattta tccactcctc cactttagtg
1409941 cgggagagat gaatgcgtgt gacttctagg aaactatgga gtggaataga agtgattaat
1410001 ccacatatgt cagcagacat ggcttctgca gaagcattgc ttaaggctga ccttctctga
1410061 ccacttaagt gctcgcttgt gttcggatgc tccttccccg cttctctgtt tcactctgtt
1410121 ccattagtgc ccatccacag gggagctgtg ggcttggctg agtggagcaa gtgtcttcat
1410181 gtctgtagta tcatcgtcct tccaggctaa agatacttca gacatgcaac tgcaacacct
1410241 gcctgcgtgc tgcagacacg ttgggtcatc ctcttggccc aaaataggga aacggtgcaa
1410301 ttaaatattt agatttgggc tcagcttgaa tatttccaca catgttccca gcagagacta
1410361 ctttttattc agccagcttc tccatttatg ctaacagctt acttccgcag acaatgagtc
1410421 acttatacat aaaatataat tataaagaat gcagacagag ttgagagctt ctgttatcat
1410481 ctaaccgaat gttagattaa gatggaatgc atgataggat tctcagcttt gggttgtcag
1410541 cagatcacgg cgttgtggtt taaaattagc atgtcaatcg aggccaagat gctacagatt
1410601 gaaagccctg caacctgaag tgataagtct gcgcaccctc agctcctcct cagctcattc
1410661 cccagcctca cgctgtccca tcattatcac atcagaaacc cagcaggaga taattattgt
1410721 tgaaatacta caaatgtttt tatatctgta ggggaagcga gcacaattct gatcaaatca
1410781 tggttctgtg tttgattcca cccagggcct tttaccaagt tcccttcatg tccaaaacaa
1410841 gcaacatgga aaaggctcat ataacaagga ccctcatgct gctgatagac cgggtacagg
1410901 agaagggaaa atgggggacc agctataata agtcttccct ggaactcaaa actgctgttg
1410961 ggaatcgcag ataatatctg gaaaccaacc ttgtaacatg ttatgtaagc gttgtagttg
1411021 gaannnnnnn nnnnnnnnnn nnnnnnnnnn nnnnnnnnnn nnnnnnnnnn nnnnnnnnnn
1411081 nnnnnnnnnn nnnnnnnnnn nnnnnggggg gggggggggg gatacattga gatccaggtt

1411141 gctggacttc atgcatgcct gtctgccctc atcatgctca tgtgaacagc gggcgctcca
1411201 caggatgtgt catgtcatta ttcaccacca ggggccaatg aatacagagc agcacaatgc
1411261 tttcaaaaca gacttttccc ccgtgcgagg gaagaccaca agtgttgtgt gtccgtgtgt
1411321 gtgtacgtgt gcgtgcgcca gtgtgtccgt gacatgtctt tgtgcattag cccatgttta
1411381 tgtgaagcgg ggggggggtt ataaacagga gtgcgaggca gtgaaggtga gcaaatgagc
1411441 aagcaaaagc aaatttctgt gtatgcttga aaccaacaac atccctccga ggtattttta
1411501 gcattgtttt tcgacatctt ctgcggggcc tgtttctcat ccagagcaat atatgctcct
1411561 ctcatgcact tccaaattat atatacatac atcgccatgg agatgcacat ttgatgtact
1411621 aaagcaagca cgcgcactac agctttcagt tgattccacc ttcacgggac ctggatttcc
1411681 ctagtaataa gtcacatgtc aaatatccat tctatgttta tcaagatgcg ccacacacac
1411741 atacacacac atacatacac atatatcccg cttgctttcc atggtttcca gcttcttatt
1411801 aatgatttca tgctgcattt actctcgact aatatgaaca aatcttgtgc gccacagtca
1411861 tggcatcatt cttcttgagg actcgactca ctgtacatga ctgtaaggca gccttgaatt
1411921 agcttatttt attacaaaaa acactgttaa ccagttggga aaaatctcac cctgaggttt
1411981 tttttttggg actattaaca aagttagact ttggtatttg atggtagcta gccttaagac
1412041 tttcggtcgc ctcagctggt tttggcggat ccaagagcat cagcgtcatt aaaagcattg
1412101 acggcatttt gtggttcacg acttcgcacc atcctgtcaa gaagagttgt aaaaaattatg
1412161 tttattgttt acaaagggat ctgcagtggc ctgaaatgct actctgcaga catgggtctg
1412221 atagcagaat cacatgtgct ggatgagcat aaatccatat tttgttagaa ttctaaccag
1412281 tgtgtactat ctaattatct aaatcgttac ttctcagatc cccatgatga cgggggcctt
1412341 catggactcc tcccccaacg acgactacag cggcgagcat tcgctcttca actcctcggc
1412401 cagcgtccac gccgtcacct cagcggcctc tgcccagggc cagcaggacg agtcgcagtc
1412461 catgtccagc gacgccatct ggctctggat tgccatcgtc gccactgtcg ggaacatagt
1412521 ggtcgtgggc gtggtctatg ccttcacttt ctgatgaacc cgagcagcaa cacacacacg
1412581 tatcgactca cacatcctgc tttttctgtg tcgttggacc cccaggtcag gacacgattt
1412641 gtgagacaca tcgtctctct gcatctttat ctctttctct tggctctcat tttgggcttg
1412701 cagacgttgt aatgctacac acatagacta tatttactgc tatatttata cataataaga

1412761 tatatatata taacaaggga aacagggact gcagagtttg acagagtagt ttgtgtacga
1412821 cagctcctta gtttgaaaca gatacatcga agcggtcacg agtgagaatt gaaggaagct
1412881 ttttttcta tttaagtttg tttccgtgtt tcccctcaga caacacctac aatttgtaat
1412941 gcggagatgg aaatagagat tcaggtcagg ccgtgccgac ttcagcatca cccagctaag
1413001 caatttggga tggtgccgtt taattatgta gccgaacagg gacgccgaat tttaaaacat
1413061 caaaagtact gggatgctgt tcgcatcagg tcctcccaaa caccacagca aagatgaatt
1413121 ataatggaat gaaaaaaggc tgatgactta aatgaagaca cttcatctgc atatctaatg
1413181 acaataaact attagtaatt tggattatcc tttacaggtt tctgccccac ccagcggaca
1413241 aagagaggaa gaaaaatgtg aaatgagagt cctcttttcc gctaactcat atttattcag
1413301 ggatttgagc ccaataatat tacaaatctg tcttgttaac acgttcaatg ttcaaatgtt
1413361 catgcacagg ggattacaat attactgttt tacgtgtacc gtgctcatgt aaagagagat
1413421 ccagataaat aatagtctag ttgctgttat tcttaatata atgtacagta cagaaggact
1413481 tttcaaggtc tgttattttt aataaatggt cgatgtagaa aatgtatact tgttgtaaac
1413541 cacacatgat gctccttttg taaagacatg ctattaaaat ttgtgtatta atgggttttg
1413601 tgtccacgtg ttgacaaatg agccatcttt agccccacat gcactgacct aggtaccaaa
1413661 taaggaatga tgtgctaaga ccaactgtag atgtgtttcc tccgcctggc atcgagccgc
1413721 tgacggacgc caccaggtaa gattgatgcg acgccgggac ggcgtcatct gtcattctga
1413781 catgctgtag tttagtcgtg tttaggaaca gcgccggcac gcccctccgt ggacctctgc
1413841 actcccagat cttcaactgt ctctatgctg gtttccactg acagtgcagt tctgctgttt
1413901 ttgcttattt tgcatcccgt ttacatcaca catacagtag atacgcagac gcgctttatt
1413961 tatatggtgt ctcaccccag tcctcaaatt ttgcctttct ggatgagcct aactgaagtc
1414021 cgctgtggct tcttttgaga tgtttgttgc gcttcagcct gagtagagag cgatctttaa
1414081 ttaattataa agatctcatc cataattcca caaacagatg ccaccacctc accttagcga
1414141 cagatttggc gagagggaaa ctgtgggctt tccttatcgc cgttgacaca ggcacctggg
1414201 gtggacatga agtcgagcgg gatatgccct tcaaatggcc ttcatgttgc tcgcgttgcc
1414261 tttccacttc attcgatcga gcaagtcggt tgtgaaaaac gtagtttccc cagggacacg
1414321 ctgggctgct gctgactttt agaggcaagg gagggtcaat ggctccttca gccccatcag

1414381 gatgaaacgc acaactgcag acactcggaa gggacctgcg tgaatgtgca ataattacaa
1414441 aaatgcacaa cggtggaaca tgtaaaataa tgaactactg atctccacca gggcttctgc
1414501 gagtgtgatg ttgcttaata atgtaataat gtaataataa taatgtatta tacattatta
1414561 ttattattaa acatatgcat atatgcaccc ttatattcct aatttagctt tgtgcaggta
1414621 cagatacaca taaaagcagc acagtcatag ataatgaaag aatatataaa caagcacatt
1414681 ataaattaat ataaatatta tgaatattca cgtgtaataa ctataacggg aggttttaaa
1414741 agtgtattta atgaaactta caagttattt ttgccttcac tttcatcaat tacgatgccc
1414801 tgaatggatg ctaaaatgtt agcatgatcg atgcacagag ctactgactt gtgctgaaca
1414861 gcgcgagcga gctcgccctc ctcatggtga agtggtttca acccagcatt tatcatagca
1414921 tggatcagct gctcgtgaag caggcggcag acgtcaaaca ccgccttctc ctggaagaag
1414981 cggctcgtgg ctgcagaaaa caatggaaga taatgagatt caagcggtga atccttccgt
1415041 tttgtgggat tctgttgtta tcagcattaa caagggtatc atgatgacaa tgtttgctgc
1415101 ttggggccac tttccaagaa aaagcttgct cccgtcggct caacaggctc cactccagcg
1415161 cacgtgtgcc tgattcgcag aggcactgat atggcgacag ccatcagagc gtggcacgaa
1415221 atatgtcaag cttgcatcat ctgtaatcga ctctgacaag gaatttgaca gggagcacac
1415281 gataaaaaga gattttaact ctgggttacc gacggctaaa agtgactaac agatgagagc
1415341 atgaagaaac atgttagctg gtggcaacaa caccaggaga gtgatttaag cttttcattt
1415401 tcaaaatgaa aacatcctct aattactctg ccgatagcct cacctcactt atgccaccgc
1415461 acacgaaggc ctttggagca caatgtcatg tgattcgaca caaaaattca gccgtgtggt
1415521 tccactgaat tcatgcatcc tcaatgataa tcggcatttc tccctctgaa atgaagtgac
1415581 agccgaaagc tttcccgatg atttattcca cttttcaatc caaccccatt tgtgtgaatt
1415641 tgggatgcgc tcacgtttga acaatgagtg tgcgagggaa cgaggagtcc tgggtgtaaa
1415701 gctgttggcc cctctgcccc ctgatgatgc agcagcctga aaaagcttca cttctgtcac
1415761 tccgtgatgg aaacacagca tcacccgtca accaaggggc aggggcgcca ttcttttgat
1415821 gtagtatccc cgatgagtgg aagctgcatc tctgtctctc tcgctctctc tctctctctc
1415881 tctcacacac acacacctct aagattcaga acagacagcc catccatctc tgtctgtcat
1415941 cctatataga aaacctccct agaaagtagg atggtcaatc tataagaaag ccgccccttc

1416001 tacaggatgg atgcatccct ggacacctgg agccacatct cgtacacatg aaccatgttc
1416061 ccgtttgtct tgttttgtta tgtagcccat attgctacct cacaccggtg tagcacagat
1416121 ctacagcagc atgctgcagc acagaggatc accagaaaca cactttttcc tccaggggcc
1416181 ataaatgtat tctccacaac tcttcagaga gaagaggagg gctcctgaga ccattgccat
1416241 agctctgtca aaacaccgaa catgattgga catcagcatg cggtgccggt ggtctgtcac
1416301 catggagact gacacgtatc actctggccg catgacgcac ccactggcag agacagagcg
1416361 cttccacgct cgacagtcca gaattacagt ggaaaccact cctgaggttc atgccggacc
1416421 cactgagcag agaggacgtg ggacccagga tggacggatg gatggacggg ctgacaggag
1416481 atgacagact gagtgaaaag agcaaaagtg caggagaatg gagaggaggg ggagaaaggt
1416541 cgggatcaag gaggaaaaat gaggccattt ctctgtgagc tgatgagcgt ctgacaggtg
1416601 ttctgtcaga gctgagtgtg atcgaagtcg atctctcatc tactctgaaa tattggcaag
1416661 tgtgtcggga agacgctgtc tcgtccctgc aaatcttata tccacggcaa caaaaccatg
1416721 tgaatcatga cccctgtcac taatttagtt ttagactcaa agtctggtta ccttccacac
1416781 atgacagatg gtggattttg ttctttaaga tcagtgatct ttgatctttt ttttaatttc
1416841 acaggggaca gtacaatttc ataaaacaca tgatttcaca ggttacaaaa gaagccagaa
1416901 taagccagaa ggctagtttt catctgcagt ccctggccag ttaattatta tgtgtgtttt
1416961 gcaatattgg ctcatttggt tatggtacat caacgtactg tacattgtac atacaaagaa
1417021 tcctcagtac atttgaaaat aattagatgt tttgcatttt tgtggtgggt agatcatttt
1417081 gacttggtca ttttataagt agctcgcatg ctgaaaaagt gtgggcaccc ctgatctaca
1417141 ggtatcgccg ccttcagagc tgcatactga cctccgaccc tgttgagcca ctcaacttga
1417201 ctctaccggc agcttacttt acctaataca atgtatttct gtatgcattt tctatgatct
1417261 atgcctattc tctcctctac ctgtcctccc ccttctcctc tctctctacc cagccagcca
1417321 tcagcaggag ggccccccta catgagcctg gtcctgctca aggtttcttc ctgtcaaggg
1417381 ggagtttttc cttaccactg ttgctcgttt ggagtcaggc ccaaccacag accaacgatc
1417441 actgtccaag ggatgcagca gctgcaacta tcaccaccca ctctggtgat aaatcccacc
1417501 gccaacgagg agaaggtgca aggagcccaa cgaacgcacc aggtcaatgt ccagcgggaa
1417561 cggcagccac caagatgagg aacccccggc caccagatga atccagctgt cacctccgcg

1417621 cccaagggcc tggaagccag caagccagca ccaaatcctg ctgtgacaca
aatcttgaat
1417681 gctttaacga cccacaggac attgccagcc agggaccaaa acgcgatgcg
gcacccagaa
1417741 ggccgacgga aagaaccacc caggaaagga gagacactgc aagagcaagg
gaggtctcag
1417801 gacgagcgac cgacacgcga gtggtgtgca ggcagggcct attatagcct
cccccaaaga
1417861 ctattaggct agatcagggg tcggcaaccc gcggctcgag agccgcatgc
ggctctttag
1417921 cgccgcccta gtggctctct ggaactttag caaatatagg gaaaagatgt
gtgaatatat
1417981 ttttttgttt taatataatt tctgtaggag gaaaaacatg acaaacattc
ttaaagtttt
1418041 ccaatgctgt aaaaatttgt ataataaata tttaatttcc acatctcatt
ataatggaag
1418101 ttaaacttaa agcaccaccg tacagcagag tggtcacgtg gtgcgtcatt
cgctccagga
1418161 tgctcgttat gtatttgtag cctaattatc attttgatag taggctaata
aagctaatat
1418221 agacacttac agcatgtgtt gccttcatta taaggcattt cagtttttgc
ggctccagac
1418281 ggatttgtta ttgttttctt ggtccaatat ggctctttca acagtttgca
ttgccgaccc
1418341 ctgggctaga ttgccaacta gaggcacgtg tctggccgag ctgggggcag
gtactgctac
1418401 acattctttc attttgaaca acaggacaaa tgtctgagca gcaacattcc
ccataacatc
1418461 ccattacagc tcagctttta ataccattgt tcccaacaca agctggttct
gtccataaat
1418521 gcaaactggt cgtccaaatc ttgcaaaagg gctatatttt gtacccagcg
agttcagaac
1418581 tgaagaagcc ttctggatag aaggcgaaac gtcttcaaaa gagaagaaaa
acagtccagt
1418641 tgacaaagaa aactaccttg aatagacaag caaaacacaa caatggctgg
gatcaaagca
1418701 tattgacttt attcagagtc aaaacattaa agaaatacca aagccatttc
gaaagacata
1418761 cataaagaca tcaaagtcaa gatggatgaa agaatcttgg catgaaaggc
aaaggtatag
1418821 tcaacaaagt tcccctgctc aatatactaa caactaacaa ctgtgcaaca
ttggctgttg
1418881 agctgtcgta cagagcagaa atgggcgtct cttccaataa acgcaaacgt
gaggaatcct
1418941 caaaatcagg tgactacacc atcagagaag ccaaatccat gtttttcatg
ctggtctgat
1419001 tcattcatac aaaagtttaa agcagactgg ttttacctca caatcagaga
acagcaccaa
1419061 cagtgctgga acggtttatt gcagttatag aagggacgat ttatttccag
tacacattaa
1419121 aacagtctct agaagtgcct tttttcacct gaggaacatt acaaggatta
gaaaactact
1419181 gacccaccat gatgctgaaa agttagtcca gctttttact tccaggtggg
atttttgtaa

1419241 ttctttatta tcagggtgtc caaacaactc tttaagaagc ctccaggtga tcaaaaatgc
1419301 tgcagccaga gttctgacag gtattgacaa aagagatcac atgactcctg tactggcgtc
1419361 gcttcattgg ctgcccgtta aatttagaat aatttttaaa acccttcttc tgacctacaa
1419421 ggtcctcaga ggcctagctc catcctacct ggaggagcta gtgatacctt accagcccaa
1419481 tagaccgctc cgctctcaga atgctggtct actggtggtt cccagagtct ctagaggtag
1419541 aatgggggc cgagcattta gctaccaggc ccccctgcta tggaaccagc tccctgtcca
1419601 ggtacgggag gctgactcca tctctacttt taaaattagg cttaaaacct tcctctttga
1419661 aaaaagctta ttgttactaa ttctgtagtt ccagttacta ttatagacag acaaattatc
1419721 atacttaggg ggtcgtctga tcgttaggtc acatcttagc tatgctgcta taggccaagg
1419781 ctgccggggt ccagaaacat gatcacctga caggcctctg tcaccccact gggtcatggt
1419841 ctcctctcct ctcctctcct ctcctctcct ctcctctcct ctcctctcct ctcctctcct
1419901 ctcctctcct cccctctccc acccccctt ctgtattcat ctgaagaatt acatgtgaag
1419961 tttgagcggt aagaagctcc accatcagag tttcagcgtt taatcctcac atacaaacac
1420021 aggaacattc aattgtcatc ataaagataa aaagattttt ttttttagtg tttttagttg
1420081 atggtgcaga aaatacaaaa acctggaagt tgctgctgca gtgcatttca gcataaacca
1420141 acgccagggc aatgttgcat tgctcctgtt tgttttatag gtctgttatt tacaaccttc
1420201 agcatgccga catgtaaaga ctcagcgggt tatggttggg ttgctgagtg tcggttcaca
1420261 ctgggtatct gtacttatgg tcgtagttgc ggtgagtcgg gttcttgctg ctgtcccccg
1420321 tttatatggc accgggagtt aactgtctga caatatcatg agctcattaa cagtgttgtc
1420381 agcatttgtg ttgccctcta agaaatgcat gattgaaaga agacacgccc ggtttatcaa
1420441 ttatttctga ttaatattca cggtgtatgt gcagaaacaa gctgcagtat atagaaaatt
1420501 ctcttgatag actcaaggac tcacatgttg aggagggaag atgacacaga ccacaaagct
1420561 gctgttgaag acaccatcta aaagtggcca aaattattta ttttatatct gatatccctc
1420621 aactatgact gaccgagttt agaggagagt atctgtcctt aaagtgccat tacacctaga
1420681 ataattcaca tttctctcct tccagcagct ttagataaac ccacagccat aaatctcagc
1420741 aaccttcgat ttcagaaagt tctgctttga tccacaaccg gctactgatc gacatcgctg
1420801 aggaatcatc taaagcagag gtgctcatta cgtcgatcgc aatctaccgg tcgatcgcgg

1420861 aggtagtgtt aatcgatcgc ggcgtgacat taaaaaaaaa ccggcccatc attcatcccg
1420921 tcacttgatt gacatacagg gcaaccagtc cgatgacaac tgaattttga cctttaaaat
1420981 acatttacaa gggaaaacat ggttacacac ctgacacagc accaatacaa gaaacatact
1421041 cacaaccaac tgccaaaggc agcacagagc acacaacacc cacacaacac aatacaagat
1421101 tataacattg tggcatatat atttttttgt ttttacaacc attttgccta tttaatgtaa
1421161 aagaaaatct aaaaaaacat tttatattta tccagcagag ggcgctgtgt ttagagatgg
1421221 aacgttcgag agcagcagtt tgttttgagt acaaatatat taatgtgcaa atgcaattct
1421281 gcagtttttc agcgtttggg gggataaata caatttacac aggcattgca actgaataga
1421341 ttatgtttat aacaaccagc atgattatta taaagcgcac cagaatttct tgtaagggca
1421401 cctgcaaaaa ataaaatatg gttcagtaac aaactactga gactatttgg ttaaaataaa
1421461 atcttcaatc attcatgtgg ggtaacagta tagttcaaaa gtgctgaact gcaggttgaa
1421521 atggtgaatt ttaaaacatt gccttggaga gggagttgat tcagatctga gataacttgt
1421581 gctgcagcat cgtctccttc ctgcttgttg tactgaatgg ttgtgacaat gtggaaatac
1421641 tcagagccct tgtttgcatc cccagggtga acgtggaccc cgtagtgcct cagtgaatca
1421701 gacacgatat cctgcagaga gacgttgcct tcaatctgct catttttcac gtactgggcg
1421761 tcacacttga caacagagca aaagcacaga gaaatgccaa gtttcataaa aaataaaaaa
1421821 aactccgcca gatgattctg tgcatcttgt gattgcaggg tcgtagcaaa aaggaagctg
1421881 agaggactat ttttattgct tttgttgcat ttgagttgca caatttctgc ctctgctaaa
1421941 atgtgaacgg catattttag ctcccactct tgtcttgtcc ttatagcagc ataaaaacca
1422001 gtgtcgagtt tgactttctg agggagtggt tgtatttccc acagccgccg gtctaggttg
1422061 catcagtgcc atcatgcgag ctgcagttag cagtttagag aggagcccgg gggttacagg
1422121 gtcactgtgt gtcagcgcct ggagctgctt tgatataatc ttttatgcca gagctcacgc
1422181 tgcctccgaa ggccctgatg gtgcagaaca gctgtgaaaa caatgtctga gtgcagttgt
1422241 tttgtctcag atctgggggt tgttgtgaaa tgtggagaca aggagcagga tcagccccgc
1422301 tgcctgggca gaaccctaaa gagacgaacc cgagaagaca ggaaattagt attttggagt
1422361 aaaagcctgg ccgccccctc gtccagacat gtacactaca tcacacacca gtcagacatg
1422421 gctgacagca acagactaag ctgtcatctg tccccaacat cactcggagg ccttaaatgc

1422481 tttcagatgt agggacggga aaacaactat ttaacaaata agagcgcttt
gaatctcaac
1422541 cttttcaact ctaatgaaac aaacagacac tcgaatttgc agtgattttt
aatagaaacc
1422601 attcaacaaa agtttgaaat agccactcac agacttctgg tgccacctca
agaccgcagg
1422661 attgtatttg tggcgctctt tccactgtaa aattacataa acgcagctgc
tgcgtgttct
1422721 caaaataagg cctgttctgt gtttggccag tataagacga gactgctgta
ttattatttt
1422781 tgtgctcggt caataaatga aaactacaat acaacagaga taacttaatt
ttctctttaa
1422841 gttctcttta agctgctttt ccctccgctt ctcctgactt ccgatgacaa
agcgttgcaa
1422901 acctagtact tttaatgatt tcgttgtgtg ctggagacgt ttcttttaac
cacgagtctt
1422961 ctaaaaaaaa taaaaaaaat aggttttgca gcatttcctt tgtcaacaag
agggattaca
1423021 aaccgcttca ttacctgcgc aacacatttg caacttcagc tatcatcaaa
agtgcagtaa
1423081 gtgcacttta atagtacttt ggaatgccag catagtcaca tgttccctaa
agttgtgctt
1423141 cctctctaat gacttgtagt gttaccagct accactacgg tggctgtttg
tgaatgactg
1423201 gtagtggtaa tatgcagtga aatgaaaagg acatgagagg tgtgctgaga
cctggaagga
1423261 tggtgtctgg atatttaaca ctcttttcag gtagtgacat tacaccagga
aatggaaggg
1423321 cactatgttg gtgaaattct gatccgacct ttaggggttg cttaggttcc
cttttcagca
1423381 ttgcaggaaa cgtcttgaga actgaacagg aagagcaaag tgagttacga
cattgcccag
1423441 atgacttttt gtgattttga tcttgtattt caatgggggg cagatgagga
agcgagcatc
1423501 tatccgtttt gcagcggtag gaaatctgat tatatggcat cattcgatta
gccgcgtgct
1423561 aacggctgca gcctgaattc agaactgctc aggttacatg gctcgggcct
cctctaaaac
1423621 caccatatat cgttatataa catttaagaa gagttgaaaa agcagaaatt
catctgtgca
1423681 tcatttcatg agcagtcaca tgaacggtca tgcattcgtc acatcataat
cacgcgaaca
1423741 tgacaccata atgtactttt agacactagc gacttctgcc tttggtgatt
ctaactgact
1423801 ctggcctcag cctttgatgt atgtaaagcc ttaatgtctt ccaaactgct
gtgctgttaa
1423861 cgtcgtaatt gtgtctccca gataaatgaa gctcagatag gataataatg
acccgcctga
1423921 tgattaagca gcaacatgca gtcattatgt ctattagagc aacagcctaa
tagacctgca
1423981 gaatctctga cagcgttgtt taaatgttgg ttattcttta cacgtgtcgg
cactgacatc
1424041 acccaggaag acaagagtca ggcagcagga aattgggaaa tgattcaaga
atcacggccg

1424101 aggcttccct gaaaggctga cgtgtctggg acgatcaact tatgaggttt gggacagaga
1424161 atggatgttc tccctgttcc agtgtgggtg ttctcgaggt actctgcctt ccttccacag
1424221 attctgtctc cagggaggat tcctggctct cccatgactg ctgggataga ctccagcacc
1424281 cccctctgtg taactaaaca ggaataaaaa gtggataaca gacaactcat cggtgaaatt
1424341 cagcatctgt caacctgaaa tgttattcat tgctagattt ggagtaaaca gtgaaaatgt
1424401 cagctctctg tttggttttc ggtgttgctc aaagctaaat tacaccctaa agtgaatttt
1424461 gtcggcacca aagtctggat ttggaccttg taaagggcag actccctcat tttggtagac
1424521 ccacagtccg cctccttcca tcctcagtgt tatctctgaa tgatttccaa gtgggattta
1424581 catttcggct gtaatttcca ggactcgggc ctctatagta tcatctttcc tacaatgaca
1424641 gaaagagccg ctaaaggcag agatggctcc atcaaaagac gcctctgctt gattgattga
1424701 cagcttccac tgaatagact cgcaggatta aatgtactaa aacatttttt ttttacactt
1424761 aaggaagttg gcagctaaca ttcatgtcaa actgtggtga tatttctaat ttacgttgta
1424821 gcttgaagct aaactcgttt tcattttatt aaatgtctat aagtcacaat ggacattatt
1424881 gaaggtgtaa taaagtgtaa atagtggtta ttcacgggat gtttaaaact tttaacaagg
1424941 aattacatta ttatttaaag gtctaattaa catctattgg tgagtctgga taccttttgg
1425001 aattaaaatg attttaagtg atggatttgt gtgtagtgtg ttgagtttaa tgtatttgat
1425061 cctaaaacaa atggactgaa attccatgat tgacagctca gtacctgttt gcctggtaat
1425121 aatgtagctt tagattaatc aagatcctgc ctgatgatcc actcagtgtt caatggtcaa
1425181 tgtgccgaca tgtcagtata taataacatg ttccatcatc caggtttgag ctttttaaaa
1425241 tgctagattt ggctttttgc ccgagcactg agggaatttg aaagaagaat gaattttgca
1425301 ttgaaattcc attgaaaaac gttgcttgag gtgtctggac acgcttgcat tcaaagatct
1425361 cttcttttct aatggaatac aaacccgaga aaaagccact cccttgatct tgaacaccca
1425421 cagagaccat ctcaggcagg cagaagtagg atggagagaa gtgcagactg caggatgacg
1425481 ttttaggtca catcgctgcc attactgttt cctttaagag agcgagtcag acgcggacag
1425541 agcgcagtga agggagggca ctggaggcgc taaccaaccg accgtcccaa catttctcat
1425601 ctggatttat tcaggagaaa atgactcttc agcccacaag gtaagctgtg tttggttcgg
1425661 acgtccgggg acggctttat tttttaagag gaaaatcaaa aagggttgaa aagttataaa

1425721 ttgggattgt ttgttgatgt aaaactgtac acagcgcaac aaaatagaac caagataaac
1425781 tccaaaagtt ctttcttctt ctcaactttc catgtcaggt ttggtgcttt tggatttctg
1425841 caaaatatgt tattaattta gaaaatgtca aacacatggt ctcagcgaga actcgggtta
1425901 cagttttgtg ggcagccact tttcttttgt cgttccagca acaaaagcaa aatgctctct
1425961 tacattcaat cgaacaaata ttaaaaatct acagttgaac tgccagttca ggaggtcctt
1426021 cagaaggttc ttggccaatg tgattataaa gatgttatcc aaccacctca gatctgggaa
1426081 accagcctgc aactgctatt gttattgctg atgataaaca ctctgtgact tggtggtgca
1426141 caagtggacg gagacgcaga aagatctcca ctttctaagg tttaaaacca agacccttcc
1426201 caggtttgct ctgctttaag acgttgtgga ggtgctcaga gcgctgcagc tcagttagcg
1426261 cagtaattgc acagtaagtc aataaatgtc agcagctgga aaataatgaa atttgcaagt
1426321 ttgactgatg acgtgctttt catgtagaac ttgtggctat aataaataaa ttgtcctttt
1426381 aaagtatttt gactggtata tgtaagaaaa gtttgaaata gacgtttacc tcattgagtt
1426441 gctgttgttt gaagatccca aggctgtttt tttaaatcta tctgccttgt gtccctcaca
1426501 gcctccccta cgtcagcacc acgccgctgc atggagacca gaactacacc aatgggaccg
1426561 agtgtccaga aacagatgcc tggaattggc tgtacaccaa ccagccggtg tacatcctga
1426621 tcatcaccgt catgggagtc gtgctaaacg tctttgtcct gatggttttc tgcctgcaca
1426681 agaagtcctg caccgtggcc gagatctacc tgagcaacct ggcggccgcc gacctgctgc
1426741 tgctgtcctg tctgccgttc tgggctgtca acatatggaa cggcttcgac tggcctttcg
1426801 ggctcttcct gtgcaaagcc gtcaacctgg gcatcaagat gaacgcatac agcagcatct
1426861 acatcctcac tctggtcagc atagaccgct atgtggccct ggtgcacacc atgtcctacg
1426921 gccagatgcg gaggcccaaa tacgccaagt tgggctgtct gctgatgtgg gggcttgggt
1426981 tccttctgtg tatccccacg atcatctaca ggacggtgga acacttccct gagtacggca
1427041 tacacgcctg cttcctggat taccaaaacc gcgccttaga gctgttctgc gacaccctcc
1427101 tggtggtgtt ctgcttcttt gttcctattt ccatcatctc gttctgcacc tataagatca
1427161 ttcgtgggct gaaggtgaag accatagaga ggctcaatgc tgagaaaacg gagaagaggg
1427221 ccacgactct ggtgctggcg gtcctgctgg ccttcctcat ctgctgggtg ccgttccact
1427281 tcgttaccac gctggacgtg ctgatgcgag ccgaagtcct gagcggctgt cacctcaaca

1427341 acgtcctcga aatcgccaac caaatcttca cctacctggc cttctttaac agcgtcctca
1427401 accccatcct ctacgtcatc gttgggaaga atttccggaa aaaggtccgg gaagtcttca
1427461 gccagtggag cagcaagaag gctgtcaact ccgagtcgac gcgatccaat ctctcctcca
1427521 ccgtgaagac agtggcctga aatgtgaacc gcagttcttc tcaggccact tcattatcta
1427581 aggataatgt aatagtagtg tgtcttctaa ggggaccaaa gatcctggct tatctcagct
1427641 tgttctggcc tgaaagggaa aaaaaacaac attttgttaa agtaaacgtc attagctgac
1427701 gaccaaaagg caggaccttt ctgaatattg acctccagat tacatgtaaa tacaggtttc
1427761 acgccgcttc gctcagttgt aaatagttga cttgtaaatg aatgtccaat aaaacttgag
1427821 agttttcctg ttacgttttt gtgatgcgtt taaatgagcc caaccttttt tccggcttgc
1427881 ttgactcagt ttctgagtca gaaccagtag tctgtcagag attttccagc agtgacacgc
1427941 aaacacacac acacacgtgc gcacacacac gtacacgctt ctttttgttg gctgtgtgct
1428001 ctggagctgt cgcaacacac cgctgctctc cccacgctgc agtgtttaac gggccggtgt
1428061 tgattcagcg tgggctgcgg ccgcggatac accctcagtt tgtctgcaga atgaaggcat
1428121 gtttggccaa acaggaaacc ttcgttggga gtcagccttc gccaccgtcg ctggagctgg
1428181 aaaatgctgc ggaacaaaaa caggcagacg gagctgcagg gtagaattac tgccaaaaat
1428241 acaccgattg aacaactttc tttaatgtca gtctggaata tggtgacagg aagacggaag
1428301 gagtgtgtcc aggcttgaac cagtagcttg gtcgggtctg gggaccagta aactacagtg
1428361 ttggaacact gctgagagga gaaatatgag cctcagttgc caagaacaac cacaagggcc
1428421 gggcaatgaa ccatcagaac atgtatagaa ccacatgttt agacccaact tgaagctcct
1428481 gcacggttga tctccagcat ctgtgatgct tcaagagtgt gtcggtttcc acggcgatga
1428541 ttttctcttt cccacaaggg tctctgcagt ggtctcacac cacagcagca tcctttttcc
1428601 ccctcagaca ttgtttggat ggaaccaaag caacaaattt cattctgtgg ttttaaatga
1428661 agattctggc aagatcctcc tatttgtttt cttaatgaga agattgctcg tgtagccaca
1428721 atagaataaa ttctctgtat ttttaggggc tttttttttt ttttttggag acagacttat
1428781 tataaaatga aataataaaa actctactac gactaacaat tctaaccatt tattcaggac
1428841 caggaccaag gttaaagggt taaaggttcc ctcccattct actggatttt gttaaaaatc
1428901 cacacaggaa tcccaacagc agtccaagaa actgttggca gagacaataa cacaagaatt

1428961 ttgaacttgt gtttataact cttattagag cgaacccttt taacatgggc atcttgcaca
1429021 tttctgcata atttgtaaca aattcctcac agcgaaaccg agttattgtc tcctccttgg
1429081 tgacttaaat gttagcgagc agtcaggcag ctgttagaaa cacctgagca catctggaag
1429141 tgagaacctt aacatagaag aaggtgtgtt gaatttctac agaacagtta ttaaaaaaaa
1429201 aaactcataa tgctcataat tagaaaaact cgagtaaagt aaataaataa acagcagtct
1429261 acctgtgctc caagaccctc ccacagctcc tggtgtatgc tctgattttc acaacgttag
1429321 catagctttg gcacctctat gatgtaggga agggatttca ctgtcacgta caagaggttt
1429381 gagcccacct gggtaacagt tacctgctgc tacagtctgt atgctaacat tagcctctgg
1429441 ctttgcctgt agctgatctg caacaccctc tatgtatttc aggtcaagta tctgctgaaa
1429501 gccaaatgtt tgggtgtctg gcagcggtgc agagtgggat gatgtaagaa tgatgatccc
1429561 cattcttgag gatcatcatc acacagtcac tcctccgatg agctgttgca gacagctcac
1429621 tgtttacatc agcttttaaa tgctgctcaa aaagtggtgg ttaaaaaaaa ccccacatac
1429681 acccttgaaa cttttagctt aaagctgtaa gccagcaggg cgtggcaaaa aaagagtcct
1429741 tactgcagaa aattgtttat taactaccat tctatgcttc ttttactgta tgatcctcaa
1429801 cttcctgtat tccgtccttt aaaataagct aggtgctctc agatataact cacttgacct
1429861 ttgactccat gtactagcag gattttagtt tggtggagct ttccgatgtt ctcttgctta
1429921 gttcctacac acatagtatg agaatacttc atacagaagt attctgtagc tcctctaacg
1429981 ttggttctga ctctgggact gttcctcaga gagtattcaa gttcgtcccc gctcttacgt
1430041 ggatggattt cctcgcacaa aatatgattc ctgtacgaca agaagccaga gagctgtagg
1430101 gaaaacgtca atgcttgtgt tgaccgtaaa acggacgcgg aaactccgtt gtttgctttt
1430161 ataataaaac gtctcatgac atcagatgaa aagatatgtg gtctgtgtga ccctcttttg
1430221 atgtttacat gattcacaac atttacagac gattgctctg ttgaagaatt acagcttata
1430281 acccgtagac aaccttctac atgaatgtgc acgatcttta cgtaagtcgc cgcctcaaaa
1430341 atgttgttta ggtataaaag ttattgggtt ggtctcacgt taaggatatt ccatccgctg
1430401 atgaaagttg ttgacagttt tatctggtca gctccacttt ggtgcgttgg cagcttcagc
1430461 ttatctctaa gacccacacc cacacacaca cccacacaca cccccccccc cccacacaca
1430521 cacacataaa attctgactc tctgataaac aaaatgatgc attgaagttc tcggccgagg

1430581 gtgtggaaga atgcaagtct gtatacaaga aggccgggag tagatctctc ccctcactct
1430641 ctgtatattc ttaccacaag ctcagtgtat acgcacacag acacacacac actcacacac
1430701 acatgagctg ggttccagat gcatcttctt gccgccacag gaatctttcc cggtgatctt
1430761 tggtaagagc cttttaactt ctctactcag cggtttgttt cttttggttt cacctttatg
1430821 gatctgacgc ctagacggtt gtttctgtat ttgttggaat ctgttgctga ctggttcttg
1430881 gagttctggc tccctttcac cctctggtct cctcatgtgc atgcgtaaac gtgcgcacgg
1430941 ttcttccagg tgaattcttg aacgcgcccg tccgttcctt tgtttgctcc ggtcttgcat
1431001 gcgtgaaagg ctgcacgttg tcgctcgcga ctcttccaga ttcaccatta cagcttttcc
1431061 gtcacaccct gtgcacgtgg gcgggcatgt gtaaacagaa tccggtgccg aacttcaaag
1431121 acaggtaaac gcaggctgct ggcttaaatt tcagcttccg catgagtgaa acgcgcagaa
1431181 acccgtgaga tgcaggcatg agggtgaaaa cgtgcacgcg ggaactgtct ttttaaaaca
1431241 ttttcagtgt ccagatgttt agtgatgagt cacgtctggc tgttcgcttc tagacatggg
1431301 ggtggaggag tttgcacctc tgaccaggct gtggcaggaa aacatttcca tcccatccaa
1431361 ttcctcagac ccttccctcc atgaggagtg ggatctgatc tatgccatca tcccccccta
1431421 catcttcacc ttatgcctgt ttgggctcct ctttaacgcc ttcgtgctgc tggtgtacct
1431481 gatccacaag gaccgactga ccgtcgccga gatctacctg agcaacctgg cgctggccga
1431541 ctttgtcctg ctgtgcggcc tcccattctg ggccatgaat atcctgaacc agtacaactg
1431601 gccttacggg gaagccctgt gcaaactggt caactcggcc atcgtggtca acttctacac
1431661 cagcagctac acgctggtca tgataagtgt ggaccgctac ctggcgctgg tgaagaccat
1431721 ggaagccgcg cggctgagac ggaccctcta cgcaaaggtg atctgcctcc tgctgtggtt
1431781 gtggggcctc ctgctgagca cgcccaacat gcttcacaga aaggtcaagt tcatcgagga
1431841 gctgaggacg acggcctgcg tgctggacta cagtcatgga gacccttgga agctggccca
1431901 ccaggtcctg ataaacgtgg tgggcttctt ggtgcctttt ctggtcattc ttttcagcag
1431961 cgtgaacatc ctcaggtctt tatcggagag gagagagagc tccggcttcc gtgacatcaa
1432021 cgacaggaag gccacgatgc tggtgtatgc cgtcacgctg gtgttcttgc tgtgctgggg
1432081 tccgttccag gtggtcgtct tcctggacat cctcagcgat gcggaggtgc tggacgaggc
1432141 gaagtggtac cccgctctga acatcgccgc ccaggtttca gtctacctgg ccttcctgaa

1432201 cagcgccctg aacccgctgc tgtatgtctt ctccgggcag tactttcaga ggaaagtgcg
1432261 agacatctgc aggaggaccc ggcacagccg cagaggttcg gacatgacga cctttcagcg
1432321 ctccgtcgtg tcgacttaca tcaacagaac ggagcaaata aagacggtgt tcatccccaa
1432381 tgaaatggtg acctgagact gtgtcatcat tgtatccagg aaatgagact cctcacattt
1432441 aatgtgcaac aattatgctt taataaagtt acgcagcact attggaggat gtcggacatt
1432501 cgattcctgg agaaaagctg ataatggacg cagattttag gatttcagct tccattagga
1432561 cactctgctg atgtttaatg tccaaaaaaa cacagacgac agcaccaaaa atcgttcaaa
1432621 ctgtttgttt ggactcgagt cagtggaatt ttttattcat aatactagtt tagaacgcca
1432681 gactactgag tccatcaaaa aatgcagttg tgaactctaa ggaccaaatt tatgcatttt
1432741 gataactgca gagatgtaaa aataaacctt ttctgtgacc cgtatgttct gaaaaataca
1432801 cattgtctgt gaaactgaac atctgcttta tttaagtgta tttactttat ttttttcagc
1432861 tctcccaacg caggaaacta gggttaaatg gcttttacgt tattacaaaa tcagatggta
1432921 gctcttaatg cagcttaaaa gattcatgga aaactctgca taaaatattt ggctaaatat
1432981 tcttttcact gcttaagaac gtgcagttta gcttaaatgc atttagtttt ctggtgtaag
1433041 tcactgatgt gtctttattt tgttgtgctt gatgctctaa tgtgaaatct agcagcattt
1433101 tttaatcaaa ggtgattttt gccacaaaaa agaacatttt tgccatttaa tgtcattaaa
1433161 aatactctat tttgcagagc tgagacctgt ttaatctcag gcttttgcag aagagacata
1433221 tatgacaaat atgcttcaga agatccataa acagagttat atcagtgttt atcaaaattt
1433281 aatgcacaca aaatgagggc attttctgaa tatgatacat ctaacatcta cttttaccgc
1433341 agagatgttc aaagaaagtg acaaatgcaa aaaaaaaaaa aaaaaggggg gggaactgtt
1433401 ttgcttgcat gcaagaaggt cttcagcctg tctgaacctg acgtcgtttt gtgggcagag
1433461 cttcgaccct gcagggccct taaactttca cgctcattca tcacgaagct gccgccagct
1433521 cacttccatc agcgtgatgc tttacagcat ttctcacttt tctttctgat ctcgtgactt
1433581 taataagcca agtctcttgt tctgctgttg cacttctggg cctctgtaaa tgatggattg
1433641 catccattca cacatgcagc caactgtttc caggtgatct gtgaatgact gaacgacact
1433701 gatgccacct ccagctgtga cacgccgcct gtgcgtgaag agcctcgact caggagtgtt
1433761 cgctaattta gaaatgcatc tgtgagagaa aagactgact ttttgcatat ttggccttcc

1433821 tccacggcgc cctgagcctg cgacttgtct ggaaacttcc tcggggacgt
cactgagaag
1433881 aggcatcaca aattttctta tatggtcaca ccaaagcgat gttgcaccct
gtaacgtcaa
1433941 cagacctgag gtcaagcaca tggaagtgct tgagggagga ttcgctgctc
taaattgttg
1434001 attttatgtt acttgcggtc caactttcaa ggttctgaga cggaagcgat
ttgtcaatag
1434061 gttgctgtaa aattagaaaa tgacataaat acaaaacagt taattttctt
ttttcctgtt
1434121 ttcatccatg tcagttcttg tgtgtcattg ttttctttct tagttaagat
tgtacactga
1434181 tttagcaaag ctccccttt agaacacttc ctctgttaat tagtgttatt
tattctcaga
1434241 tgttaattta ttgcagttca tcctcatcag tcgtatttgg aatgaaatat
ctctgaaaga
1434301 aatgggggaa aaatgacagt atttatagtg tttgatcttt acgaaccagc
agaacagctc
1434361 tgcggttgct tcaggtaact gacgtccgta tgttcccaat catgccgcaa
gagggcggta
1434421 gaatccttct tataatgtta ttcctgtttt ttgttttaaa tcttgaccac
atgacgctcc
1434481 cagcgaggcc agtggacccg ttccagtgaa aataccaaat gtgatgcttt
aagtaacaat
1434541 gtaagtctaa attatatagt aatgttttgt aaaagacacc cttctcgagg
tgataaatata
1434601 ggtgacaggc ctgactttgc tttccccata attgaatgcc aattggacgt
atgtatctgt
1434661 tttacctaat cttttatgtc attattaaaa catacccatt gtctttccag
tgctactatt
1434721 tctgctgttt gttgtatgat gttttgactc aaggtcaccc tcaaggtcac
ccccccatca
1434781 tcggagtcta cagggtttaa cctgtgcttc tggatgcagt ttgtccccag
ttggctcccc
1434841 cagtgtccag aaagtgaaga atccccaggc tggagacaga cgaggaaaag
agcccagctg
1434901 gcaaacatct gggggccaca catctgttat tgttcctgac ctggctcaga
cttccccatt
1434961 gtgcccacta tgtcaccgga ggggtgaagg aagcatgttg aggaacagat
ggtagaaatg
1435021 ctgctcacca aaagtaaata taatagctac caaaaggctt ggtttactga
taaaaaaaag
1435081 aacgcccttt gtttacattt actgtaacac agagctctga ctgtggttct
tacagtttct
1435141 tcctcttgtg ggcaacaata atcagacctt aacctgcatc tgtattgaca
aaccaaccat
1435201 aaaaacagtc gatggagcat cgaaattaaa agctgctgtg tcaccagcac
cggtagtttg
1435261 ctctatcacg tatttaatgg cactcggttg tcaggggcaa caagggggtt
actgccgctg
1435321 ccttttccct tttcccatag tcgactgaat gagcctgatt cttttttaat
gtggccgcct
1435381 cagagaaagc accttttatg gacgagacac tgtcaacaac agccacaacg
tgagtacggc

1435441 ttcacattta tttagagcgg gtttgagctt ctgcttctgt ttagatgcaa agttataaac
1435501 ttttagagct tcagctaagt ccgcaaacct cccaccaaga gcattttttt cttagattct
1435561 gtcacacctt gcttttgccc ctttttcaaa aaggcacggg cacatcatcg ccacgccaat
1435621 ggaaaacttg tccctggaag aaagcttttt cgagtgctac gtggctgaca acacatccag
1435681 gaagagggga tttatgttct tctacatatt cgtcatcgtc ctcacggtcc catccaacgc
1435741 cttcgccctc cacgtggcct ggaagcacat taggaagaag aacgagcttg gcgtgtacct
1435801 gttcaacctg gcgctcagcg acctgagctt cgccgccggc ctcttggtgt ggttggacta
1435861 cctgtggagc ggcgtgtggg tgcacggagg cttcgtctgc atcttctcca tcaacattct
1435921 cttcaccaac ttttacacca gcgaggctct gctctgctgc atcgccttcg accgctacct
1435981 ggcagtggtt catccgttca agtacatggt ttgcaggaaa atcaccacgg caacagccgt
1436041 cagcgctgcc atttgggtgg cggtggtctg tttcaacgcc accaccatca cctgggacga
1436101 ctcctaccaa gaggacggca gccactcgct ctgcttcgac atcgtcctcc ccatgtcaga
1436161 tcacctggct cggatcagct tggcacgctt cttcctgggc ttgatcgtcc ccgttgtcct
1436221 ggtgatgttt tccacctgga ggatctacaa ggcggtggga tccaaccagg ccacagagga
1436281 acaggaacgc aagcagatct tcaagctgct gacagtggtc ctgctatgcc tgttgatctg
1436341 cttcggaccg acacacgtca tcatgctcct ccagacacaa gtggacgact gcaggaacgc
1436401 aacatggctg ctttatctaa acaagatcag cgtcgccatc tccatcctca accctctcgt
1436461 ggaccctttc ctctactgct tcattaccaa atcggggaaa gcaaacatca gtcagctcat
1436521 tctcttctgt aaaaagaaaa ggacacgcag agttagcgtt gaggtgtaga acgttaagtt
1436581 acactgagac acacgaggct cgggtttagt ccaaaagtcc atttttttag tattacagat
1436641 gatgccagta cctggatttt agctggcttc tgaaatgcag ttctaagata gcaaattagt
1436701 tgggtttttt gcacaaaagg agccaagcat tcaaagataa aacatttcca ttcaatttaa
1436761 atacatagaa atatgctgac agatcagcat aaacatcaac tttacagaat tcagagggag
1436821 ttgatttgtc tctctgaccc acatctctgc tctgctatgc tttttttttt taagaaataa
1436881 atgtgtttcc attcaagaca gtttgacatc attaaacaaa tgaaacaacc ctttattaca
1436941 atgctcacac atgcagttgc ttttatcttc gccttcaatt aaaatgcaaa gcaataaacg
1437001 gtctgtttac tttgtatgta tgcctgaacc tttatcagct gtgagccaca aggcatctgt

1437061 agcgcattct gttgaccaaa atgggtcaag aaataaggaa atagaaatgt gaggacattc
1437121 gtcataaaat gtgctgtata ttgtaattgg agattaaact catctcactg ctcactcctt
1437181 atttaacctt ctcattccca cgttgacccc agactaattt tccatgtggc cagtttaatc
1437241 ctccgaactg attttctttt tctaatttgt ttgttcagat gtctacatag tagtaattag
1437301 ggaatgtgat gcccagagtc agcacgctaa cgttaaatta atggtagagt ttatacctgg
1437361 aagaaatgca gttaatacga ttgtttctaa tagaattgca tatttagtgg tgagtgtctg
1437421 ccacaggaaa atgttgcaag gaaaatgagc gtaatccaag actcctattg ggcaggtgtg
1437481 gataagagca acaatacact gagtcgggca tgaacgagcc ctctgaccat cttgaaagct
1437541 ctaaatgagc tttattgtct ttatttgctt gcagggtaat cctgggggat ccccaccacc
1437601 acctgtaatt aattgtattg tttgagagtc acgttttcaa tagcaaaggc tgtttattgc
1437661 actcatccat gtgcgtcctt gtcctgtttc attcttcgct ccatccatcc attaaaaaac
1437721 atgtctctca taacaatgaa agacctgcgt cagcaaaacc ggggagggag ccataaagcg
1437781 cacgtgaaca gccatccttt cacttcccac ctcctgctgt ccttgatgag ggaactgaag
1437841 gccttttaga atagagaggg cacacttgaa cttgtttaat gttgcgtcac accgtcggcg
1437901 cttctacctg caacgtgctt tggccgattt tcagcagcgg cacgtgtctg cgagtctgcc
1437961 gccgctgagg cgtgatgaac ggccgtggct gtaacagcct cgtaatcaaa gtcaaacctt
1438021 cagtggaaga tccgtcggta tttgtcgaac cacagtcaac gtacagggct gtggtcgcca
1438081 ttgctggctg ttgctatggg aatgagtgat gtagtatgat atactgtgcc cgagctgccg
1438141 ggttaatttg aaccaaataa gacaggtgct aaaaatatct gtgtgtgtag gaacattaca
1438201 taatgtgtga cgtggaataa gattatttta gggggtactt ttaatggaat tacatttaca
1438261 ttcaatcagc tgctcctcct acactttcac tgttgcctgt atgaatgagc cattttgaat
1438321 aaaccaagga aaataaagcc tttcaggtca ttgttatcgt atatttttgt ccaagggcat
1438381 caattagcat tcagctcttt attaatattt taaaagtgca cagtgtgtgt tgtgtttgta
1438441 cttgttgccc gaaactttat tgatctgcag aagccttaca gtcatcggct cctacttctg
1438501 tctcctctat gatttccttg tctccttctc tggcacctcc tctcctcctc taactgtgca
1438561 acagtaccgg tgtgatctgc tcctcttcac tctacaggct cacacacgta tcgtacactg
1438621 gactgctttg gaatgtcgtg ttctttcgtc tcctttgcct cgaccatctc cgctgcctgg

1438681 aggatttctc cctctcctgt gagggtgttg actgggctcc tctgggctgt ctgacactcc
1438741 ctccaatgtt tcctcctctt catcctccct tgtcacattt cgacctttct caaagcaaca
1438801 gcatctcaga atcatgaaat cagtgatggg agggtgagaa ctttgctgaa gaagtataaa
1438861 ggatgaaggt tcttttgttg gagttagatt tttgttactg tagcattttg ttgtgtatga
1438921 taatggttat gctgtgtgtg ctggatcatc ctctgcatca cagagaagtc caccaaaaca
1438981 gcaaacaccc agcatccaat cagacagcat cagcaccaga gatgacagga aggcttagca
1439041 atagaagctg tttatggaac ttggagttaa aaaacattaa gaaatatgat aacaccatca
1439101 cgagtgagaa acaataagat tcccaccctt tagaatctcc tcctcctttc attgtaacag
1439161 gtcattacag gtagtttttg gtttgacctc tgacctttcc acagacggaa taactcatca
1439221 atcaatagtt gtgtgaaatc tcactttatt ttttattctt tactcatgat atagggagtt
1439281 gggctgagca ttactttccc tgcattcact gatgacaccg tgccgcttaa tgaacataca
1439341 tcagatcagc atccgcattg gatgagtaat ttccattttt ttaatgaata atgtattttt
1439401 aaaagctttc tggccatttg tatgttgatg tactagtagg ccctaaatac tcaattcaaa
1439461 atctaagtag tcctgtgggg gacagggcta tcttcgggtc agcaacccct ggcgcccttt
1439521 ccacatttgg aacacttcag aataattagc agtctgttga caataagcac tatgttctat
1439581 atatctttat cagtggagtg gtctgtttta gtattttatg gaggaggcgc aacattcact
1439641 cataggctgg ggtaactaat ggtaaaggtc actagcaggc tgatcagtgt cacacatgat
1439701 aggatgcgtt tttaatccag cccaaaacct caaggtagtt taatctgatt ttattttgta
1439761 tatttgctgg agatgtgttt ggttaccttc gttttagcaa attccaaact ggcctttcat
1439821 gctaaaatta ttgatttatt agaaatgcgt ttgagattga tttaaaaatt agttatattt
1439881 tcggtttgtt cctcaagtca tataaacttt tcccttcgtg gtagcagtga ttaacaccca
1439941 gataaggatc tcacgccaac acctgtatga tccagcagct tctttatgtt ttcactgttg
1440001 caagtgtggc gttgattatc tttccgattt taccggtgct gctctgctgc ttcctgccga
1440061 agtgtctaaa acatcttgca catgagtcat cttgtgaaaa taggtggggt tttttttcatt
1440121 tcagattaaa ttaattcatg atccatcaga aacagcaaga tgacgggaaa cactcatcag
1440181 agggctgttg aggtcacttc caccttgaaa cacacacaca cacacacaca cacacacaca
1440241 cacacacaca cacacacaca cactctctct ctctctctct ctctattcgt tttctacatg

1440301 aaatttacag ccctgccttt actgtcctgc ctcactgatt aatttccttt gtgccgttgt
1440361 cagttgggcc catttcctgg gatgatttac acattttgac acatggaact ctaaccaaag
1440421 gcttaatgaa gtaaaaggat tttgacattg actaattata gttttgaaag tagaggatga
1440481 gacaagaaaa aaagaaagag aaaaaacaaa caaactcaag tctgttcagt ggatttgtca
1440541 tttgtactaa ggtcactgtg gtgtgatttt gttatatatt aacattaaag gacaccttaa
1440601 cagcatgcag agaaagcctg gaaaatcctt gcaaaccatt ctcccacctt tggagagact
1440661 ctgatgagtg ttttcagtag cagcagttct aacgccagat ggtcactgct agaaaaactc
1440721 aagtgggaga gaattaatga tgccaagaac ccatttagtc atttcagggc ttcctgtcac
1440781 aatgccaaaa ttaaccaact actaacaccc aacaaagttt ttatagtaat tcctttggtt
1440841 tcctatatga aaatgatatt cttgctttaa tgatcctcca gtatacgctg cagggccccc
1440901 agcagaaggt tagggttaag gtgagggttg ccactgaaag agtcaccacc tttaaaagtt
1440961 catatgtcat gtttattgtc tggaaaaaaa aggcactcac tacatttaaa gtgatccagt
1441021 taaataaaac tgtccttaat aaacgatgat gatggttaaa ggacgaataa taatcagcgt
1441081 gagagggttt gatggaagca gatttggaac tgcatgcaac gcacgcaatt tacacatttc
1441141 agacttctgt acagttcaga gtttctattg tctgtttgca aagcaagaag aacctttaaa
1441201 atattcttta aaaaacgaga aaatgtatac agtatttaaa aaaatcatga cacaaatgtc
1441261 ttttgttcat attttacaaa taaattgaaa tgtccactca agtgctccac cagtgtaaaa
1441321 aagggaataa acacagatgc aaacatgctc catgagtaca aaaaaaaact tagcatcaaa
1441381 tgcatgattt caaaagccac tttcaaaatc tgaagacatt ggtgacacag gtgttttcaa
1441441 cattattgac aatctcatgg ccaaaggtgg tcaaacgtgt gagtcgggtt ggtgttgatt
1441501 cacagcaaat aaaaaaggta ctaaaatatt tttttccatg aataagaccg gaacccttta
1441561 aaaaaaaaag aaatgaaaaa aagaagctca tttgaagcct tgatcgggtc tggttgtgat
1441621 tggaataacc atgcacgaca agtagttatg tagctctttg tatagttttt gcccacagat
1441681 gacattttca aagtcagacg aagtggagga ataactctac atgtcagctg gtcagacgct
1441741 catactgtct attaatccag cagaaacaca gccccgccct cctctcatca aacttggaag
1441801 tccctctgtg acgggtgcta aaaatagatg acaacgcaca gatcgataat gttttaaatg
1441861 atggatgaca ttaaaccacc tcttggagag tccggcccat atcgctgatg aattagattc

1441921 aacatcagcg gacagctgtt gccatggcat cctgtagaaa gtctgaacgt cgctcattct
1441981 gtagcaacat ctgacatgag gtatttagct acagaccctg tatgtctccc ccattcatac
1442041 gtttttggca acgcattttc ttttccatgg cttatggcac gatgacttga atttcctcgg
1442101 agcagatcca accgacgcat cggaaacagg aaggcacatt tctggagggc cccacggcaa
1442161 ttccgagtgg ggccgtcgct cagagcaaac ccgctccatt cacttaaaga tgatcgggtc
1442221 ggcctcaaag tagcagagga ccggtggaag atggcggtga cgcgtcagga caaacttcca
1442281 gtattctcat cactctcccg gagtcaaatt tttagcaatc aagtgtcctg tgcagatggt
1442341 ggagttgtgt ttccactgaa cagttcaggt cggatcagtg agcaagagta tttacgtata
1442401 tatgcagttt caaagcagga accgtaccaa aataactgat ccgtactgtc cttctctttg
1442461 gcaccattcc acaaggaaac cacgatgagg agatggaaaa aatcaatccg agagagaagt
1442521 aatgattctc tggactcacc acagtgagga gcattgaaga cttattgttt ttcaggttat
1442581 aaagtcgatg cgtggcagca tgtttttcgt catttaagtt attgatgagt tacactgcaa
1442641 caaggctgcg acaaggtcac tgcactcatg aagccgaagc agatgttaca tcgaaacatt
1442701 ggacgaattt cacaagatct gggcgcacaa gatccacggt agcatgttgt gcaggtctaa
1442761 actggactgc ttactggaaa tagggcatac atctggacca gaaacacctg ggatgggttg
1442821 tttttcatt tcaggatgtc tctcctctgt ctctaccagt taatagctac tctttttcaa
1442881 tatctttggc cagcgctggg gtcagcccat tcagcttgct gcggtccctc gagtgctgcg
1442941 acagatttgt caggctggtg tttccctctt tcctctcttc tttctcctca ttttcctcct
1443001 cttccgctct ctgttccccc agcatgacgc tgcagttaat gcctacgctc gcccgccgga
1443061 cgtcttcggg gatgttcctg cgcatttcgc tgttcttgtt gcgtctggcc agcttgttga
1443121 gggaaccgaa tctcatgttg aagaggttgt cctctgagga cgaggctgtc tgcgggctct
1443181 cctcgctccc cgggtggttg tactgctcgc aggcgccctt cagcttcagg ttgttgagct
1443241 tggtgtcgat gctctcctgg gaggaggtct tgaagcggtt agcgtccagg ctagcaaaca
1443301 gagcccgctt ctctggggag agcatatcca gggagtgagc tcgctgctcc aggcccagct
1443361 gacggcgttc catgctgcgg atggtcgcgg ctcgctggag cttgtcgagg acttcgacgc
1443421 tcagacgccg ccgtgtttca cgcagctccg cccgcacgtt ggctttccat tcagctgcat
1443481 gggctttaaa ctctccaacc tgcaatgaga acagcatggg ggaattaaag ggagtaaact

1443541 ggcaggaacc tgcttataaa cctggaaaca tctcctcgtt gatagtaacc agggtgagga
1443601 ggtataaaac ctgaagaata ccgtcacact tgtgtgccca gattaaatga caacagctac
1443661 agccacgttt ctcctcacgg gttgtgatct tcaccaggcc attctggcca ttaccaccca
1443721 ctcaaccatt gctgatggca tcataaacca ctgcatcatc aataacccac ccccacccca
1443781 tcattgtggg ttttttttta gatcagacca gctcccgcag ctctgacagg taatttcaga
1443841 tcttttctca tgtcatgcgg cagcgcacgc ttccaaatgt ttggatctat ttcttgagtg
1443901 gatgtgctct gtctgcagga aggatatttt tccccctcca catgtcagaa gccagggtta
1443961 gaatatgaaa cgctacacta aagacacatt tagatgcctt taactggggg aaaatatgtc
1444021 aagcaacctg cttcagaagc ttacaataag atcaaagcag cggcagagtg accttttatt
1444081 gtgtgcctct ctgattcaat accgagcggt gtaattaaat gaggcccacc tcctctttgg
1444141 tctttttaga cagcactcga agccagtctc caatcatgct gaggacggct gcaaagtacg
1444201 ccaaacccac caggatccag aaccacacca gaggcttgta ccagttcatg tactcgatcc
1444261 tcctgtttcc ccctgaaagt gcaggagagc cttcattcag tctgagaaga aggaggacaa
1444321 aagaatggga tttcgccgga ggaagactgc acctgccaca tagtctccga tgcccacggt
1444381 ggtgagcgtg atcacaacaa agtagatggc ctccagggcg gtccagccct caatgtgttt
1444441 gaagatgaca gcaggaatgg tgacaaagac gatgcagccg gccaggatga agaggatcgt
1444501 ggacgtgact ctgattttag tctggctgat ttgcctgtgt ttttgctgat tcaagtagag
1444561 aaacggagtt acagcaacaa taaaacaacg tagttgcaag ttaaagtgca caatgctgct
1444621 gaataaatga ctaaaaccaa acaaactcac tctgaaaatc ttctcaactc tcagaatgct
1444681 cttcacaaag atggttccca gctggtcacc gatccccgcc agcagaaagc caaagagcgg
1444741 gatgccaaaa atggcataaa gaatgcagaa gatctttccg ccctccgttc ttggcgctat
1444801 gtttccataa cctgttgcaa ggagattaac agaaaagact gtgaggctgg ggcgtgggtg
1444861 agtgtgtggg ggtggggggg ttattaggtg taagatgaga agcagaagac agtcaagact
1444921 ttcttatgca acataatcaa ttgatttggt tatgtaatac ttcctgcagg agtctgcagt
1444981 acataagctc taagagcaac actaattagg tttccttcac agctaattct gttaactggt
1445041 gtcctggagg tgtggacaga atgaatgaat caggggacag agactgatcg ggaacataat
1445101 aatcaaggtc acagtcagtt acatctgcat gtgaagtaaa agaaaataat aatataatgt

1445161 attaataaca acagaaccct gatgaacaca tagtagttaa gtagaataaa agtaaacttg
1445221 tggtgtggtc taaaaatgtc acctatggtt gtgatgacgg tcccggcgaa gaaaaaggcg
1445281 ctgcccacat cccagtagct ggagttgtag taggagtctc tgctggggct aactccagcg
1445341 ctcatggcgt ccatggcgtg ctggaaagac caaaacgtgt cagttaccgc cacggacggc
1445401 agcactcaag gtcagctgat ggcaccgcaa caaacgcacg tgtccgtgtg gatttgggga
1445461 gagtgagcgc gtcagccgac cccctgccaa tgtttaaggg gagctgtgta cgggagtgtg
1445521 ttatgtgatt gtgcaaccct cctgagacaa cgcgtgagga cggcacaaca ctctcagatc
1445581 cacgtgaagg gtcacgcaac ctgataaatg aggaggaaat ttcagcatcg cctgtcacta
1445641 tgtctctagt cggatgcatt gggagctgca cgctggaggc agaggtatgg atcacacgtg
1445701 cgcaaacatc tgccgggaca cgagtgtaaa ttattcagtg tttgcggtct gtttcagagt
1445761 tcacactgat ttacagcttt ctggccgttt cattttctct cttctctgtc ctgcagcttc
1445821 cctttgtctc agcgtgctgc tgttgtggga cgacatgaag aagagccgac cttccgactc
1445881 cgctgcatcg ttcctctcgc tgatcggttt cggcgggtag cggagattcc cccctatcgg
1445941 ctatgaaacc tgctgtttgt cttcctccgt gttgcagtag gttgagtatt ggtcccagtg
1446001 tcattattct ggtgaccctg ccttcctggt catggccccg ttcaaaccaa actgtaagta
1446061 agtgagctca cgtgagaggt ggaactaatg aagtatttac caactccagt gacacacatt
1446121 ccaaaggagt gcagagcctc ctcacgcatg gctgcacatg cctactcaca aaagaacatg
1446181 tcatgacctc agctccactg cagccctggt aatcagtcca gtacgggtat ctaaaattag
1446241 ccttttgtat tcatgtaatt ttataaaggt ttcagcagat gctgaaactt tgaaggggcc
1446301 agaatcaaag tggtaattat aaaataaacc ccaaataacc cacttttgga gtctggggag
1446361 agctaaaaat agctctcaaa tgttactgaa tactttataa gatggtctat ttttgtctct
1446421 cctcttcctc acagccaagt tcacgctgca ttataaatgt ggaaaatcct ccttctgcac
1446481 atgcacaacg gaggcacgag gataatgact ttcatttgcg ctgcgcccca gtaattacaa
1446541 agtcttcagc ggtttttgtt tcttgcccag cttttctctc attttgacgg ctgccgcagc
1446601 cccgcaggca ccgctgcttc ttgctaatgg ctgttttcat tttctacacc acaggttcat
1446661 tagagaatgg tgcagatctt catccaccct gcgtgtcatt gtggcccaca gcgggcgacc
1446721 cggtgctgat cctgccagcg ggtgtgagtc acactttccc ttggagctga agacgagtgc

1446781 tgaaccttca ccgtgtatcc accagcattt caactgtcac tggacaagta cttaactgca
1446841 ctcagatcag gtccaatagg aagccttgct gtatctttaa ctaaagtttt ccttgttgaa
1446901 actcacacag ggagggctgt attaaaggtg cagtatgctg cactgcacct ctgtaagaca
1446961 cttccaaaga atatgtaatg tcaatatata aataaatgat gtcagtgatt catcttcaca
1447021 cagcagtaaa aatagccgca gttcataatt atgtaactga ggctctaaaa agtttctgca
1447081 actcaacctg aatttaaata caggtagcaa agctttgtaa gcgggtgact caacaataaa
1447141 ttggtcctga atggtgtcga taacgacgca gattcacatt gtgacagcgt gagtcacccc
1447201 agtgaagctg agaaagttca ttagagtcgc tgttttcgtg agtgcacagt gggtagatgg
1447261 acaggacacg cgaggaggtc acaacacaca tacctgcgtg ggcacgccac tccccaaggc
1447321 tgccattttt ctgtcagttc ctcgaccaaa gtgtgagtca ccactgctct gttgccagag
1447381 aggatctttg tgtgtgcgtt ggcacagaca gaccaataat ggaacccatt aaaacccatt
1447441 aaaagatcaa acaaaccttg taagtctttc agtcattgtg aggcatcaga gcgccatttc
1447501 tacaaataaa taaaaatgtt tgatatatat tttaatatca gtaatctatc actaaatagg
1447561 aaatcatcat caaatctgat ttaaaaaaat gaaatagact aattcgattc ataaatgtaa
1447621 gcgttgaaaa caaaacttaa aaacacggca cagatccgaa caacactgta cgtcccaata
1447681 ttcaggattc cttttcatgt gctgcgcttg tgaatcttgt gcagttgtat ggacagataa
1447741 gctgtcagtg gctggtgcga cgcgccggcg ttgccaacat catttcatgc tactgaatct
1447801 ggatgtttgt cttagtcggt ttcgtctgtg attcagctgc tgctgcagca ggaggtctgc
1447861 gactgtgcaa tgaataattg attccctctg gacttcagtc acatcacata ttttccttag
1447921 aaaatactag aaactactta ttgatttcca cccccccctca tttttttcat tagaaaggct
1447981 tttgggactg aagggctaaa gagaagacaa ttctcttaga caagagctac agaacaaagg
1448041 tgggtgtgga gaggaaccac tggacagata cgcctcccat gtcagatgga accgttctag
1448101 cagaaagtgg agcacggcaa attgcttgcc ttatgggtta cttgaagttg agagaagcag
1448161 ggggattcta gggatgatgt gaatcattta ctcgtctcgt gcgacaggac agcgtaagaa
1448221 aaagagtgca tttttacaaa tggatgttat ttttgaacat ggggtgttgt cgtgtgctga
1448281 aatcctgctg aaatcctgtt gagatactgc tgaaatactg cttcagtagg gctcaaaacg
1448341 atgatgacgc tgataatctc ttaatagaaa acaaatacgt gattcctcta ttcctgcctc

1448401 tgctttcaag agcacttcaa ctttgctggt aaaacataga aaaatctgta aatgggacgc
1448461 tcttcattgt attttaatgc ccacagtttg attaaaacag ttcttttcaa agtcatttgc
1448521 agctgccgcc aacatctctt cagttttgac agacaatctt gactccaaat gtttcgtgct
1448581 ggtgtagctg caggctagtt tccttattta aaatgtgtgg gagttggtaa agcttcactg
1448641 tggaatttga tacagcagca ctgaatgtat tattgagagt acagtactcc gatacgcatt
1448701 tgttatcaga tatttgttta tttcacccat tgagctgtta catgttgggg aatcagtttg
1448761 ctgaatgtct cttttaataa gcaagcatag ccagtaaagg tcaaccatta ttctacataa
1448821 ggagaaaaat gtttatcaca aagacagtta tctgcccttt tctcccagat ttatgaatca
1448881 cgttatgtaa cttggaaatc tgcgctataa ggtttgccaa tgcgttagtg tcactgggaa
1448941 ttatgggtat gtctgcggtg accctgaagg tacccttcct gctaaaggat ggagctcttc
1449001 ctgagtctgc tgctgacatt tattcatcaa gtgtcaacga ccaggggggg agagagcagg
1449061 gggcgtatca tccgatggag ctctactcat tataaaccac gttttaatga ttaatcatcg
1449121 caggaatcag ccatggctta tggattataa tactataatt gcgcccattt cacctgctca
1449181 tcaagcagta atacaagagc attttattag gtaatagtaa taagtgagaa gagattataa
1449241 gttgtttttc taacctttg ttgccaatca gttaaataac ggttaataca acaactgtca
1449301 ggcggagtga tacggtctgt aaaacgtccc tctcgtctaa tcaacatact tggctggaac
1449361 taacacacat ttgtgcagag gacataaata aaaagtaggg ttcgactata atggtgcagg
1449421 atgagcagag caaagggaac cgaagtgtaa gttgcaggaa ctgtagtgca ttacagtgac
1449481 cccgaccagt gaatagaaac tgatgaggag aaaacacaca accagtatga gaaaatgtgt
1449541 atgaatatcc agttagggtc agggttgggg ttttccatgt ggcctgaatt caaagttctg
1449601 acctgtttag actgctccat gtgtgggaac tactgtaact ttcttaatga ggtgacattt
1449661 aaaacctgct gtcatgtgag aatcctggtt ccattcagtg atgaggctca tttactattg
1449721 ggggacctct aaacttcagt cattattttg agggaaatca acaaaaagtc ttgtaacaca
1449781 tctagtttga cactaaaaat aaatatatat caaatgtatt cctgttattt actttgctgg
1449841 accgatccaa tcacgcgacc ttgtaacata tagcttttca catgttaagc tgaatgtgtg
1449901 tcagcaatgc gtgtttagaa atgtaagaca tgacgctcac gtgacttcca ttgtaacttt
1449961 tcagactgtt aaccctgaca tcaccttccc tttttactac tgcagttctt ttcaaaatat

1450021 gcaaatgttt gtgtcaatga atgttttctg tcacggaggt cgaagagaac actcggctta
1450081 agaaatttta tagaaccgcg gctattatgc taatgaatac agagggtaaa gattccattt
1450141 gcttaaaggc tctctgttca tgaaaggacg cagcgttcac ctttgctgga gattatctgt
1450201 cgagcaaatg aaaccaggaa caaattagcc tgagagacac taacgatccc tgataacaat
1450261 catcatctac tgctgtttta aaggtcaatg atctgcctct cctttatcgg atctcaataa
1450321 acccttaaca tttacattta attttctcat cgccagatta caaattaatt tcttgcccaa
1450381 caaactctgt gtttatgccg ctcaccttga ttagcacctc cagctcatcg ggagtgacgc
1450441 aggggtgttt cttcaggaac agagccttct cgtgggtgat cgtggtcttc tggctgctct
1450501 caaattgctg ctccagtgcg ctgaaggcca gacctccgca aaccaggtag agcaggacca
1450561 cgatgaacac ggccagcacc gtcttccact tcatcaccga gtggagcgcc gtggccgagc
1450621 cgtcgcagtt ggtgtccatg ctggccacca cgctggcaga gcgggaggag atggacaacc
1450681 gtggcagcgg gcagtgaccg ttggccttca ggtcgcagcc catggggttc tgcatggtca
1450741 ccaggctggc ctggaccagg ctggatttta ccatgccggg ctgggccttt ttgggaggta
1450801 ccaagttggt ctggactgcc actgtggggg ggagagggag aggaagttca gtggacaaac
1450861 attgaggtca ctggtctaat ttacacaaca tgaagtaaca gtacacagaa gtgtccttgg
1450921 tgggtctgac aacctttaat gatccgcacg atgcattaaa ggtgaatcag agaggagagc
1450981 atcgactaat gtctgttgtt taaccagtgg aagcagagca gtggcgagga gcaaatgtta
1451041 gccaactgac tcaatgacta atgcgcaaaa gccaggagga atgaaaaatc tggagaaaca
1451101 tcctgaatga ggggaaataa tcagtcggtt gcttgaaatc tgttctgtct attgagaaaa
1451161 cacaagatgt aatactagct agagcttcaa tgtgttgctc taagtgacgg tgaagaaaga
1451221 ttccattttt tctactggag atttaacaac aggtaagtgg ctctttggtt gggggcttta
1451281 tataaatgca tgtgtgcagc agcaggtcaa cgaagtggta ggagaaagct gtcattttta
1451341 aattccctta atgtgtaaaa acatcatatg agctattttg agccgagggt ttcacccctg
1451401 atgccccggc ctctctctga gattggatgc tgtgcctcgg agccatgggt ggagcctgtt
1451461 atctgcaatg atgcaaccca ggaaggtagg aataagaaat tgattcactt taagggaagc
1451521 actctgttgt ctaatgggat acttatggga tatgctaggt aaaacgcgca cactcaaaca
1451581 cactcacgcc cagccctgat gagtcaagga catccaggaa gtgtctcatc ctctggtgca

1451641 gaaattacga atcttgttat ccttaatcca cgcgtggcga atcaataaca gcaaagtggt
1451701 gtcaccagtt ttggaaggtt caaaactgcc actgcaaatt tacaggacat gccttccagg
1451761 tcaatgtctg tggttatttt tagatcatca gaggaactgt tggaaaatta ggaccgagag
1451821 gtgaagcggg agaaaggcga cccaccctgg atctccatcc cattagatgg attcctgcac
1451881 acattaatgg tcttacacct caacatgtga gcagtcactt acataagatg tagctctgat
1451941 gcagcgtctc ttctacatgt tggagagtca aaaacgacat gcaaaagcgt cagatttcag
1452001 gcgccgctga agctcttttt caagccgttg aagttggttc atattgattt ttgcaaatta
1452061 gataaggaag agcgatggtt tttatttgat actatctgac actctgtttc atgggcctca
1452121 ctttctgtcg agagcaagtc agaaagaaga agaggacatg tttactggca gtccatcaga
1452181 ggatgttcca tcatttagcc tcacagaaca tgatgatagt tctgatttgg agcactgcag
1452241 attgtaaatt actacagtat ctcaggattt ctctgtgctt atcatgtgaa caggattatt
1452301 tccccgtgaa taattccccc actgatgaac aaaatttaaa aaaaaaaaaa agaatcctca
1452361 catagtaaac aacagtgtgg attttgagtt ggctcgggtc agccttctgg ctcagttagt
1452421 ccctgtggta cgagacaggt tgtaggcgtt tatatcacca gctgagcctc aaaatagact
1452481 gaacattgca aagcatccgc tgacaacaga tttcaaaatt aaacgtcatt tgagaatgtt
1452541 aagatgtacc agtagattta ctattaaccg gaaacgacag aataaagact tttaaagaca
1452601 ttccgacgtt tggatgatgg tgcttgtctc tccttagaaa gtttatttcc aaatccaaaa
1452661 aaactaactg atgaaatcgg acatctttcg aaacagttct ggtgctttta ttttattgat
1452721 cgttttccgg ttctatgcgg ctcatttgcg taacttttac tggtgcagca ctcccgtaca
1452781 gagctcacaa ctggaggatg aagaataaaa gaaataacct caaccgcttt caacaagctc
1452841 gggctcattt taacagacca gaaaatgcga ataaaaacga taaaaggtga cctgactaaa
1452901 actgtacaaa aacacatcat aaaaacccga gtaacaacac aacatcgtaa ttttccccca
1452961 gtattttaaa tgagtttgtt ggtcttcatt gaaaataaac acaaacgcat gtaacaaaaa
1453021 tgaagtatta tctactaacc ttgatccggg tcccagttaa cttgtttccg cgggttttct
1453081 attggaaatt tcattattat tttatgttat tttattttt tgttcaatca aatcctggag
1453141 gaatcagtta tgagcggtgg caaaattttg caatatcacg gtgatctttt ctcccacatc
1453201 ttcggagcat catgagttcg tgaatgtggg tgtaaactga ggaataaacg cgtccaaaaa

1453261 ccgcattgtc tgtctccgcc gtgccccctc ctttttttcc tctccgcctc gcttggacgt
1453321 gcggtgtttc ccctcacaca cggagactta tgagcccgtc agcgagctgc gccacagcac
1453381 acctcatctc tcacacaaac gcacgcacac acccaggtgt tgcggttccg aaacgcagcc
1453441 agagtgcgac cgaactttgc tgcgttatgc atccagaaaa ttcggggaag gacgcgctcc
1453501 tcccataaat tcccaaaacg caccgctgca cctgcgattc cttctaactc cccgaaatga
1453561 atcacgtctt tgcagcagct cctctggttc ccacgtaaaa attaaatcaa atcatactta
1453621 ctggtggttt ttcccccaaa tacaggtgtt atcctattcc ctcatcgttt ggtaaccggc
1453681 gagcaggcac atggcgcagg gcggtataat cagggatgta caccaaatca gcagcagact
1453741 tgaggcatta catcattcac atgtatgagg aagagtgcat tatttatgaa gaagttgttg
1453801 ccgctaccag cagctttatc accggcgata aagctcagct gaagatgacc gtcacaggct
1453861 gtcgccgcag ccacgatttg gctccaattt gtctatttta ttattctgca gagcagatgg
1453921 ctgcaggctg ctcggtgcct tttggttttc tgctcctgca gccatacgac atggttgcaa
1453981 gtttcagggg ttcaggggct gtatgttggt tcaaagtatg atatcatact ttccagcatt
1454041 tattcattct ctttaatgtt aataaaagga ccaggatata tgtcagccaa tacctgcaac
1454101 aatggttacg aaccatttta gtttatcata ctgactctac tcagaaatgg ctgtgcttta
1454161 tagtccaatg caaaggtgga aacactaaaa aaaaccaggt tacttattaa ttgcattaga
1454221 cgaggtttga atgcataagc aaaagtccaa gaaatatgga tgattcagca gaggaccaaa
1454281 aaaaaaaaaa acccccaaaa accccacttt attgacctca ctggacagta aaattataga
1454341 tataaacata ttatattatc gaaaatggga aggtgtcagc cagtcaaaga gagaatatca
1454401 gggcaataag gaaaacaata acctgttact actctaacag gatagattaa atttgtagaa
1454461 actatttcaa ctaacttatt acggccaaac tagcaaatga tgtggggatc aaaaggtcca
1454521 acaactaaag caagaacatt atgggatcca ttttgtgatg agagaaatgt ttacatttta
1454581 tttaaaaatc aaatgtgctg cgtgctctgt gctctttctg gatccgtttc ccatacatgt
1454641 cagttttccc acataataat cgaccgaatc agtgatattt agcttagtaa gtggtggaca
1454701 tgtttccaaa tgtgagcatg aagtttccaa tgtatgcaag ctctgataaa caacgacatc
1454761 atgtttatat gttgtatgtt tatgtttaag atgcttgctt tgttcggtgc tgctgtttct
1454821 tacacgatct gcgagaacat aataaattaa aaacaagtgt gctggaaggt tggggcgtgt

1454881 tcttccttac gcaagtgcgt gatgacgtag gccagcatag caaccagcat ggcgacagag
1454941 aagctttgac ttcatatacc catttgctta cagcttagca ttagtttggc gtttcgtctg
1455001 gtaaaatgtt ttcagtcgag tttgatgccg tgcctgaatg catccgggtt tcgccgaccc
1455061 ttctcttgat tatagttgat tgagaaatgg gatacgataa catggagtca agaccaggtg
1455121 agctaatgca cttaacgacg tagctgttag cattagccaa taccacggcg ctggctactg
1455181 ctaacagcta catcgccaac agcccttcgt tagaaatcaa atagtgctta aaccaagccc
1455241 tatttctggt aaatccatcc tgattgctgc atccgactgg agggaacagc aggtcattga
1455301 acgtattgat ggatcagatt agcaggatcc aggatggact accctttgcg atactataaa
1455361 gaagcgttca tttgtcttta aaatggtcaa gagtcgtatt tatttattca taacactccg
1455421 atcatgacca ataggcttac tcgagaccat ttgtgttacc aatagttcta ttttatattt
1455481 cgacagctgc tgactggttg tagatgatcg atacaagtga catagaagcc agtcgcaata
1455541 ttgtgtatat actgtatata tatcacatat ttaaatcaaa gctcctttaa ttgttgaaag
1455601 gtcaagtgac catttgctct tcagacttct ttgatcttat ttctattcct attttaaatt
1455661 atgaagctcc gctagcttaa tatgggtaat ttgggtttta ccaaatgtac aactgtcaat
1455721 ctacaacaat acaactgatc ataactttga aatttgtaaa tatccttgat aattattgtg
1455781 ttaatgataa gcggtcaatt gcattaattt aaaggagtgg tatttcctgg ttgatgctct
1455841 ttcatttatt gcagggagtg tatcatctgg acagaggtcc agttctgccg cgagaggacg
1455901 gaacctcaaa agtagccgta agtatgagag atgctcttct tgcagctgaa gtagtttact
1455961 agaagaaacg tctcttccat ggtggtacgt tcacgtggta aagtctccat gcatcgttgt
1456021 tattttagcc ttttgccctg gctcgtccag caagatggcg cagtccctcc tgaaagacca
1456081 catggtgtct cattacaaaa agatttattc agcaaaaggt aagatatttg cactcaaaag
1456141 atatattctg tacagcattt actgtatttt atcttagtac atgtatgaaa gcatactttt
1456201 ttcataagaa ttacgtgcga tcatttttct tgccagctgc tgttgatacc tcacttccca
1456261 agagcttaat acacagtgtg aagtgtaagt aactcagatc tgactttaaa agtgcagttt
1456321 tcaaccagca acagtggcag tttgtgttgg tgcaaatatt ggggcattct ttcttatgat
1456381 taaatgtaca atgcagagaa ataatgtgtg taactttaat tactgcctgg ttttaccatt
1456441 ttttcctgaa tcagtatgga acaaaatttc tactgtttaa acagctcttg tccttatgtt

1456501 tttgttgtgg tttcagacaa cgaccaaatt cgacgaaagc agttaaataa agacggtcgt
1456561 ccgcagtcgg ccccctctgt ctcaccgagg agcagcaggg catccctcta cacccccccg
1456621 gtaaggacaa ggcagacgga gtcattgttt gatgacgtca gtaacgtata ttcatgttgt
1456681 gtgttttcag agtccattat atgtccagtc tgaagaccgc ccctaccttt cctcaaggaa
1456741 ctccctctat agctcaaact ttggcacctc ctttcatgct aatgacatag tttacacgca
1456801 tcataaagct ggttcgcaga acttgttaca tactggggca tcggacgcca agtaccagag
1456861 atcagggccc tacaaggcct cccagaacac tgatcggaag acctatggtg gagacctgct
1456921 caagaaacat tctcagcact ttactcagga caaacctttc accccgaaga ccctgaagtc
1456981 agataagagt tcctacttgt ccaagtatcg ctattacaga gccccttaa agaagcaatc
1457041 tctggcaaaa gtgatgcatc aggagaaaga taacagaagg tacggaccaa atacctcact
1457101 ggcagtgact ggtgtaacat gcagcagccg taaatcatat agaatttaaa cttgttctgt
1457161 ctgtacgcag cacaaaaagc aggaaagtat cacatgcccc atctcaggtg attctgattt
1457221 gtgctcaaat gtgtctcact ggctttggtc agcagcttta gtgatgatga atatgtgttt
1457281 ttaaatgtgt tctgcttctt agggattgag tccagagccg gagtggtgtg aggatgagtt
1457341 cagcagctcg tactcccccg aaaccagcca ccagagtcca gcaaccaagg gcgggggccg
1457401 tcttttattt aactccccgc ccaggtaaaa tacgcaaaat caattcatct gttacattta
1457461 gaggtttttt tttttatgaa ggatttgtgg gttttcttta ggtcttatcc cattaacaag
1457521 gaatcttcca tcctttcacg tatatttgca gagtaagttc ttgtagattc agctgttgta
1457581 gcgtgaagtt attgtgagac atttctgctc gtctcattta cagggaagaa gagataatgt
1457641 acctcaagtt aatttctgct gtaacagaag atatcttatc caggggtcac atctctgaca
1457701 ggtttggctt ttattttttt ctcctcttgc ttcgataaaa atgagtcatt ttggcttttt
1457761 cttttctagg atcctgaccc tggtgatgca gcgacatatc gacatgaatc ggcatcacct
1457821 tgaggaagtt agtctttaac tttgtgcaga attttaacgt tgttgtccag acccggtcag
1457881 ctcctcctct gacgtcatgt ttcattttag gataaaatgc gccacctaat ggagcttctg
1457941 cgcaaagaat ttcaaagccc aacagacgtg tccagctgca gtgcaggccc cgaaaaaaac
1458001 gaaaacggac tctttgaagc attccattca catctggaat tgaggggga agaacttaaa
1458061 gaaaaggatg acctgctctg ttatgtgtcc ttcaatggct cctctgattc acccaatcac

1458121 acaccaggag gtcctcttgt gtccacgcca gcgtgctctc ccaagggaaa tacctcccca
1458181 tttgtaatta atgaagataa gcaagagaaa gacactctgg attctcagtg cggtgatagt
1458241 gttgccatta acacagaaag tgacgaggtg gacgcccatc agagcgaaat ggtctcaaag
1458301 gcaacagacg aaatcagcag tgaaatccca gaatatagtt ccaaagtgtt gaaccaggac
1458361 gaaagcatgg gctgctgtga tgaacagact gatgatttga gaggtctgga gggacagttc
1458421 tctgaatcgc ttcacgtgtc cagtaacacg gccgttactg agcaacactc agagggttct
1458481 tcctctgacg gtgagttctg actcaaggtt tagcttttca tttccaccga aagggtaaat
1458541 aaggagttga attttccaaa agtatgattt tataatgttt gctttagtca ttgtttttac
1458601 ttttctgtaa atgcacaata aagttttttg tttttaatga tcacaatcat ctgtattacc
1458661 tagtgtaagc cccccaaatg ctatatgcag tcagcgtgat gagtgattta gtttcaggtt
1458721 ggattatttc agtgcgttgg atttaacatg cctttagcat gtagctttta gcggttgacc
1458781 atcaccagtg tctctgcagc tgcaataaag tctctctaac ttcgcttggg tttcctgtag
1458841 ctcttttgta tttatttatt tttaaatcca atcttaaatc ttgatagctg ttgtaatgag
1458901 ctgcattatt gcacaacatc gtgtgctgtt tccagctgtc ggcccttgaa caagctaacc
1458961 agatgagcag caggcagctg tccagtttct ggcattttcg ccattcggca ttccaggtat
1459021 gtgacgtcat gggggccata acatccgcgt atccttccgc catagtggcc ctacaatatg
1459081 tcgcgaaaca tctttctatt tacacttaaa accccaccaa cgtcgtgtag agttagcaaa
1459141 cgctcacata ctgttcttgt ttgatttatt tagccatcat ggaggagtat gtgctgcagc
1459201 cgtagggccg cggatgtaga ctgaatccac ttgttgctct tccaatccca ggaagcgaaa
1459261 cacgtttgga acttgagtga actgttggag caggaggcgt ggagtgctgc ctcccttccg
1459321 acgcataaac aaactacaaa atcagctgat tcctaggggg aaagtggcat acttccacct
1459381 cagagacgga ttctccctgg gcccaaacca tctcggaccg ttggccaaat tcatctggag
1459441 agtttgggcc aaaactgttg accggcgacg tcagctcacc ccagggtgtc gtctattgac
1459501 tggagcgcag cgagctagcg gtaaagctac ttcagtgcct gctgatttcc cagtttgttc
1459561 cgtcaggacc gtcctaacaa ttcacttttg aattgctgag aaactgctcg gaacaagttc
1459621 gtctcacagc gaagttagct gagccgctaa ttcgtccgaa cctcagcagc gatttagcca
1459681 tgttgtgatc gactgcgtat gaaatcaagt tgcttatttc aagtatttgg gggggttagg

1459741 ctattccctg gaatttaaga tatcctacat attatttcta attataattt actttagtct
1459801 tcatggcttt ttttttttgg tagttcaacc gactttggtg ggttttgcca gtatgtgatt
1459861 gttgccactg gaagagaaaa tatgttagcg aattagatga ttctacaagg ctaatttaag
1459921 atcttcacgt ttgaacaatc gtgcacctgt tttcgttgtt gcacttttgt ggagatctgc
1459981 cccacatgtt tgcagggttt actcccaccc actgttgagc agagaggtgg cacattttga
1460041 caggtatctt gacaaccttg tccttttgtc ctgttttgca gagctcagcg cctgatgatg
1460101 agcctcacca ctcagaacag aagctcgatc tttaagaggc catccaggag aatcatttct
1460161 catgcataag tgacgacctc ccccttggag acgtgagaaa tatgcccctg ccgtttggct
1460221 taaaactcaa aaggactcgg aggtatactg tttcaagcaa gagctgtctt gtcacccgga
1460281 ttcagctgct caatggggag tttgttgagt tcacgctgtc ggtggagagc accggccagg
1460341 aatgtttgga ggcggttgca cagagacttg aattaagaga ggtatgtatg tgttttctgc
1460401 cacaagctcc gattgtgcac gctctgttta tttctttaag tcgccacctt gcacttccaa
1460461 tggccttggt caggtcatcc aattctcaac aatgatttag gtgtctgtca ataactattt
1460521 tatcatatgg tttagtggca tcctcgtgct gcttgtggca tggcgtgaat atctgacctc
1460581 tcatagagag gctctgttta ttttccagat ttttatttat attcaggcgc cagctcttgt
1460641 atagtgtggc cagtttcgtc taagaaaagt agttctgagt cagaactgag caatgtcctg
1460701 agcattaggt catagcaaca taatatgtta acctccaact aaataaaatg taaggcagag
1460761 aacggtttta aggttgtctt tgttaatggt caagaaaaaa atgattaata ttgatatttg
1460821 ttttcagagc tatcacagtc acagttgacc acagtgcttg tgattgaacc acaaccacat
1460881 gtaggtgaat aggtcaactc aaaatcaatt gcttataatg tcatcattta tggaacagct
1460941 aaaatggcaa attcttttag attgtggtgt gctagtggtc aataagtccc tttcagctct
1461001 agcgatcctc tatggcaagc ccagagggat acgttttctc accagatttt agtctgtgat
1461061 gataagacag tcacaagact gcacagagag aaggagcgag atttagacag agttgtaaag
1461121 gttaagttat ttctcagaaa gcgagaccaa gatccatatg attaacatga ttacggagat
1461181 tctggtctga gggttgttca taccgtctgc acttgtcttt cagataacct acttcagcct
1461241 ttggtacttc aataagcaga accagcagag atggatagac ctggagaagc cgctgaaaaa
1461301 gcagctggac aagtatggac tggaacccac cgtgtatttc ggagtcgtgt tttacgttcc

1461361 cagcgtcgtc caactgcagc aagagatcac aaggtgatgt tcgatgggcg acctaagcct
1461421 gagctgcaga cctgtcttga ggcaaccttt aacactctca gttgaaatta ttttttggtg
1461481 tgaataatct taatcaagtc cgagtttaga gtaaaactaa ggtgatgatg tataaagtat
1461541 acaatcgcag tgatgcacat tgtcagtgtt gtcttctggt tttatccttg gcaacatctc
1461601 tgtactagaa aagaaatgta accccgttgt ctctctctgc tcctcgcctc tctgtcctgc
1461661 agatatcagt attatctcca gctgaaaaag gatgttctgg aggggaggat ctcatgctcc
1461721 ctcgaacaag ctattcgctt agctagcctg gcagtgcaag gtacactgaa ttatttgttt
1461781 ggtttggtgt ttatggtagc ggttgtgcag ttattcatgg gtgtgtatat gggtgtgctc
1461841 gagacaaagc aggtggaaac acagtttatt tgaatttaat ctaaaattac aatcccaata
1461901 aaaccaacct ggcctttttg agctggacag ggtgtttgtg ttctcgtgta tgcatgtaat
1461961 tccccctgca tgcgcttcag ccctgtcctt taactcctac ccaggcagaa gaatctgaaa
1462021 cactgctggc aaagttatat caggcgctgc gatttattgg cctgggtttc agcatagcag
1462081 agctgctgag atcatttact gtggcagcca tgtccagaca gctgcccgcc ctgctgcatg
1462141 caccccaaga cactttgcaa aacgttttcg tttccttttg ccattgatcg cacagttccc
1462201 ctgcagattt gtaacattga tacagttgtt ttgttcccag tttgtctctt tgttgagatt
1462261 attttggaat aaagagcagt tgcagcttct aagtggcgct gatgtctggg atgcccgaca
1462321 ttgttgcgag tgttgccagc aggcggggtg aagctgggac cagttttgga gctctaaatt
1462381 accagacact gaaacaattc cctgtcaaaa caaaaaagcc ctctactctg ctaaaattag
1462441 ccttttgcta tcaccagaag cttatagcta aatgtctgaa gccacccttt aactcctgtg
1462501 cctacaacac agaccattta ccttttaaac ctcttttcat acatagctga ctttggagac
1462561 ttcaatcggt acgattccca ggacttcctt cagaaatttg tcctcttccc tatagtaagt
1462621 ctttcctact caaactttaa tctcactttt gctttttttga gttcatcctt agcattggta
1462681 tcattatttt caggactgga tccaggatga gcgaacccgg gaagaggcca ctcagaaagt
1462741 ggcacttgtt tatcagtcct tcaggtgagc gggtgctctc ggcgtcatcc tgtcatgaac
1462801 ggcacatatg atgacagcgt atcgactgca tgtcagtata cctgcggggg actgtttttt
1462861 taatgtattt ttctgccttt gttcaggggt ctgccagctc cagaggcaga gatgctctac
1462921 atgcaggagg tggaaaagat ggaaggttat ggacaggaaa gcttccaagc caaggtaaat

1462981 ttgtgaaggt ccaacctgtc atcctcaaca atccagactg agaacaatca aaatgtgcac
1463041 ataaactgta ctgaaacatt ggcgctactg catagttttg tgctgggaat agttttaccc
1463101 acaagagtgt tgtcttcatc ctctaaacag gacagcacgg gcacagacgt aactctggga
1463161 tcctgccttg atggcatcat cgtcaagcat aaaaatggca gaccgcttgt tttattcagg
1463221 tacgacagga aaaatcttta gagattatct cttcgtagta ccagtttatg ctctctgata
1463281 gtgtagatta ggtttgttgt aaaaaaaaaa aaaaaaaaac tgaatgagtg caccaccatg
1463341 actgaattat caggtagaca aattaagatt atgagatgat tacgagctct gtttgtatgt
1463401 gcttgttttt ttgtttttta aattctgacc tataaaacta tttccataat cacatgaaca
1463461 cagctgtttt gtgaccacac tgatatatct ttttcttgat tgtgtgtagg tggaatgaaa
1463521 ttaacaacat gagtcacaac aggtccttct ttgctgtgga gctgaccaac agggaggaga
1463581 gtgttcagtt ccagaccgta agtctccgct gccagggcac tttctctttc agtccaactt
1463641 tagccattat cgaattgtgc taaattaaaa ctgtgccgtg ggacgtgtaa gttatcccgt
1463701 ctcgacaact cacctctgtt tgcgtggatg tggcgcaact gggacaatct gggtatgctg
1463761 catatatgtg tggctcatgg actcgcatac cactcacaca ggaatgcttt aagatgtctg
1463821 agccagcttc accccttttc tgatgattta accacttgtg atattgtttt cttctctgtt
1463881 ttgtccagga agacatggaa acctccaaat atgtgtgtcg gatgtgtctg gctagactaa
1463941 agttttatag gattaacaag agtagcctgt aagtgcccgt gattttaact tgtctggcct
1464001 gattcccttc gctcttcttc atcatctcct gtgagtgtct cctcttcagc ctcacttctg
1464061 cactcccgtc ctcatctctc ttcagagagg actgtgaccc cctgccttct gtggtctccc
1464121 agaagtccct tcttactcta tcttttcctc gctttccaat gctctctcgt ccctctctgc
1464181 cttctaataa ggggtgagca acactgtcag ccaccacctg aataccaaca ccggtccctt
1464241 tctgaatgcc tggcccagca gcacatgggt tatttatgtc tgaaatgggt tataggtcta
1464301 agggaattat gtatgttgtt agtttctcta cttgttgggg tgtgtctgga gctttgacat
1464361 tgtgtatgca tttaaactta ctccaaataa gacctaccgt atatataaat tttaaaacaa
1464421 tctgtgctga aatgggggga aagtgagatt ctaattttga aacgctgagc atgatctgca
1464481 cctttagggt gtgctttcat aacttttaaa tgtctcgcac agaaaaacat ggttgaggta
1464541 aggtaactaa taagcctatg tcaatgtttt attgacctga atttcttgtc attagccaaa

1464601 cccagcctgc agtcatcaat ccggtcaggc gcagatcctc gacaagaata tctctggtac
1464661 tgttaaagtt gcaacacatc ttatatttag cagcctgatc ggcaatttta tatctggttt
1464721 cttcatcctt gttgtttaac agccaaagcc tcaagcctac atgatgcccc ctccacagct
1464781 gcactacaac ggccatttca cagagccgta cacgtcatca caaggtaact cgctgagagt
1464841 ggatttgttt ctgaataaga atttaaaata ggacgtaccg gtaatgtatt gttttgttcc
1464901 gtcagacaac ctgtacatga acaaccagaa tggttattac taccactccc agaccagcct
1464961 ggactgcact ccgctcgaat acggaactgg ggggcgttta cgaaatggca gcgtgtacag
1465021 cgctcacagt accagctctc taaccaaccc ccagcactac ctgcagccct ccccaatgtc
1465081 ctccaacccc tccatcacca gcgatatcac cagaccagat tacgtcccct cacaccgcca
1465141 cagcgcgctc ataccacctt cctatcgcgc cacccccgat tacgagacag tgatgcggca
1465201 gaaagcccgg ggtccaggag gggggatggt tttgtctcaa gaccaccggc agagccattc
1465261 catgaggaac ctgaacattg gaaactcgta cgcttacagc agaccagacc ctctggttta
1465321 cagccagcct gagatcagag gggagcacgg tgggacgacc caacgccacc actatccttt
1465381 ccatctgggc tctagcttcc acagtccctc tccatacccc taccccaccg agagaaggcc
1465441 tgtagtgggc gccgtcagtg tcccagagct cactaatgtc cagttgcagc aggcccagga
1465501 gtatccagcc cccaacatca tgagaacaca agtgtacagg cctccccccc cgtatccata
1465561 cgcccacccc cgacctgcca acagcacccc agacctgtcc cgtcacctgt atgtcagcag
1465621 cagcaaccca gacctggtca tcaccagacg cgtgcaccac tcggtccaaa cgttccagga
1465681 ggacagcctg cccgtcgctc actccttaca ggaggtctcc gagcctcttt tcagcggggc
1465741 ccctcagaga cacctctaca acgctcagaa gcgtaactcc attgagatcg ctgggctgga
1465801 atatagcctg gaggggatga ggctcaaaga gaggactgtg tcttcttctg ccgccgagtc
1465861 ggccacgccc cctcccgtcc tgcacggcgg ccgctcccag ggctctcagt taaatgtgtt
1465921 cctggaacat ccaaagaagg aggagatcaa ggagagcgta cagtacggcc acaaaaaatc
1465981 tctgtctgat gccaccatgc tagttcacag cagtggcgaa gaggaggagc ttgaagacga
1466041 gggtggacgc cacactccac tgacccagga agcggcggca gcattgggtc cgaaccagcc
1466101 gtcacagccg catcgcagct tgacgcctct gaccgctctg gcgccgcagc agcagaatca
1466161 aatggaaccg ccccctgcgt atcccatagg gtcgtccctc gacccctcca taaccagctc

1466221 catcacctac aaagttcacc ccatgatcca aggggagccg ctgtacctgc
tgcccgattt
1466281 acgccaaccg aggatcatac cgtcgatttc ggacggagac ctgagcggcc
aggcgaagca
1466341 gaagactaag aaagacgtga agaagcggcc tgtgtctgag ctgcctccgg
ggaagaaaac
1466401 cgtggaagga ctaccccctc cagtgagtta tgatctttct ccttcatttg
ttactcatct
1466461 gttccagctt catgctctgg ctgcagcagt gctcagtagg cagaggtttg
ttttccaaca
1466521 acaggcattt gcacctgtgt gtcttgtgga ggtcatgtga ctccacaagg
gtagtttcag
1466581 tgctcagtga cggcctcagt gtaggaatgt ggcaggactg cttcgcgagc
cgggtcgacg
1466641 acattgtaga tgacgtagag gccacatagt ccctcctgct gtacaccagc
tccaccacac
1466701 taggaaacta cacacggtca aattctctcc gtctttattc tgctgtcaca
ccagggcatg
1466761 aagaaagggc cgagatcaga ggcgaagaaa atgggcccgc tgaagatggc
ccatctcaac
1466821 ggcctgtcgg tatccaggca gccgatgtac agcgacacca aggacgagcc
ggagagagcg
1466881 tctaacgacg agcgggtagg acgccagcaa ccctcctgta ctgggaataa
ctccgtccaa
1466941 caacttttac agctgttggg tttatgtaaa gtgcgatcgt ttctttaaat
ggagctgctt
1467001 gttgcgttca gtattcatac ccttgtgagg actttagcat tgttgtcagt
aatgcttcga
1467061 cacttaaaat gcctcacacc aacgattctt caacaggacg ttggggatgt
ccagcgtctg
1467121 caacgggttt atgcgataat tgtggagatt aaatggggta atgcaagtga
tttgaaactt
1467181 tctcagcatg tagcaaaaat gtagaagatt taaatgaatt gaacttttta
agatttaaat
1467241 gaattgaact taccctgagc attctaactt tacagtggag ggatatttta
aatgtgtacg
1467301 cgggtagaaa attgtgcaat agatgatggg atttggtaaa cgcctgcatg
tgtggttact
1467361 cttcagtcct gtcctccctc tggtttcatc tctggtttca tcaaaaatgt
ggtacatcct
1467421 gctgcaacac ctcgaagtac agttacagga aaccacatcc ccttaggaca
gaaatgaaag
1467481 caagagcaca agtacaaata aacagttctg aaggcattag tcctcataac
aaagcacttt
1467541 ctgatgctct tggttgccaa gttatgcaat ttctctaata gaaaaatgac
ttccgcttgt
1467601 tgctccattg gtttgttgat tgcagtgtaa gctgctggag cagcacatgg
agcggggaga
1467661 gcttctgaag gaatacgaga gcattcccag gaggcatcct gcaggggaat
gctacatcgc
1467721 ccagcagccg gagagctcgg acaaaaaccg cttccacgac gtccttcctt
acgacagcag
1467781 ccgcgtggag ctggttccca ccaaagagaa caacacaggt tacatcaacg
cgtcccacgt

1467841 taaagtaagg agcagcattc cagggaagag gttgggggtt acactcaaaa acggaagctg
1467901 acggcttgtg ttctggcgtt agctaacggc aggtgggcag gagtggaact acatcgcctc
1467961 gcaggggccc ttgtcaaaca catgtcagga cttctggcag atggtgtggg aacagggcgt
1468021 ctccatcatc gccatggtca ctgctgaaga ggtgttttga gcgggatatt cctttagagg
1468081 attcctcatc ccagtcggga ccttaccttt tgagttctcc ttttcaggag cgtggccggg
1468141 agaagagctt ccggtactgg cccagactgg gctcgcggca caacaccgtg acctacggtc
1468201 gattcaagat cacgacgcgc ttccgcacag agtcgggctg ctacgccacc acggggctga
1468261 agatcaaaca cctcctgacg ggccaggaga ggacaatctg gcacctgcag tacacagact
1468321 ggcccgatca cggctgccca gacgatatca agggcttcct cagtgagcta ttagtctcta
1468381 gtcacgatca gatattcact caaagttcta atcattcacc aaagctttcg gcctgtttct
1468441 ttaatgactt tgttgttgtc gttgtcagcc tatctggagg agatccagtc tgtgaggaga
1468501 cacacaaaca gcatcagtga cccaaagaac accaacctgc ctgtgctggt ccactgcagc
1468561 gcaggagtgg gccgcaccgg cgtggtcatc ctgtccgaga tcctcatcgc ctgcctggag
1468621 cacaatgagg tgggttgctc tcagaataaa taagtgcccc caccaccctg agggtgtaga
1468681 ctcactggcg atgccctgtt gcagacgctg gacatcccca acgtcctgtt gaagctccgc
1468741 gctcagagga tgatggtggt ccagaccttc gctcagtaca gcttcatcta catggtcctc
1468801 atccactatc tgcgcaactc cagactcatt tgagcccgac agagcaggaa cagagaccgg
1468861 agggtgaaga ttctcgccat ctttgccagg ttccatctgc actgtggcca taacgcctgc
1468921 ctggagtcag tccgggataa accaggcaag gatgaagcaa ctttgcatgc ctttgcatga
1468981 aattggccac acaaaatgga ggataaagac ttgtgacctg cctctaaaca gtattatata
1469041 tttttgtatg tgcattgttg ctcaacaaag cccagcactt tcacagtact ttaacgtaca
1469101 tcatgaacca tttaaaaaaa atctttatat aggatgtgta aaaaaaaaaa aaaaaagctt
1469161 atttttttc tccaggtcag ttttagaaac tggggttgaa tcatgaagga atgatctcta
1469221 atcaagaaat caaacgcaaa gctttgattc cagagtccaa ctgcagtgta aacttttatt
1469281 ttgttgactt gttaagggag tcaaagcacc agagcatgtt tatattttct caaataaatc
1469341 actggatgga tgaagtacaa ccattgcaca ggatgtagga atacacgaga ggttgactca
1469401 aactggatct tttttgtagt cttgattgct gtatttcgtg ctataagatc atttgagaa

1469461 gtgcttaaac aacttggctt cttttggata atgaaggttg ataatgaagg ttctgactta
1469521 agtgtcattt gttgctggca taattgcagt gacgtgttct tgccattcac tcggacgtaa
1469581 aacccgcatg aagtagaaac tgtagtattc aactcttgaa attagagtct ggacgttggg
1469641 acatttttag tggttggggg tgattaagct gcctgcggtg ctgcgtgtca aaagcacaaa
1469701 gattgaggag cgactctggt tacagtcctc caacgccaac acttcctgct gttacagacc
1469761 atctggtgca tttacaggag cgtttcctgc tgcagacagg caacgtttaa atccctggac
1469821 agaggacgtt tacatgaatt gccattttaa ttgtgaccgg ttttgccgca ttcgttggca
1469881 aacttgagtt aagatgcagc tgaagtctta actttattga tttttaactg gagagaaaaa
1469941 aacggccgat gtattatgtt tttatggtct ggatgaaacg gctggatggt tttggttgta
1470001 ggataccgtt ttgtggccgt cacctcatcc atgtacaggt ttgttcagta tgtacacaat
1470061 gtaaaattca gcttttatga atattggata ttgtacaaat gtataaatta tctattgaat
1470121 ggacaaatga tattggaaaa aatgtagaaa cttcaaataa agaaatcttt ggaaagcgtc
1470181 tcttttacgc ccccttgcgg tagaatgtaa acctacatat tagtagaaac taagggtagt
1470241 tcagtgaaat ctaaatttat taaatatcat taaaaagtgt cagatattct agctggcatt
1470301 tcaccctaaa aaatccagaa catttcagag gttcagttga aatgaacatt tacacatcat
1470361 tccaggaaac taaagttcac atattaaaac atacagacat caatgtgcta ctcacagcat
1470421 cttaatgaaa gtaaaaatac aaactggtgc taaatgaggg ttgttgtttt taaaaagtta
1470481 gaggaagaag gttgtattta atataaaacc tggacacagc aggtaagatc agtcacactc
1470541 aatactggta ccggcctctc ttccctccat gtctaccatg tcctccataa tttccatgtc
1470601 caccttggtt ccatccacct cctctccatc ttcctcttcc tcctctacca cgaccttgac
1470661 ctccatggcc tccccagccg cctccacctc ccccatcttg tctttttgc cagggcggca
1470721 tctcaggagg cggcgctgct atctcagagg gacgtcctcc tcggtctgga acaactccca
1470781 tcaacacctg ttggtgcaac agcaaaacca ggttgtaaaa ttgaaaaaca aaccgactgc
1470841 agcagctgtg tagtacgcag ggcagctctc ccgtaccttg ttcacagcac agaccgtctt
1470901 ctccctgctg gagccgctgc tggtcttcac tacgttgcag aggtcttctc tggcagccag
1470961 cagcttggac atgtccacag cggactgcgg cctcagagcg tgtctcctgg gctggtaaac
1471021 cctcgccatg ctgtccacct tcacctgcaa acgttagaaa aaagcccttc cacagtcttg

1471081 agagtgaaca aatgctgagc tatctgcacg tttatacgaa gtacctttgc tctgtcagac
1471141 cagccaaaag actgaactgt gacgtccaga cgtttgatca gcatcctgcg ccgacactcg
1471201 tactcggacg acagagctgc gtttatgctg tccagtttct cctgtaggca tcgattatct
1471261 gtattaaatc ctaagatgtg tgtgtgagct aaaacaggag tttcccgttc ttacccactg
1471321 gtcactgtct ggtgatttct ttagcaccgg ctttccgaca gagccgtctg gaagatcttc
1471381 aagtattttc tcgacctgca acaaatgatc aggattcaga acatcagtga catgaggatg
1471441 tagaccacat ggtttcaatg aaaggttaat atctagtatt tactgacagg tactcttcaa
1471501 agggcacaat cactacagcc tgtcttttag accaggtgag tatttccaca ctctcaccct
1471561 gtcttggact tgagagaaca ctcctgctgt gtccagtcct ctgggctctg ggatattcag
1471621 catggcacag attgacagga gctcctgaca catcggactt tcttcctgtc ggctctctgc
1471681 aatgtgtctg ctcctcacca tccgtgccgc ctggagctct gagctcaaaa acacttaaga
1471741 aaagagtggc tgttgtaaat tcacgtcttt ctgaggggag tggttaaaaa gagaaggtcg
1471801 actggaaacg tacaaatgaa tttgaggtgg tcttttgttt ttaactcgct gccattcaga
1471861 atcccagaaa tgtcattagg acagtgcaac tccttcagga agccactcaa ctccgtccgc
1471921 agcccgtcca tgtcatcttc acggagaaga gagacaaaat acatgttgtg tttctttagt
1471981 aagtgcttca taaacacttt aaacttcttt gcaaaaacaa ctttggatca tcaaagaaaa
1472041 tacataaacg ttaaaaacta tggagataga ttcccggtac actatttttt tctaaagaaa
1472101 aatattttcc ttggagtacg caggtaaact tagttttaaa aagcaaaaac tgtacgtaac
1472161 tgtcttttct tcccctctaa cttacaacat gcgttacttt ttaagagtca agcgtggctg
1472221 aatgagtctc acctggccca gaagtaaggt tctccttcag atcacacagc ggctttaagc
1472281 tggatgtgag ccaccggcag agatccacat actcgggaca agacaaacct tctgccgcgg
1472341 ctgcgagcag cgccttttcg tccaacaggg ggccatcata gctacggaca acagaaaaaa
1472401 attaattcga atattttgaa ataaagtctt tataagatat gctgtgatgc ctgcaaatta
1472461 atcatgacaa aattggactt gtgatttagc gtgaatgtac ttgcaaaaca atatcgcggc
1472521 acgctaggca taaattagct tacccaagct gttctaggga gtccaaaata tcacattcca
1472581 tgtctgaatt agcacaaaag gtacgtgtat ttgtgtactt gacacatccc gaagttacgg
1472641 ggacgaaaac ctagagaggg aatgtccatc cattagtgtt ctatgttcag tcaatgcggc

1472701 agcaccattg cgtctcatgc gttcattgct tttccggtcc tctcacgaat gtgctccgaa
1472761 gagaaacctg acagttgcta atgttccgct gcaatggttc cgccacacgg tggaaaatcg
1472821 atatataaac gatgttaaaa atcatcattt taatattgta gtgctcaagt attgcttata
1472881 aatcaatgaa tgcatagaca ccaattatta tcttctttgc tagtttatgc tggctgaaaa
1472941 aaaaatatat aatgtatgtg tgtgtctgtc tgtgtgtaca cacacttttg tacagctctt
1473001 ttgtacagaa tttttctaat gttcaactaa tcaactaaaa gaggcaataa aacccacaca
1473061 gcaaaagatt aaaactcacc attttattat gataatgtta tctgatatgt caaattacaa
1473121 gtgtatgatg taacataaaa acagttacaa acatatgtat gcatatttac attatatatt
1473181 tatgaaagta acgagctaca tctgtataaa gaaaatacaa ctatgacatg agttattggg
1473241 actataaatt ttacatcata tacttggtat tctcgaaact aaatgtggaa aaaaacaaca
1473301 gaactgtaaa gatgttaaat tataaaatgg aaactaggat ggacgtgagg cacttttctg
1473361 aggttgtcgt gaaagccgat ggcactacaa aagttcaaaa caaaaaaagg atgcacgggg
1473421 aacaaagtta tgcacagagt agtgaaagaa agaaaacagg cagtgtttac agaatagtta
1473481 agagtagtac catttatact gcaagtttca aaacacaaat gttttccact attccgaact
1473541 aatcacgtaa atgttacatc aagcgctgta aggtggagga aaaaaatata taagcataca
1473601 tacagtatat cgtcagtgca ccaaagcgtc agagaattga aacagaacca ctcaactgct
1473661 gtgaaaagta atctttttaa aacaatacac tgaagagaaa ttggcttata tagatttctc
1473721 tttttaaatc tacacaatgt caagaattta cacttgtttg cattcccaaa gatatgcaca
1473781 aatttccaag attcaaagag gataaaagac tagcagcaaa gtggttaaaa ctgaagtctt
1473841 atataattgc aggtcaaaaa gtgtgtgtgt gcgtatacgt atatatacac acacacgtat
1473901 acctgtgtgt gtatatgtat atatatatat atgtgtgtat atatgtgtat acatacacac
1473961 atatatatat atttatacat atactttctt ttaaagcaga cagacacatg ttgcatatat
1474021 aaaacagtac atgacaggaa gttgcactgt gctggttaat gtagaatgag aagagttatt
1474081 taagcatttt ctacccttttt gtataaatgc tcgtcccaca ttttgcttct cagccagcaa
1474141 gttttaatca ggttcatcaa ccctcggttc agcctgcaag aacgcaggac aatccaattc
1474201 caagaggaga aaatgacaac caaccaaaca tttggttaca caccaacccc acaaacacac
1474261 acgcatgcgc aggaaactta acgcacacac ggcacgcgcg ctacacacgt gggcacacga

1474321 gaatgcttgt gtagtaacac tcgtgcaccc acacacgcat gcacaagcgt ccacttcaac
1474381 cgctcagagc aatcagacgt taaaaaaaag acctcactct catggttcga tgctgttggt
1474441 gaattaggct ttaacagtcg ttcatagcta attttctttg acaatagtaa aattagtgat
1474501 tgtatctagc agctatatat ttatacatat gcgtgtatga gcttatatat gtatacatac
1474561 acacacactt gtgtatgtgt atatacgcac atatacgtgt gtgtatatat atagatatgc
1474621 agacacatat atatgtacat acatgtatat atacacacat atatatttaa aacttttaat
1474681 gagtggactg ccactactgt accgtgttaa cactaccgtc cttctaccgt acaaaactag
1474741 agagaactct attagaagct ggattcatgt ccatcacaaa cgccctctac ttttcaatgt
1474801 gaaataatac taaaggatga cctatgtaca taatgctgtc agtccaagct acggtataca
1474861 ctgacagcaa tgataaatac acctctatct ttaacggttc aaagatgggg ctatcatcac
1474921 aatttaaata tagcgttcag tatttacaag tgtctggagt ttggtaccag ggcgtgtcgt
1474981 ggacctataa tagcattgaa gtcttctgca gatggtctgc agatttcgac gggttcctcc
1475041 tgttaggaag tttgcataga gggcaaagag aaggttggcc gggaagggga atggttcttc
1475101 cagtaacaat gtggttgagc ctggttctgg tcaggagtga cagcaagtga ggggagactc
1475161 tttctgctgt taaagatcga cattttaaag tcttgcagca ttaagtacag taatcatgga
1475221 gtttgtttgg tgtgttagtt cagcggggga gggggagggg gggggtccct ggatcttacc
1475281 tttcctcact gactaaacta gagggaacag tgtgtgcact tagcttacac tcagagcttc
1475341 aacagtgcat aggaagacct atgcacacac acttacacac gcgtagcatc tgtgttagtt
1475401 taattgctaa gatactcggc gtcccgcgaa gaatgcagcc gatccttcgc cgcctggaaa
1475461 atggaaacac ggaggggtgg aattgtgagg aataatgcag aaggacattt acacaggagg
1475521 ggagaaaaga aaagactaat gtgtgagaca aacacggaca tttgcagagg gtgctgcaac
1475581 caaagctcgg agagtagtgc ttccaggtgg gttctcccca tcccaaataa gatgttcaat
1475641 tatgtttaga aaggaatttt aaggtctgag ggcttctctg tagaaatcac ccagtttaag
1475701 tgccttgttg tttaaccgct accccttac tcaccacaaa aaaacttgct ggttgagcaa
1475761 catttcagaa ataatgaccc aaaaaaatga gcagaagtag aacaatgaag aaccaaagat
1475821 accaacatct ctactacagc agtaaaaacg ggaccaggaa tcgaatccac tgaagggaat
1475881 caaactatca tgcattggtt tcaattggca attatttttt ttatgatctg gaaaactatt

1475941 ctggggaact ggtggtgcgg taaagtcaga tttaccatat tttccctaca aatgaaatca
1476001 atcccttaac tttggaatgc aatcaaatgg tctgaatcac ctcctgaacg tgctcattta
1476061 tccaagaaaa gttctaccaa gtaccttcag agtggagctg cagccttatg gagtattttt
1476121 aacatctgat gggaaggaat acagaaaatc gggcaccgtt tctcaaatgt tcacattaaa
1476181 ctatatacgg tggttaatgt accgcaaaat gagtgttgcc ttttcttgag gggtgggagc
1476241 atttctagtg cagctttctc tgtctagtaa ttatactctc agcaaatatt taagctatga
1476301 aggaaaacca ctgttaccca actgcaaatt tacaagaaaa acagaacaga tcccttttag
1476361 catactcaaa taagtatggt tgtctgaata cagaatattt gtgtcgtctt ttcggagtta
1476421 agttgtcgct ttgtacaaaa gaattttcct tcattgagtc acacgttagt tctgacgagt
1476481 gtgacccggc tgtgtcggca gatgagcttg aatgtgtgag gtgtgtgtgt gtgtgtgtgt
1476541 gtgtacaagt gtgtaaagta acatctccaa gtctgttaat cttgatgccg cctcctcgtt
1476601 ttggatcgag ctcctgaact tagcttgatg ctcagagccc ccacaaataa aggtgtgtcc
1476661 tctatttcct gcttgtccac gacaaacaca aactgaaatc tccaaaagcc ctcagagctg
1476721 ggggtcaaca acacctcgcc ccgtctgaga gggaatgaaa aaaggatgtc ggcttttaat
1476781 tttccgcttt tatccacttc ctgtgttagc tagagggtgt ccaagagtct gcggcggggg
1476841 gtcaccccgc ggccttgtgc ttgcccccgc agcagtggca ggcctcgccg cagtggtggc
1476901 aggcgcgcag gggcaggtag cagcacatac agggcgcgat gaaggacagc gccaccagtg
1476961 caagccagcg caggcagaac tgctcgtcgg atgtgtcaca cgagcagggg tctgagaagt
1477021 ccccctcgga gtcagacatg cagtggtaca gcatgctctc cgcgcacagc atgcagctga
1477081 ctttgtaaat gcactgcttg atggggtccg gggcgtcctg acactgccct cgccagttgt
1477141 cttcgtggtt gaacatctcc cggcagtaga tgcaacgcga tcgctcgccg tcctctcgcc
1477201 gccgccggcc cttcttagat ttcagcagag gtgagggctg agtcttgata gccgtgtcct
1477261 tgcccagacc cagtcccata cctgttccaa tacccattcc agggccagag tgagagtctc
1477321 cgccgggacc gtcggggaaa aggtattcac acttcttact gtccagcttg gtgaaggcgg
1477381 tggggaagtc gaggtcgtcg cggtcggcct ccttctttag catgagaggg tggcggtagt
1477441 cttcatagcc ccggatgagc acgtctttgc gtggattgat gcggacaatc tcctcctcgt
1477501 ccatatgaaa actgacgtgt cgggttggtt taaacgccac ctttgaaata aaacaaatgg

1477561 agagaaggtc actacttgcg cagccaaacc caatctttat ttctggtatt ttcagaagct
1477621 ttgggactgg ctaaatgcag gcgcgacaga cctggggagg caggtaacgg cggttggtgg
1477681 aggggaaatc ctcaaaaggt tgtgcacgga cgtagcagcc cccaaatggt tcagtggaga
1477741 caacatggtt caggggagag aaggcctctt tcatgggtgt acagatggtg gagggatcat
1477801 cacacagcta aaggggtgga aaaaaacaca ccataaaaca ctgcagtcaa cacaaagcta
1477861 aactgacaca acttaaaaaa tcctttccat aaattttata aaacgccagc tgaaaacttc
1477921 agcagccctc tttttttacg tttttaaact agagatcatt catgtttatg accgtatgac
1477981 tcaggttgtg taggctgcgg tcaggtccac ttgtgaccac cagggggcac atcacaccca
1478041 catttggccg aacggctgct gtgtgctgat gactcaaggc cccttctgtt caaaactcca
1478101 agttcccaac agccttgcat gtgtttccac aaacatcata actataacat tggcctaaaa
1478161 agaggtttta tgtgaaaagt aaaaaaaaaa aaaccctgaa agctgtgagc attccttcaa
1478221 ctgcccttca aacacaataa ttagaaagaa acttcctctt cacaccagca agatggagac
1478281 ctcattaaat aaaactgtgg cattcactcg gaattgtatt tctattttaa atgcgaacgc
1478341 acaaacaaat aaaagtaatt cctcagcact tgtcacaaca gcagtgaaag tggtgtgtgt
1478401 tcgtgattca tgcgtgcaga ggaggcgatg agagatgggg gaggatagtt agtccatcaa
1478461 gagctggttc agcacaacct tctgtacaac tttaatgttc tctgcagtgc aaaacacgcg
1478521 ctcctaacca gaacccagaa tccttatcct gctcaaaccg atccatgaat gtggagttgt
1478581 tcctgcactc tgacgctcag aacggtgcct tcagggggtt tttgcacact taatactgca
1478641 caagacacaa gaatatggtc gtgcagcttt ttacccacaa aactggcaac acatcctggc
1478701 acagatgaga aatcctgtca ataaatctac aaagcagctc aacgcctgtt ttatgctcct
1478761 gctttgactt tataaattta aactctactg agtagtttta agacagcatt tctgatttaa
1478821 tcacagtaag acagtgacag cttttaatta tttgcctcaa aacaatctta ttgtttttaa
1478881 aacgcttcca aagttttttt gagagcattt tggtcaaagt ccactttaac caccaaggat
1478941 tctcagcctg atgttctgaa tgtaccaaac tgctgaattg tcccacactg ggagctttat
1479001 gttttattac tcctccttca tttgcatcga tatctccttg gacttcatac tcgagcagcc
1479061 aaatgccaag tcaacaccaa ccaatgccag acgttttcag acctttctga ggcagcaaaa
1479121 cttggcaatt aaaaaaaggg gtcacaagga ttaaaaatta gtttatattc aactgtaaaa

1479181 ggcgtaactt gacaacactc aacaaaactg ctccagttcc ttatcggatg ccttcacaaa
1479241 tacggttcca gcgccttcag acctcgtaca tgctggctca ccgtcccttc ttttcccacc
1479301 agccccccta cctgagtgaa gcagggtaat ttctcctctc cccctgcatg tggaggaggc
1479361 tgctggctct ggttagtcag agctgtgtgt aaagtagccc acgccagtgc tttaacacac
1479421 agcggaaatc tgtttgctcc cagctgctgc tacagtaatt actgtcctgt ggttgctaag
1479481 agatgcttcc tttgactttg ttttcccccc ttgctttttc ttcatcccca cttctccccc
1479541 tcataatgct gaaacgtggt gcgcgtgtgt gactttgttt tttctttacc gccaaacata
1479601 ccgagcggcg taacgcacat tcagagcagc tgtgggggga aagagagaag ttaaggatgg
1479661 tgggagagaa agaacaggat agggaaaaat gacagagaga gagagagaga gagcgagaga
1479721 gaggccaaac agtctgnnnn nnnnnnnnnn nnnnnnnnnn nnnnnnnnnn nnnnnnnnnn
1479781 nnnnnnnnnn nnnnnnnnnn nnnnnnnnnn nnnnnnnnnn nnnnnnnnnn nnnnnnnnnn
1479841 nnnnnnnnnn nnnnnnnnnn nnnnnnnnnn nnnnnnnnnn nnnnnnnnnn nnnnnnnnnn
1479901 nnnnnnnnnn nnnnnnnnnn nnnnnnnnnn nnnnnnnnnn nnnnggaaac agacttgcct
1479961 tccttttaaa gcacttgcct aagccccact attcaaaggc atgcgtgggg gccaatctgg
1480021 agcttagctg accccagaga tgtggatgtg tgagaggaga ggagaggagg ggagaggagg
1480081 ggaggagaga tggagacgga gatgagaccg gggGaaacct ggaacttcgc cactgatgga
1480141 tggatgttca ccataaagac tggcacacac attgaaaaca tacacaatgt gcaagttccc
1480201 aaaaaaaaac cccaactgtc aacaaagcat ctgccacagt tttaattcct ctttcttttc
1480261 atttgagaac atcaacgcca ccgaggttaa caacatcagc tgcctgccac tggtgcactt
1480321 cctgatttac acttaaatgt atttatatgt ctcaaaataa cagtattaat cattcatatt
1480381 gttgccacat gtgtaatgct gttgtgtttg tgtgttgtaa gacattaaaa acaaagatgg
1480441 cagaaaagca aagcggtttt aactttaaca aaagccactg tttcagggaa gccctgatga
1480501 ctaaacaaac tagctttatg agaagtcaac agcgagattc caaggttatt acaggctttt
1480561 attgattgtt tcactgcagg gattgtcata tttaaattcc atagattctg ccctgcccac
1480621 ttgtgttttt gtttcacaac ttccttgtgt gatttgagtg ttaaagactt tagaactttc
1480681 agacaaacct agccaagaag gagaggctaa gctaaatgtc tggttagcca tttgtcccgg
1480741 ttctactttc tgttgcagtt tcaactactc tgtcattccc gtcatgcttt ctgcacacaa

1480801 gaacagatag taatccatca tcgacgagac ctttaaacca atttcctcaa aaaggccaat
1480861 ccagtatgaa agtctcttgt aaatgaggga cattattaaa agtcagctgc aaacaaaacc
1480921 tctgtggata aactgataag agaaaacttc ctgccagnnn nnnnnnnnnn nnnnnnnnnn
1480981 nnnnnnnnnn nnnnnnnnnn nnnnnnnnnn nnnnnnnnnn nnnnnnnnnn nnnnnnnnnn
1481041 nnnnnnnnnn nnnnnnnnnn nnnnnnnnnn nnnnnnnnnn nnnnnnnnnn nnnnnnnnnn
1481101 nnnnnnnnnn nnnnnnnnnn nnnnnnnnnn nnnnnnnnnn nnnnnnnnnn nnnnnnnnnn
1481161 nnnnnnnnnn nnnnnnnnnn nnnnnnnnnn nnnnnnnnnn nnnnnnnnnn nnnnnnnnnn
1481221 nnnnnnnnnn nnnnnnnnnn nnnnnnnnnn nnnnnnnnnn nnnnnnnnnn nnnnnnnnnn
1481281 nnnnnnnnnn nnnnnnnnnn nnnnnnnnnn nnnnnnnnnn nnnnnnnnnn nnnnnnnnnn
1481341 nnnnnnnnnn nnnctagtag acacagacac acacacacac acacacacac acacacacac
1481401 acacacaggg gggggggggg ggggggggtta agaagcagaa cccaggctaa cctcagaact
1481461 tgtgctggta tgtgctagct tctttagctg cagcacccgc aactggttca cttgtctttg
1481521 tctgtagccc aggtctccaa ccgcaacatt gggatgccaa atcaccatgg tgacacccac
1481581 ggtggctcac tcactgaacc ttgcgaacac cccacttcta ccccccccaga ccctgttgcc
1481641 tagcaactgt agtcccattg gatttcctgt atgttcagac tgattcctta aagaggaact
1481701 tttgcaacag aaacaggaaa gcagcactcc catcttcacc gcccacgatc acgaatgagt
1481761 gtcgcacatg taaatctcat ttatctcaac ctgtcaacaa agacgaccag cagataagag
1481821 gggcagagta gccaaaactc cgatttcact tactgcttgt ccgtcttcag gagtgtcgcc
1481881 atctccaaat gatcgacaac ctaaaagaga aagcaaacaa cccacagatg aataaatata
1481941 cggtgccatt tgttttaaat aaaggaaatg atcatcgttt gagcgatgat attgtgacat
1482001 gcataatcac atagtttatt aaaacggaat accataaagg ctggatttca gattgtgatc
1482061 aagcgtccca caaataaaaa gccagcaagt cagataagag gctgagccgt gtcagacaca
1482121 ggctctgtac cttctacaca agcaacactt tgttaacaat tgactaaagt gataagaaga
1482181 gctcggacct caatcttagc tggtaaatga gctcatcatc tgctcataca gtcagcatct
1482241 ccttcaactc accttgtctc tacagtaagg aaatcttact ggctggtatg attcgaatca
1482301 gacagcaact gctgccagct aataaaaaag ctcaatgttt tgccatgtta ttgctcatcg
1482361 caagattcaa acattaaacg ttcttaatta ataaatcgat aaaaggtaaa ctagactgct

1482421 gcatttacag gcaatcgtga aagaagagga aatacaaata acacaatcaa atggacacag
1482481 acgtgcattt tcgctaacat aaaactggct gtattgtaaa gatggaacgt tgtaacctcg
1482541 attccaaagt ccacaattaa gcacggaagc tcgagttaat gactgaacac aacaaatcct
1482601 taagagaggg atagtgattc catcagtgct ttcttagtca gttaaaagct ttgttatcac
1482661 ttgctggatg gttctatgat tagcagacag tcaaagatct ccttccactt tgttggacag
1482721 taaaatgtaa taataaagtg acattttgcc ccaatccctt ttactactgt tagaaaggag
1482781 ataacaaagc cacgtctgat acctgctgtt ggaataaagg tactaaaatg acacaaaaca
1482841 agcagtcttt tttgcatgtg aatgctcata tctggaatat tccggcacag atcccattgt
1482901 gtcgacccca gtcagacatt cctctagact ccaccacttc tacgaatgaa gccctcaaga
1482961 agtgcaggaa aagaatggtt ctgagacaaa aaatatgatt acattttaag aagagcagaa
1483021 acctttatgg tggaagaggt cagtgcttct ctctttttaa ctggaaccta ttggagcact
1483081 gaaagctgct ttgtgtcagc tggccagaga agtttgtgaa ataagcaggc cagaaaattg
1483141 taccctacac atgactaaag catcacgtgc catttccacg cccaaaatat gcgtttgaaa
1483201 cattagtcta cttttggagc atcaaagatt tgtgtttgta atattttttc ccccccattc
1483261 tacagaaaca caaggtctga ggctgattaa agggcacatg tactgtgcat atacatccca
1483321 gaaccaaaaa ctgtgaaaaa actgatgtga tggtgtgtat ttctactatc aagtatcacc
1483381 aaggccctgt ctgaagtgcc aagccatcag atggacagac tctttgcagt tatgcgcgca
1483441 tatgacgtgc ataatgttgc acgcgcttct aaagaaagaa ctagaagtac tcaatgagcc
1483501 cgacttaaac ccagtgagtc tttcagactg ttcatctatc tgattcacat gcttcttctg
1483561 tgtgtgtaca gttctcctct gcagagacaa agaggcttcc tgcagcacga ctctggcagt
1483621 gctgggctgt gaagctgcct gagccatgtg cacagcattg ccatggcaac agagaactgc
1483681 aagactggct gacacacaca cacacacaca cacacacaca cacacacaca ggcaaaacat
1483741 attctctcac tccatccgtc tcttctctcg ccacacacat tcacacagac atccagcaga
1483801 aggatgcaac aggaatacag aagaacctgc ctgagctgaa gagaaattac acaacactga
1483861 gagaacacat gacagagaga gagagagaga cagagagaga gagagagaca cagagagaga
1483921 ggcaggcagg caggtaggca tggtggagac acactttgcc atcacctgct gttgtacatt
1483981 cacaggaatg agctgcagta tttagtagta gagaggaaat ctagagtacc tgggtgtgca

1484041 gggagatcag actaaagaac attcaacctc cattctttaa atatgttttg ctttaatcaa
1484101 tcaagcgatg aagaaatgaa caaagctgaa catttaaaag gtagagattg aattgaaaac
1484161 atgattcccg tctctgtcct taaataaaaa gtaaaaagca ctgctgcact tcaggtcgtg
1484221 aaacacattc actcggtacg ttcaacagta ctttacaaac cagacttaaa gtctcatctt
1484281 gccattaaga catttacttg cagtcctcct gctccattca taagaggata aaacactggt
1484341 gcttttcaga cttaactggg gtgacgatgt taggagatga tcaccattac ctcgacccnn
1484401 nnnnnnnnnn nnnnnnnnnn nnnnnnnnnn nnnnnnnnnn nnnnnnnnnn nnnnnnnnnn
1484461 nnnnnnnnnn nnnnnnnnnn nnnnnnnnnn nnnnnnnnnn nnnnnnnnnn nnnnnnnnnn
1484521 nnnnnnnnnn nnnnnnnnnn nnnnnnnnnn nnnnnnnnnn nnnnnnnnnn nnnnnnnnnn
1484581 nnnnnnnnnn nnnnnnnnnn nnnnnnnnnn nnnnnnnnnn nnnnnnnnnn nnnnnnnnnn
1484641 nnnnnnnnnn nnnnnnnnnn nnnnnnnnnn nnnnnnnnnn nnnnnnnnnn nnnnnnnnnn
1484701 nnnnnnnnnn nnnnnnnnnn nnnnnnnnnn nnnnnnnnnn nnnnnnnnnn nnnnnnnnnn
1484761 nnnnnnnnnn nnnnnnnnnn nnnnnnnnnn nnnnnnnnnn nnnnnnnnnn nnnnnnnnnn
1484821 nnnnnnnnnn nnnnnnnnnn nnaaacttct tatcgttgat cctccagtgg tgaaaaatgg
1484881 gggtgacctt gttgtacacc acatcttttt gtaggacgca ctctaagcac caccttacaa
1484941 cacagagttt taattaccac acaattcatt caaaaattat ggttcctttc acataacttg
1485001 angacctttt tnaattacct ttctctcaga atgttttaaa atatgtcctt tattgaaacg
1485061 acacgataaa aggcaatggt tgatgaattt gctttacaca ctttgttgcc aacgtagagc
1485121 accatcgttt tgctacatgt gaaagagctc ggtacccgtt ctcgtgtcac tgcctccccg
1485181 agatcaaaag gggcaaaggt taaacgtgtg tgctcacgca cagcgtgagg atgacagagc
1485241 tggtgtgaac aggactggct ggtacggggc tgattgagga ggtgagactc catggagcca
1485301 gggcaggtaa ggtgagtgca gagccatccc aaaagaagag gaagtatcaa cgttacacag
1485361 cagagtaaca catacccтta gaaaccaacc tgtacttggc aggatctggt cgtgacctga
1485421 acatttggtt acatttattg caggacaggc taaaatcatt gacaataaag tcaggaggct
1485481 agaccactat ttaaatagag atgtggtctg cgagccagtc atcagccaag aacagtgtca
1485541 cataacagtg aggaagaaag acagagacag aaaaaacagg atgggggggt gcaagatgag
1485601 gagtgactag tagctatatg gagaggcagc tgagtcatca ccgggacact ttgtgctgca

1485661 tcgctgcact tacacacaga taaaccatac ttctgtattc aaatacactt aaggctcaaa
1485721 aaaaggttgg gattcacaaa atacattcac agacacgacc aaatagactc cagtgccttt
1485781 tcttgttttc aacatcttat ttacacaata gtcaatcatt tactggtgaa attaaagtca
1485841 ccattcttcg aagatttgca tgtgttgtat ggtcacagat gcttccatgt tctccacact
1485901 gttatttttc tctctacttt cagacacctg tgtcattgag aaaagtcaaa cagccacttc
1485961 ctgcatgtgc aaccgtttga gcgaagcaga gagacaaaat ttgagaggaa aacctgagga
1486021 tgacgtcatc caagagaagg caccagctct tgcttctgca ttaaaacctc aaagtcacgc
1486081 atattaatga gcttgggttc cttaccaact tatctttgag cctctcgccc ttgatgtaaa
1486141 actcaacagt ggatgggctt ggactgggac tggggctggg gcttggactg ggactgtggc
1486201 tgcaggggct gaggttgttg ctgctgcctc cactgtgggt gctgctgctg tcatccaccc
1486261 gactgacctt atagatggtg acacagctaa ggcctccgcc ccccaggggc agccacccac
1486321 cgctggagtc atcccgagtc atgaccactg ctctcacacg tgcataactg tcactgtgga
1486381 acaggaagga aggagaaaat taggactagg aaggaagctg tgagagaaag ctgcttttag
1486441 caaccacgat cagtccttaa ctcgaatatt actgacatca taagctaaac aaggatacat
1486501 aaaatatcac caaacataat taagctttgc cacagattaa actggtatta cagaaataat
1486561 aatggataag ggaataaatg gtgctgtgac tgtatgcaag cgtattaacc aaatgccctt
1486621 gaatcgagga atatcttgtt gcaggagtgt gtagagaaca aatgagacca tgcagccaac
1486681 ctcaacacta tgagcagagg tataaaggta acagtagatt gtgtacatta cagataagat
1486741 aaaacataat gtaggttagg cattagaact aaaaacggca ggatctcagg actaatcaca
1486801 acgggctgtg ctggaaaggg ggaaatcaaa tgttattctt aataatgttt taattctcaa
1486861 ccgcaaagta gctatcacaa ctggacgtct ggtcagaatt aaatgtacat gaatacattt
1486921 gtagactcaa gatatcacaa aaactcagat tatacttagc aaggtgcaaa agatcagatt
1486981 atacttagca aggtattgga tttactgtat actaacattt gtctgctata acaaactgct
1487041 ggcctaaatt tgaaaagtcg aagccggctg atgtgtgttt acccccagtc tgaggtgact
1487101 gaccaagggg ccaaattcag gcttcagtta ctgctgctcc tctgacacgt tctagtcagt
1487161 cctgaaaatg ctgacccaat ataaggtcaa ataatgttgc cgttttattt gtacaacttg
1487221 aaaatacata aacctgattt ccagacatga aacataaaca aggatcactg tgtaaacatg

1487281 gtaaaaccta aattgccacc cagtaaaatt tgttgaaatg aaggtatcac aatattggtt
1487341 ccagtaaatc agtacaaagt gtgggccaca ttttaatttc tcccccctc cctcctccct
1487401 cctcctgctg tgcttttcag atgcttcccg attagatagc agagcctgaa tcaagccacc
1487461 ggaaatgaca aaggtcagtg tgtgctgatg gctacaattt gcagaacctc ctggtaactt
1487521 attaaagccg agcaagggta ttctgaaaac agaaaaagag taaaacttcc tgtgacacac
1487581 ggcggtagac atcacagttc acatgattgt gaacctgtga ttgaggagaa ccaggcgatg
1487641 actacaccag caaaagaaca aatacaccct ttccttggca gaaacatcat ccaaaggttt
1487701 ggtttgtttt ctgcacaatt ggaaaagctt gcagcctgct ctatatatat agagcaggct
1487761 ttttatatat atatatatat atatatatat atatatatat atatatatat atatatatat
1487821 gtatgtatat atatatatat ataaaatata tatatatata tatatatata tagctggatt
1487881 attactatta ggttgtttct aaagaaaata aaaacacatt aacacatctt tttgtacaga
1487941 tgacaacata ctccccttcc cctctcggcc gtgcataatc atatcctcct ttaatgtatt
1488001 tatgcctcca aatacttaag agtaacaagt ccacagaaaa aatatgaagg tctgcaaagg
1488061 agtcttgctt ttgtacatgt cattctcaca acccagtctg tacagaagcc caagtttgtg
1488121 aaaactccca ccatccattg acactgtggt gatactgtgt cctcccatca aagggccaaa
1488181 atgacaaacc attgtcatta tctggctggg aaaagtgatt ccagaggctt aaccaacatg
1488241 tgtggcatcc agtaacattt gttttaggtg atccaacagg acagcattaa gtatctcgag
1488301 gggcacatga caaagggtgt caagaatcat catcaagcat cattattata ggagtatact
1488361 gtaaataaaa gcccaaaccc ttgagcaaca tccaggagag tgcatgaaaa acaaaattgc
1488421 gctcgacctt tcaacaccgt ggacaagtac ccccgccaca gcaggaaaac gttcctaaca
1488481 gtggagcagc actggacccc ttgatggccc caataaagga tgcgggtgat gagagcagga
1488541 ggggctgtca atcaagcaag ggtccctcgg gtgaacagaa ggcaggcggt ggtgtcactc
1488601 agccatgacc agggcacaat gaccagggtc tgacccttaa cacctaccat tgcacacatg
1488661 ccagcagcca cgacactcgg agatgggggc cggtcccaga gaggaagttg agggggcttt
1488721 atacccacgg taccaaagct agcctaaaac aatagagtcg ggaaacgcgc gacgatcaaa
1488781 tactcacttt tgatatcaac tcaattataa ccaaaaccat ttcaaccaaa cagattaaat
1488841 gctatgatct tttaattgtg tatttggtat acaaacacat tttctgtggt cgtgatggtg

1488901 aatctttaat atcttaaagg atatttgagc ttcacttgtt tgaacaaata cacacaaatg
1488961 ggttagcaga gacaaaagac cggcctctac accccatgct gggagcacac agttgactgg
1489021 gcacacaact gggagcggaa gcagatcagc tggcctgggc ctgtgtgcgg ggtggggggg
1489081 tggggggtgc agagactcca gaaagaaaga aacccccatt tcaaattatg tcaagttatt
1489141 ttgctggatg actgacttta tttccgacca aaaggagggg tggaaaaaaa tctgaggggg
1489201 ggttaaaatg aggacaatgg gggtacaagt tggaaactat ggatggggaa actagggtgc
1489261 catcaaccgc ccctgctcac atttaaaggt ggctttttag ctccaaacag gacatccact
1489321 ggcttgtgtt gcatttatga agggctgagg aacccactga ggtggtacaa tcctgaatac
1489381 acatgagttc cagaactatt tagatatttc aggatctttc cttaaattgg agacctgtgc
1489441 tatgccaaga ggaggtcctc gtgtattcat tctatttaaa tgatgcatta tattaatgtg
1489501 gtcagaacaa acatctgata attgaattaa gagaagtgaa cagaagagaa gaggagaaaa
1489561 ggagaagaga agcatgtgaa aacagacgag catggttctt gtggccggct gctccagtgt
1489621 gtggtggaga agggggatcc tgggacaacc attccaagct gacaaagtag ccatttgttt
1489681 actgctccgg ctcatggctt actgagctcc agcatgccat catgctttgc ccctttcagc
1489741 cttaggcaca ccaccccaga gaggtggagc gaaggcaaag acactggtgt tgtcgggtgg
1489801 gtaatgaggg aaggactaga ggaaggagag gaatgaggag aggaataagg agaggagagg
1489861 agaaaggatg tggcctgagc cctgggggct cgccaagtca ccctcccttt cagccagtcc
1489921 agctccccct tctacccaac ctctcaccgg ctgcccgtct ccacacaatg gctgcctctc
1489981 tgctgaaatt tcacgttatt ggatcccagg cctgaagttt tgtcctgtca caatgtctag
1490041 gcagcggagc atgctgcagg ggagacacac tctgcaagtt aaaagctgga aaaccattcc
1490101 tcctttcatt agcatcaaaa aggggagaac tccccctcca cacacgcaca cgcacacgca
1490161 cacacacaca cacgcacacg cacacacaca catttgtttt cttcagcatt tctccggtgt
1490221 atcaaaccag aggaaagctg tttttaattt aatatcaaat ggagagagtt ttatattcat
1490281 ttatttgagt ttttagaaat agttgaagca tcactggagg ggggaaaaag aactaaagct
1490341 gcacagaaaa caccaaagag tacactactc acacctcatt gaagaaatta acagctcaca
1490401 ttctgggata taacatctga ttctggaatg caaactagca ggccagtttg ccacgattcc
1490461 aatctgcatt tcaaaaatga gaccagctac gcggacacca catttaatgg gaactaataa

1490521 aaataactgg gatgatgtgg aaagagtgca gcgaccagca tcacaagcct tgtgcattat
1490581 ctcctgtaaa aactgatgtg ctatagaaag agaagtgaaa gagaaattcg ccccgtctcc
1490641 tttctgcacc tcgggctggt gagtcatctt cctcacaaga acattcagct tagctacaaa
1490701 ctgagtcagt aaaaaaaaaa aaaaaaaaga gagagagaga gagaacagag aaaaaaagcc
1490761 aatcttctct gcttctcgtc tttttcacaa agaccactta atgacacaaa ttagctcatc
1490821 ccagtcactc ctgcatgcat gtgtcacagg caggaattac tggatcatat tagtccaaat
1490881 cagacagacc taaaccagca aagacatgcc aaaccggtac tttaacatgg atctcagaac
1490941 caatccctat attactggat taggcctgaa acccaaaggc taacatttga ggtgccaccg
1491001 ttccgggaca caacaaatca aaggaacgcc acatgtgtat cgatgccaca aaacgagcct
1491061 caaatgtcaa tcaaatccac atttagcaat tttctttgct caaaccgcaa gctttgagaa
1491121 tattaaaacc ttgtgttcac catcatgaga gcagaaagat aaaaaggcag accgtaaatt
1491181 tcaaccctgg ctacgtctct gatgttctct ggtgcaaggg tagtcccccg gggaccccag
1491241 tgcctacttt ttggaagcaa tttggaaagg ataaactgcc aaaccatgat gacagtctgt
1491301 gcatgtgaag agaaacagtt atgacaagtt tgaaaccatc acggcattcc ctgttcacag
1491361 gaataacatc agaaaccgac ctatttgtcc atcttagaag gaatcacagt caaagagctt
1491421 ccagtttttc ctcaaaacgc ccttcttcag atgcggtgca agcacaaagt cagctttatt
1491481 aagaaccgtg gcttcatgtc gtgcggtgag gaggtcacgt tgatgctaca gcaagagaat
1491541 acagatacac gacaatattc gtacccagat cttttccaga tgaagagatc caagagctgc
1491601 agtcattcag tcagaaaatg ttattagact gcaaacacaa gacaaccagt caccactcca
1491661 ggtttcattc tgtggtttaa gctggactgt gagacagcca aatggaccta ggacggccca
1491721 ggcaagtgag ctgatgctgt aactagctta gcagtgtaag tagcttagca cttgtcccag
1491781 gacgttagca gttggctaaa tcaacgtgag attatttact cttaaacagc gaaaggtgaa
1491841 gccaacactt tcaggacgag ctgtatgaat gtgttctaca tgtttgattg agcgtctgac
1491901 tttacattac atcactgtct ctgcagtcgt gcaggaaagg aacatggcag cgctctgagg
1491961 tcccatcctc gattcctttt tttttcctgt gcgcaccaca actgaaaaca acatctacga
1492021 ttcagacgct aatagctaga agcgcttctc aaaggcactg agatagcgtc tgggaacatc
1492081 agaggtccgt tctgacaaag gagtgcacac aaggcctggt atgcggatgt gctgatgaag

1492141 acataaaaac caacaagcat ttgtgacaat aaccacatga ctacaatacc acattcatgt
1492201 gatgcatagc acgggctgga gcgctttaca ttgaccctgt ttttgtgaat taggtacaat
1492261 cctacggcac tgttgggtgg gtccaagatt catagtgtag aacccattaa gatggctgtg
1492321 atataaacgc acagtacaga agagaaaaac ccaccggagc gatccagggc acaccatttt
1492381 agagggccgg tgtctggttc gccggataac ttttccgata actttagatc caaacgaggg
1492441 aaggggagca acatccatta ctcgtattta cagccagtca ttttggagtt acttgggaag
1492501 tgtgtggtta gcaaacaaca cactgtacac tgctcataga aaaaaaacag tcagaaaaca
1492561 ggctttgtct gagcacaaac aaacagcaca ttccatttgt catttagctg aaatgtgtct
1492621 aaatgagcca atatgacaag cttctatgat gataaaatga tagcgaacga agttagcgcc
1492681 ccttggccaa aaatgataca agttacgata cgatgcgaag tcggaaaagt ggcaagcaga
1492741 aattatgccg tttggtgaac caatcacccc tgtcggtgca tgggctgcag acagcccgct
1492801 gatgatgcac tacccccccc cccccaaact ccgtacatgc agaagcataa actgggatta
1492861 acaggagatg cactgtaggg gaaacactca caaggcaata acagcagagt gcttgtgcgt
1492921 cccaaacacc ccaggacacc cccatcgcaa acttcacttc actttttgca ttttggtagt
1492981 gaaacacaaa tggggtctgg atcacatttc agcctgatga ctttaccccc cccactccat
1493041 ttcttcactg atgctaacac ctttttatcc actgaaaacc aaaaaggaat cactgactac
1493101 ttttagactt caaaaaaacc caatccctga tgttgtgata tgcagtggaa agtgaaatca
1493161 gcattaacct ttgatacgac attattctac aacattaaat cacagggcca gacgcgcatt
1493221 taggcataaa caaacttaag tggcctcatg ctatggcttc aagagacctt tggccaacgg
1493281 acatttaaaa taataaaaca gtggagctct taaacccgag cgaccggcct tcaacatcca
1493341 caccccttata ggaaggattg tgcaagattt tccaagtctg gttgttttag aaacagtttc
1493401 tcttttgaaa tatagttgaa ttgtaagtca gatatgtgtg caatcatgct tgctcttaaa
1493461 atacacaaag aggaaatgaa aataataaaa aaggggaact gactctatac agaattgagg
1493521 ccttaaatcc aaccatggga attgaatatg cagtccctgt aaaaagccag gtcaacagtt
1493581 ggagggatcc aaatgggtca aaaagacaga tattgttcct ttaattgtga caaatgttgt
1493641 tgtccagcaa acatatccgc tcctcattta acaacattct taaagttctt ggaaaatgag
1493701 gagatgcagt tgtcaagttg cattttcacc catccgaact actcaccaat tgtagactag

1493761 ttcctgatgc tgattcagta acacagctgc attcacaaat gtcaaataca aaccttaacc
1493821 cctgaataag tgctgaatgt ggtgtgtgaa cgaacccccg tatctctgca ggccggggtc
1493881 acaagccaac acttccccat cagaaaacag aagcaaaagc tgagcgcact ttcaaaagag
1493941 aacatttgca gttcagatga gctcgctgca gcattcaggg agttgatact caagagacaa
1494001 taacttcaaa taacactgtt gtttaaaaaa agaaaagaaa aaaaagtaca gaaaatgatg
1494061 cagcttagaa taagggccaa caggatatag ccaattaccc aggccataaa gaacaagccc
1494121 caacagctga aggagctggc tgccctttgt tgttttagta catgatgggg gttttcggga
1494181 gggtgggggg gcgtctggaa agctctgcaa caccagtcca agcacaaacc agcctcaggt
1494241 gataagatca cgacacccac attgatagat aaaaggatga gttgtcaggt taaccataga
1494301 aacaggaata cgggagttga atacgggaaa cttaaagaaa ttagaccctg acaacaaagt
1494361 tcttgaagga aaaccttact gctataaact ttactgcaag caaaatgcat tgatggatgg
1494421 atgttataat agattaaaag aacccaacac taataagcat tgtattatat cataaacact
1494481 tctgggaatg aattgattcc ccttctgtga ccgtagatca ggaaatgggt agtagtcagg
1494541 gtaaagtgat tatccaaaat ggagttaacc cttcagaatt cacattagaa taaagacaca
1494601 cacacacaga gtgagaaagt gcctttaaat aaacccctca acagaataaa acaagcacta
1494661 aagtgaacct cagacaaaag cttccctttg tttttaaagg ctgaggttat tttacatttc
1494721 aggaagtcag tccagagatc tggggcttcc ttgacaatac agcagaacac tatgtcactc
1494781 ctttatcatg aaccgcatga gaaccaaata ggtggcggga tcatatcaat acttcaaaca
1494841 cgtcatccgt cacggccgag ccacgttgat cacgactgga gcctgcgcaa caccgccgat
1494901 cgttaaagag gatctgaaaa ggtcctttag attatcccaa aaaagtccgg gggaattcag
1494961 aaatggtttc atttcaccgt gtagcgaaag acgtgctgta gacaccagag ctcggatatc
1495021 aaacatgcta ggttaaccca cacattacca ggaaaccagc tccctgcacc attacaaaca
1495081 ccatttctca gtagaatttg ctattcattt ccttgtcgag gattaaaaaa aaaagaatac
1495141 tgcattaaaa gcaagcacag cacacctggg gggggggggc aacatctcgg tattaaagcc
1495201 ataatgtcgc gtgtcaatga caatgttagt gggtgaaaag gggcatatga attcctctgt
1495261 gacaaatatg tttttcacag gagttgtggg gggtgtgggg gggagtggct aaaacagaac
1495321 acggctaaat gatctggaat aaaagagaaa tggacacaaa aacgggcgtt aatgcggcgc

1495381 attgtacggc aaatgcagga aatgtgatca tcggtgcaat gcgaaacagt gtgcagccct
1495441 gtgaatgggc aggcaggtac tttatgaatg gagggggtca tgtgatcgca gcacctccca
1495501 aagcagatat ttctcactga ctgtcaacac gcacacaaga gatcagccca ttaaaacaat
1495561 accgcttcca aagccccgaa atgggcgcga cgcgcacaga aacggctcat atccgcgcaa
1495621 caccagctcg atctgcgggt ttgcgtgatg ccgtcccgtg tgcgtgatgc cccttttttaa
1495681 acggtttttt ttttttatga atgaaggctg tgatatgtaa atgagagtga ttgagaaaaa
1495741 aaaaaggcat gttgaggttt ttcgatatcc acgtcgccgc gatcgatccc aagcagctcg
1495801 cggctggaga gaatcggagc taatgcgaac acggtcggcg ttttaatcga cgattatgtt
1495861 ctgaaatcat ggcggacgac gccaagattg aagacacaaa gctttcggat catgtttaga
1495921 gcaaacacgc atttcagagc agactatgga attagaaaag tcccgaccac aattaaactg
1495981 cttcgctaat gtgtatttct tttttacccg caatatctcc tcaataaccc atttgtaacg
1496041 tagcatttct ataaagtggc cgtacgattg tcctgttaca cattataacc ctaaatgtct
1496101 attgtgcacc cgcacaatag acaatttcaa atgcattctc gttcatttgt ttcatcagaa
1496161 aaagagaata tagtaataaa cagattacca aatgacttcc gatggtgtcc ctgaacagac
1496221 aaagctgtcc gtgaactatt ttattgtagt aaaagtaaca cagtgtgtgt ctacctggac
1496281 taaaagctta gaatgtgtta ctaaatatta ataaagtatg gtgatgaaat acaacactgc
1496341 tcggttataa atgtcaatga gagctcgatg agcacgaact cgcataacgt gttgtggggg
1496401 ggggaagtcg tggatatatt agcagcaacc ttaataaaca tggaagaaat aaacatggaa
1496461 cattaaaatg cactcactcg ttgtttggat ttgttgaatc ttcactcatt tttttcctca
1496521 cggggatccg aatcctagca atagcatcga cggcgcagtg acggcgggat aaaataccac
1496581 atatatcacc attttcctca acatatagaa agcagccccg ttttgaatgg tgattaaacg
1496641 acgtcgggtc ggctctgccg aaataagcgt ccctgggcga cccaagagcg gcgttagctt
1496701 aacgttagct tcgctggctg gaactaacgt gaaacgagga gcgatgacaa tcgttgattc
1496761 attttatcct ccctggaagt aaaatcccac cacaacaaat aggccgtcgg catcatcgtc
1496821 ttcgacaaac gcgggtcccc tcacggcaaa aacgttacaa gatcccccaa agagacgcag
1496881 gaatagttac acacggcggc gttcggtggg ttatttatgg ttggcagccg agcatccatt
1496941 cgccaacttt aggtcctgga caacacttga aaaagcgccg tggagcgccg ctccgtcaac

1497001 gtgtccgagg gctccgcagt aaaggcatcc aaagccgcgg aaacggcgga gctgctctcg
1497061 gcgttaacac ccgccggtgg aacaaaacga tctagacgtg aaatttcctg gtgaagttat
1497121 cagacatctg ctttcttcga actccatttt caagtcggga gggggggat gaaacgtcgg
1497181 aaaactgggc ggatgtgtcg accctcgctt tgagaaaatt agatccttcc gcgaagtagt
1497241 aaaaacacaa cattcttgct ataaaagggg attgaattgc gctcggcgtg gatcatattg
1497301 tgaaccggtg gcggacattc tctcaaacct gtcactttgc gtccttgcaa accagaactg
1497361 agcgcgattc attgcacggt cgacccaaac tagtccctgc acttcccaaa agaagtttcc
1497421 cttcaatcac gcaaggatac gtgcgggaag cgtccgtgtg atagcacgca aagttcttaa
1497481 taatgggaag gagctggcac caacccggcc aggaacgatc tcttttcctc attaagcgcc
1497541 gtgcgtcgcg ttttaagcgt ctttatatga aacagttgcc tttaatgtgc gcgttaaatt
1497601 agacctcatg atttgtggct gaatcacgac agtgttcaag ttcagagatt aatgaaaacg
1497661 gactgagact aaaacccctg ctgcttattt tggtttgcat ggtagactga tatggtttca
1497721 ttagccgcct gtgtgagcca cgcttcatca ccattcagct gccagcaggg gtcttgaact
1497781 tgcccacttt atctggttga agggaactaa actagttttt acttaaactc ccccggcagc
1497841 tgcaaaatga ttccaaaccc acagatcatt actaatttgt tcttctatga attttatttt
1497901 cagatgtgat aaaagccctg caaagtgaat cagcagacgg ttgcgtaaga cttaaacgct
1497961 attggatttc tgcgctgtgt ggaatagcaa gagatcaggc taaatgattc tgcctggttg
1498021 tcatagtgac agcatgttgt gtgagaagtt tctttgtatt tctctggctt tcatacagcc
1498081 tgtgccacag ctttacattg cacagcgtat aaaacaccct ctgtgtcgcc tatactgtat
1498141 agcctgagta gaggacactt gcatgtgtag catttagaaa ttctgtatat ttgatttgat
1498201 taaaccttct gtccaaggtg tttactgttt actgtactga tagtcactgg agagcaaagg
1498261 ttaattgcct ctcaggtgtg ctgtcttagg ctcaatcctg gatcttagcc tgatagttta
1498321 tgtatatgtg cagtacatga gattaaaaga aaacaaaagc ctggcttctt gattggaaac
1498381 cgagcacaat aaagtggaat ttcccacttg catgcctgta ttcttcagag gatttcaagt
1498441 tcaggaagtc aacattcttt ctcatctctc aaatgtggac gctctagagc atttcctgta
1498501 ctttttcaca cgcagtgatc acaacaaact gtacttaaac aaggcaacag gatgtttggc
1498561 tgacagaggc atccctcggc ttctgtgcgc tcagtgtcac cacacttctc cttcagactg

1498621 taaacagcat tcctatagct ccacacgcaa aaccacattt agcgaaagaa
tgcaggaata
1498681 atatacaatt cattcaaggg tttctgtaac tgtcagcaac tgcccaataa
ggtgttgctt
1498741 ccattaaata gcattaacca gcaacgtgag ctgaggtgct tccgctgtga
aaaatgaaaa
1498801 ttctggataa ttccgcagga cattggacac atggttgcta ttgcacaaaa
cccaacctga
1498861 aaatatcgaa tgaagttttc attcccggcc gtggatgacg tgtgcaaacg
tgagaacaca
1498921 ggtgacgtgg tcaccatcag ataagctgct gtcacaaaga gaaggccttt
ctttctacag
1498981 caactttgaa aaggaatctc cccctctgta gttgccgcca gacagcgagc
ggagacctca
1499041 gggcttccag cgaggaaggc cggagatggc tgacaacagc gctcagtaaa
taacctaatc
1499101 ccaggatcct gtgtcccata ttaaccgacg cccccggttt tcccccaaat
aagcgaaaca
1499161 tgtttagatt ttggcagata tgtgaccttg cagattcccg ggtcgtcccc
tcctttcttc
1499221 ggtcagaaaa caacgtcgga catggcagca aacaggccag agggcggggc
tttgacaaga
1499281 tgtgctgatt tttaaaggcc tggcagctga acacacctat gtttagtgtg
ggcctggcct
1499341 cggaagcctg caactcttaa ccacactaat tgcagggtgc tgagaagaaa
aggtgtagtg
1499401 gccagagggg aaggaagtgt gtgggcctaa aggcatgttt gcctctttgg
tgtgtgctgg
1499461 ctcgctctcc ctaagtgtgt gtggatgaat ataaccagtg tgcaaccgaa
gacgccaaca
1499521 ttttacactg cccatcaaat tgctcctgaa agatctgatg tttttcagtg
aagtccaaga
1499581 tctttctctt tgtttttaag gttgcgttgt ccgaatgtgt aactatatat
acagtctata
1499641 tatggtatta gacttctggt catgtgacca cgcacacaga cattcaaaaa
cagacaggaa
1499701 ggaaactgtt agaattttaa ctggatgtaa gaatccaaaa atcatgcttg
tttaggatgt
1499761 aaacgagcca aacagtgtgg cgatttctat tttatatcac attatatcac
tttttctgct
1499821 gcatcttgca gctttttaaa aaaaatgcaa gtctacccat caagtttatt
agcatctatc
1499881 tgtctgtctc tagctgtgca gtcgaaaaaa atggtttcaa caaaagttaa
attctcagtg
1499941 ctcgagactc tgttacttcc tattgtttgc atgttgagcg tgggcttgat
tgaaacctga
1500001 tctagcctgg agaagctgtg tttagggtta ggtggacagg cctttgttgt
tgcctgaggg
1500061 gtgttttttt gggcggcttc agtcgacttc cctgtaatca tcatgcagtc
tcacaggata
1500121 gaggagatta atgaatatgt tgggggagag gaagtcgcag atggctattt
accattctgc
1500181 gaaaatacgg cacgagaagc acgtgacaag gcgcagggtg tgtgcatggg
tgaaggcagg

1500241 tgtctgtttt tgtgaaacat gcactataag atgtgtaacc agagcagtca gcgcgtgctg
1500301 aactttgagg ggtagcctaa atcatatgtt ttacacattg taaagacaaa attctcttca
1500361 ttagtgttgc ctttaaatcc tgtttaccta tgccagacat gctaaattgc gaataagccc
1500421 acagcaataa ctttttagtt tagctttgcc atactaggaa aatcaagaat acctaagcaa
1500481 ctgtatcata tgtgaatctt tttttaacag tttaactttt aatgcccatt atgtgactac
1500541 atgagctgct agtcctcttc cccctaaagc tattcagcta tttgttcttt aggaccatat
1500601 ataccttgta cctggtccat aaagaaacac aagaccttgt taaatagagt accccacaaa
1500661 acacagaacc atcaactgca cagccccatc cctccttttc cccaatctgg gcctccttca
1500721 cttaatcctc atgctcatct gggaaccacc agctcccttc caggaagaca cattatgccc
1500781 agattatttg attgttgtca gatgtcagag ctttcctctg caaaaaggga agaaccaggt
1500841 tgtggggtgc tttgccaact gctggacacc tagaaataaa aaaagaatgg gggggtgggg
1500901 gggggggga gcagggagca gacacacaca agtcaggaag tgaccctggc aatgctagat
1500961 tacaaacaat gtgctctctc tacttctgca gtccttgctg tggaacatac cactgctgcc
1501021 acagccacag gggtggcgcg tgtgcaagga gggagggttt cagctctccc actctccttt
1501081 atcctgcatc cattcagcaa gcggcgtagg gagtgggtgg gacgggtggg tagccgggcc
1501141 agaaaggcac ttcactgatg aaaagtcacc acatggaaat ggatgagaag tgggctgaga
1501201 aggcaggggg gagtgagaga agggtgggaa ttggcatgca ctaccagcag acatgaacag
1501261 gggggacaga gaggagaagg gagggaggag gtgaaggatg aaagatgagg aataggggag
1501321 cgagggataa ggagagcaac ctaggaatac aagaatggga gaagggagcg taacggtgag
1501381 ggaggaatgc cagagattaa tggtctgatt caagacttcc caagcatgga gtcccgtgcc
1501441 tgtcagatag catgtaacca acagtcttct cctctgtctg atggaggaat agcgcatgtg
1501501 gacgggaagc agcacattaa cgctgaatga agcttttgtt ctgcggatgt tgcgccttta
1501561 catttgacca ggctggatga aaccgtcttc actcccactc cttgactcac cagttcagcc
1501621 ctccatccca accttctgca gcctcagttt tactctctcc acaacacacg gaacattttg
1501681 tcatctacaa gaacataaat atgtattttc ttcattcatg tgacacttta gttattgaga
1501741 aaataaccaa aatcagacct gggagatgaa aaaagagacc tggaccaaca cacacatgca
1501801 cacgccaaca cacacactca tattccaccc ccatctgcac tgtcaccgtg actcacggag

1501861 tgttgcagta ccgtatgaac ttcaaaagga ggctggctgt acattcacag
tgtattcagc
1501921 ccttgtgctt taaatagctc aacctcctcc aaagaagaga gagagaaaaa
atcaggcatt
1501981 aaaaatgtct gaatcataca agaattaaaa gcggcagaga acttctttga
aaagaatgta
1502041 atgctcagcg gcagcagcat cgagcagaca atgcgagttt gtcttgattt
atgcctgtat
1502101 gtgtgtttgt gagagggagg tagcacggat ttatgtgggt gagcgcagaa
tgtcaatgcg
1502161 gtgatataag tttacataat tggggcttga ttaaaaacac agaccactga
aaaatgctgc
1502221 ataatctgta caggcacatg tgtggcacgc acatgtcagt ctttggtgtt
gagaccaaac
1502281 ctgttcctga gctctgtgtg agtgtgtgtg tgtgcgcgtg tgtgtgcatt
tgtgtgtgtt
1502341 ctggaatgta cctggcctga taggcaggcc gaggaatgct gaacactatc
tctcacccgg
1502401 ggaggcaggg ctggaatgcg gccctgttct tctctctgct gtgacctgca
ggttcacact
1502461 tcacacatct ccaggttggt tgcatttcca gtttagggct cggttccact
ctttcacacg
1502521 catctgtgtg atggctgcag gtgtggaagt gtcttcccgc gcgcccacgt
gtgcgcacgc
1502581 acgcgctgga tgcttcaaag tgcaaaaaat ctttagtttc tacacttttc
attacttctt
1502641 gcattgttat tgatttatcc acgtgttcca acccaagagg aacctgccag
aggtcccctg
1502701 tctttccact ccagcccctt aaaaatctaa agtcagactc tcttgtaatt
tgatttttat
1502761 tttatttttt gccacagaaa cctttaaatt aggagcaacc acagtgcaga
tttggcagaa
1502821 ccaaaccttt ccatgcatat tgcacccatg tgcactttaa tttggacttt
gttgttttat
1502881 gatgcagcaa caacatgccg cacccctcag attcaagcaa acgcagacgc
agacacatgc
1502941 ctacatctga ccaggtcctc cgtcagcatt ttcttcactt caggcacatc
tgagctgcct
1503001 ctggaatgag ctcactacct tacagtcaga tcagagagtt aattaaagag
ccacaggaag
1503061 tttgggagaa gttggtctca atggttttcc tgtctgcagc ctcccagcgg
ctctcacgca
1503121 tgctagaaaa ctgggtctcc taattgatct tacggcccag ttcctgcatt
aagggtggac
1503181 ctttcacagc ttggacattt ctgactctta ttctgaagtt ctgctctctg
aagggtcaaa
1503241 cacatgaaaa cagatttgga tggatttgtc catgaaactg atcacatgct
cccactccct
1503301 ttgacaagaa tttacagaaa agacaacaat ggtggcattt tcaggattcc
atttttttc
1503361 aaattcattt ttcttgagag gaactctaaa aaaagaaaga aatattccgc
acagtgctgt
1503421 cattcagtcc caatttactg catggcagtc ttaagacttg cacagccaac
tgatttttcc

1503481 attgatttca tttgatccct tacacagagc caggatctct agactgctcc ccagatggga
1503541 tctatctatc tatctatcta tctatctatc tatctatcta tctatctatc tatctatcta
1503601 tctatctgtc tgtctgtctg tctgtctgtc tgtctgtctg tctgtctgtc ctgattgtac
1503661 acgtgtgtta catgtatgtg agctggtgtc ttggtacatg tcaaggattg tttgtacatg
1503721 tgaaactgga aggacatcac gtgtgatgtg taggtgtgtg aatgaagaat gaagctgccg
1503781 cgttgctaag tgacccccga gcacgttcac tggtcgcgtt tatacaggca aatgctgcca
1503841 ccttgtggcc aaaagaaagt attgatcgtt gctttaaatg tggcgtctcg gagctctctc
1503901 tccctctccc accctctctc cctccctctc tctccttctc tctatctctc catctttccc
1503961 acagctcgaa cagtgattca aggattcgaa attattcatt tgaatgaaat tatatagaaa
1504021 aacataaata aacaaataac aataaactta aaaccaaaat gatcatcgag aacagagaac
1504081 aataggatcg ccccaagaaa atcaaactgc agtatagccc catcttgtgg ttgaagttaa
1504141 tagaaataaa atgagagtga ccagataaat ggattttatt ataaaaagta aatgttatta
1504201 atttactcca agtacacaga taaaattctg ctgtgatcct aataattatt attaaaaatc
1504261 ttattccaac acatttacaa ctttctcaca ggtaaaagac ataatgtcat aataaatacc
1504321 caaacagaaa ggccacagat tatgcatggt gtaaagtgcc tccaaaaaaa gtaattcaaa
1504381 ttaattaaaa gcaataatta acagtgatta aaaatcatcc aaaatagaaa tcattaattc
1504441 ctctcagtga aatgagatta agcacacttc aacgatttta aacaataacc tttgtgaacg
1504501 cacgtgtcac gctcgtgcgt gaagaatgtt gccagggcag atttgtgctg agcggcacca
1504561 aaggtgcggc tctccttctg tgcaggtgtg ccgtgtttta tcagcaggtt tactctgcaa
1504621 atgcctgtaa atgttgttcc tttcacacag atcatacaac tacactgcat tctctcagcg
1504681 aaacagaaac ttcagagcca tttgtatatt attaacaata caaataaggt gtttttgact
1504741 tgaatattaa tgtttgaagg ctgctgtggg ggattttatt catgtgagtt tgaggtacct
1504801 gacaggttta ctcaggctgg gtcacatgac caggaagtag cccctttacc gttccaaagg
1504861 aggccatctt cgtcttacct gggccttgtt tatagtctga acacagcaga aatggatcca
1504921 gatgcaccac atctctctct tttgatgttc tgtgtttaag atgatggact ggaacggtct
1504981 ggcagttcca accatcactg cagaatcggg gctgggaagc agactttgac ttgttttgct
1505041 gccttgatga aagaagagat ttacgttgta aacaagaccg aagggagagg aacatggacg

1505101 gatggcttta tcagaggcaa acacgcgttc tccgacagcg aggaaaactt tgggattgtt
1505161 ttggccacgg ctgcaccctc gtcctcagac atcaaccaca cgtgcgtctc ctcgaggcgt
1505221 cgcacgagct ttgatcaccg agatgtgctg gtggcgtcag catgaaggga tgagtgtcat
1505281 cgtcatttaa cacaccctct ctgagagata aacagcacac acacacacac acacacacac
1505341 acacacacac acacacacac acacacacac acacacacac acacacacac aaactttatg
1505401 acatcatgac atcttggcaa tacatttgcc cattacttta atgcctttta tcaaaacttt
1505461 gcccaataac tgaagcaacg gccaactttt ccaacaggtt catgcaagtt tgtttgagga
1505521 gggctgccac tggcttttca gccaaactcc aaactctaga ttggatcatt ttaacttcct
1505581 tttaattaag ttttggagga aaatattgca tatgtttctg ccactataca cttaattatc
1505641 agggcattta gtttgtggat cacacggcat caaatctctg agctcaggta caaaataatt
1505701 ttgttgcatg aaccttcatc tgcatgatga atgagttgtc agtaataaaa atttaaagcg
1505761 cacgtgtact ttaattattt aagtgaatgt gtgattaaca aaaatcatca tttcattgac
1505821 actctgtata tcattcatgg ttgcaacgtt tataggaggc attcagtact taatgtttaa
1505881 aaataatttc aaggtcacct gtatttatct agtggagatg atgtctgtat ctagtggaga
1505941 tgatgtacca cagtagttaa gtatgaccct ttgattcttc ttaagttaaa agtatactat
1506001 taacaataaa gtattaatga tatttgtacc agcgatatag tgactcagtt gctccacaag
1506061 agggcgccac agagattaga ttaaagaggc tgatcagtat tgcattctga aattgcaaat
1506121 tgtgaaaatt tcacattttt taaaagtgta aattaagatt tcatatggtt ttgtgatcaa
1506181 agaattttgc ttgctgatgc atgtctcata aaatgggggg gctcagattc aactaaaaca
1506241 gatcgaatta aaacctccga gccactgatc gacagtctgt tacagtgtta gattgtttta
1506301 actatgtatt tcagtgtatt ttaatattat taattagatt gtgactaaaa tgaaactttt
1506361 cagattattc cccttgggga cttacatcaa aggtaatata taagttactg atatgattat
1506421 ggcgataaga tgctgagact atgtaggcca cagtcatata aagcacctga tcttggattc
1506481 tctgcactgg aagtgtgccg ctgcctgcac aacagggaca aacccagcag ctcctgaaga
1506541 gcagagctcc gagtcgctcg ccttgctctc tgaaattggc atttcacctt cagctagagt
1506601 ctgagagggg aagggggggg ggattagaga ggggggcggg ttccatgcta tcagaggaag
1506661 ttcatcctct gataaataca tagctacaca tcccactgga cccgctgtct gagacggcag

1506721 cctaatgtga gcaatctgag ctgcaccact acaagagggg gagccacgtg gagccccctg
1506781 ttttgtcccc aggttgtgcg tttgagagct ttagaggatt ttaatgtgtc tctctgtacc
1506841 tcacactttt ctttcgatgg ggctctccag acgcaacttg gcgcttcaca gaagacatgc
1506901 ataatcacgg cggaggggag gggatgaaac aaagaggaga tgtgtaaagc atcatataca
1506961 tatgctactt tctgctgtgc ccctttcagg tattcttatg ggtgttttgg ttttaggttt
1507021 gattcttttt tttccttcct tcctgtgttg ttacacacaa aggaaacaga cacagctcag
1507081 tacttcaatg aggccccagc aatcccagac agaccacgtt tccactggag catgctgtgc
1507141 tgctctgccc cgcgctgcag acagacagga gctgtcatga tgaagaggag accaaggctt
1507201 ctcattaaaa aaaacaccgc cgtttcctct gcagattaca gtgctccggg aaaactacac
1507261 agggaagaag gcaccgtcgc tgcagccgcg gctgcaagac gcgctgctac cttattggaa
1507321 gtgtcaaata aagcatacag tcggaggagc cttaatgcag agggctcaga aaaatgatta
1507381 caaaacatgt ttaatggaaa tgattggcag cttgttgaca gataacgctc agcgttctat
1507441 ttattcaggg atgttgaggt ctttggggac agctgtttgc ttacagatgt gttcctgcac
1507501 cttaaacctg gtcaaggcct tgagaccaaa tccttatgat gcatgcacag ggcttccgtg
1507561 catgtgtcat gttcatgcct tcctcttctt tctgcctcat ctcctcttct ccacctactt
1507621 tactcagccg ggcatcagca ggagggttcc ccccatgtga gcctggtcct gctcaaggtt
1507681 tctttccgcc actgttgctt gtagggggg t cagactctgg gtttctggaa agctcccagt
1507741 gactactctg actttaacag acaataaaga tgatttgact tgaattgaat gacacaaagt
1507801 gcttctctct gcagcgtggc caacgttttg tttaacaaat cacaaggaca gtgcaaccag
1507861 attggctttg ggatttcact cccatctgga ttttattgca gcggtaatgc agcacaatat
1507921 gcagctagtt cccttccctc ccgataggat cggaagttat ttaaaagggt gtgaaagttg
1507981 acagtgtcaa agagtttctt tgttcacggg ttggtctttt acgaacacga acaggtcaga
1508041 ggcgatgata aatgaatgag tgtgggttgg gattgtacac ataaagaata agctgtatgt
1508101 gattatgtgg gggcagccct tgtgcggtgg tgtctgtgac acactgatct gaaatggagg
1508161 gcggtgcttg acagtaaagg tgacacaatg gctcactgtt acagcacaaa tcacacatgg
1508221 atggccacaa ataacaggac gatgacgtat agaaggcatc agaagttgtt taattacttt
1508281 tcctttttct agatgagata cagcctctct gatattgtct gtgatattct cagcacacct

1508341 gtcaaaggct cgcggaagtg gaacaagtgt aaacatggta attaccgcag ctcacggctg
1508401 cagtaacagg agcggcctct ggatggcagt gtcaggctgc gctagccgcc gccggcttca
1508461 gcccgctctc attagtgagg tctagcaggc ccctctgaca tgtctcataa atgtcaacac
1508521 caatacagca ggggacagtg ggcctggatg taagcaacac agacgctcgc aaagtccacc
1508581 ctgtctgtca aatcaatcac ggagttaagc cacaagctgc gtgttcacgc ctcacatgga
1508641 aactacctgc aagtttgcat tggccgcatt ggggaagcgg aaggtggatc cctgtaggct
1508701 cgtgagggat tattttaatc tcaaatgtaa catgctcttt tggcgtgcag gggtgcgtca
1508761 ctgttcttta gaaacgtatt tatggatgtg tgtttttaag tcacatgcgt gcctttcgtc
1508821 gttgaggtca gtagctcttc cccttgatcc cccaccctct ctttcttctc ctctttccct
1508881 tcccatcctc ctgctctttt ccattatcaa gccgtgtgtg cctcgtgcac cctcttccat
1508941 tctcctccaa tctggccaca ggagcaggtg gactggagat aagttgcgtc gtgctttgtt
1509001 ttcgctctga caccgtcatt aatcattgcc caagccacac ttcaacatat aataataagg
1509061 actggctgct gtgtgggggc tgccgcccaa ttgcctttca ttgcatgaat gccaaattgg
1509121 aggtgaacgg ccatagccag tcactacttc cctgtcccca gactgtctca tcatttaggt
1509181 atttatccat ctcagccttg ttggtggagc ttctccctgc cctcccagga ggcccaaggt
1509241 tgttaggggg gaggaacaga attgttgctt gggctttttt ttttggcttt gattctgtgc
1509301 ctataagtta gggattagaa gcaaatttcc cacagggcag cggtggattt gctggtgttt
1509361 aactgtttaa cctttctcag tcttgtgtgg ctgaggggtg tgggcgtcgg agggagggag
1509421 aggaaggagg gttttggagg atgactggtc aacaggcccg gagagaccca catgggctga
1509481 gacagatcca ggccagcatc gataagaggg gatccaattc atcatagctt gtctgtgtgt
1509541 gtgggaggat ggagagagtc catacaaaac atcatatcct gcatgtgtga cacgcatcac
1509601 ttgtgaataa gcagctatgg tgaatacagc aaacaggcca ttgagttact aatgagataa
1509661 gagcagcagc cacagtcaca gaaacatatt aggatcaagt acagattccg atttaaatta
1509721 aacacaagca caacaaacct attaaattta ccttatttac atgctgtgtt gtaggaactc
1509781 atgcctctga accataacga cttcctcaac tgactacata tgtatttttc agaaggtgaa
1509841 aataggtata aagcaaaatt ttatgcaaat acaaacttaa tgagtgcacg gccagacgga
1509901 cccctcacct ttactcctcc tcggccttct cagaacgacc gcagctaaca tctgttcacc

1509961 tcactgtctt cttcaacaga ctgcaaacta aaagcacgga tgctgacagg gtgaccctttt
1510021 ttgttaaatt agcccggtca aatacacagg aggctgatta cagcaatgct cctcagacca
1510081 gagcacaaca acacctcctc tggccttgaa tagagatgga tgcagaccca gaggagcagc
1510141 attatcttgc cagacactaa ccacagacaa ctggtggaaa ctttggaatc ttagcacatg
1510201 tctcccccc ccactccctt gttgcacgtc ttttagtaga atgagaagat gtcagcctgg
1510261 gctatttacc caccaagtat ctcacatgta aatggcataa ttgcattttt ctattcatca
1510321 gtccagacca ggtcttttct ggtgctgtag aaatatactg cactatcata ttgatcttta
1510381 attaaagaag ttcttaacct tggctggtca gaggaccccc accccccagg gcagtgagtt
1510441 aattaatgta ctaatagtca taatatttgt actgtgcacg ggaggtacat aaaagattat
1510501 ctattgtgtg ggcagtttat cagggctatg atgctgctgc cccgtgctgt gcatatatca
1510561 atattatggg caaaaaaaaa aaaaaaatct gcaatctggg agaagaggag caagagtcgg
1510621 tctttttaat aactccctcg gttccacagc actttagcaa ggccccgttt cacctcctgg
1510681 atttctgctc tgatcgtaac agtgggcatc gatgggattt tacgggcgct aatgctttgc
1510741 tgccttgaca acgcagaccg gccgtaagcc tcctaaaaga tccacttcta ccccggatga
1510801 gtcaggggtc cccactccac cgttcacccc acgacgactg accccctccag cagaaaaccc
1510861 cctccataaa aagatcccgc cgagggagag gaggacggaa ggtgatggcg gcggtggtgt
1510921 ggcagacaca cggtgatatc actctttacg gctttattga tttcttctca ggaggcaact
1510981 attaacattg agctgctgct gccgtgttgt tctctgcagg taggaacaca cccctcacac
1511041 ccctggcccc ggtgggggga gcacagggaa agcgcatgct tttataatta aggtcagctg
1511101 ggatgcatta cagcatttgg aggtgttaaa acgggccttc ctaagagagt atgttggcat
1511161 tgcccggctc gtaaattagc cccgctccct gcagatgatc tcgggaatgg ggtgggtaca
1511221 ggggtgggaa ggagcgggat tagttaggtg gggtggatgt agacacaacg ctgggattta
1511281 attgtgtgat gttgttattg gtgcccccac ctgcccccaa catcgatttt ccgtatgaga
1511341 aaaagtaacc tgaagtgaca gagtaacatg ttgcaattaa gcaaaagagg cagactgtac
1511401 ataataatga atctgggagg aattgctcca catacaagag tgggattgga tcgttgcaat
1511461 caggagatgg aaatgaaatc aaatgagaac tggacattga agcctcggcc ctcccacaaa
1511521 agttaatgaa cagccaagaa gaaaaaaaaa agctctgcag cgggcaggta cagtcaacag

1511581 atgccctcag aataagcaga ggtgtgtgcg agctctcctt ggaccaacac tggctccaac
1511641 gaggtgggta aaacaaagca gggccgttca cattcacaat ggagacactg tcggtgagag
1511701 caggggagga gagcccagaa caaacagcac acaagagttt cacaaacaac ctgtcataag
1511761 gcattcagca ggacaggttt gactgggaca cccggcccac accctgggcc aagggtgagg
1511821 gataggtcat cccacctcag cagaatgaca cgtggagagg gggggggtt aaaggtgtgt
1511881 cccggggttt cgcttggatt aattatcatg ttacattgca ttgttttctt ttataaacaa
1511941 cttccactgt taacctctca gttcatttct tgcccgagtg gtcagttctt tatctggtgg
1512001 atgaaacatc atgtgacaga cagagtccac ttagtccaga gtataatcaa ttgccaaaat
1512061 caccaattcc tatcgtccgc tggaaaatca actttcagaa aagtgataaa caccagtgag
1512121 tgagatataa ttgcttcctt acaggaggag cacatttttt gaaaacgctc atctcatcaa
1512181 taattttatc gacacatcca ttattgttat ttggattctg tcattgattt caactttgag
1512241 ctgatgaacc tgtgtgcttt gacagattgt ggattcctgt attcactttg gtcctcctga
1512301 ggctaagata agccctaaac attgcaatag aaaaccagta attggtccct ggaccaatga
1512361 ccactgagta gggagtcgcc tttgattatt ttttttagga tttgtcctag agaacttggc
1512421 tgtccttaaa gctaagagga agtgttagct cagcggcatg atgtagcact tttcctctcc
1512481 cagttttttt ttaaagaaca caattagctt ggcgtgtttc taaaatggag tcctctaaga
1512541 aatcatcaag cgttaattct tcttgagagt aatggctatg aggatgatta cgctgtataa
1512601 tgaggaacta agattagagt ttctaattat tttccctgat tattgcattt cttgttgcct
1512661 ccttaccccg ttgcctatac ccttttgctg gctgcgtttc tccaggagaa gatcaacata
1512721 cgcagaactc tgttgttgcc agatcaaatc cgttgtgcct gtcatgctaa aagtctgaag
1512781 tttggtctca aactactgcc gctattcaca ggtgattttt ccaatattca tttgacaatc
1512841 atgttaaatt gttctcataa agcctcactt atcaacatca ccgggaatac caccggggct
1512901 tttgtggaat aaggataacc agctgttaga ctgtaaaaag atcatttttt tgtgtacgtg
1512961 tgggatttga cagatggcgt aagtgttgct ttctacttta caagtgtact ctttggtgtt
1513021 ctctcttttg taaaccttta tttcaaagta tctgtggcta tattcacctg actgctcgag
1513081 gctgcagcaa cagctctgac taaacatcag tacacgtata gtatattgaa tatttcatca
1513141 aattcatcag agctcagcgt catctctcat aatgatgtca cgattctgac gcagggcaac

1513201 atgagtccaa cgggagtcaa atctggatca agcccatcat tttagaaggg cgggacggtc
1513261 ctggaaacaa ccgtacagta gaggacaaat gatagcaaag tcagaatgag acataaagga
1513321 ttgacggaag cataaaggct taaaatcagt ggggaattgc tgaggatcag tgtttataat
1513381 ataaatataa tataataata ttgtatatac aactgtaaaa tacttagaaa aagaacagat
1513441 taagggaagc gtcactcatt agagtagtgc tagtgtcact cgtaccaaac cagtttcatt
1513501 aagaaaatca cgcctactaa gtttgtccag tcctactttt ccctctcaca caaagggaca
1513561 cttgatcctt cattacaccc tgaaccgagt caccatgtgt ttttgctgaa tttctgagac
1513621 aggccatttt tctacagcaa tttacactcc tacaactata gtgttctggt gcagatatgg
1513681 tgtcagcccc tcctcagtga cctctgccct ttgtgaagcg agcaaaagtg tgtttcctga
1513741 ttaacgactg gctgaggtac acggctccac cgccggcacc ttgtcctctc aaaccgaggt
1513801 gacagtctcc tcggctcttt atggaaatta actgcaaaac agagccaatt agacctgcgt
1513861 tatttagagc gactcagctt ttcaccctga attaaaggag gcctgcataa aacctgtacg
1513921 cgcttgcagc aaattaatgt gagtttggtg gcctgccagc acgccagtcc atctaaaatt
1513981 aggacaataa agcacatagc ctgcgaatta tggaggtgat taaagctcta catctgtgag
1514041 cacatttaga cacttttaat tataagtttg gaattacggg aaatggggga agtgacggcg
1514101 atgctttgtt tttgtaaagt ttgcaaaaag ttgaacagaa ttcagtcatt tactctgggc
1514161 ttctgctttt tttttctttt tttttttttc aggttacaac ttagttcgag gacaaaaatt
1514221 ttcccaggtc ctgcagcttt tattccaaat gtgtaacagg gaattaagaa tttgtataat
1514281 ttcttttcac tccgccggct aaagataggg tgcttagcct tatacactgt cttaattttc
1514341 cctttcttag gtcactgcag agagcgaagg cattagagtg aggaggaggg tccatactct
1514401 atgtgcaaga atacactgtg gaagttgcaa agggcagagt ctctaggaaa tccactcttt
1514461 ctttgaaaaa aatgtcatgg gagagatagg agattggcac cacaaagacc ccaaaatagc
1514521 atgggctgct acgcgttgtc agacaagatt tgggctgaga cattgacttt tgttctgcaa
1514581 agtattggcc cctgaatggg agatgggagg tgtaaaaatt gtgatttttc cttttccctg
1514641 ggttgcatgc aatgcattac ccttccctta atgcacgttg caaggatggt ggcttgtaat
1514701 gtcacaatta tttaaacttt ataaaggata ctttggattg gcacagattt gagtaaaatg
1514761 gatgaaaaaa aaaatcattc acttatggtg ggtagatcta aaaacctgct ctcccataaa

1514821 cccaccatta ccaatgtctt cctctgacct tccccatgac aacaaaggct tagtatgttt
1514881 ttgcccettt ggctcatata ataggattat gaataatttc tacaaattta caaaaacaaa
1514941 tcacatggca tggcttaaag tttgctaata atttctcccc agttgataga tcaaagcaga
1515001 gcatgaggac tgcagagcca ggcagtagat tgaccctggt caggcatttt taatgaaagg
1515061 gttaatctcc tcagaggtga ctattcagga gcaagcagtg tgaccaaatg aagaattcag
1515121 gtctggggta agatggagaa tagtagtctt gagggtgctg ttgggtgaca gccttgaggt
1515181 agaactgcgc gatgctgcgt ttgaacccac cagccccatc ggtccaaaat ccaccgcaaa
1515241 aagaagaaaa aaggttaaga aacaaaagtc agagggatta gaaagaaaac agttttactg
1515301 ctgtttcacc tgtaaccaca aacaggccct gctagctctc tctctcacac acacacgcat
1515361 gcacgcacac acacacgcac acacacgcac acagacacac acacacacac acacacacac
1515421 acatacattt gtacttctat ctttgtaagg ccttccccag cctttaaccc tatccatgac
1515481 catcacaact aatgctatgt ttccactgaa ggtatctcct ctcctctcct ctcctctcct
1515541 ctcctctcct ctcctctcct ctcctctcct ctcctctact acatagacac attgcagatg
1515601 ctgaatggtt cagggtaaca taacagcagc acttctgagc atgtttatgc tgaggtctct
1515661 gaatgaactc caaaactttc ccaaaccctt ttccaggcct tgtctttagc caatgccact
1515721 gtgcaaaaac ttcaaagaaa gggagagttt aagttagaat ctggatgggt ggataagcga
1515781 aggtcaaaag atttaaagat caatccaaca cagagaaaac caacatccag aggctcatag
1515841 aggactgctg tttgttctgc actcttattt atttagttca tccatgataa aagcttccca
1515901 aaagtgtcct cactttagta tgaaatatat ctggaactca ctttgtacat ttacaagagc
1515961 aagtatacat ccatgaactt ctatctttgt gaggacattc atagtcataa tccatcccca
1516021 tcctgttgac ctaactcgga ccttgactta aacccagttc taaactttac cttataaccc
1516081 aaggtaaccc tggaatagac cactgaatca gtgaggtcct cactttgcta agtaaatgtg
1516141 tattttggtc ctcaatactt agcacacaca agtatgtaca cacacacaca cacacacaca
1516201 cacacacaca cacacacaca cacacacaca cacacacaca cacacacaca cacacagaca
1516261 cacatacaca cacacacaca cacacacaca cacagaatag tctttctcat tagaacaact
1516321 gctgcccctc tcaaaaagtc cccagctctc tggagccctg aatgtttggg ataaacttag
1516381 ttatcaagca cctcttgtgc aactgtaagc acacatgccc tcttgtgagt aattaggcct

1516441 gaattacttt caaaagaaga gagggcttaa aatgctttta tatagatgta taagccttgg
1516501 ctggttaaag aggtttagga atctcatcat gacagtggca aaatgggact cttggtaatt
1516561 aggagcaaat gaaattgctt atctgttatg gagttagtgt gccagctatt tggttgtgtt
1516621 ggaaaggatt aatcacgaag ctatcgacgt tacattgtcc cacaataatc attcatatgt
1516681 tttatgctaa ttgggcattt ttttccaggg tggaattaag cagcaattcc agacaatgaa
1516741 tacacgcaaa gaaaaacatt aatcacggcc ctaaaataac aacgatattt cacctgctgt
1516801 gatcctgcaa aagtactgaa tgaaacaatg aggatatcag tctgctcttt gagctaaact
1516861 gaaatgccac agtaataaat catcggggta aatgataatc attaaaacct acgagattca
1516921 gtgattaaaa taacggctgg atttatacag cacaatataa atgtnnnnnn nnnnnnnnnn
1516981 nnnnnnnnnn nnnnnnnnnn nnnnnnnnnn nnnnnnnnnn nnnnnnnnnn nnnnnnnnnn
1517041 nnnnnnnnnn nnnnnnnnnn nnnnnnnnnn nnnnnnnnnn nnnnnnnnnn nnnnnnnnnn
1517101 nnnnnnnnnn nnnnnnnnnn nnnnnnnnnn nnnnnnnnnn nnnnnnnnnn nnnnnnnnnn
1517161 nnnnnnnnnn nnnnnnnnnn nnnnnnnnnn nnnnnnnnnn nnnnnnnnnn nnnnnnnnnn
1517221 nnnnnnnnnn nnnnnnnnnn nnnnnnnnnn nnnnnnnnnn nnnnnnnnnn nnnnnnnnnn
1517281 nnnnnnnnnn nnnnnnnnnn nnnnnnnnnn nnnnnnnnnn nnnnnnnnnn nnnnnnnnnn
1517341 nnnnnnnnnn nnnnnnnnnn nnnnnnnnnn nnnnnnnnnn nnnnnnnnnn nnnnnnnnnn
1517401 nnnnnnnnnn nnnnnnnnnn nnnnnnnnnn nnnnnnnnnn nnnnnnnnnn nnnnnnnnnn
1517461 nnnnnngtgt taggcaaagt ccagcaaggt gtaattgcgt gtaatatatt tacagaggat
1517521 tgatttccct tctgcgaccc acccaccagg cagcgacgag ctggacccag gttactgtgg
1517581 acgaatgtct gctctgttgc ctttacatca cctaacaagc ccagaatgca caaagtgttg
1517641 aagtaatgag ctgagcagag aaagtagtga gttttccggg aggctaatac ctgtgacctc
1517701 ctggatcctg gattcctgtt cctgtcgcag gtctctggag acttttacag aaaatgcaaa
1517761 tgtccccctc ttggcaagat accattatta cacctctacc tctcacattt ctttgttttt
1517821 gctttaatta cttttcccac gtagccatga cccccccccc ccccccaact cttactctct
1517881 gaagagaaat aaaccttctt atacttaaac cacttcacct gttttcccag agaatgaagt
1517941 tctgggaaat tgacaagggc agagagaaac tacaaggagg gaaaacggat gaagaacaca
1518001 aagcaggttg atatttttcc cctctcctat cccttttctt ttctttttat caggttatgt

1518061 gactggaggc ttcattaatc accacctcga cagcctctgc cagagggaac taactgcatt
1518121 tggcccaatt aggcgctgta gagacaatag gaacggcaca agtcgcgcct ctatgccagg
1518181 agctaagcat cgcctccctg tcattaccta cagaaaggga aaaattaacc cctaccagca
1518241 tccctgcttt cctccattga tctgaagctt ttgtgcagtc cctggagcgt ggcggacaat
1518301 tacagatatt attacatctg cattgatctt ttttaaagag agcagagtgt ctccgagcaa
1518361 ttaaaaaaaa ttatggttcg gaagtgggcc aattaaaatg cctccagctc tggaggggga
1518421 ctgttggaga gaacacggga ggagagcgac ctgtcagaat gttgcagttt tttttaaaga
1518481 cagttatacc taaattcatg cacatgcatt atatttgaca gtaaacatcg gtgtgcagag
1518541 cctgtgagga ctacattagc atttagtggt gcattttgtt gggttcatat tgtcttattc
1518601 ttcatgctaa ctttagcatt tggttggtgc aaaatgtaca aaaattattg agtgttagtg
1518661 atcttttttt aaaaggaaaa acaatcctgg ctgtgtgtgt ctgactgccc gggaccaccc
1518721 ctgagcgtgt ttatgctggg gtctgtgaat gaactgcaaa actattccaa acccttttcc
1518781 aggccttgtc tttggccagt gccaccttgc aaaaagtaag ttagaatcat gatggtcgga
1518841 taagtgaagg tcaaaggatt taaagatcaa tatgatgcac agaaacccaa catccagagg
1518901 ctccttaagg tctgctgttt gttctgcact tttatttctg ggttcatccc tcaaaaaagc
1518961 tcacagtcac cttcttgaga tttctttggg agcaggtggg gtctgggatt atccggacca
1519021 gcttgtgact aagaaggtaa acaatacctc caaataagac gtctcgtaac ccagaaaaaa
1519081 acaacgggtg cagtttaagt aaacagtgtt cggcagaagg acaaaggaca atggaattct
1519141 gtctctgagc ggcagtcgtt ttatccacta actctttgca tgtgtttgaa gtggaaaatc
1519201 acagtttctt taatccgtgg agcattcgaa taagtttatt tccttccagg attaaaaaca
1519261 gtaacaatag caggcagcta caaacaccca gcagaaacaa atggcgactc caccgaggcg
1519321 ctcggctccg ttaagctctc catccaccgt gctctttgtt aatttgcaga aatgcgttat
1519381 tattgtttat ctgttctgcg cgccgataaa gcgcgccgtg cttcctctgt tggattacag
1519441 agttaaaagg tttcaggtgt aatccaccaa aattattttc cggcaacagc agcctcccgg
1519501 agatgggatg aaaaggagtt ttcgacgctc gaacatcgca acacgtttga ttaaagaggg
1519561 ctaatttcag aatatcatca aagcagaaga gagcttagag cgcgccttta taagcactct
1519621 aatagccttc taatatagga accaagggct ctaatttaaa acaaatgaca accatgcata

1519681 ctgtttgggt atacatcgcg ttagatgcac tttggggttt gtggtgcccc aatctggtgc
1519741 ttttctaagt ttcttatggg aagttgcaga ggtttaggag taaaactgag ctggatctgg
1519801 tctaagattg tgatttgttc atattctgct gatcattggg gcactttgaa gtctcatgtc
1519861 tacaatcttt ctcccttttt ggggagggaa ccaaaatatc aagaggaaaa aatattaatt
1519921 tgggttaaaa ttgcaaaggc caatgtagga gagttcacct gcaaatttgc ctggaattgt
1519981 tgcagtggga cctttttgcc aaaatgccct cccaaaaact attggttaca cttctggtgt
1520041 tggacatttt ctgtggagga aatgagttta aaggggtcta actaagaagg catgacatat
1520101 atcagtttta ttcagatgat aagctcttat gaggcagcta cgattcacgt ttctacctct
1520161 gagaaagccc ccactgcctg aatgctccct aaaaaaaaca tgaacattta aatgaaatta
1520221 tggaacctgc ttctcctgac agtcagggtg atgacacgct ttcagatccc atttgaaagg
1520281 gtcttaatgt ccccgaaatg taaatgacag gacgcatgga ggcccaaatg ggaagcagga
1520341 ttctacatgg gtccgagaca ggaagactat agaaggaaga taaacgaagg gaagagggag
1520401 gtggagattc tcatgcggat cgtttcacgt cggacacacg gtgccacttg cattatgctg
1520461 actgttgcgt caaggagcaa catgaagggg cccttttttc caatgcaccc cagggttttt
1520521 gctcttctct gaaaaaagga agtattcaga tttaagaggg cttcttaccc acatgattaa
1520581 aaaataatat agagaatgtc ttaatactaa tatttcaaaa tgctttaaat aatttcataa
1520641 tcacccgaga atcaacagac aaattcaaat atatatcata ttaaggatta ttaataaagt
1520701 ttggtggaga acaagtatct aatttaatcc ccagcgctca tgttagcatt ttactgcctg
1520761 tcactgtcat tatatctccc cgtgctgttc attcatggtg cctgacatgc tttactctat
1520821 cagcctgctg aaatgcacac cctctgctcc ctggaagcac gaggcagcct tttaatagca
1520881 gccatgcctc cttgttagcc agggaaaaaa aaggtcatgc aatcggattt ccgtgaaccc
1520941 cccccaaaaa agaaaaattc cccagtgatg taccgcagac atgtcaggca cctcacgttc
1521001 atagctcaac tttcttttct tctctttttt gtttctattt gttaatttat caaccatggc
1521061 caggaacagt tattctatgg agtctgaaat gcacggaggt tgaaattgtc catgtgcaga
1521121 cacccgcgtg gccaccaagc ccatttttgt ctatttatat atctttgtca ctttgtgatg
1521181 tgggaattca aaagcctgcc aattaaaaag aaatattcct ttcatgttta tttttgacat
1521241 tgaattcaaa tacagttctg tagatctcct gcaaattgat gtttgtcttg tattaatatg

1521301 ggcttgtatt cgtggtgtat gtattgttct tcacaaacaa taaacaagga tttgttctgt
1521361 tgtcttcata gatcaaagcc ttgtctcttc tataacataa agattaaaaa aagaactccg
1521421 tcctccctga ctcaccgctc tgcctggaaa caggcaggta ctctggaagt ttcccctgca
1521481 gccattaagt gccaacttgt gtcagaaatg acatgcatct cctgctccca tgtcatgcca
1521541 tgcatgtgat gtgatgggcg agtcaaggca cttcctgcag agaagagtag ggggtcatta
1521601 ttaaggttct taaattagcc cccatctcac acaacccttg taatttcccc aggattgcaa
1521661 ctgcacacac atccaccaag ctgcaagtat tagactcaac aagaggaatc attatttttg
1521721 aagtttttcc tggagcaaac tcatttcaaa tgtgatcaaa ttcgacctgt atttaaagtt
1521781 aaagtcacac ttggctgcat tttgagccca tttgagcaga accatgctgt tagtgctcta
1521841 taggctgggc caattgtatg tgcttataca aagcaattta catgtctctc catcacactc
1521901 acttatatgg ttcaactcac aggagctacc atttaggtca ctttaagagg gtgggggaag
1521961 gcgtaatacc acgcatgtag tgcagtggta tagaatgagt ggcaagagca gcaaaagctt
1522021 aacctctgct ccctcaactc catcggtgca acaccgacga caccgttacg atcccgaccc
1522081 gacccgaccc tcacaagtag gtctgaccgc tttagcgtct ctatggccgc cagattaaag
1522141 agacccccgc ctcagggtcg ctgttcgaag gaaacgattg catgttgaaa tgcaaccctg
1522201 cccctctccc cccacttttg ccactcattg tctctctttc attccacctc tctcactaca
1522261 tcctttccac tcttttcccc aatctgcact tccccgtcct ccctctcttc ctctcctgct
1522321 cctcgcgtat gtatatttac aagagtgcac ataactggtg tctggggaga agtaaggagc
1522381 agcggggaag ggaagccatt aggggaataa agagaggtgc tgcctcacag ccgagcttca
1522441 caggccagtg gagcaaatca aattgggtct tgttctgtgg cagaacacac acgtatgtaa
1522501 ccccctgcac acacacacac acacacacac acacacacac acacacgcac acccatggtg
1522561 acagtattgg agctacatac tgtagcggac aggcgggccg ttgggacaga gcagggctgg
1522621 gcatgacaag agcagagccg caaagggaca aattcatgac ccctcaactc tgcagtccaa
1522681 acgactctaa cagttagttc atgattgctt cttttcctat caaatgagta ttttcgtcaa
1522741 gagtataaat gctgtttaca gggtagattt ctgaacaagg gtaaacaaat aagccttaaa
1522801 caatacctta ttacttctta aatacatgtc tcatcgtgac agcctaatat ccatctgcca
1522861 tctctgcact aatacctcat caataactgc tcaaactctc tattccataa tgttatgaat

1522921 gaaatttaag ttatcagtgg gctcataaaa aagttgaaga atcggcagaa gactccaggt
1522981 aaagggcact agcgctaatg aaaaatgaga gggggagaca ttcaaggggc ttcactttta
1523041 ttagaaatag atggtaatga tgccaaaacc ttagtcaaca taatgtcccc tgccatttat
1523101 atgatatcaa tgcctacggg gtgctattca ttcacagcgg aataaaggga gatgggtaaa
1523161 atatacacgc gctgcttacg cttctttcag gtcctccgaa ggattgctac cacaaatcac
1523221 tttggctcaa tttattaggt gcctgcagag agggtgcaga tgtctgatgg atggctgtct
1523281 gcaaatggac tcatcggtta caggaaaatg cacaggcttt aattaaagag gactggacga
1523341 gcatattggg gtgtctcccc acatctgtac agacagggtc gtgcatcagg aggctctttc
1523401 ataaaccagt gcatgctcct cctggagagc tggtagcatt agcatagcgt cttttgatga
1523461 aaaaaaggaa acacgaacaa gtcgggtgtc gttatgaaaa acccactcat catggtgaaa
1523521 gggttcaaat gctttggtga tgttaagagt gtagtgtctt gaccctggaa ccatcgccca
1523581 tggcaacgcc atgaagggga aggcccaggt gaagtgtggt tcaagaagaa cccagtgaag
1523641 aggaaaatca aagatcagtg taccctgtct gaggcccatc ctgaagagaa ggccctttgt
1523701 ggagcaccta ctgcctggac ctcctcaggt gctggctaat ggaccccttc ttcataaggt
1523761 ccatgctcca gcagcacctg tgtagagctg gaacacaatt aaaagtacat gttttaaaac
1523821 atcaacacag ttatttccaa gcctgtacac acacacacac acacacacac acacacacac
1523881 acacacacac acacacacac acacacacac atgaatagtt ctacctttga gaggacacta
1523941 aaaatagcac ccagcctttc ctccaaacct caaccaccac agctaaaacc ttccacctaa
1524001 cctaatcaaa aatgtaacca taaaccatgt cttgcatttt tccctcctcc aaaaaaaatgc
1524061 cctcacttta cacatagatg aaagttcatg gcgtatctgg ggtttatttt ttgtattttt
1524121 ccccccctta aatatagagc gatcctgcaa acaaccatgt tttggtttct ttcagcacta
1524181 gttcatgtgt gtgacttttt cttttccccc ctctaaaact acctctttac ttgctacttt
1524241 tgtttaaagg ccccgttaat gaagagctct tgagctaaac acatctctat tttacgatta
1524301 gcagtggtgc ttgaagtaag cactggtgtg aaaggaggct aagacccttc agcatccaag
1524361 ccttttactg gcccgtctgc catcaatcag gccttgtgtc cggtgagtgc cgccaacatt
1524421 tatgaaatat tcatcagcca ttcacttttg ctttccactt accctaatca ttgcatttag
1524481 gcagaattgt gtcacctctc ctcttgaaaa aaaaaaaaga aagaaaaaat cccaaaaaac

1524541 ccgacaaaaa gcctacaatc atctcggtga gcggaggcga cttgaggagc tgctccttgt
1524601 tggtaggccg gttctgtcct ggtataaaaa tccattatga catccataaa ctgggccatc
1524661 tgctgggact tttctatatc tttcatcagt tttttaatga cccatgaatg caaattcatg
1524721 tctgattttg ttgttaacct ctgtctctct ttctttctat ctctatagct tagctaccta
1524781 cggctgtgat taatgtcatt ataatacaat gagtattata ttcccatcag tcatgttcaa
1524841 gcagtccgag accctgctga tgaaaggtta ttatatttat catcgtatgc tgactgcact
1524901 caaacacgtc tcagttcaca atagccaagg agggggaaaa aaatccactt tattctttac
1524961 tcatttgcaa ttcttatagc attgagaaac ttaatctatt cccaggaata actcattgga
1525021 tgttacagct taagcgaaga tgtcctcagt tgctcatctt gcattttaaa cagggcgcaa
1525081 agagccttgg tagaattttc caaacacaac tgagctaaca ctgatttatt gctccggttt
1525141 atataactcg tattaggaat tcatggacct ggcaaaaggg attagagcgt ttatttcagg
1525201 cagggagatg atcctgtcat taatggtcga tactttcatc atcgtttaaa ccagaggaat
1525261 ttgcccggtg acgaatcgtg gcggacatag ctatcagagt tgtgcagctg tactcgttat
1525321 ctgccgacga cccgctctgc tcggcggacg tggacccgcg cccatcaaac ttggattagg
1525381 ctgtcaatca agcgtcggcc tcgtatattg tgttacaatt tcttctaaat gtcaccaaga
1525441 tcagagtaat ttctcggcta attgcatttc gtctcccttt aatgctgcac gtttaatggt
1525501 tttttctttt acatacgtga caagatggtg tttctctcac agtggcacgg tgacgagcgt
1525561 aaggagggcc gcgcttttgt ttggagagga aaatgaaaga ataagtgact gctgaggttc
1525621 agagggcgga tattctgaaa tggcagcaaa taaagagagg aaactgagaa atgatcccaa
1525681 tcttgcaata gcgtgacatc aattactcca aaattcttat tagcagaggg ttactctgta
1525741 atctgcgggc tttgcagggc tcgttttgtt ctagaaatgg tggcacgttc tgcaaaagat
1525801 cggtggcaga agtaatattg aggattttct cgcctctgtc acctcctttt caaaaacatg
1525861 tcactctgtt taaacaggac aaatacaatt ccgagcgtct cacaatgtcc cggtgcctct
1525921 ccacagctaa taatagtagc cacaatagac tcattaatta cctcctcgca gaaacctgtg
1525981 aggtgaaata ctattaagaa tattaaacac actgaaacac caatttcact gcatgtcagc
1526041 agcaggatta cagtccatca ttagactgga gggaagcaaa caaataaaac tgcatttctg
1526101 gagttaaaaa ggccaaatgt atatgtagcg taaggcaaaa tggaaaagaa acattacttt

1526161 tagtgcattt taacacagac acaaggtgaa aatctcaaaa aacaaccatg agtggcactg
1526221 aaaactaaag ctcaagactc gttgctaaca aacagttaac tgctttccat gagcacactt
1526281 aaccttctct acatgttcag tcatcccact tttcagtccc tgtggctaac tgtgtattgc
1526341 taattcaaaa atgagacaac gtttggagcg gccctcaatc tccccagtga ctaaatgccg
1526401 caattaactt ttccgccttc ccccccaaca aaaataagga gtttttaatg actgttacag
1526461 gcaatatcat cgtcttgcaa agtctacgct agtttttttc aatgtttctg tggcacttaa
1526521 tatcacagag taagaggtta tgagattatt caagggcaag ggaaaaataa tctaatagtc
1526581 atcagatgtt gtcagcagaa atgaatatgc aactgttaag attttattta gatagcagga
1526641 aacagttgtt gctaactcaa gttagcatga gttgaattag aatgttcggg tttatgggca
1526701 ccactgttag acttttattg tggtagaact tgatcatatt ttaaagtcca gtctgggttg
1526761 atcttctccc aaattacccc tcttgtcagt gtgtgagtga atggtgtttg ccctgcgatg
1526821 ggcaggtgaa gtatattctg gtctctagtg aacacaagat gggccctagc aacccctcat
1526881 gaccctgatg aggaataaat gattatctat cttctctgtc atcaaactat gtgtgtcaac
1526941 ttctctgaga gcttaagcgg cacaaattat attctgaaat taacttaaag ggcccacaaa
1527001 gtgattgatg atgtgatcag gattcgtttc cataaaaaaa gagaatgaat cactgcttgc
1527061 agtgcaactg ctgacatcac atttgtaaat aataaccgga tgtcaggatt aaacccgatt
1527121 tacgctgtaa tgatgtcatt aacatgtgca cagtgtgtga gagtatgtgc gtgtgtgtgc
1527181 ctgcgtgtgt gtgtgtgagt cattacaaga ggagctggtt gcatccataa gcaaaatagc
1527241 tgcataactg gtgatgtcca tgacactgtg ctctctgtag ctgctgtccc cccccccgca
1527301 tgttagccag aatctaatta ctggggtttc agcagggagg taaagcaggc gctgaggaga
1527361 cgaagcctgc aaagcctgat tctgtcaact gacgggttgt tctgtcggag acacaggcag
1527421 gcagcctgga atccctgggg tgcgtttggg agagacccag ggactggtca tatcaacaaa
1527481 ttttaatcaa aagcctgaaa ttgtcagctg tcatacactt ttttgtgatc tctgatacct
1527541 tacccctctt ccgacctgat aatagcaaca cacatgcaca tgcacaacgt taacggacac
1527601 ataataacac ctacgtacac acaaagaata acgagatggc atggatgcta acttgcgcac
1527661 atgcaaacag gaaagtaaca acctagaata tcagggaatg cagctacatg tgtttttatg
1527721 gcgtcattaa ggctaaaaat caaaagaatt gtccgtgtaa ctgaaagaaa tgccatttgt

1527781 taacgtccaa aggccttttc ccaacaaagg ttccctgagt aatccaggct gtacttaata
1527841 aacaggccta acgtgtccac gttccgccgt gcatcagctt aacagctgca cattttggtt
1527901 tgctagctaa ggtcgggggc aaacaatgcc attttatttt gcccccaagt ccccttttcaa
1527961 ccttgtgctt cagttcagga gcggatggaa aagctgtggc agctttgatc gccgaactgt
1528021 tactaagaag ctttgagggg ctttaaagga cctaaaatgt ataaaatgga cggatcaaga
1528081 gcaggctgaa atttgtcggt tcaggggcag gttaaaaaag taagagcatt cccatacaat
1528141 atatatccag ttagtgcata ttaggggacg gcagatgaca aaagtggaat ctgagagccg
1528201 attagcattg tagttaaacc ttctcatgag cggggggcaa aaacagctcc atcctccgtg
1528261 tttctgactt taaagggcag atttttttta tttaaatgta cacaaaagaa aaccagaaca
1528321 ataatgaaaa aagggttgag tccactgtga aagcagctct gaggaagaag caaataaggg
1528381 taacatagag aaatgtgtcg tagcttagca acatcgctaa cactgcaact gagaaacagt
1528441 tcaaagttct gtaagcgcct ggcaatatgg tttattcaat ccaaaacatt catgattaat
1528501 taatgctttt gtaatttatt gattggagag ctgcagaaac acatttctct tctgttaata
1528561 aggacacatg aaagaaggcc ctgccctcga taatgaaaca ggctcggtct gctaatgata
1528621 actgcaattt attccactgc tacccgggct tacatgggcc cgtgtgccac catcaacctc
1528681 tctcactttg gtaattaagc gtgttgatac tgcaggatgc ttacctgcaa ttacgccatt
1528741 ttaatgagca gcagcggatg gaaaaggggc aaggcgaggt cagccagaag cagcaaagca
1528801 ccggtgtttt taatagatac ttaaatggtg ttaatttagc tcccagtaat ggagaagacc
1528861 tctctgagga tataaccaca gttgtgcgct tggtgagaag gggtttttga gtacaaagag
1528921 gaaatcctta aactcttcgg aaacttttat tactcccctg aggaggttct gcttcttttc
1528981 agctgctttt tgaggatctg ttttcgaccc tccaggcgtt tgacgggttt tagcaatagt
1529041 ttggtgggac tgtgtcggtg tcacgctgag agttggagtc cgcttccctc cgtccagcgt
1529101 atgggcatca tgggcagcct ctatatgcag atgtttgaag gagatctata ataggtttcc
1529161 acgcttggct ctgagggtcg tgcatcacct gcaagaacac ggaaaacacg ccggagagct
1529221 acagcgcgca ggttattata actcctgcgc cggctcctgc gaaggtggtg tgataacagt
1529281 cattagggga gccgtgggag gaaacggagt cttattaaaa gcgcgtggag attcattgga
1529341 aaaacaagca ctttcattaa caggtgtgaa gctatgcaaa tacataaaca caccacggta

1529401 gcccacctac ctcacacctg caggtggaaa tggctgctta ttaacacaac aagcctgcaa
1529461 ttagttctga agaatcagat tcaattaaac tggacaagaa cggatgcgta aaagccttga
1529521 tgcacaaaag agtgatactt acttgattta gtgaagtgta taatgatttt atataggaag
1529581 gaatgtttta gataaactat ataaaacaca tgatactcat ctgaaatgtt cccttgagac
1529641 catctgccca gtgtgtgata tctatggttg tgcttttata agaaaattaa atataataat
1529701 tacttttaac atgtcgccta ctgatccctg tctttttgct cacagaaata agatgaatct
1529761 tgaggtgagc gttcctccaa gggtagtacg acatatactg ctgtgtttct acagtagcct
1529821 aaaatggaca aacttcatgg aatttgtttg cattaatcta ttatcattgc taaaatgaac
1529881 gctaaatgtt agttttagaa tattgctaca ccatatccac accatctgca tttcataaag
1529941 aattaacatt taacaaaaca aactaaatga aagtatgggt aataaacaga ggttttcacc
1530001 ttttcgtctt tgttcagctc atgtttgttc atttcctccc tctctttttt acagtaaggc
1530061 tggaaaatcg tgatcagaag atgatttgct acctgacaca tttaatgtat aacatgcttc
1530121 cagtgagggc tcttgaatgg atggattttt gaaaggtgcc agagtggctc cctgctgagc
1530181 ggtaagctcg ctctttgatg tatcgcgctg taatgtggtt gttatccata atcggctcga
1530241 attcaggagg gccgtggcta aaaagagagg tgcgacggta acacgttctg gtgggacatt
1530301 aaaatgcgct cgtgctcctc cacccttcgc ccgctccacg gtaaccaaaa ctaaacgctt
1530361 tattgcataa gcacctgcaa atatgaattc tttagggaag caaaaaagca ctaatgtcca
1530421 cagccgctga caggataatg taaaatatca gtaacagaac cttgtatatt attacatcct
1530481 gtgaactcat aagatggaga ttaaattgtt tcctgatgct tatgctaatg tccccggttt
1530541 gtccaatgcg ataggtttaa gcaagaaata atcccaaagc catcacatta agctgcgatc
1530601 tgaaaggtgg gacgcagcca cagagtttgt gatatgaatc ggtttggaat ggaatatggt
1530661 gatgatagct ggaagaacaa tatacagacg agggagcgaa gaagaaggtc tgaaatgtga
1530721 gtctttgttt ttgcattaaa cgctgccagt gtaacggggt tttaatgctg caccgggctg
1530781 gaaaacgcag tgattggatc acaggaatta atgtaataat gccgcaaaat cccatgcttt
1530841 cacctgcagt cgtaaacctc ttctatagtt tgttgtcatc catagatttc agcggtcatc
1530901 tgtatgataa acgcagcagt gcgggtgttt taatggtgct gttattgctc ctgaagccat
1530961 ctgatagcct ggctagcaaa aaataaatag agttgagcac ccgggttgtg attgttgctc

1531021 cacagatatg tgatattacg accttcttac gatgcgctct gggttttaat
ttagcagtga
1531081 taatttgctt atgcaggcct ccgctgatta gaactctttg ttgcaccaac
tgttttttaa
1531141 tatagtgtga attacccact gcgcttgttt atgccaccca aacagggcgg
aaaatcagct
1531201 accagcctgt ttgaaattaa cagcggcgga ggcagaaagg gagcaggaga
caggcagcca
1531261 gacagaaggg aagggaaggg atgagatggt gggagtggtc gctgggggac
gagtgtttgt
1531321 aaagaagcgt aatcaaacct cgggaaaaaa gaccaaaata taatattgaa
tggcggtgac
1531381 tccctgacat tagcaaagct ataatagtta gttcggaggg gccgtcctta
ttgaaaccca
1531441 ccggagtgaa tggaagtgtt ccagttatgc atcattatgg ggggagagct
gtccacttcc
1531501 tgctgggaaa agatgccctt tgctttcaca ctaaaaggag tagttcacaa
aataacagca
1531561 tccccggcgt ctgcccccca cacccccacc cctacctccc cctccctttg
acagataggg
1531621 ctcaaatgaa gagccctcag aaaaggatat gcctccattt aaacttaaaa
gcgggcgcgt
1531681 aattcggggc ttacaacgcg cagtgctcat aatggcggtt aaaatggcag
atgagggaag
1531741 gcgacaacag ttcgttgaaa tgcacaatta cgacggcaaa aacatgcaat
ttggtttttta
1531801 ttttatttta gcaaaagcag cagagagaga gagaaagcat gaaaacgcgt
aagattttac
1531861 tcgcttgcat gcacgcacat gcctccccat atcatcagcc tgcattgtga
agagctcctt
1531921 ctttttttcca aggtcttatc cacttcaggg gttcaacaga ttagcgatcc
gctggggtgt
1531981 gatggctttt cagtgccgca ctttacgtcc aatttgaagt gtctaagagc
atgcttgtca
1532041 ttagtgttta ctcaagactg ttttttcttt ctccaggaga tcccagacag
actgtgacat
1532101 taaagacaaa taaaagtgca tcagccccgt gttgatcttt ttcgaaggct
ctcggagaaa
1532161 caggaccaac attttatctt ggcaggcagc agcttcagag gttatggata
ggccagcccg
1532221 cgacgccatt atttaccaat ggagcagagt gtcagccctc ctctctctgc
tctttctctt
1532281 tggattgttt gctccctgtc tgtttgaaag gggcacttt atggaggtga
acggcgagag
1532341 aaaatatttc tgcccggcca tcagcggatc aatggagatg tgtttacctg
cctatgggca
1532401 caatggtcac tccatataat ggatatttaa cagcttgttg accgatcgct
tctcgtcgat
1532461 cccttaaact tgttgaccag tggtgggaca cgctaccaat gtggggtcac
gccatgtatc
1532521 tacagagact caatggcccc ctttgtcctg cactggtcca aacatagttt
gtcaagtcca
1532581 ttttgtttgt tttgttagta ggtcatttta aacccccacg tccaaaaagt
ccagccggag

1532641 gggtttgtag gagatcaaaa ctgaatctga gcataaattc tttagtgaac taaggaactg
1532701 gaacctcagt attataaaag ccttcaaaca tggaagcgtg atttgtgatg gggctcgtct
1532761 ccatcgagat gcatcgctgt ttatactgat gtttctgaat cccatttaaa ctttagataa
1532821 atggcaatga gtcctcacat ccactcaggc acctgaaaag ttgccagaat cgatgacgga
1532881 tacatacaga acattagcac agtcagctaa aacctcgacc cccacccggg aagacatcga
1532941 gatggagtcg ttcccttttt aaaaaaatct cgatttcacc tcctcttcac cgccctgttc
1533001 cccactcaag cctctcctgt catggctcgt agcaccttgg aacaaacaca acgaatgtcc
1533061 gccatcattt ggtaaagcct cgctttggtg cagcggaggt ggccgaaatg aaaaggctgg
1533121 gttactttta cataaagcct gtagccgtca gggctccgcg ggacaggcgc agagtgggcc
1533181 cgataaatca cgacctgcat tcattcattc agcacattta cacccagaca catttctctc
1533241 gcagccagct ctgctgactc ctcatttctg ctcctgcttt aatatctgac ctcggttagg
1533301 aatgtagctg cactgttcag ggggaagaaa aagcgagatt gctgacaata gagctgtaag
1533361 tttccactct tacatttatt tactgagggc aaatcaactt tttttcccca catttcaaga
1533421 atggactggc tgaacatcgc cgcttattca agtgctcgtc agccactggt atgtgtgtgt
1533481 gtgtgtgtgt gtgtgtgtgt tttccacctc ctttaggtga atgcacggca ccctttaggt
1533541 ttaatgcaat tcaccgcggt gagtgacagg ctttgaataa gctggagaaa atggggaggt
1533601 aaaaggggca ttaggagtag ctaaagaggc ggggcttgtc taaagactga caccatctta
1533661 gggctgcgac actcctacac aaacactgca tatattcaat aaaaggtcac acacaataca
1533721 ttcaatacac ttgtgctctg tgtgtgtgtg tgtgcgtgct tgcgtgtgca tatatagatg
1533781 tatggatgag aagaatactt ttcaggccat gttctacgga agatgaagct ctgcataaag
1533841 gctacttgct atttgctcat aagaaagtaa caaactttag tgtttggatg gaaaaaagga
1533901 aggaacgaaa gtctttagct tagtcaaaat agcttaaggg atatgagact aatttttgct
1533961 atctgtaaca ctgtatgaac tctgtataaa ctaagatatt caaataaagt catcgtcaca
1534021 ttaaggatgt tgctgacata gagaggggaa gcttgccttc agtcattaca ggcagatttg
1534081 ctctgaaata acctggcggg aacatgtgtt tctttggatg gagcgtgcaa ccctggacgt
1534141 atcttaaata aacgcgttac atgcaaaacg ttcaggactg gcagacgtct gggcgaaaca
1534201 gacagcggcc tgtgataagc gtgggcttcc tttgttatta atttgttatt tgtcatctgt

1534261 ttggtgcctg gctggattat catgtcgagc acacagtcca aattccactg taaacattag
1534321 caattaatct tcttcccccc cccaaatgtc tgttttacat aaagctgatc aaattaagtt
1534381 gtttgctgtg ggtaaattct gtcatcactt tgtctgaaaa ggctacgata ttcattcctg
1534441 aaagtccgca atcatattat tcagtcacag taactgtcgt ccaatagttt tctttttttt
1534501 aaactgcatg tccagtgcga ctgacattaa ccaccagaac cctgaaccag aatggatatg
1534561 cataatagac aacagccggt gaaatttata ttcgaggaat cctttaattg cttcatggaa
1534621 aaatgtctgt accatattaa gtaatttaat actgccagtt aatggaaagg cagggagaga
1534681 tcatgttttg cataaaacct ttaaagccag tgcttcgcaa tgtggtctgt ctccctcgtt
1534741 ttgatcacgc tgacaccttc acattcgagg taatccccaa tctattttgt ggtctggact
1534801 gaagatcccg cttcctaaga aggttgggca tgttaccata taaagctgga attactgggt
1534861 ctttaaaact gctctttttg tacttagatg aacatacaga ttagcataat tcacaacagt
1534921 ctggcattaa acttattttt cgcatctcag ccttcctaat ccatgatctc tgataggcta
1534981 gtccaggggg aaaaacaatt aaaaagaaat tacgaccacc ttttgcagca caatcgggat
1535041 tataatgatg gtctgtcact tgtgttcaga ggcccaagga aaatatatat cctcaatgta
1535101 tgttttacaa acctgtcagt catttgcaga ttgctggtga tggcctgagg gacgttttcc
1535161 atgcaagaag acatcaggaa ccctgttgac taatgcctgc agaagcacgc gcattcagcc
1535221 gccgcttcac aaggatgact gacaaaaaca gcggcggcgc gagcagcgcg ccttaaaaag
1535281 catctgaccc gcatctgcat agcaaacccc cgtcatgtcg taatgaaagt ttcgacagca
1535341 ttcaaagagg acatgatgga aagcagcaaa ccgtagctgt tgtttgaaga cacgttcaag
1535401 tctcctggga gagagccgtc ttgttgtcgg acctttcttt ggtaacagag gagccggtgg
1535461 gcctgagtct tttggaattt tcaaatgtat gcatgcgcgg tgcataatga gctccaatta
1535521 agtggctcct cttaagatag aaagcccagt ttctcttgtc acaggaggat aaattaaata
1535581 ccgtggttgt gtactgttgc atttttatgt tcaagtggat gtatagagaa cattctttgg
1535641 cagagattga caaagaaact gctgaaaggt ttgatcaaag catcaaagac aagtgaaaca
1535701 gtcgacatgt ggctgtatga tataaattcc ctaaaaataa cagcttttag gccctcttgg
1535761 aatagagaag ggaccatgtt ggccagaatc tgcctcacat attgatggag agcaaaagtg
1535821 tgacagcagt ggcttttcaa agctatctaa atagtgctgc tcaagccgtc cattgtcaag

1535881 gcaaatgggc ggtcggtaaa ggtggtgatc gctctgtaaa acaagcttag accagaggtt
1535941 caaccaagct ctcagatcaa tggagcttct ccagcaggcg tcggggtccc aggggtccca
1536001 gtgacgtaca ccctggtcta aagctacgga tgctaacgac gcgtcggcct gaatctccaa
1536061 caaaagtcca atggacacgc agagtttgag ttacgggtca ggtggattat tgaagagccc
1536121 gccatctttt tgttgtttgg tggataatcc tgtgggaaca aagaactgct ggaattaaac
1536181 ttctcaacct gtccatcaga tttgagatca tgggtggaag ataatggagc taaaggccag
1536241 atccttggga atttaataaa taaatataaa gtaagcacta taaatgagaa acagctgtgc
1536301 aggtatagtg tctcctgaca ctccagtcat gtagaccatg tgagagaata gagaaagctt
1536361 tgtagctcca gttctccccc ttctaagttc aagatttcaa tctgccccag ataacaaagg
1536421 acatggtagg ccaagaagac aatggatgca ggccagggga cagggatctc atatttagct
1536481 cgtgattgca ctcagggtag acaataagta agattcatta ggtcattttt tacttgcgtc
1536541 tgccaagatc tggaatgaaa tgttaaatgt ttcatgggct aagaaaaggg cagaatttgt
1536601 ctttgtgttt gcctcagaga gaaagagaga ggggagaatg atagacagca agatataaaa
1536661 caatgaaaat aaggaatgac tggatgaagc ggaatgctgg ggggatactg cgggagaaag
1536721 tctggaagaa aggagaaaat gggagggtaa aaaagaaggg atggagccgg gaaggggagc
1536781 agggaagaga gggagctggg ctgggtgttt tgggctgtct gatggatgag ggtgtagaga
1536841 ggttggtggg ggcggtgtct cctgaatgat gatggcctaa tatcatcatc cccctgtcag
1536901 gcctaatagg ctttccttgg tagaaatcac tcacacagag ccgccataat gcccaggccc
1536961 ctgacagcat catccatcca ctcaagccaa gcgaacgtct ccaaactaat aaggaaatat
1537021 atggttcaag gagagctgaa aaattaaaca ggtagcttat gaacaatttg tttcgagggg
1537081 atgtttatta catttatcga atgttgccga ggtgtctttc tcgctccgtc ttcatgtagg
1537141 aaacgccagt tcggtgcatg atgtgctgac agggcgggtc tgtaaagtat gaaataaaca
1537201 ccctctcctg cacctccgtg ctggccttgt cctccagtgt tgctcggcct tacttctgtc
1537261 atcctgcaac gccccaggac aggcccttcc cctccgtaaa tctatatatt tccactgtgg
1537321 agggtcatta tcagttggaa taaaaatact ttccgactct ttaatcctcc gatctaacag
1537381 ctcacttttc agatcttctg aaatattctt attttagcgg cagagcctcg taggagtcgc
1537441 ccattcttat tttcctcgcg gcgagcctat ctgcgataca cctgtcattg cgttccctca

1537501 tgcaatggct gatcacagca cactttaaac atggttactc tcttcttatc tctacccata
1537561 ccctaccgat cccgctatct ccaatcgtct ctttaatcat gtgacacctc ctcggcgact
1537621 cccccccac ccgcacccac aagggagaac tgctgccgtc aataacaaca ggaacttttg
1537681 caacatctac tcttaaaagc accttcaaaa cggtcagatt ttattagcga tggcacggcg
1537741 tgaaagccag atggtatcgc gtgttagcca atcattgttt tggttctgct ttgaggagcc
1537801 gtgcgagcat cgcgaagacg tcctccgtct ttcttatgca tatagagtaa ttacttgcat
1537861 actcttcact cattgtgacc gtcaatattt gacattggag gtggggatga agtggccctc
1537921 acacacgtca catgcacgtg ctttgagacc tgctttgcat agagggatgc tttaggagta
1537981 cgagtccctc agattgtttc gtactttaat gactctgttc ttttgcccct gaatattctt
1538041 ttgttgtctg ttctgtgtat tcagctgcac ttcagacgtg gtcatccgta tccttgtttt
1538101 tactgcgcac gtgccgtatg ttccacagga tatatctgca gtacagaggg tgcctggtaa
1538161 taacagaggg cttgatgagg tgtgtttcga ggccattgtg gtctccacgc tcattggttt
1538221 cttttgtacc atgaaagccc ggcgatacag taacagccag tggcagaaag gaactgtctg
1538281 tcgtcaagat ggctgctaac acttgtttta accagccttc caggaggctg cggagaatag
1538341 aagagaggca gctggtaggc ctaacaggaa atgcaatggt aacagtgtca acagctgccc
1538401 ctatggccgc gcaagccaaa cggttggcaa caacaggcca taagaaaagc tacttgtttg
1538461 catagctgga gggatttggt tcatacagta cgtgtaatgc agggatgcca aatgagcacc
1538521 tttgtgtgtt ttctcattag agcattgtac ccgcagctta gtcatttcac aggtgatgac
1538581 agcatggaat gttgagtaaa cagggcaaaa accaacagct gaaggggagc agagctctgc
1538641 tgcgacaaga gggactgctg cccccccca aaaaaagcac acgtttatta caaagtctgt
1538701 aaattgttac ccagatttaa tctaaaagtc taaatctcac acttgtttaa cacaagacta
1538761 aagaacaaaa accattttca attatttagt cttttttct ttaaaactat ttttatgata
1538821 tattatagaa gaaattaatg catatatctt tccccactac cacgcaaaaa tgatcagttt
1538881 taaaaatgag acaattctga attgataatt gagttttata gtaatccaaa ctgtttttga
1538941 tatgtgaaaa ctttaaattg caataaatat acagtatgat ggatagaaag tgactggaat
1539001 gggaagtaac agtgccattt taaaacataa aagggcaaat aattgatcaa accaaaactg
1539061 acgctaagtg cgttgctgat tttgagataa tatacaggca tacataatat acaggctgta

1539121 cgacaataaa gttagatgag tccctggatt aatctcgcaa ggctaattta gcgtagcaga
1539181 cattaatctg actcataagc aaactgcatc acttgtttta aacaaagata agggcataca
1539241 ttttttttccc attacttgtg gcagctacag tgacctcaaa ttcaggtcaa gatgaccccc
1539301 gaggagccga taaatcacag tcgcacctct gctgcccagt ccccctcttc gctatcgcta
1539361 actttgagtg tccttagcat tttctcactt cccccgctcc ttgcacgacc ccctcgtcca
1539421 aacaaaccgc ctgtcagcaa tgcagcgaca ggtacaacaa ctccagctta acgcgaggag
1539481 tgattccccc cacaaaaaga aacactcttc tccgtcctcg actttctgtc tccggcactg
1539541 tctcaccctc actttgtcac tcaggaataa ctcaaacctc agttttgaag ccttttcctt
1539601 ccgcttaatc acagttgagc tgtgaaaata tgtaattatg atatgtacac taagctattt
1539661 gaagatggaa tttgttttta gtctgtatcc aaattccgtg taagagtttt caaataagag
1539721 acgtgagaga taatagtata aattccagaa tacaatatca gggtctttat ttaaatcctg
1539781 gttacatcca atttgcaggg agctttaatt cacccctaca tgatcattgc tggacataca
1539841 tttttgaatc tggtggtatt atctaccatt tgctaactat aataccttgc tgagattgct
1539901 ctgcataaga gcttattcca catctgactt gaagatttgg agctcttctt atgtacaatt
1539961 cattactata atgtgaacga ctcattgggg tcatctgcag ccatcagttt acatttttag
1540021 ccatcattag acgaataaat atcaatgaga ttacctcata agcctcggaa agttttcccc
1540081 tgcaaaccct ttactaccaa aatcttttcc gctgtgcagt attaaagggc tatttctgtt
1540141 agctcgtgtc atcttagcat tgaaatagcc ttttttcatg tggcaaaggg accaaaagac
1540201 cgctttctaa tttagagcga aacacgattc tgataacagt aaagagggag aggaacagac
1540261 attgggatct tctttcaatg accttaaata ccttatctgc acctttgaag ccagagagag
1540321 gtgattggca ggtcaatttg tgatagcatc agtcgatgta cttggcgttc ttaatgctct
1540381 ttgccacaga taagtctgct taatatgaaa actgtcataa ttcatccgcc ttttcagcaa
1540441 catacatctt ctcttgcaat catcaatcca gtcacctgtc aggaaagacg catcaggatt
1540501 cccgttcccc tatccagcct catgtgatta cactttatgg agcttatccc acaccatgaa
1540561 acagtcgggt ttattccttt agtatgttga atcaaagttt ttacgtgagt gcgttgagat
1540621 aaaaaagaag tgagaggaag tgggagctca cacagcgcgg aatatttcac gtgtccagtc
1540681 tcctgtcaca cccgtcatag tcccatcatc tcacagttgg tagtttcctg tgtgaattat

1540741 caaactcagt ggtgaggtag gttccaattt tgcaggaaag tctgcaaatt agccatggtt
1540801 tttttttttt taattttaat taagcccagt gcgtacagca agggaagcag ggccgctgtt
1540861 ctggatggga ctggtgtaaa tttgttcccc gggggagctc tgcgatgtcc gtcctccagc
1540921 ggggactcat cctcagccta atcagtcccc agcttgaaaa atgtgcgggc tttggcggcc
1540981 tatgctgaga ataactgctg agagtggtca gaaattattc attgtttaag ggcctcattt
1541041 gtaacatttc cattaaaagt tgccaccgca gagccagggt ggacatttga tgtttaatat
1541101 ttatagctgt tggacaacag catcatcttg ttttcatttc aagagggtct ttttattgga
1541161 tttctctgcg gagacccctc aggtgtagga ccatgcccat gaggatgctg tcgatgtctt
1541221 ggtttttgtt tgctaaaaat gtaaaacaca tgtttatttg caattgtttt gttattcagt
1541281 gatcatatag cagttacttc attttaatta gtcttattat tgggttaaat acgtttattc
1541341 agaaaggaac ttgaagaaga taggatatga aatttgtcaa aggcaggtcc tcctttggat
1541401 gacaagtacg tcttctcttt gggttgcaca cgttgctctg tgggggcttg catcagacag
1541461 agtagccaca gataatgagc tgtctttaat cacgggagac agtactttga gatcccttgc
1541521 attgcatttg gagatgaggg ggaggggggg tgtctaatta ctgtatcgca gaaaaaaaag
1541581 gggggggggg gggggttgcg cagcttcctc gccattcgaa ctgatgcaga gtcctaagga
1541641 acgctgacag gaagtgacaa caaagaccgt ctcggctccg aatgccggag acaaagaaaa
1541701 gtaagagatg gccaaactag agacgctgat tggaatagat gggaatccca taggtgcaat
1541761 tgacagcata taacataaca gatgggagtt ttgacatttt ttatcctgat aggatgaatc
1541821 ggaagcaaaa atccatcagc agctgagctg acttcgactc agattctgac tgatattgac
1541881 agcagaagac tctcaagaaa cagcaaatat gtttcctgat tgagacataa aatacttcct
1541941 cattgtctca acgccaattg aatgtgacac cttgtctgga ttttgagacc cactcagtcc
1542001 atgaagcaga aattaaattt atcagatgat tataataaaa caacctgact ttctactgtg
1542061 gcgcgtcaga taagtccacc aatctctccc atatttcatt cctgtccgtg tccacgggca
1542121 gcagcataga tttgtcccct aaccctattt gatcattttg catctaaaac gatattcgac
1542181 atttgttatt ccatccatca ataacatctc cgccattctg tgcgcgcgta atctcagcgc
1542241 gcacatgcaa aacgcttaac aataacacgt atgttgtaca cggagccata tatcatccct
1542301 ggatgcattt acatcctgtt aacaatttct cagggcagat tagagggcgc ggagacagca

1542361 gcccggatcc catgagaggt gggctttgga agctgataag gccagccaca ggaaggcagg
1542421 attaatacgt atgaccagga cacatggagg ccctctgatt attccagcag aaaaacggtc
1542481 ctatcccatc agtccccgca tgattaaata cagccctagt gcggacgggg cggcaaaggg
1542541 atggggagat aaccgatttt atttatgcaa gccggttagc ctggcagatc acgtcttagg
1542601 attgttcacg ctacaggggt gagggatcgc accaggggag ctcacccccg atcacacgca
1542661 gcgctgcctc ggctgtcact tttccacctt gacttctctc ctctgcagaa tatagatcaa
1542721 gcattcccaa aatatttcat gtcaaggatc cccaaacaga aatatgtaaa taggcgcctt
1542781 cagttgattt agagtttcct tttaggagtt ggaaatatcc ttcaccttct agttttttac
1542841 ttcttttttt ttaatttcta aagtgaccaa gaaaaaaaaa aaattccatt tgcagatgaa
1542901 gaactcctca gggtgccccc ttgacaaaga cttcagcaat attttgccat ttgtgtagta
1542961 cacaagactt tgtgaaagcc aggagtgcct aattaacttc cccaaaaaca tgtactcgtt
1543021 atagatttaa ttccttttgg tagttagggg ggtacttaat taattaattg tgttaatgac
1543081 ccattaatag tagtgcgcaa gaaattctaa tgagccacct taattaaata ttatctcact
1543141 gtttaatcaa cgtgtttaat tattcacatg atattttctc tggaatcaat ttggtcgcag
1543201 gggtaaaatg ctgattgtgt gttccccttg tgagtcatcc cgggattggt cattccgttt
1543261 aaaattctct tttaatcatg ggaatatacg gtaagccatt tggctcgcat ccatacattt
1543321 tttttttgtg tgtgtgtaag taattctgat cattttgata tcaattttaa ttgacccggg
1543381 ttcacatttt acatctcttt gatagacgtt ttaattagcg atatgagatt ggtgcaatta
1543441 tattgccatg gtcgcagcgt gtctcttgcc attagcaaag ataaatggtg gggttccata
1543501 tattttcata tattaatggg caatctacct ataattttcc ctgttgcatg gtgtacagta
1543561 ttatgttaat gacagcctga tatgactttt atatttttga cggacttgag cgctgtgtgg
1543621 cgccataaca ctaaatgttt aggctccttc ccagaaatgt cgagggcaaa tgtctcatct
1543681 gtacaacaat gtgttctttt ccaggataaa cgcagcagcc agagtgtttg cgtatttcgt
1543741 tgtcgtgcca tcaccaagtt ttttttttta tcaatcttga gcccatgaac ctctgctctt
1543801 cctttctcat ccctcctccc cctcccccgc ttctgcagtg caaactggaa gacctcactc
1543861 atcgtcctcg ttttagctca aatttggtgg gatcaaaggt ggccagctgg gtgtgggcgg
1543921 ggagcagatg gccgcatgct caagggaacc cggcaaaggc ccttcaggtt ttaacacctc

1543981 ctctcccctg ggtgcctggg tgggggggat ccggcttggg gaacctacct
gagctcagtg
1544041 gcatgaccag caggcttcca ctgcaccacc ccctgctttg ctcgcatatg
gcgccggtcc
1544101 acaggcgcca gcctttagat gagattgcga gcagaggaaa ctaaactgta
gaagggcaga
1544161 ggaaggtaag ccttgctcat taggatgagt gttcaagaac aaatccatta
attatcaaag
1544221 caaaaattaa ctttgggttc aaggagcttt ggattaaatt cgaacaggaa
aagttacctc
1544281 gggtctcggg gtgatgcctt tgaagaacct caaagtgatt cgattactgt
ttcaaagatt
1544341 cattcttata attttacctt gcctcttgct cttgcagcga gccaccaatg
gccgcttaca
1544401 aaaaaatgca ggtgctcggc agattaatcc ctcgccaaat cagctccgcc
tcaacaatct
1544461 caaagttgca ggaatggagc ctgctgggaa cgtgaagtgg tgcttgtggt
ctgggattat
1544521 gactttcaga ctttctggcc ttgagaacac aaatgtgggc tcttaagatg
aagttgcaat
1544581 gggaataaac tctctggagg aggggtgaaa ccgggtggcg gcagcagtga
gggggagtca
1544641 tcaatgtttt tctggggata actatttttc cataaatcca tcttaagtag
tcacattttc
1544701 cctttggtta ataggatcat gatgagaata atctttccaa atctgacaga
aaaaaagaat
1544761 gtcctggcct tcaacttctt cctctcggta aatgaacaat ttcaagagtc
tttcctgtgg
1544821 gacaaagggg gtgggtggac ttgtactggc gagcggacaa aaaaaaggac
aagaaaaaca
1544881 acattatttc tctggaatag gttttgtctg tacaactgta aaagaatacc
tgtgtctctt
1544941 aacttttaag atacaactgg tgtgttcctg agggttcgag attgaaatga
tgctccgttc
1545001 tgacatttgc tccccattct ccacttgtca ctcacacccg ggtctacatt
gccacacaat
1545061 agctctggat gcacacaata tgacaaagca aacctcttat tgtcgcgtca
agctttgaca
1545121 gcacgggaat acgtgcatgt ttgctcacct gcaccgacgc acaaacacgt
gtttttgaag
1545181 gctttcgcaa tttcaaacgg caaacaaaga aataatctat ccctggtatg
atttccccga
1545241 cccacgtctg gcctcggagc agaataaatg ccgcatgttc cataatattt
caacaccgtc
1545301 attaggaaaa catcataact aataatttcc ttatcctttt tgtctcgcac
ttgtttgaat
1545361 ataaagtaca aaatggacgt tatcgccttt gatctgggta attaaaaaaa
aaaaaggctt
1545421 tctgggggaa caaaagttaa aggacggaaa tgtctgcagt gctgaaaacc
tgccagcaag
1545481 cgatagaatt gttaatggag gaaaatcctt gaaatgatca gaaatagcca
ttgtgagtct
1545541 gaaatgtttc acaaacctaa gagccaacat tctccccaaa gctagcttcc
cgctaaacaa

1545601 gctaatatgt ttatatgtgt gtgtgtgtgt gtgtgtaaac acaattttgg gacagaatct
1545661 tgccagttta gtgagctcca caacttaaca aagaccaaaa tgctgatgtg ccttttctaa
1545721 tgtagaacaa aggcaccctc cttgacctgt ctacccttct caatgagaga ttgctgttga
1545781 attagacggt gtgtgttttc caccggggcc tttgtgtgtc ctggatgtgt gtctgtacgt
1545841 gtatctgatg tgtgactgac ccattgtgcc cagacacctg cccaaaggcc tcttgtctga
1545901 ctgatgaatc agtgtcttca aggtgtcttt gtagaagttt gtctgctttt gttctatggt
1545961 cctaagcatc ctgtcctgtt cctccatcca ctactgagga gacaataaat ccgctgctga
1546021 caccgccaca ataaacggcc ggggccatcg ttccatcagc tcagagcaga cagagaaaga
1546081 taatggatgt aattgtaata attgtgtgta caggatctaa caataggcct tcatctctgt
1546141 tctacccatc ttaaataaag gaactctttc ctttttgtt tcactttcac ttctatctta
1546201 tttgtgatgt cacacctgct atcattcatg tgccgcgcgg cctgtcaggg cagattagaa
1546261 gcaggggccc gcgcctcgac agccatcgcg cagtatcaat attgtgacgg ctgaggagct
1546321 gcctgtcctc acatctcttc ggagtgcaaa gacaattgaa aagaggaaac gtcacaaacc
1546381 tgacacccaa gtcacttcaa ggaatccctt tcatgaggaa acaaaacctc acctgacaaa
1546441 gcaaagctat cccaggacaa gagtaaatag catcaaacag aaagggtgtt tccactttca
1546501 actggaggaa atgcacccgc tggaatcaat tatgatgaga gaagcgaaat tatctgtcct
1546561 cttaaaacta ttgtggaatt gttgaggcaa tgttgatgaa attgcacgtg tttggatgtc
1546621 gtttttttgc taaaagctaa cgcagacgca aggaattttg cttggctcag accagatgta
1546681 gaatgaggtt ctcccaaagg cagtatcatt gaaaacttcc tgttagggat aatttatgaa
1546741 tgtacattct tcagatcctg tatcattagc cactgccccg gatttttttt tacataacgt
1546801 ttaccgctgg aatttttcat agtttgatca aatgtaatca tttcctccac cacagcacca
1546861 ctcattaacc ccaattattc ttccaggagg ctatcaaaat gactctttct tttctagtct
1546921 tctaataact ccagctgctc tcctcatcaa ccagtttaat ttgggcaagc tggactggga
1546981 tgtaagccaa atgaagaaat gtaacatgag gttaatcacc caaatgcatc ctcaaagagt
1547041 gcaatttgtc gattttctgg cttttccccc cttattttgt acatatcccc acactgctga
1547101 atgcactatt acaagtagct tttgcttttc agaaacagat tagaacccag tgacttatca
1547161 cgattctgct agcggatcca atagtgaagt gaataaaagc tgtttgctgc ctgtggtcga

1547221 ttgggtgcaa caaaattccc agtttttctc tgaaagagat gtgttgctaa catttagttc
1547281 ggatcttttg tttaaccttg gttctgacct gcagtgcaac tctgcctcct gcggaaagta
1547341 aagtccagaa aaaaaaatag gaggaagcta aaacataaaa aataaatgaa caagtaaaag
1547401 cccatgtgtg attccaagat cagcatgcca cggcacaatt actatcactt tgaatttgaa
1547461 atggccattt aaagggcagt agagcatcct ggaaaattaa actggagcct aattagcaca
1547521 ctccaacctg acaacggagc agctttcagc ctgagcgagc agagcagtca agcacgagga
1547581 agtgactgac tggaggactg aagaactgcg gaggccccac cgttccagtt ctgaactcat
1547641 cacacatcca actgtggagc tcacttaggg cacattcgct ccagaacaaa tgcatcatcc
1547701 atcataaata cccacacggt ttaatccgcc cccctccaaa aaaataaatt tcacagccag
1547761 gaaaagagaa cacggatgac actgctcatt ctgtatccat gcttttgctt gtacgtgtaa
1547821 tgtctgtaaa aatctaatct ttttagtcca tgttgttgtt gctcaagcct gtcttatatc
1547881 tctcagcttg ccagcggtgt ttgggtcagg cccatgacct ctgacccctc actgttaaat
1547941 taactctgct cccgcttggg tgttgtgcca aatgtcagga gcggcctcta attccgtctc
1548001 gccgtctcgt tgaccgctgg gcagcagact acctttcccg cccttgcata ccaacctctg
1548061 ctcagatccg ctgcgggctg ttcctgcgcg cagacaggac cacctactta ttcagcccga
1548121 gtcctgtcgt ccaacagcaa caagggaaaa gttgttgcag aggagttctg agcgggtgaa
1548181 aattcagact gtacaatcct gccgcaggga tggaaaagtg ctgaggggag gtgaaaattg
1548241 atactgtgaa ggcaaccgtg tgtgtgtttt acctctgtca ttctacagcc ctggggtctt
1548301 taggacagac tggtcgaaga gaggaatagc cccatggcca tacctgtcaa ggaatacaag
1548361 aggccaactt tagccttcct gccaaaagtt agcacagtga actcaataat caaataagtt
1548421 tacgttacca gagagtaaca tttattaaga tgtcataggc gcttgcacgg tgtgacttac
1548481 cttgttaaag gttttcagtc aaccatattg attaaggctc cagcaggaaa taacttcctc
1548541 tgcacagatc gatccaaacc tgttttggag acctgcgcaa cctccccata ccaccgtcat
1548601 gtggtggcga acatcatctg aaggcgcgcc taatttgatt tgagtcaaat acggggctca
1548661 atcagtgcct attagttcag atttaattaa cccaaatcac gttattgtgt gacaaagagg
1548721 ttttttgtt tggaaacctc attgtcagat cattgttcca ttgtttcact gagctcagtc
1548781 aaaggttgag gaagaatggc cggctgtcga tacgaagaga ctaaagcctg agattatcgt

1548841 taatgtgtga tattgactcg atgggaccag agtgactaag ttatagagaa caaaagtcac
1548901 aaatatcacc tggcaaatat gaattcaagc agaaattagc atcagaaact ataattcctt
1548961 ttgactgtca ctaatggcta ccactgaagg gcaaaatatc tgccatttaa acaggacttg
1549021 aaaagcttta ttgccagctt ggtaaagaag gactctgcac ttgaaatcaa agttctgcca
1549081 aagaccaatt aattggaaaa gaaaagatgc aaaaagaaca aagactgtaa atcaatcacc
1549141 tctgaacaaa atgacccaaa tgccattaaa ttccagaaga tcttattctc ctttgcttcg
1549201 ctctctcatc tggagtgaca tgatattttt tccgcgccac gacgcacgta attgacaaaa
1549261 gcccgcctgc actttgccga agcgtattga gtcgctatca ggtcacagaa tacaaaaatc
1549321 ggaggtgctc gacaaaactg ctgccaatta tctgaagccc tgtaaatgat aactcggcgg
1549381 cccctcgagc gtgaaacaag aagatgaaga tgaatctgac tcttccccat catttcatcc
1549441 cgttttaatt gacaaggcct cattcgctgt taaatttaga ggattacaaa tatgagaatc
1549501 tcgagcgccc catccaacca ctctaatcac acccagaacc ggccgcaaat taatctgcat
1549561 attttaatga ggcaacggcg tcgtggaagg tgtgtgcaga cgtttaatgg attatcattc
1549621 cccatccctg gagttcagaa attgaataaa tatctttgct ataaacacac aagaatggct
1549681 gatagcagag agctttctgt tttaccatat tctcttcctc ctctctctct ccgtctcaca
1549741 gatgaacgtg taggctttac agggctttca ctccactccc agtccggtct gctttggtcg
1549801 attaatagtc caggcactta ctgaacaaag aatatgtaat ttcccggctc tcctaatacc
1549861 agtggctcct gggtttctta cttccagcgt aatctttacg agcacagaag agaatatgcc
1549921 tttcctaaga attaaacctg cttttggaga cgagggagct tgaaatgccc acgtgtaaaa
1549981 cactgccata acttgtagat ttaaaagtta atatgcaatg ttagagctta ttggggttta
1550041 ctactgccgg tggcgaacag aagcgagggc ctgattaaga tgatcccttt tttcccctct
1550101 ccaccaatct tctttcagtc tgctggcact cttggaactt ttcttggagt ttaataaaag
1550161 accatacttt aaataattgt tgaatatcca atggatatgt tttctttgca tctatgcctc
1550221 tacacttcat cattgaaatg tgcttttatt taacagaagc aactttaacc cagaagctag
1550281 atcctcttaa tgcatcgcgg atgacaaagg gggctacagc tcacgacaaa ggaagatgac
1550341 gcacgatacg tggtcacttt taaagcattt tcagtgcgaa acaaagatgt tttaacatgc
1550401 tgtcagtgga tgtgacattg acggtgcact gatgcaaaca gggaccgact caagtcacta

1550461 acaccttaga gcaacactct ttcacaaacg tggcaaattt caagagaaga catcggaaaa
1550521 ctgtaaggac ggctggctgc acgtgctgag gatttgagca tattgttttg catgttaagt
1550581 atgtttaaaa taaaatgtaa atgagtccga ttggtggcga gcaaacggct agggttgcaa
1550641 gcaatattca tgtccagctc ttagacaaat taatcctgaa atttgtgagc caccagtgtc
1550701 agcatttaac tattttaatc gcatctccat tgagatgaac aaggttgttt ttagtttaca
1550761 gagaaagcgt aacaccggca gccatatata ctgtgtaatc ctttataggt tccatatatc
1550821 tacaccgtca ttatgttttc attgtgccca gcgttaatcc ttgctccaag caaacacgca
1550881 aacacctacg gagcaaaaac ctacagctta accttctctt caccctcttc aaggcaccca
1550941 ttttctttga gaaacactga aaatataagc ttcctctttc ggagcaaata aagcggtgat
1551001 gggagtgttt aagtggcagt acaaagagtt ataaattgtt ttcttttttc accttttcac
1551061 aatgccagga agaatatggc cccattacac tgctgagggt cacttttagt gtctgaaggc
1551121 agtgcttaca cagactttac agcgtgtatc attatacttc accatataag gaagcagccg
1551181 ggttgggtgg aggaaaaaag acgaggtcac cgaagctgtc agtttcacgg tgaaaggagg
1551241 aacaaagatc atcgctcaat catcaccagt taaccgaaat gaatctccca ataagacacc
1551301 tgtttgtgaa gtcatacagt agcctcacag ggtttttggt tatccgttgc tttaaagttc
1551361 actttagtgg ggacatttcg aaagcaacaa tcaggtgggg ggaaatcaca aatatcacac
1551421 ctaagcattt tatcttcaat cggcggggac atatctcggg acaaaacggt gataaagcgc
1551481 cggcgcgaca ctgatttgtc atttagaagt gattcgtcta ttgccgtccc aactcctggc
1551541 ggagtgagac gtcacatgtc agcggtgatt gacagttccc agcgctgtca gttagctgca
1551601 gggaagctgc agggaaagat aaggacatct atcagtgtag tccctaatgg catgccgggg
1551661 tcacgtgaag gtcccctcca aacatgatga tatcagtatc tggtaggtct ggttcacttt
1551721 tcctgtaaca ccaatcacca gctgaccttt caaccctacc tgaaccctac agttcatttt
1551781 gattgatctt tgtcagctgt aattaaatac aagatttgac agatcacagc aaaagtaaat
1551841 gagggaaaat ctctattcat aaaaacggtc attatccaac gtgaaaaatc taaaatgtaa
1551901 taataagaag cagacctgat gattttatcc acagctgagg tcccttatat tggatttatg
1551961 tacaagtctt tatcctgcat tcacagtatg gagcaaagct aaaactgact ggaccagact
1552021 gaactacacc atacgtgtgt tatcagattt aactgtcctt tacttttatc tcttcaaatc

1552081 ttcttatttg ggtttataca aatgcaaatg aagaatcaga cactgcaagg
ggtactgggg
1552141 gagggagggg ctgcaccact taaccctaaa gcaccttgtt tgtaaatccc
tcaacatatg
1552201 tagtctgggc ctgagatctg tgtcagggtt tgtgtatttg tgtatttaag
tgattaacta
1552261 tttttgtaat taatgccaat gaggctgcat tactgcatgt tgactgttgg
cggcctgcac
1552321 tccacaccaa cctgctaaag acacccaagg tcacatgacc attagaagta
ataaaaacag
1552381 tttgaacagc aacggatttt aattacggct tagattttga cagtgcatct
gtcactacac
1552441 gaaaacctct tgtgagctca aagatgccgt gcgtgcgtgt gcacgtctgc
ggacgtgctg
1552501 acatgtttga ctgtttctca acaggaggga cctctgaggt caaagaggga
ccctgtctgt
1552561 tgttacatgt tacagttaat aagagtgcca tttgagggaa atgagggaaa
gaggatcaat
1552621 accgaaatat ttccacgtca aacaaatgct gagacggctg aggaaaacgc
tgtaaaatca
1552681 aataacaaaa ggagaaatca gagagagaaa gaaaatgatg aagaaacaac
aggactttta
1552741 aaggaaccag aaagggcgtg tgtgtgtgtg tatgtgtgtg tgtgtgtgtg
tgtgtgtatg
1552801 tgcgtgcgtg tgtgtgtgtg catggggtgg ggggtgggag tgtctaaaga
aaaaaaaaag
1552861 ctttacctat tatctcagtc atcgagcgga ataatgggac aacaaaaaaa
gcctaagtgt
1552921 gcccaaaccc accagccagc ctggctgaca ggcggtactg acgtggttaa
aaagactttg
1552981 gctaatcgac taagcgctcg ccgtccccac ctcggtgacc cagaacagcc
atcttcctcc
1553041 tccactcccg agacccctac agagcaaaca ctgcgggcac attaaacact
catacacatt
1553101 tttaaccatt aacctaaatg gcaaggactg ctcctttctc cttccataag
gtttgagcca
1553161 ttgaataaac tcaaatgata atccatcaat ttatttctac ttaggtgggt
gaataatggc
1553221 gcaatatcgg tcctggaggg agcatcgggg agtacggaca agttcatgaa
aaattgacat
1553281 cgtttgcttc agaagggatc tggagtttcc cttcaaagct ttatgtttga
ccattagtct
1553341 ccaccagcgt tgtagccctg ctccctactc gtcatttagt ttctaaatct
gattgtttgt
1553401 gaggattatc ggtgtctctt tcacctattg gaaggtattg tgaacagaaa
ccagcagacc
1553461 gtttcctgtc ccagtcagga caatagctcc accacattat ggttcattgt
acacatttcc
1553521 aatcttgtag ccgagggaca gttggaaatc cgaaataaag cacaaggtgg
aggatttgtg
1553581 atgttaacgg atgagtgaaa tgattccagt gaaatttcat cttagttttt
ggtgaaattc
1553641 tgtcattaga agccgtgcca gagatcagca gaggatgttg gaatgagacg
atgttattgc

1553701 ataaatactg ctgcactttt gactccagga agctacttcc tgaggcgtaa ccttggtga
1553761 ttcaggggac tgttgtacag taaccggtca ggggttcgac atgcttttct aacacctcat
1553821 tccacccctc tgtgctcctt ccctcccctc tctccctccc tctctttccc tcacagatac
1553881 ccaggttttc tcttctaatt ttaggacgct ctcactcttg acatgtcctg gggtcgattt
1553941 tcacctcgac caccggcaca gctgtccgtc ctcgctgtgt ccggaggaga gatgttctgc
1554001 tttattaaaa cgtgtgaatg taaaaaaaag aggagagtga gaaaaagcct gacacgcaca
1554061 ccacaggacg aggcagaaac tgtatctttg agacgtagca gtgcgccatt taggtgtgtt
1554121 taaaagagag ctgcagaaga aattgtgccc ttgcagaagc ctccatctga caaacttctc
1554181 tggcgaactc actgcaaatc tgaagataaa aacaaaagct ccctcgtcgg ctgtctgtaa
1554241 aagcaccgac tccgcggcag ctgattgtgt accgaccaac tgcaattaca ttcctgagct
1554301 cgcaggaggg gcaaaagcga gcccaagaga ggtgattaac aattcaattt ggattcagac
1554361 gcagcccggg cactgacaga cacggtcccg ccgggttccc tcatcagaga agtcaaaatg
1554421 tggccaccag gcgggaaaaa acaaagcatt ctgctgtcgt aaagcataaa cacagaaacg
1554481 tcccaacagt ttttcggcca ccgctgcatt tgatgagagc gcagtgtttt aatttaaatg
1554541 tgaaattccc agactccccc ccgtgagtcc tcagagaact cctcagctcc cctgtgcctc
1554601 tgtaactcct aatttatttc aagagtgtgt gtgattagaa aaaagggggg taaaaaagcc
1554661 taaattactg tatcgcacaa aatgctataa ttaaattaat attggatgta ataattactt
1554721 tgtggattct catttttcat acacacagct gctttgttgc ctgaaccatt agcatttgtc
1554781 tgctgcaatt aacaaaggcc ctaatgagat tccagagcat gtacgtgtgt gtgcgcgtga
1554841 gcgtgacttt gaggggcatc actctggcat gctctggcgt gtgtgtgtgt gtgtgtgtgt
1554901 gtgtgtgtgt gtgcgtgtgt gtgtgtgtgt gtgtgaatgc ttatgcgtgc agttttaact
1554961 gtatggcgaa cacgtgcaca aatacattca ctgcaccttt taaaggggtt tttgtcacgg
1555021 aaggcatttt tcacaagtac atttgggtgg agtaactcag cctcaatgag agcgtgaact
1555081 ttaaggaaaa ggtggaaaag ttcccacttc ttaatgacta aaggttgagg aatctcggcg
1555141 gtgccacgag aggtacatta ctctcaatct caactgtaat gtattccacc gctcgtgaaa
1555201 aatagctaca tttgtctaat gcacgtcaag gaaccggctt aatcctaagc gatgatcctg
1555261 tccgtcgatt tcattcacat ttcggcggtt agcgagggcg tgagtcacag tcgttgaact

1555321 tcagcgggat tttatggagt ttaaaagcgc agtcgtcaca atcggagtca aatattcaat
1555381 actgaaaatc agcaccaaat gaattccatc agcgtgtcaa gctgcctcag ttagtccgta
1555441 agtgctggta ggtttgagct gctgcctttt tgtcccgtct gttcatttgt gagcgtgtca
1555501 cagttgcatt tgtgtattct gagtagcagc ctctccccac acaagactcc agtgttcctc
1555561 tctgtcttct tctctgcctc tgtctacaaa agatactctt ctgtgccagc actgtcactg
1555621 ccgatgaaaa cgtccactag ttatcggcct gtgtaattgc cgtcttgttt ggttaggaga
1555681 ttagagggct gaaactcttt gtgtggtctt gctgattgaa caaacaggca ccagttggag
1555741 ctcctgtcca agaaccacaa agacggactc tcaactgtgt gatgatagcg agcctgatgg
1555801 actggagcag gtgataattg tgatggttgg tgggggttga taaatgttcc ccttccttta
1555861 tatatttcca tgcatttttc ctgctaagac aacccattac tgccgtaaat tgccaatttt
1555921 agcgacactt taactgatag ttaaagttga ctactttcag gcctttcgtt agcgtgcttc
1555981 ttagaggcgc tgcgtccttg caagtgactg aaatctgcgt tggtcctctg tgtgctgttg
1556041 ctagtcagca gtgacaacct ttatttcgac agcctgcact tgtctacccg tctctgaggg
1556101 tggtatccca tcagcagctc tgtctcagcc tctgccctat acaataccac acccccgtcc
1556161 tcaccattaa ggcatatttc caacccaagc cgtccaaact cctggcttgc gtgcttttac
1556221 ttccctttttt ctgctccctt ttttgtcttt cctttctgct gtccaccctc tcgtgaaaag
1556281 cgtagagagc gccgggctcg ccctgtcacg gtggttccaa tcagccagac aaagctcgca
1556341 gatcctgttt gatactgtca gatggcctgg aaaattaaaa acccaattaa agtggcagtc
1556401 cagaggtcag ctcctctgca tatcaaaagc gctcctctgg ccattctaca caaattatat
1556461 ccgttaacag ctcactttgc ttaggctgca ccagcagtgc ggttggggag taataagtgc
1556521 tttcccatct cgctcttcaa tttcaaaagt aaacctccag ccggaggaaa cagcgatgaa
1556581 taaatcaatg ccaaatcact attgttgttt ctgtatttga gcaagagaaa aggctcggct
1556641 ggtattttaa gatatcaatt taagagaatt acaacaactg aaggaatcta ttttgggatg
1556701 ttggccgcct cactcttttt ctccgttttc tttttgccca cacatgacgt cctgcatttc
1556761 tgctcatatt catctcctgc ttttttgaga aatagccacc aaccaagccc cctcatatct
1556821 ctcccactgc cgctcttttg tgctcgcgac tttgcctacg attcattaat ttctgtacgg
1556881 tggttgggga agaggacaga gagaaatggc cgagcgggct taatggcatg catctcgggc

1556941 caggccatta atattcagct taatgtttct gataacaggg ctggggggcc
aataggaact
1557001 gctggcccag acagccttat taagcagctg aagtggaatg atgctcgctc
acgtcgggga
1557061 tccagcgcga gtgtaagttc agaccccatg cgtgcacggc acacacaagc
acagtctcag
1557121 tcaaggcagt cttcgggtct tcccactaaa tcgcttccac catccgccct
ctatttatcc
1557181 agttgccagc agcagtttcc aaggtttgat aagttgctgc aggtctttgt
aatcgtgtca
1557241 cgccgctaac tacgctgcat ttcattgtca taggttggtc agcttattaa
atgtttatgt
1557301 tcctgcaaaa taagttagtg cggctcgcct tgacatttct cgttttattt
atctgcctca
1557361 cttggttgtg aagcgagcca agtcctcggc tgggtctttt agctccatct
catttacatt
1557421 taatgacatg ataatacaca ttagcagact atttcaaata attacagcat
ttcagtcgct
1557481 ttttagcctc acctcttccc ccaaatgttc ttttacattg gagacactct
gtcctttccc
1557541 ccctaaactt tcatatttct aacagcaatg acagaaaatg cttccccccc
cccgaaacaa
1557601 tatataataa tatgtcacaa ggacaggagg aaaagaaatg attgagcaat
tttcaaaatg
1557661 atgcagggcg gaggggtgaa taggacatta agttcttttc tgtgggagag
gtagtggata
1557721 tgtttttttc ctgtcatgct gggggtcttg cgttaaaact aacagcagac
ggccatggag
1557781 tggtgacagc tctcctgtca ggtcagccat ggttgatgtt gaataagagc
cgccgtaatt
1557841 gtcaagttca aagaatccat tcagaaaaca ggctgtggat tgaatgtatt
atcgtgtcac
1557901 gtactgtccc gcatggaggg aggggagggg tgcacgtgct gcgctgctgt
tgcccatgat
1557961 gagcaaacaa atgataaatc atcttatttt tgttgtttca ttgtatttga
gagagcatat
1558021 gaagtgtgga aacttggagt caggatgttc ttggactgtg caagtaaaaa
gacctgaaag
1558081 agagtttgta gattctacta ttatgatgct agctcgaact ttctttgctc
ttttctctga
1558141 tttaatctgg gaaaaggctt ggatttgatg gagtattgcc gagcgccagt
tggaaataac
1558201 gagtctgatg tcgccatcct gacccggcgc tctgctgaac aggccaccgc
ttcatgcaat
1558261 gtacgcgtgt catgtcccag ctaatggcgc tacatccggt tgactgcagg
atgtgtagtt
1558321 tctttctgtg tagctcacag aaacatcatt tccaaagctg ctgctgccct
gttacagtct
1558381 cattgcagca tttgacagac gcatcagtga tttaaactga tgacagataa
gtgatatttg
1558441 actggacttt atgtaggatt atgccagatc catactgggc agcactggca
cagaggggcg
1558501 agcatgggag ttacaatagt ttaaaataga gtttaaactg ggagagtggg
aagacaaata

1558561 caacaaaaaa gggatgaatg attgtcagga gaggtagcaa cagtctatag gcaactatac
1558621 agcagttcag aggctggaac agccctgcac aaagatggtt ttaactgatt gttgaacttt
1558681 ttatgtcaat ataatgggga gttgaagacc actgcctgaa aaacagtgta gccccaacgg
1558741 agcaggcagg tcaatgtcct gatagtgaca gagcagactg ttacactctc ccactgaagc
1558801 catcctggaa cactttacag cccagtccat aaagctgcag acacctgcac accagatttg
1558861 taaagtaaca gtgccctgaa gccatagtgg agacctgcca ttgcatcttt gcacttagag
1558921 gaaatcctgg ccaattacta gagggaacca accggattga ctctcaggtt accatagact
1558981 gatgcatcct aaactgaaac atccatttga atgaagaatt ctgaagtttt tccacaagaa
1559041 attatagatc ccacaaacaa ctgatggtgt ttttttgtt gatgtgtttc gacagtagct
1559101 taatatttac cctgtaatat cacattcgag accacacatg gattaaggat cgtatatatg
1559161 cacccagatc ctcaggttaa tgtttcctct ttaagaagtg ggggtagtaa atggaagtac
1559221 gagctataca gagtcatttt tttctttatg tatgaaaaaa ttagccatgt ggaagagtac
1559281 actgtgtggt gcctgcatga atctattcct cacagtcgca cagcactggt caaacaaatt
1559341 acattactac aacagtatgt aagtaatttt atgtaaatta tggtgttaat gttctgcaac
1559401 gtgctgttga tgatgggctc ttctggtggc tgtcgtctgc gggcccagca gccactgact
1559461 ggaaatgaac gtgtacattt gccagtaaga gtcatcagta cgccctcaat tcgcttccgc
1559521 cttgctgctg ctgccgtaca ttcaaaaggg atttaccata ttttatctgg cttgaggctg
1559581 gaacagggga gggactttgt cggaactatt tctgtcagtc tgggccaatc tgaactcagg
1559641 atttccgtga ccaagagaaa tgcgaacaca gaagagaaca tcgggaaccg tcctcctaca
1559701 atgtagattt taaaggtcag taaataccaa ctacaaaatc aggttcattt tatatgtcac
1559761 aaattctgga agatcgggaa aaaactataa acccacaagt gctgcagtta gaaacaattt
1559821 ttcgcattat gagcacaaag caacctcaat ctcgtctctt ttatcacctt caccgtcgag
1559881 ttgcctccct cctctggcca gccacgattt acacggccac agaggagtgc aggatttatg
1559941 ctggattttt gtttcagcca tctacagaat taagaagtct atccttgcat ttcattagat
1560001 cccgcctaat gagatgactg tctctcccca aagagcagat gatgaagcac cctatgccac
1560061 ggtgcatgaa ttagtacggt ggggttaaat actggaactt actcatgttt cacaagaggg
1560121 cccaaaccag cagttaagcc aacatgcatg aaaaaaaact gcaggctgaa tgatctccag

1560181 tggaccagcc gcttccagtg aattcagatg gatccctaac ccagaaatct tgatgttatg
1560241 cattcagatt gatatgatgt gcatgcattt aggataaatc tattctactg taataacatg
1560301 tcgagtacat tcactgacag ctggatcgtt gcttctagct ggaatatagt tttcgcaaag
1560361 cctttcaaat tagctgacgt cgtctgccct tcatttctgt ctatctcaac atctctttca
1560421 ccatatattt ttatgcgatt cataagacgc ccctggcagc aaagcagcca gaggatgccg
1560481 agagtctgct gatattccag gtattttaag ctttccggtc cgcatggatg taatggtctt
1560541 ctcaagcggt gctcgtctct ctctaattag tggtcccccc tcgagccgta acaagggcac
1560601 tgacgagaga ggctttttcc caatgataat gtcaatggat acctgcaggc ggagatttcg
1560661 gcgctgttcc tcatcctgtc agccactctg ggtaacactg gggcttaatg aacctgaaaa
1560721 caagacactg tgtgtggtgg tgggggtata cagtatgtgt gtgagcttgg tcctgtacag
1560781 cagagagaga tgactgtggc gtctttcctt gacagagaga ctgtacataa attagtattc
1560841 tgcatagatg atgaagttaa ctggagcacc tcggctgaga tactgggctn cctgagacat
1560901 ttacagagag gcgtttatct tgagtaaaaa ccatacacat catgctgatg cacgtgtcac
1560961 tgaacctgga atgcgatatg gatgccctgc acgtgagaag gcttgactga tacgtcgaca
1561021 ccacggagat gcatacctgg gatgcatgca atgtcacaaa tgtcacatgt gatcgattga
1561081 aggctgcgct gtctgagtga gaggtcattg ctctcgatat acagccagag ggaagggatg
1561141 tgaggggcgt ccaggagcaa atgcctggtc agcacatggt cacatttgga tgggcttctc
1561201 ctcccccact gtcctgagta ggtaatgaga tgagctgttg tttggtggag cacatgtcac
1561261 tccatgtgac acttgactcg ctccgtgctg ccagggatga cctggcgcct catctcggac
1561321 actcggccct tccaccgggc agccaagact accgtacgca agcttgttga cattgaatga
1561381 cgcctgtgtt tattgtggcc gatgggtgtc atgttaggga ttcataagtg ggaaaagtga
1561441 gccgtgcaag tttacattcc aggtaacggg cagcccacgt gcaccacagg aaaaaaaact
1561501 tgacctggaa atttaaacct gcacaaaaaa agcaaaaact agagaaatat gtatgtgaag
1561561 cattcatcaa ggcatagaca caaagaggga aagactgaga cacataaatg gaggatgggg
1561621 accacagatg tggcagggcc tggagcaata caagtcatta tggtgtaaac cgctccaatg
1561681 gcgatgctgt gctaaaagca ctgaggtcac gagaaagcga tacatgcgtg catacgcagg
1561741 agcatgcaca cacggaggaa atcgagagaa catctggatg tgtgccacct gccgaaagga

1561801 agccatctag tttacacgca gtgcaagggg ggggtccctt cctggatgtg caaccttcat
1561861 gctagcctgt accgctggga gaggatggag gacaaagtgg agagtgaaga gcgtgcaaac
1561921 taaatgacac actttgcagg gattaagtgt ttaagtaaac agggatactg tgaggaaggg
1561981 caggaaatgg agagaaatac acaccacgcc acatctcccc tgcagacccc tatcatgtca
1562041 ccacagttgc ttgttccact cagctgcagg gatttgttcc cgcacgaatg ctcactgagg
1562101 agggttacag aatatccaga ggaccctttc aaggaacaga tcaattaaaa atcaaagagg
1562161 ggaatgttac aatctaattt gtgtgaaaaa aaatcaagga cgaaagaagg aaaaatggaa
1562221 agatgggggc tgcaaactcg gcataagtat tgacatctgg ctcagcccaa gctagcgtgc
1562281 ctcaaaacaa ttgcattcat taggttaatt tatcatggtg gtaagctcaa tttacttttg
1562341 cctgtgtgag cgtggctcat taagattcat ctgctgtttc ctcgggcctc gtcatgatca
1562401 ttcacgtagg gttagtgcat ttcggtacag ataggaagca ctctggggtt atctgccgga
1562461 cctaaaccgt ttaaataaag gcggcaagca agaaaagaga ggagaaggat tatggtggca
1562521 aatgagtgaa tgtcacagag atggtggtgt gtacgtgggg gggtgtcagc ggaggagaca
1562581 cttcggcagc attctggaga agaatgccga tgaggagaag tgggcagaaa gaaagaggct
1562641 cggccagagt tgacagaatg gatgttgcgg ggcagtggtg gaagaggaat ggagcgaggg
1562701 cgaggggaag ggaggcacgt gactaaatgt attctgataa ctgaacagca gatgcctcgc
1562761 atggagctcc tttgttgcct gaacagcagg accctgcact tcagtgggct cacttcacac
1562821 cgcatttatt tgcctgccag ctgccaattt agggtggaga gagtgaaaaa gcaggagaaa
1562881 ggaagagaaa gagagagaga gagggaaaag ggagggaggg aggctacacc aattcctccc
1562941 tccctcagca cgtcttgtgg gaagctgcca ttcccttact cttcccccgg tggcctgcac
1563001 aattagcgat ctgcataacc acccacacct ccacacactc ggaacagagg cagagcagcc
1563061 tttgtctttt ggctgctgca caataagtcg ccacagactt ggctgaaagc acgaaactgc
1563121 atgttctcgg cggatgacga atgggatctc gaatcttgtg aaatgcggca caacaaagac
1563181 ggcacgagtg tcctacgctc agaagagttc atcgctccag cctcaagccg tagagatcat
1563241 taacgcctct gcaaaaacaa aatcacccct tccccccgtg ctaagtggta caagcccagg
1563301 caggaagggc taatgggttt gaccagggtg ggggtgcaag gtgagactca ggtgactgca
1563361 ataacttcaa cagctcacct gaatcctgtg acgacattaa ataaagcaaa tgacagctca

1563421 cgtttgatac acctccacct tttcagccag aaacgaagaa cgtgttactt tctatttata
1563481 aaatgaaact ttttttact gctggagtcg aaagctctgc tcatttgacc ttcagcatcg
1563541 tgttttttca gtgaggcgat gttggatgga tgccgaagga ctgctgaccc tgatctgacc
1563601 catcattaag gcttgtatag gggaaataat acaagtattg atcaggggcc accagctcat
1563661 caaaaccgga gccgatccca ccctcagggt cactgtcccc acttcctgtt cgggctagcg
1563721 tggagatgat taaaactttg tgatcagggg ctgggaacat tccatttccc tgtgatttgg
1563781 ggaggaagtg ggggtggatt aatcttaagg ccatcatttt actgccggtc cccactcctc
1563841 tctcagagct gaggaagcgc aagtgaataa tggcgtgtcc gtcagccgtg ggctcaggcc
1563901 cagatcgggc ttttctccgg ggataatgca aagcgcggtg acaatgggtg attaaaaatg
1563961 tacgatttta gcccgatgat actgcgcact attctggtgc agtgggaaag ggtcttccag
1564021 tatcggccag agtgctgaga ataaacatga tggtcctgaa gaatcctcat tcagtcctaa
1564081 ttgcagaata ttttcctcat ttataatctc ttaacaatct ccatcagttt gttcttttca
1564141 gtacaagcaa cggtcccact aacaaagttg tcaggctccc caccaagcca gcacctgtcc
1564201 tgtcagacag gggaggcaga ttccgtgcca ggcgtccacg gcaggagcgc catccatcag
1564261 aggttccatt tgatatttaa tttagacttt tatgagcctc agacgatggg ccctgtcatt
1564321 tttctcctcc ttctgtaggg atggaggata aattccttcc atccatgcag cagtcttgcc
1564381 aaggtttctt cagatttata actgtatatg tgatagcgcc aatgcttgtg gccacacacg
1564441 ggccacacaa cttctcacgg gtcgtatctg ccccatcttt gctcaaaagt ggattaaaga
1564501 aaaggacact ctgatttatt gttaggattg gtcacggaca ttaataagaa agagcagtta
1564561 tggtgttgcc gtggcggggg aataataata tcacattaaa agtagcagac cagaatcatt
1564621 taatgttgaa ttaatcaaga gaagttgata ttttgcatac atgtttttat gtccatgagt
1564681 tatggtcgag gtagaacatg caatattctc agagggtaat tttaaagagt agcaaagagt
1564741 tttataaact gtcctctctt aatgttctgt ttgaacctaa tttatagctt cattcattac
1564801 ccctttgag taagtaatta tttctaaaat accaaagtgc catcttttct ttccaggttc
1564861 ttctcaggct ctgatttatc accccgtcca tggtcggatg cagtcttccc aaggtgtgac
1564921 atttcaaaat atttatagct ccagcatatt aaagtccttc ctttaatggt ggccaaccac
1564981 tcctcctcac tagtgtaatt agtggagcct ccgatgtgcc gtgttatttt tccactgcgt

1565041 tctgtcggac ctgtacggaa aacacggcgt tcgctctgct gacagggaag acgtttcctg
1565101 tcgcagccac aaaactttca aatttaatta gtttcatcaa acctctgcgt ccaaagtggc
1565161 aattaccatt caattaagct gactgaaggc attatattaa atttaagctc tttatgagct
1565221 ccatgatcat tctggaggat gtgaatataa tcataatctt tctggatata tattatcctg
1565281 ttcattcact ataaaagtga cagttttagg tatactttca atatttctta cctataccaa
1565341 taatatttta ttgccattta atggacttta atgagtattg tactcagggg attactttag
1565401 gtatttattt agatgttttt ttaagtaaaa acaggtggaa cagagattgt gcagctaaat
1565461 tattataata taatgactat taataatgac aaaaataaca atctgcagta ttttcctggt
1565521 ttggaattag ctgacacagc gctagcggtc tgggcaacgc accccctggt ggaaacgcaa
1565581 gtcagatgag tgcaaccgaa cgtaccatgt aatagtgcac tgctaggtgg gaatgaacca
1565641 ctgagtggct catatttacc ctcctatcac agcaaatgta catatgcgtg aataaaacct
1565701 gagcaaatac aactttcagt aagaaatcat agcaaacatt tgaaaagaaa aaaaaaactc
1565761 ctcctgtttg accaccccgt cttctcattt gtcctctggg ctcatcaaag gtcagaggtc
1565821 aaggctaagc cacagaaggc cattgatccc gcggcgtcca cggccagtca gcaggctatt
1565881 taggcgtaca cagagagcag tgtgggagcc tgagagaggc tggtgttcct gagcgcttct
1565941 ctgtttgctc tgtgtgatta ttctctccag ctcccggagc ggtccggctc ccgcctcggt
1566001 gataaagata ttcatttacc tgatatgaaa actcttgctc atgttgatcc caaatgactc
1566061 tttggagatg gaggaaggaa gcaggaaggt gaaggaggag ggaagaggcg ataggatgga
1566121 agctcacgag ggaaggcaga gaggcaggca ggggagaatg acatgaaata ttaatatatg
1566181 atcccagccg atgtgacact gaagtttaca tgataacaaa ataagcaggt cgggagtttt
1566241 ttattgactt ggccgagacg cacaagtcat ccgaagtgga aaatatgaga atatgaattt
1566301 ctttatttca atcggtccat cttattggga ttctgggaaa tgaaagagtg gatttttct
1566361 gtgttctaat gttcctgcgc tgttgtcagg gagtatttct ccgcacacac gtcctcccct
1566421 ctctggtaat aaaacattca ttccatttta actgctcgtg aatgaggctt tacgcgccga
1566481 gctggcatcc atccaccaag ccgccacaaa tatgagaccg agagagatag atttgatgac
1566541 gaggggctta aataaagcca aacgtgtcct ttcgaatgca tgcttgtggt taaaagggta
1566601 aaacgagagg caggaacagt ccctcctctg tcacggagcc agcctgtccc ttctttcttt

1566661 cctttgcccg agcgtacggc tcactcatct caggttttat tgtctctacc ccggcgagcg
1566721 ccgcatgttc gccgcgcgca acgcatcatg caagttcttg attctgtgct cggtaattta
1566781 aagcctttat ggaattgcaa tttcagggct cagtgcttgt ttttctaaaa tgtactcaag
1566841 gaattagcta ctccagccac aaatcagttg aatttatgca cagttgtaat acactttatg
1566901 tgctcatggt acattgcttc aaaaacagca gatgggaaat tatgataaat ataatgagga
1566961 tttttccccc cctctttgct gcctctctct gcccccccctc ttgactatag gagagaagga
1567021 tgtgaagcgt gtttacttac cccatacaat gcatttaatg tcttagtaag ggcagccaac
1567081 aaaacctgat taatgtcctc atctctttta ttaaagataa gagtggcact gcagtcagga
1567141 gatagagaga gcaccataaa tctcaggcaa aatgagcagt caaataatca ctcatcagaa
1567201 atgaaatcta catacattaa cactcgcaga cgtacggcgg agaatataaa cacggggcca
1567261 cgggcgagga agccggatcc atttatccac atattcatgg ttaggggaag gtttctggag
1567321 agggttctca gccggtgatg atgtttaagg gagtctggct ctgcaaagtg acagtccacc
1567381 atgtgagcaa gggagcagac gctacgatca ttagggcaat ggatgggtta gatggcgaag
1567441 gcaattagga gtcagcctga atgggacgta atgttgatcc atccctgtga ttaatcatgg
1567501 catgcattag acagtgatgg gtgaccctct gcctttgtgg caaaatgatt cttcatcctg
1567561 tgtttgggga atgggattct gaggtcccta atgcgcctta gaggtgcccc aatgggtctc
1567621 cgtcatttat tctctgtccc tccctgacag ctatcaacct gtcttcccct agccaagaag
1567681 cattatcgga actgctgtgg tagttagcag caggaactta gcatgcatga gtggtgatag
1567741 cagacagagt gatatgtgaa gcaatgctga tgctactttc cctttgtcct ttcacaatcc
1567801 ctgttaacct ggagtttagc ctccacttgg aaattggaaa agtccaaaaa agttcatctt
1567861 ctttcaggaa taaagtttgc tgtaccggag ctgaataaat aggtgagcag tatttagcca
1567921 taacgagcaa agttactcat atttacatgt gtgacacaaa tacagataga caggaaagag
1567981 cagtatttct ctgaacgttc acaccatata agttgcattt agtgaatcat aacaatgaat
1568041 agcaatagca caaatgcagc tgtgctattt ccgtcacaca tccaaggcct tgctgtgaca
1568101 aataccaaat gtcaacaatt ccaaatgaat ccacaatgag tcaggactgc tctttgtttt
1568161 ggtgtcagtc atatcttgat cgtcaaaatt attattttca ctcctctggc tgccaccagt
1568221 gtggcatctg gcagggatgg gatgattggg tgtttttacc gcgcaaatga caaggaatgc

1568281 gttttcgatg cctgataata ctgtcaatcc acctcgcgta aagttgtgca aagtgcagca
1568341 gctttgctca aaatattgat agtgatgggg agcggcgccg acggtctcgc tgtgataatg
1568401 atttacttgg cagcggatgt atgtgcctga ctgacatgac tctaaatctc atgttgctaa
1568461 tctttccgaa gtctttaact ttttcaatta ttgagattta gcatttagct cctaatgctc
1568521 ccgtctccct gtaatttccc ctccgtccac atggttacct tttcacctgt gctgaaggat
1568581 catgagagtc attacctcca tcattgagcc caagcatctg agagcccgct agcccagaaa
1568641 aggctgtgcc atggcgcgct gatggtacaa tatgctcctt gttattgcgg gatcattggc
1568701 gctaaagccc tggcaatcaa gccagttttg ttgaagctca ttggctcagt ggggtttcat
1568761 ctcatcagtt tggaatgagg aaaggagtat tcgggttagt tgggtcgtta ggtaggaatt
1568821 ctttgataat ggtgcatgtt acattagagc cccacaaacc aaggataagt gtttcagact
1568881 ctgtgtaatg cagtttatag aatggctata gctgataaga cattctcctg aggtggaaaa
1568941 tatggagatg aatactttct tattaaacct gagtgacttc tccggctgta gagagagatg
1569001 agacactaat gtctggacag gtatgtatga tatcaaggtg cagtacagag caaagcagca
1569061 tttggcgttt gactgaatgt atgtttccag attgctggtg tgagtgtgac tgctactctc
1569121 ccggcattaa taagacattc ataaatgacc attaggggga aaccatctct cagcgctgac
1569181 aggagcctga tgagatttac atccatgatg aaatggaaac agctgagcag ctgtggtcag
1569241 aggcgacctg cagccaccag attcccctca gagttctgcg aaacacagtg accggagaca
1569301 ctttccacac caaatgtctc ctttaggtct gcggcaccag ccttgcagtg ggcgtgctgt
1569361 agcggggtga agatattggt acggtccaat attgagaatt gtgttactca tgataacggc
1569421 cacaactgga caccattcaa gctcacacat acatgctatt ttacctctct gtgttctgtc
1569481 atggcccgga atgacgtgct aatgctaaaa aagtgcaaag agagtaggag aatttatgag
1569541 aggccacgca taccgctttc tttataaaag atgaaagatt ttaagatgta ttgactgctt
1569601 ttcttctctg tgcactggac gacccgtttt ggctgtcggc ttctttaaac agccatcctt
1569661 gttggtcact taggcctgga aacatcctgg tggttacgtg cttttgcccc atcatgcatt
1569721 tcagtgtcat caactcctgg aacccagcta attagaatca agaagcaaat ttataaaatt
1569781 ttggacactg ttgtcttgag gtgtctcgcc gtccgtgccg atttcaggtg ttccgagcgg
1569841 ttccccccttc tcgaaaagtc taacgagagt gcagaagtaa aagccgtttc ccaggggggc

1569901 tcaggagcga gttgtcgcgt aatgctcgcc acagcccctc ggcagcatct ccgcttcatc
1569961 cctctccccc tctgggatgt caagaggaga gggggcatcg cggaggaggt ggcgtcatgc
1570021 agctgccgcg gcccagcaga gctcctggag gaaagacgcg ttgtcagtga gcattaatca
1570081 tcgggcccac catcggaaac agattcccct cataagcaat ttcactcatt agagttacag
1570141 tgctgatttg ctgattgaag ccgtgctgag gccaccgcgc tgcgaggaag gggggggggg
1570201 ggcaagggag ggagggcatg acacatttac agtgacgtga aaactgagat gttgattaat
1570261 gcgccttgtt cccgtcacat aaactgaagg aataaatatc aaacccaagc cgaggcaggc
1570321 gctcatttcc aaagcaggaa aggaaagttc catttggagg caggctggga ctactggtga
1570381 cactaactgg gtccagagag acacacagag gaacgccgac tgttcctccc aggtcctccc
1570441 aggtcttccc ctcccataag tggccttctt gttctgcaac cagctcttca aatgaataca
1570501 cactttttta cgcacaattt atcccactgg catgacgcgc tgggtccctg caacagttgc
1570561 ggcccctgtt atcatttata tttatgctcg gctgtttatg ttgatctaca tatcctattc
1570621 gcaaacactg ttgtttacat aaaggcacaa tattatgttt gctgccatgg aaacagttta
1570681 cccgggcctc aaaaatcacc gtttaatgga gtgagatgtt tactcggcag ggaattctgc
1570741 ctaataatgg catagctcaa ctgtcctatt atggcttttc ataagttgga tatgtgttca
1570801 tgcaggctct cgtggcaacg ctgttcatgc tattgcgtgc atagctgcga ttctagcgat
1570861 ttttggagca tcagcggtgt tctccttaca gtagagatgt acgtttggaa gggaaacaga
1570921 cttttggtcc ttattcgggt atcaaatttc agtttatttt cactttgttc ctgacacaat
1570981 gtctgagcag ttgtttcctg ccgtggcgaa aacccacgaa cagatgcaac agactgagtc
1571041 cattgtttaa cctttgccaa cttgttttga tttgatgcta tccattacac cccccccccc
1571101 cacacacaca cacacacttg gatccctgag tgacccccac cccacgagtg ctcattagct
1571161 cgtttctcca ttttaggaag cagcaaaaca tccgaggcca tccgagcttt tcctgtggag
1571221 cggagccaac aaccgcatga cttaataaac ggtgacaaac ataagccagc gctcacatca
1571281 gtagcattac gtagcaacaa agacgcggcg cgagttcaac caaataaact gttactacag
1571341 tgcgcgcatg tgtctgtgtg gtgtgtgcgc gttgtcttca tcatttctct gctttgatgc
1571401 tttgatctat ttcaaacggc tcctgacgtg gttcttctct gacggcgttc ggcgcgctaa
1571461 ttaagcgtcg gtttgtcagg cgagtgatgg ctgctgatgc acctctgtcg cttccaccgt

1571521 gacagaacaa cactcgggca ggaattcctc agcctccttt attctccctg gtttcacaga
1571581 tccccgtcgg cccggcagaa gtcattttac agttcaatca ggaattctaa taaactgacc
1571641 tgtgctttaa tgactaattc attgtctcct ttactacctt catgggtata aagtggatta
1571701 atgacttgag cagggttaaa aacagtacca ctgtgctttt ccttcatcta ttatcaatca
1571761 tcctttagat tctttggaga gcaacggaaa ttgaaggaaa attagcaatc cttttgatgg
1571821 atgatggcac aggagtgcac gctcacagta tagcttacct atagggttaa atggaaatcg
1571881 ccttataaat agcatcacag taggacatag caaattataa cgtcatacga tgggaaattt
1571941 aagaccaaca tagatttatt caaggaatca aatggaggga agccgttttc tcgatggaca
1572001 caaataatct gccgcagttc tatagcctgt ttacaacaaa gtgctttttt atttttattt
1572061 tttttttag aaatactcca aggtagaagc cgaaaaaatg ggagttgagt ttcatggaag
1572121 ttgttaaact ttgtttttga cagtttcttt acttcccccc cccctcactg tacctctttc
1572181 ttgttgtttt ttttttcaaa aatcccagtg aagagagaaa aaaggaagta aaacaaagga
1572241 gaggcagaag gagaagagcg aggagaagtg cagagtgggt ataaggctgc gtctctttgt
1572301 ttaccgctcc atattcccca tcatgctcct cactcctcgg ctgcatctcg gggctcttcc
1572361 cattcttggc cccagtcatt tctgacaatg ttaatgattt agcggggcgc ccgccacggc
1572421 ggatgatttg tgacacgtag gaaggctcag aacgggggag gactgggcag gtttctgcgg
1572481 ggagtgtttg ggatccaggg atgccttgaa aaaagtgatt cattatattt aagacaagtg
1572541 tgacatccta cttcaaggaa gcagcacaca tacgtatgga ggaggagtgt tccttttttt
1572601 ttcgggtggc caaagtgggg agggggtagg tgagtacaaa ggaatgcctg ggtgtttttt
1572661 tgagatgaca taggaatagg aggcaggcat ataagacggc agactgggaa ataatgggta
1572721 atggaggaat ttagcccggg gctatatgcc tgctttgcct acttctgtat aactagtata
1572781 tttataattc aaatacatac ctttaataat taacaactgg cgaatattaa agatgaatgc
1572841 aggatgtctc tctgccgatg gagtcaggtg aaagatgcca ttcggtttca aatgtgacaa
1572901 caacagaaca aaagagggag gccataacat tgtaaaaaaa ataaagtaaa atataaaagt
1572961 cggccgccat tttgggactg actctgtatg ttggtcggaa atggatggga aaaaggtata
1573021 tttactggct ggccattaga tagattataa catcgtagga tttttggaat gtattgacag
1573081 cttttgtttt ttccttatgg aatcaccggc tgcttgttca gtagcattgt tgttttgtgc

1573141 gttgttgttt tgtgcgttgt tgttttgtgc cactgactcg tggcgtcagg ttgactccct
1573201 tgaacgatca agcaacccaa ccatcttgct gacagtcgcc tgtgaaattc cagcagcgtc
1573261 accgaaacgg aaccggtcag gtattgacac tgttttgtct acagcgtgac agccgcgtca
1573321 ccctcgcacg tgacgctcct gtgactcgac gatagcgatc ggctcgcatc aaacgcaccc
1573381 gcaccccccg tgtgatgtgg attcaaacgc atttgtgccg cagcaatttg gaagcattga
1573441 gattccgagt gtttatgccg agctctgagc gcgcctttta aagtaaacta tcccagagga
1573501 cggtgtgttt tgtagcgccg cagaaacaca ccctcagact gcgtcgaccc aagttatgca
1573561 aacacgagag ttcaacaaaa accaacagat ttgcatattc tggaagtgaa atgtgttccg
1573621 cttttgtgca ggcctcattt ctgcctccgc gctttgatgt agctcatgca tattgacgac
1573681 ccgccgaaga acccgaccac gttaccgaga gcacgggctc atcccctttt tgaaaaacag
1573741 cggcgcattc gtttgtccca tttatatttc cggggttaat tagagactga gagattggac
1573801 aattagctca tccgtgctaa ttgattacaa tattacagcc cggcgatgat aggatgaaca
1573861 tagattcctt gttccagatt cctcagaggg gttccagaat acatacaatg cttgtgtgta
1573921 atgagggttt taaaaagagc aaatcagctg ggaaattgtt aattcaattc cagtcagacg
1573981 gcactgaggt tttcagggca ccaatgaact gaaatagata gcagggcgct gacacgcgtt
1574041 tgatttcgac tcgtgtcgac gcagatcagg tgcggctctg cttcactccc gtctgtggaa
1574101 caatttagtg ccaagtccag aagaactgat cagatcccta aaagagcccg cctcgtgtct
1574161 gcagccctcc aacaccagtg tgttttcaca tggacgcggg accacctcag tcatcacctg
1574221 tcaggaacct ggagagggac gctcaggtgt ggacgtccaa ccagctctca caggaggtcc
1574281 cacttgagct ctggcatgcc gtgacagaca gtccggctat gcggaggaaa ggtttaagct
1574341 gctttacctg gaaagttgca caggaagtgc tcaaacttcc acacggcgca gaaaactcaa
1574401 agcagggttt atattttaca gtcgcctgcc gttagatttg atggaaaact tgggcctcct
1574461 ctgcctcctg gctcagatta atcatgcaaa agcatcgctc ttgcatgatt ataactcttt
1574521 atcgcttaga aggcctcggc gccgagaaga ttccactctt ccggccccgt tttgtgaata
1574581 atgtttgctt tcacctcaga atgtccgtcc tgaggtgccg cggatgaaag agcaagacac
1574641 gctaatgttg aagcgctgcg cttctttact gcgcttaata attaataatc caacacactg
1574701 ccctatgaat attaacatgc aaagtgtccc tgggagccct gcgagtgcca gaggggggcg

1574761 gggagtgtgt gtttaccgca ttaaacgctc aaacatcttc ggcagccgta tttcgcctgc
1574821 aaccacgaga agcagccact aaagcatcgt gagcgttatt tgttgttgct cattgtaacc
1574881 tattcaactt ccatttagaa gtggcaagtc aaatcacagg tactctcgcc gcgctcggcg
1574941 gaagtcccag gacgccgctt gtctcgcgga agctgttttt ttctttcctt aaacgcgtct
1575001 ttccagagcg ctggcatcat tcactgggat caacatcccc ttacggtgtc caggggtcaa
1575061 aggtcccgtt gttagacctc cctggacaag gcgtgattct gaaggaaagg gcatcaatta
1575121 aatttagcta accgagctaa tcggactctt cagttgtagg cttgacaggt tcacctctat
1575181 ctcacgtttg atttcataaa agcattaaat ccagattaaa gacgagcaga gaagcaccgg
1575241 atgaccgctc cactcgtgac agcttcagag cacatttgga ttcatgcctc cccgacctct
1575301 gacctttgga gagacgcgca gcgggaagca catcagcaag gtctgtcagc acagcgtcgt
1575361 cgctcttgtt gaagttgctc tgcagccacc atcaaggata ccagccgcta ataatggaca
1575421 agcgggctgt gtgataccaa taaacacgct cgggtgaacc cggacatgtt tggctctgtt
1575481 gccgacattg gtgatattta tgtaggaatg gaaggctgaa tgttgtccct gcctccctgc
1575541 agctccccg agctctcaga tctcctctcc gtctcctgcg tggatgagcc gccgccgcaa
1575601 aggcaacggc gctctttgat atttcactgc tcatttgaat cagtggccgc tttatttgac
1575661 aatgccaggt aattacctgg gtggcaggct tgatcatgtg aactcccgcc caaccagttg
1575721 agatagaggg ggagggaggg agggagggag tggggaaggt ggcgggagtg ggaacgcgta
1575781 aacaggaagg aaataaaagc aggacaaaac caaaacacac aaacaggaga cgtctgcatt
1575841 tgtttgcctg ctcatttttc tcaagtgaag gccgcacggt gtcgggttaa acaactttac
1575901 gttttattac attcattgta aatgcaaaca cctttttag aatgattttt ttgttctttt
1575961 agcacgtata gctttgttgg ctagttatct agcatttgtt atgaggaaga aaacacacac
1576021 acaaataacc cattctttaa aaaaataata cattttaatg tcacagcatt aaaacatcca
1576081 taatttcaca gaagcagttg tttcaacatg atacagattt aatgctggtt aaaataaaac
1576141 ttccatttac aaatgaaaac ggcagtgaat ttcaatgggc ggatatggag actcgtcaat
1576201 gatgtttatt gaacgggaaa gcagtgttta ctctgttgtg tgactggaat atcaaaagca
1576261 gccagcccat gtgaatgcag ctgcgttcag gctgactgcc tgagccctcc ttggaggaaa
1576321 ccctgttggg caaccctcaa gttgagctga caacacgggg tcagggttgc ttcaacaaat

1576381 aagacaactt tagaattaga aagacatttt tagaaatctt taaaaaaaaa aaaaaattct
1576441 aatacagcga agactctaag cgaagctcgt gattattgaa aggtagaggt agcttagagt
1576501 taaatacagc acagttatct attcagtttt ttttaattac ctgcatttat tcataaatcg
1576561 aatgttattt tctgatcgca catgcacagc agtttttttt tttttacagc cgtgtgaaat
1576621 atctgctctc cgtgcgtaat ctcggacact cgcgcacttt caactttgta gttttatacc
1576681 ctttctgatc ttagcttgaa aacgtcgaca cttcacattt ccccgctccc aggctggaca
1576741 aacaaggcga gggtggaatt tgtgttcatt ttaaagctga gtgggggaat taagacaatt
1576801 taatgaaggc ttaaatcgcc ggggttgatt ctccggcgcc cggagccata tttcctcaga
1576861 tcagcggagg agggcgactt tattggacat gtttctattt ccaaatccgc gtaacaggca
1576921 acgatgcatg cacttcctat aatgcagata agggagagag ggtttgatat gtaataaaag
1576981 gtgggcctgg gctgcctgtt taagcccctt aagcctgata aatgatgagc gacagaggca
1577041 cagcaagagg gtcaccgcta ttaactcctc gctttcccgg cctccactta aacttctctc
1577101 aattacatct ggtaatgtgg ctggatatgg gcctttatga gcctcgatct gtttcaatcc
1577161 aacctttaaa aaagatatct aaaattatag ccattttccc gcgcgccttt gtggctgcgc
1577221 gtatcgagct gtccccaaaa ccacgtgtta aggaggaaaa acaggaggat tacggtctgt
1577281 ggcgttccta acacatgcac accaggtatt ctgggacaaa cacccggcgc aagctcatta
1577341 aatcagcatt tggtgtatcc acacaggcgt taacgacggg ctctaatctc ggcggtgacc
1577401 tgctcaaagt gtgtcacagc tgagcagaac tgacactcgg agctccacaa ggtgattttt
1577461 atttgttttt acgcgttcag ataatagaaa tgaaaagaat gacttagtct acaaatttga
1577521 cgtgtgaact tttagcatta aatttaccgt cggctcattt ctgttattaa atagtataat
1577581 aaacaataac ttaacctgac actacgtgct tcgattattt ttatatttaa acaaatatat
1577641 ttgtttcaga tttttcaaat gaaataatta aaaccagata caaaaataac aacaggcttt
1577701 aataatttcg gcctcggtgt tttttctcga atatttaatt ttccatatcc tcgactttca
1577761 tttttctttt atttctattg tttcactttt ttaacgtagg aaataatttc atttgaatcc
1577821 ttaattcaca ctttcagaat aatggcgcct tcacctgaag acaaatcatc gcgttaaaag
1577881 ctgcttaaat tacagcattt ccagacgtgc tgggttccaa caggatccga gctgcaaacg
1577941 agtgaaaact ccatgagcca aaaatgcatc cacacgggca gagacatttt cttctctaaa

1578001 ttttaatgtt cgtgcttctg tgtcgttacg aatctgaatg aactcgccag cattacaatc
1578061 taaatcaaat caaacgcaat ttaaaaaaaa aaaaagtact aatcgcgcca gtttagctaa
1578121 tgacttattt tgttttagtt ttattggtct ctgatttgct gcaacgcttt cagcagatcg
1578181 atgccctctt aaaccgccag gtgtcgctat atggccgcct atttccaatg agagccgctt
1578241 ttacgcaggg agaagccgcg gttcccctca gcgatggtct ccccccctctc tctccctctc
1578301 tgtccctcct cccctgcact aacccgctga caccagagtc tgagaatcgg tggaggggaa
1578361 aatgaggctt cggagccgaa gaatttacag ttagtcgcga aacttttaca agtatttgag
1578421 ttgctcacgc tactttctga aaccctatag tcacatttca ttccagggtc agagagagtt
1578481 cagatccaga tgactaatgc gttcagcatg agcaaaacaa cgaggtgcca aattatcatc
1578541 agtaaatcaa actttcactc tagatgcaat ttttaaatat acctaaccgg aaagagggca
1578601 aagacgcaaa attgaaatga gcagaatttg gcctcagtag gtgtataaga caatgtaaag
1578661 ctctgtgtta aaaataacgt tttatataaa atgactgagc gggtatcgct ttattcccgt
1578721 ttttgactgg acaggctgga agaggttccc gtgaactcca gtatatgagg ctgtgatcca
1578781 ctcttatctc ggcgcctcta tacattagtt tagctgattt gcataatctc aaatagtgaa
1578841 gagggccaaa gaagaacaaa gttacaaaca agattagggc gattttattt gttcctgtgg
1578901 ttgagcgcca attgtttctc caggagtgtt taaggtcact aagaggattt gaagcccgtt
1578961 tcatatcagt aatctttttg agggaattaa atcaacaatg catttctctc catatttagc
1579021 aacaggctgg cctgtaacag ccgagccctg cagggcagga gacggagaga ggacgtgtaa
1579081 gtcccgcgaa cgagtaaaca gaggaggggg ggaggtctcc gtgctgccgc ccgaaagcag
1579141 gcaggcggcc ccgcgcacct ttcaggatgt gacattcagt ggatctttag cctggtgatt
1579201 gtcccactag tgttttcctt gaaatggatc ttgccccccc ctcctccaga tgaacccaac
1579261 cgcagctggc catgtcccac acggacaccg agagctttaa ccctaaactg accccaattt
1579321 gacaccacca gatgaaattt taccgcctgt taatgtcatc ccctgatttt tcgtccaccc
1579381 ccccgctttt tttcatatgc gtacgaatag tgagcgcgca cctggaaacc caaactagtt
1579441 tttaaaaacg gatcatcgcc tctgagagtc ctccttctgg aattatttgg cttgcaaacg
1579501 ccgtcatgtt gcagacaaaa ggggatttaa gttggcacgg gtcttgttta tgatgaaaat
1579561 taattttaat cacctattca gtgaatggtg attaaaataa aattagaaaa tgaaacagcg

1579621 gctcgcgtaa tgtggcatca ggtggcgtta atgggccaaa attaaccaat
ttcgaaacct
1579681 gaacgtagct aatggtgaag gcttgagttt atagctgcta ttaattagtc
caacaaatag
1579741 ttaacaaaag atggagtccc aaatttcccg tcgttttgag tcgttgctgg
aagttttata
1579801 ggcatgggtg tctttctaga gccgtggaca cgcataatta gcctccgcta
ttttcacaca
1579861 ctcttcgggt gtttacatca cttgcctcgg ggccaaatca catatttcaa
caataacttt
1579921 ggaaaaatgg cgcacgatct gtagcctgtg cgttaaaatc agcgagaggt
ggcttttaag
1579981 agtctaaaaa acccaaaaca ataacgttat cataaaatac aaaaatcgag
cctggaaagg
1580041 cgtcttttct acttaaatgc gcgcgactgc aacaaatcgg caaataaagt
acaacacaag
1580101 gttgattcca cttaccggga ttgtccagaa agagggtgga aggggggaaa
aagtcaagcg
1580161 ttagcgcaca aatcaacgtg aacgatagta tttagcttat gaaaatatca
gtcttcagtg
1580221 gtcgaggctg ccgagcggct tgtgtttaca gtggaagaga gtcttggaga
cggtgaatgc
1580281 cttggctctc tgcgcaccgc tctgcagctc cttcccgggg tattgcgcgt
tgaaatgtcc
1580341 acacagtcgt ttgtcaaacc cagcagtccc tcacttccca cccccattta
tgccaacaaa
1580401 cataactgtt gccacggttt catttattaa atcttttttt tttcacccac
tattttttaa
1580461 gacccccgct atttctgtaa ttgaccctga gcgactctgc tggctatttt
gtttgttggg
1580521 tagggcggga gtttattgac cctccaacgg tagccgcaac ccatctccct
gagcgcgtgt
1580581 cttctttaat cctaacctgt gaataattaa tttcgaccat caaaagtgtt
acgaagccat
1580641 aaatggagct tttatgagag tctgtcaatg cggccacggt gccatcttag
tactgatgga
1580701 gctaaatctt aaaggggatg cgtggcctgc ttctgttttg tgcgtaaaag
tgcccaagac
1580761 tccaaacagg gctccactgc aatcgatgac cgcggctttg ctcctcctct
aaacttcaat
1580821 ctccttacat gattgatgat cttttggggg gtggtggtgg agggtggagg
gggctttgat
1580881 aattgactac tccgaggtga ggcgcggatt tctatctgaa cagccgattg
gacgaaagaa
1580941 atatacaaaa ttacttcctc cacaaaagct tttttcgcct ccaggaaaag
ttcattctga
1581001 gctcgcaggg gtgcggtgtg tggtttaatt tgctacaaaa aaattaaaaa
aaaaagaacg
1581061 agagagagac tggagcaata attagctgcg agattaggag gcattactga
catattaggc
1581121 acaccagctt ataaccacaa aacagataac aagtaagcgt catgctcata
tattaaagtc
1581181 agtattcatg agatatcctg ataatttcag ctcgggacaa tcctttgttt
aacttacgcg

1581241 ccggctgcaa ttttacgcat tcgctgttta tccgtgcgta aaagtttatg tgcgtcattt
1581301 tgtgttaaaa catgattcgg tgtcgtgtgt tatgtgatga atggccgcca aagtgtgcgc
1581361 gctgcttcaa accttcctca cacactcttc tctgctcctc tttctttaca ggactcttct
1581421 ctgcgaggcg gaattggggt gggagttgga gctgtgctgc tttggctttt ttaccccag
1581481 tgtggagttg aagccgtccc cgttcctcgc acacactctg catatcacaa gatttggtct
1581541 ctcctctctc ttttctggag ctgctatagg actggctatg gccaccgcta cttccgggat
1581601 ccgtcatttc attcgcccac cgctcagcgc tgcgaactga ctctgtttct ataagcaagc
1581661 tagcagccaa cctgccaaca ctcgctgaca ctcactcatc tcagtttcgc cagatagacg
1581721 aaatacctac ggggggaaga aaggagagat ccgaatcgca atcgcaattt ctgctgcctc
1581781 ttttttctcc attttttaaa agaaagagtg ataataaaac aagatagcga tctggaagct
1581841 ttaaaggaaa cgcggacttg ccggatgaaa acggactaaa aagattcgtc cttctctcga
1581901 acatcatcac caaccgtcat tcattcatta ccttgacaac ctctggcttt gattgacagc
1581961 tggagtggca aaaagccatg agacacgaca gtttagttac atgcaggttg ttgatgggcc
1582021 gattgtaacc ctcactttgg cgcatttttt cttcttttgc cgaggaaaga ggggaaacga
1582081 gactaattga gaaaaaaaac tccccgatgc aacggtctca actgagccac actggcgaat
1582141 aggagaattc gcaaagttgg ttgaaagctg actttcttac cgggactggg atattaaaca
1582201 cacgacagat ccaggagttt attggagcgc agcctgatgg cgcaaagggt aggttttaaa
1582261 tctcctacac ttacctgttc agatccacag ggaggccctg ctatcgataa cctctcgtcg
1582321 gctggtggca gatttacaaa gtattcctat ccaagccgcc gtannnnnnn nnnnnnnnnn
1582381 nnnnnnnnnn nnnnnnnnnn nnnnnnnnnn nnnnnnnnnn nnnnnnnnnn nnnnnnnnnn
1582441 nnnnnnnnnn nnnnnnnnnn nnnnnnnnnn nnnnnnnnnn nnnnnnnnnn nnnnnnnnnn
1582501 nnnnnnnnnn nnnnnnnnnn nnnnnnnnnn nnnnnnnnnn nnnnnnnnnn nnnnnnnnnn
1582561 nnnnnnnnnn nnnnnnnnnn nnnnnnnnnn nnnnnnnnnn nnnnnnnnnn nnnnnnnnnn
1582621 nnnnnnnnnn nnnnnnnnnn nnnnnnnnnn nnnnnnnnnn nnnnnnnnnn nnnnnnnnnn
1582681 nnnnnnnnnn nnnnnnnnnn nnnnnnnnnn nnnncgcggg gcagtttttt ccacgggaga
1582741 ctccaaacta attcgctctt ttccttcgca ccaaactttc ctccgcggag caacccgaat
1582801 tataatgttt ctggtaatgg caaaccatta aacacatttc atatgtggca cttttatgcg

1582861 tcggcgctgc gcgttttcag cgcaacagct tatttgtttt tccgctgcgt aggttcagaa
1582921 tgagacggcg tgctttgtgc tccttacaca cttgttttgt tttcttcttt cttatttttt
1582981 ttttttttta taagttaccg ggcagctcgt gttgactttt gtttgatttc attcccagta
1583041 cgatgagctg gcccattacg gtgggatgga cggagttacg gcgtcgatgt acggggaccc
1583101 gcatgctccc cggccgcttc cccaggtcca ccatctgaac cacggaccgc cgctgcactc
1583161 cagccagcac tacggcaccc acgctcccca tccgaacgtt atgcccacca gcatgggctc
1583221 tgctgtcaac gacgttttaa agcgggataa agatgcaatt tacgggtaag ggtcgcccga
1583281 gccgaagagc ctacacctaa gttttttaat ttttttttta gatttcctat tttactccag
1583341 tttggtttgc agaagtttct atccggccac taactgggag tcgtgctttg tgccggagcg
1583401 ggcgtcattt tttggtcccc attgtttggt caggggagtt ctgttgatgt gtaattttag
1583461 acgcattgta gaagtaggac taagccttga gcctgaatta caaactataa aataatgtgg
1583521 agtctcgctg cgcgcattcc gagcccagag gtcacagtct gcacgaaaac aaactaaagg
1583581 gcgagttcca aatataaacc aaaaaaggga aaattaaacc gtataaagtt tgcgttaatt
1583641 ataattttca tcagggcgac gttttggaat agtccggcag tgttgtggct gcccataaaa
1583701 ctcatctggt gttttctcat gctgcctgga cacaaaatcg cgttaaaatc tgaacgtgta
1583761 attatatttt atatcaggtt attcttaaag cgcgtaagag acgccacaga gacaatggca
1583821 cgactttgaa aagggtcttc cgaaacctgc ccatttcata gaaagagtta taatcccagt
1583881 gtgtgtcatt ataatctcgg gtgttgtgcg gagcannnnn nnnnnnnnnn nnnnnnnnnn
1583941 nnnnnnnnnn nnnnnnnnnn nnnnnnnnnn nnnnnnnnnn nnnnnnnnnn nnnnnnnnnn
1584001 nnnnnnnnnn nnnnnnnnnn nnnnnnnnnn nnnnnnnnnn nnnnnnnnnn nnnnnnnnnn
1584061 nnnnnnnnnn nnnnnnnnnn nnnnnnnnnn nnnnnnnnnn nnnnnnnnnn nnnnnnnnnn
1584121 nnnnnnnnnn nnnnnnnnnn nnnnnnnnnn nnnnnnnnnn nnnnnnnnnn nnnnnnnnnn
1584181 nnnnnnnnnn nnnnnnnnnn nnnnnnnnnn nnnnnnnnnn nnnnnnnnnn nnnnnnnnnn
1584241 nnnnnnnnnn nnnnnnnnnn nnnnnnnnnn nnnnnnnnnn nnnnnnnnnn nnnnnnnnnn
1584301 nnnnnnnnnn nnnnnnnnnn nnnnnnnnnn nnnnnnnnnn nnnnnnnnnn nnnnnnnnnn
1584361 nnnnnnnnnn nnnnnnnnnn nnnnnnnnnn nnnnnnnnnn nnnnnnnnnn nnnnnnnnnn
1584421 nnnnnnnnnn nnnnnnnnnn nnnnnnnnnn nnnnnnnnnn nnnnnnnnnn nnnnnnnnnn

1584481 nnnnnnnnnn nnnnnnnnnn nnnnnnnnnn nnnnnnnnnn nnnnnnggac atcgcagtct
1584541 ttgccaaaca ggttattctg aacgcaaatg tcacgcagat atctgggact gttgcggcgg
1584601 atgcagcctc tgctttaaaa gtttctgttt ctagcagcag aggagagaga gagagagaga
1584661 gagagtgtgt gtgtgtgtgt gtgtttcaaa cgtcccccat cctgcaacat attagaatgt
1584721 gaattgatgc acaattttat tttgtgccaa actaacggtg acgtttgcgc tattttaggt
1584781 ccgagcagaa aaacctttat tttcatcaaa tccagagctg gacaatttgg taagccacac
1584841 tctgacatct ctgtcttcat aattgccgcg acgctgcggt tgcacccgtc cttgtttcag
1584901 cggcgctcac gattgtgcgc aacattaggc ggcattgaag tgaacgtatg atttaaatgt
1584961 cagatccggt ttgcatgtga agctgcgcgg ccacagtcgc cgctgtcacc ggtcaaactt
1585021 tttttattta attcatcagg aatgaaggac tgtgcaaatg ccaacttttg aaataattaa
1585081 cgctgagtgg acagcgtgta aaatatttaa taaaatacaa caaatattaa ttagcccttt
1585141 ttgactctct tgcagatgat acaagccata caagtattac ggtttcacct tttggaatta
1585201 gaaaaggtat gtatgtatga ctctatattt gtgtgtgtgt gtacatatat atcaacataa
1585261 ttaatgaatt atgcaaattt atgtagccca ccacgcgtat aaaataagtt agacctttgg
1585321 gatgacggtt ctgattgtta tcgatcagca gatatagttt tacctgttca ttacacccag
1585381 ctgacggcgt gttgcttttt gctttttgtc gttcgacttg ttgctcagtc tcaggagaat
1585441 aataagcaca gcaacaattt gactattgtt tcctcgccgc cgataatgcc cccttnnnnn
1585501 nnnnnnnnnn nnnnnnnnnn nnnnnnnnnn nnnnnnnnnn nnnnntttat gccaacaaac
1585561 ataactgttg ccacggtttc atttattaaa tctttttttt ttcacccact atttttttaag
1585621 acccccgcta tttctgtaat tgaccctgag cgactctgct ggctattttg tttgttgggt
1585681 agggcgggag tttattgacc ctccaacggt agccgcaacc catctccctg agcgcgtgtc
1585741 ttctttaatc ctaacctgtg aataattaat ttcgaccatc aaaagtgtta cgaagccata
1585801 aatggagctt ttatgagagt ctgtcaatgc ggccacggtg ccatcttagt actgatggag
1585861 ctaaatctta aaggggatgc gtggcctgct tctgttttgt gcgtaaaagt gcccaagact
1585921 ccaaacaggg ctccactgca atcgatgacc gcggctttgc tcctcctcta aacttcaatc
1585981 tccttacatg attgatgatc ttttgggggg tggtggtgga gggtggaggg ggctttgata
1586041 attgactact ccgaggtgag gcgcggattt ctatctgaac agccgattgg acgaaagaaa

1586101 tatacaaaat tacttcctcc acaaaagctt ttttcgcctc caggaaaagt tcattctgag
1586161 ctcgcagggg tgcggtgtgt ggtttaattt gctacaaaaa aattaaaaaa aaaagaacga
1586221 gagagagact ggagcaataa ttagctgcga gattaggagg cattactgac atattaggca
1586281 caccagctta taaccacaaa acagataaca agtaagcgtc atgctcatat attaaagtca
1586341 gtattcatga gatatcctga taatttcagc tcgggacaat cctttgttta acttacgcgc
1586401 cggctgcaat tttacgcatt cgctgtttat ccgtgcgtaa aagtttatgt gcgtcatttt
1586461 gtgttaaaac atgattcggt gtcgtgtgtt atgtgatgaa tggccgccaa agtgtgcgcg
1586521 ctgcttcaaa ccttcctcac acactcttct ctgctcctct ttctttacag gactcttctc
1586581 tgcgaggcgg aattggggtg ggagttggag ctgtgctgct ttggcttttt tacccccagt
1586641 gtggagttga agccgtcccc gttcctcgca cacactctgc atatcacaag atttggtctc
1586701 tcctctctct tttctggagc tgctatagga ctggctatgg ccaccgctac ttccgggatc
1586761 cgtcatttca ttcgcccacc gctcagcgct gcgaactgac tctgtttcta taagcaagct
1586821 agcagccaac ctgccaacac tcgctgacac tcactcatct cagtttcgcc agatagacga
1586881 aatacctacg gggggaagaa aggagagatc cgaatcgcaa tcgcaatttc tgctgcctct
1586941 tttttctcca ttttttaaaa gaaagagtga taataaaaca agatagcgat ctggaagctt
1587001 taaaggaaac gcggacttgc cggatgaaaa cggactaaaa agattcgtcc ttctctcgaa
1587061 catcatcacc aaccgtcatt cattcattac cttgacaacc tctggctttg attgacagct
1587121 ggagtggcaa aaagccatga gacacgacag tttagttaca tgcaggttgt tgatgggccg
1587181 attgtaaccc tcactttggc gcatttttttc ttcttttgcc gaggaaagag gggaaacgag
1587241 actaattgag aaaaaaaact ccccgatgca acggtctcaa ctgagccaca ctggcgaata
1587301 ggagaattcg caaagttggt tgaaagctga ctttcttacc gggactggga tattaaacac
1587361 acgacagatc caggagttta ttggagcgca gcctgatggc gcaaagggta ggttttaaat
1587421 ctcctacact tacctgttca gatccacagg gaggccctgc tatcgataac ctctcgtcgg
1587481 ctggtggcag atttacaaag tattcctatc aaagccgccg tatttgacat ttttgcagcg
1587541 cccctgctct tcagctgaag tgacagccac tgcttttgcc gacagcctcc accagtcgcg
1587601 gggcagtttt ttccacggga gactccaaac taattcgctc ttttccttcg caccaaactt
1587661 tcctccgcgg agcaacccga attataatgt ttctggtaat ggcaaaccat taaacacatt

1587721 tcatatgtgg cacttttatg cgtcggcgct gcgcgttttc agcgcaacag cttatttgtt
1587781 tttccgctgc gtaggttcag aatgagacgg cgtgctttgt gctccttaca cacttgtttt
1587841 gttttcttct ttcttatttt tttttttttt tataagttac cgggcagctc gtgttgactt
1587901 ttgtttgatt tcattcccag tacgatgagc tggcccatta cggtgggatg gacggagtta
1587961 cggcgtcgat gtacggggac ccgcatgctc cccggccgct tccccaggtc caccatctga
1588021 accacggacc gccgctgcac tccagccagc actacggcac ccacgctccc catccgaacg
1588081 ttatgcccac cagcatgggc tctgctgtca acgacgtttt aaagcgggat aaagatgcaa
1588141 tttacgggta agggtcgccc gagccgaaga gcctacacct aagtttttta attttttttt
1588201 tagatttcct attttactcc agtttggttt gcagaagttt ctatccggcc actaactggg
1588261 agtcgtgctt tgtgccggag cgggcgtcat tttttggtcc ccattgtttg gtcaggggag
1588321 ttctgttgat gtgtaatttt agacgcattg tagaagtagg actaagcctt gagcctgaat
1588381 tacaaactat aaaataatgt ggagtctcgc tgcgcgcatt ccgagcccag aggtcacagt
1588441 ctgcacgaaa acaaactaaa gggcgagttc caaatataaa ccaaaaaaaa ttaaaccgta
1588501 taaagtttgc gttaattata attttcatca gggcgacgtt ttggaatagt ccggcagtgt
1588561 tgtggctgcc cataaaactc atctggtgtt ttctcatgct gcctggacac aaaaaatcgcg
1588621 ttaaaatctg aacgtgtaat tatattttat atcaggttat tcttaaagcg cgtaagagac
1588681 gccacagaga caatggcacg actttgaaaa gggtcttccg aaacctgccc atttcataga
1588741 aagagttata atcccagtgt gtgtcattat aatctcgggt gttgtgcgga gcaaatcctc
1588801 atctgctctg ctcacgcgtc ccatcaccgt ctttannnnn nnnnnnnnnn nnnnnnnnnn
1588861 nnnnnnnnnn nnnnnnnnnn nnnnnnnnnn nnnnnnnnnn nnnnnnnnnn nnnnnnnnnn
1588921 nnnnnnnnnn nnnnnnnnnn nnnnnnnnnn nnnnnnnnnn nnnnnnnnnn nnnnnnnnnn
1588981 nnnnnnnnnn nnnnnnnnnn nnnnnnnnnn nnnnnnnnnn nnnnnnnnnn nnnnnnnnnn
1589041 nnnnnnnnnn nnnnnnnnnn nnnnnnnnnn nnnnnnnnnn nnnnnnnnnn nnnnnnnnnn
1589101 nnnnnnnnnn nnnnnnnnnn nnnnnnnnnn nnnnnnnnnn nnnnatcacg cgtttaaatt
1589161 ttattttcct gcatgactcc tggcgcagct tggataccta gtttttttatt cacctcctca
1589221 cgcttttgca tcgttatgat gtgcaatgta atgcatttct gtaaaaatat tttcagaaca
1589281 agtttatttg attttaaact gtaaacgcgc tgcctcaatt tgccccagtg ttgttttaag

1589341 ttgcgttcac gtcccagccg tttttatttt gtagcctact gcaagaaatg ccagcctcaa
1589401 caagtcgatt aatatcagac cgggacttaa tcccttaaat atcccaaagc taggaaataa
1589461 aatattccag atgcatttat tcatgctgca aactgaaaac tttcatcccg gctcttgttt
1589521 gcattatctg actgtccttt tttggggggg ggttgtttta acaaggtggc tgcttgtttt
1589581 aataaactcg gagaaaacag tttaaataaa acaaaatgga ttaaaataat tgatgttgta
1589641 ttcgcagtca ggaacgtgtg gggctggtca gagacgcagg gtgcatgcgg acattttttg
1589701 cagtggagcc ggtgagcagc ctgtgctcag actgtgtttc ccttcttgca gtcacccatt
1589761 attcccactg ctcgcgctgg tttttgagaa gtgtgagttg gcgacgtgta ctccgaggga
1589821 gcccggtgtg gcaggaggcg acgtctgctc ctcagactcc ttcaacgagg acatcgcagt
1589881 ctttgccaaa caggttattc tgaacgcaaa tgtcacgcag atatctggga ctgttgcggc
1589941 ggatgcagcc tctgctttaa aagtttctgt ttctagcagc agaggagaga gagagagaga
1590001 gagagagagt gtgtgtgtgt gtgtgtgtgt gtttcaaacg tcccccatcc tgcaacatat
1590061 tagaatgtga attgatgcac aattttattt tgtgccaaac taacggtgac gtttgcgcta
1590121 ttttaggtcc gagcagaaaa acctttattt tcatcaaatc cagagctgga caatttggta
1590181 agccacactc tgacatctct gtcttcataa ttgccgcgac gctgcggttg cacccgtcct
1590241 tgtttcagcg gcgctcacga ttgtgcgcaa cattaggcgg cattgaagtg aacgtatgat
1590301 ttaaatgtca gatccggttt gcatgtgaag ctgcgcggcc acagtcgccg ctgtcaccgg
1590361 tcaaactttt tttatttaat tcatcaggaa tgaaggactg tgcaaatgcc aacttttgaa
1590421 ataattaacg ctgagtggac agcgtgtaaa atatttaata aaatacaaca aatattaatt
1590481 agcccttttt gactctcttg cagatgatac aagccataca agtattacgg tttcaccttt
1590541 tggaattaga aaaggtatgt atgtatgact ctatatttgt gtgtgtgtgt acatatatat
1590601 caacataatt aatgaattat gcaaatttat gtagcccacc acgcgtataa aataagttag
1590661 acctttggga tgacggttct gattgttatc gatcagcaga tatagtttta cctgttcatt
1590721 acacccagct gacggcgtgt tgctttttgc tttttgtcgt tcgacttgtt gctcagtctc
1590781 aggagaataa taagcacagc aacaatttga ctattgtttc ctcgccgccg ataatgcccc
1590841 cttaattgga gttattcttg gactggctga aacgtttcct catcctgaga aaaaaaaggt
1590901 gtttttcctc cctctgtttt tctaggtgca cgagctctgc gacaattttt gccaccggta

1590961 catcagctgt ttgaaaggaa aaatgccaat agatctagta atcgacgaac gggatggtag
1591021 ctcgaaatcg gatcacgaag aactctcggg atcttcgacc aacctcgcag atcacgtaag
1591081 tgaccgagct tctccttcgt gtttgtgctg gtttccaagc gcctgcgtgc gcaaacgcat
1591141 ttcccagtga ataatcggtc cgtataaaaa tgagattaga cgccaagttc tctgctgcca
1591201 ctgtgctcgg tgcgtgaccc tgttgtgcca tcggtaaata gagcaggctg tgttctgcaa
1591261 ggaaaatgtg ccgatcgatg ttctgaggtc aaaagctaga tgtgatacaa tagacagagt
1591321 tattgtttta gtcttttgat taggggcagt catgtgtgca taatgcgtcc cagcgcgcgc
1591381 gtgtgtctgc gtgtgtgtgc atgcgtgtgt gtgtgtgtgt gcgcgcgcgc gtcactgctg
1591441 ccgtgcatta cggagagttg cacgctggag gaaagaaaaa agtcaggcaa ttatctgtta
1591501 cgcgggttta ccgttaaagc agggtatcag cgctaagctt tagctttagg ctaatattta
1591561 aaaagaaaaa ggctttatat ttggggctgt gggagctgga tatggcctgt gcgtaaagcg
1591621 gagagggttt tctttttttt ttttttaccc gcagaggtgg agaaagtggt agcccgttga
1591681 aatagatctc acagctgaag gcttatgcct tcaatggcaa gttcacatgg cgcaggatgg
1591741 cgcgtctgga ggccttccat tgcccctgat gttaatgtgt ttaattgttt cctattgatt
1591801 gctgcgtggt tccctctgca cttcccctct ccctcgttct ccattttatg actggaggct
1591861 ccataatgct gaattcaatt atatgcacct gaagtacagg agctttaaat ttgaagatca
1591921 attctttggc ttaaatcgtg catttttagt gtaattaaac acacacacgc acacacacac
1591981 acacacacac acactcttgt tcagctgaca gtgtatcata aagttcatat aggtgaatta
1592041 ttgccttgtg cgcatctgcg ataaaactaa tgttttggtg gaggggaggg gggtaacaaa
1592101 gtgactagtt aatatctcag ctcaaggcca agcctattag gaattaaagc agatttaagt
1592161 gttatgcaaa gtcaccaccc tgcatctgtt gggtctggct ataggcctga ttccctctga
1592221 aaggcgtttc cggagccacg caccacaccg cacaggccca gtccaacacc acgttacgga
1592281 tgttgcaccg agaaaacggt cagaaaagac aatattgcgc gcgtccgagc cgagtgtaga
1592341 aatgaggcat gctgttgttt atattgtaga gattagataa actggcctgt ttagtttcat
1592401 ggcagatggt tgatactcaa gccaatttcc accggcgctg cgctgctcag acaccagcct
1592461 ttgtcatcag gacacttgtg ggaaatattc gcgataatct ggaaaataag gagatgccag
1592521 taaagtgagt aaacacaggg ggcaaagcat ttatcctgga gtcagctggc ctgaccactt

1592581 catcggctat ttaggctttg aaagatttag tcatgtgaac tacatctaac cgctgagttg
1592641 atttgacaaa aaagtccccc ccccccgcac acacacacac acacacacac acacacacac
1592701 acacacacag acacacacag acagagacac gcgcgcatgc acacacactt gtttacttct
1592761 ggagctctga gtcagctcag tgcctcatgt catggagacg ttaatgaaga aatgctgtat
1592821 ttcttgtgct tcacgacgcc ttagtgaaaa tagttcagag ttaatttgtt ggatgttcag
1592881 ctaaaagctt tgaagcgtgt tcatttcctc aactgcagcg ttcatacctc attaaagagg
1592941 cctttcctgc ccaatatgtg tttggaatat tagaaaaaag gtgaaaaaga accccagtcc
1593001 tacttgtttt gacttgttta ttttatttat tcatttgtgt ttgcctctgt tttattattc
1593061 atttctgtcc ttgcttctag tggaaggtgg ttctgatagt taggtatgct aatttattta
1593121 gttggaatct ctcacatcca tttttctttg ctctgatatg catttttaat gtatagcccg
1593181 gggctacatc tcatttggtg aaagttggtt tttctggaga tgatgaggct cttcactgtg
1593241 aaggaggcat gtttttatgc atgtatgtat gtatgtaatg tatgcatatg cacaaatata
1593301 tggtgttggt gacattctct atggttacta cctgacagca gagcactgtg aattcttatt
1593361 tacacctaaa tgcagcactg tcttacggat gattggatta ttatagaaag ggagcagcgc
1593421 gagtccttca ttctctcatt ggctcttcct accccaccgt ccttcctcag ataaacctca
1593481 tctcaattaa aagatttaga aaaaaaaatt ctgtcttaca cataaagcat gatggaagga
1593541 gggagaaaaa aaccacgcag cgctgtaaaa tcgtaaatgg ttggctagtg tgtgacagcg
1593601 gtttcctctc ttgtctttta attaatgaat aaaatttctg tctctcctct gctacatcca
1593661 attagggagg caaatagaat taacagtgtc tgaatattat ctgttctaat gaattagcct
1593721 ttgtgtctat tgtggagctc cttcactgaa gctcacattt taaagaaagc cttttcattt
1593781 cctcatatgc atttggcctc tgaaagcgtc tctggattac cgcgtagttt tcagatttgt
1593841 tgcggagaca ttctgtagga tttgcaggtc ccgctacttt gttttagcga gagctttcct
1593901 tggggagtga tagcggttcc cgtagaggtt gtggtgaagc ttatctttac tctgcatggt
1593961 tggaaaggag ggagaaaagc tccctttgtt tacatttaag tctctggtgc agactgcgag
1594021 ccattggctg gacgtggtga tgtcacgggg tcaggagagc cggccaacgg ctcatctcca
1594081 cctataagtg gagatggttg ctccgcaggt aactatgggc acagcgtagg cgcgcttttt
1594141 tccgccggta ttaccgcgag cctgcgggct gggccggcct gttgaactgc tggtgtttta

1594201 cttcaaacaa gtttggctct tcccgcgcag ttttagctgc gatccgttcc acgcaattat
1594261 ttcgtgacag cggggacatc tggccgagca tttcctgttt tactggcact caggaggcac
1594321 tgttggcagc catgtctaat catcactacc gtggtgagag agaggcgaag gccacttgtg
1594381 gagtcctccc gacgaccccc tccacaatca ccaccccttc ccttaactgc acatatgctc
1594441 taaatgactg caataaaaca cagttgctgt acagtcatct caggatcatt acatgcatgt
1594501 ttgctgacat gtattcaaac attgtgtcgt tagattcggt acggacgtac tctgagctgc
1594561 cactgtagtt acacccggca gcctgctgca gcaattccac ttattttctc acgcaagaca
1594621 catttttgtt attaatacga tgcatggtta atattggtca tgcaaaatag cattaggttc
1594681 taattaaata cagtaaatat cctcattaga gtgctcgtta catggatttc ttttacagcg
1594741 taggtggtta ctgtctttat tttccagcct gtattttagg atttgccgca aggggaagac
1594801 atcttattat ttctgtatgt catgtcttat tttgaaaggg ctctgtcatt ctcctgagcg
1594861 gcgaaaactg tggatcctgc cccttcattt gtcatttaat ctcgtctttg tgaaaacaca
1594921 cgtttgcgat tgcatctaat cttttacaaa agtgcttttg cccgagagaa agttttcaaa
1594981 ttgaattgca atcagcggat gccttttctc tgccgagaca cgccgttgcg aattattagc
1595041 tgctaaattt ggcacatgtt acattctgga taattgaggg tgttgtaaat gctcagctgt
1595101 tcaatagagt tgcacctact cttcactgga gcgggcgtgt ataattacaa gctcatccca
1595161 acaaaaagag ggggagttgt ttttttctct ctgtctgttt actccaagtg gcacgatgta
1595221 ccttgaaatg tcaaccggct tcatgattta tggtccactg tacttctggc gtgtcagcat
1595281 ttccgtacat gttgagcaca ggatccctga tgctgctctt cgctgtacaa cacgcacggg
1595341 gttggccctc agcagcctgc tttacgccgc agatttctcc acttccctgc aggttccagg
1595401 ggaatttcag gcaaattcaa tgatgctgct aaattcaatt ttatttgaaa ttcctaggga
1595461 tgtggtgtgg agtgaagtga gattgtgcca ggcaggtgtt tgtaaatgag aaacagggga
1595521 caggagcaga ggagccggca gaattctcct gctctcctgt ggcctggctc caccacgagc
1595581 cttccttccc atattgtctc ctgcttttat attaattaca agttgtatta tcagctgcca
1595641 gttgctaacc ccggcaaata gaaaaggctg cccccagaga ccgtgcttct tttgtatctt
1595701 attctcaatc tagtcctatt gtgtgcgtgt ctgtatgagt gtgtgtttgc ttgctaccgt
1595761 tcccttcctc cttctttctc ctccctcctc ctcctcctcc tcctcatggt aacactgctc

1595821 tttaacacaa ttagtgaaga gcatggaccg aaccatgttt gactcctaat tatacgcagc
1595881 ttcagggaat agtcacatgt ctagaggatc tttccttttt ccttttgcat tttcttttgc
1595941 ttcgcgtgtg tgtttctttt tgcgagactg tttgggcgac acgatttttt tttttttatt
1596001 atttccaact tgtttgaact ttatcagacc cacgagggtt gagacaaaat gcttgcgttg
1596061 tgcggtgcac cctttgagaa gcacactaaa ttattcatgc agaggaaaga ctgttccttt
1596121 tgtcaccttg ttgagcttgg aaagttaacg cttttgtggt ggtgtttgtt gtggtgtttt
1596181 gcagacggga ttataaatga gacatgactt acagatatta aaggactgct gtgtgcctga
1596241 tcaaagatat ttgctgcgtg tttactgcgc cgaggaccca ccgggtgatt gttattaggt
1596301 catataagcc gttgtccttt tccctctggt aaaataacta acatagtctt ttccatttcc
1596361 ctgcatacag gcctattaaa cctacagtag caaaatataa ctccctttaa caccattagc
1596421 cgtggcgtct ttgaatttaa tttagtgcca gcaccattaa aaatgacaca tcactctgca
1596481 atgatagcta aattaagtcc tatcgattca gctgttgcat taaaatcggg ccttggtcgt
1596541 tgctggcaac cgtttccagg gatgagagac agagagggag cgtacatgtg aaccagagag
1596601 caacaggaag gatttttcag gtttgttttg catcttgctg gcattgactg cctcactgtg
1596661 tgcaagtaga acccggtcat gttctcctct gggagcagca gccttttctg cctaaataca
1596721 ttatgtacat tggaagtcta ggactttcct gtccactttt ctgtttgtgc tattttacaa
1596781 atagataaat taatctgctt tgggttgccg cttggtgact ttacttagtg aacactgttg
1596841 ggaatagaaa agtgctacag caacagatgt tgttctcatg aattagagtc cagcatgtgc
1596901 tgatcagttg ttgagagtta acgcatcaca atcggtggcc tccctcagag cagggacact
1596961 tttccctcca ctcaccggcc tgggattaaa cccacgcatg gcctattgtt ccccttgtct
1597021 tctctcccct cttctggatt attctccagg ttctttatct gattgccatc cggcaaacag
1597081 ctgacgctga gagcgccttg agtggagcgg agggtctgaa gggccttgtg tgtttctgtc
1597141 tgctgttttc agcctcaagc tgtttgagaa aacagcccct ttccaaaaaa acttagcaga
1597201 taaaagggcc tcgctcacag gcatcacggc agcctcgcct ccgcagcagt acaggcaaca
1597261 gcaaatcagg actgtgcacc caggggagct gagcggctgc ttgtcttata gcctctgttt
1597321 ctctgcaaac ctccgctggt ctccaggcct tctcctgtct gtcctccacc agtacagctc
1597381 actctgactg ggacaccaac acaggcaggc aggcaggcaa gcaggcaggc aggcaggcag

1597441 gcaggcagag gggcaggcag gcacaggggc aggcaggcag gcaggcaggc aggcaggcgg
1597501 atcttctctc tggcagaagt tgaggaaaag aaagtcatca gaaatggtaa aagcgttctc
1597561 tttcttggaa tattctgtgg cacgtaaatt ttccttctgc tttcgtttgt atgtatatgt
1597621 gtttatatgt gtttgaggga gagagagaga gagaacggga actgagaagt gggttgaggt
1597681 ttgtgggaag ccttggtgca ggctcgtgtt tgtttggctt gggctccact caaggggccg
1597741 cgttcctacg gccccagacg actctgggct ctcgttggcc aacattttgt catcagactc
1597801 aggtgacagg ttaaattggg aaagactggc cccaagttcc tgtgagccac aaaagctgta
1597861 tcaaccttgt gtgagaggat gtcttaggcg gattgagtct gcggcagtga cgcgatcgct
1597921 ctgttttacc agcggctgaa tggcagagga aattccacct ctagcctgcc ttcatgtggc
1597981 gctgataaag cccttcctgt acagtagaaa atcctgtccc cccctgatca gggtaaacct
1598041 tgagagacca ggaaagaggg gaggagatgg agagaaaaag agcccagaag aagggaaggg
1598101 aagggaagaa aaaagagaaa aacaaatcct taaagtttcc tatttgttta tggacctctc
1598161 tttgtagctt gccagcagat atttaacact ttgaatcagc atggccttgt atatacaaaa
1598221 gtaagtgcat gtgcatttgt gtttatactt cttgctctat tatgtcatta gcatgtgtgc
1598281 acgtaagtgt attcctgcag gtctgagagg gtgtgtgcac atttttggca gattagaggg
1598341 agactcactg tcatatctta ttttcatttc tgacagtggc agtgattaat ggtggctgtg
1598401 gcgtgtttgg acttcttacc ttgatatttc tcacttgcca cggaagtctc aattggtcat
1598461 ggtataaatg aactccgctg ggccctgcca tatgtgttta gacaattact cacgggcgct
1598521 ccacacaaaa tcaaacggca aattaacggc ggtctgcttt gctctcgtgc attgatgaaa
1598581 cactttgatt agagtgtttt gacttattgc cagctgctgt cgtgcatcgc tttccttcta
1598641 ccccgggttt gattttggcg gcgtcctttg ttgctcacca gcttggaaag cagcggcagc
1598701 actcgttgcc tttgccaaga taatggaagc aggccaggga tcctcactca gtcaggcgac
1598761 gcttacagat ggcactctca cttttccagc atactcatgt ggtctttctc tttttcctcc
1598821 atccctacct tccccccccc ccccctttcct gctctctccc gttcctggtc tgcggtttgg
1598881 tcgggaatgt ggatggatgc acggacgaat ggatggatcc cacgctcgct ttctctcctc
1598941 tcaaaacctc agaacccagc ttcctggaga gaccacgatg acgccacctc cacacactca
1599001 gccggcacac cggggccctc cagcgggga cacgcttcac agagcggcga caacagcagt

1599061 gagcaaggtg agtgctacat agaaattggc accaactgtc cacacacaca
cacacacgac
1599121 cacaggagca ctccatatat tcgtgcgctc aagcgtgaac acataaatgc
acgtagatgc
1599181 gggcgcagat ccatgcagaa catattaatc atcacggttg aatagagttg
ccaacacttt
1599241 aaccccatct caagtgtgat caacagtgaa tggatggcac atccttacac
agacgtccca
1599301 gtccttcctc tcacgcgctg gattatcctg ctttttgagt ggcatccatg
tcaacacaca
1599361 gtcacaccta aaaatgaaca ttctcagccc ccgttgcact tgaaagcaag
cgtcagtgcc
1599421 tgtaactctc cttccaaaat gttgctattt acggagctga acaaaagctg
gtgattaata
1599481 gtatattcct gccagttctt gtttgcgttt aaagcatatt aactttgctc
gcacaacggc
1599541 tcctaattat gtctcagcga gccttgtgtt atctcgtaca tgacctgcca
agcagcaata
1599601 atgcatttca aaacaaatgc atttgaattt attacagaca gcgcgagcaa
atcacagccg
1599661 tctgtcttgt gtttttgtca tggtccacat caaatttgac gcgcaaattc
tccaaagcgt
1599721 atgctattta ttgaatttag aggttaggcc aagtatcaat atgttttttt
gggtttggaa
1599781 ttgttcctta aacgttgctc ttgcgtgtcc taggagagca aatgaggagc
tctcggcctc
1599841 cctttgaaca cgccgaggtc tcacacactg tcggtgcagt gcctgtgtgt
ttatgtggac
1599901 ctttgtatgt actgtatccg tatatctacc ctctggcagt gtccatgctt
ttgcatgaaa
1599961 tttcatcaca cagaacccct ctgatagtcg aaagcggctg cgctcactgc
gggaggctgt
1600021 gcgctgcgtt ccttcagatc tgtggttttg ccaataggcg atcagatgga
tcgatgggat
1600081 gagtgtctgt ggtgaagctg tttaaatcat cctctcctct tcagacagaa
actgctccag
1600141 ttttagcctt tgaaacgtta ttgaccacag gccaagggcc acggcagacc
tctgtggtcg
1600201 gccatacgag gaccagctgg atcacaggct gtttctcagg caggttgatt
attgctaggc
1600261 ctctgctcag caatgttaac acaaaataat caactgatgt gcaatatttt
gcaaccattt
1600321 ttcctgaatg gtggccagag cagggtcagt tcagtaggac aaaggactat
ttagtgtact
1600381 gttttcacaa taacttatta gaaaaaaact gtgtggcagt agcagaagat
gggaggatac
1600441 tttgcagagc aagtgctgaa gatggagact gcaagaacaa cagaggggag
gggccaccag
1600501 agaaagaaat gcactgccta ttgattatct tacacacata agtacaagtg
acctgtaatc
1600561 cacagagcac ttatcgactc ctctccccac cgtatccagt aacagcagat
caatacagac
1600621 caaagacagt tacggtacag caccgcgcac cgggccaaga tgggccaaga
ctagatcagg

1600681 gcccgagtct gacctgaact gaagagcgct aaatggtaca agcagtgcag
ccttttattt
1600741 tgaaagccga gcaagtagcg tggaataaag gcagcgcttt gaaaggaagc
agaggaaggg
1600801 ctattgtggt gctgaggtga aagaaggtga gaaagtaggc agagtgaggg
aaaaaaagag
1600861 aaaagacaga gggcagctga tttaatggtc taatgaggcc gtcctccagc
cagttgtttt
1600921 gtgtgaggca tactctgacc tttcgctccg ctcgggagcc tcgctgcctc
atttttcaca
1600981 ttctttcaat tacgttaacg cttgaagcgc tcgctggctc ccgactctgg
cccttccatt
1601041 ctgacagagc cttctctccc caacctctcc acctttccct ccacggcact
ccactccaca
1601101 catgaactcg cctccaatct caatatattt ctacataaaa tttgtgctcc
acactttcag
1601161 gtccgcagag gaaatgcgaa atgtgcgctt cacttcagta atggggtctt
taacagcttg
1601221 gcctctgtta aaaggggtga cgctgtggat cagttgagga tagactgaat
agatgaggaa
1601281 gagggagagg gagcagtgag taggtgagac acactttggc ttctgccaag
aaaaacatca
1601341 actttctcct gcccacttcc atgttgatag gtgttttgtt tgtgaccccg
tggcttgaaa
1601401 aaaaaaattc acctcagaat atttaattcg cacaaatccc agtggtgaat
atattacagc
1601461 tacatcattc ctctaaaaag ggtaaaggct tttcccaact gggtgtattg
gcaataaaat
1601521 acagctgaaa aagttttgtt tgtacagtgc acacgcaact tagcttggtc
actgtgagaa
1601581 aaatccactt cctcgatatt taagttgcgt atgaggccct gccaaaacaa
caatcctact
1601641 agtgtgttgc acctctcgtg gaataattaa gcgtcaaaca gtaaaagaga
ggtgaagcgg
1601701 gctcagtagc tgtcagtaaa acatgccagt taaggtttat acatgtgaat
aagggtttca
1601761 gtagaatgca cagatcttta acttttcccg tccccagtct ctaaccaaaa
tcttttttta
1601821 tatatataac tttctcagaa tttcccaccc cccccaaaaa aagaagtatc
agcctgaggg
1601881 caaattttgc actctcactt actgaatctt agcgagtgac tccctatgtt
ctggaaaaat
1601941 aaacaaatac aagcctttaa tgggagacag ttcagaaggt aacccacaac
gcactaagtc
1602001 cattggctcc tcatgcacat cattttgatt caaagccttt tggatgtcct
ccaaacttat
1602061 ctcaaaacat cagagatatc agttgtaggg agttaaagtt gatgaatgta
cgtgccttca
1602121 gatagcactc ctttccaagc ccgcgtctgt aacagtttaa acacccaagc
tatgatggag
1602181 cgctgtgttg aaatcctctg cacatttgga cacggcaaat aaagaggggg
gtcaaatgct
1602241 cccagtcgtg aaccgcgaat ctctcaaccc cgattctgtg tgagcgcgca
tgcctgagtt

1602301 ctattccacg attgtaatta ttagctatgc atgaggacca aatccacgtt gacatcttga
1602361 gtggagagca aagggggctt tcagagcaac tggttgattc cgcactagat ggaatagctc
1602421 aacaaaagag aggagagagc attttgtaca ttttctcagc aattgaacaa tccccacgct
1602481 ctgtggatag acagactccc cagagagctc tagaaaggct gggatggaga ggaggggagg
1602541 aacagaggca gcgctcgatg gtatggtgtg tgcgtgggga gtgctgtgag ctcccatggc
1602601 cctggccagg ggctccactc agcaagggtt tgtgtggctg agctcaacag agcaggaggt
1602661 cactcctggt tggaacccga cgccctgaca gtcccggcat caagttctgt caaatctgac
1602721 attttggctt ttgcacttcc ttcaactaca caaaaacctt aacggaatgc acattggcgt
1602781 caaaacagaa tgtgtgatgg aacccttgca atgtttctgt tgcctttatt ctggctctca
1602841 agtgatcaca gaggcgtgtg tctgtacata acatggacaa tacagtgagt gtggaggcac
1602901 aggtacgtgt gttagctgct tccctctaag agcctgttag agtttaattt agtgttgtaa
1602961 ttaaagctga acactggccc ataaacaaca gataaacaat aaagccgacc ccagccctgc
1603021 tgtttcatgg ccctcgctcc cagctgttga acgctgataa gttgttgttt ttttacgcag
1603081 ctcttgccta atcacagcac atcgcccagg ttacgagcgt gcgcatatgt ggaacgcgta
1603141 ctggggtgtc taactgggtg tctaacattt tagaaagtct aaagaaatac gaagggcaaa
1603201 agcaaataag gagttccaca gcggagcgat cccagcgcgt aagaggacca gtctgccctg
1603261 atggccaatt acttactaag ccaactcagt ggcaatagtg atcacataaa cattaaacaa
1603321 agtgcagcca tgaggtgcat ccgcctcaca atgtacggca attcactgac tggcaaagtg
1603381 taaatgtttc ctcagaaaac aggttaaaga ggcttaatgc acagagcggc gggagtgttt
1603441 ctcaatcagc caacacgggc acttgtagtc acagcgccac gcgttggcct cgccgtcgct
1603501 acgtgtctgc gctgtggacg gagcagcgag gctgtcggat gggttgttgg gaaggggcag
1603561 ggcgattgtt gaaagctgtg gagggggaga aaacagtgta caaaggggat actttcatga
1603621 tcccattagc ataagagagg ctaaattatt catgtggagt gcttaatcta tggggaaaag
1603681 tatagttaat gcacatagtt ttaaaatgga tctctctctg tctctcctct aacaccagat
1603741 tcttgctgga ttcagcattt gttgggcatt tacgggaatg agtgtcactc tccctcacac
1603801 acacactcac gcacacgcgc acaaaaacac agacacacac agacacgcac gggaatgttg
1603861 gtactcttgc ttaagcgctg cagcacttca aactgcttca aggggctctg ctctcaggct

1603921 catcacctct ctcccttttt ccttaagtcc ccttctcatc ttctccctct ctccagctct
1603981 ttccatccat tccttcatcc tccctctcac attgtctccc tcgcactcct ctcctcactc
1604041 accctctccg tcttgctctc ggtaccacag actacctggc ttccatccaa aatgtgatta
1604101 aagagaatga tcagctccct cagctccctc cttccttcct cccttccgct ctttcttccc
1604161 cccctttttt aatgtctctc tgtctttcgt ctgaccctgt gttatgttct tggaaactgt
1604221 ggaaggaaac aaatgaaaac agaaaatgca gacacactgt taggagagga gtagtgccaa
1604281 tggagtcagg ttcatttcaa ggaatgtggc cctctttaca caaggtaacc cccccattct
1604341 atagctctag atgattgttt tggttcttat ctttaaccgt ccccccgagc atgatctggt
1604401 gtgccccagc attggtgtat gttaggtcag cctgtgacca gatttattcc aactgcaaca
1604461 ggcgcagatg tctcttttac tgattttctt ttatttctgt attttttttt ttatccacgc
1604521 agcccttcta tcttgagcat tgattaaaac gcagctagaa caagatgctg ctggagaatt
1604581 gtgattgtac gattgtaaaa cgtttgaact ggactgcaaa caaaggctga gcgacgtgct
1604641 gatgtatctt ttcattatcc aacgagttta cgctcccgtg tccctctgct gattactcct
1604701 gcgaatgtgc cgatgaggac gagtgcacaa aaacactgag agggatttga gcacatgccc
1604761 tgaagggtat atccacgtcc attgatgccg ctgtattttc acgaggacga cagtgcttca
1604821 gttctttaaa gagcaaatgt tttcccagga aattttctgt catgttttag cgggctagcc
1604881 tgaaggggt cagccaagaa gtcaccctta agaggtgata atccaattgc tctagtcctc
1604941 accagatttg ttttcccctt gtctggcagt gtggttgcgt gtgtgtttac agagagtgta
1605001 agcatgcgtg ggtgaatgta acagacagag aatggagatt tggagagggg gggtgggcag
1605061 aggcagcaag atgccaccag tttcttctgt gactcaccct gggccccagt ctaaaccagt
1605121 gagaggttat gggaggctag gtctgtcaaa agcacacctg cagggaacct ctgaaaaccc
1605181 tgactgccag cacagacccg tttaaggtgt gcttagttgg agtttaaccc tgacttcaga
1605241 tgtcctttcc ctctctccct ctttgtgttt gaccaatggg ccttgtgctc ttttactttc
1605301 tgttttcctc ctcccttgtc tctttaatcc cctccacctc ccccaccctc ccagttctcc
1605361 tccccctaat atcccacacc gcatcacagc aaaggtccct cccaaaagac gacatgcggt
1605421 tgagtcgggt ggcttagcga gggcagccat tgcctgttca cttgctgtgt ggttttctcc
1605481 cttgtaaacc agggtttcca cgcgaatgct tacattcata tggaactgag cagagcagct

1605541 ataccctaca ggtagtgctt gaaccgtgct accaccatcc aagagagtgg gaggatgaga
1605601 aagaaagtgt gtttcaatcc gagttcttcc tctccgctcc tgattttcat gcacacatct
1605661 ttcacgttta tgcaaagctt ttgagggcat tatacaagat aagctcattt tctagtctga
1605721 ttcctactct agcacatgct ttaatgactc ttaaagccaa gggctgcttt tttaggcata
1605781 atagcactcc cgcagtctca gttaccgtgt agccagagtg ctttttccac acctgatcac
1605841 ttttgttcgc ccattttatg ggtattgggg agcaggcaga ggtgagcgat ttgacagtag
1605901 ttcctgccat gagccctaat gtttattgga agcaagcgtc taataatttt tgattacccc
1605961 agaggaggta ttagccatgt tagtcagcaa ggataatttc ttatttacat gccccaagca
1606021 gtgcacactc tgaaagaagg ctggggataa atgtgcaaca aaacgaatca aacatgccaa
1606081 accaagatgc tcaattctgg ctggctgcag ggctgaggct gagcgaacct tcattatgca
1606141 tttaacataa ttcatttaga gggttatcaa tttgcagact atagaagaga tggttggaag
1606201 gggaagcaaa acagcatagc aggcacagca aaggaattgg ggaacgcaag atgaaaagga
1606261 gcaagatatt caaggctagc tctctgcttg cattttttttt taaaaaaatg ctccaattac
1606321 acacttttcc atgttttgag ggagaacttg catgttaaac actgcaatct ccaacggtgc
1606381 cgtcacactc cgccccctaa aaatcagaaa ctgatttcct tttaatgaaa gctttttttt
1606441 cagcgcgaga gaggatgtag tttaaatatt acccttgtgt tcatttaggg tccctctttg
1606501 tttgacttct gcctggctta cacaacggct ccttgcatca gtcccccagt gtggattgag
1606561 ggacagttac aacatgtttt catttttctc ccctggcaac atactgattg aagaaaaaga
1606621 cctctaaaag tatctttccc tctgccatca ggcagagctg acggcccctt tagaacaatt
1606681 ggaggaaact tagggagaca gggagcattt aaaagaatga gtgcaccact atcttgcgat
1606741 aaccagagct tttctacatt atctaagcac aaaaccgtcc accttggaaa agtacactca
1606801 gactatggcc cgattcaaaa ataaaacaat gcaaagtgag aggtggaaaa aagggggaaa
1606861 aatgcaacaa agtaagggga aaaccttcat tgtttagtgt gaatgcttac tcccagggat
1606921 gcaatcctta gacaactgag tgtctgcagg agacatctgt gaaggatgac cttgtgaccc
1606981 cggaaagaat gctcttaagg tgttggtggt gtttttgggg gtgtaagggg ggctgcctta
1607041 gccactgcat cacagcctac aggatagccc agcagccaca caacaaacag aaatgctgtt
1607101 gtgagaaaaa aaaaacagga ataaagtggg aaaaaaacac agggcagaga tgaagaagtc

1607161 aagaaaggct gataagattc acagcataca cagacagcct tattgcaccc
ccacagacct
1607221 ggctgtctcc caccgcaacc cccagtcacc ccgccatgag atcttttttcc
tcctttatga
1607281 gtcaaaagga agcctggtcg aaaatgaaag gttacataag ccagaaccca
agacctcaga
1607341 gcgagcgaga ggagcaaggg ccctgctgcc attcaaagca cctgccattg
tgtgggcgtc
1607401 cattcaggcc tattgttgtg cagaaattag tcgtgctccc ttcaggtcca
gaggcatgcg
1607461 gcggacccccc ttgttgttcg gggttcatat atgtggttgc aaagatgcgt
ggccccggct
1607521 accatcacca ctggctgtgg ccctttgccc ctttcctaaa gcacagtatg
attgcgtttt
1607581 ttttttccct cacccactcg gtgttgttca ttttttaaga acacttgccg
gccaacattc
1607641 cttcagtttt ctctctccct cttttgtact cctactctcc tttggtgtat
atgcttattg
1607701 tttttcagag cacctcatta tacacatggt tatattttaa gggcctctct
ctttgatttt
1607761 ggcctcggtg ggagtgagcg gtgggctggt gggagctgta gggctggacc
agcttacagg
1607821 gctccccagc cattgtctcg gtgagtgggc ttcagggtat tgtcgggctg
ggcactcctg
1607881 cagaagcact ggtgctattg ctagatcagg agttggatgt gaggggaaaa
ttgagaagtc
1607941 aataatgagg gccgcaaaaa aaaaaaaaag aaaagagagg ttgagacaaa
aggagatgaa
1608001 gagcttcaaa aagacgagcc atagagcact tctaattaat aacatcagag
gaaccccccg
1608061 cccccccttc cttctggaac ttcagcgttg catattaact tgcggggtga
gacgcatcgt
1608121 gagtcaggtc tatcacactg ccagtctatg tccatttcac ctctatgcag
tctctggcat
1608181 gaatcttgaa ggcacgtttg tattaacatg cactggttgg accagattgt
acccttaatt
1608241 tttaatagat tgccatgttt gtcgtcactc aaccctatca gctaatcaaa
agataaattc
1608301 ttgaaatggg acagattata aatcttggtg ggtcagcgag gtagagtggc
agtaatttga
1608361 gccaagcctt tgcaatttct atttttcaca gccgtaatag gctctcctct
cgctgttcac
1608421 ttccacagcc gttcccactg ccaacagctg tcaggctcct aaaatcaaaa
ttagtggcat
1608481 ggatttgggc cctaatgaaa tgacctgctg gatttacctt accgagcagg
gcggacagtc
1608541 gatactagcc atgtcagaag cctctctctg cacactgcag ccccattaat
ggaaggaaat
1608601 aggctcaagt aaatgtatta accactactt ccaccttcta attatatttt
tttcttctct
1608661 cttcataatt cattcactct ttcattagcg ctcaatattg agggaaaaaa
gtcatttaac
1608721 tggctatttt ggcctcattg ggacaccaat tatttgtccc ttgtcaagca
gggacacaga

1608781 tgagtccatg ttgtaaatgg gttgaaattg ggcagaaaat tgctgaggct tatattttgc
1608841 ttcaattcag ttctgttctt atttatctgg gtttccattg tgggaaattg taaataatta
1608901 gatataaccg atccaataaa tcctactttt ctgcatcctt tgattgggtt accaagatca
1608961 gggcctcttc caatggagtg aaggaacctg taacagacat gtggagtagg tccaggcctg
1609021 ctgttgtaca gggctgaaaa gtaagcctgg aaaatggtat tagtgtccca ccacagccat
1609081 gctgttggac cggatggagg acactgggcc catgttattg acttgtaaag gagctgttag
1609141 gtgaggggtc tcggcaagac atgtgcagct gacactcttg taaagatgta tctgatttct
1609201 atgcccgctg gctccctgga gagtcgtcct tatgttgatc agatgcgctc tcgcggctct
1609261 atagaaaaaa agcactgcaa taaaaagaat gaaattatag ttgtgtgaaa gaagacactg
1609321 cttttctaca tgttttaatc catcattaag gtcaaaatca tagtgaggga gggagtgaaa
1609381 gaaggaggta aaatagtcag ctgctggttg aaagagctgc cttttggcta catgcatgtt
1609441 ggtttgccaa aaataaacca tttcaaatta ctgcttcggt gagttgtgta acgaggatat
1609501 ctaaatccac atttctgtag gtgagcatag aaaaatagtg aattgatgta gcaagaaggt
1609561 ggacataacc agtatcaagt gaacaagaac cgtactcctc actgatctga ctaatttatg
1609621 agtgtaaact gcactggcag agagagatgc aggtggaggg aggaagtttt cattgactcc
1609681 agagcaggcc gagcaaaagc ccaggaggga tctctctcag tttgaacttt atcaggggct
1609741 acgcttaatc aatacactat cggaaaaaga attaattact aaagcagaat aaaacagctg
1609801 cattttaaac caaggattca tcaattctgt aatctcttgg ttacagctca ggtgtcagga
1609861 ggatcatttt aactccatgt aatgaggaac tgtgggagtt taccccaacc ctttgctgtc
1609921 ctcctcctcc tccacccctc tatttctaac cttccttcta acactcactt gcactcttct
1609981 atcacctcat caagacatat tccatagctt aaccttcatt ctcatccagc cgtgtttgac
1610041 acagttttgt ttttaacatt cccctcattt ccgtcccttt gcattgccca ttatatcaat
1610101 ttattccact tgcatagctc tacattccca ttccatcatc tttatctttg caagatggaa
1610161 tgggacattt ccactgttgc atcgatagag ttggacactc tttgctatgg ctgggacgcc
1610221 gctggagttt cagaagattt gtgtataaag tcaatagagc atgacgattt aagtagaaga
1610281 ttggttggaa ttaatttcat cataggacat ttacatttgt gaggacaaaa tgtaagccac
1610341 ataggaagca tcccttgttc caaaataacc ttggctggtg ttgccttgtc catcgccttc

1610401 cacacagccc ttgacccaag ctcaaagcct gtaaacggac tgtccttgac
tagccataat
1610461 gggggatttc aagggaagtt gaaaaggggt tatgggtgat gaattgggtt
tttagtgagc
1610521 tcattaggaa gccaaacaga gtttagactg gcggagcagg gcagcagcgc
agtaatcagt
1610581 tctgctcgct ttgttccctg cctccaggac atttgtctaa aaaaaaaaaa
aagagcgact
1610641 gaggaaaaaa acttcatggc atttggctga tgagagggct gctgagcaga
ggctgcaatc
1610701 tgatggttga cttgttgatt tataaacctt tttcatacat ttactctacc
aaagccctgc
1610761 agctgtagga aagcctcatg tctgtgaaag tttgatggcc ctgtttatca
agggtgccaa
1610821 tgatgtctcc tggaaatctg agaacccccc ccccccatca gtctgaaaga
tttgtttata
1610881 tagttcattg cccatattgc tatcagacat agtctaaaat gtctaagctg
tttaagttca
1610941 caaaaccaca ctgagacagt aaataaatgt tgaaagatgt gtttacagtg
caatgagtta
1611001 gattgtagat gtcaccgagc aattttaata ttgggtctaa aaagctcaat
taaggaagta
1611061 tcagagaagt ataaaataaa tgaaaaaatg aataggacaa gggaactggc
aggtttgtgg
1611121 ttctttttct tccccattct gaaacaatgg gcgaaataag gttacctgag
agaccaatta
1611181 gcctccattc gatctcgact atgagggcag accaagcggt cgtgttgaag
tgcaggggga
1611241 gcagggtgga gttgtcagag aggcaaagag tgtgaaatgc ctggtgcgga
cccaaagccc
1611301 ttcattagcc actgatccct gtctgttgag tgaagagcag tcatgggcat
cacaactcct
1611361 tttttagttc cccccaaaaa gagtgtgggg aggcagtgtg gcgggaggct
accccatccc
1611421 ctgcaattta gccctttgcc attcatgcaa agtgggcctt gacatctgca
acagcccgtc
1611481 tcctttcttt tatacatctt tatccgtttt ctttcatcca atgctctttt
cctcaaataa
1611541 agcacagatt tgatggtact gtcagccgac atggctcctt tcatgcatat
cccacacaga
1611601 tttttcttgc gtttggaagg aagtattagg aaaactcaag atcttaaaag
ggtagaattt
1611661 tgcaatctgt ttaaagttta aaattatgat gcaataagaa catttttgct
tcaggtatac
1611721 gtgcaattgc accaacagtt aattcagtgt ggggctctga aagttgtgct
aaccttgaag
1611781 ttttggcaga tgatactctt tagaaaaaag atcaagagag ttcgaagcaa
tggctttgga
1611841 gaaaaggctg taaccaggca gcctgacccc cccgaaccac ttttctttgg
cttcaggggg
1611901 gaggtgaaag gggaggatat ctttgctcca gccccttttt taggttaaaa
ggtaaaatag
1611961 cctaactggt tgttaaaacc aggttttacc tccagcacat cccctcccct
gtctttttg

1612021 tgctctcttc acttcaacct tgccctctat ggtagggatg tgcacagtgg ggtcaacatt
1612081 gtgactgtga gaggagagag gccttgtatc actgagaggc gatagttatc tccagcattc
1612141 ctgggatgac agagagatat ctgcctgatg gcagggttag ggagtttaga gaaagggcgg
1612201 gtaagggagg gagaagagga ggtacaggat ccgttgagca actacccct caaaactacc
1612261 cctgctacca cccttcgtcc ttccctcttt gcctttctta ctgcctgact gcttcgcttc
1612321 cccacacata ggaaaaaaaa catctaatta gaagctctta ttccacttca tttaagggaa
1612381 cacttaatga tgtgtttttg ggggaagaca actacacatt acttctcgcg taaagggaac
1612441 cactcagtcg tgccactcag gatgcagata cagctgcatc tgaaatggag gctttcacct
1612501 catttctctt gggctctatg accactggaa gagagagcca aacacagcag tcttgtgtcc
1612561 ttcaccttta gccgttgttc atgtggtaag agtgggcagt aaataggagg tgggttgtga
1612621 caactgactt tcgcaaggag acaggctgaa tgagagagtt tgctgtaatt gtactgacag
1612681 gttttagggc tgttaatgac tacagcctga gcgctaccaa tttgtcagca tgcttgctgt
1612741 ttactaagct gttgttttat ggtccagtgc taggggctgc tgcctgttta agcacagatc
1612801 aggaaataat cagcgtaaaa cacaggctgt ggtggtgcag ccccacagaa acagtttact
1612861 ttagatccct catctactgt accaatgatt gcagtgagcc atatggtgtg ttgaaggtgt
1612921 tcaagagcac tgatgggcta cttattggta caaacagtga aatatttgtt agttaaaaat
1612981 gattactttc tttaactatt gtctgtgttt aataatacat atacataaag catattgctt
1613041 acatgctggt gagccataat tctctgtcat tacagggtgc gtgctgacaa ttccactcat
1613101 gtgaacagaa ccagtacatt tgtgtatgtc tgtatgtgag agaaggtggg tgcgaatgtg
1613161 tgtgtgtatg tgtgcatcaa gagcatgtgg tcccagagcc ggcaaagacc ccttcgtaaa
1613221 tccagagtaa ataaaagggg taaccggacg aacaaaagag gagcccccgc ggcttcactc
1613281 attcgcttcc atttggggga ctgaatgtgc taaacggcgc ttatgttggc tgaaaagttg
1613341 aggagaaaaa gaggggaaag gaaggctcta aatccctctc ttcttttctc ttcattctct
1613401 gtggccaggc ctatggggcg agaggtggtg taatggaagg gcattggagg gagggttgga
1613461 ggcctatggc aaagcagaga aggccaaaag agccctggtt cccattgttt ctatcccata
1613521 tctactcatc tttttcttat aactttcctc acaggttgta ttctcatttg tttgagcttg
1613581 actgagcaca gttgactagt ttgggctgac agggggcttg gggggtctgt tgtaattagt

1613641 cactttaacc acagcttcca taagccactg cctctacact cttgcttcat taccctgaaa
1613701 caaatgctgg ggcctcccag tgcaaaatca ctgcgcaggc cacggaataa tatgcaaaag
1613761 tttgtctaga ggctgtctag taggtgtatt aatagtacag agacggttaa atctgtagcg
1613821 ttgtttcata agttactggc tatgatgcta ggatggagca gcattcaaca acaaagggca
1613881 gggaggcatt cttggtgtcc tgacagggtt tggatatgtg ttactcacag tcatatccat
1613941 aatgctccca gggttgacaa gactttggtg actcccccag ggccagcgtg cctcctggac
1614001 atgaagcatg atctccagca tcattaggcc ttctccaaat cttacacccc ccccttcttc
1614061 agcacagact gtagaacatt ttgtgttaaa tcagttcttt gggcacaaaa tcctgtagcg
1614121 agcccgaata tgccaacatt agataagcag agttgccgtg tgtgtttaca gtcctttccc
1614181 aacccgttaa tgagcgcagc cctgagacca atttactcac agggagatat atgtgaatgc
1614241 atttttaata accacccact ggtgatgaga tgagagaggg aatgtatgtg agtgtaatgt
1614301 gctcttggac ctgcgtgctt gtttgttggc tagcattttc tttcactctc tcttccttat
1614361 tgtttctggt cattgtcttt catctagctt gattacgcca tctcagattc tttgcataga
1614421 acatatgcac aaagtttccc agcaagcact ttacgtcaag atattggtgt agcgggtgag
1614481 agacgagagc agcaaagagt gagggaagac agaaggaggg gtgggggct gtcttttttt
1614541 ccgtctccaa gggcgctgct tttccaaaag tgcctggggc tacactccag cacctcggct
1614601 ccccgaggcc tttcggctgc ctcctaatag ccaaccccca tctgtgtcac atggccagat
1614661 gatgggacgc ttccccctgc tctgccctgc actttttct ttacccattt tttttctca
1614721 gagtgcacat ccacacaggt tagaatggca gagttgccag ggaacagtca gaaaccatta
1614781 gcgtttggga caatttgccc tgcagcgttc cttctcttgc tcataagaaa gaagtggggg
1614841 tggatgcaaa gcaccagctc tcctttaaat gtaatgaggt tgtgcaagtt gggtcttctt
1614901 tcttcttttt tctttctctc tcttctaatg tttgttcttc tttcccctct ttgtatgttt
1614961 tatttctctt ttctttttag ctttgttctg cctttgttct ttattaatgc actttgcgta
1615021 gaaacgtaaa acctttataa aaataatcag gaatcgcttt tgatgtcaca cgtttaatct
1615081 gggtaaacat aaggcacgac acacagcaga gagagagcga agtcgcaagc cttggagtcc
1615141 ggtttcacct ttgcaggcaa gggcaagcat gctcggtaat gaagtgacag gtcagtctgt
1615201 aaatacgcat gggtcgctct ggcccaggct ggagggaggc tatcacattg cagtgaaaat

1615261 ggaaatgtca ataaaattaa tctgccagcc ttttggccac tgtgtggttc cattggatga
1615321 gcctgaccgc tcacagcagg agtggggatt tggaggctag cttaaaaatc aacatcgagg
1615381 ctagcggcat gcaaacaaac aaaccaaact gtgttacaac ttcattcact ggaacttcaa
1615441 ggtggagaaa caagaggagg gtggttgaca ttcacagctc actagttgtg ttgttattga
1615501 agtttggaag caaactgggg gactcagact tgacttcctt ctgggtcaca tttctcagga
1615561 agccccttca cagccagaga agggcaatgg ccagaccaaa gtttattgtt ttaggctttc
1615621 ctgaacaagg attcatactt agcctgggat ttacggcatt tgcctctaat gaattcccat
1615681 tctacgatta caacacccct agtctgtccc tattcccata tgtccttcag atgggctgtt
1615741 gcttctctaa cttcacacag ggagcacaaa gtcggccctt ctgtttttcct gtttcgtgtg
1615801 gctctgcgct aatgatgtgg aaggaaaaca ctgcacaaaa agagggaaca aaaacgggaa
1615861 aaacaaggca gagaaagaag ataaagaaag tagggaaagt agttagaaga gagggcccct
1615921 gctgtccttg tattattcat ttattttgca tttgattatg ccaccagtca gatccttttt
1615981 cttttgcccc ttttaatttt attagttcaa atatttatgc aagattacga catgtatagg
1616041 tggttgtgat tgatgcctca ctaaaaacag gaagacatct gaggctagat tttgagtgtc
1616101 cgggtccttc actgtcttag tggaggagtt tttcagatac ataatttaga tgccctcaag
1616161 gaaggctttc aaggcatttg tgcccggggg caagaaaaaa taactatgac tgttaaaagt
1616221 aaatcatttt tgctaatgca caattaatgc cggaggaagc cgaacaagga aaaggcaagg
1616281 gacaaagatt gaaagttaga tttgaaggag aaattctgtg tgtgtgtgtg tgtgtgtgtg
1616341 tgtgtgtgtg tgtgtgcgcg catgttttcc tcttgaaatc aaacagtgac ggacattaat
1616401 gcaggcaaag ccgccactgg tctgtcatgc taatcttctg gcggaatggg gaggggttgc
1616461 tgcgagggaa aggagtggga gtggaggcct aagagagtcg ggttctatgg ggaggagggg
1616521 ggcagacgag tccattatgt gcatcgaagt gcaacaatga gcaaccccaa atcacatccc
1616581 tactttaata tttgtgcatc tcaaatggat gctgtgaatt atggtgtttc tgggcgagca
1616641 tgtgtccgtg tattgctgga catgtgggcc taactccttc ccgtcccctt tggctttctt
1616701 caaactatga aaactgctcc acattctgtc aagcatgcaa atttaattga attaatgtta
1616761 tgagctcaag tctctactgt atgcgaaggc tccctctcct ttttgttgtt gccttgctcc
1616821 agtttttcct cgtttcagtg cagttctcca acaaggaacc caatgtttca tcgtccaacc

1616881 ataatagtcg agttaataac gtccgttccc ctcttctgtg ctccagaata tagacaaact
1616941 ctgcaaggcc tatgtctaaa ttcctcaccc actgtacagg ccttcatcta aggctctaaa
1617001 tcccccagcc tccatatctt ttctacacaa cacgggcttt gctgcagccg caccacagag
1617061 cttttgaatt ctttcatagg catttgtggt atttgtccat atatccatct tccacgtgcc
1617121 ttaaacttct tgagcctcga ctttctccat tttagttttg gcttcttgtg gattttactt
1617181 ttgtgctctc agtctgtggc gttgcccgcg atgttattta atgctggctt tggtttccgt
1617241 ccaggagatg gcttagacaa cagcgttgca tcacctggca caggggacga cgatgatccg
1617301 gaaaaagaca agaggaggca gaaaaagcgc ggcatcttcc ccaaggtggc caccaacatc
1617361 atgagggcgt ggcttttcca acatctcacg gtaagagcct aaacctaaat aaacgtccca
1617421 tacagaggac acacacacac acacacacac acacacacac acacacacac acacctgtca
1617481 cacaatcatt agtgaaggag taagtgacag cagtgctgtg atgctcacct gtcagccagc
1617541 tgtcagagga acacagtgac caggtcaatc taatcagttt ggattgttta ctcccctcaa
1617601 cacactctga gctgtttttc tacagcacag agtaggtccc agcacagatg caggggtatt
1617661 tggtaattaa tccacggcaa ttacctattt tcctagttaa acacggttgc aaaacgaagg
1617721 gcatgacggt gaagatgcaa catgtgcaac gatgggaaga ttttgtcagc agctggtcac
1617781 agggccaggt ggtagagcaa tctaaaacac aacaggatgt ccactgtatg cctgcagttt
1617841 ccccaataaa tcctgctcaa ataaaccttt tttctctatt gttttctgtg ctctccaagc
1617901 aaccaacact tcaagcaact caagtagcaa cacatgtgcc taaattacaa ttagattacg
1617961 cgattcaatc gctagtacgc gcgctggtac acgcgctcac acacccttca ctaatgtcag
1618021 gacattatca gaacatcagg ctgtattgat tttgcaacac aggtcagatt tcaggaggag
1618081 tcggctgtga tgcaggacac aagctgctca catgtcccag agatggcagg acaataaaaa
1618141 gagtgtgaga gtaaaagaag aaagagctgc acaattgtaa ctcgggtctc ccctgcttcc
1618201 ccctttcttt cccagtgccc gctccttacc aatggccctt gtaccctgat taccgaacca
1618261 aaagccgaag taatgtgtgg ccagcaaata cggtctaatt gaaagggctg taaatccatt
1618321 acatattggt cagggataat caggagtgct ttaatcactt ttatctattc aggagcagtg
1618381 aagctgcaac agcaaagccc tgagccttgc aggccttagc agttccacca cgccagtctg
1618441 cgtgcttatt attcccgtca ttatcaaaat gacttcacag ctgttcacag ctccatctct

1618501 ccctgactcc cagacctcgc accacactaa acaaacccgc ttccattcac acgttcaggg
1618561 caggcgcgac accgtttttt tttttttata gtggcaattt atttgcgctt gaagggaaca
1618621 aattgtattc taaattttat tatttatttt aggtgtgttc aaatccagga atttttgccc
1618681 tggaactaca ttgttatact tctaatacat ttcgggttgt aatattatta tcttaattgc
1618741 tactgctggg ggaagcagat ggaaaagaga tgttgcatca tctcctgaca attaaacttt
1618801 aaatggaaat ggaaacactg ctgtggtggt ccaaaaatta gtcatttctt tagaattgtt
1618861 taaaattcag ttaaaaaggt gaaagggaaa ggagaggggg gaaaccccag tgttgggcac
1618921 aggcacattt tttgcccagt tcattcagag agctttcaag gccctcgttt tctctgtctt
1618981 gctccccttt atcgcatcca ctgctcctac atgttctctc caatctttat ttgtttcttt
1619041 tgttcaaata acaatccctc ccttttaacc tgccttcatt tccctactga ggaagaggac
1619101 taattgaatt atccccottc tcttctcacc atcaaattct cccacccata aactctctcc
1619161 ctcttttaca cacacacaca cacacacaca cacacacaca cgattcttct ttcttttaat
1619221 ttgttaagca aatgtatttg ctattgctgg atgcatttgt gcaagggtga attcttgggg
1619281 actttggccc ctgaatggcc tatagtggca ttctgctatt catgcattca ttaactgagg
1619341 ggtgaggagt gaaaggagcg gccttgtatt ttctcaaact gtgagggcca gcatggagct
1619401 cctcgtccat gagccacagg ggtctctcta catatgttct caacacccag ccattgtcag
1619461 ttcttctgaa aagctcaaaa ggactcgacc cccctgtgct cgcacacaga aaaacaacag
1619521 aggccaatag taacaaatgt tggcttttgg attcagtata acaaaattgc cctctcccta
1619581 tctccttcaa gccctctgtg caccatattt ggctggaaga aatgacaaag tgtcatcaca
1619641 tgattatgaa gtaatctttg tgtgtccctc atcccaaatg agaggatata ataaaggaca
1619701 catatagatt taattctagc tatattcaag ctgactgtac agagtgcatt tgaggtaaca
1619761 accctaaatg ccccatatac tgacaaatgg ctggacagat cagctggagt tagccggcag
1619821 aagagggtca gccggtctct ccaatcccct ttctatccct cccactccca tgtctttctc
1619881 tctttctaat tcccccctcc ctctccatgt gtttctggat gtcttccagc cacattaggg
1619941 ctaggaaaat cccagactgt tggtataagc ttttcaatac tgcagggaga ttatgttatc
1620001 tgaaaggtaa acttcactgt taggggtaaa atgttctaga gcgcttcaaa agcactggca
1620061 acaaaactgc acttgaggat atgggtttag aaataatagg gtagataatg aagtgaaaat

1620121 agagattttt gcagatggtg caagcaatgt ccttttgttt ggtgaaaact ctaaaatcta
1620181 caattagccc ccaggtgccc ctggccttct tctctctcac cccctgccag ccccctgtaa
1620241 gcccctactc acccttgtaa agacaagggt cggcctcgca atgaaaacag gaagcactca
1620301 aatccaaaga taccattctg ggactgtggc cttgcctttg acaagggtaa accctcaaac
1620361 acatctgaaa ataaataaga gacctaagaa gcaaactagg ccagcggaga gcgcgtgtgc
1620421 tagatagatg gtgggaggga ggcacggagt ggccagtctg gtcagtaagt ggggatccgt
1620481 aggaggtatc aagcttctcg ggttttgttt cgggacaccc tttggccggg gtgtttgggg
1620541 gaatttctgt taagctgtta agccaggtgt attggtcccc tgggctcctt tgaagggaag
1620601 tgttatttgg aaaagccttc agctgtgaac tttaaaaccg ccgtttgagt ggaataccgt
1620661 gtgcgtagat gaggttagac ataaatatgt ttcttgttag gtgaccataa acatattctg
1620721 ggtctgtagg ggcgctgtgt gaatatgcag gtgtcaacca cactcacgcc atgtgatgag
1620781 tgtattgggc agaaaacctc attagacacc cgtgacagtg gctacccccg cgtccctgcc
1620841 gccaccaaac accaatctta gcccggcata gcgttggtct ccatggcgac ggggtgtcat
1620901 cgccaggtcg agccctcctg gctgcccttt atagatgcac ataaaagagt ccgccagggt
1620961 cattcacaag acatgtgtgc aggcaaggcc gaaccctggc aacggatgtt tgtcctgtat
1621021 gataaacaag tgtacgagtc catccatttg aaaagagagg gcatgttctc ggtgtagact
1621081 aaacagtttg ctctgtagta tccccagttt tgtttaagct cctccagtcc atctaatcat
1621141 tgtgtgttgt taccatttaa ctggtgggga tgcacatctg ctaacagttt gaaatacctg
1621201 agctatttct ccctctaagc tgtcaaaaac tgttgaattc atttttttgg attcaacttt
1621261 atctttttct cttaaaacaa aatttttttc accgtttggt gactttatgt gtctgatttg
1621321 tttgatttta gcaattgtga gtggcattgg ttttagctaa ctgtttcaaa agctgtttgc
1621381 ttttcagcgt tcttagactt tggatgaggg gagggtggag acaaagaacg agtggggagg
1621441 ggtctcctga agtctcctaa agagggggct cctaagggtc acagagctgg aagctctgga
1621501 ggaggcccac acactaatct gtcatgtgct tgtttgcgtt ggagcccaaa agtcagctct
1621561 ccaatcaccc gtagaaagcc aggcgaccgt ttggttcact gccgctgccc gagtgtgtgt
1621621 gacgatcagc gaggggagaa aaagagtcat cacattgcct gtaaggctta ctgctttgtg
1621681 ggtttacaaa agagacctca gtgtcaatat gacagcataa ttaagcgaca tttagtgcag

1621741 aaatatttaa aagtcattgt taaggagaag atctgcggag aggatcgtcg gtggtggtga
1621801 tggtgtgtgt tgggaagggg gtgttgtgcc tggctattgt ctagtcactg ttctccagca
1621861 ctggctgctg cagagcagag acacagccca cctcagtctg ccatctgttc ttccacaagt
1621921 gggccacctc tgtctgtctg tcacagcaaa cacagacagt cgggctgcag agtggcaacg
1621981 acggcaacat aattattctt tttattttca tcgccatcgc ccatgctgga actcatgtga
1622041 tgaagttgtt gttttcaaga gcccagagaa cagagtgaag atttagaagt gctgggggag
1622101 agctagcgac atcccatttt cacaggctgt ataattatat ggcagaccgc atcaactttg
1622161 ccatttcaga gcagagcggc tttgtggtga tttcccagtg ttagcatgtc acagctctgt
1622221 ttacagtagg ctctggctga gctggtagct gttccacagc tttagtgctg cattagaaag
1622281 caataaatcc cagtttattt catgcctctg catcagaaca tcacacttca cctccaagcc
1622341 agatctgtga aagttccagt gtgaactgac cctacatcga atcgggtcgc acataaaacc
1622401 aagcacatta gccatgcacg tgctgccatg tggttattgt tgtgtgttcc ctttccatgt
1622461 catttacaag cggagcgccg ttttactcat gtaaatgcag caatctttgc atattccaat
1622521 acttctccac cctccctgtt aattttcagc cctgtcagct gagatgagtg ctttctggga
1622581 agggactgct tgtgccatgt tcccactgta tggaggtgaa taagctgtgt gaaggagcaa
1622641 gatttgagaa aaaaagatga aaacaacgcc aaacccaagc atatgagcaa aataaataca
1622701 gtccaactat agaatatata ttagatttaa tataatattc catgcgcaac atcagtgtca
1622761 tggaagtctt ggagcacagc aggagtttgg cccccggcag gggtgactgc ctgtgtcctg
1622821 accgcagcag aatcccgtgg aggtcagatt tataaactca cagctccgta catagaccct
1622881 tccaagaaca ctcgcagaca tgtttgctat tgcaaaaaaa aaagagcaga ggaaacttct
1622941 tttaaggccc actgagaaaa ggccccagaa gggggatgtt tccctccctt tagcacggca
1623001 aggcagtccg ccgctgctgt agtcataaca caaatctgtt cgctggggtc tggctgagct
1623061 gactctgtaa gaaataaggc tgaatgacat catgtgtagc ctgtgttagc atatatgccc
1623121 ctggtgggaa agcagttatt aagatgctgg tagcatgttt tggttgtgtt ccctataagt
1623181 ccataagagg attttggttt cattcttgga gctagttttc ttaaattacc cgtgcagagg
1623241 taccaccctg caggctagcc tcatcttccc acagttgatt gtggggttgt ggcaagagga
1623301 gctagagcgg cgactgctag catgccagcg gatctcggac gccaatgaga ttttgctgca

1623361 agcctccaag ttggtgcagt agtgctttga tattcatctc acaccaaaag aaaaaaaaat
1623421 gcaaagggga ataggagcag cacacgcatg gaaacggcaa accaggccaa aaggagggaa
1623481 ggaggaacag atagaaaagg tttgagggat gaaggtatgg ggcggaaatc aaaaacagac
1623541 ctgatgtttc cctgtttggt ttgtcatggg accctggctg caccagatcc tgctccagcc
1623601 atttttttct ctgcccctca ccttcctctc ccgcacatac attcttttgt tttgccagtc
1623661 cagtggcagg ctgtgggatt cctgcacagt gggctcctta taactgggag ccatgtctct
1623721 tgttctcacc tcttcccccc acttcctcac cttttcattc ccccccaccc cctcagatat
1623781 cctcccctga acatatggac tgatttttaa gagacagccc agtccacatc aaagtccgag
1623841 gctgagcggg tcaatttgtt gctcttttcc cagactcttc cccctcgaca agcctcggtg
1623901 gaccctccca gtgctgcagc catttaccca gaatacacca ttgctcaagc gctccagctt
1623961 atatcgagcc atatgtcgtc ggtgtcccta ttttttttccg catggaccaa acctcattct
1624021 cttcaaatgt ctggatttct ctgcagccca ggaagatgag aaggctgtag aaataagcac
1624081 gtgtgatgga taacaaagct ggtatcaaaa atagctccac cacatgcaga taagaaaagg
1624141 cgtctggatg gatgatgatg agaacaaaga gaaaaaaatt gcataaataa attcaaaaag
1624201 agtgtaaata agtatatata aatccaaatc caggcagagc aggtcttggc tctagatgtg
1624261 aatctctgaa tattgatcat ggggaaagct agtccctgat atccccgggg ctccagcgaa
1624321 tgacacacaa agcgcccctg taaaacccat aaaccagctt gtacaattat actgataaaa
1624381 caggcgacaa cgccagtgca tgcctcccaa cccatccttc ttggcgctcg tcctcctact
1624441 acaacgctgc tcctcctttc cccctttctc agaaagatga agaaattaaa aaggggcaaa
1624501 tgtgtaatta gccacacatt tctgaatcct ccttaatgtt cattaatgaa acccggggca
1624561 attacaagtt caaaggggtt tggggcatca gcagcccgca taatcagtgg taaattaagc
1624621 acagctcatt tgttacagcg cagcatgcgt gctcgtatgt gcccacggta acacgtatgg
1624681 atgcatactg tcagagttga ggcacagagg agctgacagc catcaatttc tgcacaggca
1624741 caaatctctg ctaccccaag gctctaggtt aggttgcaca atcctcttag agtctgccgc
1624801 tttgttttca ctagacaaca gcaaacacca ctgacacctt cacctcggct cgctcaacat
1624861 ttcactggat ccaactgcat tcatcctctt ataagtgtcc tgtttgctcc tacaatccct
1624921 ctccgccacc tggcctaccc tccctgagtt tataggttta ttctaaacct atatcctcca

1624981 ccacgtctgt ctccactgtc ggaagacgtg ctcgggcagt gttcttacca gtatatactg
1625041 tatatatata tatatatata ttgaatattc cccatatcat tagtattccc cgaaaatcct
1625101 tgttttgctc ctatcatcga cccactcaca cggttaaact gttgatatat tcccataaac
1625161 gtggctaagt tgttgtcaag tgcaatactt aggccaagtt atttttcatt gtgtgtccct
1625221 ctccacctgg ctgtgtctct gtgacacgtg acagtgtgcg tttcacgtct gtccccggag
1625281 gcgtagttca gttacctctg tggccaggtc acaatagcta atcctgtctt caggtggctg
1625341 tttagcacag tcagaggaga gctcacagaa ccggaggtga gctcctgtta caggactccc
1625401 tgatcagggg tcacgtccca caccgccacg ggacgtgcgc gcgtgccctg gtgaacacgc
1625461 atgcacacgc accggtgttt tcaaacacca acactgtcac tcaaaccctc agtccctttc
1625521 taaatcccag caggcctagc cttcatctgc tgtcttcttc cttatctttg cgctgtgcta
1625581 cttctaaact ttccctccct gcccagatca gtcagacttt tgttccatta gggttccctg
1625641 atatgatctc catggagact tgctctggtg gccatgcgtt gcctgtccta cccggcaccc
1625701 tcattgttta agctgctctc acacggggac gctgtcaggt ctccagcgga ttgaatagag
1625761 ccctatcatg tgtggctcag cacagggaga gggaactcat ttttcactgg tggaacgaca
1625821 tccatctcag cagccaaacg caggctgttt cattaagtag cccatccagg cagacagatg
1625881 tgttccccac acagccgtgc ttgcctatgc aagagtgtgt attaacccac aatcctttgg
1625941 gggagattaa taggctacca gctcctttcc attgattatc tcgctgccat tttctcatcc
1626001 cttccttggc tagatattaa aaactaatga agcctgttga aaacgggaac cgggaagctt
1626061 ttcacggatc attttaggtt atttggactc aaacagagag ggatctcttt aaggcacagt
1626121 ttgagttgaa ttaagaaaat aaatgcggag agcggtggtg aagaattggg ggtgcaagac
1626181 ggagagagga agctgagagg ctgcgtctgt gttcaagaaa gctccgctct gacacatgga
1626241 gcggagatgt ggcagatgtt agcaattcca gcgcatagaa agcattcctc acattggaac
1626301 catgtcttaa ttactgtaat ggaggtggaa agcactgaca gtaatggatt tgtgaggatc
1626361 catcctcaga atcctgtcaa aggggccact tggtagatgc tccttcatat ggcctttgat
1626421 tttcattatc taatctctgt atcaagctgg aaataattct cataatcagc tgttcgccat
1626481 tgtcctggcg ctagaagtag gtggttcagc tgtgggagtt gccagggtga gggtacgata
1626541 cggagggaga tagacggatc agagaaagca gggtagagta agcaaagagt taaaagcatc

1626601 catgtgatgg gttcacctct gcaccaggcc tggtgaaagg cggcagggtg ctgtcgggaa
1626661 aatgacttcc tgagacatta ccatcaatca ccagacaggt caggcccccc aatcccggca
1626721 tcaggatgga ggagaggaga gggggatggg gcaggattag actagaggga ggaagaaaac
1626781 agaaaagaag actgtgtaaa agcacgttgg ggaacggact attaacattc tccttttcta
1626841 agcgcctagc tgctcttttc ctggacccat ctgtctcccg ttgtctttgt cttctgctgt
1626901 tttcctgttc tttatgaaac agctaacgcg gctcgcagga acagctcctg ctggtcaaag
1626961 tcctctgctg gactcaattg catcattaat tattccacag ccagtggact agaagtgcct
1627021 ctatgaacca agagataaat agaaagcaac agtgacattg gaagaccggg tagtgctgtc
1627081 tttacacccc cagttgatct agattttgca tttctttccc tctccccccc ccatgccttg
1627141 ctcattctgt aaaatgatgg acgaggggag gggcttgggt tacctcagtg agcggttgtg
1627201 ttacattcct gagaagggaa ggggtagtgt ggcggttcaa cgagttgttg aaaagaacac
1627261 agatgcttgt caacagcctt cgggatgggg agccatcctg ccttgcctta gagccggagg
1627321 tcagtgacct tatattgtac ccagatagag acagagacat agggaagcac cagaaagtgt
1627381 aagagctcat aatgtggtgt gtgtcgtggc tcgtccgtga caaaggtcag acaaaagtcc
1627441 taaaaaccct actttttctc tcttcaccca taaaatattg tacatgagct gtgaattagc
1627501 tttctcccta gcgcccatat aggaaaagaa agtggggaga caggcgtaga gcggctggga
1627561 acacggtaag caccgctcgc agtcatggcg gaatggccca ttagatttaa ggagtaaaag
1627621 ttcatcagct ttattaatgg caagatatat aaatgaagta tgaattacct gacttttatg
1627681 gagtggtgtg agtgaaacta cagcgtctta tgatataatg tttcagtgag ctgttctgag
1627741 cgctctgatg agcaggctgc ggagaagagg aggagggaac aggcagaaag agagcgagag
1627801 agaagtgtgc tggggtcccc tgagtccagt ggctggaagg ttgcctgatt gttgctaaca
1627861 tgcgtagctg atcgggttag cttggagaca gggacgtttg tagcttccag ggttattctc
1627921 tgcgtgtcat tagctatcgg tgtgggggaa aaaaaatacc gagacacata aaaaaggtca
1627981 gacactgatt ttgttgctcc tcctacattt gcgttaaatt tgccaaacag gcatgcagat
1628041 gcgtgtgtga acaccagtgc atgcgaggga acaagcgcac ccacagacag acacacaaaa
1628101 cacacatgct ccccaccatc ctgtaataag cggtaagctg agctagctga tggtcatcat
1628161 cccatggggc tgctgggtgg ctgccaactt cctgactgct ttgtcagtgg ataaagcccc

1628221 taacaaacga gagggctgta gctaggcagg ccagccaggt cgaaggtcac ctcggccccc
1628281 gggcaggatt gggcctaaga gcactacaag gagagaggac ggaagaatgc ccttaatgat
1628341 tgactagtat tagcgttaaa agctacatag ctcctcttct cctcttctcc tcctctcttc
1628401 tcttctcctt atgagggtgt ctgtcattct gcacatacga tcagctgcct cgggtgttga
1628461 gcaatgacag gagaaatgct cttgacttcc acacctatag atgaaaagag aatggcaaaa
1628521 gtggtattcc agtaaaaaga gcagtcacaa caggtctggt cggggtggtg actgataagg
1628581 gttcggctgg ctttgttcca gggaagcact tgtgttctca aacgaatgca cctatggata
1628641 gcgccagcca tgtttctctc actgacgggg acatgatctg aggagcgtgt cagtcgtggc
1628701 ctgagagctt ctctcaccag aagaggacag aggagaaacg ggaggcaata gaggagagaa
1628761 aatctttcca tcccagctgt cctttcaaag ggagagaaaa tttgccatgg ttgacttgaa
1628821 cacatttagc atcattagca aacaggaagt gtcccctgca gctgtttttt cggtggagga
1628881 gggggcttgc ccttcttcca ctgtaatgga caaatgttac acacaattgt cgcctctgat
1628941 ttttgacctg aaaacaaaaa ttgacattac aaatgtccta taatatcgtc caatttgcct
1629001 tgacattcca tagctggccc cgggggattc ttgtttgtcc tctttaagtg gttgtgctct
1629061 tgttgttgtg tcccgaggct cactattaga acgtactggc ctttctttca agaatgcaga
1629121 gccttaaaga gacagaggga gagactacaa agagagagct aaaagaggtc ctagttgtgt
1629181 gtttgaatac ttatgctcat taacttaatg cacagtgaaa gggagaagca tcgagtggga
1629241 actctcagaa gcttctattt gaagagtaac ccgactgctt ctctctgctc agtccgcctc
1629301 tccacagatt aatctgtttc tgtttcaatc gaaagatcag tcccactgct ccggcttggg
1629361 gagtttaacg aagtctaaag aacacaacag ataaagagga caaacctaaa gagacactgt
1629421 atgttaatac ttggtcccaa cagacctcaa caggcaagtc tgttcatcag ccccctccac
1629481 cgtttccagt agtctatact attaaaagtg ttaaaactgc atatggaaca taaacaaata
1629541 aatataagga tttttaacac agtcggggtt gcagtggcga taatatcaca ttaataggaa
1629601 caaaaaaaaa actttttctc caccttagac ttttcttaca tgtctcatta gaaagccatt
1629661 gctttttttt tttttttaca atatgtacac accccattaa accagtgctg gtgtttattc
1629721 agaggctttg ctcattcagc acactttaac tacgttccca ccgactttat gaccaacaag
1629781 tgtggagatg aggatcgtcc cgcgcagaca agtgggtggg tcttgtattt tgactggctc

1629841 gcccgcttga tctcctcaat gtccctacac acagcatcac aaaaaccatt attttaaaaa
1629901 ggcattaaca cgtgtctgta gcaattatta taactactaa aacaggccca gttgttatct
1629961 ttacaagtct tttcaatgga gcaagtagca agcgtggctg tgtgttgaac tcaggcgggc
1630021 tggagagcgg tgcagaacag agccgggtgc ccctgagcag gcagttacca caacaaagcc
1630081 aacagactgg tctgaaaaac caggccaaat tcctgccctc taggacctgg cttaaagctt
1630141 gagcggaagc tttgaaaaga gggggacgag atgagatgag aggagaggag agaagaggag
1630201 gagaggtgga gggaaggaga cgaggccaga cgggttagcg tgcagagaga aagagatagc
1630261 tgctggggcc cgcggagtac ggcaaggaga aggggagaga gcgagaacag acctggcccc
1630321 cactgaaatg atggagggat cgtattcctc ctcttgaaag tggaggggca gccgtgtggt
1630381 ggaaaaaaaa gaagccatga acttgatctg atgattaaat aggcacatct ctgtggcctg
1630441 cagcctcgac caggttcaag ctcagtttgt gcgcatatgc atctgcacgc aaatttcaaa
1630501 tggtctctgg gtcgagctcg gcgaggcttt ggtcacgttt ctacacaaat aatcaataat
1630561 ttattcgagc attttgcttc ctttattgga taaaggtggt gcaaaatgat caccgtagcg
1630621 tttagaagcg gatccgctaa atctggagct gcgttgggat tgtgtgggag tgattgctat
1630681 ttagcctcat gcagcttgga acaatcagat gcctataact tacatatcct ccagggctcc
1630741 atcctcatcc tccctctcca ttcattcctc gctcccgccg actcccctcg cccactttca
1630801 ctgtagccgt agctttggca ggcgagcggg gcagttatgc cgtggagctt ctctacaacc
1630861 ttgcaccatc tctagtgaac ataaattaat ggtgtagagc aaacaggctg ctggctgaca
1630921 gccaggagct ggcttcagga gcccagccag caattgttgt ggctggccgt ggcagcaaca
1630981 gcagcgctag tgctgatgag ctgccaaacc agccagccag ctagcccgtc agagaggcag
1631041 acagacagac tgggggccct tttgctcgat aatagatgcc gtgagcctca ccaccgcgcc
1631101 ttccttgtat ttgcaatcat cggtgtattg ttgtcagaat gcagcctgtc gagagccctc
1631161 gtgcttaagg tggaaactct ggaggctgga gcgagagagg caggagtgca gcggtatgtt
1631221 ctgaatgcag atatggacgc gctggcgttt tcttttatca caggttggtt ggcatgtggg
1631281 gcgtgcagtt ggcaacatgc gagctgtgtt tataagttgg ctccatgagc gcgcgtgttt
1631341 gtatgtttgc aatcagcaga aggggaaggg agttgcaggg ttagatcact gagtcatgcg
1631401 gcattcttgt ctgttttcag cctgctgtca ggaagctgtg tgaggctcat tataataaaa

1631461 caaactcccc aaactccttt ccgtttaagg agaaaaggaa aaagcagatt gagagttgaa
1631521 gctaaaagga ataaagtata tttttacaat tatgagtttg tcctcacgtg atttggttcc
1631581 ttgaatcagc cttaatgcaa caaacacagg aaacaggcgc tattcccggt tatcagaaac
1631641 atttgaaaaa taattatttt aacatgaaat tctcatatat tccttttaag ttatgattca
1631701 ttcaccgtat gtttccacaa atgacacgga atctgtcaga aaacagcggc tgaaaagcca
1631761 ggtatcccca gcaatcgtgc aggctccgga atctccggct ggattcatct gtcatatctg
1631821 tttgatgtcc ccgtaatgcc gatatgcatt caatcccatc gtcatttgtc agagaagcca
1631881 attgtggcat tattaacaaa ggcaagtcta tatttaaaat gtgccaatct cgcctccatc
1631941 caatgcagat tccatattaa aatgtcattc ctatatttga ttgtgtttgt ttctgacagt
1632001 gaatattatt atcatcctcc agtcactttg attaaaaata aataagattc caatcaatgc
1632061 aaagagggaa tatatgaaag gaaacaccag aatgggctgt tgctgtaaca ttagacgcaa
1632121 gctcacagca aagccaatca aaagcccatg aaagagaaaa aaacaatacc attacaatca
1632181 gtatcgagca aaatatgagg gcttctatag aataaaaaag cgtatatgca aatcatctaa
1632241 taaggctctc cttaagacaa aataaatatg gttaaatcac agaagtcatc atcagcaatt
1632301 gctttctgtt gccgggatac cgaggtaaac agccactggc ctgattatca atttcctgag
1632361 agtcattagt gtggaatagg tttgcaagcc tccgcgaccc cgtgatccga ctaataacta
1632421 ataagtcatg tcacacgctg atgatgtcat ccatcagctt ctcggtcagg caagggggca
1632481 gggcgcgaaa tgtgtgaggg ggaggtgggt gaagacatgc ggtgtgaggt aagagcgggc
1632541 taaaagtagg tgtggttatt gtatgtacgg tgcaacccca agctgggctc aggggggggtt
1632601 taagggccga gcacagagga tgatgaagct tcctcaggtt gtaatgttcc cgcaaccaca
1632661 ggggccatta ctcccagcgc tatcagagag cagagagcgc atcacaccct gtaatttagc
1632721 tggcagttgg tcgcacaaac acacacacgt acacacacat gtacgcagat ggcagaatgt
1632781 gatccacccg tgcacgcgtg gtatgaatcg gacaacacgt gtcactttag tccagcgcac
1632841 gctcaggcac attaacgggc tttagcgtaa ttaacggagg tgtttcgatt ggaggccagt
1632901 gctgcccaga cagcaaattg aagccgctca tttctggcct atttattgac tggctgtttt
1632961 acatgttgcg gtcaatcctc ttttaaggat attggcctcg gtgttcggaa ctgagcgtat
1633021 tgagcgatcg ccgagctgct ggggagatta tcaatagcat ctttgatcgc ggccggtgtt

1633081 tacgtttcat ccattaaata accatgttgt tctgcttcct gggggtgaaa aagagcagtc
1633141 gtgggaacca aaaacatttt ctgatagctt ttgcatttgg actttatttg atcacttcgg
1633201 tggtggctct cattttccct catttttgca ttaatttgga tgatttacta agtttttttt
1633261 ttaaactgag gtaattaaca ttaaaaggtt acatagaggt ctgagtagcc ctactccaat
1633321 ttcatgcatg caggagcact gcgaccactg ggccaccatg tgttggctaa cctctcatgt
1633381 ccctgtgtgt gtgtgtgtgt gggggggggg gggtccattg ctaccacaca tcattnnnnn
1633441 nnnnnnnnnn nnnnnnnnnn nnnnnnnnnn nnnnnnnnnn nnnnnnnnnn nnnnnnnnnn
1633501 nnnnnnnnnn nnnnnnnnnn nnnnnnnnnn nnnnnnnnnn nnnnnnnnnn nnnnnnnnnn
1633561 nnnnnnnnnn nnnnnnnnnn nnnnnnnnnn nnnnnnnnnn nnnnnnnnnn nnnnnnnnnn
1633621 nnnnnnnnnn nnnnnnnnnn nnnnnnnnnn nnnnnnnnnn nnnnnnnnnn nnnnnnnnnn
1633681 nnnnnnttgt gggggggggg agtgtgaaga aaggctgtac gtaggcacat tgtgtgtgtg
1633741 attgattaca tttttggtgt gtgtgcatat aagaaaatgt atgcacgatt tgggatggga
1633801 ggaaggcgaa ttgtctcaga cacaggaagc caaggaattg gggaatggga atgagggtga
1633861 ggagaagtgt aagctgaaca aaataggatt tttgtgcatg tgtgtgtgtg tgtgtggtgt
1633921 ctctgtggtc agagtaaatc tccttactca ggggttttgt gcacgaaggg ggctttaacg
1633981 tccccactct ccttgatgag acatctttct tgtttgttca aaccattcat tacgctgtgg
1634041 ctgttggagg agcaacatgc cactggctgg tgtatttatc acccagctaa agcgcgctct
1634101 ggctgacagt gggcgattga tgaccgggcc cgggcctgaa agcccgagga agccttaata
1634161 caatgtagat gacccactcc catttccaat ttagactgct cttctcccca cacctcggca
1634221 ctgcatctca gctagtgtac gtctcaattt gtgtgctggg cacgggggtg tgtggtacca
1634281 ttgtgagtgc atgtgacagc aagcgagagc gtggaggctc tataggcatg cgggtttgat
1634341 ggtgactgag gtcagagcta atgtgaacgc tgaggaagct ttgcagtgta agagggaggg
1634401 gaaaggggt cgcactcttg ttcagtcctg ttcacacaca cagacacaca cacatgttgt
[truncated: 460,098 more chars]
